# NR-2L: A Two-Level Predictor for Identifying Nuclear Receptor Subfamilies Based on Sequence-Derived Features

**DOI:** 10.1371/journal.pone.0023505

**Published:** 2011-08-15

**Authors:** Pu Wang, Xuan Xiao, Kuo-Chen Chou

**Affiliations:** 1 Computer Department, Jing-De-Zhen Ceramic Institute, Jing-De-Zhen, China; 2 Gordon Life Science Institute, San Diego, California, United States of America; University College Dublin, Ireland

## Abstract

Nuclear receptors (NRs) are one of the most abundant classes of transcriptional regulators in animals. They regulate diverse functions, such as homeostasis, reproduction, development and metabolism. Therefore, NRs are a very important target for drug development. Nuclear receptors form a superfamily of phylogenetically related proteins and have been subdivided into different subfamilies due to their domain diversity. In this study, a two-level predictor, called NR-2L, was developed that can be used to identify a query protein as a nuclear receptor or not based on its sequence information alone; if it is, the prediction will be automatically continued to further identify it among the following seven subfamilies: (1) thyroid hormone like (NR1), (2) HNF4-like (NR2), (3) estrogen like, (4) nerve growth factor IB-like (NR4), (5) fushi tarazu-F1 like (NR5), (6) germ cell nuclear factor like (NR6), and (**7**) knirps like (NR0). The identification was made by the Fuzzy *K* nearest neighbor (FK-NN) classifier based on the pseudo amino acid composition formed by incorporating various physicochemical and statistical features derived from the protein sequences, such as amino acid composition, dipeptide composition, complexity factor, and low-frequency Fourier spectrum components. As a demonstration, it was shown through some benchmark datasets derived from the NucleaRDB and UniProt with low redundancy that the overall success rates achieved by the jackknife test were about 93% and 89% in the first and second level, respectively. The high success rates indicate that the novel two-level predictor can be a useful vehicle for identifying NRs and their subfamilies. As a user-friendly web server, **NR-2L** is freely accessible at either http://icpr.jci.edu.cn/bioinfo/NR2L or http://www.jci-bioinfo.cn/NR2L. Each job submitted to **NR-2L** can contain up to 500 query protein sequences and be finished in less than 2 minutes. The less the number of query proteins is, the shorter the time will usually be. All the program codes for **NR-2L** are available for non-commercial purpose upon request.

## Introduction

Nuclear receptors (NRs) are key transcription factors that regulate crucial gene networks important for cell growth, differentiation and homeostasis [1], [2]. They function as ligand-activated transcription factors, thus providing a direct link between signaling molecules that control these processes and transcriptional responses. Many of these receptors are potential targets for the therapy of diseases such as breast cancer, diabetes, inflammatory diseases or osteoporosis. Nuclear receptors form a superfamily of phylogenetically-related proteins, which share a common structural organization. The N-terminal region (A/B domain) is highly variable, and contains at least one constitutionally active transactivation region (AT-1) and several autonomous transactivation domains (AD); A/B domains are variable in length, from less than 50 to more than 500 amino acids. The most conserved region is the DNA binding domain (DBD, C domain), which contains a short motif responsible for DNA-binding specificity on sequences typically containing the AGGTCT motif. A non-conserved hinge (D domain) is between the DNA-binding and ligand-binding domain, and contains the nuclear localization signal. The ligand-binding domain (LBD, E domain) is the largest domain. It is responsible for many functions, such as ligand induced, transactivation, and repression. The F domain is in the C terminus of the E domain, whose sequence is extremely variable and whose structure and function are unknown [3]. Not all the NRs contain all the six domains.

The importance of nuclear receptors has prompted the accumulation of rapidly increasing data from a great diversity of fields of research: sequences, expression patterns, three-dimensional structures, protein-protein interactions, target genes, physiological roles, mutations, etc. These collected data are very helpful for data mining and knowledge discovery. NR superfamily has been classified and assigned seven subfamilies based on the alignments of the conserved domains [3], [4]. As a rising branch, the recognition of subfamilies of novel nuclear receptors is crucial for developing therapeutic strategies for the diseases mentioned above because the function of a nuclear receptor is closely correlated with its category.

Although the sequence similarity search-based tools, such as BLAST [5], are usually applied to conduct the prediction. However, this kind of approach failed to work when the query protein did not have significant sequence similarity to those of known attributes. Thus, various discrete models were proposed. The commonly used feature extraction methods are based on the concept of pseudo amino acid composition (PseAAC), which was proposed by Chou in studying protein subcellular location prediction and membrane protein type prediction [6], where a detailed description about PseAAC was elaborated.

In 2004, Bhasin and Raghava [7] have proposed a nuclear receptor subfamilies predicting method with the predictor of SVM and the input features of amino acid composition and dipeptide composition. Recently, Gao et al. [8] reconstructed the NR predicting dataset, and introduced the PseAAC [6] as the feature expression, thus enhancing the predictive quality. However, the existing predictors have the following shortcomings: **(1)** The datasets constructed to train the predictors cover very limited NRs subfamilies. For instance, the datasets constructed by these authors [7], [8] only cover four subfamilies. **(2)** The cutoff threshold set by them to remove homologous sequences was 90%, meaning that the benchmark dataset thus constructed would allow inclusion of those proteins which have up to 90% pairwise sequence identity to others. To avoid homology bias, a much more stringent cutoff threshold should be adopted in constructing the benchmark datasets. **(3)** The existing predictors could not filter the irrelevant sequences, and all the input sequences would be assumed belonging to NRs regardless and hence might generate meaningless outcome. **(4)** No web-server was provided by the existing methods or the web-server provided by them is currently not working, and hence their application value is quite limited.

The present study was initiated in an attempt to develop a new predictor, called **NR-2L**, by addressing the above four shortcomings. To extend the coverage scope for practical application and reduce the homology bias, new benchmark datasets were constructed and a two-level predictor was developed. The new datasets cover seven subfamilies in which none of proteins included has 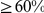 pairwise sequence identity to any other in a same subset. Included in the new benchmark datasets are also the non-NR sequences for training the predictor to identify non-NR proteins. To make the predictor more powerful, more sequence-derived features were utilized. These features are capable of capturing the key information through PseAAC [6] as well as various physicochemical properties of proteins. The resulting feature vectors are finally fed into a simple yet powerful classification engine, called fuzzy K nearest neighbor algorithm, to identify NRs and their subfamilies. For the convenience of users and dealing with the situation that some link might be occasionally down, the web-server for **NR-2L** has been established at both http://icpr.jci.edu.cn/bioinfo/NR2L and http://www.jci-bioinfo.cn/NR2L, by any of which Multi-Fasta protein sequences can be input and handled in a batch mode. Furthermore, the source code of the algorithm is available for educational purposes and basic researches by e-mailing a request to the corresponding author.

To develop an effective method for identifying protein attributes such as NRs and their subfamilies, the following five things are indispensable [9]: **(1)** construct a valid benchmark dataset to train and test the predictor; (**2)** formulate the protein samples with an effective mathematical expression that can truly reflect their intrinsic correlation with the attribute to be predicted; (**3**) introduce or develop a powerful algorithm (or engine) to operate the prediction; (**4**) properly perform cross-validation tests to objectively evaluate the anticipated accuracy of the predictor; (**5**) establish a user-friendly web-server for the predictor that is accessible to the public. Below, let us elaborate how to deal with these steps.

## Materials and Methods

### 1. Benchmark Datasets

Protein sequences were collected from the nuclear receptor data base (NucleaRDB release 5.0) at http://www.receptors.org/NR/, which is a part of a project devoted to build Molecular Class-Specific Information Systems (MCSIS) to provide, disseminate and harvest heterogeneous data [4]. The database have collected and harvested all the seven subfamilies of nuclear receptors marked with **(1)** NR1: thyroid hormone like (thyroid hormone, retinoic acid, RAR-related orphan receptor, peroxisome proliferator activated, vitamin D3-like), **(2**) NR2: HNF4-like (hepatocyte nuclear factor 4, retinoic acid X, tailless-like, COUP-TF-like, USP), (**3**) NR3: estrogen like (estrogen, estrogen-related, glucocorticoid-like), (**4**) NR4: nerve growth factor IB-like (NGFI-B-like), (**5**) NR5: fushi tarazu-F1 like (fushi tarazu-F1 like), (**6**) NR6: germ cell nuclear factor like (germ cell nuclear factor), and (**7**) NR0: knirps like (knirps, knirps-related, embryonic gonad protein, ODR7, trithorax) and DAX like (DAX, SHP). For detailed information about the database, refer to the NucleaRDB (http://www.receptors.org/NR/). Because the NucleaRDB has not provided the nuclear receptor sequences in FASTA format, we read Web content at the specified URL and extract all entries by the text-parsing method. The initial data set had 727 sequences belonging to seven subfamilies of nuclear receptors. To avoid any homology bias, a redundancy cutoff was imposed with the program CD-HIT to winnow those sequences which have 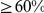 pairwise sequence identity to any other in a same subset except for the subfamily NR6 because it contained only 5 nuclear receptor protein sequences [10]. If the redundancy-cutoff operation was also executed on this class, the samples left would be too few to have any statistical significance. The final benchmark dataset, 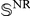, thus obtained contains 159 sequences classified into seven different subfamilies of NRs as shown in **Table 1**, where 500 non-NRs protein sequences were also collected in 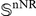
for training the predictor to identifying non-NRs. The protein sequences in 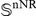
were randomly collected from the UniProt at http://www.uniprot.org/ according their annotations in the “Keyword” field, followed by undergoing the similar redundancy-cutoff operation to assure that none of the proteins in 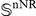
has 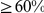 pairwise sequence identity to any other. The accession numbers and sequences for the benchmark dataset thus obtained for 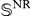 and 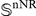 are given in Supporting Information S1. Meanwhile, for the purpose of demonstrating the practical application of the current predictor, the corresponding independent testing datasets 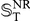 and 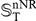 were also constructed (Table 1) in a way that none of proteins in the testing datasets occurs in 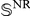 and 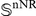. The accession numbers and sequences for the independent testing datasets 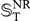 and 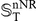
are given in Supporting Information S2. It is instructive to point out that the results derived from such independent datasets are only a kind of demonstration that cannot be used to objectively measure the accuracy of a predictor; the real criterion for measuring the accuracy of the predictor should be based on the jackknife test as will be elaborated later.

**Table 1:** Breakdown of the learning dataset 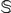 and testing dataset 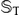.

Attribute	Training dataset 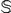			
	Set	Subfamily	Subset	Number
NR	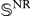	NR1	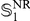	50
		NR2	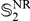	36
		NR3	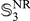	37
		NR4	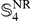	7
		NR5	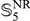	12
		NR6	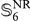	5
		NR0	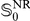	12
Non-NR	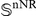	N/A	N/A	500
	Independent testing dataset 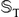			
NR	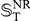	NR1	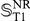	231
		NR2	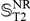	127
		NR3	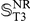	148
		NR4	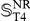	23
		NR5	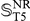	33
		NR6	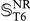	0
		NR0	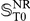	6
Non-NR	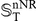	N/A	N/A	500

### 2. Sequence-Derived Features

As pointed out in [9], to develop a predictor for identifying protein attributes, one of the keys is to formulate the protein samples with an effective mathematical expression that can truly reflect their intrinsic correlation with the attribute to be predicted.

A protein sequence 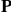 with *L* amino acid residues can be expressed as
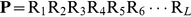
(1)


In order to capture as much useful information from a protein sequence as possible, we are to approach this problem from four different angles, followed by incorporating the feature elements thus obtained into the general form of PseAAC [9].

### 2.1 Amino Acid Composition (AAC)

As mentioned in the introduction, AAC was widely used to transform protein sequences into 20-D (dimensional) numerical vectors (see, e.g., [11], [12], [13], [14]). The AAC of a protein is defined as the normalized occurrence frequencies of 20 amino acids in that protein; i.e.,
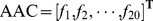
(2)where 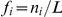 with each 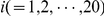 corresponding to one of the 20 native amino acid types, and 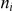 the number of type 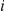 amino acids in the protein; while 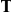
is the transpose operator.

### 2.2 Dipeptide Composition (DC)

**Figure 1:**
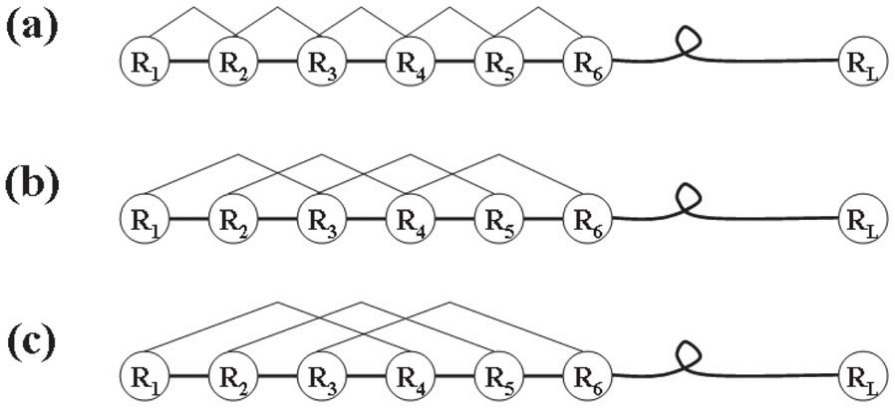
Schematic drawing to show dipeptides with different gaps along a protein chain. (a) The traditional (0-gap) dipeptide, (b) the 1-gap dipeptide, and (c) the 2-gaps dipeptide, where represents the amino acid residue at the sequence position 1, at position 2, and so forth. Adapted with permission from Chou [6].

Traditional dipeptide (amino acid pair) composition was used to capture the local-order information of a protein sequence, which gives a fixed pattern length of 400 (20×20) [15]. The fraction of each dipeptide was formulated as
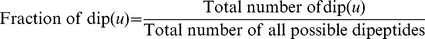
(3)where 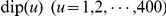
is the *u-*th dipeptide. In addition, to express the interaction of the amino acid for a pair with higher sequence gap than for the dipeptide pair (**Fig.1**), let us consider the following general equation
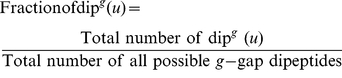
(4)where *g* = 0, 1, 2, or larger, and 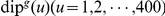 is the *u-*th dipeptide with *g* gap between the two residues. When 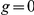, Eq.4 is reduced to Eq.3, the formulation for the conventional dipeptide. Accordingly, the dipeptide compositions with different gaps can be generally formulated as
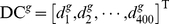
(5)where 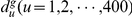
is the
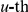
normalized occurrence frequency of the dipeptide of gap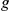. Since the couple effects among the local residues are usually stronger than those among the distant ones [16], [17], here let us just consider the cases of 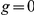 and 1 as denoted by DC(0) and DC(1) respectively. Thus, we obtain 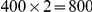
elements for using DC to formulate the protein sample, in which 400 elements are from DC(0) and 400 from DC(1).

### 2.3 Complexity Factor (CF)

A protein sequence is actually a symbolic sequence for which the complexity measure factor can be used to reflect its sequence feature or pattern and has been successfully used in some protein attribute prediction [18]. Among the known measures of complexity, the Lempel-Ziv (LZ) complexity [19] reflects the order that is retained in the sequence, and hence was adopted in this study.

The LZ complexity of a sequence 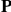 can be measured by the minimal number of steps required for its synthesis in a certain process. For each step only two operations were allowed in the process: either generating an additional symbol that ensures the uniqueness of each component 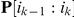, or copying the longest fragment from the part of a synthesized sequence. Its substring is expressed by
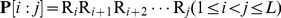
(6)The complexity measure factor, 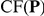, of a nonempty sequence synthesized according to the following procedure is defined by
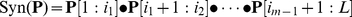
(7)Let us assume that 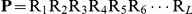 has been reconstructed by the program up to the residue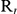, and 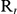 has been newly inserted. The string up to 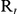 will be denoted by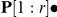, where the dot denotes that 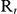 is newly inserted to check whether the rest of the string 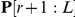 can be reconstructed by a simple copying. First, suppose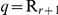, and see whether 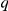 is reproducible from 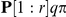, which means deleting the last character from the string 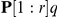. If the answer is “no,” then we insert 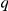 into the sequence followed by a dot. Thus, it could not be obtained by the copying operation. If the answer is “yes,” then no new symbol is needed and we can go on to proceed with 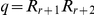 and repeat the same procedure. The LZ complexity is the number of dots (plus one if the string is not terminated by a dot). For example, for the sequence 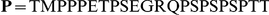, the LZ schema of synthesis generates the following components 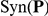
and the corresponding complexity 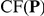:
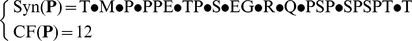
(8)


### 2.4 Fourier Spectrum Components (FSC)

Given a protein sequence 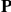, suppose 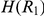
is the certain physicochemical property value of the 1st residue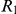, 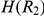 that of the 2nd residue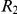, and so forth. In terms of these property values the protein sequence can be converted to a digit signal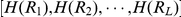, for which we implement the discrete Fourier transform, obtaining the frequency-domain values,
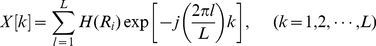
(9)where *j* represents the imaginary number. For each 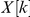 we can calculate its amplitude components 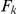 and phase components 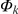


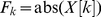
(10)

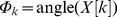
(11)Where **abs** gets the complex magnitude and **angle** gets the phase angle. Thus we can generate 2L discrete Fourier spectrum numbers as given below:
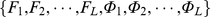
(12)The 2*L* Fourier spectrum numbers contain substantial information about the digit signal, and thereby can also be used to reflect characters of the sequence order of a protein. Furthermore, in the *L* phase components 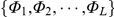, the high-frequency components are noisier and hence only the low-frequency components are more important. This is just like the case of protein internal motions where the low-frequency components are functionally more important [20]. For certain physicochemical property, accordingly, we only need to consider the 1^st^ 10 phase components as well as their corresponding amplitudes, i.e.
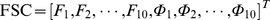
(13)As for the physicochemical property values, we adopted the hydrophobicity of each constituent amino acid, and its hydrophilicity and side-chain mass as done in [6]. These values can be obtained from the web-site at http://www.csbio.sjtu.edu.cn/bioinf/PseAAC/PseAAReadme.htm. Thus, we can obtain the 60 Fourier spectrum components.

### 2.5 Features Fusion into Pseudo Amino Acid Composition (PseAAC)

Finally, we obtained a total of 881 feature elements, of which 20 are from AAC, 800 from DC, 1 from CF, and 60 from FSC. Thus, according to the general formulation of PseAAC (cf. Eq.6 of [9]), a protein sample can be formulated as an 881-D vector given by
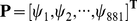
(14)where
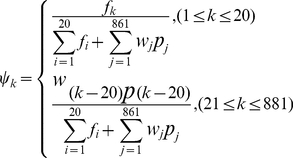
(15)where 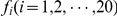 are the amino acid composition, 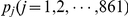 are the remaining 861 ( = 881-20) feature elements from dipeptide composition, complexity factor and Fourier spectrum components; 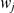 are the weight factors. In this study, the weight factor was set at 20 for all the feature elements from DC, 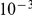
for those from CF, and 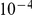
for those from FSC.

### 2.6 The Fuzzy K Nearest Neighbor (FKNN) Classifier

The *K*-nearest neighbor (*K*-NN) rule [21] is one of the simplest but quite powerful methods for performing nonparametric classification. The main idea of *K*-NN can be stated as following: Given a test sample with unknown label, its label is assigned according to the labels of its *K* nearest neighbors in the training set. Recently, the *K*-NN classifier has been successfully used to predict protein subcellular localization [22], membrane protein type, protease type, among many other protein attributes (see a long list of papers cited in a recent review [9]). For an intuitive illustration of how *K*-NN classifier works, see Fig.5 of [9].

Fuzzy *K*-NN classification method [23] is a special variation of the *K*-NN classification family. Instead of roughly assigning the label based on a voting from the *K* nearest neighbors, it attempts to estimate the membership values that indicate how much degree the query sample belongs to the classes concerned, Obviously, it is impossible for any characteristic description to contain complete information, which would make the classification ambiguous. In view of this, the fuzzy principle is very reasonable and particularly useful under such a circumstance.

Suppose 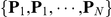 is a set of vectors representing 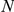 proteins in the training set which has been classified into 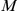 classes: 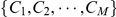, where 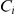 denotes the *i-*th class. Thus, for a query protein 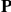, its fuzzy membership value for the *i-*th class is given by:
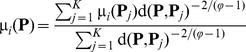
(16)where *K* is the number of the nearest neighbors counted; 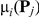
is the fuzzy membership value of the protein 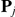 to the *i-*th class (it is set to 1 if the real label of 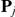 is 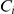; otherwise, 0); 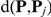 is the distance between the query protein 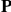 and its *j-*th nearest protein 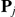 in the training dataset; and 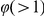 is the fuzzy coefficient for determining how heavily the distance is weighted when calculating each nearest neighbor's contribution to the membership value. Various metrics can be chosen for 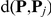, such as Euclidean distance, Hamming distance, and Mahalanobis distance [11], [24]. In this paper, the Euclidean metric was used. The values of 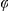 and 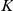 will be mentioned later. After calculating all the memberships for a query protein, it is assigned to the class with which it has the highest membership value; i.e., the predicted class for the query protein 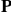 should be
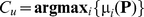
(17)where 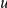 is the argument of 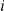 that maximizes 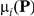.

**Figure 2:**
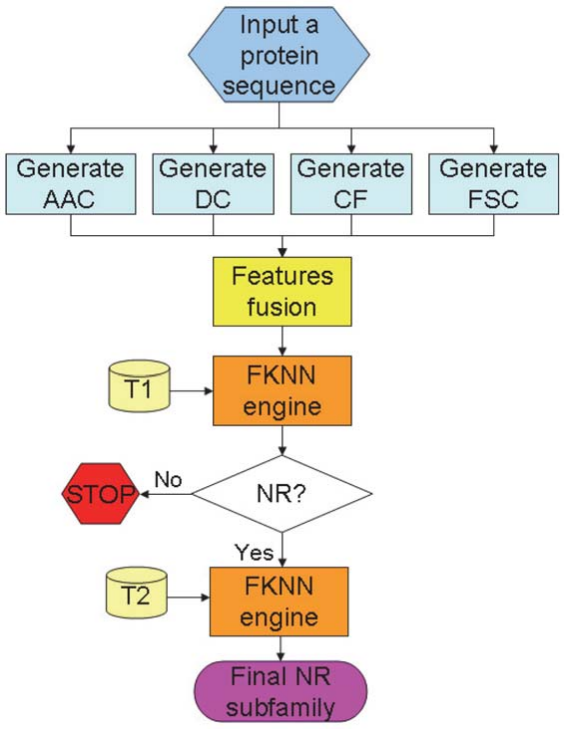
Flowchart to show the operation process of NR-2L. T1 represents the data taken from the Supporting Information S1 for training the 1st level prediction; T2 represents those from the Supporting Information S1 for training the 2nd level prediction. See the text for further explanation.

The predictor thus established is called **NR-2L**, where “2L” means the prediction consisting of two layers. The 1^st^ layer is to identify a query protein as NR or not; if it is a NR, the 2^nd^ layer will be automatically continued to further identify the NR among the seven subfamilies. To provide an intuitive picture, a flowchart to show the process of how the classifier works is given in **Fig.2**.

## Results and Discussion

In statistical prediction, the following three cross-validation methods are often used to examine a predictor for its effectiveness in practical application: independent dataset test, subsampling test, and jackknife test [25]. However, as elucidated and demonstrated by Eqs.28-32 of [9], among the three cross-validation methods, the jackknife test has the least arbitrary that can always yield a unique result for a given benchmark dataset, and hence has been increasingly and widely used by investigators to examine the accuracy of various predictors (see, e.g., [26], [27], [28], [29], [30], [31], [32]). Accordingly, the jackknife test was also adopted here to examine the quality of the present predictor.”

**Figure 3:**
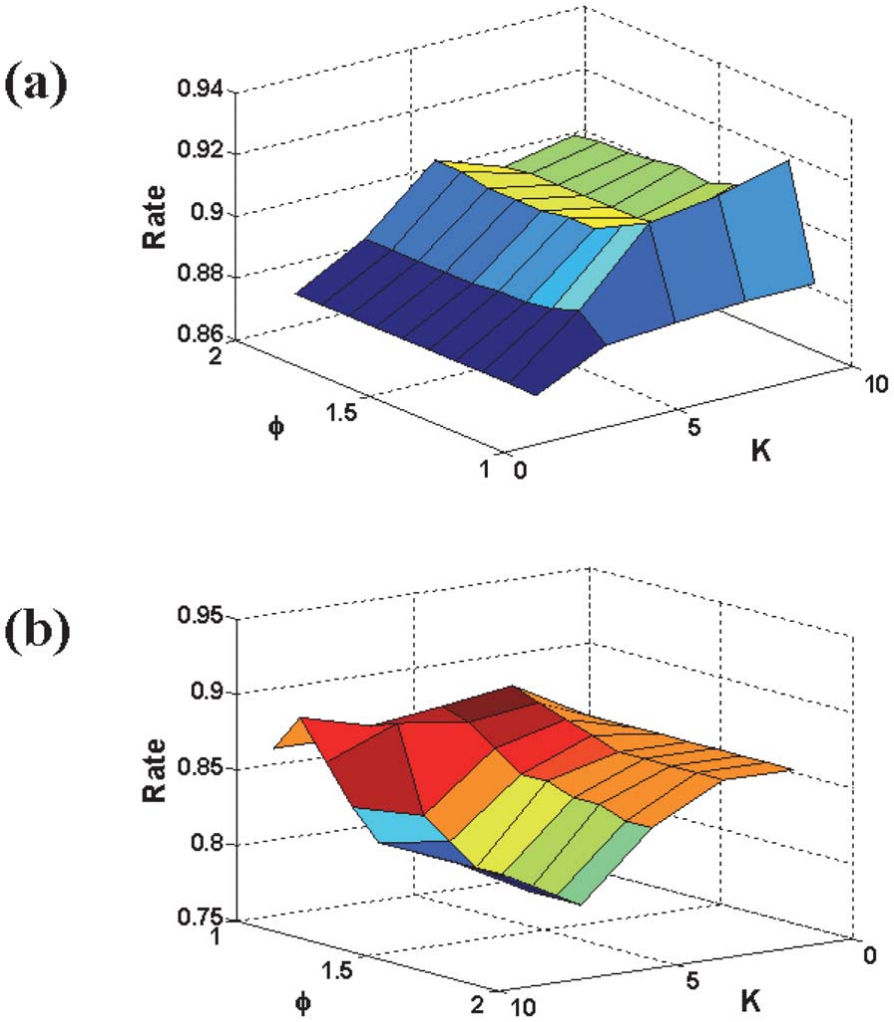
3D graph to show the jackknife success rates with the different parameters. (a) The results obtained by the 1st level prediction, and (b) the results obtained by the 2nd level prediction, where the parameters and are defined in Eq.16.

The values of parameter 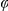 and 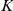 in **Eq.16** were determined by optimizing the overall jackknife success rate thru a 2-D search (**Fig.3**). It was found that the highest overall jackknife rate was obtained when 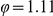 and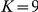 in the first level, while 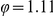 and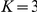 in the second level. Thus, with the optimized parameters, predictions were further made for proteins in the independent data set. The success rates obtained by the jackknife test and independent test are given in **Table 2** and **Table 3** for the first and second level, respectively. The prediction result by the jackknife test for each of the proteins in the benchmark dataset 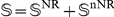 is given in Supporting Information S3, and the prediction result for each of the proteins in the independent test set 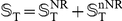
is given in Supporting Information S4.

**Table 2:** Prediction success rate and MCC index in identifying NR and non-NR by the jackknife test and independent dataset test.

Attribute	Jackknife test		Independent dataset test	
	ACC	MCC	ACC	MCC
NR	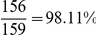	0.83	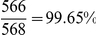	0.96
Non-NR	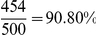	0.83	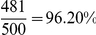	0.96
Overall	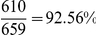		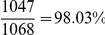	

**Table 3:** Prediction success rate and MCC index in identifying NR subfamilies by the jackknife test and independent test.

NR subfamily	Jackknife test		Independent dataset test	
	ACC	MCC	ACC	MCC
NR1	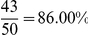	0.88	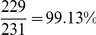	0.99
NR2	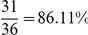	0.85	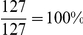	1.00
NR3	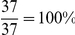	0.86	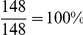	1.00
NR4	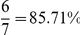	0.70	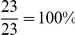	0.98
NR5	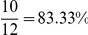	0.86	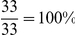	0.98
NR6	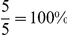	1	N/A	N/A
NR0	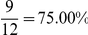	0.86	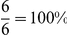	1.00
Overall	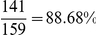		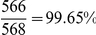	

As can be seen from the **Table 2** and **Table 3**, the success rates in identifying NRs and their subfamilies by both jackknife test and independent dataset test are very high, indicating that the **NR-2L** predictor is quite promising in generating reliable results for both basic research and drug development.

**Figure 4:**
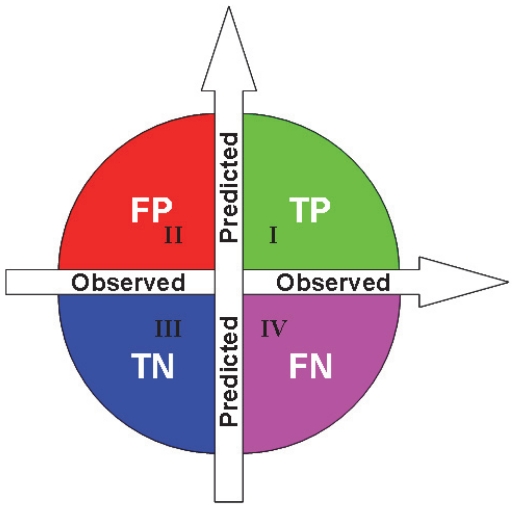
Distribution of predicted results in four quadrants. (I) TP, the true positive quadrant (green) for correct prediction of positive dataset, (II) FP, the false positive quadrant (red) for incorrect prediction of negative dataset; (III) TN, the true negative quadrant (blue) for correct prediction of negative dataset; and (IV) FN, the false negative quadrant (pink) for incorrect prediction of positive dataset.

To further evaluate the performance of **NR-2L**, the Matthew's correlation coefficient (MCC) index, another widely used criterion in statistics, was also used. The definition of MCC index is given by
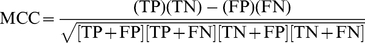
(18)where TP represents the true positive; TN, the true negative; FP, the false positive; and FN, the false negative (see Fig.4). The corresponding MCC values thus obtained are also given in Table 2 and Table 3, from which we can see that NR-2L not only possess high accuracy but also quite stable even though the subset sizes are very different.

Also, it is instructive to see the results in Table 4, where the success rates obtained by using different features are separately listed. It can be seen from the table that, among the five feature combinations, the contribution from AAC+DC(0) is the highest to the successful prediction.

**Table 4:** The jackknife success rates obtained in identifying the NR subfamilies by separately using different features on the benchmark dataset of Supporting Information S1.

Feature mode	AAC	AAC+DC(0)	AAC+DC(1)	AAC+CF	AAC+FSC
Success rate	66.67%	81.76%	80.50%	72.33%	73.58%

The results listed in Tables 2, 3, and 4 were obtained for the benchmark dataset with 60% cutoff threshold to exclude those protein sequences that have 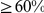
pairwise sequence identity to any other in a same subset. To show the impact of such threshold values to the predicted results, an extensive study was performed on the datasets constructed by following exactly the same procedures as described in the “Benchmark Datasets” section with, however, cutoff thresholds 40%, 50%, 60%, 70%, respectively. The results thus obtained are given in Table 5, from which we can see that the larger the cutoff threshold value, the less stringent the benchmark dataset, and the higher the overall success rate by the jackknife test, fully in consistency with the elucidation as elaborated in [9].

**Table 5:** The jackknifing success rates obtained in identifying NR subfamilies with different redundancy reduction cutoff thresholds^a^.

RedundancySubfamily	40%	50%	60%	70%
NR1	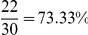	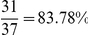	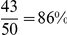	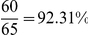
NR2	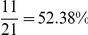	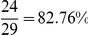	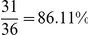	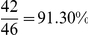
NR3	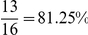	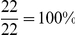	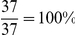	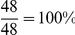
NR4	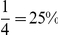	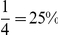	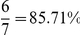	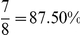
NR5	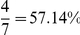	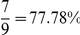	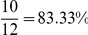	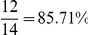
NR6	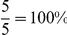	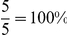	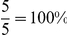	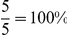
NR0	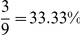	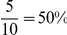	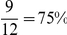	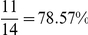
Overall	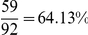	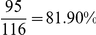	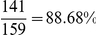	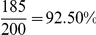

a We did not eliminate the redundancy of NR6 subfamily because it contained only 5 nuclear receptors. If the redundancy-cutoff operation was also executed on this class, the samples left would be too few to have any statistical significance.

Owing to the functional importance of NRs and the rapid increasing of their sequences, it is important and feasible to develop a reliable predictor for identifying NRs and their subfamilies based on the sequence information. The NR-2L predictor developed in this study can be used to address this kind of problems. The high success rates achieved by NR-2L have once again indicated that it is indeed an effective approach by fussing several different kinds of sequence-derived features into PseAAC to formulate protein samples for identifying their attributes. It is anticipated that NR-2L may become a useful tool in speeding up the pace of characterizing newly found nuclear receptor proteins or at least may play an important complementary role to the other methods in this regard. For the convenience of biologists and pharmacologists in using NR-2L, a user-friendly web-server for NR-2L has been established at http://icpr.jci.edu.cn/bioinfo/NR2L, by which users can easily obtain the desired results in a short period of time even for a large number of query protein sequences. Furthermore, as a backup, the web-server for NR-2L can also be accessed at http://www.jci-bioinfo.cn/NR2L in case the former link is down. All the program codes for NR-2L are available for non-commercial purpose upon request.

## Supporting Information

Supporting Information S1 The training dataset S contains 500 non-NR proteins and 159 NR proteins classified into the following 7 main subfamilies according to NucleaRDB (http://www.receptors.org/NR/): (1) NR1: thyroid hormone like; (2) NR2: HNF4-like; (3) NR3: estrogen like; (4) NR4: nerve growth factor IB-like; (5) NR5: fushi tarazu-F1 like; (6) NR6: germ cell nuclear factor like; and (7) NR0: knirps and DAX like. Both the accession numbers and sequences are given. None of the proteins included has ≥60% pairwise sequence identity to any other in the same subset except the NR6 subfamily.
(PDF)

Supporting Information S2 The independent testing dataset ST contains 500 non-NR proteins and 568 NR proteins classified into the following 7 main subfamilies according to NucleaRDB (http://www.receptors.org/NR/): (1) NR1:thyroid hormone like; (2) NR2: HNF4-like; (3) NR3: estrogen like; (4) NR4: nerve growth factor IB-like; (5) NR5: fushi tarazu-F1 like; (6) NR6: germ cell nuclear factor like; and (7) NR0: knirps and DAX like. Both the accession numbers and sequences are given. None of the proteins included here occurs in the training dataset S.
(PDF)

Supporting Information S3 List of the jackknifing results obtained by NR-2L on the 159 NRs and 500 non-NRs in the dataset S (cf. Supporting Information S1), and the corresponding observed results as annotated in NucleaRDB or UniProt.
(PDF)

Supporting Information S4 List of the results obtained by NR-2L on the 568 NRsand 500 non-NRs in the independent testing dataset ST (cf. Supporting Information S2), and the corresponding observed results as annotated in NucleaRDB or UniProt.
(PDF)
