# Supplementary material for: NR-2L: A Two-Level Predictor for Identifying Nuclear Receptor Subfamilies Based on Sequence-Derived Features
Source: PLoS One. 2011 Aug 15;6(8):e23505. doi: 10.1371/journal.pone.0023505 (PMC3156231; doi:10.1371/journal.pone.0023505)
Supplement: Supporting Information S1 — The training dataset S contains 500 non-NR proteins and 159 NR proteins classified into the following 7 main subfamilies according to NucleaRDB (http://www.receptors.org/NR/): (1) NR1: thyroid hormone like; (2) NR2: HNF4-like; (3) NR3: estrogen like; (4) NR4: nerve growth factor IB-like; (5) NR5: fushi tarazu-F1 like; (6) NR6: germ cell nuclear factor like; and (7) NR0: knirps and DAX like. Both the accession numbers and sequences are given. None of the proteins included has ≥60% pairwise sequence identity to any other in the same subset except the NR6 subfamily. (PDF) [file pone.0023505.s001.pdf]

Supporting Information S1. The training dataset $\mathbb{S}$ contains 500 non-NR proteins and 159 NR proteins classified into the following 7 main subfamilies according to NucleaRDB (http://www.receptors.org/NR/): (1) NR1: thyroid hormone like; (2) NR2: HNF4-like; (3) NR3: estrogen like; (4) NR4: nerve growth factor IB-like; (5) NR5: fushi tarazu-F1 like; (6) NR6: germ cell nuclear factor like; and (7) NR0: knirps and DAX like. Both the accession numbers and sequences are given. None of the proteins included has $\geq 60\%$ pairwise sequence identity to any other in the same subset except the NR6 subfamily.

---

1. 159 NR proteins $\mathbb{S}^{NR}$

(1) 50 NR1

>THB2_RAT
MCMDVRCPSICTAPGSRGLASACMERVCICKAGLHLDTKMNYCVPEVHEVCPAAGSNRYMQVTDYLAYLE
DSPAYSGCDVQAVPGSSIYLEQAWTLNQPYTCSYPGNLFKSKDSDLDMALSQYSQPAHLPEEKPFPQVRS
PPHSHKKGYIPSYLDKDELCVVCGDKATGYHYRCITCEGCKGFFRRTIQKSLHPSYSCKYEGKCIIDKVT
RNQCQECRFKKCIYVGMATDLVLDDSKRLAKRKLIEENREKRRREELQKSIGHKPEPTDEEWELIKTVTE
AHVATNAQGSHWKQKRKFLPEDIGQAPIVNAPEGGQVDLEAFSHFTKIITPAITRVVDFAKKLPMFCELP
CEDQIILLKGCCMEIMSLRAAVRYDPDSETLTLNGEMAVTRGQLKNGGLGVVSDAIFDLGMSLSSFNLDD
TEVALLQAVLLMSSDRPGLACVERIEKYQDSFLLAFEHYINYRKHHVTHFWPKLLMKVTDLRMIGACHAS
RFLHMKVECPTELFPPLFLEVFED

>Q64895_9RETR
METVIKVISSAPVVAMPVVIKTEGPAWTPLEPEDTRWLDGKHKRKSSQCLVKSSMSGYIPSCLDKDEQCV
VCGDKPTGYHYRCITCEGCKSFFRRTIQKNLHPTYSCTYDGCCVIDKITRNQCQLCRFKKCISVGMAMDL
VLDDSKRVAKRKLIEENRERRRKEEMIKSLQHRPSPSAEEWELIHVVTEAHRSTNAQGSHWKQRRKFLLE
DIGQSPMASMLDGDKVDLEAFTEFTKIITPAITRVVDFAKNLPMFSELPCEDQIILLKGCCMEIMSLRAA
VRYDPESETLTLSGEMAVKREQLKNGGLGVVSDAIFDLGKSLSAFNLDDTEVALLQAVLLMSSDRTGLIC
VDKIEKCQESYLLAFEHYINYRKHNIPHFWSKLLMKVADLRMIGAYHASRFLHMKVECPTELSPQEVGPD
HCMKCAHFIDGPHCVKACPAGVLGENDTLVWKYADANAVCQLCHPNCTRGCKGPGLEGCPNGSKTPSIAA
GVVGGLLCLVVVGLGIGLYLRRRHIVRKRTLRRLLQERELVEPLTPSGEAPNQAHLRILKETEFKKVKVL
GFGAFGTVYKGLWIPEGEKVTIPVAIKELREATSPKANKEILDEAYVMASVDNPHVCRLLGICLTSTVQL
ITQLMPYGCLLDYIREHKDNIGSQYLLNWCVQIAKGMNYLEERHMVHRDLAARNVLVKTPQHVKITDFGL
AKQLGADEKEYHAEGGKVPIKWMALESILHRIYTHQSDVWSYGVTVWELMTFGSKPYDGIPASEISSVLE
KGERLPQPPICTIDVYMIMVKCWMSGADSRPKFRELIAEFSKMARDPPRYLVIQGDERMHLPSPTDSKFY
RTLMEEEDMEDIVDADEYLVPHQGFFNSPSTSRTPLLSSLSATSNNSATKCIDRNGGHPVREDGFLPAPE
YVNQLMPKKPSTAMVQNQIYNYISLTAISKLPMDSRYQNSHSTAVDNPEYLE

>Q5RZV7_PSEAM
MSEPAENCSPSWKDEAIQNGYIPSYLDKDELCVVCGDKATGYHYRCITCEGCKGFFRRTIQKNLNPTYAC
KYEGKCVIDKVTRNQCQECRFKKCIVVGMATDLVLNDSKRLAKRKLIEENRERRRKEELQKTVWDRLEPT
QEEWDVIRMVTDAHMSTNAQGNHWKQKRKFLVEEAMLLNEITCNLFYTSDQSAAGVKEDKPEEIGQASMA
NTPEGNKVDIEAFSQFTKIITPAITRVVDFAKKLPMFCELPCEDQIILLKGCCMEIMSLRAAVRYDPESE
TLTLNGEMAVTRGQLKNGGLGVVSDAIFDLGVSLSSFNLDDSEVALLQAVILLSSDRPGLSSVERIERCQ

EEFLLAFEHYINYRKHKLAHFWPKLLMKVTDLRMIGACHASRFLHMKVECPTELFPPLFLEVFED

>RRG_NOTVI
MMKFSDTASCRDGGERPEEEGKGAGGRSKLRMGKEEFTGSVGKEEAAAVASMSSSKDRICSTSTQLSQLH
GFPPSMYPFAFSSNMRGSPPFDLTNGGAYFRSFPTDLPKEMASLSVETQSTSSEEMVPSSPSPPPPPRVY
KPCFVCNDKSSGYHYGVSSCEGCKGFFRRSIQKNMVYTCHRDKNCQINKVTRNRCQYCRLQKCFEVGMSK
EAVRNDRNKKKKEIKEEVVTDSYEMPPEMEALIQKVSKAHQETFPSLCQLGKYTTNSSADHRVQLDLGLW
HKFSELATKCIIKIVEFAKRLPGFATLTIADQITLLKAACLDILMLRICTRYTPEQDTMTFSDGLTLNRT
QMHNAGFGPLTDLVFAFAEQLLPLEMDDTETGLLSAICLICGDRMDLEEPEKVDKLQEPLLEALKIYARR
RRPNKPYMFPRMLMKITDLRGISTKGAERAITLKMEIPGPMPPLIREMLENPEAFEDDASPPPKSEQKPI
KVEEKPGEKTSTKDP

>Q15156_HUMAN
MEPAPARSPRPQQDPARPQEPTMPPPETPSEGRQPSPSPSPTERAPASEEEFQFLRCQQCQAEAKCPKLL
PCLHTLCSGCLEASGMQCPICQAPWPLGADTPALDNVFFESLQRRLSVYRQIVDAQAVCTRCKESADFWC
FECEQLLCAKCFEAHQWFLKHEARPLAELRNQSVREFLDGTRKTNNIFCSNPNHRTPTLTSIYCRGCSKP
LCCSCALLDSSHSELKCDISAEIQQRQEELDAMTQALQEQDSAFGAVHAQMHAAVGQLGRARAETEELIR
ERVRQVVAHVRAQERELLEAVDARYQRDYEEMASRLGRLDAVLQRIRTGSALVQRMKCYASDQEVLDMHG
FLRQALCRLRQEEPQSLQAAVRTDGFDEFKVRLQDLSSCITQGKAIETQSSSSEEIVPSPPSPPPLPRIY
KPCFVCQDKSSGYHYGVSACEGCKGFFRRSIQKNMVYTCHRDKNCIINKVTRNRCQYCRLQKCFEVGMSK
ESVRNDRNKKKKEVPKPECSESYTLTPEVGELIEKVRKAHQETFPALCQLGKYTTNNSSEQRVSLDIDLW
DKFSELSTKCIIKTVEFAKQLPGFTTLTIADQITLLKAACLDILILRICTRYTPEQDTMTFSDGLTLNRT
QMHNAGFGPLTDLVFAFANQLLPLEMDDAETGLLSAICLICGDRQDLEQPDRVDMLQEPLLEALKVYVRK
RRPSRPHMFPKMLMKITDLRSISAKGAERVITLKMEIPGSMPPLIQEMLENSEGLDTLSGQPGGGGRDGG
GLAPPPGSCSPSLSPSSNRSSPATHSP

>Q86WD1_HUMAN
MEVLESGEQGVLQWDRKLSELSEPGDGEALMYHTHFSELLDEFSQNVLGQLLNDPFLSEKSVSMEVEPSP
TSPAPLIQAEHSYSLCEEPRAQSPFTHITTSDSFNDEMTMVDTEMPFWPTNFGISSVDLSVMEDHSHSFD
IKPFTTVDFSSISTPHYEDIPFTRTDPVVADYKYDLKLQEYQSAIKVEPASPPYYSEKTQLYNKPHEEPS
NSLMAIECRVCGDKASGFHYGVHACEGCKGFFRRTIRLKLIYDRCDLNCRIHKKSRNKCQYCRFQKCLAV
GMSHNAIRFGRMPQAEKEKLLAEISSDIDQLNPESADLRALAKHLYDSYIKSFPLTKAKARAILTGKTTD
KSPFVIYDMNSLMMGEDKIKFKHITPLQEQSKEVAIRIFQGCQFRSVEAVQEITEYAKSIPGFVNLDLND
QVTLLKYGVHEIIYTMLASLMNKDGVLISEGQGFMTREFLKSLRKPFGDFMEPKFEFAVKFNALELDDSD
LAIFIAVIILSGDRPGLLNVKPIEDIQDNLLQALELQLKLNHPESSQLFAKLLQKMTDLRQIVTEHVQLL
QVIKKTETDMSLHPLLQEIYKDLY

>Q658K5_HUMAN
MYESVEVGGPTPNPFLVVDFYNQNRACLLPEKGLPAPGPYSTPLRTPLWNGSNHSIETQSSSSEEIVPSP
PSPPPLPRIYKPCFVCQDKSSGYHYGVSACEGCKGFFRRSIQKNMVYTCHRDKNCIINKVTRNRCQYCRL
QKCFEVGMSKESVRNDRNKKKKEVPKPECSESYTLTPEVGELIEKCAKRTRKPSLPSASWANTLRTTAQN
NVSLWTLTSGTSSVNSPTKCIIKTVEFAKQLPGFTTLTIADKITLLKAACLDILILRICTRYTPEQDTMT
FSDGLTLNRTQMHNAGFGPLTDLVFAFANQLLPLEMDDAETGLLSAICLICGDRQDLEQPDRVDMLQEPL
LEALKVYVRKRRPSRPHMFPKMLMKITDLRSISAKGAERVITLKMEIPGSMPPLIQEMLENSEGLDTLSG

QPGGGGRDGGGLAPRQAAVAPASAPAPTEAARPPTPRDRPRHMDTALALRPGFSLPFYRPCDPAPALPPP
ALPGSTGDLPWGTGREEAATPWTEAWALSGLPAPTAWADVRGRGQELSEAPGPGSQDGSWGPRVHQDTPL
PSSPHLHHQQTPGLGSPILRTHKPLLPSWGTSTSPLPRLVTEGVGQGRGVPPVHTLPYQPQVLILAGFVF
ILIFLF

>NR1D2_HUMAN
MEVNAGGVIAYISSSSSASSPASCHSEGSENSFQSSSSSVPSSPNSSNSDTNGNPKNGDLANIEGILKND
RIDCSMKTSKSSAPGMTKSHSGVTKFSGMVLLCKVCGDVASGFHYGVHACEGCKGFFRRSIQQNIQYKKC
LKNENCSIMRMNRNRCQQCRFKKCLSVGMSRDAVRFGRIPKREKQRMLIEMQSAMKTMMNSQFSGHLQND
TLVEHHEQTALPAQEQLRPKPQLEQENIKSSSPPSSDFAKEEVIGMVTRAHKDTFMYNQEQQENSAESMQ
PQRGERIPKNMEQYNLNHDHCGNGLSSHFPCSESQQHLNGQFKGRNIMHYPNGHAICIANGHCMNFSNAY
TQRVCDRVPIDGFSQNENKNSYLCNTGGRMHLVCPMSKSPYVDPHKSGHEIWEEFSMSFTPAVKEVVEFA
KRIPGFRDLSQHDQVNLLKAGTFEVLMVRFASLFDAKERTVTFLSGKKYSVDDLHSMGAGDLLNSMFEFS
EKLNALQLSDEEMSLFTAVVLVSADRSGIENVNSVEALQETLIRALRTLIMKNHPNEASIFTKLLLKLPD
LRSLNNMHSEELLAFKVHP

>Q6IBU6_HUMAN
MSSPTTSSLDTPLPGNGPPQPGAPSSSPTVKEEGPEPWPGGPDPDVPGTDEASSACSTDWVIPDPEEEPE
RKRKKGPAPKMLGHELCRVCGDKASGFHYNVLSCEGCKGFFRRSVVRGGARRYACRGGGTCQMDAFMRRK
CQQCRLRKCKEAGMREQCVLSEEQIRKKKIRKQQQQESQSQSQSPVGPQGSSSSASGPGASPGGSEAGSQ
GSGEGEGVQLTAAQELMIQQLVAAQLQCNKRSFSDQPKVTPWPLGADPQSRDARQQRFAHFTELAIISVQ
EIVDFAKQVPGFLQLGREDQIALLKASTIEIMLLETARRYNHETECITFLKDFTYSKDDFHRAGLQVEFI
NPIFEFSRAMRRLGLDDAEYALLIAINIFSADRPNVQEPGRVEALQQPYVEALLSYTRIKRPQDQLRFPR
MLMKLVSLRTLSSVHSEQVFALRLQDKKLPPLLSEIWDVHD

>Q922A5_MOUSE
MTTLDSNNNTGGVITYIGSSGSSPSRTSPESLYSDSSNGSFQSLTQGCPTYFPPSPTGSLTQDPARSFGS
APPSLSDDSSPSSTSSSSSSSSSSFYNGSPPGSLQVAMEDSSRVSPSKGTSNITKLNGMVLLCKVCGDVA
SGFHYGVHACEGCKGFFRRSIQQNIQYKRCLKNENCSIVRINRNRCQQCRFKKCLSVGMSRDAVRFGRIP
KREKQRMLAEMQSAMNLANNQLSSLCPLETSPTPHPTSGSMGPSPPPAPAPTPLVGFSQFPQQLTPPRSP
SPEPTMEDVISQVARAHREIFTYAHDKLGTSPGNFNANHASGSPSATTPHRWESQGCPSAPNDNNLLAAQ
RHNEALNGLRQGPSSYPPTWPSGPTHHSCHQPNSNGHRLCPTHVYSAPEGEAPANSLRQGNTKNVLLACP
MNMYPHGRSGRTVQEIWEDFSMSFTPAVREVVEFAKHIPGFRDLSQHDQVTLLKAGTFEVLMVRFASLFN
VKDQTVMFLSRTTYSLQELGAMGMGDLLNAMFDFSEKLNSLALTEEELGLFTAVVLVSADRSGMENSASV
EQLQETLLRALRALVLKNRPSETSRFTKLLLKLPDLRTLNNMHSEKLLSFRVDAQ

>Q6VA69_AEDAE
MYRLNIVSTNPSGSVQQQQQAQGQQVISSVVRPQQQQPPPQLALVQTGGSGGTTTTIIGLTSLNALNATT
ITGLVAGAAGSSTSAIAAAGASNSGSGPSTATTKHILKAATTNNNISIVKIVDDIMLKAVKVEPLPMDTG
GGGGGVSMIPSSATTSGGVTVTAIPASVAPMPPVAAGTNVSSNGSVTVYASGKRRLESNEEWISSPSPGS
VPGSAPPLSPSPGSQSTTYTTTMSNGYSSPMSTGSYDPYSPNGKMGREDLSPSSSLNGYTDGSDAKKQKK
GPTPRQQEELCLVCGDRESGYHYNALTCEGCKGFFRRSVTKNAVYCCKFGHACEMDMYMRRKCQECRLKK
CLAVGMRPECVVPENQCAIKRKEKKAQKEKDKVQTNATVSTTNSTYRSEILPILMKCDPPPHQAIPLLPE
KLLQENRLRNIPLLTANQMAVIYKLIWYQDGYEQPSEEDLKRIMIGSPNEEEDQHDVHFRHITEITILTV

QLIVEFAKGLPAFTKIPQEDQITLLKACSSEVMMLRMARRYDAATDSILFANNRSYTRDSYRMAGMADTI
EDLLHFCRQMFSLTVDNVEYALLTAIVIFSDRPGLEQAELVEHIQSYYIDTLRIYILNRHAGDPKCSVIF
AKLLSILTELRTLGNQNSEMCFSLKLKNRKLPRFLEEIWDVQDIPPSMQAQMHSHGTQSSSSSSSSSSS
SNGSSNGNSSSNSNSSQHGPHPHPHGQQLTPNQQQHQQQHSQLQQVHANGSGSGGGSNNNSSSGGVVPGL
GMLDQV

>Q6PAY0_XENLA
MSTIMVDTNSELCILTPLDEDDLESPLSGEFLQDIVDIQDITQTIGDDGSTPFGASEHQFLGNSPGSIGS
VSTDLTDTLSPASSPASITFPAASGSAEDAACKSLNLECRVCSDKASGFHYGVHACEGCKGFFRRTIRLK
LVYDRCERMCKIQKKNRNKCQYCRFEKCLNVGMSHNAIRFGRMPRSEKAKLKAEVLMCDQDVKDTQMADL
LSLARLIYDAYLKNFNMNKVKARAILTGKASNPPFVIHDMETLCMAEKTLVAKLVANGIQNKEAEVRIFH
CCQCTSVETVTELTEFAKSIPGFTELDLNDQVTLLKYGVYEAMFAMLASVMNKDGMLVAYGNGFITREFL
KSLRKPIGDMMEPKFEFAMKFNALELDDSDLSLFVAALICCGDRPGLVNIPSIEKMQESIVHVLKLHLQS
NHPDDSFLFPKLLQKMADLRQLVTEHAQLVQTIKKTETDAALHPLLQEIYRDMY

>NR1H3_HUMAN
MSLWLGAPVPDIPPDSAVELWKPGAQDASSQAQGGSSCILREEARMPHSAGGTAGVGLEAAEPTALLTRA
EPPSEPTEIRPQKRKKGPAPKMLGNELCSVCGDKASGFHYNVLSCEGCKGFFRRSVIKGAHYICHSGGHC
PMDTYMRRKCQECRLRKCRQAGMREECVLSEEQIRLKKLKRQEEEQAHATSLPPRASSPPQILPQLSPEQ
LGMIEKLVAAQQQCNRRSFSDRLRVTPWPMAPDPHSREARQQRFAHFTELAIVSVQEIVDFAKQLPGFLQ
LSREDQIALLKTSAIEVMLLETSRRYNPGSESITFLKDFSYNREDFAKAGLQVEFINPIFEFSRAMNELQ
LNDAEFALLIAISIFSADRPNVQDQLQVERLQHTYVEALHAYVSIHHPHDRLMFPRMLMKLVSLRTLSSV
HSEQVFALRLQDKKLPPLLSEIWDVHE

>Q9PW01_PLEPL
MVDTQQLLFWPVGFSLSAVDLSELDDSSHSLDMKHLATLDYTSISSASVPSSLSPQLMSSISSVGMAYDP
SPPQSEEHLTNMDYTNMHSYRTEPNVHNSIKMEPESPPQYSDSPVFSKLQDDTTAASLNIECRVCGDKAS
GFHYGVHACEGCKGFFRRTIRLKLVYDHCDLHCRIHKKSRNKCQYCRFQKCLNVGMSHNAIRFGRMPQAE
KEKLLAEFSSDMEHMHPEAADLRALARHLYEAYLKYFPLTKAKARAILSGKTGDNAPFVIHDIKSLMEGE
QFINCRQMPIQEQQQASVLTATHRGLTEHHMGSDYGVWGTTSISGQEPQNALELRFFQSCQSRSAEAVRE
VTEFAKSIPGFTDLDLNDQVTLLKYGVIEVLIIMMSPLMNKDGTLISYGQIFMTREFLKSLRKPFCQMME
PKFEFSVKFNTLELDDSDMALFLVVIILSGDRPGLLNVKPIEQLQETVLHSLELQLKLNHPDSLQLFAKL
LQKMTDLRQIVTDHVHLIQLLKKTEVDMCLHPLLQEIMKDLY

>Q9GPH1_CALVI
MMKRRWSNNGGFAALKMLEESSSEVTSSSNGLVLSSDINMSPSSLDSPVYGDQDMWLCNDSASYNTNSHQ
HSVITSLQGCTSSLPAQTTIIPLSALPPNSNNNNSLNGGQNQNYTNGGGSLNTNLAGNGSNNTGGGGGGV
PGMTSLNGLGGGGGIVGGGVNIQVNNHSHIHNHHNSNSNHSNSSSHHTNGHMGVGGGVRRRLDAWWFVLV
NINGPNIVSNAQQLSSLQASQNGQVIHANIGIHSIISNGLNHHHHHHMDNSSMMHHTPRSESANSISSGR
DDLSPSSSLNGFSTSDASDVKKIKKGPAPRLQEELCLVCGDRASGYHYNALTCEGCKGFFRRSVTKNAVY
CCKFGHACEMDMYMRRKCQECRLRKCLAVGMRPECVVPENQCAMKRREKKAQKEKDKIQTSVCATEIKKE
ILDLMTCEPPSHPTCPLLPEDILAKCQARNIPPSSYNQLAVIYKLIWYQDGYEQPSEEDLKRIMSSPDEN
ESQHDVSFRHITEITILTVQLIVEFAKGLPAFTKIPQEDQITLLKACSSEVMMLRMARRYDHNSDSIFFA
NNRSYTRDSYKMAGMADNIEDLLHFCRQMYSMKVDNVEYALLTAIVIFSDRPGLEEAELVEAIQSYYIDT

LRIYILNRHCGDPMSLVFFAKLLSILTELRTLGNQNAEMCFSLKLKNRKLPKFLEEIWDVHAIPPSVQSH
IQATQAEKAGPGSPGYHFGSVAHGHFSGRHLIVLHKYLDGDIILIISYQPSAVSLSAASTPNGGTGVDYV
GTDMSMSLVQSDNA

>O97095_LOCMI
MELFRGADGALPSASASASASASGAPAASPLAVSVPLALPLPGHASPASAADALVVKTEPREAGALFAAI
SSPGQGPGPAKRARLDSDWLSSPGSNAAPSPPPHHLFGAAASASAGAPAALPNGYASPLSSGGSYDPYSP
GGKIGREDLSPLSSLNGYSADSCDAKKKKGAAPRQQEELCLVCGDRASGYHYNALTCEGCKGFFRRSITK
NAVYQCKYGNNCEIDMYMRRKCQECRLKKCLTVGMRPECVVPEYQCAVKRKEKKAQKDKDKPNSTTNGSP
EVMMLKDIDAKVEPERPLSNGIKPVSPEQEELIHRLVYFQNEYESPSEEDLRRVTSQPTEGEDQSDVRFR
HITEITILTVQLIVEFAKRLPGFDKLLREDQIALLKACSSEVMMFRMARRYDVNSDSILFANNQPYTKDS
YNLAGMGETIEDMLRFCRQMYAMKVDNAEYALLTAIVIFSERPSLVEGWKVEKIQEIYLEALKAYVDNRR
RPKSGTIFAKLLSVLTELRTLGNQNSEMCFSLKLKNKKLPPFLAEIWDVIP

>Q7T030_PLEPL
MEGVQPTVSEQHDRVNGYCEPKSPQDAAEVRWTSPKRESGGSDSCGGASVSELTDEQELKARESEDEEDN
EKKKRVPVSKGLKGDQKSKEKEHLDQEKNNHSKQKSSGASSYTDLSHTSSPSLSEQLRLGREDSTGAGIS
VECKVCGDKASGFHYGVHACEGCKGFFRRTVRMKLEYDRCERSCKILKKNRNKCQYCRFQKCLSLGMSHD
AIRYGRMPEAERKKLVAGLLAEELTVSKPGGSDLKTLAKQVNAAYLKNLVMTKKRARSILTGKTSSTSPF
VIYDVDTLWKAESGLVWSQLLAGAPLTKEIGVHVFYRCQCTTVETVRELTEFAKSIPGFQDLYLNDQVTL
LKYGVHEAIFAMLPSLMNKDGLLVANGKGFVTREFLRSLRKPFSEIMEPKFEFAVKFNALELDDSDLALF
VAAIILCGDRPGLMNVKQVEQSQDNILQALDLHLQANHSDSLYLFPKLLQKMADLRQLVTENALLVQKIK
KTESETSLHPLLQEIYKDMY

>ECR_DROME
MKRRWSNNGGFMRLPEESSSEVTSSSNGLVLPSGVNMSPSSLDSHDYCDQDLWLCGNESGSFGGSNGHGL
SQQQQSVITLAMHGCSSTLPAQTTIIPINGNANGNGGSTNGQYVPGATNLGALANGMLNGGFNGMQQQIQ
NGHGLINSTTPSTPTTPLHLQQNLGGAGGGGIGGMGILHHANGTPNGLIGVVGGGGGVGLGVGGGGVGGL
GMQHTPRSDSVNSISSGRDDLSPSSSLNGYSANESCDAKKSKKGPAPRVQEELCLVCGDRASGYHYNALT
CEGCKGFFRRSVTKSAVYCCKFGRACEMDMYMRRKCQECRLKKCLAVGMRPECVVPENQCAMKRREKKAQ
KEKDKMTTSPSSQHGGNGSLASGGGQDFVKKEILDLMTCEPPQHATIPLLPDEILAKCQARNIPSLTYNQ
LAVIYKLIWYQDGYEQPSEEDLRRIMSQPDENESQTDVSFRHITEITILTVQLIVEFAKGLPAFTKIPQE
DQITLLKACSSEVMMLRMARRYDHSSDSIFFANNRSYTRDSYKMAGMADNIEDLLHFCRQMFSMKVDNVE
YALLTAIVIFSDRPGLEKAQLVEAIQSYYIDTLRIYILNRHCGDSMSLVFYAKLLSILTELRTLGNQNAE
MCFSLKLKNRKLPKFLEEIWDVHAIPPSVQSHLQITQEENERLERAERMRASVGGAITAGIDCDSASTSA
AAAAAQHQPQPQPQPQPSSLTQNDSQHQTQPQLQPQLPPQLQGQLQPQLQPQLQTQLQPQIQPQPQLLPV
SAPVPASVTAPGSLSAVSTSSEYMGGSAAIGPITPATTSSITAAVTASSTTSAVPMGNGVGVGVGVGGNV
SMYANAQTAMALMGVALHSHQEQLIGGVAVKSEHSTTA

>Q90WP6_SALSA
MVDTRRAAWSLLSFGLGTLDLVEMDNKMNSFDMKTLSTLDYPYLPSLEYSHNSPHHHHSPDRSHSCNHSP
DRSHSFNHSPDRSHSFNHSPDRNHSFNHSPDRSHSFNHSPDRSHSYNDTYSVYQGSVNDKPLSPSQSSDC
SIVSLSRPRPHSNPPTYTDASSLLNIDCRVCGDKASGFHYGVHVCEGCKGFFRRTVRLKLVYDHCDLHCR
IHKKSRNKCQYCRFQKCLLVGMSHDAIRFGRMPQVEREKLLQAEFMDVEPRNPESADLRALSRQLCLSYH

RHFPLTKSKAKAILSGKTHGNSPFVIHDMKSLTAGQYFINCRQLPVLERQRSVLPPEEPAEELELSVFRR
IQFRSAEAVQEVTEFTKSIPGFTELDMNDQVILLKYGVIEVMTTMLAPLMNKDGTLFAYGQIFMTREFLK
SLRKPFCEMMEPKFEFAAKFNLLELDDSDMALFFAVIILSGDRPGLVNVKPIEDLQETVLQALELQLKTI
HPDCPQLFAKLLQKMTDLRQLVANHVRHIHLLKKQELQMCLHPLLQEIMRDLY

>Q6GMI3_BRARE
MESTKAGGVIAYISSSSSASSPESCHSDSSNSSFQSCSPPHTGQGQGETLHVASHSRPPRHGGGKARSLS
STKSGITKINGLVLLCKVCGDVASGFHYGVHACEGCKGFFRRSIQQNIQYKKCLKNESCPIMRINRNRCQ
QCRFKKCLLVGMSRDAVRFGRIPKREKQRMLLEMQNAMNNMMNNSHGNQSLNNQTSAADGTSEDGTSEDS
TSGPSSRSPSSSPRSNRSESSPDPELQIAMDTSSSSDSSSATDSGEEEVIGTVTRAHQETFMYNQEQGSM
ASEALNNYSHCTEKTQEVLNHQNNVSSVSEQNPQSSCGPQGPEDSGQDYSTLSSCPVRLSNNSSRGPSLQ
TLTFRAHNDHETHANGSYIVPTFPRANRMHLVCPMNTSPYVDPQKSGHSIWEEFSMSFTPAVREVVEFAK
RIPGFKDLSQHDQVSLLKAGTFEVLVVRFTSLFDVKERKVTFLSGRKYSVDALRSLGAGDLLNSMFDFTE
KLQALNLSEEEMSLFTAVVLVSADRSGLENVNSVEALQETLIRALRSLITKNHPNEIAIFTKLLLKLPDL
RSLNNMHSEQLLAFKVHP

>Q8WSA1_BOMMO
MQCYPKLSPKREPPEGLYEIEMLPGARRLELPAPPGKEFRAPVLLAGPSLAPTHSVIQCMRPPPPPPPPP
PPRLLKPPSFEEPSSSIPDLEFDGTTVLCRVCGDKASGFHYGVHSCEGCKGFFRRSIQQKIQYRPCTKNQ
QCSILRINRNRCQYCRLKKCIAVGMSRDAVRFGRVPKREKARILAAMQQSSSSRAHEQAAAAELDDAPRL
LARVVRAHLDTCEFTRDRVASMRARARDCPTYSQPTLACPLNPAPELQSEKEFSQRFAHVIRGVIDFAGL
IPGFQLLTQDDKFTLLKSGLFDALFVRLICMFDAPLNSIICLNGQLMKRDSIQSGANARFLVDSTFKFAE
RMNSMNLTDAEIGLFCAIVLITPDRPGLRNIELVERMHSRLKACLQTVIAQNRPERPGFLRELMDTLPDL
RTLSTLHTEKLVVFRTEHKELLRQQMWNEEEGVSWADSVVEESARSPIGSVSSSESGEVPSDCGTPLLAA
TLAGRRRLDSRGSVDEEALGVAHLAHNGLTVTPVRPPPRYRKLDSPTDSGIESGNEKHERIIGPGSGCSS
PRSSLEEHTEDRRPTAPADDMPVLKRVLQAPPLYGGTSTLMDETYKPHKKFRAMRRDTGEAEARPVQPTP
SPQPLHPHPASPAHPAHSPRPPRISLSSTHSVLAKSLMEGPRMTPEQLKRTDMIQQYMRRNEAGSSVEGC
TLRTGGLLTCYRGASPAPPPVLALQVDVTDAPLNLSKKSPSPPRSYMPQMLEA

>Q7T031_PLEPL
MAGDLYSPPSPLGESLLGSPLCGDLMEDYCDISQSMEDSALGFDIPEYQSTGSGSESSTALDTLTPASSP
LSGVCGAAPGTEESLNLECRVCSDKASGFHYGVHACEGCKGFFRRTIRLKLEYDKCERSCKILKKNRNKC
QYCRFHKCLSVGMSHNAIRFGRMPQAEKLKLKAESNMVEKEAESPMLADHKILVRQIHDAYMKNFNMSKA
KAKLILTGKTSKPPFIIHDMETFQLAERTLAAHMVNGDQAEPECGLQSRAVALDAECGELEQREAEARLF
FCCQSTSVETVTELTEFAKAVPGFQSLDLNDQVTLLKYGVYEALFTLLASCMNKDGLLVARGGGFITREF
LKSLRRPFSDMMEPKFQFATRFNSLELDDSDLALFVAAIICCGDRPGLVDVPLVEQLQESIVQALQLHLL
ANHPDITFLFPRLLQKLADLRELVTEHAQLVQEIKTTEDAALHPLLQEIYRDMY

>O44338_9ACAR
MVDDALQLGSGAEQQLDAHLLELAAVALHGLARIFVAAKRDERVALLATHDVHTAHRDVEPLEVAADVQR
VCRPGQVLQTDDDGGDGGPVPPKRVRQDDAGAWISSPSSQMSVGSLSPPPPLLNGVANSSGLSPVSNCSS
YDTYSPRGPCKEEMSPSSGGGGLNGYFVDSFGDPKKKKGPAPRQQEELCLVCGDRASGYHYNALTCEGCK
GFFRRSITKNAVYQCKYGNNCDIDMYMRRKCQECRLKKCLSVGMRPECVVPEYQCAIKRESKKHQKDRPN
STTRESPSALMAPSSVGGVSPTSQPMGGGGSSLGSSNHEEDKKPVVLSPGVKPLSSSQEDLINKLVYYQQ

EFESPSEEDMKKTTPFPLGDSEEDNQRRFQHITEITILTVQLIVEFSKRVPGFDTLAREDQITLLKACSS
EVMMLRGARKYDVKTDSIVFANNQPYTRDNYRSASVGDSADALFRFCRKMCQLRVDNAEYALLTAIVIFS
ERPSLVDPHKVERIQEYYIETLRMYSENHRPPGKNYFARLLSILTELRTLGNMNAEMCFSLKVQNKKLPP
FLAEIWDIQE

>NR1H4_MOUSE
MVMQFQGLENPIQISLHHSHRLSGFVPDGMSVKPAKGMLTEHAAGPLGQNLDLESYSPYNNVPFPQVQPQ
ISSSSYYSNLGFYPQQPEDWYSPGIYELRRMPAETGYQGETEVSEMPVTKKPRMAAASAGRIKGDELCVV
CGDRASGYHYNALTCEGCKGFFRRSITKNAVYKCKNGGNCVMDMYMRRKCQECRLRKCREMGMLAECMYT
GLLTEIQCKSKRLRKNVKQHADQTVNEDDSEGRDLRQVTSTTKFCREKTELTADQQTLLDYIMDSYNKQR
MPQEITNKILKEEFSAEENFLILTEMATSHVQILVEFTKKLPGFQTLDHEDQIALLKGSAVEAMFLRSAE
IFNKKLPAGHADLLEERIRKSGISDEYITPMFSFYKSVGELKMTQEEYALLTAIVILSPDRQYIKDREAV
EKLQEPLLDVLQKLCKMYQPENPQHFACLLGRLTELRTFNHHHAEMLMSWRVNDHKFTPLLCEIWDVQ

>E75C_DROME
MHGGGPGSSGSNIIRRSSGSFPGSGSGSASKLIKTEPIDFEMLHLEENERQQDIEREPSSSNSNSNSNSL
TPQRYTHVQVQTVPPRQPTGLTTPGGTQKVILTPRVEYVQQRATSSTGGGMKHVYSQQQGTAASRSAPPE
TTALLTTTSGTPQIIITRTLPSNQHLSRRHSASPSALHHYQQQQQPQRQQSPPPLHHQQQQQQHVRVIR
DGRLYDEATVVVAARRHSVSPPPLHHHSRSAPVSPVIARRGGAAAYMDQQYQQRQTPPLAPPPPPPPPP
PPPPPQQQQQQYISTGVPPPTAAARKFVVSTSTRHVNVIASNHFQQQQQQHQAQQHQQQHQQHVIASVSS
SSSSSAIGSGGSSSSHIFRTPVVSSSSSSNMHHQQQQQQQQSSLGNSVMRPPPPPPPPPKVKHASSSSSGN
SSSSNTNNSSSSSNGEEPSSSIPDLEFDGTTVLCRVCGDKASGFHYGVHSCEGCKGFFRRSIQQKIQYRP
CTKNQQCSILRINRNRCQYCRLKKCIAVGMSRDAVRFGRVPKREKARIWRPCNRAPRIAASSDPSPPSWM
TSHASSPPCCCAHLETCEFTKEKVSAMRHGRGLPSTPCHTSGLSAEPAPELQSEQEFSQRFAHVIRGVID
FAGMIPGFQLLTQDDKFTLLKAGLFDALFVRLICMFDSSINSIICLNGQVMRRDAIQNGANARFLVDSTF
NFAERMNSMNLTDAEIGLFCAIVLITPDRPGLRNLELIEKMYSRLKGCLQYIVAQNRPDQPEFLAKLLET
MPDLRTLSTLHTEKLVVFRTEHKELLRQQMWSMEDGNNSDGQQNKSPSGSWADAMDVEAAKSPLGSVSST
ESADLDYGSPSSSQPQGVSLPSPPQQQPSALASSAPLLAATLSGGCPLRNRANSGSSGDSGAAEMDIVGS
HAHLTQNGLTITPIVRHQQQQQQQQQIGILNNAHSRNLNGGHAMCQQQQQHPQLHHHLTAGAARYRKLDS
PTDSGIESGNEKNECKAVSSGGSSSCSSPRSSVDDALDCSDAAANHNQVVQHPQLSVVSVSPVRSPQPST
SSHLKRQIVEDMPVLKRVLQAPPLYDTNSLMDEAYKPHKKFRALRHREFETAEADASSSTSGSNSLSAGS
PRQSPVPNSVATPPPVAASAAAGNPAQSQLHMHLTRSSPKASMASSHSVLAKSLMAEPRMTPEQMKRSDI
IQNYLKRENSTAASSTTNGLGNRSPSSSSTPPPSVQNQQRWGSSSVITTTCQQRQQSVSPHSNGSSSSSS
SSSSSSSSSSSTSSNCSSSSASSCQYFQSPHSTSIGTGEPDGAPVRDRTAPRPCWNCRWTLLTRRTSQFV
QEIAHAAAQQAARSGGRRQCRSKVSHIVRRRHSDSLQWRSSVGGGESGAQQQSAGECGLPQSGPERRRAQ
GNAGGVRAGGGRWFYAEKWERQRLGVAVQRSRKQDHLERRELN

>Q6GND0_XENLA
MTSLDCANNTGGVISYVGSNGSSPNRTSPVSLYSDCSTGSPQSGVTHYSNYLPPSPSNSYSANSSGSGGE
SSPRSSYGLASSPGGLHVALDDGSRVSPSKTSSSITKLNGMVLLCKVCGDVASGFHYGVHACEGCKGFFR
RSIQQNIQYKKCLKNETCSIVRINRNRCQQCRFRKCLSVGMSRDAVRFGRIPKREKQRMLAEMHSAMNHI
TSGQSHFGRRSPVPLQPHQQLHPSSSPLMGTAPTSLPTQAYSQYPQQLTPPTSPSPDHNDEVISQVAMAH
QQIFVYANDKVGKKTSWDHQHGNQPPPCWSHDSEESHDIRLACPMNALHQGGPVRSVQEVWEHFSLSFTP
AVREVVEFARHIPGFNDLTQNDQVTLLKAGTFEVLMVRFASLFDARERSLRFLSGATYSLPELHAMGME

LLGSMFDFSEKLSSLSLSQEELGIFTALVLVSADRSGMENSSLVEQLQETLIRALRSLILKNSPNDTSRF
TKLLLRLPDLRTLNNLHSEKLLSFRVDAH

>ECR_BOMMO
MRVENVDNVSFALNGRADEWCMSVETRLDSLVREKSEVKAYVGGCPSVITDAGAYDALFDMRRRWSNNGG
FPLRMLEESSSEVTSSSALGLPPAMVMSPESLASPEYRALELWSYDDGITYNTAQSLLGACNMQQQQLQP
QQPHPAPPTLPTMPLPMPPTTPKSENESMSSGREELSPASSINGCSADADARRQKKGPAPRQQEELCLVC
GDRASGYHYNALTCEGCKGFFRRSVTKNAVYICKFGHACEMDMYMRRKCQECRLKKCLAVGMRPECVIQE
PSKNKDRQRQKKDKGILLPVSTTTVEDHMPPIMQCDPPPPEAARIHEVVPRYLSEKLMEQNRQKNIPPLS
ANQKSLIARLVWYQEGYEQPSDEDLKRVTQTWQSDEEDEESDLPFRQITEMTILTVQLIVEFAKGLPGFS
KISQSDQITLLKASSSEVMMLRVARRYDAASDSVLFANNKAYTRDNYRQGGMAYVIEDLLHFCRCMFAMG
MDNVHFALLTAIVIFSDRPGLEQPSLVEEIQRYYLNTLRIYIINQNSASSRCAVIYGRILSVLTELRTLG
TQNSNMCISLKLKNRKLPPFLEEIWDVAEVATTHPTVLPPTNPVVL

>Q8MX80_BRAFL
MWEDASMSQGEKLLPRLARAWEANIAEVFWQTHHAGDSWDDQRSSNHQSDRCITDQSSSEEMEPSPPSPP
PPPRVYKPCFVCSDKSSGYHYGVASCEGCKGFFRRSIQKNMQYVCHRDKNCVINKVTRNRCQFCRLKKCF
DVGMSKESVRNDRNKKRKDKTQSLEKHTLSYNWTPEIQTIITTVREAHMATLPDMGKLPKYKVKNAAEQR
GPTDIELWQHFSDLCTETIIKIVQFAKKVPGFTTFGTADQITLLKAACLDILILRLATRLDKESDTVTFI
NGMMLSRTQMHNAGFGPLTDGVFTFAEGMQKLLFDETEIGLMCSICLVCGDRQGLEDIQRAENLQEPLLE
ALKAYSRRRIPDDPQRFPKMIMKITDLRSISSKGAERVITLKMELSSPMPPLIAEIWEKQNEALS

>Q6TNS3_BRARE
MTLLGLNMTTAVDTNNTGGVISYIGSCGGSPNRTSPVSMYSENSNSSMQSLTQPCFGSSFPPSPNGSHDS
SRMYTSSSSSSSSGSGEDGNSSCSGGSPRGRDDGGSARNSPNKSVATLTKLNGMVLLCKVCGDVASGFHY
GVHACEGCKGFFRRSIQQNIQYKKCLKNETCTIMRINRNRCQQCRFKKCLSVGMSRDAVRFGRIPKREKQ
RMLAEMQNAMNNMVNNQLQNEFQLASITSNTPCPSSSSPSNTSSSSSSPCPGLTVGPQPQPPAVPVAQSP
SSPAPTSPTVQLAQSPPPLTSTPPPCTSPGVDKTIAAITRAHRETFIYAHDKLGSPLLPHNSELDNRSNN
RCMAGYHLNGHNTIYHHDNNVAHHCNNFEVQQDNSLHFQASQTPEKHQQHNNNSQRPPNSNFYSIHHGTR
DEQRIPGSELSMEKHKEILLACPMNMHPYSDPNKTPQEIWEDFSLSFTPAVREVVEFAKHIPGFSTLSQN
DQVTLLKAGTFEVLMVRFSSLFNVKEKTVTFISGATYSLEALKSMGMGDLLGTMFDFSEKLNSLELSAEE
LGLFTAVVLVSADRSGIENVNSVEMLQESLIRALRTLVSKSAPTDASRFTKLLLKLPDLRTLNNMHSEKL
LSFRIDA

>O76246_UCAPU
MAKVLATARVDGMFVLGSGVATLNLSTMGDESCSEVSSSSPLTSPGALSPPALVSVGVSVGMSPPTSLAS
SDIGEVDLDFWDLDLNSPSPPHGMASVASTNALLLNPRAVASPSDTSSLSGRDDMSPPSSLSNFGADSYG
DLKKKKGPIPRQQEELCLVCGDRASGYHYNALTCEGCKGFFRRSITKNAVYQCKYGNNCEMDMYMRRKCQ
ECRLKKCLNVGMRPECVVPESQCQVKREQKKARDKDKTYPSLGSPIAEDKAAPISPVSKDMSAAPRLNVK
PLTREQEELINTLVYYQEEFEQPTEADVKKIRFNFDGEDTSDMRFRHITEMTILTVQLIVEFSKQLPGFA
TLQREDQITLLKACSSEVMMLRAARRYDAKTDSIVFGNNYPYTQASYALAGLGESAEILFRFCRSLCKMK
VDNAEYALLAAIAIFSERPNLKELKKVEKLQEIYLEALKSYVENRRLPRSNMVFAKLLNILTELRTLGNI
NSEMCFSLTLKNKRLPPFLAEIWDVSGY

>RORA_HUMAN
MNEGAPGDSDLETEARVPWSIMGHCLRTGQARMSATPTPAGEGARRDELFGILQILHQCILSSGDAFVLT
GVCCSWRQNGKPPYSQKEDKEVQTGYMNAQIEIIPCKICGDKSSGIHYGVITCEGCKGFFRRSQQSNATY
SCPRQKNCLIDRTSRNRCQHCRLQKCLAVGMSRDAVKFGRMSKKQRDSLYAEVQKHRMQQQQRDHQQQPG
EAEPLTPTYNISANGLTELHDDLSNYIDGHTPEGSKADSAVSSFYLDIQPSPDQSGLDINGIKPEPICDY
TPASGFFPYCSFTNGETSPTVSMAELEHLAQNISKSHLETCQYLREELQQITWQTFLQEEIENYQNKQRE
VMWQLCAIKITEAIQYVVEFAKRIDGFMELCQNDQIVLLKAGSLEVVFIRMCRAFDSQNNTVYFDGKYAS
PDVFKSLGCEDFISFVFEFGKSLCSMHLTEDEIALFSAFVLMSADRSWLQEKVKIEKLQQKIQLALQHVL
QKNHREDGILTKLICKVSTLRALCGRHTEKLMAFKAIYPDIVRLHFPPLYKELFTSEFEPAMQIDG

>Q9PTN2_BRARE
MLTENSAVNSGGKSKCEAGACESTVNGDATSLMDLMAVSTSATGQDQFDRNAPPICGVCGDKATGFHFNA
MTCEGCKGFFRRSMKRKASFTCPFNGNCTITKDNRRHCQACRLKRCIDIGMMKEFILTDEEVQRKKDLIM
KRKEEEAAREARKPRLSDEQMQIINSLVEAHHKTYDDSYSDFVRFRPPVREGPVTRSASRAASLHSLSDA
SSDSFNHSPESVDTKLNFSNLLMMYQDSGSPDSSEEDQQSRLSMLPHLADLVSYSIQKVIGFAKMIPGFR
DLTAEDQIALLKSSAIEIIMLRSNQSFSLEDMSWSCGGPDFKYCINDVTKAGHTLELLEPLVKFQVGLKK
LKLHEEEHVLLMAICLLSPDRPGVQDHVRIEALQDRLCDVLQAYIRIQHPGGRLLYAKMIQKLADLRSLN
EEHSKQYRSLSFQPEHSMQLTPLVLEVFGSEVS

>HR3_MANSE
MNNNQFHELFGSQWPPDQHGGHSSASTMLHQQTMPQTMQLKREPHTEVGVMHNQMGMDITSGSVADSTSP
PPGSSEGMFGPISGMFMDKKAANSIRAQIEIIPCKVCGDKSSGVHYGVITCEGCKGFFRRSQSTVVNYQC
PRNKACVVDRVNRNRCQYCRLQKCLKLGMSRDAVKFGRMSKKQREKVEDEVRYHKAQMRAQNDAAPDSVY
DAQQQTPSSSDQFHGHYNGYPGYASPLSSYGYNNAGPPLTSNMSSIQAQPQAQQPYDYADSTTTYEPKQP
GYLDADFIGQVEGDISKVLVKSLAERHANTNPKLEYINEMFSKPQDVSKLLFYNSMTYEEMWLDCADKLT
AMIQNIIEFAKLIPGFMKLTQDDQILLLKSGSFELAIVRLSRLIDVNREQVLYGDVVLPIRECVHARDPR
DMALVSGIFEAAKSIARLKLTESELALYQSLVLLWPERHGVMGNTEIRCLFNMSMSAMRHEIEANHAPLK
GDVTVLDTLLAKIPTFRELSLMHLGALSRFKMTHPHHVFPALYKELFSLDSVLDYTHG

>Q9UNW4_HUMAN
MTVTRTHHFKEGSLRAPAIPLHSAAAELASNHPRGPEANLEVRPKESWNHADFVHCEDTESVPGKPSVNA
DEEVGGPQICRVCGDKATGYHFNVMTCEGCKGFFRRAMKRNARLRCPFRKGACEITRKTRRQCQACRLRK
CLESGMKKEMIMSDEAVEERRALIKRKKSERTGTQPLGVQGLTEEQRMMIRELMDAQMKTFDTTFSHFKN
FRLPGVLSSGCELPESLQAPSREEAAKWSQVRKDLCSLKVSLQLRGEDGSVWNYKPPADSGGKEIFSLLP
HMADMSTYMFKGIISFAKVISYFRDLPIEDQISLLKGAAFELCQLRFNTVFNAETGTWECGRLSYCLEDT
AGGFQQLLLEPMLKFHYMLKKLQLHEEEYVLMQAISLFSPDRPGVLQHRVVDQLQEQFAITLKSYIECNR
PQPAHRFLFLKIMAMLTELRSINAQHTQRLLRIQDIHPFATPLMQELFGITGS

>Q86BC1_DROME
MASLLGSAPPAQILANQPIIVKIEPTQSFHIVDEGDTRVLSLPLSDADKLGASWIDLKDIAGLQAGGGAT
LLDVCFEQANEDGTIIATVQPDLENELEAELKAEGEPEDETEPEPPAPKRLATTRPAQSRPQQQQQQQQQ
QVKFLSDPPALARSSSFSSLSSFSSISNISSVCKNMASNTSQEGSLKRTKERTPPPMPPLTTHKPAATTT
ATSATSAAAATSAATASATATSARENSREHSSSSSGSNGAMTAQIEIIPCKVCGDKSSGVHYGVITCEGC
KGFFRRSQSSVVNYQCPRNKQCVVDRVNRNRCQYCRLQKCLKLGMSRDAVKFGRMSKKQREKVEDEVRFH

RAQMRAQSDAAPDSSVYDTQTPSSSDQLHHNNYNSGGYSNNEVGYGSPYGYSASVTPQQTMQYDISADYV
DSTTYEPRSTIIDPEFISHADGDINDVLIKTLAEAHANTNTKLEAVHDMFRKQPDVSRILYYKNLGQEEL
WLDCAEKLTQMIQNIIEFAKLIPGFMRLSQDDQILLLKTGSFELAIVRMSRLLDLSQNAVLYGDVMLPQE
AFYTSDSEEMRLVSRIFQTAKSIAELKLTETELALYQSLVLLWPERNGVRGNTEIQRLFNLSMNAIRQEL
ETNHAPLKGDVTVLDTLLNNIPNFRDISILHMESLSKFKLQHPNVVFPALYKELFSIDSQQDLT

>Q8JJ27_XENLA
MRQREQLEQTMANSYVTVSDAYCLAEPLTYYDVLPDHINYQLPDSEFQTASCCQYTNMAYSSGLQSPSSQ
CHYTSYGLEAAYGDGQYLLSTCELSKPSTYMTHSMDDVFPTMKRPRVSHTSIKVKGHEELCVVCGDKASG
YHYNALTCEGCKGFFRRSITKNAVYRCKNGGHCEMDMYMRRKCQECRLKKCKAVGMLAECLLTEVQCKSK
RLRKNCKQNNSILSNVKTEDDGSDSRHVSSTTKLTKLPSQLELTGEEYKLIDHIVTAHQKCGISLDDMKM
FLEESADPEEIFYHFSEAAVLHVRAFVEFTKSLPGFEMLDPLDQIALLKASTVEAMLLRSAQIYNQSVMG
STLQTTEGLARYPSHSVDFSQIQEFDKCPLYSLEAHPQEEDSTSSTDLTEEFITPLFNFFRSMGSLNVTE
AEYALLSAVTVYFSDRPLLQSKPHVEKLQEPLLGILHKYSRLYHPEDPQHFARLIGRLTELRTLHHNHSE
VLVLWKARDTKLTPFAVWVLESTMSLESNCKFNLNLLQPA

>E75_METEN
MFCDQDMYEIPADCQVLVDKTVIEFDGTTVLCRVCGDKASGFHYGVHSCEGCKGFFRRSIQQKIQYRPCT
KNQQCSILRINRNRCQYCRLKKCIAVGMSRDAVRFGRVPKREKAKILAAMQSVNAKSQERAVLAELEDDT
RVTAAIIRAHMDTCDFTRDKVAPMLQQARTHPSYTQCPPYLACPLNPRPVPLHGQQELVQDFSEALLPAI
RGVVEFAKRLPGFQQLPQEDQVTLLKAGVFEVLLVRLAGMFDARTNAMLCLNGQLVRREALHTSVNARFL
MDSMFDFAERVNSLALNDAELALFCAVVVLAPDRPGLRNAELVERVHRRLVNCLQAVVSKHHPENPNLQR
DLLSKIPDLRTLNTLHSEKLLKYKMTEHTAAGAPWDDSRSSWSMEQESSVGSPSSSYTTDEAMRSPVSCS
ESICSGESASSGESLCGSEVSGYTELRPPFPLARRRHDHSEGASSGDEATESPLKCPFSKRKSDSPDDSG
IESGTDRSDKLSSPSVCSSPRSSIDEKERGGPARTICRCCARLQRRPSSTRICSWRKPTTSPIKSSVRNV
GKRSLTPHSPPPPRSWSRRCLSLHSTRALWLRHTPPWPPVWRRPLA

>E78A_DROME
MDVYQIELEEQAQIRSKLLVETCVKHSSSEQQQLQVKQEDLIKDFTRDEEEQPSEEEAEEEDNEEDEEEE
GEEEEEDEEEDEDEEALLPVVNFNANSDFNLHFFDTPEDSSTQGAYSEANSLESEQEEEKQTQQHQQQKQ
HHRDLEDCLSAIEADPLQLLHCDDFYRTSALAESVAASLSPQQQQQRQHTHQQQQQQQQQQQHPGQQQHQ
LNCTLSNGGGALYTISSVHQFGPASNHNTSSSSPSSSAAHSSPDSGCSSASSSGSSRSCGSSSASSSSSA
VSSTISSGRSSNNSVVNPAATSSSVAHLNKEQQQQPLPTTQLQQQQHQQQLQHPQQQQSFGLADSSSSN
GSSNNNNGVSSKSFVPCKVCGDKASGYHYGVTSCEGCKGFFRRSIQKQIEYRCLRDGKCLVIRLNRNRCQ
YCRFKKCLSAGMSRDSVRYGRVPKRSRELNGAAASSAAAGAPASLNVDDSTSSTLHPSHLQQQQQQHLLQ
QQQQQQHQPQLQQHHQLQQQPHVSGVRVKTPSTPQTPQMCSIASSPSELGGCNSANNNNNNNNNSSSGNA
SGGSGVSVGVVVVGGHQQLVGGSMVGMAGMGTDAHQVGMCHDGLAGTANELTVYDVIMCVSQAHRLNCSY
TEELTRELMRRPVTVPQNGIASTVAESLEFQKIWLWQQFSARVTPGVQRIVEFAKRVPGFCDFTQDDQLI
LIKLGFFEVWLTHVARLINEATLTLDDGAYLTRQQLEILYDSDFVNALLNFANTLNAYGLSDTEIGLFSA
MVLLASDRAGLSEPKVIGRARELVAEALRVQILRSRAGSPQALQLMPALEAKIPELRSLGAKHFSHLDWL
RMNWTKLRLPPLFAEIFDIPKADDEL

>Q80ST6_MOUSE
MANTYVATSDGYYLAEPTQYYDILPEQFHYQLCDTDFQEPPYCQYSTAQFPPALQSPSLQSHFNTHGLDP

QYSGGSWCGLDARESGQSTYVVVHDDEDEFPGAQRCRATCSLRWKGQDDMLCMVCGDKASGYHYNALTCE
GCKGFFRRSITKNAVYSCKNGGHCEMDMYMRRKCQECRLKKCKAVGMLAECLLTEIQCKSKRLRKNFKHG
PALYPAIQVEDEGADTKHVSSSTRSGKGVQDNMTLTQEEHRLLNTIVTAHQKSMIPLGETSKLLQEGSNP
ELSFLRLSEVSVLHIQGLMKFTKGLPGFENLTTEDQAALQKASKTEVMFLHVAQLYGGKDSTSGSTMRPA
KPSAGTLEVHNPSADESVHSPENFLKEGYPSAPLTDITKEFIASLSYFYRRMSELHVSDTEYALLTATTV
LFSDRPCLKNKQHIENLQEPVLQLLFKFSKMYHPEDPQHFAHLIGRLTELRTLSHSHSEILRMWKTKDPR
LVMLFSEKWDLHSFS

>RORG_HUMAN
MDRAPQRQHRASRELLAAKKTHTSQIEVIPCKICGDKSSGIHYGVITCEGCKGFFRRSQRCNAAYSCTRQ
QNCPIDRTSRNRCQHCRLQKCLALGMSRDAVKFGRMSKKQRDSLHAEVQKQLQQRQQQQQEPVVKTPPAG
AQGADTLTYTLGLPDGQLPLGSSPDLPEASACPPGLLKASGSGPSYSNNLAKAGLNGASCHLEYSPERGK
AEGRESFYSTGSQLTPDRCGLRFEEHRHPGLGELGQGPDSYGSPSFRSTPEAPYASLTEIEHLVQSVCKS
YRETCQLRLEDLLRQRSNIFSREEVTGYQRKSMWEMWERCAHHLTEAIQYVVEFAKRLSGFMELCQNDQI
VLLKAGAMEVVLVRMCRAYNADNRTVFFEGKYGGMELFRALGCSELISSIFDFSHSLSALHFSEDEIALY
TALVLINAHRPGLQEKRKVEQLQYNLELAFHHHLCKTHRQSILAKLPPKGKLRSLCSQHVERLQIFQHLH
PIVVQAAFPPLYKELFSTETESPVGLSK

>O02643_CHOFU
MQRTPRLRRARRILCLLPPLPTTTASRQIEIIPCKVCGDKSSGVHYGVITCEGCKGFFRRSQSTVVNYQC
PRNKACVVDRVNRNRCQYCRLQKCLKLGMRRDAVKFGRMSKKQREKVEDEGRFHRAQMRGQTDAPLDSVY
DAQQQTPSSSDQFHGHYNSYPGYASPISSYGYNNTAPSLTSTMIQPHADSAAIRRTSGLCGLTTAYEPKQ
GGFLDADFIGHDEQKGTTVVRTVRINIHVTATTSTTSMRQSTINEIPRRLQDFDRYEPDRIQSPASISSG
VINIKQEIKPETSMGVDNLVASYVDSTTFLHSPSNLQHSPMDIQNSVLVSGQSSVSLTSDELSPDDLTSS
SGHGRLIDPLNLNMSGMGMVNPNAVSNRRHQGSSHSGSSNEDLPSEGDISKVLVKSLAEAHANTIPKLEY
IHEMLRKPVDVAKQLFYSSMTYEAMWLDCADKLTTMIQSIIEFAKLMPGFMKLTQDDQILLLKSGSFELA
IVRLSRLIDVNRETVLYGDVVLPIRDCVHARDPRDMALIVGIFDAAKTIARLKLTETELALYQSLVLLWP
ERHGVRGNHEIQCLFNMSMTAMRHEIETNHAPIKGDVTVLDTLLAKLPTFRELSLMHLEALCRFKAAHPL
HIFPALYKELFSLDSVLDYHV

>Q7ZZY9_PETMA
MMATQNMTVSTSNALEDDEEGGVPKVCGVCGDKATGYHFNAMTCEGCKGFFRRSMKRSASFTCPFEGKCN
ITKDNRRHCQACRLKRCRDIGMMKELIMTEEEVQRKKEIIMKRKLEDSAREVHTPQLLEEQERLIATLIE
AHRKTYDASYSDFSQFRPPKRGDGSPECRNATNPFLMSLLNSDMDELPKASASGAEAAAGDELSMLPHLA
DLVSYSIQKVIGFAKMIPGFKELCTEDQISLLKASAIEIIILRSNESFTMEDNSWTCGSNEFKYQIGDVM
QAGHKLELLEPLVKFQVNMKKLDLHEAEHVLLMAICLFSPDRPGVQDRCRVEEVQEHLTETLRAYIACRH
PLSCKHMLYTKMVEKLTELRSLNEEHSKQYLQISQDAVNKEDLPPLLLEVFGNPTA

>Q9DF24_XENLA
MSEVQEAVVLEEEEEEEEDPCNSCGTGEDEDDGEPKICRACGDRATGYHFNAMTCEGCKGFFRRAMKRNL
RLSCPFQNSCVINKNNRRHCQACRLKKCLDIGMRKELIMSDEAVEQRRALIKRKQNLSLSPPTPPGASLT
PEQQHFITELVEAHTKTFDFNFTFFKNFRPIRRSPDPTQDPQATSSEAFLMLPHISDLFTYMLKGVISFA
KMLPYFRSLAIEDQIALLKGSVLEVCVIRFNRMFNPKTNTWECGAFTYNADDMTMAGFSQQFLEPLLRIH
CMMTKLNLESEAYALMATMALFSSDRPGVSDCEKIQNLQEHIALMLKAFIESHRPPSPQNRLLYPKIMEC

LTELRTINDIHSKQLMEIWDIQPDVTPLMREVFGSLNE

>Q9DFH3_CHICK
MSQSSPSDPDSPGAQRCPNVTDVTEEELKVCAVCGDRATGYHFHVMSCEGCKGFFRRSILKGVHFTCPFT
RSCPITKAKRRQCQACRLQKCLDVGMRKDMIMSEEALGRRRALRLQRRLAQAQPGGLTAEQQELISILIA
AHKRTFDSSFSQFQHYQPAVRLCIPGPCSQSPPGPGVPSASLSPQLDCLDEDVLPDVFSILPHFADLSTF
MIQQVIKFAKEIPAFRGLPIDDQISLLKGATLGICQIQFNTVFNEETNAWECGQHCFTIKDGALAGFQQI
YLEPLLKFHISLKKLRLHEAEYVLLVAMLLFSPDHASVTQRDFIDQLQEKVALTLKSYIDHRHPMPEGRF
LYAKLLLLLTELQTLKMENTRQILHIQDLSSMTPLLSEIIS

>Q60Q78_CAEBR
MQAVEPKLVGPTLPPSLQTPSSSSPPSPVHFQQLRSIPSSPQSSQQSSAPSQAPPVESPDGGKSKENENG
AENQHQPPRPMLHPHMIKSEAADYRYSPELKPNTIFGAQGDHPSHAFAKMLTCVIDPNQVNMSGMMPHAS
AFAAMHNHHQYESKPLQVSAQIEVIPCKVCGDKSSGVHYGVITCEGCKGFFRRSQSSIVNYQCPRQKNCI
VDRVNRNRCQYCRLKKCIELGMSRDAVKFGRMSKKQREKVEDEVRMHKQLAEASGLGYHIYGDYSPPPSH
NYIFDQSMYGHYPSGTSTPVNGYPIAMPATPVTPMPQNMYGANPSVATGSHYVAHQATGGSFPSPQVPEE
DVVSRVISSFDQQHLDYRTNNAVCEVDPETFSRLDRADGWELFSLQLDPLVKTIIEFAKCVDGFMNLPQE
TQIQLLKGSVFELCLIFAAMYYNMDTHAVCGERGTIPFACVVTEDVDEMNLINEIHGTLHDIVALQPNTS
ELALMAAGLLLEQATTSSSSSNGQGDPSTLATAELLKTALHQSMVARTGCMDTTITRIQDVEQKIRQTAR
LHQEALTKFRLSDPRASEKLPDLYKELFTADRLSPTI

>NR1I3_RAT
MTATLTLETMTSEEEYGPRNCVVCGDRATGYHFHALTCEGCKGFFRRTVSKTIGPICPFAGRCEVSKAQR
RHCPACRLQKCLNVGMRKDMILSAEALALRRARQARRRAQKASLQLSQQQKELIQTLLGAHTRHVGPMFD
QFVQFRPPAYLFSHHRPFQPLAPVLPLLTHFADINTFMVQQIIKFTKDLPLFRSLTMEDQISLLKGAAVE
ILHISLNTTFCLQTQNFFCGPLCYKMEDAVHVGFQYEFLELIIHFHKTLKRLQLQEPEYALMAAMALFSP
DRPGVTQREEIDQLQEEVALILNNHIMEQQSRLQSRFLYAKLMGLLAELRSINSAYSYEIHRIQGLSAMM
PLLGEICS

>Q9XXU7_CAEEL
MSFETKPNYLLLTNPDTPLSVCTSPYYSPSGKTASIPSSEASKPEGTNGQWSHLPTGATYVTDEFSSEFQ
IQNGSTAAQSGNANNYADPLSHRRYFNNVNGYNHHQFYDTASQASVSSPATSVTSSLSPPDSLSNGHTTQ
RHHIGKAISFCKVCGDKASGYHYGVTSCEGCKGFFRRSIQRKIDYRCLKQQVCEIKRESRNRCQYCRFKK
CLDSGMSKDSVRQMKFRNAMRDDKSPDSVFVPEISTLERQEEVDAVYEAVLRAHTTFSFYTDIKIRSIVA
RPFNVRINEDSKMNRLNAWQIYAHEIDVDIKEVVNFVKEIPKFNFINGNDKAVLLRKNAFPLYLLRIVRG
MSNRGLMLRDGRLIDFKSLQLLYGSLADEMLAFANHIITIGCTDGDIALFIVLILCQPLTTEQQFSTNFK
SQLQLLEMFDFYKKVLFQKMTCRIDGCDTYKQLMKCIHELNRLNELHKQQLNILRENLSFLNLPPLVVEM
FQLSTLPLPVNHNNQENHILLLQSIRALNHSSQRQINNRLPFTASHLCQILIRFHSNTIHLQIISL

>Q6GZ85_HUMAN
MASREDELRNCVVCGDQATGYHFNALTCEGCKGFFRRTVSKSIGPTCPFAGSCEVSKTQRRHCPACRLQK
CLDAGMRKDMILSAEALALRRAKQAQRRAQQTPVQLSKEQEELIRTLLGAHTRHMGTMFEQFVQFRPPAH
LFIHHQPLPTLAPVLPLVTHFADINTFMVLQVIKFTKDLPVFRSLPIEDQISLLKGAAVEICHIVLNTTF
CLQTQNFLCGPLRYTIEDGARDRPGVTQRDEIDQLQEEMALTLQSYIKGQQRRPRDRFLYAKLLGLLAEL

RSINEAYGYQIQHIQGLSAMMPLLQEICS

>NHR1_ONCVO
METKESTMNDQNTHTRSRNKAIPYLPRYMKPGQPCVVCGDDATGLHYRAITCEGCKGFFRRTVQQKIVYK
CKSIERCEISKISRNICQFCRFQKCLRNGMTKSLVLNETERIAKRKMIIDNRERRKLEHLRTLLKASSLA
DKQDEFQSRIDQVTANYCKIMDNSLEYKFKSNKKSERLIELTKLVSQQVRQFAETIEICDTLNPSEKEEI
IAKSWLVVKILQIIHEFNPTECCLMLANNTTYIPAKGNYSELDKTTKIFENLINLAISFNCMQLDNRQLA
LLSALLIYNPENVKRSKEKIDKIHVELWKCLQSISEMHDDDSSDLLHWPNFLVRIPYLILTVSKMQDFFQ
DENNINAIANILLFKFT

>HR96_DROME
MSPPKNCAVCGDKALGYNFNAVTCESCKAFFRRNALAKKQFTCPFNQNCDITVVTRRFCQKCRLRKCLDI
GMKSENIMSEEDKLIKRRKIETNRAKRRLMENGTDACDADGGEERDHKAPADSSSSNLDHYSGSQDSQSC
GSADSGANGCSGRQASSPGTQVNPLQMTAEKIVDQIVSDPDRASQAINRLMRTQKEAISVMEKVISSQKD
ALRLVSHLIDYPGDALKIISKFMNSPFNALTVFTKFMSSPTDGVEIISKIVDSPADVVEFMQNLMHSPED
AIDIMNKFMNTPAEALRILNRILSGGGANAAQQTADRKPLLDKEPAVKPAAPAERADTVIQSMLGNSPPI
SPHDAAVDLQYHSPGVGEQPSTSSSHPLPYIANSPDFDLKTFMQTNYNDEPSLDSDFSINSIESVLSEVI
RIEYQAFNSIQQAASRVKEEMSYGTQSTYGGCNSAANNSQPHLQQPICAPSTQQLDRELNEAEQMKLREL
RLASEALYDPVDEDLSALMMGDDRIKPDDTRHNPKLLQLINLTAVAIKRLIKMAKKITAFRDMCQEDQVA
LLKGGCTEMMIMRSVMIYDDDRAAWKVPHTKENMGNIRTDLLKFAEGNIYEEHQKFITTFDEKWRMDENI
ILIMCAIVLFTSARSRVIHKDVIRLEQNSYYYLLRRYLESVYSGCEARNAFIKLIQKISDVERLNKFIIN
VYLNVNPSQVEPLLREIFDLKNH

(2) 36 NR2

>RXRB_HUMAN
MSWAARPPFLPQRHAAGQCGPVGVRKEMHCGVASRWRRRRPWLDPAAAAAAAVAGGEQQTPEPEPGEAGR
DGMGDSGRDSRSPDSSSPNPLPQGVPPPSPPGPPLPPSTAPSLGGSGAPPPPPMPPPPLGSPFPVISSSM
GSPGLPPPAPPGFSGPVSSPQINSTVSLPGGGSGPPEDVKPPVLGVRGLHCPPPPGGPGAGKRLCAICGD
RSSGKHYGVYSCEGCKGFFKRTIRKDLTYSCRDNKDCTVDKRQRNRCQYCRYQKCLATGMKREAVQEERQ
RGKDKDGDGEGAGGAPEEMPVDRILEAELAVEQKSDQGVEGPGGTGGSGSSPNDPVTNICQAADKQLFTL
VEWAKRIPHFSSLPLDDQVILLRAGWNELLIASFSHRSIDVRDGILLATGLHVHRNSAHSAGVGAIFDRV
LTELVSKMRDMRMDKTELGCLRAIILFNPDAKGLSNPSEVEVLREKVYASLETYCKQKYPEQQGRFAKLL
LRLPALRSIGLKCLEHLFFFKLIGDTPIDTFLMEMLEAPHQLA

>Q9BMU6_AEDAE
MYGGGVLCPSPSTATGFYNPRGQGSEIGALELGFPRGMALVPPPPHGAWRDPTGLGSHLPVSSTSEDITT
LGGGNAPPTVSSMQSNTSIDKKDFISSASAGSQHNAGSLAVSQHTSVDKKEFLSLQQSTTPGSQSMNSQN
GSQQDIKNQNIECVVCGDKSSGKHYGQFTCEGCKSFFKRSVRRNLTYSCRGNRNCPIDQHHRNQCQFCRL
RKCLKMGMRREAVQRGRVPPSQPPGLPYGQYSIPNGDAVTGFNGHSYLSSYISLLLRAEPYPTSRYGQCM
QTNNIMGIDNICELAARLLFSAVEWARNIPFFPDLQVTDQVALLRLVWSELFVLNASQCSMPLHVAPLLA
AAGLHASPMAADRVVAFMDHIRIFQEQVEKLKALHVDSAEYSCLKAIVLFTTDACGLSDVAHIESLQEKS
QCALEEYCRSQYPNQPTRFGKLLLRLPSLRTVSSQVIEQFFFVRLVGKTPIETLIRDMLLSGSSFSWPYL
PSM

>Q8MX78_BRAFL

MESLGSPGGAPASSTTPNPTTQHQPMHYSAPPHIPSMTSSGPHITSPPPTLSSGQPPLTSNPSTMTSPHL
VQTPSVLTSSHPLHLHPGFGMPGVNQVSSSMQEDVKPVISQLGPTPLQNVSPHMMNTPLMVNTGQLTPPA
QPLQSPRPSQTPMGLSKHICQICGDRASGKHYGVYSCEGCKGFFKRTVRKDLTYACRDNRDCVIDKRQRN
RCQYCRYQKCLAMGMKREDVQDQRQGSGNSAVQEERQRSKEGKDGEVVSTTNPNEDMPVEKIQEAEMAVE
PKDGNMVEQPNDPVTNICQAADKQLVTLVEWAKRIPHFSDLPIDDQVILLRAGWNELLIAAFSHRSIDVK
DGILLASGLHVHRSSAHQAGVGTIFDRVLTELVAKMRDMKMDKTELGCLRAIVLFNPDAKGLTDPSLVES
LREKVYASLEEYCKQQYPEQPGRFAKLLLRLPALRSIGLKCLEHLFFFKLIGDTPIDTFLMEMLEAPGLG
QTAASQGQGPMAQAAAQHREAQQQAQQVQPPS

>Q6GN21_XENLA

MASIEEIAHQIIEQQMGEISRSQTEVSQTALMDGTTQRIQLVPSESNVSVPQRIQIVTDPQTGQKIQIVT
ALDQSGVSKQFILTNNDGSLPSKVILARQDSNQGKVFLTTPDAAGMNQLFFSSPDVPAQHIQILSDTQSL
DQNLNKQLVELCVVCGDKASGRHYGAVTCEGCKGFFKRSIRKNLVYTCRGSKDCVINKHYRNRCQYCRLQ
RCMSLGMKQDSVQCERKPIEVSREKSSNCAASTEKIYIRKDLRSPLAATTTFVTENKTPRTTSLLDSSML
VNIQQSGVKNESILITPNKVEACQGDLSTLANVVTSLANLNKTNDLPQTNTELSIIESLSNGDSSLSDLA
QDDQSSSEVTRAFDTLAKALNQSENSTQGSSECLGSNANLLHDVNVEIEGPLLNDVHIAFRLTMPSPMPE
YLNVHYICESASRLLFLSMHWARSIPSFQSLGQENSISLVKACWNELFSLGLAQCSQVMNVETILAAFVN
HLQNSMQHDKLSSDKVKLVTDHIFKLQEFCNSMVKLCVDGYEYAYLKAIALFSPDHPGLENVSHIEKLQE
KAYMEFQDYVTKTYPEDTYRLSRLLLRLPALRLMNAAITEELFFAGLIGNVQIDSIIPYILRMETSDYNS
QIIGLAV

>7UP2_DROME

MCASPSTAPGFFNPRPQSGAELSAFDIGLSRSMGLGVPPHSAWHEPPASLGGHLHAASAGPGTTTGSVAT
GGGGTTPSSVASQQSAVIKQDLSCPSLNQAGSGHHPGIKEDLSSSLPSANGGSAGGHHSGSGSGSGSGVN
PGHGSDMLPLIKGHGQDMLTSIKGQPTGCGSTTPSSQANSSHSQSSNSGSQIDSKQNIECVVCGDKSSGK
HYGQFTCEGCKSFFKRSVRRNLTYSCRGSRNCPIDQHHRNQCQYCRLKKCLKMGMRREAVQRGRVPPTQP
GLAGMHGQYQIANGDPMGIAGFNGHSYLSSYISLLLRAEPYPTSRYGQCMQPNNIMGIDNICELAARLLF
SAVEWAKNIPFFPELQVTDQVALLRLVWSELFVLNASQCSMPLHVAPLLAAAGLHASPMAADRVVAFMDH
IRIFQEQVEKLKALHVDSAEYSCLKAIVLFTTGKLLDILYKDVPALLTKVSALLGKGSTASNDDVLAVVR
DHLDELNRQEQESQAQQQAPLHLAAFMNCVAGVEAAVQQAEQAQVPTSSASASVSAPLVPSAGSAFSSCQ
AKSAGSEMDLLASLYAQAQATPPSSGGGDASGHNNSSGLGASLPTQSQSGSSSRNLTASPLSTSLATAPA
PASASAPAPVPTSSVAQVPVPAPVPVTSSASSSSLGGGAYQTPSAAAAAAAMFHYQTPPRAAFGSAFDMF
HHSTPFGVGVGHAHALAHSSGSGSASFGSPSYRYSPYSLAGSRWQL

>O76241_UCAPU

MIMIKKEKPVMSVSSIIHGSQQRAWTPGLDIGMSGSLDRQSPLSVAPDTVSLLSPAPSFSTANGGPASPS
ISTPPFTIGSSNTTGLSTSPSQYPPSHPLSGSKHLCHPLSGSKHLCSICGDRASGKHYGVYSCEGCKGFF
KRTVRKDLTYACREERSCTIDKRQRNRCQYCRYQKCLTMGMKREAVQEERQRTKGDKGDGDTESSCGAIS
DMPIASIREAELSVDPIDEQPLDQGVRLQVPLAPPDSEKCSFTLPFHPVSEVSCANPLQDVVSNICQAAD
RHLVQLVEWAKHIPHFTDLPIEDQVVLLKAGWNELLIASFSHRSMGVEDGIVLATGLVIHRSSAHQAGVG
AIFDRVLSELVAKMKEMKIDKTELGCLRSIVLFNPDAKGLNCVNDVEILREKVYAALEEYTRTTYPDEPG
RFAKLLLRLPALRSIGLKCLEYLFLFKLIGDTPLDSYLMKMLVDNPNTSVTPPTS

>Q8IPF2_DROME

MVRKSGRVKISSRDRVAVGNILLRGKVGGGRVAVAAAEEAEAGRRRRRRDSSASRTASSDESESHIMHAD
ALASAYPAASQPHSPIGLALSPNGGGLGLSNSSNQSSENFALCNGNGNAGSAGGGSASSGSNNNNSMFSP
NNNLSGSGSGTNSSQQQLQQQQQQQSPTVCAICGDRATGKHYGASSCDGCKGFFRRSVRKNHQYTCRFAR
NCVVDKDKRNQCRYCRLRKCFKAGMKKEAVQNERDRISCRRTSNDDPDPGNGLSVISLVKAENESRQSKA
GAAMEPNINEDLSNKQFASINDVCESMKQQLLTLVEWAKQIPAFNELQLDDQVALLRAHAGEHLLLGLSR
RSMHLKDVLLLSNNCVITRHCPDPLVSPNLDISRIGARIIDELVTVMKDVGIDDTEFACIKALVFFDPNA
KGLNEPHRIKSLRHQILNNLEDYISDRQYESRGRFGEILLILPVLQSITWQMIEQIQFAKIFGVAHIDSL
LQEMLLGGELADNPLPLSPPNQSNDYQSPTHTGNMEGGNQVNSSLDSLATSGGPGSHSLDLEVQHIQALI
EANSADDSFRAYAASTAAAAAAAVSSSSSAPASVAPASISPPLNSPKSQHQHQQHATHQQQQESSYLDMP
VKHYNGSRSGPLPTQHSPQRMHPYQRAVASPVEVSSGGGGLGLRNPADITLNEYNRSEGSSAEELLRRTP
LKIRAPEMLTAPAGYGTEPCRMTLKQEPETGY

>USP_CHOFU

MSSVAKKDKPTMSVTALINWARPAPPGPPQPQSASPAPAAMLQQLPTQSMQSLNHIPTVDCSLDMQWLNL
EPGFMSPMSPPEMKPDTAMLDGLRDDATSPPNFKNYPPNHPLSGSKHLCSICGDRASGKHYGVYSCEGCK
GFFKRTVRKDLSYACREERNCIIDKRQRNRCQYCRYQKCLACGMKREAVQEERQRNARGAEDAHPSSSVQ
VSDELSIERLTEMESLVADPSEEFQFLRVGPDSNVPPRYRAPVSSLCQIGNKQIAALVVWARDIPHFGQL
ELDDQVVLIKASWNELLLFAIAWRSMEYLEDERENGDGTRSTTQPQLMCLMPGMTLHRNSAQQAGVGAIF
DRVLSELSLKMRTLRMDQAEYVALKAIVLLNPDVKGLKNRQEVDVLREKMFSCLDDYCRRSRSNEEGRFA
SLLLRLPALRSISLKSFEHLYFFHLVAEGSISGYIREALRNHAPPIDVNAMM

>Q9U3Y3_AEDAL

MLKKEKPMLSVAAIIQAQGRWDRTLPLAGLAGFDAALVGHMGPLSPQDMKPDLKPDISLLNGSVGPFSLR
HNCGPASPGAFNQQVAAAPQQQQQNVNNSLNSQQQNSGGGGGGGTPTTPTNMSQQYPPNHPLSGSKHLCS
ICGDRASGKHYGVYSCEGCKGFFKRTVRKDLSYACREDKNCTIDKRQRNRCLYCRYQKCLACGMKREAVQ
EERQRSSKFSIKSEEINSTSSVRDVTIERITAAEQLSEQKSGDNAIPYLRVGSNSMIPPEYKGAVSHLCQ
MVNKQIYQLIDFARRLPHFTNLHRDDQVMLLRCGWNEMLIAAVAWRSMEYIETERSPDGSRISIRQPQLM
CLGPNFTLHRNSAQQAGVDTLFDRILCELGIKMKRLDVTRAELGVLKAIILFNPDIRGLKCQNGDDGMRE
KIYACLDEHCKQQHPSEDGRFAQLLLRLPALRSISLKCLDHLNFIRLLSDKHLDNFIIEMLDMPM

>EAR2_HUMAN

MAMVTGGWGGPGGDTNGVDKAGGYPRAAEDDSASPPGAASDAEPGDEERPGLQVDCVVCGDKSSGKHYGV
FTCEGCKSFFKRSIRRNLSYTCRSNRDCQIDQHHRNQCQYCRLKKCFRVGMRKEAVQRGRIPHSLPGAVA
ASSGSPPGSALAAVASGGDLFPGQPVSELIAQLLRAEPYPAAAGRFGAGGGAAGAVLGIDNVCELAARLL
FSTVEWARHAPFFPELPVADQVALLRLSWSELFVLNAAQAALPLHTAPLLAAAGLHAAPMAAERAVAFMD
QVRAFQEQVDKLGRLQVDSAEYGCLKAIALFTPDACGLSDPAHVESLQEKAQVALTEYVRAQYPSQPQRF
GRLLLRLPALRAVPASLISQLFFMRLVGKTPIETLIRDMLLSGSTFNWPYGSGQ

>Q9NG48_APIME

MMKKEKPMMSVTAIIQGTQAQHWSRGNTWLSLDNSNMSMSSVGPQSPLDMKPDTASLINPGNFSPSGPNS
PGSFTAGCHSNLLSTSPSGQNKAVAPYPPNHPLSGSKHLCSICGDRASGKHYGVYSCEGCKGFFKRTVRK
DLSYACREEKSCIIDKRQRNRCQYCRYQKCLAMGMKREAVQEERQRTKERDQSEVESTSSLHSDMPIERI
LEAEKRVECKMEQQGNYENAVSHICNATNKQLFQLVAWAKHIPHFTSLPLEDQVLLLRAGWNELLIASFS

HRSIDVKDGIVLATGITVHRNSAQQAGVGTIFDRVLSELVSKMREMKMDRTELGCLRSIILFNPEVRGLK
SIQEVTLLREKIYGALEGYCRVAWPDDAGRFAKLLLRLPAIRSIGLKCLEYLFFFKMIGDVPIDDFLVEM
LESRSDP

>USP_DROME
MDNCDQDASFRLSHIKEEVKPDISQLNDSNNSSFSPKAESPVPFMQAMSMVHVLPGSNSASSNNNSAGDA
QMAQAPNSAGGSAAAAVQQQYPPNHPLSGSKHLCSICGDRASGKHYGVYSCEGCKGFFKRTVRKDLTYAC
RENRNCIIDKRQRNRCQYCRYQKCLTCGMKREAVQEERQRGARNAAGRLSASGGGSSGPGSVGGSSSQGG
GGGGGVSGGMGSGNGSDDFMTNSVSRDFSIERIIEAEQRAETQCGDRALTFLRVGPYSTVQPDYKGAVSA
LCQVVNKQLFQMVEYARMMPHFAQVPLDDQVILLKAAWIELLIANVAWCSIVSLDDGGAGGGGGGLGHDG
SFERRSPGLQPQQLFLNQSFSYHRNSAIKAGVSAIFDRILSELSVKMKRLNLDRRELSCLKAIILYNPDI
RGIKSRAEIEMCREKVYACLDEHCRLEHPGDDGRFAQLLLRLPALRSISLKCQDHLFLFRITSDRPLEEL
FLEQLEAPPPPGLAMKLE

>O45117_CHITE
MLKKEKPMMTVAAIIEQAQNRWMDHPLVYNSRSLQFQGSYCIDSSLLGHMGPLSPPDLKPDISLLNCNNN
NNNTNNNNSNSSHNNLNHHNTSPLPVLGANTFSPIQSLNNNGPSSPLSSIGNGSGTIVTFNQIKLQSPSP
SNASSSSTLSGPLTTTPPATNANNILGMGNGNCGNTANGKQSQYPPNHPLSGSKHLCSICGDRASGKHYG
VYSCEGCKGFFKRTVRKDLSYACREERNCVIDKKQRNRCQYCRYQKCLNCGMKREAVQEERQRGGKSQKG
DDMSISSTQSLVNNGPGRDITVERLMEADQMSEARCGDKSIQYLRVAASNTMIPPEYRAPVSAICAMVNK
QVFQHMDFCRRLPHFTKLPLNDQMYLLKQSLNELLILNIAYMSIQYVEPDRRNADGSLERRQISQQMCLS
RNYTLGRNMAVQAGVVQIFDRILSELSVKMKRLDLDATELCLLKSIVVFNPDVRTLDDRKSIDLLRSRIY
ASLDEYCRQKHPNEDGRFAQLLLRLPALRSISLKCLDHLFYFQLIDDKNVENSVIEEFHKLN

>Q8CFY1_MOUSE
MRLSKTLAGMDMADYSAALDPAYTTLEFENVQVLTMGNDTSPSEGANLNSSNSLGVSALCAICGDRATGK
HYGASSCDGCKGFFRRSVRKNHMYSCRFSRQCVVDKDKRNQCRYCRLKKCFRAGMKKEAVQNERDRISTR
RSSYEDSSLPSINALLQAEVLSQQITSPISGINGDIRAKKIANITDVCESMKEQLLVLVEWAKYIPAFCE
LLLDDQVALLRAHAGEHLLLGATKRSMVFKDVLLLGNDYIVPRHCPELAEMSRVSIRILDELVLPFQELQ
IDDNEYACLKAIIFFDPDAKGLSDPGKIKRLRSQVQVSLEDYINDRQYDSRGRFGELLLLLPTLQSITWQ
MIEQIQFIKLFGMAKIDNLLQEMLLGGSASDAPHTHHPLHPHLMQEHMGTNVIVANTMPSHLSNGQMCEW
PRPRGQAATPETPQPSPPSGSGSESYKLLPGAITTIVKPPSAIPQPTITKQEAI

>O97120_SCHMA
MIMSIFANHDAIHMNHGVSDESVLYTHSYLIPPINQLENDILKPDQDMLTCVQSNRSHSPSQQIYDMLDS
TVLSDNPIQTIANSSCKSQTSLKSSSCDTSNADLGVDIKPSFMYNDNPQVISQSFLTDLHNHTDIHTVSS
DTNPNLCPIANSINSTHLQELVYNTPQKFPHMLPPDAHQHTVYSDNVVSEPFTRLLASGNSNETSTSYLP
QVTKVETNSLTVSQSPILLFVDPNKTKPESRECFPTQNPSELASQSSSATSVNTTNLNPICVICGDKASG
KHYGVISCEGCKGFFKRTVRKQLVYVCRESGQCPVDRRKRTRCQHCRFEQCLAKGMKKEAVQEERHRQPS
SNPVPLISKPPKSEKKGPGRRSTFGNKSAESIVTDQPPNINQXSTPNISITPTTTDCVQPNQVKSXSSTT
CIQSNNVLLSDXTDLPNLTLRCLLSAELSMDPKLAVSERGEAIYEDIPGDDDTGLHPLTIICQSIEQQLP
RIVNWARQLPVFSSVYLSFDDQFCLIKAAWPELVLISSAYHSTVIRDGLLLSIGRHLGREVAKSHGLGPL
VDRILHELVARFRDLSLQRTELALLRAIILFNPDANGLSSRHRVEAVREQLYSALHSYCTTNQPQDTSRF
TKLLLRLPPLRSIASKCLEHLVFVKLAAEDPTSCRLINLVEHGVWPIQEKSFELATLPSDSASTDSVPSQ

ITMVPTPQYIQHQDDNSLPTSSHTDLSYTHTLPNFHTVQNYPF

>O96562_TRICY
MAVQCNSSTANDVVSKEVSEETKLQIVKEEETSAPSCDSSVSAMSKEGGLAMVDSCLKEASPLESIHPYS
PLASDASGSSTSPIASSSLLQLPSLTADSQRPVQPCSVCSDKAYVKHYGVFACEGCKGFFKRSVRNNRKY
SCLGKRHCDTDKKSRNRCQYCRFQKCVQVGMKPEAVQDETLKKERKDYRKRLPSTPKGSPAEVTSSKVDL
PMIPIESIIAAETLVDPGIQTFASANTDPIRHVCLAADKQLASLAEWAKRLPHFRDLSIADQVVLLQWSW
PELLIGGFCHRSCAVKDGILLSTGLHLTRDNLKKAGVGAIIDKIFSEVIEKMQEIQMDRAEWGCLRAIML
FSPDAKGLTAIDQVENYRELYTSTLEDHVKRKHPEQPDRFTKVILRIPALKSIGLQALEHLYFFKLIGDV
PMDTFLLDMLEVDRS

>Q6ZMP8_HUMAN
MFRAGAEGAEKEPSPRPECRADPGPGLGFPLGSGLPWPSLLESPGGRILDIPCKVCGDRSSGKHYGVYAC
DGCSGFFKRSIRRNRTYVCKSGNQGGCPVDKTHRNQCRACRLKKCLEVNMNKDAVQHERGPRTSTIRKQV
ALYFRGHKEENGAAAHFPSAALPAPAFFTAVTQLEPHGLELAAVSTTPERQTLVSLAQPTPKYPHEVNGT
PMYLYEVATESVCESAARLLFMSIKWAKSVPAFSTLSLQDQLMLLEDAWRELFVLGIAQWAIPVDANTLL
AVSGMNGDNTDSQKLNKIISEIQALQEVVARFRQLRLDATEFACLKCIVTFKAVPTHSGSELRSFRNAAA
IAALQDEAQLTLNSYIHTRYPTQPCRFGKLLLLLPALRSISPSTIEEVFFKKTIGNVPITRLLSDMYKSS
DI

>O46174_BOMMO
MRSDVLAPSELEGTDYLLSSTMRLEDSFLQILDSDMQLETSSSEASAASSTTISQHCAICGDRATGKHYG
ASSCDGCKGFFRRSVRKNHLYTCRFSRNCVVDKDKRNQCRYCRLRKCFKAGMKKEAVQNERDRINCRRPS
YEEPTQANGLSVVSLLNAELLSRKVIDETVNVSDAEINNRKLAKINDVCDSIKQQLLILVEWAKYIPAFT
VLHLDDQVALLRAHAGEHLLLGCARRSLHLKDILLLGNNCIITKHNIDGRMDIDISMIGMRVMDEIVKPL
REIDIDDTEFACLKAIVFFDPNAKGLSQPQKIKQLRYQIQINLEDYISDRQYDGRGRFGELLLCLPPLQS
ITWQMIEQIQFAKLFGVAHVDSLLQEMLLGGATTEAVLEEPSPEPAAASPSPPLVPLVPLVPQLPQLPGN
DSAFLEPMPFKQEPNI

>O77099_AEDAE
MKSIVEVFCDYDRSDFSLGTSLNIEDTLFQHILDHDANDSLSPTHTSESRFTPEQSVDSNKNYTLDNCQS
SANNLCTICSDRATGKHYGAASCDGCKGFFRRTVRKNHSYTCRFSRQCVVDKDKRNQCRYCRLRKCFKAG
MKKEAVQNERDRISCRRPSMEDIDTSNGLSVKFLLLAENRSRHFGAALDDAYDGDGDLSNKRFASINDVC
DSMKQQLLILVEWAKSIPAFAELQLDDQVALLRAHAGEHLLLGLSRRSMHLEEMLLLGNNCIITKQSPDS
KMAPNLDISRIGARIIDELVSAIKDIKLDDSELACIKALVFFDPTVRGLNQPQKIKALRHQVLNNLEDYV
SDKQYDSRGRFGEILLLLPVLQSITWQMIQQIELAKMFGVAHIDSLLQEMLLGGETIENTAPPTPPLNSF
PNSSNSPPHMMSCDTTQRPSNAMEISRSNPTTTSSNCDAIDSESIDGANDMMAPAIIEDISNYNIPQTTN
SFQRDENVQNYIHPSNDDVYSNQYISPASSGMHPVHASANRHQQTNVLLPVNQLSREDYLKLERELKREP
EANGY

>Q26622_STRPU
MGMVSSQATPVEAYKVQQVQINDQTVEQLVRVQTSAGTTVTTIDAATAERLVKLREEDLIKFSAQGMSQA
QMMAKPGMTSPRPIELCAVCGDKASGRHYGAISCEGCKGFFKRSIRKHLGYTCRGNKDCQIIKHNRNRCQ
YCRLQKCLDMGMKSDSVQCERSPLKTRDKTPGNCAASTDKIYIRKDIRSPLTATPTFVTGSVLAGGDMKS

PQGNRQGLFDQGILLNVQTTPTSSPSASTSDSTTDLSTLASVVTSLANMNKKTEEGPSHSQQIYSPSQTL
QIISNGDQDVQGGGDNVSKAFDALTKALNTSGDSEAGDLSIDQSANGGSSEVELVKLDSPMLSDHHMQFK
LTTPSPMPQFLNVHYICESASRLLFLSMHWARSLPAFQVLSADTHTSMVQKCWSELFTLGLAQCAQAMAL
STILTAIVNHLQTSLQQDKLSADRVKAVMEHIWKLQEFVTTTSKLDVDQTEFAYLKTIVLFSPDHPGLSN
VRQIEKFQEMAISELHDYEAQTYPSKLNRFSKLLLRLPTLRLLSPAIMEELFFAGLIGNVQIDSIIPYIL
RMETADYNSAQITMSASPGSLIG

>Q8ST32_DIRIM
MDLCASDWFASNLITTPTSIDLTQNGIRNKRKRDTTSRIGIGDLEQPEEQFNHVLNNYFHFSNPTSTTWF
QQEQMLPSNTQLLRPITDRSQEFTVSNDEVSSTTPNMTLYASQKSSTSMNVGTITKHICAICGDRASGKH
YGVYSCEGCKGFFKRTVRKDLIYLCRENRNCIIDKRQRNRCQYCRYRKCQSMGMKREAVQEERQSSRADI
TKMLTSGGGQRHITTSQMEAESTSTYGGLEIQLERIAAAEEASEALFSNIKIESNDISSYESLEWQMIRM
VEWALMLPSFNEILVEDQARLIRFGWHELILADIAYRSTINKLLLWPERVMERNDAEILGYRIIFDRIIN
ELTVRLKDLDVDRMEIAALRCAILYNPSVSGLRNVSVVESLRDKVMVCLEDYCRQHHPAQTQRFAKLLLR
MPALRSLSLHCAENNGFIIAAPTIPVIFPEYYHLTTLMTHNMNIPTTKVLQGGY

>Q6PHZ7_HUMAN
MTSPSPRIQIISTDSAVASPQRIQGSEPASGPLSVFTSLNKEKIVTDQQTGQKIQIVTAVDASGSPKQQF
TLTSPDGAGTGKVILASPETSSAKQLIFTTSDNLVPGRIQIVTDSASVERLLGKTDVQRPQVVEYCVVCG
DKASGRHYGAVSCEGCKGFFKRSVRKNLTYSCRSNQDCIINKHHRNRCQFCRLKKCLEMGMKMESVQSKR
KPFDVQREKPSNCAASTEKIYIRKDLRSPLIATPTFVADKDGARQTGLLDPGMLVNIQQPLIREDGTVLL
ATDSKAETSQGALGTLANVVTSLANLSESLNNGDTSEIQPEDQSASEITRAFDTLAKALNTTDSSSSPSL
ADGIDTSGGGSIHVISRDQSTPIIEVEGPLLSDTHVTFKLTMPSPMPEYLNVHYICESASRLLFLSMHWA
RSIPAFQALGQDCNTSLVRACWNELFTLGLAQCAQVMSLSTILAAIVNHLQNSIQEDKLSGDRIKQVMEH
IWKLQEFCNSMAKLDIDGYEYAYLKAIVLFSPGKICGGDA

>Q8I748_SUBDO
MPILWKIVPQVDISTYASPSSTPASSPGYEQIQMLDMELKPFEDMSSCKLMHPTSPDPQSPATFNPRPHT
FGGMASYQSQQYMNDYKDPMYSPNTAAPPHYCADNRYMHNGVEPPPYGKNYGMINGSSFYFSNYPQSVEG
MSPSGQNLSLQQTICKICGDTASGNHFGVQSCEACKSFFRRSVRANARYACRGSRNCAIEKHTRNRCQYC
RLQKCIANGMRKEAVQEERTPQGAKLQRASTPTPLGFPNLNFIPPSIPYFPQSYDSPTSPVPRMLSHAAS
RMYGSLPNLKMEFDRSTPMSTPTGSLTPQQRSFTPPSLDASNSHVSVSVLVNADMQSEGQSEPIKTPASN
IKLEDLFEGLKTSLLKVIEWAKRIPAFVSLSLDDQVKLLKSSWCEHCTLKLAAQNGPKADTVLLANGLSC
NRDQIEDPEVRRVINRVFNELAYWLDYLNVDRVELACLKGILLFNPDAKGLSPASKKRVEIFQEQILQSL
ETRTKTMYPVSPRRFSKLLLRLAPLKAISLEVMQHMEVQRALGNTKMDNLFGELLDFE

>Q8MLA0_DROME
MATGRSLLFRVPWYVCLCVCAESAEPGVYWRLRLRLGLPTLAGPHTNTLTLTARTSSCRSIKKERIKASQ
QANAPPELPLKVSVDVNIIIAAHSQRRRIGLVRFHQRESEDRPLAVASPRLQINMEPTAMNPKKLHSPQR
HCYTPPPAPMHGQAPPPTSTGVAPPTQPPPPHPAAPNVPNGRLLSWNHSAAAAAAAAAAQAAANSMNHSS
AAEGSSMTRIKGQNLGLICVVCGDTSSGKHYGILACNGCSGFFKRSVRRKLIYRAGDDWAKNGCMDFDGF
RVGGMALSPDLIKVPPGQNKGREVDETEERNSCQAGTGRCVVDKAHRNQCQACRLKKCLQMGMNKDAVQN
ERQPRNTATIRPETLREMEHGRALREAAVAVGVFGPSNRPKSAGNSNLQTTRATVSALLRLRTPAAALTG
QFADGTIAAPQPPHLLNAIGCEPHGRRQLSHVQRSRRAPLAQGKGLRHGDGHQWQCLPLDQLEQQPLHRP

ELTGAGKRQGDKHRRRQCLLHDSIDVTNDNEEPHAVSRSDSSFIMPQFMSPNLYTHQHETVYETSARLLF
MAVKWAKNLPSFARLSFRDQVILLEESWSELFLLNAIQWCIPLDPTGCALFSVAEHCNNLENNANGDTCI
TKEELAADVRTLHEIFCKYKAVLVDPAEFACLKAIVLFRPETRGLKDPAQIENLQDQAHHTKTQFTAQIA
RFGRLLLMLPLLRMISSHKIESIYFQRTIGNTPMEKVLCDMYKN

>Q6P0E0_BRARE
MKISGSSLDLSVTEYGITLDPTFTMLEFDGLRVLPVQTDPLMPGHASASVPVVVPQQSSMSLCAICADRA
TGKHYGASSCDGCKGFFRRSVRKNHAYTCRFSRQCVVDKDKRNQCRYCRLRKCFRAGMRKEAVQNERDRI
SCRRENQGVGTLTIDVLMQAEAYTHQSLSQNLMRDVGSKKIAGVGDVCESMKQQLLLLVEWAKRIPEFCE
LSVDDRVALLRAHSAEHLILGVARRSLPYSDIILLGNDFIIPVGGSEQEMSKVAVRILEELVRPLKELNI
TDTEFVCLKTIVFFAPDCPGLQCAQAVRRLRFQAQVLLDEATSEQRGRFGELLLLLLTTLQSVAWQMLEQL
QLMRLLGRASVDSLLMEMLLGEEATRGSESLPATESNAEPLLFSTATESVLSRGILTEHIPMPNFTSVIL
PVPPSALAEDALLSTHTEVFTHGQTEDMPREMPNSLAMPTVHPCL

>Q9V7B3_DROME
MATGRSLLFRVPWYVCLCVCAESAEPGVYWRLRLRLGLPTLAGPHTNTLTLTARTSSCRSIKKERIKASQ
QANAPPELPLKVSVDVNIIIAAHSQRRRIGLVRFHQRESEDRPLAVASPRLQINMEPTAMNPKKLHSPQR
HCYTPPPAPMHGQAPPPTSTGVAPPTQPPPPHPAAPNVPNGRLLSWNHSAAAAAAAAAAQAAANSMNHSS
AAEGSSMTRIKGQNLGLICVVCGDTSSGKHYGILACNGCSGFFKRSVRRKLIYRCQAGTGRCVVDKAHRN
QCQACRLKKCLQMGMNKDDDSIDVTNDNEEPHAVSRSDSSFIMPQFMSPNLYTHQHETVYETSARLLFMA
VKWAKNLPSFARLSFRDQVILLEESWSELFLLNAIQWCIPLDPTGCALFSVAEHCNNLENNANGDTCITK
EELAADVRTLHEIFCKYKAVLVDPAEFACLKAIVLFRPETRGLKDPAQIENLQDQAHHTKTQFTAQIARF
GRLLLMLPLLRMISSHKIESIYFQRTIGNTPMEKVLCDMYKN

>Q9U9R6_SCHMA
MNFSETAAHQSECSIVVSQLSTKKLTTPVLCCISSNSNNIPIISQQSPNNNNNNNNNNNNVVFKTDNTKQH
IIDQNILTKTATTTSIVQPSELCHIQPILISSPVKNNNNNTHNLGTGSYIDSNCLSNQSNNDKSHSITPS
SPLLTNCNSPSTTLQSSTVCCLLRPSGRSTSTSSGSSCGSSCSGTGGGVSVSSGQYICSICSDRASGKHY
GVFSCEGCKGFFKRTVRKELTYICRDSQECQIDKRLRNRCQYCRYQKCLRAGMRREAVQEERQQQQLQSE
VQRSPTPPEQNCDLSVNSMIMSDTKITNAMNHSCLAEKQEIMLKTTNCTSSSSPHLLSNCSDSSINYFYS
ASNEKSQQPSINDNFNLTVNDAATYPPQELSLIGNKDSNNVTLPLADIHALKLPTTTSAAIPPPPDALEF
IRTAESTISSRRKQWLSAFNKQQCHAEIAKCFQDSMENLKWLENNFEKCTTNHLPLFDLVIWSSKLPYIC
QLSCGVHLDLLKSACMQLIIVNLVYWLANDHKPRSLSTSNSTSKLPDTTPTINSTDISNITDDPPENSIS
DISKDCTIQMKKINKSVPLDEKMDYYYSNFPEFHLLNNLTKPMDNNNNDSISSKPTNINDNSVDDDMIRK
RNTNVYKLIYNLAIKLRMLNLDPVELGCLKLILLLNPDSMTCLNNIRSLIELLRDQVYAGLEYYCNQVWP
NAPHGRMGRLLLKLSNFQSVAARIEKLICSNELNNLLNNLESIFSYLSKKKVDHSNYISTTTTTTTMSS
TTTSTSNSIHLEFS

>NR2E3_MOUSE
MSSTVAASTMPVSVAASKKESPGRWGLGEDPTGVGPSLQCRVCGDSSSGKHYGIYACNGCSGFFKRSVRR
RLIYRCQVGAGMCPVDKAHRNQCQACRLKKCLQAGMNQDAVQNERQPRSMAQVHLDAMETGSDPRSEPVV
ASPALAGPSPRGPTSVSATRAMGHHFMASLITAETCAKLEPEDAEENIDVTSNDPEFPASPCSLDGIHET
SARLLFMAVKWAKNLPVFSNLPFRDQVILLEEAWNELFLLGAIQWSLPLDSCPLLAPPEASGSSQGRLAL
ASAETRFLQETISRFRALAVDPTEFACLKALVLFKPETRGLKDPEHVEALQDQSQVMLSQHSKAHHPSQP

VRFGKLLLLLPSLRFLTAERIELLFFRKTIGNTPMEKLLCDMFKN

>Q5U3F3_BRARE
MSMVPFSSPTESSRSNSTDDSQDGKSPAPGKVLNTGLLCKVCSDTSSGKHYGIYACNGCSGFFKRSVRRR
LIYRCQAGTGMCPVDKAHRNQCQACRLKKCLQAGMNKDAVQNERQPRSTAQVRLDALDVDKDKEHLATTL
EPTSSSTCSVISRPLLGSSISSSISSTDGSQHSNSPKNGHRFMASLMTAETCAKLEPEDVDENIDVTSNE
PERSSPMYGSALYPSREPESVYETSARLLFMSVKWAKNLPVFSHLPFRDQVILLEEAWSELFLLCAIQWS
LPLDNCPLLSLPDLSPTGQGKGSPSASDVRVLQEVFSRFKPLQVDPTEFACLKAIVLFKPETRGLKDPEQ
VENLQDQSQVLLAQHIHTLYPSQVARFGRLLLLLPSLHFVSSERIEHLFFQRTIGNTPMEKLLCDMFKN

>NHR64_CAEEL
MIFFQNFDHTKLKIPKNQFLSKFQLFPTHFPFFDSKTVLFSEEMTLEEKEEVSTSTSQSPQSSSFENVFC
AICGDRATGKHYGAMSCDGCKGFFRRTIRKRHSYVCRFGEKCQVDKAKRNSCRKCRFDVCLRKGMRRDAV
QTERDRIRPANPLSNGSNGGIVPDDPLLDTLIRAEASTRGLRTTVITKTAEARKQATTNDVTDSMNQQLT
LMVEWAKVLEGFQRVDNITQVALLRHFSAQHLVMCAAFRSIHLSDAVWLTNETCLHKDSPKIPDMNRVAE
RIIDQVTNPMRSLHMNEIEYIALKAIAFFDPLAKGITSESYSDVEEMRQRILESFERHVRYVSPYKDMPL
RFANLLLLLPPMLAISRDLVEDVQLAKLFGLASIDNLMLELMLPNEGKNTTDKTSPPIMCHQ

>Q7YTB9_SACKO
MNKTATTSSRILDIPCKVCGDRSSGKHYGVYACDGCSGFFKRSIRRNRQYVCKNKGNGPCPIDKTHRNQC
RACRLKKCVQVDMNKDAVQHERGPRNSTIRKQMALYLKETTGRDDVTATIPSNLRHGPTFMNTLLGGESP
YMGITYPYELTPSYCSNPEAICETAARLLFMSVKWAKNVPAFLSLPFRDQLLLLEEGWRELFVLGAAQWQ
MCMEIGPLLAAAGLSTEHTNPEKIVAIMSEMRTFQEIIAKFKQMQVDATEYACLKGIIIFKSVFPDSPQE
IRGVRDFHGVATLQDQAQLTLSKYIHTKYPTQPFRFGKLLLMLPQLRAIRPSTIEELFFRKTIGNIPIER
LLCDMYKANDF

>Q8IPT4_DROME
MDGVKVETFIKSEENRAMPLIGGGSASGGTPLPGGGVGMGAGASATLSVELCLVCGDRASGRHYGAISCE
GCKGFFKRSIRKQLGYQCRGAMNCEVTKHHRNRCQFCRLQKCLASGMRSDSVQHERKPIVDRKEGIIAAA
GSSSTSGGGNGSSTYLSGKSGYQQGRGKGHSVKAESAATPPVHSAPATAFNLNENIFPMGLNFAELTQTL
MFATQQQQQQQQHQQSGSYSPDIPKADPEDDEDDSMDNSSTLCLQLLANSASNNNSQHLNFNAGEVPTA
LPTTSTMGLIQSSLDMRVIHKGLQILQPIQNQLERNGNLSVKPECDSEAEDSGTEDAVDAELEHMELDFE
CGGNRSGGSDFAINEAVFEQDLLTDVQCAFHVQPPTLVHSYLNIHYVCETGSRIIFLTIHTLRKVPVFEQ
LEAHTQVKLLRGVWPALMAIALAQCQGQLSVPTIIGQFIQSTRQLADIDKIEPLKISKMANLTRTLHDFV
QELQSLDVTDMEFGLLRLILLFNPTLLQQRKERSLRGYVRRVQLYALSSLRRQGGIGGGEERFNVLVARL
LPLSSLDAEAMEELFFANLVGQMQMDALIPFILMTSNTSGL

>TLL_DROME
MQSSEGSPDMMDQKYNSVRLSPAASSRILYHVPCKVCRDHSSGKHYGIYACDGCAGFFKRSIRRSRQYVC
KSQKQGLCVVDKTHRNQCRACRLRKCFEVGMNKDAVQHERGPRNSTLRRHMAMYKDAMMGAGEMPQIPAE
ILMNTAALTGFPGVPMPMPGLPQRAGHHPAHMAAFQPPPSAAAVLDLSVPRVPHHPVHQGHHGFFSPTAA
YMNALATRALPPTPPLMAAEHIKETAAEHLFKNVNWIKSVRAFTELPMPDQLLLLEESWKEFFILAMAQY
LMPMNFAQLLFVYESENANREIMGMVTREVHAFQEVLNQLCHLNIDSTEYECLRAISLFRKSPPSASSTE
DLANSSILTGSGSPNSSASAESRGLLESGKVAAMHNDARSALHNYIQRTHPSQPMRFQTLLGVVQLMHKV

SSFTIEELFFRKTIGDITIVRLISDMYSQRKI

>Q9NCL0_TRICA
MSEMQSVEGAMVHHLEPHRMQIKPQSPSSSSRILDIPCKVCGDFSSGKHYNIFACDGCAGFFKRSIRRNR
QYVCKAKDEGSCIIDKTHRNQCRACRLKKCQNVGMNKDAVQHERGPRNSTLRRQQMSSYYNESRVMMSPP
GNVLNLTMPKYEPNPSIIDPGPALPPTGFLCNNYPPLPQVPPLPLPPIFPPTMINPSAICESAAQLIFMN
VQWVRSIPAFTCLPLSDQLLLLEESWLDLFVLGAAQFLPLMDFSVLVEACGVLQQEPHRRDAFLKEVADF
QETLKKISQFQLDAHEFACLRAIVLFKTSFEKPSSSSNQEKTTTESAKISVIQDDAQMRLNKHVTTTYPK
QPLRFGKILLLVSSTFRTISGRTIEDLFFKKVIRDTPIVAIISNMYKNQILGNNNV

>O96680_DROME
MGTAGDRLLDIPCKVCGDRSSGKHYGIYSCDGCSGFFKRSIHRNRIYTCKATGDLKGRCPVDKTHRNQCR
ACRLAKCFQSAMNKDAVQHERGPRKPKLHPQLHHHHHHAAAAAAAAAHHAAAAHHHHHHHHAHAAAAHHA
AVAAAAASGLHHHHHAMPVSLVTNVSASFNYTQHISTHPPAPAAPPSGFHLTASGAQQGPAPPAGHLHHG
GAGHQHATAFHHPGHGHALPAPHGGVISNPGGNSSAISGSGPGSTLPFPSHLLHHNLIAEAASKLPGITA
TAVAAVVSSTSTPYASAAQASSPSSNNHNYSSPSPSNSIQSISSIGSRSGGGEEGLSLGSESPRVNVETE
TPSPSNSPPLSAGSISPAPTLTTSSGSPQHRQMSRHSLSEATTPPSHASLMICASNNNNNNNNNNNNNNG
EHKQSSYTSGSPTPTTPTPPPPRSGVGSTCNTASSSSGFLELLLSPDKCQELIQYQVQHNTLLFPQQLLD
SRLLSWEMLQETTARLLFMAVRWVKCLMPFQTLSKNDQHLLLQESWKELFLLNLAQWTIPLDLTPILESP
LIRERVLQDEATQTEMKTIQEILCRFRQITPDGSEVGCMKAIALFAPETAGLCDVQPVEMLQDQAQCILS
DHVRLRYPRQATRFGRLLLLLLPSLRTIRAATIEALFFKETIGNVPIARLLRDMYTMEPAQVDK

>P90892_CAEEL
MSDEDEPLNFSTSKATEESKEGILGVRSIFNTPLLFPPPMFNAGVISPHIAAALAMSFNQQRMNASVSPP
LDHTTISVNSFPSMGSVKTDSPPTASSPTLCCAVCGDVSSGKHYGILACNGCSGFFKRSVRRRLIYRCQA
GTGNCVVDKAHRNQCQACRLKKCLNKGMNKDAVQNERQPRNTATIRPALDMDPQNFFREYAGAVSAIMGH
SNMMKREDSPSSASDGKTEDEKKDSLQETTMSQLESVLQWAQQFRLFTVLTNSEKRQIILTQWPRLLCIS
LCEQAEDVSFDDHLTSLMLKFRRLDVSPAEFNCLKAITIFMKRELSLWRAGWDNRASIITVYPAGERGAR
LVAAALLLAEHSVMGFGNCVIPLALVFSTKSRYVIQRHAINSLPACVPGGTSAHPVLRCSMGSRREKVI

(3) 37 NR3

>ANDR_HUMAN
MEVQLGLGRVYPRPPSKTYRGAFQNLFQSVREVIQNPGPRHPEAASAAPPGASLLLLQQQQQQQQQQQQQ
QQQQQQQQETSPRQQQQQQGEDGSPQAHRRGPTGYLVLDEEQQPSQPQSALECHPERGCVPEPGAAVAAS
KGLPQQLPAPPDEDDSAAPSTLSLLGPTFPGLSSCSADLKDILSEASTMQLLQQQQQEAVSEGSSSGRAR
EASGAPTSSKDNYLGGTSTISDNAKELCKAVSVSMGLGVEALEHLSPGEQLRGDCMYAPLLGVPPAVRPT
PCAPLAECKGSLLDDSAGKSTEDTAEYSPFKGGYTKGLEGESLGCSGSAAAGSSGTLELPSTLSLYKSGA
LDEAAAYQSRDYYNFPLALAGPPPPPPPPHPHARIKLENPLDYGSAWAAAAAQCRYGDLASLHGAGAAGP
GSGSPSAAASSSWHTLFTAEEGQLYGPCGGGGGGGGGGGGGGGGGGGGGGGGEAGAVAPYGYTRPPQGLA
GQESDFTAPDVWYPGGMVSRVPYPSPTCVKSEMGPWMDSYSGPYGDMRLETARDHVLPIDYYFPPQKTCL
ICGDEASGCHYGALTCGSCKVFFKRAAEGKQKYLCASRNDCTIDKFRRKNCPSCRLRKCYEAGMTLGARK
LKKLGNLKLQEEGEASSTTSPTEETTQKLTVSHIEGYECQPIFLNVLEAIEPGVVCAGHDNNQPDSFAAL
LSSLNELGERQLVHVVKWAKALPGFRNLHVDDQMAVIQYSWMGLMVFAMGWRSFTNVNSRMLYFAPDLVF
NEYRMHKSRMYSQCVRMRHLSQEFGWLQITPQEFLCMKALLLFSIIPVDGLKNQKFFDELRMNYIKELDR

IIACKRKNPTSCSRRFYQLTKLLDSVQPIARELHQFTFDLLIKSHMVSVDFPEMMAEIISVQVPKILSGK
VKPIYFHTQ

>GCR_RAT
MDSKESLAPPGRDEVPGSLLGQGRGSVMDFYKSLRGGATVKVSASSPSVAAASQADSKQQRILLDFSKGS
TSNVQQRQQQQQQQQQQQQQQQQQQQPDLSKAVSLSMGLYMGETETKVMGNDLGYPQQGQLGLSSGETDF
RLLEESIANLNRSTSVPENPKSSTSATGCATPTEKEFPKTHSDASSEQQNRKSQTGTNGGSVKLYPTDQS
TFDLLKDLEFSAGSPSKDTNESPWRSDLLIDENLLSPLAGEDDPFLLEGNTNEDCKPLILPDTKPKIKDT
GDTILSSPSSVALPQVKTEKDDFIELCTPGVIKQEKLGPVYCQASFSGTNIIGNKMSAISVHGVSTSGGQ
MYHYDMNTASLSQQQDQKPVFNVIPPIPVGSENWNRCQGSGEDSLTSLGALNFPGRSVFSNGYSSPGMRP
DVSSPPSSSSAATGPPPKLCLVCSDEASGCHYGVLTCGSCKVFFKRAVEGQHNYLCAGRNDCIIDKIRRK
NCPACRYRKCLQAGMNLEARKTKKKIKGIQQATAGVSQDTSENPNKTIVPAALPQLTPTLVSLLEVIEPE
VLYAGYDSSVPDSAWRIMTTLNMLGGRQVIAAVKWAKAILGLRNLHLDDQMTLLQYSWMFLMAFALGWRS
YRQSSGNLLCFAPDLIINEQRMSLPCMYDQCKHMLFVSSELQRLQVSYEEYLCMKTLLLLSSVPKEGLKS
QELFDEIRMTYIKELGKAIVKREGNSSQNWQRFYQLTKLLDSMHEVVENLLTYCFQTFLDKTMSIEFPEM
LAEIITNQIPKYSNGNIKKLLFHQK

>Q6XLI8_CALJA
METKGYHSLPEGLDMERRWGQVSQAVEHSSLGSTERTDENNYMEIVNVSCVSGAIPNNSTQGSSKEKHEL
LPCLQQDNNRPGVLTSDIKTELESKELSATVAESMGLYMDSVRDADYYEQQNQQRSMSPAKIYQNVEQLV
KFYKENGHRPSTLSCVNRPLRSFMSDSGSSVNGGVMRAIVRSPIMCHEKSPSVCSPLNMTSSVCSPAGIN
SESSTTASFGSFPVHSPITQGTPLTCSPNVENRGSRSHSPAHASNVGSPLSSPLSSMKSSISSPPSHCSV
KSPVSSPNNVTLRSSVSSPANINNSRCSVSSPSNTNNRSTLSSPAASTVGSICSPVNNAFSYTASGTSAG
SSTSRDVVPSPDTQEKGAQEVPFPKTEEVESAISNGVTGQLNIVQYIKPEPDGAFSSSCLGGNSKINSDS
PFSVPIKQESTKHSCSGTSFKGNPTVNPFPFMDGSYFSFMDDKDYYSLSGILGPPVPGFDGNCEGSGFPV
GIKQEPDDGSYYPEASIPSSAIVGVNSGGQSFHYRIGAQGTISLSRSARDQSFQHLSSFPPVNTLVESWK
SHGDLSSRRSDGYPVLEYIPENVSSSTLRSVSTGSSRPSKICLVCGDEASGCHYGVVTCGSCKVFFKRAV
EGKCSWQHNYLCAGRNDCIIDKIRRKNCPACRLQKCLQAGMNLGARKSKKLGKLKGIHEEQPQQQPPPP
PPPPQSPEEGTTYIAPAKEPSVNTALVPQLSTISRALTPSPAMVLENIEPEVVYAGYDNSKPDTAENLLS
TLNRLAGKQMIQVVKWAKVLPGFKNLPLEDQITLIQYSWMCLSSFALSWRSYKHTNSQFLYFAPDLVFNE
EKMHQSAMYELCQGMHQISLQFIRLQLTFEEYTIMKVLLLLSTVPKDGLKSQAAFEEMRTNYIKELRKMV
TKCPNNSGQSWQRFYQLTKLLDSMHDLVNDLLEFCFYTFRESQALKVEFPAMLVEIISDQLPKVESGNAK
PLYFHRK

>PRGR_CANFA
MTERTGKDARAPHVAGGAPSPAPAAEPESRRRDGGRLRASQTSDAPRVAAAAAAAAAAASAAPSAPSDRL
LFSRRGQGADPGGKAQDAQPRPDVARADPRLEAASGAGADSPGPPRQDRGPLHGAPSTALRPAGPGQGRS
SPAWEPRSPRCPSGPEPPEDPRGARSSQGAACPLMSRPEGKAGDGCGTAGAHKGPPRGLSPSRQPLPLCP
GAHAWPGAAGKAATQPAALGVEDEGGFAAEGSPGPLLKGKPRPPAGPAAAAGAAPAAPGTAPGGTAPVPK
EDSRLPAPKGSLAEQDAPAPGCSPLATTMMDFIHVPILPLGSAFLAARTRQLLEAETYDAGAFAPPRGSP
SAPCAPLAAGDFPDCAYPSDAEPKDDAFPLYGDFQPPALKIKEEEEGAEAAARSPRPYLAAGPHSCVFAD
APPALPALPPLPPRAPSSRPGEGAPAAAAAAGCSASSASSPGPALECVLYKAEGAPPPQGPFAAAPCRVP
GAGACLLPRDGAAAAASAGAAGASPALYQPLGLGALPQLGYQAAVLKEGLPQVYQPYLNYLRPDSDASQS
PQYSFESLPQKICLICGDEASGCHYGVLTCGSCKVFFKRAMEGQHNYLCAGRNDCIVDKIRRKNCPACRL

RKCCQAGMVLGGRKFKKFNKVRVMRTLDAVALPQPVGIPNESQALSQRISFSPSQDIQLIPPLINLLMSI
EPDVIYAGHDNTKPDTSSSLLTSLNQLGERQLLSVVKWSKSLPGFRNLHIDDQITLIQYSWMSLMVFGLG
WRSYKHVSGQMLYFAPDLILNEQRMKESSFYSLCLTMWQIPQEFVKLQVSQEEFLCMKVLLLLNTIPLEG
LRSQNQFEEMRSSYIRELIKAIGLRQKGVVSSSQRFYQLTKLLDNLHDLVKQLHLYCLNTFIQSRALSVE
FPEMMSEVIAAQLPKILAGMVKPLLFHKK

>Q8TDS3_HUMAN
MTELKAKGPRAPHVAGGPPSPEVGSPLLCRPAAGPFPGSQTSDTLPEVSAIPISLDGLLFPRPCQGQDPS
DEKTQDQQSLSDVEGAYSRAEATRGAGGSSSSPPEKDSGLLDSVLDTLLAPSGPGQSQPSPPACEVTSSW
CLFGPELPEDPPAAPATQRVLSPLMSRSGCKVGDSSGTAAAHKVLPRGLSPARQLLLPASESPHWSGAPV
KPSPQAAAVEVEEEDGSESEESAGPLLKGKPRALGGAAAGGGAAAVPPGAAAGGVALVPKEDSRFSAPRV
ALVEQDAPMAPGRSPLATTVMDFIHVPILPLNHALLAARTRQLLEDESYDGGAGAASAFAPPRSSPCASS
TPVAVGDFPDCAYPPDAEPKDDAYPLYSDFQPPALKIKEEEEGAEASARSPRSYLVAGANPAAFPDFPLG
PPPPLPPRATPSRPGEAAVTAAPASASVSSASSSGSTLECILYKAEGAPPQQGPFAPPPCKAPGASGCLL
PRDGLPSTSASAAAAGAAPALYPALGLNGLPQLGYQAAVLKEGLPQVYPPYLNYLRPDSEASQSPQYSFE
SLPQKICLICGDEASGCHYGVLTCGSCKVFFKRAMEGQHNYLCAGRNDCIVDKIRRKNCPACRLRKCCQA
GMVLGGFRNLHIDDQITLIQYSWMSLMVFGLGWRSYKHVSGQMLYFAPDLILNEQRMKESSFYSLCLTMW
QIPQEFVKLQVSQEEFLCMKVLLLLNTIPLEGLRSQTQFEEMRSSYIRELIKAIGLRQKGVVSSSQRFYQ
LTKLLDNLHDLVKQLHLYCLNTFIQSRALSVEFPEMMSEVIAAQLPKILAGMVKPLLFHKK

>Q6QB13_DICLA
MDQGGLKRSAFFEMRSDIWGGRSWSRQSIQADTAGSLLQSAMHLPGPGSVPQPTVCTNGQGGTKDQGELG
GLFESPQHHVMSDMKEGEIIRMQKQQQQQQQQHQDMDIFNMEDSLPLLKQSISDLNQKSTSVISTSDTSV
LGNLPLPDLFPQHIKQEGNFSLDKDMETYSGHIGAGPCDMDGNSGRLIEDTEIWQDLDLPSSLPEISDFE
LDSKVAHLNNILHDSMGGSGPVSGLLKETKVPTWVTGVNCTNGKDGGHGTTPISGIHHPIQHHSHQQKPH
HLLQHQHQQHQLQHQQPGSLLSSVMIKEEKDPDDSFIHISTPGVVKQEKQEGAGFCQAQCLQSSLHGGGP
MSSPMGVGAGPGYHYRANPSPTVGLQDQKPFGMYSNLPLVGESWARGNRYGESSGIQRGDDGLPSAAAVA
AFSVSFSSSSPRAGETSSSVVPVQSKPSGQTHKICLVCSDEASGCHYGVVTCGSCKVFFKRAVEGWRARQ
NTDGQHNYLCAGRNDCIIDKIRRKNCPACRFRKCLQAGMNLEARKNKKLIKMKVQRPSGSSEPISNMPVP
VIPRCMPQLVPTMLSVLKAIEPEIIYSGYDSTLPDTSSWLMTTLNRLGGQQVISAVKWAKSLPGFRNLHL
DDQMTLLQCSWLFLMSFSLGWRSYEQCNGSMLCFAPDLVINKERMKLPFMTDQCEQMLKICNEFVRLQVS
YDEYLCMKVLLLLSTVPKDGLKSQAVFDEIRMTYIKELGKAIVKREENASQNWQRFYQLTKLLDSMQEMV
ESLLQICFYTFVNKTLSVEFPEMLAEIITNQIPKFKDGSVKPLLFHQK

>Q8JJ92_HAPBU
MDKGGVKKIAYRRDDHLSKLVYTESPEEGGLLRVAPHSAMSVTSPASVVLPSSSLMQPGQVPNGLNNSTL
PEELTSASVTATVGSLIDSPQPRGLTKDQRPQHQQLLQTQTTFGHQTLSENLSQLDASMADITQSSMDSL
IGGSDPNFFPLKTEDFSLDKGEQDPIDLDNAFEPIGKDMDVNQKLFSDNTLDLLQDFDLSGSPSDFYVGD
DAFLSSLADDSLLGVTSERDIKPAVVDSSNTTGAVPVALNGSSVTSPDLSSPTISTTTSLSPTTTLFAMV
KKEKDADFIQLCTPGVVKQEKTSGGQSYCQISGTASRDMAGTNAISVCGVSTSGGQTYHFGVNTLSSDTP
LQNEQKPVSSLFLPVTTIGGIWNRGQGIGNNSLVQRAGEGFSSSPSYPTSFTRQEGSTATSSTQGKSGTH
KICLVCSDEASGCHYGVLTCGSCKVFFKRAVEGQHNYLCAGRNDCIIDKIRRKNCPACRFRKCLMAGMNL
EARKLKKNRLKGVQQSNPPEVTPSPPVETRSLVPKCMPQLVPTMLSLLKAIEPDTIYAGYDSTLPDNFTR
LMTTLNRLGGRQVISAVKWAKALPGFRNLHLDDQMTLLQYSWLFLMTFSLGWRSYQQCNGNMLCFAPDLV

INEERMKLPYMTDQFEQMLKICSEFVRLQVSHDEYLCMKVLLLLSTVPKDGLKSQAVFDEIRMSYIKELG
KAIVKREENSSQNWQRFYQLTKLLDSMHEMVGGLLSFCFYTFVNKSLSVEFPKMLAEIISNQLPKFKAGS
VKPLLFHQR

>GCR_ONCMY
MDPGGLKHSKDKGLAFGKLSESSVEGSFSGDTGGSKSTTSTSLMHLPGSRPQPPARDSANGLNVTTTQME
LSTGGLTIEEAEVKVMEKAIRMQQPQKPQQNQQLFENFALLEASIADLNRSNTPGSSVLGRPHDLFSLKT
ENFSPMDKDRLDMGSVSFGQSQKDLDVNERLLGDNTMDILQDLDLPGSLSDLNEFYVSDEAAFLSSLSVE
DVLLEDGNMETKPIDCSNGGNCTNVDSADQQKQLLEAGVSMPVIKTEEDADTSFIQLCTPGVIKQENDRR
SFCQISSLDLPSTHNSAGSISGPSYPYGANTSTAVSLQQDQKPVFGLYPPLPSVSDSWNRGNGYATGSGM
SSSSFPVGFSSPKARPEASGSASSAPAKPSGPTHKICLVCSDEASGCHYGVLTCGSCKVFFKRAVEGWRA
RQNTDGQHNYLCAGRNDCIIDKIRRKNCPACRFRKCLQAGMNLEARKNKKLIRLKGQQTTMEPNPPPPDE
RACALIPKSMPQLVPTMLSLLKAIEPEAIYSGYDSTIPDTSTRLMTTLNRLGGQQVVSAVKWAKSLPGFR
NLHLDDQMTLLQCSWLFLMSFGLGWRSYQQCNGGMLCFAPDLVINDERMKLPYMTDQCEQMLKISTEFVR
LQVSYDEYLCMKVLLLLSTVPKDGLKSQAVFDEIRMTYIKELGKAIVKREENSSQNWQRFYQLTKLLDSM
QEMVGGLLQICFYTFVNKSLSVEFPEMLAEIISNQLPKFKDGSVKPLLFHALNHDTMP

>Q8JJ89_HAPBU
METKRYQSFFDGKDTENRWPQVPGTMEYCCSTEDSSLTGSDILMDIVNVNCPAGSPATDCKDNNTKKQEQ
PMLQLSQNQPFVLPHFNNSLLGHKQEMDSKELSKTVAESMGLYMNAAREADFAFNQQGANTSPGKLYPAC
GRPLEENQCGPTKSPKLKPFGFQQPSATPRECTAGTPVSSASMLASSLSCSPQTSSAISSPGGSNNMVSS
TTSPPTCFAPLCSSVSSPVSQTSCAATLANIKRRNSVTCSPVESSTVGSPPLTSPLNVMRSPMSSPQSMS
SVRSPPSCSTTCNIRSSVSSPTAGSCSSKTTNNCNTAMPSISSPATAGNMTASSPQNPSSGGFPVSSPAD
GLGLVQNDTSSPEAGLTRETDFKNFEFPKVEMVDGEVFNVGLDQMGMVKYIKNEPGTDFRSMCLGSSKCN
ASNTPFITQIKSEPNKSEGCMNQQPYGEQSPSIGLFSASETTYLSLRNNIDEYSLSGILGPPVSSVNGNY
ESDVFSNNVLSKGVKQEATDGSYYQENNSMSTSAIVGVNSGGHSFHYQIGAQGTMSFTRHDVRDQTNPLL
NLISPVTALMESWKSHPGISQGSLAARGEGYPGQNCISDGMSSSPLRQPSSTAKVCLVCGDEASGCHYGV
VTCGSCKVFFKRAVEGQHNYLCAGRNDCIIDKIRRKNCPACRVRKCLQAGMNLGARKSKKLKLKGVSEDL
QGSKDGQTATGGAGGYLSSEKELNASAANALVPHRPGVVTPFLPPSICSVLELIEPEEVYSGYDNSQPDT
TDHLLSSLNRLAGKQMVRMVKWAKVLPGFRSLPIEDQITLIQYSWMCLSSFCLSWRSYKHTNGQMLYFAP
DLIFNEERMQQSAMYDLCLGMRQVSQEFVRLQLTYDEFLSMKVLLLLSTVPKEGLKNQAAFEEMRVNYIK
ELRRSVGKATNNSGQTRQRFFQLTKLLDAMHDLVGNLLDFCFYTFRESQALKVEFPEMLVEIISDQIPKV
ESGLTHTIYFHKK

>P70048_XENLA
MEVHIGLGGVYKQPPGKMIRGAFENLFLSVREALQGERRALEGSQAPAGWSEAPGTHRWSEASPQDGTPL
NPWVTHPPAPWREAQAEAAPQNPAGRTEGAQFPALGDCPTELKEILGEQSGRILESEETPAEKEGFSGPP
EGISDSAKELCKAVSVSLGLSMEALEHLSAGAGEAQQRGDCMYAHPPDTHKCQVAEEDKSDTRDGPFRRS
SQSNFATGKSPEDGGGGGGGSSSAGGSEEKEQPCTDLALPEPAGGYRHRAMELTPSLTLYKPTAFMEESP
GYPSRDFYSFQMALAPHGRIKVENPMEYGGGAWGAAGRYSELSGFAHCGATAGWHTLFEEGQSSGSFAEA
GPYSYPRSHGPAGADGEFPSDAWYPAPTMIGRVPYSGPMKTEMAPWMEGYPGAFGEMRLEGGRDHLLPID
YYFPPQKTCLICGDEASGCHYGALTCGSCKVFFKRAAEGKQKYLCASRNDCTIDKFRRKNCPSCRLRKCY
EAGMTLGARKLKKLGNLKAQEELDGSSVQGEGSKELSPGMGIPQLEGYSCQPIFLNVLEAIEPVVVCAGH
DNNQPDSFALLLSSLNELGERQLVHVVKWAKALPGFRNLHVSDQMTVIQYSWMGLMIFAMGWRSFKNVNS

RMLYFAPDLVFNEYRMHKSRMYSQCVRLRHLSQEFGWLQITPEEFLCMKALLLFSIIPVEGLKDQKCFDE
LRMNYIKELDRVISCKRNNPASSSRRFFQLTKLLDSVQPIARELHQFTFDLFVKAQMVSVDYPEMMSEII
SVQVPKILSGRVKPLYFHIS

>Q5WP02_ONCMY
METKRYPSFFEGSTDTEKNRWSHVPSSAMDYCCSGAEEADSLNLNSNSDVLMDIVNVSCSPSSNTADSKE
SNNNNEEKKKPEQQPTLKLTQNQHQPFVLPLFNSSLHGRKPEMMDSKELSKTVAESMGLYMNAAREATDF
GGFGQQGGHCSPGKMYPAGVCGRPCLEDSQCAASGGSPKLKSPSTGFPKQPSSTPGDCCSSGTPAGSAVL
GLSLSCSPQTPSSISSPGGSNNLVSSTTSPTCFGGPFTCTTISNPVNQHNAAGPALAHNHVYRRNSATCS
PAGSSTVGSPPLTSPLNVMRSPISSPQSMSSVLSPPSCSTSTNMRSSSVSSPTGSTNNMRASLSISSPTT
GGPMAMSSSPRNPSGGGGGFAVSSPASELGLVQNDSNSPEGRRDQQDFKEFEFPKVESVDGEMFNVGLDH
MGMVKFIKNEPDTDYRSMCLGNGSNNTKCNQATGCPGNGSPFITQVKSEPNKDGGGCMNPQCYTEQQQQH
SMGLFQSGPSEITYLSLRDNIDEYSLSGILGPPGTEMNGSYEAGVFPHNLLSKVKQENNDGSYYQENNNN
VVPTSAIVGVNSGGHSFHYQIGAQGTMSFSRHDPRDHGTNPLLNLISPVTALMESWKSRPGMSQGPRGEG
YPGHGCMPDSMSSASLRHPSSTAKVCLVCGDEASGCHYGVVTCGSCKVFFKRAVEGRKSQHNYLCAGRND
CIIDKIRRKNCPACRVRKCLQAGMNLGARKSKKLGKLKGVNEDSTPTKEGGQTCPGSGGGYLSSGEKELS
TSPTNALVPHGPGGGLVTPYLPPSICSVLELIEPEVVFAGYDNTQPDTTDHLLSSLNQLAGKQMIRVVKW
AKVLPGFRGLPIEDQITLIQYSWMCLSSFSLSWRSYKHTNGQMLYFAPDLVFNEDRMQQSAMYDLCLGMR
QVSQEFVRLQLTYQEFLSMKVLLLLSTVPKEGLKNQAAFEEMRVNYIKELRRSVGKATNNSGQTWQRFFQ
LTKLLDAMHDLVGNLLDFCFYTFRESQALKVEFPEMLVEIISDQIPKVESGNTHTLYFHKK

>Q8QFV2_CARAU
MDVPGEACESPDAVFHRTYQSVFQNVCVARANNAESLDFSSSEKRGCLQETDSTEKRPSKLSPSKGIICC
PKKECESASSXMRSSIGSKSDTSLSCSGRADATESSGSRAGFLRGAESGQKSCATAEVHSRRDARVASSS
RACNTTTSSSSSSSISETARELCKAVSVSLGLAMESSELGDVEPHHVPPPLTTKSSSERIYLFGMPLLNC
SVSERXAGGKEREYALAAGRDRGAELRGRDRLLEMFKSGDLEQLAGEVTTLQCSSASRSHLTADAQGVHE
FASVSGDIANLSSEGTTGPDMDETRAASCQFEQLLPVSMAHFVQPELENGPNQSFAKPAEMSGEFAGPVE
DYVNLYNVKIKAEMMPRELNDTWAYPHRYAEDCNGQYGSPKQRTPYASGHDTHFICNPYEYGRNEALVPR
ERPPPEQWYPGGMLTRPPYPNMPCLKNEMGNWLDVTSLTDGRFDGRRSDIFPMEFFLPPQRTCLICSDEA
SGCHYGALTCGSCKVFFKRAAEGKQKYLCASRNDCTIDKLRRKNCPSCRLRKCFEAGMTLGARKLRKIGQ
MKGPEEVGPVQGPSETIQCLSPKPSLTFHSQLIFLNILESIEPEVVNAGHDHAQPDSAVALLTSLNELGE
RQLVKVVKWAKGLPGFRNLHVDDQMTVIQHTWMGVMVFALGWRSYKNANARMLYFAPDLVFNDHRMHISS
MYEHCVQMKHLSQEFVLLQVTQEEFLCMKALLLFSIIPVEGLKSQKYFDDLRLTYINELDRLINYGRKTN
CAMRFQQLTRLMDSLQPIVRKLHQFTFDLFVQARSLPTKVSFPEMIAEIISVQVPKILAGLSKPILFHK

>PRGR_CHICK
MTEVKSKETRAPSSARDGAVLLQAPPSRGEAEGIDVALDGLLYPRSSDEEEEEEENEEEEEEEEPQQREE
EEEEEEEDRDCPSYRPGGGSLSKDCLDSVLDTFLAPAAHAAPWSLFGPEVPEVPVAPMSRGPEQKAVDAG
PGAPGPSQPRPGAPLWPGADSLNVAVKARPGPEDASENRAPGLPGAEERGFPERDAGPGEGGLAPAAAAS
PAAVEPGAGQDYLHVPILPLNSAFLASRTRQLLDVEAAYDGSAFGPRSSPSVPAADLAEYGYPPPDGKEG
PFAYGEFQSALKIKEEGVGLPAAPPPFLGAKAAPADFAQPPRAGQEPSLECVLYKAEPPLLPGAYGPPAA
PDSLPSTSAAPPGLYSPLGLNGHHQALGFPAAVLKEGLPQLCPPYLGYVRPDTETSQSSQYSFESLPQKI
CLICGDEASGCHYGVLTCGSCKVFFKRAMEGQHNYLCAGRNDCIVDKIRRKNCPACRLRKCCQAGMVLGG
RKFKKLNKMKVVRTLDVALQQPAVLQDETQSLTQRLSFSPNQEIPFVPPMISVLRGIEPEVVYAGYDNTK

PETPSSLLTSLNHLCERQLLCVVKWSKLLPGFRNLHIDDQITLIQYSWMSLMVFAMGWRSYKHVSGQMLY
FAPDLILNEQRMKESSFYSLCLSMWQLPQEFVRLQVSQEEFLCMKALLLLNTIPLEGLRSQSQFDEMRTS
YIRELVKAIGLRQKGVVANSQRFYQLTKLMDSMHDLVKQLHLFCLNTFLQSRALSVEFPEMMSEVIAAQL
PKILAGMVKPLLFHKK

>Q800S7_ACASC
MSQTSRQLSCTKVWPRGEKIKTGDAASARSMAQNTEESPLRVSKHLTGNGAGRMRGSDNADPNTYGFGHM
IPLACGMEKHCCQTAAASQEELLNADCRVGDSRSFSACATISETARELCKAVSVSLGLAMECSDSSDMDA
ALPQCAANDHVRGEYLFGVGNAPLSCPGGQAAVSDYKCAEERPLHGHKQQQQQLMEMFKSSETAAHLQHL
TSARTPVDEQNFTMCKAEDSTPEETAHLDPVRAASCPYAQSAQPGSMTHFDPPAQERPWRLYKPPDEAGD
FMEVMESSFATTSGYQPEQYSMKIKCEDTESAGALWGGNHSFNDRYNSQCWGPRHCVSAHGAGADSALCN
PYERSAARPEHWYPGGMLRSPYPNSSYMKSEVGEWPDVPYNDPRFDASREHMFPMEFFFPAQRMCMICSD
EASGCHYGALTCGSCKVFFKRAAEGKQKYLCASKNDCTIDKLRRKNCPSCRLRKCFEAGMTLGARKLKKI
GQQKNPDGDHPPQEPAEVMPNISPKTGLSFNSQVVFLNILESIEPEVAYAGHDYGQPDSAATLLTSLNEL
GEKQLVKVVKWAKGLPGFRNLHVDDQMTVIQYSWMGVMVFGLGWRSYKNVNGRMLYFAPDLVFNEHRMHI
SSMYEHCIRMRHLSQEFLLLQITQEEFLCMKALLLFSIIPVEGLKSQKYFDELRLTYINELDRLISYRMS
ANCSQRFYQLTRLLDSLQMTVKKLHQFTFDLFVQAQSLPTKVSFPEMIGEIISVHVPKILAGLAKPILFH
D

>O93244_ONCMY
MEIPVGLGGVCDSTNLVFRGPFQNVFHNVQALPSNTTVTESLDFCSSKFSFLQNKQPWEMRQASRQSSRK
EIWSGTARNSDIEVREDDSVGFSRTLESVACRIHFAKSSKGNKAGFSSLNESDNPNANEYGSCRSGQQVS
NTEQQSCQPSVSHHGNELNHNSEACVASSCSSSACSTISKTARELCNAVSVSLGLTMDANEMTDLGPNHA
PSSVNHQSQGNYLFEVPLLKCSGAGENVSITEYTCPSERNAKPLQSDKQLVEMFQRSPANDLTEKVATIQ
HLSSGHPCTDKQEFRLNENRDDPTSKETDHFLNTGARSRSYHFDQLLPAHLAHFSQTETDRNSSHVFYKP
PAHVGETGETMEDKYADYLQQQYSVKIKYEAFNRHNELPEPSWDFQYRYNDNDNTQYGLNSYIAGPDSAL
ICNPHEYERGGGLVRRERPTPEQWYPGEMLGRMPYPNSPCIKNEVGDSLDVSYTDARFEGGSDHMYPMEF
FFPPQRTCLICAEEASGCHYGALTCGSCKVFFKRAAEGKQKYLCASRNDCTIDKLRRKNCPSCRLRRCFE
AGMTLGTRKLKKIVQLKSPEEDLPTQGPTDTIQGVSPQSGLSFHSQLVFLNILESIEPEVVHAGHDQCQP
DSAAALLTSLNELGERQLVKVVKWAKGLPGFRNLHVDDQMTVIQHSWMGVMVFGLGWRSYKNANCRLLYF
APDLVFNDHRMHISSMFDHCIRMRQLSQEFVLLQVTQEEFLCMKALLLFSIIPVDGLKSQKYFDELRLTY
INELGRVINYGRKSNCSQRLYQLTRLMDSLQPVVRKLQQFTFDLFVQAQSLPTKVNFPEMIAEIISVHLP
KILAGLAKPILFHK

>Q76LM5_ORYLA
MSQTSRQLSCSRNCSQGTRGKAGEAAGALTMARKSEESGGFFTKTRAGGRLRDSESGGYGSERGNPQVRD
METHCCQAAAAPKEKLLSADCRVGDSRAFSACATISETARELCKAVSVSLGLTVESSETSSVDAHAALPP
CAPSDQMSAECFYGVDCPGAPVAEYRCPDRAPHEHKYQLKMFRSSETPSPYHHPSSTRTSAQNFPPCEVD
DITSAAHCPGYVPPASSSDHLAHFGHTAAGRPYGGFEPPEGAGDVGDGAEEKSDGYAPEQYGVRVKCEGS
ESTGAPWASNYTFNKRYNTQFWGSRHCVNSQDAGANPAFISNPYEGSVVRPEQWYPGGMPRTPYSNSNYM
KSEVGEWLDVSYSDAGRFEAGREHMFPMEFFFPPQRTCLICSDEASGCHYGALTCGSCKVFFKRAAEGKQ
KYLCASKNDCTIDKLRRKNCPSCRLKKCFEAGMTLGARKLKKIGQQKTSEDDLPVQEPAELPHHTSPQSG
PNFNAQLVFLNILESIEPEVVNAGHDCGQPDSAASLLTSLNELGERQLVKVVKWAKGLPGFRNLHVDDQM
TVIQQSWMTVMVFALGWRSYKNVNGRMLYFAPDLVFNEHRMHISTMYEHCIRMRHLSQEFSLLQITQEEF

LCMKALLLFSIIPVEGLKSQKYFDELRLTYINELNRLINCQMATNCPQRFYQLTRLLDSLQMIVKKLHQF
TFDLFVQAQSLHTKVSFPEMIGEIISVHVPKILAGLAKPILFHK

>Q9YGV9_ANGJA
MEIPVGLGGVSDATNAVFRGPYQNVFHSLQVAFQSHGAVSRSLDFPNTKYGFLQNRHFCEMRQENKQPPC
KGLGLFYGNHRNSDTGTNEDDIACFSRQSDAEARPGIFSESSLDTGDEITCKLQSDNQGVRASGPLLPGS
SGCNSGQKSSLACTSQQRETTSQSDTCAGESCSEHQATTISETARELCNAVSVSLGLNLDLNDMNDLSSN
QISSTESDTSQAIYLFESSPGYTGVGLNALVRDCKCQSAREGTSTQQYDRGAMFKINRVNDLPLQPAPPR
HTSISDAKWDMEAGLCAQMEHKDSEKCANMDGAHSTSVFSQFDQLLPVNASHYSQNVSVRVEPQSDFSPI
LYKSPGIQKNAEKYNVQYDATIKSEDGKTTSEREWGFQYRYNESCSTPSAPPRHCAHQNRAGPYNQFFFN
PFEYAKRGVVSREGYSLEHGFPNNLARTPYSGSLKNELGDRLSGPYPDVSYRYEGERENVFPVEFFFPPQ
RTCLICGDEASGCHYGALTCGSCKVFFKRAAEGKQKYLCASINDCTIDKLRRKNCPSCRLKRCFAAGMTL
GARKLKKIGQMRAPEDGQGQGPAEAELSVSPKYDLGFHTQSMFLNILEAIEPEVVNAGHDYGQPDSAASL
LTSLNELGERQLVKVVKWAKGMPGFRSLYVDDQMTVIQHSWMAVMVFALGWRSFKNVKSRMLYFAPDLVF
NEHRMQVSTMYEHCIRMKNFSQEFAMLQVSQEEFLCMKALLLFSTIPVEGLKGQNFFDELRRSYINELDR
LVSFRSKSSCSERFQQLTRLLDSLQPVLKKLHQFTFDLFVQSQNLSNQVCFPEMISEIISVHVPKILAGT
VKPILFHK

>Q9DDU9_XENLA
MEEISQTPGDDPARTGTQVLPEDSSLDSLLYQRPEHEGSYLKGEGPSSPDNNDLENVLDSILDSSSASSH
LHSWTHFAAPEVSRVTPHMCPSDEAKALSIHHTSSLEETSLWAAPTLVERKEPGDSMPLKGKNSLEETKE
PNSSLTMDSDETVTSILPILPPDLNLNNMPILPLNPAYLAVRARQILGGEEGGQRSSPDTFQTSIPYANL
QPNMKQISYFYPDTQPDFRIKKESSTVSLYKAAAKESTLGQDYGSSPQNPSTPDPDPSLNFILYKNDSAC
DYDSFKISYGNTSEGSGCVLPSTSAQTIYQPLSLNGHQHITFQPTSMKETYLSEIQLPYVSYIRSDGNPD
PGSPFSFETFPQKICLICGDEASGCHYGVLTCGSCKVFFKRAIEGQHNYLCAGRNDCIVDKIRRKNCPSC
RLRKCCQAGMVLGGRKFKKFGRIKTGREIDTVVLQSPPTLSLECQQILIRRISNSSAQEIQFTPELLQIL
QSIEPEVVYAGYDNTQPETPSALLSSLNQLCERQLVCVVKWSKSLPGFRNLHIDDQITLLQYSWMSLMVF
ALGWRSYQHVSGQMLYFAPDLILNEQRMKDSSFYTLCLSMWQLPQEFMKLQVTHEEFLCMKALLLLNTIP
LEGLKSQTNFDEMRSNYIRELAKAIGLRHKGVIASSQRFYQLTKLMDSMHELVKQLHLYCLNTFLQSRSL
SVEFPEMMSEVISAQLPKILAGMVKPLVFHKK

>PRGR_RANDY
MEDKSKQCLQDPMCLGIESKHYIGSHGRHQVDFLEEMVDPGEISLDSLIFQRPEDEVSYQKKGGGPASSD
NNELEKVLDSILDTSSSSHLHPWDDYVIPEFQEISVTSASSTKTLGDLTTDKKEAGVWTGTTAVENKSIP
SLVPKEQDTLEYSIEGQLEGKGRRHGVLASRHPTLPPDGKMPMLPGSAVYLPGQPRHIEEDNDNYDQKHL
SPYPCHSSCSHHDGKIKQEDSLYAEYGMPSSNPATPGPDPSLEYICKSETAAAYDNFRSTLGKSSDKAIP
VLPTSSTTSQNLYQPLSLNDHQQNAYQSGTVNDAFVPQMHLSYEQYSRPDDPDQSSQYGFDALPRKICLI
CSDEASGCHYGALTCGSCKVFFKRAIEGQHNYLCAGRNDCIGDKIRRKNCPSCRLKKCCQAGMVLGGRKF
KKYNRLKPGRELDRIATSSPMECQQALTRRVSNSSAQEVQYFPELLQILQSIEPEVLYAGYDYTKPETPS
ALLCSLNQLCERQLLCVVKWSKSLPGFRNMHIDDQIILLQYSWMSLMVFAMGWRSYKHVSGQMLYFAPDL
VLNERRMKDSSFYSLCIAMRQLPQEFVKLQISQEEFLCMKALLLLNTIPLEGLKSQSYFDEMRSNYIREL
AKAISLRHKGVVASSQRFYHLTKVLDSMHELVKQLHLYCLNTFLQSRALSVEFPEMMTEVISAQLPKILA
GMAKPLIFHKK

>Q6RKQ3_ONCMY

MDSLIGDSDPNLFPMSMRTETAFSRDQNPMDMDQEGYIGKDQKLFSDNTLDLLQDFELTGSPSDFYVGDD
AFLSSLADDTLLGDESQDRGVSNSTSKPAATMTNSGSFSGSNTTTLNGSSLLACQDESNSSTSMTTTATF
PMVKMEKESGFIQLCTPGVIKQENTSAMRSSSCQMSGSTGGSTSSSPSELSSSSPSPISICGVSTSGGQS
YHFGGNSSINTTLASTTSGASQQKDQKPSVFSLYPPLVTVGEAWNNISYGDGASGMQGLSSPTSAFSSSY
ASSTSKLGGGAASCTTQGKAGTTHKVCLVCSDEASGCHYGVLTCGSCKVFFKRAVEGTGARGQHNYLCAG
RNDCIIDKIRRKNCPACRFRKCLLAGMNLEARKTKKLNRLKGVQQPTTAELTPRPLPEARSLVPKSMPQL
TPTMLSLLKAIEPDTIYSGYDGTLPDTSTRIMTTLNRLGGRQVVSAVKWAKALPGFRNLHLDDQMTLLQC
SWLFLMSFGLGWRSYQQCDGNMLCFAPDLVINQDRMKLPYMADQCEQMLKISSEFVRLQVSHDEYLCMKV
LLLLSTVPKDGLKSQAVFDEIRMSYIKELGKAIVKREENSSQNWQRFYQLTKLLDSMHEMVGGLLDFCFY
TFVNKSLSVEFPEMLAEIISNQLPKFKAGSVKPLLFHQK

>Q9IBD5_ANGJA

MDNNHQDKMESLYTPARASPTPDAESIKRARNLIKTYSESFGSYVEEIVRDDSNNIQSLSSVPLLMRNFG
NMDTLTCAPGSGSDSEIWKDFVVPGNSVVSKDTCGHVEISTKAENLSWAAAPLSREETLAKGTVTVPATV
PKESFTATSNTSSASGISIKDEQQSLLKMEPQSSDFCPYTANIPKLNPSYLTNTASTKQLGYGEQPDTSA
HSSPPAQKIVLDTARYSADLCSDNPLPQATNIKTDPCSSFSSFVGEGILTRASMGYSQQAIQTLPVHKSE
PFRLSASSAPADSPFWCQSTGPSEDHHLQIDYLSPAGLHSTCKYSSTNAYSSYLGVLPQRVCVICGDEAS
GCHYGVLTCGSCKVFFKRAVEGHHNYLCAGRNDCIVDKIRRKNCPACRLRKCYQAGMILGGRKLKKLGAL
KAAGLTQALVAHSLTPRRLSGDSQALMPLGCLPGVRELHLSPQIISVLESIEPEVVYSGYDNSQPDMPNM
LLNSLNRLCERQLLRIVKWSKSLPGFRSLHINDQMALIQYSWMSLMVFSLGWRSFQNVTSDYLYFAPDLI
LNEEYMRRSPIFDLCMAMQFIPQEFANLQVTKEEFLCMKVLLLLNTVPLEGLKSQPQFDEMRQNYIHELT
KAIHLRENGVVACSQRFYHLTKLMDHMHDIVKKLHLYCLSTFIQADAMRVEFPEMMSEVIASQLPRVLAG
MVKPLLFHTK

>Q6A4C2_DICLA

MCKRQSPAQSRQHCGPVVRPRISPAGFTELETLSPQRPSPPLRAPLSDMYPEESRGSGGAATVDFLEGTY
DYAAPTPAPTPLYSHSTPGYYSAPLDAHGPPSDGSLQSLGSGPTSPLVFVPSSPRLSPFMHPPTHHYLET
TSTPVYRSSVPSSQQPVSREDPCGTSDDSYSVGESGAGARAGGFEMAKDMRFCAVCSDYASGYHYGVWSC
EGCKAFFKRSIQGHNDYMCPATNQCTIDRNRRKSCQACRLRKCYEVGMMKGGVRKDRGRVLRRDKRRTRA
GDKEKASKDLEHRTVPPQDRRKHSSSSSSSSSAVGGGGKSSMIGMPPEQVLLLLQGAEPPMLCSRQKLSR
PYTEVTMMTLLTSMADKELVHMIAWAKKLPGFLQLSLHDQVQLLESSWLEVLMIGLIWRSIHCPGKLIFA
QDLILDRSEGDCVEGMAEIFDMLLATASRFRMLKLKPEEFVCLKAIILLNSGAFSFCTGTMEPLHDTAAV
QNMLDTITDALIHHISQSGCSAQQQSRRQAQLLLLLSHIRHMSNKGMEHLYSMKCKNKVPLYDLLLEMLD
AHRIQRPDRPAQSWSQADGEPPFTITTNNNNNNISGGGSTSSAGSSSGPRVSHESPSRGPTCPGVLQYGG
SRSDCTHIL

>Q8QGX5_ANGJA

MDSVRKDKSGATSPTASRPRDTFMKTDNDLTEGFSDSTSNYMAGSCSTANSMYSLSGVSSTMRNSGNALS
GVSSTMRNSGNVDTTRHGANSTNDTTESVAVAENAARYNDSREAGRTESKANNSPWTTSLADNEGLALPP
ASGSKASLSGVSSSSVGNCKFIKDEQDSSSSMEPQSPYFHPSGNITTSNSSYGTCEEDSATHHPPHMFTD
YNRTTALPLIPEITEDQFSFPYPVGEVVANSCLTGYGQRSPQNSLRFKSELCKLSLPTSSPESQSWCQST
GLSEDQHFETGYLPPGEIRNICETHNSLKSHSVYMGMLSQKFCLICGDEASGCHYGVLTCGSCKVFYKRA
VEGHQNYLCAGRNDCIVDKIRRKNCPACRLRKCYQAGMTLGGRKMKKLSALKVLGLTQSLAVRSPLGASY

EGQALATLPSMPMVRELQFTPQILSILENIEPETVYSGYDATQPETPHLLLNSLNGLCERQLLWIVRWSK
SLPGFRSLHINDQMTLIQYSWMSLMVFSLGWRSFQNVTREFLYFAPDLILGEEKMRNSPISDLCMAMQII
PQAFDNLQVTKEEFLCMKVLLLLNTVPLEGLRSQAQFDEMRHGYIRELTKAIQLTERGVMASSQRFYHLT
KLMDAMHEIVRKVNLYCLSTFIQAEAMQVEFPEMMSEVITSQLPKVLAGMVRPLLFHKK

>Q8BG65_MOUSE
MSICASSHKDFSQLRPTQDMEIKNSPSSLTSPASYNCSQSILPLEHGPIYIPSSYVESRHEYSAMTFYSP
AVMNYSVPSSTGNLEGGPVRQTASPNVLWPTSGHLSPLATHCQSSLLYAEPQKSPWCEARSLEHTLPVNR
ETLKRKLGGSGCASPVTSPSAKRDAHFCAVCSDYASGYHYGVWSCEGCKAFFKRSIQGHNDYICPATNQC
TIDKNRRKSCQACRLRKCYEVGMVKCGSRRERCGYRIVRRQRSASEQVHCLNKAKRTSGHTPRVKELLLN
SLSPEQLVLTLLEAEPPNVLVSRPSMPFTEASMMMSLTKLADKELVHMIGWAKKIPGFVELSLLDQVRLL
ESCWMEVLMVGLMWRSIDHPGKLIFAPDLVLDRSSEDPHWHVAQTKSAVPRDEGKCVEGILEIFDMLLAT
TARFRELKLQHKEYLCVKAMILLNSSMYPLATASQEAESSRKLTHLLNAVTDALVWVISKSGISSQQQSV
RLANLLMLLSHVRHISNKGMEHLLSMKCKNVVPVYDLLLEMLNAHTLRGYKSSISGSECCSTEDSKSKEG
SQNLQSQ

>ESR1_RAT
MTMTLHTKASGMALLHQIQGNELEPLNRPQLKMPMERALGEVYVDNSKPAVFNYPEGAAYEFNAAAAAAA
AGASAPVYGQSSITYGPGSEAAAFGANSLGAFPQLNSVSPSPLMLLHPPPHVSPFLHPHGHQVPYYLENE
PSAYAVRDTGPPAFYRSNSDNRRQNGRERLSSSSEKGNMIMESAKETRYCAVCNDYASGYHYGVWSCEGC
KAFFKRSIQGHNDYMCPATNQCTIDKNRRKSCQACRLRKCYEVGMMKGGIRKDRRGGRMLKHKRQRDDLE
GRNEMGTSGDMRAANLWPSPLVIKHTKKNSPALSLTADQMVSALLDAEPPLIYSEYDPSRPFSEASMMGL
LTNLADRELVHMINWAKRVPGFGDLNLHDQVHLLECAWLEILMIGLVWRSMEHPGKLLFAPNLLLDRNQG
KCVEGMVEIFDMLLATSSRFRMMNLQGEEFVCLKSIILLNSGVYTFLSSTLKSLEEKDHIHRVLDKINDT
LIHLMAKAGLTLQQQHRRLAQLLLILSHIRHMSNKGMEHLYNMKCKNVVPLYDLLLEMLDAHRLHAPASR
MGVPPEEPSQSQLTTTSSTSAHSLQTYYIPPEAEGFPNTI

>Q9W6F4_HAPBU
MSFHSVLEGTNSLTMNEGDRLDTTIYACPAYELSKAVSVSLGLDSVSSPPNNMNQSSSSAFAECDSTVAD
TSRGVPELRRTGNMNSDSSLLVLGDSSLREDDFGEVCQGIQQVSCMDLFGSGEMDGAQTVTRGSVISRYV
CRESNVFMNPTLELPATPQVAEVVPLKPPSSYYASSDLYRDFPPQMWCANERAYIDRSQPQPQPQPQRGE
SGEHNFLCKYCNCGQAPRGNRQECRCIWYGRGEKGGKGGMRATAQGYGQMESYPSAIPQGQSTFSTIKSE
PSVWMNCTDRSLRHEDFFPGVYLSERRVCQVCGDDASGCHYGAVTCGSCKVFFKRAAAGKQNHLCASRND
CTIDKLRRKNCASCRLKRCFMSGMSLKGRRLKGTGQASNGEEEQQPASWGHGEKEERAAKKDAVLESRNA
AVRAQGASQALVAAIPPPLHSCLSLLSILQAIEPALVNAGHDPAQPDSPVSLPTSLNELGERQLVTVVHW
AKAIPGFRDLYVDDQMSLIQLSWMGVMVFALGWRSYTLTNSSMLYFAPDLVFNDQRMQVSSMYEHCVRMK
LLSQRLCMLKVTQEEFLCMKALVLLSIMPVQGLKSQSCFDKLRTSYIKELDRLASHHGETTRTQRLFQLT
QLLDYLQSVVRKLHQFTYDLFIQAQSMQMHVSFPEMISEIVSVHVPKILSGMVKPILFHNAA

>Q6R7S4_SALSA
MSQYRRLPGLPSELPQSPMAASPLPERDSATLLKLQEVDPSRVGRGGRILSPIFSAPSPALPMEAHPICI
PSPYTDIGHDFNPLSFYSPTLLSYAGPALPDCPSAHQSLSPSIFWPPQAHVGPPLSLHHRPQSRPQQGQP
TRVSWAEPHALSESSKPLRKCSQESEETVISLEGKAELHFCAVCHDYASGYHYGVWSCEGCKAFFKRSIQ
GHNDYICPATNQCTIDKNRRKSCQACRLRKCYEVGMTKCGMRRDRSSYRGHQPRRVGRFFTRGTASGPKR

VLAEGGEPIKELRPTVLTPEQLIGRIMEAEPPEIYLQKDMRRPLTEANVMMSLTNLADKELVHMISWAKK
IPGFVDLCLFDQVHLLECCWLEVLMLGLMWRSVGHPGRLIFSPDLSLNREEGSCVQGFVDIFDMLLAATS
RFRELKLQREEYVCLKAMILLNSNMCLSSSEGSEELQSRSKLLRLLDAVTDALVWAIAKTGLSFQQQSAR
LAHLLMLLSHIRHVSNKGMDHLHCMKMKNVVPLYDLLLEMLDAHIMHSPRLPHQANSAGPCPEVSHPQPT
TSAAAPARHGPPAAEASLNSRSHWTAGTPVERQW

>Q7T3U4_9TELE
MSSSLGPASASAPPAMDSGNANRGDSPNTLPHLYTSPLGMDSRTICIPSPYVEACQDYSPPHGGEISHGA
LTLYSPVSSTVLGYTHPPVSESLVPLSPTIFWPPHTTHPALSLHCPPPLAYSETHAHTTWEDAKTHMINQ
GSSVLTHAKLFGQQLDGDGGLNPSPGVLGKGDAHFCAVCHDYASGYHYGVWSCEGCKAFFKRSIQGHNDY
ICPATNQCTIDKSRRKSCQACRLRKCYEVGMMKCGVRRERCSYRGARHHRNPQIRDSSGGALGVRRHSQP
QLEFPLSPTHPLFPSGDRAEGCGRSLSPEQLVNCILEAEPPQIYLREPIKKPYTEASMMMSLTNLADKEL
VLMISWAKKIPGFVELTLSDQVHLLECCWLDILMLGQMWRSVDHPGKLMFSPDLKLNREEGNCVEGIMEI
FDMLLATTSRFRELKLQREEYVCLKAMILLNSNNCSSLPQTPEDVESRRKVLRLLDSVTDALVWTISRTG
LSSQQQSIRLAHLLMLLSHIRHLSNKGIEHLSTMKRKNVVLLYDLLLEMLDANTSQSSRMLAAHTEASLQ
SDTQQTTEILHTSRQQPALKESYQEPWHSPQAEETVDKILHCSLHRVDMDTD

>Q762D6_CONMY
MMAKMTGSPGNELPLLQLQEVDSSKVGENGGSAGLLPSMYNGALPTLSMENHAVCIPSPYTDSGHDYTTL
TFYRPPILGHAGPAVPEGPDVHPSLSPSLFWPSHGHHGHVPQLALHFQQPLLYREPPHSPWGDPKPLEQG
HAQTSKLTKRVAESEEGASSGGGCFAGKGDMHFCAVCHDYASGYHYGVWSCEGCKAFFKRSIQGHNDYIC
PATNQCTIDKNRRKSCQACRLRKCYEVGMMKCGVRRERCTYRGARHRRLPQIRDLAGSGGGARAHRRGEG
PATQVHTSALTPEQLIHRIVEAEPPEIYLMKELKKPFTEDSMMRSLTNLADKELVLMISWAKKIPGFVEL
DLSDQVHLLECCWLELLMLGLMWRSVDHPGKLIFSPDLKLNRDEGSCVDGILEIFDMLLAATSRFRELKL
QREEYVCLKAIILLNPNLCSASATDNREETESRSKLLHMLDAVTDALVWTIAKRGLTFQQQSARLAHLLM
LLAHIRHVSNKGMEHLSNMKRKNLVPLYDLLLEMLDANTMHGTRMSESYSQPPPWPPTAQSQPSPQPSCS
GEGPCPPKESITAAVFGHGEDRVIPGLHTGTTSRRD

>ESR2_MICUN
MASSPGLDPHPLPMLQLQEVGSSKVSERPRSPGLLPAVYSPPLGMDSHTVCIPSPYTDSSHEYNHSHGPL
TFYSPSVLSYSRPPITNSPSSLCPSLSPSAFWPSHNHPTMPSLTLHCPESIVYNEPSPHAPWLESKAHSI
NASSSSIIGCNKSLVKRSEEGVEDMNSSLCSSAVGKADMHFCAVCHDYASGYHYGVWSCEGCKAFFKRSI
QGHNDYICPATNQCTIDKNRRKSCQACRLRKCYEVGMMKCGVRRERCSYRGARHRRGGLQPRDPTGRGLV
RVGLGSRAQRHLHLEAPLTPLAPILQAKHVHLSAMSPEEFISRIMDAEPPEIYLMEDLKKPFTEASMMMS
LTNLADKELVFMISWAKKIPGFVELSLADQINLLKCCWLEILMLGLMWRSVDHPGKLIFSPDFKLNREEG
QCVEGIMEIFDMLLAGTSRFRELKLQREEYVCLKAMILLNSNLCTSSPQTAEELESRNKLLRLLDSVIDA
LVWAISKMGLTTQQQTLRLGHLTMLLSHIRHVSNKGMDHLSTMKRKNVVLVYDLLLEMLDANTSSGGSQP
SSSPSSETYSDQHQYPQPPSHLHPGSEQTTADHAIVPPLGPTDDPILDGHLDAMPLQSSPPFQSLVVPHM
DTNDYIHPEQWSLGTGDAAPSVEPTDYITTERVVMETALVTQP

>Q90WH6_CLAGA
MSGEQTRTEALAGAKQRRRSELEGYSASLASLKLSPMYPEEEQQATGGMSSTAHYLDGTFDYASNPPNPS
VDYYSAAPDLQVAPEPQEENLQPLGANGSASPMVFVSSSPQLSPFLSHPPAGQHVAQQVPYYLEPSGTPV
YRSSVLASAGSRVELCSATSRQDVYTAVGASRPSGASGTSGAIGLVKEIRYCAVCSDYASGYHYGVWSCE

GCKAFFKRSIQGHNDYVCPATNQCTIDRNRRKSCQACRLRKCYEVGMMKGGYRKERGGRAMKHSRRHGGL
KERERGYNEAQSGSDAREAPPQDGRSTSGIGSAVAGVVCMAPEQVLLLLLRAEPPTLSSRQKLSRPYSEL
TIMSLLTNMADRELVHMIAWAKKVPGFQDLSLHDQVQLLESSWLEILMIGLIWRSIHSPGKLIFAQDLIL
DRTEGECVEGMAEIFDMLLATVARFRALKLKSEEFVCLKAIILLNSSAFSFYSSPVEPLRDGFMVQCMMD
NITDALVHYISQSGIPVQLQSRRQAQLLLLLSHIRHMSYKGMEHLYNMKCKNKVPLYDLLLEMLDAHRLR
PLGKVSKSWADRVSNMPVTSTLTQTATTTTTTTTNQQSSAPPCPADLPSNPPCTNQSPSP

>Q90ZE6_SQUAC
MATSPRKESSQMPELQELRPGTVENHIKNSPTGLTTQPQYSSTLPGLSEHGPVCIPSSYVENRHEFPTLA
FYSPSILGYSMPSDASGPDGTIVRQSLSPSMYWSSTGHVSPITLHCQQPIMYAEPPKSPWDDLRSGDQHL
LNRENLKKKPAPPGSSVNGVCSRRDAHFCAVCNDFASGYHYGVWSCEGCKAFFKRSIQGHNAYICPATNQ
CTIDKNRRKSCQACRLRKCYEVGMMKSETRRDRCSYRLTRQNRLSSAQAQHWSRGKRSGESDTTNISDTC
LNELAPEKLLSSLLEAEPPNVYSLNHPNKPYTEVSMMMSLTNLADRELVHMIAWAKKVPGFVELDLHDQV
QLLECCWLEVLMVGLMWRSIEYPGKLLFAPDLILDRDEGQCVEGILEIFDMLLAATSRFRDLKLQHEEYL
CLKAMVLLNSSMFPRSGVTEEHENREKLHKILDTITDTLIWCMSKSGIPPQQQATRLAHLLMLLSHIRHA
SNKGMEHLYSMKCKNVVPFYDLLLEMLDAHVIYSRTKPSDDHDCGQSKGDNL

>ERR1_HUMAN
MGLEMSSKDSPGSLDGRAWEDAQKPQSAWCGGRKTRVYATSSRRAPPSEGTRRGGAARPEEAAEEGPPAA
PGSLRHSGPLGPHACPTALPEPQVTSAMSSQVVGIEPLYIKAEPASPDSPKGSSETETEPPVALAPGPAP
TRCLPGHKEEEDGEGAGPGEQGGGKLVLSSLPKRLCLVCGDVASGYHYGVASCEACKAFFKRTIQGSIEY
SCPASNECEITKRRRKACQACRFTKCLRVGMLKEGVRLDRVRGGRQKYKRRPEVDPLPFPGPFPAGPLAV
AGGPRKTAPVNALVSHLLVVEPEKLYAMPDPAGPDGHLPAVATLCDLFDREIVVTISWAKSIPGFSSLSL
SDQMSVLQSVWMEVLVLGVAQRSLPLQDELAFAEDLVLDEEGARAAGLGELGAALLQLVRRLQALRLERE
EYVLLKALALANSDSVHIEDAEAVEQLREALHEALLEYEAGRAGPGGGAERRRAGRLLLTLPLLRQTAGK
VLAHFYGVKLEGKVPMHKLFLEMLEAMMD

>ERR2_HUMAN
MSSDDRHLGSSCGSFIKTEPSSPSSGIDALSHHSPSGSSDASGGFGLALGTHANGLDSPPMFAGAGLGGT
PCRKSYEDCASGIMEDSAIKCEYMLNAIPKRLCLVCGDIASGYHYGVASCEACKAFFKRTIQGNIEYSCP
ATNECEITKRRRKSCQACRFMKCLKVGMLKEGVRLDRVRGGRQKYKRRLDSESSPYLSLQISPPAKKPLT
KIVSYLLVAEPDKLYAMPPPGMPEGDIKALTTLCDLADRELVVIIGWAKHIPGFSSLSLGDQMSLLQSAW
MEILILGIVYRSLPYDDKLVYAEDYIMDEEHSRLAGLLELYRAILQLVRRYKKLKVEKEEFVTLKALALA
NSDSMYIEDLEAVQKLQDLLHEALQDYELSQRHEEPWRTGKLLLTLPLLRQTAAKAVQHFYSVKLQGKVP
MHKLFLEMLEAKAWARADSLQEWRPLEQVPSPLHRATKRQHVHFLTPLPPPPSVAWVGTAQAGYHLEVFL
PQRAGWPRAA

>Q5XTQ9_BRAFL
MTSQQDLYIDSTAVKKEPLSPAHNGMLTSDIVSATTEAHHNYARTVIPNGTDYHHQENGHHMQNGHNSSD
EENRYDSNSSSEILTNGAAEDRMMKCEYMLHSLPKRLCLVCGDVASGFHYGVASCEACKAFFKRTIQGNI
EYSCPATNECEITKRRRKSCQACRFTKCLKVGMLKEGVRLDRVRGGRQKYKRKIDADPSSYVQQAPALKK
PSNGPVNKIVAHLMVAEPEKLYAMPDPTTPDSELKTLTTLCDLADRELVVIIGWAKHIPGFSNLSLSDQM
SLLQSGWMEILILGLAFRSLHYDSRLVFAEDYIIDEEQSRAAGLEELSRHILRLVGRLKALGVEKEEFVV
LKAMALLNSDSVYVEDHEAVQKLQDVLHDALQDHDLNAHPSDSRRIGKILMMLPLLREVATKAVQHFYTI

KMEGQVPMHKLFLEMLDAKISEVVCKRCTSYCIGT

>Q5XTR0_BRAFL
MTSQQDLYIDSTAVKKEPLSPAHNGMLTSDIVSATTEAHHNYARTVIPNGTDYHHQENGHHMQNGHNSSD
EENRYDSNSSSEILTNGAAEDRMMKCEYMLHSLPKRLCLVCGDVASGFHYGVASCEACKAFFKRTIQGNI
EYSCPATNECEITKRRRKSCQACRFTKCLKVGMLKEGVRLDRVRGGRQKYKRKIDADPSSYVQQAPALKK
PSNGPGRTRHVHNSVPKKFVLAGGGTKTKRQVVTCPTNSHKQHGRKVNKIVAHLMVAEPEKLYAMPDPTT
PDSELKTLTTLCDLADRELVVIIGWAKHIPGFSNLSLSDQMSLLQSGWMEILILGLAFRSLHYDSRLVFA
EDYIIDEEQSRAAGLEELSRHILRLVGRLKALGVEKEEFVVLKAMALLNSDSVYVEDHEAVQKLQDVLHD
ALQDHDLNAHPSDSRRIGKILMMLPLLREVATKAVQHFYTIKMEGQVPMHKLFLEMLDAKISEVVCKRCT
SYCIGT

>Q8WS79_DROME
MKFYAGEGQGTNMSDGVSILHIKQEVDTPSASCFSPSSKSTATQSGTNGLKSSPSVSPERQLCSSTTSLS
CDLHNVSLSNDGDSLKGSGTSGGNGGGGGGTSGGNATNASAGAGSGSVRDELRRLCLVCGDVASGFHYG
VASCEACKAFFKRTIQGNIEYTCPANNECEINKRRRKACQACRFQKCLLMGMLKEGVRLDRVRGGRQKYR
RNPVSNSYQTMQLLYQSNTTSLCDVKILEVLNSYEPDALSVQTPPPQVHTTSITNDEASSSSGSIKLESS
VVTPNGTCIFQNNNNNDPNEILSVLSDIYDKELVSVIGWAKQIPGFIDLPLNDQMKLLQVSWAEILTLQL
TFRSLPFNGKLCFATDVWMDEHLAKECGYTEFYYHCVQIAQRMERISPRREEYYLLKALLLANCDILLDD
QSSLRAFRDTILNSLNDVVYLLRHSSAVSHQQQLLLLLPSLRQADDILRRFWRGIARDEVITMKKLFLEM
LEPLAR

(4) 7 NR4

>Q6ZMM6_HUMAN
MTSAQYKIKILIEGLHHGQRPGPAPPRQPGSFCWALKADGIMWLAKACWSIQSEMPCIQAQYGTPAPSPG
PRDHLASDPLTPEFIKPTMDLASPEAAPAAPTALPSFSTFMDGYTGEFDTFLYQLPGTVQPCSSASSSAS
STSSSSATSPASASFKFEDFQVYGCYPGPLSGPVDEALSSSGSDYYGSPCSAPSPSTPSFQPPQLSPWDG
SFGHFSPSQTYEGLRAWTEQLPKASGPPQPPAFFSFSPPTGPSPSLAQSPLKLFPSQATHQLGEGESYSM
PTAFPGLAPTSPHLEGSGILDTPVTSTKARSGAPGGSEGRCAVCGDNASCQHYGVRTCEGCKGFFKRTVQ
KNAKYICLANKDCPVDKRRRNRCQFCRFQKCLAVGMVKEVVRTDSLKGRRGRLPSKPKQPPDASPANLLT
SLVRAHLDSGPSTAKLDYSKFQELVLPHFGKEDAGDVQQFYDLLSGSLEVIRKWAEKIPGFAELSPADQD
LLLESAFLELFILRLAYRSKPGEGKLIFCSGLVLHRLQCARGFGDRIDSILAFSRSLHSLLVDVPAFACL
SALVLITDRHGLQEPRRVEELQNRIASCLKEHVAAVAGEPQPASCLSRLLGKLPELRTLCTQGLQRIFYL
KLEDLVPPPPIIDKIFMDTLPF

>Q98TQ3_ORYLA
MPCVQAQYGSSPQGASPASQSYSYHTAGEYSCDFLTPEFVKFSMDLTNTEITATTSLPSFSTFMDNYNTG
YDVKPPCLYQMPHSGEQSSIKVEDVQMHSYHQQSHLPPQSEEMIAHTGPMYFKPSSPHAPSTPNFQVQPN
HMWEDPGSLHSFHQNYVAATSHMMEQRKTPVSRLSLFSFKQSPPGTPVSSCQMRFDGPLHVSMTHDNPGA
HRGLDGQSFAVPSAIRKQAGLAFPHSLQLSHGHQLVDSQVPSPPSRGSPSNEGLCAVCGDNAACQHYGVR
TCEGCKGFFKRTVQKNAKYVCLANKNCPVDKRRRNRCQFCRFQKCLVVGMVREVVRTDNLKGRRGRLPSK
PKSPQEPSPPSPPVSLISALVRAHVDSNPSMSALDYSRFQANPDYQMTGDNTQHIQQFYDLLTGSMEIIR
GWAEKIPGFSDLPKQDQDLLFESAFLELFVLRLAYRSNPVEGKLIFCNGVVLHRLQCVRGFGEWVDAIVE
FSSNLQSLDIDISAFSCIAALAMVTERHGLKEPKRVEDLQNKIVNCLKDQVTFNSGGLNRPNYLSKLLGK

LPELRTLCTQGLQRIFYLKLEDLVPPPAIIDKLFLDTLPF

>O97726_PIG
MPCVQAQYSPSPPGSSYAAQTYGSEYTTEIMNPDYTKLTMDLGSTEITATATTSLPSFSTFMEGYSSNYE
LKPSCLYQMQPSGPRPLIKMEEGRAHGYHHHHHDHHHHHHHQQQQQQQQPPPQQQQPSIPPPSGPEDEVL
PSTSMYFKQSPPSTPTTPVFPQQAGALWEDALPSAQGCIAPGPLLDPPMKAVPTVAGARFPLFHFKPSPP
HPPAPSPAGGHHLGYDPTAAAALGLPLGAAAAAAAAAAGSQAAALEGHPYGLPLAKRAAALAFSPLGLTT
SPTTSSLLGESPSLPSPPNRSTASGEGTCAVCGDNAACQHYGVRTCEGCKGFFKRTVQKNAKYVCLANKN
CPVDKRRRNRCQYCRFQKCLSVGMVKEVVRTDSLKGRRGRLPSKPKSPLQQEPSQPSPPSPPVCMMNALV
RALTDSTPRDLDYSRYCPADQAAAGTDAEHVQQFYNLLTASIDVSRSWAEKIPGFTDLPKEDQTLLIESA
FLELFVLRLSIRSNTAEDKFVFCNGLVLHRLQCLRGFGEWLDSIKDFSLRLQSLNLDIQALACLSALSMI
TERHGLKEPKRVEELCNKITSSLKEHQSKGQALEPTEPKVLRALVELRKICTLGLQRIFYLKLEDLVSPP
SIIDKLFLDTLPF

>Q6INY4_XENLA
MPCIQAQHGSLSQCAGPCDNYVPDILNSEFGKFTMDLVNSEIAASTSLPSFSTFMDGYTGEFDAFLYQIP
SSNQQSSLKVEEFQVFGCYPGSFTNQLDETMSSSGSDYYGSPCSIPSPSTPGFQNPQLPTWECSYGAYSP
TQNYDNMRHWTEQQKNSISQQTFFSFGTPAHSPNMAANPLKIAPATHRLDQQLVDTDVFALAQNSSGGFP
AVPLGQAPGVLDSSVLLDSPLSPSKTRSPSSNEGRCAVCGDNASCQHYGVRTCEGCKGFFKRTVQKNAKY
ICLANKDCPVDKRRRNRCQFCRFQKCLVVGMVKEVVRTDSLKGRRGRLPSKPKQIAESSPVSLINSLVRA
HIDSIPSSSKLDYSKFQETVPLQLEKESSVDVQQFYDLLSGSLEVIRKWAEKIQGFVDLPKEDQDLLLES
AFLELFILRLAYRSRPEEGKLIFCNGVVLHRTQCVRGFGEWIDSIIEFSHSLQRMNIDVPSFSCLSALVI
VTDRHGLKEPKKVEELQSQIINCLKEHIPSSMNEQNRPNCLSKLLGKLPELRTLCTQGLQRIFYLKLEDL
VPPPPIVDKIFMDTLPF

>HR38_DROME
MMRDRLASLIVVKQEGGSNTSISHHQATAIKCEASLYTESSLFQEINNNSCYRQNLNAPTHQQSHTSHLQ
HAQQHQTHQQHPLLPPPLPTLPLIYPCRNLFPDGCDINHLACCSSSNSNSNCNSDSNSTSSSPGNSHFFA
NGNTCAAALTPAPPATEPRKIKPLGAGKLKVGKTDSNSDSNSNCDSRAAAAASTSATSATSATTLAATAA
ATAAAAEAGGAASAAAAAKISQVRLTNQATTSMLLLQPNSSFSSLSPFDNFSTQTASTTTTTSASAAGHH
QHHNHLLHQQHHNQQQQQQQQQQQQQQQQQQQEHLQQQHQQQLVSPQQHLLKSETLLSHEEDQLISNLTD
SSVVSHSELFSDLFFPSDSNNSLLSPTTSGYPDNPAEDLTSSIENLTKLTCLRDKRLSSIPEQQLSSEQE
QQLCLLSLRSSSDPAIALHAQQQQQQQQQQQQQQQQHQQQQQHLQLQLISPIGGPLSCGSSLPSFQETYS
LKYNSSSGSSPQQASSSSTAAPTPTDQVLTLKMDEDCFPPLSGGWSASPPAPSQLQQLHTLQSQAQMSHP
NSSNNSSNNAGNSHNNSGGYNYHGHFNAINASANLSPSSSASSLYEYNGVSAADNFYGQQQQQQQQSYQQ
HNYNSHNGERYSLPTFPTISELAAATAAVEAAAAATVGGPPPVRRASLPVQRTVSPAGSTAQSPKLAKIT
LNQRHSHAHAHALQLNSAPNSAASSPASADLQAGRLLQAPSQLCAVCGDTAACQHYGVRTCEGCKGFFKR
TVQKGSKYVCLADKNCPVDKRRRNRCQFCRFQKCLVVGMVKEVVRTDSLKGRRGRLPSKPKSPQESPPSP
PISLITALVRSHVDTTPDPSCLDYSHYEEQSMSEADKVQQFYQLLTSSVDVIKQFAEKIPGYFDLLPEDQ
ELLFQSASLELFVLRLAYRARIDDTKLIFCNGTVLHRTQCLRSFGEWLNDIMEFSRSLHNLEIDISAFAC
LCALTLITERHGLREPKKVEQLQMKIIGSLRDHVTYNAEAQKKQHYFSRLLGKLPELRSLSVQGLQRIFY
LKLEDLVPAPALIENMFVTTLPF

>Q9U4L1_AEDAE

MDEDCFKIAASPHHQSVGYGHHGYGHHAAAVHQPHYEFPQHTTAPPTQYSNPSPGGSFYPTPAYEQHAAV
SQPRNTTQTNTSTIVSFPQNYGAVTPQDSYSLPPFPTIAELHVSTTCYRRASLPLQRSESTSSSESPKPP
RVGIYKYSALPSASSSASSSPGYINNNNPNTINNNEVAAAAAAAAVAAAAVAGSATPAPRGPTPQSPSQL
CAVCGDTAACQHYGVRTCEGCKGFFKRTVQKGSKYVCLADKACPVDKRRRNRCQFCRFQKCLAVGMVKEV
VRTDSLKGRRGRLPSKPKSPQESPPSPPVSMITALVRAHVDTTPDLASLDYSQYREPGQNEPIISEAEKV
QQFYNLLTTSVDVIKQFADKLPGFSDLSSDDQELLFQSASLELFVLRLSYRARIDDTKMTFCNGVVLHKH
QCQRSFGDWLNAILEFSKSLHSMEIDISAFACLCALTLVTERYGLREPKKVELLQMKIISSLRDHVTYNS
EAQRKPHYFNRLLGKLPELRSLSVQGLQRIFYLKLEDLVPAPPLIENMFLASLPF

>NHR6_CAEEL
MEQLSIQTDELQDQFSNCSPASVDSSYSSCSSVEDEIEIYTRLVRNEEPLRRDFFREMSKNSSCSSSFDY
GEFGPSSSSRKGSKTTDADLDSLFHSLVETSDQVNTVPKPTKTEVESIPEEFEQKPSSSSHRLPSEMNAS
ITHIKSELDPTMQAFQMPHNDLFLATAAPHYNPFALSNDFMPNPLMPSFTSPFYPQHFPVSDSRRGSQGT
TSSSNNTGGTPSPHSSSLPTSPPQLQGFLRSFLNPDNLSTPTSFGVPSETALDADKMCAVCNDRAVCLHY
GARTCEGCKGFFKRTVQKNSKYTCAGNKTCPIDKRYRSRCQYCRYQKCLEVGMVKEIVRHGSLSGRRGRL
SSKTKLARSEDQPSPPLPLLALMGKAIEDHTNMTVVRQFMQPFDETIALRILHGELHATKKLLMAMPQIS
EIQPADFQILLSRSFFAIMAIRVANRCGNSTDTIMFESGELFSLNAFPACFQQIIRFMVDKARTFSSLVD
WEPQAFAAFIALQFLAGNTEHNVLGLTNKPLVDQVQSTIINALKDHCSGSQNKLAKIVRLTQEFDVFHAL
GLQALDILYPSHQLPEEFMFLINLTRAPLRSTDAPPACGSPVAPSGSSLFNFQMGPAAF

(5) 12 NR5

>Q9QWM1_RAT
MSASSITGDFQDFLKHGLPAIAPAPGSETPHSPKLEEKHREKRAGLPDRHRRPIPARSRLVMLPKVETEA
SGLVRSHGEQGQMPENMQVSQFKMVNYSYDEDLEELCPVCGDKVSGYHYGLLTCESCKGFFKRTVQNQKR
YTCIENQNCQIDKTQRKRCPYCRFKKCIDVGMKLEAVRADRMRGGRNKFGPMYKRDRALKQQKKALIRAN
GLKLEAMSQVIQAMPSDLTSAIQNIHSASKGLPLSHVALPPTDYDRSPFVTSPISMTMPPHGSLHGYQPY
GHFPNRAIKSEYPDPYSSSPESMMGYSYMDGYQTSSPASIPHLILELLKCEPDEPQVQAKIMAYLQQEQN
NRNRQEKLSAFGLLCKMADQTLFSIVEWARSSIFFRELKVDDQMKLLQNCWSELLILDHIYRQVAHGKEG
TIFLVTGEHVDYSSIISNTEVAFNNLLSLAQELVVRLRSLQFDQREFVCLKFLVLFSSDVKNLENFQLVE
GVQEQVNAALLDYTLCNYPQQTEKFGQLLLRLPEIRAISKQAEDYLYYKHVNGDVPYNNLLIEMLHAKRA

>P97782_MOUSE
MDYSYDEDLDELCPVCGDKVSGYHYGLLTCESCKGFFKRTVQNNKHYTCTESQSCKIDKTQRKRCPFCRF
QKCLTVGMRLEAVRADRMRGGRNKFGPMYKRDRALKQQKKAQIRANGFKLETGPPMGVPPPPPPPPDYML
PPSLHAPEPKALVSGPPSGPLGDIGAPSLPMSVPGPHGPLAGYLYPAFSNRTIKSEYPEPYASPPQQPGP
PYSYPEPFSGGPNVPELILQLLQLEPEEDQVRARIVGCLQEPAKSGSDQPAPFSLLCRMADQTFISIVDW
ARRCMVFKELEVADQMTLLQNCWSELLVLDHIYRQVQYGKEDSILLVSGQEVELSTVAVEAGSLLHSLVL
RAQELVLQLHALQLDRQEFVCLKFLILFSLDVKFLNNHSLVKDAQEKANAALLDYTLCHYPHCGDKFQQL
LLCLVEVRALSMQAKEQYLYHKHLGNEMPRNNLLIEMLQAKQT

>Q8JH98_CARAU
MDYSYDTDLEELCPVCGDKVSGYHYGLLTCESCKGFFKRTVQNNKRYTCAESQDCKIDKTQRKRCPFCRF
QKCLNVGMRLEAVRADRMRGGRNKFGPMYKRDRALKQQKKALIRASGLKMEATPPLMSSPQSDYSFSTAL
STPAPKNTHLNIGTSVAPTDYERNLYASSSLSLTIPIPAHTPLPAQYPAYPTLPSRAIKSEYPDHYTSSE

HYTSASSPESIPGYTYVDQTQVSTSPPLAPLGLTVPPLVLEFVRCEQDELQVQSKISAHLVHLQQEQNSR
SAAANQEQNTRLAAKQERLSTFGLMCHMADQTLFSIVEWARSCIFFKELKVGDQMKLLHNCWSELLVLDY
IARQLHHGKEDSVLLITGQEVELASLLAQAGVTLNGMIQRGQELVHHLRELQLDRRETACLKYLILFNPD
VKLLENQPYVESVYEQVNAALLEYTLCAYPQFPDRFSQILLRLPELRALSTQAEDYLCYKHLSGEVPCNN
LLIEMLHAKRTCI

>Q8UV26_CLAGA
MLEAQSKGSVMEFTTEEDLEELCPVCGDKVSGYHYGLLTCESCKGFFKRTVQNNKRYTCNQNQDCGIDKT
QRKRCPFCRFQKCLSVGMRLEAVRADRMRGGRNKFGPMYKRDRALKQQKRALIRASTFKLEHNPPLVPSN
QAEYPFPGSVPGLLAPLGPPDYDCPPACPPSLGVALHSYGSFPAQYQYTTPTVPGRSIKAEHPDPYSGSP
DSSLGYSYAEGCIAASPQTSPLNPAVPSLVLELLSCEPDEEQVRAKICAYLQQEQSGRGKLDKPRPSNLL
CVMADQTLFSIVEWARSCIFFKELKVGDQMQLLHNCWSELLLLDHVFRQVHHGRDNTLLLITGQEVELAG
VVSDSGLTLSSLVQRGQELARRLQLLQVDRREMACLKFLVLFNPNVKLLENQALVESVQEQVNSSLLEYT
LTSYPQHVDRFSQLVLRLPEVRALSAQAEEYLSSKHLSGDVPCNNLLIEMLHAKRATTI

>Q98ST8_BRARE
MNISSYPLDPPQPSVHLAAEYTQDHASQERPSTSQELKPDPESSATQEEGCPVCGDRVSGYHYGLLTCES
CKGFFKRSVQNNKRYTCAEAQNCPMDLAQRKRCPYCRFQKCVAVGMKKEAVRADRMRGGRNKFGPLYRRD
RQLKQQRGTYHQANIAPYRVKIEGPQQLVPALPHDFHVINPNPASLGTDHYHPTQHFHPNMSQSEFPVLL
DCTMPRDRTFSDPSLPIHTVCYPGIHGYPPDKREIPFNYNPASVTPPGPMSPTVLSTPAPTLTPASTPSS
TPTPSQSLTPTSTTAPSSFFLNQLLQAEPDEKQLCMRVLASLQREQACRGKHDRLNTFSIMCKMADQTLF
GLVEWARNCELFKELKVDDQMVLLQSCWSELLVLDHLCRQVAYGRDGSICLITGQQIEASTILSQAGVTL
SSLVSRAQDLVTKLRSLQLDREEFVCLKYLVLFNPDVKSVQNRRQVEQTQERVNKALMDHTMQTHPGHSD
KFGQLLLRLPEVRSISLQVEEYLYQRHLLGDFPCNSLLTEMLHAKHT

>Q6W954_EPICO
MEHRHDVGLEELCPVCGDKVSGYHYGLLTCESCKGFFKRTVQNNKKYICAEKQDCRIDITQRKRCPFCRF
QKCLHVGMRLEAVRADRMRGGRNKFGPIYKCDRALKLQRKALIQASGFRLDSNPALVSSTHQRDLTFTGD
LHLAPILHSTPLPSTQNSHMSYQPPSLCSLLPSSSPGATQYQCTSLSNWTIRSEHIYNCASSPGFTAGIN
SGKIYLRPFSPQGPGMPQLMIELLHCDPDELQLQTQITARLLQEHMSWEKHGNPSTFSLMCLMADQMLFS
IVEWARTSVFFKQLKLNDQVKLLHSCWSELLLLDIISRQVLCGKEGSLLPFTGQEMDLSDMASHAGLTLA
SLVQRGQELVEKLHILKVDRQEFACIKFLILFNPDVTPLEDHQLIESVQEQVEGALLEYTLCTSSHPPGH
FAHLLLCLSELRCLSSFAEDYLYCKHLSGEAPCNNLLTEMLHAKHSWA

>FTZF1_DROME
MDTFNVPMLAESSNTNYATEATSNHHHLQHQHQQHSHQQQQQQQLLMPHHHKDQMLAAGSSPMLPFYSH
LQLQQKDATATIGPAAAAAAVEAATTSANADNFSSLQTIDASQLDGGISLSGLCDRFFVASPNPHSNSNM
TLMGTATAATTTTTNNNNNNNNTNNNNNNNVEAKTVRPSNGNSVIIESVTMPSFANILFPTHRSANECIDP
ALLQKNPQNPNGNNSSIIVPPVEYHQLKPLEVNSSTSVSTSNFLSSTTAQLLDFEVQVGKDDGHISTTTT
TGPGSGSASGSGSGSGSGSGSIARTIGTATPTTTTSMSNTANPTRSSLHSIEELAASSCAPRAASPNSNH
TSSASTTPQQQQQQQHHMQSGNHSGSNLSSDDESMSEDEFGLEIDDNGGYQDTTSSHSQQSGGGGGGGGG
NLLNGSSGGSSAGGGYMLLPQAASSSGNNGNPNAGHMSSGSVGNGSGGAGNGGAGGNSGPGNPMGGTSAT
PGHGGEVIDFKHLFEELCPVCGDKVSGYHYGLLTCESCKGFFKRTVQNKKVYTCVAERSCHIDKTQRKRC
PYCRFQKCLEVGMKLEAVRADRMRGGRNKFGPMYKRDRARKLQVMRQRQLALQALRNSMGPDIKPTPISP

GYQQAYPNMNIKQEIQIPQVSSLTQSPDSSPSPIAIALGQVNASTGGVIATPMNAGTGGSGGGGLNGPSS
VGNGNSSNGSSNGNNNSSTGNGTSGGGGGNNAGGGGGGTNSNDGLHRNGGNGNSSCHEAGIGSLQNTADS
KLCFDSGTHPSSTADALIEPLRVSPMIREFVQSIDDREWQTQLFALLQKQTYNQVEVDLFELMCKVLDQN
LFSQVDWARNTVFFKDLKVDDQMKLLQHSWSDMLVLDHLHHRIHNGLPDETQLNNGQVFNLMSLGLLGVP
QLGDYFNELQNKLQDLKFDMGDYVCMKFLILLNPSVRGIVNRKTVSEGHDNVQAALLDYTLTCYPSVNDK
FRGLVNILPEIHAMAVRGEDHLYTKHCAGSAPTQTLLMEMLHAKRKG

>Q9NB03_AEDAE
MHEEASSTASSSTKPTNIIYTNINGGGGGGSVLSGGGGVTHLNGNLLITTTPTVQDASSSLYELVHHHHQ
QQPSFQQQHHQQVMTTTQPLHSTKIIITNQPLISTTTATSPAGVRTLQHHMQHQPQNQHHHVQSTSSCSS
SSGPTSSGTTTILINTDDGCSAFDTVAAAAAAIFESDPGGPSAEVLTADTAVAAAAAALIDLHPSSSCSS
AASVPTADGTLHIQELGIASTTVAGTTTSTSSSTSASSSTVADPGAVGAGGVALHEPIEIKIPSEGSFAE
GSDGGYHDNISQTQSNYLAIPPSGTTAIEPTSLNNLNVVSAIQCDQTDFKYFIEELCPVCGDKVSGYHYG
LLTCESCKGFFKRTVQNKKVYTCVAERQCHIDKTQRKRCPYCRFQKCLEVGMKLEAVRADRMRGGRNKFG
PMYKRDRARKLQIMRQRQLAIQALRGSIGGGVGIGQLGSDGASAQLQGIDYHQPYSNMHIKQEIQIPQVS
SLTSSPDSSPSPIAIALGQVNPQTTISLSSGVGGNGSSGGGSGGGGSNNSSGAGTGGSNSTSTAIQNQLS
ESKLWMSANSTTASPHSLSPKAFSFDSGTNPTSTADSGNPADTLRVSPMIRDFVQSIDDREWQTSLFSLL
QSQSYNQCEVDLFELMCKVLDQNLFSQVDWARNTIFFKDLKVDDQMKLLQNSWSDMLVLDHIHQRLHNGL
PDETTLHNGQKFDLLGLGLLGVPSLAEHFNELQNKLQELKFDVGDYICMKFLLLLNPVEVRGITNRKTVV
EGYENVQAALLDYTLTCYPSVPDKFSKLLSIIPEIHAMATRGEEHLYIKHCAGSAPSQTLLMEMLHAKRK

>Q8WSJ3_MANSE
MTMDQQTNLMSLNMSPFDLSPGPEGSGSGGGPSGASQQYVPQGAAYQCPPEQQSFGYANLDASYLFPTGP
GGEAGAYLPAAGAVCDQTDTKDVIEELCPVCGDKVSGYHYGLLTCESCKGFFKRTVQNKKVYTCVAERAC
HIDKTQRKRCPFCRFQKCLDVGMKLEAVRADRMRGGRNKFGPMYKRDRARKLQMMRQRQIAVQTLRGSLG
DGGLVLGFGSPYAAVPVKQEIQIPQVSSLTSSPESSPGPALLGTQPQPPQPPPPPAHDKWEAHSPHSPDA
FAFDAPTNAAATPSSTAEPTSTETLRVSPMIREFVQTIDDREWQNSLFGLLQSQTYNQCEVDLFELMCKV
LDQNLFSQVDWARNTVFFKYLKVDDQMKLLQHSWSDMLVLDHLHQRMHNGLPDETTLHNGQKFDLLCLGL
LGVPALADHFNELQNKLAELKFDVPDYICVKFLLLLNPEVRGIVNVKCVRDGYQTVQAALLDYTLTCYPT
VQDKFGKLVMVVPEIHALAARGEEHLYQRHCAGQAPTQTLLMEMLHAKRKPNGGEMVNRSAEHTSTLTDY
LETHENKETLIPPALRIRSRYY

>Q9Y0D1_METEN
MDSGLFPGVATTTLDYTSLAELPDTKEGIEELCPVCGDKVSGYHYGLLTCESCKGFFKRTVQNKKVYTCV
ADRSCQIDKTQRKRCPYCRFQKCLEVGMKLEAVRADRMRGGRNKFGPMYKRDRARKLQLLRQRQLSQQGI
LSGGARHTSSGVASRTPPGATPRRHPHTSTSRKRSRVPSSPRPHLRQTPRRPPWQLGGLVAGSGGRGRPS
ASGPVAPILAGPDPALWVTNAQSTAGGVTTGTPPTGGRRRWRRRRRRRGRRTHSTPLYPFTIIRELVETV
DDQEWQALLFSLLQNQTYNQCEVDLFELMCKVLQQNLFAQVDWPRNSCFFKDLKVDDQMKLLQHSWSDLL
ILDHLHQRIHNRLQDETTLPNGQKFDLLSLALLGTTQFADRFHAILNKLRDLNFDISDFVCVKFIILLNP
DSIADVRLLSDRRAVIAARPGATGIDGIYSQCLPRVSSGEIQKLMDLLPELHFLAENGEKYLYYKHINGA
APTQTLLMEMLHNQKEIEEVVTPCFQPSSAPHVYIKGGSVRVSSHHVRPEGGSPP

>Q9BPL0_SCHMA
MLRTSGIGINNNQTVNTSSVNLSSADRSIILVQQPVAGTSIHVCSTGTIVPGSSSGSGTTLVPLRQLGVS

TCGGNGLVSSAGSTRLIGSNIGIELFSNTGAVGSRSSQGQGGSMGLVTISANAGVSGSSSSGTPQRESTW
QQYVKQFTKLGPCPICGDKISGYHYGIFCCESCKGFFKRTVQNAKRYACHRPNASSRCEINVASRKKCPA
CRFLKCVDKGMRIEAIRSDRTRGGRSMYPGSRYLRQIAARVSGNRSTSGLAISSSCMEFSTSDLDGNMLG
GLTDQSIMSDDADQLSCSVVGLAAHTLGPDGLPASEDAGGGVYLDPGVLGTHDNDEEEIDSGSLRVEPNI
LECGSGIVGGTNLSTNVVSPYSGRQVIIGGSNESIPRTREQLPKIIRDILLVEETIEAEPEDALEIDSAV
ASETAAPEGVSDDEAAVYRALLNLADPRLYRTVRWSRALPDFSLLDTDDQILLIQNCWADLLCLDCCWRS
LPTPSEIRLTSSKCINLEAAREMGAEEIVERILQLTQSLTRLQLDIVEYACLKVIVLMQADLNNLKASSQ
VRSYQESVRRLLMDYVTKSSPDINDKFNKLINRIPELRKTSQAARLMLVDLDLSSYLSTNSLLMELLRSD
IQRYTPNNGTNNSTESTTTVSMQGSANSGLKTVSVTLGADTAENQEENTTVTIVPSSGVTDLLSLPNHTT
ITTVTESRLDETITSNSNNSLTTSGVDVISK

>FTF1B_DROME
MPNMSSIKAEQQSGPLGGSSGYQVPVNMCTTTVANTTTTLGSSAGGATGSRHNVSVTNIKCELDELPSPN
GNMVPVIANYVHGSLRIPLSGHSNHRESDSEEELASIENLKVRRRTAADKNGPRPMSWEGELSDTEVNGG
EELMEMEPTIKSEVVPAVAPPQPVCALQPIKTELENIAGEMQIQEKCYPQSNTQHHAATKLKVAPTQSDP
INLKFEPPLGDNSPLLAARSKSSSGGHLPLPTNPSPDSAIHSVYTHSSPSQSPLTSRHAPYTPSLSRNNS
DASHSSCYSYSSEFSPTHSPIQARHAPPAGTLYGNHHGIYRQMKVEASSTVPSSGQEAQNLSMDSASSNL
DTVGLGSSHPASPAGISRQQLINSPCPICGDKISGFHYGIFSCESCKGFFKRTVQNRKNYVCVRGGPCQV
SISTRKKCPACRFEKCLQKGMKLEAIREDRTRGGRSTYQCSYTLPNSMLSPLLSPDQAAAAAAAAAVASQ
QQPHQRLHQLNGFGGVPIPCSTSLPASPSLAGTSVKSEEMAETGKQSLRTGSVPPLLQEIMDVEHLWQYT
DAELARINQPLSAFASGSSSSSSSSGTSSGAHAQLTNPLLASAGLSSNGENANPDLIAHLCNVADHRLYK
IVKWCKSLPLFKNISIDDQICLLINSWCELLLFSCCFRSIDTPGEIKMSQGRKITLSQAKSNGLQTCIER
MLNLTDHLRRLRVDRYEYVAMKVIVLLQSDTTELQEAVKVRECQEKALQSLQAYTLAHYPDTPSKFGELL
LRIPDLQRTCQLGKEMLTIKTRDGADFNLLMELLRGEH

(6) 5 NR6

>NR6A1_MOUSE
MERDERPPSGGGGGGGSAGFLEPPAALPPPPRNGFCQDELAELDPGTNGETDSLTLGQGHIPVSVPDDRA
EQRTCLICGDRATGLHYGIISCEGCKGFFKRSICNKRVYRCSRDKNCVMSRKQRNRCQYCRLLKCLQMGM
NRKAIREDGMPGGRNKSIGPVQISEEEIERIMSGQEFEEEANHWSNHGDSDHSSPGNRASESNQPSPGST
LSSSRSVELNGFMAFRDQYMGMSVPPHYQYIPHLFSYSGHSPLLPPQARSLDPQSYSLIHQLMSAEDLEP
LGTPMLIEDGYAVTQAELFALLCRLADELLFRQIAWIKKLPFFCELSIKDYTCLLSSTWQELILLSSLTV
YSKQIFGELADVTAKYSPSDEELHRFSDEGMEVIERLIYLYHKFHQLKVSNEEYACMKAINFLNQDIRGL
TSASQLEQLNKRYWYICQDFTEYKYTHQPNRFPDLMMCLPEIRYIAGKMVNVPLEQLPLLFKVVLHSCKT
STVKE

>NR6A1_HUMAN
MERDEPPPSGGGGGGGSAGFLEPPAALPPPPRNGFCQDELAELDPGTISVSDDRAEQRTCLICGDRATGL
HYGIISCEGCKGFFKRSICNKRVYRCSRDKNCVMSRKQRNRCQYCRLLKCLQMGMNRKAIREDGMPGGRN
KSIGPVQISEEEIERIMSGQEFEEEANHWSNHGDSDHSSPGNRASESNQPSPGSTLSSSRSVELNGFMAF
REQYMGMSVPPHYQYIPHLFSYSGHSPLLPQQARSLDPQSYSLIHQLLSAEDLEPLGTPMLIEDGYAVTQ
AELFALLCRLADELLFRQIAWIKKLPFFCELSIKDYTCLLSSTWQELILLSSLTVYSKQIFGELADVTAK
YSPSDEELHRFSDEGMEVIERLIYLYHKFHQLKVSNEEYACMKAINFLNQDIRGLTSASQLEQLNKRYWY
ICQDFTEYKYTHQPNRFPDLMMCLPEIRYIAGKMVNVPLEQLPLLFKVVLHSCKTSVGKE

>Q66JK1_XENTR

MDTWEDDRVDQRACLICGDRATGLHYGIISCEGCKGFFKRSICNKRVYRCSRDKNCVMSRKQRNRCQYCR
LLKCLQMGMNRKAIREDGMPGGRNKSIGPVQISDEEIERIMSGQEFEEEANTSWSNNGDSDHSSPGNGVS
ESNQPSPVSTPSSSRSMELNGFGSLRDQYLGTPGPMHYQYLPHLFSYSAHPTLIPTQSRSLDPQSHTLIN
QLLTAEDIEPLSTPMLIEDGYKVTQSELFALLCRLADELLFRQITWVKKLPFFCDLSIKDYTCLLSTTWQ
ELILLSSLTTYSKQIFGDLADVTSKYSPSEDELHRFSEDGMEVMERLIYLFRKFSQLKVSNEEYVCMKAI
NFLNQDIQGISSVSQVEQLNKRYWYVCQDFTEYRYPHQPNRFPDLMMCLPEVRYIAGKLVNVPLEQLPLL
FKAFLHSCKTSLTKE

>P70033_XENLA

MDTWEDDRVDQRACLICGDRATGLHYGIISCEGCKGFFKRTICNKRVYRCSRDKNCVMSRKQRNRCQYCR
LLKCLQMGMNRKAIREDGMPGGRNKSIGPVQISDEEVERIMSGQEFEEEANTSWSNNGDSDHSSPGNAVS
ESNQPSPVSTPSSSRSMELNGFSSLRDQYLGTPGPTHYQYLPHLFSFSAHPTIIPAQSRSLDPQSHTLIN
QLLTAEDIEPLNTPMLIEDGYKVTQSELFALLCRLADELLFRQITWVKKLPFFCDLSIKDYTCLLSTTWQ
ELILLSSITTYSKQIFGDLTDVTSKYSPSEDELHRFSEDGMEVMERLIYLFRKFSQLKVSNEEYVCMKAI
NFLNQDIQGISSASQVEQLNKRYWYVCQDFTEYRYPHQPNRFPDLMMCLPEVRYIAGKLVNVPLEQLPLL
FKAFLHSCKTIVTKE

>Q9PU65_BRARE

MDSWEDDQAEQRSCLICGDRATGLHYGIISCEGCKGFFKRSICNKRVYRCSRDKNCEMSRKQRNRCQYCR
LLKCLQMGMNRKAIREDGMPGGRNKSIGPVQISDEEIERIMSGQEFKDEANMPEHTWGNNGDSDHSSPGN
GVSDGNQPSPVSTLSSNRSVELNGYTTALREQYIGNAMAQHYQFLPHLFGYAAQPRSLYPQSHTLMRQLV
AAEDLAPLGTPMLIEDGYRVTQVELFALLCRLADELLFRQISWIKKLPFFCDLSIEDYTRLLSATWQELI
LLACLTVYSAQVLGDLANVTHKYTPSDDELHSFSEDGMEVMEKLIYLFRKFHQLKVSNEEYACMKAINFL
NQDIRGLTNVTQLEQLNKRYWYVCQDYTEYKYPHQPKRFPEIMMCLPEIRCIAGKLVNIPLEQLPLLFKA
VLHSCKSSLSSYRTSSSPCVTKGTAPAN

(7) 12 NR0

>KNIR_DROVI

MNQTCKVCGEPAAGFHFGAFTCEGCKSFFGRSYNNISTISECKNDGKCIIDKKNRTTCKACRLRKCYNVG
MSKGGSRYGRRSNWFKIHCLLQEHEQAAAAAAGKAPGHATGSPMSSPGFGDLAAHLQQQQQQHQQQQQQQ
HQHQQQQQRHPHLPPLLGYTGYHLPEHFGARHPADAAAAAAALPFFSMMATPQSAFQLPPHLLFPGYHAS
AAAAAAADAAYRQEMYKHRQSVDSAASAESHSRYTPPTVATVPQQSQPQPAASPIDVCLGADDDVQSQHS
HSHSHSQSQSPHTIHTPVAIRATPPQQLLPGLTTASHSSSASPTPSKSQSSSPLSFTAKMQSLSPVSVCS
IGGETAAANAAASAAAAAQDGPMDLSMKTSRSSVHSFNDSDVCSLQDEHELAARRKYYQLEAECTTITNT
TNSCSSSTSTSSSNSSTSSTEAAVAVKRQKLNPIGGESPPFGGFAVTHNASSAMRSIFVCV

>KNIR_DROME

MNQTCKVCGEPAAGFHFGAFTCEGCKSFFGRSYNNISTISECKNEGKCIIDKKNRTTCKACRLRKCYNVG
MSKGGSRYGRRSNWFKIHCLLQEHEQAAAAGKAPPLAGGVSVGGAPSASSPVGSPHTPGFGDMAAHLHH
HHQQQQQQQVPRHPHMPLLGYPSYLSDPSAALPFFSMMGGVPHQSPFQLPPHLLFPGYHASAAAAAASAA
DAAYRQEMYKHRQSVDSVESQNRFSPASQPPVVQPTSSARQSPIDVCLEEDVHSVHSHQSSASLLHPIAI
RATPTTPTSSSPLSFAAKMQSLSPVSVCSIGGETTSVVPVHPPTVSAQEGPMDLSMKTSRSSVHSFNDSG

SEDQEVEVAPRRKFYQLEAECLTTSSSSSSHSAAHSPNTTTAHAEVKRQKLGGAEATHFGGFAVAHNAAS
AMRGIFVCV

>KNRL_DROME
MMNQDNPYAMNQTCKVCGEPAAGFHFGAFTCEGCKSFFGRSYNNLSSISDCKNNGECIINKKNRTACKAC
RLKKCLMVGMSKSGSRYGRRSNWFKIHCLLQEQQQQAVAAMAAHHNSQQAGGGSSGGSGGGQGMPNGVKG
MSGVPPPAAAAAALGMLGHPGGYPGLYAVANAGGSSRSKEELMMLGLDGSVEYGSHKHPVVASPSVSSPD
SHNSDSSVEVSSVRGNPLLHLGGKSNSGGSSSGADGSHSGGGGGGGGGVTPGRPPQMRKDLSPFLPLPFP
GLASMPVMPPPAFLPPSHLLFPGYHPALYSHHQGLLKPTPEQQQAAVAAAAVQHLFNSSGAGQRFAPGTS
PFANHQQHHKEEDQPAPARSPSTHANNNHLLTNGGAADELTKRFYLDAVLKSQQQSPPPTTKLPPHSKQD
YSISALVTPNSESGRERVKSRQNEEDDEARADGIIDGAEHDDEEEDLVVSMTPPHSPAQQEERTPAGEDP
RPSPGQDNPIDLSMKTTGSSLSSKSSSPEIEPETEISSDVEKNDTDDDDEDLKVTPEEEISVRETADPEI
EEDHSSTTETAKTSIENTHNNNNSISNNNNNNNNNNNSILSDSEASETIKRKLDELIEASSENGKRLRLE
APVKVATSNALDLTTKV

>TRX_DROVI
MGRSKFPGNPSKSINRKRISVLQLEDEAASAAAAAAAATAATTEQHQQSEQSAGSSASREKGNNCDNDDD
DNAPSGAATSGNRGASSGASDAAPEGGNSYGNGSSTGSKTTNGGNVNGGSHHKSATAPAELKECKNQGNQ
IEPNNCIAAEPDGTEDTNNDDDDDSSNDKKPTAAAAAAAAAAFVPGPSALQRARKGGNKKFKNLNLARPE
VMLPSTSKLKQQQQQLQLNCPSASASSLSSSAAAAAAAAAAPTTTTTTASASATLTATATSTSTSSLPGT
PLSVIAGGGGGAAAAALLLANPFASVETKVVEVNAAATAAATAAATAAAGAGEDVGMLKASIEMANEAGL
EAPAVAVKSSGSSPNPNHNPNAVAGSTSAAAPGAPTATKQKKTVTFKNILETSDDKSVVKRFYNPDNRVP
LVSIMKKDSLNRPLNYCRGSEFIVRPSILSKILNKNSNIDKLNSLKFRSVHASSNSIQESSSSTTNLFGS
GLSRAFGAPIDDEDAVSGGVTFRKQEPQHKTPEDNDDDGSASSDAIEDDEDIDDDDAEENEEAASEKSAE
TTASVDEKEADDRQLVMDKHFVLPKRSTRSSRIIKPNKRLLEVGGICSKRSPSDANGKPKPKNYFGLATL
PAKCTPRRRRSAATALSQKLGKETFASFATAKVNSSFVLRQPRLQFQTDKSRSFVSAKPTLPTTTVLPAS
SSAITSANVLSFGALNNANSAVAAASTCAVCSAPVNNKDAPLARKYGVIACEVCRKFNSRMTKISKLSTP
MHSNPSTSTAQSGQQLKCTDGGNCSILSLKSQLKNFKKLYKERCKACWLKKCLATLQLPAGHRSRLSAIL
PASMREEVAPKDDKCPELLSPTASLRFTAPTSSASSGTTIKWKSSAETAVNSIKSNPLAENNVTFGGTPL
LRPAILEKPLFLKIGSDNKKAKESKEALGLSPVPSTSEAAVAPGKTTRKAKQDKEKARELEAEKPLSPNA
KKTTEANTPETQKDEQPASTTTTVSAASSSTSHTSSAATNSSQLETTEAANASAVPDNLKRQRIDLKGPR
VKHVCRSASIVLGQPLATFGDEEEELAAAEAGPAPTTTTTTTSPEVIIKKPKSPQPMQMIIDENDNCASC
ILTPTEATAEAQPAVKSVLESRSSKSNTQTEAKKTPATSGSSKGKVTTRNATATVTSVASSLVATKKQRN
IEVSSSISSSQAAATQSRRALAKEVNRLKALISIDFWENYDPAEVCQTGFGLIVTETVAQRALCFLCGST
GLDPLIFCACCCEPYHQYCVLDEYNLKHSSFEDTLMTSLLETSNNACAISAATNTALNQLTQRLNWLCPR
CTVCYTCNMSSGSKVKCQKCQKNYHSTCLGTSKRLLGADRPLICVNCLKCKSCATTKVSKFVGNLPMCTA
CFKLRKKGNFCPICQKCYDDNDFDLKMMECGDCNQWVHSKCEGLSDEQYNLLSTLPESIEFICKKCARRC
DVSRNKADEWRQAVMEEFKSSLYSVLKLLSKSRQACALLKLSPRKNWRCCSAGAQPAKAHSQGKLQPKAL
QFTYNGLGSDGESQNSDDIYEFKEQHSTNRKPSTPVPCSCLQPLSQSPSFSLVDIKQKIASNAYVSLAEF
NYDMSQVIQQSNCDELDIAYKELLSEQFPWFQNETKACTDALEEDMFESCGYEELKESPTTYAEHHTASQ
APRTGLLDIPLDDVDDLGGCAVKTRLDTRVCLFCRKSGEGLSGEEARLLYCGHDCWVHINCAMWSAEVFE
EIDGSLQNVHSAVARGRMIKCTVCGNRGATVGCNVKSCGEHYHYPCARTIDCAFLTDKSMYCPAHARNAL
KANGSPSVTYESNFEVSRPVYVELERKRKKLIVPAKVQFHIGSVAVRQLGSIVPRFSDSFEAIVPINFLC
SRLYWSSKEPWKIVEYTVRTTIQNSYSSTLTLDAGRNFTVDHTNPNCSLVQLGLAQIARWHSSLARSDLL

DTDWAEFPNSYVPADENTEEEPQQNADLLPPEIKDAIFEDLPHELLDGISMLDIFMYEDLGDKTELFAMS
EQSKDGTTATSQAGGASVIICDEDTRNSNSLNKHLVLSNCCTASNPVDDAMLCAARSSSQEKECGDVLKK
TDTAPTRSWPKLDGGSVAAFKRRKLSKNIAEGVLLSLNQRSKKEMATVAGITRRQSVCGSSELPAEGSAT
MRTKSFTWSAAKCLFEKNESREEPAKLTIMQMDGVDDSITEYRIIGSDGNLSTAQFTGQVKCERCQCTYR
NYDSFQRHLGSCEPMSTSESESETATGTAQLSAESLNELQKQALAAATLSNTGGLNYLQTSFPQVQNLAT
LGQFGVQGLQGLQTLQLQPQSLGNGFFLSQPNAAQATSNGNDVLQLYANSLQNLAANLGGGFTLTQPTMS
TQAQPQLIALSTNPDGTQQFIQLPQSNGATTQLLQTAAPLRCNATYQTLQATNSDKKIVLLFLEAGDPLQ
EVVTQAAQQATAAAHQKQLKSGHGVKPIQAKLQGQQQQQRHQQHQQHQQHQQQQQQQQQQQQQQQQTPITV
AQHGGTTQLLGQNLLQPQLLFQSNAQPQTQQLLLPQTQAQNIISFVTGDGSQNQPLQYISIPTTNDFKPQ
QTTSTPTFLTAPGGGATFLQTDASGNLMLTTAPANSGLQMLTGQLQTQPQVIGTLIQPQTLQLTTGADGT
QTATAQQPLILGGATGGGTTGLEFATAPQVILATQPMYYGLETIVQNTVMSSQQFVSTAMPGVLSQNSSF
SATTTQVFQASKIEPIVDLPAGYVVLNNAVDASGNTSWLQQSQTQATDDATAQLLQNAGFQFQTTPTTST
QQTMSTDYAPPLVVTAKVPPVAQMKRNTNANKSPISVLSKVQPQPQQSQVVNKVLPTNVIQQQQQQQQQ
QQQQQQMQPKQQLAGNANLKLTSQFQRQQQANELKNKQAAGQQTGSTCGAPPSIASKPLQKKTNLIRPIH
KVEVKPKIMKQAPKLATSAASMQHHQQQQSPAAINQVAKVALLQQRLAPAPQPQQQEPQEEQQHLHQQQQ
QQQQQQQHMQQHQQQQQQLSMPQLLRAQQPIISIVNTAEPQAATQFVIRPALQAQAQPIQLQEQQSQQQQ
QQPAEQLINGKAARLQRYASNSLPTNVVNPLQQQRCASANNSSNSNVTQQNSTITINSRPTNRVLPMQQR
QEPTPLSNDVVVQSPTPPKPIEEPVPAGASTQKPIVKCYAQLEQKSPGYETELKTNITLDNLEQTNSITT
MQLQQPQQGPIYGEQIFEKQSEAQVQLEKPKHNDLMLLEATSCQQQQQQQQHMEMVVDNGFQLTSNESCL
LEKHGFNVEAVPMDTEDHYASMKNGSGGGAAEGIGQVDDAEEDEDDDDDFSLKMATSACNDHEMSDSEEP
AVKEKISKILDNLTNDDCSDSIATATTVEASAGYQQMVEDVLATTAAGSVSTDDETFTATAEAVEAAASY
INEMAEAHELQLKQLQAGVELDLKKPKLDVPQQQPDTVPPNVVPTAAAPQQPPPMRDPKKISGPHLLYEI
QSEDGFTYKSSSIAEIWEKVFEAVQVARRAHGLTPLPEGPLADMSGVQMIGLKTNALKYLIEQLPGVEKC
VKYTPKYHKRNGNVSTAAGGGHARTAGSNPAALAAGAESLIDYGSDQEELQENAYECARCEPYVSRSEYD
MFSWLASRHRKQPIQVFVQPSDNELVPRRGTGSNLPMAMKYRTLKETYKDYVGVFRSHIHGRGLYCTKDI
EAGEMVIEYAGELIRSTLTDKRERYYDSRGIGCYMFKIDDNLVVDATMRGNAARFINHSCEPNCYSKVVD
ILGHKHIIIFALRRIVQGEELTYDYKFPFEDEKIPCSCGSKRCRKYLN

>TRX_DROME
MGRSKFPGKPSKSINRKRISVLQLEDDAANPAEPQQPAPESQQPSGSGSGSSAAREKGNNCDNDEDDNAP
GGASISGNTASSSAGSGNSGNGSSSGSSTGSGSSGSGSTNGGSVNGGTHHKSAANLDKEAVTKDQNGDGD
KTRGNVSSAPSGKLSAAASGKALSKSSRTFSASTSVTSSGRSSGSSPDGNSGASSDGASSGISCGKSTAK
STEASSGKLAKTTGAGTCSSAKSSKASSLEQLVKQQPLVSGACLKALFVATPATSTGLACALVSPGGSSQ
GGTFPISAALLRARKNSNKKFKKLNLARGEVMLPSTSKLKQLNSPVVDNPSPSPPILSGSTPSVEGGIGV
GGVVSPGEDAALKRVLTEMPNEVARDPSPSSCTAAANGAASGKGSASNGPPAMASSGDGSSPKSGADTGP
STSSTTAKQKKTVTFRNVLETSDDKSVVKRFYNPDIRIPIVSIMKKDSLNRPLNYSRGGECIVRPSILSK
ILNKNSNIDKLNSLKFRSAGASSSSSNQESRSSSNVFGLSRAFGAPMDEDDEGGVTFRRNDSPEDQNNAE
DDEMDDDDDDEEAEEDDQNEDDNDEAASEKSAETEKSAGADERDPDEKQLVMDSHFVLPKRSTRSSRIIK
PNKRLLEEGAISTKKPLSLGDSKGKNVFGTSSSSAGSTASTFSASTNLKLGKETFFNFGTLKPNSSAAGI
FVLRQPRLQFQADNQQATFAAPKACPTSPSAIPKPANSLATSSFGSLASTNSSTVTPTPSACSICSAVVS
SKEVTQARKYGVVACDVCRKFFSKMTKKSISANSSTANTSSGSQQYLQCKGNEGSPCSIHSAKSQLKNFK
KFYKDRCTACWLKKCMISFQLPAAHRSRLSAILPPGMRGEAAAREEKSAELLSPTGSLRFTSTASSSSPS
VVASTSVKWKSSGDSTSALTSIKPNPLAENNVTFGSTPLLRPAILENPLFLKISNAADQKLAAAEAISPS
LTKKNSKQEKEKVKESEQSEKLLSPTQAGTKKSGAAEAQVEEVQPQKEEAPQTSTTTQPSASNGASHGVP

QAELAGETNATGDTLKRQRIDLPGPRVKHVCRSASIVLGQPLATFGEDQQPEDAADMQQEIAAPVPSAIM
EPSPEKPTHIVTDENDNCASCKTSPVGDESKPSKSSGSAQAEVKKATALGKEGTASAAGGSSAKVTTRNA
AVASNLIVAASKKQRNGDIATSSSVTQSSNQTQGRKTKEHRQQRTLISIDFWENYDPAEVCQTGFGLIVT
ETVAQRALCFLCGSTGLDPLIFCACCCEPYHQYCVQDEYNLKHGSFEDTTLMGSLLETTVNASTGPSSSL
NQLTQRLNWLCPRCTVCYTCNMSSGSKVKCQKCQKNYHSTCLGTSKRLLGADRPLICVNCLKCKSCSTTK
VSKFVGNLPMCTGCFKLRKKGNFCPICQRCYDDNDFDLKMMECGDCGQWVHSKCEGLSDEQYNLLSTLPE
SIEFICKKCARRNESSKIKAEEWRQAVMEEFKASLYSVLKLLSKSRQACALLKLSPRKNVRCTCGASSNQ
GKLQPKALQFSSGSDNGLGSDGESQNSDDVYEFKDQQQQQQQRNANMNKPRVKPLPCSCQQHISHSQSFS
LVDIKQKIAGNSYVSLEEFNYDMSQVIQQSNCDELDIAYKELLSEQFPWFQNETKACTDALEEDMFESCS
GGNYEDLQDAGGVSASVYNEHSTSQAESRSGVLDIPLEEVDDFGSCGIKMRLDTRMCLFCRKSGEGLSGE
EARLLYCGHDCWVHTNCAMWSAEVFEEIDGSLQNVHSAVARGRMIKCTVCGNRGATVGCNVRSCGEHYHY
PCARSIDCAFLTDKSMYCPAHAKNGNALKANGSPSVTYESNFEVSRPVYVELDRKRKKLIEPARVQFHIG
SLEVRQLGAIVPRFSDSYEAVVPINFLCSRLYWSSKEPWKIVEYTVRTTIQNSSSTLTALDVGRNYTVDH
TNPNSKEVQLGMAQIARWHTSLARSEFLENGGTDWSGEFPNPNSCVPPDQNTEEEPQQQADLLPPELKDA
IFEDLPHELLDGISMLDIFLYDDKTDLFAISEQSKDGTQAMTSNQAQNQNQQAGGANSVSICDEDTRNSN
TSLGNGWPASNPVEDAMLSAARNSSQVQMLKTLAWPKLDGNSAMATAIKRRKLSKNLAEGVFLTLSSQQR
NKKEMATVAGVSRRQSISETSVEGVATTSGSVRSKSFTWSAAKRYFEKSEGREEAAKMRIMQMDGVDDSI
TEFRIISGDGNLSTAQFSGQVKCDRCQCTYRNYDAFQRHLPSCSPTMSSNETESDVSGQGMTNNATQISA
ESLNELQKQLLANAGGLNYLQSATSFPQVQRLGSLGQFGLQGLQQLQLQPQSLGNGFFLSQPNPATQANT
DDLQIYANSLQGLAANLGGGFTLAQPTVTAPAQPQLIAVSTNPDGTQQFIQIPQTMQATTTPTATYQTLQ
ATNTDKKIMLPLTAAGKPLKTVATKAAQQAAVKQRQLKSGHQVKPIQAKLQPHPQQHQQQQQTQVQQPIT
VMGQNLLQPQLLFQSSTQTQAPQIILPQAQPQNIISFVTGDGSQGQPLQYISIPTAGEYKPQPQPTATPT
FLTTAPGAGATYLQTDASGNLVLTTTPSNSGLQMLTAQSLQAQPQVIGTLIQPQTIQLGGGADGNQPGSN
QQPLILGGTGGGSSGLEFATTSPQVILATQPMYYGLETIVQNTVMSSQQFVSTAMPGMLSQNASFSATTT
QVFQASKIEPIVDLPAGYVVLNNTGDASSAGTFLNAASVLQQQTQDDTTTQILQNANFQFQSVPTSSGAS
TSMDYTSPVMVTAKIPPVTQIKRTNAQAKAAGISGVGKVPPQPQVVNKVLPTSIVTQQSQVQVKNSNLKQ
SQVKGKAASGTGTTCGAPPSIASKPLQKKTNMIRPIHKLEVKPKVMKPTPKVQNQNHSLLQQQQQQQPQL
QQQIPAVVVNQVPKVTISQQRIPAQTQQQQLQQAQMIHIPQQQQPLQQQQVQVQPSMPIITLAEAPVVQS
QFVMEPQALEQQELANRVQHFSTSSSSSSSNCSLPTNVVNPMQQQAPSTTSSSTTRPTNRVLPMQQRQEP
APLSNECPVVSSPTPPKPVEQPIIHQMTSASVSKCYAQKSTLPSPVYEAELKVSSVLESIVPDVTMDAIL
EEQPVTQSIYTEGLYEKNSPGESKTEQLLLQQQQREQLNQQLVNNGYLLDKHTFQVEPMDTDVYREEDLE
EEEDEDDDFSLKMRTSACNDHEMSDSEEPAVKDKISKILDNLTNDDCADSIATATTMEVDASAGYQQMVE
DVLATTAAQSAPTEEFEGALETAAVEAAATYINEMADAHVLDLKQLQNGVELELRRRKEEQRTVSQEQEQ
SKAAIVPTAAAPEPPQPIQEPKKMTGPHLLYEIQSEDGFTYKSSSITEIWEKVFEAVQVARRAHGLTPLP
EGPLADMGGIQMIGLKTNALKYLIEQLPGVEKCSKYTPKYHKRNGNVSTAANGAHGGNLGGSSASAALSV
SGGDSHGLLDYGSDQDELEENAYDCARCEPYSNRSEYDMFSWLASRHRKQPIQVFVQPSDNELVPRRGTG
SNLPMAMKYRTLKETYKDYVGVFRSHIHGRGLYCTKDIEAGEMVIEYAGELIRSTLTDKRERYYDSRGIG
CYMFKIDDNLVVDATMRGNAARFINHCCEPNCYSKVVDILGHKHIIIFAVRRIVQGEELTYDYKFPFEDE
KIPCSCGSKRCRKYLN

>EGON_DROME
MNQLCKVCGEPAAGFHFGAFTCEGCKSFFGRTYNNIAAIAGCKHNGDCVINKKNRTACKACRLRKCLLVG
MSKSGSRYGRRSNWFKIHCLLQEQQTTSGLGGGSSVGSGSGGGVSSASLEQLARLQQASNQARQTYQDKT
NPCIKSATATTSPRIEGAAVGTGIGGGASPSFLQAAKLHHQRQLKLDSRLSNTPSDSGASSAGDPNEDGV

TSVLGGQIATPSSTNATSLPKLDLRHPNFPATSEPDADMQRQRHQELLEIFRSHSEPLYSSFAPFSHLPP
VLLAAGVPQLPIFKDQFKAELLFPTTSSPELEEPIDLSFRSRADHASPMAHNSNSPSLSEPAAASHCLGE
STNFVRKSTPLDLTLVRSQTLTG

>ODR7_CAEEL
MIVPDTEGLLIYSYGLMYGSYCMACQMLIPHFQCIPGIFPNFRISTELIKTMTDKLEQPNNNVPQQPWGP
FPPAFGGRPSGEQTDGNPGEFDNDAAHQQTAPFMTHFFPRIGLQFPDFTEYQRFNGFQRNAFFPNPFGSQ
FTGQAFAQSFPLHNSMTTMDGFNLTHAPHPFSTNTNSTKPKDIENTVQSTIKHSSENIQDKPPVLSVEYP
VKYDSELKFDANVDFTAVPKQESSDDSTLKNLKKSDQQLQQPQQFTFPPPLLAEKSFEQPRMREDVLPFH
PQFYPAPLDMGTNFKQEMRTPPIDGHIDYRKFDASGKRMEFQPPGALHDCQVCLSTHANGLHFGARTCAA
CAAFFRRTISDDKRYVCKRNQRCNNASRDGTGYRKICRSCRMKRCLEIGMLPENVQHKRNRRDSGSPPRK
TPFDTFFNGFYPSFQPSGSAAQPITVSSSESPRHTTN

>DAX1_MOUSE
MAGEDHPWQGSILYNLLMSAKQKHASQEEREVRLGAQCWGCACGAQPVLGGERLSGGQARSLLYRCCFCG
ENHPRQGGILYSMLTNARQPSVATQAPRARFGAPCWGCACGSAEPLVGREGLPAGQAPSLLYRCCFCGEE
HPRQGSILYSLLTSAQQTHVSREAPEAHRRGEWWQLSYCTQSVGGPEGLQSTQAMAFLYRSYVCGEEQPQ
QISVASGTPVSADQTPATPQEQPRAPWWDASPGVQRLITLKDPQVVCEAASAGLLKTLRFVKYLPCFQIL
PLDQQLVLVRSCWAPLLMLELAQDHLHFEMMEIPETNTTQEMLTTRRQETEGPEPAEPQATEQPQMVSAE
AGHLLPAAAVQAIKSFFFKCWSLNIDTKEYAYLKGTVLFNPDLPGLQCVKYIEGLQWRTQQILTEHIRMM
QREYQIRSAELNSALFLLRFINSDVVTELFFRPIIGAVSMDDMMLEMLCAKL

>Q8QGS4_RANRU
MACLDKCHCAVDNRRHGSILYNILKNEEHKDSHNSSKISKEDHSRLYGQGCSCGSQKKVTLKSPQVTCKA
ASAVLVKTLRFVKSVPCFQELPLEDQLLLVRSCWAPLLVLGLAQDKVNFETVETSEPSMLQRILTNSQGG
ENKLHHEHSEQDFLFGNSQHQQHNKLSQLPSATEIRWIKEFLEKCWSLAISTKEYAYLKGIVLFNPGLPG
LHCAQYIQGLQQEAHQALNEHVKMIQRWDNARFTKLIIVLSLLRSINANAISELFFRPIIGTVNMDDMLL
EMLGAKI

>Q8AY13_ORENI
MATLEGCRCRGASGRNNNSSSSSSILYSILKSDDRLVTAEEQQQHPQQHTLQHLFQKPPSSSASASLQEV
RQQACSCGSTRRRGVLRSPQVTCKAASAVLVKTLRFVKNVPCFRELPEDDQLVLIRSGWAPLLVLGLAQD
RVDFETTETVEPSMLQRILTGCPDRQSEAVGGQNRGAPGVSVVDIEAIKAFLKKCWSVDISTKEYAYLKG
AVLFNPDLEGLRCLHYIQSLRREAHQALNEHVRLIHREDTTRFAKLLIALSMLRAISPPVVAQLFFRPVI
GTVNIEEVLMEMFYGK

>P97947_RAT
MSSSQSGVCPCQGSASHPTILYTLLSPGPRTRPMAPASRSHCLCQQHRPVRLCAPHRTCREALDVLGKTV
AFLRNLPSFCLLPHEDQRRLLEGCWGPLFLLGLAQDTVTFEVAEAPVPSILKKILLEEPNSGAQGAQPPD
PPQPSLAAVQWLQHCLESFWSLELGPKEYAYLKGTILFNPDVPGLHASCHIAHLQQEAHWALCEVLEPWY
PASQGRLARILLMASTLKNISCTLLVDLFFRPVIGDVDITELLEDMLLLR

>Q8AUM4_ORENI
MDSACRCSTSSDRTSNPILYNILSQMDGSRLSQGNFSYNSVPHRCKCEARRTVCLKRPSEICKEASAVLV

KTLNFMKNLPAFNQLPPNDQFALLKSCWAPLFILGLAQERVDFEVTDIPTDSMLKKILLNRPESPEVERE
QPTMAGVSKLVSCLKKFWSLDLSPKEYAYLKGTTIFNPDVPDLKAALFVEGLQQEAQQALSKVVQLLHPG
DGDRFARILLTASMLQSITPSLITELFFRPVIGQANLLELLVDMLFCR

2. 500 non-NR proteins $\mathbb{S}^{nNR}$

>A0A178_CARAU
MQRVAMLLFCCLLLSATSSPVYPLRFGQRAAAILMTSSIEDPMQLPADTSSQTPDAEFRFGRHADAIFTN
SYRKVLGQISARKFLQTVMGKRLGPETQNYVKRHSGIYGDTFNQDVDMDVIEREQSYREPQRLKFSVVTQ

>A0A180_XENLA
MRRTLCLLLLHFALCVQCYIFHPKYSSYQTPGDFNIETIEPLQSQDWSSLDEKKEYVRGLSENRVERHVD
AIFTNTYRKFLGQISARRYLQNMMGKTLGQDTQKKADPEDGVLGEEVITLLSDSGIPDWRAEEHRETRL

>A0A181_DANRE
MQVGVIQRTALLVLCCLLLSASSSPVYPALKFGRHADAIFTNSYRKVLGQISARKFLQTVMGKRLGPETE
SNVKRQSSMYGNTYKQDVDMNVIESEQSYRDPQKFKFALIMH

>LCE6A_HUMAN
MSQQKQQSWKPPNVPKCSPPQRSNPCLAPYSTPCGAPHSEGCHSSSQRPEVQKPRRARQKLRCLSRGTTY
HCKEEECEGD

>EYS_DROME
MSNVHQFDTQTMAESPQIRRDMGRLCATWPSKDSEDGAGTALRAATPLTANGATTTGLSVTLAPKDMQRN
HLLKMPTATIEKPTITATIASSSSTSTSTTRKSVTATRSLKLNPNILLPTLRILARGLLLPALILAILVG
SSQAGFACLSNPCVFGVCIDGLNSSYSCYCIDGYTGIQCQTNWDECWSSPCQNGGTCVDGVAYYNCTCPE
GFSGSNCEENVDECMSNPCQNGGLCRDRTNGYICTCQPGYLGSHCELDVAVCETGTGARCQHGGECIEGP
GLEFTCDCPAGWHGRICQEEINECASSPCQNGGVCVDKLAAYACACPMGYTGINCEEEILICADNPCQNN
ALCLMEEGVPTCYCVPDYHGEKCEFQYDECQLGPRCMNGGVCIDGVDTFSCSCPPLLTGMLCECLMVGEE
SLDCNYTAPATQSPPRRTTTTSTMAPPTVRPVTPPETTVSPSRASEEVEIIVVTTSAPAEVVTSVLSPSS
SSSSSEEGVSVEIKTPTVAPPESGSHSISVEQTTAVPAQPEPESEQEPESKPHPESESASESETETEEEI
IPGTTARPPTSRSSSSSEESPSIFTTLPPLPGKPQTSASSESSGEVVTSEEYTTVPHFEVSGSKSESGSE
EVTTVRPTAAPSITISVDITSSGSSSSSSESVEVFTTPAPVFVQRVTTIETSISIDYVTPTPLPETTTPR
VVPVPRPTFAPEPPLDVVETTASTHHLWTEVPTTAAPFFTEYPAEVLITTHRTSAGRFTTVQPPAGVTTT
SPTEDSSVELPTPHTPQIVVTILDSNEVIPSLITTTGSPTTHHHHHHPHHEAEGTTLQPLEEDEHHHHH
HHDEFTTPQPVEITTGHPLQTEDLIGVQEPAVVTTESPFAPAETTVVPVVVPATIAPLGTAAPPATPAPV
PPATTTPPPSPPSLATETPTLPPTLPPVTLPPVTQPPPTIPPTPPSTQSAQTLPPPTSAINVYTTPDGPP
TASQTKPSVTESSEEVEGTNTVSTGGRGSGGVPEEKAGDVDCIKLGCYNGGTCVTTSEGSRCVCRFDRQG
PLCELPIIIRNAAFSGDSYVSHRIYKDIGGHESLDAVLPMHIQLKVRTRATNGLIMLAAAQGTKGGHYMA
LFLQKGLMQFQFSCGLQTMLLSELETPVNTGHEITIRAELDFSRNYTHCNASLLVNDTLAMSGDQPTWLK
LLPPRLHTPEAILNTWLHLGGAPQAPIGLIIELPPAQSGSGFTGCLHTLRINGQAREIFGDALDGFGITE
CGSLACLSSPCRNGAACIKIETNDLDENGEKAEKWKCKCPTGYMGPTCEISVCEDNPCQYGGTCVQFPGS
GYLCLCPLGKHGHYCEHNLEVALPSFSGSVNGLSSFVAYTVPIPLEYSLELSFKILPQTMSQISLLAFFG
QSGYHDEKSDHLAVSFIQGYIMLTWNLGAGPRRIFTQKPIDFRLDAPRVPYEIKVGRIGRQAWLSVDGKF

NITGRSPGSGSRMDVLPILYLGGHEIANFNTLPHDLPLHSGFQGCIYDVQLKAGQVTVPLQETRGVRGRG
VGQCGTRECHRHACQHDGACLQHGATFTCICQEGWYGPLCAQPTNPCDSFNNKCYEDATCVPLVNGYECD
CPVGRTGKNCEEVIRSLSDVSLTGRRSYLAVRWPYLYDGGDKLGAKRSQMVSYRNFTKKLMPPKPITTPS
SHFVMKLLNEVEKQRSFSPVPLMGSKSFEEHHRVQFFFIEFQLRPLSERGLLLYFGTLNNNQDKKIGFVS
LSLQGGVVEFRISGPSNHVTVVRSVRMLAIGEWHKIKMAQRGRWLTLWVEGSASSALAPSAEVLVEPDSL
LYIGGLKDVSKLPHNAISGFPIPFRGCVRGLVVSGTRIVLNETNIVESRNIRDCDGTACGGDSCESGGHC
WLDEKLQPHCICPEYAKGDRCEYSETCKLIPCKNNGRCLRSGRCSCPNGWGGFYCEIAMSKPTTPSFRGN
SYLILPPPRIPMKDKRRGPSLYVRPREAIQVSLNFSTIEPDGLLLWSEHERSKFLGLGLEAGHLKLASNL
LGSTNDTVRAPASGFIADGAWHWTSVLLDRSRLELQLDGEVIFTERLPEGGRSLGSTTPRSTLAGRRKNS
SKEPTISYEDVFYLGGFPNSDSVSRRTKGRFFDPFKGCLQDIQFGAEPTAIISDFSTYQGENIGSCDLHG
DEPLTV

>A0A1G5_9BIVA
MRVRTVTRFTSLVLLLWMSCVKSEYLFTMPRSLRGGSQGKYCLTLRNLDHENPAGYNCQVSLHFLTSESI
ITSEHIYAFNSTESDWSQCVNFDAPFKEDRYTATVTVHIDGKKLFMHDEKVDVWTAKNITLIQTDKPQYK
PGQTVKFRILRMDYRLLPLTDLFELITIDNPGGVRVMQWKNVDVSKGLVSLEMKLSDDPVLGTWNIKALG
ADDTVQSFKVEEYVLPKFEVKIMPPKYLLPTTTSISGKVCADYTYGQPVKGSLTMKVCFGPEYYPSYYPE
QPCVDVIETNFDGCHSFSISPSELKLGVQGYPTWGSLKISATVRETATGIELNGTSTGPPLTNDPLKIEI
SDESDGYFKPGFPYKGKVTVTLPDGNPASNEVIRIKAEKYNLQYYWSREFTTDSSGIINFSLSNLGRDVY
SLSLMATAVKYEKQAEIGYRPYSLRVFTPNGYRTITQWFSPSLSSIYIPTIKDSVPCDSTVDLDVIYSTD
GSSTNVFNFVVKSGDDVIHMVRKKVVFDGVDIPMNVLQFNPAEELEYHEKEVTKAPRPPRPPFVTKGSPL
PKVPEPEPVPVVEEEIIPKVSTPLGGGSNQGAEVQEELVVPEVVKSLGVEKAIEGGKNPKRRSSDEPGMS
YGIGYFKMPVPIKTSGSEVTVLVYYIRQDKEVVATSLKIPVESCFKNKVKMEFASAKVRPGEETMFRLQA
SPNSLCSVGMVDKSVNLMGGDHQLTPARVLDEVKKVQTGGRYHSYWFDDEKYCMKKNKNETVSKGMYHHH
HHFTSGRPETKDSIEAFRSSKMLVVTDVLLETRPCSEPSPYYPIAYSSVVAETMGLPGLAGERSLNFRAK
SHPKGNEMERESAEDTQVQTVVRSYFPETWLWDLHTIGDDGVVNVTTEIPHTVTEWVGNTLCSNSKDGVG
ISPMIGITVFQPFFLSFTLPYSAIREENLPVLVTVFNYMTECLTMEVRMKETKDFRIQSVSGAILKMCVC
GGDSKSAKFHIVPLTVGEIDLEATAVSIEDDATCVNQIISKEGVGVQDGVRRKLLVEPEGIPQEYTTSFY
LCPEGRLLSQDIDLPVASADKLVPDSQRAKVNVIGDIMGPTLSNLKDLLKMPYGCGEQNMASWSPNIYVL
QYLTNTNQLTDAIQDEAKGYMRVGYQRQLKYRHHDGSYSAWGDNEYQNSTGSTWLTAFVVKSMAQSRPFI
DIDSKDLHLSMQWLLKHQNGDGCIQSVGKVFSSYLKGGLADGENVGGLTAFALIALLEAGIDKNDPAIVN
GFTCLSKQQTNADTYTLTVMAYAYTLYNVDSPKRGQIMAELEARTRVPNPGQKHWIREEEEKKEKDSNYF
YWRAPSAEVEMTAYVLMAYIAGGQEGAVSTAQPVVQWLTKQRNAQGGFSSTQDTVVALQALSMYATLVYQ
GGLDISVRVDTPSKSYQTGINDSNSLVLTTWDLSPQTTKLNVQVQGKGCTMVQANMKYNIYKDEEKETGQ
ASFEVKVSVYRSRTNIDNCKRRTLRICARYALPNFSNMAIVEVKMITGWIPVKSTVKELLAAKQIQKYEI
NPDNVDFYFDEFDSQERCFAFEVEQTDIVVTDPKPALIKVYDYYETKDSVMILYDIKTTCGTKEELPFPK
P

>A0A4R1_HUMAN
MRCVASNLGLCSFSRWPAGF

>A0A4W4_DANRE
MDIMIAVQDACVSGQWLTAIILSLCCFLPSCLPAGQTVDYTTSSESVVSRQGDTALLRCYLLDGISKGAW
LNRSSIIYAGNDKWSGDPRVSIVSNVGDKHEYSLQIQKVDVTDEGVYTCSIQSERNLHPKLIQLIVKVPP

KIYDISSDITVNEGSNVSLICAASGKPEPKISWRHISPSARKYESGEYLNITGISRDQAGDYECGAENDI
ASPDTKTVRVTVNFPPAIHEMKSHGVRPGQVALLRCEAAAVPSPVFEWYKGEKRINMGQGIVINNLSSRS
VLTVKNMTQDRYGNYTCVAVNRLGTANASVPLNPSNPAMYGSTGGAEVLLACWYLILALSSLVTVY

>B5DVZ7_DROPS
MEAALVDIIGGIMCTDTRRIHECTTALGQAFRNPQTLTGLCQILVSPREAPVRQLAVLLMNKRLQKLHHW
QMAAPEQQEEIKSCMLQALIGEEQKGVRNAIGKLIGTLVRHEADKEDSWLADLLAISFRFCNMPDRKKSE
LGASIFCTLAEAAPDHFLSRMPAAFELFSCVLVAAQAKGDMATTTVANMMMGMCFLVPLVDSHTEETLEN
IVPLMLFALQAFAQKGVVSEFSTGFDMLDSMVENTPKLLNKNIQNVVQFCLEILRNKQFYAPIRIEVVDF
VGRVVSVKKRTIVKQKLLGPILVAIFEMICSVFDSDEEEDDYFTGTFNSPGSAATQALDNMAFDLSSEKL
LRALLPIIEPSLQSPDPLRRRGAFMCIAVISEGCSEYIKRNNLEILLSLIIQRGVIDPDPRVHNVAFFAL
GQFTEHMQPEISTFAPQIMPVVIDFIHQVVVEAKMTHSVEPNKLNRIFNALEDLCDHLEDEILPHLPVVM
ECLFECMDQENHVHIRKLALISISTVASVSKTNFSRYLNPIVHILSHYLVYECSAPLNELRIVAIDTLAS
IASSVGKENFTHLTDCTLQFSLTMLDQGPDDPDLRRAIYSLLSGLSFVLTDNMDTAFPRFVARMLQSVAS
TECDNESNNHDVGDGSDDLQVENDFVLEKEEATLALRDFAVNSSKSFTPYLTKAFEVVHKNIDHNQEVIR
KASIDALYAFVIALGYTADIDGVKLACTILVPHFTHLIKKDEEPDIVCTILENLGALFKAVKKAALPLAQ
LAEAVVEGITDVLLSKTACQYSEHVDDGEGDTDTEESEDDEMVIESAANLVVTISYALDPETYSMYFGRL
YKLLLTQLEKAKKNDDLNQRTLVYRVLSECIRPLGIRVVTYFDDLLPVFLEGSTDCQPKARHCCFFGLGE
LVYNAEEHSFGSFSVILQALSDAIARETDAFAVDNICGALARLIITNCNIMPLGFVLPVFMHNLPLRQDP
EDYDIVLMAFRVLYMNARPSVVDFIGQMVAVTLNALVNGKLSDSESTASAVSFVKELKDDYPHHFNNVAS
TCNSAEAFEIVQNLLN

>B5DW00_DROPS
MHSDCCSRKCMTYLNVCARRIDEGFSLEIPHLSNSIYDIDVKQSAKEFPDIVDIVTLATESKQTPQTEQG
RQCQPVGGQAVQVHIPVECLPLPPGKGASVDAKLPSISSVPGQQMNPLNAEDITVFTNMFLSLFRGIGDW
RNQVAAETVQSVWQSPPIVGLSHRLFAA

>B5DW05_DROPS
MSINTETFGKLIGVIGDEDTCVGFLLGGIGEVSEDRERNFFVVEKDTTAAQINASFKKFLERPDIGIILI
NQVYADMIRPTVDAHVVPVPTVVEIPSKQHPYDASKDSVMKRAYGIINPPKRRH

>B5DW07_DROPS
MDLGPLVKIQDSEDENESDNASCASYTTKANRRQRDGNRLQQEQQHQQQPSSHISHQP

>B5DW09_DROPS
MHSGVHGSGRGQASRRKNKDENGNPNKDANANADEDEDADAVEDDVPRVTAGQ

>B5DW10_DROPS
MSWKPQAKPVTSKGDRPVTKVNKENKTAAKQHPVTRTPPSRPNTHDRIWKIMKM

>B5DW13_DROPS
MADIDEKHSKIRSSSTSSIRYNIAKQIYPKQLLTIKAGPIKDELGFTLCEKVALRQAWNLIRPRERRFGQ
DVFYTFLNEWYWSISKFKKGEDINIALLHAHALTFIRFVGALINESDPIMFQVMINENNQTHSRCRVGAD
YIAMLGQALTDYILKVLDKVRSPSLEQGLQRIVEKFKSYQDIQMDRSKTSYRRGVSKSNFSLHRITTEK

>B5DW14_DROPS
MNDSQDVPNQANDLVAEEPIYAEDLNPNDEEQRPTIVLPVYPKPLPERDVSHKLDDNGFSILEKSALRNA
WRFIEPFQRRYGQNTFYDFLTEHELLINTFRQQGKINLTKLHGHATAMMRLLSKLVQTLDMNLQFRSSLD
ENLPFHLKTGIDLDDMKMLANALKDYLLSSPVIEKHNSCTLTTALEKLVTIVGGYAEAEEARKKAMSGFY
RNTNTSVEPKSERASSIKSAKKSVIMD

>B5DW17_DROPS
MSTTLQLIMLTLVGVTIGQANNMSYRQVLVPVLSQLKGGVELHLRSSKDGDHDFVQFLLRQGKYSMSISR
TLELDSALPHHVLLFAGLEQLQEAFPQLTPRGGFYILALESSAPAEDPQLLQFMAEVWRHHNHSQIYYIQ
LDRGRLLLHNPFNQSIVLVDGTQAYTQIYQNLNGYPLRIYIFDSVYSAVVGDGASKKILSITGADAKLAK
TVAKQLNFTPDYIWPDDEFFGGRRSDGSYSGGVGRAHRREVDIIFTGFFVKDYLSTDIQFSAAVYMDDLC
LYVQKAQRIPQSIMPLFAVHVDVWLCFLMVGFFGTLCWLCLRALNRGLGIPRIFDAQKSRGRKVSSWSCA
LRIFIDTWVVWVRVNIGRFPPFHSERIFVASLCLVSVIFGALLESSLATVYIRPLYYRDLNTLAELDESG
KPIYIKHPAFKDDLFYGHDSAVYRRLDAKMTLVAEGEERLIEMISRQGKFAGVTRSASLQLNDIRYVMTK
KVHKIPECPKTYHIAYILPRPSPYLEELNHVVLHLVSGGLIQLWTGEMKERAKWSIQRFPDYLAQLDVGR
WKVLTLSDVQLAFYSLAIGCLVAGSVCLVELFFGRRKRK

>B5DW18_DROPS
MEQMERKRKLIRERFAARKRHRPAARPRSSDLENDSIEGLEEDPNDSSEEGEEDAESEDNNNNNGTDGIN
EATAVAVDDGTDDSSSTSMTSMEAQLSQAPSTSSSYPFLVETEANNNNQMASNNNHKPPAAANGSTVDMC
NTESSSASASSSTSN

>B5DW19_DROPS
MSLMLCRFERPSRQQPSKKKKPVALDVSAGSSSPSSTVKNQSKADSKTKPKKIRKSMGTLSQPMAKPKVN
NKAVKQLVAGKSKDSTKDLGEDIDANMLEPQASLKPQMVSKG

>B5DW21_DROPS
MEKSGLDWSGVEWRGVLGLLSQERQQAANFNAQQQQQLTRWQHVSTSKCHEEAQPPPPHCCLPRWAWAGA

>B5DW23_DROPS
MNEKIKSPSTQGGAGAGTPAAAPPSASGSAAAAAAAAAASSGSGGASVGANSASTPPTSGPPTPNNNGSDP
IIQQNVGVPHPYGAPPPPGSAPGAPPDPAAVMHYHHLHQQQQQQQQQQQQHPPPPPHMQHHGGPAPPPG
APEHAPGVKDEYGAAVAHLPPPHAHPAYARYHSGDPNMDPYRYGQPPPGGKLPQPHQQQQQPQQQQQQPP
GAGGSPNRPPQQQQQRYIPGQPPQGPTPTLNSLLQSSNPPPPPQHRYANSYDPQQAASAAAAAAAAAQQQ
QQAGGPPPPPPGHGPPPPQHQPSPYGAQQGGWAPPPRPYSPQLGPSQQYRTPPPTNTSRGQSPYPPAHGQ
NSGSYPSSPQQQQAQAQQQQAGQQPGSAGPGGPPPGAAQQQPQQNTPPTSQYSPYPQRYPTPPGLPAAGP
NHRTAYSTHQYPEPNRPWPGGSSPSPGPGHPLPPASPHHVPQPLQQQQQPPPPHAAAGGPPPSSPGHAPS
PSPQPSQASPSPHQELIGQNSNDSSSGGAHSGMGSGPPGTPNPQQVMRPTPSPTGSSGSRSMSPAVAQNH
PISRPASNQSSSGGPMQQPPVGAGPPPMPPHPGLPGGPPPQQQSQQQQQASNSASSASNSPQQTPPPGPP
PSQGMNNMGTPPPPPQGASGGGYPMPPHMHGGYKMGGPGQSPGAQGYPPQQPQQYPPGNYPPRPQYPPGA
YATGPPPPPTSQGAGGANSMPTGAQGGGYPGRSMPNHSGGSPHGQYPPYQWVPPSPQQQAGAPPGGTMVG
NHVQGKGTPPPPVVGPPPPQGSGSPRPLNYLKQHLQHKGGYGGSPTPQQGVPQGYGNGPTGMHPGMPMGP
PHHMGPPHGPTNMGPPTSTPPQSMLGGQGQPPGGPDSGGPEHISQDNGISSSGPTGASGLHPVTAVVTTG

PDGTPMDEVSQQSTLSNASAASGEDPQCTTPKSRKNDPYSQSHLAPPSTSPHPVVMHPGGGGPGEEFDMS
SPPNWPRPAGSPQVFNSHVPVQQEPFRSTTKKSDSLCKLYEMDDNPDRRGWLDKLRSFMEERRTPITACP
TISKQPLDLYRLYIYVKERGGFVEVTKSKTWKDIAGLLGIGASSSAAYTLRKHYTKNLLTFECHFDRGDI
DPLPIIQQVEAGSKKKTVKAASVPSPGSSNSQDSFPAPPGAAPNAAIDGYAGYPVGGSPYPVGASGPQPD
YAAAGQMQRPPSQNNPQTPHPGAAVAVAAVGDNISVSNPFEDPIAGAGPGSGPGPGPGPGPGAGGAAVAA
AAGGALPPPPPHSPHATQQSAAQQQQQQHPQHPSLAGPPTQQQQQSQSQPPQPPGAPPAGAPQQQQHGPG
PGPVPPSPQHLPQQQQHVRPAAGAPYPQGGSAYPTPVSRTPGSPYPSQPGAYGQYGASDQYNATGPPGQP
FGQGQGQYPPQNRNMYPPYGPEGEAPPAGANQYGPYGSRPYSQPPPGGPQPPAQAVAGGPPTSGSPVAPP
TGGYAPGTPTQQDYYRPPPDQSPQPRRHPDFIKDPQPYPGYNARPQIYGAWQGGAQQYRPQYATSPAPQS
WGSAPPRGAAPPPGAPHGPPIQQPAGVAQWEQHRYPPQQAPPPPPQQQQQQQQQPPYQQVAPPGQQQPQA
PPQWAQMNASQPTQPGIAPPGSPLRPPSGPASQQQRMPGMPGMPPQQQAPGSQQPPQQPPHGGGIPSPGM
PQVPGGMVKPPYAMPPPPSQTVGQPVGQPAPGVGMIPQKQPPIVGPGMPQPLQQPPPHQQHPHPHSHQQH
PQHPQHPQHPQHQHQLPPNQQQQPGGYAPQVPVGGPGAQLVKKELIFPHDSVESTTPVLYRRKRLTKSDV
CPVDPWRIFMAMRSGLLTECTWALDVLNVLLFDDSTVQFFGISNLPGLLTLLLEHFQKNLAEMFDERCEE
PLDEAEDDADSGTVMCSGGLERRVRGGRQTRCVRSICSYNRKRHYENMDRKNGGSDSEDADEGIDLGQVR
VQPNPEERSLLLSFTPNYTMVTRKGVPVRIQAAEQDIFVDERQKAWDIDTNRLYEQLEPVGSNAWTYGFT
EPDPLDGIIDVFKSEIVNIPFARYVRSEKAKTRQDTDTATSPKPDIKQEENTTNSTEDGLQESFNKKRRL
VSGDSSSGEAGEGSAGVKKSKLLADEIAPPNAEVKKEPGTSGTTDASDSDCRAVDMEIESPSLQQQQQQR
LTNGIASASPPLGAFDPRGTVRDAAQVLQRKRDSSYEDECYTRDEASLYLVNESQDSLARRCIALSNIFR
NLTFVPGNETVLAKSTRFLAVLGRLLLLNHEHLRRTPKTRNYDREEDTDFSDSCSSLQGEREWWWDYLIT
IRENMLVAMANIAGHLELSRYDELIARPLIDGLLHWAVCPSAHGQDPFPSCGPNSALSPQRLALEALCKL
CVTDANVDLVIATPPFSRLEKLCAVLTRHLCRNEDQVLREFSVNLLHYLAAADSAMARTVALQSPCISYL
VAFIEQAEQTALGVANQHGLNFLRENPDSMGTSLDMLRRAAGTLLHLAKHPDNQSLFMQQEQRLLGLVMS
HILDQQVALVISRVLYRVSRGASRMHSVEFRLLQQRQQQQQLQQLRPSENQALAASAEAAAAAATAAAAA
ASGSGVKLESGSGEPNAEPLSGADVKPSPAATSTTLSTSGSGMISSAINDENSNSSQQLPPAATFNDVSN
SSTNSNSCGTASSNQTNNSTTNSSSNSSNSLGSQSTSTMNNAPTTPAAHITPPSVTEQQQQQQQQQQQQQ
QVSKAAATAALVSSNLSGSLSNATSAASAAPPPSSSSSASAASATAVSQPAAAPPPTNAGTTTAVA

>B5DW25_DROPS
MISNYDITDSKSSSKVLRPPGGGSSDIFGSDMPQTPRNVKNRMVSNIFSVEKDNSVKNTVRQGAHRFYFI
VYIRNMSRVEDNEKSKQTKTDTAGCPLTPVASAPAPPPADVLGIDLPCLDLEVGDVPKDNEIYTETGKHD
VNIQTRRDSGSNVEQPHSLEKMRSTANLKEPLALCPDYVKEVHGPCNARNPITGLGLNGDGVGGLKPVKQ
KIREGNPVTGEGYRAGGTDYIKAAGSTNSGSVGNGDNGGNSVVNKNRVPPGGYSSGLW

>B5DW26_DROPS
MEGMDLWTSMFSADFEEGEEEIEQYANENGLDSNPAGDSDDILPEGDGDDEDEENEEEDDDDDNDVEDD
AEEETCRDSGKTKEPDQDDDAIFQFDLEPIVSIGEPQAQPPLLAPPGPLGAPPVRPMHRHSLPRASQGRP
KEGRLSGVLTRPTTSQINATDANGIPINYELGVIYICPACGAEFRQQDLWKRHMNQMHQFNTRRGLNFMA
MDKLYHRCLECNKRIAMHSRENLLKHKFTHLPFRCTKCYICKREYKYRQDLMVHLRMVHCDEVVAMMREG
FNLAGRKTRVREPRTEQMQRKDPFPEEDDERNDLLEPETETVPPMLEEEAPPRRRKKRYQGGPSGDGGAD
LCEDYIHYMCPDCGTECDTHAQWSQHIEFVHDYVSRRGLNFISVDMQMQCLECKKIVIPNTIKTLRAHKF
THLSQPEWLRCKLCYKGYTEHHELVSHLLKQHHLESLMPEDSPDPSPVKKTDGGGGDDWEDNGNGSVFDD
VSPHFEDVPRRGGRIGSDDMYEPHIDYLCPQCGKEFIEKKHWRTHVVQVHGMNDVTKLNFEIINDRQLKC
TECAKIITNAYGIQNAQQHRITHLPFKAYARCRKCHKSYTDRKGLVKHLATYHRVGYVPRKTLKRGGRSA

APFASPKQQRRQIVTLGKETYEIIFLDEELMHSSVDEENDFGEQMQMADEEDLPTVSAGVPPPSPNRYKC
VECGTLFPTQVALKLHISEAHTFRDPPQYGGSKKLDKSQGKEESPKKRRLGAGPAAALTPSSSPAATSTP
SSGTVEQNYIFLCPSCGEEYKTQFEWRRHINEAHNFDKRQYLNMRQLDKYRYQCTQCNDIVCNSKLKGLQ
DHHFRHLPFRLYLKCLVCGTCYNHKPNIAAHLRTRHNIYERETPWRELQTKPQYSYVQSKEKDRDTAVKS
RPQSPSHAVVPSSSGSSSTSSRPLWPQSEKRPTRPAGLNTLEDSISYHNAVDMDFITYYCPKCNKNFDSH
AFWRKHIVEHHNFNSREGLNFRQIDIHHYLCLECYKRVTVTHTKGAIGQLQSHKFRHLPYRSFKCLTCNG
EFVRKQMFFKHLNRDTNRCDNKPSAAEVFDDEEQDAIGVTAAEDEDVEASAYRLMCPQCGDDFTTCNQAV
WHNHLNVSHGLDKQLLHIRKVGEGLYRCHDCNEELATKSRRVLQQHRFKHMPHAGYIRCQLCESGTDEKA
AEVHSMRELHQHIREHHPAVDESDERMLKVRQLMDEEETDEDRQPTANSDNESGSTPNNGPYMPLPPHLL
DEVEDLDFEDQYLLG

>B5DW27_DROPS
MQSMKANELPENPRETSNPLSSLMFCFAMPVLFKGRKKTLEQKDLYQALKEHKSDSLGDRLCAAWDEEVV
RNKNPRLGRVMTRVFGWHLVLTGVLLFLQEFLTKVTQPICLFGIMAYFAGDDTDLFKAQLYATGLIAASV
FTVFFGHPYMLGLLHLGMKMRIALSSLIYRKSLRLSRTALGDTTVGQVVNLLSNDVGRFDMVLINVHYLW
VAPLELIVVTYLMYIEIGVSSLFGVAVMLLFLPFQSYLGKRTSVLRLMTALRTDERVRMMNEIISGIQVI
KMYAWEKPFGKLVEFTRFKEMQCIKQVNYIRGILISFAMFLSRIFISTSLIAFVLLGNVLTAEKAFFVTA
YYNILRRSVTMFFPQGISQFAELLVSIRRLETFMHHPETQVRDKSKIQKEEPVIGDSPKANGLPEKLLDF
SGFTARWDSQSAEPTLENINLQLGRRKLVAVIGPVGAGKSSLIQAVLGELPAENGSLRVDGSYSYASQEP
WLFTGTVRQNILFGLEWDKHRYRTVVKKCALERDFQLLPYGDKTIVGERGASLSGGQKARISLARAVYRR
ADIYLLDDPLSAVDTHVGRHLFDQCMHGYLRSELVILVTHQLQFLEQADLIVIMEKGRISAMGTYSSMKR
SGLDFARLLTNPNNEDDTMDELEVAVGDQMDRLSVPSLSRRGSGKISRPTSRNNSFTSLSSMAESMAQEA
ALQMEEPRVEGKIGVGLYKEYLTAGSSWFMISFMLFLCLATQIVCSAADIFLAYWVNKNSNKAEMSSDPA
DMYYFAALNVAVVVFTLVRTMLFYKMAMRSSTTLHNAMYRGITRAAMYFFNTNPSGRILNRFSKDLGQLD
ELLPTVMLDVVQLFLILAGIVVVICITNPYYLILTLTLAIIFYYIREFYLKTSRDVKRLEAVARSPIYSH
LGATISGLPTIRALGAQKALIEEFDNLQDLHSSGYYAFLATNRAFGYYLDLFCTLYIVIIILNYFINPPE
SSGEVGLAITQAMGMTGMVQWAMRQSAELENTMTAVERVLEYDEIEPEGEFESDPKKKPCDTWPEEGEII
AEDLSLRYFPDPQSKYVLRALNFRIRPSEKVGIVGRTGAGKSSLINALFRLSYNEGTIHIDHRDTADIGL
HDLRSKLSIIPQEPVLFSGSMRYNLDPFEEYSDAKLWDALEEVELKPLISELPSGLQSKISEGGHNFSVG
QRQLVCLARAILRENRILVMDEATANVDPQTDALIQATIRNKFRDCTVLTIAHRLNTIMDSDRVLVMDAG
QVVEFGSPYELLTGSASKIFHGMVMEGGQSHFDGLLKVAERAHQESQKPKSE

>B5DW28_DROPS
MWKVQALVVQILLLSCLLKIYFEGSRLLPNLKPQKVLPEWGIGPPANRLVVFLTDGLRAETFFADNCSSV
PDLREVFVKQGLVGISRGSVPTLVRSGQIAIFGGFYETPPLLRTGFQWNPSTFDTVFNRSKTSLCWIESQ
TNVYTKSLTRGMFQHHSSDPKPKDPWVLLQLRSYLGINETVDKLRSKTSLILFVYLGNIATEGPLSKRYL
ERLHSAQRGIRETYDLIESTFNDSRTAYLMTSSHGISHLGSNVAGSRDETDTPFFLWGSGVRQTAHNVAP
TFAANNAGLQRPLIDLQQIQLAPIISALIGEPPPVNNLGRFPLGFLDVSMEQETQAAHLNALQLLAQAQL
LMRHHERGFFDKFLPQFKHLNQSQAESYILEMDREVRMGRTQEALQTIEKVAALAQECLRYYHSYYRIPL
LIATMAGLLGWIYCLLVQASQQARNSSEPPESVELISWTNVMMGALGVVVSVIILLLKVPFLIGFCLLLP
IYIWCMALAGRPLQGKPIRYGLLHLKLILVSIVCFIVPIFRRGLLCLLYVGLVCFYNRRHFKKMSPSFLA
WLGLVCCLSVLLFCLHCPSTLSLTINYGMYFQGASMLLAILRPLILGEKYGASVWIINAGTLLIGGYGMY
LKETGATVPLLLHTVCWAYLFFAFGSVPYGKRLPPKRRLELICFNLMTLHTLLSDSFASLFAQALIFEYQ
MGIDVYQETKQPRNALEQRADENEVQNEPKIVCPEENLARASYFSVSILLYFLLALLGTGHWISSFTFAP

STARLFAKDCCYGLTAFFVLLKIFIPSIIILSSMYARSEYARRNIRLIFISLILTCNGSLCLFLFVDHGQ
FWPAAHPSVVHVVLINTAVVFLVVCSCVTNFFFRGSLQARPTSSSTKINEEVPSSQSDVQTNV

>B5DW29_DROPS
MFCDWLNELQRFLHWGPVIVFSLMTCVTWTTVHVNAMWWPPAESFNAAINFVLIVFFNLITLYHFSMSIL
VGPGLLPKHWRPENPEDEQFLQFCKVCDGYKAPRAHHCRRCKRCVLRMDHHCPWINNCIGWANQANFFYF
LFFFMLGSIQSALIIGFSCYEGIYRRALLKQGDTNGIRVVLTLRSAMACMYSLGIVVGVLLATLKLMQLQ
IKAIIWNLSEIEGWIVKKAEYRRYASSSIGIAIPTFDYPYDMGVWRNILETFTPSNDGINWPVRPGCDQY
TMTREQLAQKVQKRARTRTYRCIASATGYWLPILSQGLMVTICMPCADDPRIVVHKGDLVQVTRVQEYWL
YGERVLSKREQKLQKKLQLKGHIRGWFPRCCAVEQFDEDQSAGDTVDVTHNTDQEKTKTGSKIKKRKPKP
NTFSLN

>B5DW32_DROPS
MALAAKAIPATKPDTKLAEATATATTAAAATTIWRPKATSTPTTMTLRPVDVQQNGQKP

>B5DW33_DROPS
MTDLFVLVQLLAESSTDLVNGCIQQDCPSDSGSTPVYPRIADHTLGFSFET

>B5DW38_DROPS
MMMMDTMDTTSQSQPMDVAPAMAVAATTVGTTVDFTATMVSMAATAESNNNHIDMAEYKEHRKNKKKKRD
KREKEGKEHRHHKHRDREHREHREHRRHRDREQREREREVVSSHHLHNSSQHSTSASSSPSSASTTPSAT
IEYVGGSASASPSYLGAATAGGVSGASGGATTATATTTYPHNLKIRFLLSGQRTELSPPTHQTSATAEVN
ATLAPAISATIAATASATTAAVTSVAGGTTTGSSTLSSNATSSSTAGGAGGSAAAPAVGGAAGVGASGYP
KTDSSRANTSGSSSKHSSSGSGSGGIKESSSSNPTPPASGSGSSNSNAPSLYVSVPLSTANVPGINLPSS
NSSSAATNPSGDPHVTAASRSTQQQQANQSALSTAGTAGGAYATATSGGAASTVIQHQSGKSSPALGGSG
IGTLVSGNSGGSIISASTGSSSSIGSGALSTGNLTATTTESGNLKISYEKQTTRVQQLQEQEALPATSRR
SRSRSGESSSTSSHHHHHHHHSQNHHHTNVVPPNHPTQGQQQHLTSLQSLHTAASVGSTSLTTPIAPVSS
SASNASNIKATSGGRSGKKRGAALNKNETNAPGAASATGNVAAAAATTAIAEKHSRHSSPHQSPSAAGLP
GGIASPVVLTLSSSQSSIDSPPAVAPTTTPTPPNSRSTRNSSNSLLLHSNHNGDQTSSSSSSSTSSSSMS
SSGGASSVPVVNLLDSPVNMSTSVPSVGYRVGNTGTSTPTGKGTTTSDGNYGGSSSNGGGTTKPLNKKML
RAQQTQLFQQQQQQQQPPQTYAPIIPARTTSPTNLTSASPSSSASPQQQQQSQQQNQHEDLEILQFTNS
IPTNPSSTSTTTTTITKSNRTPDLATTSAATTSSLPSTTTLTSQQSSQSMMPLVGGLKFSYESQTQHQLM
DVAMLPVSSIKDSPPSSPGSEIGGATQLHTTAALSATAVAVAAPQPGNVRKRGRKAKDTTIAAAAAAAAQ
AATVAAAAATNVQHQPDLKDVRILQNGAASSSSSSGPISTPTPPATPAAASGASASSIIAHTATHMLGNH
VNPNSSVAQKLSEQLHMEVQDHAIYTSDAMSSQFTGVPFPGKQRNNTSAASMNAAPAPNPLQSMFSGGGM
NSNMSIPQSLEQLLERQWEQGSQFLMEQAQHFDIASLLNCLHQLQSENLRLEEHVTSLIARRDHLLAVNA
RLQIPLNPIANKSEAHAATTTSSTTTSTTTTTTATSSTTCTTSVLSTLAASNMSALNAATTIAAAVSLAT
TAINTTTPTMVNNRPTVAPASSSGVLDYRGTGTATGGGSNNNGNMSNNPGVAAGGVATSSSNVSSGTTTP
MSLSGTMSNAASLGLRHVSAVHAAYNNSNSGSGSVGNSLHQHPSQNQQQQQHSHPSQQQVVQQHLQQQHQ
QQQSSSSHHASAAVAAAHMLGVGVGVGVGMGMGGAGGVAAAAGGNIGRGGSATALAGATSTTTTTTTFTT
TVGMPLRVGGVGVGGIGHGVNLGLGDLSSTTTTSTSSYAAHHAALYSTHHQSQLRRDDNLAKTS

>B5DW41_DROPS
MVKIILILLTIYGFAQIHGGFPEDALARHNEHRKNHGCPDLKLDSDLSNECAEYAKTLAGKGKLEPTKLD

GYTENLCMTIKKPLECIESWYIEKNLYDYNAPRLSSETEHFTAMIWKASKTLGIGLFTKDENHYVVARYK
PKGNVKSDFKENVPMATYVFVSFCEFSFIILISLCFRGNADHSTSFCLAVLILVAIFIERN

>B5DW42_DROPS
MPAPGSRPWVPCRAVRESILVRPGLCIWPLQLLGPHLPQRHANHEQSLKRRQQQQQQEAGGATAKQKLPQ
RVDKSKCSRKT

>B5DW43_DROPS
MIPIRNSLPFATLLLLLLLLATAKARARPNEATGEASLYNEADNVINADTNTLRNHLGTVPKGKLVQFIN
IFCGDCQRFAPTFKDVARDLYKWQRVLSIYAVDCAQEKNVQICRDFQVLKTPTLRYFPPVYTGNGTGIDI
PTVKPNEIKDLLAGYLAKEMNWNLLYFDPLRSDSNAKTTIGDHKGPGQAAEYIALVLQPKGSNIGRDTIF
ELLPYPAVVVRLVDDAQIFANFGLTPQGQKLAILDLAGNVQALKAAQETSQAYAASIAEYLAQKGHTPVP
PLPTTVAPKVRTVRNKEQQAILATVLRGGPAKIYRADLEQAIDKLLHIELPKADLIQGSNLTALRDIIAV
LRHLNPLNNNGQELLTNLHGFLLPINRLTGSEFADLVKSTEKKLEGNVFKAKRYVGCIASRPFLRGFTCS
LWTLFHYLTVAAAKPPYYLQAGSVLSAIHGFAKHFFGCRDCADHFLALAERKHIDRVTDHDAEILWLWEA
HNEVNKRLAGDTTEDPKFPKIQFPSKKYCPACSNENSQWNRTEVLKYLKIIYDNKNLSPYGLPTTRGYP

>B5DW44_DROPS
MKIVLKFLAYFGLSGAVVQGRQASILQYTTEDNIFLDSNLDDLLANNPLNAVVFYSHSKSDPVILKETVE
SLLVLRRLLNVYMLKCNVEVNKKTCADYHMESSHGMRYFRRRFPIRQGKKGLEIPMDTPLQITDSVWNVM
SRQELPLPDGSPNFRPIGPHDDYKSLFDQAVLSHFTHLVLVEQIEHYSKLGRDTILALLSLDNVIVRIVM
NPYYFEHFDLEFNSGEDKLGIIDRRGRAVVLHPKETNGLAFAEAVVSYLESKGLI

>B5DW45_DROPS
MTAPSDSFGALSVIVGPPKEKEEQKVQEEEKEHQEEEDLELEQLQLWKRRKLVKM

>B5DW46_DROPS
MHISSYEICLERVAEEFMGRRKWKNYQEQATRSQLNIEEQQPIATAGSDEDEDNKDNRNHCKPENQHHRL
EQQFEEDFDNQTSQDSSRTATPISIPSPPPEPNDWRPSDKCNFCVNGRLLTVNAQGQLVAETVAPTATVH
SSSSSTSKGPIYHNGQHDSDSNSSASLHQSGARNIANSGNSLELYKLLTQRAAKMTSMDSMAAQLAQFSL
LADFNLINSLASQQQQQQQQQQQHPPHNSVTTTTSELLSAAAISAPLKDTPSPSADAPLDLSSKPSPNSS
ISGDVKSIRALSDCPTPTPSSGRRTYSEEDLNRALQDVTASKLAPRKAAGQHRSSLMNKLGHHEQRLERL
ERLEQDEPDEPEDSADDAEVDSNASTPAPVYPELAQRIAATAQLHKLSSHLSEHNGSEDLGEDLEGGGQH
SPKLSRIPGPASAVAPIPIDTNVLLHTLMLAAGIGVMPKVDETQTVGDLIKGLLVANTGGIVNDALINLL
AASQQETSNGNAASLLLQQHQQQLNAFHHRLPKSETPETSSSLDPNEASEDPILKIPSFKVSGPPSISPS
ISHSHHQQQQQQHLLQQQHHHQHPHHHNNHSSSSNSHAHGHAHAHSNSSNRNGGGGGGRERSPHSASPML
AAAVSNSSASAAAAAAAGLLSASPTSIQKMMASNIQRQINEQSSHEAAAAAAATASSSLRNGSNSAGSDC
SNNGAASGNNNSSSSSSYKKPSISVAKIIGGTDTSRFGASPNLLSQHQHQHHHAHLPAGHHAAAAAAHSH
QAQLSAQEAAALAAGKGTRPKRGKYRNYDRDSLVEAVKAVQRGEMSVHRAGSYYGVPHSTLEYKVKERHL
MRPRKREPKPQPDLVGLTGPANKVCPEKLKPGNHINHTGNHHGVGGSKLSNALKSNPSSQAAAAAAAAAA
AAASATVPNGLKIFDGVAPQLGFQPNIFWTQQNAGNAYGLDLHRMAEAMRQQTNQTNAHERPMKSALEIA
EHMYDGIIRKTLQSSGDGNGNGSGSGSGSSSTSNGHALGHGLGHHGNALLDQLLVNKTPLPFTNNQSNDY
RGAACSSGGGERIKRSASPLGVYADIKRERLSADSASSDEERDRERDRDRDHDHSSNNNNNNSDLAHNKN
KSNSSNNNGNSSSRNNNNNGNGRSRMTSRDSDATDASSLKSDHSRHRHRHNKMMDLNGSNSSSSQNDGDG

DGDGDGEGEMTHSQMPSPNPSGSPNPHPCPSRSRSPGCSSSAVGGSILHEKLAQIKAEQLEQQQQQQQL

>B5DW47_DROPS
MSEPTSHLLPLHRIIMLGGEGVGKSALTLQFMYNEYDDEYEPTKADIYRKTIQLDGEQVQIDILDTAGQN
VNVVIRDSYYRRADGFLCVFSIADEGSFQATQAARDLVLQLRKDESVPFLLVGNKSDLKEERQVPLGECQ
ARAHKWGVSYVETSAKLHQNVHLVFYDLMRQISSRKASGSMATNGGRKCKKKGCHACAIL

>B5DW48_DROPS
MFSQAPVLSSQKSKLLSLDEQLIRQLFYKYHTLELSKALDKQSDESKGLSVYGKRLEGGSKRRTSTTSVN
PKVSQKGSVASVRPNSSFSHHSTAPRRHFESSFRDSSRHNLSATSRLFNMSHCLRPNASATSHISNVSKR
LQELAKAKQSPKVLNTRPPWHLTWSMRTFKASDRLTLLAKSRMPSIEKLYNPFSVAQRALTYKATERINL
LAVPRKRTPAVVEYSDIQVRSTRKGFRPGHLTLRLYNLAAPKLREDTTIRRKPFTVRRSAMRATLTPRLE
QLSQPRQQFSRNWE

>B5DW50_DROPS
MSKPKLDEEESEEAAATTEQLDFLPADLTATTTTIAAATPTTAAAAAAVRNLIGLQRTPFVPAIDCAASA
TASASVLPMGLLNFLEQGQGPEEEEY

>B5DW51_DROPS
MQHNNNSGSIGGSGNDSHVQQQQQQQMLHQQQQQFQQDFVWANIGNISGSNSPISNSLQLQLQQQQQRSA
SWTAECNKQRQITNNNNNMNVNYNQHLTQQQQQQLQQYMQPTYNNYTQLQLQQQQQQPQQLVPATTSQSN
NCYYACQQQQQQQHQQQQHQQQQHQQQQQQQFLAPTTTPSAVAATAIAATRQVIQRHRPKVYLAKALSSA
STANSQQSAPNDRQTASTRHSGPRALDPSLFQIRCRLRPEVTNV

>B5DW53_DROPS
MMASLDFRVLLALLLLGSTKATPSTETPSALLPLEARNADLSGEEDIISTSYVLPNQIFNEGKPYYPRHD
PVSGQLDFSAKKQAGIQPEINEVLDPNEKIVLSGASSPNIHDFLNLPVKYSSSKFVYPLVSSSYANLKYQ
GSNKNYITNKKPTSVVAPVTPPPPSYHSSNYFTVPTTKLTAVTPTAGAYYPHPSSSSSSSTSTTTKATTT
AATTTQGTLPPSRRPIPVTTTTTTTTTTTVPAAKYTTTSRRPIPLHAAPTTTQPPRTSTTRKKFVPTKKY
SPTLPSSSSRTPIEQEERRPSTTQQPRPTPSSSDVAFTTHHATPQAAIFPTEPMTTAKIYTTSSTSPPVV
FTEGPTPPPPFSPIPGTGIPNLDPADVYHTLGQKNTQAEEQQQQKEQQLQKEQEQQYQQQQQQKFQQQE
QQQYMQQQHMQQQNMQQQHLQQQHLQQQQQKQQQQKQQQQQQQQQQQQQKQQQQQQQKQPQQQQVQQQHQ
QQQVQQQQQQQQQQQQLQQQQLQQQQQQQQQQQHQQQQHQQQQQQQQQLQPPPRPPMTLSDIFNSLAEEE
SAVAHNYQQNQGFDSQGNLIEPIAPSKPSPFAMQQAVGQPLSQPGAPQRPSTAGAGTTPGQSPYPNDQKV
ISGSQENYSNEYVSYQVQQPNVMQYRPVPGQINNVVISPGQHSASFVLGSQVQQVSVGHPSVEKEPLFAK
DTPGVQYGQVINEDIGNIKRPVPVPYKPAAPQEPPMPTSYQQMPNVQQNSNFHQNGNMATAGTTVPAGSY
QEPPPEAPSPYQQLPLIGSNIRPANKPVAPKPEQPSPSYQPTKASTPHQTPPTRPAMHGDDTQDLLVSSN
IRFPVLAEETSVELASSAVGPGPPAGPHINGHAQPLSLQQIHNSNPVVFPKVKDEETPGANVQIQQHEVV
NLNQQQQQLKFPAQQPGHGEQPSRDMEPPPRYPTTSSGPQVPSTPGNRPPFYNEFNRKPQGGPRPSNLPN
ILPQFRPNAKISSGHPPTLKQDPGNIRLPTQGMKRPYNPSAPPHFAHRRQPLQQQMLQKRYPMNRISEYP
VGPQGVGGDINRRVYRLPPYGGQNMPYRPDHMYARRPNGPGPANGPLRSVDGYPAERHAPSAGSEPYQTI
NAEGAADFEEEDLVINDPPQPVTPSKDRMGVEETKLEPVVTLQMLQSQKKAVSLPGDDTGAGEIQVTADN
DPQEAEASSLSQQKLDPSGMYVVFPLKGSEKQQIEGEPPSAPAEYQNTPFSVIRDQPQEPILKNKKPQSL
QQQNKAQQMPKEKFPYPIEKPDPSYSELNPSPESQGHVPGVLIAPRIIHGAFGTGTETPIAIAYTPTEPS

VFRRTSAAGDQDQKFSNINLATPVISEIRQDTQTEEGLSSDFDLRGQNYEKNFMAPFYPSVSLGGGGPGA
SGAAAVNWNIVPSTTDQAIYEKNSINRADVEPAEETKEKESATASALTAEKNPELDSFQPQLQGGFKPIY
PPGYQHVEQQIDHEDAKNRDQDQPLALPLVAVTTSTAKPATAAATSSSSSSTTTTTSTTNQPGTTKAAAT
ATTTTTTQAPAGSVKPTQRKKSTFETSLAALLFGDEDEEDGARKSAEPPKAQAGPRNVPRMGPRSLTLS

>B5DW54_DROPS
MQLVTPPDGQKDNNKRQQQPPNRHNPNQAGRQLQSGNGKPKRGALSFSIEVALGRACGAGSPDTSAYTDY
RDSPCKELVVESLKERILDMVQRGSSGHDTEPFPGAGGS

>B5DW55_DROPS
MDNTSKVDNSEPSFQDAVVFKFTNIVGESYNQSWFVYHNYRLKAVSRNKVLLNINGTILHPVNDASIHGR
IFKKANGYKPWLFDIYIDACQYLRKRNQPVANIVYGLFKQFSNINHSCPYVGPQIIKDFYLRPELLRIPM
PTGDYLLALQWYFDKKLQFDTNVSFTFVEDLMKSK

>B5DW63_DROPS
MAMTGDDDDNEDNGDDRQTTYPYIRYNMHQRQRQSQHQNQSWTLEPGPRPDLFGGDRDGDRDKGMHCLPS
FKPKPKIVDWDRLGLCLGLRLRVSRTAIGHVAASVAAPWPNVAPIATHLRAPRRL

>B5DW64_DROPS
MHVASVWSTFLGLFAGSIAADIPDCNFFDTVNLTNSWKFPNGSYSYRGLIIPLSLAGEYDYEIQFDGASR
SVASHTRGCVCRLKPCIRFCCPLSKVMQYKRCSPQSQNKYAYNMTLDITQDNGTVTSKHVLEDFVVQEYL
PLPCGNHYALSRHNYTTDLWTLFENGTLLRQYDKRYLSKQDYCLQPRRSREQIGYNYTIVPYNCSIDPDE
QWLMSISVLFMAITIAVYLWLPQFQSLHGKCCNLYFICLTATFLLNVFSIFNVFDSKMACSQGWSGMLYF
YSPLAILLSFNLTIFSVTTRHIYLLDKISSKKLNGIEQKQMSKNRANYGVYLHLFIIMGGSWVLEIVAFI
CETQKFCKPLIVAADIIKCSQGIIIFLVTFCNRDMIRAIRERTHERRLSGVESATCSLTQDFQLKQDMK

>B5DW66_DROPS
MAGHAELKELGVFELRPLITNLSCKLSAVTAGFDEKQLLAIVSEENEIVLRYISAAGQEVVKMISWFRHR
VIHDVCFDPAGIWLLVLCFDNTLHIVPALALVDKTNILAACSLYSTTQVTSYIIPFVGPHECPNSKKCPN
HSTASTEQLQPAGSNQEEQLAEKFANVMQLEENGEPEPLGHELPDHMLVKNNEQDPAEVQQQPVQPQYDA
TGNSQVMAASFMASKTCPYPLSVVWWKTNQGLNRAIIGYSDGSICFVGLSPNCPMIASTAIENGSVMRLV
ICKDKTYDGVMLLITSSLKEQFKLLLEQKAIMYVYPGDSSPTTREGAWQIVMSVQGKHQSHSGMAAGPRQ
SNSSDPASDSSQLEEDVFAPPSSAEDVPPSFQAPTVPTDVSTVPAPGDSRPATADVQPQPQPPPPAPPSY
SEAVARQSSENMGVRFNQQQQLPTAGNSIDGILPATRARLASLKSLGSKKLHAIKMRLSDQRQKFDELMP
DSNLGSFAIMDSPSVTPEPLGNTTGSFYTIQNLRSTFLLSALHSNSHSLTVHSIDISLKPLFVYKIPKHT
QEILISSNILYGINYVNLHSRSDSAISTAASSLEEPLFANTDKENRDADTEKLEQQGERETSAHKSDTTS
ATTTATSSIDHSEMPSNAINAVSVISSAMASMHTSDEDRFNTLAQIGLFRFEQETVLHLYQMAQYRQPAG
QPETLTKEEKAKAFEIKDIHTPMDYVQFYETADVSSEFSLRQMEIMQSEFPKINYDPSYVITNKNVYTIQ
LRKEPFELFLDAALKSEWNQCRDFCNTFDLSFELCIEFAGDYLLRRKKITQALLTYNVARVKPIRTALKL
AMYGHTYALMQLSAMALKSTYLLRSRHLGHPLLKGMMGDITYRHSEDVRMILPQQREQGVVNSGAPCSDY
CYGVDESISALQMSPSSQFHLANLLLITLAEKAVNDKNYMPLWNFLVTNSKYHTNMTSIVLCQSGLFSSA
IILAKTRGVCLDTFNALISVVAQEFGWYNELNVCLYNLSETIFMETITYLPHVSMDYFAFIQEKLEHIRP
CVLLRLSRQLNPFSAALRPIVSHSSGCGANSNDNNSPHLFEFCKSLIETYLAVLIHMESQRVKHDSLFAI
ESSRFKPISAGCAYCACVVDGIAYFWGSSGIPSYFSAQKSTEPPEPAHAVKTLELLSQLNLQVHAIKCGR

QHTLILTNNGLYSVGNNSLCQLGIGRHMQMALQPMLVTALDGMNITMLEVGQYHNAAVADGRLYMWGWGV
YGQLGQGSCENIATPQLVSFFKYKKILQISLGHAHSLVMCQASKSYKEAYNCSNELFVFGSNHFGQLGTG
NSELGSDTKVNLPVRLETGGSSLRLIHTKFFTNLAVDDQERLYTWGSSPQALRLANQIKRRANAKQKLEE
NHQRELLSRQLEATLLPRPNSSSSTLVEPTTSYAAVVAGATPKVANETKDSQLDNEAAPSDGSERLGSKC
EAEKDKPTATPVAPAPATEPDPTEHMKPHLVDTSEVAGQTLQISSGLFHFALISSTCTLYTWGKNLEHQL
GTEDKDRRAVLKPSPIDSIERPMYVDCGADFTLVMTTDHIVRAFGGNSNGQCGRDQGAGLERMRQRVVCL
PTTKRLMRFESQCVEAPVEINLPRPRIRLDQDPIRYLKVMPPYQPHFLQPANISFDQRYSIGTPIYKSST
EQAATGQDSDDLLSSLENLSQNDVSFSYTAPPMMQTDANSSLDMDYSPEEQSYAPLAEQMTAGGIGTGIP
ANAAALLGGDDDVSTFTASSEGTLGGTALQQLRHYIHYCLYAFHGLYNPDKIGEYARLHRNEYRIRICML
NFRFVEAFRLCLQSCDSAQRTLKLFEYFSKDTGYVPLRRSDLKHLIYHMCLHCLERKFDMLECERYFMAD
LDYYLLELAYVFYFNNNNSTLEQNLNQKFKMLIAAAADNNQAALANSASGQQQLLQDTDVIFDSFTVKFK
TILCQRLLTYSDCEVSTAN

>B5DW68_DROPS
MMADSHWLNWCRLCAKDDVRGNVKVYTNEKGQGTWDNVMIMAIRRYFEVHMRLEDELSSVLCTECYTLIS
ELIDFAEHVTKVQAIFEVLRRTETEQNKQLDVLALRQQYGLCEDDWSHIIKPMPVPETEPNLIYQDHVEV
LNESGSLPKQAKRIQGAEESQKQQFKNTPSDNKQEFVDLSPFFQGEKLLKNNMDAIINDLEAEDVLIAEP
DVIKRHHERIDSSLEAPCGADDEDIKPDVSINYDDDQEFLHEQTSPATIAIEPPISASPPDENSRTDNDE
TKSKPIRLEHPSKRCRMECKRCGKTYRNRASYEKHIDQEECQRIVRKVKMDKNTSCDLCHKTLSSVSALR
LHKEGMHENVKPFVCDSCGKQLKTLTALNEHKLVHTESRPFECPVCKAGFKNRARLKVHCQIHEDPSFIC
NICGKKLQTRRTWNMHKVVHKEERNLKCEVCGALFKRSKTLKTHLLSHTGLRPYICNYCGKTFACNANCR
SHKLKKHSQEIKQGDREGMPSRLSVPTLEELRVITQKLPKDEKQP

>B5DW70_DROPS
MKIYKQKLRDAWLQMPEFRPWLRRDPEDSYRAHCRFCKCGVNTKICDLRAHARTMKHRKRDSEAVVETKP
NHMTDAEDAIEISICHNNINVVQPPKVKSKCPAVKREKIRKVVKEEKTQPIDYEQILQNLALYEPEQVMF
STCPLTGPEEHNPEEDIEAVATTDALIDEDMINKAVANAINNQKDSSQIFGDFVADRLRQLSIDASEFAK
DKILKAILEAASIDRTVSYN

>B5DW73_DROPS
MKYLARAKYEKAIKYRPRNAEEENDEEEEEEEEEEEEEEEDKEDEEEDEEEDEEDVDIDHYQKTNTIQYT
DPPVWMDTRYHFQHNSRQPRLRLESNPLLEYYPDDKNKEVVCRDQSTIIGTDIVTSFIKPYIYQKSYGKR
NLYRKWRDDLTRLQLLGYCHTPGQTSVFSFLECITDRHMAFESLLPKYPYHQVQAGPQSKTVDSKSKAKV
QGIGNEKRLVKLDHRPTTNSRNFSTPSGRKSDINIEKENEDKRLSKKRSHVRVVVPLSSTRLSVGAKDPK
TRKIGVTPNTSLFPATRNQGLLLLHPRPATNAQNLSASSIQKSHIKGEGRTQTKNRFQIRRTTSLTSIHP
SVGVDDPKTREIGVTSNASTFPVTSNLGLMLLDPRPITDFQNLSSPPVAPGFRSQVQKTTRKIPMELTIP
MRYLTSPQNVLMMDAAPEDDQMIHTEAPVWKDTRYHFQHQSRQPKIRLESSPIMEFYPEDGKINTVCWNK
STIRGTDMVMNYIKPHIYKKPYGKRHLYPAFRKANGTSGMPLLFCHSSDSAPVQMFLKCMIMRHMMMESL
VPKYPYHQVQPGP

>B5DW74_DROPS
MYKISVMVFLCLCCFITTIPLNRRHLVVSQGTYPGNRSRLVVDYDLGLSDQAKDSISAGRGIRLFQMPTF
ANAQTRREYTGAINTTGDITFSHRSTKNYDEKRKPRARDGEGEGGGGEGEEVVNEAQEKRRARDGDGDGD
GEGGDVVQSQEKPVDDVLAMDQEDDEEGSDKDEEEVVENKLSTVSSTRKELKVTELEPEPELLPEQEAED

EEDRDDDDVEEIEDLAKKPDVAEQNMGFKGLLGPKTPRGYGFLKKQGESGFGEAISYAASWKDTRREEWR
SALARAGVMSNPLEDYYPSNTKNISTVCKDRSSIMNFPRVTKWLHKVLTNITYTQRPIFVELRRELFKFG
KSELCHKTTMVKWKDYQYCALMRNQRMEYMIPHYPLHQIGYRLLFKAKKGA

>B5DW75_DROPS
MPMISNEGVTKLGEETGVSSARHPHSEQLLMDQQDMQMAADNSDLGLRMFPIVYHSEPPPREAPRSSGPP
QLENKWTRRSSVDPLLNFWAKAEDKVTKGTTKLLPRKYDEVEAEAGKESDGAGASASAGSGTGAGAVEQR
AGTEMTISLPRYVTDHMGKWVFNPWGVTFGDVWEDTRFQFDGKHSVPWILVDHGDPGAYRMEKLHGSVCQ
GKSTIYNVPKIYKWFPRYIDTKKRYHRNILTNLRKEIYKDGRSENCHTADLDDWYAYMHCALLRNMRMDY
FVPNYPLHRWYNTD

>B5DW76_DROPS
MRPFMVDAMPMKANESVTKLGEETVVSSAKHPYSEQLLMKGVTEAEYLDRQDMQMAADNSGLGLRMFPKV
YRSETPPRKAHRSTAPPQLVKKWTKRSSVHPLRTKGLLNFWAKDKATKGTTKLLPRKYDDVEAEGGNESD
GAGADSGADAGAIEQRAGTEMTISVPRYVTDHMGNWVFNPWGVTFGDVWEDTRFQFEGRNWLPWVQIDLP
ERGEYRMEKLHGSVCQGKSTVYNRPRIYKWFPRYIDTKKRNHRNILVNLRSEIYTDGRSENCHTADLDDW
YAYMHCALLRNMRMDYLVPNYPLHRWYNTK

>E1FMB3_LOALO
MESVELVLEPVGNDYPGLVREALAGREPNSSCSGSLSSCGYAWLIVGQSLYVWKYDAEENYTSAPAVLNL
PPSGLPYNAQTVCVYKRQGFSALGIIAVTPEGIVRHWSLPDRQFSDSSVDLDREVALSLGRINLQSSEGI
CFLLATTTGSLFLLNVNANVTPTPGKRQRLSSEITIKIIHTNTRNGIGGKLASAIFGEGRQSGAKLVKAL
VYEQSSGDLVDARSSASSNSNTGVTKNMHILAVTEHHFHSYSIRDSAKMWSCELANMASEHFALNLWNLY
DVEEHRHWHEEIKTWILDAVNIRNGALVLFACNNKAISTQIYFAFGYISEYDGAHPPVALEWFCVLKALS
NKYELSEKNVVANITLRLPTGSEQLLHDKMEGVLVVTQKAVYSFCLPDRLLKKTPFKLILCAKYNATFIS
SGRDARYCYIFLEESGLQRVRLLPRGFDANLAQTIAQMSKMEVQPVNLSSAVSDRRALALAFYLFSKADL
YEASNVMQELLENRHAGIAQICVDYAIDLADDIPNDEPRAVTNQISSKSSFLLEVHLEEQKKRVLAMFIL
FIKSLALDDKLNRTVKTHLCQSRSARSQIVELIEKIDIAIALYKWSKQNPSTVFDAALERVLSRRKESID
GERELLTRYDCFFRKVSRIEELFSGLFEEEEDIVANDNELLDYKLECIEYVGAALIIGKEAIDERRDDIV
LDIENDLRWTQEKHILRPFIKHLSILFNYVNQAGRDCPKYVALLKQGVLIAAFIMNEQAFDDRQNSPIVA
RFLEISEHAIALELAKRFQDYKTIIRLACALPDLERKAKIQEYKEFFSSAKFCNMLYEYYLENGYVKDLL
EMKEPDADLFFATQTNVGWMRDLENGDFSKACRTLKTLSQKSNDDVVLKRRLLSFAKLSALCEDEVDNNF
LEGIKRDLNLIKLQQKIDPNLEMKFDSSEPASKIRSCTAEEIIRAHLNNASCDVNRCFDALLTLSTLMDE
EASDRTGELVHSLQTKIWIAAIKANSDYWKKVTRDDDSRYPTVYSELLDRITAYAQLSSERKLELIPDTK
KLAECLTQFAHNKLFGVLLRTSEEAARRSISSREGIKVTSEAILDSMRS

>E1FMB5_LOALO
MDSLYRSEEMCLAQLFLQTEAAYTCVAELGELGLVQFRDLNPDVSAFQRKFVNEVRRCDEMERKLRFLER
EIKKDLIPMLDTGENPDAPQPKEMIDLEATFEKLENELQEVNQNEEMLKKNFSELTELKHILRKTQQFFE
EVEYGRWPYARREENRRHFIPEEEENLLSESRSTTVTLRLGSEKTIVNCCGLCFSGSKAGAETIVPPNAP
VGSGLPEQIVLQETEGIGIELTGAGVTGQMFANFGFVAGVIQRERLPAFERLLWRACRGNVFLRHSEIAE
PLIDATTGDPVINSVFIIFFQGDQLKTRVKKICEGFRATLYPCPDTPQERREMSIGVMTRIEDLKTVLGQ
TQDHRHRVLVAASKNVRMWLTKVRKIKSIYHTLNLFNLDVTQKCLIAECWCPVADLNRIQLALKRGTEES
GSTVPSILNRMSGITEAPPTFHRVDKFTRGFQNIVDAYGIASYREINPAPYTMITFPFIFAVMFGDCGHG

LIMLLCALFFIYREKQLEAARINDESVGPYPIGVDPIWNLAEGNKLSFLNSMKMKMSVIIGVAQMTFGIM
LSYENYKYFGSRLDILYMFVPQLIFLSCIFIYLCVEILFKWLLFSAKSGHVLGYEYPSSNCAPSLLMGLI
KMFMMTSRPSGFVNSEGNVYPQCYLNLWYPGQSFFETLFVLTAAACIPIMLFGKPYMQWKKHKEQSTLGS
SNLSVRAESNGDDAHIIHNDLSRSSVMRIEEKFDFGDVMVYQAIHTIEFALGCVSHTASYLRLWALSLAH
AQLSDVLWTMVFRQAFMLNGYMGAVATYVLFFLFASLSFSILVLMEGLSAFLHALRLHWVEFQSKFYKGL
GYAFIPFSFDKILEEARGAEENM

>E1FMB7_LOALO
MTEATKIDKSIVAYCNEEKSWLHVHHDEDILPNYFENSIGLDEFGDVEFSSIQFQHNFRLHRKSIQAVIE
VGSSLQSFVPMIVYHRHVWWNYGYKLTLEKTNPGKKAPRGAFGNNSPNNQKPPPASNVRVTLGVKKNGIL
EAIIEEELVESPHGTGFICCNLINRSILHELIENHEFIVLHISMNIHKNYFNIEELIESSVTLPIKTSMN
PENYDLLEAVLACEPNRDSDFVFTRGSGANNDATSYHVHQNILKRRSFMLEGILRQKYSLPTDQLLVIDA
EDRIIFPYLTDNDMKFLLTYLYTGEIMLPKFDGFARVGRVLSLLIDREQLINIFMQWQKLIVQNLLQIEK
KNNNDLIVEESFKALISVFSAPYGALPYAKRMALSLLADQITKSNETLAEKYDKQSQYGRYQVGHFMETA
LKLKRFITSVKKIPHPL

>E1FMC1_LOALO
MFNRSCGSVLLGISRQRIAFPRIRRSFDTSNDGNAGSQTESLQRSKTSDKNISIRQESKNMAPKATTNEN
LKPEDIRKKLLEVKRKKEWKKPKPNEVATEEILAAVNSVVKDLHHSGISDKRKTRNELIGKLIEYEKEKF
EAATSAQYSELLSDRAFATFLESLKEEKNRPPPSAVEKRQMRQGLLLLKREIFYQALQSGHKAEDARRIA
ERAVKIAEQKVVGKNEILMQNYTEMQKKNMLEEIEQTERESAFYNIALQLASKMLYPDDLSSEESHIALS
NVIHPDCRVENIFKNIDNKLNIFKIADIPKLKDDSMKQWCEWDKNAAAIWNSSFGPANAFEEMIELTEQG
KMWPYPIDNEYQIGEEEDVDFYDHIFLDKYLAQYNLPEEGPVTQFMELVCVGLSKNPYMSVKKKHAHLDW
FANYFKEKAEEIKRIQESAGVLVEGSI

>E1FMC2_LOALO
MTEWKQFRWDRYDENEGRGSRTYSCIALYYFDNKGHSQCVEAGRRLTLIEYHPAHRHQPSPPPYHLLLLF
YYYYNNIITIIILSYYYLLLFTTILLLLLYYYYYYTTTTTTTTTTTTTATTTTTTTTTTTTTTTTTTT
TTTTTTTTTTTTTTTTTTILM

>E1FMC3_LOALO
MKLPTGGPRTTGSPQGRGEIILIKSVIKEDTEGNLSCKVNSFLNVNENCPYKNDSSIEFRRKETMEMRCR
ISCRKSVPFGCLSIIAPYSNCRRHCTNVGVCVWMYVWVDTGVHMDDIALIDGFHIVAVSVATHPRAQSVK
FFFPLSPPPLPSI

>E1FMC4_LOALO
MAENPLNFVNYGTNNQALWQQHPEASASVTAFSNQSIQNSSWTTKMLPSNNPMSSQGYFFDNIAVGSSVN
VLSYLQQHPNSFPVNDSVQPLFHDSSAEALTYNYPPSCSVTHHLVNSVRTNPVGQSDTSFQPQMLPNFST
TPFSSTILPLDAPAPISAVPLQASSFSCLPDQDTNPDSVVSYAQCLPPVSTQPLTSVAYRSNARNESDLL
HNFPIINKHSQSQLLNDHYQMNGNYPGGMGSLQQEFAKMNYGIKSIRYVDLMQERDLMKYGTEDEEIALP
PTVCNPDARCNPSIFRCTLSSVPQTQELLKKSRLPFGLTLQPFRDMKNLNIIQTSSIVRCRYCRTYINPY
IYLPDSRHWKCNICYRVNDLPDDFNWDPATKSFGEPTHRPEIKNSTVEFIAPSEYMLRPPQPAVYVFVMD
ISQNAVETGYLYTFTEQLLITLEQLPGDDRTMLGFIGVDSAVHFFQFRGKARPQQLVVEEYSDIFLPVNC
GLLVNLRKNIDVIRLFVQSLPALYESNSSTSCCLGAALTAAHKLIADIGGRITVLLTVIPDLGPGALKSR

EDPNQRAASEVSNLYPANDYYKRLALECTGHQIAVDLFLMNTDMSKFSTGCIFHFPNYHINHDQVQVKRF
QKQLNRYLVRKIGFEAVLRIRCTKGLSLHTFYGNFFVRSTDLLAMANINPDSAIAVQVQMEENLIGINTA
CFQAAVLYTSSRGDRRIRIHTLCLPVTKDLSTIFSQFDVKCAVSLLSKMAVERTFMGVSLTDSREAMVNT
VVDVFGAYNSAVSRMNRASSMLSPVTSIRLLPLYVLGMLKHRAFTAGQSIRLDNRVAALLIFRSAPLEVI
DLELYPALYELNHFVENEADPPRLHLSFEHISRDGVYLLDTGSYVYIYISSNVESSIIKRLFDVNTFEKI
DDDAKVTIAMQLVLKLVWIFLLLDDGLLSYTFLLQNTFEALDNPLSNRVHSFLRKLSIYRTVFAPVILIR
EDSPVRSLFSRRLIDDRTESSHSYIEFLSYIRQEMRK

>E1FMC5_LOALO
MDPLVSYLGPSFGTTDSSIQTAKCQMEYRGPNKNQWKQWLPLRDIVPVGGEGKGWEGGGGVSACYRSFPD
SSSWSICGREAYDMASAILCPFTAVVSNRSRKGWKALFYGFGSHTSLCVYSCHSHDVVIPNKIWLGKRYC
KNKDEWHSYWPSSDIPHA

>E1FMC9_LOALO
MTTEINTNETPEINTNATPLKKFNVGILGFNSSNFPNSISHQYITINNHILDKYISSTKVAISIQSALMH
GSFEHLKNATFGFDFDTAKKTHWHENIQQNY

>E1FMD1_LOALO
MVALQTTADDVENNENLPGMICTPGEKSSTNVIHSDRTDTIVPAQLTLMEQNALLQTVKQSGRLKAVLKG
KKPPKITRVITYATSDDITNADCSARDDNVPLPCVVYGDIHGQYSDLHRWFNLNGWPSQVRSLFLGDFVD
RGSHGIEVVALLTALKVRFPKNIFLCRGNHEEESLNRAYSFYEEVCARFSGNFEGKDGRALYPYFRSLFS
NLPLAALIGGRILGMHGGISPRLTSLQAIREIRRPLEEFEVGSLECDLVWSDPDTSPDRTGFRSNFEREP
LYGIGQLFASDTVQNLCKKLGIDMLIRGHQA

>E1FMD2_LOALO
MKLNKSSNNGLGEAKVFPKEIDKNKSDVGIPELKRAAQDINIEQRISSGNSMMTNKVNKTSWGLVSGVVC
TAVVIACLVGAIIVLINKRRQKRQAVY

>E1FMD4_LOALO
MMMPSSLGGGSGSAKHHQQLMQSASMLPSPYNFLPSVFANAQLDMPLFRPPESNLGAMYLSQLAAAAALN
QGQNGSNGGTTNEQQEETVGENNKAELVASTSSEVAISSCDAESPLPPKPFGAVDRETESTNERNSVDNA
ALSPTKVNTEDIALVESENLQPSSVSELQQLDLSKWISDFFPGLPNNVSALNMAALTQRMPPMLEQIQLM
NQYFSPGSVEDSLRVLAALGSTNGSAGMVPSTNCSPSSILNSPFASSAGHSKDTYCEICDKNFCNRYFLR
THKWKKHGIPFPGKNSPSECNVTSLASPTVPQNLPLDLPTTSTGINLSPAEFIAALCNQNVLDASQISPS
KTTSHPTSVIVNCLSSADSSPTKRARTTDEGSKDDDELLGAGRSESVRTDEEALASVASQLPNTGDLFAM
IARQAQENAIAQELLSSFKADGIQVQQQSSPSKHSSASKEACEMCGQECENRATLQHHLLLQHNILNSFL
SSFNLLSPLSQLQPQQQSQKQQNNQDNDADRSRNATAMPGMVTSPISASRQPKKQYTTTGKNYCDVCNKE
VCNKYFLRTHMLKMHGIVIDEHKTVIANIDTLEKEKSGTIAFRCDICNTNVGQTRESLKQHKQEVHNVVS
LPTSRGHRGSLSSTSSITSTSTLSQALKTADQSGNNIE

>E1FMD5_LOALO
MTNEQCNMEMIGTTGNIPTEREVGGEEGRNKHTCAYHSPISAITEGCDVTVTYRCNPERKKETIAGKSHP
PPHALSSTFRQHQHNQSLADNNPQLRASFCAHCIWVGGGAVILIAGYHVGHQAGMVSRNEGMCKQFTPSP
TLRASNVEIN

>E1FMD6_LOALO
MHRKHLYEVVMARSSNKKLIVDERYRNPSECCCCHPATPSLHTIILQMPNNAEHHLFSLQVIASELRTYV
RISYKVERLELELFLSENEISYTLILPNSSKETKNFMKAFK

>E1FMD7_LOALO
MNDHQQLHIESAADQDAGRYSCVAENKPGRAEKDLIVAVLKPPQMDGQYRVVELAENETITLTCPIDDPS
VEIQWTKNGIPITTSNNLQLSTSGLKLHILHGQLYDAGRYVCRAWNDAGEAIAYINVVVLVPPKITGSAF
RTIESVLNQTVNIECKKTGIPTPNIVWSFNGRTIFPSEKIQILDNGTLLVLQEVQVNQEGRYSCTATNKV
GKAEADTFLQVTAPPRISTFVDELKVIQGQGQTIRCEVSGTPVPKVEWLKNGQRFNATTAQSSSNLHYIH
LREAQVSDAGRYTCIARNRAGEHRMTTQLYVLVPPTILEGERVVQVKENATLTLECVATGNPKPMIVWKR
DGRPLVTRDSRFVIESSKASDAGRYTCEARNEAGTVSTDFEVDVFIKPRFRDLKAEVRVRNGDRARLECK
VDGHPEPIITWMRGGRPIEDMKNIILSPRGETMMILKSRRADSGSYSCVAKNFAGEAEASFTVIVLIAPH
IEEQIDQNLRVVQGTRVIMHCPVKGNPKPKVKWLYNGKPITIDSTKILGETDLIIHESKQYDKGRYTCIA
ENEAGILNTNYELEIIGPPKFHHRGEAVYEVILGKTVTMDCDVEADPKPEIHWFRGDSPLYLRENIHISP
DGQQLTIRGVELSDGGKYTCKTENEAGAADIDLTLKVLVPPSIDTSNIIGNPLAVMGKSIYLECPVSGIP
HPSVKWYKDDVPISIDDDRFVVEQNNQTFGIKEVKVSDQGQFRCVVENKGGRVEQNFNLEVLVPPQLETV
QPQKYTKQEKDSLTLFCPVKNDGDSATQTEILWYKDGRPIDGLTLPNIKITSDGQRLHVARMSVSDAGNY
SCVALNRAGESSLDFYVEILCTYLNSEYC

>E1FMD8_LOALO
MFFIAAAPQLDYSRNEQQPHVVAGRPITLWCMVSGYPLPTIRWIKDGYLVPINENSGIRMIDNGQGLEIL
EAKREHAGVWTCEASNAAGKTDYELTLDVWTLPTVFIHPEDNVRPVDSVITIQCQATGNPEPSLSWSKDG
QPLITSAEGARISIKGTRLDIPRLKQSHVGEYTCTAVNEVGTSSATVHIDVLVPPRINRDNIEMSPRLPT
SQTLTLICDASGKPLPQIFWYVNDTMIKETTSNVIIGEGGRYLQVKDVTLNDNGAYKCVASNIAGKDVLL
YTVAIVQAPKILNGGNYQVIEGEEARISCYADGEPPPVVTWQRNGIRIETGMRYITEDKMLRITDARSSD
SGLYVCLATNEAGTAQQAFTLEVFVVPRIITISPNESLVPVDSPFSLKCGVRGFPPPDITWSLNEQIIGK
DKAEYSIAEDGTLFVAKAPKQARLTFKCIAKNNAGSDSKEYVIKVISPPVLREEGIKIINATEGDPSLLI
CEIEGEISEIYWYKDNEPLVLKPNLELSPDRTQLRIHHSKLHDEGMYSCVAINPAGNATQIQQLYVGVPP
RITEKPRRITVKSGQQAELWCEAVGIPKPHITWLKDDKALSQTALDDYTDVLKSTAFFPNVSSQDGGVYT
CKAENWAGTSYKDVDLVVLIPPEIHPERLNMTANVGETIILTCNTTGVPEPAVSWVKMPNIDIIGNEKKY
QIYGTALHIRNLKPEDDGFYHCVAKSNAGQAIGSRRLIVHDARKDYKVIWVECDEFGQPIKTTFVPARGD
VPEGDNNLLPWKQDLQDLPQNGTDGILIRCLPESRGPRRAALTLPRFIRSPRTQKVRQGAVAHLHCSAIG
QPKPHIIWIRNGALLAGISQSSDSYSILRVKVESNEDLGDYTCLAQNAVGFVTSIATLLLDQIAEKTDLL
GYQCYATNRKQAIEISFLEIQDAAPRVQVSPKQVFVNLNESVILSCKLMSNPLTTMVQWTRNDVKLVNSS
RTRVLANNSLHITKLLPSDRAIFKCVASNRYGKSYDDVKVIVRTGLAGVINKVKGVVDGRRLRRETVLVS
VKPEMEANVVSLNVNGIFNDQGVTAETVLGYMVVASPQLAFNPNQENSLASVEFHRVIDYRFESGETMRV
YQKGLGLDGEYAKFEIIFNGRVPHFDCETYSWVDQAKEEMVEQEPGMIRGNGFAILRIGNQANIPF

>E1FME3_LOALO
MPSSVGSEIDDTLYTSHPKTGVLLLPQLYCRGQKYSKEDFGSTIEILRVASKSALSHGFGYIAKVW

>E1FME4_LOALO
MDGSDEVDEPIDDVEDFHMSRWHSPYQLFGSEKIRGSAKMPASHIAREDNGSR

>E1FME5_LOALO
MADGEDDSSPNLSISLPRQTLIRGKKRKITLHSMVLRRRNSGQADPAEPPTVQTSTPVSSSVSYLQSVPV
QTDITDMAARKRYELRRRRSLQHSEEDGCIMNSMVAITDELNLVNILRVVPKSMLQFSLEWILGYDLMDT
RRKN

>E1FME8_LOALO
MRTEIVFILIIIIEGIFCNDTRQRLITLLRTDQKFHDQWIHLVELQHRQVHEQNMGGALEEVTDLLLNCA
PKLDSKLDHIDYVCPGDIELYAELGQLTRYCNDSIMELIKGRVDSCQHASYTSSLPSIVKFLMKSIQNLT
VIETSKATKLENQTQYLVGQFMEHKSWRNKWKLIVIMPNIEDGELREPEQSAVEVMKSIKLLYEVIPKRT
ILIVVRSSTLKLWQDASNTHRACQTLLEPWKLYEKLNPVSIWDQVEKICGLHFQSSLFTVQILPLMKDAS
LPFLPGSNQMDLSLLGHDCVHFSPRGLSLLHIAIWNAMLTRLPDRSQAFNFSLERPLCADPQCPFIRTAK
NSALCVWNIQKADGNGRRSEQLIAIGVLIIATVLFVIVLGTICCFTRYHNKVNEVAENLPKKPPVGADWI
SWKYIDEDSSASYKF

>E1FMF1_LOALO
MKSPKNREICLSPKPKIICDFFAGNSDSQIIELLSGSNPSKRLHKKLLNSKEKYRWIAYMKVIKKRAKKY
AIRNIRRKKSNKILEDKMPEKESSGSFCSMEEG

>E1FMF2_LOALO
MCICLYVLYLYVCICVHMYVCIYMYVCMYVCVYVYACIYIYIYMYMCVCMYVCMYSCIYICVYVCMCMCL
SVNVSASASVSICVGMIDLCVPYTIQAIGPVVHR

>E1FMF3_LOALO
MVLELKKCGGAAYHSMSISAGDGTPNRSLTANRHLQWKSQKVFIDRIYQISNNCHPGIGLDQAAAEHIQE
ILICLFLNYWNVIRKQSKIWNECQNTAEALHRIPRKKLHDKRNANSKSLMSLLHTLQPKLKELLGYKLDE
EVMLYLLSVIEYIAADILKWTGNYVKNIRKCDPTIGLQNLKIALSADTSLMELTEMLYNDEETSTAGILN
DNVEDIVQEMSYEEASRDFNRDEAQYLRDLNLIIHVFRRRFETVFEDSDEHYLDEMFGNILELHELTVKV
QRMLEDAIEMSDTPCVGAGLWELAEAHEFDVYIAYMDLFKRSLTTVVEKVLNDAKYEQFFMLEDRAHSIT
PGGETFRLAVKYVLPSLLEIPVIHFFRYVEFTNILCHLARPPDEVEDLKSAKSYFSGLAVKVESLCPLFL
INHLKTEQSIRALPESNCSRQRRRIQEIQRSIELWEGKEIGYKCAEVIREGDLLLLRSGPVSSADTLKKN
RGTTVRHAFLFDHLLVLCKTLRSSRPEKPLYKFKDKMLIRKTDIFDLTDTEELQNAFKIVSRSITHDRAD
NTSWVLFCRTPEEKTSWMCDLVKIQAKSSLDRMLDASLKEEEKRVPLILPTSDQYRFADQDCDENIVFED
YTSSSGIPVVKHGTVLKLVERLTYHLQLLIERFQVPTPPQLQVAGANNTSNLESRELLNGPYATVHSQGL
RTQVSPTLLNRIERSYQRFRKEYEQPIQCRVLSVIRQWVNNHWYDFEHNPILLQDLCSFLEETDSHGKVI
NQYKKWKKRADTIPSVAHSASQSEPSPSTITSYGPQKPKILWHIAEKGAIETYDLLSLHPIEIGRQITLL
QFDLYKAIKPIELVGSAWTKRDKDLRSPQLLKLIDHSTMLTYWVARCIVETASLDERVYMFSRVLEVMSV
FEELNNFTGLVALYSALNSSSVYRLKACWEKIDREKQVWYEKFKKLCNPHWKEMIERLKSINPPCVPFFG
HYLSKIFFYEEGNSTFVQSEDLTHEQINAEGNTSVVGATGRKVMVSFVKCRRIASIISDIQMYQNESYAL
EVEPSVRVGKCTDMIVKEYIESFGKNFFVMRHALPPCRLFSFLR

>E1FMF4_LOALO
MVLAESLLRFVCQSLDGWPAHSARGHFVSDPSVKTSLAGYIPWRRHKTLPAWLAALIMARKRARGEMNSI
STLDCPLLHAK

>E1FMF5_LOALO
MDRDKATVKAVTYTFNNNAISKLLKLMLKKMPADYAGKMRKKSGLIVGAYRASMESLFPLCTRKKQAIFP
AKLLFASQQLMRGAVNPFTLLKRNMAQMVFALAIKDDESGNKQRFAPIIIGCLSTASQQSNMLTSMIFNF
IMHLCAVKKDTMFQYGAVQLITFLSAGNENLYGVTIEPRSEIDIDNLNTLISSSEEILMKYAFWITFTGK
MTAKTKVAQKLREWLPKASVDLSSVVESDAKVADLKLEDFDKMFSLLHTELNDDFTHINELRNFYAHFRS
ATENAKFAD

>E1FMF7_LOALO
MFSLQKIMELLMLIIIFIQLWDTCDTFGCCPIAIPCMAHRCKPCIPIPCPPPLPPPTCPPPLPPCPPLPV
CPPLPPPVICPPLPPPCPPPTICPPPPPPVICPPPPPPCPPPPICPPPIICPRPIICPPPSLPPPPPPPP
PPCPSPSLVPICPVPAPKMIPTYAVPVINDCCCTCITPCVNSQMRIHGAKIFSASLAVDLEHDSKCNNPV
LQSIMEENMADDATIAKRAIQREAEKKLFKKFNVICSEDDFSYIAYTDTFCQHSNDDITCYAFSPLLEI

>E1FMF9_LOALO
MKKLKRRISAAFRGSDMSSTLPVRNWRLSDSMSELAERLAADGVIIEECDIPSPKVPFPVFYMKHNRLPV
PVSRHFRPDYPKNRKAYSPTQHQYSLFPRKPCAHTSLVIYVHKFSF

>E1FMG1_LOALO
MSFISDINNTGLLCQHAAWNAVVVHDPVNASSKCGFLYSVALLSGFIGHKQRAPTDSRKIRKLLMTQRIE
NQNELLACGFIHVFNGQQISYKKQRTTNHQSYEPSCRRLEIVGIAWDCNHSTSKAGNRRIVLIGIYVHDS
SEKTKADSTVLSAAADFRIVQSFTHVFRSFEFFAWSGRSVFMLWLSIVAIISLSVTDTHV

>E1FMG2_LOALO
MIFGVFRQVHERELRMITYNSVLMDMQLEALDLSYNDIKIIPSIDLRQLGLRTLSLHTEIGLSYSSKLRI
LEQFVFSHIPNLRAVNLIYSKSFTYLSPHIFIKNTLGVLHCTPEILRDELLSVMSQGFFDHNPLRCGCII
KFIAKDITHLRNL

>E1FMG3_LOALO
MKKVAVYLNYLQRQCDIAFTHSKDKAAFRQEKMNYTVTAGAIFGRIRSRATATKRYPFWQQDYTSLQKGI
FKDKKNYFEKTNELIRSQHFCSITINDFSTVRGIVDIDDRNLSICIIFCPTVVHANPTLWM

>E1FMG4_LOALO
MVELVRAQSAVVLRPSYSTTIYRTRSVPDLNLYSRYTSKWPYRYYRDWDLHDDYWYDRHYYYSPLYRSTF
FPRRYYYNNYVLNPYSYWNYPYSYWTRYRGYLYGYPSYYSRYYYYYDDYPRYRYAYSYPYRYGYYTPFDR
YWLSSTYWDGRFRVSITKYIPNYQIYNVD

>E1FMG5_LOALO
MFGKKLSLCTRQQLAQQLPDIRKQSAGKHDMMASSTRNYDAVAHHEANKTIKLKIKRVKTSDPIVSDTCI
PPQQLLEEDLFTTKIKMNAKRQLHIFVENSKDN

>E1FMH1_LOALO
MFESEQTGAAVNVALNEPDEIACYPRMFFDNKEEFYLTLKISNEQAKESNFFIPYEVRDHYLKGHMGLKV
RKHLTFTFGIIIELSSSQPPSDNNLILISNSTDDQALMSKQQNK

>E1FMH5_LOALO
MQAATWKPFSNADASHILFLQKGPHDIINYKLHCLCPRKYTPYVEISSNSGTLARNFRKKIRIRTDSSTI
RDAEFKMFIGNFNHFLNIVKV

>E1FMH6_LOALO
MKNLMNATLGYVENTMKRLVNVTEVADKGSDKRGSDITHRIYNINDTSAMIRINGTEQKDERMSPTRRMR
RIVEALLSEMRMSMNMRLKLEVGSYDLLVSKKETPTDSICQN

>E1FMH7_LOALO
METLNRHQFEILEQCLYYYLAAEAQRKAQYFNPIQALAAFPPETGNMKGSRVKVFFHQITLQHFIMNEFL
KELNINGYIIIFWDDNRLSWNEEKWKIKKLEINSVSHVWVPILTAQTFDTAVRNGDLMEIRRVSINNNGT
IRAIINFSLRTFCDDSDFKNFPNDMYKCCYQIEPHVNQGGIDFATSGRPVFTDTKYFRDYGWYISGSMPT
IQIMQDSRVPQLGFCLNLKRSTKSLHIQLSLPNTITTMLFLLTPLLGQIHLQIFAKMFILFLQFSTLQLY
SLLISPYFGSSSATPNMLRFLEFATIINVISVSVSITLWMCSKIRRNLPPWNWLIRSSEFINRCVCVFNS
TDSGISLNENDKSSTTNNYQQDWTNAFVAMHGAAVFLLSLIFILGYLMLS

>E1FMI1_LOALO
MYGSRKSFSEYPLHFLINARKKDSHKGNLNTSKCMKILLLSSFAAYSNFHPFEVCTEQIVIMKPLSENVD
ILKCHSKGIDENLEIPISNVQSADDQETVFRNINPCSFLPAFHPLTSFFISGNELPEFCFKIFGGPVEMK
RWAIYNVIILEKKSCQRI

>E1FMI3_LOALO
MHPTPLYRITDEQKESVYEPAEDTFLLLDALEEDKEALEQLEPNVVVEIGSGSGIVSVFCQQLLRVPVFT
LTTDMNFKALQCTRTTAQLNNVSVEAVQCDLLSALNHRLCGLVDVLLFNPPYVPTEQEATSDSVRCWAGG
PTGRGAIDRLFAQLPEILAPGGFFFVVALHSNDIARMLAFNQSIFSSKILLERRCGIEHLFVLKFTKSKY

>E1FMI5_LOALO
MIPISEDWSEWSKCSDSCGGCGTQNRTLKLPNGTRIVHLRHCNPEPCLDRKEPCCEPFKFINGKCLIPQK
TEGNEVRSKDQKKEAVVAWESIKDDDKPKFNNLEEQRKEAVLAWEIIKGTEFSDNKKFGNINLKETDILL
NLTTGISIGLNDTRNSDNDDFTESSGELDEEMDSKINTTETFNLGITNADAPHASMKKNKSVKKGYNRRN
YKQRQRRNRQHLPRMASKSQNAKKSGKSQKMFDAYYIYDYYDFYYYY

>E1FMI9_LOALO
MGSRRCPFFLLMRLILNMMVSVTSSGIQSIARVRFDPPYIHDLMVGTNKTVKVAIDVDTNRQEFAKLPPE
KPFAVILKSADEDIATAAKERKQFKWGEYSSKRNDCQTYEVNITLTGHFLGKTAVKVRLLSTDETTDDDR
FLYEIDKNRNIFDQSNTLDIWVIQDNKRLENRLFLSALVILIIIANVLMGCELDGRVMLEVIKEPVAPMI
GFCTQFIAMPLLAWGIANVMFTANGLHSFALGLFVTGCVPGGGASNYWTVLLDGNLPVSLTMTFCSTLAS
LVMIPLWMWLLGFHFLDSFHPEAVIKIPYVKIISSLVIMIVPLIFGITISYFKPALRFQARKVMRPFIIF
VLVFLISFGTVANIYMIHLLTWTTVIGSLLLPWCGFAIGCLTAVILRQSPPNVTAIAIETGIQNTGIAIM
LLKFSFPDPDADISALIPVICASMTPVPLLFIAAIHWLCRKLEKRQEIAECSDFEETAVKIDPANDNCNG
ITAVKYKSSPPLMNINGKGYILREISRC

>E1FMJ0_LOALO

MQEILCRTIRSKARIELISNEIESKLVSKIKNPCFALYDESIEVPNCNADNIIKEELLILRGWKQRQKVS
TL

>E1FMJ3_LOALO
MSRLKKHERSRQLSSRSSDSGSYGRRTRSRSRDRRSSRSRSRGRRSSRSRSRGRRSSRSRSRGRRSSRSI
SRGRRYSRSKSQDRRYSRSRSGDRRSNRSGSHGRHGSSGGRRRSRGSGRRRSRERSQSYLWRSRDRSKSR
SSEKIGREKHDKRSTRHGHSKQRGRSSQEKMKDTMKEIELMKSRMRSALESAQAKMDDVVEARVTETVAR
HRDIERIEEEGFHQSTFISSSGGAGGRLRKEDATDRIAMVTKKEDEHDKAMFGPKWRDAEKGYSDEKRET
VTDVEVDKETPLANARFSEDPAVREERWLKIYRERRAKLLGL

>E1FMJ5_LOALO
MSGGVYGGDEVGALVFDPGSHTFRVGFAGEEFPKGDIPSQVGVREVLDEMDDTVDQNAQKAKMKEYFIGR
TFVNVPRPRTEIKSYMKDCMIDDWDIFEQLVDYSYSRVLFSESQYQPVLFSEAAWNTKANREKLTELMFE
KYNVPTFYVVKNAVLSCYANGRTAGLILDSGATQTSAVPVFDGYCITHAVVKQPVGGDMIAEQCRLMLEE
QKIDLVPSYKIASKEVVNEMEPPVWTEKKNLPEVTKSYETYMEKQILEDLAASVLQCCDTPIDVEFAEKL
PSSPFCFPNGYSKEFQAERIKIPEGLFDTTYLKSVNTSSTMSVSQIAATACGMCDIDIRPTMYSGLMVTG
GNSLLMGFTERLNHDLAHKCPPTIKLRVYAAPTPMERRFGSWIGGSILASLGAFQQMWISRAEYDDEGKS
IVSKKCA

>E1FMK6_LOALO
MKTEQEATDRTERCFNRLQTALRRDGSIPEIGLPLQAIEGFLTRLASLDSNNRFNTVCIGAGEREGRVLS
PLVQRLHYGLAHGIGRSGNLTERQPKAIGSSILSEITNKLALDAIRCLGIPSTKAAMVVPVATGMALSLC
LGAWRHTKRRAKFVVFLRIDQKSCFKSIFTAGFEPIIIDGIQETMISDGLITDLTALKVVLEKQKDEIIA
VLSATSCFAPRQPDDLIAIGELCTKYGVKHLVNNAYGLQSPECRNRIEEAGSAGYIDAFIQSTDKNFLVP
VGGSIVATFDEKSLNIIANFYPGRASIVPSRDLLITLLQLGKVGLRDLYNMQQTNFEILRTAMREYAETI
GQKVMEVKMNRISLAVTLKGWTNQEQNAFGAQLFSRGVTGARTVPNGLSKTIDGYEFLNFGSHSSRQHDG
YLNVACAIGMTTREIEELVKVLKEETPKWAFSPSNNAHAKDSNSALRSSANCITALSTDDMQADESYA

>E1FMK9_LOALO
MESGLIKDNQLSASSSHDKDTTGPQNSRIRTERGSGAWCPRKQINSEVVEWLQIDFDMDMVITAVETQGR
FDSGRGLEYAPGYMLEYWRESLGTWARYKDGRQNEVLAGNSDTQSAVFQELDGGIVARNLRVIPVSDVTR
TVCMRVELYGCSYKDQILSYTIPEGDIIDGLNLKDISYDGITNSSGYLINGLGKLYDGAIGVDNFEKYPE
QWIGWSKEKHGATITIEILFVKKKIINAILFHASNFLKSGAQVFKRAHIWFSSQGDGQYSPRTLYFNYVP
DKNFQSARWVRIPVPSRIAKELRVELTLSENSNWLLLSEIKFEFKNGMLESDDLDDEEFDLDHLSNRGDT
LTYFAINDMSEDSARWISFAVITSLLFLFCALIVLFYLLWIYRRTFPRKGPFVVLKKNSKDLRMTIGGRT
AKRTSPNAYRMTNDNMQNSLLEKLHANQSSGGEYAEPYYVRYTSSFCNLPHQLALTQSNMLRYDLSDM

>E1FML0_LOALO
MPYRRSQSRTRRGRSRSRTSRRNVASLSLLSMRRRRGLRLRSRSQVGFDLGRLLSSRRRRPTYRSRSRRS
SYRSRSIRSLRRHRPSNIRRRRHSSSRSRRRQFRSRRRSRSRRLSRSRRLSRSRRRSRSRSGSVDDYDET
RVVSLKR

>E1FML2_LOALO
MRYCAPEVGLSYLWILVISCSASIHYPEPTHVKHPASPTIMSSSIVQLCSVADQPFLGATSTPLIHLSSR

VIQRSMKRTAFVIFITLTMRRVEHNFSSNTAPFSIAQMKASDKIARNAVRNSSNRDRAWSC

>E1FML9_LOALO
MLTSSYICANKERYTNVRSIKILILNKAERIKGWFSPIKLKGIKIVRGLTEFGIREALRRNEFFGGSMLE
VTTVSSNRVSDGDVEESGIAKFKRDAGKSHSWNTLFLGANAVAERLAEKLDLLLGHRVLVHV

>E1FMM1_LOALO
MLTRIHLLACKSIVASNSAHYTTIGNKRIGYNPDYDKDVEGIQKVKTDKSVGIDGIPSEVWKHGGHALQA
NFHELVLLDRTLESEYSILSFLSLEGLLCRSMCKI

>E1FMM4_LOALO
MEVNPIVTMLQFLHDISSSRWRSLRAMLSVACASSSWMSSNKKWTNSVHAVSHLKNPLRKLEDKLAFVVF
ATSMSSTLIKSSFAKSNHDDDDDDDDDDDDDDDY

>E1FMM7_LOALO
MPTYTCVCTQTHICTYAQTHENVHERAQLPQYKHSRMSAYYMQSLNRDM

>E1FMM9_LOALO
MDIYYLFEADASTILISYQMHIFLLWFIFFLITKKKTLGLTGCCVLTFRDADSNDDSHITTAIMIMIMDG
WMVIIVKQEGREISETELENKLKRILVEYYLKAQSREREIMSGEIVFCRFTQIYERFDFKCSECSLPSKM
AYQNLEIAYELLPYGWKKNLYMEIYLSL

>E1FMN0_LOALO
MKLVNFLIGLANFAEISPLRKAEISQLPVAIKADNAKRNALPPGLRFDHKLEMVLPEGINESESTKDPRS
KRTKMYSGYCLRPFPAARGNYAVDYSRKTNIKLRKEISQGDQEDAFLLHIVEGYSSTTLPTKRAGNGTQS
SSVHEYDAPQLIVAENEKMFVEEMIGDQVVVKEKSLVDTVYALRDQLNNVQKELLQLNKTVDREQKSRRR
LEEMVRRLCQQLQQQQQSQQQQQGPKFSANLTSKAPLSARD

>E1FMN1_LOALO
MDEGAKISIDSSKYPLARKDNSILDDFHGTKVADPYRWLEDPDLNETKEYIMQLNSISQPFIASSPYREK
IKKRLTKLWDYEKFGCTSRRGDYYYYYHNSGLQNQSVLYRQKTLLDESEVFLDPNKFSEDGTTSIRNQCF
SHDGSILVYGISEKGSDWMTLKFKKADGTDLEDTVVGVKHSDLDWLIDNSGVFYSKYPEHKSALKGSSTE
KHEYHSLYFHKLGTPQSDDILVADFRSHPEYMCSGTVTEDGHYLIVDVSRGCDPYNMLYYYDVQEAQQKI
TGKVPLKPLFDKLDAKYELIDSDGDKALILTNHDAPMFKLIRVKISTANEGPSAWETIIPEDKNNKLEWV
VNVGGDRLVVSYIEDVKSKLYVHCPKTGQRFYEIPLEIGTISGFFGRKNHTEMFLLFESFLTPGVVYYVD
FKGIEPSHQVKLKEIRRTAIKDVDTSKFAIKQVFCKSKDGTKVPLYIVHNKNLVLHGNTPVMLYGYGGFN
VAVMPHFAVSRILFLDNFKGVFALANLRGGSEYGEKWHEGGMLKRKQNVFDDFIAAAEYLVNYNYTLPRK
LAIRGGSNGGLLVAAVSQQRPELFGAVVNQVGVLDMLRYHKFTIGNAWIPEYGNPEEASDFEFIYKYSPL
HNIKIPNKGYQWPSTLLMTADHDDRVVPSHSLKYMARLYEAAHSAGSFQKNPLIIRVDVKAGHGAGKPTS
KVISEIVDMYCFLEKVLDLKWYESE

>E1FMN4_LOALO
MSMYCPLAVHLDYDPPIPSATTPVTTISNCVLSHNGFPDRSDLIQTVCQLYDVVQGERVHSRKIALIQHG
SPLHPYSVCNSNYGGHELCLPLIVDYVSPDLAPPTMAHHFYYLHLRDAKIPNTLKNDIYRQLYHTAPFDL

FTKYTELRSRFDLVPLKECRPLMLTILDFWIQRCKRLKMCLDATEFFPVRLKWTDSVFEGTRLKRDQLSQ
NWEIGCEVDEYGWAVEMVRAEHVTAFTENCPLMFMGDSTSTRLARYIEWGKRFSDIDGRFLQQFGIDNRK
LEESERMSTLFLLLQKIHFGNGCIAVVLSSGAHELAEGMAPSKYGKILKDILVYLLQFQLRILVVPPTPC
VENQFLWFQYMQQQVELSVKLPTVEFLIRPIHQTSDRCFLDALIIGSRTLDSFMCGDAGFTDFGIKKLGF
FLIEVMQIPKTNHNGEILKREMIIATTSRQDDAIFAYDCNKESSLGLVETQIKIWNEPIIDYKSIDNVPS
FPSMACTNLLSTDVAYLNVKSYSELAGYAGPSARNSLTSLATNIQNSRHRKKKTKIMGGSHNGYLWGQKQ
RAIISSSKKLGQFPKLTHGNYFTLAQERQDRRRRARTTEQLNK

>E1FMN7_LOALO
MGAGHRAGPLKQSNKRHKSGRHRTKGALDAVAGGKVGCKAVTGKHRFLIGKKARRLQAQQLRENKHAALK
LSEGYAASNGYLKQPPIFITVLSFDPNYTADELIALLCSCDETSYRNTSYNGCLNHLCIKRLKSRYVFTA
PNYVNTDVILDYVKISDVIAFIWPANGEITVKDDILMSTLLAHGLPVTMHFVPGLCASATTKQKEIIRKN
VTKLIANWSFGNEKLMHCDSENDGLLALRLISTMKKKLPCMQRMRSHLIAENVEKIEAEGDLCTLKVTGY
LKGRSLDVNNLVHVPWLGDFQMTQIVMEQDPFAPDKKVRIGIKTSGTTFKPDVNLQTSLQSEIIPDPMDA
EQTWPTEDDIVKEAFKVKKIVKNVPVGTSAYQAEWIINDEDEISDEEIEDELADNNVMKEAVNSEESDVE
NEMEVEKTSVISDTESFMEIKDEEINMAEVEKYREARENEQFPDEIDTPMDVPARIRFQRYRALKSFRTS
PWDPLENLPKTYSRIFKFSNYRHSKKVALSNTATENEYSAPRGAFISIYISKVPTNLIDIWPRSKPLIIY
GLLAHEQKMSVLNMVLKRHPSCTVPITNKQRLLFYMGYRHFEAEPVFSQHTSGDKFKMERFMPNDGAFVA
SVFAPIMFPPVPVLVYRLDSRGNQQLVATGGVLNVSPDRIILKRIVLSGHPFKINRRSVVVRYMFFNRED
IEWFKPVELRTPRGRRGHIKEALGTHGHMKCVFDQQLNAMDTVMMNLYKRVFPKWTYRPVVLHPSSGSPN
ESELMEI

>E1FMP1_LOALO
MSGRKEKVAVPRNPVIVARHLKEAKNIIFENGRMSNDDFTNEFHSLPCKIEYDGPAKVSQYFVTEELEDN
QKVATFRGRILNGVQQQFPDAYRLYIAVEKEDRVSELVSFSSRRKERSAAENRLKVDSGIQEFADNSRVF
EVSGSAASFTRWEYDRSSSYQSPLVRAIGYLNIAETFAKDDD

>E1FMP4_LOALO
MERSVRNVKRMLRENVTRFLEHADGREVTITVTARLVKKHLSSLVILGLGSLVSLMAWYFLVLIH

>E1FMP5_LOALO
MVCPKIETCLEQCFLEDVLHFNSCAGKRCNFYCYDDDCSYCTYVAKRIFLRICRENNIPNLPNVKFHGNE
MWNEAQFSFKNGKISFEMEIGFQILLFKMN

>E1FMP6_LOALO
MNDDDDDDDGNDDDDDNNNNNNNNLQMFEERALLKDYMDKSLAIILISQIIVSVSDKVRRYAKTGIFYHT
MGRRTKTKAAMHTMSAVGMFSTMVDQFSFVCIMTKCRDACTACEQCNYALDQLSKITSGIKTSNALVDI

>E1FMP7_LOALO
MNTHWNKFVNYRVTNSQSGALIDDHPCENPQNLTCNGLCYIFQISGINTVDGTKQPFALAQGCSVGLFDG
EVRCFERSITLRGRGGNFQLDGHYCLCGGNLCNRNSKIWIQHDPKKFYAQGQKAGS

>E1FMQ0_LOALO
MFFVLEADSAHILEGSSSVISNVFLCLPIRGHSGPLLAVYMAKAECSCVLSSPGLTLVTLPCARTVTLVT

LVKKSDQWVFRIARRQRWWKRSRSRIGGGRRPMIQIFPKTLYVFADKSSILVSEDMILPRPWTVQRRM

>E1FMQ1_LOALO
MLQTSCRNLLTLPPFAVSVTRLESGIPSVNDMKKSENDYHPSKYTILGNKRRDPMHYTDRKVGRGLDIRL
RNAQRIFVQKLLITTYHIPRSLFWMHLHGNEKLKIELHGEGRISQRNDPRDIIC

>E1FMQ6_LOALO
MIGRSGLFADIMCPYDEDCIRPHCHFWHSKDDISQQEQQSSVSLMSATLPEQPFVGYVGYMENQKTTIET
TMPLCSQSIYAPLDPVVYTQRPSTSAVQQSSVTYVPTKITARPMCKRMPSPEIPKPRMPVELSVDDILDP
ANDKPKTSVAPSINNLSLIENTKVSEMSLNDYAKSVADIDTRIEELQRKIEIERQQKEKIVHDIRALVKP
PAAVNSDSLTPKKHSGLNSKSKLTYTGGYTPTPIALLKSVQKAKNEARQKKKSKTEESDNEKEDVVNLKN
KVKGGKMKDKNKNVKVKEVTNVEASKLRVALKQQQSTEGKTQSVAKKKLDEQLSIEDLFETGVPATKKQR
NIDNDVKSKAGNLPEKMEKQKEIEIKMEVEKRITHSSVQPTIQMCVTATKVRAPTMAQQLMNRYQKLQKE
KEELLKNMKLKEEVKAKKITTAEEKYDYKLGAVAATLGKGEKRMAHAPKISKSTKVNLVPLDPYSCGKVP
YALRARYLSLFYIECQKFCGSMSDAVVQAQQEEKTIKDRAVTKGGYTSAAVNVLRRLRDVNSPQVAALKS
VSHSAILAGRHHGSITVGQRKYSAPRKQSVSESEFYGLLLSKFVLDEQKLEQNGFPLWEGPDKKRVKIKT
SELDCKKKLFVEESDLKRICCRCGAEFSVTPKGEYAALKQCVYHWGKAFKTKSPPVFF

>E1FMQ8_LOALO
MFPNNPWTKHPNIAKIIHYTVPSISAESVVVDDNTTDASAPHVPRADRDRLFANRSLLVLVWFMAEFLGA
SSGITVGWIVGSFDFGVDEIIVGVSERADCPM

>E1FMR0_LOALO
MNLRRPEFPKIEMLCKCCIIIFIISSKEFEKKAAGVSKQTLPRIFYLDLRHSLGQLTANYWYGFNYIWNK
CQPLMPAKYGQSDNIIMHFVLHCNQSEYEQMDISKLYGYMIHSNLCLLQLTHDAFE

>E1FMR2_LOALO
MTTVLSCRTMWKMCSSDHSLKPIKTFTHQWIAFTESDDLATNRAGRNELIARYIKIRCGKTRTRKQVSSH
IQVLARKKARETQAKLKSAEASPPSAVSATVPGTSAEKEKPMSAALNNLSMLAMSKQNVLPRALYPTVWP
AGFPAPTAAQTAFSAQDDLKTYQRIVEINGYNNGQVSLNGQSPAEKLSPVTSLDDRAIASSMLTLCGFTA
YVEPIPNNVASRVDLVRIPKLADEPLEVSCCNAGGIVRFRFFRSSNYILINADFPVYNPSLTHYQTFKLE
DIAEKYPPVLGELFAAGPRDGFFLVKCWANIEFTLPDDNSALYAVDSFYESTQRFDITVSTKVCSFGKQV
IEKVEIYSPIENGRKFNFRLEGSPMCEYMVKFVAELKKLQSHDLMNSVLYNFTVLQVVTNRDTKEPLMVI
GFVFEVSPVPESTCRLYRLVAG

>E1FMR8_LOALO
MAVLAAQQYYIYHEDDAINIDKLENSLMMYLPEDDILDTNENNHERWLHLVLHAFRKKFQQNHPSAEEVK
ADVVTFAKQKWPLLFSRYFEAFSYASSSLPDSQLLIAVNWQGIYVIDGQDDVILQLTYPQISRIVSTTND
RDGIEMLTIEMIGGEEYCFESLSTKDIKQIVEFFLNGLKNRSRYLLALQDYFTGEGKEEWNTCLNFKKGD
LLFLNDEYCGASLRNNQFVKGENMRTGNQGMIPVNLVHVLPTICKPSVEAMDMLSRSQELGCPINRQMSL
MTHHLVVNREHTLERFANDNFRAAPRPPQGLSESFYEHYAPYSLWSYNREPIKMPLLKKLQGKREPSQAA
IKGYLAILKYMGDYPCRRIFTITELTDEEIIRDELYCQLMKQLTRNYNPISMEHGWELMWLCTGIFPPSQ
SLLNEVELFFRTRQHPLALHCLVRLRRIQRIGRRKFPPHDVEVEAVRQRTVQIFHKVFFPDNSDEIIEVD
STTKARDFCHRIATHLGLNSVDGFALFVKVNSKGNEMLVISIPDNEFFFDFLRQLFEWMHPKNGTVFNFS

YQLFFMKKLWINTVPGEDRIADLIFHYPQELPKYLRGYHAVTRKQVVELAAILLRARTRDDKAAPLSQLA
QIIADIVPRDMIKIYSGTEWKKFITNAYVGRYENIPSNEAKIHFLKTIAEWPTFSSTFFEVKQSSDLAIP
DKLLVAINKTGVHLYNPSTKQRIVHYPFTILSNWNSGNTYFHMTIGNLIKGGRGTRLLLETSLGYKMDDL
LTSYIRLFLSTLNRPSTETTQSNTIDYCNK

>E1FMS2_LOAL0
MKTVFVVTTVGSSCAIILSLIVVVLIFHDISELYTNAVIEMEEFRTTAEDTWREITYASVNPYGAIKTAP
FTLSSIFRAKRQNNQCNCGLSPKKCPEGPPGPRGKIGEKGADGLPGEDGMPGPPGVTLLATYNIPGGCIE
CPSGEQGPPGSEGPTGPPGPNGPPGAPGKNGKPGQKGPTGRKGNAGETGPDGPKGKPGRDGKDGKRGTGP
QGKKGPTGQRGPEGPKGDDGKDGEPGPHGEPGPDGKPGADGRDGRDGKPGISGIGGMPGKDALYCPCPPR
PQKTPAPPPNHYEQQKSDEPQGGYKQKKKMQKKQ

>E1FMS5_LOAL0
MMQKRSEKMQAILHDIARWDRSNAAGVFQRNGGCSVLPTTGYPLTFHPDRPTPSPASVIETERSIQNSEM
KDSAYRMGGVRASDESTPRRDSAGKREIGLEQCLIPRMA

>E1FMS6_LOAL0
MGWAAWRSLFHCYYCRCCAAICRAEQYNDGRWEGTKSKDAILRGSDRENFHYKPLRASSQDSTNNQSSIT
TVSFDPKTETFPENSPFLFSTFRLLQPFVAVY

>E1FMS7_LOAL0
MSSFPFPSEVSAVPPEKSIQDDQHAKMSLLSSLDLLVPFAQQKNSNTERTTASANAHIYVYQCPKEIPMV
VRIQNTQNSAQFLKMCQRNYVGNKEFQRQRLIPKTNRESLKKTLHSSSVKKTSYTTLQIPTLQ

>E1FMS9_LOAL0
MDVAMDERLRTYVQEVESRVRCQLDDRKKLEDAVPLSEEQLRKMDSTLKRTTAFMKKLKNIGCIQLTSVL
QDVDKVNLSKFVEEMTATIAEAKIKYSEVQAVVKVCVHLSCYYAEFSSLLLLEFRKLLPSRRNDKIQNPS
KLRVDIRLLGELCLHGVFGKEGVQLLGSVVSFLTLTDRTEHINIPIVLPFCKAMNVDLLGLHPYSIKQVA
SAANIKLPETSVISVDHKKTFAQLLLDYRSSLVDHIKQDLLEMNQLLLSIKRQTRTRGDASAEDRNHLGE
LRARYEKILTSGTQLSEYLGVEMEAMKEEQSEDEEEEVSALALSRALQEGTITIWPDEDTRQFYETRMEL
RQLVPAILFQESEQRTLDLVEGKIEDVDLSGLDAIAEEAEDEGDEHSEGIDDEETKIDLNEMDLPSVAMS
RIDAPKISGQDLKLRMIAFMEQLPWLINRDLIDKAALDFVTNLNTRNNRKKLCQMMLEQHRNRLDLLPFY
GRLVATLEPVMPDLALELSHSLLQQFRAAVQSKKKQRVDWKIRCARFISELVKFEIIPKGEGLSCLRMVL
FDFRGHNIDMCCAMVDAMGQFLYRSTDSHGKMKILLGVMMKKRDRVSDPRQQMLIDNAFYTCIPPPVQEK
PKPASDPFQDFIRYLISNLSRYRMEITVRCLRKLDWSDPVCANYAVECLSNPSLPRYNNVPYLASLVASL
SSCHDWIGIRVLDATLEDIRIALEFNSPSLNQSTVLSVIFLGQLYNYSVCDSPVIFKTLYQLITFGAFDP
LLDDWSNLARIGLVCELLLVCGEYFNAGSAKKKLDCFLAYFYRYLLAKEEAFKARDIVFPKHVRFRVEEM
SDYVRKGVKIPESFDEAQEVVDGIQQQYGKMVDAAREGKIAQQIEEEIMDEEEEEPRKINCAAEAGGDEN
ISSSDEIVVSIYDENESVRVHTNRVLLPEDELFSRANVVSVPVSEIVVPTSARQKFHRSIAFDANARSTK
SDDTSNSGTETRMAILTRGKGNKTVLKAIAMDAPSFLTETWNAQREKERMELTVHKHITLSINERMMMDD
DESLSE

>E1FMT0_LOAL0
MVAPTAYTSMLPSFMHHHYQQKRLNSSGSNSKHIPLSDNTIAIFVLLQIVSTCSALTCMTCATSSSTHQN

AIQLDKFRIIAQLPYPGCPMEPIRCDRDQDVCVTITMNIRGDGLYWIGAGCDRQEYFQHIACENVRTLTR
NVQLGMAQELRVLQRVCLASQYQRFGGHHPVQ

>E1FMT2_LOALO
MSLRISAEYSADTSKEMDGALKEEDWCYKYRSAFTYAPYNDLDKMGYEAPKRYASQVGSRYSRYSYSFSG
TEFMEEAPLTVHTTAVESIFVVLAFLLFLMTLPFSLIFSLKFVGDFERLVVLRLGRAQKTRGPGATVVLP
CIDTYTKVDLRVNAFNIPPMQIITFDRGLVELGATVFSQVKDALAAVCAVQERNRSTRVLSIATLHRLVC
KQRVSDVTSVVGRRQLCENLQVELDVLTTAWGVEITKVELSEVKVIKEGENMALATFNKVLKSELGSRII
ETIKGAAQEFVVQQQQKRQSVHQQQIGNHTERDRADLSGELRKKNELRCTLNNSLKFLDIDHLLCSLTMV
IDNQLVASVGYIFQVNCTEFGNFYVDLKNGSGNCCKGISPTADVTLCLNRELLFKILKKEVTPMQAYLDG
SLRILGSVKAAVRLTLLSDRFSCLL

>E1FMT5_LOALO
MSRGNLGDSCSTDDQCSSGLICKNRTESKDGLCLFGTTLISHLAKKNKTDKSETYPDDYVIKTGATVETK
LESILTQKDEADITNKTTSEFTSNFLTENIKIGKTKITAPPLSVKETNTSREMTEGSVSLSAVKNNTVGK
MTTKVSSFPATEITTETFPSFPAIEITAEKIPTFPLSSVTQITVEDTLSTKAPSSPATEITMRIHYRRKF
LHLQRQKSLLRIHYRRKFLHLP

>E1FMT7_LOALO
MNTMKALICLLNGKGCRRITKEEDQFYDDTSSKKAGISRKDGKALEHIPLQPGNTSHNSEKEHKYNKFLA
WTKMDREINERYAMIFGAPILPIECNSSLPGHSETKSVQYTIKTDTHEFCNHLGTDQRDKITVESLWHVP
KHI

>E1FMT8_LOALO
MKKRKKRMNAYCIWVLVVAVLVIFYLSVFTEVSYHLHIHKRNTNLFEQFVALTNGERNRSDNEHRSQHCQ
VLHLALVVCGAVQLSYPLSTLIKSILRYRQQAIVLHLLVDDITMSIISLLFSTWRLPAVKVTFYNASQYL
DRFSWIPNRHYSGRYGLLKLILNDILPADVDKVIALDTDVLIMGDIAQLWSFFSKMANLQAIGLVENLSD
WYLFNRSTPQRTVWPAWGRGFNSGVMLLDLAKLRNMSWSHIWEESASKNVKEYGPVELADQDVINAVIND
HRWIVQKLPCEWNFQLGFQSQQNLCAVEISHLKLVHWNSPLKTRIVNRYAVLLRRYYDSVRDIDGSMFRS
NTIHCRYGSQREHSTLDKVSEYDDNDDGCSEIRHARWITYRTLLYVRPYNFTAAPKNVVLITQFSVDRLM
HFNALLQYWTGPVSAAVYVTDSELSLLIQFFDDTLVNRTNVALHAVYKEGTYYPINYLRNVALNNSNDAS
FVFLADVDFTPAPGLYTMLYKKLVKTDSTNKRAFVVPAFEYTGNRVPAVPLTKNELLRELDARRMQIFRR
SVWIQGHAATDYDRWRHADQEYSVSWKADYEPYVVVRRSGLPPYDQRFVGFGWNKVSHVMLLNAAGYEFT
VLPDAFVVHQPHHPSFEMVRYRSLATYRKCLKALKGEFVRDLIREQRKNWISNFSGDPLNDYSVLTIA

>E1FMU1_LOALO
MIRSLSIILVWILAVEAGRITLYSGLAVEDRGNGTEEEKWNNEIDNGDNDDDDENFTNENSQDSDIYVRN
KFMPQILFAQKDKIIEWKDSDYEKGHLINDEFQNDNNTITEIIINNVKGIGSNGENVSKRGKDEIVRTFP
HM

>E1FMU4_LOALO
MTDNPKKLFLKFDSVKVLFYAIFENFTNVQREELKKTVAYDVTKVGVLIQRHRQKNPLLVRNDPHLRDTV
MESQRVFNRTNTLKIPTIPTLKIRDDRSQVTRQVSFLSAILNQ

>E1FMU5_LOALO
MDILNHHHYYKSSKCAHHIDQPPCQALAAQMSLAPRIMLHFSVEVEAVRSWEYVFVTGSAPALGGWMPSD
AFPLLPSQDSARQRWEGVVEVGSDPVKFRYFIGYYLHSGKEEEKNASLFYQLLNRSRVCAICMLPTNLAG
VNNSGISTVSAGKSLVSEASLLGKNQNEILLRFHGNPFKFYKKRYAERSYCIKVTPFDLRHREAGSVDDD
DNEEGQDVVQPVVPSYSVAHIAILSSDDPHYREQSPSGDRFKVGSDYFIFRTRSVAVEFLGFRIELFAYD
EEHGPSTERFAIAYALPSSFSGTFGVAGEPLLVKNRPVGQINVEYLFIRPLLTSCSVQKMNISYAKHWKK
RQPLEVGHRGMGNSYTKMNAGRENTIHSLNTAAKRGADYVEFDVQLSKDKIAVIFHDFHVLVTVAKRRTP
RLEAQPAEAANDYHEIAVKDLKLKQLQLLRLEHYKANQTQTRQNYTKLSAEADEQDERLPFPTLLDALHQ
VDSSVGFNIEIKYPMMQKNGLHECENYFERNDYIDIVLSDVLSNAGDRRIVFSSFDPDCCALLAAKQHLY
PVLFLCVGVTTRYEPFVDLRSSTSNAALNFAACIDILGVNFHTEDLLRDPSPIQRARKFGLISFVWGDDL
DNKENIDYFKKVLRVDGLIYDRIGEIEARRNVFLVEREAKASLFRQSISPIASRTVSLDDGPPSPAVSEN
SHSSGSTVSSNNSFYLHRPQTSLSSRKVSVTLEDLQVNKSGNVIRDESLFTSNNCLHSTPYS

>E1FMU8_LOALO
MTLKTKVRETGSENFTPTLKNHGMANKKRVPKGIISSSENSVGRKVFMAHLFPCMKKFFPIFDIKYLITK
REEMKTNGTSNGKKCDIYRFKTTAITNLHHALGKLACDVSQLSSADVEVIVNIQFKQNTTYVRFSKYHHN
EFGKEKVTMSKQ

>E1FMU9_LOALO
MAKKCAPRMRHNYEVTPGVMRFSAARMYAKRGAYAKKPYPAVEKKIERKTKFIVKPIGGDKNGKERKVFI
KKRPKYVKEGRTTRRAKRSPKKTVLRRSITPGTILIIILAGRHKGKRVIFLKQLEKSGLLLVTGPMKLNST
PLRRIAQAFVIATKTKIDISRLKLPEHINDTYFRRFSSKKHLKKAMPIYLHKGQLKLESCGNLEYTVSDQ
RKADQKAVDSVVLEVIREHPEKKYLFGYLGSRFALGKNQFPHQMLF

>E1FMV1_LOALO
MVVRASDVDKNFFTPRDYQVELLDKACKRNIIVPLGTGSGKTFIAVLLIKEYTSKLVIPWKSGGKRAFFL
VDKVSLVGQQAAHIEHHTTLNVGKMHGHLNQDIWSEPAKFDAFIALHEVTVLTAQIFLDLLDHGFFNMSN
AAVIIFDECHHVLGSKHPYRLIMHRYGQLTEVDRPRILGLTASLISSKIPPNSLEQLLEKLERIMYSSIE
TASDLLSISKYGAKPKEYVIMCHDFFCCSCEISKKVVDTLENLRTFCLKCTEFHPEFDVDPRKPVLEAVN
RTKSVLEQLGPWCAWKVCQLFQRQLKKHSGQGFLPEKQVIFLQMAYTTMRFTKRLLDAKVASVRCFNDMK
PILPDRLSRLFEILKFYSPSNMEKVDPDFTFCGIVFVEQRYVAYVLNTLIRAISRWDSDKFGYLVSDFII
GYNSANISAEETMALHKRQELVLRKFRQRHLNLLIATSVLEEGVDVRQCNVVIRFDRPTDYRAYVQSKGR
ARKDGASYFLLVEERDREQCSRDLKDFLQIERMLLKRYQNVHNPPEPTISPSIEAMDDIIAPYTVESTGA
QVTLTSAIGLVNRYCAKLPSDIFTRLVPQNTIVPETVGDHVMYRAELLLPINSPIKETIKLKKPLGSKKL
AQMAVALEACRRLHKRKELNDYLLPVGKDTIMLTALDEDPDEFIPNMNYKVGSARRRQLYDKRMAKALHN
AIPHVDEECYIYVMEMDLVKAVTGAANPKNRRIINPLDTEFCFGFLSNKKIPKVPSFPLFLRQGRMQANI
FPVKSRLLVDAQMLELLKAFHHYLFDNVLRLVKGGLVFVPDKAPVNVLVVPLRRERNNKTNEVDFKLDYA
YVRNVVSSIDELPRIPTEVERRAFKFDAANFQDAVVMPWYRDRDHPSFYYVAEIIDAKPSSKFPDDKFAT
FNDYFIQKYGIVIYDQEQPLLDVDYTSSRLNLLMPRHWSRSRNRTTEERGSESGGMSQGQILVPELVDVH
PIAASLWNVIAALPTLLYRINSLLLADELRELIMREAFSNPDYRTSDDVYWMPLDYPTPMDDLEMKPVQR
ICDLKKQVEEKSLENNDAKDSGSGMADFEIGVWDPELAKGLEGFCPNRGRRDEIRGFEDIDGDALGLMVN
GSTLRQHGDMSDDDDEDAIVLFDFINSVHERLGKSGDIFAPRENITSSGWDDLIVIEESAPNGINMPLSV
NSGDSQIDSRGLMADLSRMSWLLNMPTALPLAAVDTTNQGSVDVKKEIMKNGKKQHHPSIARKPAQLYLD
SLERLEDSDRGSLKSNRQEECIDLIEFCDEIDEIVSSEYDSTLPFDLRYFKGCLLDTDVELNTVEMLSPT

KISLHQDGKLMDEEMVRSSSDLELFLQMSHNSDVAKLACSTANAYEVNRKQEISAPVLDWMTFSFEEDTF
NDHPDGVSPCTLLQALTLSNASDGINLERLETVGDSFLKYAVTDYLFHTNPEQHEGKLSFARSKEVSNCN
LYRLGRKHNLPSLIIGSKFDPNDGWLPPCYAPTSDFKAPNTLDAEERDKFIENVLEGKTVERQEPVRVPT
GWDEADRNSQVRRIADGIETIEFPKNMITSWDGEEITPLPYNLLTQQSLGDKSIADAVESLIGAHLLELG
PTATLRFMKWLGLKVLTEPVQVEPPLLRFIDTTEQPDKSLRKLNDLWVQFQFSKLEDFIGYRFNDRAYLL
QAFTHASYYKNRITGCYQRLEFLGDAVLDYVITRFLFQHSSHYSPGVLTDLRSALVNNTIFASLAIKHNF
HKHFIAMCPRLHHMIEKFVCLCTEKNLSSANFNEEMYMVTTEEEIDEGEEEDIEVPKAMGDIFESVAGAI
YLDSGRSLDIVWRVFYNLMKETIDECCSNPPRSPIRELLEMEPERARFSKLERILETGKVRVTVDIQGKC
RFTGMGRSYRIAKCTAAKRALRYLRSLKKEKERAAGKQ

>E1FMV2_LOALO
MNCARGESSYGDGSNHCGLKDDEDGNLVKFTCFLCGLTENCRYGLVEVSGRTHNYRYKDEMYYMLDPFRN
RNTVNERRLTRTKANTCSKTSGSKTASILDILVLGAICSICGQPVCIGEDCGVFYAKAFCIVCINREKAH
FPKNFIEQIDATRKQRKSERKEVTVFV

>E1FMV6_LOALO
MASCGTYDKIIRHEWNVLFTNNKSELCFCGIISEYFLHKDKYTVPAVLTSSTDFLGNEIRERERERGGRGE
CSVFGCLDCLLACQGHGVIRAITKYRSITDDIDTFKEVGDERVQSGNLNQQGKSGIVAADLTTVYKQNMQ
FAASVDSLDSGPSCSEPIAISSSAPTVPVDSQKEPLSPVSERYRLKKFHLQQKYDYIYMSNNRLRNRLYH
IKKEINHLKRLKRVLCQRLFTFRDPFMDSCLEIPDNDPTSPTIERISSDATGKPSGSSKKKKAAEAKRVN
QVAEKIRAAMGSRKSQVPGCQRIPTWAFRYGHLWKTRRIRGMYTIRKVAVRCLQKPKEVGEVDLVTMEAD
TPRLAHMLSFGHNSKLIQVNEVSSMVEIAKNSVISIIDSVITESHEERVRANQNSSGQKTPQQSPNVPAL
GEVMRNEAGIEGVSEDCEPVIERMLRASSIDHLLPVDPDSDTNNVLTGFGENLEENFMAED

>E1FMV8_LOALO
MFSRPWSFRCVKFRTTQSLKASSTQKIKKKKEEKEGNREKSECRKTQVASWNLIKFDIHTVLCASTNGLM
SRKRSKSRKRQRQELRGLNFAPIKTVLRQL

>E1FMV9_LOALO
MLLSCQYHYYHETDIYIYVIICIFVLTFIFIFMVIFAFIFIFIFIFIFVFVFTLIFIFVFIFILIFIFIF
IFIYIYVYIYIYIHIYIYIHICIYIVYPQ

>E1FMW1_LOALO
MELGEMNLEVELEDEDQEYTGTPEPDMTLPLRDQHCSTQSRDMKNIVPARSAWYAFVSCWACWINVSFQL
NQKLKLNLQEQKYNKNKSAATVD

>E1FMW2_LOALO
MDGSEGVELSNKPEILVESRRDYIGADEESEKDSREMARNDEELDKCESPKQEVDAHILRHAIQKLYSDP
SFAVICSFFNKFAVFLGLKPQCFTKMENMFTSFHVTGKVDRDLIDLHLMLMRKLTFKSARLEVWEKYLLK
FCSLVPSLETEYLQLERYGYLHTTAATKLAILKALCESQFDFNVKFKESLLNSCAASDLRLLPIGYDKEG
LAYSYQQDADLVIRIYSAEQDDHSGGSWNLVAKCKEELENLVMELKNRLASKYLSNLLKQHEDSLALVQG
ETDEQTNVLSTFTTKRGALLDTYQDESAIKRKLVKPSQMRKKADVEEASKKENSAPPVEITEDIPDEVKP
FLDRRILPRRSARSAAINQLKELTAPLKPQCGKRKNQQNKKQVEVENAKQLKILDKNDDQGDSEDNDENE
KDDNDVDDFILAETSTSSDDDFMPLSELKKKNQNSRRRRGRKAIVEKDPFEMIESEEDEESDDSQEKKAK

KERKKATEETLCMKCSKSSNPEVLLLCDLCDEAWHTWCLHPILWYVPDDDWFCPNCQQAMLIEKFSKVLT
VLAEQVKRKAAEDKKKEAAAERLKREMEYIGASLNNIIHTVRDTKVESSESSEESGEDDGERKSKKKQSG
KFTYQRLGVHPQKPIIPIALSRSRRHVAKVDYKFGAYDELIQLENVCVDVAGSVRLKGGAGRGKDMSNII
NAENKQIPTGVEDSYQNDGLESHIRIQSKIGKPVKSRHRLNDLNVDEMTESDTDEYQGSDTASVSDPSDD
TDEYLPSDASCRRRTTKRNYSNKRTQSDDDFVVSGSESEYEPVRKKSHKSKNTRKIKGKRKREKWTTSDD
EDSTSEVSDNYQSEESSSSSVDRRAKRRTTTKWAPRASSSDAEDSGHSVQKTATGRPLRKAVAKRPSLTM
DDGEDEEEEDEEMEERLKVIRPVASGRKTESSDEFEPDDEEEEEEEEAEDEETADEKEMENKEKTTAVDD
EKQRQNDEEENVEGKRKKSVKVENGIEVMKEATSSATKALSVGCHKKQTEDTDTNDICPSTSNDVNVMAE
DRYRCQLTSETARKVKSEQMEQEVFYAASTKTEDLKEVEVKTAEKTTNIAVTSSNVTSFVPPSCFPSTSA
DLSSLNPSSLMQQMARLASSSATPVQQFDPSSAAYREMLQTAAPTFISNGAPLHIYSGPAPVTVYQVQQP
PLVRPPTGMVQPSIGPLSQGWPPYPPPNPFPTNAAAPPMVTATTIPSPTTDSETLGNVLASAMDY

>E1FMW4_LOALO
MLCAIEGISMISMRLKHFITTPIFYANGPPHIGHLYTALLADASNRWRLLRDGNADLDDSLFTTGTDEHG
LKIQQTATAAGCDPQSYCDNMSDKFKDLFCAFGIQPNDFIRTTETRHKEEELDKRGQIQQGKHEGWYSTT
DECFYASDEVEKLSEQNSMIKMNRYVHYIVEDKNLVSKITGSVVEWVQEENYVFPLSKYLGKVRSWLSSC
DIIRPKVYFPEAFQQASIERNLSMSRDRKRVTWGIAVPNDESQTIYVWFDALISYLTVSVIWPAFLIALD
LPLPKRIFVHSHWLVNGAKMSKSVGNVVDPFIASKSLSEEGLRYFLLRQGTPQNDANFTMLKAINVINTD
LINNVGNLLQRSTIEKLNPSQRYPAFYPDSFHNSLLELGEPLVHSVNSLAELYEEQFDELMIYKALELLM
EVMRQANGFFQFYEPWNELDDRKPAVPHYADHLLNRLGIKKNERGLGNVKLITDSKYFGRPLGEYSGPLM
ERIVCRASEV

>E1FMW5_LOALO
MSDDDRPRKRAKKALSHECTVILIDVGANMNRKGIATTDMQLAKDTVEWIITRKIFTESADEFTLVLFGS
ELTQNPVTVDENIFFCEEEMQQAKIDWLRLIDKEIKPSKSTNGDFLAALIAALDYMRNHLESWPKSNITA
RNILLVTNLGGFNENVDEECIGAVINGMKALEINFNVIGPSIGMVSEDEDKIISNEESTIQPEEKLSNAM
RSFKIEPAERVLTDILKQTDGVIYSFAEALPVLQHFVSRKVNLRGQKFNLELGIDLKLPLQMYKKIQTTD
FKLAAEKYASITGTRLKRKTLYEKCVKDGEVDDGLTAMDVDVGRSDCASQGSSKIFAKEIVKGYKFGTTI
VPYNAEDQKEYGWKHENRCLKLIQFTKRSQILEHYLMDGGACYFIPPALDKNACVAISALVNAMIAEDSV
ALTRYVYNAASQPRIMGLFPRRSKKGVDMFVGIQLPFYEDFRGLNFPPLNSPATEPKNDHLSAMHSFVQA
MDLTKAHFNSQTGQFEESLRPRDVPNPKLQNVCKAIKYRALHPNAPLPAFEDKLLGDLLEPNALLLKRAN
ESLVYLKTNLPMFESPNKKQHIKEVKEEILPRMSADNSVLPEGGKFIKTDEVDA

>E1FMW6_LOALO
MSADVMSLAKRLDLQPKEINCLKSEKGKLLIFETKEYVNSLSEQNLRSKLRLEARRRMWKHKLESLPSDE
DIELFCINLQNKATYSRSEDDKLVDYNSYKQVINLAPKALQDFLSAEIFMNLLQISNTKDEGRIAVDSII
DYLLTKKDKLSKLIHLHYYDSASKGYLSIEDLRDFLEAEVLPLIPSLANLNEEEPMLQDYYLCMATRKIF
FLLDTMQQKKVRIVDIVASRLLEEFETLNESSNGNAGDDESVEMEQGTSQTTGTLNWFSKRNCLRIKNIY
EQLDTDGNGLLSFSEMTDFQRITNSFMQRVFEVQQTYDNDELDLRGFCDLLFAIEHKSDRSALSYYFRVL
DVDGDNLLSASDLNFFYRDLAKMLEDCFSDGSSQKAPLFDDIKNEIFDMCHPKNPKGITLKELISSGKGG
TVVGLLTDLDAFLEYENREDVIYEE

>E1FMW7_LOALO
MGESGFILLPTEVDKIVDPFVDAGNLLIVDNDPLIDEDSKKKPSEEELSARVRDNAQFLFNKIWELERKR

IDEAICAKLPGPLFRLPREKPLPSERQLTKWEQYAQQKGIRKRKKDRKVFDEQTQEWKARYGYKSVKDNN
AKEWLIEIPDNKGNMIFFF

>E1FMX3_LOALO
MWIFGYGSLLWYTDFPYQSVIPGIVRGYSRRFWQLSPDHRGTTSSPGRTVTLVEDANGSCWGLAYEVAEE
QASNTIKYLDVREKAGYLRKEVMFYPDNGSSFFPINVYLAAENGNPYFTGPTDEKSIVHTILRARGISGT
NIEYVLRLAECVHRMAPHINDEHLFAIEREVVDGCHQLNIQDDYLAKYLNQKRTIESLTKKP

>E1FMX4_LOALO
MYSVRMVVMFLLIVLVSISIIIRNTEGDNKHVIIDERFGDNISKYQSNNTRYDENNHEDIKYEEFDIKKS
KRKSLKQDIFMLHGSNKFKVSYPPAKYRFFKTMLNKDLCDQYTLECIYYRSEYPRSRLSFDWKTGKEYNP
CHRFVEPCFGISEYDYKIARKFKSLKDY

>E1FMX7_LOALO
MPMLQLSDSFLGSQQNMQEVAKYRSAETGSFQLSSYEILRRVKPTPRYLYELILCEPIFETDQIGPTKLN
ITDDV

>E1FMX8_LOALO
MKKAKEFSRRNENREGNRVRRRMDLLEMLRCAMNEERQKFLTSFRKKKGKKDEEEKEALPACFVNSFKTS
FANVVLMIVLIASTLFVGRIASFFDKIVLLELLNE

>E1FMY0_LOALO
MDRIVSYLSDRLLKNSSENWNTCHIAGNATWLTINSSGVINKLHAEHLSSRQDFNIATAVEIPDELVDGS
FLADYRSKVRDSRIWESWNDRCLTLQRRISPCHFGLYVCLWPNNNEHQLMRSSPLVD

>E1FMY4_LOALO
MMLTMIGMLQEFSRRKRAKWMITKEAKLEVYLKWFAFDSFSPPLQRQLLIYSLWDNESDKIELYSTENGA
EVEHIYRQQIHYKYIDFFMNIIYCNNILILIQKQKHLDPKTRKKCRIRISPSDTQHTLKVR

>E1FMY6_LOALO
MTPIQKPQAVKIMGLAFVLAITIPLSMFIKLRFIEYENFTANFALNTEVLMTLDIALMVQLYSPFNILIQ
GINEEMLVSLIMSIKVISEYESYAVNSNGCRVRLLFPQRLSLTGTLGPEISGGLCD

>E1FMY8_LOALO
MRGIGGTSLSERNLRYPGECQVSRPDLMHAPPSVKLLHPILLIHAFLQLTDKLEGVETGWSGAVMQLRDH
RIGEQKCHWVVTIVLEKRRVG

>E1FMZ2_LOALO
MLTQVQFTPTPEYKTVLLGNGSVGKSALVKKEIAIDGTICIVKVLDIAGTISFDSIYIANSSFESQECSS
NARFIHKNGDGFIVMYSLIDAATLTDTT

>E1FMZ4_LOALO
MNGEQRRKRSPAANSARISLWDEELRNNAWENLAISQLLPWRTISREQTGHPEGRQPEHHIVLRSANNFK
NCYFSPIQCVLVEEQ

>E1FMZ6_LOALO
MAKAHLFSSIFALLLFGAALNAQISKKGSFSSRVRFVITDDNNKMHLINNHCTTIRRLTALKLLEKPAIV
EFVTQCEKGPIDLIFLVDTNTRSIIDFNEQQNRITDTIRWALWSLLPDCSLRMYKGMKKHQDA

>CLDZ_DANRE
MSTGLQLLGTTLGTLGWLGIIISCAIPLWRVTAFIGNNIVTAQTMWEGLWMSCVVQSTGQMQCKVYDSML
ALAQDLQASRAILVISAIVGLIAMFASFAGGKCTNCLADNSAKALVATTGGVAFIIAGILGLVPPSWTAN
TIIRDFYNPLVAEAQKREFGAAIFICWGAAVLLVIGGGLLCSSYPKGRTSSRGRYTPASQNGRERSEYV

>CLD7B_DANRE
MAHKGLQLLGFTLSLLGLIGLIIGTIMPQWKMSAYVGDNIITAIAMYQGLWMSCAYQSTGQQQCKVYDSV
LQLDSALQATRALMVVAILLTVAGLGVASMGMKCTNCGGDDKVKKSRIAMTGGIILSVGALCSIVACGWF
TSQIIRDFYNPFTPVNTKYEFGAAIFIAWAGAFLDIMGGGMLASSCSKGQSSPNYPKSSRPVKSSRPPSS
SKEYV

>CND3_XENLA
MVKEGKAMEIKEAFDLSQKAHQNHAKLVSSLRAAYNKTEDKSIFLEEFIHFLKFPLIVYRREPAVERVMD
FVAKFVTSFHNSGGENEEEADEENSPVNCLFNFLLQSHGASSMAVRFRVCQLINKLLVNLPENAQIDDDL
FDKIHDAMLIRLKDRVPNVRIQAVLALARLQDPSDPDCPVSNAYVHLLENDSNPEVRRAVLTCIAPSAKS
LPKIVGRTMDVKEPVRKLAYQVLSEKVHIRALTIAQRVKLLQQGLNDRSAAVKDVIQKKLIQAWLQYSEG
DVLDLLHRLDVENSPEVSLSALNALFSVSPVGELVQNCKNLDERKLIPVETLTPENVLYWRALCEHLKSK
GDEGEAALENILPEPAVYARYLSSYLQTLPVLSEDQRADMTKIEDLMTKEFIGQQLILTIGCLDTSEEGG
RKRLLAVLQEILVMQNTPTSLISSLAELLLFVLKDDDKRIQTVAEIISELREPIVTVDNPKDAAQSRKLQ
LKLADVKVQLIEAKQALEDSLTNEDYSRASELKEKVKELESLKTQLIKEAEEPEMKEIRVEKNDPETLLK
CLIMCNELLKHLSLSKGLGGTLNEICESLILPGITNVHPSVRNMAVLCIGCCALQNKDFARQHLPLLLQI
LQLDEVKVKNSALNAVFDMLLLFGMDILKSKPTNPDDSQCKAQENADEDISEQEKPGSVDENLTNEEVQE
ETATVNGILHLFSGFLDSEIAEIRTETAEGLVKLMFSGRLISAKLLSRLILLWYNPVTEEDTKLRHCLGV
FFPIFAYSCRSNQECFAEAFLPTLQTLFNAPASSPLADVDVANVAELLVDLTRPSGLNPQNKQSQDYQAA
MVHDGLAIKICNEILKDPTAPDVRIYAKALCSLELSRENSTDLLPLLDCAVEDVTDKVCERAIEKVRSQL
RSGREEHRVSKETEPQVSKETEDRTNLQENEEGKQKDEANCDENTDTVKEKAARGKATKGRRKGPAAAAT
RRKASKAEEAEAEMERQEESQCVPVNTRPSRRAKTAALEKTKKNLSKLLNEEAN

>BAG6A_XENLA
MAANEKMEVTVKTLDSQTRTFTVETEISVKDFKAHISSDVGISPEKQRLIYQGRVLQEDKKLKEYNVDGK
VIHLVERAPPQTQPSTGGPSTSSSTSPTSSNAAPVPGAPERNGNSYVMVGTFNLPHVMSGLVRQQRPSVS
TVNGNDGSTLDVHINLDQQLPVQSEPRVRLVLAQHILQDIQRILDRLEGQAVNEQAAEPMDTAESEGEAS
SRETLPQTTQNTDGQSNTTPTSHPSPSEYVEVLQSLSRVEERLAPFMQRYREILSSATSDAYENQEEREQ
SQRIINLVGESLRLLGNALVAVSDLRCNLSSASPRHLHVVRPMSHYSGPMLLQQAAIPIQINVGTTVTAT
GNGTHAGHMPSDGNAAQPPSTNTSEPQRPNTENQPPSNGERPASDAPPTSVPHPHPRVIRITHQTVEPVM
MMHMNIQDSASGGPTTIPPPTAGHGGSAHIHMPGLPPEFMQAISHQITQQAMAAASGQQIPGFQAPPRFV
FTRPAAPSFQFQPGTATTPPGPGGATTTVPGATVGPAGNASLAQMISGLVGQLLMHPVVVAQGGSSTSSS
TSSSTFTSTSSSASSSSSTDTTSTTTTSSTANPTVSSVPSSQPPPGTDQHLSQLLGSLLGTASSGMSNIT
MGSPSITVTVPGMPAFLQGVTDILQATQTVPVSTAPTQSASQAPPPSSPPPPPAHSSPPPAAAPESLPPE

FFTSVVQGVLSSMLGSLSAADQSGTESIAAFIQRLSGTHNIFQPDAEGPGGFFGDLLTLICHNFSLVDMV
MLLHGHSQPLQNLQPQLRSFFLQEYLHQVDPTPNNIQMASRNLTNGLEEYIRESFASVTVRDDVDITRTN
IEFLQDQFNRITTHILHCADSTFGQRLLEMCNQSLFEWLALNLYCLRGDQNALTSVINERIRRLSLDVSP
VLVSWVTSVLSLRLQVLLGQMPVTEGEIQRHVRRVGDAPQVPEPSSQEQPMETMPVDCQNGAASPVLATH
SGGVLFLPPQSSVPTICPDSDHPTQEDGGSEQWAASVPPEWVPVIRQDMQNQRKIKQQPPLSDAYLSGMP
AKRRKTMQGEGPHLSLSEAVSRAMKATGAKPESSAECVRRELDNSEAQGRYREQLCQDIQKTLQDNESYS
AQRFPNTQRAFRGDP

>Q9YHT5_XENLA
MRPLVMLTALIGAAFSLPLPPQPQHPGYVNFSYEILSPIKWYQSMMKNQYPNYGYEPVSGWLQSPMIPVP
PMMQQQQLPSQNAVPKLPSHHPLLIPQQPLVPVPVHHPLIPLTPQHTHQLKPIYLFNSDGQYPTNTQLLE
PSKPDHESQNGQPTFPLHPLPPLVEERPQEPWQEAGNDKQEELD

>NOGG3_DANRE
MDNIPYFLATVLIFSLGFRIEEGMCQHYYLLRPIPSDSLPIVELKEDPDPVLDPKERDLNETELRAILGS
HFEQNFMSINPPEDKHAGQDELNESELMKQRPNGIMPKEIKAMEFDIQHGKKHKPSKKLRRRLQLWLWSY
TFCPVVHTWQDLGNRFWPRYLKVGSCYNKRSCSVPEGMVCKPPKSSHLTVLRWRCVQRKGGLKCAWIPVQ
YPVISECKCSCPN

>Q9YHV6_TAKRU
MSASYPTPVYSHVGRGDFRDVYEPAEDTFLLMDALEKDAERLRQSCCTDVNPAAAQCTAKTSSSNKVSLQ
PVITSLVDSLLSRLSGKVDVLVFNPPYVVTPSEEVGSRGIEAAWAGGSRGREVTDRFLPVVAQLLSTKGS
FYLITIAENDPEEIITSLCQQGLQGESFLTRRAGNERLSVLRFLKG

>Q9YI01_DANRE
MPCRKENYIFLEQSVTVDSKEVDALVSKIGEALQLHNNSSSQKAMSRLHGFTGSKPNGGGGSSTGGQRCI
RLRSRSLRTSRASPYNPPGSSDQEWDHFKSSWNRKKIDEDDPHQLLQELILSGNLIKEAVRRLQFSSEPS
DHDIPKSVD

>Q9YI32_CARAU
MPSSSRCCATGVFILPSLLLLPLFFLSLICGVQGATLREHRLSESEPVSYGPASQIRPPPSAEMLRALRY
IQSLSQRTPPDTLDDERQQDTSDDMESIRSMMQLAAPSRMDRGMDESKEREKDKTQELLQAVLTTLQQTE
EHMMPQKNSQKVITPSQPRYAVHPFPGPKQQLEMETSSENYGRTSWTNPDTRRQHRKLPPMFDNEEDMEQ
PLKRTNENAEEQYTPQKLATLQSVFEELSGIAASNANSKREDGEDDDDNDNDLYRQRKMVLEDIMGTDEW
APLEEQTETQEEERERHGFNRNLEDDEQEEDEEDDDVKRSIQPDWFQTEKEEEPEDIAKLVDYYLLKILE
KKEQEQQKRQEAGKSEQESDREVEEKDADEDEDEEEDDKEEREIKPVHSTLPESLSQIIKISQMLQIPPE
EVLELLQNEKQKDLFRIPTKSRTPYATAHKTFAASPHRRPIEASEGNSVDEDIMNILELVNAAQQNNRPV
QAKTSSYYERETGRNDYDDTVDEDDELANYLAPKMVAHGQRRARLTPSRDEDQPVYERADYFEKSAPEKR
PGNHENAAAGLDNNTMLQILRFLDPESDDADEIDSEGKTVPEM

>TYRO3_DANRE
MEVSLCILLFLLHFNEGIHGVRFTQKPFHQTVTQGNMVRLGCAFEGLSEPEIIWMKDGEKLFSTDQMYIT
LDPYHWETFHSVKSVQQQDAGKYWCEVEYHGAIISSEPAWITVAGVPHFGVEPEDVAAFAGESFNLTCAA
SGPPEPVEVLWWLGGEQNGDFTPSPSVLFVKGVNESIKFHCEPRNARGISVSRTGTVHIKARPDSLGRTG

HHVSDFNITLTWSPGFTGHSQLSTCTIQLSRGPGEVKLPDVVVEVPPFQHVFEGLRSYSNYSVRVRCDNE
VGSSPFSPWVDFHTPQAAPSAAPKNFTFDLSEQQLTLSWATLEQEELRGRLQEELRGRLLAYKLQWNQGG
ESQDPLLFKENVAHLSGAGRFFNATFQVAACTMAGCGPWSQPVLVMPVSAMQAQTQRGHMWVGLLFGLLV
ATMVGLLLIVLIRNRGKETQFGSAFAAQGAEVPVSFTAARSFNRQFPELPESTLDSLGINSDLKAKLQDV
LIFERLLTLGRMLGKGEFGSVREAFLKSENNSGQKVAVKVLKTDINSSSDIEQCLKEAAYMKDFHHPNVI
QLIGVSLHRRAQQRLPIPMVILPFMKHGDLHTFLLMSRLGDEPFTVSQQILIQFMLDIARGMEYLSNKNI
IHRDLAARNCMLNENMSVCVADFGLSKKIYSGDYYRQGSVSKLPVKWIALESLADNVYTTQSDVWAFGVT
MWEIMTRGQTPYPGVENSEIYEYLIKGERLKQPPDCPADIYEIMHSCWSPVPKCRPSFQHLIDQLELLWA
KLNPAPVKEPLLYVNLEEEDGEQANSGTRSSEEPSWGVPWQCAGIEEDEKDWLMVSSGAALAIGGDYRYI
IGPSVSAIDEESRHSEDGLSEDIREEEEDVIINV

>Q9YI90_XENLA
MADLGQNFSLQDAMTDGPAEIESEVKQDFITSLENEKFDDEVGETCDKSNYVPLLDDDDVKEPKNKSERS
AAPHDSIMENGEHNLGENEVTDPFGSNYDENVLSDLPPFDTTNQPALSEHFEAERTVPAEELNPFEEGWL
TDSNSKTDSPSGDISEITTATCGDTPSDSSIQVPLPLTQSDNMAGIWQPTAEEQLALSPHQPAEPLRSSE
EATLLLPEGQASFDAYLQEGSRDFLSHDQEKLECGGAAELISCISFDAPLDPYAEPLPGHEFPHESEEHA
KESNIVEPADSTVNEDSPEYLSVDAQNAQLVEQVDEAALLLAEGQLNEVGQPEDTANNPVVEERDREGST
TNEELLDSEEVEHQTAETPSEKLDLVLEECKSDTSTPHGGDTPSEQELILEEENQNYEAQLPCEDFLPSK
TEDKDLLQYKTEDEDLSPLKTEGQDLLPLITEDEVLNPATPEAEVLTSHIPEAEVLTSPIPEAEVLTSPI
PEAEVLTSPIPEAEVLTSPIPEAEVLTSPIPEAEVLTSPIPEAEVLTSPIPEAEVLTSPIPEAEVLTSPI
PEVEVLFAPIPEEDVLSAPISEVEVLSAPIPEAEYFSAPTPEVEYFSAPTREAEVLSAPIPEAELLSPIT
PEAEVLSNPKTDLLDENITENLLVPEDIPAQQAVLHFEEALLQQTSSEGSPVEEQSSVVETAPEKNDEHK
AVPHVEFPQDIQAKPETAEPAAAAATENEKTSPPNKELPPSPEKKTKTSAATPSKTPLSKSKPTGAATAP
SPKRALSATPTQKKSASPAPASTTTPKRPLSSASRVTSATPKDSKPKTLDLKSPVKSPDKKPAALKQTPT
SATPRTSVKASPVASKSSTATTAAPTTGSTAPKAAVTPKRPTAPKIDVKAADVKRVPSTKSTIDASRPKS
VPADLTKANGAAAAPTRPRTTKPAVPKTTGASNTAMEAKKLPTARTAPLAKPSTAPLSKTSTAPLKTAAA
PKQPRPATVPDLKNIRSKIGSTDNIKHQPGGGKQVEKKPVPISTARKAVPPAVPKTATTTKSTDTKEAAQ
KQSNGKVQIVSKKVNYSHVQSKCGSKDNIKHVPGGGNVTNAAKPATGGTRPQASGAHKPGSANVQILNKK
IDLSKASAKCGSKPSMKNKPGAVETNTGNSKKQETAKETQEEIVKVVESQNIGEQDLSSQNGDLVTPSEV
PRQESQENGVGERSPAEGDNQRESFNTLIPETSI

>Q9YIB1_ANGJA
MRADRLFIGAIVLSLFGCLCNAQFQTRNVDGFGTNPRLASKPVGSQAPITPRPTLGRPWFTQAPVTPRPT
FGGPGITQSPVTPRPTFGRPGITQAPVTPRPTFGRPGITQAPVTPRPTLGEPTHTVEAPTAKPDAVKVHC
GESSVQMEVDMDLLGIGHLNQPSDITLGGCGPVAQAKSTRALLFETELHGCGSVLAMTEDSLVYTFAFNY
QPSAIGATPIIRTSSAVVGIQCHYLSLHNVSSNALKPTWIPYHSTLSAEDLLVFSLRIMADNWQLERTSN
VFFLGDLINIEISVVQANHVPLRVFVDTCVATLDPDMNAVPRYAFIENKGCLMDSKLTNSRSQFLSRVQD
DKLQLQLDAFRFAQETRSAIYIFCHLKATAALPDSEGKACSFPLGKERWIEASGNDQACSCCDTSCGGRK
IRSVNSGIQYEGGAVLGPIVVQEAVKDVPESISPLNADHQAEGASEVVFMAGVMAAVGLVCIIVLGMVLV
WRRYKPVL

>LEG2_CONMY
MSDRAEVRNIPFKLGMYLTVGGVVNSNATRFSINVGESTDSIAMHMDHRFSYGADQNVLVLNSLVHNVGW
QQEERSKKFPFTKGDHFQTTITFDTHTFYIQLSNGETVEFPNRNKDAAFNLIYLAGDARLTFVRLE

>SYUG_MOUSE
MDVFKKGFSIAKEGVVGAVEKTKQGVTEAAEKTKEGVMYVGTKTKENVVQSVTSVAEKTKEQANAVSEAV
VSSVNTVANKTVEEAENIVVTTGVVRKEDLEPPAQDQEAKEQEENEEAKSGED

>CDC7_MOUSE
MEEPMAFSSLRGSDRCPADDSLKKYEQSVKLSGIKRDFEELGEAVPQLVNVSKIKDKIGEGTFSSVYLAT
AQLQEGHEEKIALKHLIPTSHPMRIAAELQCLTVAGGQDNVMGLKYCFRKNDHVVIAMPYLEHESFLDIL
NSLSFQEVREYMYNLFVALKRIHQFGIVHRDVKPSNFLYNRRLKKYALVDFGLAQGTRDTKIELLKFVQS
EAQQEDCSRNKYHGVVGHKGLLSRPAPKTVDQQCTPKTSVKRSYTQVHIKQGKDGKERSVGLSVQRSVFG
ERNFNIHSSISHESPAEKLIKQSKTVDIISRKLATKKTAISTKAMNSVMRETARSCPAVLTCDCYGSNRV
CSVCLSRRQQVAPRAGTPGFRAPEVLTKCPDQTTAIDMWSAGVIFLSLLSGRYPFYKASDDLTALAQIMT
IRGSRETIQAAKAFGKSVLCSKEVPAQDLRALCERLRGLDSTTPRSASGPPGNASYDPAASKNTDHKASR
VQAAQAQHSEDSLYKRDNDGYWSHPRDCTSNSEGWDSVPDEAYDLLDKLLDLNPASRITAEAALLHVFFK
DMCS

>WDR46_MOUSE
METAPKPGRGVPPKRDKPQAKRKKPRRYWEEETTPAAVATSPGPPRKKARTGESRPPRSKSAHIAQKSRF
SKKPPISKTAPDWKKPQRTLSGAQDPFPGPVPAPLEEARKFCRIDKSKTLPHSKPKTQSKLEKAEAQEEE
ASVRAARAELLLAEEPGFLVGEDGEDTAKILQTDIVEAVDIASAAKHFDLNLRQFGPYRLNYSRTGRHLA
LGGRRGHVAALDWVTKKLMCEINVMEAVRDIHFLHSEALLAVAQNRWLYIYDNQGIELHCIRRCDRVTRL
EFLPFHFLLATTSETGFLTYLDVSVGKIVTALNVRAGRLSVMAQNPYNAVIHLGHSNGTVSLWSPAVKEP
LAKILCHRGGVRAVAVDSTGTYMATSGLDHQLKIFDLRGTFQPLSSRTLPQGAGHLAFSQRGLLVAGMGD
VVNIWAGQGKASPPSLEQPYLTHRLSGHVHGLQFCPFEDVLGVGHSGGFTSMLVPGAAEPNFDGLENNPY
RSRKQRQEWEVKALLEKVPAELICLNPRALAEVDVVTLEQQKKERIERLGYDPDAKAAFQPKAKQKGRSS
TASLVKRKKKVMDQEHRDKVRQSLEQQQQKKKQDMAMPPGARPSALDRFVRRAREGGLQVDP

>CST9_MOUSE
MSCPLRKKALPLTMLLLLLSFHVLITPVSKANKETNRSVHFIPTVEFAVNTFNQESQDEYAYRMEHIMSS
WREKVNFPTVYSMRLQLRRTICKKFEESLDICPFQESHGLNNTFTCLFTVGTYPWITKFKLFRSVCS

>Q9Z0I5_MOUSE
MKDIQLRRKEAKSILQAVCTLLNSGGGVVKAHIKNQNYSFTRDGMGLDLVNSLPGIMHLPHDYLDFMQHK
DYFFVFVKPLKPNQKGPGITTLKTNLYRRINSISDEVKVANAVQLLKSRTDPEEKAESRPSSPGKIVCNE
TLNECLSLFNRDWLAYEETFCFTKSIHAEVKLTPKEKIFPKEKILELLPQTVSAFANTDGGFLFIGLDGK
TQQIIGFEAEKSNLVLLESEIEKCIQQLPVTHFCGEKEKIKYTCKFIEVRKSGAVCAYVCALRVERFCCA
VFAAEPESWHVEGGCVKRFTTEEWVKRQMDATAVMPGKVICCPEALYMKPFSQHEGYEHLVRTELGSLLK
GTLVISKSWALDLGLQEKQEVIWDVLHISQGSLLTLYVFVQGDENLEGNSSLLGELGAELKGYYKQIALT
LKQMLLNHCGYTAEIGIIVKITYLGHKTMCLYDTSTKIRYPQKYYLSAKAVKDLEKALVEILGSYESFYS
LPRRNWDSFMSAFLNVGSYIVPVLRNV

>ITSN2_MOUSE
MMAQFPTAMNGGPNMWAITSEERTKHDKQFDNLKPSGGYITGDQARTFFLQSGLPAPVLAEIWALSDLNK
DGKMDQQEFSIAMKLIKLKLQGQQLPVVLPPIMKQPPMFSPLISARFGMGSMPNLSIHQPLPPVAPIATP

LSSATSGTSIPPLMMPAPLVPSVSTSSLPNGTASLIQPLSIPYSSSTLPHASSYSLMMGGFGGASIQKAQ
SLIDLGSSSSTSSTASLSGNSPKTGTSEWAVPQPSRLKYRQKFNSLDKGMSGYLSGFQARNALLQSNLSQ
TQLATIWTLADIDGDGQLKAEEFILAMHLTDMAKAGQPLPLTLPPELVPPSFRGGKQVDSVNGTLPSYQK
TQEEEPQKKLPVTFEDKRKANYERGNMELEKRRQVLMEQQQREAERKAQKEKEEWERKQRELQEQEWKKQ
LELEKRLEKQRELERQREEERRKEIERREAAKQELERQRRLEWERLRRQELLSQKTREQEDIVRLSSRKK
SLHLELEAVNGKHQQISGRLQDVQIRKQTQKTELEVLDKQCDLEIMEIKQLQQELKEYQNKLIYLVPEKQ
LLNERIKNMQLSNTPDSGISLLHKKSSEKEELCQRLKEQLDALEKETASKLSEMDSFNNQLKELRESYNT
QQLALEQLHKIKRDKLKEIERKRLEQIQKKKLEDEAARKAKQGKENLWRESIRKEEEEKQKRLQEEKSQD
KTQEEERKAEAKQSETASALVNYRALYPFEARNHDEMSFSSGDIIQVDEKTVGEPGWLYGSFQGKFGWFP
CNYVEKVLSSEKALSPKKALLPPTVSLSATSTSSQPPASVTDYHNVSFSNLTVNTTWQQKSAFTRTVSPG
SVSPIHGQGQAVENLKAQALCSWTAKKENHLNFSKHDVITVLEQQENWWFGEVHGGRGWFPKSYVKLIPG
NEVQRGEPEALYAAVTKKPTSTAYPVTSTAYPVGEDYIALYSYSSVEPGDLTFTEGEEILVTQKDGEWWT
GSIGERTGIFPSNYVRPKDQENFGNASKSGASNKKPEIAQVTSAYAASGTEQLSLAPGQLILILKKNTSG
WWQGELQARGKKRQKGWFPASHVKLLGPSSERTMPTFHAVCQVIAMYDYMANNEDELNFSKGQLINVMNK
DDPDWWQGETNGLTGLFPSNYVKMTTDSDPSQQWCADLQALDTMQPTERKRQGYIHELIQTEERYMDDDL
QLVIEVFQKRMAEEGFLTEADMALIFVNWKELIMSNTKLLRALRVRKKTGGEKMPVQMIGDILAAELSHM
QAYIRFCSCQLNGATLLQQKTDEDTDFKEFLKKLASDPRCKGMPLSSFLLKPMQRITRYPLLIRSILENT
PQSHVDHSSLKLALERAEELCSQVNEGVREKENSDRLEWIQAHVQCEGLAEQLIFNSLTNCLGPRKLLHS
GKLYKTKSNKELHAFLFNDFLLLTYLVRQFAAASGHEKLFNSKSSAQFRMYKTPIFLNEVLVKLPTDPSG
DEPVFHISHIDRVYTLRTDNINERTAWVQKIKGASEQYIDTEKKKREKAYQARSQKTSGIGRLMVHVIEA
TELKACKPNGKSNPYCEVSMGSQSYTTRTLQDTLNPKWNFNCQFFIKDLYQDVLCLTMFDRDQFSPDDFL
GRTEVPVAKIRTEQESKGPTTRRLLLHEVPTGEVWVRFDLQLFEQKTLL

>CLD13_MOUSE
MVVSKQEAISFSVTSLGWVGAIVSCVLPVWRVTFPDDETDPDATIWEGLWHICQVRENRWIQCTLYDTRI
LVAQDIKVSRVFMVICTIGTWLGLLLCVLGDWRINCFMNFTIEENLLKVAGGMFLSVGLLMLVPLSWVTH
NIIHGFFNPLLGFSKKVQMGSSLSLAWTSSLLLLLGGILLCVNIPVCRDFPRCIETPSARPSGANNDTLD
V

>TI17A_MOUSE
MEEYAREPCPWRIVDDCGGAFTMGTIGGGIFQAFKGFRNSPVGINHRLRGSLTAIKTRAPQLGGSFAVWG
GLFSTIDCSMVQIRGKEDPWNSITSGALTGAILAARNGPVAMVGSAAMGGILLALIEGAGILLTRFASAQ
FPNGPQFTEDHSQLPSSQLPSSPFGDYRQYQ

>PA21B_MOUSE
MKLLLLAALLTAGAAAHSISPRAVWQFRNMIKCTIPGSDPLKDYNNYGCYCGLGGWGTPVDDLDRCCQTH
DHCYSQAKKLESCKFLIDNPYTNTYSYSCSGSEITCSAKNNKCEDFICNCDREAAICFSKVPYNKEYKNL
DTGKFC

>IRS4_MOUSE
MASCSFSGHQALRRLRASAAAAASAALAAVATTPLLSSGTRTALIGTGSSCPGAMWLSTATGSRSDSESE
EEDLPVGDEVCKRGYLRKQKHGHRRYFVLKLETADAPARLEYYRNARKFRHSVRAAAAAAEAAASGAAVP
ALIPPRRVIILYQCFSVSQRADARYRHLIALFTQDEYFAMVAENESEQESWYLLLSRLILESKRRRCGTL
GALPDGEPAALAAAAAAEPPFYKDVWQVVVKPRGLGHRKELSGVFRLCLTDEEVVFVRLNTEVASVVVQL

LSIRRCGHSEQYFFLEVGRSTVIGPGELWMQVDDSVVAQNMHELFLEKMRALCADEYRARCRSYSISIGA
HLLTLLSTRRHLGLLPLEPGGWLRRYGLEQFCRLRAIREREEMLFTRRFISPREPPPPFRRGRGHLPRAR
RSRRAASVPPSLFRRSAPSPGRIPQPEDVPNDRAREASGSSSGNTEEKDKEGEEGNRGDCIPMNNWGSGN
GRGSGGGRGSSGQGSSSQGSGGRQGSGGGQGSGGQGAGGNQCSGNGQGTAGGHGSGGGGHGSGGGQRPGD
GHGSGGGKNSGSGKNSDDGDRGKSVKKRSYFGKFTQSKQQQTLPPPPPPPAAGATGGKGKSGGRFRLYF
CADRGTKERKEAKEVRDMETSGGATRGPYRARAFDEDEDDPYVPMRPGVAAPLACSSDYMPMAPQNSSAS
TKRHSRSPFEDSRGYMMMFPRVSPPPPVPSAPKAPDTNKGDDSKDNDSDSDYMFMAPGAGAIPKNPPNAQ
GGSSSKSWSSYFSLPSPFQSSPLGQSDHSEYVPMLPGKFLGSGLHKEASFSQGTKNVSSKPSTEASFSKP
EDKGSSAKPSDDVPPMNKAKEPNHLSFIAKGTQVKPKPLNPTQERREAAGSRDYINIDFIKRERLVLPSS
AQGLPDMRGVVTDPAPTAFSGYLNVEFGVPFPNPTIRLSDLLRVLPGANSIPLAGTRWPFPGSAIGSIVE
AGEYIEVIFNPAMTPAMSFADSAICYDAQTGQIYVVDPFSECCMDVSLSPGRCSEPPPVARLRREEAQER
RRPQSRSQSLFASTRAAVSAFPTDSLDRDFPAASAVIAAPAEAPLLAVSRALAVVSALAAAPSIGDVFAG
FRAAAGVDSASARGFQPVAGAQAVREFQDLAAGWNPGALNHRARGEDLAAGAAAPPPPPRQIWVLRPQER
ADSEDDDDDDDDIYVRMDFARRDYRK

>STAU1_MOUSE
MYKPVDPHSRMQSTYSYGMRGGAYPPRYFYPFPVPPLLYQVELSVGGQQFNGKGKMRPPVKHDAPARALR
TLQSEPLPERLEVNGREAEEENLNKSEISQVFEIALKRNLPVNFEVARESGPPHMKNFVTRVSVGEFVGE
GEGKSKKISKKNAARAVLEQLRRLPPLPAVERVKPRIKKKSQPTCKTAPDYGQGMNPISRLAQIQQAKKE
KEPEYMLLTERGLPRRREFVMQVKVGHHTAEGVGTNKKVAKRNAAENMLEILGFKVPQAQPAKPALKSEE
KTPVKKPGDGRKVTFFEPSPGDENGTSNKDEEFRMPYLSHQQLPAGILPMVPEVAQAVGVSQGHHTKDFT
RAAPNPAKATVTAMIARELLYGGTSPTAETILKSNISSGHVPHGPRTRPSEQLYYLSRAQGFQVEYKDFP
KNNKNECVSLINCSSQPPLVSHGIGKDVESCHDMAALNILKLLSELDQQSTEMPRTGNGPVSACGRC

>Q9Z117_MOUSE
MEQNKETWKMERQEAVAKDPGPQASYSLFFKEKIKEETQKEMAGSPLIMSQRLLTFGDVAVEFPQEEWEC
LDSAQRALYIDVMLENYSNLVSVENYCISDTVHQHVKTEKESCQGNELGEMLHEPTNCALYIRRDTIETS
NNYRCSKDRDGSVDLLNQDRHKSTHTGEELCKSEDCEKSLSLSSNLTENQRLYAANKLQSQGEYDDDFSS
VYSLMQQTIYIGDTPHQCEKCRKCFSTASSLTVHKRIHTGKKPYKCSVCGKSFTQCTNLKTHQRLHTGEK
PYKCKECGKSFPQLSALKSHQKIHTGERPYKCKECDKSFAHCSSFRRHQKTHRAEEHCSCPECGKVLHQL
SHLRSHYRLHTGEKPYKCNECDRSFTYYASYRRHQKTHSLDKFYKCKECGKSFLELSHLKRHYRIHTGEK
PYKCEVCDKSFTVNSTLKTHLKIHTGEKPFKCRECDKSFTKCSHLRRHQSVHTGEKPYRCKECDKSFTEC
STLRAHQKIHTGEKPYKCRECDKSFIQRSNLIIHQRVHTGERPYICKECGKSFTKCSTLQIHQKIHTGEK
PYKCMECNKSFTQDSHLRTHQRVHTGERPYICKECGKSFTRCSYLRAHQKIHTGEKPYKCKDCDISFIQI
SNLRRHQRVHTGEKNTIATIVT

>Q9Z161_RAT
MVQSWLQGPKSSPGLSLDTCRSEAPKGAVHAHHLAVHMCPSQTCLLTHSDRDVIVPIVLSYEGHHWPVLG
GISGIDCDEFLSVILGQPVHLDGVANSIGKKEHFNLFKAASVSHLVQVVPQELFRVV

>P2R3A_MOUSE
MPERPPIRALRRDPDDPAVAQALASLARGSDLVFPSRFQKWLRDFRQVHAHRKEEPPPQSPPPGHTVPAF
YFPCGRPPPRPQDTEDAIALVECAFEGLPRGRAGLGDMAVVAKACGCPLYWKAPLFYAAGGERTGSVSVH
MFVAMWRKVLLTCHDDAARFVRLLGHPGCSGLIQEDFVPFLQDVVNSHPGLAFLRAAKDFHSRYITTVIQ

RIFYTVNRSWSGMISREELRRSSFLQAVSQLEVEPDINRMTSFFSYEHFYVIYCKFWELDLDRDLTIDRS
DLARHGDGAISSRMIDRIFSGAVTRARLPRKVGKLSYADFVWFLLSEEDKTTPTSTEYWFRCMDLDGDGA
LSMFELEFFYEEQAQRMAARGVEPLPFHDLARQVLDLVAPRCPGRITLRDLKQCGLAGEFFDAFFNVDKY
LAREQREQAGTPQDTDSDPAASAWDRYAAEEYDFLVAEEAMAEDDDDHDEGSDPIDLYGLADEDCDDLEP
L

>Q9Z190_MOUSE
MIFIVAERRQGVLTLGAV

>EYA4_MOUSE
MEDTQDLNEQSVKKTCPEADVSEPQNSRSMEMQDLASPHALVGGSDTPGSSKLDKSGLSSTSVTTNGTGV
SLLAVKTEPLHSSESTTTTGDGALDTFTGSVITSSGYSPRSAQQYSPQLYPSKPYPHILSTPAAQTMSAY
AGQTQYSGMQQPAVYTAYSQTGQPYSLPAYDLGVMLPAIKTESGLSQTQSPLQSGCLSYSPGFSTPQPGQ
TPYSYQMPGSSFAPSSTIYANNSVSNSTNFSSSQQDYPSYTAFGQNQYAQYYSASTYGAYMTSNNTADGT
SSSTSTYQLQESLQGLTSQPGEFDTVQSPSTPIKDLDDRTCRSSGSKSRGRGRKNNLSPPPDSDLERVFV
WDLDETIIVFHSLLTGSYAQKYGKDPPMAVTLGLRMEEMIFNLADTHLFFNDLEECDQVHIDDVSSDDNG
QDLSTYSFATDGFHAAASSANLCLPTGVRGGVDWMRKLAFRYRRVKELYNTYKNNVGGLLGPAKRDAWLQ
LRAEIEGLTDSWLTNALKSLSIISTRSNCVNVLVTTTQLIPALAKVLLYSLGGAFPIENIYSATKIGKES
CFERIVSRFGTNITYVVIGDGRDEEHAANQHNMPFWRISSHSDLLALHQALELEYL

>ZSC12_MOUSE
MTSTSDTKVCKNQGGLLEIKMEEECKYTTRQDRNLQKNTYNRDVFRKYFRQFCYQETSGPREALSRVREL
CRQWLRPDLNSKEQILELLVLEQFLTILPGELQAWVQEQNPESVEEVVTVLEDLERELDELGYRASVQTE
EQVTFQEVNALATEQKPSVSLQFVKAKPGCELAGREAQEEQVSGVETGNEPRNVTLKQGLWEGTEAEQNP
ASRLAKDALECEEAHNPGEESSGISHEDSQPLRNENGVNSPANSEYAKHQSICPGRKVHGCDECGKSFTQ
HSRLIEHKRVHTGDRPYKCEVCGKTFRWRTVLIRHKVVHTGEKPYKCNECGRAFGQWSALNQHQRLHSGE
KHYHCNECGKAFCQKAGLFHHLKSHRRNRPYQCLQCNKSFNRRSTLSQHQGVHTGAKPYECNDCGKAFVY
NSSLATHQETHHKEKPFTQSGPIQQQRNHTKEKPYKCSVCGKAFIQKISLIEHEQIHTGERPYKCAEGGK
AFIQMSELTEH

>DEF3B_RAT
MRTLILLTTLLLLALHTQAESPQGSTKEAPDEEQDISVFFGGDKGTALQDAAVKAGVTCSCRTSSCRFGE
RLSGACRLNGRIYRLCC

>Q9Z1F3_MOUSE
MPGQLKPSLQQSLAQYPNHKEIAATVIGHVQVIPCSREGFDVTHHSTWHGVTQLTSVGGMNNLGDPEKAT
RRLGSQVSD

>SAE2_MOUSE
MALSRGLPRELAEAVSGGRVLVVGAGGIGCELLKNLVLTGFSHIDLIDLDTIDVSNLNRQFLFQKKHVGR
SKAQVAKESVLQFHPQANIEAHHDSIMNPDYNVEFFRQFILVMNALDNRAARNHVNRMCLAADVPLIESG
TAGYLGQVTTIKKGVTECYECHPKPTQRTFPGCTIRNTPSEPIHCIVWAKYLFNQLFGEEDADQEVSPDR
ADPEAAWEPTEAEARARASNEDGDIKRISTKEWAKSTGYDPVKLFTKLFKDDIRYLLTMDKLWRKRKPPV
PLDWAEVQSQGEANADQQNEPQLGLKDQQVLDVKSYASLFSKSIETLRVHLAEKGDGAELIWDKDDPPAM

DFVTSAANLRMHIFSMNMKSRFDIKSMAGNIIPAIATTNAVIAGLIVLEGLKILSGKIDQCRTIFLNKQP
NPRKKLLVPCALDPPNTNCYVCASKPEVTVRLNVHKVTVLTLQDKIVKEKFAMVAPDVQIEDGKGTILIS
SEEGETEANNPKKLSDFGIRNGSRLQADDFLQDYTLLINILHSEDLGKDVEFEVVGDSPEKVGPKQAEDA
AKSIANGSDDGAQPSTSTAQEQDDVLIVDSDEEGPSNSTDCSGDDKARKRKLEENEAASTKKCRLEQMED
PDDVIALD

>VPP1_MOUSE
MGELFRSEEMTLAQLFLQSEAAYCCVSELGELGKVQFRDLNPDVNVFQRKFVNEVRRCEEMDRKLRFVEK
EIRKANIPIMDTGENPEVPFPRDMIDLEANFEKIENELKEINTNQEALKRNFLELTELKFILRKTQQFFD
EAELHHQQMADPDLLEESSSLLEPNEMGRGAPLRLGFVAGVINRERIPTFERMFWRVCRGNVFLRQAEIE
NPLEDPVTGDYVHKSVFIIFFQGDQLKNRVKKICEGFRASLYPCPETPQERKEMASGVNTRIDDLQMVLN
QTEDHRQRVLQAAAKNIRVWFIKVRKMKAIYHTLNLCNIDVTQKCLIAEVWCPVTDLDSIQFALRRGTEH
SGSTVPSILNRMQTNQTPPTYNKTNKFTHGFQNIVDAYGIGTYREINPAPYTVITFPFLFAVMFGDFGHG
ILMTLFAVWMVLRESRILSQKHENEMFSMVFSGRYIILLMGLFSIYTGLIYNDCFSKSLNIFGSSWSVRP
MFTQGNWTEETLLGSSVLQLNPAIPGVFGGPYPFGIDPIWNIATNKLTFLNSFKMKMSVILGIIHMLFGV
SLSLFNHIYFKKPLNIYFGFIPEIIFMSSLFGYLVILIFYKWTAYDAHSSRNAPSLLIHFINMFLFSYPE
SGNAMLYSGQKGIQCFLIVVAMLCVPWMLLFKPLILRHQYLRKKHLGTLNFGGIRVGNGPTEEDAEIIQH
DQLSTHSEDAEEFDFGDTMVHQAIHTIEYCLGCISNTASYLRLWALSLAHAQLSEVLWTMVIHIGLHVRS
LAGGLGLFFIFAAFATLTVAILLIMEGLSAFLHALRLHWVEFQNKFYTGTGFKFLPFSFEHIREGKFDE

>NEK4_MOUSE
MPQAAYCYMRVVGRGSYGEVTLVKHRRDGKQYVIKKLNLRNASSRERRAAEQEAQLLSQLKHPNIVTYKE
SWEGGDGLLYIVMGFCEGGDLYRKLKEQKGQLLPESQVVEWFVQIAMALQYLHEKHILHRDLKTQNVFLT
RTNIIKVGDLGIARVLENHGDMASTLIGTPYYMSPELFSNKPYNYKSDVWALGCCVYEMATLKHAFNAKD
MNSLVYRIIEGKLPPMPKVYSTELAELIRTMLSRRPEERPSVRSILRQPYIKHHISLFLEATKAKTSKNN
VKNCDSRAKPVAAVVSRKEESNTDVIHYQPRSSEGSALHVMGEDKCLSQEKPVDIGPLRSPASLEGHTGK
QDMNNTGESCATISRINIDILPAERRDSANAGVVQESQPQHVDAADEVDSQCSISQEKERLQGNTKSSDQ
PGNLLPRRSSDGGDGEGSELVKPLYPSNKDQKPDQDQVTGIIENQDSIHPRSQPHSSMSEPSLSRQRRQK
KREQTAHSGTKSQFQELPPRLLPSYPGIGKVDIIATQQNDGNQGGPVAGCVNSSRTSSTASAKDRPLSAR
ERRRLKQSQEEMLPSGPAVQRTPSAVEPLKPQEEDQPIPAQRFSSDCSITQMNHTLPREKEKRLMHGLSE
DELSSSTSSTDKSDGDSREGKSHTNEMKDLVQLMTQTLRLEAKESCEDLQVLNPGSEFRLHRKYRDTLVL
HGKVAEEVEPHCTELPTGIIPGSEKIRRIVEVLRADVIQGLGIQLLEQVFDLLGEEDELEREARLQEHMG
DKYTTYCVKARQLKFFEENVSF

>NDUA7_MOUSE
MASATRVIQKLRNWASGQDLQAKLQLRYQEIAKRTQPPPKLPVGPSHKLSNNYYCTRDGRREVVPPSIIM
SSQKALVSGKAAESSAMAATEKKAVTPAPPMKRWELSKDQPYL

>KANK3_MOUSE
MAKFVLNQNLPDLGGPPLYPGPTGSARSPSSPYSVETPYGFHLDLDFLKYVEEIERGPASRRTPGPPHAR
RPRASRTGLAGARSPGAWTSSESLASDDGGASGALSPGAFPGLSLPPLSPRSLSRNPRVEHTLLETSRRL
EQAQARERALSPARAVTRSPRGSGRSSPAPNPALASPGPAQLQLVREQMAAALRRLRELEDQARALPELQ
EQVRALRAEKARLLAGRVQPEQEVEIEARPDKLAQLRRLTERLATSDRGVRSRASPRAEDPDGLAARRSE
GALQVLDPGSRTPDGEPRTRETGTEVVPETREVDAQAVPETGEAGVEVVPETVEVDTWVTEELLGLPEAA

ERELELLRTSLEHQRGVSELLRGRLRELEEAHEAAVTRPQSRDVAAQTTLGCTEKTTQTELPVENQPRPT
AGDEMAPVGILKSIMKKKDGIPGAQSSQGPKSLQFVGVLNGEYESSSSEDGNSDDEDGVAEHPRSSSSGS
DDSSGGSDAGTPGPHNDKDAGDCELETHPELTAGREGRCELNPRLREACIALNQQLNRPRGVTSRDGNAA
RLVAQEWFRVSSQKRSQAESVAGVLRGVKSLGPELLAYVVNLADGNGNTALHYSVSHGNLAISSLLLDTG
VCDVNHQNRAGYSALMLAALTSVGQEEEDMAVAQRLFSMGDVNAKASQTGQTALMLAISHGHQDMVAALL
ECGADVNVQDADGATALMCASEYGRLDTVQLLLAQPGCDLTILDNEGTSALAIALEAEQDEVAALLHAHL
TSNHQGQSSTGSPTAKECNDK

>BAG6_MOUSE
MEPSDSASTAMEEPDSLEVLVKTLDSQTRTFIVGAQMNVKEFKEHIAASVSIPSEKQRLIYQGRVLQDDK
KLQEYNVGGKVIHLVERAPPQTQLPSGASSGTGSASATHGGAPLPGTRGPGASVHDRNANSYVMVGTFNL
PSDGSAVDVHINMEQAPIQSEPRVRLVMAQHMIRDIQTLLSRMECRGGTQAQASQPPPQTPQTVASETVA
LNSQTSEPVESEAPPREPMESEEMEERPPTQTPELAPSGPAPAGPAPAGPAPAPETNAPNHPSPAEHVEV
LQELQRLQRRLQPFLQRYCEVLGAAATTDYNNNHEGREEDQRLINLVGESLRLLGNTFVALSDLRCNLAC
APPRHLHVVRPMSHYTTPMVLQQAAIPIQINVGTTVTMTGNGARPPPAPGAEAATPGSAQATSLPPSSTT
VDSSTEGAPPPGPAPPPASSHPRVIRISHQSVEPVVMMHMNIQDSGAQPGGVPSAPTGPLGPPGHGQTLG
QQVPGFPTAPTRVVIARPTPPQARPSHPGGPPVSGALQGAGLGTNTSLAQMVSGLVGQLLMQPVLVAQGT
PGMAQAQAQAQAQAQAQAQAPAPAPAPAPAPATASASAGTTNTATTAGPAPGGPAQPPPPQPSAADLQFS
QLLGNLLGPAGPGAGGPGMASPTITVAMPGVPAFLQGMTDFLQASQTAPPPPPPPPPPPPAPEQQSTPPP
GSPSGGTASPGGLGPESLPPEFFTSVVQGVLSSLLGSLGARAGSSESIAAFIQRLSGSSNIFEPGADGAL
GFFGALLSLLCQNFSMVDVVMLLHGHFQPLQRLQPQLRSFFHQHYLGGQEPTPSNIRMATHTLITGLEEY
VRESFSLVQVQPGVDIIRTNLEFLQEQFNSIAAHVLHCTDSGFGARLLELCNQGLFECLALNLHCLGGQQ
MELAAVINGRIRRMSRGVNPSLVSWLTTMMGLRLQVVLEHMPVGPDAILRYVRRVGDPPQTLPEEPMEVQ
GAERTSPEPQRENASPAPGTTAEEAMSRGPPPAPEGGSRDEQDGASADAEPWAAAVPPEWVPIIQQDIQS
QRKVKPQPPLSDAYLSGMPAKRRKTMQGEGPQLLLSEAVSRAAKAAGARPLTSPESLSRDLEAPEVQESY
RQQLRSDIQKRLQEDPNYSPQRFPNAHRAFADDP

>GRP1_MOUSE
MGTLGKAREAPRKPCHGSRAGPKARLEAKSTNSPLPAQPSLAQITQFRMMVSLGHLAKGASLDDLIDSCI
QSFDADGNLCRNNQLLQVMLTMHRIIISSAELLQKVMNLYKDALEKNSPGVCLKICYFVRYWITEFWIMF
KMDASLTSTMEEFQDLVKANGEETHCHLIDTTQINSRDWSRKLTQRIKSNTSKKRKVSLLFDHLEPEELS
EHLTYLEFKSFRRISFSDYQNYLVNSCVKENPTMERSIALCNGISQWVQLMVLSRPTPQLRAEVFIKFIH
VAQKLHQLQNFNTLMAVIGGLCHSSISRLKETSSHVPHEINKVLGEMTELLSSCRNYDNYRRAYGECTHF
KIPILGVHLKDLISLYEAMPDYLEDGKVNVQKLLALYNHINELVQLQEMAPPLDANKDLVHLLTLSLDLY
YTEDEIYELSYAREPRNHRAPPLTPSKPPVVVDWASGVSPKPDPKTISKHVQRMVDSVFKNYDLDQDGYI
SQEEFEKIAASFPFSFCVMDKDREGLISRDEITAYFMRASSIYSKLGLGFPHNFQETTYLKPTFCDNCAG
FLWGVIKQGYRCKDCGMNCHKQCKDLVVFECKKRIKSPAISTENISSVVPMSTLCPLGTKDLLHAPEEGS
FIFQNGEIVDHSEESKDRTIMLLGVSSQKISVRLKRTVAHKSTQTESFPWVGGETTPGHFVLSSPRKSAQ
GALYVHSPASPCPSPALVRKRAFVKWENKESLIKPKPELHLRLRTYQELEQEINTLKADNDALKIQLKYA
QKKIESLQLGKSNHVLAQMDHGDSA

>SEPT3_MOUSE
MSKGLPEARTDAAMSELVPEPRPKPAVPMKPVSINSNLLGYIGIDTIIEQMRKKTMKTGFDFNIMVVGQS
GLGKSTLVNTLFKSQVSRKASSWNREEKIPKTVEIKAIGHVIEEGGVKMKLTVIDTPGFGDQINNENCWE

PIEKYINEQYEKFLKEEVNIARKKRIPDTRVHCCLYFISPTGHSLRPLDLEFMKHLSKVVNIIPVIAKAD
TMTLEEKSEFKQRVRKELEVNGIEFYPQKEFDEDLEDKTENDKIRQESMPFAVVGSDKEYQVNGKRVLGR
KTPWGIIEVENLNHCEFALLRDFVIRTHLQDLKEVTHNIHYETYRAKRLNDNGGLPPVSVDTEESHDSNP

>AP3B1_MOUSE
MSSNSFAYNEQSGGGEAAELGQEATSTISPSGAFGLFSSDWKKNEDLKQMLESNKDSAKLDAMKRIVGMI
AKGKNASELFPAVVKNVASKNIEIKKLVYVYLVRYAEEQQDLALLSISTFQRALKDPNQLIRASALRVLS
SIRVPIIVPVMMLAIKEASADLSPYVRKNAAHAIQKLYSLDPEQKEMLIEVIEKLLKDKSTLVAGSVVMA
FEEVCPDRIDLIHRNYRKLCNLLVDVEEWGQVVIIHMLTRYARTQFVSPWREDGGLEDNEKNFYESEEEE
EEKEKSSRKKSYAMDPDHRLLIRNTKPLLQSRNAAVVMAVAQLYWHISPKSEAGVISKSLVRLLRSNREV
QYIVLQNIATMSIERKGMFEPYLKSFYVRSTDPTMIKTLKLEILTNLANEANISTLLREFQTYVRSQDKQ
FAAATIQTIGRCATSISEFTETCFNGLVCLLSNRDEIVVAESVVVIKKLLQMQPAQHGEIIRHMAKFLDS
ITVPVARASILWLIGENCERVPKIAPDVLRKMAKSFTSEDDLVKLQILNLAAKLYLTNSKQTKLLTQYIL
NLGKYDQNYDIRDRTRFIRQLIVPNEKSGALSKYAKKIFLAPKPAPLLESPFKDRDRFQLGTLSHTLNIK
ASGYLELSNWPEVAPDPSVRNVEVIESAKEWTPLGKTKKEKPMKKFYSESEEEEDEDEDEDEEEEEKEDE
DENPSDSSSDSESGSGSESGDTGTEDSSEDSSSGQDSETGSQAEAERQKVAKRNSKTKRKSDSENREKKN
ENSKASESSSEESSSMEDSSSESESESGSDSEPAPRNVAPAKERKPQQERHPPSKDVFLLDLDDFNPVST
PVALPTPALSPSLIADLEGLNLSTSSSVINVSTPVFVPTKTHELLHRMHGKGLAAHYCFPRQPCIFSDKM
VSVQITLTNTSDRKIENIHIGGKGLPVGMQMHAFHPIDSLEPKGSVTVSVGIDFCDSTQTASFQLCTKDD
CFNVTLQPPVGELLSPVAMSEKDFKKEQGTLTGMNETSATLIAAPQNFTPSMILQKVVNVANLGAVPSSQ
DNVHRFAARTVHSGSLMLVTVELKEGSTAQLIINTEKTVIGSVLLRELKPVLSQG

>Q9Z1W6-4
MAARSWQDELAQQAEEGSARLRELLSVGLGFLRTELGLDLGLEPKRYPSWVILVGTGALGLLLLFLLGYG
WAAACAGARKKRRSPPRKREEVTPPTPAPEDPAQLKNLRSEEQKKKNRKKLPEKPKPNGRTVEIPEDEVV
RTPRSITAKQPPETDKKNEKSKKNKKKSKSDAKAVQNSSRHDGKEVDEGAWETKISHREKRQQRKRDKVL
TDSGSLDSTIPGIENTITVTTEQLTTASFPVGSKKNKVSSGLNENITVNGGGWSEKSVKLSSQLSAGEEK
WNSVPPASAGKRKTEQSAWTQDPGDTNANGKDWGRNWSDRSIFSGIGLSSADPSSDWNAPAEEWGNWVDE
DRASLLKSQEPISNDQKDSDDDKEKGEGALPTGKSKKKKKKKKKQGEDNSITQDTEDLEKDTREELPVNT
SKARPKQEKACSLKTMSTSDPVEVLIKNSQPIKTLPPAISAEPSVTLSKGDSDKSSSQVPPMLQDTDKPK
SNAKQNSVPPSQTKSETNWESPKQIKKKKKARRET

>HNRPC_MOUSE
MASNVTNKTDPRSMNSRVFIGNLNTLVVKKSDVEAIFSKYGKIVGCSVHKGFAFVQYVNERNARAAVAGE
DGRMIAGQVLDINLAAEPKVNRGKAGVKRSAAEMYGSVPEHPSPSPLLSSSFDLDYDFQRDYYDRMYSYP
ARVPPPPPIARAVVPSKRQRVSGNTSRRGKSGFNSKSGQRGSSSKSGKLKGDDLQAIKKELTQIKQKVDS
LLESLEKIEKEQSKQADLSFSSPVEMKNEKSEEEQSSASVKKDETNVKMESEAGADDSAEEGDLLDDDDN
EDRGDDQLELKDDEKEPEEGEDDRDSANGEDDS

>Q9Z271_MOUSE
MHALDDVTAVVEDTADVLSVHGAGKVRVAVVAPISTSSADSQKLIPNEVLGPGHAWVLSGLGSRILRSGV
ASELWKVVLNLRFASEDFLSKQVLLVEEENHRDGAQPSVVPDALEEVQSLLQAVSLVVLPNDHVVAAAGH
HEDDGSHIIEALDPLAAFIALAAHIEHVEVDFVHLELGLKDSRGQDTAAKQVLVTWHVVGLLDHINLVQE
VLGTVNQLVLIGALVAGTHSLILPQSLGMLIEFRWEVKVRQVHHAQNVVHSELVLRVGQLHRGHQVAHGG

HNGFNGLLQVVFDVLHFSGLLTIAVAPCSLRVLFAALR

>TULP1_MOUSE
MPLQEETLREVWASDSGHEEDCLSPEPPLRPKQRPAQGQKLRKKKPETPDSLESKPRKAGAGRRKHEEPP
ADSAEPRAAQTVYAKFLRDPEAKKRDPRENFLVARAPDLGGEENSEEDSDDDDNDDDEEEEEKKEGKKEK
SSLPPKKAPKEREKKAKALGPRGDVGSPDAPRKPLRTKKKEVGEGTKLRKAKKKGPGETDKDPAGSPAAL
RKEFPAAMFLVGEGGAAEKGVKKKGPPKGSEEEKKEEEEEVEEEVASAVMKNSNQKGRAKGKGKKKVKEE
RASSPPVEVGEPREFVLQPAPQGRAVRCRLTRDKKGMDRGMYPSYFLHLDTEKKVFLLAGRKRKRSKTAN
YLISSDPTNLSRGGENFIGKLRSNLLGNRFTVFDNGQNPQRGGGGDVGSLRQELAAVVYETNVLGFRGPR
RMTVIIPGMNSDNERVPIRPRNASDGLLVRWQNKTLESLIELHNKPPIWNEDSGSYTLNFQGRVTQASVK
NFQIVHADDPDYIVLQFGRVAEDAFTLDYRYPLCALQAFAIALSSFDGKLACE

>PLD1_MOUSE
MSLKSETRVNTSTLQKIAADMSNLIENLDTRELHFEGEEVEYDASPGDPKAQEGCIPFSSIYNTQGFKEP
NIQTYLSGCPIKAQVLEVERFTSTSRVPSINLYTIELTHGEFTWQVKRKFKHFQEFHRELLKYKAFIRIP
IPTKRHTFRRQNVKEEPREMPSLPRSSENAIQEEQFFGRRKQLEDYLTKILKMPMYRNYHATTEFLDVSQ
LSFIHDLGPKGLEGMIMKRSGGHRIPGVNCCGHGRACYRWSKRWLIVKDSFLLYMKPDSGAIAFVLLVDK
EFRVKVGRKETETKYGLRIDNLSRTLILKCNSYRHARWWGGAIEEFIRKHGADFLKDHRFGSYAALHENT
LAKWYVNAKGYFEDIANAMEEASEEIFITDWWLSPEIFLKRPVVEGNRWRLDCILKRKAQQGVRIFIMLY
KEVELALGINSEYSKRTLMRLHPNIKVMRHPDHVSSSVYLWAHHEKLVIIDQSVAFVGGIDLAYGRWDDN
EHRLTDVGSVKRVTSGLSLGSLTAASVESMESLSLKDKHEFHKKEPISKIVDETDMKLKGIGKSRKFSKF
SLYRQLHRHHLHNADSISSIDSTSSYFSHCRSHQNLIHGLKPHLKLFHPSSESEQGLTRHSTDTGSIRSV
QTGVGELHGETRFWHGKDYCNFVFKDWVQLDKPFADFIDRYSTPRMPWHDIGSVVHGKAARDVARHFIQR
WNFTKIMKPKYRSLSYPFLLPKSQATAHELRYQVPGAVPAKVQLLRSAADWSAGIKHHEESIHAAYIHVI
ENSKHYIYIENQFFISCADDKVVFNKVGDRIAQRILKAHREGQRYRVYIVIPLLPGFEGDISTGGGNALQ
AIMHFNYRTMCRGESSILEQLKPELGNKWINYISFCGLRTHAELEGNLVTELIYVHSKLLIADDNTVIIG
SANINDRSMLGKRDSEMAVIVQDTETVPSVMDGKEYQAGRFARDLRLECFRLVLGYLSDPSEDLQDPVSD
KFFKEIWVSTAARNATIYDKVFRCLPNDEVHNLIQLRDFINKPILAKEDALRAEEELRKIRGFLVQFPLY
FLSEENLLPSVGTKEAIVPMEVWT

>Q9Z284_MOUSE
MALGRLSSRTLAAMLLALFLGGPALPSEIVGGRPARPHAWPFMASLQRRGGHFCGATLIARNFVMSAVHC
VTGNFRSVQVVLGAHDLRRQERTRQTFSVQRIFENGFDPSQLLNDIVIIQLNGSATINANVQVAQLPAQG
QGVGDRTPCLAMGWGRLGTNRPSPSVLQELNVTVVTNMCPRRVNVCTLVPRRQAGICFVSTLCRRRV

>KR121_MOUSE
MCHTSCSSGCQPSCCVSSSCQPSCCVSSPCQASCFVSSPCQPSCCVSSSCQSACCRPAICIPVRYQVACC
VPVSCGPTVCMAPSCQSSVCVPVSCRPVCVTSSCQSSGCCQPSCPTLVCKPVTCSNPSCC

>RGR_MOUSE
MAATRALPAGLGELEVLAVGTVLLMEALSGISLNGLTIFSFCKTPDLRTPSNLLVLSLALADTGISLNAL
VAAVSSLLRRWPHGSEGCQVHGFQGFATALASICGSAAVAWGRYHHYCTRRQLAWDTAIPLVLFVWMSSA
FWASLPLMGWGHYDYEPVGTCCTLDYSRGDRNFISFLFTMAFFNFLVPLFITHTSYRFMEQKFSRSGHLP
VNTTLPGRMLLLGWGPYALLYLYAAIADVSFISPKLQMVPALIAKTMPTINAINYALHREMVCRGTWQCL

SPQKSKKDRTQ

>E2AK3_MOUSE
MERATRPGPRALLLLLFLLLGCAAGISAVAPARSLLAPASETVFGLGAAAAPTSAARVPAVATAEVTVED
AEALPAAAGEPESRATEPDDDVELRPRGRSLVIISTLDGRIAALDAENDGKKQWDLDVGSGSLVSSSLSK
PEVFGNKMIIPSLDGDLFQWDRDRESMEAVPFTVESLLESSYKFGDDVVLVGGKSLITYGLSAYSGKLRY
ICSALGCRRWDSDEMEEEEDILLLQRTQKTVRAVGPRSGSEKWNFSVGHFELRYIPDMETRAGFIESTFK
PGGNKEDSKIISDVEEQEATMLDTVIKVSVADWKVMAFSRKGGRLEWEYQFCTPIASAWLVRDGKVIPIS
LFDDTSYTASEEALGDEEDIVEAARGATENSVYLGMYRGQLYLQSSVRVSEKFPTSPKALESVNGENAII
PLPTIKWKPLIHSPSRTPVLVGSDEFDKCLSNDKYSHEEYSNGALSILQYPYDNGYYLPYYKRERNKRST
QITVRFLDSPHYSKNIRKKDPILLLHWWKEIFGTILLCIVATTFIVRRLFHPQPHRQRKESETQCQTESK
YDSVSADVSDNSWNDMKYSGYVSRYLTDFEPIQCMGRGGFGVVFEAKNKVDDCNYAIKRIRLPNRELARE
KVMREVKALAKLEHPGIVRYFNAWLETPPEKWQEEMDEIWLKDESTDWPLSSPSPMDAPSVKIRRMDPFS
TKEQIEVIAPSPERSRSFSVGISCGQTSSSESQFSPLEFSGTDCGDNSDSADAAYNLQDSCLTDCEDVED
GTVDGNDEGHSFELCPSEASPYTRSREGTSSSIVFEDSGCGNASSKEEPRGNRLHDGNHYVNKLTDLKCS
SSRSSSEATTLSTSPTRPTTLSLDFTKNTVGQLQPSSPKVYLYIQMQLCRKENLKDWMNRRCSLEDREHG
VCLHIFLQIAEAVEFLHSKGLMHRDLKPSNIFFTMDDVVKVGDFGLVTAMDQDEEEQTVLTPMPAYATHT
GQVGTKLYMSPEQIHGNNYSHKVDIFSLGLILFELLYPFSTQMERVRILTDVRNLKFPLLFTQKYPQEHM
MVQDMLSPSPTERPEATDIIENAIFENLEFPGKTVLRQRSRSMSSSGTKHSRQPSCSYSPLPGN

>ATOH7_MOUSE
MKSACKPHGPPAGARGAPPCAGAAERAVSCAGPGRLESAARRRLAANARERRRMQGLNTAFDRLRRVVPQ
WGQDKKLSKYETLQMALSYIIALTRILAEAERDWVGLRCEQRGRDHPYLPFPGARLQVDPEPYGQRLFGF
QPEPFPMAS

>OASL2_MOUSE
MDPFPDLYATPGDSLDHFLEHSLQPQRDWKEEGQDAWERIERFFREQCFRDELLLDQEVRVIKVVKGGSS
GKGTTLNHRSDQDMILFLSCFSSFEEQARNREVVISFIKKRLIHCSRSLAYNIIVLTHREGKRAPRSLTL
KVQSRKTDDIIWMDILPAYDALGPISRDSKPAPAIYETLIRSKGYPGDFSPSFTELQRHFVKTRPVKLKN
LLRLVKFWYLQCLRRKYGRGAVLPSKYALELLTIYAWEMGTESSDSFNLDEGFVAVMELLVNYRDICIYW
TKYYNFQNEVVRNFLKKQLKGDRPIILDPADPTNNLGRRKGWEQVAAEAAFCLLQVCCTTVGPSERWNVQ
RARDVQVRVKQTGTVDWTLWTNPYSPIRKMKAEIRREKNFGGELRISFQEPGGERQLLSSRKTLADYGIF
SKVNIQVLETFPPEILVFVKYPGGQSKPFTIDPDDTILDLKEKIEDAGAGGLT

>Q9Z2G2_MOUSE
MHLFAEATTKFTLELYRQLRESDNNIFFYSPISMMTALAMLQLGAKGNTEKQIEKVLQFNETTKKTTEKS
AHCHDEENVHEQFQKLMTQLNKSNDAYDLKAANSIYGAKGFPFVQTFLEDIKEYYQANVESLDFEHAAEE
SEKKINSWVESQTNGKIKDLFPNGSLNRSTIMVLVNAVYFKGQWNHKFDEKHTTEEKFWLNKNTSKPVQM
MKQNIEFNFMFLEDVQAKIVEIPYKGKELSMIVLLPVEINGLKQLEEQLTADKLLEWTRAENMHMTELYL
SLPRFKVDEKYDLPIPLEHMGMVDAFDPQKADFSGMSSTQGLVVSKVLHKSFVEVNEEGTEAAAATGVEV
SLTSAQIAEDFCCDHPFLFFIIHRKTSSILFFGRISSP

>LETM1_MOUSE
MASILLRSCRGRGPARLAPPRAASPRGSLRDRACLSCTRTLGLTSRESVLSRCCTPAHPVYLCFKGEPLS

CWTQRPECQGTAARTTWTPASARLVVTGPQYLPVRGWHSSSPLGEDSVIEKSLKSLKDKNKKLEEGGPVY
SPPAQVVVRKSLGQKVLDELRHYYHGFRLLWIDTKIAARMLWRILNGHTLTRRERRQFLRICADLFRLVP
FLVFVVVPFMEFLLPVVVKLFPNMLPSTFETQSIKEERLKKELRVKLELAKFLQDTIEEMALKNKAAKGN
ATKDFSAFFQKIRETGERPSNEEIMRFSKLFEDELTLDNLTRPQLVALCKLLELQSIGTNNFLRFQLTMR
LRSIKADDKLISEEGVDSLTVKELQAACRARGMRALGVTEDRLKGQLKQWLDLHLHHEIPTSLLILSRAM
YLPDTLSPADQLKSTLQTLPEIVAKEAQVKVAEVEGEKVDNKAKLEATLQEEAAIQQEHLEELKRASEAV
KDIQPEVAEATLPGRPGPEPQPPVDDVILPSEVLTDTAPVLEGLKGEEITKEEIDILSDACSKLQEQKKS
LTKEKEELELLKEDVQDYSEDLQEIKKELSKTGEEKYIEESAASKRLSKRVQQMIGQIDGLITQLETTQQ
DGKLGPSQSTPTGESVISITELISAMKQIKHIPEHKLISLTSALDDNKDGNINIDDLVKVIDLVNKEDVQ
ISTTQVAEIVATLEKEEKIEEKEKAKEKAEKEAAEVKN

>PMM2_MOUSE
MATLCLFDMDGTLTAPRQKITEEMDGFLQKLRQKTKIGVVGGSDFEKLQEQLGNDVVEKYDYVFPENGLV
AYKDGKLLCKQNIQGHLGEDVIQDLINYCLSYIANIKLPKKRGTFIEFRNGMLNVSPIGRSCSQEERIEF
YELDKKEHIRQKFVADLRKEFAGKGLTFSIGGQISIDVFPEGWDKRYCLRHLEHAGYKTIYFFGDKTMPG
GNDHEIFTDPRTVGYTVTAPEDTRRICEGLFP

>Q9Z2P0_MOUSE
MSVPAAVSASRPVRSQAAGCHLPRLAISPCRRCRQTPTADVTNAFTSLKRS

>VAMP5_MOUSE
MAGKELKQCQQQADEVTEIMLNNFDKVLERHGKLAELEQRSDQLLDMSSAFSKTTKTLAQQKRWENIRCR
VYLGLAVAVGLLIILIVLLVVFLPSGGDSSKP

>Q9Z2R2_MOUSE
MKGLAGEWHQDSGLDIREKAEDFSLPWLLPRLMALVMQEEGRFRSDRNHGYLREWLRIQALTACLPSPLG
RVHYAQCSPKQKGRLPRGWASLPSLSVLVRALRASNAFSLGNYYCCPWRGTRWAKGQPGEWARPRASSPS
AHREGVAVT

>APOC3_CAVPO
MQPRVLLAVTLLALLVSARAEEIQESSLLGVMKDYMQQASKTANEMLTKVQESQVAENAREWMTESLDSM
KGYWTSLIGRLSGFLDSTPSS

>Q9Z2R8_MOUSE
MGTVSRAALILACLALASAASEGAFKASDQREMTPERLFQHLHEVGYAAPPSLPQTRRLRVDHSVTSLHD
PPLFEEQREVQPPSSPEDIPVYEEDWPTFLNPNVDKAGPAVPQEAIPLQKEQPPPQVHIEQKEIDPPAQP
QEEIVQKEVKPHTLAGQLPPEPRTWNPARHCQQGRRGVWGHRLDGFPPGRPSPDNLKQICLPERQHVIYG
PWNLPQTGYSHLSRQGETLNVLETGYSRCCRCRSDTNRLDCLKLVWEGTLDGYCERELAIKTHPHSCCHY
PPSPARDECFAHLAPYPNYDRDILTLDLSRVTPNLMGQLCGSGRVLSKHKQIPGLIQNMTVRCCELPYPE
QACCGEEEKLAFIENLCGPRRNSWKDPALCCDLSPEDKQINCFNTNYLRNVALVAGDTGNATGLGEQGPT
RGTDANPAPGSKEE

>ZN292_MOUSE
MADDEAEQERLSGGGCAAELRRLGERLQELERRLCESREPAVEAAAAYCRQLCQTLLEYAEKWKTSEDPL

PLLEVYTVAIQSYVKARPYLTSECESVALVLERLALSCVELLLCLPVELSDKQWEQFQTLVQVAHETLME
SGSCELQFLATLAQETGVWKNAVLSTILSQEPLDKEKVNEFLAFEGPILLDMRIKHLIKTNQLSQATALA
KLCSDHPEIGTKGSFKQTYLVCLCTSSPSEKLIEEISEVDCKDALEMICNLESEGDEKSALVLCTAFLSR
QLQQGDMYCAWELTLFWSKLQQRVEPSVQVYLERCRQLSLLTKTVYHIFFLIKVINSETEGAGLATCIEL
CVKALRLESTENTEVKISICKTISCLLPEDLEVKRACQLSEFLIEPTVDAYYAVEMLYNQPDQKYDEENL
PIPNSLRCELLLVLKTQWPFDPEFWDWKTLKRQCLALMGEEASIVSSIDELNDSEVYEKVDYQGERGDTS
VNGLSAAGLGTDSGLLMDTGDEKQKKKEIKELKDRGFISARFRNWQAYMQYCLLCDKEFLGHRIVRHAQK
HYKDGIYSCPICAKNFNSKDSFVPHVTLHVKQSSKERLAAMKPLRRLGRPPKITATHENQKTNINTVAKQ
EQRPIKKNSLYSTDFIVFNDNDGSDDENDDKDKSYEPEVIPVQKPVPVNEFNCPVTFCKKGFKYFKNLIA
HVKGHKDSEDAKRFLEMQSKKVICQYCRRHFVSVTHLNDHLQMHCGSKPYICIQMKCKAGFNSYAELLAH
RKEHQVFRAKCLFPKCGRIFSQAYLLYDHEAQHYNTYTCKFTGCGKVYRSQSEMEKHQDGHSHPETGLPP
EDQLQPSGNDVNPDSGATAAGGRSENSIDKNLGSNRSADWEKNRAEPAVTKHGQISAAELRQANIPLSNG
LETRDNTTVLRTNEVAVSIKVSVNHGVEGDFGKQENLTMEGTGEPLITDVHKPGIGAGVQLCHPGFQEKK
GHECLNEAQNSLSNSESLKMDDLNPQSLERQVNTLMTFSVQNEAGLEDNSQICKFECGGDVKTSSSLYDL
PLKTLESITFVQSQPDLSSPLGSPSVPPKAPGQKFSCQVEGCTRTYNSSQSIGKHMKTAHPDQYAAFKLQ
RKTKKGQKSNNLNTPNHGKCVYFLPSQVSSSNHAFFTPQTKANGNPACSAQVQHVSPSIFPAHLASVSAP
LLPSVESVLSPNIPSQDKHGQDGILCSQMENLSNAPLPAQMEDLTKTVLPLNIDSGSDPFLPLPTENSSL
FSSPADSENNSVFSQLENSTNHYPSQTDGNINSSFLKGGSSENGVFPSQVSSADDFSSTSAQPSTPKKVK
KDRGRGPNGKERKPKHNKRAKWPAIIRDGKFICSRCYRAFTNPRSLGGHLSKRSYCKPLDGAEIAQELLQ
TNRQPSLLASMILSTSAVNMQQPQQSNFNPETCFKDPSFLQLLNVENRPTFLPSTFPRCDVSNFNASVSQ
EGSEIIKQALETAGIPSTFESAEMLSQVVPIGSVSDAAQVSAAGMPGPPVTPLLQTVCHPNTSPSNQNQT
PNSKTLKECNSLPLFTTNDLLLKTIENGLCSNSFSSSTEPPQNFTNNSAHVSVISGPQNTRSSHLNKKGN
SASKKRKKVAPAVSVSNTSQNVLPTDLPVGLPAKNLPVPDTNTRSDMTPDCEPRALVENLTQKLNNIDNH
LFITDVKENCKASLEPHTMLTPLTLKTENGDSRMMPLSSCTPVNSDLQISEDNVIQNFEKTLEIIKTAMN
SQILEVKSGSQGTGETTQNAQINYSMQLPSVNSIPDNKLPDASQCSSFLTVMPTKSEALHKEDQIQDILE
GLQNLKLENDTSAPASQSMLMNKSVALSPTPTKSTPNIVVQPVPEVIHVQLNDRVNKPFVCQNQGCNYSA
MTKDALFKHYGKIHQYTPEMILEIKKNQLKFAPFKCVVPSCTKTFTRNSNLRAHCQLVHHFTIEEMVKLK
IKRPYGRKSQSENLSSPQNNQVKKQPSMAEETKTESQPAFKVPAATGDAALANATVIPEKQLAEKKSPEK
PESSSQPVTSSAEQYNANLANLKTKGRKNKRHRKEKEEKREKNPVSQAFELPTKYSSYRPYCCVHQGCFA
AFTIQQNLILHYQAVHKSNLPTFSAEVEEESEAVKESEETEPKQSMKEFRCQVSDCSRIFQAITGLIQHY
MKLHEMTPEEIESMTAAVDVGKFPCDQLECKLSFTTYLSYVVHLEVDHGIGTRTSKAEEDGIYKCDCEGC
DRIYATRSNLLRHIFNKHNDKHKAHLIRPRKLTGQENISSKANQEKSKSKHRTTKPNRSGKDGMKMPKTK
RKKKSNLENKSAKVVQIEENKPYSLKRGKHVYSIKARNDALAECTSKFVTQYPCMIKGCTSVVTSESNII
RHYKCHKLSRAFTSQHRNILIVFKRYGNPQGKEISEQEDEKNDKKDPDSSVLEKNDNSEPAAAPQEEGRK
GEKDEMDELTELFITKLINEDSTNAENQGNTTLKGNNEFQEHDSCTSERQKPGNLKRVYKEKNTVQSKKR
KIDKTEPEVSLVVNNTRKEEEPAVAVQTTEEHPASFDWSSFKPMGFEASFLKFLEESAVKQKKNSDRDHS
NSGSKRGSHSSSRRHVDKAAVAGSSHVCSCKDSEIFVQFANPSKLQCSENVKIVLDKTLKDRSELVLKQL
QEMKPTVSLKKLEVLSNNPDRTVLKEISIGKATGRGQY

>Q9Z2U9_RAT

MFMATQITSGMKYLSSLNCVHRDLATRNCLVGKNYTIMIADFGMSRNLYSGDYYRIQGRTVLPIRRMSWE
SILLDKFTTAMMCGGFGVTLWETCTSRQEQPSSQLSDEQVIENTGEFFRDQGRQTYLPQPAACPDSVYKL
MLRCWRRETKHRPSFQGNTTFCFLQARS

>Q9Z2V0_MOUSE
MELQRTSSISGPLSPAYTGQVPYNYNQLEGRFKQLQDEREAVQKKTFTKWVNSHLARVSCRITDLYTDLR
DGRMLIKLLEVLSGERLPKPTKGRMRIHCLENVDKALQFLKEQRVHLENMGSHDIVDGNHRLTTLELLEV
RRQQEEEERKRRPPSPDPNTKVSEEAESQQWDTSKGDQVSQNGLPAEQGSPRVSYRSQTYQNYKNFNSRR
TASDHSWSGM

>HDAC6_MOUSE
MTSTGQDSSTRQRKSRHNPQSPLQESSATLKRGGKKCAVPHSSPNLAEVKKKGKMKKLSQPAEEDLVVGL
QGLDLNPETRVPVGTGLVFDEQLNDFHCLWDDSFPESPERLHAIREQLILEGLLGRCVSFQAWFAEKEEL
MLVHSLEYIDLMETTQYMNEGELRVLAETYDSVYLHPNSYSCACLATGSVLRLVDALMGAEIRNGMAVIR
PPGHHAQHNLMDGYCMFNHLAVAARYAQKKHRIQRVLIVDWDVHHGQGTQFIFDQDPSVLYFSIHRYEHG
RFWPHLKASNWSTIGFGQGQGYTINVPWNQTGMRDADYIAAFLHILLPVASEFQPQLVLVAAGFDALHGD
PKGEMAATPAGFAHLTHLLMGLAGGKLILSLEGGYNLRALAKGISASLHTLLGDPCPMLESCVVPCASAQ
ISIYCTLEALEPFWEVLERSVETQEEDEVEEAVLEEEEEEGGWEATALPMDTWPLLQNRTGLVYDEKMMS
HCNLWDNHHPETPQRILRIMCHLEEVGLAARCLILPARPALGSELLTCHSAEYVEHLRTTEKMKTRDLHR
EGANFDSIYICPSTFACAKLATGAACRLVEAVLSGEVLNGIAVVRPPGHHAEPNAACGFCFFNSVAVAAR
HAQIIAGRALRILIVDWDVHHGNGTQHIFEDDPSVLYVSLHRYDRGTFFPMGDEGASSQVGRDAGIGFTV
NVPWNGPRMGDADYLAAWHRLVLPIAYEFNPELVLISAGFDAAQGDPLGGCQVTPEGYAHLTHLLMGLAG
GRIILILEGGYNLASISESMAACTHSLLGDPPPQLTLLRPPQSGALVSISEVIQVHRKYWRSLRLSKMED
KEECSSSRLVVKKLPPTASPVSAKEMTTPKGKVPEESVRKTIAALPGKESTLGQAKSKMAKAVLAQGQSS
EQAAKGTTLDLATSKETVGGATTDLWASAAAPENFPNQTTSVEALGETEPTPPASHTNKQTTGASPLQGV
TAQQSLQLGVLSTLELSREAEEAHDSEEGLLGEAAGGQDMNSLMLTQGFGDFNTQDVFYAVTPLSWCPHL
MAVCPIPAAGLDVSQPCKTCGTVQENWVCLTCYQVYCSRYVNAHMVCHHEASEHPLVLSCVDLSTWCYVC
QAYVHQDDLQDVKNAAHQNKFGEDMPHSH

>Q9Z2X7_RAT
MVRLTSNFLLFCIIYIFKISCQFYYVCSSIHQSPQKEVSPQNRHLRLCPKECVLIFARIKENKLCTHSRF
FICRQTSLFII

>CORIN_MOUSE
MGRVSFSVRVSSVRRARCSCPGRCYLSCRVPPTTALRALNGLGCAGVPGETAGGAVGPGPLGTRGFLSGS
KFQAPGSWKDCFGAPPAPDVLRADRSVGEGCPQKLVTANLLRFLLLVLIPCICALIVLLAILLSFVGTLK
RVYFKSNDSEPLVTDGEARVPGVIPVNTVYYENTGAPSLPPSQSTPAWTPRAPSPEDQSHRNTSTCMNIT
HSQCQILPYHSTLAPLLPIVKNMDMEKFLKFFTYLHRLSCYQHILLFGCSLAFPECVVDGDDRHGLLPCR
SFCEAAKEGCESVLGMVNSSWPDSLRCSQFRDHTETNSSVRKSCFSLQQEHGKQSLCGGGESFLCTSGLC
VPKKLQCNGYNDCDDWSDEAHCNCSKDLFHCGTGKCLHYSLLCDGYDDCGDPSDEQNCDCNLTKEHRCGD
GRCIAAEWVCDGDHDCVDKSDEVNCSCHSQGLVECTSGQCIPSTFQCDGDEDCKDGSDEENCSDSQTPCP
EGEQGCFGSSCVESCAGSSLCDSDSSLSNCSQCEPITLELCMNLLYNHTHYPNYLGHRTQKEASISWESS
LFPALVQTNCYKYLMFFACTILVPKCDVNTGQRIPPCRLLCEHSKERCESVLGIVGLQWPEDTDCNQFPE
ESSDNQTCLLPNEDVEECSPSHFKCRSGRCVLGSRRCDGQADCDDDSDEENCGCKERALWECPFNKQCLK
HTLICDGFPDCPDSMDEKNCSFCQDNELECANHECVPRDLWCDGWVDCSDSSDEWGCVTLSKNGNSSSLL
TVHKSAKEHHVCADGWRETLSQLACKQMGLGEPSVTKLIPGQEGQQWLRLYPNWENLNGSTLQELLVYRH
SCPSRSEISLLCSKQDCGRRPAARMNKRILGGRTSRPGRWPWQCSLQSEPSGHICGCVLIAKKWVLTVAH
CFEGREDADVWKVVFGINNLDHPSGFMQTRFVKTILLHPRYSRAVVDYDISVVELSDDINETSYVRPVCL

PSPEEYLEPDTYCYITGWGHMGNKMPFKLQEGEVRIIPLEQCQSYFDMKTITNRMICAGYESGTVDSCMG
DSGGPLVCERPGGQWTLFGLTSWGSVCFSKVLGPGVYSNVSYFVGWIERQIYIQTFLQKKSQG

>Q9Z322_RAT
MMSYSERLGGPAVSPLPVRGRHMVHGAAFAYVPSPQVLHRIPGTTTYAISSLSPVALTEHSCPYGEVLEC
HDPLPAKLAQEEEQKPEPRLSQKLAQVGTKLLKVPLMLGFLYLFVCSLDVLSSAFQLAGGKVAGDIFKDN
AILSNPVAGLVVGILVTVLVQSSSTSTSIIVSMVPSGLLEVFFFFLLYPHLFMLNLFPCHELLFLQFLLG
YLFLLCNLLFWLWNNLFLLSEDHLDVAGGAHVWVNPAVSSVGSSAHLRSLVHLDVFNDQRVYIQTLKFSI
TLCIFKHVQQKFSTLFWPPSLCPAPLFGLGAPTDSTIIPPEWHTLLL

>KCNQ2_MOUSE
MVQKSRNGGVYPGTSGEKKLKVGFVGLDPGAPDSTRDGALLIAGSEAPKRGSVLSKPRTGGAGAGKPPKR
NAFYRKLQNFLYNVLERPRGWAFIYHAYVFLLVFSCLVLSVFSTIKEYEKSSEGALYILEIVTIVVFGVE
YFVRIWAAGCCCRYRGWRGRLKFARKPFCVIDIMVLIASIAVLAAGSQGNVFATSALRSLRFLQILRMIR
MDRRGGTWKLLGSVVYAHSKELVTAWYIGFLCLILASFLVYLAEKGENDHFDTYADALWWGLITLTTIGY
GDKYPQTWNGRLLAATFTLIGVSFFALPAGILGSGFALKVQEQHRPKHFEKRRNPAAGLIQSAWRFYATN
LSRTDLHSTWQYYERTVTVPMYRLIPPLNQLELLRNLKSKSGLTFRKEPQPEPSPSQKVSLKDRVFSSPR
GMAAKGKGSPQAQTVRRSPSADQSLDDSPSKVPKSWSFGDRSRTRQAFRIKGAASRQNSEEASLPGEDIV
EDNKSCNCEFVTEDLTPGLKVSIRAVCVMRFLVSKRKFKESLRPYDVMDVIEQYSAGHLDMLSRIKSLQS
RIDMIVGPPPPSTPRDKKYPTKGPTAPSRESPQYSPRVDHIVGRGPTITDKDRTKGPAETELPEDPSMMG
RLGKVEKQVLSMEKKLDFLVSIYTQRMGIPPAETEAYFGAKEPEPAPPYHSPEDSRDHADKHGCIIKIVR
STSSTGQRNYAAPPAIPPAQCPPSTSWQQSHQRHGTSPVGDHGSLVLRLERSAGMMSCH

>THIO_RICPR
MVNNVTDSSFKNEVLESDLPVMVDFWAEWCGPCKMLIPIIDEISKELQDKVKVLKMNIDENPKTPSEYGI
RSIPTIMLFKNGEQKDTKIGLQQKNSLLDWINKSI

>CSTA_HELPJ
MQKSLVSLAWVFVAILGAICLGVLALHKGESINTLWLVVASACIYSIGYRFYSHFIAYRVLKLDDSRATP
ACVRNDGKDFVPTDKAITFGHHFAAIAGAGPLVGPILAAQMGYLPSILWILIGSVLGGCVHDFVVLFASI
RRDGKSLGEMIKLEMGKFVGMIASLGILGIMLIIIAILAMVVVKALAHSPWGFFTIAMTIPIAILMGLYM
RFFRPHKILEVSVIGFILLIIAIYAGKYVSLDPKLASIFTFEAGSLAWMIMGYGFVASILPVWFLLAPRD
YLSTFLKIGVIGVLVVAIVFVAPPLQIPKITPFVDGSGPVFAGSVFPFLFITVACGTISGFHALISSGTT
PKMLAKESDARLVGYGSMVMESVVALMALVCAGILHPGLYFAINSPEVSIGKDIADAASVISSWGFSISA
EEIREMTKNIGESSILSRTGGAPTFAIGLAMIVYHILGDPSVMAFWYHFAILFEALFILTAVDAGTRTAR
FMIQDLLGNIYKPLGDLSSYKAGIFATLLCVAGWGYFLYQGTIDPKGGIYTLWPLFGVSNQMLAGMALLL
VTVVLFKMGRFKGAIISALPAVLILAITFYSGILKVMPKSNDSVLNNVSHVAQMQIIKEKIALTTDEKAL
KTLQKSFFNHAIDAILCVFFMLVALLVLIVSVRICSNAYFKNQIYPPLAETPYIKAA

>DHE4_HELPJ
MYVEKILQSLQKKYPYQKEFHQAVYEAITSLKPLLDSDKSYEKHAVLERLIEPEREIFFRVCWLDDNHQI
QVNRGCRVEFNSAIGPYKGGLRFHPSVNESVIKFLGFEQVLKNSLTTLAMGGAKGGSDFDPKEKSEHEIM
RFCQAFMNELYRHIGATTDVPAGDIGVGEREIGYLFGQYKKLVNRFEGVLTGKGLTYGGSLCRKEATGYG
CVYFAEEMLQERNSSLEGKVCSVSGSGNVAIYTIEKLLQIGAKPVTASDSNGMIYDKDGIDLELLKEIKE

ARRGRIKEYALEKTSAKYTPTENYPKGGNAIWHVPCFAAFPSATENELSVLDAKTLLSNGCKCVAEGANM
PSSNEAIELFLQAKISYGIGKAANAGGVSVSGLEMAQNASMHPWSFEVVDAKLHHIMKEIYKNVSQTAKE
FKDPTNFVLGANIAGFRKVASAMIAQGV

>Y944_HELPJ
MKEVIHSTLAPKAIGPYSQAIATNDLVFVSGQLGIDVSTGEFKGADIHSQTTQSMENIKAILKEAGLGMD
SVVKTTILLKSLDDFAVVNGIYGSYFKEPYPARATFQVAKLPKDALVEIEAIAIK

>SCOB_HELPJ
MREAIIKRAAKELKEGMYVNLGIGLPTLVANEVSGMNIVFQSENGLLGIGAYPLEGGVDADLINAGKETI
TVVPGASFFNSADSFAMIRGGHIDLAILGGMEVSQNGDLANWMIPKKLIKGMGGAMDLVHGAKKVIVIME
HCNKYGESKVKKECSLPLTGKGVVHQLITDLAVFEFSNNAMELVELQEGVSLDQVKEKTEAEFEVHL

>Q9ZNT0_ARATH
MYSNFKEQAIEYVKQAVHEDNAGNYNKAFPLYMNALEYFKTHLKYEKNPKIREAITQKFTEYLRRAEEIR
AVLDEGGSGPGSNGDAAVATRPKTKPKDGEGGGKDGEDPEQSKLRAGLNSAIVREKPNIKWSDVAGLESA
KQALQEAVILPVKFPQFFTGKRRPWRAFLLYGPPGTGKSYLAKAVATEADSTFFSVSSSDLVSKWMGESE
KLVSNLFEMARESAPSIIFVDEIDSLCGTRGEGNESEASRRIKTELLVQMQGVGHNDEKVLVLAATNTPY
ALDQAIRRRFDKRIYIPLPEAKARQHMFKVHLGDTPHNLTEPDFEYLGQKTEGFSGSDVSVCVKDVLFEP
VRKTQDAMFFFKSPDGTWMPCGPRHPGAIQTTMQDLATKGLAEKIIPPPITRTDFEKVLARQRPTVSKSD
LDVHERFTQEFGEEG

>APC10_ARATH
MATESSESEEEGKISGGNYKLIIDDDLREMGKNAAWSVSSCKPGNGVTTLRDDNLETYWQSDGLQPHLIN
IQFQKKVKLQLVVLYVDFKLDESYTPSKISIRAGDGFHNLKEIKSVELVKPTGWVCLSLSGTDPRETFVN
TFMLQIAILSNHLNGRDTHIRQIKVYGPRPNPIPHQPFQFTSMEFLTYSTLR

>VATF_ARATH
MAGRATIPARNSALIAMIADEDTVVGFLMAGVGNVDIRRKTNYLIVDSKTTVRQIEDAFKEFSARDDIAI
ILLSQYIANMIRFLVDSYNKPVPAILEIPSKDHPYDPAHDSVLSRVKYLFSAESVSQR

>RABD1_ARATH
MSNEYDYLFKLLLIGDSSVGKSCLLLRFADDAYIDSYISTIGVDFKIRTIEQDGKTIKLQIWDTAGQERF
RTITSSYYRGAHGIIIVYDCTEMESFNNVKQWLSEIDRYANESVCKLLIGNKNDMVESKVVSTETGRALA
DELGIPFLETSAKDSINVEQAFLTIAGEIKKKMGSQTNANKTSGPGTVQMKGQPIQQNNGGCCGQ

>Q9ZUX0_ARATH
MPVSVHSVIATNLATTLSSNYRNVSRRRTISTSVNCSVEITEADRSVKLKNGNDSLEICRVLNGMWQTSG
GWGKIDRNDAVDSMLRYADAGLSTFDMADHYGPAEDLYGIFINRVRRERPPEYLEKIKGLTKWVPPPIKM
TSSYVRQNIDISRKRMDVAALDMLQFHWWDYANDGYLDALKHLTDLKEEGKIKTVALTNFDTERLQKILE
NGIPVVSNQVQHSIVDMRPQQRMAQLCELTGVKLITYGTVMGGLLSEKFLDTNLTIPFAGPRLNTPSLQK
YKRMVDAWGGWNLFQGLLRTMKTISTKHGVSIPTVAVRYVLDQQGVGGSMIGVRLGLAEHIQDANAIFSL
VLDEEDVNSIQEVTKKGKDLLQVIGDCGDEYRRI

>Y1461_ARATH
MGCVFGREAATTTTAEAKQAKSSKASSGVVVVGESSVTKSNGVIADDVEKKKNEEANGDKERKSSKGDRR
RSTKPNPRLSNPSKHWRGEQVAAGWPSWLSDACGEALNGWVPRKADTFEKIDKIGQGTYSNVYKAKDMLT
GKIVALKKVRFDNLEPESVKFMAREILVLRRLDHPNVVKLEGLVTSRMSCSLYLVFQYMDHDLAGLASSP
VVKFSESEVKCLMRQLISGLEHCHSRGVLHRDIKGSNLLIDDGGVLKIADFGLATIFDPNHKRPMTSRVV
TLWYRAPELLLGATDYGVGIDLWSAGCILAELLAGRPIMPGRTEVEQLHKIYKLCGSPSEDYWKKGKFTH
GAIYKPREPYKRSIRETFKDFPPSSLPLIDALLSIEPEDRQTASAALKSEFFTSEPYACEPADLPKYPPS
KEIDAKRRDEETRRQRAASKAQGDGARKNRHRDRSNRALPAPEANAELQSNVDRRRLITHANAKSKSEKF
PPPHQDGGAMGVPLGASQHIDPTFIPRDMVPSFTSSSFNFSKDEPPTQVQTWSGPLGHPITGVSRKKKDN
TKSSKGKRAVVA

>PCNA2_ARATH
MLELRLVQGSLLKKVLEAVKDLVNDANFDCSTTGFSLQAMDSSHVALVSLLLRSEGFEHYRCDRNLSMGM
NLGNMSKMLKCAGNDDIITIKADDGSDTVTFMFESPTQDKIADFEMKLMDIDSEHLGIPDAEYHSIVRMP
SGEFSRICKDLSSIGDTVVISVTKEGVKFSTAGDIGTANIVLRQNTTVDKPEDAIVIEMNEPVSLSFALR
YMNSFTKATPLSETVTISLSSELPVVVEYKVAEMGYIRYYLAPKIEEEEDTKPE

>NU5M_RHISA
MFINWTMMLISFSIVFLYMFFMTFYFNIFFIFEYNLMSILSFEYKFYILLDWMSCMFSFTVLMISSMVLW
YSHSYMSSDKNKTSFCWMVLMFILSMLLLVLMPNVFMLILGWDGLGLTSYCLVIFYQSSNSYNSGMMTII
SNRVGDVMVIMMIIFAINFNSFELTSIKSFELIWGLLIIIAGLTKSAQIPFSAWLPAAMAAPTPVSALVH
SSTLVTAGVYLLIRFDLLFNNNIFSAFLMKISLMTMIMSGMNAFFENDLKKIIAFSTLSQLSIMMLTLSL
SLTNLSFFHLIVHAIFKSMLFLCAGFVIHNLMGNQDIRFLSDFFKFSPLILSCMMIGMFSLMGFPFIGGF
YSKDVIMEFFFLKSNNMIEMNMFIVGIIFTFLYNFRLFYMLLLKGTLFNMMSKDSINIFMNYPIFNLTVY
LLITSNLMSWLLLPEYTMIFLSFTQKLMLLFLVPFCTFMFMILLKFIKFFPYLKLNFLMTMWNLSELTSF
IMLSNNKFLLKMSINDWTWLEMYGPLGIKSKIEHNYNFSMTKEINVITIAFSLTILMMIIVY

>NU4LM_SQUAC
MSPVYFSFSSAFILGLMGLAFNRSHLLSALLCLEGMMLSLFIAIALWSMTLNSTSCSITPMILLTFSACE
ASAGLALLVAATRTHGSDHLQNLNLLQC

>NU6M_CANFA
MMTYIVFILSIVFVMSFVGFATKPSPIYGGLVLIISGGIGCAIVLNFGGSFLGLMVFLIYLGGMLVVFGY
TTAMATEQYPEVWVSNKAVLAAFITGLLSELLTACYILKDDEVEVVLKFNGMGDWVIYDTGDSGFFSEEA
MGIAALYSYGTWLVVVTGWSLLIGVLVIMEVTRGN

>A3LT2_RAT
MALEGLRAKKRLLWRLFLSAFGLLGLYHYWFKIFRLFEVFIPMGICPMAIMPLLKDNFTGVLRHWARPEV
LTCTSWGAPIIWDETFDPHVAERARRQNLTIGLTVFAVGRYLEKYLEHFLVSAEQYFMVGQNVVYYVFT
DRPEAVPHVALGQGRLLRVKPVRREKRWQDVSMARMLTLHEALGGQLGREADYVFCLDVDQYFSGNFGPE
VLADLVAQLHAWHFRWPRWMLPYERDKRSAAALSLSEGDFYYHAAVFGGSVAALLKLTAHCATGQQLDRE
HGIEARWHDESHLNKFFWLSKPTKLLSPEFCWAEEIGWRPEIHHPRLIWAPKEYALVRT

>A0A504_MOUSE

MQSLRTEQTQGLLPRDSRAWEKPCHSTFPKDWEAVEVGASSCDSDEKDLSSQETGLSQEWSSVEEDDESE
DSQGFVEWSKAPQQTTIVLVVCVLFLFLVLTGMPMMLHI

>A0A5F3_MOUSE
MLTYPDFADLILVDGNVGVESGHSDLSHVGHSGATPVLVLGCDSEVSQALGIDPRTERSCQQVVAGALEV
KAPAIEGVVVRVTPQPTRGIIVGHTHTLTTLNFAGLLWSAGDTMAWVCEERRKYREGLPTTSMSAPAICL
SVLGMSSLMVAAAVTRP

>A0A7V1_PAROL
MRSLGTLGVALHASLLVLLAQGFQRSRDGVYVRPLHRLPVSDRSTGHHLPTYMVHLYRNFKANLSGPLDT
TEQDAARQADIVKSVMAKSFTYRHRRWVATFDLHTLLADKQIQAAELRIRLPRTPSASNISVEVYHQQGQ
ACHMHQGCQEQQLVGLLAESSLVTSSQSWKVFNMTSLLLDWLRQKSTTRIQHKRVSRRKKTVKSNRGLSL
PDQPVYYVANPGRDQDVSDRALLVIFSRTGSGKNSKAKASLLHTAEQSKFLSPAEIKKARWPKRRRTKRGH
REPIARGPQVSKRGSEKTLCRKVDMHVDFNQIGWGSWIIFPKRYNAFRCEGSCPGPLGEDLNPTNHAYMQ
SLLKHYHPDRVAPPCCAPTKMSPLSMLYYENGEMLLRHHEDMIVDECGCQ

>A0A8M4_9CRUS
MRFLKKLKRVAVLVAQHVTRVVKVIWSFLATQEKADDRKFLWNENRLGDGNILPKAKPFSQIPGPLPIPF
FGSQWLYSWIGPYFLDKLHLANEDKYFKYGPIVKEHYLWNFPIVHLYDKNDIETVLKYSSKYPIRPGLEA
QIFYRNSRPDRYKSVGLVNVQGLEWHHLRSKLTTQLASTALGDHTVKQLSIISEELIEKIREERDAESLI
EGFEKYVVYSCSLEVIFAILLGRRLGALNKSSIPPIAERLMFATENLFEVSHETMYGLPWWKYFPTKSYRK
LAECENIIYDVFSDLVKEALNSDEATGTQSPVLNQILTAEGVDTRDKIVSLIDLVAAGIETTGNATLFLL
HNILNNPEIKVRVYEELDRVLPSPEDTITPQLLLELKYLRACIVESLRMTPVAPNVARILEKPFTFQGYH
VPAGTLVVCETWVASLQEENYLNAKSFIPERWLDSDKTNRYPFLAVPFGVGRRMCPGKRIAENEMLIITA
KLLRAFDISFHKPLEQVYKFLISPKGPINVILRERY

>A0A8M5_9CRUS
MDVTSGGESSVWISSFSVYTVTTILVTLVVLAVVKRYNFMQRCNKVLGSPTDIPLFGGGSLIFVPPEEIM
NLLLLLHVVFGRLSPSGIIRAWIGPLPMFFATTAEAVEAVLSSNKIITKSREYDFLHPWLNTGLLTSTGS
KWQTRRKLLTPAFHFKILEDFVHVFNEQSLILVNKLNQAVAKDKDLNIFPFVTLCTLDIICETAMGRNVE
AQSKTDSAYVQAVYNMSQLIQHRQVRFYLWLDWMFKLSSHWPEQRKTLGILHGFTNKVIQERKAEHQQRS
SDIAEPSKDVTEDAVFSKRRLAFLDLLIEFSQGGTVLSASDIREEVDTFMFEGHDTTSAAITWSIFLIGS
HPEVQEMVNEELDRVFGDSDRPATMADLSELKYLECCVKEALRLYPSVPIISRTCVEDTVIGGDEIPAGT
SVSICSYYLHRDPKYFPDPELYQPKRFLAEHAERRHPYSYVPFSAGPRNCIGQRFALMEEKAVLSAILRN
FHVQSLDKREEIILLAELILRPRDGIRVRLEPKKKQ

>A0A9I9_MARSA
MVVLVICGGGNGAHVLAGIASSNPNADVRVLTLYADEAERWTKAMEGNDFVVTVNNQDKSQTILKNKPRL
VTKDPALAANKADMIVFTVPAFAHRQYLDALKPHIRPGTVLVGLPGQSGFEFEVWTAWGDLAKQCSIMSF
ESLPWACRFTEFGRAATVIGTKENLAGAAWYGSVPPKADPTLVLQGCLGPHPVLLTRGALLGITLMATNG
YIHPSILFGRWHKWDGNPVNEPPLFYNGLDEFSAQTMSSVSDEILAVASSLMKQRPQVDLTNVAHIWQWY
LRVYADDIGDKTSLFTTIRTNAAYSGLTHPTTKTDDGKFVPDFKHRYLMEDIPFGLLVSKGIAEVAGVPT
PTIDSVISWAQQKMGKEYIVNGKLAGKDVSSTRCPQRYGLTTVDAILGL

>A0A9P7_TAKRU
MASNSLFSSTSPLICWDPVVKHRPMPSPGHHRMENQDVGTIQQPRMGIPRGLVQTDSPNFLCTSLPQHWR
CNKTLPRPFTVFALGNDVPDGVVVTVMAGNEENSSAELRNATATMKQGFAHFNDLRFIGRSGRGKSFTVS
INVLMSPPQIATLQKAIKVTVDGQRQPRRQRQKEVKSGAFRPGTCSTASADCRSFSSSLWTSEPSFLGQV
TSLSSPFTPSPRMHHLPTFSYATQPTTYTSYLSSPPPPPLNHSSSFQPGSFYYGQNQQFYSMAEDRNVVT
ALTNYIEGACLSMRGEEPVWRPY

>A0A9Q6_9ASCI
MEQIKKKMTSLKAQAEMAEERADQLATDLKAKEQENEDLLEENASLQRKMASIQDESDKSQDNYDKIMQE
LNEKRKEIQDLEEINKSMENKISIAEDKIEDLEVKLENTTRDLDAIRQEKEESIRSLRSLENSEANAAMQ
LELHEDRLKEATAAAQASDSKYEEIHRKYCILEVENDKNEDALELLTREKIELNAQIDSLNEQCQSYRHM
ENQFTDSSDKNEEKTRKFMDTIRDLENELDEKKAKCKQQAIEIETLEADLEKAEDERDDAKKELEHTLSE
LSEI

>A0A9Q7_9ASCI
MLKFQHKNALHTLVSCMTGRPCTKCDPGICPGFALHEWRKVCAHCKCGVDYHVGDDTNQTSENNSVDLPA
ISNKFEANDIMVGHGHNATPNNNNIDHYDRNQRSHKTGTRGLFNDTDSGCVLEECAWVPPGLSPKQAQAY
FSKLPEDRIPFIDSIGEKHRIRQLLQQLPPHDNEVRYCNDLGEEEKHELKIFSEQRKTEALGRGTARPFP
PNIPPAICENCGYHINGGDIAVFASRAGCAVCWHPNCFVCSVCDELLVDLIYFHQDGQLYCGRHHAETLK
PRCSACDEIIFADECTEAEGRHWHMNHFCCFECEVVLGGQRYIMRDGKPYCTSCFEQTYAEYCDTCGDII
GLDAGQMQYEGQHWHATDRCFSCARCKKSLLERPFLPKHGQIFCSKACSHGEDQLHSESDSQYEKATTPV
SHNVRHSLNLENLSLHEKNWDNSSSVEKSQDSDLPVDLNDLYPSDAIVASQHNKCLRLAKNGRIDDYERT
KNTKNVASESAAQPVASFPQNTYNSTDSSGYNSSSTIDAMDHKSRGRNGQTKVVPSDTLNSTCSTASQAT
TCSAMSNSDKAFCMGGHVPASEFVPYRVNCSESNKNNNIVQKRIESILSSTQDTQNSSVWQTRRPSVEKI
SAFKSVSDSNNILKHPIINGPIPAPRAKYPSKQSDWSDANQSLSKNEPARRVSDITPPSNYFKDTPRGAT
RRLSENNIGLRLNTTSSTGSNNSQPRGILKKSRSQVESGNISDLHTPTDETPVSPIFPEPNTPPFNRAGR
LNQSARFPNAPGSPESKSTTNYFSECEKKTCSKKLRRTKSTDFTSKATGASKKKRQARFANDVPDEHDSW
CSTCTSSSDDSDYERWDKFDDNVSMTSSPHHQRRSTEFNLTHLQNLQQQAKLRYGVQSTSALPKYHHSRH
SRRHHKKNCVIM

>A0A9Q9_9ASCI
MLIANPIKDFYEKILHERVLVLVSLDVDSLTACKILQSLFKCDHVQYTLVPVATKVDLKNAFDEHKEQYQ
HVIMLNCGGNIDVLDFLQPPEETVIYIADSHRPIDLVNFYCDQQVYVIMKHDDTEEMSAIPEYDALYHED
DEEPASDEESSEEQVAKRPRLDEEELKKRRRRREWEEKRKEILFDYEEFSYYGTSTAFLLFELAWKMSKD
NNDLLWWGITGVTDQYQNKKIGRDKYVSSILDLQGHMSRLNHRYEDQDNTTSVNCMKINFEHDLNIAMYR
HWSLFESLRHSICSATAFKLWTSNGMKRLHQFLAEMGIPLTQCKQTYNFMESGIREKLSGLMEESAQKFG
LDNIKFQSFSAQCGFNHKVCASDVVFAVSSILEDFSSSEDANSYVNFVKALDSLAKISTKQLQSGIDQAK
VQLTSMVSNVSTAIEIGQVISYGPFLYTFIKEGMRDYKNFGGAIFLNAFTHHLLHCYLRSLGKSKRDRAK
NLPLVVCASHDTDNGVCIITGVPPLAEDSKKNLFGRAFTHAAERINSRIIDEYFDPCTITLKSEDRSKFL
DALIAIMS

>A0A9R0_9ASCI
MVMEIGMDTTDSGQRVENNMVTQILVQSSGIGQASGTATTSIPRSHNNVMSHHNFVSSSQISSHDDTVEY
GRLIVLGTNGCLPGGDRGRRKSCFTLRRRSLANGVKPSDKHQVVERASHSEEFSSKEHHSVSYTLPRTVV

VVPYVRDSKTDMFQIGRSTENPIDFVVMDILPGATIPSNDTKHAQPQQSTISRFSCRIVCDRDPPYSARI
YAAGFDTSMNIILGEKAPKWQDKTNRMDGLTTNGILLMKPNKSDGWRETSVGGGAYNLRESRSAQVPGAK
VEDCDNVLEDGTLIDLCGATLLWRSSSTLKLMPTQRHIDQIIKDINLERPQCPVGLTTLAFPTRSRANKP
TEKQPWVYLACGHIHGWIDWGWRGEERMCPLCRSVGKYVPLWMGNEPAFYKDHGPPTHCFVPCGHVCSEA
TAKYWAQTPLPHGTQAFNAACPFCATEIEGTPGYVKLIWQQPLDG

>A0A9R2_9ASCI
MIQNKKKMLIESVICCILSIIIAANRIQATSVTYQPFEENFETGVTAYWNNDWENAIVEIEKSLVMHRLF
ESGIYDCYMKCKDTRNIENTDRHSQFADIKFIISKASCSRSCLDEIFGDARLITQVSEHIKHLFHIKRPY
VYLQFAYFQVGNIEQAVKTAHTYWLRHVGDDVIEDSIEYYRNLTEVKPEFFIDLEASEHIYVYDTAVEIY
LDEDYEAAIPLFMRALEAYYTASDKCNALCEGEHVFDYDYPDVPEFHMQTADHYIQVTECSLECVKKIAT
DSSGVHISDFLPLHYHYLQFAIYKEGNISAALSNAKTYLLFHPEDEVMVNNVNLLSKKVDSKTEAIPEAV
LYKKRIDGQIALLRYSYQAFGYEYRTNLLTPFADLTENEDGIDEFFSGHTLQKPNILPSNPEEQFYQSPD
EKQKDIIEKKSMEEHILNELEEQRSQQKVAKPDNDVDVSNVSLGEALVDSKDMVTIRDAANETPTAEGNL
LFEDYVLVANSTQLNGQERFLVDNLITQNDCDDLINLELSRGLFGDGYQDKKYPHTEYESFQGISIYTAV
ELAKKSEIPLRIARLYFDTSEKVRAQVQEYFHLPNLYFDYTHLVCRTALPKSSSEREDLSHPVHADNCLL
QDNGECLKKPPAYVWRDYSAILYLNNKFEGGNLIFVDSTAKRISAQVEPKCGRMAAFCAGKECFHGVKPV
SKGQRCAMALWFTTKKEKQEVQRQIAEETLASLENSKDEL

>A0A9R3_9ASCI
MYEITRWYYFDDNYLYDIINDEPKVLLIGQWAEEITAVTQVALEYINTNEILGRTWKIIKRENGYLKSDV
FQGREYIFDIKAESMQDGIVRSFRIQLLHPFNHQNEVEVVSYVQNIQKPVHIIVPLTGVLNNAKNIMLVF
QQTAKLKEGLCLTLVLFKKRGIYQQLFDPNIWEYLENFKQNNKEMCFHMREINSDYNFFDGIKQSIEEIE
ENNIILTLKDDIEFNSDAIENCQNLALQNIRVYFPLSFSQFNPKNIEDGMPFGKPKNVDKKSFTKFTGYW
MHNMHDFICAYSDDLKSILSKTESLVYEENPNNIFIEQSENGDNVYNKFLLANYKVISSVEPGLLRLYNN
INCTNIRNKLDRRKCKIQMMETSGSKQELNILYLNENASKLR

>A0A9R4_9ASCI
MDQLKKDVNEIVGLDILIKAADYIETCENEKRHRESDHGYASTYRYIDDNEPLHKRVKITKKNQSRTSHN
ELEKNRRAHLRNCLDNLKAIVPLGQDASRHTTLGLLNKATSLIEDLERQQKIYNKQKSDLSNHNEQLKHL
LLGLELSSRIRHDSTNSIGLSDLSTDSDKEEIIDVTECSSSGDELIQELIGMKTMPAEMVIMS

>A0A9S0_CAEEL
MLMSIREDVVEATSSIVPEKIHSDARNFSLVTSSSQVRSPLYLKSLDADGEDNSTVEKQITHSKKVADTE
LDRSLECLKENENDVKTNGEVELSAVEDALIQVVPEVSENQNPRKIGPYDFDQHANFLNS

>A0A9S1_CAEEL
MRRFLLLFHVFFAFSHSFPVRNDDRGVLPNIDALFCTNKDLRQVVFNALQNITNPKAIAVDQVQAAIQTA
LIPNFASSGGTWLVSATSYHRVNGYVDDRATSMDTFCAVNDINLIRSEQNLQKKGVQGANTLDTFPRRRI
LFGQGASKKMKKLELFSLFTHKDIYPSRPFRYYLST

>A0A9S2_CAEEL
MSGGLGFAIDRGGTFTDVIVFKPNSEVEVLKVLSVDPANYTDAPTEAIRQVLEREGGKKIPRGVALPTDS
ISWIRMGTTVATNALLERKGERIGLLITKGFKDLLFIGNQARPKIFDFNIQIPEVLYEDVVEVDERVLIL

EQTKELLDENAQIETTINGLQIVVEKKVDELELKEKLGELKEKGVKSVAILFLHSFIYPNHEKEAGDIAK
KFGFDYVSLSHQVMPMIKVVPRGFTVCADAYLTPKIMEYLDGFKAGFSDISSVRVNFMQSDGGLCEMDKF
RGSRAILSGPAGGVVGIASTAYKASDKKPVIGFDMGGTSTDVCRYSGYLEHVMETTTAGITIQAPQLDIR
TVAAGGGSRLFFRDGLLIVGPESASAHPGPVALGFLAVANEEMCRPIRTLTQSRGFNPSEHVLACFGGAG
GQHACAVAKVLGISQVRIHKYASLLSAYGIALADVVDESQTPAQVVYEEENFPKLYSQFMDLRSKSLAGL
KTQGFTESQIETKYFMHMRYEKTDTAIMISCDIGKSEDLASFRDEFRTTYRREFGFVLEDRNIIIDDVRI
RTRGKSGCHVEKTIKQAAEDQRQAVLKSVSSVYFENLKFVETGVYLLEEMLAGQIINGPALLIDKNSTIV
IEPSSTVTITEHGNVELQIGNDVEKDLTTEVDPIRLAIFSNRFMSIAEQMGRILQRTAISTNIKERLDFS
CALFNPEGGLIANAPHIPVHLGGMQYTVKFQIDHRGIENIKEGDVYLANHPTAGGCHLPDFTVITPVFFK
GHKTPVFFVANRGHHADIGGLVPGSMPPNAHHIDQEGASFISFKLVDEGVFQEQRLIDALKAPGKVPGCS
AARNISDNIADLNAQIAANRKGIQLVTSLIEEYSLDVVHAYMQHIQNTAELCVREMLKKVGRKVLEKTGK
SQLSGEDFMDDGTVIKLTVDIDSEEGTAIFDFTGTGPESYSSCNAPRAVTMSAVTYCLRCLVEKDIPLNN
GCLAPIQIKIPEGTLLSPSETAPVVAGNVLTSQRLCDVIFKTFDIVAASQGCMNNLVFGDETCGYYETIA
GGAGAGNGFHGRSGVHTHMTNTRITDPEILENRFPVILREWKLRDGSGGSGKWEGGDGVVRQLEFTRKLT
LSLLTERRAFQPYGLHGGQPGQRGLNLLKRGGRAVNIGSKASFEIQKGDILCIETPGGGGYGAPENS

>A0A9S4_CAEEL
MLEALASLLASNKYFFDVNEPSWLDCKAFAVLAQFKYTPLQNEARVKQFMKDRTPNLMTFVTRMKEEFWS
DWCTTSED

>A0A9S5_CAEEL
MNRKTALLTTLPRTVTVPYYAPSCLFLETFLRSKLIPYETTQCSLYNVLPREHLYPLIDVDGYFFKNLME
GLDYLLAKYGKSLDSGLTPKERAQALALSALLDELTWMLAYSRGQDFTWLRDDRKIIEDFGLVQLYFWRN
WIVPQMQKRVDY

>A0A9S6_CAEEL
MQFTYFPRVSIKLYMLALLILLLAAQQITPQCQQCGNRRIVQQYRSYPQKKFNFNFEQFFSRFPGFTSPQ
RQPPSQPRVFNSYGQEAETFGSAIGKSIGRRIQQAMAAQDPAFDIEFAQPSSPASTSSTAYSPPPATSST
TYGSSIPSTYPPSTFAPSAYPTSSSWGSTHPPEDLPTVQQAFQQQQQSYGTVQPQVSLAQSLIGNSNGFG
GLPGLLPPPSNGDCPWCSGYRGKRGPDTEKEKKNEKI

>A0A9S7_CAEEL
MPGLIGLGLLILLGFHVTYSTQCDCNSSCAIYTSPVQCVRCCTHYVKRRSLPLHPAHRKRQFLLTESTKI
KHPIWRRPVEPMVEESLPRLIRLLLRKPL

>A0AAB8_CAEEL
MNGNGENGTDEKQKLLSVVGVVSEEVVEMNRVSSARFQVSKTDEDEKTSDDNLRKLKSGSEPPPPTQNHE
AVTLVTRKMSATGRFMVTSDTNPGQLVAEVADELGIDVPIDPPAEKSPASRAKGVHFSVGDKDSGSMSED
GDEKRNITHENQTTFNMKSWRNMKTIEHPPIIDFYRNSIDTDGVFNRPSMAQLIHGKEHKEQDLGFEELN
AQHHHLEDSPVHETKNEYRMEKMHAPPSQNRVKFGWIQGVFVRCLLNIFGVMLYLRVSWVSGQAGVGLGS
CIVLLASVVTTITALSTCAICTNGDVKGGGAYFLISRSLGPEFGGSIGIIFSIANAVGAAMYIVGFAESF
RDVLIDYNIGTIFDGGLWDVRVIGFVTCVILMGIVFIGSEFESKMQLGLLVILLVSIANYVFGSFFPPNE
IAMNRGATGYSLDTLQSNFMPHFTENNTFFSVFSVYFPAATGIMAGANISGDLANPQQAIPLGTLLAILV
TTIVYLLTVWMTGSTVVAFSNGTEPAVFNNSYFVPPDCTPDCPFGLVSYYQVVEMSSFWGPLITAGIFAA

TLSSALASLVSAPKVFQAVCKDRLFPRIDYFAKTYGKNEEPKRAYVLGFFLAMGIVAIGDLNVIAPIISN
FFLGSYALINYACFDQSFADSPGFRPGFTYYNMWISLVGALLCVFVMFIIDWFSALVTFFCFGAIFMYLL
HRKPDVNWGSSTQAHSYKNALSAMIKLSTTEEHVKNYRPQVLVLSGNPASRSCLVDFANNITKGSSLLVC
GQVVPYEPSDRVYMVMKRLDENINVWLRKRHLKAFYRAVANSNFRKGAQSLIQLTGIAKMKPNIVLMGFK
SNWYKGGPTETNLNEMNEYFGTIQDVFDWNMAMCVLRNGQVGLDFSEAMRNLNLIEPNRLNVPPIEEKEK
EKEKTNENSPETVHLIEKDETARTDKSGDDGSSSISINETYVSDENQEDADGDDDNDNDGEESGADDEHQ
KEDDVELGVIDEQEKRHFSLRRRGSRRHTVEQKALLSSIQRFNRKVKKGTIDVWWLYDDGGLTLLIPHLL
AIPKSYLEGARLRIFTISTSSRTMEQEQRGMAALLSKFRIDYSDVYVIADIGKKPRQETMNTWQSVIDPF
IAQDGSCPIGMTTKSELSAQRDKTYRQLRAGELLNEHSINADLIVMTLPVPRKGMVSSSLYMSWLEVMTR
NLPPVLLVRGNQQSVLTFYS

>A0AAB9_CAEEL
MEALPIMPMLAPQSRSSTLSYDNIPNFDFRLRMKEYILLTFNADPHDYDSAFDELTQMKFEANVPTPSVE
QTLKLKRYYGQLCMMQKRFPMGAGEQLETPFAWHDGLIDIRSAQSEVTICDIEFEKASVMFNIGACHAQY
AAEQTRDTQDCIKAAFMHFQYAAYAFEQLNSFRNSDIFYPSVDLDANVISFYYKVMIAQAQECLVQKSLL
DNRSAIVIAKLSLWLQEAYDSAAKIVDEWSVNIPESVQRYYAKICSLKSAMYAVIAYMSFGDNLEKEDKK
MGWRLQYYNIANKYMELLSSNHSKMRERYPELFVTSSFLFDVISAKQRNAEKENDFIYHDRVPKQEDAID
AVQKDGGGAMCKVKTLSFDPLDPSVCGCDLFGKLLPTFVQDAVKKYSDDKDQALREIKECVRSYDEHLNY
QLQLAEFDKLRFMLNEGKRSREAWFEISEDLMKRNADMTSYPDCVPNLIDKMGESSDTARVAEAKLNTLL
SKLRAIDLQKSSIRSDEGFILIQKELERLAEHLEQAKAHNVSLNKAIAQHSANLQLLTLPCREMWMKIVP
PEQQGEMRNGSSPEELQVRKMIEKVMEMQAQRRKLVEQFEADLKADNISNKLMGTNERGAEEIMKSELTK
HTNIQQLIRLNITAQDAILRAFADANADFFEERLAMSTKKEEYEKRVSELCASYEVYRDVSKKVDEGEQF
YRQLMARCDQFAIPVHAMEEQYREEMEKKERAKKEAEHHLNQIRMSRDAQNALMDFGGGRGGGAPGARAQ
QQAYQPSRGAAGAGAGAGAPRGPRLGDFMDSYRARKQAPQAQIPEDQGPPSPTPSSICDFPVQSQRSQRFGA
FEGAQGAPGAQRGYQGPHSPVPGALTGSQASGPFGAPPGAPHGYGAPLQAPPQAPYGYDPSRYQDPSTYP
APPPILPGSAAYEHAIRPYPGDTGSYQVPRIVPGAQYGAQAGSGAYFGAQFGAQGAQGAHFGAQGVQFGA
QGAQGAHFGAQGAQGAQFGAQGAQGEQFGAQGAQGAHFGAQGAQGAHFGAQGAQGTQFGAQGAQGPPPAS
YGAPTPPQASYGPAPGAHGYQNGAQGPPGAEVGAQGPPGAHFGAHGASAPPPTSYGAPTPQRPPQASYGA
APGAQGPPGGQFEAHGAAALPPTSHGAPTPQGPFGAAPGAQFGAQGPYGQQGARYEAQKSPGAAIFGAPG
APPQHQGSFGAQFGVPPPQNSAPGAQFGAKPEASSHAPTPPPQPHPSYQAPAPPPALSVFQHSPQGAPIT
APPPASSHHEHIAAPQARFTPTPGAPSPWHATPAELKTPWNTTPQYHAPPGAQAAAPEAPAAPAPGAPAA
RSNVDLLSDLLGDFNLPPQPIQPTVHSTQQLQHQAPQQQEMSDFRIAAATLNPPPESVKPVKPAAAVQPM
PQMTAQQVPAHGAQETPILKSSELSSSASTSSSITELSDPSKFQLGVGNVQKLEKRMLHQSFRNQGPPPP
MNQSDPLNQIDAFSSFMTPRHQ

>A0AAC0_CAEEL
MSDFDSEERPTFDGWSCRLGFMLLTFNLLVITGIATGISNMIRICVDPPECKTGEVYVIIALVIIFLGFI
GFLIGYNFYNWTKKMYLPLLTLNRASIVTFGFLLLIYQAIDSNVFLLYLYSISWGLIAACYFNSLCAQLV
SSIIPEERLIILNGYLCIFFSGAAGFGLDRFTVKLKYGGFKLGSFILLGPLVMSYLITYIVSKNERKEGH
LTHCNSAYRLPHKLTNSLANIAAAQIARHIEDGHYKDPNIFQELDTKSHEAIFSKLLLLNPKAYKFIAKA
TQIQESVVIGKRVHAQHYYARNIDIMTSLTSVLYGKSLAKIHSLDIGECRSQLTPGWIGTMLPSLQSLDI
SNKPLSKEDFSQLCNFFQNLQKLNISDTCVKKLQGISKLKNLKVLSMRNLQFRTATDMLDLFKLKGLVAL
DVSRDIAKANIKTIRQFVECGNVLPSLTFLDASGTDINQDLLDSLEYQPNLQKILAIGIQIIEDSNTPKV
LNFATLETTLKALAHYTNLKNSAATRRCLASIKSQFKKNHDDGERYDVVNCLKHVIEAIETFSPDGSLDV

NTMWSGHTYIQGVMCLTEISINKSHLFHQIDAKLVLEMLLVASERLTILKSSLSQHPCQEVWEAIENLVE
QEFDWLDYDRIGKISMRFAFKVGLRSDWFEHQAMPVVDMCTERSPSQDFRDFPARLHTKYVIDLRKQAIA
ERNTRECMSLLKILHSTTLDSKLECEDILECKRFCKLVWCLENGESDELQLGVLRVIRNIIVVHKSSNFT
ERFTNVEYTVFQQLLIDWTSNKAYNIFTIIALRIFLTEKDWTPCEFADEANDLLIDRFQFFQPTEITEDL
EIYMGTVEKVLKNSKLDGPVLWALLTLQILAGRSKENVWTIRGQPEMLKFIGNPRVASEAVRNATESLLE
LIN

>A0AAC1_CAEEL
MMRINGFGKKLSAKQMRAKFERQTECDFDELKESRKWREPPEKALRFENVIIQDTNLPFEQVEESKKMLG
DALQLCGIENEIASFMKRKFDAKYGGHWQCVVGRNFGSHLDPIQFIHFTVSKISVILFR

>BR2GC_RANGU
MFTMKKSLLLLFFLGMISLSLCEQERGADEDEGEVEEQIKRSIWEGIKNAGKGFLVSILDKVRCKVAGGC
NP

>A0AMI7_DROME
MPTKKCYCPVTHVIFDCDGTLIDSEGIYLKTVQDLLAKYGKTYTKVDQTQHMGMPVGTFSQHIVKDLKLP
MSPAEFQKEFEAAVDKSMGSVALLPGVRDLILHLHEYRIPFCIATSSFRKLFKVKAESFKDIFLAFHHVV
CGDDPALGPGRGKPYPDIYLLAASRFNPPADPKKCLIFEDAPVGLIGGKAAGSQVIFIPTDNVSKQQKKG
ATMVLKSMADFKPELFGLPPFDTCSKFEFG

>A0AMQ8_DROME
MEKVCQFFRNNYVLANCEDAIYKKAFSLNLSHYQISDVPGIIEKCETLMKLFLNQNKLTKIPSSIGSLMR
LQVLTLDYNKLDEFPLCICRLVRLKFLNISCNSISSLPPELGYLTQLETFWCNNTGLLELPNEIRNCEHL
ETLGVRGNPLKKLPDAIGALSSLRWLTAEGCELSEVPLTMALLGNLVHLNLKGNRLRRLPRMLMAMQKLR
FAFLNENRIDEMPTRSQLEELRTLHMLNLSKNPISLHRDLQLMALRQTNLYVELPSDPANICDGLASRAS
LNAQEQQEDQARGAGQDLDSSDWANSVRTSELDTTDESALENNIEDLSVMLPEMSRFVTTF

>A0AMT9_DROME
MATITKRKNQTAKDRSRTDATTALRRLVIPRPDILHTYLDYKQVNQYLQYLAQRYAHFVHVHILGHTHEK
REIRALEINWMNSENVELSPQMREHSPRLFDIGPNGRFTVPVIHVGEHCRKTVFIEAGTHAREWISVSTA
LNCIYQLTERYTRNIEVLRKLRFIIVPLVNPDGYEYSRTKNPKWRKNRRPHKSAKFVGTDCNRNYDIFWN
SGPSKINRNTYKGESPFSEPETRAMRCILDRMSSNLLFFLSLHSYGQSIMYPWGYCRDNPIYWRELSSLA
NSGKSAIKSYNGREYRTGSISCLTKRTIAGSVVDYVYGVLKVPMALVMELPSRELGFQPPVEMISQIGHE
SWYGIREMCKRSFDLRHQIVREKEPPLPWPSRHESSNEGVAIENTSTPTEETNTAGGDFAKTTSKKKRSK
RTTIRAQHENILRLQRSLKAREAQDLPEGIQALPPGLFPRQMEPQYPPIKQSGLSRDGQGGGDGVILTSR
GKPTISSPVSLVLL

>A0AMX2_DROME
MAFVARRSSDGSVVDRQQVSGREKVRPASQSDISDEDSESLLGYTNHTYGAPTVRYVGQGEDREAVEPQL
DYEDFGDSLGDHVDNSESETDSILSFCKELDEEEANDALLNGRMSFTDEKAKVEDKANADDVFHRVMNKG
DEAAATDTETVMNFDHDVQSLSSLGSCTPTVTQRGEDVDNLDNISGRTFFKEEKSHSINTRTFCKDNNLD
ALSGRTFYKQASVKETNQSQISSRTYSLSSSGNNQNHDASSMSTCSWSDMYRGALQSTDGCTGMPPLSLA
SVVSDSSTCYKQLEEHKERPGPQAFEDWKARKAAEKQKNLLAAKQEREKREAEAALRQKYAQEHFQEWCR

RKEEQQQQQRQKQKKSSTSSSSLGSVSGSSTAPTRKVSPENTKNRLQKWEQAKREQQQQEKQRLIRLQCN
KQKLEEERRQRRQGAWQNWMKQVDKRAKPVPLNQGFDTLRGTISNIYINPVQWVSNIDPQDSGRSR

>A0AN03_DROME
MAFCGHVTSSGQIVLRDEIFSSRTCIARSPSSGGSLNSMSSPSSLAQNTTRTYFFKDDGGNPSPQSTDNW
SLGGLKRSEIQSNLVPSLCLSSQESCLNYLSHCKGIKVDRQPEAAYQNWYSAKQQQLLEKQRRIKEEQEF
KQQRTEERKQLARMCYEQWLKDKARQAANLQLESHIQDAAMKASIALRKNPLESLRKNPVVSSLSGLGSL
GSGPSSSTAPRRIRKVSKDEIRRVVEEWWLKKQSQQQAQREEKRRAMLSKALKEEQRRQLAQDAWSKWMS
NVDAKPKPVPLNQGMDSLRGTISQLYVNPTPWMGPVKQTQM

>A0AN67_DROME
MQSPAFAYREHAEHSPRAMYMRDWRSALRTPTYRIGNRTVRFNYHFALLIVCAFVLVLLFYFARQGSHSS
SDGYWLRRSYTDARSMDNGGPVVQAYNATYPLTSPMVLRGGVINYRIAMIADLDTSSKVSKGDGSSTWRS
YLKKGYLTYTVARSEIQISWDDGAPIVLESAFALKGRGMELSELVTFNGRLLTFDDRTGLIYEIVNDKPI
PWVILLDGDGHSAKGFKAEWATVKEQTLYVGSMGKEWTTSAGDFENNNPMYVKAITPSGEVRSLNWVDNF
KQLRLQSMQITWPGYMIHESGTWSEERNRWFFLPRRCSKEKYNETKDEHMGCNVLVSADESFTNFETVRL
DPENTTPTHGFSSFKFLPGTDDSIIVALKSEELNGKTATFITAFDIAGKTLLPETRIETDYKYEGFEFI

>A0ANA1_DROME
MASAQVQSFDIMKQQDQQHQLNSIHKHHHHHEGVPLESNSPEPIDEEIYQVEYKHPLEHVWTLWYLENDR
TKHWRDMLNEITEIDSVETFWSLYHTIKTPAELKIGCDYSVFKKGIKPMWEDEANIKGGRWLITVSKSAK
AELDQIWLDILLLMVGQNFEYSDEICGAVINIRNKSNKISVWTANGSNEMAILEIGQKLKILLHLQSHSL
QYQLHSDAMSKFNSGVKSVYTL

>A0ANB3_DROME
MARQRLLGSILSKGGAQVLRNFQSFVQGTRLEYVSYTSPRNQMQAPPIVVMHDLNLSLESWRQVAVNLSQ
VGLRQVITVDARNHGLSPYITGHSPMHLAADVEALMSHQRLNKIVALGHGMGGRAMMTLALTQPQLVERV
ILVDITPAPVPSNFYLTRQVFEMMLQVAPSIPSNLSLSEGRTFILPLFQDVVHDASELARIIYNLRKMQD
NTFGWAVNPQAVLSSWGEMMINYEATLGGLRPYMGEVLLIAGSQSEFVTTTSIAVMQRYFPNTVVQILDA
GHCVYEDQPEQFVELVVEFTQTCLVC

>A0ANE1_DROME
MAKKCQYVAILITVMVILSGAHRMKRLSSPEFHGDETLELAKYVVSIRSRTPNKYFGDNHYCGGGLLSNQ
WVITAAHCVMGQSKIMYKARWLLVVAGSPHRLRYTPGKSVCSPVSSLYVPKNFTMHNTYNMALMKLQEKM
PSNDPRIGFLHLPKEAPKIGIRHTVLGWGRMYFGGPLAVHIYQVDVVLMDNAVCKTYFRHYGDGMMCAGN
NNWTIDAEPCSGDIGSPLLSGKVVVGIVAYPIGCGCTNIPSVYTDVFSGLRWIRHTAYDWASITKTNPTL
MFILLYVLHSCLNRI

>A0ANM3_DROME
MSEEAASTSSFGFKKRNLKKGAGIRRKKETSSDESAKSSDEETKGKASALVRAENRRKRTNPNFQSTKTM
SKAKRQAGGVGDDGSSSASDDDKLGIAYKSKREALPSGPQDQGATSINEMDTELDRDAQAIHARALKINE
ELEGKADDKIYRGINNYAQYYKKQDTAAGNASSGMVRSGPIRAPAHLRATVRWDYQPDICKDYKETGYCG
FGDSCKFLHDRSDYKAGWQLEMDHENQRTGDVDSDGDDIKYEIHSDEETLPFKCHICRQSFVNPVVTKCK
HYFCEKCALAQYKKSQRCIICSQQTNGIFNPAKELIARLKTNPMENLDSDEEEEVGKKEDKAGAGEEAQE

ISSDDSD

>A0ANU3_DROME
MHRHNAESSFCFINRINGNTNDELQQPGEHEPTSALLDYCKRKFLRPYVAILTLVGLNPISTDMTSIRAC
CSYVQALMVLFVLLVGYLLRYICGYRSDRGFTSYRDIRPITNGTDGGATTPNTIGELLFGFIVPSGFNLL
AFVSAVLVCKVIDHEQLQNLIERVFLMSAKPKRLCRMLWLFLAIALALLILLFGYACCVVMMQPSQIVKV
SWLAEILSDWEMWLRVGLLFTILLQDLVEIIILSNYYIECYLLRMHLETLSHKLLMHSIDSLDWMREVLE
FRKLLERLNQHVSIPVCFLIVMNLAYAFAGLVYLFKDFDFHYCALKLVLLNIANVMLWLFLGLLPFFVAS
SVTRVCQNAQANGHQIRVRPFVYHNTSAEDLNSTLLFAASLDMSAKLFRMPIQPNYLCFAILVVVIVILT
LGMCLNLTALGKI

>A0AP24_DROME
MERYNRVYRDSASPLTPLTPLSTEAFTFEDVTPTGGVGRKGTARYGLFGMPKNNNLTVPNSRPALSGLKR
LSESTLPRRFSQKFMRTRSVFSPTSQSTLINGETRLLGESGVSKLLRTKREDRPKPDIRLQQETRLRQEE
SKLKSARKIKVEDPRSPTPSIHHSRYKPCSPVEHPTLSPRVKSLLDRTGNAHLTELFTRQEIDIEVLIQM
TLEDLAALGVRGAREIRLAMNIIQLAKQFF

>A0AP42_DROME
MLITQIIMKQIRDYPIVSTISIAVSTVLASEVIWKLVQCSRSKREKASRVHEVIIFNELGEICAAVHMRN
SSMGSQKPQVSPCCNMHCSLRNVAKIVEQIDRAVYSIDLAIYTFTSLFLADSIKRALQRGVIIRIISDGE
MVYSKGSQISMLAQLGVPVRVPITTNLMHNKFCIIDGFERVEEIRLLRKLKFMRPCYSIVISGSVNWTAL
GLGGNWENCIITADEKLTATFQAEFQRMWRAFAKTEGSQIQLK

>A0APF4_DROME
MSPKKALDDCMIDFDRCVLVTDLLEEYKLSYKEVNDVLEAYIKEQEPATKFEKRFLVHGKRKTQGSDSGE
DLYSVVLESRMQDWLAKVQDAESQLYSVKIAGGTKAPAAIFKPMQHLEVKLAKVEQRPGAGKIVPSANGT
TPHNGVKSEPTKSEPSKSAVKLEPSKSSLKSEPAKSKAEKPVASKASPEDKKTSPKEQASKAKPAAAKKG
SINSFFTAAASKPKDVKATPSKSTSGTVDNFFKKQPAGAKKSPPESEDKSKKDASNSNKKEASKKKSPSP
TKKPTTANTSMQLFDEESAESSDEEEKLDMLRRKVIESDNDSDQEKASSSKRRRISDSEDEEQPPKKSAD
EETITVDEKMDTEPANETYLDEDGFVITQRKPTKAQPANKKVSPKAAAPVNKKKSPPSAAKAGKDAPKTK
QAGIMNFFSKK

>A0APM6_DROME
MVHQLSILCCALLGVAAASYIPHGGYSHEHGVGYSIQTHHEAPKQWQDHHQAHHQWVDVPKAHSWGTDQD
HHHQQWAPVAAHDSHHQWSHHEEPKHHPKYEFNYGVKDTKTGDIKQQWETRDGDKVKGGYTMKEADGRTR
IVEYTADSHNGFQATVKHVGHASHLEHSHSYGQQYGHGHGHATSYVDVKQDTSSKWEDKSNKWW

>A0APN6_DROME
MPTCPVSMAYGWGLGFVVLCMLHIAHTQHGPAGHIEFYSDYGRNHDEERLHYSHQYHISDVASRVHILHQ
EQRNGDYVSGSYSHLEPSGHIRSVHYEVRGANRGFKAVVEQRTGNSRVHQTLEFRSRQPIRALAIAEPVA
FVI

>A0APP3_DROME
MGLTEQLLLIGVVALGGVFGRLNCEFELESVTQNPLVTDVYVKARQPSGYTPHRIVGGADIPPGEHVPYQ

VSLQYRTRGGQMHFCGGSIIAPNRILTAAHCCQGLNASRMSVVAGIRGLNEKGSRSQVLSYSIHPKYQEL
VTSDLAVLSIKPPLKLNNSTISAIEYRSQGKDFVGGGVPVTLTGWGLRLPVPFPFLDNVNYPNVLQRMSY
HTISNSECRNAGMESVTDTEICARGPFRGACSGDSGGPLVMESKNGLQQVGIVSYGLVVCGLYISPDVYT
RVSTFSDWIGNQTKS

>A0APQ1_DROME
MEVNQKWNWDTKPILVKCGGFSSQLAKNVTEKVDGDPLWGSRFDATNPEAVIQTHLDFLRNGADIILTNT
YQSSVEGFVKYLGVTRERGVELIQKSVQLAKQAKEQYLSEIGSEAESALPLIMGSIGPYGAYLHDGSEYT
GNYADKLSKEELRAWHKTRIEICLAAGVDGLALETLPCLMEAEAVTELVLDNFPDAKFWVSLQCMDEKHM
ASGENFAEAALSLWRLVQSRKAENRLLGIGLNCVNPLFVTPLLSSLTKVAGSDRIPLVVYSNRGEIYDVE
QGDWTGTGEEVVKFVPEWIQLGVRIVGGCCRVYPTDVLAIRKYVDGLNIKP

>A0APW6_DROME
MEYGRFQTIGCLLIILGMANSQPLSWCDPDLCPDNTVHIACNNDGKFHESCSPDATMVDLKPYRKLIVNE
HNKRRNYIASGSLPGYYPATRMATMVWDEELEYLATLNLKTCYLEHDDCHNSYRFRNLGQNLCGVDRRRN
WDLNVTNLVEQSMGLWFGEHKLIDSSYITDFKLTKDLEKYGHFVETVLDRNTHVGCAMMRFTNPQYPFLY
IYNTACNYASVYAIGVPVYNAGKPASECRTGSNPEYPALCSIKEQYNPNHNFY

>A0APX3_DROME
MLCQVQWYLSLTYKFNEAVAPGEHWFQGKLEFMLSQRGKPLLVHDGHTFGIQYVRKDKKYWQCNLSRKFN
CKARVTTTDTGDIIVTNNEHCHTEIRQHLRKDYQRNKVMMNALINLSTSHMRRNNMGGLGALPMVPEVLT
QGTPIHEMVQNLHDMAAKVSAGQQHHHQQQQQQRAHDMEEPLDCGLRSSAALEEMARSFMTNNKPSIAFP
GSESGHNQHGSDTKEEPSWKEKIEGDA

>A0AQ14_DROME
MNKITSAFIRVLRQSSSSGGTGNLLGRQLSYKSDLSLDKIYPGARLQIYTPPPPPSGSDKFSGFIPMDRL
EITYSRSSGPGGQHVNTVNTKVDVRFKVAQADWIPEQTRQKLLKVLANRITKDGYFYIKSDLTRSQQMNL
ADALEKLRTIIRSQEAVVPAPPSEETLEKLRRRQERAVRERLQLKRGRAQVKADRQGPSGLDL

>A0AQ96_DROME
MLLCDGLGPEPPRQRHRNRTSAARIRKRPKCCCGDGGSGNQAEQPGGIVSNPISYGQSLTTLARVTAAAL
TTAAMLHTTNALAATGSSSASNSSTGGIALPLGTATPATHELNATQPFGGTGLNFNESGAGLSDHHHHQQ
HNPDEDWLDNIVWVFKAFVMLLIIIAAICGNLLVIISVMRVRKLRTFFYSPASSCHCSSFVVGSHADSVS
FIYSPHN

>A0AQD3_DROME
MRKLVISLAVLGLISLALGHNNPWEQQPTPIKKVPVPVPVPVPVPVYPKEGGGGGGGGGGSGGGGGGGSG
SGDGGGGAIGGTCPRPSPEALRCLREWACQYVIRLDLRCLLTLNGNPLLGNLISIPGITGGGSSSGGGGI
LGGLLGGK

>A0AQD7_DROME
MRKLVISLAVLGLISLALGHNNPWEQQPTPIKKVPVPVPVPVPVPVPVPVYPKEGGGGGGGGGGSGGGGG
GGSGSGGGSGSGDGGGGAIGGTCPRPSPEALRCLREWACQYVIRLDLRCLLTLNGNPLLGNLISIPGITG
GGSSSGGGGILGGLLGGK

>A0AQL9_TRISP
MSSEEKVEMADVPLTAAVDGEDTTKEAGVNEAVAKTKKSMFHNLFVKKQKHDALINTEADQSEHDKIPKN
KCPVKKWFFCQARSCQEQADSAQSQMTIGINMLDRDEKQLNEHIKLNFEDILAEPDGYHSWDWVWRGTYH
TFNTLRRFIYRFLALLFAVPCAIVWAMLFALLTSINVWILTPLAIAISIPAVWLAKTWNFIIRSLLDPFF
KSCGLMRAAHSESTTNQTV

>A0AR24_HUMAN
MNRIVPHISIVMLNVNGQNAPLKRYRMAEWIRIHQPSVCCLQETQHIRKGWKKIFHANGHQKQGGIAIHI
SDKTNFKATAVKKDKEGNYIMIKGLVQQENITVLNIHTPNTGASTFTKQLLLDLRNEIDGNTVILGDFNT
PLTALDRLSRQKLNKEKMDLNYTLEQMDLKDIYRTFYPTTTEYIISTWYILQDRPYDRPQNESQ

>A0AR27_HUMAN
MVAQTIFLSHPQQVITKEYELFEFRRTEVPPLLLILDRCDDAITPLLNQWTYQAMVHELLGINNNRIDLS
RVPGISKDLREVVLSAENDEFYANNMYLNFAEIGSNIKNLMEDFQKKKPKEQQKLESIADMKAFVENYPQ
FKKMSGTVSKHVTVVGELSRLVSERNLLEVSEVEQELACQNDHSSALQNIKRLLQNPKVTEFDAARLVML
YALHYERHSSNSLPGLMMDLRNKGVSEKYRKLVSAVVEYGGKRVRGSDLFSPKDAVAITKQFLKGLKGVE
NVYTQHQPFLHETLDHLIKGRLKENLYPYLGPSTLRDRPQDIIVFVIGGATYEEALTVYNLNRTTPGVRI
VLGGTTVHNTKSFLEEVLASGLHSRSKESSQVTSRSASRR

>A0AUH1_HUMAN
MQAAWLLGALVVPQLLGLGHGARGAEREWEGGWGGAQEEEREREALMLKHLQEALGLPAGRGDENPAGTV
EGKEDWEMEEDQGEEEEEEATPTPSSGPSPSPTPEDIVTYILGRLAGLDAGLHQLHVRLHALDTRVVELT
QGLRQLRNAAGDTRDAVQALQEAQGRAEREHGRLEDSECMTPPPSLPGPAHRLPEGAAPGPQVLPALARL
RSSGGGAGAVHGAGREPGAAGRPPADGGAHSVPARGARSLQLARVAGRARSARRGPLPLRKRPARVLLRL
ASLTPPRARRPAQRLAASAQPGPAQRWHARELRGAGL

>A0AUJ2_HUMAN
MPSFPTPIQHSIGNSGQGNQAKERKKGYSNRKRGSKTLFAGDMILYLENLVVSAQKLLKLISNFSKVSGY
KIKVQKSQLFLYTNSRQAESQIMNELLFTTAKRE

>A0AUP8_DANRE
MEKFDDDSSKKTSEAEPVNVQSLYTTLGLSSEDVDALAKIPESEISVETLPFLIMQIKTKKATDTSSSAD
TGHKDKSKDSEEKQETPKSCTDAAKPKSKSPPKVPQKRSGSHEQKKKAESHKKTERHSGGRRDSKYSSSR
EKQSGDHAAAESECADNPTVFPHECTRCKCVVNSIKTWKEHLSGSRHKSHDSQSSQRSSRPHPPPKRPYS
SDPFAGDSFTSGMPDHLFPPKPNTRVVVAKFPMGAVTVEDLLVLGKPFGTVVKHLVLPAKGFLEFSAHKE
AVNMVNHFQQKPAFVKDTRVGLYLSPRVEGIHSPPKYDEPVPKRLKRTNNPSVVCFSHLPPGEDNQAEVL
ELAAMFGEVWQSKFLDGKALIEMVDWRDADIMVKYYYSNPLKIQGKSIKVTMSYIKSLREMPGELTPRKS
DSGKHSRQKEESSVKDKADDTNQESSEKETKTIKTPEQSQEEGIQLEEGTDGSVVKTEEEEEKVEDEDKQ
EEGEEVQEEQEKMEVGEEEEEKGKEEDEELEVTEELELTEEPSVAKEENRDQESEHNPEEQDDMEFPEDI
EDFVTLDELDNTAGGDSALDSSEPQDGKVVVIWPIKKSPAETMRELISNLSDLCVPFGGLVRYTLSIIKQ
QAMLELETVEKAQEMVKFYKSHKAMINGRPVSVTMCFAMTTIEVPSGRTVFMGNLPNKWYMRQKYKQSTL
LRMAKRYGNLTGFCLNRHQGMCYVQFDSSESAEHMVAQYRRRRRSFSVFGQILNVAICKIGDSQIQWRDP
VAEEETSRNSGPEQDTEEKESKDNGQTTKPSTDGNGKARKRGAKQNQQRPAPECEGVGGDPDSEIISEED

GHEEKTPLEPYQPDQTFGVSYVIPVTGFFCKLCNMFYTDENKAKSEHCRSLDHYNNLKLKHAVEEQADGQ
PDGE

>A0AUQ0_DANRE
MVHVYNCHPFAAQRIVSAEQEPGLVCCGGGALFVVSAGGCKIEAFDLQREESPVICRFSCAGTVLSLQHS
AVGDYLVTIEEKNNATYLRAYTNWRYQAVEKTRVGVRLLGHLLRGSSFRGAPKEQMEIIEIPLFEPPLCV
ACCSLTGDLLVGSAKSLVVFSLKRQTLSEQLSVLDFERVLILHIQGWSPSQVAFSAGYLALQTELEVLVI
KLVQQQKSAQTAEENALLPEDIMVETSLEDDAEMKKDGFSPLHEQEDFFVFPKHQEMLGSEAQDCGITLV
PECTAMDSESRGETTVVYVLFRRFAPDFFQGCSVEETRLHSLQLHPIFTGKQEESSISRCGDPDCMFVFF
SLPNTGYMYSLKSSVELVSTYQYPEKAHQAVLSDQFLYVITRNALQCFTVRCSAVAARVQDPYIDTTMKA
CPPFSMEVCALRIQLFIGLKVLCQTPMTQDSVFMCSAPPNWASMRTPSTDCWTAAHRPSSHTHNTSCTTT
HCVCGGRNYSLRSVTGSGKQEMTTQSSSQLSKRL

>A0AUR7_DANRE
MSLRESPFPNGFLEGLHAVGWGLIFPCFWFLDRLIAVCISTTLERMWRLEQECYLHPLKVVFGSILFFIL
FVISTPFALLGFILWAPLQAIRRPFSYHKQEQSIPMENRNARWEEMGKISFGFLTANLCLLPDGIARFNN
LGHTQKRALVIGKSIVQGVTRPHIRIFVDSPSSCGTVTPSSSLIPQPNASSYGSVDASGELPDAIEVNEI
TPKPNCNQNSNHQKHPPRSLRTLLRDADIPMEVSALFPPSVDIVCLQEVFDKRAARKLTQALGPLYGHVL
YDVGVYACHPAGSCSSFKFFNSGLFLASRFPIMEAEYRCFPNGRGEDALAAKGLLTVKVDIGLQKGEKRM
VGFINCTHLHAPEGDGAIRFEQLNMLSKWTSEFQTLNRRDDELPLFDVLCGDFNFDNCSPDDRLEQSHSV
FDEYTDPCRAAAGKEKPWVIGTLLEQPTLYDENMRTPDNLQRTLESEDLRKDFISPPVALDGVPLVYPEA
DQPWIGRRIDYLLYKEKSGHRTEVEELTYVTQLAGLTDHIPVGFRLSVSLDSEQN

>SMKY_MOUSE
MPAHIEEELPPPSPQPSNPEDGELYSQYKVVRTLGHGTYAKVLLAKHWLTGTPVAVKVLLKNKPCFQPAM
KEANIMKKIKHPNIVSLLQVFETKTRGYLIMELVEGQELYEYIKSSGHIEEDEARQIFLQILSAVSYCHG
LGIVHRDLKPDNIMIDDKGSIKIIDFGLSTQVKPGDLLDEHCGAYAFGAPELFLWKSYDGTKSDLWALGV
ILYYMVVGKVPFDSYIIPELQRQILAGVYPAPCGVSNELKDLLSLLMTVNPKYRPTVTEVMKHPWLRGHC
KGLTNIHEEPVPVRPDPDIVDAMQYIGFQAKDIRESLTKEKFNEMSAAYYLLEEQALQREVRSTQAPTVS
QVKAPFPSMDAGEASCLKIKRSGSASILGRSVWPPSIDQEPAYVQKVRQRAGRRSSGHGLLFEANQMTPT
QDQHHIRAMSVPCMLSTSSISEESVSEKREENLSHIALAEDKPIRSRGWCRGIMRWTRRVGNAIRTLCCC
IPSRKTPQLGQSRVSPQK

>CB067_HUMAN
MTPALREATAKGISFSSLPSTMESDKMLYMESPRTVDEKLKGDTFSQMLGFPTPEPTLNTNFVNLKHFGS
PQSSKHYQTVFLMRSNSTLNKHNENYKQKKLGEPSCNKLKNILYNGSNIQLSKICLSHSEEFIKKEPLSD
TTSQCMKDVQIILDSNITKDTNVDKVQLQNCKWYQENALLDKVTDAEIKKGLLHCTQKKIVPGHSNVPVS
SSAAEKEEEVHARLLHCVSKQKILLSQARRTQKHLQMLLAKHVVKHYGQQMKLSMKHQLPKMKTFHEPTT
ILGNSLPKCTEIKPEVNTLTAENKLWDDAKNGFARCTAAEIQRFAFSATGLLSHVEEGLDSDATDSSSDD
DLDEYTLRKNVAVNCSTEWKWLVDRARVGSRWTWLQAQISDLECKIQQLTDIHRQIRASKGIVVLEECQL
PKDILKKQMQFADQAASLNILGNPQVPQECQDPVPEQDFEMSPSSPTLLLRNIEKQSAQLTEIINSLIAP
LNLSPTSSPLSSKSCSHKCLANGIYRSASENLDELSSSSSWLLNQKHSKKKRKDRTRLKSSSLTFMSTSA
RTRPLQSFHKRKLYRLSPTFYWTPQTLPSKETAFLNTTQMPCLQSASTWSSYEHNSESYLLREHVSELDS
SFHSVLSLPSDVPLHFHFETLLKKTEIKGNLAENKFVDEYIISPSPVHSTLNQWRNGYSPICKPQIRSES

SAQLLQGRKKRHLSETALGERTKLEESDFQHTESGSHSNFTAVSNVNVLSRIQNSSRNTARRRLRSESSY
DIDNIVIPMSLVAPAKLEKLQYKEILTPSWRMVVLQPLDEYNLGKEEIEDLSDEVFSLRHKKYEEREQAR
WSLWEQSKWHRRNSRAYSKNVEGQDLLLKEYPNNFSSSQQCAAASPPGLPSENQDLCAYGLPSLNQSQET
KSLWWERRAFPLKGEDMAALLCQDEKKDQVERSSTAFHGEIFGTSVPENGHHPKKQSDGMEEYKTFGLGL
TNVKKNR

>S12A8_HUMAN
MTQMSQVQELFHEAAQQDALAQPQPWWKTQLFMWEPVLFGTWDGVFTSCMINIFGVVLFLRTGWLVGNTG
VLLGMFLVSFVILVALVTVLSGIGVGERSSIGSGGVYSMISSVLGGQTGGTIGLLYVFGQCVAGAMYITG
FAESISDLLGLGNIWAVRGISVAVLLALLGINLAGVKWIIRLQLLLLFLLAVSTLDFVVGSFTHLDPEHG
FIGYSPELLQNNTLPDYSPGESFFTVFGVFFPAATGVMAGFNMGGDLREPAASIPPGSLAAVGISWFLYI
IFVFLLGAICTREALRYDFLIAEKVSLMGFLFLLGLYISSLASCMGGLYGAPRILQCIAQEKVIPALACL
GQGKGPNKTPVAAICLTSLVTMAFVFVGQVNVLAPIVTINFMLTYVAVDYSYFSLSMCSCSLTPVPEPVL
REGAEGLHCSEHLLLEKAPSYGSEGPAQRVLEGTLLEFTKDMDQLLQLTRKLESSQPRQGEGNRTPESQK
RKSKKATKQTLQDSFLLDLKSPPSFPVEISDRLPAASWEGQESCWNKQTSKSEGTQPEGTYGEQLVPELC
NQSESSGEDFFLKSRLQEQDVWRRSTSFYTHMCNPWVSLLGAVGSLLIMFVIQWVYTLVNMGVAAIVYFY
IGRASPGLHLGSASNFSFFRWMRSLLLPSCRSLRSPQEQIILAPSLAKVDMEMTQLTQENADFATRDRYH
HSSLVNREQLMPHY

>A0AV37_HUMAN
MAAPGAPAEYGYIRTVLGQQILGQLDSSSLALPSEAKLKLAGSSGRGGQTVKSLRIQEQVQQTLARKGRS
SVGNGNLHRTSSVPEYVYNLHLVENDFVGGRSPVPKTYDMLKAGTTATYEGRWGRGTAQYSSQKSVEERS
LRHPLRRLEISPDSSPERAHYTHSDYQYSQRSQAGHTLHHQESRRAALLVPPRYARSEIVGVSRAGTTSR
QRHFDTYHRQYQHGSVSDTVFDSIPANPALLTYPRPGTSRSMGNLLEKENYLTAGLTVGQVRPLVPLQPV
TQNRASRSSWHQSSFHSTRTLREAGPSVAVDSSGRRAHLTVGQAAAGGSGNLLTERSTFTDSQLGNADME
MTLERAVSMLEADHMLPSRISAAATFIQHECFQKSEARKRVNQLRGILKLLQLLKVQNEDVQRAVCGALR
NLVFEDNDNKLEVAELNGVPRLLQVLKQTRDLETKKQITGLLWNLSSNDKLKNLMITEALLTLTENIIIP
FSGWPEGDYPKANGLLDFDIFYNVTGCLRNMSSAGADGRKAMRRCDGLIDSLVHYVRGTIADYQPDDKAT
ENCVCILHNLSYQLEAELPEKYSQNIYIQNRNIQTDNNKSIGCFGSRSRKVKEQYQDVPMPEEKSNPKGV
EWLWHSIVIRMYLSLIAKSVRNYTQEASLGALQNLTAGSGPMPTSVAQTVVQKESGLQHTRKMLHVGDPS
VKKTAISLLRNLSRNLSLQNEIAKETLPDLVSIIPDTVPSTDLLIETTASACYTLNNIIQNSYQNARDLL
NTGGIQKIMAISAGDAYASNKASKAASVLLYSLWAHTELHHAYKKAQFKKTDFVNSRTAKAYHSLKD

>RBM47_HUMAN
MTAEDSTAAMSSDSAAGSSAKVPEGVAGAPNEAALLALMERTGYSMVQENGQRKYGGPPPGWEGPHPQRG
CEVFVGKIPRDVYEDELVPVFEAVGRIYELRLMMDFDGKNRGYAFVMYCHKHEAKRAVRELNNYEIRPGR
LLGVCCSVDNCRLFIGGIPKMKKREEILEEIAKVTEGVLDVIVYASAADKMKNRGFAFVEYESHRAAAMA
RRKLMPGRIQLWGHQIAVDWAEPEIDVDEDVMETVKILYVRNLMIETTEDTIKKSFGQFNPGCVERVKKI
RDYAFVHFTSREDAVHAMNNLNGTELEGSCLEVTLAKPVDKEQYSRYQKAARGGGAAEAAQQPSYVYSCD
PYTLAYYGYPYNALIGPNRDYFVKAGSIRGRGRGAAGNRAPGPRGSYLGGYSAGRGIYSRYHEGKGKQQE
KGYELVPNLEIPTVNPVAIKPGTVAIPAIGAQYSMFPAAPAPKMIEDGKIHTVEHMISPIAVQPDPASAA
AAAAAAAAAAAAVIPTVSTPPPFQGRPITPVYTVAPNVQRIPTAGIYGASYVPFAAPATATIATLQKNAA
AAAAMYGGYAGYIPQAFPAAAIQVPIPDVYQTY

>TTC26_HUMAN

MMLSRAKPAVGRGVQHTDKRKKKGRKIPKLEELLSKRDFTGAITLLEFKRHVGEEEEDTNLWIGYCAFHL
GDYKRALEEYENATKEENCNSEVWVNLACTYFFLGMYKQAEAAGFKASKSRLQNRLLFHLAHKFNDEKKL
MSFHQNLQDVTEDQLSLASIHYMRSHYQEAIDIYKRILLDNREYLALNVYVALCYYKLDYYDVSQEVLAV
YLQQIPDSTIALNLKACNHFRLYNGRAAEAELKSLMDNASSSFEFAKELIRHNLVVFRGGEGALQVLPPL
VDVIPEARLNLVIYYLRQDDVQEAYNLIKDLEPTTPQEYILKGVVNAALGQEMGSRDHMKIAQQFFQLVG
GSASECDTIPGRQCMASCFFLLKQFDDVLIYLNSFKSYFYNDDIFNFNYAQAKAATGNTSEGEEAFLLIQ
SEKMKNDYIYLSWLARCYIMNKKPRLAWELYLKMETSGESFSLLQLIANDCYKMGQFYYSAKAFDVLERL
DPNPEYWEGKRGACVGIFQMIIAGREPKETLREVLHLLRSTGNTQVEYMIRIMKKWAKENRVSI

>A0AVG2_HUMAN

MQAINTSIKNKIPCVVVEGSGQIADVIASLVEVEDALTSSAVKEKLVRFLPRTVSRLPEEETESWIKWLK
EILECSHLLTVIKMEEAGDEIVSNAISYALYKAFSTSEQDKDNWNGQLKLLLEWNQLDLANDEIFTNDRR
WESADLQEVMFTALIKDRPKFVRLFLENGLNLRKFLTHDVLTELFSNHFSTLVYRNLQIAKNSYNDALLT
FVWKLVANFRRGFRKEDRNGRDEMDIELHDVSPITRHPLQALFIWAILQNKKELSKVIWEQTRGCTLAAL
GASKLLKTLAKVKNDINAAGESEELANEYETRAVELFTECYSSDEDLAEQLLVYSCEAWGGSNCLELAVE
ATDQHFIAQPGVQWYVNGVNYFTDLWNVMDTLGLFYFIAGIVFRLHSSNKSSLYSGRVIFCLDYIIFTLR
LIHIFTVSRNLGPKIIMLQRMLIDVFFFLFLFAVWMVAFGVARQGILRQNEQRWRWIFRSVIYEPYLAMF
GQVPSDVDGTTYDFAHCTFTGNESKPLCVELDEHNLPRFPEWITIPLVCIYMLSTNILLVNLLVAMFGYT
VGTVQENNDQVWKFQRYFLVQEYCSRLNIPFPFIVFAYFYMVVKKCFKCCCKEKNMESSVCCFKNEDNET
LAWEGVMKENYLVKINTKANDTSEEMRHRFRQLDTKLNDLKGLLKEIANKIK

>FR1L5_HUMAN

MLRLVVQSAKIDPPLAPLPRPCMSIDFRDIKKRTRVVEGNDPVWNETLIWHLWNRPLENDSFLQVTLQDM
GSQKKERFIGLATVLLKPLLKQPSEVLFVKDLTLLNHSMKPTDCTVTLQVAHMSNQDIEKTGAEDHLGIT
AREAASQKLMVPGSTAHRALSSKPQHFQVRVKVFEARQLMGNNIKPVVKVSIAGQQHQTRIKMGNNPFFN
EIFFQNFHEVPAKFFDETILIQTDIGFIYHSPGHTLLRKWLGLCQPNNPGSGVTGYLKVTIYALGVGDQA
LIDQKLLYGTDDTDIQIFKSAVVPINMAYLQLFIYCAEDLHLKKHQSVNPQLEVELIGEKLRTHMQTQTD
NPIWNQILTFRIQLPCLSSYIKFRVLDCRKKDCPDEIGTASLSLNQISSTGEEIEGKQSLEPTSYTPRVY
SGFLPCFGPSFLTLHGGKKAPFRIQEEGACIPDSVRDGLAYRGRVFLELITQIKSYQDSTIKDLSHEVTR
IEKHQNRQKYGLCVIFLSCTMMPNFKELIHFEVSIGHYGNKMDLNYKPLVSSTPYSPVIYDGNIYHYVPW
YNTKPVVAVTSNWEDVSFRMNCLNLLHFTRDRLKANLDTLKSTRNPKDPALLYQWEKLLRELAEDCKRPL
PCMTYQPKATSLDRKRWQLRSLLLQELAQKAKQAKPKDMVATAEDWLYRLNTVLPEPQMGLPDVMIWLVA
KEQRVAYAQVPAHSVLFSPAGALHSGRLCGKIQTLFLQYPEGEGQKDVLPAHLRVCMWLGNVTDSKDLQL
LRQGDTAVYAEMYENQAKYKDQWGQQGLYHCPNFSDVMGNKTLPMTDFQPPLGWHWQDSWTVEPQRRLLL
DIDINKSQVLEEVYENQGRDTRGAWGPAAIPNTDVNGQPMEARENVKCPQGWHFKKDWVVELNHAVDSWE
YGVGIPPSGLPQVWSPVEKTYHSCRRRRWARVRFRNHGELSHEQETLSFLQLGLAKGEEEGWEYDTFGSK
FHLNPQPQSRFRRRCWRRRLAPNKDKGIAPIFLLEGSLACEGGSVREEGDWAWARGNRSSTQEPQGRLSW
VQAMDLKYHAGKEEDSKTWPWGLDRQFRDPQRQDTRPPNLPFIYCTFNKPHYYQLFCYIYQARNLVSNQI
LTFQGPFIRVVFLNHSQCTQTLRSSAGPTWAQTLIFQHLLLYENPQDTKESPPLVVLELWQRDFWGKESL
WGRSVWPPMVWLDLQDRILPPMRWHPLVKELGKEEGEILASCELILQTEKLGEKQLPILSVPWKNGAYTL
PKSIQPTIKRMAIEILAWGLRNMKKASSPQLLVEFGEESLRTEPIRDFQTNPNFPESESVLVLTVLMPTE
EAYALPLVVKVVDNWAFGQQTVTGQANIDFLQPYFCDPWAQDYMHPKLPTLSEKKHQDFLGYLYRKFWFK
SSKAEDEYEHEVDWWSKLFWATDEHKSLKYKYKDYHTLKVYECELEAVPAFQGLQDFCQTFKLYQEQPKL

DSPVVGEFKGLFRIYPFPENPEAPKPPLQFLVWPEREDFPQPCLVRVYMVRAINLQPQDYNGLCDPYVIL
KLGKTELGNRDMYQPNTLDPIFGMMFELTCNIPLEKDLEIQLYDFDLFSPDDKIGTTVIDLENRLLSGFG
AHCGLSKSYCQSGPFRWRDQMPPSYLLERYAKRKGLPPPLFSPEEDAVFYNGKKFKLQSFEPKTPTVHGL
GPKKERLALYLLHTQGLVPEHVETRTLYSHSQPGIDQGKVQMWVDIFPKKLGPPGPQVNINPRKPKRKAS
EHSGHRYELRCIIWKTANVDLVDDNLSREKTSDIYIKGWLYGLEKDMQKTDIHYHSLTGEADFNWRFIFT
MDYLAAERTCVQSQKDYIWSLDATSMKFPARLIIQVWDNDIFSPDDFLGVLELDLSDMPLPARHAKQCSI
RMMDADPKWPYFIQYKHFSLFKKKTVTGWWPCQVLDGGKWRLSGKVKMSLEILSEKEALIKPAGRGQSEP
NQYPTLHPPLRTNTSFTWLRSPVQNFCYIFWKRYRFKLIAFMVISIIALMLFNFIYSAPHYLAMSWIKPQ
LQLYPPIKIFNIINSLNTSNASSSILPTQDPNLKPTIDHEWKLHPGPTNHLSDIFPELPAPGD

>TM129_HUMAN
MDSPEVTFTLAYLVFAVCFVFTPNEFHAAGLTVQNLLSGWLGSEDAAFVPFHLRRTAATLLCHSLLPLGY
YVGMCLAASEKRLHALSQAPEAWRLFLLLAVTLPSIACILIYYWSRDRWACHPLARTLALYALPQSGWQA
VASSVNTEFRRIDKFATGAPGARVIVTDTWVMKVTTYRVHVAQQQDVHLTVTESRQHELSPDSNLPVQLL
TIRVASTNPAVQAFDIWLNSTEYGELCEKLRAPIRRAAHVVIHQSLGDLFLETFASLVEVNPAYSVPSSQ
ELEACIGCMQTRASVKLVKTCQEAATGECQQCYCRPMWCLTCMGKWFASRQDPLRPDTWLASRVPCPTCR
ARFCILDVCTVR

>E2F8_HUMAN
MENEKENLFCEPHKRGLMKTPLKESTTANIVLAEIQPDFGPLTTPTKPKEGSQGEPWTPTANLKMLISAV
SPEIRNRDQKRGLFDNRSGLPEAKDCIHEHLSGDEFEKSQPSRKEKSLGLLCHKFLARYPNYPNPAVNND
ICLDEVAEELNVERRRIYDIVNVLESLHMVSRLAKNRYTWHGRHNLNKTLGTLKSIGEENKYAEQIMMIK
KKEYEQEFDFIKSYSIEDHIIKSNTGPNGHPDMCFVELPGVEFRAASVNSRKDKSLRVMSQKFVMLFLVS
TPQIVSLEVAAKILIGEDHVEDLDKSKFKTKIRRLYDIANVLSSLDLIKKVHVTEERGRKPAFKWTGPEI
SPNTSGSSPVIHFTPSDLEVRRSSKENCAKNLFSTRGKPNFTRHPSLIKLVKSIESDRRKINSAPSSPIK
TNKAESSQNSAPFPSKMAQLAAICKMQLEEQSSESRQKVKVQLARSGPCKPVAPLDPPVNAEMELTAPSL
IQPLGMVPLIPSPLSSAVPLILPQAPSGPSYAIYLQPTQAHQSVTPPQGLSPTVCTTHSSKATGSKDSTD
ATTEKAANDTSKASASTRPGSLLPAPERQGAKSRTREPAGERGSKRASMLEDSGSKKKFKEDLKGLENVS
ATLFPSGYLIPLTQCSSLGAESILSGKENSSALSPNHRIYSSPIAGVIPVTSSELTAVNFPSFHVTPLKL
MVSPTSVAAVPVGNSPALASSHPVPIQNPSSAIVNFTLQHLGLISPNVQLSASPGSGIVPVSPRIESVNV
APENAGTQQGRATNYDSPVPGQSQPNGQSVAVTGAQQPVPVTPKGSQLVAESFFRTPGGPTKPTSSSCMD
FEGANKTSLGTLFVPQRKLEVSTEDVH

>UBA6_HUMAN
MEGSEPVAAHQGEEASCSSWGTGSTNKNLPIMSTASVEIDDALYSRQRYVLGDTAMQKMAKSHVFLSGMG
GLGLEIAKNLVLAGIKAVTIHDTEKCQAWDLGTNFFLSEDDVVNKRNRAEAVLKHIAELNPYVHVTSSSV
PFNETTDLSFLDKYQCVVLTEMKLPLQKKINDFCRSQCPPIKFISADVHGIWSRLFCDFGDEFEVLDTTG
EEPKEIFISNITQANPGIVTCLENHPHKLETGQFLTFREINGMTGLNGSIQQITVISPFSFSIGDTTELE
PYLHGGIAVQVKTPKTVFFESLERQLKHPKCLIVDFSNPEAPLEIHTAMLALDQFQEKYSRKPNVGCQQD
SEELLKLATSISETLEEKPDVNADIVHWLSWTAQGFLSPLAAAVGGVASQEVLKAVTGKFSPLCQWLYLE
AADIVESLGKPECEEFLPRGDRYDALRACIGDTLCQKLQNLNIFLVGCGAIGCEMLKNFALLGVGTSKEK
GMITVTDPDLIEKSNLNRQFLFRPHHIQKPKSYTAADATLKINSQIKIDAHLNKVCPTTETIYNDEFYTK
QDVIITALDNVEARRYVDSRCLANLRPLLDSGTMGTKGHTEVIVPHLTESYNSHRDPPEEEIPFCTLKSF
PAAIEHTIQWARDKFESSFSHKPSLFNKFWQTYSSAEEVLQKIQSGHSLEGCFQVIKLLSRRPRNWSQCV

ELARLKFEKYFNHKALQLLHCFPLDIRLKDGSLFWQSPKRPPSPIKFDLNEPLHLSFLQNAAKLYATVYC
IPFAEEDLSADALLNILSEVKIQEFKPSNKVVQTDETARKPDHVPISSEDERNAIFQLEKAILSNEATKS
DLQMAVLSFEKDDDHNGHIDFITAASNLRAKMYSIEPADRFKTKRIAGKIIPAIATTTATVSGLVALEMI
KVTGGYPFEAYKNCFLNLAIPIVVFTETTEVRKTKIRNGISFTIWDRWTVHGKEDFTLLDFINAVKEKYG
IEPTMVVQGVKMLYVPVMPGHAKRLKLTMHKLVKPTTEKKYVDLTVSFAPDIDGDEDLPGPPVRYYFSHD
TD

>A0AVV0_DROME
MWKCSPTPSPRKSLYVGHTDYKYLRWQVIDTPGILDHPLEERNVIEMQAITALAHLRACVLYFMDISEQC
GHSLEEQVKLFESIKPLFTNKPLILAINKIDILTPEDLPEERRAIITKLQEDKNIPVMLMSTVQETGVME
VKTEACERLLSYRVDQKMRTKKVDNILNRLHVAMPAPRDDKLRAPCIPEKASARLLQNADKAERKRKLEK
EIEEEMGDDYTLDLKKNYSEIPEEERYDIIPEFWQGHNIADYIDADIFDKLEELEREEGLREESGVYKVP
DMTMDETLKEIREIAKQIRGKRFELRDEKRLSSRKNKPVIPRNKQPKVRDRSVQKLVSTMEGLGVDMSGS
ENANFTKSVVDLRRGQVAVGSKKVPMQPLLDKESSAVVRKTGQPLKRAPSRDTLGIKNLAIRKKAQIMAK
RDIAKKVTSRGLKGEADRFIGTKMPKHLFSGKRGNGKTDRR

>A0AVX5_DROME
MNDEFKLCWKNFQDNIASGFQNLYDRGDLVDVTLACDGKLLHAHKIVLAICSPYFQEIFTTNPCKHPIII
LKDVSFNIMMELLEFMYQGVVNVKHTELQSFMKIGQLLQIKGLATNSNSSPGSSVSEKSSSQPPAEESSN
TNSTNHHNSSTNSNNNSKSETDHNESKGQSNSRTTSPGGASRASNDASHLYTSNKRPMPSDFGSDSLSIY
SGKQLRRSLKDHGSGGSEGGGGDHADTAAGLDNSMNSEEFFLPPIPQITMGEQRYDLGGLKRESDGHHHG
PLSAGGGNSGSVGSLTSSASPSAPIRNPFAPNFMDSFNYYKGGNSSGPSGSSNNSVGPGGVNNNGSVGLG
SGAEYPNELYMPNDYSKSFANHMDIPSSGSNMVMLSTTSLLHGNCVFNRNNTVATQQGMKTYWLCKSYRI
SMCRARCITHLGRIISATGVHNHTPHMRGGQGSSAPSTASVSGNPAPTPISSQAGYSLTDGGGHLGPDLQ
HGNRVTNMFANQTPPPPPPPPPLPVASTPHHPHHHHHLGQPLPNLLVQHHPAPSHMEQHGSSASLLHNPN
LMHLQTPTHHHQQHHQQHSPHNPPPPQQHNLELGGGSGSGAVLLSPAHLQQSPQSNRSSHSLQAQSPPP
QSVEEQQPHQQQQQQQEGATTEVTTDPSTAHVINSITISPNTRHTFKMENM

>A0AYH9_BURCH
MRTLSARRRMSVRCLSREDSNMELRHLRYFVAVAEERNFTRAAERLHIAQPPLSRQIQQLEEALGVPLFE
RTARPLKLTDAGRFFYSHAVQLLAQTTELESMTKRVGKIERSMSVGFVGSTLYGMLPKIIRRFRSEYPAV
ELSLHEMSTMDQIKALKEGRIDVGFGRIRHEDPSVRRVVLREERMIVALPVGHVLDSAKPVLSLHDLVND
TLIIFPKAPRPSYADQVLAAFHDRALQPRRIYETRELQIALGLVAMGEGVSVVPQSVYGLKRDDISYKPL
DDPNLVSPIIMSMRMLDESEDIRAMQALIYRLYDEAQMEYLPPQPE

>A0B2G5_BURCH
MNWQGIRAIYRAEMARTRRTLMQSIIAPVISTSLYFVVFGSAIGSRISDVNGIGYGSFIVPGLVMLSLLS
QSISNASFGIYFPRFTGTIYEILSAPVSYWEIVIAYVGAAASKSILLGVIILATAGLFVPLHILHPFWMV
LFLVLTAVTFSLFGFVIGIWADSFEKLQLVPLLIITPLTFLGGSFYSVDMLPPAWRVVTLFNPIVYLISG
FRWSFYGIADVHVWISLAATALFLVILLAIVAWMFRTGYKLKN

>RL72_PARTE
MSQKKQKIQVEQKVPENVAKKTQRDSKLRDAVAKRRTERLAANKTRRAQWEKTAQAYEAEYKAADKSLVD
NLRKAKTEGGFYVPAEAKLILVVRIRGINTLNPQVRQTLRLLKLRQLHNAAFVRVNKATIEMIRKVEPYV

TYGYPSRAVIKNLIYKRGYAKINGQRIPITNNNVIEQQLGKVGIHSVEDLIHEITTVGPHFKEANRFLWA
FKLRGPRGGFIAKRRSFINQGDWGNREDLINDLVKRMI

>A0BJ67_PARTE
MQLDESNKTKKNEQSILKETSKNRNIVSKLQGNPARLQGLKDSFKYFTLLKKNQYLERKQYRVSRDETLV
CSCIMCPEDQIQSRPQGPQYSYNCGERCLNRFTCTECDVELCPCAEQCKNRRFQKHDDACVYPLRCGGKG
MGLFAGERILKGQFIMQYVGEIFQINSAFGRRRVQEYSKSTCTYLMKLNNQEVIDPTSKGNLARFINHSC
EPNCITEKWNVLGEVCIGIFAIRDINEDEELTFDYQFDVFHTPLTKCLCGANKCKGYLGLKPTDVTQEEW
EEHLENMVCKICQTKTPQDDEQLLLCDKCNCGFHLLCLVPPLSSVPKDAWYCQECQDEKRILAESEKEKE
KIEGLLQQKVKQLNNEKKNKKKMDSSSSSSDSDSEYDNRYKMMKSLEKQTVREYLQQKQHQQQQINKLEE
DVNYNKKEDLDTNTSDLPLTSKGIKSTGTSLNNLDQFVKQSFEFQSQNSGKLQQMQSIVDVAQKKFEEQY
NDNNELQQQIEQIQVQNIQNIDTKKECFKINILEQKVVKKNAPLIYKIGNKISFEQIDQQYADFFKKENN
LTVIGSVQQISIFRSILIMIEQIIKELKKELGIIEGQIKVPIIYLKRLIMKFNSLDKKQEVNIIYNKSLA
HSEEIFPMDKATPIKIKGSKQNIESTVQEISKILKTLCVQRLYISRSETKTVQQNMYHLKQHAEIRISRD
SVYNAKGEQTQRDINHPFFYIQYREKEVCLIGTLQQIQDTSNAIKLLLEQETNNEKEMTYFTVLISPQYK
EQCKQVKQRIEKDSSSTKVLLFEASHPRKNMTILILCLRNQIKQTKQMFFEMIQEDCQLKLEQFQDQMSM
QMCRYVFKYLQNTMMTNDMAFMKNWDLITPYFYQFNIMKYREQKFDNWFVKRCYPSLLRDYETQLYIQYC
LGKEIQKNLTDQEQMLRKRNLILLTRKILNAILGFKRHQPEQAQIDGNFQQDSFRRYNSFQEDDTPKFQN
LSLISLISHTSQFSKKSDRQKESSDNSSSDSSNNQHQKRQSSSESMKSRDLSTKQKHKSHHHRYNYNQES
SIDRYDKYKYEKSHNYDKSKPRTSRRYYEDNDHSRNRGYNKRSNNLNQFKDLAVKAHKNHKITEEKDRNL
QTLINIKIVSIIVDFNLKEDIPRVTNMIMKSLQKCKKAKKDLDQDKETNQIVAMVIEEDLNQLINPRNTI
GSIIKPKVFFVLGGPGAGKGTQSAKMVERYHFVHLSAGDLLREERSKKTQNAELIEEIIKSGQIVPSHIT
ISLLESAMQAKGLDKMFLIDGFPRNQENFDVWIKLMGDKVEFKKLFYFECDEETLKNRIKIRAQESGRSD
DNDETLVKRLKTYNESTRPIIQHFDSLSQCIHIKADKPIEEVFQEITKKLDEIL

>A0BST8_PARTE
MRIPQAYYESMVRRYKKSKHVDSTDYSWIETTKKPLVERIQDFAQCKQLQDPMYTRIICETIDTIFEAAV
ALFQDQDQTLCIQLLGESDSIQIWDLCLLCCDYILKSTNSKILDMFFSYEHILTLIFHNPDYEIQKIYKL
VQCMQYLIESMNASKACKFKIEQFEKVFIRFMYISAFLQRNIYNLSDQEYQELFQYSTDNYNLKITIPNE
DMNIYQRYLELSNIRKILKSPPVILDIPFDCTKNPLDQSLQIAAEIKVQKDSILFDQIFIRLLCQYSIDE
KLLQKMFLRNFIFHFKCTFSELQLINPKFMTDITNRLIDQKDFLFLLIRKIASEKDLFLEDTLVESYKFI
KSKQIQLYEHNTFLDKLWLFELQGIQDTLNWWKKEFKITEGNFYDSKMELNDKLKQLFFQQLCDQQFTQM
FKKQPNYTQWNFIIIQIIRKSLVQDDGTKLPYYILNPILTYSVNLITELPANNQNQQLDYQLESQIIQGL
MEILKNCIKKPQIQLQIRAIQLLNQLVTYYKQEFDQVQIQNAQSEFLIPVPERYHKLLTEIWSNKILEGT
LLAAQELNETLYTYLITLLAITMGENPARVRQFCENNSLQLLIDKIFELLKICSRASPLIVTIINLLIAI
STNEQAISLTQQSNNSGDKQEKDNLIKRIFIKCYLPSVELINDPTVSDDFAKKIIMLYSQSMLAKKQIEK
VIENTFVQISSLVDLSKDKIEEIVSNQDKLQSFYSSSKSLQALLQLFMQLQPERKSSYRSFQGYEELQLE
EAQRQKDILFNSLLKNQKFVEALEKIIMSPILIGSKNKDTYVEHYVGILCKKNKLNPYTSLWKELDLISQ
NYMVIGDQKIIDIGKLVDYDALSIHMSDKYFFRKIIKHISPQTYRGFYILSRFHLIHYLIKMCVNQGVGQ
NQDAHLMISQLLEIYFLSSQHAGFHESIMMFMSAKQQVQKHQTSYDEDSQIRRRISKDNLIPLQSSCLDL
ISKLQQHAEQKDFVVKKYGEITQLFFQNFSEKCQQLKQAQGIQIVSIVTYLQDMLKSFKFLQNLFLSLEQ
QYQPLQFAVICFQNSNFFLSLVEILQHLDKIQRQQQFNGTKEQEIIRKILPGFIEAIHQHIDNIIKLLIF
KKISIDKIQLSDQLIQDPQQLDIVCNEIFVAQASIVNQMIDLLLYNNCEGSLKTLLEQLIQRFYSIKDLQ
VQDMRKIAGTETKEQIIGNKDINTILSGTDGRQEQKKSLFDRPEIKAMIDDICDMGFKQQLIKKAIQRIE

IPEPTMIIDMLINERISDDEEEQEDPFELVKNAEQNQQVQDQQDQQQQNVETETQKEQKEIQALDQEQFM
KILIQNFPLANTQLNITERFLGFIGDFQSKLDQNLSKLLDQNLHNQKSSGNLLEYLFTIAKRSQHINKII
EYIFMILNPLKQDKSSQDLNATMIQYQKALQWLNLLQQIAPQEHEKLYSNLALIDQEPQSKYVLQFQSMI
TIGFALSNDIFDQFKKIGKLELDQLKQLKISEMTYTEIYQQAQTITNNLNMLFETKQTTLKLFCKELLEA
QKYLTKFFLNLQNKHDQLIERYWNQILIYLEFLIQVCTNNLELQEYSQQTQNFLDELETIISNNQQLKQQ
RDQSQNNIQLRQLIQRRFNSDKQLITLLLQCLNNFGLHNQGKLFSMLTKKQTNQKQSLLDTLLQLKFYSS
HLEYEESQPVFLENLIYLVAIDSKTTNDLVFQILKQVMLNEHIDWHAQQLKNTEDLSQIQFPQKFIECPL
QYLLQFHPVLINNPIVYNMIIEQQGLSITDKFSELQRIHAPKQSLPQPTGKLTKEQERLLKEREKELQAQ
PQTDIIIRYEVEQLIEPQLNEHFIDLVNIIAQTTIDRFFEENCILIEQIFKIQCNQYKDDNRIYLYGWDQ
LLRIIEMLIIKIPQLVLYLRQYQFVLPSNSKTDQKKITFIEFVIKYLTWVGGDKLANFMHNYISDINLLS
IGYGVTIAQEVLQLLLNELQIDINQEQMIQKYYHILQLLTLFTQTLTMQNCCMILTDQRSQLNIIPQLID
TLNKIKNCENKQFVKTYQQTIAKALEPFLQSQIYYDHIQYGRYGEVFRLQQSITDNPFKYHYLHCLKNQL
ICYSLYPGFKSTIGSAQHFISHQQVQQTLKDEHQQELVWPLYQSRKNYNNQRQNNQKQQEEDLSDSDSDQ
DILQVDNNQIKYKANYSNINSWGSDYYYYDNNDFMSKYSMNRDDDRVQRENQQNIFPQKDLNILFCKVDP
QTITFNLPPLQITKASIQYNSSDYKQWITSIVGLYYSSNEYFENKKSLISKSYENQLKITVENINQINRI
LLSSNYYRNRSNDFMQQYLSYYLNGTLNNLQVQASICFYSKFGQPPNQLQQDYQANSLSININESKQQQH
RILCQISCNILQSRVVLNGMPNDLQDQNLLKAKLSSLLHPEEHLIIQPIILQIPKRQFAQPDPQQLFTYD
NLVLEIFINLLLDANNFPQFPFNLFRQICKCPRLGFKLLIQLLLLFQEQKHNLDLTLRILYLVSQKIPST
ILRDLLSEQLQVSPILNRREKANLAKVQEIKEQFVIEEEENVDEQPQDEDPENPLVFLINQLNTYCEKNI
SFMKLFHNPESYIPLEKNILLTPLLASQTGMHLSYLLQIIRGSLIVITEDPKDKNRKHDEDYLSKIQRRQ
ATIFTNNNQDEPNHKLFDLVSQLNKHEQDVNFEEMIFKTFGDRYSDNQYDQWEPISYFSKQPFSFRQHEL
QQQQQQQQQQQQQQQQQQQQQQQQQQQQQQQQYQQQLQQQQQQQQQRFILQNTSIFSMPQPPQPLLPQTL
PQNHPPIPSISILQQQQQSLQQFQQQRQNQSQILQQSGAQQVRQFYDKFFKMNENQQDQSQSYQQYLKQF
YDNIIQSRQQQQNQQQQEQQQNQNQQQNLIQEQQPQQAEEKFLNNIEELDESDSKQQKRERKVSAKISHV
EIDIITALCKILNNHLLRDCNSTEIINILNTYANDRLKIEHAINELTKYIIQQSKIFNYELDRKRKQLRE
ITSQLEKVAEHGPERQQQLSQQENEIIRELNQKTPDPSIIQRCFIAITGFLNISLSISDQQKQSANKPSS
QQTRQQIQLDKKKYKLHQQGKNVDDSQEVDILSKKDIRKQFINILQTRQTHHLWLNVVETLLLIFNCKGQ
RSLELLQKKLYPLLECFLRLYQNVHDDSNNSDSLISGSSDPNFFERNASGSATNSLQFGLLQTQNIHLKR
SFSAVRNENEMQMKIDELFTYLCINGKRLINHLISQRLQQVTIDHKQNQNSNLIKLFKETDPLAFVFFKM
SQIISFENKKHLFELDLEALKISEKSNTRSRGRQNETIEINVVRETRLQQSLEKVINIDKAKFRKCDIKI
RYQGEKGQDEGGIRKEWMTNLVKDILQTDKFELTPKRFYKINPNKKAQEDLNHYKLLGKIVAKSIYDGLL
LPVYFISPIFKQILQKKPSFDDLEHYNEGYYDTFIKFLNDETQVQYKDLFEFWIPEEYASLIEQNKKLIF
ADLLQDYIEKNPIEIKQEQQSEQDSKQEPLQTQEQDQPKQQSQEQQQQQEQQEQQQQQQEQQEQQQEQQ
QQQQQQQQQQQEQQQEQQQEQQQEQQQEQQQQEQQQKQQQQEKKQQQQQEQQQQQEQQKQQEQQEQQEQ
QQQDSQQQESQEEKQQILEWEQQKYEQEILLQQQLQQQYHFQKYLQIQEELKKEQLLIQQQQQLLDQYL
ISETQKLQFLTQNALQQKQEDLQQQEASLLELNFEQYEQQQQLLLYLQLQQEHQRQSQKLMEEEQQRLEQ
QQKQQAEQAEQNQVESEVMVPLLKAQSEGKQYKIKKESEDITAEDMENREKKRLIRKNKKKQLNDEVVKC
IKLQEVFDKQTALVSIKHKRLICEFISKRIMLEEIKNQISALREGSFDILSKELFSYMDWRDLQKLIIGV
PSVDRGRFISKY

>A0BUY5_PARTE
MKSSQSDSIEEESGNWNLSSPQGPQISLRNTFANYQPDSSVHSYESERIQDKSEEQKQQFTIEFNPDAQP
IFKRTMIRTNPLQKLHQIAAKNRLVKQFKQNLFMNSYILSKDYQDKILQFEQFSQNKNLDYLISKTQSSI
LPVFEAYSNLMKGWDVIMILQQLIQLWFLPFIISFYGFDSNIYVIREFLILMTLLDIIVSMNREIFYKGS

YIGNRQQIFRQYTKNGLVGDIIQLITWFCFLFIYQNIEYKAYLILLGILMVVCCIKSLLRKTEYYIDSYY
NKGNLSNFLDLFILILQIYFVAHYMACLWHFVGQMGIQVEKATWLSEYGFLDESITTKYNYSFYWATMTM
ATVGYGDITGRNNYEILVSNIMMILSSCIFAYSMNSIGNILKSINDSKLNYRYHQTIILQEDYIINQQFX
XXXXXXXXXXXXXXXXXXXXXXXXXXXXXXXXXXXXXXXXXXXXXXXXXXXXXXXXXXXXXXXXXXXXXX
XXXXXXXXXXXXXXXXXXXXXXXXXXXXXXXXXXXXXXXXXXXXXXXXXXXXXXXXXYLPKVLQKEMKED
IAKKINQKIKKLQQNFSHSTSNLLVQHLKIQNYAPGEYIYHQHEQQYCFCYINQGEVQIKEEQSQTQIQV
LKQNSSFNEYSFFTEQATKSNALSIGFTQIVKINRRAFLSILKENQKDLEQFYNIKDTMLLYQDYSLLQK
KCFYCGYIKHETIDCPLLTYQPKKMSVIQKHHKNSQLQSRKYIQRHYHYIPSRKQYQEIKNTISYAREYY
FSDLQMNDSQQFNKIQLLQKEDTNMNEIISPSQKKDEIDQNPQNISKSRLPSVSADANNLRRISKFQIPF
IANPDEKRQSKTQLEFFQINFSGLEIDQVQNYEKYMIQNNLINILKSFQKYKRKTKIATRKQKLIESFNN
ED

>AOC2K3_PARTE
MKQIQDGYSKYIKQSKDWRIHYFWVNLISDIMCYRPIFLKTEMKQLSQSQTVDEETWNQLVIKLPYNKFA
GKLKLFSNNNFTLKKYATLKLFQEYLIQKNQSDIQLLPNYINFNFNAQERKKFQDEDLFIQSLTNQSNLE
ILKALMKQLRYQNDQATSNFVIAKEKVSQHDTYLRGEKHIEFKIFSQELQFLMKQIKQSSLLQLYLINEI
NLLVNKELQNNLIIQTIFQEQFKILGQDSKKIKEQTLKDIQEIENKYASEFLGNLQNVSQFWSQIVQYTT
FSHIELIEGNIELEKSLQIQLDQNIKYFTNINNYLDKFQLQISNVKTKFLKILEQQKHKDLMKESISQRQ
LLDIMIKLFNPKLTLKLINESIQYYQKLLQEEIQIENVKDVRKILIVIQSNINIYKGLQMLFKQHHKKLL
SIQTQLQGAFEKISVAHIKTQQNCEIDIKYHRTAFILEMMDQLQLLFQKYQNQEXXXXXXXXXXXXXXXX
XXXXXXXXXXXXXXXXXXXXXXXXXXXXXXXXXXXXXXXXXXXXXXXXXXXXXXXXXXXXXXXXXXXXXX
XXXXXXXXXXXXXXXXXXXXXXXXXXXXXXXXXXXXXXXXXXXXXXXXXXXXXXXXXXXXXXXXXXXXXX
XXXXXXXXXXXXXXXXXXXXXXXXXXXXXXXXXXXXXXXXXXXXXXXXXXXXXXXXXXXXXXXXXXXXXX
XXXXXXXXXXXXXXXXXXXXXXXXXXXXXXXXXXXXXXXXXXXXXXXXXXXXXXXXXXXXXXXXXXXXXX
XXXXXXXXXXXXXXXXXXXXXXXXFETIRSNQQNTKIKNLQKNNQQTSTQYQIKLFMDWPDQIKTHHILL
LSEIKLQLELLSLVQQDRNIIYIEISKLLDGYLKEINSEDKPNEHQPLTKGIKQSSTNDEILILKLKDFF
DCEGPLNILEKLITKSKKIKSIFRQNHLNAASQNLQTNFDLLNKLKDAQTLSLKQAITDLLQQTLVFCQM
INTHEQIIFDQGFIEKSQNQPNLDQISALLHILNFDTLNFQNQRMEHGVEKVDIIQNMIAELEKDMKIIQ
EENIFDTLSDQSYKVRELVVFNLIKIQTIIQEQPIQEFCENLLKQIWIIEKHSSVRSLLKNEEMIKMQKK
LFSQDLQNFSLKIKKEMQTKFKQIEQLETQVLLSGNQDEIKKSLQQQYDEFEIYLDNITDMSQRLDISLV
FLREISKDLKNIKSSIDSILISVKGVEDDIRRLRGKNFMELLTIRKQKVLYQKLENEQDQIHIEIKTQEY
DPISGNKKKNKGGEFESFLMKSQCDNYDGEVNEFLWSGYEKLKDVMLIKGKAGSGKSRASRNIEEFIWIC
DSISPNWIPIYASLPQLKDPNHNLIEQALESENYNFDGIQIREFKDAVINGKLKIVIILESYDEMKYDYI
GTNLYNRNRVAYDLNLQAQDLKVKFIITTREEILNTIGYQTWFYGSSLQNLKEVEILPFRQEQSSQYLNI
YAEISIKRTIKRFYEFLKQLKGQNFQLEEFKLIWSQIENTINSIILQRRKTDMLFLNSDIERIIKAIQNV
QFFSFIQSNQMLSLKKELLQLWGEQKFQSVVKNVNITHLLSTPFMMEIIVYVLPKMSSLFSKANRIRELL
KKNYMILKKEANNSQVQLEIYQQYSNNSINFEAENYRKSKTMKEIEITQQFTQILEDLDNSNFFESFSIA
DSLECVNNTTIFSNKTFNVRFDANYVVSAFKLQQYTAFDFYEIFVNFYHNQQLQKLKDLGISIKEESMLQ
DLNDFSTFLAIDMSLRQLTQVNYKQKRKLYISSVKEERKVEVSWDDSYFDENQDDFEYKSLLRKCMLINS
KRSLYAFNHKSIQEFFVANYIINLIEIIFSQGNGEVNQNFLNQCSFNKNEFNLSLEHYSGTLELLKPKII
QIEDIKNKLIQITQLSRKNSQNNLKRSASNSIYILGSLREHFEKIDLSDLCIEYTKLDGMSFYQCNFNNS
QFHNVSIDSCNFNCSTIENATWTNLICKEKPSIKAHTSSIKYVTFSDNGKFLISGSETGVIIKQELLADS
EPKKTILPNNDNDKLRTLSTGKNLLACLSNKSLYIYSLSDLKELQSFPATSIYFKDVDIIRFSPNDKYLA
VTLRKENLLQQQAQQFKFVSQQNQDYINRIAISPNQQLLATGGYKVKIWNSTNISDVQQILELPGEKIKI

NSIAFSMDNQARSQNFGIFRILIKFFQNIGLTHQMKQFKYAYSKNGQLFASKSEKVLKIYEVQKLPDQQD
FFRINSELPVDLIEISSDCQILAMSHIQISKNDSPKITIWDIKNLHNVKMISVLKEQSENISALKIRNDN
LVLGSGSLDHSICLWDLLKLELIVKLTSHKVKILDLAFSSDIQQMASCSVDETIIFWDTTNIGKPQIKMT
QQQQLGVKKVLYCPNRSLLVSIQGETYNEIKLWNSAEFKLISTNTNQAQLIDINFSEDGENMLSLNQNTI
LTIWKIDDQGFEIQKQIKIDGEKYVSQAYFLNSQSIIAHQEIQKKHKYGFSNTPNSIQVSQNKKIIVMKD
DGDPNKIEIIVIENHNRYNNVDFQDKIKDFEFSNDASLLAVATDKGAFIKDIIKNQILQNFQQNQCCQLI
CFIGKEQLAIMLEQQLVLYDIQDINSSKTISIIQLYDKPQKMIFLEQRQEICIYSKSSFVLISLLELQQI
KLISFEEDAQSSDVIFDSEQLFMGIGIKQHIQFISLSNAIKIEKIIKLYDTNDDNPHTYTNFSQDCNIFS
ILTPKLLYLQLDISSNQILKKVSFKSYRNPCALNNQETLIIVNDIGIQKKNLFLIDLETQKTVTCFEEVD
QRKFRCLTIVFSQDGQNFVTSYSNLIIKFWDTKSCKLLSTFKTDTNSIQILTISIKGILAQTSDDIIKLW
DLNALKQQQQEMDGHTSSVNEICISSDGLQMVTGSEEEIIRWDFIELKQLDILIKGKSLPSKFCFSPNSQ
YFIALGEQSIHIWKFITKYIIEFHKIYWCDSIKEQKLGVGQNHVIYKNGESKIFIMNLEQISKQIQIQFQ
SDLKNQTRSIILSKNLLIKTNPLEIIHINNKLELKNEQMVGISASQITKIAYCANSSRFAFENEDQSIII
WSIEKKQQLGILNSNENKKTQLVTMIFSGNSKILFSYHDDKKIRLWNITDKFELIEIQDLKNDKYLVYSL
NLHLHSSKDEEYILIWGETINWSDFEVGAIFFQIGTIVKQLGQASVDCKKKFSAAFNVSNNVIALQTESI
LQLYDISQEEIIVIATFEDNPLNDLLCKSNLIFSSDGKILLSLGTDFTVRLWDISDQSSIKVKVNMKKPI
QAVAIQFINSETIRILSESEYLEIGVIKDKDQFDFRAAKQQLGISKYSGKLNNRTLEILDSQTSQLQYTL
NKFSSKINAIQFTPSQEQFILGMEDGSILFYKINQQTIQFYGIPACYHIFAKNPLLSAQNCNIRQSTFKT
IENENFEKVLCEKGAKK

>PPX4_PARTE
MSQSDLDRQIAQLRNCENITEGEVKALCTKAREILVEESNVQRVDAPVTICGDIHGQFFDLMELFKVGGD
CPDTNYLFLGDFVDRGFNSVETFLLLLALKVRYPDRITLIRGNHESRQITQVYGFYDECLRKYGSLNVWR
YCTDIFDYLSLAAVIEEKIFCVHGGLSPSIKTMDDIRAIDRKQEVPHDGAMCDLMWSDPDEIEGWNLSPR
GAGYLFGGDVVDDFNRKNNIELICRAHQLVMEGYRVMFNEQLVTVWSAPNYCYRCGNVASILELDENLAK
SYKIFEAAPQENRGLPAKKPIPDYFL

>A0CFU8_PARTE
MKGQAEQQKGATKTVKKVKTTNKRAPVRLWVKAVFTGFRRSKVQQNENQALLKIEHVNDVASSRFYWGKR
VAYIYKAHSLKNNTKFRAIWGRISKSHGSNGVVTARFGRNLPPRAIGSTLRVFLYPNRA

>A0CP12_PARTE
MDLIEHNTKEIIEICNIKAKKQSYRFKMIFFNFYNLLFFLFLLISSQEISEKCVCGHARSQTQCQNSGLC
IWQNGGCVLSSARTYIKENQDESICKNFAEEDCRIQKQCGFHLGHCNSFIDCLVFKKDQCQQSSYRCVSD
GTKCVEMLECNKYKTEIGCSNKNLNGGYCFWVKDMVWKCRDVIICEELPIFLTSHIMCTQGLKGCTVNEQ
GYGCIKQKDQCTQYFKDFQCFESKEKTQNCFWDIKNGKCVERVCENLPFTQDYECKSYLSECTSNGIHCI
KRKQCSDAQNKFGCVTDAQGNKCEYHKNQCQIKSCDTALDSLKNYQQCQEYDNYLDCVTSENGGCKQRPQ
ICEGYVSQVDCYSIKQQDCIWQKNKCKKRECIDAPLYFSQKDCKQYGNCIGKVDGGCQQTPEVCDEILQE
QFCEFNYNSQSCIWLQGKCTLLECNKLKLPTYKNHKLCQEASSSCTFNLDNFGCKDFICENIQEIEFCQI
DSKGTVCQINQGCIDKKCLTAPPNYESNSQCEEWLPFCTVNVQELSNSKLLIGCVDKKSQCEVAQEEQCY
STSSGIKCKWDKLGKMCIYSICTDADPNIFLTNTDCNSYKVLEGTCIIGTLGFGCQLWSNTCDDLISQQQ
CELNLQDGTKCFWTGSLCKLLECSDASPINYTNNIECNAWLAYCIFNHIMGGCMIRPSNVDCTSSPNDIM
YDTHFECQAWNPTCTVSSSFNLEGCELKKANCSDYIRQRNCKTNLAGQQCYWTDFYQKCMDQNDDGDCSM
RIYGDLSHQNCENFLEKCTVQNIIRTCTNLSYYCDYKLEQQCVITMYRQPCKWDARNKLCKNLLCSDNMT

AQTEAECLKFILYRQCQLKIHSSGSFGPGCEDRPYSCNYITDPLICKLTLTQQHQRCYYSKFQCYAVGYQ
QCEAIEDSKSNEECQLHNSQCVLQSSGQGCYSIYGCQVLSNNVCKSAIMKFNHKCNNYGGRCYLNYHCNN
LSTDNCYGRKAGLGQMCYRECGYYSCDSSCITKTIEYRIIFQSFQTFQERSKLCQDYSSNYRYVTSCQCC
QSMNDCSQQQGGQQLCDSSIAIQSSISSSISRCGYDYQTNTCQKRICEHISYINYPVITDQICFDWGYDC
VLGVSGCIVFTGNCTLIKLIYQCYSQNCYWQDSKCVNHIDCQINTTAVTTRECLLVNSDYCRLNYTKGFG
CAFYRCSHITDETICNSSNLDDGQNCRWINGQCYFRSCEDYLQQSECESSYGSIFQTLTKCFWCLDDLKC
SKNKYCNQSSMISPKSHQDCNSLNYQYTIQFESSVICKIKESFCSSYTYEDACVSTIDGVDCYWSSNICQ
NKCEAVTTNPFTNQQCYDWNSNCMYINFSCKLLNCLQLTVMSDCNIYHTKCFWESSACKRISDCSSYSTS
TVCSNNNNSKGIPCFWNSTQCMEKTCQNIPTTPSSFQDCNSWLTNCQFNEYDISCVEDCTSADILHITHE
QCESYYANKSCTVKLDLIQCVNLPISCGLAKETQCYLDKDGNQCYYSKSTQMCLNLTCSNLDTDFTSHEK
CNQKLNQCTVNATLNGCQQLNDCNTYSIQEQCYLDKDNFECQWINSKNICTIKECSSAQFNEYTAFSCRQ
YFDDSCTVNKSLDGCEVGQSFCMNYNYQQCISDGQINLKGVDCFWNEERSICQERICTNGPSNATSHSEC
ILFLSTCQKGGCRQKGCFDYYYAIDSACASIFEDKRCVTNGFRCVLRNDCQDVSMIDGCTFDINLNPCIW
IDEKCQAKTCQTAQVTLIKYEECNSYLPYCTVKQDGGCTTKQSCSDYEIKEACYTDFENFQCIWDINLSK
CFSYQCIDYCGDGIVTNKDEQCDDGNYLPYDGCYKCQVQCPQGCNQCNGRLCQECNKNGWKLVEGVCTSK
CGDGITVGNEQCDDGNNIEFDGCYQCSFQCDEMCVDCFQGQCILCKDGYVEDGPKCQNICGDGYLVYQQE
QCDDGNRQNNDGCSNTCKVENNWKCSTDNNFSFCSYALLPKIILTRLSKTDTTSQEFKLSFSEPVCLNEK
AISEEQFLQLIFIEILDLKDNDYDIEIKSMISISTQLSDVAYKILINFKTNVKNPVLKVQVNSENIVNNQ
GNTLVSKEAKLEFRSPYKMSSYQLSLMSKTSMLSKIVLYFIIIISGISFLCGNLEILWNLLDLLQQLSYM
KFHNIVFPQNLESYFEIFTIGSFTPIFDKLQIDQNLQDIFNFQIPVIQAKWKFQYYEINCYFLQNLQTLL
IMLMMGFTYYIISYMFQKFLILINYQNWPTVYQKDYYFKIAKFTFFLQKIARKYYQYFIYSGLIRIFTSS
FYELTYSSILQLVNFNTLTTLNTTISLLALITLICNIFLLAIFSCYLSKKNTVAMNLSVLVEGIKSQTNQ
GAKQYFTILLIKKSLFIVNLVAMQGLMGAQSLVTACLSGVFSCYFYIFKPFKNNFENIKIIITEVLIVLN
LLIFSLYEILKLNQDKEQAERLGWINICGFTLILLTTLSIDIYQQFLQYKELVITKVNMCLRISHIKTQN
SRILFF

>AOCPX7_PARTE
MDLDFHSDCQYVTEPTMIISNGNEMLCHPRLRQYIVWPLQFFEKVLFCQNGAILYEYSENTDLVLSCQFV
HANCIVAQKSGGIVKCQTCYEYRSGQYCQVNKHIGCGSNCESCETNYCKTCLEGYSPSSETDFYCSLVAC
QTGHLKCSLTNNVYAFEGCKKGYELVDNQCVACSNNCSVCVMGXXXXXXXXXXXXXXXXXXXXXXXXXXX
XXXXXXXXXXXXXXXXXXXXXXXXXXXXXXXXXXXXXXXXXXXXXXXXXXXXXXXXXXXXXXXXXXXXXX
XXXXXXXXXXXXXXXXXXXXXXXXXXXXXXXXXXXXXXXXXXXXXXXXXXXXXXXXXKGCSSQCQTCLYT
DPDYCTTCNLKEKFLSSTIVPGKCICDYPNGYIEKDGGCGKCSDGQCQTCGLDYYDCTSCKPISNRTLDI
LKLVILTKYVKVCFNNKLECYVSCYDCEGGYENDCTECGDPSIYDKYFQDGKCFCFERTLLEVQPDGNSI
CKPCHPRCERCSMPFDNTSNQYCNMCIAGQRRVLSNDYRCVCQDGYGEDGNSDVCFRCHYSCLSCKGPLQ
TDCISCSIVAHRHMTVDYKCNCDQGYYDPGFKDPNCYLSCHHSCASCTVFGQDQCTSCPSSRHADRVGTT
FQCLCNDTHYYSDPFFLECQQCHLTCKTCKGSSQTNCLSCDTTYRQLIISRCDCYPGYYSTGQLQCQQCH
YTCLTCYSANEDGCITCSSAKNRVLKANKCVCKDNTMEASNTDAMCSKCSYRCSSCIIAADHCTTCPDQS
YREVGTNNSCSCPAYYYDQPDNPICIKCYNTCYGCKGSKNNECTACNPLSKRELSINGECVCMSKYYDTG
IQECSICSTDCLDCITSPTNCTSCNPEKYLLGNSCVCKTKLQGSFLTTYFVPSKNKCQSCHYSCLTCSGP
LVNQCLSCFNAEQRILSGTSCICIENYFDNGFPNCKQCDFRCYECTTFSTLCKSCPQSSLRIYNSLNSSC
DCPNSYYDDGVNPVCQECDYTCSTCKIMSSRCESCQVNTYRVYDSLLFTCLCDTHYYDSGIPICQQCHYS
CLLCNNYGADSCISCQPQTISFRVLNGNVCECLLGYYDNGYSSNCSQCYYKCLSCINSSTYCTSCEQTRH
LDQNQCLCNTGYFENGSSNCSKCNSNCYNCNFNSKQCTECDPNTLRSLNTNNNTCQCQSGTTEIDGLCQL

CDQNCLTCSNALTNCTSCGLMKFLTNSKCTCIDGTYLLNADNKCYYCNSTCETCFGNDSFCQSCSSDKNR
ILDYATHTCICKAGYYEDAVNNSCNQCHQTCLTCFGISTYCTQCDSSLNLTLNYQNRCVCKSGFFFNLIT
QQCEACNVSCSECLTQTQYSLTMTHHNVYAEMVSYSSYVIKKCLCTINEYIIECHITCKTCQIQSNQCLT
CESSNFRVLQTNTCPCLDGYYDVGVGMCQKCSDICKTCQTNSTKCYSCYPSHHRILNQNSCTCTPGYFDN
GQQLCEKCSNSCLTCKNQRDYCTSCDINQNRLDQSIIHKCPCVSYFYQDSNETSQKCHVKCSGCVYERDN
CLSCKFVQGSNRLTISNQCNCKDGYYDDDIQIICKKCNNRCKTCEKDSNNCLSCLSNLKINPPNCFCMNG
YFETDQLNCERILFIINQACEIQCDTCETRASNCITCKEGRINQKCDCEEGYFESGQPLCIECDFQCQTC
SKFANNCLTCKGDRYQIPVCRCQDGYYDDFQSLNCLKCDYTCKTCTLNKCLSCNGNRILSDEMTCDPPPN
SVSSLLTPWCSNCEVAVLKIKLSDDLKSIFVHFDFPINPNFFSSYLDSNTCFNILKQTTLLKLGLNPQCK
IDPNNKRQLILNLGHNPTIMLEDSIEFLENSFGHNYCDGKLQYFIFNTLEQPSNPFAPLIKIQQNIILIE
SKLYDGLRSFVSISWSFIVQGQNGSADMINFVTELTNFQLLDLTIPEKTLPIQSNITLFVEVANFVSKKS
VFEILIETHKGQFPSIFQKFKQSYYPFESIKMDFTINKKNCIENLQISNNTNSQYQINFSEIDRNDSRSR
PSNIKFDQIISSNLLELNIERYSLTAYTAYTFLLTVSDSSIQYNSQQNITIKILSGGILCQFNGTKKLQN
YQSATNIYILCKDLDVQYDWNEDPDLTIQVSCLELTSQEECKDSQKRKLQYNSTITTQKFPKGTFQPYTI
QSWDVIATKNSLTYSYNINIVYMEYDFKILDIDYNSGYLVRPVNNYEDLQFTFNIPFQERQYLLDLSVAI
IYDYQLISILQPQYFKYSFQIYDHFQQFNKGNKFNLKFLAQFTNDIIPNQEDLILLLNQPPICNLKILEE
NVYALEPLKMAINCEQSEDQPYNYQMKVFLLNNDLEEYLNKSSDNSLLYYSFQQSNNFVVYFPSSEINVI
LQIIDQRGSITNIQRSLNISKKQVTYQLSLIQKISWIFEILLNQNDKQNCTILKDELYNSVEQKLNSEAM
YEKLLVYQTINLFKKLSIKQANSNSTKKRLLEQKYQNQCYNNETSLFIITNQEPTEKNAINISSLIASSQ
KAQSQIVDLIQMKINLEKQNTQNNLIIDSQLVTIIKSVIQVLFGSVQLIDDQCLIISQNETSAEYQEEVM
KISEGLIQLIENITIQISDNLQVNGKVLSFYGAMLQLQLQKITKSVYNSQFQIQYDYLDNLIAFIQKSQL
KISFNYYNLSQSYRSMLEIYLNRSDFEIDQKYFVKSLLTNFLYTGTQINQLQLNTHYKIDMVEFQNCDIS
KGISEIFKYNYVCINQLENNQFEKCDVEMEKIDNQTTQLYCKCQFFGNLFLIKVANNSMNQSNDTLVNXX
XXXXXXXXXXXXXXXXXXXXXXXXXXXXXXXXXXXXXXXXXXXXXXXXXXXXXXXXXXXXXXXXXXXXXX
XXXXXXXXXXXXXXXXXXXXXXXXXXXXXXXXXXXXXXXXXXXXXXXXXXXXXXXXXXXXXXXXXXXXXX
XXXXXXXXXXXXXXXXXXXXXXXXXXXXXXXXXXXXXXXXXXXXXXXXXXXXXXXXXXXXXXXXXXXXXX
XXXXXXXXXVMKQVFQYQRLLGESYLLISLLIILVFRAISKINQAIYMFEGKIAIMMIALYLFLPVTYFF
LMILAFNQIEMNQNYIDLQITLNLLSTLLLIYFIYEPIAIYLRIVIYRPFFESIRNNEFNPINHFVYFFI
YDSKINQTYDQLVI

>AOCT56_PARTE
MFTDVSGFTNLTESLSKLGNEGPELTAFVINRYMELLVKAISQSGGDILKFAGDAMIVVWPPSYKTDIVQ
VHEELRLTCRMALQNALDIQNKLNDTCILPDLKLSVKIGFGIGDINIIYVGGVYNRSEYLATGEPLLQAF
QSEHCATKGGETIISKEVYEFVKDYFTFETINHENKQFYQVKKLNAYSKVKLKAEATLIKNSIILNPKIQ
QQLAMFVPAALKPYMEIGLERWGSELRRVTTMFINLSIDLSDAKTDSGLQRIQNVIKTVQYCVYTFEGSL
NKLLMDDKGSTCIAVFGLPPVSHQNDPVRAIQSAFLLESDLKKIKCKVAIGISTGIAYCGVVGTSGSRRE
YSVLGDCVNLAARLMQIATQPFSPCILIDQTSASDAQTKIQSIFWSERKVKGKEKGVLIYEPLTLQEDFK
KIKDINTHLEFRMMNTQSMVSLSNFNSTFSSKSLQYHNNLQSVIVFDKYHSHCREYDEELQPVGRKQEIN
QIKNFIQKFLDEFKKNKHVQNRLVLIKGEYGIGKSFLAKTVLKNIESLQNKEALLEIMISSLNPSSRSKL
LNSIKIILRNIFLSYAQRLNKAANIELIQQIYECDSHLANIISQVLSLTVNDIKANTIREDLTLAKSIKK
FVYRLFSLYLEQEQGQIQNEMQNEDQIQEYYSQALIIMIDDMQDTDDGSLYLLKSILKNFKNILVVGCIR
NKFKEFSFFDNSNNQSDSQSEFLTKMIKQQTNNLTINLKGLKQTEQLQDLFEKFFKIKSFVCMEEDQKNQ
DQTMFARRKRTSSIDDIYEMVELLKKVRDPLMFWESKPELEEMDFNSVCINYLYVITCGHPLKVIYLMKH
LLNNHYIILDEQRNLGMASKKFRTLIKHEEWLDIDSPLMAVQINGPYIDKLTALQQLILKQACVIGDIFD

IQTLNYVNPFKDFIKQKLLINELNSLYEQGLLDILNMEVENIYYRFSYSFFRETIYQRMTYAQRRQAHKN
VALALQKIPQPFDYHNNKEYTRMQYHWTQAQNRNSVIVDEGTLLPKLTLYEKKQLIIKKIKQLLDQNRRN
VLLKSGYINKRSEKGLQWNWRFCVISSECLIVSQEDDNPEHILTIYLKHIQLIEREFNSQYFTLTVTTSQ
WSRGNQVFENYRKVQFALETEDELNEWVTYLEFAKAYAIYMDFVNNFGKISFPLSSNEYDFQLKSELQHD
NRRIARLGLIDSSMGSEKIQVRHTKASTSRPTMQRKHKPRKSSLFADGYRGSTSSSQQNTSLQQQKGYDQ
SELVLNIKTLLLNAQLQLLALIVNKATSQKQIVLGQKLF

>A0CZW3_PARTE
MQSRLFIQKQYIQFNNRQNPIAILRQIWKGGDNYYQIQLNNNIYKCSRSSSCPQKIIQLYAKYQNKSQKV
KKFQLQEHTFMFEDNYSPVEIIGSGAYGCVIQADDKNAKVEKDRQVAIKKIERAFEHRLYAKRTLRELKI
LRLMKHENIVELKTLLLPKSREEFEDVYMVTELLETDLAQVIKSDQVLTDEHIQLFLYQILRGLKYLHTA
GILHRDLKPRNLLLNRNCDLKICDFGLGRAMADPSSSNNANIMTYYVETRWYRAPELLVSFKNYTPAVDM
WSVGCILAELLLRKPFLRGDSTKRQVKLIFELLGTPNEAYIQSFQDEKVQNNLRKVIKETGPKQGIPLEQ
LFKNASKNALDLLRKFLTFDYRQRITVQQALEHPYLAQLHFEADEPSAQLVNQLEFEFEKYEMTREQIKD
LLYEEILLYHFPEFQTSYEAKKKSGQSLISHVVNNENAKIFDPTADDDLDRD

>A0CZY2_PARTE
MNQDHVSQYTQSQTPHNHEPNPQLSSNQSLHIMNPEQLQLIQEIEEEQIQPQKQRLRLSLDSDRDRDRES
VGNQWIKQRPGSSSPSDQKTVKNITNKSGSQALVSGNGRSSKTPQDFQEEIIPQVRSILKQGTFISHSSL
PRSRGLRLSRLQSLHSLPQSIKKNRVYPEEIRDNIGSNQNDSFSDDSFFEETEQNELGDETRYRFDKRYA
NLKYIIFPDDPTKLFWDILVVIALLYICIMVPYDISFKDDNQEETPTQFGLGLAIDLLYGIDIIINFLSA
YVDDQDELVVDKTTIIKHYLKSWFLLDLVCVVPLDYILDNNETSGYQKFAKLPKAYKMIKLVKMSRMLKF
CVQKKKFGELITSFSNITVTIFVLKQQNIRVMIISLFSVILVSHLFSCFWYFIGTVSSESETWITHYVED
QTNFERYIMSLYWVFQTMATTGYGDISATNSTEQMITIFIMIIGVVFFSVTIGSVSSLLTQLDTQNLKYK
EKIDTLNEITKNHKIDNALYAKICKVLKQGYKNNQNEVVEFLHLLPQNLRTELSQAMYKNVFLGIDLFKQ
KPLRFTAYIGPLLTILRIPEGDVIYNEGDYASEIYFIREGGVSLCIKECDYHPFVTIDAGQYFGEIELIK
ETQRKYTAIAQKQSELLALSKSHFFKIFFSEFREIGEELHEDARRKKRDYEDKYTKTKAYLQGLEQKAEQ
QIIEANNQPKQKQGLEQFKKNLIFQAQTKKGFLDKAIKDAEQLASCELKRKMTKLKTVLLQKGIINKFDE
NSPTKISPKMMKRNTLNSEYQPRKIDKRNTFLPMEILQQAQQLSSEDDNKQSQNNQQEQNAFRFPKKAMT
AFISVDQNQQQEEFITSQDGMNNSQHPSSYNTNIQFLKKRKKQLYL

>A0D7E8_PARTE
MNQFNKEEYLFLAKIAQQTERFNDMIEFVKQIDELELTKEERNTLQSAYNNVIGIKRAELRVLQEIEERE
SNRQNDGQVLLYIKNYLIKIEKELQQKCQEIIILVRDKLLQNAKQTESKVFYLKMIGDYNRYLAEFHLEN
DQHNSIDQAKEAYKEAVLMAQTKLSPTLPLYLRLMLNTSVFICDILSDVEGARELAKESYEKGILFLDHV
KEENIKDYQCLLQLLRNQFRNEYIKLIMNKFSREEFLFLAKIAQQTERFNDMIEFIKHFLDQELNKDERS
ILSAAYKNVVGNKRAELRVLTAIEQKESRKQTDQYTLNYIRNYKHKIEGELKNSCAEILNLIDSTLYPNA
KQVDSKVFYLKMKGDYNRYLAEFLLDNEYHAAVEQATQAYKEADVLAKSNLSTTSPIRLGLHLNQSVFYY
EILQNATEAIRIANDAFEQAIAQVDTVNEENYKDCTLIMQLLRDNLTLWNNPEEEANDDQ

>A0DCA3_PARTE
MEVELHQQIPPQLNVQVAIRVRPFNEKELKQKEGVCLETSEKQVTLLQSARLFTFDYVFDQNASQDQVYK
KCVSNLVQRCFEGYNSTILAYGQTGSGKTHTMGTTGVDQLANKNNMGMIPRVITQIFEEIEKIDQEILIS
CSYLELYNEQIIDLLQETSISSQPTIREEKDRTITILNLTTILVNNPNEMLQVLNRGAVHRTTAATQMNM

TSSRSHAIFTIYFKINPKDDSEDGTLNAKFHFVDLAGSERLKKTLAQGKTMEEGININQSLLVLGNVIKT
LSDQKKKSQTHIPYRESKLTRILQDSLGGNSNTCMIACVSPAESNYEETLNTLKYASRAREIQNKPTQNR
DPHAIQILGLRQEIAVLVEQNKQFQELLQVNGVKFDSIKKVSINQYSSNIPHTCEEHLEMIQKQKQALLV
WERLGSQHKLEVQKLQSQLNDQEIELHNLKKKKDIIFKQFQEAKKILQKFNIPFTNYDEDDEIEDLYDEV
DKLKQESKEKDYKILTMQKEIDELLQVVYIVQILGQDAHRDQYILCKKQKELMVLQRKLEKLEPKDNFES
PAQDSECDEEVVCNDSMIKETNLQIEEFESNLQVMTIKNFELKRQLAEDMKKEFQQQISILEAQKSSLMK
QVNQKQDATQLNALKVKLQEYESKITEMRLKESKYEINVEEIGRIGKLGQGFKDQYRKNEKIESRVGQEN
EDKEEKQKELILIKKLKIQQDAALSKLKNENCKKEVLLRRKEDEILKQRNEKVIDKGSSLSFKKSQKAQT
YDSMEKQIESLFSQLISGGQAEDQIKREITKLEQLQEEVNQIEQKICQLQIKREQTSFDQNNKNSLNQIE
LELSDIIIIRENINETIDYQLHKIEQLRKSCNNCTEVYTNFLTNSELRDLPNWCIKIFKYVIDNWIKDHL
QMQNFSQQNDEFQQIIQELQQQLQKPLQEINCNSPTFKKKIIKSQVSSQPSDALELRKQLNDQKRKYQFL
QNENKNMISDLEYYKKFYTEHINKVQKDKCIGLSDSFQLPQSQSYQQIRDAYKLERTKQKELSLNLSFQK
PYLQNSALKMAKVCKSDREGYTDQFSQAQEEQELRYDLIKSIPGHENPILCVYTMQNLLCSSAFRSVKIW
DMDAQSHLISLDANTHVKSICHWPERNAIAVSHGSLISLYDMTSFQLQSVLKSSIEEIRVMTRHNGLLVA
GGKGINYAMNVWDARSNNQIHEFEKQSDVMCLQQSDQTQELIWGTFNHNVRKMMMNSNKCGQIAQMTPPH
YDKVTGVGLLDNWIVSCSFGKGMRMWNQVNGQQGQANSDIHKDSILTMAIDKNLKVMYTGCKDGQIKATR
ISENKFQLVSDISASVQQINSLNIINDSSLIVSGGQDRMIKIWKPSKFTFDYLKQFTPNIGDFIIEEEQY
MFS

>A0DHD2_PARTE
MSLNSRYVCNSLYKSHQENLNQIIEQYCDQASANSTECDQSFYEGFKFQVAHQQISDLISQVERSSKLDE
GKRQLSTQILSQAQQILKNYSSQECQIKIKITQSLFNLQRDTIRTIQEVHEAFALLESIKWPCKGPANKQ
YDKQLRALEKLCYFWEQVLMLENRATSTISTNEFKQRLNQWFEQFTNEIRTKNFDNLKKSKKGYLDTQLR
NAHEMYVGLGIEKNRDSALQIYKRLSEESHPIAQAIMGQVLMDGELGEKDYDQAFNYFKLSADQGHTFSV
YWASRLILEEKVFDKFFEKDEQQSRKSSVTTIKTKDCDLAIKLLRKAAELNHVPSMIYLGDLYTSGLQLQ
NYSLEKDYQDAEYFYKQAQKRNSVEATYKLSLLYQEMSKQSLNKNRRQLIFPLLSQAKNQDYLPAFYDLG
MLLLNGLGEELQANPIMADLIFEQGAFNGDFKCAKKLLNLRFQNLQREEGQLTDFLSLLDQLEDILKDQT
VINYMRGKIYYKGISCEKNLQKAVEQFRIGSWRGCLKCKAQLEKIFKDKEDLPTSSPQSSIQKLNIQGGF
IDQVKLSDQRKAYQIQKGKVSLNNKSIPFEQENQSTRQDRHTIMTISIVKPNEANDPDRKRGGSEYHSFK
RAESGVMLSQLKLQIQSPQQQSSTRFEQQQQSQYGSPRQPSLINISSNSSKKDLFNFSKFPSHSSINSSK
YLFNQP

>A0DJ92_PARTE
MFILICAIFLILQQINCTWVRISSYVTAIKQFSNTDTDGFISQFGNPNIPANYMTCSAEGSYITLNAQSQ
SATYMNSFSANSYSMYLITYDLIFFNLWNNLDFVLYSIGSNQKQITYNEIEQVSVSKGFCNNQVAIIQSI
NTTINSTTISGYHKFQINSLSASVSIKNLVVSALQCHGTCRICFGSKFNQCTNCFLYGTLTPENTCTYSC
PSTAMYYVKNQGCKPTCQISQQKYVKGVCEPYPITNFVGLYIQQYKSTEQFRWQIIYDFQQLNTALPENR
QYDGYFIYGLFKNNQGLYRVLTLPTNPGSFMIAFTLELVLFNKMPPLSSIQFLINDIYYGQLYTDDQGIL
KADKLLRYYNETRQNTVFNGISYTDNLYNILYFYANITNTGTIRKFIQYFRDANAGWGLRSFYVYSGYCP
TNCQRCDEKFNCAQCQGGYLKSKWGTCSLCLGSYQQKINTTHCQEDDDQTNCKDHHYNLDSKYIIKEFIG
LELDLEAYPQYTLISVVGQNFLKGSGIYYSIWKSQYRIFGGPFIWAQAKFQRVFAIEDPHHAITISFIVI
FGPNFPEDGSFTFGLDGIPQTELTSSSLAEIPIQHKQLHNFNSITIEFECKGINNEPIYGYCGFYQLYVT
VHQCKPNCNSCSNDSDCSDPNYIEQIILCSDDQYFDQYQQKCQSCPTTCNTCTSFQNCLTCVATFSNPTQ
GCICASNQYLESNICNNCPQNCNQCISSLKCTECVPSLFRILVDNQCVCQDSYFEENQGQVCQKCIQNCL

KCSYLNQCEICLVGYQLSNIGTCDQNPNTYFMATLQKYLDCPVSSTCNPCQGINLSCDCGDMIITGDEFC
DDGNSIQYDGCFECKFQCQPQCTKCIDGICYECATLGWYLDVSQSTYQCKEVCWDGLVVGNEKCDDAIST
TNCKDCKYYCRTDCVDCDYQNGKCIGCREGLIPENNYCRNICGDGIVVSAIDISFNEECDDGNLFDYDGC
SKICQLQCQNISICDDCQNNKCFHCINKYKLNINLHRCECDQFCQQCDLINGQGCVLCKPGFILIDRECV
AICGDSLVTPVEQCDDGNLIFGDGCHQCYFSCENQCLTCTMGYCLVCAIGYQNIEGRCQELYIQTSSVVQ
PKNDIYSNALLQSFYQDYQYNNYLNQISLEALDNKSLQQIQQDEIVYFTFLFVNLIQSSNQQIFFDADLS
IINYQRNQESIMYSNCFMLDNKCMNHCLEFTKQEGLILKPLEICQYEKLITIEYLDDSFQVCNGNGFECL
QKCPIYCQICSQDACLSCNAGYNLDIIGNSCTPVCGDNLITVEELCDDGNNLIYDGCSLCQFQCQDECLD
CQYGKCKQCQDTSRCFDRKSCNEKDGLYYDDKNNQCFTKCGDSIKAGTEQCDDGNEIQYDGCFQCLVSCD
KFCAVCQLKGCEQCQTGYKKIDQECILDCGDGLIIGNEECDDGNSLKXXXXXXXXXXXXXXXXXXXXXXX
XXXXXXXXXXXXXXXXXXXXXXXXXXXXXXXXXXXXXXFNNFKLLQQNLQLFILYSYIFNHIQFNIFIIL
ILQIIIYSQILYFINYINFKRLYQNIFFFQFNPECADCELKNGQLVCLQCSKGYFISFGECLKCSEHCLE
CQSSPNNCTECMIENCQKCDKKLGYYNDLSSKKCTTICGDGIMAGTEECDDGNNFNQDGCNQRCEIEKGS
QCVLNFCNKKEEKQIEYHYSNSSTANSLFLKSEINFEDCCTRIKVNIEYFNSSDFNYSLMPIQLKSNNKS
QLDTKFHECQIQFNFFKTITEINLIHLLIPLSQSSSRLLKEDDIREIIVTPRKQVYYNQAQIAQAQAVVS
TSNQLQFLLQLIGPLTILFGGLNFFWTILDLLTWINNFYLLNVDYPLNVKLFFQHLQWDDIFNIPEFISL
NQPTDPYYFEAPPKFNEKNINPLFLNNIQVFSCLILLAILIYLLSLTVIKIFEKKFTKINVPRKSIAVFT
VCYVESNMQTQEQQQPKYPMNEKLQRLPSIVVLVIRQSYKYKLTFLSKLFAITNLLLLDIFMACLLQLIC
EKKYDYFIVTINNFLAIFGLIFILGMYQLHEFVSSKHQLLLNHFSFSNKYSSIYEGIDYENKIAKKYCYF
NLIRKIAFIVSLVILYEKPVLQTTLCCLSCFINQAFLLYQNPFDSKIQLIQVGVPDFSIFSIILLTVLIS
VDDLTNILSFDQKYNIGWAIICLISLSILIQLIFLLIEFYKNLLANLRGLKQLFCK

>A0DLP9_PARTE
MRLIIQRCLSGSVSVGDQLVSQIGKGLVVLLGIHERDTKEVAKKLAHKLSKIRLWEKENKAWNGSCVDFN
YEILIVSQFTLYAYMKGNKPDFHYAMDADKARDLYEYFVDECGKAYKPEKIKKGAFQQYMAVNIVNDGPV
TIEIDEMEVEKQQKQQKQQIQQQQQQQQQQQQPQEEEDNKIQKIN

>A0DLR9_PARTE
MDIQNLIQQFGAGQAVNPEASIPDTAEQVTISALALIKMLKHARAGIPFEVMGLLLGDIVDDYHIRVYDV
FSMPQTASECFRGICGAQFFNKKMVELLNLTGRMENCIGWYHSHPSYGCWLSSVDINTQQSYEQLNKKSI
AVVIDPIQSVRGKVVIDAFRLIPQQNMLSQQEPRQTTSNTGHLQKPGLEALLRGLNRYYYSINIKFKCND
LEQKMLQNLYKNSWTEGLKCNSASENSKRNESCVEDMSKLALDYQKLIEDESKKGEQETKIKNTGKKDPK
RHLGLKVDELLDENLNAILGRMMATKGF

>A0DMR3_PARTE
MSFLFCQSIQLNQIRDLSIQIVFLYCYQLFKGIFFIVVQLSFQLNICLLLLIKYIRFRIQQDVFTLCQFQ
NIENYFRFNQVLQVKSSIIKKKTLSRLVSQFNNCNLMYFCRTLSEILLLSTIFQMVKSSCDVFKFITTPD
TGAASVSLDLSTTLYTDFGIGVWTRFQSQLEDNDSLNKKILNHFVFAQLLDGSFPLIYFIKQDREAQVIY
FEVILQNDQYFEMRELELKQDLVGQKWVFFYFCYSKSIKQYNMFVYVDKDTINVQMAGGGEYLNQHNLII
PFTVGGELQYSNEKFNVNLDLSPFAGEISDLDIRTDVQSFYPNIPTFLSHLEDTNCELYKCPATAQTTVL
TDFYNGGFILNKQVTFFNKRFVLFGWVKLNELADIRSLETILVRATFRKIYYGDKYQGEKALYWKYFQST
MRDQDNGFEVSTYHDVKGASNIKYQTLESDTISQKSEYFQSAITSWHWFVYQEGLPNSKIELSIYFGNYK
ELKTYSMTQIHYEKSTYYFNIGGDKFIKTFSGEVRNLTFSYCQANDNKPDRYCHYSCNTCFGPNESECID
CKPVSKRELNGTYCPCIINYLDVGLPTCINKFTVLGGITIQEGLIEDSSGCQRNQFLVKMKGNSYCLDCP

GAITPYGLYCIDCIYNPNTWYLNPICKIDYLMPFTDKTTYVFQRRDRKKKDYEYFLINYNSIQDQAVLET
CFGCLGQTDSTINFIEKYTMNQLSKIICKNCYVSLNGECVNLQPYCDECDDNQLCTTCQQNYTLFQSKCY
QCPSYCPDCKYNSTGYYCNSCIDRYYYNSTLEQCQQCGAFCEICFYQSRLNLLQCVKCVDNEKYFVAAEY
TQCLPKNLDNCKYQVQEFFMTLSNQTNVYATYILSIDLHSQYFSYPTFDLCLKCNPHYVNFMKCGQQYGC
YTVDEIESKSGKPLPQALRQKVLNDPKFELGYHEKQLKDTMENYYILFGDSVVDNNISYNPIVLEQKEIG
QQVCRDQYCLYCIQNYVYSQEYCVKCFKPYYAHKLQGNCIICPPGCVECEHGNKFYKDGWKSDLLPGYQL
RTKQEWHHFNLFVLSTTIDEYEVTCLKCAEGLMQQNGKCYPKCPCESCIIENEQVKCVTCQNSIKTVIND
KCTQCPQFCELCREFTPQEISQINPYFNQQNPAYKGYAKQCLKRDIQDPPQGILYNDPSLGQELNCKNAD
QKDCYFFVELQQQVHCGTTQFQQRLNQETDEDARQYFMKYNLPLQNLFSSSNSSHFYIETDYLFTELNSK
SVKLIRYIVNILPNSGDCIINKNTFIQSQIRQNVFTVKYIELIIQSQNDIPIHMEGDVSFLEFSTVTLKN
VQIIPTSKYLSLNVNSVFGVSFLIDQFSIKNAVDLQFQIKLQNPNSISIKNFEISDSKLNNIDGIIRYYF
TQPIKNTLIYSIDTILIQNCQILNSNLFVQILGTDQGNQKFIANNFKSYNNKYTNSIVLYTEFPNQLRES
EFTIQQFLSDQEQLTQSSLFILHGALSVILTDITVQNGDFFAEVKLFKLPLFTINNMLIEGNTFESADNR
VITNVVDALYQDSISISSLTLNTIAFKNNFYQGSKVFIELIQSQNFKDLRIQIDDLSLESNQFTLQQYSS
LMTSSNSSIYFDVNQMTILNLNIVRAFTLPEISINNVDKLVLKNIKATLNSGFKIQLIHQQLSCIQQSLE
IGYGSMLQIFNTKTIEIDKLQVLGLIVLNLPIITIKSLDGSNYRQKETIMIQQVQFSNNTMILTKLAEQP
TILSIISEQQQEITLNKMICFNNHHHNYQEDLLFKQSSTILIQNPNSNIVLAESTFSNNVLTNNQGSNLV
LISNTLKVSNCIFQNQNDLQFKYLRDRLIWGYNKGDVVYFENLYSLFSIKTKGGNGYLSANQINLQNISS
NNSLALQGGAFYFGTQSSGSIAIQNCVFNFSQANLQMKEKSQGGTLFIDASQSDLHLSILNNSFSNSYSR
NEGGTIFIEPSRNNNSIIIYYNTVQNVFSFQNAFLKLPVTFSSKSLILQFTVFDLSIKNTYIGYLEYLGK
IQNLSSSEISLQTQNYLISIKRGNLTLRSCNIFDIFDYGALEVLEASQIKLDTVLIQNITIISGSILSLQ
LNKKYLTQVQLINVKLKDISEVIGDVSLPNTPISTVPESFYKCDLTSNQPTSLQALYDQEQRYIISLNNF
YAIIKNKTNPNFIFEIDSVSANHYILLDNMNIQKINCANCQKGVLKFTNIDEEQNQNLIYINGFILKDNQ
CGIFSCFVIAAKSTNEIQLFSKFKSNRILGQEKAEFDKQISTIKLEKSLFDNNQATFGGAILISALSSVI
SNCQFTNNIASSVAGALYFDYELDTQLIVYDSVFANNQAKVGGALYLSDFQMQSPSKMNNVFQGNQASYF
GDNLANQPTQLTLQIGSKIMQKKILEKNSTNKTEIIDVKQYKMGSLEYDNIMLPSGQPIGGYQIFDEITQ
SYIPYNFTFRILPLNDENSQIKSLEGSKCFIRGRQIIDKQEGEFLTNFTSITEVLFNSTSQDYNLDQMEI
TFDPDYSSIGYLQLEITCNSIKIPIFEEKPPYLLQDYFTNYRLRVNLQTFPCQRGEYKTRLGTCKLCDSN
ADQYNVKAGDQCQIKDPIKMQQVSSARVMLRPEYWRPFESSDKIEYCLNLPENCVGGWNPGNDLCSQAHV
GALCEQCDIYNIRGQGAHSVSTSYKCGSCNNIGDNTLKVILVSIWTMISIFLSVKGTVETVDKMITQSKM
HHLKIFRKDPKAGYGSVLIKVLTNYLQIIGAVSTFQLKLPSALQSSVRSVGNPTEAMSFSLDCFLVNLVD
INIIYFRMIWALFMPMLYIFTFLIIYAVVILIRLAKPNKSAITTTAIYLFTYLQPTLIGGFISLLSFRQI
SNLYWIQGNVAYRYDTQTHFNWILTFILPSTLTLAFFIPAFMFISLYKQRHHLDKENTRKNWGYLYNEYQ
TEAYFWEIVKILEKGFIIIFLTFYEDLIIIKGALVFIIVFIYQVLTRSYRPYKLPFLNLIDEFSTLICGT
SIVIGMTIYQSSLSNNQEIVWPFYMLLIIFNLVFIIIILWEIVLAQLEDQQENIDKVRDMINKKYPRLIN
SNWIFKRLLTNRGQQQKRVKNRFQMIRRYLMKLVRNNPGIYKLPTPPPSIQEIPFINQEKQNLLPAHQQS
NQENMIAPQSNGAKVFPVDHDQRTFESVRYDDKRQSP

>A0DPG3_PARTE
MVVKALAHKRIVHKRTKRFVRFESEDYPHKLRPSWRRPRGIDNRVRRRFRGNRPMPKAGHRGDKKTRYLD
QSGFRKLLITNEKDLELLLTNNRTFAGELAHNLSARKRATLVRRAAELNVRLTNGKGKVRAEEKKE

>A0DVJ0_PARTE
MYNGMNMYHTLENWQSIKCTIQVQQRTVSVMLQNLPFKDPNKKGNPNLESLKVKFILIPDTYIEGVVGQR

IQLKVYEADLCIRFPNVRYLSKGDPYVTFFLTEDMERNYNWKIKKRLSNIQLVNDKFDLENKSYCQKLQF
NKLDLFKNQVSRLTLFIIFYTSVYFPIFSTQNIFFSNIFNLIYHLIQQIMQELGDIENQPHKKVKTNETP
LEFTVLATHNFARASIMRLPHGEVLTPVYMPVGTKGAMKGVTYSEMDDLGCKLLLANTYHLAYKPGGNLL
EKVGGLHNFVNWKHNILTDSGGFQMVSLSQLSEVTEEGVTFESPYDKSTMHLRPEDSIHTQNQIGADIIM
ALDDVVRTTTVGPRMQEASERTTRWLDRDIAAHKRKHDQNLFPIVQGGIDPKLREQSLNDLIQREANGYA
IGGLAGGEDKVDFWKTVAQCTATLPVNKPRYLMGVGYPVDVVVCSCLGVDMFDCVFSTRTARFGTAFTDN
GFLKLKNKEAANVFEPIQKGCQCQACKSYTQSYIHYLIAREEVACHLISVHNLNYLIQLMLNLRQSIIDG
KLIEFINEFLKKWFKNEGQIPVWIIEALEYAKIPVEQL

>A0DVQ0_PARTE
MRKKVSTLQDYEECKQQLLLDYLSTLEEKDKEKLLEKLESINIRNLIDVYSHYKEKPNENRELNPIKNVL
RVASTPKDTLQQYQKLGEKLISEGKVCVAMMAGGQGTRLGFNKAKGMFDIGLPSHKTLFQIFCERILSLQ
NMIQSRIGQCLPIQFFIMTSDVNHEETTQFFIENNYFNLQSDQITFFQQDSLPILSINGEIMLSNSTAIL
EGPDGNGGIFSSLYNQGYLDYMKCLGIKYIHICPVDNALCKLCDPIWIGYVESKNLTICSKFVKKAHAEE
KVGIHALINEKPCVIEYSEMTQEDLHKKNEEGELIYDAGGIAQMICTVEFAHKIIEDPQTSNNYHVAQKK
YDYYNINQRQIVKPDQINALKFELFFFDCFPLCPKEQFGLIEVKREDEFAPVKNAPGDKSDTPETAKKLY
LDRDQKWLKYYGLQFPQQVEISAKITYFGEGLENILPKYLGNKQNPLIITNDRQSAPKSPQLVKQPQPKQ
QPLQQPQQQQQQQQQQQQQPKQPQKQIQQTSVLNQQIYPQVVYSSLINSVHPTVLQPNYYPYQSTPQNS
KNILVQENPIHQSQVLNPRLSMQHQTQSQQQLKQIKANAFPTQGIAVATPKLINPQTRYQPIMYSQNLLN
QKQSIIQPVQINSSLSPQRVTSYFKQRAVTVQRPTMFGQQRPLIISAQQYIPIATIKK

>A0DX60_PARTE
MNTEELKEAIKRIGQCQQEQVECGSNRSQQDLEIVWQQIQHPVGIGFIVSMCKERNRIQTIHNSANKILM
HIVKVCLNNRDQLKAWETLRLTIDPEKQFYKCHELVQMDEDQFSHLEFTQQEFCANLEEGQDVDALYEDE
KGNVIGWCRANIVKINEKYVWVKWYENEEKRKIARYSMDLAPYKSKVTDDEWQWRHSLTAGDVIDCFDNR
VWQNATIVTAFNEEDKEYVVGFRVYDEKGNQYDSLNRRYFGWNQQYDERIKAVSPRIQKRNAFSRGQPSN
PHCQEEIIHDCNDFLYPNTDYAVPRFQTKQARRSIVICEMINEFGRAGLFQYIMDQILNKCTIDFLHSYI
VLLNNFQEMLNREFVQTYIPQLFEAVQNNILQSADNNLRNFSTQKITDILQSLSNLLKRVFALQKRQEMI
DRLDLDIAYKCFTSDFLERKIQGLKAIQELIKKTKDQYNQYGMQEWQKQAAIQMILEWLNEKKIFESLYV
GSGNSHLVQRSAEFFKFLIEEKMISINSFKDIWYSLEKAEYEHKLAIFKLFKDVSNSLEKEWLDFLTDAV
CSKDPKDVTKDDLELLLDIIKMNYRYKDEYIQKCCNYYWTVLKSGGQLNQTMLEQYISYYIDQVTQYEMRP
HKGTQISMIGESIVNGEQVNVGLKVLIKLIEKLNIQPEAWEQYTRNVALTDLESKFKIVEEIIKSIPELK
KQKDYLDEIKIRMQFIQFFYQNISTYEYRLTFNIISSIWEQLVCQSSSQQEKDLVYKWFSALSTQDGQSQ
LTSMVPLQDLKSFFYEKMTNDLIQLTEEGFNCFKTVMTAINKQEDNGFGLDVLWQMILETENEKVSQSAI
DFYLSFESTLDVLFNKLQENRNNNQKLHRCLLVLEGFIDQSEVNGVGNLKSLNALSQGEELQIQISYEHG
NQKKFTIKINDNQTIIELRLAISKVIKQQWDSIGLNSLKGEIKFTENGKMIKDLRLKKGEIIMVFRKQVK
EIQEANLLDGDVLSEGAKQVFGEIFSEYSSEGKMSKEDCTRFVTGCTGNPCSIDDANIQRTFEQYDKDKD
SILTLSDFLDFYTDSARTKKSTVWLNLQTLHYRNDLIRGDRVPLPQVNAQLLPRGQIVQNQRYLDLLFEL
LQNSSNEVQEKTWYLLRRLPPSPQLIKQMLTFENIQQPTDWDQILVSSHYRLLYSLYIIEFLMNQYDSNN
LQALIDDQEILVLKDKWMSKFLQLGGFDKLLQFFKQYQGRSVSTLPQIEKEILSFLLKTFQNYVIAACAT
NVPNLYKASRGIQIIKPLDQVLLDIRQSEDPEEFKLLVQKLKESRLGDSITEKIEKFISVLINLIQELLK
SNELEQEDRQIIEHSVIVIIVILLHNQELLTNSIENVEFINIFFSGIFTDKSDSVRNLFSRAILVLCHES
QKRNNQPTKIILQQLISMQNQQANSSQYYELLSQLIDSAFESEDSSQFIDYQQLTQQVLDGLINHKSIET
RQKSTAVDKILIGLLNLLTKLHKFIKQPIHDIIFNDCLFSLSDEKEIKCKSNESRQAAFKLLYQLSNQQS

NCENILLNLQQLSQKIPVLNRWNYIPSSDMRSSFGYSGIRNLRCICYMNAMLQQFYMTLPFRYGILQADD
GQEPDMQQSKTGFQFDDNVLHQLQQMFSYLELSDRVDYNPQEFCAAFKDYAGEPVNIFIQQDAQEFLNMI
FDKLENLLKNTVYKNILDGVFGGKTCTQIECQNCKSVKNKDEIFYNLSVPIKNLKNLQECFDKFVQGEII
SDFKCESCNQKVDVNKRQLLAQLPNVLIVHLQRIVFNLDTFMNEKINSRLEFPINLDLAQYTINQDQCTQ
YKLVGIVVHLGTADVGHYFSYIDIKNQDQWLEFNDHKIKEFKLKQMENECFGGQSNLEYNDNDVWGNGFR
ENSQSAYMLIYEKVQKDKIKLEFNSEEELQKSLSKFDNYTIQPNNELLVDYNSFKQHIPTQYHKKVNSDN
QQFLLERNLFNTEFMKFILDLSDFVNNDNANVIIEILIRFNYDLLARSYDNSISEQFNTKILQLIQQYPN
SNYLDLIYFGKQTKVQDLLLVCPESRTRKYFGKLLSTLFNQAIQIQGEITEKIGEGLNQLFLMLQDQVPK
NWTRFDQYFQFWLDFLQEGKLQVQYCLEKDMILYMIDFILDKSSPLQLYEKKTQMGNAYFPINCQIPMQI
ISTLLQQNHQLTLQEKKLLYSPKFYDKALKSAKIEELTPIILKFAYNNRYFSEIITGCIMRGLGGGDSDE
FKNYLQIAKPFLLIKDNLQVERLEWLVGIPSSKQKESAKVYDNQFVEYPQFGLYGLTSLEEDYWTFSSPL
GWQNSLFDQFCSNRTTKNLDNQCLLILKLLLMISLECETCFDYISKLPCVNYQYKYMEEIFKAFIETYQV
DTKRFYSAFPRKQETEETKAYLDQYCNKLSCAKHVCQQSILLHNXXXXXXXXXXXXXXXXXXXXXXXXXX
XXXXXXXXXXXXXXXXXXXXXXXXXKNKAYLDQYCNKLSQIHNEFQPQFDYIIGKSIEVTRVRKTYFLYN
PCTKEEMQIDEGSEGFVEIKQQIEEGNQHKVLTMEEKIYKTHICDNLPNGTTNEALPAQYVKGTQIHNYS
VDPNCQAANFIQSKAWSTDSDDKAIVTNPRIADSVKQIQLQNHTNRNLHVILEIKGEPNPCHYIPKSKIQ
SLMSSKSSTTMFTAIKHNCTQEFPPLQLTLVHKKQEPRQDSYLYLSSQDEMNLELL

>A0DZ45_PARTE
MEVERNTGNDKLRQQAEEHKNKGNDYFKRGLYSNAAEEYEKAIELCTNEANYYGNRAACFLQMKKYSKCL
KDCEQALSLDPNNIKFLRRKALSLQYLGLLTEAKPIFEQIANLDNSEQSLKEHKLIKELITYLQQARQKL
DDNQYKEALTFIERVAKEVPDAVDIQILNCECLARTSNINQAQEQLRLIQDKHGPRVETYYLKGLIELYG
GSPDKAKSILQEGLRQDQKNKKCLAAFQMAKDQDSYKSKGNDCLNSNRFDDAIDFYTKALAVDSNNFKFN
SIIYANRGLAYQKKKDHQKAVNDFDKSIELNDRYFKPYLRRGDSRQELGDLDGAQGDYQKVMELDQGSIQ
QMRQKINDLTRKQKQLSKKDYYKILDVEKNATDADIKKAYRKLALQWHPDKNKENEEQKKLADKKFREIA
EAYSVLSDKNKRQQFDMGVDPNDPMGGAGGFETNIDPSQIFKMFFGGEGGGDFGIGNMGGGDFPGGFKTM
FTTNLGGMGQNMRGGQGFPFQFGDFSQQGGAGFPGFQFPGMQFTQQQQRRK

>A0E3C0_PARTE
MLYHMYYQLSLFSFEIEIQLEQNDNWEMQMKEIKELWNAFESKLKLPQKPNFLQVVKNRNEQCILEMMVS
LLQAIIHSPYSSELVTPMMQLDQETQMVLLKLIQEEQSSLTLDQESKISDEVLRKFEELEYENQKLNQDL
MLSKEQYEMEKKKMKEDMEIVQTDIETRQKTINSLNEDLNQIYEVTQTNSVSEVCEYIHNRIDDIKGMQQ
IIETLKNDQQQEKDVHEEIVRDLRNKLEAGYRKYTQMQKLEKYCEQLKTQLSESLQEQTKNKENLKQNSK
LKEENIELNNKVNQLQQKLDSQKQLQKQNQKIQSQQEFDISKARSENESLSRQLKLREQEIFKLKQEIID
LEHNIHNKTEQSQLENSSVMLHSLNDGEERRNFSGIFTPKRNLGLELSQLITKSNYLIEGDQKLKLDVHT
QTDDNYESFTLLKKQDSSFDEEILSSQLSFWKTQCSDLLAERALYEEKQNQLHKEMKELQIKLEQFFIEQ
HKYEQELQDAKDKVLSLIEDNNVIRSQFEQSLQKIALQDKQLVEFPQMKEQLEGALQKLEKTEEQLRMKK
KKKVPINQSSRLMRNCYQLNRKSCSCWLHRSKIQSLIIMFPKSCDELYQCRLFKKLFLGSSTRYFYVFSD
RVIANKDSNRKRADRIFSNTNTNRLIWRYTNEQPLKLKGLIFDCGDIQFEYFGEDQTLRELKKNVSNLFF
QAKVQEEYMAQQVIGQGNYALVLELQHLHSDQRFASKCIDKKKVQAIEQGEQSVQNEIKIMRTLSPHRSL
INLIEVYEGDNNIYLIMDLAQGGSLYKEMKNKVSLYSREEVQNIMYQILSGLHYIHSKQIMHRDLKPENI
LFREKGNLNTLTIADFGLSVEIDAFPYLYPKCGTPGFVAPEVANLIDKTQPYTAACDIFSAGVIFHILLL
GEGLFVGNGHQEILRMNKEFQVDFSKPKYQQLDYDAKDLLFKMTAHNFLQRYTSEQCLQHPFFQNNGKQQ
SQQKTTQFNTSQIEESPNKDSFNQYLQNYNSPNQKLISQDSKKLSIVTRTPLYGPKAVTPQVQIQNDILE

ELSPLSHFSLDQEQQGEQEYDNHQIANTK

>A0E9R7_PARTE
MFYFQGKILLLFHIFNFAFGSGCQQELLEQNDYEIQHTSSIYQTIGIWFQWNPTFQQMKGLQGYNPNYLL
YIEKNEIDATTSASRIFQELEYNYFLIQHYAFQSYASAPQNAQHQQIERVFSFRLDPFLMEGNWGFHCFS
YDSTSKEYKVYLWFAKKGSMTEDFIFESKGYNLDRDPGTQYTYIVGDIQTPPFGQVYRKMDDEVIPLKFR
GLRSKLYFSDTYLGTLAEFLALDQLSCQKYNYCNAMGITRAFVPLIDGITSNFPQVSIQNYREERFSFSG
WVKLNKMYGASQDITIVRVAIYEFYSNDYIMGDRLFMMSYHYDPEYLQNSSIQIDTYSYDYPYISFYTTT
LFDTYKKQGLSMFELMTKWHYFMFEAGVQSYVSNVNVRIRDGAISDPNYYDIKYNYQRRNQFKQSQVVVN
LGGDKFSPSPMNGFLANVAMEYCYSSDQVFEFRCHSICQTCFGPDKTHCLSCKDTTTKVLKNNQCLCIRN
YYEDENNACQSIYYRIQNIAHNSYLGSLQLVQTNRNNACQEDQFLIPGDSSTICQKCPSSKSDFQIMCSD
CLLNQKTWYQNPICSQDYIQHQEGDVFDGSSAYQLIKRDKIDQDYYLIGKDGSLSLCEGCFGVVTDDTQS
HILQYQLDKLTKIYCKPCHQIINNSCVNLNLNCLECDDDFKCLTCQSDFQLLNSDCIKCPSHECSTCIIN
QGRVKCASCPKATYLNNGACVKCGQNCQICDIDRCQRCVDINQYYLSLDGLNCYPNPIPNCLIAFERVNG
QRTSSLQFNYPLLQRDQTTVVECALCKSNYINKLTSCSYYDQMDETEQSLDINNDGNILQLDIQIAKATQ
CQNIEAKNMKIDCQSPCFQCVKQNKYLLDGWKQQLMPFYQSYFSSSKFDNAPDPLNTNLVYSKDNLHIHC
TSCREGYELYITECIPICSSNCECKIVNNQNMCYKCKSNQRGQPLSLFDGQCIECPYNCAHCKIRTKTEI
KLINQYFDPEYSDLQIYGHQCLLPYADTTLYYDIDIGVYVECSSSLCENSLKLPINYYCDLTSYNTAKSS
YSGTLDEFLKYNVLLTDYESFKPFYETQFFYLIMNQRTLKTISFEVTLISSNAYCELPKNFTLATNLRRN
IFTLINLPIVLKSNVNQLQLFTKTLQIIGYSDVTIKNLQITNFPLADVSIFISDTFGFVVTMKDMLFSCQ
NVKLQVTINNPKQIKCDSLTFKDMIVPNTYGIFMYQGLDNDKIVQVEFINVKVINTILTNSSLFVLSLFE
TFQNQNVKFNTFLVQDSQFTDSTILQTNYQTSIRKAVVEFTQLKTITSTFISSVFLHFEGFLNVKLIDCQ
LDQSAFKDGSIWLLLPLFQLQNIKITDNTLETSNDRFITNKGTVAYSDSDNDQNFNQIQNLLFENNQFAS
GKAFIEIYQNVFIGLSIEITDLNIHSNQLFGSSSKYKNRNVVSENSTIYLDVYKIALKNVDIIRKQSFPE
LAILNSIDVQVLNLNARHSELKTSFLHNSYSCAQKSIFVQGYTALMYIYNAQNIKFEKIYLNNLRSANLP
FIFIKSSDKMKVRQNESVLIQNSYFSQNLLLLTAISEQQAIIQISSEQYQNVIIRNVTFFHNYQHSYVQD
LSFISSSTILFSSPYSTIKIEQVTFERNMITSGQNSNLVLKCSTIKVINCSFLQSNLPPLDTIQQNIYWG
IKQDQVLYFENYTQSFPIYSQGGSAYLSANSLEIENVLVLESRSQKGGAFYIAPVQYGVINIQNITFLDC
RASLEKVSSAQGGTMYIDASLAQLDITIKDVEITRSYSRREGGVFFINPSKTKNSVRLINIMVSESYSLF
YSFLRIPQTSSADVYIQNFVVRNSYQGFKDYLGLLFYLSEIELSTFQSSYFISLMGNLTIDISTISNQFS
GLFEIQSGQAIFKQMSISNCFMYSQPMMIFTSVNLLTFQDIRMYNISNHQYDEDPVDQFEITYQALQMGC
TQETSKPSYLYDALNSSIINYYGQQNIKSISASFAYPTSLIQIDSIEKDFKITYKDSQFSMLTCSNCQKA
LILHTFNTYYNMGVNIVQIDNIKFHQNICGYSCIVFLQENSNRLLSLTSSSKENLIYTVSVRSSEFLRNT
GQNGGVFYVDDINFLIDSCVLNYNQASQSGGVVYFISKSAAFNIYDSDLTSNSAQIGGAFYLNNYTLNSP
DRLNLHLINNRAIDYGKNYAEYPSQLTVSLDGEQTQLDKKVIFSNSSTIIDLITIQPYSFNNQTKDFIVL
PSGGQAIRNYKYYNETTQQFVSYNLTFRILALNKQNNKIQNLQGTKCTINSFLVSKASSNQEIPNVLSDAN
ATFTNLKEVAFDTDNQDYNLDDMIIYFDPTKNSEQVLQLEFFCDSVRIPQFDPEPPYLLNTLIQDYRLRV
NVQSLDCQLGEQKRLDDGTCVVCDSTLDQYSVQPGQQCQTRDSISTEAVTSASVKLRPGYWRPYTYSVRI
EYCLNMEINCEGGWIPGNPSCFTGHIGALCEQCDVYNIEGNGSWSISGQYKCGSCDSIGDNTIKVALVSA
WTIISIMLSVKSTMEMVENMVMGKKLQKFYIENPKTGYGGILIKVLTNYLQIIGVVATFQLSLPSALSEA
FKTVGSPVESMSYSLDCFLINMSKIDILYFRMIWALIMPLIYIFTFCILYIIAIIVRIVIPNRSAITTTL
IYLFTFLQPTLLGGFISLLSFRQISGIYWIQGNVSYRYDTSTHLGWLITFVLPSALMLGLIIPTYLFLSL
YKIRNKLDDENNRKNWGYLYNEYQPKAYFWEIVKVYQKSFIITFLTFYEDLIIIKAALVFIIVFIYSGLT
KRFRPYKLPFLNQVDDVSTLVCGTSIVLGMTLYSANLSNNQEIIWPFYILLILINASFIFVILWEILWAN

LENQQAALDKLRDKLNAKFPNLVNKNWLTKRLFTNRGEQLKRNKRRWKMIRNYLFDIIRKQGLVQTEDND
SKLDRKSLPGIPKYYNKIYPENFIDQFGSDIEMSV

>A0EAD3_PARTE
MRKQQALISKRIRTNYQIQYKQKLTINIKFHQQILMKIIFLVINVIQVICLSPQFQLQENQEISLYPTEG
EIYKYYIDSIVADTQLMCKIDPQVPNVQVLNQCEEIFQMQGDEFKSISSNNTHFSTLSYENEVTFYEWKN
QILEKIWESEIIDSSFNCFTINLSEIFSILVDCYQNNEFLLIQLMDKQSKITYQIQSSMPTSTKIQSIVN
GTNNFIVYAQYFEDYSILSLISSSFQNLSSLNNQFIDFDIPITITPNIYVITSQELFQLSISSDSQFCFK
ANFSQQNLNNFITINVYYDLSIYSQCDQILLAYYYQYQPCLILLLGCENSIIQTQARFNWLQFESILNIF
QNSYFIIIQLNSQIFIDEKQSHFLFAYHLTNQDNSLLYFNSDKLTVMIVYKIQLPSLQVNLTNLESARNN
STFRLICQNQGKKIFYQSKIYLQVLSQNDTNIYVMFKQQFPQYQFSWSIIVQNSFLSFSGQLLQYKQNPD
GIPLNFTLLSQQQAGQINYRYCLVQSLSLIFQQNTYFYIIGYNNYSIDIIGSYGIQSIFQFSQICSFNIS
INASSLQVAYSINPQMIIIGLSANNTIYLFQYYNSNNSIISYSNYTFEQQFSDFVVTFNNIIILFANQQI
KIMTFDFTNNFTLNQSSINHQFNNLQFNPIQIVVNTQLQSSLLYINNINEVIIISIDQNSIPKPISLIKV
NYTIKQINLISQQLILSYLCNDYQNMCFQVYNLQNLPKYYYVKNLYSVNVDNKAIIQSDNLFLYVTFSNY
TVYVYNPFLTYHQSLYYMLKLTSPIQCVQAVKSYYFYVPQFQYISSIILLQNNTILQLYRKQEFQINVEF
YNEDFNNSIQYPQFIYNYNVTSSLNETAFQQTPNQSIVLYSNFTVFLNQRNLSINLTKDNIINNTKIFSY
PMNLILDRQVGYCGPPNLAQTNNLNKHCTFLIIATFLQLLRQIMNALLYRITLTYKQQIVXXXXXXXXXX
XXXXXXXXXXXXXXXXXXXXXXXXXXXXXXXXXXXXXXXXMLQPPPLCWKELLLSILCIIQFGLIILVSS
NNINYIAQGKFEQQKCSTNNNCQGLGVGFKTIPAFGDLINNGNSYYQNGVLTQQFKQNTKYIVGIYYLNN
LFDGNLMEPILMQSSFTTTVSNYAMVINSQYQNGTALYIYKNHLYNYTIGTWNVTCLLNKKLQSQVNVSI
FCINEFSNGTYNISFKPPPLEKKSKRWIYTLVSIIILLLLYFYLKVRQKTIELSYIQQEVEQ

>A0ECE3_PARTE
MDQKPEQQVEEQVQNKAQQKQQQGKQQPKEKQLAVKNPKGTRDFLPQQMVIRRQAFKIITDVFEQHGAVE
IDTPVFELKETLIGKYGEEGGKLIYDLQDQGGEILSLRYDLTVPFARYLATHGYKKLKRYHIGKVYRRDN
PSIQQGRFREFFQCDFDIAGENDPMVADAELIKIIDQIFTKLDLGPFQIKISNRKLLDAMIQIAGIPKTQ
FKTVCSSIDKLDKQPWKEIRQELTEKKGVTEEQADILSKMVQLRGEPFQLIQQLKEQKIFEQQGKEALDE
MELLFNLLKYMNGLKNVIFDLSLARGLDYYTGLIQETVLLGGQLGSISGGGRYDELVGMFSKNSIPCVGA
SIGIERIFAILEEKYKKSGVVLRENQSDCVVATIPSKNIDMNAEKFKIYDLLWSNGIKADVCYKLNWNLG
KQLTYANDQQIPFAIVIGEDEVKQEVVKFKDFVEKKEETVNLKDLVQFIKQRVGKEQYMLLLLIYYQCQQ
YNNFIIMNGKQQFIHQITDESIDLNVAKDMLQEAVIENPKVLGEIFEQKLNMHALCILHSTLKEYTPLSS
LIAEKFLNKNIYAQSSIVRIYYSYLQKTASVHELYQAIKNNKVEKLDNPQIVVITAHKVQNLSNVGFQIL
TLYTPAFKNMQIQVYDDVYDYGQFVKDQFIHIIGMTIKIGIDSYPTFSSSATISLYQIYNDVLIQDAELN
DFRRCCKDDALREPLKLNIQESLLSDFLSLSSSLMLVSANTLRPLNIPTKLLPCGGQEQQVMRYYKKFYS
IS

>A0ELU3_9BIVA
MAMADIENLIESMKDVHVNSLNNGCRRRIAMYLNPEGSLIPDSDMFNDWCGYAELLNFSQPEIENMKRHK
SPTEEMLHLWSTRNDPEPKVGNLISFLCKLERFDVISDCRTMIERDVEKWRQNQLSLQKINDDPTFREPQ
SRPRSPQSEETQTRADVGSKDEIYYDCFVIYNPDASDQLEFVKQMSRILEGPEYNFRLFIPWRDDLVGTA
MHSVSAAIIEKKCRRCIVVLSKSFNNSPAADFQLKFAHALSPGARQKRVIPVLIENIEPPSILNFVAPAP
FYNVGIRDWQWPRVAATIKSELRPDPETWLPKVDAWNIALDTSDVTKRELWGITVATQIYPEEDPVGIPD
VDAQRKKHKNKNKKRGE

>A0EM56_BOMMO
MAVTSTRGIVNLKEKGHFERENARVEKYTLGDELPAQLSSLEIRPRKMSVPAYTIQRLTYNDRDLVLKFL
RRFFFRDEPMNLAVNLLETPESRCTELDDYAAATLSDGVSVAAVDENGDYVGVIINGIVRREEVDYTDKS
EDCPNPKFRRILKVLGHLDREARIWDKLPETCDSVLEIRIASTHSSWRGRGLMRVLCEEAERLAKAMGAG
ALRMDTTSAFSAAAAERLNYKMAFGVRYADLPYAPQPEAPHLEARVYIKEL

>A0EM58_APIME
MANVVPWLLSICLFGLQEIAIQPGSNALSRNVEASGQRSVSSGFRSSLRNYKTLISSHDELPGHINCDSS
KFEEDLMNKLPTTPEYNNHLYGSTPDSRDYFSRPFEKRLHSRLPGRNFNLLRADANSVNELESQGFNYDP
GRGHIEDDYVGPAMELVYAWSTIDYTYDSIEARDSAIFDGDFITENNLPLGLEVWRDKVFITLPKWKDGI
PVTLTTVPKHSKTKSPKLRPYPNWEWHTVGNCDGLTSVFRIQVDECDRLWILDSGKVDIAKGGKLACPPA
IFIFDLTTDTLIRKYIIPKEQVKEDSLYTNIVVDIRNEDCGSAIAYVSDVFRYGLLIYDFFKDSSFRIQH
HFFYPDPLASKYELHGLKFQWTDGIFGMALSPVDIHDDRTLFFHPMSSFREFAVSTSILGDKKTAEENTD
YFMPIGRPRAKDYGHSSGSVIDRNGVMFFNMVTRDSVWCWDTRKEYIPQNLGVIGTSNLSLVFPNDIKVD
HEYDQNVWVLSNKLAMYLYGSIDSSKINYRIFKANVKEAVKDTXCDPNYVVPDSEHGYDEIC

>A0EM59_APIME
MRQFYFSAILFLLAISDSQAQEKLKNIYSWKALEFAFPNGYAKLAAIKSGSYIPGASLPIDVDVYNTEQQ
STVFVAIPRIQDGVPLTLGYVTREVSIDGNPLIAPYPNWSYNDVKYCDGLTSVYRMQVDKCGRLWILDTG
ILGEKQICRPKIHVFSLHDNKLITMYRFPQNQFKESSLFVTIAVDVRDTEDKCKDTFAYIADVTGFALLV
YDFRNSRSWRITNNLFYPYPPYGTFNIKGDTFDLMDGILGLALGPIRNNDRILYFHSLASRVESWVNTSV
IRNYTLFNENSEAAARSFVPFSIERSSQSVAEVMDRNGVLFFGLLSDLAIGCWNSEHFFEYGGNNIEIIV
KDPETLQFPSGMKIISSKKDRQELWIFTISFQKYMSGTLNSNETNFRIQAGLVDELVRGTKCDVSLLGRF
IPSQ

>A0EXG3_MOUSE
MPRSHRQPPPAPRAPAANAAEPKSDGVLAMTFKIFLLFAGXMVKVPVGLYFSCKLLLFQSLMLMSPEDSA
FYATIVSVVGLHVVLAIFVFIVWKEGLPQWRENKND

>A0EYL8_NEMVE
MDSYSFSGQGRVNQLGGVFINGRPLPLVLRKQIIELAQLGVRPCDISRRLRVSHGCVSKILYRFQQTGSI
EPGAIAGSSTPRNVTPEIEEKIDEYRRENPGMFSWEVRDRLVKDNVCSRCTVPSLAAISQVLKNRIASTS
SAASEDDDVFIDVEETDDNSNIVAGVILKTKKERKDEEDDKGKEDEKVEKNSSLPSRHNFSSSYSIASIL
KKPSAEEDGSAEIVKGIKVEAIESPPPSTSPSRMHFSLDPDFLLTRKQRRSRTKFTSKQVDELEKAFLKT
QYPDVYTREELAQRLNLTEARVQVWFSNRRARLRKKKINGDDNDSTNKRKYCYPQGMCSCHPTAAPIRYQ
VVPINDRVYTLPVL

>A0EYM1_NEMVE
MLQNNIEQSLGLGKAPNQYVLGDVIGLEALSSGHPSVVYRSYQDHPDQSHTPDHGSDSENPATVRRKQRR
NRTTFTKQQLQELEKVFEKKHYPDIALREELAAKINISEARIQVWFQNRRAKWRKLQNPNHSLLKKNRLH
HEKLQGQLPIAPRPGLPCGGPNYFLSLPTGMPTVSAHSSYPGLLTPLSASSNYMSSPQLPVHNAPLWTCQ
TAFQSDQPKMALEALRFKAKTHSSAFEFVPTYH

>A0EYM2_9BIVA
MAWEPSQAALALVAFCGVLYFAAGEEGRTKCTNTKNHTVHDFSFLNVYGNETIDLRYYRGEVLLVVNVAT
YUGLTVQYHGSNALQGKYRNDSFRVLGVPCSQFHFQEPAFTSEELMNGLKYARPGHGFVPNFNLTQKTEI
NGHKEHPLYTYIKSECPPARDRFVQPILYEPIYTSDVRWNFEKFLIGRDGHPVYRYASTIDPRTSQMLDA
DIAVEIKKTLHGHGKDIVDIVG

>A0EYM5_XENBC
MPQLKFSLWMTMFITTWTFMMMTALFNFNEATANTPMTKDTKAKNSKKWQNQF

>A0EYM6_XENBC
MTKSILMTNLFHQFEAPNLMGVSMFTLTMMLPAAMLMMTKPLKMMTPPRPTTLTNMIKDGMIKNMLLPIK
NQNHWFWVLNTLLMFILTINLVGILPYTFTPSSHLALTMTLAIPLWSASIILGMRVKTKEAISHLLPEGT
PLAIVPFMIMIETLSIIARPIALALRLAINITAGHLLLKLISSATIFMTTQNLIPVGMMMIVMILLTILE
LAVAMIQAYIFTILTTLYLEENL

>A0EYM9_XENBC
MPFMNLVLMMMFMCSVTGLMMNRPHFLTMLLCLEAAMVSTLMIMMIKSHMNPSNTMMLTLTMITMAVCEA
SVGLSLLASITRTHASDMVTNLNMLKC

>A0EYN0_XENBC
MLITSITMSTLQMISPWVSKTPWKTCLTLSTMTATLVMTTNSFQTKEMTFSHLMSLDFISSPLMILSAWL
APLMMLASKAHMKDKTKVQKKIFLTITSLLQMTLIMTFTVNEWVLFFIMFETTLIPTLTLITKFGAQKER
LIASSYFMIYTLCGSLPLLTSLMILNTKTGTSNMMVLTLQTDGVNMYMKNMWWMISLTGFFIKMPLFSVH
LWLPKAHVEAPVAGSMILAAVLLKMGGYGLLRMAPIFNPFSTLLTNKIIMALALWGTLMTSMICLRQTDL
KSLIAYSSVAHMSMTTAAILIMTSWSWQGAYNMMIAHGLTSSLLFALANTIYERSHTRNMTITRGYKILM
PSMYMWWMLAMLSNMSLPPTMNFLGEIMMIFSLMNWSVLTVLVTAVATVMTALYSLYTVIMTQHGKPMNN
NKNPTNSMKSTEMMNSLMHILPLTLMITMPEIIKIT

>A0EYN1_XENBC
MVSPMSLIIITSILMTTIIMMTSYNKTSNKLLIPMIISMPLLANITNSSKIINLTEPKWLSVTYMELPFN
INLDVQSITFSLVALFITTNIITFSNYYMEKDKNIKTFKNMLIIFLMFMLILLTANNMVQIFIGWEGVGI
MSFLLISWWTTRNLANAAAMQAIIYNRVGDMGLIVAAAFAITCSSSMNFQHMTLNSTNSLMLSIGLLLAA
AGKSAQLGMHPWLPAAMEGPTPVSALLHSSTMVVAGVYLMMRVAPLMAKINFLSNLAILGASTALFASTV
AMFQNDLKKVIAYSTTSQLGLMMFSIGVGQPLLALFHMTTHGFFKALLFMCSGSLIHNNTDDQDARTNNN
TMKESPISLACMVTGSLALMGTPFLAGFYSKDLIIEFAAKSSTNMFAFTMITMATALTAAYSTRLIKLIT
EKTSNNTPLKMKNEPKKLMYPLLTLSGGALISGWMLINFINENTQEEHPLPTSVKTLALIISILAVTTTI
QWKSETKSKFFYKTWFFTDMNHTNPANMTMKNMLAGSFMTGDQGWLEIMGPMGXTQSLTTLSKKTTIKPS
KIKTHMGVLILSLMMALIMF

>A0EYN3_XENBC
MVMNFVYLFPIFFLLSVILIVSSESPYFSIVGVIIYSILASFMMFLWGYSLLGWIFLIVYVGGMVVVFSF
TASMSSSVFPKVGGMFFIVFFSLGLVVLSFYAYLSKWWAFGEVFSFGHNSVFLLERLSYGFHLAYSSSTL
LLILIISLIASMVCVYMSCLCYSKGQILRY

>A0EYN5_XENBC
MMKKLSLMMIFLSVGINLFGLMLITLSNHYILMFVGLELMTFSFITMISPTSPRQVEAAVKYFIVQSTAS
IILLLSLIFPNTQISLVLMMMLMMALSMKLAAAPFHSWFPEVVQGVKWKPAMIILTWQKLGALVMMSRLM
TQDNKMVMIAVALISASLGAISGLNQVQTRKIMAYSSISHMGWTIMLLTINQPLALTYFIIYSLITASXM
LTMEYHNSNHLSKMHLDSFLXPWTLAMMLSLILSLGGLPPLGGFLNKLLAFKEMMVYNMGMTAAILILSS
LMSLFFYLRVSYSTTLTSSSQHSISFMAMRIKKKTFKNMLINQPPLTMMITSSHIVPILGLMFLPAMMVS
LMTMTS

>A0EZR6_AEDAE
MSSTNLRVSSHLGLGSTMMHWGLMVLMVGTVVQAADQRFCGKQLVLTLSMLCDEFPDLHYGAKKSLNDYD
KDYSTDEWLAMIGQDPESIVSTDLMVPHMDQQTVQQQQKVPLWMAMMYPQGYGFRSSASRNDLIPPRFRK
SPRGIVDECCLRPCSINQLLKYCKTIA

>A0EZR7_AEDAE
MQSSSINLFRALLIVAMQLALLSLFVGRAHGSPLASPGSGSNADGLLHHTRSRYCGRKLTETLALLCQGR
YPMMSHHRSEYLSDQPQAQVEVEVATLPDSGSHGFPFSRSTGHRRVRRGGIYDECCKKSCSYAELKSYCE

>A0EZR9_AEDAE
MTCRFYILVFLCLGGLLVVAVKSERVCGPKLVKTMYNVCPNGFYGPQTKRNNVFDRGPFYKLFGPTWTRF
ITDKAQLSVMLRTRRNIPTGLAHECCQKSCTYEEMESYCIT

>A0EZS2_AEDAE
MAYHFTKANVSLWLLVFCVFGILSSRIESVDGQRFCGKVLTDTLTAYCEIFPTPRPSKRRVMEAADFRYV
LFPNLPPALDGQSNDPESLRSMQKMNTDSKWMRAFYRIKDGANYDKTKSRVADNDDELAPDGFQLGKRGV
VDDCCYKPCTLQYLLKNYCG

>A0EZS5_PSEMX
MKAVLCLAALAVFVCSVESKYSPPKVQLYSRDPGENGKDNTLICHNWQFHLTKSVPFLPNDGQMYSCKVT
HGSNVKDYAWESNM

>A0F019_PSEMX
MATPVSVLRLPKGPDPNSRGFDPRSPRFIALCRTSICTSAASEADPEQLQREQHARSVLRESFLRCLLSM
TDKKVQFHMHEKVKVAATFGASDIDVLNFQVSNLHTPIGVQKEALIRCQDVISFTFDA

>A0F0C2_MESMA
MKILTVFMIFIANFLNMMQVFSVKDRFLIINGSYELCVYAENLGEDCENLCKQQKATDGFCRQPHCFCTD
MPDDYATRPDTVDPIM

>A0FDP3_SACKO
MDGGLELGFINTQPCMAEFLTSVGRNINESTYCSQSITHNSTSATDKNSVNISSCAQTTCSAPELQHTLQ
TKSNHVPSVKLPEYPWVEEPCSKTDEEGDDKSVNGKEAGHHRRVRTAFTNTQLLELEKEFHYNKYLCRPR
RIEIASMLDLSERQVKVWFQNRRMKHKRQIMKAAVSGSPIPNNETQNSMVSATSSPSGSDQGKQGVNGNV
NGLSPSPDSRSTPCVTSNDDGKQREPYLFTSNVCCANTVSPTSPSLNFASSAAPIHRNPETYTSFNYQPA
NNNCVIVSAPHTDGYLGRDSHGNVGNMAAAEYDTSSPTFRNVPGYGHQRSATDNRTTFSGNGRLVISSNH

GDDDHSPSSLFTYNSYRAASNHHPYYVGNAVQF

>A0FDP4_SACKO
MSSYFVNSSLTSRYQNGQDFQVQHYNTTGRYNPAPAAAAVNYNTGYQYSNHHDASYYPSDATTALLQPQQ
PTTTNRLSHTPSSHPSYSLTGNLENVTMSANSTSSPSPNSDSSLCSGVHGKEKIGTGSTQDGVYPWMRRM
HMSSGTNGMEAKRSRTAYTRYQTLELEKEFHFNRYLTRRRRIEIAHALGLSERQIKIWFQNRRMKWKKEH
NVKSISQIMNPDTKDNSCETGGLSATGSVSPTN

>A0FDP5_SACKO
MSSYFPSLNSYQTTQSEYPSSQVGLYSANYDPIRQLQSYSVPENRGYAGYSDKYNYSEQRSTAGSAASNY
AAVGYVTETNNNNYHNRQLAHNANLPHYDLSCNSTRNKDVVVDAMEAKKDKDSDSDSGKTPTIIYPWMRS
NNYCGSDQRRGRQTYTRYQTLELEKEFHFNRYLTRRRRIEIAHTLGLTERQIKIWFQNRRMKWKKEQKTG
GAPSPKQFASSQTQNAMQVNGDSNDGSNGEIS

>A0FDP6_SACKO
MTTAGSAFCVNSLISSEENEALLRNPADIPSAGNQPIDSKCGLLIPKTTEPAMASAAAQGMSLHSSSANL
YPSGGDQNTYTNAWYYPSTEQNYASMQVTGLHDTEYDSNPYGFSIGAQKNYDSSSLMSSNSNFYPFQARQ
GYDRYHTNGYPTTNGTGIHHVEYGNLKYNTITQRQRYSGSYPYENTNSYSSPMSSSSSSACRLQNSKTNL
AATSPTSTGSESRSPAKSEGSPIGTNNNNNSTNSTTPKTEVTRNSPSDIGDQPTWLTTTASGRKKRCPYT
KFQTLELEKEFLFNMYLTRERRVDIARLLNLTERQVKIWFQNRRMKLKKQNQRNATMLH

>A0FDP8_SACKO
MHASIQRDSCTDWFVDPKNSMDRYSSLLREKQTATDNYLSNAMASSNAVTSDQGSVFPCRYPQYYNNMDT
TNANYNDTLPSWNSYDFNQSRQCLNTFQSPPIFNGGGAISQHTYGINPTNYGAHPASNYTGNVPGVTDVS
NCSWLTTFTTTPRRTKRKPYTKMQIFELEQAFQNNMYLTRERRTKLSQQLSLSERQIKIWFQNRRMKLKK
MTEREKLEEKELREHQAL

>A0FDP9_BOMMO
MGNLKKNLEKLKHPNSRKTMTLAKKLKKSEKKDQSKLGTHIKQNLIGEKIMWFKERIPEECEVFDKEQTL
KLIESYLSRFDEELEQIALKNSIGQRKNRQHASREDVINMTRKKEQDEFETCGIEIPDLMDPQQMNVVRN
WNGELRFLHHFKLKRFTKKNLM

>A0FDQ1_BOMMO
MNTINFQTFVNSFQNRLEPPVRQHLKNVYATLMMTCVSASAGVYVVDMFTRFQAGFLSAIVGAGLMLMLIA
TPDNGKNTNLRLGYLLGFGLTSGMSMGPLLEYVSVVDPSIIITALLGTTLVFVCFSAAAMLAERGSWLFL
GGTLMTLFTSMSLMTLVNLFMQSHFLYQAHLYLGLMLMCGFVLFDTQLIIEKRRMGSKDFVQHALELFID
FIGMFRRLVIILTQKGNKTAVASVTKNILRRYYLPTSVW

>A0FDQ4_BOMMO
MIVGGKTYEGRLIQGLRINTPGDDENKPVIFIESGIHAREWITPATTTYFINQLLTSLDPNITALRDQFD
WRIFPTVNPDGYHYSINYDRMWRKTRSKSSSTCRGADPNRNWDYNWLKHGASSNPCDYQTYGGSRPFSEV
ETRTLSQYISSIENLMAYVAFHSDAQMLLLPYSDSTEHNDNYDDLVTIGKTSLNYGYAVNGAKYKGPATA
AEILYKASGGSMDWVRHTLGTPIVYTYELRGTYFYWPPERISEQGDEVTQMILGLVQEARNLGYV

>A0FDQ8_BOMMO
MKVLLLCIAFAAVSLAMPVAEEKDVVPAQPILEVAPKIDDSVKPTEVAAATEEKKAEPAPVSNDEVPAIP
EAKKDDIAPEDSDIAKPETVPEVKTEEKVPEAKSSEIPDAEAKSADIKVEEPAAQPEDSKTEVQATVAEI
SKEEKPSATDAEGSADSAAIIPNMVKKIDLAPTVESDAAAVPEIKTPEAADAPKLADNPVDEDKPADISP
DAPKAEAKSADDSATTAKDDIPVAPETSDDKNKETKTKDKDSSSSSESSQSSDESKSEENKS

>A0FDR0_BOMMO
MASSRLERIGTIFTRVEGLLSRGAMKPDDRPLWFDVYKAFPPITEPKYARPNLVVKEIRPILYKEDVLRA
KFHSNGYGLAPVSLLNQSNETQTKRLVQQYDELKAEGIPEDEIIEKAAQAVAVERHSYAAQKLNVTPKNP
DSVTAQVLAEADIKNIFNNK

>A0FDR2_BOMMO
MAQTAGVKPMTIVGRVASERERCLGMTDAERAWRKQWLKDQVLAAHEPVHVEEYWRERTNPIRRFYRKPL
DVLFAKLTPMLGEQRAAHYRYISGKLGLIAVAMLSTHYYFKYLGNDWTKKGGWKVLKTKPMVLPGQPGFP
FKSEKTDSDYAERKFKSSVI

>A0FDR3_BOMMO
MMLIVLLLQISLVNCVNITENTLKIKSLIMKYKNNRVHRSPMTSLDGFVQDALMFFDRTERFKDVLNVYN
YSECVGDEGLMEKHVLKGLLIHEHLPRRHWHEYKAIHNKLYSSTHHEMAALMKWRQNLRRVARHNREYLA
GIQSYSLHLNHFGDMHVTEYFGKVLKLIKAFPLFDPAEDHHKTAYRHNRRCKVPKRIDWRDQGFKPRREE
QWQCGACYAFAVTHALQAQLYKRHGEWNELSPQQIVDCSIKDGNMGCDGGSLRGALRYAAREGLVMESHY
PYVGKKGYCRYDSNLVRARPRRWATLPSGDEEAMEKALATVGPLAVAVNAAPFTFQLYSGVYDDPFCVSW
HLNHAMLLVGYTQDYWILLNWWGRNWGEDGYMRIRRGLNRCGVANMATYVEL

>A0FGR0_XENLA
MHKSHLDSVAPQLRSDLQCIKASSSSLCDQMECMLRVLQDFKRSSPPPSPDIEKPCVVPPRRAPRRDNRI
SHRTSDLSEADSACCMDLPSDVSPGSCGQRGLEWDSGYSEVSGGSLRGEEDDIVEEESETSVPTVVLRRL
PTPSCQRLSSGGFLNSRQGRIRPKSTSDVCLEQWRGIGLGSDSQDWTGCLLSQSRSRQPLVLGDNSFADL
VKQWMDLPENVDEEGRRVRDGGRWLHKPHGFLISLSGNVKRRLGNMSRLRRSEQEAVKRMSCPQLGCRPL
SLYYHQSLSDIAEASTNLLHCRSRQPIICNEGAGFL

>ESYT2_HUMAN
MTANRDAALSSHRHPGCAQRPRTPTFASSSQRRSAFGFDDGNFPGLGERSHAPGSRLGARRRAKTARGLR
GHRQRGAGAGLSRPGSARAPSPPRPGGPENPGGVLSVELPGLLAQLARSFALLLPVYALGYLGLSFSWVL
LALALLAWCRRSRGLKALRLCRALALLEDEERVVRLGVRACDLPAWVHFPDTERAEWLNKTVKHMWPFIC
QFIEKLFRETIEPAVRGANTHLSTFSFTKVDVGQQPLRINGVKVYTENVDKRQIILDLQISFVGNCEIDL
EIKRYFCRAGVKSIQIHGTMRVILEPLIGDMPLVGALSIFFLRKPLLEINWTGLTNLLDVPGLNGLSDTI
ILDIISNYLVLPNRITVPLVSEVQIAQLRFPVPKGVLRIHFIEAQDLQGKDTYLKGLVKGKSDPYGIIRV
GNQIFQSRVIKENLSPKWNEVYEALVYEHPGQELEIELFDEDPDKDDFLGSLMIDLIEVEKERLLDEWFT
LDEVPKGKLHLRLEWLTLMPNASNLDKVLTDIKADKDQANDGLSSALLILYLDSARNLPSGKKISSNPNP
VVQMSVGHKAQESKIRYKTNEPVWEENFTFFIHNPKRQDLEVEVRDEQHQCSLGNLKVPLSQLLTSEDMT
VSQRFQLSNSGPNSTIKMKIALRVLHLEKRERPPDHQHSAQVKRPSVSKEGRKTSIKSHMSGSPGPGGSN
TAPSTPVIGGSDKPGMEEKAQPPEAGPQGLHDLGRSSSSLLASPGHISVKEPTPSIASDISLPIATQELR
QRLRQLENGTTLGQSPLGQIQLTIRHSSQRNKLIVVVHACRNLIAFSEDGSDPYVRMYLLPDKRRSGRRK

THVSKKTLNPVFDQSFDFSVSLPEVQRRTLDVAVKNSGGFLSKDKGLLGKVLVALASEELAKGWTQWYDL
TEDGTRPQAMT

>ESYT3_HUMAN
MRAEEPCAPGAPSALGAQRTPGPELRLSSQLLPELCTFVVRVLFYLGPVYLAGYLGLSITWLLLGALLWM
WWRRNRRGKLGRLAAAFEFLDNEREFISRELRGQHLPAWIHFPDVERVEWANKIISQTWPYLSMIMESKF
REKLEPKIREKSIHLRTFTFTKLYFGQKCPRVNGVKAHTNTCNRRRVTVDLQICYIGDCEISVELQKIQA
GVNGIQLQGTLRVILEPLLVDKPFVGAVTVFFLQKPHLQINWTGLTNLLDAPGINDVSDSLLEDLIATHL
VLPNRVTVPVKKGLDLTNLRFPLPCGVIRVHLLEAEQLAQKDNFLGLRGKSDPYAKVSIGLQHFRSRTIY
RNLNPTWNEVFEFMVYEVPGQDLEVDLYDEDTDRDDFLGSLQICLGDVMTNRVVDEWFVLNDTTSGRLHL
RLEWLSLLTDQEVLTEDHGGLSTAILVVFLESACNLPRNPFDYLNGEYRAKKLSRFARNKVSKDPSSYVK
LSVGKKTHTSKTCPHNKDPVWSQVFSFFVHNVATERLHLKVLDDDQECALGMLEVPLCQILPYADLTLEQ
RFQLDHSGLDSLISMRLVLRFLQVEERELGSPYTGPEALKKGPLLIKKVATNQGPKAQPQEEGPTDLPCP
PDPASDTKDVSRSTTTTTSATTVATEPTSQETGPEPKGKDSAKRFCEPIGEKKSPATIFLTVPGPHSPGP
IKSPRPMKCPASPFAWPPKRLAPSMSSLNSLASSCFDLADISLNIEGGDLRRRQLGEIQLTVRYVCLRRC
LSVLINGCRNLTPCTSSGADPYVRVYLLPERKWACRKKTSVKRKTLEPLFDETFEFFVPMEEVKKRSLDV
AVKNSRPLGSHRRKELGKVLIDLSKEDLIKGFSQWYELTPNGQPRS

>A0FH50_DANRE
MKSKGKGSKRGRQSWAEQDDPDFTLETEPGSSEQEGSEASVRLGGSWRTLRGEGHSRTRVGARRRRQHGN
TKERNVRRLESNERERQRMHKLNNAFQALREAIPHVKTEKKLSKIETLTLAKNYIKALTTIILGMSNGCL
PDAESQMEANASRLLQCYQQHLREEGEEDLSNYLNHIHSFSQDS

>A0FIK6_HUMAN
MASPSLERPEKGVGKSEFRNQKPKPENQDESELLTVPDGWKEPAFSKEDNPRGLLEESSFATLFPKYREA
YLKECWPLVQKALNEHHVNATLDLIEGSMTVCTTKKTFDPYIIIRARDLIKLLARSVSFEQAVRILQDDV
ACDIIKIGSLVRNKERFVKRGQRLVGPKGSTLKALELLTNCYIMVQGNTVSAIGPFSGLKEVRKVVLDTM
KNIHPIYNIKIDKELASGEYFLKANQKKRQKMEAIKAKQAEAISKRQEERNKAFIPPKEKPIVKPKEAST
EAKIDVASIKEKVKKAKNKKLGALTAEEIALKMEADEKKKKKKK

>A0FIU8_9DIPT
MKLLIVAFAIFVALVVADPHGHGHGHGRGRGRGRGGHRGHHEHPQPDNSTDQQPEVQGLQSPRIWEIIAQ
EINQAVGEARNSSDAVPTAANSTDEGY

>A0FIU9_9DIPT
MFCLIVILGVITGGFALVLCPPNFDPAVTIHIPHPTNCSKFITCVGSQPVEQDCPQGLEWSESATRCDYQ
QNANCEHRVRVRRSENATVETTTVSASKVENTTHSGGSIRIISSLAILIVTITVSVL

>A0FIV0_9DIPT
MVMPVNNGSTIFLVSLFGMPTYTSGTSSTSSTSSSTARSPVNATISLGNRENTSAVEDLGTNIDGLTISR
SKRFVPLLIPNVTGFSFFQNLPGIFTRQKRAAESIPPLSNESVIRVGNQIIKLPPRGISNLTIHIIDGGS
YSEKFTPFQTPANGTWAPFVDQIKNFFDGLFV

>A0FIV1_9DIPT

MKSLFVLILATLLLAVTISTVEVESRRIWHPIIYPYAARNATSDGSSRDAQDSIAFQPRILPWFGEVGER
IPEESLQIQPEFN

>A0FIV2_9DIPT
MRTLLKIFVAICLISFALAIPHPRPDDSDEESSGTQRGRDSVSDEDHNPAERRHNFGVNADSDERGRQEP
RRRNDS

>A0FIV3_9DIPT
MKLLFFAFALMVAMVIADPARFGPPRRPGGPGHRGHHGHHGHGNRTANGTEINDRFIPPSVWERIRQEIQ
RSILLSRNASSADLAAANATGN

>A0FIV4_9DIPT
MKLLIITLVVFVAFAMAEPHRWGRPGWWGPQPGAPGGRQPPQQQPQQPVNVTEIVRDIWRIIAQEIRNAV
NAARNSTLSNSSATDASNSSSSN

>A0FIV5_9DIPT
MKLLLITFAAFVAFASAEPIFRWWSRPIWWGPDYYQQPQQQQQQQQSQQPQQQQQQQPQLPDFGLQRIAG
FWRNVADGIRPVVQAARNSSRAAAAAFAESASASLNSTYSDSDAASSNNSSDSTNSTSRK

>A0FIV6_9DIPT
MHRYFALATLIAILAIHGGTEARTFTECQLAKLLRTTYKFDTAKVNNFVCLAAAESSLTTSKTNRNRNGS
TDYGLFQINNRYWCSTPGFRSSNECRVACSELMKDDISKAVTCANKVFARHGYYAWEGWKAKCKNGVKDL
SSCM

>A0FJI1_DANRE
MTYIIHLTVFMLIVNIERTCCLNPPQDLKIVKSQLLWKPPEVDGDLRYSLQYKLDSKAEDKWYNVSSHIN
KNVFNITDEFYGALFRVRAEKGYHISEWAISNRVNCVNVNSCAPVVNLSEKPGIVSLTLAHMDQSLEKEH
GEHLEFNISSWSVNSRENPEEDVVINSKDYIFNDLESGQIYCFQVEYLLYHKPYGKASKERCVFIPETPE
AKKKRVLIYGPLITCFVLMVCGFCIFLFCKCKSNKRVQSVVKEWFQPFKLDLPDHYKEFLSSEFPVGLAP
SPSIPSLQSDDFIILKENADVEENGQEAEQQKTEIL

>A0FJI2_DANRE
MDWTLWICFLVCCTPGLIAGEQKKLNLTFDIWEGNVTVLWDPPNKAPVPAFYQVKLALDTGPHAKWNIVQ
ECDMITETKCNLGNFIFKPTMKVKIELISGNNESSWLVIRRIRLDYIKLLSPDFDLFSGPNTVKVKIHRK
PFLKELFPYGLSYSAILYPRGQESKAKTLSDDDDEDGEVEFTDLSYWQTYCVRVRLEITSIAVSNTSLPR
CIHPPTDLSVVISIGVIGVVGVITLFMLFVCFFLRRPGKMPAVLKLAVNGWLPMNVGQTEVESVTQKGWL
LNSNKIAEKTKAFDEIEELSEDEKERRESTDSGVSIGQQDSIKNRPQREEDSGCGSLTGTEDSLSSGRRS
LEELPFLDGGGNSSSVEGTREDSGLGIQTQDISDKPKPMHDDLLSEIVVIGDGYRSQSPSAEAETETTIQ
CDEDANMVSRTNGYRSGQVTCLCSDSETCMWCKTRKHLTDCDSFSHKQTVNDNDRSSYLKKSPLETVNMF
GLDDLSCHSDKTEESSLFITCPLLLKEPYKLDTLPLTLGDVELTFT

>A0FJI3_DANRE
MDVVLSTCLCFLLRALSASSADGPPEPRDVHFYSESLRNVVRWTAGHGSPSDTVYTVEYAIYGDADEKIP
EQVRWRPVDQRISVSQTECDVSQETFDLRDEYFARVRATSKHGQSVWSESIGRFRPLSDTVLGAPLVDVT

VRQNHIDITLKGPFRWRMKRMKKEKSLWKIIPNMIYKVSVFNSRSNRTDVFRLTNGSLSLGELEFSTQFC
VVAQAQSESIPLSYIPSQKQCVHTPKDPFRDQLLAAMLGGVLPSALCLCVMAVLGALIRCYITDHRQKLP
KSTQLVQLSEKLQTFKPEVPQTVIFNIKLDDSSVLRPALPLPFSVEDSSEEPVNAVPQPAGSYAQQQPLP
PAGPASLSGDLQDGDSLSLDSEPLADHHSEAGDYAFVIRAKVDHSEVEVSQYRTQGHTADPQDHHIEEEA
QIFVDWSPESGELKIPLMGLLGLEDESGVQTEAVSLLPNLILTQTSVDGCEQEDDFSMMERNWGLVIHSS
PE

>A0FK01_XENLA
MNSPVPVPYCLSLSGLRREMGGHVHQPVGYYLPSQCCPSPHPPPSPVQAAAQLENKELWEKFNSVGTEMI
LTKSGRRMFPEFSVSLSGLDANLLYTLCVQAVPEGDARYRWRDGAWSKSDRAEPGPPTHLYVHPESPASG
NRWMERPICFSKIRLTNNTLSQGGQIVLQSMHRYSLRLYVIPTSNQGVHSAASVSFTFPETSFIAVTSYQ
NPKLSLLKIEENPFAKGIKFFKSQQESHSPQKRLGRLEPEEESSPGCKRSRESREEENVGSEESAQETQR
VSEESAEREEKYQSFMAERTKMEEAGENRNDGRLSRHFMGRIGQNVKEQEGRPHINMSVPPITEHYDSIA
QRLAWLSPPAVPQGPMGPYSPAASVTGYRFAIQYHANIPPFHSMASGALLHSLGPNFPPNHWTSGQLNQC
VSGPHPQLFQHILPPPSLFLPPQPPCLGI

>A0FK59_CHICK
MDPPAEDFQQVLSIDQIRSIRASNNYVERPATCFQQARSNPSLSQPPHKQEWTQDRLASSTLQDLHRSHS
QQPHMPPLQQAMSHSSTASSVSQSTTASEQRLLSALTPSHSGHSLIRTQPRAGELKAEESPLKGAVEKGS
LHGGHLFVCEECGRCKCARCTAARGLPSCWLCNQRCLCSPESLLDYGTCLCCVKGLFYHCSSDDEDTCAD
DPCSCGPGSCCARWAAMSFLSLLMPCLCCYFPTLGCLKLCQRGYDGLKRPGCRCQTHTNTVCRKISSSSG
TPFPKALDKPV

>A0FK72_ARAVE
MGGPGGPGGAAGGAGAGGVGPGGGSSGGAGGAGPVSVSSSVVVSGAGGPSGPGAGGVGPGGAGPGGIYGP
GGAGGLYGPGAGGSFGPGGGAGGPGGPGGAGGLGGPGGPGGAGVSGGVVPVAGGAAPGAGGSGPTTVTEV
VSITVSGGQPSGGVLPGGSYTPAAGGSSRLPSLINGIMSSMQGGGFNYQNFGNVLSQFATGTGTCNSNDL
NLLMDALLSALHTLSYQGMGTVPSYPSPSAMSAYSQSVRRCFGY

>A0FKC7_CHICK
MAQSAEVQPSFEDIFNILSQVPAEKLLSLKHKLKHLIFAPSSKLLQAMVLLTLGQEADARICLNALGDNL
AALYIHQTKLGTAAVQKDGGNSQHPQLDAGAMAFLAQIYLLLANEKLCSHEAVVKAEQAANNASRDAQRD
TLNSIPVGDQERYGLAISTVDSDSKFRTLRSDVSTGFLRMTSPNNTVKSSPMKIRKTSDLSGTQTLQSSG
ISDSFTSLLISQSPTAIFCTPTPSCQSSRLCEVSTSDAGQPDGERQSHSLQETGRASSPSSHSRQDTNPQ
VPHLGKTLQVSSSRLSLPIVETQLPILGAVGQPVESNDISSTVIAEPQVPKESRDQKQKLSTSLPYSRMT
INTGPACIPIEDSYIPAGTSNSAPASTSVCSFPPQTYFSSAILRPLQSIPYNVPFPPPLHSSPSPTGPPP
LKTVEASLAPEPNGEKKKFFTFVVLHAWEDEHIACRIKDLLENMGVPNGATFCEDFLVAGHNQLTCFQDA
MENSAFLILLLTKNFLCHQCMFQTNSALMESIQRPSKHNSVIPFVPKENPLERSQIPSMLSVLVALDENS
PVFARTVQNTFTPEKINERKAMWCQIQQVQEQKRKLELYQDHCQTLQNLGALTLGSLPQMSPSAMQLNQS
SLEQLLEQLLPLQSSQQCHPPVSVSAATRPLPRAGHPTSAQVGPSPSQPLNLPSGQHYTTTDPGGARSII
IQHARMVQIGNHNTMHVETAAPGPQDSEEESRENA

>A0FKH2_LEPDE
MPLTLHGYNLSPPVRATLLTLKALGLEYNHIPVSPAAKQHLTPEYLKLNPLHTIPTLEDGDFVIYDSHAI

IAYLADKYADGGALYPKDVEKRAIINQRMYFDCGTLFPKFIEVIRAFFAGAKTISKDFIARITEVYTLLE
TLLENSTYVAGEELSLADFSVVSTVTSMNTVVPLATNRYPNIIKWIERLQELPYYFEGNQEGVDKLAAAI
KSMMS

>A0FKN4_HUMAN
MKKSIGILSPGVALGMAGSAMSSKFFLVALAIFFSFAQVVIEANSWWSLGMNNPVQMSQDVGGHGWLRAH
VLRPWLRPVQDRADGALPLQVPLVLLRQVQEVHGDRGPVCVQVVGATQHSAPLPGPAYL

>VMPA_LOXIN
MIKYIGVFAFLVGGFCHDFETVISNQDPIVDGMRLVEGDMLFDDGPLFTERNAVKYDQQLWPNGEIVYEI
SPGLRQYEQIIREAMRTYEDNTCIKFRRRTNEADYVNIHVGDRCYSRVGKSFRGGPQPLSLGRGCTDFGT
ILHELGHSVGFDHEHSRADRDEFLIIHKENIKNGSEHNFDKLWENNTRTIGPFDYDSIMLYGAYAFSKDT
RKFKTMEPVEPGLPMKSVIQKGKLSYYDIVKVNKLYKCPPVNPYPGGIRPYVNV

>A0FLQ5_CAEEL
MASNSTVGMVTEATVSLIEAVSQEAVEASNKGGGLSGGAIAGIVIAVVIGVLLIGVVGFFAFKYVKDRRK
NHGEYRPQFEEQHHAKDLPYLQPPNVEGLI

>A0FLR2_CAEEL
MADVWNRSSTSSFIRKRLRASRKSKKKIEKEGGHESSEASVHQAQYSCENNAEATKSGMLLPCYKRLLKF
VNRR

>A0FLR4_CAEEL
MEFSQELISIAVIVLVCVILSICCICIWMCRCRQDQVRANEEKNDRATQLQNFEEGQAKNGVVQASIKPG
NRLNIKDISKSTDTVRSHREESSRSHETIGQYEERITGVSNASNNNSRSGAATGIAQRFRDEEAAKNKRL
DTISEIAMSNDSLQRNDSKTNVFNNIDGSRKTISPMEVDLNPGLSSSINLEKI

>A0FLR5_CAEEL
MNFQAIICSFILGVGLVLICTSTGTVDEDTNFTKTTCKIIFIIGCVMTAIGGLAYVYTIHEVLVRAREKF
RAKKLRKLEQQNAQLKTVSIRIEGEKNVPA

>A0FLR6_CAEEL
MRSNSSSRLWVGPLMLVIIIALIYLLSLMPTTPPHLANNGMIVNDAHTEYAMKGQAQVDWQEIYKNHASI
EDNLGFDPCRHGGVTNETDGSCVCTRWYSGDKCETPVCMNDGYFNHTLGRCVCTSNWVGEHCIFRCNSGV
VNKTSGLCECLFGRPCTIQKCINGHFFDGKCACYEGFTGPACTICDGTVPTIQCDEVIRKRGSVNSRLTL
SGLSFCIITIGLLCVGAHRRRSAMNPVTEETWYRVFHPNNNRPFRCRHDYMCGGSWVPRDRALIVAPGRV
SMSSTNSRVHRLATPPPSYTSVDDLNSAEAQTPPTYEEATRIEIENVIAEEIESEEAKDDVGVTIEIPTS
SDEIVTEETTELNTEST

>A0FLR8_CAEEL
MHTIPITTDSAASWDWPLQHNDGVVKVTNTSEKFEVGLDAGFFGPNDIDVKVNGIEIIIHLRHDLLQNRP
TEYGIVNREVHRTYKLPEDVDPSTVRSHLNSSGVLTITANKL

>A0FLS3_CAEEL

MFGLAFTCLTVLVTVAAAGGCSAPSNIVSETFSSAKQCFLDYSNDAYLYGCGVTASWVFSTTSECFVSEY
YKNKYNYVGAVSAKYNGSTICTKTPCGAVIKVQSNCSQAFYDRINKIPSDDYENEC

>A0FLS4_CAEEL
MKENLRKPPEHFPLGSHSTILHVPKSSTNYSFRIRNIHNVKWKLNVETLNSVPTDKINLKLSKELNKNTD
TNKIDSIQIDYGFTIKNHVNSNNSIVVKGTFTFGGNENELNVEMSENEKIIEDSNGFYDLKTQKLELECS
FIVRSAELCDFVEYLDLYNFNPVIHDTEANVLGVRMFLNKKIIALQSPTFLALIENQTVLSSEQLANCKF
EDIHDFLQLIHGVDLSLDEDNIDTFLMLAKRLDVPRVIDYCKRELIAGCGGISKKKVTEIAIKWAFWDVM
PEIVASAHTLADLKKQNWDLNELPLNVFDMITRKCFE

>A0JCK6_PLUXY
MSVIALLFVGVSVVFAQEQCRTPNGDAGNCILLEKCEPLLAINRIEVKTPEDILYLRQSNCGLFMKIKPK
VCCPPKTQWSSFTTTKPFVHPSLTSALPTTPTTTEAPVAQKTPDVYDDTEDGDYTCKPGVKPPKAESFCC
GVESSSGSDRIIGGNIAGVDQYPWLALLEYNNTAKKTACGGSLISSRYVLTAAHCLGQTAWGYAVKVHLS
EFNTSSYPTDIVETDGGGFEYVKNIVIRIERHLPHPGYVSRVEPVLHDIGLVRLARDAPYTEFIRPICLP
TSDITAIPHSYLDFWAAGWGSDGFRFSELKKHIKLPYVASQKCKNAFYSHRKPDLIQDTHLCAGGEKDRD
TCGGDSGGPLMYSSGDTWIVVGVVSFGSLVCGTEGKPGVYTPRLQVYGLDCSEYGVIIDDLLLLALKYS

>A0JCS2_9HYME
MSTTSLLLLLSVFGTAWSEVAPSIVFGMQETRIRNNITVNENGLPPGSVVIGGSVLKQQNNEYLTIADTE
KLTVVMADMIKLPSQLSVLGLNDSDISNLLNVSAQERNAQVIRDLRQSLTALEEICEDMSQLVDRSLIKN
RDYPKKNSLFYNQKYVNEAMWYTTLSLYWSKALSCRLTLEKSMDNIRRNRKITDSDIQRARSKMVSLLEY
YGKLARRSHDVISPA

>A0JCS3_9HYME
MFTKLLIVLFLIATISTTWAEPTTLRNLSLKRQKRDTGDQPRRVVYGINSREVKNNFGLIKSTGDGASLN
SGVSIVTGSNANQTENSFSNIQYAGSVYVVLPQWYKIENYQAPDICERTKGDKISSERSQIIQRMITFLE
DKITALKNQSRVLDKTHNLNTVYEAPNDRKDDLKKRLSMLIRKLDTLSSRLNYQQERLNQLNNNDSKGLE
DIFDRDFGHLTLVVEITQLDLLDLATSFNN

>A0JCS4_9HYME
MLSMKLVLVFLVAVFGTSCSAQWNSASRNYPYPTATQSPTVRHDRYQSQVPAKPSIAYEDSHPNQATAEL
PTVRHDRYQSQVPAKPSIAYEDSHPNQATAELPTVRHDRYQSQVPAKPSRAYKDSHPNQATAELPTVRHD
RYQSQVPAKPSRAYKDSHPNQAAAKLPTVRHDRYQSQVPAKPSRAYKDSHPNQAAAKLPTVQEDGNQNQG
TTDTLSVHRNGNQNSMTAELSTAQRNGNQNQGTTDTLSVHRNGNQNSMTAELSTAQRNGNQNQETTNSLS
VHRSGTQNSLPADSSTQPSYLTLLTNNLPGTEGDGYQNQDSLLSKILKHSGPVNIQTGNTYMTDKNPVEC
SGFIAQEPQKKDLSTSAQAQSPLFNYFQLQEPLAQESINAEKLRSVGTKVSDLSHDINRLKSEYSNLSYS
IKKVDQFSDGIDGILETLLKRNY

>A0JCS6_9HYME
MALAKTSIFLLFFIVGTSMFLDGSSTRLAYAKPGLANAIYDDITSGLAMMIPMMPSPSQQPRSNLHKRKY
YGSITNQEGATFHDENVRGTVSYKSITIQNGNHFA

>A0JCS8_9HYME

MKRSGNNRRAFYVVQFIELPFEGIDDYVCVPYTWMVVRRATDQKAAVAYPKGEDPFNTRDRVKRQERYDD
EWRFYMAAVKYESDTYRDAEYWIATRNDYGPSVEKESTMSATPTRLTVNKKLRSANQSNSSKSNNNPRKP
LPRISIKRPALSEPKKHFNEKRLKLDEAAQSSSVVVNDANAQPGCLKREEPIIIQDNQSMECNLTEGTAA
AASTGESSSSQRTNKSVQESSPTQEQQELNMPLMNDVTPTVVIDDDEKVQPTSVIEHPLFSIQVLSQVAE
QPELVQGPNISVAPSMKMTHNDQELHTNDVVNNESTSLPLPNLLKMSANLYSHITGARRLVPIQEPVEKR
IRLMNFERNPVLAAQNQSNFMDQTGSLSTHSVDKQSSVVSQNQSLLAHDQYVTRPLQNQYRLTNQDNSEQ
ELLRRPTESNNFKHRPNSRTIRMRTPSNRQNSTHSSNPSYQISELPQQRQSQSNQHDSQRQQQRPKIRVG
ISITPKNNRQKPTLQGVTKTTPAPILSKVRNSIRQSLSIPHNDYLLNEHQLQASQNESLLVNGNHQNVMN
NACQTDECMVEEPLSPYHEETSDSDAVTDHEVISDHEMSSDEAPVETMNNRYCSGIASERSSANRPTATQ
NLTSPNNQTHPRVVLEQQMLDNFSTLFTQMGSTLRYTCDMYNNLRSSILETADTYRNLLDAVERFNGGRN
STSNASSLSNLTPIVPQATEERHIEVTARAISSHENQHSNNVSDKAPKKKHNNLRRFVLPPEYDPHDTRW
TLKYPTNLPGLVELMPQSDIYVSYGELKYCQQVSKDCKSLARRLLPEVFNRKALGVCLSMSEKAQASNNV
GSNLRPELDEHASKVLLNFVIDYGLQCGWNTDLKPILKTLHSKVQEIRLRSGVMVKC

>A0JCS9_9HYME
MIVLKAVVVLCVFILRVSWTQAMPDGKKTVEEGRESLFFTYVERLFTQKSTPSPMTAEDRSSKVSTSRSI
RSAEEDRFTAAAVPGTMTTKDQSGFIKFLPIVLLFRTAREKMADVDLTPMTILLLVAFVLIVVYIFVISS
YLLRKTSQKRYDVKQPPIQLCKLVS

>A0JCT0_9HYME
MAEESTGDSKQGHLSPRSPSGAGVAGGVVTKPSSPRSPGAKSTRRVRLTDPQLEAASKEELAGKWREQES
YVEILESQAAAQEGDLASLKESEEKFRQQFTEASYREKILVRRLASKEQELQDYVNQITELKAAQAPSVS
SLRSALLDPAVNILIQKLRQELITTKAKLEDTQNELSAWKFTPDSNTGKRLMAKCRLLYQENEDLGRMIS
SGRIAKLEGELALQKSFSEEVKKSQSELDEFLQDLDEDVEGMQSTIYFLQQELRKAQDSMKLLQQENSAL
KSLPIENNLSNGLSPHTPPIIKKEEEQDELENEVMTKALVIEPEEYKEADEDGARTPPLKNQVAIPATQE
NDESSSDSAALIIKVENEELTDRENEEEESKSGRTLRRTKGAKCCSSGSSSAGDKTNGDDSTSSRTTNAL
RLRDRGDCRTVDSRGDLNRERTKRDKSAHSSDEEQHHQIRDCGRLKKKRRESILSIDYNDGDDDAIVLTN
GETLQSDPE

>A0JCT1_9HYME
MSRTLGSFFIKTVKTNSVYKKSISRDFGVSCPRNGRGLMYTGYGEPAEVLKLTEVADKKPEANQVAVKWL
LSPVNPADINTLQGKYPSRPSLPAIAGNEGVGEVAEVGGNVTDLKVGDRVVPNANNIGTWTTRGTYQADL
VMKIPKTFGPVEASMLNVNPCTAYRMLKDFEKLSPGDTVIQNGGNSAVGQLVIQLCKAWNFNSVNVVRDR
ENIEVLKKDLKAIGATEVLTESEVRTTDLFKSKKLPAPKLALNCVCGQNAVDVLRHLRAEGTMVTYGAMS
REPLTVPASALIFKNISIKGFWMSAWKKAHGNSEANTTMYEEIGKLFEVKKLQPPPYKVIPLSDEKAVAQ
ALKMDGKIGVKFIIDLTT

>A0JCT2_9HYME
MSLHCNPLIYLNMGGEMLYVLQQRLKAQKINSRKTAIVLDDITAALVHPKMISAVFTDSPISSLSWVRST
LETIALCSIMRLDQNSMNKLFDLMMMMVKFQLSTATGPREIVLLTLNHVDALRGMITRSGTHERITVIHE
LLIKVTIKYGKLTCNGIWGARNECLDLLGDINVRVSILLKLGLQNEDTSFNLNPRNYNEKFDLMSGELGA
IELLEIPQNLRVGSLQLIGERVTFLGRNM

>A0JCT4_9HYME

MKSHRKLRLLLIFVSNLFVNGLSLSINKLNDRKIRENSAVDNPGNGEWRLRIVHTNDMHSRFNQTSKSST
DCSEKDAKKEKCYGGFARIASKVREINETSTSPVLFLNAGDNFFGTPWFDIHREKIVLDMMNLLKPDAMS
LGNHEFEHGPQKLVPFINNVSAPVLCCNLDMSKEPELQNSKLLPYVELSVRGHRVGIIGYLTRETMDVAQ
VDNVKVLDEIDCLRKNVKELKDKGVDIIIGLGHSGYVRDLEIAKAVEGLDLIVGGHTNTFLHNGPPPDQE
KPEGPYPTVVVQPNGRKVYVVQAYAYTKYLGDLEVTFDAGGEITHVEGNPILINHKVPEANDVVKALDNY
RKEIQELGKKVIGETLVPLDGPKRCKMHECNSANLLADAMVDYVSALHYLEKDKWTDAAVAIVNSGSFKS
EHEAHTNVIVYSIIVYDEIMVTVDFTGKQLLEAFEHGVVNITYNDVNRTDYVDGRLLQVSGIKVRCLREI
IFEKTQANFFSYQKLHYNFLIFKISYDLLKPGGSKVIPNSVSIRCADCQTSLYKPLDENKTYKVLTGDFL
RRGEDGFTFIKDLPWQSSGRIYKPNQTNFKVPHVLMSN

>A0JCT5_9HYME
MTANKVVIVLCVGILSVSWIQAIPDQQETVDHVIESPLIEYFKHLLDQPSTPEPMTAKDRTSEDSTLRSI
TSAAKDNVFVPASTAPMTIEDYIRLTSLFSLKMTQKKTAVAVLSPITIKLLAASVIVIGFFVGILFYLLR
KSFQKMYDVEQPPVQMSKLVETRH

>A0JCT6_9HYME
MCYSEELLNLWSNSGPILSPSDGLDKQVFIGVNNLRDVNRWETIEGESLPYDNWSSWWAGGRQPSRPNEQ
RCGSLLRQGGMDDVECYLKLGFICESS

>A0JCT8_9HYME
MMMIIKTVLILWVAILSATCIEATSEEDTVLRKWLETDLFSPKLLLNHNLALSDENGAIKPFFPLIIPVE
FSPTPQPEQVSSLVAPPVHPEAVSHPTPPLVPNSVTNPPKTPADNRPPKTSTSPPPSSTTDDDFPKQLLP
PSSDVTENRPFKETPFIEVSTTEDSIFKGKSSSATDTVKDFVLDELTSLSAPFAAEEITSKKIKPPTGVL
SIEIIIAGLISAVVFLIILLIIIQSHWRVHKIAETKYEKCNEHSHIVYQNTVEKV

>A0JCU0_9HYME
MNKFAYLMLLPLVLGQDLLVSRNRGRATIGVSKSYEFHTTPATFEDARKICKQQGGDLAIITSQDEEHKL
LDLWSNSGPILSPSNGYDKQVFIGVNNLRDVNRWETIEGESLPYDNWSSWWADGRQPSRPNEQRCGSLLR
QGGMDDVECYLKLGFICESS

>A0JCU1_9HYME
MQISQLTSSKTTDDLKTRSEWKFYQAQTKCRTFCQLNTYGIQAMPDRQEAVELDRRLPLPEDIAKWFTQK
PALEPTTGKNRSSDDSSSRLMTSTPNPSFPEERSGTNESFSGDGSGEDDDFTEDGSGLPTLTHKLLY

>A0JCU3_9HYME
MSLNNSAFVLVSALFSLSCTQALPTHHQWEAIQENPVADPNLIDSMAKLYSTRSDSIADGIMPPQDDVTK
SAGYNRGTSMILDSYPEVPPKRPYSENFLSGYPPIYNLDYDARSRFVDRPHPLVRTISEPEYLTVYRAIS
GPGFIAGNGAVAGNGVFAGNGVSAKPELFSTPRSIVYQPENVTEKTKSCLSSHEDLLSKVRAIEKALQEL
TSAITCNEDEVTSNTATTSSSPPVLMEATVKFNPETIKSSEKRVTTSKPATTTTSTARPLFPIDPDVAFE
LENDKSSEEKEAPLDTDTTTPSAPGLVPTEPNAAFELGPVESPVETPYGNDATAKIERPF

>A0JCU4_9HYME
MSLNKSALVLFSAILSLSCIRGLPTNREIKTIQENPNASITLNGLLSKLPITDLGSPISKLDHAVFGLPI
GFVLPLSQQILDDLAVNSNKIESTVERPGMAVVIPLSSLTNIYALKEAIQIMADAGSHSEDKGTLSTVTT

TETLPVLPAPILTGKKPQQPTEKRKFFSEAVMGSAKSMTLSDIVARLYGNVTNH

>A0JCU5_9HYME
MLLFTILSIRYSCPECTQQELCRHLMLMVRHSTDHLIFILRYSINFFYCYTGITTVQHRIFNTAEEHRHT
GVDTQVLHSGVKMSPFLTPLFLQCTSNIVFPWK

>A0JCU6_9HYME
MKRESTMFNTSLFGFLVLFLGITSAEETPSRLMYVLGLNNTRAGNTLEPGTPSNGSHSAEGVTIIGGSRV
DQANNTYLTLEKVKDAQIFLPPAYQSENGEPKSLAELMTGPIIHLNVMNPNRRLIVQRNIDALKKLSLKF
KDQLYGLMMKDRKNISKLSSVLQSTISAELRLQDQQKMLDDFNIVVPGSPPKIDHFDRDLTTLNLTIESA
IAATITSA

>A0JCU7_9HYME
MIFMCITLSALYLDQVFCGFPGHCAKGQGTDECEYTIESRPQLRKSTRGPLKWGKEKGELIEEELKSHVS
RPNRLKR

>A0JCU8_9HYME
MLYCHCDIKAIMLSTKTAVVLLVTIIKTSCTQPLSTGRNGYQNGKTTDSLIVNRSGNLNPVATDSSSYPS
SYYLGLLNSQSSSDQTDGHRVESSLLSEILKHPGPVNIQTGNTYMTDRNFTDVFGSISQDTGHPESQVHT
QDLRALENSQRRHHEELISEIAFLKDELQTIKIYLSTILQSQNTMTLALQKYNEIHK

>A0JCV1_9HYME
MLSMCITLSALYLNQAFCSSPEHCAKRRNTDKCQYTIKILKTAEYCLRDYNVRQWGIESFPRKNCFESIS
NFVEIDSIISSVVVCITNINSETTNR

>A0JCV4_9HYME
MISMCKTLSALYLDQAFYGSPGHCAERRSTDKCQYTIQCIRTLDWLRVCSYKKLGGCRRITYALVYFTNF
RIFKQLQIILLTTDMLI

>A0JCV5_9HYME
MSGSKVVLLVLAVGLISSFNGVSGRSANDLPSSSLMVEDGGVPPGYYDSMFKTVETALKTESHGARTIGD
QNTVQESKSSGFLNSGSKIDHSDNRHDENGSDDKGPLAFTLGTHNTFQSSNSNMTTNMGSWVTYGNNIYS
GKSSNP

>A0JCV6_9HYME
MLLVKTTLFLFILTAGIEFFTYDNSKGVANAKAVILEEDAVKNAVALYNLIRIRKNSIDYENIRKPTSLN
IFQSVGIGNQTGSDISIDHGAITVRHENIDRYINSKGAISHGSIFMDDGNEHNKSNSSDYHYSGSNTLEV
NNIFEGINSSGNKTYGSITQQENNVYNGSNIEGSILGSGTLIENNRFSAVNATSNNEYGSLTIQKNNTYD
HSESKHHIYGSHTVIRDNIFMQRNSNANISWGSDTTQEENKY

>A0JCV7_9HYME
MLSYKVVVLLAVFGAPCIKAAPADYNEDYEDAMLLLDEVENDLEFSGTAYGSQSVQSGNTYSGDFNGTIN
YAGQSIQHGNIYEGNVIDDVQFAPLSIQENNTYGANIIGTVNYGANVDQRNNSYNINVVGNVRQGSTTVQ
RNNSYNGTVIGVIDVGSMTSQTDNTYQGNITGNIIRGSLTDHTKNIHDGDLVGKISHGSSTYQIGNTYGE

NVVGNINAGSLTSTLNNTYLMNEAGNVRHGSYTNQTGNVYEEQVLGNVHRGSSTVVHYNLYTGNPIGNIT
HGSWTHQSQNSASKARANKTESGSHTDVEFNRAIAVGET

>A0JCV8_9HYME
MKLYKNSLLVAAFEILWLQNVSAGLVPKINPAGDLKIDDPFPDIDSILRFYGTLNNKTNSEKTGANISWG
SQDVQSDNDYSKDDGGETLYGSRDIQIGNIYPENPDGTNVYGAIVVQKNNVYHGTGKNVLYGASVTQIGN
VYPENSGEKDIYAPVVIQEGNIYPENSGDSAVFGAIVNQINNTYPKSVEANKDALKPIIYQSGNVYPPTS
QTNSVVGAVVKEEGSRFHPSQGHHERPIAGMTIDMVIRQYKEIKRIIDHHLRTDQPTSKRCTDGMMDLKK
LDQIESLYLTVGTLPAQLKALQEINRNRKIICDSIGNQCSKHADMQKWIASSKQIVMKSTERSIERWESL
QKC

>A0JCV9_9HYME
MLCRTILTLLIASIGISRIEAREIGERTAQLNKTPVDGVNTAIEALTVQNDNTFNTGLNDQSIYGSVVKV
DNNIYESPNSRQPTSTQTRIVVAPGGPGHDGVSYAKSEERDQQGEPKNNVSATTYMKGNTFNLAPDSKNV
FGSYIEQNNNVYRSRGGNNQTQPNEEHKSSNRGNIGLTAETILTNSVFQTSGNMEINAGSSITRNGNVYT
SDKNSQTGEKTINGTERQDNSSDIMTVLLKDIQTREKIYKDWKNQLYFGNQSNIQQDPIESKPNSSFIFY
DSYPNSETEKNAEDTKEKEASAEKHNGPNGQTSLSPNLFYSFTYFLIILIKLCVFNSNS

>A0JCW0_9HYME
MALPKSILVLLAAVVGLTWVQAFPEHRGKIDYPRQFFDVTDENTIIQDDNDNGSIYVAALTTSKSYESNK
MAIRQATENGLSEAATLHSLPKSKIRLSLQASLRLVQSIYETNDEMEATQKSLSSSDRDIRSNLRQTLCN
LVSGVDMPLDVQEIQNVKDAICAPLFSNNA

>A0JCW1_9HYME
MASLVALSIIIGALCQGIEAFSMQGGQNNGTIEFPTIVYGPINQQLEHEYQSAKPVHTLGEPATQEGSVI
YGSTTSQNNYKYLTIGQAGNVCIMVSTINEPLFVPIPGLSIIQDASKGGLILDSKDPTGTQIKKIIDAFN
TFQQSFDHIMRIASPFLTQNEISLLQQKSDKDDFTRQTKEIKWLTTVASQRMKLVKLYEQIIFISNSLRN
NLEISREEIVSARGVQATPMQHQWIEKQLNPTSIIQALPSKVTSLPFNPIDGSDFGNEQDNSQRFDINIG
SNYVLPFIDSIIGDTNRFNSEEKATIMAFITSSIQNLLSELKIQSDDLVHLLDTLIVKDEVTTLQRLSMM
TDNRKQVRFVDLLTVIKVHQSNIAAVQDVLKSYQTIDVWRTSLQRNEMTYELVALYYTVSSISNANNLLW
REFYLILNM

>A0JCW3_9HYME
MFNTSLLVLFVVILDIKWAQALPKGIDIVIGTRNTQNGNDIDIGRGPKKINSTAGSEYVFGSVTEQKGGK
YVILGRVNKTHILMPEFSDPNGVPRYFVESIDGPSDHVNVMNPERRRVVQEKLKSLTDLCQGLKNTMYQV
LYGGKNSPATINILYDIISTEAKLQVQQNLFREFNAVMPFSRSKPDNFDGDLKIIKLEIEQLVLRSLAVT
LSNN

>A0JCW4_9HYME
MLSTKAAVVLLIATIWISCAHSTYTYGSATLTDQKYDITSDMKPAVSSHGSIDEAFGESLEKNYNYCIKE
MAFLVSELSVIKRDLLILSRSQRRIGLNLERAISLPS

>A0JCW5_9HYME
MFVNNTLLFLFIGIIGSSWLIDERSGVASALIAKSRRTYGRPTSSFGRGYTRPSSRPASQNRWMPNPTRR

NAWLPKPGVQNKWPMPKPNFGVNNPGLPKPGVQNKWLMPKANFGVNNPGLPKPGVQNKWSMPKANFGVNN
PGLPKPGVQNKWSMPKANFGVNNPGLPKPGVQNKWSMPKANFGVNNPGLPKPGVQNKWPMPKPNFGVNNP
RLPTPGVQNGLLSPHSKVFNTQPGLNEYENVGNPHSQTSANSLVDSVLAGFQSMMNNPMNYNSNRQPAGI
YDGTYSQPPGTYDGTYSQPPGTYDGTYSQPPGTYDGTYSQPTGTYDGTYSQPSGTYDGTYSQPPGTYDGT
YSQPTGTYDGTYSQPSGTYDGTYSQPPGTYDSTYSQPTRTYDSTYSQPTGTYDRTYSQPAESYGGTFPQS
SVPNDGVSLQSAGTSNETDDSTYLQPAGTYDSTYSQPAESYGGTFPQSSVPNDGVSPQSAGTSNETDDST
YSQPAGTYDGTSSQPPGTYDSTYSQPAESYGGTFPQSSVPNDGVSPQSAGTSNETDDSTYSQPAGTYGGT
YSQPAESYGGTFPQSSVPNDGVSPQSAGTSNETDDSTYSQPAGTYDGTSSQPPGTYDSTYSQPAESYGGT
FPQSSVPNDGVSPQSAGTSNETDDSTYSQPAGTYDDISSQADDTYNGFDSESSELSSDDYPQPARMDNTS
EQSLLLNGNSDGPNPEPNENINVENNLISQYDNSESSVNQHSTYYNACVELLGRDLCVGLDSANSS

>A0JCW9_9HYME
MLLTKAAGVLLVTIVAISCAEPLNDGRSRYQNSVRTEPSNGRSSDDRKPVATDATQFYGSVTVQTGNTFS
TDPKSSNMYGSFTQMTDTVHPQPRTPQSSDQQTKQMATLTGLASSNAELISSLETKVSQLTDEINFLRVE
LSNMKSVTDKVATHLNTIYWKIYRKLNFQ

>A0JCX0_9HYME
MLYITIATLLIASNGISKNSIYYFFVQGIQSEEQGDRRAQPSGVQTSLTGGDSVIEALLVQKNKTYNTGP
NDTSKDSSITMVETDIYESPDSRQLSSTQFPTVSATGSPGYREVIYVRSEEQDHRRQPDQNQIPGKRTGI
VLGSETLQIGNTFIVEPGSRKVFGAVTDQRNRVFRFVGGQNQTQPNGGDIHYGSSTIQINNTFKTNKGAK
YSPRSHLYQKVDVYPEMNVTRKEKKQQNIESQAQLSNSSNLQLSLSYKKFYYLFVLFNCIVQLLSVVK

>A0JCX2_9HYME
MVFAKTSILLFCLIIGISVFPNGSSSGLVYAKPPPDYMNQGLRELQNRIDSQVPVNGGPRSYGSLTNQFG
NEFVVTGVKGEVTFGSNAQQKFNDFNDDQSPALKTYGSITNQGFNSYQVANVGGSMTVGSNTYQEKNKF

>A0JCX7_9HYME
MWALSLNINVNNSCYCSQTFSVPLRVMFTLLCLGLRKAYATILIRYKRKIWMIHHYRNPLAVETKVFRRI
KHEKLIETILLIMLAIQSIIKRIYILTVTSLDDTLLWHSSPHSTILQ

>A0JCX9_9HYME
MKRNGYNRREFYVVQFIEIPFEGIDDYVCVPYTWMVVRRALDQKAAVAYPKNENPFDTRDRVKRKERYDD
EWRFYMAAVKYESDTYEDAEYWIATRNDYGPSVEKKSTISNTPSRFRLNKKLRSANESNSSKCNDHPRKP
LPKISIKLPALSGPKKQLDGKRLKLDESAQSSVVNDANADPGCLKKGQSLSIQDNQLMECAQTKEAAAAF
NGDESTEPRTQTQPTEENKQALSLPVIDVDEPTEVININGKDHSQDFKMLDENSIISQRHLSNNTTDALI
IAAEPSTLAEQTTQPNQHDNQEHVLDSGSSLSNFQPRIENVRSMAPDYNNHELHVNDVLNNEPASHRVPD
LSKMSAHLYSQMNRAQRLVPTQEPVKKRIHPMNFTKNLPLAAQSPLNSMEKNGSPSTQSVAEQSSVVTQS
RSQNSLRVQEMCTKDRREQTTPSRSISISSQDIQHVPKINVGQSQNVAAVDPKNVLQSGTNVALALQTPA
SSLRKRPSVDQRLDRHYNLVAQEQNLTRAHQNQYMSINQNSFKQDLLIGSPENNIYNHQSNSRTIRTTIP
SMKQNSTHPSNPMHQTSELSQQRQSQPNQQDSQQHSHRPSMRIGISIKPKNNRRIPTLQGVTKKTPPTVR
PNVLSSIPQALSTTNNNYLLNALQSQATLSTKSTEYNHTSLPTGPHNVAASAAHDNHNLEMSTCSFQQSP
GIQQSSAHRNINQNLRTQRESVNQYTGTNLTTKRVHFMNDVATSTIDENYLKVTDNACQTNECMMEESLS
PHDVVTSDSDAVTDHEVISDHEISTDEAPVETMNNQTHPKIVLEQQMLDNFSTLFTQMGSTLRYTCDMYN
NLRSSILETADTYRNLLDAVERFNGVSNSTSNASSLSNSTPIVPQATEGRHIEVTARANSGHENQHSNNV

SDKAPKKIHNNLRRFVLPPEYDPHDTRWTLKYPTNLPGLVELMPQSDIYVSYGELKYCQQVSKDCKSLAR
RLLTEVFNKKALSVCLSMSEKAQASNNVGSNLRPELDEHASKVLLNFVIDYGLQCGWNTDLKPILDTLHS
KIQDIRLRSGVMVKC

>A0JCY2_9HYME
MFFSKLSLLCFVAILGTSWFSEGGFTAVEAYPQQYSSQFGAGNQQATTQDGWIWQQYIGQYYNQQPTVVY
RFPETRVNPYENRPVLNFLHNLHNWKMCMLSGFHNCYQ

>A0JCY4_9HYME
MFLKEVVVLLVVTQVGIARGGWNGYPYQQPSEPNENLDLQPKASNGYPYPQPENGRCEYPQLNRPSGYPY
PQPENERNEYPQLNKPSGYPYPQPENERNEYPQLNKPSGYPYPQPENERNEYPQLNKPSGYPYPQPENER
NEYPQLNKPSGYPYPQPENERSEYPLLNKPSGYPYPQPENERSEYPQLNKPSGYPYPQPENERNEYPQLN
KPIGYPYPQPENERSEYPLLNKPSGYPYPQPENERSEYPQLNKPSGYPYPQPENERNEYPQLNKPSGYPY
PQPENERNEYPQLNKPSGYPYPQPENERNEYPQLNKPSGYPYPQPENERNEYPQLNKPSGYPYPQPENEK
SEYPQLKRSSGYPYPQPENERSEYPQLNKPSGYPYPQPENERNEYPQLNKPSGYPYPQPENGRSEYPLLN
KPSGYPYPQPENERSEYPQLNKPSGYPYPQPENERSEYPQPNGPNKYSYSQFDRPNRYPYAQPKSGSGYP
YAQPY

>A0JCY5_9HYME
MFFSKLSLLCFVAILGTSWFSEGGFTAVEAYPQQYSSQFGTGNQQATTQDGSIWQQYIGQYYHQQPTVVY
RFPETPKPYPDSLNSQTISCTDSGLLKTRILFESTKSENEPINVDSIQKQGSGLIH

>A0JCY6_9HYME
MFSKVVVFVLATLIGTILGAASPTTQVDKEKRQVVQYLNCSVLTGNRIVISNTEFNDIESPCRINVSEIV
LNNNVFPTFEKQFDISAENVTIINNLFYGSQQDHRIVGNQINLSRNIYAGQHQIHEVAGTSVMVAVNLYD
GDYQVVKLMGNTIMEAGNKHTGNLVSHNLTAVTTEELFKEYSFELPSYLKNTVLESKAAMVTDNEFGGTH
TNSLPSGSIVKYLLRVFSGLKIFRNFDAPILRQMSELYDQNV

>A0JCY7_9HYME
MLFTKTLFVFLVAILSTSLVPGGRTNGLVSAHSLSARSLDRYGKAIVLATHDVPPEDEEFTPLTVGIWAG
KMYYRLPNAPFYVVVIKEGIQNLTKFIPNPFYQESTYGKELNSTIDDIFPKYNEFTPLTIGIWVGKTYYW
LPNAPSYVVAIKEGASSVTGYIPNPFYRGSISILPF

>A0JCZ0_9HYME
MKSKATGTILTSIKTCTREDNKYKRVGNEEGAEGSVTCQHPLSTSERQMDSAVSTCVAMVSLMIQAISVT
IRIRTSTLFFTKGPFGYSSLIIRIRIVSLGICYKTDLFIYSSLIQRICRYTHLLQYGPV

>A0JCZ2_9HYME
MLSLPIFLLSIGQLEDTNYKTRDYKVYWNVPTFMCHKYGMYFDDLEKFGIIQNTNDHFRGEKITILYDPG
MFPALIKNPNGTITRRNGGVPQEGNLQSHLDLFKTHLEEQVQENFSGLGVIDFESWRPIFRQNWASLAPY
RDLSIELETASHKNWSKNAIKQHAVKLFESAGRLFMEETIKLAKQLRPYASWSYYAFPYCFNLTPNQPNA
SCDPRVFKENDQLSWLWKIEDSILPSVYLRKSLTSHQRSDFIAKELQESVRLSKKFSNRPILPYVWYKFQ
DQPDVYLSENDLLNSFKAILDNGASGFVVWGSYKDVNTRKKCFNFKTYLKRMLGPAVVTIKAIALTYTPA
DDINSDEL

>A0JGX7_TAKRU
MEATKGEHGGQNPGGQTDIPVRYHILESMIPKVVKRRVHALKRLQVQCANIEAKFYEEVHELERKYAALY
HPLFEKVVSKRSST

>A0JGZ6_TETNG
MSFLGVMCSCELLNKKRGEEQTGVCPLPAIKVSLDWLKLRPGVFQEAALDTRQHVWPWLVSVLNSFQPKE
DMTSSAVSPLPEEFELQGFLALRPALRSLDFTKGHQGILEQEDPRGNIRHHRLISLGKWVADNQPGLIQY
RMSEAGLVFITDIPEVALEEPSEKEAPVLQESSNSEQTSNEGGNVGLKSVLSVGKTQSSFSDGSERPVVT
FKENIKPREQSRDAARNQPLKDGGKERRDFSKGSGAAGKLEPKRDGKRKGELKKAAHDKDSESGKQVKPQ
AELRKTPVSEVKKTPGTQTQTTCSSQFIPIHHPGAFPPLPSRPGFPPPAYVIPPPVAFPGLQVNPGFTFS
PGVSVPGPFLQPGIHTQAGSQTTKQTHIPYSQQRPSGPSPAPGLGQGPCGGTMSQGQPPQAQAPSQPNLQ
QSVQLLNQQQQSPTKLVQQAAIGKSPPHHPGLQQWNQAPAALSKIPPLQMSIKQTQHPQQTFFMAAQDPL
KLYEHQMAPPGQAPLSNVEKKAKFPNVKMPDFYWDPSYQMGDGPSVLSDRMKSPVAACPKQDSSAGPRGP
PFENRLDPSTPEPSSQSSSLLPLSGFPAQEFGQSSIFRQGYGKAQPTNAKPSAPTMHQEPSLYSLFPWSP
SLPASSDHSTPASQSPHSSNPSSLPSSPPTHSHGAPPFSTFGPIGTPDSRDRRLVDRWKPDKTGPVSTLG
LDYLPTSATSASSDSSWHQIGPAGSSWTNQESPMEESSVLLDSLKSIWSSSMMQPGPSALEQLLLQQKQK
QQRGHGTMNPPH

>A0JI74_SCHMA
MHLMTSTYLSIRDADRVLNEFRLIVPDLPTSIRSILRTCTSVQPKSISSGVYYHLGLKTNLLRYVELWLC
TCDFDSLQLYINVDGLSMSRSSSQHLWPVLGRIVAPRLSDVFMIGIYGGNTKPAQFNEISADTISEIKEM
TETGLLSVRFNKYIAIKLSAVICDAPARSDVRYTVNHNGKAGCDRCVVNGRRLDGKMTFPNGEYTLRTDD
SFRNQTQYIHHKGHSLFESLSIDMILTFPLDPMHMVYLGVTKKLVTLWIELGHKRLKNMNSCVIRTINKL
ISRCVESTPSDFPRKCRTLDYLSVWKASECRLFLLYLGPVILQNILPESLYINFKCLALSMYLLAHPKFY
NRVTESVRMDLRNFLREYEWCYGCENLVYNVHSLQHPLDDVLAHGPLDSFSAFPFESYMRQIKQSVRSGY
SVAKQAAQRYAEQMSFCDRLQVDISTNDNPVIGMADSRKQVIMFRNSQIKSFHPDNVVVVKGKPGLITDI
QDSGLLRFRQFTDPQNYFTDPFPSTDIGIFKCSVVSSAYSWVSIRDVDCKCMSINCVNHLVIVPLLHTVI

>A0JI76_SCHMA
MSSWQMVEYQCSICNCHCHGPGISRILYLHQACCTMESQTILANWLYP

>A0JL26_DANRE
MRMLYLIFLFVLQKVHSTAVFCPETDDLNSDKLLIGCLGVRLTWLYAVFEDLRSVLEFAVSLRCETGLCP
ADIQDHGHYCSNANTRDLQQIQPVDALDGCCFTHLRCSQELKERNCSRRAPSNNNYTCSYNSSCDMLDFC
DEGFCRCDRMVIDCISFNRPITKQDRDNQLITSQTVSPFVTDLLENDTYISDVVNVTESVDLPLSDIDME
TMFYEVNGTLNETMSNFTNELVPVNDSLSSNFQSTEYPGIYQSGREEEENEAGEKELGRNPDRESTLERE
EEPVEYEDGLSNVTAVLTTEEPAGDNVEAPTTNNPVQWNKLDWTNNEPTVSMALTESQNVTDLPDKNTPH
MTPPTTYVQTHSQPDHHPFDEDDYESDEDKLQLFTSNTLKPTTHTFEHTTLTQTHTTTNYTHGITKPHVD
THVITEEESEESGEEGEEEVQSTQSPYMVLMTTPTKLTTNQSSHHKTTSSVVTSKNTAASVQFPSMKESE
EKYISDESKPESKEDEETEKERDLLADPPNESLESSTEQNYKLSTTKPTTPTQKMDKEESKEDLQSHEED
DKSTTSQTTTIPYRVSQISPFSWVIPAPIPKPVVHSVSPAPTVVKPTTNHKSTTVVPQKPISTRTTSSGR
ATVETISHPQKPPHPITDRSPSLTTTERQESAEEEEEEEEEEEKEPSDSSQESKKIESLRVRGRALPFFTL
SLLEAAGLTDLQQQKDSEECSMSFLQYSASGRVWRDMSALGEMLHCLTGRCPHEYQHYGCYCGQQGTGQP

VDRLDRCCFLQQCCLEQLSVFGCRTNRKLNAHISCHKAKPQCFGVSVCDRLQCVCDRSTAECMAASHFNS
SASSSCSGPRPLCLRTAHSSAQSTNQESSEESDEMSQSQPQINTQSDTVDRKPTQSKPQQDVREEEEDEE
GKEKEEEEEEEEKEQEEEEEEEKEQEEEEEQ

>A0JLZ8_XENTR
MGQRKKACPRLLDYLVIIGARQPSSDSISQTPELLRRYPLEDHPEFPLPPDVVFFCQPEGCLSVRQKRMS
LRDDMSFVFTLTDKDSGVIRYGICVNFYRSFQKRLPKDKSRKPPKPASENGSEGSDGGHPMQPHSAESTP
DINQSPRGKRLSKGMQRSRNSTLTSLCIISHYPFFSTFRECLYTLKRLVDCCSERLVSKKAGVTKGVQRD
TMWRIFTGSLQLEEKPNVLLHDLREIEAWIYRLLRSPTPVPGQKRVDVEVLPLDHQPPLTFALPDPSRFS
LVDFPLHLPLELLGVEACLQVLSCIMLEHKVVMQSRDYNALSMSVMAFVSMIYPLEYMFPVIPLLPTCMA
SAEQLLLAPTPYIIGVPASFFLYKSDFKMPDDVWLVDLDTNKVVAPTNADLLPVLPEPEASELKKHLKQC
LHSMSVITQQRLLASDNKALASMSLNTQPILNLDRFHEGPDAALLKAPSDAQGTPSTEFNPLIYGNDVDS
VDVATRVAMVRFFNSPNMLHSFQMHTRTLRLFPRPVVAFQSSSFLAARPKPSLFTEKLSKTQAVEFFGEW
SLNPTNFAFQRIHNNMFDPALIGDKPKWYAHQLQPIHYRVYDANSQLAEAFCVPAERDSDSDPTDDSGSD
SVEYDDSSSSYSSLGDFVSEMMKCDINGDTPNVDPLTHAALGDASEVEFDDFQEYSGEIEENNGDSENSQ
DNNQPRSGSSTTASSSPSTVIHGANTESADSAEPEEKVMGVLQNHLPVLPPTFTRLAMERRESDVSLGGG
EARRREYDNPYFEPQYGFPPEEENDEREESYTPMFNQSVNGTRSPKPLLRPNSLKLASDSDAETDSRGSS
PTSTVSNNSSNNEGFGGIMSFASNLYKTHSSSFSLSSLTLPSKSRDKATPFPSLKGSNRRALVDQKSSGI
KHSPTVKRESPSPQGKSNNSSENQQFLKEVALGVLEGQGVGWLNMKKVRRLLESEQLRVFVLSKLNRSIQ
TEEDARNDVIQEVEITRKVYRGMLDLLKCTVSSLEQTYANAGLGGMASVFSLLEIAHTHYYNKGTKPAPP
LVDSGETEEKKSQISADSGLSVTSGSQRSDLDTGSIGPSILIRSSSQDSEISTVVSNSSGETLGADSDLS
SNAGDALGGPGGSHLSGSRGAVSDSEIQMNSFTGAIFGKPQMLRPGEKERNVSPGRPQQDLSQRIYLYEG
LLGKERSTLWDQMQFWEDAFLDAVMLEREGMGMDQGPQEMIDRYFSLGDHDRKRLEDDEDRLLATLLHNM
IAYMIMMKVNKTDLRRKVRRILGKCHIGLVHSQQINELLDRVTDMNGRDLSIRPSGSRHIKKQTFVVHAG
TDTSGDLFFMEVCDDCIVLRSNIGAVYERWWYEKLINMTYCPKTKVLCLWRRNGQETQLNKFFTKKCREL
YYCVKESMERAAARQQSIKPGPELGGEFPVQDMKTGEGGLLQVTLEGINLKFMHSQVFIQLNHIKKCNTV
RGVFVLEEFVPETKEVVSHKYKTPMAHEICYSVLCLFSYVAAIRGKEAELKSRPPRPVSS

>K0284_XENTR
MSVTSWFLVSSSGTRHRLPREMIFVGREDCELMLQSRSVDKQHAVINYDSDKDEHRVKDLGSLNGTFVND
VRIPDQKYITLKLSDNIRFGYDINTYVLEQIQHQVPEEALKHEKYTSHLQMCLKTAAAGREDQFKEHGAH
VDSAQAKQDKADKKATSDIPAYRTPLYGQPSWWGEDDDNKLDKEGRRQDEHYSERPNDMTQHEEEINGNM
SYRDSQDQCVYPFRREPSYFEIPTKDFQQPVKPPETQVYEIPTKDTDAVPPVTPPVMQSHASFTIEFDDC
QPGKIKIKDHVTKFSMRQRKTAGKEPAPTEMVSAESKVADWLVQNDPSLIRRPAPGEDVYSTKSDLPIHN
RTLKGNRHEDGTQSDSEDPLVAQPEQEIGTPDHPQLQRQIKREPEELLHNQQAFVIQFFDDDDAPRKKRS
QSFTHNANSPQNDTDPVLKAKAEKRKGTLHVEKVSTNGMGSTAPASKSLSSPSFPQRSNSFRREKTEDRI
SSAPTTAKLPGKNYGSVGKKSKLAQEFAAEYMREQVEVVKQAAEKPMTLPLSTSILQQPASQVQISSQAQ
TMQLTSDVRTGKVKNEEEDNLSDAGTYTIETETQDREVEQARKMIDQVFGVFESDEYSKIASTVYRPVIK
LGEEEPLASEPSVPHKPIMSSTPPVKLSNGLPAETLIERTGSSSKQSQKWVSRWASLADSYPDASPTSPL
DSQKQGLIADDEDVIERTEHQGEADPTVPSRTRRLLPQLPPENVMPTIFVCQESFSDESQRKSLEEPEKR
ISEENSSLLLVQEELDPDSLSDTSKSDDGVISVRKSAKASNTYRNGWKGEESHSREPSVQRTSVPSNEKK
STCFYVGNDDVGSVKQTSFGLSSKDVIKGPESHIKMKVNLHITAETPSSGKAVNQFPKKDNGSAKDPLSF
VRQESFTKETSSSNVLPNKLPHISTHPLLKDLSVTKSNHDYSKETRLILKETETALAALEAKLFTQSHLD
EIENTPCLRDDSLSGDSDVDTASTVSLVSDKNVPSHSQKNRIVSLQKEKSSSTSSIQEQYCQPSARERLS

EKRRTVPADAGTRNVTKRLGMTRSTGARGSLDFTDEERCSSLPYMPVSETVSSDYEHSSSRHISRRKPFG
QTCKDESSRSSNAQKVQQALTRSNSLSTPRPTRASKLRRARLGESSDNESADVERSNISPGTSSANSSSA
KSSTETKKPSRLDILAMPRKRAGSFNVPSDSETTSSVRGMFSGRSIDQSYTSRKPAVAESKQPPKKPLPP
TQKQTPRPRSSSVKYSSSSTSRRRQQGSDYVSTSEEECGSNHSTPKHKHSRASTATQTSRSSSVSRRQMP
NSRQDEEEDEQEVYNNFMAQSVEIAEIARLSQTLVKDVASLAREIHDVAGDGDSQSSSGTGQSTSISSVP
NTPASTISAREEIIKRSLQRTCSSQLVHHIPEASLNYQKIPPGSTGLEDFDQNMNDSREDPSKRRARNIE
EVIFDNLMLNPVSHLSHTICANTEVLTEKMKILFQNTEKNWEEIEAKINSENEVPILKTSNKEISSILKE
LRRVQKQLEVINAIIDRSGHLDVPSSNKKTSSTILTSNPLSRTTNNSAARTESQTPGHVRNYMHKSSSSS
SRSPGSSFSRDDEETYIV

>C2CD3_XENTR
MKSRKVKSVRPGRGRSKGLTDVTPSSSLPPLVEGQVRSFLQVTVSKILWTVPKPPPSVLVRLRWWGETAN
GTVFRPRDSSQTEQKGAKTTTLYAVRCGPKQFTSYLTDMGMLVFEVMTKLDHFPIGRAQISGIAQLSLAH
PVSGFFTIVSPTSEKLGELQVSVQLEPLPETYDSSSSAPNTDISFDHASESYGKALTGHPTTMNDPPQPI
ILSLASADKRESESSSRVTTPRGRDHLYFQENADPGKDSYRGTQDHVTVGWSNVTEGVQSIKPIYSEGTT
AGKDALTVNSGPATKDLLSALLDQGSKLRDAMVVSALKSSPDLDHNPDIKLPLALNNYSLSQARATSEIP
SPTLMRNLLNSRHSPQTRDILLQPADSFIPDMEAPSDAKAIELLLGSSVLSPGHYWDGTGSPPESISGSD
FYNESELNDPLYDQSLLEKLFYKAPKSDSSASDFMSDDENTRSQNKKNKIALDRGRHRDNSPSEYKEDAK
QTKGNDSLRDSKTSEKSTSKCEGISLSMDQAALLGQIHVAHVVVESLRVPLDGTAVTPSKTNSRGRPPRP
VRPAKQTFFVEFQFPVLSKSRSGEVNSATEITRLVSSKVVNGSIKFQQRFTFPVLFSGQMIKHWWNTDLT
FRIFLRKGTQNKPGPVGSATLPLRDVLQSPGLSVTCSLPVSCTAEDSHTAAGPLKISVALAGDNKNIHDI
SEKTLEPENQAPVLPAVTSKAELENSASYTDLRPVAEKSSPLRQSLPPHIGNGGPKVSFSQNPQQTAEED
GLLLHIVLMVPEGKGLVAAGGDSSGICNSYLNCKLFSAQEATRSSVVWGSTQPQYNFSQVAPLTLNARLL
ERMKNNVMIIEIWNRVASPGQDQLLGLAKLPLHQFYMSFSDPKITRLLLQAQYPVVAVDSYVPIIDVFSG
CDRGKLKVLLAIGSGDQVVALQRLKNEEGTSQTAMPRPAHFLDPPLSSSQMGRPQEGMTDHIFEIHVENV
KGLTPLQSTVWGEADCFVQYYFPAHGPDSHTAIDLPEIAMTLKPVRTATTLCVPDPVFNDRQSHTIVAQS
DTPVQRLLLGAYSMQGLSGGGGVPFEIWCRYYYPNVRDQMVAKGVLPLSRLCAMVTMQHREDVGIQAFSL
PLIPRSEKSAELPPQPSGLLNVNVTYRRSMRNPVGMLATRMASISVQIHRASGLQAAARLVAQQDASFQY
SADVGVNAYVTIHPAFLPDVELRNTRTVARTFCPEFDHHYEFPCNMVIQRNNGEACSLAEVLYFSEIVLS
IHHQNVASVGSTRPQPVRDYHLGMVRIPCRQLITKRSGVSGWYPVTVPEDSKLPTDSTILHSVVGGLELS
VHFAHHSDRDRVLEVARGLGWNEYNEDFQEAIATEADEWHKREDLVNLSVNIPKIWLPLHCLLLAGHKHI
HKSTYCYLRYKFYDREAVCSPLRRPRLSEDGQQATIMFELSENRELIKHQPLVWYLREERMEIQVWRSYG
KDTNGPRPQDTDRLIGCAYVDLKALSENTSRTLAVSGVYPLFKRNVSSLWGAAVRVHLALSSAYHPSNSS
RRLSCAGERSQSEGEEWAPTSGDSFEEKQDDSAKNDKPEAKTEVPLLDAKEQPVGEVDLKNTFAASIVVE
RAMHLSLKGTPLTERAAATPTCCVSYPVAGCSEPVTTPVIANTDSPLWNFQHQARLHKELLLDPQQRLVF
KVWHKTDVERVVGFASVDLSPLLSGFQSICGWYNIVDFVGQCQGQVKVSITPLEGVAHLKAKVTSQRSSS
YQSRPAFCSSFSYNPSQSEVPAFIPHIPTLHHQLSDRENSGPLFPFLRHEEHMENVRRFHESLQQAERNA
HTVEGLDSLSQSSRSSLLSALRKNLGELDEIQKYFNQKLYRSISNAETSRCASVQIPQVQPPNSEPAEED
SDAKMLLQKSSFLVSQVSNLITGLQGIPKFAPAFSSTEQRLDVQGQTSVQHQQVDNMEPMAPERSEAYER
EGQRSDTPPFSPLGSLNVSGEKLMEFIGTDMDEKERHLFAEKHQEEQDKHIIHGSSDEEYEEDVIEPRTL
NEITTMTDRTSPWSSILSERDSDSMDHPQDQPVNPLAAENNRVITDFFSSFHQNDPSSVLSSAGSTDSEV
LAEGSRVRKISSSSGSEAETVGVQLSVDPAEQGALGEAESEAESQEMDGDPEANRTEEEQQDPVTVFSAL
SSGSDESEHITYGASEPDCSPPQDEPETDYPCSEPLEGNMHHIDEEEQPAGSESPQVPPSNLLSDPVIVP
NFFLPPQHLEASMRHLSLSAVREGRSDTPGIPFRRSKRQKPRLAPADLPKEETNRIARIFAAQFPGPPTP

P

>A0JM29_XENTR
MKESCRICGRELCGNQRRWIFHTASKLNLQVLLCHVLGREVPRDGRAEFACSKCAFMLERMCRFDTVIAR
IEALSIERLHKLISEKERLKHCLAGLYRKHNSEEDGPDTGPDTGPDSRPAAEGGTVDISELPDVRYSALL
QEDFAYSGYEYWAELDQQSQEPQHCQHTDTGGVRPRRCRGCSGLRVADSDYEAVCKVPRKVARSPSCGPS
TKGSASFCTEETMVTQPPPQPLPEAKAPPEGDGLRLSSGSSAESLATHMDAAATPSHKEEEPCKPPGDDA
KCDYCSINHVAHSPVNGSRSEFAFLLAKVFNYKPVQIPRGCRIPVKSRLHDFKPNIMVASGLSATPSPGL
GFLGIIAESPPSNAQDFVPELYDVQDLWQDVYEEYIPLHTQNLLEKQHQNVSQYEALSAQHVSDLQTLQG
RLQETEASNKMLQESLHQVTAELNSARELSCNQERIIQSLRETLQSRDSEVADLYHIVEGHNDTIVKLQN
MLQKSQTEQLQASQVTPSQQQLQLLDLQNTLFCTQRELQKQQRTLRQKERQLTDLEQSQRLLEADLLEGQ
QQKETTWKHNQELHGVLHKLQTELQEKSQLLQNSEEEKCTKLRAQENCIQKLQQTLAQKEQLQQEYMDLL
KYQQSLGKHPGGSEHMLDKLRQRIKDRDAALEQAVDDKFCALEEKEKEIQQLKMIIREKQRDLERLQSVL
SGNEETINSLDNLMKSKDLELEHISAAYKNLEWLKQEMEEKNQRSLKERDSIIQQLQQALQDRSKEIQDM
MATFLQKSEMGSFDLIQELQACLECKEKMLQEALHNRSQQADEHMRELEELLTSMASEKMGQGFVCKSCA
LKEKQNSEMDQSPACQKTVKQLAKAKPLSDHPISVCVTSLSADLKDPVAQSNQTEALESELAKAKDDLQL
VLRKERENQIEVSALQSVIREQSEQLQEQAADMDALNRSIQIKEDLIKDLQMQLVDPEEVPTVEHLTQEV
LSLKEKLAISGVPEPEHGIDHYQKLSKLLEELVADRSRLNDALHAEKQLYSSLVQFHSDPDSIKRTSALQ
EELLTAQTLTCQLEDTLEMATERLRQLDSERGLTAITFGGGEWGSLSVPHMHTHAF

>A0JM54_XENTR
MHWCLCINVLILFSCCINGAIRSDSQTVVGRVGESTVLGCSLLHQDAGRPPLYVIEWVRFGFMLPIFIKF
GLYSPRVDPQYLGRTRIEEGASLHIESLRSEDQGWYECRVLFLDRHHGEEDFQNGTWVHLTVNSPPSFRE
TPPTYVEVRVGDTLTLTCVAYGNPQPVVTWKRDGVTLESGDKVQASNGSLSIVGVERGNAGVYTCHAFSD
EGEVTHTSRVLVQGPPIIVVPPENTTVNVSQDAFLTCQAEAYPANLTYTWFQGSNNVFLLNRLQPRVRIL
VDGSFLIQRVTPEDAGKYTCIPSNGMWKSPSASAYLTVLHPAYVTSMPAETYLPIGMRGVIKCPVRANPP
LLLVNWTKDGHALELDKYPGWYVDEDGSLVIATGNDDALGIYTCTPYNSYGTGGVSLPTEVILKEPPSFI
DVPSEEYFQDVGRELVIPCSAVGDPPPLIRWSKLGLPGKSAAQMDANSSLVFRPLTKEEHGIWECSATNH
VATISARTAIYVLGTSPHAVTNISALSLVSSVNVSWEPGFDGGYFQRFSVWYAPLVKRLTRGHHDWISLS
AAVGATHLLVGNLQADTAYQFSILSQNKLGTGPFSEIVTATPLNFPVTTVPPPVPTSEPSDSLSPPRYLS
ANETSEGVFLQWLPPLRSSLPLRGYGLEFRRDEGAWELLDESIPESQTELVVRKLVKDTMYEFRLVAFAG
DYISEPSNSVNVSTEGMKVYPSKTLNPEVQRKPLLAGVVGGACFLSLTIILSTVVACMMNRRRARRRLKR
RQQDTPMVFSQPKKASPSQNSNGSNSPDSLMRVKLHASPYNSLRRSLQVADRPGNSVGITAGGKYTIYES
HIGDALPLERISRGPDGRFVVETEMSSGNLRGFPYVTETDLYPEFNARDSSDLSQCSQVKTYLQACQLPE
GGAVSWRDDVKMRPQATGQARKEARDSGYRRGRYFGCSSSPLEEAIPFRISNISPVTSSVPLPYANIEEA
RCRDSAQAEGDLERTIYTLKGSEHDGYSALSGVGREPSSLLQKPLGGNEAESRLRHSLSTQSGILQYLSL
PFFKEMSVDGEWPAEEDKHQTKSSEHNSLGAAEQDPPSAVALEDRTLMDVEGRSGVRNLETPCVPHTLLP
LDYVTSKQELSVPGKSFLKPPEPNLGPAKAVLPGSFMWAHPPENSERLKVGQRAPWGAGEVPLDSRAEGH
LPSDCECPLKDQPLSVFTDFQALDKQRAPLDKSLFLHGSEKVMRSSLTSQSSGRGSVSFLRPPSLAQSVG
GSYLNSPLGETSSWHSGGGSQGSSADDYRHRKDSLLATISNRRNTSVDENYEWDSEFTLEADLLDALQVY
RSTNGGRPISTVEEGEVKRQSVKIPSEGPGSPAGSGSMDALDGRYPQPLSSPEERCAALKEEFLEYQRRT
RAAQKVHTKSKDFEECYEQATLL

>UIMC1_XENTR

MGCRGQNCSEKRRAIRDHFIVISDSEGEEEKEVIRFGKNIAQPDGRRTSVKRKIAQMTEEEQLALAVRMS
EQEANHVNYSQEEEDELLRKAIEESLHSCTVSEPCNTTTQQNTNTLHSTNRTLAAEDCSEKHPISQNCSL
SEPPNNITVQQVYKETLSTNEPVKLQEHAEEKSFSQVSALFELPSGAAEQQMNKEAHSTERPVVTGDVVE
EGHLSQTFTVSETPLSATQEMNTEDFTTSATFVTEEVGEALTQYSTVSSQSRHKSPVVLLMRLSQDIVES
SSVILSPKCRDPFSDMESGMASSCPSNSSNFVSMLPHKALTLSPVFPKQLPRRLGLSPRKLFQGASLTSD
SKEQEVDDQCSHCSESSELDYSVLPNSLQAEPFKSNTATEVLKENNTLLNGVTTEEQTGASNSCLDSQRQ
SGSTVHYYWGVPFCPKGEDPNLYTQVILCQLEVYEKSLKKAQRQLLRKMNFGEPVQLSAPPLRRTERGKA
DSQDSLSQESEDLKDEDSPRPVDDEEVDSEKPMNQCVSSSSRQPAESLEEESPIPAQDEQDNNSQLILFL
NMNNSHSTSLPL

>CA174_XENTR
MRKRKLSDGVRCSARQKNRSCSGAQSSTDHEADTYGPKKKAASSNNTEKESSKKLRKDEKGPVEADENEL
LNKIDNAASNESSNVNDSQQSEKSITNTKDNGTRCSKLRANTRLPSSPVSDLNEVSCNGLTDDSGDGTGF
IHKTCSEPSKLREIYLNGSPFVDEDSNQPMPLGLFFENADLMQDLPPAVPSCASMSRRELRNLHFRAKEE
DEDDDDYVDGLANEGNI

>TDRD5_XENTR
MEQERIMQRIQKDVRALLIASKNGLSIHELEQDYRMMIGSHIPIRSLGYKSTMELLLDMPSVVKIHTQLD
GTVKLSAVVDETTRRIADLVSCQKDRCTARTRNRRRNIRPRCPVDLVRRGRVSPVLPATVKSDLRDLLSV
SPLLLSELEKAFSSRFGRSFQYTRYGFYSMLEVLRSITDIVEVKQTRAGSLLVLRNSETGRISASLCVKQ
SQLAIDPKKTSCVLEQCLKEPRTPEKKTSETVTYLKVSPPEPQKLSSKESPSVTRSTSPLINSSNKEQRL
KLNVLLDESLKSNENKAPPVTNFTSAVPSHNSVLSNSLVGNSVSSETLFTKMSRPTSHVEADHSAASEPD
ASVLEWLEKKLEKELKLCLARKGAGGSVSDALRMDIQHVVNQHSDGLKISQLPTVFKSFTGKDLPFKELG
FMSVMELVGSLGDILCVESTVDGKDWKLFDAKKKDLVDEFSAGLPSTSSILSSWNSSQQTTTPVKPVGTI
FSKVDEKLWWGPLELKLCSTEQIDIPPDAVRNQKLHCLPSMKRGFMIGVYVENIESPSQFYVRCCGKDTS
EKLEDMMIEMRHCYSNECVSERYIVTDNCISVGQIYALRVPGDVWWYRVIVHDIKNSELLDVFYPDFGNV
ATVKKSWLRFLKNCYMKIPAQAVPSSLPYVTSTEDQWSAQAIKKFQQLCSCIPLVGMVLQYVQDILHIFL
CDTSSEEDLYLHQLLISQGLAKMEPEHACKKISKKRNPFMQYLTPSQEQPQEESSQLSVPSESSQSEIPC
AKEVKDEDDLGMPYLEAFPSGSDVWDENWAFSDGACDYDTAPTIPKVDTQKQQENKVSQETQPFKFCIDS
ADASVVHHPLEEFYISLIKSRKSQESTDIQQSPPTEEQHVAEISHRSATEQLQGGSSSSMLCEKKLYYEK
EDLPYCQQSKCSFSPLLGFQRLQIPRSATPVALGPAARLATAGRLLYWASEPR

>A0JMB8_DANRE
MSKQGDRQSVFQTTKVRTALKGDGSWIQRQEPENTDVEKPWLAEVRANRSSSVFEETSPVSSPTTKSPQP
KTDTEKSKTNTSGYIIRGVYTKTDSKPPSTSFSNGFVGVNNLAKKPSESYKKIAPHTIRSSNGKTAQSEP
TLSTEEMDKRMEAASSVLRGSSGNRRSYVMSATKKYESTDKPNNSDDISASFVAKRVVISDDDDTPATVK
SVPQQSVKSSNEESPVKKPVVKEVNPAAPETKPAISTESKTTETKPTTDSKPAVENSTQPKTAPEAKSAS
QPKPASETTKTPDSKSAPTVTEPKPLAETKPTTQLAATSDPKLAPENKPTTQITTVTEPKPLAETKPATQ
LAAASDPKLAPENKPTTQITTVTEPKPLAETKPATQLAAASDPKKAPETKPTTQTTTVTEPKSTSQPKPQ
TETKPTIQLISASDPKLVPETKLTTQTTTVTETKSTSQPKPLAETKPTIQITAASEPKSATETKPTTQTT
TATEPKLTSETKTVVETKPTTQVTKTTEIKPIMKTREASDSKPTLIPTVVVTKPTETTEVKPTTQTKTAT
DSKLVEETKTTIVTASDPKPTSEPKAVETKPATTAEPKPTAQAKTAAESKLMEEIKASIKTTTASDAKST
SEPKPVEIKPTTTADPKSSTETKPTAQTKTATESKLLEEIKTTIKLSTESDSKPTSEPKPVETKPSTTTE
SKSTTEPKPTAQAKTATESKLLEEIKTTIKMSTESDPKPTPEPKTVETKPTTTAEPKSATETKPTTQTKT

ATDSKLVEEIKTTIKIPTASDPKPLEIKTAPQAVETKPKTAEAKPAVEIKTSTDPKPTSEPKPVTETKTT
PEPKSGTQLKPEPKPEPVPDKSAESLIALSDTLISFNTEPARSPRKADSGKDLLSQDLLTGNGFLPETKT
SLDFLSDDLIPFNTSKTSSVDSFDPILTMGEPTKSSQYSPHHLLDSRSTWRTEMESTKTSTSTEERDRKL
PESESKKNFVYVKEYVDNSAVDGSSSDYVSSTTSSYNYSSPSYYTRSEMTACTYCGEKVGSDAKITIEHL
NISCHPSCFKCAICSKPMGDLLYNMFLHRGTVHCESCYSNLL

>A0JMC6_DANRE
MHEHGGFRCEPSPCRGFPQKSSIRFFCEPGYTLPKRYQSSKCHHGVWTPKVPVCMPRPDGHGEQQEKIIS
SIPSVATTAIGVSIFLLTTTACLVIKSRLFSCRSHRRSSDQLDLVVDGLPVSLPTYEEAIYGSWGQRIPP
FRGPTQLLLAQDPSEHSPLTSLIRSDTNRCNDTANQNTETPPPYEEVQSRSRDSVNDSDGQAMQSALPAD
KNN

>A0JMC9_DANRE
MYQFPVKGLEKIRRTRRKVKNILGEFGLEACLNLGQELQEYYPGDNIFDATDWCNLSDGFDGRWRKKWGY
RTQGLCCTLCNFSTRSWHTYKTHVQRYHDEEEHMCKLAACASCPFIAHPRLVCKHLKLFHNEDTKSETSA
APQLPPRAVNGTTFQCRRCHIQDTLLYSVKKHVLLYHYTSTLNKYAGQRTEQELVALGDRSQKFYCKKCY
ISAESSEHLLYHLLTSDKHKELDVHIRSLIFETDNKKQFPALAPKAQGPSPVMMMKDQPAATATLAAGGI
TGVENGGSAVIATPGTSQAFLPTQASALVQLASAEAKGLLRPGVPMGFQNTQMPRPALPPPPNSIPPNQM
PVRVGLPNQAQLQPVSRQIVLPPGVRLNVPGVRPPTPQSFSANPRLPLNQPPSGGTMLTSQSLLSHLIPT
GNKVDGLPTYTLAPLQVLSVQANNSQGVSKPQLPPSQNNPAAQQNKQSSGLSTPKQTKKWITCPICNELF
PSDIYESHPEVHKEAAKLPKVGLAARAPFLKKMPDKTVKCLTCKILISEKGVFEHLLHGMNCLFCSGLFY
SIKQLVDHIQIEHNVSRKSNCDFMRREYRLYTDDSGYLLFPYFDIRTTAPKLIMGEKELNLALVTSSLDL
IFVKMLPNNPQGVNQQYTPCKMPTPKPDSTECPFCSEKLLNKECYHMHLKEKHFIVPTLHAILKTPSYRC
LYCGGVYTGKTTTKAIIVHLAKCRSAPKSVRESDKLTLGLAVTPKANRAHGSYPAHPRQITGPTPTSAQA
SVPSTQPPESEAEMQSKMRLEIAFREAMEANRKEREERLARKRKLERDRLAGLTLPSPDIVVDPSVKLAL
DPAGMELRTFEDRREFVNKYFNTQPYPLKKEIIALSARLLLNKTDVACQISSKRTRCMKNIQKMKAVVLL
GFNMTEVMKLKHDLLIPEIEPEKVATMADTDMEVDQE

>K0664_DANRE
MVSKTDDIPASVPNCSPADFARDGETANSKGTTSKKEASCACGHGVETAVMNGDAGHDQAEEADSKQDGS
GDADQAEDANEQEVIVIQDTGFTVKIQAPGTEPFDLQVSPQEMVQEIHQVLMDREDTCHRTCFSLQLDGN
VLDNFAELKSIEGLQEGSLLKVVEEPYTVREARIHVRHIRDLLKSLDPSDAYNGVDCNSLSFLSVFSEGD
LGDTGKRKKKGSELEQIDCTPPEHILPGSKERPLVPLQPQNKDWKPMQCLKVLTMSSWNPPPGNRKMHGD
LMYLYIVTVEDRHVSITASTRGFYLNQSTTYNFSPKPANPSFLSHSLVELLSQISAAFKKNFTTLQKKRV
QRHPFERIATPFQVYSWTAPQIDHAMDCVRAEDAYTSRLGYEEHIPGQTRDWNEELQTTRELSRKNLPER
LLRERAIFKVHSDFAAAATRGAMAVIDGNVMAINPGEETRMQMFIWNNIFFSLGFDVRDHYRELGGDSAA
HAAPTNDLNGVRAYSAVDVEGLYTLGTVVVDYRGYRVTAQSIIPGILEREQEQSVIYGSIDFGKTVVSHP
KYLELLEKTSRPLKVQRHAVLNEKDSAVELCSSVECKGIIGNDGRHYILDLLRTFPPDLNFLPVEGEELT
PESQKLGFPRQHRHRLACLRQELIEAFVEHRYLLFMKMAALQLMQQKANKDKTAALQDSNAAGAGSENKP
LALESCDGTPDSPTSSESTLTPEDSEATTVSENSSAENQEAMTEVPVASINGTHEPLAAERQNGGCDGPL
EGKEADENIPGLAQAKELAESLAAEDGSGIDPKSREVVLNACKAVGSISNTSFDIRFNPDIFSPGVRFPD
DSNDDIKKQKQLLKDAAAFLVSCQIPSLVKDCLDHSSLPMDGATLTEALHQRGINVRYLGTVLEFMDNMP
AKAQLEHIYRIGISELITRCAKHIFKTYLQGVDLSALSAAVSYFLNCLLSSFPDAVAHLPADELVSRKKS
RKRRNRVPGGGDNTAWASLTPSELWKNITSEAHGYYNFSLQCESVDQAVEKYGLQKITLLREISIKTGIQ

ILIKEYNFDSRHKPAFTEEDILNIFPVVKHVNPKASDAFHFFQSGQAKVQQGFLKEGCELINEALNLFNN
VYGAMHVEICACLRLLARLNYIMGDHPEALSNQQKAVLMSERVLGIEHPNTIQEYMHLALYCFANGQLST
ALKLLYRARYLMLVVCGEDHPEMALLDSNIGLVLHGVMEYDLSLRFLENALAINTKYHGPRSLKVALSHH
LVARVYESKAEFRSALQHEKEGYTIYKNQVGEAHEKTKESSEYLKYLTQQAVALQRTMNEIYKNGSNASI
MPLKFTAPSMASVLEQLNIINGIIFIPLSQKDLENLKAEVQRRQLMQDSGKIQEQQGSHLELDDKLPVDD

>LST2_DANRE
MMNRFRKWLYKPKRSDPQLLAQFYYADEELNQVATELDSLDGRKDPQRCTLLVNQFRSCQDNVLNIINQI
MDECIPEERANRDFCVKFPEEIRHDNLAGQLWFGAECLAAGSIIMNREIESMAMRPLAKDLTRSLEEVRN
ITRDQALRDLNHYTERIKEALRHFDGLFAEFELSYVSAMVPVKSPKEYYIQQEVIVLFCETVERALKLEY
LTQDMIDDYEPALMFTIPRLAIVCGLVIYSEGPLNLDRKPEDMSELFRPFRTLLRKIRDLLQTLTEEELM
TLERSLCISQDGEFPTSSTNDPSASTGPDSQTEELEKEKGVEEVVDLTLFVTQEDSVWKEEEEKQVLPES
SSESEEEEPIDADLACSMQYDEEEIEQLNMMVHQVGDEMSTLLSPPSQNQSPAHRPRPYNGSSLEGSSAT
SSTQASPRRAPGSYHDDDRVFFMDDLESGLSSELCRGQLPLPTVCLRSPEGSSCNGWLTVCQSSDATNLG
CQRKLSQSTESVGNSDRMVNGWEGLQDEDSVQTAEEIANRTGGMKLSATVIFNPHSPSLSDLAVVLPQSA
DAPEGGEGGALVATQCLLNSCVCCAGGCVDNHEDAMEPAGRSMALGFEKHKLTITSSVIQSAVAAGSPGK
GNGHLPLTLPPSQGHLTHSVPNCSVQNQAREDEGSQDGIHYPCCEKCSPGVLLAQDRGSGHEGGPSCTLQ
DTGCQTQHNASVKGRSECFGKQSKDDNRKINSSSQESPLSSVPSSDIDGVSVTTCSLSSSYAPSPVSSLT
TSSDMSEDLDHQEIQVALQAAKLAAHNKIRSRFHSSSDLIHRLFVCISGVADQLQTNYASDLRSILKTLF
EVMATKTDQGDNEKPKKGPCLGSAVLEDCALCQETISSSELAAKAREGQFEDPPEWVPDEACNSCIACKA
PFTVIRRKHHCRSCGKIFCSRCSSHSAPLPRYGQMKPVRVCTHCYMFHVTPFYSDRTGI

>A0JMD3_DANRE
MWLFCAALLALGSVDSVIGDSKAVTTTLTTKWPSTPLLLEASEFLAEESQDKFWVFVEANQNIENDHDDT
DQAYYDLILKRAGELLSPVQLNLLKFSLSLRAYSSTIHSFQQIASNEPPPSGCKAFFNVHGQKSCDSERL
QGMLDNALERPKPNLFKGDHRYHSANPDAPVVILYAEMGTKEFSRLHQLMLSKANKGMITYVLRHFLASP
SKSKVHLSGYGVELAIKNQEYKAKDDTQVQAGADANATVIGENDPVDEVQGFLFGKLKTIYPELKEQLKE
LRKHLIESTNEMAPLKVWQMQDLSFQTAARILAAPSVDALNVMKDLSQNFPTKARSITKTVVNSEIRKEI
EENQKYFKGTLGLQPGDSALFINGLHIDLDVQDIFSVFDVLRNEARVMEGLRSLLIETPYIHDILKLNVQ
PSDSDYAVDIRNPAVHWINNLETDGRYASWPSNVQELLRPTFPGVIRQIRKNFHNLVMILDPTHENTAEL
LGVAEMFYSNNIPLRIGVVFVVNDSDDVDGMQDPGVALLRAFNYIADDVDGQMAFDAVISIMNRIPSGDK
LKVEHVVGVLEKRYPYVEISSILGPDSAYDNNRKEGKAYYEQTGVGPLPVVLYNGMPLQREQLDPDELET
VVMHKILETTSFFQRTVYLGELNSDHDVVDYIMNQPNVVPRINSRILSTSRNYLDLSATNNHFIDEYARF
LFLDAKDKNAAVANSMNYMTKKDDGIIRPVTFWVVGDFDQPSGRQLLYDAIRHMKTSNNVRLGLINNPSE
NPSNENSLIARAIWAAMQTQTSNNAKNFITKMAKEETAQALYGGSDIAEFAVGGMDVPLFKSAYESPNVN
FLLAHSAYCRDVLKLQKGQRAVISNGRIIGPLEEREVFNQDDFLLLESIILKTSGERIKGKIQQMGMVED
RASDLVMKVDALLSSQPKGEARIEHTFAEDRYSAVKIRPKEEEVYFDVVAVLDPVTRDAQKLAPLLLVLK
QLVDVNLRVFMNCQSKLSDLPLKSFYRYVLEPEIVFLTDSSFAPGPMAKFLDMPQSPLFTLNLNTPESWM
VESVHTRYDLDNIYLEEVDSVVAAEYELEYLLLEGHCFDVTTGQPPRGLQFTLGTASDPVIVDTIVMANL
GYFQLKANPGAWMLRLRKGRSDDIYKIYSHDGTDSPAEADDLIVVLNNFKSKIIKVKVQKKPDMINEELL
SDGTHENESGFWTSIARGFTGGSNPEEPKQEKDDVINIFSVASGHLYERFLRIMMLSVLKHTKTPVKFWF
LKNYLSPTFKEFIPYMAEKYGFQYELVQYKWPRWLHQQTEKQRIIWGYKILFLDVLFPLAVDKFLFVDAD
QIVRTDLKELRDFDLEGAPYGYTPFCESRREMDGHRFWKSGYWASHLAGRKYHISALYVVDLKKFRKIAA
GDRLRGQYQGLSQDPNSLSNLDQDLPNNMIHQVPIKSLPQEWLWCETWCDDASKKKAKTIDLCNNPQTKE

PKLQAAVRIVGEWSDYDQEIKRIYNNFLDEKERGTLEGYQSTDRKHPGGDTHSEL

>A0JMD5_DANRE
MTAPKTQKPKGPNKKKIIITVLMVVVVLAILAVAAFLIKQLIDSKYFFCTKSWKFIPLEKVCDGKNDCSE
AEDESACVTMFKPNTTFPLRLYSANNVLQVLSPSDNTWKSVCSESFTQQHAETACQLLGYSVSPVFSSIA
VGPLPSDLKISFCMVGTTKPQTFQSAVSDRKVCSTGTVISLSCSADCGLSRNQDRIVGGKDADIANWPWQ
VSLQYSGQHTCGGSLVTPNWVVTAAHCFNGDGRKALSRWTVVSGITYLSSTPSSYVKEIIVNSNYKPAES
DFDITMIKLQSPITVSESRRPVCLPPQNLGLKGGDGLVVTGWGHMAEKGGSLSSMLQKAQIQVIDSAQCS
SPTVYGSSITPRMICAGVMAGGVDACQGDSGGPLVHLADRWVLVGVVSWGVGCARPGFPGVYTNVDQMLD
WAHSVMQTYK

>A0JMD6_DANRE
MDPVVRRKIPACVRSLLTDITPKQEENLSDWPETHSESITSKDGIALPKRGTSETLLTPADSTEGVTTDG
NPSSIKPCGLCLIKPSSYTCPRCNIPYCGLACYKSQSHSKCSEEFYKESVLQELKSRGATDEEGKSKMQE
ILLRLRQSADTEGGMQNFLRHLDGTNVTEGDAQALDLLCKLAELQSGGDENSHEAQEILAKLEEADDDDD
DEDLAEKLAGLDVDSLSEEQLWSLLSTQEKEKFENLLKVGSIAGMVVLWRPWWEQHEKETKTLIEELRFE
SQEEVRTAKKGKPQKVASDAKQSAVPPISAKIPPLHRLTSNPSPLVQFNLINALYGYTFSLCLFNGDISD
TLLEFSQALVSISEFLGAGRVFNSVPEALDAGIRSVSAGGYFDREDSSAPIRAVEAVAHVLTGRSREDAE
GYTLSALSQVRSALSKAKMAAAKDDDERMRQGFFQAGKKCEFFQSWVKENPEVLRRLAGSVWMDFERREV
ERETLEEDRKLLDEARGKSRGKGALIEEVE

>A0JMD7_DANRE
MTHVRQHGGPSANFTKMTSDMEPLKAVNKFPDNVTEIVQYVPASDTKKVQKRKVTIRGIVTLGLPTALAI
IALITGLLVWHFHFRYNGKVRKMFSGHLTISSQTFSDAYENNNSTEYKELALKVSKQLKSIYGQVHVLSR
YHVASSVQGFSEGNNNGIIAYYLSEFNVRESKVSALEEAIASMDDTENTRKSRRGFSRMTDSLIIDGITS
GAVDARLADRNLKRSSKRSNHAHANQTDIIRSPGFPDNSYSPNLYTEWQIRADPEHRVRLEFDTLDLEKD
CHNDFIKVYDSLAPSEKLVITEKCGYRLPNEKPPKTVYTVKSNQLTVKFNSDRSYVSEGFSAEFEAFEPT
NPCPGRFECDNDLCISSDQHCDGYNDCGDMSDERGCMCNETQIQCKNGFCKPSFWGCDGVNDCGDNTDEE
NCGNCKTWEFRCRSGRCISAQKQCNGYNDCGDGSDESRCAKSIAVHCSDSTYKCKNKQCISKLNPMCDGE
TDCVDGSDEAECKCGKKPPKSTRIIGGKDSDEGEWPWQVSLHMKTQGHVCGASVISNSWLVTAAHCVQDN
DQFRYSQADQWEVYLGLHNQGETSKSTQRSVLRIIPHPQYDHSSYDNDIALMELDNAVTLNQNIWPICLP
DPTHYFPAGKSVWITGWGKLREGSDAVPSVLQKAEVRIINSTVCSKLMDDGITPHMICAGVLSGGVDACQ
GDSGGPMSSIEGNGRMFLAGVVGWGDGCGRRNRPGVYTRVTDYRSWIREITGI

>A0JMD8_DANRE
MFLKTVPPAVAIHSEWRSSKSCSLPLYFNDSASDSDLASVSGTPFPQKIQMIIESLHSTQSSGMSENEQA
EKAAHSSHEAGYKGQMRLMDMSARTRRSGSDTKLQNNRSDTGDDSDSDDSVDRGIEEAIQEYLKEKVDHK
RKGDPVTSSPPAPKLQRREPDAPKQQTHSSSAKVLTASNHIQRLSSGMVALKKKVKKKQLSKENPFKKAD
VSKVSPLKSLPPPRAKKGSSSSSEMDKSPPRLVIKEEEEWLDSSSDDGIEEEIQRFQQEKKEKQEGDRDA
QRSPQKIKDSDSSSDEGIEEAIRRFKEEKHKQKKNKSLLKPAQLVPAQRSKPAVASPERISTQPLKVLSK
KNNIKKLSTNKSTSLPTPLSVSHFLNKCTSQPAKAKTFTASEDPSSSKEPKKKLKEIQPNKGVRLSLSKK
RKFKEEQCKLSRDQDMVLNVKEEPPGTPLIHSDSTRLELSTTPTAISHSSSVKNSKLKQNSPPRKGADHV
TKDNVSSCSASSPKAAIGSERNDSSDKSSSLDSDEDLDAAIKDLLKTKKKVKKKVRDLKARKSDKPFEAS
SLDAIKKQKPLTEQKCIPLNKLVKSSILKGGKESLNIQAKNDKVPKSKPAKGKCEVQNFKPSKSLAQTDK

VTGNGGISSAPGGDTLSPSLHADDDSDVDSDDSIEREIRRFLAERAKVSAPVTTNIKSEEKGINSSSAET
EENPDLQKTRTGTPVSAATSTSGRLWKTELTGTSTKSTETPAELNKGPVLTPGSSCIMGFSRTESQKPEN
SAPRDPTNGSSQTGKDTPTGHVIKPNSFTPPCVNSETARPLGHQHQNLFLMRPVNNRMSESSSRNSNDIP
ISHQSRSAPRIPLREVITSLCPSPLSKTQINSPTVDLSTSVPPLSRTEGVYMHNRLKRDRHFSDRPHISF
STSHLCQPSPELQSRQGVSVIQVQRDQAVLFQATNRLQVSQTEATASLGETQREGGSVSKEDEEEKCIDE
TNVESDEERKDQKTKDTSKQTNNQ

>A0JME7_DANRE
MSGGKKRSGFQITSVTSDYNQSSAESHPVELLSPGTQRATPPSARNPAHGRGSESQTTSPTQFISNGLSL
RQSPALQTQQSCSQPTTPVTARKQNSLDAGGSRFRVVRLDQGPGEPYRRGRWTCVDVMEREVEERGLRRV
IDSMRHAHSLESLETVGLGGAEGAVGGARLKPLAVHPGHMVHSQGTTHLLSQCRTEYAHSGPPSPTYNHT
LYDTQPITGDVVQLEKSPEEGKGRRKCVQY

>CTC1_DANRE
MEEFLNNFKSYSRADQRWLSAVFEAVQQQFSPLLDSDGFSVARLAQSVVEKVQRAVGSDCADLPVSYRIV
SVSELIRDQRTACCSSLCWSSAHYRDQAKKAEQTLPNYKALPRDNLLLIGFLCDGRADGKSEGEWRVRDA
GGSVPCMMLKSSCLWLGRLMLFPTWNYIPQNAPGAAGYLELVDPPVCVTLEAMTFDPGGALTEVMSVRHA
AELLAHSCEDQVCVCVGGQVCVVGPLLLIRGKRFFCLVLSDGGSSVALIIRELKHQYWRQCVCVGQSVWI
SSLRVCSLGALEGRRVLTDSCQSRLHPHTPLQESSEEQDTEDDTHTPADTTHTLADATRTPADDTRLASD
PAVRVKPSTIISFKGTVTKILNAEAGLYELDGQVGLCLAYQPTQKWGGGLRPGAEIQLHNVHFTYQASAF
APRAVLWACLRSSVQITAFSRLCSELQVTEPHGALQRLLLERNLGPSQYLWLCYCQKAIAERLSPRWVRP
DRVCVVAGRLLDAVCDAEQKTKKKRNIYTEMIQEPHHCPISMYCVRWPSAALWSVQQLCVWMQREVWSSL
SLPALLSASAPHMTALELNSALRWSSVELRLEEASPDPLLLLGVLELQAESATLQLRDHTHKLHCLCVCR
DHSPNISTAWQGCLVCVRRCTLVMERFMKTNFPSWKHLDQPSYITLKHCRVYLQLCMDDLIIISPSSSMS
HQMAESKLTHGHTHRSCGESSKRLRSADAGVCERADSSAVCVSLLFRLENKHGVTLQNVSADTHTRQLGF
KCRAVCVGGVQRWSHDPKNCRIQERERDGGGQTVELHFRGSCVRWFPVLHPGCVYRLIALNTEDVGVLEA
KGVSARGGVTQLSSPSLLLQPQWRVHTLPEEPADAQAAAPALMSVSEVLHSSSAPEVVSFHGIISQRITL
QEDNEAQPKIKSVSPTADTQEDLRVRLTVQDAECPGLMVHVYLDLSCGLYTLGLITGAALQVHHVQRKVS
RACNVYCRSLPISYVSVTGLRSVCSGPAAAAPPPPMMLLGSWASVGSQQCLVAQVKGHVVCVLSLRLQWI
CSLCGSIFTQTTCGRTHPPCDSTSAVFQAEAKVVLEDGTGEAHVWFSSESVCGLLMLNAVQWEGLQRLVR
VKGHVKVYTRGRTMTCDDSPDDSIVQYLSCLCSSVSVCRQIHVTCRRRAQKTGKSQLRKVHRGQTEFICK
FPPALQLQCQHIHTR

>A0JMF2_DANRE
MGDPFTRTVQRRSRMRNFNWDAIPRHSVLGKRNVWTAHRNLENFELDTKRMEELFSHNEHHGLIRKGGTV
RKSVWGLSQIAAESENVSILNSKKSMNIGILLKQFKRTPKDIVEAVRNGNMCFASGKLRELNKLLPDDVE
TKKLMSFNGDLSALNDADRFMVMMVQVPGYKVRLKSLLLREEFFPFIEEIKHSIAVMTTAANELLACDDL
HSIIRLVLKAGNYMNAGGYAGSAIGFRMASLLKLVDTKANKPGMNLMHYVTMQAQQIDEALLHFTDQLQH
IGIAARIQKQEVEMDFQRELEKIKEAKIDASKQPDLLHQMEAFLRMADIRLMDVEASLQELEAISTSVAE
YFCEDAATFKLEECCSIFHSFCERFEKAAQENRDREAAETRKRQQREREILTKTAKRRSTGSCSTQDGNN
DNSTLESVLTSFLKQRPSRRRPGGPTSVANSLVKNIRPQVEKREDNENLDSPIDSNEEKFEIPRVEEPEN
ICQEELPPCVINDVQRARAASKRGQCIDDELIPESSDRKQDEGSYTSIKQKLEDGVVDKKEVEEVEDLNK
EEDKEKIEEADKMPDLSGKVFRFQDCGLNELSTPDRSRIVCTSTPRQRDIKEVDLALQTGLGGLGSPWTI
LSPRVSPRNTPHRRHSFSISRVDILDDGVWALPDTPVRDKPSFSHKSAASSSLPDCPSKRTPAQGMFPRS

ASLTDEKERAPNFKLGQIFQRRIGTEVLPDKRQEGSGLVSFFRRFGERNRAGTVG

>A0JMF5_DANRE
MTVVVLRLQGLNTEAGSEDIRRFFHGLHIPEGGVHIIGGEMGEAFIIFKTEREGQLAMRYSGKTIKGSSV
SLHISSVAELKRKMESRLKKPKPTELETNVVPSSPTDTNAALLLSLMGAIYGLHSNNKDNQQGQTQQTPG
NVNQNSPEMQTWPLTGKQMNTDAQHSLNHELASTEQQTGRGENLNLSKPGYLRLYGFPDCVAKQEIFQFL
RGLPVLEVITKVRLQLGWCCLVKLASFSDAEEGLKYSHRKFKEFNVEVRLAHEKMWTDAVEESKNCTQNL
KPHSSSEQNEANNNRNAVRAFTNKRIAEEQPSPGSPKKFCRNDPSPQKEYSVIVNNLSRNITKTEIKNIF
GCHAIQNYRIKHLLNKWGERTSTAFITFDNSEDQASAMKMNGMNVGLKNIEVLSITKEEILDILSKNRFV
KSWRPPPYGNTHLSMSCIYARNFPADVRKEEVKGFFMPYNIGDDAVRLLVDNQGNGIGEAIVEFRFQNIA
KQAQRLHGKYFMGAKILLTCITHQQMEKILGKR

>HAUS6_DANRE
MSKLKKTDGKYLWWCLLCLKFKPDNVSVTKTTKHLNLGMNMFDKPNKEAFYIVIHFLFNKLNPTRAQDVF
RNCWLVWDHKSDAEFRKVAFAWLQEIANEEGSAFPKVAASHLLSAFGPKFINLMLHLAKHVMLKTMKTFN
TDGMWVPEAAAVPASSEEMELKRFQLVKRQFQRVTVEQDFLIQDYQKRAKVLEKSLRDLDAENAKYDSLL
KEHDSITDLKEVILAKVQKLRGLWNEVDRFLSNHQGKRDIVASVINGQVDQYILCGQELNVKMPAALRER
MERPSHQSSAVRLYEGGQPVLVRLLALLNEGLHVLREEREKIVGPSVQLQNQDVQEHALLFTRSKENLNL
IRWKLVKEDAAEIKVSIGKLEEEWECKWANCLKNTPLTCFLEEDPVLDLVSPMVALSFEPASEASFQASV
ISQYPCKPHDLLEQPSKEKLDTTVAVVHLEQEVHLKPEIKISSVATEDQDVLVPSDSCFSPEHVTCGTPP
CETILSVIETPSPKQPCHTNLHSKPKASVLKTKAQILDLECDNLASQFADAVIMSPGNQRSDVDLGQLLN
AISDPFSSRKQLCRTPESLIKDVRSSWRKAVEEGLAEKKQASWNQQDSLSWLKTPMANMNSPCKTLNLES
SMSFSTNLDNCTSPPVQQGSTLHSTLSWDSSQMEVLTSQCSSDVIKFSIAQEEPPDLFDVSFNSDSSVEN
SGEKARDEELCLPSVGLCYLDETPQLTEQLCLGASSDKSVFKDSPVHLSMSKDLSHWTTDHFSLDLDRLE
SLSSPPRAENLTLPNLVNVNLDEY

>A0JMG0_DANRE
MGGLTEERVRVVAIIQALKAVGIRVKNWKNFLNGNSNCEQTLCQENASQRFAFGRDLHLLPQHDLPDNGG
TVPRFLVEACVFLSQHLNTEGLFRKTGSLTRIRALRADLEQGKPVFHPPHSSLLQPSDVASLIKQFLREL
LSPLIPTDLQIPLIQAQGLEMTHDQEGARNRTTLLITALFPSSNACALRYLCTFLRQVADRCSENRMDAS
SLAVVIAPNLLQCPTQPNKLTLDTEKHLDQQTSVIKSLILNADRIGVVPSCLLEPSKSTGGIGTPSPVGG
TMFSKRTGLSVYRSLRRQRRRSVGEIFVDAFAKLKPCRTPTEPAIVLGVASETLLLKSPTPQSPVTVKRK
AAEESLPELEGSARKRRSLHDLREDTTTTITQESVDSRSPLEDNCSNEDPTPTTTKKRSHTRDQRKSLKP
PGQEEKVNRRRKSLRFFTVSSGSNSSNLSVSPADKVLENGLEEQPNTKSGAPFTNAELSSNIPFILIDGP
EGVVVGSEVDDDPGLLNCSFAESPNDCPNTRSEVSCIQGQDSEVPCDDECWTVLESEKFEPLAECELIQP
VTQHEGFQVNAGTEVKNEPEVKQLEEQPRKESKKVKNSFKNRSRPRRSISLPEVTLESCTHALADLEKEV
VGAEPEMEDSVWPMFESLTLSNEHDNVTAEKKVGSKEAQETKGSVKTLKNYKQDKMADSTLGFKRPHQRL
SMAERLRGFSALTLLLRTSRAAPQFRDKPQESLQKAAARLKRQGARRFGRSISHDGVPEREAEQDPVGSP
PANQAYLGLCDASPDSGVESVDHEPINDLISQDICKGPQNSPKAQPTNEVDVDSLISFENPNQDLILSAS
DKTECEFQCKKTNQDVEELDLHKLLDQKCQIGGQEEDDCHDSDVLTEVLSPDIASPMFLQEMVPLNLFGD
VDDLRRSDLSSA

>A0JMG4_DANRE
MPKKKKNGHSPSRAPMQSAEYGSGSESRAYGTNGSFVTRAGATHLGSSTHYSSGRVESQSRDEIIQRMQE

MFSHLDPEVICMVLSEVDFKVDNAMDSLLEMSDAAEGIAPPSPPLSGFEQAAALLRTNTTQTDLLSGTAQ
RFVFSNQPRSQPELSPDETCLTEEFDALLDQELQTLTTQQMQASSHSPVSSIPLSSLPSSSQLLSLASQP
QCPAPQSLAEHAEHVSYSDPGLSEARGGSSPVNELSFGGGHIPETKTISLDFSHLTLEANSTEPRPSAFQ
VYRRPDQLHNHAGAKKHQESLSTPAMFWNTQAAEFHPHSMGPPFITPVIPNPTPWSSNPIPASQWLTHAT
VRQAPLKPSATVPKSWTLPTRHRLKLEGQVLVLLRGAPGSGKTTLANAMLAQNPGGVVLSTDEYFMRNGH
YYFEPNLLGEAHEWNHQRAKDAFEKGRSPIIIDNTNTQCWEMKPYVAMAQKHRYKVLFREPDTWWKTKPR
ELEKRTRHQVTKEKIRRMLERYDRFVSVQTIMNSNKPEPVPSVAALTQIETEQPQQFLHPGLTHPDLVGD
SDLSKLNLHLSSSLPDVSSVSQIYNTTSAGEEESEMALYTCKESMLTCENVQEDARTPQSDLLDSEGLDS
ELDACLVDTDKEFSNLRSGINEEYIVSEHETFHEPPVAFSESIAQRIRKVRENDRKPQVKSVIDQPVNLD
PILDSSEPKKEKRCEGEPFVEDKVVRPELLDFVGDWPLVSQDQRQGRCQNSRNNIFNNSGLEASVVEHQD
NKDKDKDGQDKDVSTDSTAEFQKLVDLLQGGGRNSFPDFYHTPASNTLTTLQSSLLSVKESDRDLEPMVL
PELPDCVLERKYSEGTNPRKDYIYSPSTPEQGADPIGPSNQMSSEKSTQDEIEQEDNGKGSKVVIDSEGS
LERRKGLSRRSGKSCKLALTFTNQSTSSPCSQSPVVSHLPSEITPAGGTPSLPMLCSSTSAQTYPQDFAL
LWRIDQQKSFELASDSSNSRMFVLEGNSLRFTPKINEEESAWQHRVPYRVVHEKGSQVEDNDFRESNSKQ
QNLEILSGHFRHVSVDILEDLYEKCHQDIEWTTNLLLDSGERLYKEDNEVDLGSLVPMQESCELSIKPTE
TVVCSQSDQARALLSDDSSNSSLSSISPKSQETNPSEVSPASDDWSPGQSSLSDAEGYDEESMTEEICES
EPSHETVKQLKEDTKIQLAVPAIKEELAEKTGEQDAGLQDTLVSQSEQIVRLLEEDLKEWKEEEGGGEER
EDDETKAEVNALTQLLLSQVDEMEQKEEEERKERENERRRTGLGKREQSSMDIQSLELKLPTELALQLTE
LFGPVGISPGAFSSDDCAVQIDLNLAKLLHQKWKETIQEKHRLAALSYQLMQECPTQWGSKSKLRDQTAL
HEEIPFMDHWNASCAPVSLRDIMIEEQVMQDSVEKCRSSRKDLDKKDSAAKLKENQLFSMFPTIDRHFLR
DIFRDHNYSLEQTEQFLQTLLDDGPVKNVVAPEPSPQRNGTYRAPSKERKWKSRDGEVDAAQFQDSEDPE
YDDFRTEATLQRRQQIECFNKAAEAHRQGRKDVASFYAQQGHMHGEKMREANHRAAMQIFQQVNASLLPQ
NILDLHGLHVDEAIHHLSQVLLDKCLEFHQGVCPPQLSVITGRGNRSQGGVARIRPAVLDYLKNQHYSYT
EPKIGLVLVTLH

>AOJMH5_DANRE
MEGNRDEAKKCLNIAVKAIEDGNKNKALKFLYKAEKLYPTEKAKVLLEALLNNGNSTGNSTAYCGKSSNG
SKPGSQTQHEAPKQDSAAADLTKGFTKEQAEGVQRIKKCKNYYEVLGIRKDASDDELKKAYRQLALKFHP
DKNHAPGATDAFKKIGNAYSVLSNPEKKRQYDLSGGEEPSTPNYSSHEGFDFHRGFESDITPEDLFNMFF
GGSFPSSNSHEFTNGRTYSHHTEETRGERVEERGDGGFSMFIQLMPIVVLVLVSILSQLLVSTPPYSLYS
RPSTGQTVKRQTENLHVDYFVTTDFKSEYKGSALLKIEKSVEEDYVSNVRNNCWKERQTKTDLLYAAKVY
RDERLQRKAELMTMENCKELDRLNDLFRGG

>AOJMI1_DANRE
MNSAIKVTNSTAIRAEVRRHENLQKTINKFIKQLERVEDQQLRTGLKVFLHSIQASCANSQEWTLTRTIP
ELRLGVLGSIRSGKSALVNRFITGSYLPLESHEGGRYKKEVLVEGQSQLLLIREESGPPSAQLCNWLDGL
ILVFSLENEASFQEVYKNYSELSAHRNIAEIPIIAVGTQDKISSTNARVIEDKRVQQLCIDVRRCTYYET
CATYGLNVDRVFNEMTQKIVAAKKQAALLASCKSLPNSPSHSGASTPVSGPGQASNGAQSSDYPSSLPST
PVISHKDIRGGASRDGVTQRNPPRRRTSLFANRRGSDSEKRSSDSRSDTSRSVPIKQGTLWKRSERSLNK
EWKKKYVTLSNSGMLTYHSNINEFLQNAQGKEMDLLRVTVKVPGKRLHRAAAPGAPSPGPALVPVPGVNG
LSKDKQTPEGGAAPNLLTVEEASRAGLSYPNDQKVKRCPSSVSNKGFSVDSSIEGVTSPPLGKEHMPSSP
MTDRKKKKRNRSINLKGDAAAGQAEEEESVDFIIISSTGQSWHFEAQSQEDRDAWVQAIESQILASLQSC
ESRNKARRNSQSEAVALQAIRNAKGNDLCVDCAAPNPTWASLNLGALICIECSGIHRNLGTHLSRVRSLD
LDVWPSELTKVLSAIGNHMANHIWETCTQGCQKLTPEATREQRESWIRAKYEQRAFVSPLPAQCSEDTMS

TWLLKAVINRDLPNLLLLLAHSTKELINIPPEGAGQQHHSALHAACQLGDVVMTQLLVWYGSDVKSKDPQ
GRTALTLARQAGSKECAEILVQHGCPSETSPTSPTPVLSRKSSITSVGRVNSRRRVS

>RRF2M_DANRE
MLLSLTFPVLRGCTGHLVNRSLQAPRWRVTWKRSYSLLQDEVKSLRTVVNPDISKIRNIGIMAHIDAGKT
TTTERMLYYSGYTRALGDVDDGDTVTDYMAQERERGITIQSAAVTFDWKDYRINLIDTPGHVDFTLEVER
ALRVLDGAVAVFDASAGVEAQTMTVWRQAEKHQIPCVCFLNKMDKPAASLRYSLDSIKAKLKANPVLLQI
PIGSGKSFTGLVDLITRQKMMWQGNALTNDGRSFEINSLQPSDDPNVLLAVSEARAALIEQVADLDDDFA
ELLLGEYGENFDAVPAVKLQEAVRRVTLARKGVPVLCGSSLKNKGVQPLLDAITAYLPAPNERNHDLVRW
YKNDLCALAFKVVHDKQRGPLVFVRIYSGSMKAQSSVHNINRNETEKMSRLLLPFADQQIEIPSLSAGNI
ALTVGLKQTVTGDTIVSSKASAAAAIRRAQAEAESRSNSHSAALAGVEVPEPVFFCSIEPPTMAKQADLE
HALNCLQREDPSLKVRIDPDSGQTVLCGMGELHIEIIHDRIKREYKIETHLGPLQVAYRETILQSATAKD
LLDRILGEKRHVVSVELTVHPLKENSSASCDITFEEDVKAMLPADVREAVENGVQSAYLQGPVLGFPVQG
VQTVIQNVRLESGTSAAMVSACVSRCMLKALKQAGGQVLEPVMALEVTVGEEHLSSVLADLSQRRGTICD
IQSRQDNKILLADVPLAEMMGYSTVLRTLTSGNATFSLELSSYEPMNSQDQNILLNKMAGLT

>A0JMK6_DANRE
MMLSLPKLSAGGVVVLLATLLHTLTVQGASVRHHRLRGGDQGGFLAPSSDMIKALEYIESLKQRADGPES
PTGDYDEVDKFRFLVQLASLQDENTPTHEDATRWPDNKVPQWVRSLLRVLDQAGESPESQAAGNERRLHK
TRRPVADGESPAGDYAGFVKPHKKYPLMFEDEENGRDNKRATEDLDEQYTPQSLANMRSIFEELGKLSAA
QKRDDEEDGEDDDDLYRVRNAAYEDVTGGEEWVPLEEQLETEELVKGSHEEYKRALGDISEQGMENMERR
GEEEDENPDDDTKLVDYYLLKVLEMTDQAQKRDLMEGRRRLLSRPSLIDPRAIKQLLSAISMKLQVPPED
LVGMLFMEETRKQQQRLPEPQLARNPSQPRYKSRVIKYYNGRQPEVTVSDIPHDVKTEDILKVLGLGNLA
NKNAKFSLLKQRPYKTAMTNYFNPNGRRGSLFLSELNKAPSKRKDDYDDDDAVDEDEESTFLAAKLLTEY
PDTSSSNRKRAIDSAANGQLPYELYEEAMKDFFDQVDNGKSALAKRDTQGKEEPEGPQKPPAQDPAQETV
DQTPPESGTEDGKEYHGKIVAGM

>BICR2_DANRE
MAPTMGVDDLLASPQDDRSPTLLEKDLILAAEVGQALLEKNEELASQIMQMESEMEAMQQEKHMVQRRLE
VRDLEASQREAELQADISALRAQLEQKHIQGRDRRREESEQLIQLSNHNQKLVEQLAEAVSLEHTLRTEL
RTLREEMEDTSFSKSISSARLDSLQAENRVLKERCTHMDERLKSTQEDNERLRSERDGLRERAIELQTSL
KDKETELEQEHSTVFQLRTVNRTLQQRVQALGEEASLGEATCFPLSLQSEIQQCQAKETILAHSSVLREK
EEEIQRLQKELQSRETELEGLREEVKLFRNSPGKPTYKALEEEMILARQERDALNQQLLNTIRHKVALSQ
EVESWQEDMRLVICQQVQLQQQEKEKENNKERTGFQRGTRTTKSLRLRGEEGRKGFFSALFGGD

>CAF1A_DANRE
MVVVMLAAEEPLASTPRRDMDCVGKANTNKKLVQARLPFKRLNPEPKECNEPKRTKGPVAPKCSEPSDQE
NDQDSSSISHHGPALVNGRGPLDCFMSRRKRSPLRSAPEATIDLTEDSNDSAKQQPAPPIAATCPLSEEK
TKTSEGTTEPTIPLTEEETEKDEAEDVDALPLLDITQDSDTEEEEEEEEEEEEQQQEAEVSHGNESVLST
GSTSSASVIASSPEPSKSAPTTPASTSRINAANKVKRRSLKSVQEQEEKQRQRDEKERLKQEAKAAKEKK
KEEARKMKEEKEREKKEKKEKDEKERREKKERDEKEKADKLKAKEEQRQMKIEAKLEEKRKKEEEKRLKE
EKDRIKAEKAEITRFLQKPKTQLAPKTLASACGKFAPFEIKAHMSLAPLTRVQCEDSVLEDLDRYLAQPD
STLNGLKDWTGHKPRSSGPTRPRHSAQGDCVVITESQKADDGPDRSRYGRMKLLHFHDNYRPAYWGTWSK
KSTHISPRCPLRLDKDLLDYEVDSDEEWEEEEPGESLSHSEGDDDDEAGEDDDDDDGFFVPHGYLSEGEG

ALEDEEGGDPEKQKVRQRMKAREWENELMSKGKVKVLEAVVRGCFWEGEKPLPDFLQPYAVCMLEPLSKD
EASTPEQDASRQQRNEKLLTMLLPLLHGNVNSNKVIITEFLEFCRQQTSSPTESLLNTTESIPPRIHVRR
IIKENAVYEKRSTFKRCCWYVHADVLARYSQEALPVPCQWSYLTSGANVTRDEHSGSQGNSPTTNSSTTP
SNKRKSASSHSITKYMKKCGESEQTEAMETDGFQADTEDDEDDDCIIIGEQSGSSEQDINTSLPQTDRET
EPMDTSASETAALALPCPTPATA

>A0JML0_DANRE
MPRGHKQHCEFAQFDISAILNFLSACKHFKPFLTKGENVKKVINVRNVVMHSPDLKMNNEDMNRHLETIF
QFADMLNSKVSALSVLREKIEQFNNILDKNFNQTEVDGQHKDLKTMVDFQEVLNREQQALKDRIEYLITH
FEGNLDKNENSPDMTTLMEFLHQNKDLLENLGPEVYKLKGMQTKLNQHEKQINNLTNRVDQLEKVKETTN
TAGQSSSQITNYPKFIKDNRSWLINTVKNIDQILDDLSELHSESVANVKAKQTKQAMMRELLLYVNCERI
AKDLFNALLKHEKRPMEERLKGL

>A0JML6_DANRE
MNRMGSMCTFDEIRMDDRLNGHRTLRLTLHEQNQQQQQPVTDKPVGRAFALIQPTYERKPLKSDLIKPSD
DQGEVIKVYLEDRKEAQARHQQSLKMLSEEVSQIQEVRYCLKSLREQMAAKSHRAEHKLVLSGARKVNGT
HSALTQSLNGLERQDSMDEQEKEKMREASKRLYGQLQEAEKKHQEEREKLLMEARQYKQQLSEQSEHLKR
IQQSKQQQDQQIDDLRRLMSGMEQESSGLREQLMSREAEILQLRELRDEGHVGTQRLEELEKDNAILKEK
IHHLDDMLKSQQRKMRQMIEQLQNSRMVIQERDRAIRELEEKVAMLEAENKQMRDQMDYYLGSQRSNSFL
PSDSNAQIVYSKPLRPSSQSNKSLPFIKVIEIKS

>DALD3_DANRE
MEESAPFSIVSTVKALSGALRGDAGHEADISREREKLWFKESSAKNLRNRDFLAPNTVLTTLYAGGEVPS
DILSAVHTLRGSGVLPIGATEVTTEGLRVCVDRCAAFRGVLCGIMPYLKPAAQRQGCVLINCPALHTKQS
VPSPDTLALGQLRTILIADHLGAQLRRQGYTVSFCPALPQESDIVNFLKTLGIDWPTVPVSWTNEDREEK
MKKALENSAYRDRETEKGKRRSRGEEIEGEKRGLKEDVRINLKQVVQDENLNGYDPSLGTCTVQREALCH
LAQLDSATADFPASTTTALHVTSCQDEFRQQQTAMLWRAAGATALQRLVICGPVKTPGVQMNAAQYFQLR
KAQMKEASEMKYGDQVEGQTWDDIIRVMTSATVRFELLSTVHTSPVTLDVQRDSGVSTKGPRGGVFVMYN
CARLHTLFSSYEKGVEQGLYPEIPGGTELDFSALKEEGEWLLLFNYLIPFSEILDQSAQTLEHEGGGARV
QLRTEQVCRFLVSLSKDFSSYYNRVHVLGEPLPHLFNQMFCRLLLLRALRELYHSALDSLNLPPIPQL

>A0JMM0_DANRE
MAEPRFNNPYFWPPPPSIPGQLDNLVLINKIKEQLMAEKIRPPHLQPTSVPSQQPLLVPSAGTEGGQHNI
STPKLQQMPGLHAHGTSQPDIALHARPASSSVTGRILGDVNLNLDDKTAIKARGLWEDWHLRQIIDQPSR
ANHLSGLSLASSRNANHNTSESVTPSTPTSPTIGGQSRQGIPTANLLSGLTGGPGMEALKNSGALMGPPM
KSIGRGRKKIKAENTSGPLLVVPYPILASGAEQSAVITAKEGKTYRCKVCPLTFFSKSDMQIHSKSHTEA
KPHKCPHCTKTFANASYLAQHLRIHLGVKPYHCSYCEKSFRQLSHLQQHTRIHTGDRPYKCLQPGCEKAF
TQLSNLQSHQRSHNKDKPYKCSNCYRAYSDSASLQIHLSAHAIKNAKAYCCSMCGRAYTSETYLMKHMSK
HTVVEHLVSQQSPPRAESPSVPVRISLI

>A0JMM2_DANRE
MDAVWMLPVLLVLPLLLWTSSTFVFYFKKCFYVAYMMLLAVIAIPICILKSGGRDIENMRVIRFLVRHVK
YFLGLRYQVSGWEHLQTEGPYVIISNHQSSLDVLGMVEILPDRCTMIAKKELIWAGTVGMICWLGGIVFI
NRKKTSDAKNVMSDAAKTMLTDKIRLWVFPEGTRNQNGGLLPFKKGAFHLAIQAQVPIIPIVFSSYSKFY

LRKEKEFKSGTITLKVLPKIETKGLTADDVTTLSDQAFGVMRSAFMEISGQSAQSNGPSTH

>A0JMM5_DANRE
MFYAQLFTSKRGTLAKIWLAAHWEKKITKAHVFECDLETTIKEILSPQIKIGLRTSGHLLLGVVRIYSRK
TRYLLADCSDALVKIKVAFRPGQTDLPDDAMEATLKTITLPEDFTDFDSQLPDLNTIDVVDHFSLNQCRT
EDITLKENFGNLFLSLERIGEEMQSYQGLFDQSFSVHGDCFGDEEMAVDLIDIIANTAEDTLMSDFNGNS
INELPATPPPTAVNAVEPEKPETSCLKERSSPDVTETTFLVNEDEGFALAPVSATPCSAKKRKVRKRKLV
IDRSKQLTNSAIRDQLADCSDILSPLEIAPPTRQLMEWRESGGVKQLISSFCLPVLHPDLKQLFPSEVSP
RRQGLKGGARQQTDPEEMREQTREAVEEDTVQPVMDDWSLLQDSVAQEQPVDADTTLEDSVCHPEPPPSW
NSTIESRLEVSYPEPQSEISTLSHPSVEMRETPLVTKTQSVLGSQDLEDRRMTSRAHDLLQALKSQDSSP
DAVYSLHALCESGSRSYVAALFFCMLVLRKQQFLNLHQSAPYSDIIATPGPLFHSI

>A0JMM6_DANRE
MWKSVVGHDVTVETESQGDDWETDPNFENDVSEQEQRWGAKTIEGSGRKEHISIADLRQNVSREHEVVKK
KELDQGPKASYGYGGKFGVEKDRMDKGALGHSYVAEVEQHSSQTDAAKGFGGKFGVQKDRVDKSAMSYEY
KAQVQQHASQKDYAQGFGGKYGVQKERVDKAAMGYDYKGETEKHQSQKDYAKGFGGKYGVEKEKVDKAAM
GYDYKGETEKHQSQKDYAKGFGGKYGVEKEKVDKAALGYDYKGETEKHQSQKDYAKGFGGKYGVEKEKVD
KAALGYDYKGETEKHQSQKDYAKGFGGRYGVQEDRMDKNASSFNKMESPSSSYEKPQAFEGSSVGAGNLK
ARFENMAKASDEDNKKKAEEERTRRLAREKREREEARRKQEEQSQHVEEEKQSPPPVPVSQKPQQEFRKL
PEIPREEIEPDVEEENQPENEEPPSLPPRPSDLLEDETYAECEPPPVPQEEDYEDVGSYSAQAGENDYED
LGGGGTTARAIYDYQGEDSDEISFMPDDIITNIEMVDEGWWKGMCNGRTGLFPASFVELL

>A0JMM7_DANRE
MDHDTQWLYQLLADVQLERFYLRMRDGLNVTRMEHLSYVKEADLEQIGISKPGQRRLWEAVKRYKTNMRA
RSWMVKATNGRAPEGNDQSNAAGSGQGPVRALTCLIQDRELLFGDRLGSGSFGVVRKAEWHTPTGRVLPV
AVKTLRSGQVDLVSDFLQEVTSMQSLDHPNIIHLYGVVLTHPLKMVTELAPLGSLYDTLRLRHGEYPLSR
LWLFSTQIAAGMEYLESRRFIHRDLAARNVLLAAREQVKIGDFGLMRGLDDRDHYIMTAHRRIPFAWCAP
ESLRTGSFSHASDVWMFGVTLWEMFTYCEEPWLGLSGRQILYRVEREGERLERPPDCPQELYAVMRKCWA
CNSTERPTFAQLTTMVSEAQPMEVRSVKDIAEPRRLSLQANDLVTVIDHGLDMCEWKGQNQRTLSVGWFP
PALTAPALTAVVPASGPTLISSPVRGSLHHTGHGDTDPGRSRGTPERIDDNRRWRVPLAKEKEGSNLKKM
AGMSRSLESVLGGPQDKGRGVGVNAAQRSDQRRNMQNNNVIQDPRRFSDAVVIPPPRPPPPNIKSIKPQM
MMMFLDRRAANPSGWSPQTQAQPQFQPQQQGLGTSNLTRMTHLAKSSPQLDDAIEKDKEREKEQERERER
EKGKERYPPLVDKEAVIAQVQAAVHGVTNEEVQKALHRCDWNPAKAEQQLKTDQLYYMNQCTREECQKVL
SRYNWDLQIASRYVIRMAKERDRVLDRRGERV

>A2LDC_DANRE
MIAIHILLLVFSNIVLPALCINVFVYGTLKKGQSNYHELTNTTHGQAEFITCARTKDPYPMVIATKDKFP
FLLNVPGSGQQVYGEIYNVDQNMLDFLDEFEECPDLYQRTSIQLKILKGNGDSEEAFVYSTSTFDPDWLN
KPTFSVYDATGDPGNNCVSRE

>ATHL1_DANRE
MGGVYNGDGGTCHRGNIPCPLAAQMKTGEVGRQLYELNMHTGVFSHTVVTSDFEAIQVLYAHRNQSNLLV
MEILLKRIKTSAEPITIQLESSFKPQSEDIAFQNAPDYKGGRHIFGQTASSEVPGGVRPVVHLIWTPVTP
TLTLPANQSQSSWTFLVAVARSNESAQSFYDSGLALINTGDLRPSHQRSWAELWKGSSIEVIGAESLNRA

LIGCMFYLLGSFPYVNKEASAAFEFGGVSPGGLSNGSEDEDYHGHVFWDQDTWMYPSIALFYPALARAVL
QYRVETLEGAQVNAQQMGCKGLKFAWESAVTGVDVCPEDVYSQQELHINGDVILAFQQYYYLTQDLELFQ
SGRGSEVVWGVADFWVSRVTWDSADQQYHIKGVIPPDEYYFTVDNSVFTNAVAQRSLEFAVELSALLAEV
PPPAWQDIADKIKIPFDPELKFHPEFDGYKPGNKVKQADVVLLGFPLAFPMSPEIRRNDLEMYEAVTDPL
GPAMTWGMFALGWLELGEAEKAQKLLQKCFKNVQKPFQVWSESADGSGCVNFLTGMGGFLQAVLFGYTGF
RVQKEQLAFSPLLPLDVSALSVKGVCYLGHKMDWTITSEEVKVSVRKTDSKETFTLQVVLNSGSTLLLTP
GQSVSFPRQPGHICQLKSSSSCWPI

>A0JMP5_DANRE
MWFSWMLLLATLASSSASSRTREWAHHGAMMFADLTPQEMYAVRDYLYSCSELGLTSARGTSLKKNSILL
MELHVPRKHEALRALDKGQAKPSRQARVVVQFGNQAVPNVTEYIVGPLPFPKTYHLKTFKNNKNIRFESR
PISAVEYEHLSGVLDKVGSKVNKILQESTGFTYGNCTKRCLTFSDIAPRGLTSGERRTWIMLQKFVEGYF
IHPVGFEVLVNHKDLDHEKWTVEKVWYNGQYFDSLDEFVEKYEKGTVDKIKLPEHDEEDLFSTYIPRGDM
NTRTNIHGAKLVEPQGRRFQVDGNFVEYAGWSFAYRVRSSAGLQIFDLRYNGERIAYEIALQEAIAFYSG
DTPAAMQTKYIDAGWAMGTSDYELSPGIDCPEIAHFVDLYHYYDTDKPVRYRNALCIFEMTTGIPLRRHF
NSNFQGGYNFYGGLENHVLVIRTTSTVYNYDYIWDFVFYQNGVMESRVSATGYIHATFFTENGLNYGTRV
YNYVLGNLHTHLIHYKVDLDISGRENSFESIDLKYVNFTNPWSPGHTIMQSKLHRTQYETERSAAFRFGK
KFPKYLHFYNPNQLNKWGHKKGYRIQYNSHANSVLPRGWREENGIPWSRYPLAVTRHKDSEVTSSSIYTQ
NDPWEPLVSFEEFVRNNENIVNQDLVAWVTVGFLHIPHSEDVPNTATPGNSVGFFLRPFNFFNEDPSLAS
RSTVIVRPDEKGQPKVQRWTPEVVGHCVSDKPFFYNGTYAGV
