# Supplementary material for: NR-2L: A Two-Level Predictor for Identifying Nuclear Receptor Subfamilies Based on Sequence-Derived Features
Source: PLoS One. 2011 Aug 15;6(8):e23505. doi: 10.1371/journal.pone.0023505 (PMC3156231; doi:10.1371/journal.pone.0023505)
Supplement: Supporting Information S2 — The independent testing dataset ST contains 500 non-NR proteins and 568 NR proteins classified into the following 7 main subfamilies according to NucleaRDB (http://www.receptors.org/NR/): (1) NR1:thyroid hormone like; (2) NR2: HNF4-like; (3) NR3: estrogen like; (4) NR4: nerve growth factor IB-like; (5) NR5: fushi tarazu-F1 like; (6) NR6: germ cell nuclear factor like; and (7) NR0: knirps and DAX like. Both the accession numbers and sequences are given. None of the proteins included here occurs in the training dataset S. (PDF) [file pone.0023505.s002.pdf]

Supporting Information S2. The independent testing dataset $\mathbb{S}_{\mathrm{T}}$ contains 500 non-NR proteins and 568 NR proteins classified into the following 7 main subfamilies according to **NucleaRDB** (http://www.receptors.org/NR/): (1) NR1:thyroid hormone like; (2) NR2: HNF4-like; (3) NR3: estrogen like; (4) NR4: nerve growth factor IB-like; (5) NR5: fushi tarazu-F1 like; (6) NR6: germ cell nuclear factor like; and (7) NR0: knirps and DAX like. Both the accession numbers and sequences are given. None of the proteins included here occurs in the training dataset $\mathbb{S}$.

---

1. 568 NR proteins $\mathbb{S}_{\mathrm{T}}^{\mathrm{NR}}$

(1) 231 NR1

```
>THA_CHICK
MEQKPSTLDPLSEPEDTRWLDGKRKRKSSQCLVKSSMSGYIPSYLDKDEQCVVCGDKATGYHYRCITCEG
CKGFFRRTIQKNLHPTYSCKYDGCCVIDKITRNQCQLCRFKKCISVGMAMDLVLDDSKRVAKRKLIEENR
ERRRKEEMIKSLQHRPSPSAEEWELIHVVTEAHRSTNAQGSHWKQKRKFLPEDIGQSPMASMPDGDKVDL
EAFSEFTKIITPAITRVVDFAKKLPMFSELPCEDQIILLKGCCMEIMSLRAAVRYDPESETLTLSGEMAV
KREQLKNGGLGVVSDAIFDLGKSLSAFNLDDTEVALLQAVLLMSSDRTGLICVDKIEKCQETYLLAFEHY
INYRKHNIPHFWPKLLMKVTDLRMIGACHASRFLHMKVECPTELFPPLFLEVFEDQEV

>THA1_SHEEP
MEQKPSKVECGSDPEESSTRSPDGKRKRKNGQCSLKTSMSGYIPSYLDKDEQCVVCGDKATGYHYRCITC
EGCKGFFRRTIQKNLHPTYSCKYDSCCVIDKITRNQCQLCRFKKCIAVGMAMDLVLDDSKRVAKRKLIEQ
NRERRRKEEMIRSLQQRPEPTPEEWDLIHVATEAHRSTNAQGSHWKQRRKFLPDDIGQSPIVSMPDGDKV
DLEAFSEFTKIITPAITRVVDFAKKLPMFSELPCEDQIILLKGCCMEIMSLRAAVRYDPESDTLTLSGEM
AVKREQLKNGGLGVVSDAIFELGKSLSAFNLDDTEVALLQAVLLMSTDRSGLLCVDKIEKSQEAYLLAFE
HYVNHRKHNIPHFWPKLLMKVTDLRMIGACHASRFLHMKVECPTELFPPLFLEVFEDQEV

>THB2_MOUSE
MNYCMPEVHEVCPAASSNCYMQVTDYLAYLEDSPALSGRDVQAVPSSSIYMEQAWAVNQPYTCSYPGNLF
KSKDSDLDMALSQSSQPAHLPEEKPFPQVQSPPHSQKKGYIPSYLDKDELCVVCGDKATGYHYRCITCEG
CKGFFRRTIQKSLHPSYSCKYEGKCIIDKVTRNQCQECRFKKCIYVGMATDLVLDDSKRLAKRKLIEENR
EKRRREELQKSIGHKPEPTDEEWELIKTVTEAHVATNAQGSHWKQKRKFLPEDIGQAPIVNAPEGGKVDL
EAFSHFTKIITPAITRVVDFAKKLPMFCELPCEDQIILLKGCCMEIMSLRAAVRYDPDSETLTLNGEMAV
TRGQLKNGGLGVVSDAIFDLGMSLSSFNLDDTEVALLQAVLLMSSDRPGLACVERIEKYQDSFLLAFEHY
INYRKHHVTHFWPKLLMKVTDLRMIGACHASRFLHMKVECPTELFPPLFLEVFED

>THB1_HUMAN
MTPNSMTENGLTAWDKPKHCPDREHDWKLVGMSEACLHRKSHSERRSTLKNEQSSPHLIQTTWTSSIFHL
DHDDVNDQSVSSAQTFQTEEKKCKGYIPSYLDKDELCVVCGDKATGYHYRCITCEGCKGFFRRTIQKNLH
PSYSCKYEGKCVIDKVTRNQCQECRFKKCIYVGMATDLVLDDSKRLAKRKLIEENREKRRREELQKSIGH
KPEPTDEEWELIKTVTEAHVATNAQGSHWKQKRKFLPEDIGQAPIVNAPEGGKVDLEAFSHFTKIITPAI
TRVVDFAKKLPMFCELPCEDQIILLKGCCMEIMSLRAAVRYDPESETLTLNGEMAVTRGQLKNGGLGVVS
DAIFDLGMSLSSFNLDDTEVALLQAVLLMSSDRPGLACVERIEKYQDSFLLAFEHYINYRKHHVTHFWPK
```

LLMKVTDLRMIGACHASRFLHMKVECPTELFPPLFLEVFED

>Q6F3J6_CONMY
MEHMPKEQDPNLSEGEEKRWLDGPKRKRKNSQCSVKSMSVLSLSVPGYIPSYLEKDEPCVVCGDKATGYH
YRCITCEGCKGFFRRTIQKNLHPSYSCKYDGCCIIDKITRNQCQLCRFKKCIAVGMAMDLVLDDKKRVEK
RRLIEENRERRRKEEMVKTLQNRPEPSGSEWELIHLVTEAHRHTNAQGSHWKQKRKFLPEDIGQSPVAPT
SDGDKVDLEAFSEFTKIITPAITRVVDFAKKLPMFSELPCEDQIILLKGCCMEIMSLRAAVRYDPDSETL
TLSGEMAVKREQLKNGGLGVVSDAIFDLGKSLAQFNLDDSEVALLQAVLLMSSDRSGLTCVEKIEKCQET
YLLAFERYINYRKHNIPHFWPKLLMKVTDLRMIGACHASRFLHMKVECPTELFPPLFLEVFEDQDV

>THB1_RAT
MTPNSMTENRLPAWDKQKPHPDRGQDWKLVGMSEACLHRKSHVERRGALKNEQTSSHLIQATWASSIFHL
DPDDVNDQSVSSAQTFQTEEKKCKGYIPSYLDKDELCVVCGDKATGYHYRCITCEGCKGFFRRTIQKSLH
PSYSCKYEGKCIIDKVTRNQCQECRFKKCIYVGMATDLVLDDSKRLAKRKLIEENREKRRREELQKSIGH
KPEPTDEEWELIKTVTEAHVATNAQGSHWKQKRKFLPEDIGQAPIVNAPEGGQVDLEAFSHFTKIITPAI
TRVVDFAKKLPMFCELPCEDQIILLKGCCMEIMSLRAAVRYDPDSETLTLNGEMAVTRGQLKNGGLGVVS
DAIFDLGMSLSSFNLDDTEVALLQAVLLMSSDRPGLACVERIEKYQDSFLLAFEHYINYRKHHVTHFWPK
LLMKVTDLRMIGACHASRFLHMKVECPTELFPPLFLEVFED

>THAA_XENLA
MDQNLSGLDCLSEPDEKRWPDGKRKRKNSQCMGKSGMSGDSLVSLPSAGYIPSYLDKDEPCVVCSDKATG
YHYRCITCEGCKGFFRRTIQKNLHPSYSCKYDGCCIIDKITRNQCQLCRFKKCIAVGMAMDLVLDDGKRV
AKRKLIEENRQRRRKEEMIKTLQQRPEPSSEEWELIRIVTEAHRSTNAQGSHWKQRRKFLPEDIGQSPMA
SMPDGDKVDLEAFSEFTKIITPAITRVVDFAKKLPMFSELTCEDQIILLKGCCMEIMSLRAAVRYDPDSE
TLTLSGEMAVKREQLKNGGLGVVSDAIFDLGRSLAAFNLDDTEVALLQAVLLMSSDRTGLICTDKIEKCQ
ETYLLAFEHYINHRKHNIPHFWPKLLMKVTDLRMIGACHASRFLHMKVECPTELFPPLFLEVFEDQEV

>THB1_MOUSE
MTPNSMTENGLPAWDKQKPRPDRGQDWKLVGMSEACLHRKSHVERRGALKNEQTSPHLIQATWTSSIFHL
DPDDVNDQSISSAQTFQTEEKKCKGYIPSYLDKDELCVVCGDKATGYHYRCITCEGCKGFFRRTIQKSLH
PSYSCKYEGKCIIDKVTRNQCQECRFKKCIYVGMATDLVLDDSKRLAKRKLIEENREKRRREELQKSIGH
KPEPTDEEWELIKTVTEAHVATNAQGSHWKQKRKFLPEDIGQAPIVNAPEGGKVDLEAFSHFTKIITPAI
TRVVDFAKKLPMFCELPCEDQIILLKGCCMEIMSLRAAVRYDPDSETLTLNGEMAVTRGQLKNGGLGVVS
DAIFDLGMSLSSFNLDDTEVALLQAVLLMSSDRPGLACVERIEKYQDSFLLAFEHYINYRKHHVTHFWPK
LLMKVTDLRMIGACHASRFLHMKVECPTELFPPLFLEVFED

>Q7T0K4_AMBME
MDQNPSDLDCLSDPDEKRWLDGKRKRKNSQCLLKNSMSGYIPSYLDKDEPCVVCSDKATGYHYRCITCEG
CKGFFRRTIQKNLHPSYSCKYDGCCIIDKITRNQCQLCRFKKCIAVGMAMDLVLDDSKRVAKRKLIEENR
ERRRKEEMLKSMQHRPEPTSEEWELIRVVTEAHRSTNAQGSHWKQKRKFLPDAIGQCPMTNVPDGDKVDL
EAFSEFTKIITPAITRVVDFAKKLPMFSELPCEDQIILLKGCCMEIMSLRAAVRYDPDSETLTLSGEMAV
KREQLKNGGLGVVSDAIFDLGKSLSTFNMDDTEVALLQAVLLMSSDRSGLTSIDKVEKCQETYLLAFEHY
INYRKHHIPHFWPKLLMKVTDLRMIGACHASRFLHMKVECPTELFPPLFLEVFEDQEV

>THAB_XENLA
MDQNLSGLDCLSEPDEKRWPDGKRKRKNSQCMGKSGMSGDSLVSLPPAGYIPSYLDKDEPCVVCSDKATG
YHYRCITCEGCKGFFRRTIQKNLHPSYSCKYDGCCIIDKITRNQCQLCRFKKCIAVGMAMDLVLDDSKRV
AKRKLIEENRVRRRKEEMIKTLQQRPEPSSEEWELIRIVTEAHRSTNAQGSHWKQRRKFLPEDIGQSPMA
SMPDGDKVDLEAFSEFTKIITPAITRVVDFAKKLPMFSELTCEDQIILLKGCCMEIMSLRAAVRYDPDSE
TLTLSGEMAVKREQLKNGGLGVVSDAIFDLGRSLAAFNLDDTEVALLQAVLLMSSDRTGLICTDKIEKCQ
ETYLLAFEHYINHRKHNIPHFWPKLLMKVTDLRMIGACHASRFLHMKVECPTELFPPLFLEVFEDQEV

>THA_RANCA
MDQNLSGLDCLSEPDEKRWPDGKRKRKNSQCMGKSGMSGDSSVSLLSAGYIPSYLTKDEPCVVCSDKATG
YHYRCITCEGCKGFFRRTIQKNLHPSYSCKYDGCCIIDKITRNQCQLCRFKKCIAVGMAMDLVLDDSKRV
AKRKLIEENRERRRKEEMIKTLQQRPEPSSEEWELIRIVTEAHRSTNAQGSHWKQRRKFLPEDIGQNPMA
SMPDSDKVDLEAFSEFTKIITPAITRVVDFAKKLPMFSELPCEDQIILLKGCCMEIMSLRAAVRYDPDSE
TLTLSGEMAVKREQLKNGGLGVVSDAIFDLGRSLSAFNLDDTEVALLQAVLLMSSDRTGLICTDKIEKCQ
ETYLLAFEHYINHRKHNIPHFWPKLLMKVTDLRMIGACHASRFLHMKVECPTELFPPLFLEVFEDQEV

>THA_SALSA
MEPISNVEDPNSSEGDEKRWPDGPKRKRKNSTCSVKSMSALSLSVQGYIPSYLEKDEPCVVCGDKATGYH
YRCITCEGCKGFFRRTIQKNLHPAYSCKYDGCCIIDKITRNQCQLCRFRKCIAVCMAMDLVLDDSKRVAK
RRLIEENREKRKKDEIVKTLQARPEPDSSEWELIRHVTEAHRHTNAQGSHWKQKRKFLPEDIGQSPRAPT
PDGDKVDLEAFSEFTKIITPAITRVVDFAKKLPMFSELPCEDQIILLKGCCMEIMSLRAAVRYDPESETL
TLSGEMAVKREQLKNGGLGVVSDAIFDLGKSLAQFNLDDSEVALLQAVLLMSSDRSGLTLVDKIEKCQET
YLLAFEHYINHRKHNIPHFWPKLLMKVTDLRMIGACHASRFLHMKVECPNELFPPLFLEVFEDQEV

>THB2_HUMAN
MNYCMQEIYEVHPAAGSNCYMQSTDYYAYFEDSPGYSGCDAQAVPSNNIYMEQAWAVNQPYTCSYPGNMF
KSKDSDLDMALNQYSQPEYFTEEKPTFSQVQSPSYSQKKGYIPSYLDKDELCVVCGDKATGYHYRCITCE
GCKGFFRRTIQKNLHPSYSCKYEGKCVIDKVTRNQCQECRFKKCIYVGMATDLVLDDSKRLAKRKLIEEN
REKRRREELQKSIGHKPEPTDEEWELIKTVTEAHVATNAQGSHWKQKPKFLPEDIGQAPIVNAPEGGKVD
LEAFSHFTKIITPAITRVVDFAKKLPMFCELPCEDQIILLKGCCMEIMSLRAAVRYDPESETLTLNGEMA
VTRGQLKNGGLGVVSDAIFDLGMSLSSFNLDDTEVALLQAVLLMSSDRPGLACVERIEKYQDSFLLAFEH
YINYRKHHVTHFWPKLLMKVTDLRMIGACHASRFLHMKVECPTELLPPLFLEVFED

>Q6F3J5_CONMY
MEHMPEEQDPNPSEGEEKRWLDGPKRKRKNSQCSVKSMSGYIPSYLEKDEPCVVCGDKATGYHYRCITCE
GCKGFFRRTIQKNLHPSYSCKYDGCCIIDKITRNQCQLCRFKKCISVGMAMDLVLDDSKRVAKRRLIEEN
RERRKKEEMVKTLQNRPEPTNSEWELIRMVTEAHRHTNAQGSHWKQKRKFLPEDIGQSPVAPTSDGDKVD
LEAFSEFTKIITPAITRVVDFAKKLPMFSELPCEDQIILLKGCCMEIMSLRAAVRYDPESETLTLSGEMA
VKREQLKNGGLGVVSDAIFDLGKSLAQFNLDDTEVALLQAVLLTSSDRSGLTCVEKIEKCQETYLLAFEH
YINYRKHNIPHFWPKLLMKVTDLRMIGACHASRFLHMKVECPTELFPPLFLEVFEDQEV

>THA_HIPHI
MEPMSNKQDSNSSEGDEKGWPDVPKRKRKNSQCSMKSMSALSVSVPGYIPSYLEKDEPCVVCGDKATGYH
YRCITCEGCKGFFRRTIQKNLHPAYSCKYEGCCIIDKITRNQCQLCRFKKCISVGMAMDLVLDDSKRVAK

RRLIEENREKRKREEMVRTLQVRPEPDTAEWELIRMATDAHRHTNAQGSSWKQKRKFLSDDIGQGPMVPT
SDGDKVDLEAFSEFTKIMTPAITRVVDFAKKLPMFSELPCEDQIILLKGCCMEIMSLRAAVRYDPESETL
TLNGEMAVKREQLKNGGLGVVSDAIFDLGKSLAQFNLDDTEVALMQAVLLMSSDRSGLTSLEKIEQCQEA
YLLAFEHYINYRKHNIPHFWPKLLMKVTDLRMIGACHASRFLHMKVECSSELFPPLFLEVFEDQEV

>THA_NECMA
MDQNLSDLDCLSDPDEKRWLDGKRKRKNSQCLLKNSMSGYIPSYLDKDEPCVVCSDKATGYHYRCITCEG
CKGFFRRTIQKNLHPSYSCKYDACCIIDKITRNQCQLCRFKKCIAVGMAMDLVLDDSKRVAKRKLIEENR
ERRRKEEMLKSMQNRPEPTSEEWELIRLVTDAHCSTNAQGSHWKQKRKFLPDAIGQCPMTAVPENDRVDL
EAFSEFTKIITPAITRVVDFAKKLPMFSELPCEDQIILLKGCCMEIMSLRAAVRYDPESETLTLSGEMAV
KREQLKNGGLGVVSDAIFDLGKSLSAFNLDDTEVALLQAVLLMSSDRSGLTCMDKVEKCQETYLLAFEHY
INHRKHHIPHFWPKLLMKVTDLRMIGACHASRFMHMKVECPTELFPPLFLEVFEDQEV

>THA1_BRARE
MENTEQEHNLPEGDETQWPNGVKRKRKNSQCSMNSTSDKSISVPGYVPSYLEKDEPCVVCGDKATGYHYR
CITCEGCKGFFRRTIQKNLHPSYSCKYDSCCIIDKITRNQCQLCRFRKCISVGMAMDLVLDDSKRVAKRR
LIEENREKRKKEEIVKTLHNRPEPTVSEWELIRMVTEAHRHTNAQGPHWKQKRKFLPEDIGQSPAPTSDN
DKVDLEAFSEFTKIITPAITRVVDFAKKLPMFSELPCEDQIILLKGCCMEIMSLRAAVRYDPESETLTLS
GEMAVSREQLKNGGLGVVSDAIFDLGKSLSQFNLDDSEVALLQAVLLMSSDRSGLTCVEKIEKCQEMYLL
AFEHYINHRKHNISHFWPKLLMKVTNLRMIGACHASRFLHMKVECPTELFPPLFLEVFEDQEGSTGVAAQ
EDGSCLR

>Q766D2_ORYLA
MEHMPKEQDSNSNPSEGEEKQWLNGPKRKRKNSQCSVKSMTGYIPSYLEKDEPCVVCGDKATGYHYRCIT
CEGCKGFFRRTIQKSLHPSYSCKYDGCCIIDKITRNQCQLCRFKKCIDVGMAMDLVLDDSKRVAKRRLIE
ENREKRKKEEMVKSLQTRPEPTVDEWDLIRMVTEAHRNTNAQGAQWKQKRKFLPEKIGQSPVAPTSDGDK
VDLEAFSEFTKIITPAITRVVDFAKKLPMFSELPCEDQIILLKGCCMEIMSLRAAIRYDPESETLTLSGE
MAVKREQLKNGGLGVVSDAIFDLGKSLAQFNLDDTEVALLQAVLLMSSDRSGLTCVDKIEKCQETYLLAF
EHYINYRKHNIPHFWPKLLMKVTDLRMIGACHASRFLHMKVECPNELFPPLFLEVFEDQEV

>Q5RZV6_PSEAM
MEPMSNKQDSNSSEGDEKGWPDVPKRKRKNSQCSMKSMSALSSSVPGYIPSYLEKDEPCVVCGDKATGYH
YRCITCEGCKGFFRRTIQKNLHPAYSCKYEGCCIIDKITRNQCQLCRFKKCISVGMAMDLVLDDSKRVAK
RRLIEENREKRKRDEMVRTLQVRPEPDTAEWELIKLATDAHRHTNAQGSSWKQKRKFLSDDIGQGPMVPT
SDGDKVDLEAFGEFTKIMTPAITRVVDFAKKLPMFSELPCEDQIILLKGCCMEIMSLRAAVRYDPESETL
TLNGEMAVKREQLKNGGLGVVSDAIFDLGKSLAQFNLDDTEVALMQAVLLMSSDRSGLTSLEKIEQCQEA
YLLAFEHYINYRKHNIPHFWPKLLMKVTDLRMIGACHASRFLHMKVECPSELFPPLFLEVFEDQEV

>THAA_PAROL
MEPMSNKQDSNSSEGDEKGWPDVPKRKRKNSQCSMKSMSALSVSVPGYIPSYLEKDEPCVVCGDKATGYH
YRCITCEGCKGFFRRTIQKNLHPSYSCKYEGCCIIDKITRNQCQLCRFKKCISVGMAMDLVLDDSKRVAK
RRLIEENREKRKREEMVRTLQIRPEPDTAEWELIRMATDAHRHTNAQGSSWKQKRKFLSDDIGQSPMVPT
SDGDKVDLEAFSEFTKIMTPAITRVVDFAKKLPMFSELPCEDQIILLKGCCMEIMSLRAAVRYDPDSETL
TLNSEMAVKREQLKNGGLGVVSDAIFDLGKELGQFNLDDTEVALMQAVLLMSSDRSGHQCMEKIEQCQEA

YLLAFEHYINYRKHNIPHFWPKLLMKVTDLRMIGACHASRFLHMKVECPSELFPPLFLEVFEDQEV

>THBB_XENLA
MPSSMSVRLFTASAAQRKKIQEGDCCVVLAGKTQGRFILIGAVARVSGYIPSYLDKDELCVVCGDKATGY
HYRCITCEGCKGFFRRTIQKNLHPSYSCKYEGKCVIDKVTRNQCQECRFKKCKTVGMATDLVLDDSKRLA
KRKLIEENREKRRKDEIQKSIVQRPEPTQEEWELIQVVTEAHVATNAQGSHWKQKRKFLPEDIGQAPIVN
APEGGKVDLEAFSQFTKIITPAITRVVDFAKKLPMFCELPCEDQIILLKGCCMEIMSLRAAVRYDPESET
LTLNGEMAVTRGQLKNGGLGVVSDAIFDLGVSLSSFSLDDTEVALLQAVLLMSSDRPGLSSVERIEKCQE
GFLLAFEHYINYRKHNIAHFWPKLLMKVTDLRMIGACHASRFLHMKVECPTELFPPLFLEVFED

>THA_HUMAN
MEQKPSKVECGSDPEENSARSPDGKRKRKNGQCSLKTSMSGYIPSYLDKDEQCVVCGDKATGYHYRCITC
EGCKGFFRRTIQKNLHPTYSCKYDSCCVIDKITRNQCQLCRFKKCIAVGMAMDLVLDDSKRVAKRKLIEQ
NRERRRKEEMIRSLQQRPEPTPEEWDLIHIATEAHRSTNAQGSHWKQRRKFLPDDIGQSPIVSMPDGDKV
DLEAFSEFTKIITPAITRVVDFAKKLPMFSELPCEDQIILLKGCCMEIMSLRAAVRYDPESDTLTLSGEM
AVKREQLKNGGLGVVSDAIFELGKSLSAFNLDDTEVALLQAVLLMSTDRSGLLCVDKIEKSQEAYLLAFE
HYVNHRKHNIPHFWPKLLMKEREVQSSILYKGAAAEGRPGGSLGVHPEGQQLLGMHVVQGPQVRQLEQQL
GEAGSLQGPVLQHQSPKSPQQRLLELLHRSGILHARAVCGEDDSSEADSPSSSEEEPEVCEDLAGNAASP

>THA_PIG
MEQKPSKVECGSDPEENSARSPDGKRKRKNGQCSLKTSMSGYIPSYLDKDEQCVVCGDKATGYHYRCITC
EGCKGFFRRTIQKNLHPTYSCKYDSCCVIDKITRNQCQLCRFKKCIAVGMAMDLVLDDSKRVAKRKLIEQ
NRERRRKEEMIRSLQQRPEPTPEEWDLIHVATEAHRSTNAQGSHWKQRRKFLPDDIGQSPIVSMPDGDKV
DLEAFSEFTKIITPAITRVVDFAKKLPMFSELPCEDQIILLKGCCMEIMSLRAAVRYDPESDTLTLSGEM
AVKREQLKNGGLGVVSDAIFELGKSLSAFNLDDTEVALLQAVLLMSTDRSGLLCVDKIEKSQEAYLLAFE
HYVNHRKHNIPHFWPKLLMKEREVQSSILYKGAAAEGRPGGSLGVHPEGQQLLGMHVVQGPQVRQLEQQL
GEAGSLRGPVLQHQSPKSPQQRLLELLHRSGILHARAVCGEDDSSEAGSLTSSDEDPEVCEDAAQATQPL
PEAPPRADGEGGGGGS

>THA_RAT
MEQKPSKVECGSDPEENSARSPDGKRKRKNGQCPLKSSMSGYIPSYLDKDEQCVVCGDKATGYHYRCITC
EGCKGFFRRTIQKNLHPTYSCKYDSCCVIDKITRNQCQLCRFKKCIAVGMAMDLVLDDSKRVAKRKLIEQ
NRERRRKEEMIRSLQQRPEPTPEEWDLIHVATEAHRSTNAQGSHWKQRRKFLPDDIGQSPIVSMPDGDKV
DLEAFSEFTKIITPAITRVVDFAKKLPMFSELPCEDQIILLKGCCMEIMSLRAAVRYDPESDTLTLSGEM
AVKREQLKNGGLGVVSDAIFELGKSLSAFNLDDTEVALLQAVLLMSTDRSGLLCVDKIEKSQEAYLLAFE
HYVNHRKHNIPHFWPKLLMKEREVQSSILYKGAAAEGRPGGSLGVHPEGQQLLGMHVVQGPQVRQLEQQF
GEAGSLRGPVLQHQSPKSPQQRLLELLHRSGILHSRAVCGEDDSSEASSLSSSSSDEDTEVFEDLAGKAA
SP

>Q80Y90_MOUSE
MEQKPSKVECGSDPEENSARSPDGKRKRKNGQCPLKSSMSGYIPSYLDKDEQCVVCGDKATGYHYRCITC
EGCKGFFRRTIQKNLHPTYSCKYDSCCVIDKITRNQCQLCRFKKCIAVGMAMDLVLDDSKRVAKRKLIEQ
NRERRRKEEMIRSLQQRPEPTPEEWDLIHVATEAHRSTNAQGSHWKQRRKFLPDDIGQSPIVSMPDGDKV
DLEAFSEFTKIITPAITRVVDFAKKLPMFSELPCEDQIILLKGCCMEIMSLRAAVRYDPESDTLTLSGEM

AVKREQLKNGGLGVVSDAIFELGKSLSAFNLDDTEVALLQAVLLMSTDRSGLLCVDKIEKSQEAYLLAFE
HYVNHRKHNIPHFWPKLLMKEREVQSSILYKGAAAEGRPGGSLGVHPEGQQLLGMHVVQGPQVRQLEQQL
GEAGSLRGPVLQHQSPKSPQQRLLELLHRSGILHSRAVCGEDDSSEASSLSSSSDTEDTEVCEDQAGKAA
SP

>THA_MOUSE
MEQKPSKVECGSDPEENSARSPDGKRKRKNGQCPLKSSMSGYIPSYLDKDEQCVVCGDKATGYHYRCITC
EGCKGFFRRTIQKNLHPTYSCKYDSCCVIDKITRNQCQLCRFKKCIAVGMAMDLVLDDSKRVAKRKLIEQ
NRERRRKEEMIRSLQQRPEPTPEEWDLIHVATEAHRSTNAQGSHWKQRRKFLPDDIGQSPIVSMPDGDKV
DLEAFSEFTKIITPAITRVVDFAKKLPMFSELPCEDQIILLKGCCMEIMSLRAAVRYDPESDTLTLSGEM
AVKREQLKNGGLGVVSDAIFELGKSLSAFNLDDTEVALLQAVLLMSTDRSGLLCVDKIEKSQEAYLLAFE
HYVNHRKHNIPHFWPKLLMKEREVQSSILYKGAAAEGRPGGSLGVHPEGQQLLGMHVVQGPQVRQLEQQF
GEAGSLRGPVLQHQSPKSPQQRLLELLHRSGILHSRAVCGEDDSSEASSLSSSSDEDTEVFEDLAGKAA
SP

>THAB_PAROL
MAQWPEKEEEEQPMFGEEYTGYIPSYLEKDEPCVVCGDKATGYHYRCITCEGCKGFFRRTIQKNLHPSYS
CKYDCCCIIDKITRNQCQLCRFKKCIAVGMAMDLVLDDSKRVAKRRLIEENRERRKKEEIVKTLQNRPEP
TGAEWELIRMVTEAHRHTNAQGAQWKQKRKFLPDKIGQSPVAPTSDGDKVDLEAFSEFTKIITPAITRVV
DFAKKLPMFSEQLPCEDQIILLKGCCMEIMSLRAAMRYDPESETLTLSGEMAVKREQLKNGGLGVVSDAI
FDLGKSLAQFNLDDTEVALLQAVLLMSSDRSGLTCMDKIEKCQETYLLAFEHYINYRKHNIPHFWPKLLM
KVTDLRMIGACHASRFLHMKVECPNELFPPLFLEVFEDQEV

>Q800D8_SPAAU
MSEPAEKCSPRWKDEAMQNGYIPSYLDKDELCVVCGDKATGYHYRCITCEGCKGFFRRTIQKNLNPTYAC
KYEGKCVIDKVTRNQCQECRFKKCIAVGMATDLVLDNSKRLAKRKLIEENRERRRKEELQKTVWDRLEPT
QEEWELIRMVTEAHMATNAQGNHWKQKRKFLSAAWVKETKPEEIGQASMVNPPEGNKVDIEAFSQFTKII
TPAITRVVDFAKKLPMFCELPCEDQIILLKGCCMEIMSLRAAVRYDPESETLTLNGEMAVTRGQLKNGGL
GVVSDAIFDLGVSLSSFNLDDSEVALLQAVILLSSDRSGLSSVERIERCQEEFLLAFEHYINYRKHKVAH
FWPKLLMKVTDLRMIGACHASRFLHMKVECSTELFPPLFLEVFED

>THB_BRARE
MSEQADKCNSRWKDEAMQNGYIPSYLDKDELCVVCGDKATGYHYRCITCEGCKGFFRRTIQKNLNPTYAC
KYEGKCVIDKVTRNQCQECRFKKCIAVGMATDLVLDDSKRLAKRKLIEENRERRRREELQKTVWDRPEPT
QEEWEMIRVVTEAHMATNAQGNHWKQKRKFLPEDIGSAPIVNAPEGNKVDIEAFSQFTKIITPAITRVVD
FAKKLPMFCELPCEDQIILLKGCCMEIMSLRAAVRYDPESDTLTLNGEMAVTRGQLKNGGLGVVSDAIFD
LGVSLSSFNLDDSEVALLQAVILLSSDRPGLTSVERIERCQEEFLLAFEHYINYRKHKVAHFWPKLLMKV
TDLRMIGACHASRFLHMKVECPTELFPPLFLEVFED

>THB_PAROL
MSEPAENCSPRWKDEAIQNGYIPSYLDKDELCVVCGDKATGYHYRCITCEGCKGFFRRTIQKNLNPTYAC
KYEGKCVIDKVTRNQCQECRFKKCIAVGMATDLVLDDSKRLAKRKLIEENRERRRKEELQKTVWDRLEPT
QEEWDLIRMVTEAHMATNAQGNHWKQKRKFLSAAGVKEDKPEEIGQASMANTPEGNKVDIEAFSQFTKII
TPAITRVVDFAKKLPMFCELPCEDQIILLKGCCMEIMSLRAAVRYDPESETLTLNGEMAVTRGQLKNGGL

GVVSDAIFDLGVSLSSFNLDDSEVALLQAVILLSSDRPGLSSVERIEPCQEEFLLAFEHYINYRKHKLAH
FWPKLLMKVTDLRMIGACHASRFLHMKVECPTELFPPLFLEVFED

>ERBA_AVIER
EDTRWLDGKHKRKSSQCLVKSSMSGYIPSCLDKDEQCVVCGDKATGYHYRCITCEGCKSFFRRTIQKNLH
PTYSCTYDGCCVIDKITRNQCQLCRFKKCISVGMAMDLVLDDSKRVAKRKLIEENRERRRKEEMIKSLQH
RPSPSAEEWELIHVVTEAHRSTNAQGSHWKQRRKFLLEDIGQSPMASMLDGDKVDLEAFSEFTKIITPAI
TRVVDFAKNLPMFSELPCEDQIILLKGCCMEIMSLRAAVRYDPESETLTLSGEMAVKREQLKNGGLGVVS
DAIFDLGKSLSAFNLDDTEVALLQAVLLMSSDRTGLICVDKIEKCQESYLLAFEHYINYRKHNIPHFWSK
LLMKVADLRMIGAYHASRFLHMKVECPTELSPQEV

>Q6F3J8_CONMY
MSEQAGKCSPRWKEHELMQHGYIPSYLDKDELCVVCSDKATGYHYRCITCEGCKGFFRRTIQKNLHPTYA
CKYEGKCAIDKVTRNQCQECRFKKCIAVGMATDLVLDDSKRLAKRRLIEENRQRRRRREEVQRKVWSKPE
PTPDEWELIRAVTEAHVATNAQGNHWKQKRKFLRAVEVKESKAEDIGQAPIINAPDGSKVDLEAFSQFTQ
IITPAITRVVDFAKKLPMFCELPCEDQIILLKGCCMEIMSLRAAVRYDPESETLTLNGEMAVTRGQLKNG
GLGVVSDAIFDLGVSLSCFNLDDSEVALLQAVILMSSDRPGLTSVERIEQCQEDYLLAFEHYINYRKHKV
SYFWPKLLMKVTDLRMIGACHASRFLHMKVECPTELFPPLFLEVFED

>THB_RANCA
MPSSMSGYIPSYLDKDELCVVCGDKATGYHYRCITCEGCKGFFRRTIQKNLHPSYSCKYEGKCVIDKVTR
NQCQECRFKKCIAVGMATDLVLDDSKRLAKRKLIEENREKRRKDELQKTLVQKPEPTPEEWELIQVVTEA
HVATNAQGSHWKQKRKFLPEDIGQAPIVNAPEGGKVDLEAFSQFTKIITPAITRVVDFAKKLPMFCELPC
EDQIILLKGCCMEIMSLRAAVRYDPESETLTLNGEMAVTRGQLKNGGLGVVSDAIFDLGVSLSSFNLDDT
EVALLQAVLLMSSDRPGLSSVERIEKCQEGFLLAFEHYINYRKHNVAHFWPKLLMKVTDLRMIGACHASR
FLHMKVECPTELFPPLFLEVFED

>Q90Y21_SALSA
MSEQGDKCTTPRWKHEAMQNGYIPSYLDKDELCVVCGDKATGYHYRCITCEGCKGFFRRTIQKNLNPTYA
CKYEAKCIIDKVTRNQCQECRFKKCIAVGMATDLVLDDSKRLAKRKLIEENRERRRKEELQKTVWDRPEP
SQEEWDLIRMVTEAHMSTNAQGNHWKQKRKFLPEDIGQAPVINAPEGSKVDIEAFSQFTKIITPAITRVV
DFAKKLPMFCELPCEDQIILLKGCCMEIMSLRAAVRYDPESETLTLNGEMAVTRGQLKNGGLGVVSDAIF
DLGLSLSSFHLDDSEVALLQTVILLSSDRPGLTSVDRIERCQEEFLLAFEHYINYRKHKVAQFWPKLLMK
VTDLRMIGACHASRFLHMKVECPNELFPPLFLEVFED

>Q90Y22_SALSA
MSEQGDKCTTPRWKHEAMQNGYIPSYLDKDELCVVCGDKATGYHYRCITCEGCKGFFRRTIQKNLNPTYA
CKYEAKCIIDKVTRNQCQECRFKKCIAVGMATDLVLDDSKRLAKRKLIEENRERRRKEELQKTVWDRPEP
SQEEWDLIRMVTEAHMSTNAQGNHWKQKRKFLSAVGVKETKPEDIGQAPVINAPEGSKVDIEAFSQFTKI
ITPAITRVVDFAKKLPMFCELPCEDQIILLKGCCMEIMSLRAAVRYDPESETLTLNGEMAVTRGQLKNGG
LGVVSDAIFDLGLSLSSFHLDDSEVALLQTVILLSSDRPGLTSVDRIERCQEEFLLAFEHYINYRKHKVA
QFWPKLLMKVTDLRMIGACHASRFLHMKVECPNELFPPLFLEVFED

>THBA_XENLA

MPSSMSGYIPSYLDKDELCVVCGDKATGYHYRCITCEGCKGFFRRTIQKNLHPSYSCKYEGKCVIDKVTR
NQCQECRFKKCIAVGMATDLVLDDNKRLAKRKLIEENREKRRKDEIQKSLVQKPEPTQEEWELIQVVTEA
HVATNAQGSHWKQKRKFLPEDIGQAPIVNAPEGGKVDLEAFSQFTKIITPAITRVVDFAKKLPMFCELPC
EDQIILLKGCCMEIMSLRAAVRYDPESETLTLNGEMAVTRGQLKNGGLGVVSDAIFDLGVSLSSFSLDDT
EVALLQAVLLMSSDRPGLASVERIEKCQEGFLLAFEHYINYRKHNIAHFWPKLLMKVTDLRMIGACHASR
FLHMKVECPTELFPPLFLEVFED

>THB_CHICK
MSGYIPSYLDKDELCVVCGDKATGYHYRCITCEGCKGFFRRTIQKNLHPTYSCKYEGKCVIDKVTRNQCQ
ECRFKKCIFVGMATDLVLDDSKRLAKRKLIEENREKRRREELQKTIGHKPEPTDEEWELIKIVTEAHVAT
NAQGSHWKQKRKFLPEDIGQAPIVNAPEGGKVDLEAFSQFTKIITPAITRVVDFAKKLPMFCELPCEDQI
ILLKGCCMEIMSLRAAVRYDPESETLTLNGEMAVTRGQLKNGGLGVVSDAIFDLGMSLSSFNLDDTEVAL
LQAVLLMSSDRPGLVCVERIEKCQEGFLLAFEHYINYRKHHVAHFWPKLLMKVTDLRMIGACHASRFLHM
KVECPTELFPPLFLEVFED

>THB_CAIMO
MSGYIPSYLDKDELCVVCGDKATGYHYRCITCEGCKGFFRRTIQKNLHPTYSCKYEGKCVIDKVTRNQCQ
ECRFKKCIFVGMATDLVLDDSKRLAKRKLIEENREKRRREELQKTIGHKPEPTDEEWELIKIVTEAHVAT
NAQGSHWKQKRKFLPEDIGQAPIVNAPEGGKVDLEAFSQFTKIITPAITRVVDFAKKLPMFCELPCEDQI
ILLKGCCMEIMSLRAAVRYDPESETLTLNGEMAVTRGQLKNGGLGVVSDAIFDLGMSLSSFNLDDTEVAL
LQAVLLMSSDRPGLVCVERIEKCQEGFLLAFEHYINYRKHHVAHFWPKLLMKVTDLRMIGACHASRFLHM
KVECPTELFPPLFLEVFED

>Q7T0K3_AMBME
MPISMSGYIPSYLDKDELCVVCGDKATGYHYRCITCEGCKGFFRRTIQKNLHPTYSCKYEGKCVIDKVTR
NQCQECRFRKCTYVGMATDLVLDDSKRLAKRKLIEENRQKRRRDELQKTLIHRPEPNLEEWELIHMVTDA
HMATNAQGSHWKQKRKFLPEDIGQAPIMNAPDGGKVDLEAFSQFTKIITPAITRVVDFAKKLPMFSELPC
EDQIILLKGCCMEIMSLRAAVRYDPESKTLTLNGEMAVTRDQLKNGGLGVVSDAIFDLGMSLSSFNLDDT
EVALLQAVLLMSSDRPGLLNVEQIEKFQEGFLLAFEHYINYRKHNVAHFWPKLLMKVTDLRMIGACHASR
FLHMKVECPTELFPPLFLEVFED

>Q6Y9T0_NECMA
MPISMSGYIPSYLDKDELCVVCGDKATGYHYRCITCEGCKGFFRRTIQKNLHPTYSCKYEGKCVIDKVTR
NQCQECRFKKCTHVGMATDLVLDDGKRLAKRKLIEDNREKRRRDELQKTLIDRPEPTSEEWGLIQIVKEA
HMATNAEGSQWKQKRIFLPKDIGQAPIVNAPESGKVDLEAFSQFTKIITPAITRVVDFAKKLPMFCELPC
EDQIILLKGCCMEIMSLRAAVRYDPESKTLTLNGEMAVTRDQLKNGGLGVVSDAIFDLGMSLSSFNLDDT
EVALLQAVLLMSSDRPGLLSVERIEKFQEGFLLAFEHYINFRKHNVTHFWPKLLMKVTDLRMIGACHASR
FLHMKVECPTELFPPLFLEVFED

>Q766D1_ORYLA
MSGYIPSYLDKDELCVVCGDKATGYHYRCITCEGCKGFFRRTIQKNLNPTYACKYEGKCVIDKVTRNQCQ
ECRFKKCIAVGMATDLVLDDGKRLAKRKLIEENRERRRKEELQKTAWDRLEPTQEEWDLIRLVTEAHTTT
NAQGSHWKQKRKFLSQAGGKETKPEDFGQSSVVSSPDGNKVDIEAFSQFTKIITPAITRVVDFAKKLPMF
CELPCEDQIILLKGCCMEIMSLRAAVRYDPESETLTLNGEMAVTRDQLKNGGLGVVSDAIFDLGVSLSSF

NLDDSEVALLQAVILLSSDRPGLSSTDRIERCQEEFLLAFEHYINHRKHKVAHFWPKLLMKVTDLRMIGA
CHASRFLHLKVECPTELFPPLFLEVFED

>P97513_MUSSP
MASNSSSCPTPGGGHLNGYPVPPYAFFFPSMLGGLSPPGALTSLQHQLPVSGYSTPSPATIETQSSSSEE
IVPSPPSPPPLPRIYKPCFVCQDKSSGYHYGVSACEGCKGFFRRSIQKNMVYTCHRDKNCIINKVTRNRC
QYCRLQKCFEVGMSKESVRNDRNKKKKEAPKPECSESYTLTPEVGELIEKVRKAHQETFPALCQLGKYTT
NNSSEQRVSLDIDLWDKFSELSTKCIIKTVEFAKQLPGFTTLTIADQITLLKAACLDILILRICTRYTPE
QDTMTFSDGLTLNRTQMHNAGFGPLTDLVFAFANQLLPLEMDDAETGLLSAICLICGDRQDLEQPDKVDM
LQEPLLEALKVYVRKRRPSRPHMFPKMLMKITDLRSISAKGAERVITLKMEIPGSMPPLIQEMLENSEGL
DTLSGQSGGGTRDGGGLAPPPGSCSPSLSPSSHRSSPATQSP

>RRA_MOUSE
MASNSSSCPTPGGGHLNGYPVPPYAFFFPPMLGGLSPPGALTSLQHQLPVSGYSTPSPATIETQSSSSEE
IVPSPPSPPPLPRIYKPCFVCQDKSSGYHYGVSACEGCKGFFRRSIQKNMVYTCHRDKNCIINKVTRNRC
QYCRLQKCFDVGMSKESVRNDRNKKKKEAPKPECSESYTLTPEVGELIEKVRKAHQETFPALCQLGKYTT
NNSSEQRVSLDIDLWDKFSELSTKCIIKTVEFAKQLPGFTTLTIADQITLLKAACLDILILRICTRYTPE
QDTMTFSDGLTLNRTQMHNAGFGPLTDLVFAFANQLLPLEMDDAETGLLSAICLICGDRQDLEQPDKVDM
LQEPLLEALKVYVRKRRPSRPHMFPKMLMKITDLRSISAKGAERVITLKMEIPGSMPPLIQEMLENSEGL
DTLSGQSGGGTRDGGGLAPPPGSCSPSLSPSSHRSSPATQSP

>Q6F3J7_CONMY
MSGYIPSYLDKDELCVVCGDRATGYHYRCITCEGCKGFFRRTIQKNLNPTYACKYEAKCVIDKVTRNQCQ
ECRFRKCIAVGMATDLVLDDSKRLAKRRLIEENRERRRREELQRSVWEKPEPSPQEWDLIHLVTEAHMAT
NAQGNHWKQKRKFLRDVCVPECKPEDLGHPPILSAPEGNKVDIEAFSQFTKIITPAITRVVDFAKKLPMF
CELPCEDQIVLLKGCCMEIMSLRAAVRYDPESETLTLNGEMAVTRGQLKNGGLGVVSDAIFDLGVSLSAF
NLDDSEVALLQAVILLSSDRPGLSSVERIERCQEEFILAFEHYINYRKHKVAHFWPKLLMKVTDLRMIGA
CHASRFLHMKVECPTELFPPLFLEVFED

>RRA_HUMAN
MASNSSSCPTPGGGHLNGYPVPPYAFFFPPMLGGLSPPGALTTLQHQLPVSGYSTPSPATIETQSSSSEE
IVPSPPSPPPLPRIYKPCFVCQDKSSGYHYGVSACEGCKGFFRRSIQKNMVYTCHRDKNCIINKVTRNRC
QYCRLQKCFEVGMSKESVRNDRNKKKKEVPKPECSESYTLTPEVGELIEKVRKAHQETFPALCQLGKYTT
NNSSEQRVSLDIDLWDKFSELSTKCIIKTVEFAKQLPGFTTLTIADQITLLKAACLDILILRICTRYTPE
QDTMTFSDGLTLNRTQMHNAGFGPLTDLVFAFANQLLPLEMDDAETGLLSAICLICGDRQDLEQPDRVDM
LQEPLLEALKVYVRKRRPSRPHMFPKMLMKITDLRSISAKGAERVITLKMEIPGSMPPLIQEMLENSEGL
DTLSGQPGGGGRDGGGLAPPPGSCSPSLSPSSNRSSPATHSP

>RRA_CHICK
MASNSSSCPTPGGGHLNGYPVTPYAFFFPHMLGGLSPPSSLPGIQHQLPVSGYSTPSPATVETQSTSSEE
IVPSPPSPPPLPRIYKPCFVCQDKSSGYHYGVSACEGCKGFFRRSIQKNMVYTCHRDKNCIINKVTRNRC
QYCRLQKCFEVGMSKESVRNDRNKKKKDVPKTECSESYIVTPEVEELIEKVRKAHQETFPALCQLGKYTT
NNSSEQRVSLDIDLWDKFSELSTKCIIKTVEFAKQLPGFTTLTIADQITLLKAACLDILILRICTRYTPE
QDTMTFSDGLTLNRTQMHNAGFGPLTDLVFAFANQLLPLEMDDAETGLLSAICLICGDRQDLEQPDKVDK

LQEPLLEALKIYVRKRRPNKPHMFPKMLMKITDLRSISAKGAERVITLKMEIPGSMPPLIQEMLENSEGM
DTLGGQPGGPRTGGLGPPPGSCSPSLSPSSTRSSPATHSP

>RRG1_HUMAN
MATNKERLFAAGALGPGSGYPGAGFPFAFPGALRGSPPFEMLSPSFRGLGQPDLPKEMASLSVETQSTSS
EEMVPSSPSPPPPPRVYKPCFVCNDKSSGYHYGVSSCEGCKGFFRRSIQKNMVYTCHRDKNCIINKVTRN
RCQYCRLQKCFEVGMSKEAVRNDRNKKKKEVKEEGSPDSYELSPQLEELITKVSKAHQETFPSLCQLGKY
TTNSSADHRVQLDLGLWDKFSELATKCIIKIVEFAKRLPGFTGLSIADQITLLKAACLDILMLRICTRYT
PEQDTMTFSDGLTLNRTQMHNAGFGPLTDLVFAFAGQLLPLEMDDTETGLLSAICLICGDRMDLEEPEKV
DKLQEPLLEALRLYARRRRPSQPYMFPRMLMKITDLRGISTKGAERAITLKMEIPGPMPPLIREMLENPE
MFEDDSSQPGPHPNASSEDEVPGGQGKGGLKSPA

>Q91YX2_MOUSE
MATNKERLFAPGALGPGSGYPGAGFPFAFPGALRGSPPFEMLSPSFRGLGQPDLPKEMASLSVETQSTSS
EEMVPSSPSPPPPPRVYKPCFVCNDKSSGYHYGVSSCEGCKGFFRRSIQKNMVYTCHRDKNCIINKVTRN
RCQYCRLQKCFEVGMSKEAVRNDRNKKKKEVKEEGLPDSYELSPQLEELITKVSKAHQETFPSLCQLGKY
TTNSSADHRVQLDLGLWDKFSELATKCIIKIVEFAKRLPGFTGLSIADQITLLKAACLDILMLRICTRYT
PEQDTMTFSDGLTLNRTQMHNAGFGPLTDLVFAFAGQLLPLEMDDTETGLLSAICLICGDRMDLEEPEKV
DKLQEPLLEALRLYARRRRPSQPYMFPRMLMKITDLRGISTKGAERAITLKMEIPGPMPPLIREMLENPE
MFEDDSSKPGPHPKASSEDEAPGGQGKRGQSPQPDQGP

>RRG1_MOUSE
MATNKERLFAPGALGPGSGYPGAGFPFAFPGALRGSPPFEMLSPSFRGLGQPDLPKEMASLSVETQSTSS
EEMVPSSPSPPPPPRVYKPCFVCNDKSSGYHYGVSSCEGCKGFFRRSIQKNMVYTCHRDKNCIINKVTRN
RCQYCRLQKCFEVGMSKEAVRNDRNKKKKEVKEEGSPDSYELSPQLEELITKVSKAHQETFPSLCQLGKY
TTNSSADHRVQLDLGLWDKFSELATKCIIKIVEFAKRLPGFTGLSIADQITLLKAACLDILMLRICTRYT
PEQDTMTFSDGLTLNRTQMHNAGFGPLTDLVFAFAGQLLPLEMDDTETGLLSAICLICGDRMDLEEPEKV
DKLQEPLLEALRLYARRRDPAKPYMFPRMLMKITDLRGISTKGAERAITLKMEIPGPMPPLIREMLENPE
MFEDDSSKPGPHPKASSEDEAPGGQGKRGQSPQPDQGP

>Q9I8T3_AMBME
MYDCMEAFMLAPHPLYDVTNPGACMLRKARLSPCFGGLDPFGWPQPASLQSVETQSTSSEEMVPSSPSPP
PPPRVYKPCFVCNDKSSGYHYGVSSCEGCKGFFRRSIQKNMVYTCHRDKNCQINKVTRNRCQYCRLQKCF
EVGMSKEAVRNDRNKKKKEIKEEVVTDSYEMPPEMEALIAKVSKAHQETFPSLCQLGKYTTNSSADHRVQ
LDLGLWDKFSELATKCIIKIVEFAKRLPGFATLTIADQITLLKAACLDILMLRICTRYTPEQDTMTFSDG
LTLNRTQMHNAGFGPLTDLVFAFAEQLLPLEMDDTETGLLSAICLICGDRMDLEEPEKVDKLQEPLLEAL
KIYARRRRPNKPYMFPRMLMKITDLRGISTKGAERAITLKMEIPGPMPPLIREMLENPEAFEDDALSSPK
AEEKPIKVEEIPAEKPSSKEL

>RRB_HUMAN
MTTSGHACPVPAVNGHMTHYPATPYPLLFPPVIGGLSLPPLHGLHGHPPPSGCSTPSPATIETQSTSSEE
LVPSPPSPLPPPRVYKPCFVCQDKSSGYHYGVSACEGCKGFFRRSIQKNMIYTCHRDKNCVINKVTRNRC
QYCRLQKCFEVGMSKESVRNDRNKKKKETSKQECTESYEMTAELDDLTEKIRKAHQETFPSLCQLGKYTT
NSSADHRVRLDLGLWDKFSELATKCIIKIVEFAKRLPGFTGLTIADQITLLKAACLDILILRICTRYTPE

QDTMTFSDGLTLNRTQMHNAGFGPLTDLVFTFANQLLPLEMDDTETGLLSAICLICGDRQDLEEPTKVDK
LQEPLLEALKIYIRKRRPSKPHMFPKILMKITDLRSISAKGAERVITLKMEIPGSMPPLIQEMLENSEGH
EPLTPSSSGNTAEHSPSISPSSVENSGVSQSPLVQ

>RRB_CHICK
MTTSSRTCPVPAVNGHMTHYPAAPYPLLFPPVIGGLSLPSLHGLQSHPPTSGCSTPSPATVETQSTSSEE
LVPSPPSPLPPPRVYKPCFVCQDKSSGYHYGVSACEGCKGFFRRSIQKNMVYTCHRDKNCVINKVTRNRC
QYCRLQKCFEVGMSKESVRNDRNKKKKEPTKQESTENYEMTAELDDLTEKIRKAHQETFPSLCQLGKYTT
NSSADHRVRLDLGLWDKFSELATKCIIKIVEFAKRLPGFTSLTIADQITLLKAACLDILILRICTRYTPE
QDTMTFSDGLTLNRTQMHNAGFGPLTDLVFTFANQLLPLEMDDTETGLLSAICLICGDRQDLEEPMKVDK
LQEPLLEALKIYIRKRRPNKPHMFPKILMKITDLRSISAKGAERVITLKMEIPGSMPPLIQEMLENSEGH
EPLTPTSNGNTAEHSPSISPSSVDNSSVSQSPMVQ

>RRG2_HUMAN
MYDCMETFAPGPRRLYGAAGPGAGLLRRATGGSCFAGLESFAWPQPASLQSVETQSTSSEEMVPSSPSPP
PPPRVYKPCFVCNDKSSGYHYGVSSCEGCKGFFRRSIQKNMVYTCHRDKNCIINKVTRNRCQYCRLQKCF
EVGMSKEAVRNDRNKKKKEVKEEGSPDSYELSPQLEELITKVSKAHQETFPSLCQLGKYTTNSSADHRVQ
LDLGLWDKFSELATKCIIKIVEFAKRLPGFTGLSIADQITLLKAACLDILMLRICTRYTPEQDTMTFSDG
LTLNRTQMHNAGFGPLTDLVFAFAGQLLPLEMDDTETGLLSAICLICGDRMDLEEPEKVDKLQEPLLEAL
RLYARRRRPSQPYMFPRMLMKITDLRGISTKGAERAITLKMEIPGPMPPLIREMLENPEMFEDDSSQPGP
HPNASSEDEVPGGQGKGGLKSPA

>RRB_COTJA
MTTSSRTCPVPAVNGHMTHYPAAPYPLLFPPVIGGLSLPSLHGLQSHPPTSGCSTPSPASVETQSTSSEE
LVPSPPSPLPPPRVYKPCFVCQDKSSGYHYGVSACEGCKGFFRRSIQKNMVYTCHRDKNCVINKVTRNRC
QYCRLQKCFEVGMSKESVRNDRNKKKKEPTKQESTENYEMTAELDDLTEKIRKAHQETFPSLCQLGKYTT
NSSADHRVRLDLGLWDKFSELATKCIIKIVEFAKRLPGFTSLTIADQITLLKAACLDILILRICTRYTPE
QDTMTFSDGLTLNRTQMHNAGFGPLTDLVFTFANQLLPLEMDDTETGLLSAICLICGDRQDLEEPMKVDK
LQEPLLEALKIYIRKRRPNKPHMFPKILMKITDLRSISAKGAERVITLKMEIPGSMPPLIQEMLENSEGH
EPLTPTSNGNTAEHSPSISPSSVDNSSVSQSPMVQ

>RRA_NOTVI
MASNGGSCPSSGGHMNGYPVPHYAFFFPHMLGGLSPPGSLAGIPHPLPVSAYSTPSPATIETQSTSSEEI
VPSPPSPPPLPRIYKPCFVCQDKSSGYHYGVSACEGCKGFFRRSIQKNMVYTCHRDKTCIINKVTRNRCQ
YCRLQKCFEVGMSKESVRNDRNKKKKQEAPKQECTESYIITPEVEDLVEKVRKAHQETFPALCQLGKYTT
NNSSEERVSLDIDLWDKFSELSTKCIIKTVEFAKQLPGFTTLTIADQITLLKAACLDILILRICTRYTPD
QDTMTFSDGLTLNRTQMHNAGFGPLTDLVFAFANQLLPLEMDDAETGLLSAICLICGDRQDLEQPDKVDK
LQEPLLEALKIYVRKRRPNKPHMFPKMLMKITDLRSISAKGAERVITLKMEIPGSMPPLIQEMLENSEGL
DSLTGQPPRASSLAPPPGSCSPSLSPSSNRSSPTSHSP

>Q8VHB6_MESAU
MYDCMESFAPGPRRLYGAAGPGAGLLRRATGSSCFAGLESFAWAQPASLQSVETQSTSSEEMVPSSPSPP
PPPRVYKPCFVCNDKSSGYHYGVSSCEGCKGFFRRSIQKNMVYTCHRDKNCIINKVTRNRCQYCRLQKCF
EVGMSKEAVRNDRNKKKKEVKEECSPDSYELSPQLEELITKVSKAHQETFPSLCQLGKYTTNSSADHRVQ

LDLGLWDKFSELATKCIIKIVEFAKRLPGFTGLSIADQITLLKAACLDILMLRICTRYTPEQDTMTFSDG
LTLNRTQMHNAGFGPLTDLVFAFAGQLLPLEMDDTETGLLSAICLICGDRMDLEEPEKVDKLQEPLLEAL
RLYARRRRPSQPYMFPRMLMKITDLRGISTKGAERAITLKMEIPGPMPPLIREMLENPEMFEDDSSKSGP
HPKASSEDETPGGQGKVGLKPPT

>Q5U645_HUMAN
MFDCMDVLSVSPGQILDFYTASPSSCMLQEKALKACFSGLTQTEWQHRHTAQSIETQSTSSEELVPSPPS
PLPPPRVYKPCFVCQDKSSGYHYGVSACEGCKGFFRRSIQKNMIYTCHRDKNCVINKVTRNRCQYCRLQK
CFEVGMSKESVRNDRNKKKKETSKQECTESYEMTAELDDLTEKIRKAHQETFPSLCQLGKYTTNSSADHR
VRLDLGLWDKFSELATKCIIKIVEFAKRLPGFTGLTIADQITLLKAACLDILILRICTRYTPEQDTMTFS
DGLTLNRTQMHNAGFGPLTDLVFTFANQLLPLEMDDTETGLLSAICLICGDRQDLEEPTKVDKLQEPLLE
ALKIYIRKRRPSKPHMFPKILMKITDLRSISAKGAERVITLKMEIPGSMPPLIQEMLENSEGHEPLTPSS
SGNTAEHSPSISPSSVKTVGSVSHHSCNKTFSSYFKHSPVPSVPGFKMQEKTFLLLLSFWTEKILKLKKD
QEVFICINIYTPHCVTYLEIQTFPILKNQPFHATRN

>RRG2_MOUSE
MYDCMESFVPGPRRLYGAAGPGAGLLRRATGSSCFAGLESFAWAQPASLQSVETQSTSSEEMVPSSPSPP
PPPRVYKPCFVCNDKSSGYHYGVSSCEGCKGFFRRSIQKNMVYTCHRDKNCIINKVTRNRCQYCRLQKCF
EVGMSKEAVRNDRNKKKKEVKEEGSPDSYELSPQLEELITKVSKAHQETFPSLCQLGKYTTNSSADHRVQ
LDLGLWDKFSELATKCIIKIVEFAKRLPGFTGLSIADQITLLKAACLDILMLRICTRYTPEQDTMTFSDG
LTLNRTQMHNAGFGPLTDLVFAFAGQLLPLEMDDTETGLLSAICLICGDRMDLEEPEKVDKLQEPLLEAL
RLYARRRDPAKPYMFPRMLMKITDLRGISTKGAERAITLKMEIPGPMPPLIREMLENPEMFEDDSSKPGP
HPKASSEDEAPGGQGKRGQSPQPDQGP

>Q9I8T2_AMBME
MYDCMEAFMLAPHPLYDVTNPGACMLRKARLSPCFGGLDPFGWPQPASLQSVETQSTSSEEMVPSSPSPP
PPPRVYKPCFVCNDKSSGYHYGVSSCEGCKGFFRRSIQKNMVYTCHRDKNCQINKVTRNRCQYCRLQKCF
EVGMSKEVVAAAAVRNDRNKKKKEIKEEVVTDSYEMPPEMEALIAKVSKAHQETFPSLCQLGKYTTNSSA
DHRVQLDLGLWDKFSELATKCIIKIVEFAKRLPGFATLTIADQITLLKAACLDILMLRICTRYTPEQDTM
TFSDGLTLNRTQMHNAGFGPLTDLVFAFAEQLLPLEMDDTETGLLSAICLICGDRMDLEEPEKVDKLQEP
LLEALKIYARRRRPNKPYMFPRMLMKITDLRGISTKGAERAITLKMEIPGPMPPLIREMLENPEAFEDDA
LSSPKAEEKPIKVEEIPAEKPSSKEL

>Q9QWJ1_RAT
MYESVEVGGLTPAPNPFLVVDFYNQNRACLLQEKGLPAPGPYSTPLRTPLWNGSNHSIETQSSSSEEIVP
SPPSPPPLPRIYKPCFVCQDKSSGYHYGVSACEGCKGFFRRSIQKNMVYTCHRDKNCIINKVTRNRCQYC
RLQKCFEVGMSKESVRNDRNKKKKETPKPECSESYTLTPEVGELIEKVRKANQETFPALCQLGKYTTNNS
SEQRVSLDIDLWDKFSELSTKCIIKTVEFAKQLPGFTTLTIADQITLLKAACLDILILRICTRYTPEQDT
MTFSDGLTLNRTQMHNAGFGPLTDLVFAFANQLLPLEMDDAETGLLSAICLICGDRQDLEQPDKVDMLQE
PLLEALKVYVRKRRPSQPHMFPKMLMKITDLRSISAKGAERVITLKMEIPGSMPPLIQEMLENSEGLDTL
SGQSGGGTRDGGGLAPPPGSCSPSLSPSSHRSSPATQSP

>Q8VHB8_MESAU
MPSLRAPQAMPPPSCLAGALLRGPSPATIETQSSSSEEIVPSPPSPPPLPRIYKPCFVCQDKSSGYHYGV

SACEGCKGFFRRSIQKNMVYTCHRDKNCIINKVTRNRCQYCRLQKCFEVGMSKESVRNDRNKKKKEAAKP
ECSESYTLTPEVGELIEKVRKAHQETFPALCQLGKYTTNNSSEQRVSLDIDLWDKFSELSTKCIIKTVEF
AKQLPGFTTLTIADQITLLKAACLDILILRICTRYTPEQDTMTFSDGLTLNRTQMHNAGFGPLTDLVFAF
ANQLLPLEMDDAETGLLSAICLICGDRQDLEQPDKVDMLQEPLLEALKVYVRKRRPSRPHMFPKMLMKIT
DLRSISAKGAERVITLKMEIPGSMPPLIQEMLENSEGLDTLSGQSGGGTRDGGGLAPPPGSCSPSLSPSS
HRSSPATQSP

>Q8VHB7_MESAU
MFDCMDVLSVSPGQILDFYTASPSSCMLQEKALKACLSGFTQAEWQHRHTAQSIETQSTSSEELVPSPPS
PLPPPRVYKPCFVCQDKSSGYHYGVSACEGCKGFFRRSIQKNMIYTCHRDKNCVINKVTRNRCQYCRLQK
CFEVGMSKESVRNDRNKKKKEPSKQECTESYEMTAELDDLTEKIRKAHQETFPSLCQLAKYTTNSSADHR
VRLDLGLWDKFSELATKCIIKIVEFAKRLPGFTSLTIADQITLLKAACLDILILRICTRYTPEQDTMTFS
DGLTLNRTQMHNAGFGPLTDLVFTFANQLLPLEMDDTETGLLSAICLICGDRQDLEEPTKVDKLQEPLLE
ALKIYIRKRRPSKPHMFPKILMKITDLRSISAKGAERVITLKMEIPGSMPPLIQEMLENSEGHEPLTPSS
SGNTAEHSPSVSPSSVENSGVSQSPLLQ

>RRG_XENLA
MANSSKERLCGAGAPLGHANGFPPSVYPFAFSGGIRRSPPFEVLANGGFFRSFPTDLPKEMASLSLTMGA
AERSAHSDCISTVETQSTSSEEMVPSSPSPPPPPRVYKPCFVCNDKSSGYHYGVSSCEGCKGFFRRSIQK
NMVYTCHRDKNCQINKVTRNRCQFCRLQKCFQVGMSKEAVRNDRNKKKKEIKEEVVLPDSYEMPPEMEEL
IQKVSKAHQETFPSLCQLGKYTTNSSADQRVQLDLGLWDKFSELSTKCIIKIVEFAKRLPGFTTLTIADQ
ITLLKSACLDILMLRICTRYTPEQDTMTFSDGLTLNRTQMHNAGFGPLTDLVFSFADQLLPLEMDDTETG
LLSAICLICGDRMDLEEPEKVEKLQEPLLEALKFYARRRRPDKPYMFPRMLMKITDLRGISTKGAERAIT
LKLEIPGPMPPLIREMLENPEAFEDGAATPKPSERSSSESSNGSPTGEDSSGSKTP

>PPAT_PIG
MGETLGDSLIDPESDAFDTLSANISQEVTMVDTEMPFWPTNFGISSVDLSVMDDHSHSFDIKPFTTVDFS
SISTPHYEDIPFPRADPMVADYKYDLKLQDYQSAIKVEPVSPPYYSEKTQLYNKPHEEPSNSLMAIECRV
CGDKASGFHYGVHACEGCKGFFRRTIRLKLIYDRCDLNCRIHKKSRNKCQYCRFQKCLAVGMSHNAIRFG
RMPQAEKEKLLAEISSDIDQLNPESADLRALAKHLYDSYIKSFPLTKAKARAILTGKTTDKSPFVIYDMN
SLMMGEDKIKFKHITPLQEQSKEVAIRIFQGCQFRSVEAVQEITEYAKNIPGFVNLDLNDQVTLLKYGVH
EIIYTMLASLMNKDGVLISEGQGFMTREFLKSLRKPFGDFMEPKFEFAVKFNALELDDSDLAIFIAVIIL
SGDRPGLLNVKPIEDIQDNLLQALELQLKLNHPESSQLFAKLLQKMTDLRQIVTEHVQLLQVIKKTETDM
SLHPLLQEIYKDLY

>Q8HZ56_SHEEP
MVDTEMPFWPTNFGISSVDLSMMDDHSHAFDIKPFTTVDFSSISAPHYEDIPFPRADPMVADYKYDLKLQ
EYQSAIKVEPVSPPYYSEKTQLYSKPHEEPSNSLMAIECRVCGDKASGFHYGVHACEGCKGFFRRTIRLK
LIYDRCDLNCRIHKKSRNKCQYCRFQKCLAVGMSHNAIRFGRMPQAEKEKLLAEISSDIDQLNPESADLR
ALAKHLYDSYIKSFPLTKAKARAILTGKTTDKSPFVIYDMNSLMMGEDKIKFKHISPLQEPSKEVAIRIF
QGCQFRSVEAVQEITEYAKNIPGFVNLDLNDQVTLLKYGVHEIIYTMLASLMNKDGVLISEGQGFMTREF
LKSLRKPFGDFMEPKFEFAVKFNALELDDSDLAIFIAVIILSGDRPGLLNVKPIEDIQDNLLQALELQLK
LNHPESSQLFAKLLQKMTDLRQIVTEHVQLLQVIKKTETDMSLHPLLQEIYKDLY

>PPAT_BOVIN
MGETLGDALIDPESEPFAVTVSARTSQEITMVDTEMPFWPTNFGISSVDLSMMDDHSHAFDIKPFTTVDF
SSISTPHYEDIPFPRADPMVADYKYDLKLQEYQSAIKVEPVSPPYYSEKTQLYSKPHEEPSNSLMAIECR
VCGDKASGFHYGVHACEGCKGFFRRTIRLKLIYDRCDLNCRIHKKSRNKCQYCRFQKCLAVGMSHNAIRF
GRMPQAEKEKLLAEISSDIDQLNPESADLRALAKHLYDSYIKSFPLTKAKARAILTGKTTDKSPFVIYDM
NSLMMGEDKIKFKHISPLQEPSKEVAIRIFQGCQFRSVEAVQEITEYAKNIPGFVNLDLNDQVTLLKYGV
HEIIYTMLASLMNKDGVLISEGQGFMTREFLKSLRKPFGDFMEPKFEFAVKFNALELDDSDLAIFIAVII
LSGDRPGLLNVKPIEDIQDNLLQALELQLKLNHPESSQLFAKLLQKMTDLRQIVTEHVQLLQVIKKTETD
MSLHPLLQEIYKDLY

>PPAT_MACMU
MGETLGDSPIDPESDSFTDTLSANISQEITMVDTEMPFWPTNFGISSVDLSVMDDHSHSFDIKPFTTVDF
SSISAPHYEDIPFTRTDPMVADYKYDLKLQEYQSAIKVEPASPPYYSEKTQLYNKPHEEPSNSLMAIECR
VCGDKASGFHYGVHACEGCKGFFRRTIRLKLIYDRCDLNCRIHKKSRNKCQYCRFQKCLAVGMSHNAIRF
GRMPQAEKEKLLAEISSDIDQLNPESADLRALAKHLYDSYIKSFPLTKAKARAILTGKTTDKSPFVIYDM
NSLMMGEDKIKFKHITPLQEQSKEVAIRIFQGCQFRSVEAVQEITEYAKSIPGFVNLDLNDQVTLLKYGV
HEIIYTMLASLMNKDGVLISEGQGFMTREFLKSLRKPFGDFMEPKFEFAVKFNALELDDSDLAIFIAVII
LSGDRPGLLNVKPIEDIQDNLLQALELQLKLNHPESSQLFAKLLQKMTDLRQIVTEHVQLLQVIKKTETD
MSLHPLLQEIYKDLY

>RRA_FUGRU
MAGKGNPVPGPHLNGFPVPTYSYFFPHMLGSLSPPALPGLPISGYSTPSPATIETQSTSSEEIVPSPPSP
PPPPRVYKPCFVCQDKSSGYHYGVSACEGCKGFFRRSIQKNMVYTCHREKNCIINKVTRNRCQYCRLQKC
LEVGMSKESVRNDRNKKKKDEKKPECIENYVLSPDTEQMINRVRKAHQETFPSLCQLGKYTTTNSSERRV
ALDVDLWDKFSELSTKCIIKTVEFAKQLPGFVTLTIADQITLLKAACLDILILRICTRYTPEQDTMTFSD
GLTLNRTQMHNAGFGPLTDLVFAFANQLLPLEMDDAETGLLSAICLLCGDRQDLEQAEKVDILQEPLLEA
LKIYVRRRRPHKPHMFPKMLMKITDLRSISAKGAERVITLKMEIPGSMPPLIQEMLENSEGLESGATGSR
PSGAPPGSCSPSLSPSSAQSSPPTQSP

>Q95J17_MACFA
MVDTEMPFWPTNFGISSVDLSVMDDHSHSFDIKPFTTVDFSSISAPHYEDIPFTRTDPMVADYKYDLKLQ
EYQSAIKVEPASPPYYSEKTQLYNKPHEEPSNSLMAIECRVCGDKASGFHYGVHACEGCKGFFRRTIRLK
LIYDRCDLNCRIHKKSRNKCQYCRFQKCLAVGMSHNAIRFGRMPQAEKEKLLAEISSDIDQLNPESADLR
ALAKHLYDSYIKSFPLTKAKARAILTGKTTDKSPFVIYDMNSLMMGEDKIKFKHITPLQEQSKEVAIRIF
QGCQFRSVEAVQEITEYAKSIPGFVNLDLNDQVTLLKYGVHEIIYTMLASLMNKDGVFISEGQGFMTREF
LKSLRKPFGDFMEPKFEFAVKFNALELDDSDLAIFIAVIILSGDRPGLLNVKPIEDIQDNLLQALELQLK
LNHPESSQLFAKLLQKMTDLRQIVTEHVQLLQVIKKTETDMSLHPLLQEIYKDLY

>Q8MHZ0_MACFA
MKGFSEITMVDTEMPFWPTNFGISSVDLSVMDDHSHSFDIKPFTTVDFSSISAPHYEDIPFTRTDPMVAD
YKYDLKLQEYQSAIKVEPASPPYYSEKTQLYNKPHEEPSNSLMAIECRVCGDKASGFHYGVHACEGCKGF
FRRTIRLKLIYDRCDLNCRIHKKSRNKCQYCRFQKCLAVGMSHNAIRFGRMPQAEKEKLLAEISSDIDQL
NPESADLRALAKHLYDSYIKSFPLTKAKARAILTGKTTDKSPFVIYDMNSLMMGEDKIKFKHITPLQEQS
KEVAIRIFQGCQFRSVEAVQEITEYAKSIPGFVNLDLNDQVTLLKYGVHEIIYTMLASLMNKDGVFISEG

QGFMTREFLKSLRKPFGDFMEPKFEFAVKFNALELDDSDLAIFIAVIILSGDRPGLLNVKPIEDIQDNLL
QALELQLKLNHPESSQLFAKLLQKMTDLRQIVTEHVQLLQVIKKTETDMSLHPLLQEIYKDLY

>Q95KZ8_MACFA
MGETLGDSPIDPESDSFTDTLSANISQEITMVDTEMPFWPTNFGISSVDLSVMDDHSHSFDIKPFTTVDF
SSISAPHYEDIPFTRTDPMVADYKYDLKLQEYQSAIKVEPASPPYYSEKTQLYNKPHEEPSNSLMAIECR
VCGDKASGFHYGVHACEGCKGFFRRTIRLKLIYDRCDLNCRIHKKSRNKCQYCRFQKCLAVGMSHNAIRF
GRMPQAEKEKLLAEISSDIDQLNPESADLRALAKHLYDSYIKSFPLTKAKARAILTGKTTDKSPFVIYDM
NSLMMGEDKIKFKHITPLQEQSKEVAIRIFQGCQFRSVEAVQEITEYAKSIPGFVNLDLNDQVTLLKYGV
HEIIYTMLASLMNKDGVFISEGQGFMTREFLKSLRKPFGDFMEPKFEFAVKFNALELDDSDLAIFIAVII
LSGDRPGLLNVKPIEDIQDNLLQALELQLKLNHPESSQLFAKLLQKMTDLRQIVTEHVQLLQVIKKTETD
MSLHPLLQEIYKDLY

>PPAT_HUMAN
MGETLGDSPIDPESDSFTDTLSANISQEMTMVDTEMPFWPTNFGISSVDLSVMEDHSHSFDIKPFTTVDF
SSISTPHYEDIPFTRTDPVVADYKYDLKLQEYQSAIKVEPASPPYYSEKTQLYNKPHEEPSNSLMAIECR
VCGDKASGFHYGVHACEGCKGFFRRTIRLKLIYDRCDLNCRIHKKSRNKCQYCRFQKCLAVGMSHNAIRF
GRMPQAEKEKLLAEISSDIDQLNPESADLRALAKHLYDSYIKSFPLTKAKARAILTGKTTDKSPFVIYDM
NSLMMGEDKIKFKHITPLQEQSKEVAIRIFQGCQFRSVEAVQEITEYAKSIPGFVNLDLNDQVTLLKYGV
HEIIYTMLASLMNKDGVLISEGQGFMTREFLKSLRKPFGDFMEPKFEFAVKFNALELDDSDLAIFIAVII
LSGDRPGLLNVKPIEDIQDNLLQALELQLKLNHPESSQLFAKLLQKMTDLRQIVTEHVQLLQVIKKTETD
MSLHPLLQEIYKDLY

>RRB_MOUSE
MSTSSHACPVPAVRGHMTHYPAAPYPLLFPPVIRGLSLPPLHGLHGHPPPSGCSTPSPASVGQACQRTTG
GSQFAASTKWTPSLNAAIETQSTSSEELVPSPPSPLPPPRVYKPCFVCQDKSSGYHYGVSACEGCKGFFR
RSIQKNMIYTCHRDKNCVINKVTRNRCQYCRLQKCFEVGMSKESVRNDRNKKKKEPSKQECTESYEMTAE
LDDLTEKIRKAHQETFPSLCQLGKYTTNSSADHRVRLDLGLWDKFSELATKCIIKIVEFAKRLPGFTGLT
IADQITLLKAACLDILILRICTRYTPEQDTMTFSDGLTLNRTQMHNAGFGPLTDLVFTFANQLLPLEMDD
TETGLLSAICLICGDRQDLEEPTKVDKLQEPLLEALKIYIRKRRPSKPHMFPKILMKITDLRSISAKGAE
RVITLKMEIPGSMPPLIQEMLENSEGHEPLTPSSSGNIAEHSPSVSPSSVENSGVSQSPLLQ

>Q866S0_CAPHI
MVDTEMPFWPTNFGISSVDLSMMDDHSHAFDIKPFTTVDFSSISAPHYEDIPFPRADPMVADYKYDLKLQ
EYQSAIKVEPVSPPYYSEKTQLYSKPHEEPSNSLMAIECRVCGDKASGFHYGVHACEGCKGFFRRTIRLK
LIYDRCDLYCRIHKKSRNKCQYCRFQKCLAVGMSHNAIRFGRMPQAEKEKLLAEISSDIDQLNPESADLR
ALAKHLYDSYIKSFPLTRAKARAILTGKTTDKSPFVIYDMNSLMMGEDKIKFKHISPLQEPSKEVAIRIF
QGCQFRSVEAVQEITEYAKNIPGFVNLDLNDQVTLLKYGVHEIIYTMLASLMNKDGVLISEGQGFMTREF
LKSLRKPFGDFMEPKFEFAVKFNALELDDSDLAIFIAVIILSGDRPGLLNVKPIEDIQDNLLQALELQLK
LNHPESSQLFAKLLQKMTDLRQIVTEHVQLLQVIKKTETDMSLHPLLQEIYKDLY

>Q5RFE5_PONPY
MTMVDTEMPFWPTNFGISSVDLSVMDDHSHSFDIKPFTTVDFSSISTPHYEDIPFTRTDPMVADYKYDLK
LQEYQSAIKVEPASPPYYSEKTQLYNKPHEEPSNSLMAIECRVCGDKASGFHYGVHACEGCKGFFRRTIR

LKLIYDRCDLNCRIHKKSRNKCRYCRFQKCLAVGMSHNAIRFGRMPQAEKEKLLAEISSDIDQLNPESAD
LRALAKHLYDSYIKSFPLTKAKARAILTGKTTDKSPFVIYDMNSLMMGEDKIKFKHITPLQEQSKEVAIR
IFQGCQFRSVEAVQEITEYAKSIPGFVNLDLNDQVTLLKYGVHEIIYTMLASLMNKDGVLISEGQGFMTR
EFLKSLRKPFGDFMEPKFEFAVKFNALELDDSDLAIFIAVIILSGDRPGLLNVKPIEDIQDNLLQALELQ
LKLNHPESSQLFAKLLQKMTDLRQIVTEHVQLLQVIKKTETDMSLHPLLQEIYKDLY

>PPAT_RABIT
MVDTEMPFWPTNFGIGSVDLSVMDDHSHSFDIKPFTTVDFSSISAPHYEDLPFARADPMVADYKYDLKLQ
EYQSAIKVEPASPPYYSEKTQLYNKTHEEPSNSLMAIECRVCSDKASGFHYGVHACEGCKGFFRRTIRLK
LIYDRCDLNCRIHKKSRNKCQYCRFQKCLAVGMSHNAIRFGRMPQAEKEKLLAEISSDIDQLNPESADLR
ALAKHLYDSYIKSFPLTKAKARAILTGKTTDKSPFVIYDMNSLMMGEDKIKFKHITPLQEQSKEVAIRIF
QGCQFRSVEAVQEITEYAKNIPGFVSLDLNDQVTLLKYGVHEIIYTMLASLMNKDGVLISEGQGFMTREF
LKSLRKPFGDFMEPKFEFAVKFNALELDDSDLAIFIAVIILSGDRPGLLNVKPIEDIQDNLLQALELQLK
LNHPEASQLFAKLLQKMTDLRQIVTEHVQLLQVIKKTETDMSLHPLLQEIYKDLY

>Q92019_XENLA
MYENVDVSPTHYHMMDFYSHNRQCLWPEKRINPYGTPLGTQHWSSSNHSIETQSTSSEEIVPSPPSPPPL
PRIYKPCFVCQDKSSGYHYGVSACEGCKGFFRRSIQKNMVYTCHRDKNCIINKVTRNRCQYCRLQKCFEV
GMSKESVRNDRNKKKKEPPKVDVMESYVLSPETQDLIEKVRKAHKETFPALCQLGKYTTNFSSEERVSLD
IDLWDKFSELSTKCIIKTVEFAKQLPGFTTLTIADQITLLKSACLDILILRICTRYTPDQDTMTFSDGLT
LNRTQMHNAGFGPLTDLVFAFANQLLPLEMDDAETGLLSAICLICGDRQDLEQPDKVDKLQEPLLEALKI
YVRNRRPKKPHMFPKMLMKITDLRSISAKGAERVITLKMEIPGSMPPLIQEMLENSEGLDTLGGGASSQT
PVTPVAPGSCSPSLSPSSTHSSPSSHSP

>Q8JHU6_ANAPL
MVDTEMPFWPTNFGIGSVDLSVMDDHSHSFDTKPFTTVDFSSISAPHYEDLPFARADPMVADYKYDLKLQ
EYQSAIKVEPASPPYYSEKTQLYNKTHEEPSNSLMAIECRVCGDKASGFHYGVHACEGCKGFFRRTIRLK
LIYDRCDLNCRIHKKSRNKCQYCRFQKCLAVGMSHNAIRFGRMPQAEKEKLLAEISSDIDQLNPESADLR
ALAKHLYDSYIKSFPLTKAKARAILTGKTTDKSPFVIYDMNSLMMGEDKIKFKHITPLQEQSKEVAIRIF
QGCQFRSVEAVQEITEYAKNIPGFVSLDLNDQVTLLKYGVHEIIYTMLASLMNKDGVLISEGQGFMTREF
LKSLRKPFGDFMEPKFEFAVRFNALELDDSDLAIFIAVIILSGDRPGLLNVKPIEDIQDNLLQALELQLK
LNHPESSQLFAKVLQKMTDLRQIVTEHVQLLHVIKKTETDMSLHPLLQEIYKDLY

>Q8HXA4_RABIT
MVDTEMPFWPTNFGIGSVDLSVMDDHSHSFDIKPFTTVDFSSISAPHYEDLPFARADPMVADYKYDLKLQ
EYQSAIKVEPASPPYYSEKTQLYNKTHEEPSNSLMAIECRVCGDKASGFHYGVHACEGCKGFFRRPIRLK
LIYDRCDLNCRIHKKSRNKCQYCRFQKCLAVGMSHNAIRFGRMPGAEKEKLLAEISSDIDQLNPESADLR
ALAKHLYDSYIKSFPLTKAKARAILTGKTTDKSPFVIYDMNSLMMGEDKIKFKHITPLQEQSKEVAIRIF
QGCQFRSVEAVQEITEYAKNIPGFVSLDLNDQVTLLKYGVHEIIYTMLASLMNKDGVLISEGQGFMTREF
LKSLRKPFGDFMEPKFEFAVKFNALELDDSDLAIFIAVIILSGDRPGLLNVKPIEDIQDNLLQALELQLK
LNHPEASQLFAKLLQKMTDLRQIVTEHVQLLQVIKKTETDMSLHPLLQEIYKDLY

>PPAT_RAT
MGETLGDPPVDPEHGAFADALPMSTSQEITMVDTEMPFWPTNFGISSVDLSVMDDHSHSFDIKPFTTVDF

SSISAPHYEDIPFTRADPMVADYKYDLKLQEYQSAIKVEPASPPYYSEKTQLYNRPHEEPSNSLMAIECR
VCGDKASGFHYGVHACEGCKGFFRRTIRLKLIYDRCDLNCRIHKKSRNKCQYCRFQKCLAVGMSHNAIRF
GRMPQAEKEKLLAEISSDIDQLNPESADLRALAKHLYDSYIKSFPLTKAKARAILTGKTTDKSPFVIYDM
NSLMMGEDKIKFKHITPLQEQSKEVAIRIFQGCQFRSVEAVQEITEYAKNIPGFINLDLNDQVTLLKYGV
HEIIYTMLASLMNKDGVLISEGQGFMTREFLKSLRKPFGDFMEPKFEFAVKFNALELDDSDLAIFIAVII
LSGDRPGLLNVKPIEDIQDNLLQALELQLKLNHPESSQLFAKVLQKMTDLRQIVTEHVQLLHVIKKTETD
MSLHPLLQEIYKDLY

>PPAT_CRIGR
MVDTEMPFWPTNFGISSVDLSMMDDHSHSFDIKPFTTVDFSSISAPHYEDIPFTRADPMVADYKYDLKLQ
EYQSAIKVEPASPPYYSEKAQLYNRPHEEPSNSLMAIECRVCGDKASGFHYGVHACEGCKGFFRRTIRLK
LIYDRCDLNCRIHKKSRNKCQYCRFQKCLAVGMSHNAIRFGRMPQAEKEKLLAEISSDIDQLNPESADLR
ALAKHLYDSYIKSFPLTKAKARAILTGKTTDKSPFVIYDMNSLMMGEDKIKFKHITPLQEQSKEVAIRIF
QGCQFRSVEAVQEITEYAKNIPGFINLDLNDQVTLLKYGVHEIIYTMLASLMNKDGVLISEGQGFMTREF
LKSLRKPFGDFMEPKFEFAVKFNALELDDSDLAIFIAVIILSGDRPGLLNVKPIEDIQDNLLQALELQLK
LNHPESSQLFAKVLQKMTDLRQIVTEHVQLLHVIKKTETDMSLHPLLQEIYKDLY

>PPAT_MOUSE
MGETLGDSPVDPEHGAFADALPMSTSQEITMVDTEMPFWPTNFGISSVDLSVMEDHSHSFDIKPFTTVDF
SSISAPHYEDIPFTRADPMVADYKYDLKLQEYQSAIKVEPASPPYYSEKTQLYNRPHEEPSNSLMAIECR
VCGDKASGFHYGVHACEGCKGFFRRTIRLKLIYDRCDLNCRIHKKSRNKCQYCRFQKCLAVGMSHNAIRF
GRMPQAEKEKLLAEISSDIDQLNPESADLRALAKHLYDSYIKSFPLTKAKARAILTGKTTDKSPFVIYDM
NSLMMGEDKIKFKHITPLQEQSKEVAIRIFQGCQFRSVEAVQEITEYAKNIPGFINLDLNDQVTLLKYGV
HEIIYTMLASLMNKDGVLISEGQGFMTREFLKNLRKPFGDFMEPKFEFAVKFNALELDDSDLAIFIAVII
LSGDRPGLLNVKPIEDIQDNLLQALELQLKLNHPESSQLFAKVLQKMTDLRQIVTEHVQLLHVIKKTETD
MSLHPLLQEIYKDLY

>Q7ZTI3_BRARE
MYESVDVVGLTPSPNPFLSMDYYHQNRGCLIPDKGLVSGAARGFRNPHWSGSNHSVETQSTSSEEIVPSP
PSPPPPPRVYKPCFVCQDKSSGYHYGVSACEGCKGFFRRSIQKNMVYTCHREKSCIINKVTRNRCQYCRL
QKCLEVGMSKESVRNDRNKRKKDDKKQECLENYVLSPDTEKMIEQVRKAHQETFPSLCQLGKYTTNNSAD
HRVALDVDLWDKFSELSTKCIIKTVEFAKQLPGFTTLTIADQITLLKAACLDILILRICTRYTPDQDTMT
FSDGLTLNRTQMHNAGFGPLTDLVFAFANQLLPLEMDDAETGLLSAICLLCGDRQDLEQSDKVDELQEPL
LEALKIYVRNRRPHKPHMFPKMLMKITDLRSISAKGAERVITLKMEIPGSMPPLIQEMLENSEGLEGGGS
KGAGGGGGGGGGKGAPPGSCSPSLSPSSAHSSPSAHSP

>Q90272_BRARE
MYESVDVVGLTPSPNPFLSMDYYHQNRGCLIPDKGLVSGAARGFRNPHWSGSNHSVETQSTSSEEIVPSP
PSPPPPPRVYKPCFVCQDKSSGYHYGVSACEGCKGFFRRTIQKNMVYTCHREKSCIINKVTRNRCQYCRL
QKCLEVGMSKESVRNDRNKRKKDDKKQECLENYVLSPDTEKMIEQVRKAHQETFPSLCQLGKYTTNNSAD
HRVALDVDLWDKFSELSTKCIIKTVEFAKQLPGFTTLTIADQITLLKAACLDILILRICTRYTPDQDTMT
FSDGLTLNRTQMHNAGFGPLTDLVFAFANQLLPLEMDDAETGLLSAICLLCGDRQDLEQSDKVDELQEPL
LEALKIYVRNRRPHKPHMFPKMLMKITDLRSISAKGAERVITLKMEIPGSMPPLIQEMLENSEGLEGGGS
KGAGGGGGGGGKGAPPGSCSPSLSPSSAHSSPSAHSP

>Q91391_BRARE
MYESVDVNPFLMMDYYNQSRGCLIPDKMPHPFSSSIRHQHWSGSNHSIETQSTSSEEIVPSPPSPPPPPR
IYKPCFVCQDKSSGYHYGVSACEGCKGFFRRSIQKNMVYTCHREKNCIINKVTRNRCQYCRLQKCLEVGM
SKESVRNDRNKKKKEEKKPECTENYTLSPDTEQMIDRVRKAHQETFPSLCQLGKYTTSNSSERRVALDVD
LWDKFSELSTKCIIKTVEFAKQLPGFTTLTIADQITLLKAACLDILILRICTRYTPEQDTMTFSDGLTLN
RTQMHNAGFGPLTDLVFAFANQLLPLEMDDAETGLLSAICLLCGDRQDLEQADKVDVLQEPLLEALKIYV
RNRRPHKPHMFPKMLMKITDLRSISAKGAERVITLKMEIPGSMPPLIQEMLENSEGLESSSGAQGSRASA
TTPGSCSPSLSPNSAQSSPPTQSP

>Q90271_BRARE
MYESVDVNPFLMMDYYNQSRGCLIPDKMPHPFSSSIRHQHWSGSNHSIETQSTSSEEIVPSPPSPPPPPR
IYKPCFVCQDKSSGYHYGVSACEGCKGFFRRSIQKNMVYTCHREKNCIINKVTRNRCQYCRLQKCLEVGM
SKESVRNDRNKKKKEEKKPECTENYTLSPDTEQMIDRVRKAHQETFPSLCQLGKYTTSNSSERRVALDVD
LWDKFSELSTKCIIKTVEFAKQLPGFTTLTIADQITLLKAACLDILILRICTRYTPEQDTMTFSDGLTLN
RTQMHNAGFGPLTDLVFAFANQLLPLEMDDAETGLLSAICLLCGDRQDLEQADKVDVLQEPLLEALKIYV
RNRRPHKPHMFPKMLMKITDLRSISAKGAELLITLKMEIPGSMPPLIQEMLENSEGLESSSGAQGSRASA
TTPGSCSPSLSPNSAQSSPPTQSP

>RRG_BRARE
MFDCMEALGMGPRQLYDVTNRGACMLRKASPFYAGLDPFAWTGTASVRSVETQSTSSEEMVPSSSPSPPPP
PRVYKPCFVCQDKSSGYHYGVSSCEGCKGFFRRSIQKNMVYTCHRDKNCQINKVTRNRCQYCRLQKCFEV
GMSKEAVRNDRNKKKKDVKDEVIPPESYELSGELEELVNKVSKAHQETFPSLCQLGKYTTNSSSDHRIQL
DLGLWDKFSELSTKCIIKIVEFAKRLPGFTTLTIADQITLLKSACLDILMLRICTRYTPEQDTMTFSDGL
TLNRTQMHNAGFGPLTDLVFAFAGQLLPLEMDDTETGLLSAICLICGDRMDLEEPERVDRLQEPLLEALK
IYARRRRPNKPHMFPRMLMKITDLRGISTKGAERAITLKMEIPGPMPPLIREMLENPEAFEDQSEATEKK
PEPEPPAPPPPALLTMKKEQEDEDDSWATENGSEPSPEEEDDDDEDGEEERGTDSDGEAWGGQEPNADVS
RKSHGGRAQ

>Q9I878_CHICK
MVDTEMPFWPVNFGISPVDLSAMDDHMHSFDIKPFTTVDFSSISSPHYEDIPLGRADQTSIDYKYDIKLQ
DCQSAIKMEPPSPPYFSEKVQLYNKPHEESSNSLMAIECRVCGDKASGFHYGVHACEGCKGFFRRTIRLK
LIYDRCDLNCRIHKKSRNKCQYCRFQKCLAVGMSHNAIRFGRMPQAEKEKLLAEISSDIDQLNPESADLR
ALAKHLYDSYIKSFPLTKAKARAILTGKTTDKSPFVIYDMNSLRMGEDQIKCKHASPLQEQNKEVAIRIF
QRCQFRSVEAVQEITEFAKNIPGFVNLDLNDQVTLLKYGVHEIIYTLLASLMNKDGVLISDGQGFMTREF
LKSLRKPFCDFMEPKFEFAVKFNALELDDSDLAIFIAVIILSGDRPGLLNVKPIEDIQDNLLQALELQLK
LNHPESSQLFAKLLQKMTDLRQIVTEHVQLLQIIKKTETDMSLHPLLQEIYKDLY

>RRA_XENLA
MSSKDNTCPPPGPGHINGFHVPHYAFFFPHMLGGMSXTGGLPGVQHQPPLSGYSTPSPATIETQSTSSEE
IVPSPPTPPPLPRIYKPCFVCQDKSSGYHYGVSACEGCKGFFRRSIQKNMVYTCHRDKNCIINKVTRNRC
QYCRLQKCFEVGMSKESVRNDRNKKKKESPKPEAIESYILSPETQDLIEKVQKAHQETFPALCQLGKYTT
SFSSEQRVSLDIDLWDKFSELSTKCIIKTVEFAKQLPGFTTLTIADQITLLKSACLDILILRICTRYTPD
QDTMTFSDGLTLNRTQMHNAGFGPLTDLVFAFANQPVPLEMDDAETGLLSAICLICGDRQDLEQPDKVDK

LQEPLLEALKIYVRTRRPQKPHMFPKMLMKITDLRTVSAKGAERVITLKMEIPGAMPLIQEMLENSEGLD
TLGGGASSDAPVTPVAPGSCSPSLSPSSTHSSPSTHSP

>Q98SF8_XENLA
MVDTEMPFWSNLNFGMNSMDMSALEDHCQPYDIKPFTTVDFSSINSHYDDILDEKTFLCRNDQSPIDYKY
DLKLQECQSSIKLEPPSPPYFSDKPQCSKAFEDTPNSFIAIECRVCGDKASGFHYGVHACEGCKGFFRRT
IRLKLIYERCDLNCRIHKKSRNKCQFCRFQKCLAVGMSHNAIRFGRMPQAEKEKLLAEISSDIDQLNPES
ADQRVLAKHLYDSYVKSFPLTKAKARAILTGRATDKTPVVIHDMNSLMMGEDQIKGQCVSPEQNKEVAIR
IFQRCQSRSAEAVREITEFAKNIPGFVSLDLNDQVTLLKYGVHEIIFTMLASLMNKDGVLVAEGQGFMTR
EFLKSLRKPFSDFMEPKFEFAIRFNSLELDDSDLAIFVAVIILSGDRPGLLNVKPIEDIQDSLLQALELQ
LKLNHPDSAQLFAKLLQKMTDLRQVVTEHVQLLQLIKKTEADMCLHPLLQEIYKDLY

>Q6PA31_XENLA
MVDTEMPFWSNLNFGMNSMDMSALEDHCQPYDIKPFTTVDFSSINSHYDDILDEKTFLCRNDQSPIDYKY
DLKLQECQSSIKLEPPSPPYFSDKPQCSKAFEDTPNSFIAIECRVCGDKASGFHYGVHACEGCKGFFRRT
IRLKLIYERCDLNCRIHKKSRNKCQFCRFQKCLAVGMSHNAIRFGRMPQAEKEKLLAEISSDIDQLNPES
ADQRVLAKHLYDSYVKSFPLTKAKARAILTGRATDKTPVVIHDMNSLMMGEDQIKGQCVTPEQNKEVAIR
IFQRCQSRSAEAVREITEFAKNIPGFVSLDLNDQVTLLKYGVHEIIFTMLASLMNKDGVLVAEGQGFMTR
EFLKSLRKPFSDFMEPKFEFAIRFNSLELDDSDLAIFVAVIILSGDRPGLLNVKPIEDIQDSLLQALELQ
LKLNHPDSAQLFAKLLQKMTDLRQVVTEHVQLLQLIKKTEADMCLHPLLQEIYKDLY

>NR1D2_MOUSE
MELNAGGVIAYISSSSSASSPASCHSEGSENSFQSSSSSVPSSPNSSNCDANGNPKNADISSIDGVLKSD
RTDCPVKTGKTSAPGMTKSHSGMTKFSGMVLLCKVCGDVASGFHYGVHACEGCKGFFRRSIQQNIQYKKC
LKNENCSIMRMNRNRCQQCRFKKCLSVGMSRDAVRFGRIPKREKQRMLIEMQSAMKTMMNTQFSGHLQND
TLAEQHDQSALPAQEQLRPKSQLEQENIKNTPSDFAKEEVIGMVTRAHKDTFLYNQEHRENSSESMPPQR
GERIPRNMEQYNLNQDHRGSGIHNHFPCSERQQHLSGQYKGRNIMHYPNGHAVCIANGHCMNFSSAYTQR
VCDRIPVGGCSQTENRNSYLCNTGGRMHLVCPMSKSPYVDPQKSGHEIWEEFSMSFTPAVKEVVEFAKRI
PGFRDLSQHDQVNLLKAGTFEVLMVRFASLFDAKERTVTFLSGKKYSVDDLHSMGAGDLLSSMFEFSEKL
NALQLSDEEMSLFTAVVLVSADRSGIENVNSVEALQETLIRALRTLIMKNHPNEASIFTKLLLKLPDLRS
LNNMHSEELLAFKVHP

>Q8C598_MOUSE
MELNAGGVIAYISSSSSASSPASCHSEGSENSFQSSSSSVPSSPNSSNCDANGNPKNADISSIDGVLKSD
RTDCPVKTGKTSAPGMTKSHSGMTKFSGMVLLCKVCGDVASGFHYGVHACEGCKGFFRRSIQQNIQYKKC
LKNENCSIMRMNRNRCQQCRFKKCLSVGMSRDAVRFGRIPKREKQRMLIEMQSAMKTMMNTQFSGHLQND
TLAEQHDQSALPAQEQLRPKSQLEQENIKNTPSDFAKEEVIGMVTRAHKDTFLYNQEQRENSSESMPPQR
GERIPRNMEQYNLNQDHRGSGIHNHFPCSERQQHLSGQYKGRNIMHYPNGHAVCIANGHCMNFSSAYTQR
VCDRIPVGGCSQTENRNSYLCNTGGRMHLVCPMSKSPYVDPQKSGHEIWEEFSMSFTPAVKEVVEFAKRI
PGFRDLSQHDQVNLLKAGTFEVLMVRFASLFDAKERTVTFLSGKKYSVDDLHSMGAGDLLSSMFEFSEKL
NALQLSDEEMSLFTAVVLVSADRSGIENVNSVEALQETLIRALRTLIMKNHPNEASIFTKLLLKLPDLRS
LNNMHSEELLAFKVHP

>Q7TNH1_MESAU

MVDTESPICPLSPLEADDLESPLSEEFLQEMGNIQDISQSLEEESSGSFSFTDYQYLGSCPGSEGSVITD
TLSPASSPSSVSCPVIPASTDESPGSALNIECRICGDKASGYHYGVHACEGCKGFFRRTIRLKLAYDKCD
RSCKIQKKNRNKCQYCRFHKCLSVGMSHNAIRFGRMPRSEKAKLKAEILTCEHDLEDSETADLKSLAKRI
HEAYLKNFNMNKVKARVILAGKTSNNPPFVIHDMETLCMAEKTLVAKMVANGIQNKEAEVRIFHCCQCMS
VETVTELTEFAKAIPGFANLDLNDQVTLLKYGVYEAIFTMLSSLMNKDGMLIAYGNGFITREFLKNLRKP
FCDILEPKFDFAMKFNALELDDSDISLFVAAIICCGDRPGLLNIGYIEKMQEGIVHVLKLHLQSNHPDDT
FLFPKLLQKMVDLRQLVTEHAQLVQVIKKTESDAALHPLLQEIYRDMY

>PPAS_MOUSE
MEQPQEETPEAREEEKEEVAMGDGAPELNGGPEHTLPSSSCADLSQNSSPSSLLDQLQMGCDGASGGSLN
MECRVCGDKASGFHYGVHACEGCKGFFRRTIRMKLEYEKCDRICKIQKKNRNKCQYCRFQKCLALGMSHN
AIRFGRMPEAEKRKLVAGLTASEGCQHNPQLADLKAFSKHIYNAYLKNFNMTKKKARSILTGKSSHNAPF
VIHDIETLWQAEKGLVWKQLVNGLPPYNEISVHVFYRCQSTTVETVRELTEFAKNIPNFSSLFLNDQVTL
LKYGVHEAIFAMLASIVNKDGLLVANGSGFVTHEFLRSLRKPFSDIIEPKFEFAVKFNALELDDSDLALF
IAAIILCGDRPGLMNVPQVEAIQDTILRALEFHLQVNHPDSQYLFPKLLQKMADLRQLVTEHAQMMQWLK
KTESETLLHPLLQEIYKDMY

>PPAS_HUMAN
MEQPQEEAPEVREEEEKEEVAEAEGAPELNGGPQHALPSSSYTDLSRSSSPPSLLDQLQMGCDGASCGSL
NMECRVCGDKASGFHYGVHACEGCKGFFRRTIRMKLEYEKCERSCKIQKKNRNKCQYCRFQKCLALGMSH
NAIRFGRMPEAEKRKLVAGLTANEGSQYNPQVADLKAFSKHIYNAYLKNFNMTKKKARSILTGKASHTAP
FVIHDIETLWQAEKGLVWKQLVNGLPPYKEISVHVFYRCQCTTVETVRELTEFAKSIPSFSSLFLNDQVT
LLKYGVHEAIFAMLASIVNKDGLLVANGSGFVTREFLRSLRKPFSDIIEPKFEFAVKFNALELDDSDLAL
FIAAIILCGDRPGLMNVPRVEAIQDTILRALEFHLQANHPDAQYLFPKLLQKMADLRQLVTEHAQMMQRI
KKTETETSLHPLLQEIYKDMY

>Q99ND3_RAT
MEQPQEETPEAREEEKEEVATGDGAPELNGGPEHTLPSSSCTDLSQNSSPSSLLDQLQMGCDGASGGSLN
MECRVCGDKASGFHYGVHACEGCKGFFRRTIRMKLKYEKCDRICKIQKKNRNKCQYCRFQKCLALGMSHN
AIRFGRMPEAEKRKLVAGLTASEGCQQNPQLADLKAFSKHIYNAYLKNFNMTKKKARSILTGKSSHNAPF
IIHDIETLWQAEKGLVWKQLVNGPPPYNEISVHVFYRCQSTTVETVRELTEFAKNIPNFSSLFLNDQVTL
LKYGVHEAIFAMLASIVNKDGLLVANGSGFVTHEFLRSIRKPFSDIIEPKFEFAVKFNALELDDSDLALF
IAAIILCGDRPGLMNVPQVEAIQDTILQALEFHLQVNHPDSQYLFPKLLQKMADLRQLVTEHAQMMQWLK
KTESETLLHPLLQEIYKDMY

>PPAR_RAT
MVDTESPICPLSPLEADDLESPLSEEFLQEMGNIQEISQSLGEESSGSFSFADYQYLGSCPGSEGSVITD
TLSPASSPSSVSCPAVPTSTDESPGNALNIECRICGDKASGYHYGVHACEGCKGFFRRTIRLKLAYDKCD
RSCKIQKKNRNKCQYCRFHKCLSVGMSHNAIRFGRMPRSEKAKLKAEILTCEHDLKDSETADLKSLAKRI
HEAYLKNFNMNKVKARVILAGKTSNNPPFVIHDMETLCMAEKTLVAKMVANGVENKEAEVRFFHCCQCMS
VETVTELTEFAKAIPGFANLDLNDQVTLLKYGVYEAIFTMLSSLMNKDGMLIAYGNGFITREFLKNLRKP
FCDIMEPKFDFAMKFNALELDDSDISLFVAAIICCGDRPGLLNIGYIEKLQEGIVHVLKLHLQSNHPDDT
FLFPKLLQKMVDLRQLVTEHAQLVQVIKKTESDAALHPLLQEIYRDMY

>PPAR_CANFA

MVDTESPICPLSPLEADDLESPLSEEFLQEMGNIQEISQSIGEDSSGSFSFTEYQYLGSGPGSDGSVITD

TLSPAPSPSSVTHPAAPGGAEEPSSVALNIECRICGDRASGYHYGVHACEGCKGFFRRTIRLKLAYDKCD

RSCKIQKKNRNKCQYCRFHKCLSVGMSHNAIRFGRMPRSEKAKLKAEILTCEQDPEDAETADLKSLAKRI

YEAYLKNFNMNKVKARVILAGKASNNPPFVIHDMETLCMAEKTLVAKLVANGIQNKEAEVRIFHCCQCTS

VETVTELTEFAKSIPGFANLDLNDQVTLLKYGVYEAIFAMLSSVMNKDGMLVAYGNGFITREFLKSLRKP

FCDIMEPKFDFAMKFNALELDDSDISLFVAAIICCGDRPGLLNVGHIEKMQEGIVHVLKLHLQTNHPDNI

FLFPKLLQKMADLRQLVTEHAQLVQVIKKTESDAALHPLLQEIYRDMY

>NR1D2_RAT

MELNAGGVIAYISSSSSASSPASCHSEGSENSFQSSSSSVPSSPNSSNCDANGNPKNTDVSSIDGVLKSD

RTDCPVKTGKPGAPGMTKSHSGMTKFSGMVLLCKVCGDVASGFHYGVHACEGCKGFFRRSIQQNIQYKKC

LKNENCSIMRMNRNRCQQCRFKKCLSVGMSRDAVRFGRIPKREKQRMLIEMQSAMKTMMSTQFGGHLQSD

TLAEPHEQSVPPAQEQLRPKPQLEQENIKSTPPPSDFAKEEVIGMVTRAHKDTFLYNQEHRENSSESMPP

HRGERIPRNVEQYNLNHDHRGGGLHSHFPCSESQQHLSGQYKGRNMMHYPNGHTVCISNGHCVNFSSAYP

QRVCDRIPVGGCSQTESRNSYLCSTGGRMHLVCPMSKSPYVDPQKSGHEIWEEFSMSFTPAVKEVVEFAK

RIPGFRDLSQHDQVNLLKAGTFEVLMVRFASLFDAKERTVTFLSGKKYSVDDLHSMGAGDLLSSMFEFSE

KLNGLQLSDEEMSLFTAVVLVSADRSGIENVNSVEALQETLIRALRTLIMKNHPNEASIFTKLLLKLPDL

RSLNNMHSEELLAFKVHP

>Q6I9S1_HUMAN

MEVNAGGVIAYISSSSSASSPASCHSEGSENSFQSSSSSVPSSPNSSNSDINGNPKNGDLANIEGILKND

RIDCSMKTSKSSAPGMTKSHSGVTKFSGMVLLCKVCGDVASGFHYGVHACEGCKGFFRRSIQQNIQYKKC

LKNENCSIMRMNRNRCQQCRFKKCLSVGMSRDAVRFGRIPKREKQRMLIEMQSAMKTMMNSQFSGHLQND

TLVEHHEQTALPAQEQLRPKPQLEQENIKSSSPPSSDFAKEEVIGMVTRAHKDTFMYNQEQQENSAESMQ

PQRGERIPKNMEQYNLNHDHCGNGLSSHFPCSESQQHLNGQFKGRNIMHYPNGHAICIANGHCMNFSNAY

TQRVCDRVPIDGFSQNENKNSYLCNTGGRMHLVCPMSKSPYVDPHKSGHEIWEEFSMSFTPAVKEVVEFA

KRIPGFRDLSQHDQVNLLKAGTFEVLMVRFASLFDAKERTVTFLSGKKYSVDDLHSMGAGDLLNSMFEFS

EKLNALQLSDEEMSLFTAVVLVSADRSGIENVNSVEALQETLIRALRTLIMKNHPNEASIFTKLLLKLPD

LRSLNNMHSEELLAFKVHP

>PPAR_HUMAN

MVDTESPLCPLSPLEAGDLESPLSEEFLQEMGNIQEISQSIGEDSSGSFGFTEYQYLGSCPGSDGSVITD

TLSPASSPSSVTYPVVPGSVDESPSGALNIECRICGDKASGYHYGVHACEGCKGFFRRTIRLKLVYDKCD

RSCKIQKKNRNKCQYCRFHKCLSVGMSHNAIRFGRMPRSEKAKLKAEILTCEHDIEDSETADLKSLAKRI

YEAYLKNFNMNKVKARVILSGKASNNPPFVIHDMETLCMAEKTLVAKLVANGIQNKEAEVRIFHCCQCTS

VETVTELTEFAKAIPGFANLDLNDQVTLLKYGVYEAIFAMLSSVMNKDGMLVAYGNGFITREFLKSLRKP

FCDIMEPKFDFAMKFNALELDDSDISLFVAAIICCGDRPGLLNVGHIEKMQEGIVHVLRLHLQSNHPDDI

FLFPKLLQKMADLRQLVTEHAQLVQIIKKTESDAALHPLLQEIYRDMY

>PPAR_MOUSE

MVDTESPICPLSPLEADDLESPLSEEFLQEMGNIQEISQSIGEESSGSFGFADYQYLGSCPGSEGSVITD

TLSPASSPSSVSCPVIPASTDESPGSALNIECRICGDKASGYHYGVHACEGCKGFFRRTIRLKLVYDKCD

RSCKIQKKNRNKCQYCRFHKCLSVGMSHNAIRFGRMPRSEKAKLKAEILTCEHDLKDSETADLKSLGKRI

HEAYLKNFNMNKVKARVILAGKTSNNPPFVIHDMETLCMAEKTLVAKMVANGVEDKEAEVRFFHCCQCMS
VETVTELTEFAKAIPGFANLDLNDQVTLLKYGVYEAIFTMLSSLMNKDGMLIAYGNGFITREFLKNLRKP
FCDIMEPKFDFAMKFNALELDDSDISLFVAAIICCGDRPGLLNIGYIEKLQEGIVHVLKLHLQSNHPDDT
FLFPKLLQKMVDLRQLVTEHAQLVQVIKKTESDAALHPLLQEIYRDMY

>Q62879_RAT
MEQPQEETPEAREEEKEEVATGDGAPELNGGPEHTLPSSSCTDLSQNSSPSSLLDQLQMGCDGASGGSLN
MECRVCGDKASGFHYGVHACEGCKGFFRRTIRMKLKYEKCDRICKIQKKNRNKCQYCRFQKCLALGMSHN
AIRFGRMPEAEKRKLVAGLTASEGCQQNPQLADLKAFSKHIYNAYLKNFNMTKKKARSILTGKSSHNAPF
IIHDIETLWQAEKGLVWKQLVNGPPPYNEISVHVFYRCQSTTVETVRELTEFAKNIPNFSSLFLNDQVTL
LKYGVHEAIFAMLASIVNKDGLLVANGSGFVTHEFLRSIRKPFSDIIEPKFEFAVKFNALELVDSDLALF
IAAIILCGDRPGLMNVPQVEAIQDTILQALEFHLQVNHPDSQYLFPKLLQKMADLRQLVTEHAQMMQWLK
KTESETLLHPLLQEIYKDMY

>Q9I8W4_CHICK
MVDTENQLYPLTPLEEDDIGSPLSGEFLQDMENIQDISQSLGDDSSGALSLTEFQSLGNGPGSDGSVITD
TLSPASSPSSINFATAPGSIDESPSGAENIECRICGDKASGYHYGVHACEGCKGFFRRTIRLKLIYDKCD
RNCKIQKKNRNKCQYCRFQKCLSVGMSHNAIRFGRMPRSEKAKLKAEILTGENYVEDSEMADLKSLAKRI
HDAYLKNFNMNKVKARVILAGKTNNNPPFVIHDMDTLCMAEKTLVAKLVANGIQNKEAEVRIFHCCQCTS
VETVTELTEFAKSIPGFSNLDLNDQVTLLKYGVYEAIFAMLASVMNKDGMLVAYGNGFITREFLKSLRKP
FCDIMEPKFDFAMKFNALELDDSDISLFVAAIICCGDRPGLVNVGHIEKMQESIVHVLKLHLQTNHPDDI
FLFPKLLQKMADLRQLVTEHAQLVQIIKKTESDAHLHPLLQEIYRDMY

>NR1H2_HUMAN
MSSPTTSSLDTPLPGNGPPQPGAPSSSPTVKEEGPEPWPGGPDPDVPGTDEASSACSTDWVIPDPEEEPE
RKRKKGPAPKMLGHELCRVCGDKASGFHYNVLSCEGCKGFFRRSVVRGGARRYACRGGGTCQMDAFMRRK
CQQCRLRKCKEAGMREQCVLSEEQIRKKKIRKQQQQESQSQSQSPVGPQGSSSSASGPGASPGGSEAGSQ
GSGEGEGVQLTAAQELMIQQLVAAQLQCNKRSFSDQPKVTPWPLGADPQSRDARQQRFAHFTELAIISVQ
EIVDFAKQVPGFLQLGREDQIALLKASTIEIMLLETARRYNHETECITFLKDFTYSKDDFHRAGLQVEFI
NPIFEFSRAMRRLGLDDAEYALLIAINIFSADRPNVQEPGRVEALQQPYVEALLSYTRIKRPQDQLRFPR
MLMKLVSLRTLSSVHSEQVFALRLQDKKLPPLLSEIWDVHE

>PPAT_XENLA
MVDTEMPFWSNLNFGMNSMDMSALEDHCQPYDIKPFTTVDFSSINSHYDDILDEKTFLCRNDQSPIDYKY
DLKLQECQSSIKLEPPSPPYFSDKPQCSKAFEDTPNSFIAIECRVCGDKASGFHYGVHACEGCKGFFRRT
IRLKLIYERCDLNCRIHKKSRNKCQFCRFQKCLAVGMSHNAIRFGRMPQAEKEKLLAEISSDIDQLNPES
ADQRVLAKHLYDSYVKSFPLTKAKAPGHPDGQSHRQNSRGYTRHELADDGGGSDQGAVREPRAEQGGGDS
NLPALSVALRGGVREITEFAKNIPGFVSLDLNDQVTLLKYGVHEIIFTMLASLMNKDGVLVAEGQGFMTR
EFLKSLRKPFSDFMEPKFEFAIRFNSLELDDSDLAIFVAVIILSGDRPGLLNVKPIEDIQDSLLQALELQ
LKLNHPDSAQLFAKLLQKMTDLRQVVTEHVQLLQLIKKTEADMCLHPLLQEIYKDLY

>Q8QGC1_9AVES
MVDTENQLYPLTPLEEDDIGSPLSGEFLQEMENIQDISQSLGDDSSGALSLTEFQSLGNGPGSDGSVITD
TLSPASSPSSINFATAPGSIDESPSGALNIECRICGDKASGYHXGVHACEGCKGFFRRTIRLKLIYDKCD

RNCKIQKKNRNKCQYCRFQKCLSVGMSHNAIRFGRMPRSEKAKLKAEILTGESYIEDSEMADLKSLAKRI
HDAYLKNFNMNKVKARVILAGKTNNNPPFVIHDMDTLCMAEKTLVAKLVANGIQNKEAEVRIFHCCQCTS
VETVTELTEFAKSIPGFSNLDLNDQVTLLKYGVYEAIFAMLASVMNKDGMLVAYGNGFITREFLKSLRKP
FCDIMEPKFDFAMKFNALELDDSDISLFVAAIICCGDRPGLVNVGHIEKMQESIVHVLKLHLQTNHPDDT
FLFPKLLQKMADLRQLVTEHAQLVQIIKKTESDAHLHPLLQEIYRDMY

>Q9QWI0_RAT
MEQPQEETPEAREEEKEEVATGDGAPELNGGPEHTLPSSSCTDLSQNSSPSSLLDQLQMGCDGASGGSLN
MECRVCGDKASGFHYGVHACEGCKGFFRRTIRMKLKYEKCDRICKIQKKNRNKCQYCRFQKCLALGMSHN
AIRFGRMPEAEKRKLVAGLTASEGCQQNPQLADLKAFSKHIYNAYLKNFNMTKKKARSILTGKSSHNAPF
IIHDIETLWQAEKGLVWKQLVNGPRPYNEISVHVFYRCQSTTVDTVRELTEFAKNIPNFSSLFLNDQVTL
LKYGVHQAIFAMLASIVNKDGLLVANGSGFVTHEFLRSIRKPFSDIIEPKFEFAVKFNALELDDSDLALF
IAAIILCGDRPGLMNVPQVEAIQDTILQALEFHLQVNHPDSQYLFPKLLQKMADLRQLVTEHAQMMQWLK
KTESETLLHPLLQEIYKDMY

>Q866Q2_PIG
MEQPPEEAPEVREEEKKKEVAEAEGGPELNGGPEHSLPSSSCTDLSQSCSPPALLDQLQMGCDGASCGGL
SMECRVCGDKASGFHYGVHACEGCKGFFRRTIRMKLEYEKCERICKIQKKNRNKCQYCRFQKCLALGMSH
NAIRFGRMPEAEKRKLVAGLTANEGSQHNPQVADLKAFSKHLYSAYLKNFNMTKKKARAILTGKASHTAP
FVIHDIETLWQAEKGLVWKQLVNGLPPYKEISVHVFYRCQCTTVETVRELTEFAKSIPSFDHFFLNDQVT
LLKYGVHEAIFAMLASIVNKDGLLVANGTGFVTREFLRSIRKPFSDIIEPKFEFAVKFNALELDDSDLAL
FIAAIILCGDRPGLMNVSQVEAIQDTILRALEFHLQANHPDAQYLFPKLLQKMADLRQLVTEHAQMMQRI
KKTETETSLHPLLQEIYKDMY

>Q9I8W3_CHICK
MEQLQEEVPEVREEEEEEEEAVTVPSGASDPSAGPDSSLPSSSYTDLSQSSSPSLSDQLQMGCEETASGA
LNVECRVCGDKASGFHYGVHACEGCKGFFRRTIRMKLEYEKCERSCKIQKKNRNKCQYCRFQKCLSLGMS
HNAIRFGRMPEAEKRKLVAGLTASEISCQNPQVADLKAFSKHIYNAYLKNFNMTKKKARGILTGKASSTP
QPFVIHDMDTLWQAEKGLVWKQLVNGIPPYKEIGVHVFYRCQCTTVETVRELTEFAKSIPSFIGLYLNDQ
VTLLKYGVHEAIFAMLASIMNKDGLLVANGNGFVTREFLRTLRKPFNEIMEPKFEFAVKFNALELDDSDL
SLFVAAIILCGDRPGLMNVKQVEEIQDNILRALEFHLQSNHPDAQYLFPKLLQKMADLRQLVTEHAQLVQ
KIKKTETETSLHPLLQEIYKDMY

>Q6P6S7_RAT
MTTLDSNNNTGGVITYIGSSGSSPSRTSPESLYSDSSNGSFQSLTQGCPTYFPPSPTGSLTQDPARSFGT
VPPSLSDDSSPSSASSSSSSSSSSFYNGSPPGSLQVAMEDSSRVSPSKGTSNITKLNGMVLLCKVCGDVA
SGFHYGVHACEGCKGFFRRSIQQNIQYKRCLKNENCSIVRINRNRCQQCRFKKCLSVGMSRDAVRFGRIP
KREKQRMLAEMQNAMNLANNQLSSLCPLETSPAPHPTSGSVGPSPPPAPAPTPLVGFSQFPQQLTPPRSP
SPEPTVEDVISQVARAHREIFTYAHDKLGTSPGNFNANHASGSPPATTPQCWESQGCPSTPNDNNLLAAQ
RHNEALNGLRQGPSSYPPTWPSGPAHHSCHQPNSNGHRLCPTHVYSAPEGKAPANGLRQGNTKNVLLACP
MNMYPHGRSGRTVQEIWEDFSMSFTPAVREVVEFAKHIPGFRDLSQHDQVTLLKAGTFEVLMVRFASLFN
VKDQTVMFLSRTTYSLQELGAMGMGDLLNAMFDFSEKLNSLALTEEELGLFTAVVLVSADRSGMENSASV
EQLQKTLLRALRALVLKNRPSETSRFTKLLLKLPDLRTLNNMHSEKLLSFRVDAQ

>Q9N2H4_RABIT
MEQPPEETPKVREEEEKEEVAEAEGAPELSGGPEHALPSSRYTDLSQSSSPPVLLDQLQMGCDGASCGSL
NMECRVCGDKASGFHYGVHACEGCKGFFRRTIRMKLEYEKCERSCKIQKKNRNKCQHCRFQKCLALGMSH
NAIRFGRMPEAEKRKLVAGLTAVEGHQHSPQVADLKAFSKHIYNAYLKNFNMTKKKARGILTGKASHTAP
FVIHDIETLWQAEKGLVWKQLVNSLPPYKEISVHVFYRCQYTTVETVRELTEFAKSIPNFSSLFVNDQVT
VVKYGVHEAIFAMLASIVNKDGLLVANGSGFVTREFLRSLRKPFSDIIEPKFEFAVKFNALELDDSDLAL
FIAAIILCGDRPGLMNVPQVEAIQDTILRALEFHLQANHPDAQYFFPKLLQKMADLRQLVTEHAQMMQRI
KKTETETLLHPLLQEIYKDMY

>PPAR_PHACI
MVDTESQICPLSPFGDDDLESPLSEEFLQEMGSIQEISPSIGDDSSGTFAFAEYRCLGSGPGSDGSIITD
TLSPASSPSSVSYTPIAGSADDSSSATLNIECRICGDKASGYHYGVHACEGCKGFFRRTIRLKLAYDKCD
RSCKIQKKNRNKCQYCRFQKCLSDGMSHNAIRFGRMPRSEKAKLKAEILTCEHDLEDSEVADLKSLAKRI
YEAYLKNFNMNKIKARIILAGKASNNPPFVIHDMETLCMAEKTLVAKLVANGIQNKEAEVRIFHCCQCTS
VETVTELTEFAKSIPGFSNLNLNDQVTLLKYGVYEAIFAMLSSVMNKDGMLVAYGNGFITREFLKSLRKP
FCDIMEPKFDFAMKFNALELDDSDISLFVAAIICCGDRPGLLNVGHIERMQESIVHVLQLHLQNNHPDDV
FLFPKLLQKMADLRQLVTEHAQLVQVIKKTESDAALHPLLQEIYRDMY

>Q8BP65_MOUSE
MSSPTSSLDTPVPGNGSPQPSTSATSPTIKEEGQETDPPPGSEGSSSAYIVEPEDEPERKRKKGPAPKML
GHELCRVCGDKASGFHYNVLSCEGCKGFFRRSVVHGGAGRYACRGSGTCQMDAFMRRKCQLCRLRKCKEA
GMREQCVLSEEQIRKKRIQKQQQQPPPPSEPAASSSGRPAASPGTSEASSQGSGEGEGIQLTAAQELMI
QQLVAAQLQCNKRSFSDQPKVTPWPLGADPQSRDARQQRFAHFTELAIISVQEIVDFAKQVPGFLQLGRE
DQIALLKASTIEIMLLETARRYNHETECITFLKDFTYSKDDFHRAGLQVEFINPIFEFSRAMRRLGLDDA
EYALLIAINIFSADRPNVQEPSRVEALQQPYVEALLSYTRIKRPQDQLRFPRMLMKLVSLRTLSSVHSEQ
VFALRLQDKKLPPLLSEIWDVHE

>Q90970_CHICK
MSQRELRQRFPVILFACTIFSEQLLLGKQLNGRSSEGSDGAPKSDRLEEAVKPSQSGVAGLTKGHNGVTK
FNGMVLLCKVCGDVASGFHYGVHACEGCKGFFRRSIQQNIQYKKCLKNNNCSIMRMNRNRCQQCRFKKCL
SVGMSRDAVRFGRIPKREKQRMLIEMQSAMKTMMNSQFSSHLPNEALAEHQDQAPQEDLSSKPKQERETL
KSPSPPSSDMAKEEVIGMVTRVHKDTFMYNQEQSQNPAEMMQPQSGERVSKNTEQYMLSSEHCVSGLSGP
QYPDSEQHLGGQYKGRSTMHYPSGHAICFTNGHCMNFTNGYTQRLCDRIPEDVFSPNRNTTYSCNTGGRM
HLVCPMSKTPHVDPNKSGHEVWEEFSLSFTPAVKEVVEFAKRIPGFRDLSQHDQVNLLKAGTFEVLMVRF
ASLFDARERTVTFLSGKKYSVDDLHSMGAGDLLNSMFEFSEKLNALQLSDEEMSLFTAVVLVSADRSGIE
NVNSVEALQETLIRALRTLIMKNHPNEASIFTKLLLKLPDLRSLNNMHSEELLAFKVHP

>NR1H3_RAT
MSLWLEAAVPDVSPDSATELWKTEPQDAGDQGGNTCILREEARMPQSTGGALRIGLESSEPTALLPRAET
LPEPTELRPQKRKKGPAPKMLGNELCSVCGDKASAFHYNVLSCEGCKGFFRRSVIKGARYICHSGGHCPM
DTYMRRKCQECRLRKCRHAGMREECVLSEEQIRLKKLKRQEEEQAQATSVSPRVSSPPQVLPQLSPEQLG
MIEKLVAAQQQCNRRSFSDRLRVTPWPIAPDPQSREARQQRFAHFTELAIVSVQEIVDFAKQLPGFLQLS
REDQIALLKTSAIEVMLLETSRRYNPGSESITFLKDFSYNREDFAKAGLQVEFINPIFEFSRSMNELQLN
DAEFALLIAISIFSADRPNVQDQLQVERLQHTYVEALHAYVSINHPHDRLMFPRMLMKLVSLRTLSSVHS

EQVFALRLQDKKLPPLLSEIWDVHE

>PPAR_CAVPO
MVDMESPLCPLSPLEAEDLESPLSEYFLQEMGTIQDISRSLGEDSSGSFGFPEYQYLGSGPGSDGSVITD
TLSPASSPSSVSYPEVPCGVDEPPSSALNIECRICGDKASGYHYGVHACEGCKGFFRRTIRLKLVYDKCD
RSCKIQKKNRNKCQYCRFHKCLSVGMSHNAIRFGRMPRSEKAKLKAEVLTCDRDSEGAETADLKSLAKRI
YEAYLKNFHMNKVKARIILAGKTSSHPLFVIHDMETLCTAEKTLMAKVVSDGIRDKEAEVRIFHCCQCVS
VETVTNLTEFAKAIPGFASLDLNDQVTLLKYGVYEAIFTMLSSTMNKDGMLVAYGHGFITREFLKNLRKP
FCDMMEPKFNFAMKFNALELDDSDISLFVAAIICCGDRPGLLNIDHIEKMQEAIVHVLKLHLQSNHPDDT
FLFPKLLQKLADLRQLVTEHAQLVQVIKTESDAALHPLLQEIYRDMY

>NR1H3_MOUSE
MSLWLEASMPDVSPDSATELWKTEPQDAGDQGGNTCILREEARMPQSTGVALGIGLESAEPTALLPRAET
LPEPTELRPQKRKKGPAPKMLGNELCSVCGDKASGFHYNVLSCEGCKGFFRRSVIKGARYVCHSGGHCPM
DTYMRRKCQECRLRKCRQAGMREECVLSEEQIRLKKLKRQEEEQAQATSVSPRVSSPPQVLPQLSPEQLG
MIEKLVAAQQQCNRRSFSDRLRVTPWPIAPDPQSREARQQRFAHFTELAIVSVQEIVDFAKQLPGFLQLS
REDQIALLKTSAIEVMLLETSRRYNPGSESITFLKDFSYNREDFAKAGLQVEFINPIFEFSRAMNELQLN
DAEFALLIAISIFSADRPNVQDQLQVERLQHTYVEALHAYVSINHPHDRLMFPRMLMKLVSLRTLSSVHS
EQVFALRLQDKKLPPLLSEIWDVHE

>Q91X41_MOUSE
MSLWLEASMPDVSPDSATELWKTEPQDAGDQGGNTCILREEARMPQSTGVALGIGLESAEPTALLPRAET
LPEPTELRPQKRKKGPAPKMLGNELCSVCGDKASGFHYNVLSCEGCKGFFRRSVIKGARYVCHSGGHCPM
DTYMRRKCQECRLRKCRQAGMREECVLSEEQIRLKKLKRQEEEQAQATSVSPRVSSPPQVLPQLSPEQLG
MIEKLVAAQQQCNRRSFSDRLRVTPWPIAPDPQSREARQQRFAHFTELAIVSVQEIVDFAKQLPGFLQLS
REDQIALLKTSAIEVMLLETSRRYNPGSESITFLKDFSYNREDFAKAGLQVEFINPIFEFSRAMNELQLN
DAEFALLIAISIFSADRPNVQDQLQVERLQHTYVEALHAYVSINHPHDPLMFPRMLMKLVSLRTLSSVHS
EQVFALRLQDKKLPPLLSEIWDVHE

>Q9U3Y4_AEDAL
MMKRRWSNNGGFTALRMLDDSSSEVTSSSAALGMTMSPNSLGSPNYDELELWSSYEDNAYNGHSVLSNGN
NNLGGCGAANNLLMNGIVGNNNLNGMMNMASQAVQANANSIQHIVGNLINGVNPNQTLIPPLPSIIQNTL
MNTPRSESVNSISSGREDLSPSSSLNGYTDGSDAKKQKKGPTPRQQEELCLVCGDRASGYHYNALTCEGC
KGFFRRSVTKNAVYCCKFGHACEMDMYMRRKCQECRLKKCLAVGMRPECVVPENQCAIKRKEKKAQKEKD
KVQTNATVSTTNSTYRSEILPILMKCDPPPHQAIPLLPEKLLQENRLRNIPLLTANQMAVIYKLIWYQDG
YEQPSEEDLKRIMIGSPNEEEDQHDVHFRHITEITILTVQLIVEFAKGLPAFTKIPQEDQITLLKACSSE
VMMLRMARRYDAATDSILFANNRSYTRDSYRMAGMADTIEDLLHFCRQMFSLTVDNVEYALLTAIVIFSD
RPGLEQAELVEHIQSYYIDTLRIYILNRHAGDPKCSVIFAKLLSILTELRTLGNQNSEMCFSLKLKNRKL
PRFLEEIWDVQDIPPSMQAQMHSHGTPQSSSSSSSSSSSSSSNGSSNGNSNSNGPHPHPHGQQLTPNQQQ
PPHPQQQQHSQLQQVHANGSGSGGASSNNSSSSGGLGVPVGLGGGALDHV

>ECR_LUCCU
MMKRRWSNNGGFAALKMLEESSSEVTSSSNGLVLSSDINMSPSSLDSPVYGDQEMWLCNDSASYNNSHQH
SVITSLQGCTSSLPAQTTIIPLSALPNSNNASLNNQNQNYQNGNSMNTNLSVNTNNSVGGGGGGGGVPGM

TSLNGLGGGGGSQVNNHNHSHNHLHHNSNSNHSNSSSHHTNGHMGIGGGGGGLSVNINGPNIVSNAQQLN
SLQASQNGQVIHANIGIHSIISNGLNHHHHHHMNNSSMMHHTPRSESANSISSGRDDLSPSSSLNGFSTS
DASDVKKIKKGPAPRLQEELCLVCGDRASGYHYNALTCEGCKGFFRRSVTKNAVYCCKFGHACEMDMYMR
RKCQECRLKKCLAVGMRPECVVPENQCAMKRREKKAQKEKDKIQTSVCATEIKKEILDLMTCEPPSHPTC
PLLPEDILAKCQARNIPPLSYNQLAVIYKLIWYQDGYEQPSEEDLKRIMSSPDENESQHDASFRHITEIT
ILTVQLIVEFAKGLPAFTKIPQEDQITLLKACSSEVMMLRMARRYDHNSDSIFFANNRSYTRDSYKMAGM
ADNIEDLLHFCRQMYSMKVDNVEYALLTAIVIFSDRPGLEEAELVEAIQSYYIDTLRIYILNRHCGDPMS
LVFFAKLLSILTELRTLGNQNAEMCFSLKLKNRKLPKFLEEIWDVHAIPPSVQSHIQATQAEKAGPGSSG
NNIGHFSSRHLIFLHKYLDGNIILIIVIAIGASTPNGGAVDYVGTDMSMSLVQSDNA

>NR1H2_RAT
MSSPTSSLDTPLPGNGSPQPSTSSTSPTIKEEGQETDPPPGSEGSSSAYIVVILEPEDEPERKRKKGPAP
KMLGHELCRVCGDKASGFHYNVLSCEGCKGFFRRSVVHGGAGRYACRGSGTCQMDAFMRRKCQLCRLRKC
KEAGMREQCVLSEEQIRKKKIQKQQQQQPPPPTEPASGSSARPAASPGTSEASSQGSGEGEGIQLTAAQE
LMIQQLVAAQLQCNKRSFSDQPKVTPWPLGADPQSRDARQQRFAHFTELAIISVQEIVDFAKQVPGFLQL
GREDQIALLKASTIEIMLLETARRYNHETECITFLKDFTYSKDDFHRAGLQVEFINPIFEFSRAMRRLGL
DDAEYALLIAINIFSADRPNVQEPSRVEALQQPYVEALLSYTRIKRPQDQLRFPRMLMKLVSLRTLSSVH
SEQVFALRLQDKKLPPLLSEIWDVHE

>PPAR_XENLA
MSTIMVDTNSELCILTPLDEDDLESPLSGEFLQDIVDIQDITQTIGDDGSTPFGASEHQFFGNSPGSIGS
VSTDLTDTLSPASSPASITFPAASGSAEDAACKSLNLECRVCSDKASGFHYGVHACEGCKGFFRRTIRLK
LVYDRCERMCKIQKKNRNKCQYCRFEKCLNVGMSHNAIRFGRMPRSEKAKLKAEVLMCDQDVKDSQMADL
LSLARLIYDAYLKNFNMNKVKARAILTGKASNPPFVIHDMETLCMAEKTLVAKLVANGIQNKEAEVRIFH
CCQCTSVETVTELTEFAKSIPGFTELDLNDQVTLLKYGVYEAMFAMLASVMNKDGMLVAYGNGFITREFL
KSLRKPIGDMMEPKFEFAMKFNALELDDSDLSLFVAALICCGDRPGLVNIPSIEKMQESIVHVLKLHLQS
NHPDDSFLFPKLLQKMADLRQLVTEHAQLVQTIKKTETDAALHPLLQEIYRDMY

>Q6AXA9_XENLA
MSTIMVDTNSELCILTPLDEDDLESPLSGEFLQDIVDIQDITQTIGDDGSTPFGASEHQFFGNSPGSIGS
VSTDLTDTLSPASSPASITFPAASGSAEDAACKSLNLECRVCSDKASGFHYGVHACEGCKGFFRRTIRLK
LVYDRCERMCKIQKKNRNKCQYCRFEKCLNVGMSHNAIRFGRMPRSEKAKLKAEVLMCDQDVKDTQMADL
LSLARLIYDAYLKNFNMNKVKARAILTGKASNPPFVIHDMETLCMAEKTLVAKLVANGIQNKEAEVRIFH
CCQCTSVETVTELTEFAKSIPGFTELDLNDQVTLLKYGVYEAMFAMLASVMNKDGMLVAYGNGFITREFL
KSLRKPIGDMMEPKFEFAMKFNALELDDSDLSLFVAALICCGDRPGLVNIPSIEKMQESIVHVLKLHLQS
NHPDDSFLFPKLLQKMADLRQLVTEHAQLVQTIKKTETDAALHPLLQEIYRDMY

>Q8IW13_HUMAN
MPHSAGGTAGVGLEAAEPTALLTRAEPPSEPTEIRPQKRKKGPAPKMLGNELCSVCGDKASGFHYNVLSC
EGCKGFFRRSVIKGAHYICHSGGHCPMDTYMRRKCQECRLRKCRQAGMREECVLSEEQIRLKKLKRQEEE
QAHATSLPPRASSPPQILPQLSPEQLGMIEKLVAAQQQCNRRSFSDRLRVTPWPMAPDPHSREARQQRFA
HFTELAIVSVQEIVDFAKQLPGFLQLSREDQIALLKTSAIEVMLLETSRRYNPGSESITFLKDFSYNRED
FAKAGLQVEFINPIFEFSRAMNELQLNDAEFALLIAISIFSADRPNVQDQLQVERLQHTYVEALHAYVSI
HHPHDRLMFPRMLMKLVSLRTLSSVHSEQVFALRLQDKKLPPLLSEIWDVHE

>Q5U0N9_HUMAN
MSLWLGAPVPDIPPDSAVELWKPGAQDASSQAQGGSSCILREEARMPHSAGGTAGVGLEAAEPTALLTRA
EPPSEPKEIRPQKRKKGPAPKMLGNELCSVCGDKASGFHYNVLSCEGCKGFFRRSVIKGAHYICHSGGHC
PMDTYMRRKCQECRLRKCRQAGMREECVLSEEQIRLKKLKRQEEEQAHATSLPPRASSPPQILPQLSREQ
LGMIEKLVAAQQQCNRRSFSDRLRVTPWPMAPDPHSREARQQRFAHFTELAIVSVQEIVDFAKQLPGFLQ
LSREDQIALLKTSAIEVMLLETSRRYNPGSESITFLKDFSYNREDFAKAGLQVEFINPIFEFSRAMNELQ
LNDAEFALLIAISIFSADRPNVQDQLQVERLQHTYVEALHAYVSIHHPHDRLMFPRMLMKLVSLRTLSSV
HSEQVFALRLQDKKLPPLLSEIWDVHE

>NR1D1_HUMAN
MTTLDSNNNTGGVITYIGSSGSSPSRTSPESLYSDNSNGSFQSLTQGCPTYFPPSPTGSLTQDPARSFGS
IPPSLSDDGSPSSSSSSSSSSSSFYNGSPPGSLQVAMEDSSRVSPSKSTSNITKLNGMVLLCKVCGDVAS
GFHYGVHACEGCKGFFRRSIQQNIQYKRCLKNENCSIVRINRNRCQQCRFKKCLSVGMSRDAVRFGRIPK
REKQRMLAEMQSAMNLANNQLSSQCPLETSPTQHPTPGPMGPSPPPAPVPSPLVGFSQFPQQLTPPRSPS
PEPTVEDVISQVARAHREIFTYAHDKLGSSPGNFNANHASGSPPATTPHRWENQGCPPAPNDNNTLAAQR
HNEALNGLRQAPSSYPPTWPPGPAHHSCHQSNSNGHRLCPTHVYAAPEGKAPANSPRQGNSKNVLLACPM
NMYPHGRSGRTVQEIWEDFSMSFTPAVREVVEFAKHIPGFRDLSQHDQVTLLKAGTFEVLMVRFASLFNV
KDQTVMFLSRTTYSLQELGAMGMGDLLSAMFDFSEKLNSLALTEEELGLFTAVVLVSADRSGMENSASVE
QLQETLLRALRALVLKNRPLETSRFTKLLLKLPDLRTLNNMHSEKLLSFRVDAQ

>NR1H2_MOUSE
MSSPTSSLDTPVPGNGSPQPSTSATSPTIKEEGQETDPPPGSEGSSSAYIVVILEPEDEPERKRKKGPAP
KMLGHELCRVCGDKASGFHYNVLSCEGCKGFFRRSVVHGGAGRYACRGSGTCQMDAFMRRKCQLCRLRKC
KEAGMREQCVLSEEQIRKKRIQKQQQQQPPPPSEPAASSSGRPAASPGTSEASSQGSGEGEGIQLTAAQE
LMIQQLVAAQLQCNKRSFSDQPKVTPWPLGADPQSRDARQQRFAHFTELAIISVQEIVDFAKQVPGFLQL
GREDQIALLKASTIEIMLLETARRYNHETECITFLKDFTYSKDDFHRAGLQVEFINPIFEFSRAMRRLGL
DDAEYALLIAINIFSADRPNVQEPSRVEALQQPYVEALLSYTRIKRPQDQLRFPRMLMKLVSLRTLSSVH
SEQVFALRLQDKKLPPLLSEIWDVHE

>ECR_AEDAE
MMKRRWSNNGGFTALRMLDDSSSEVTSSSAALGMTMSPNSLGSPNYDELELWSSYEDNAYNGHSVLSNGN
NNLGGCGAANNLLMNGIVGNNNLNGMMNMASQAVQANANSIQHIVGNLINGVNPNQTLIPPLPSIIQNTL
MNTPRSESVNSISSGREDLSPSSSLNGYTDGSDAKKQKKGPTPRQQEELCLVCGDRESGYHYNALTCEGC
KGFFRRSVTKNAVYCCKFGHACEMDMYMRRKCQECRLKKCLAVGMRPECVVPENQCAIKRKEKKAQKEKD
KVQTNATVSTTNSTYRSEILPILMKCDPPPHQAIPLLPEKLLQENRLRNIPLLTANQMAVIYKLIWYQDG
YEQPSEEDLKRIMIGSPNEEEDQHDVHFRHITEITILTVQLIVEFAKGLPAFTKIPQEDQITLLKACSSE
VMMLRMARRYDAATDSILFANNRSYTRDSYRMAGMADTIEDLLHFCRQMFSLTVDNVEYALLTAIVIFSD
RPGLEQAELVEHIQSYYIDTLRIYILNRHAGDPKCSVIFAKLLSILTELRTLGNQNSEMCFSLKLKNRKL
PRFLEEIWDVQDIPPSMQAQMHSHGTQSSSSSSSSSSSSSNGSSNGNSSSNSNSSQHGPHPHPHGQQLTP
NQQQHQQQHSQLQQVHANGSGSGGGSNNNSSSGGVVPGLGMLDQV

>Q8MYA6_9NEOP
MRRRWSNNGGFQTLRMLEESSSEVTSSSALGLPPAMVMSPESLASPEYAGLELWGYDDGITYNATQSLLG

NTCTMQQQQPPTQPLPSMPLPMPPTTPKSENESMSSGREELSPASSVNGCSTDGESRRQKKGPAPRQQEE
LCLVCGDRASGYHYNALTCEGCKGFFRRSVTKNAVYICKFGHACEMDMYMRRKCQECRLKKCLAVGMRPE
CVVPETTCAIKRKEKKAQREKDKLPVSTTTVDDHMPPIMQCDPPPPEAARILECLQHEVVPRFLSEKLME
QNRLKNIPPLTANQQFLIARLVWYQDGYEQPSEEDLKRVTQTWQSNEDEEEETDLPFRQITEMTILTVQL
IVEFAKGLPGFSKISQPDQITLLKACSSEVMMLRVARRYDAASDSVLFANNQAYTRDNYRKAGMAYVIED
LLHFCRCMYSLSMDNVHYALLTAVVIFSDRPGLEQPQLVEEIQRYYLNTLRVYIMNQHSASPRCAVLYAK
ILSVLTELRTLGMQNSNMCISLKLKNRKLPPFLEEIWDVADVSAAQAPAIMDVATNL

>Q7T029_PLEPL
MVDTQQLLAWPVGFSLSAVDLSELDDSSHSLDMKHLATLDYTSISSASVPSSLSPQLMSSISSVGVAYDP
SPPQSEEHLTNMDYTNMHSYRTEPNVHNSIKMEPESPPQYSDSPVFSKLQDDTTAASLNIECRVCGDKAS
GFHYGVHACEGCKGFFRRTIRLKLVYDHCDLHCRIHKKSRNKCQYCRFQKCLNVGMSHNAIRFGRMPQAE
KEKLLAEFSSDMEHMHPEAADLRALARHLYEAYLKYFPLTKAKARAILSGKTGDNAPFVIHDIKSLMEGE
QFINCRQMPIQEQQQASVLTATHGGLTEHHMGSDYGVWGTTSISGQEPQNALELRFFQSCQSRSAEAVRE
VTEFAKSIPGFTDLDLNDQVTLLKYGVIEVLIIMMSPLMNKDGTLISYGQIFMTREFLKSLRKPFCQMME
PKFEFSVKFNTLELDDSDMALFLVVIILSGDRPGLLNVKPIEQLQETVLHSLELQLKLNHPDSLQLFAKL
LQKMTDLRQIVTDHVHLIQLLKKTEVDMCLHPLLQEIMKDLY

>Q9W712_PLAFE
MVDTQQLLAWPVGFSLSAVDLSELDDSSHSLDMKHLATLDYTSISSASVPSSLSPQLMSSISPVGMAYDP
SPPQSEEHLTNMDYTNMHSYRTEPNVHNSIKMEPESPPQYSDSPVFSKLQDDPTAASLNIECRVCGDKAS
GFHYGVHACEGCKGFFRRTIRLKLVYDHCDLHCRIHKKSRNKCQYCRFQKCLNVGMSHNAIRFGRMPQAE
KEKLLAEFSSDMEHMHPEAADLRALARHLYEAYLKYFPLTKAKARAILSGKTGDNAPFVIHDMKSLMEGE
QFINCRQMPIQEQQQASVLTAAHGGLTEVHMGSDYGVWGMTSISGQEPQNALELRFFQSCQSPSAEAVRE
VTEFAKSIPGFTDLDLNDQVTLLKYGVIEVLIIMMSPLMNKDGTLISYGQIFMTREFLKSLRKPFCQMME
PKFEFSVKFNTLELDDSDMALFLVVIILSGDRPGLLNVKPIEQLQETVLHSLELQLKLNHPDSLQLFAKL
LQKMTDLRQIVTDHVHLIQLLKKTEIDMCLHPLLQEIMKDLY

>Q9VVM9_DROME
MEAVQAAAAATSSGGSSGSVPGSGSGSASKLIKTEPIDFEMLHLEENERQQDIEREPSSSNSNSNSNSLT
PQRYTHVQVQTVPPRQPTGLTTPGGTQKVILTPRVEYVQQRATSSTGGGMKHVYSQQQGTAASRSAPPET
TALLTTTSGTPQIIITRTLPSNQHLSRRHSASPSALHHYQQQPQRQQSPPPLHHQQQQQQQHVRVIRDG
RLYDEATVVVAARRHSVSPPPLHHHSRSAPVSPVIARRGGAAAYMDQQYQQRQTPPLAPPPPPPPPPPPP
PPPQQQQQQYISTGVPPPTAAARKFVVSTSTRHVNVIASNHFQQQQQQHQAQQHQQQHQQHQQHQQHVIA
SVSSSSSSSAIGSGGSSSSHIFRTPVVSSSSSSNMHHQQQQQQQQSSLGNSVMRPPPPPPPPKVKHASSS
SSGNSSSSNTNNSSSSSNGEEPSSSIPDLEFDGTTVLCRVCGDKASGFHYGVHSCEGCKGFFRRSIQQKI
QYRPCTKNQQCSILRINRNRCQYCRLKKCIAVGMSRDAVRFGRVPKREKARILAAMQQSTQNRGQQRALA
TELDDQPRLLAAVLRAHLETCEFTKEKVSAMRQRARDCPSYSMPTLLACPLNPAPELQSEQEFSQRFAHV
IRGVIDFAGMIPGFQLLTQDDKFTLLKAGLFDALFVRLICMFDSSINSIICLNGQVMRRDAIQNGANARF
LVDSTFNFAERMNSMNLTDAEIGLFCAIVLITPDRPGLRNLELIEKMYSRLKGCLQYIVAQNRPDQPEFL
AKLLETMPDLRTLSTLHTEKLVVFRTEHKELLRQQMWSMEDGNNSDGQQNKSPSGSWADAMDVEAAKSPL
GSVSSTESADLDYGSPSSSQPQGVSLPSPPQQQPSALASSAPLLAATLSGGCPLRNRANSGSSGDSGAAE
MDIVGSHAHLTQNGLTITPIVRHQQQQQQQQQIGILNNAHSRNLNGGHAMCQQQQQHPQLHHHLTAGAAR
YRKLDSPTDSGIESGNEKNECKAVSSGGSSSCSSPRSSVDDALDCSDAAANHNQVVQHPQLSVVSVSPVR

SPQPSTSSHLKRQIVEDMPVLKRVLQAPPLYDTNSLMDEAYKPHKKFRALRHREFETAEADASSSTSGSN
SLSAGSPRQSPVPNSVATPPPSAASAAAGNPAQSQLHMHLTRSSPKASMASSHSVLAKSLMAEPRMTPEQ
MKRSDIIQNYLKRENSTAASSTTNGVGNRSPSSSSTPPPSAVQNQQRWGSSSVITTTCQQRQQSVSPHSN
GSSSSSSSSSSSSSSSSSTSSNCSSSSASSCQYFQSPHSTSNGTSAPASSSSGSNSATPLLELQVDIADS
AQPLNLSKKSPTPPPSKLHALVAAANAVQRYPTLSADVTVTASNGGPPSAAASPAPSSSPPASVGSPNPG
LSAAVHKVMLEA

>Q8MYA7_9NEOP
MDLKHEVAYRGLPRQVKAEPGVSHNGHQVNGHVRYWMAGVVGGGSPSPGAATQPPNNGYSSPLSSSSYGP
YSPNGKIGREELSPASSVNGCSTDGESRRQKKGPAPRQQEELCLVCGDRASGYHYNALTCEGCKGFFRRS
VTKNAVYICKFGHACEMDMYMRRKCQECRLKKCLAVGMRPECVVPETTCAIKRKEKKAQREKDKLPVSTT
TVDDHMPPIMQCDPPPPEAARILECLQHEVVPRFLSEKLMEQNRLKNIPPLTANQQFLIARLVWYQDGYE
QPSEEDLKRVTQTWQSNEDEEEETDLPFRQITEMTILTVQLIVEFAKGLPGFSKISQPDQITLLKACSSE
VMMLRVARRYDAASDSVLFANNQAYTRDNYRKAGMAYVIEDLLHFCRCMYSLSMDNVHYALLTAVVIFSD
RPGLEQPQLVEEIQRYYLNTLRVYIMNQHSASPRCAVLYAKILSVLTELRTLGMQNSNMCISLKLKNRKL
PPFLEEIWDVADVSAAQAPAIMDVATNL

>O76827_CERCA
MMKRRWSNNGGFALRMLEESSAEVSSSSNGLVLSTTLSPSPLDSPVYGDQDLWYDAAFNGHGGGGGGGAH
SVITSAHSCAALLPPQTSIIPLGGGVNNTNMNGAGNPGINFGGSGGGVNGIVGGGSLAHGSQLGNGHLGN
QLNSHNNNNNNMNMHNNNINTTNMLHATLNQGLINGLMSGVLGVSGGMQHNGLNMQQHTPRSDSANSISS
GRDDLSPSSSLNGYSANDSCDVKKIKKGPAPRLQEELCLVCGDRASGYHYNALTCEGCKGFFRRSVTKNA
VYCCKFGRSCEMDMYMRRKCQECRMKKCLAVGMRPECVVPENQCAMKRREKKAQKEKEKQTTGNSPIICK
TESIKNEILELMNCEPPSHPTCPLLPDDIVAKCKASNIPPLTRNQLAVIYKLIWYQDGYEQPSEEDLKRI
MSTPDENESPNDISFRHITEITILTVQLIVEFAKGLPAFTKIPQEDQITLLKACSSEVMMLRMARRYDHN
SDSIFFANNRSYTRDAYKMAGVADNIEDLLHFCRQMYSMKVDNVEYALLTAIVIFSDRPGLEKAQLVEEI
QSYYIDTLRVYIINRHCGDSMSLVFFAKLLSILTELRTLGNQNAEMCFSLKLKNRKLPKFLEEIWDVHAV
PPSVQSHIQATQAEREGLEPNTAVATTSTSAAASSSPVRPLTW

>Q8IQS3_DROME
MLMSADSSDSAKTSVICSTVSASMLAPPAPEQPSTTAPPILGVTGRSHLENALKLPPNTSVSAYYQHNSK
LGMGQNYNPEFRSLVAPVTDLDTVPPTGVTMASSSNSPNSSVKLPHSGVIFVSKSSAVSTTDGPTAVLQQ
QQPQQQMPQHFESLPHHHPQQEHQPQQQQQQHHLQHHPHPHVMYPHGYQQANLHHSGGIAVVPADSRPQT
PEYIKSYPVMDTTVASSVKGEPELNIEFDGTTVLCRVCGDKASGFHYGVHSCEGCKGFFRRSIQQKIQYR
PCTKNQQCSILRINRNRCQYCRLKKCIAVGMSRDAVRFGRVPKREKARILAAMQQSTQNRGQQRALATEL
DDQPRLLAAVLRAHLETCEFTKEKVSAMRQRARDCPSYSMPTLLACPLNPAPELQSEQEFSQRFAHVIRG
VIDFAGMIPGFQLLTQDDKFTLLKAGLFDALFVRLICMFDSSINSIICLNGQVMRRDAIQNGANARFLVD
STFNFAERMNSMNLTDAEIGLFCAIVLITPDRPGLRNLELIEKMYSRLKGCLQYIVAQNRPDQPEFLAKL
LETMPDLRTLSTLHTEKLVVFRTEHKELLRQQMWSMEDGNNSDGQQNKSPSGSWADAMDVEAAKSPLGSV
SSTESADLDYGSPSSSQPQGVSLPSPPQQQPSALASSAPLLAATLSGGCPLRNRANSGSSGDSGAAEMDI
VGSHAHLTQNGLTITPIVRHQQQQQQQQIGILNNAHSRNLNGGHAMCQQQQQHPQLHHHLTAGAARYRK
LDSPTDSGIESGNEKNECKAVSSGGSSSCSSPRSSVDDALDCSDAAANHNQVVQHPQLSVVSVSPVRSPQ
PSTSSHLKRQIVEDMPVLKRVLQAPPLYDTNSLMDEAYKPHKKFRALRHREFETAEADASSSTSGSNSLS
AGSPRQSPVPNSVATPPPSAASAAAGNPAQSQLHMHLTRSSPKASMASSHSVLAKSLMAEPRMTPEQMKR

SDIIQNYLKRENSTAASSTTNGVGNRSPSSSSTPPPSAVQNQQRWGSSSVITTTCQQRQQSVSPHSNGSS
SSSSSSSSSSSSSSSTSSNCSSSSASSCQYFQSPHSTSNGTSAPASSSSGSNSATPLLELQVDIADSAQP
LNLSKKSPTPPPSKLHALVAAANAVQRYPTLSADVTVTASNGGPPSAAASPAPSSSPPASVGSPNPGLSA
AVHKVMLEA

>E75A_DROME
MLMSADSSDSAKTSVICSTVSASMLAPPAPEQPSTTAPPILGVTGRSHLENALKLPPNTSVSAYYQHNSK
LGMGQNYNPEFRSLVAPVTDLDTVPPTGVTMASSSNSPNSSVKLPHSGVIFVSKSSAVSTTDGPTAVLQQ
QQPQQQMPQHFESLPHHHPQQEHQPQQQQQQHHLQHHPHPHVMYPHGYQQANLHHSGGIAVVPADSRPQT
PEYIKSYPVMDTTVASSVKGEPELNIEFDGTTVLCRVCGDKASGFHYGVHSCEGCKGFFRRSIQQKIQYR
PCTKNQQCSILRINRNRCQYCRLKKCIAVGMSRDAVRFGRVPKREKARILAAMQQSTQNRGQQRALATEL
DDQPRLLAAVLRAHLETCEFTKEKVSAMRQRARDCPSYSMPTLLACPLNPAPELQSEQEFSQRFAHVIRG
VIDFAGMIPGFQLLTQDDKFTLLKAGLFDALFVRLICMFDSSINSIICLNGQVMRRDAIQNGANARFLVD
STFNFAERMNSMNLTDAEIGLFCAIVLITPDRPGLRNLELIEKMYSRLKGCLQYIVAQNRPDQPEFLAKL
LETMPDLRTLSTLHTEKLVVFRTEHKELLRQQMWSMEDGNNSDGQQNKSPSGSWADAMDVEAAKSPLGSV
SSTESADLDYGSPSSSQPQGVSLPSPPQQQPSALASSAPLLAATLSGGCPLRNRANSGSSGDSGAAEMDI
VGSHAHLTQNGLTITPIVRHQQQQQQQQQIGILNNAHSRNLNGGHAMCQQQQQHPQLHHHLTAGAARYRK
LDSPTDSGIESGNEKNECKAVSSGGSSSCSSPRSSVDDALDCSDAAANHNQVVQHPQLSVVSVSPVRSPQ
PSTSSHLKRQIVEDMPVLKRVLQAPPLYDTNSLMDEAYKPHKKFRALRHREFETAEADASSSTSGSNSLS
AGSPRQSPVPNSVATPPPSAASAAAGNPAQSQLHMHLTRSSPKASMASSHSVLAKSLMAEPRMTPEQMKR
SDIIQNYLKRENSTAASSTTNGVGNRSPSSSSTPPPSAVQNQQRWGSSSVITTTCQQRQQSVSPHSNGSS
SSSSSSSSSSSSSSSTSSNCSSSSASSCQYFQSPHSTSNGTSAPASSSSGSNSATPLLELQVDIADSAQP
LNLSKKSPTPPPSKLHALVAAANAVQRYPTLSADVTVTASNGGSSVGGGESGRQQQSAGECGLPQSGPER
RRAQGNAGGVRAGGGRWFYAEKWERQRLGVAVQRSRKQDHLERRELN

>Q9I8F6_SALSA
MDNKMNSFDMKTLSTLDCPYLPSLEYSHNSPHHHHSPDRSHSCNHSPDRSHSFNHSPDRSHSFNHSPDRN
HSFNHSPDRSHSFNHSPDRSHSYNDTYSVYQGSVNDKPLSPSQSSDCSIVSLSRPRPHSNPPTYTDASSL
LNIDCRVCGDKASGFHYGVHVCEGCKGFFRRTVRLKLEYDHCDLHCRIHKKSRNKCQYCRFQKCLLVGMS
HDAIRFGRMPQVEREKLLQAEFMDVEPRNPESADLRALSRQLCLSYHRHFPLTKSKAKAILSGKTHGNSP
FVIHDMKSLTAGQYFINCRQLPVLERQRSVLPPEEPAEELELSVFRRIQFRSAEAVQEVTEFTKSIPGFT
ELDMNDQVILLKYGVIEVMTTMLAPLMNKDGTLFAYGQIFMTREFLKSLRKPFCEMMEPKFEFAAKFNLL
ELDDSDMALFFAVIILSGDRPGLVNVKPIEDLQETVLQALELQLKTIHPDCPQLFAKLLQKMTDLRQLGG
QPRSAHIHLLKKQELQMCLHPLLQEIMRDLY

>ECR_MANSE
MRRRWSNNGCFPLRMFEESSSEVTSSSAFGMPAAMVMSPESLASPEYGGLELWSYDETMTNYPAQSLLGA
CNAPQQQQQQQQQQPSAQPLPSMPLPMPPTTPKSENESMSSGREELSPASSINGCSTDGEPRRQKKGPAP
RQQEELCLVCGDRASGYHYNALTCEGCKGFFRRSVTKNAVYICKFGHACEMDMYMRRKCQECRLKKCLAV
GMRPECVVPESTCKNKRREKEAQREKDKLPVSTTTVDDHMPAIMQCDPPPPEAARIHEVVPRFLTEKLME
QNRLKNVTPLSANQKSLIARLVWYQEGYEQPSEEDLKRVTQTWQLEEEEEEETDMPFRQITEMTILTVQL
IVEFAKGLPGFSKISQSDQITLLKASSSEVMMLRVARRYDAATDSVLFANNQAYTRDNYRKAGMSYVIED
LLHFCRCMYSMSMDNVHYALLTAIVIFSDRPGLEQPLLVEEIQRYYLKTLRVYILNQHSASPRCAVLFGK
ILGVLTELRTLGTQNSNMCISLKLKNRKLPPFLEEIWDVAEVSTTQPTPGVAAQVTPIVVDNPAAL

>RORA_MOUSE
MESAPAAPDPAASEPGSSGSEAAAGSRETPLTQDTGRKSEAPGAGRRQSYASSSRGISVTKKTHTSQIEI
IPCKICGDKSSGIHYGVITCEGCKGFFRRSQQSNATYSCPRQKNCLIDRTSRNRCQHCRLQKCLAVGMSR
DAVKFGRMSKKQRDSLYAEVQKHRMQQQRDHQQQPGEAEPLTPTYNISANGLTELHDDLSTYMDGHTPE
GSKADSAVSSFYLDIQPSPDQSGLDINGIKPEPICDYTPASGFFPYCSFTNGETSPTVSMAELEHLAQNI
SKSHLETCQYLREELQQITWQTFLQEEIENYQNKQREVMWQLCAIKITEAIQYVVEFAKRIDGFMELCQN
DQIVLLKAGSLEVVFIRMCRAFDSQNNTVYFDGKYASPDVFKSLGCEDFISFVFEFGKSLCSMHLTEDEI
ALFSAFVLMSADRSWLQEKVKIEKLQQKIQLALQHVLQKNHREDGILTKLICKVSTLRALCGRHTEKLMA
FKAIYPDIVRLHFPPLYKELFTSEFEPAMQIDG

>Q6GMA2_XENLA
MSATAVDGSDVSLDGDSFHSVGTVLQDSAGSVKLQIKEERLESQESASLPNENEVLGTDGEGSSQSAAEE
PERKRKKGPAPKMLGNEVCSVCGDKASGFHYNVLSCEGCKGFFRRSVIKNAQYSCKNNGKCQMDMYMRRK
CQECRLRKCREAGMREQCVLSEEQIRSKKIRKQQDEDVTRSSSALVPPSPCMLSQEVVQLTPQQEKMIEQ
LVSAQQQCNKRSFSDQPKVTPWPPGTDPNSREARQQRFAHFTELAIISVQEIVDFAKQVPGFLELSREDQ
IALLKASTIEIMLLETARRYNHETECITFLKDFTYSKDDFHRAGLQVEFINPIFEFSRGMRQMQLDDAEY
ALLIAINIFSADRPNVLNHQHVENLQLPYVEALHSYTRIKRPQDHLMFPRMLMKLVSLRTLSSVHSEQFF
ALRLQDKKLPPLLSEIWDVHE

>O77255_CHOFU
MRRRWSNNGGFQTLRMLEESSSEVTSSSALGLPAAMVMSPESLASPEYGGLELWGYDDGLSYNTAQSLLG
NTCTMQQQQQTQPLPSMPLPMPPTTPKSENESISSGREELSPASSINGCSTDGEARRQKKGPAPRQQEEL
CLVCGDRASGYHYNALTCEGCKGFFRRSVTKNAVYICKFGHACEMDMYMRRKCQECRLKKCLAVGMRPEC
VVPETQCAMKRKEKKAQKEKDKLPVSTTTVDDHMPPIMQCEPPPPEAARIHEVVPRFLSDKLLETNRQKN
IPQLTANQQFLIARLIWYQDGYEQPSDEDLKRITQTWQQADDENEESDTPFRQITEMTILTVQLIVEFAK
GLPGFAKISQPDQITLLKACSSEVMMLRVARRYDAASDSVLFANNQAYTRDNYRKAGMAYVIEDLLHFCR
CMYSMALDNIHYALLTAVVIFSDRPGLEQPQLVEEIQRYYLNTLRIYILNQLSGSARSSVIYGKILSILS
ELRTLGMQNSNMCISLKLKNRKLPPFLEEIWDVADMSHTQPPPILESPTNL

>Q6RVD3_PLOIN
MRRRWSNNGGFQTLRMLEESSSEVTSSSALGLPPAMVMSPESLASPEYGGLELWSYDDGISYNTATAQLL
GNACTMQQQQPPTQPLPSMPLPMPPTTPKSENESMSSGREELSPASSVNGCSTDGDARRQKKGPAPRQQE
ELCLVCGDRASGYHYNALTCEGCKGFFRRSVTKNAVYIYKFGHACEMDMYMRRKCQECRLKKCLAVGMRP
ECVVPETQCAIKRKEKKAQREKDKLPVSTTTVDDHMPPIMQCDPPPPEAARIHEVVPRFLSDKLMEQNRL
KNIPPLTANQQFLIAGLVWYQDGYEQPSEEDLKRVTQTWQPTDEDDDDMPFRQITEMTILTVQLIVEFAK
GLPGFSKISQPDQITLLKACSSEVMMLRVARRYDAATDSVLFANNQAYTRDNYRKAGMAYVIEDLLHFCR
CMYSMTMDNVHYALLTAIVIFSDRPGLEQPQLVEEIQRYYLNTLRVYIMNQHSDSPRCAVVFGKILSVLT
EIRTLGMQNSNMCISLKLKNRKLPPFLEEIWDVADVSTAQQLPLVDAGSAL

>Q8JHU1_CHICK
MGPTQLSTQDHGKRVASVFEMEEEGLSLFSGSENPPKHAENPPLKRKKGPAPKMLGNEVCSVCGDKASGF
HYNVLSCEGCKGFFRRSVIKGAQYVCKNGGKCEMDMYMRRKCQECRLRKCQEAGMREQYVLSEEQIRLKK
LKKQEDDQARTVVVRPNPPQPPSPSHQLTPEQLNMIEKLVAAQQQCNQRSFTDRLKVTPWPQVPDPNNRE

ARQQRFAHFTELAIISVQEIVDFAKQLPGFRELTREDQIALLKTSTIEVMLLETSRRYNPEIESITFLKD
LSYNRDDFAKAGLQFEFINPIFEFSKGMNELQLNDAEYALLIAINIFSADRPNVQDQSLVERLQHTYVEA
LHSYICINRPNDHLMFPRMLMKLVSLRTLSSVHSEQVFALRLQDKKLPPLLSEIWDVHE

>Q5RGZ2_BRARE
MVDMPSLYSPSSPLGDPIMYSPLSGELIGDMQVLEDISQSLSDDTFNSFHMLDYQNCDTAVDNSSILDVL
TPASSPSSEVFSASTGQDENSSGSLTLECRVCADRASGFHYGVHACEGCKGFFRRTIRLKLEYDKCERNC
KIQKKNRNKCQYCRFRKCLAVGMSHNAIRFGRIPQSEKQRLKAEQDVSGKEEHKCQQPDMRSLARQMHEA
YLKHFHMNKAKARVFLTGKTSTPPFVIHDMDTLQHAEKKLVTQLLGNVASGDISTLQEREVEARLFLFCQ
YASVATVTELTEYAKAVPGFADLDLNDQVTLLKYGVYEALFTLLASCMNKDGLLVAQGGGFITREFLKSL
RKPFSDMMEPKFQFAMKFNALELDDSDLALFVAAIICCGDRPGLSNVPQIERIQESVIHSLRLHLTSNHP
DNSLLFPKLLQKLADLRQLVTEHAELVQEINKTEDASLHPLLQEIYRDMY

>O77240_CHOFU
MDLKHEVAYRGVLPGQVKAEPGVHNGQVNGHVRDWMAGGAGANSPSPGAVAQPQPNNGYSSPLSSGSYGP
YSPNGKIGREELSPASSINGCSTDGEARRQKKGPAPRQQEELCLVCGDRASGYHYNALTCEGCKGFFRRS
VTKNAVYICKFGHACEMDMYMRRKCQECRLKKCLAVGMRPECVVPETQCAMKRKEKKAQKEKDKLPVSTT
TVDDHMPPIMQCEPPPPEAARIHEVVPRFLSDKLLETNRQKNIPQLTANQQFLIARLIWYQDGYEQPSDE
DLKRITQTWQQADDENEESDTPFRQITEMTILTVQLIVEFAKGLPGFAKISQPDQITLLKACSSEVMMLR
VARRYDAASDSVLFANNQAYTRDNYRKAGMAYVIEDLLHFCRCMYSMALDNIHYALLTAVVIFSDRPGLE
QPQLVEEIQRYYLNTLRIYILNQLSGSARSSVIYGKILSILSELRTLGMQNSNMCISLKLKNRKLPPFLE
EIWDVADMSHTQPPPILESPTNL

>Q8JIT8_SALSA
MKSHDEEVVQQEQEDQQEKGNSNSHSTSNTDSPALSSSCTDLSQTSSPSLSDQLLLGREDVTGAGINVEC
RICGDKASGFHYGVHACEGCKGFFRRTIRMKLEYERCERSCKIQKKSRNKCQYCRFQKCLLLGMSHDAIR
YGRMPEAEKRKLVAGLLAGERAPTTNPNGSDLKSLAKEVNNAYLKNLNMTKKKARSILTGKTSSSPVEYP
YPFVIHDMDSLCQAENGLVWKQLINGTTPNKEIGVHVFYRCQCTTVETVRELTEFAKSIPGFVDLFLNDQ
VTLLKYGVHEAIFAMLPSLMNKDGLLVANGKGFVTREFLRSLRRPFSEIMEPKFEFAVKFNALELDDSDL
ALFVAAIILCGDRPGLINIKQVEEIQDSILQALDQHLLANHTDSKYLFPKLLNKMADLRQLVTENAMLVQ
KIKKTESETSLHPLLQEIYKDMY

>Q8AXU8_CHICK
MGPTQLSTQDHGKRVASVFEMEEEGLSLFSGSENPPKHAENPPLKRKKGPAPKMLGNEVCSVCGDKASGF
HYNVLSCEGCKGFFRRSVIKGAQYVCKNGGKCEMDMYMRRKCQECRLRKCQEAGMREQYVLSEEQIRLKK
LKKQEDDQARTVVVRPNPPQPPSPSHQLTPEQLNMIEKLVAAQQQCNQRSFTDRLKVTPWPQVPDPNNRE
ARQQRFAHFTELAIISVQEIVDFAKQLPGFRELTREDQIALLKTSTIEVMLLETSRRYNPEIESITFLKD
LSYNRDDFAKAGLQFEFINPIFEFSKGMNELQLNDAEYALLIAINIFSADRPSVQDQSLVERLQHTYVEA
LHSYICINGPNDHLMFPRMLMKLGSLRTLSSVHSEQVFALRLQDKKLPPLLSEIWDVHE

>O44337_9ACAR
MLSELQLQPLGRRSAAAAPSSDEVASMKPMLLQAAQGGAGGLAAGSPPALSPNLPSVVKVEPRLPSPCVG
GAASGDGGPVPPKRVRQDDAGAWISSPSSQMSVGSLSPPPPLLNGVANSSGLSPVSNCSSYDTYSPRGPC
KEEMSPSSGGGGLNGYFVDSFGDPKKKKGPAPRQQEELCLVCGDRASGYHYNALTCEGCKGFFRRSITKN

AVYQCKYGNNCDIDMYMRRKCQECRLKKCLSVGMRPECVVPEYQCAIKRESKKHQKDRPNSTTRESPSAL
MAPSSVGGVSPTSQPMGGGGSSLGSSNHEEDKKPVVLSPGVKPLSSSQEDLINKLVYYQQEFESPSEEDM
KKTTPFPLGDSEEDNQRRFQHITEITILTVQLIVEFSKRVPGFDTLAREDQITLLKACSSEVMMLRGARK
YDVKTDSIVFANNQPYTRDNYRSASVGDSADALFRFCRKMCQLRVDNAEYALLTAIVIFSERPSLVDPHK
VERIQEYYIETLRMYSENHRPPGKNYFARLLSILTELRTLGNMNAEMCFSLKVQNKKLPPFLAEIWDIQE

>Q68CY8_HUMAN
MSSPTTSSLDTPLPGNGPPQPGAPSSSPTVKEEGPEPWPGGPDPDVPGTDEASSACTTDWVIPDPEEEPE
RKRKKGPAPKMLGHELCRVCGDKASGFHYNVLSCEGCKGFFRRSVVRGGARRYACRGGGTCQMDAFMRRK
CQQCRLRKCKEAGMREQCVLSEEQIRKKKIRKQQQQESQSQSQSPVGPQGSSSSASGPGASPGGSEAGSQ
GSGEGEGVQLTAAQELMIQQLVAAQLQCNKRSFSDQPKVTPWPLGADPQSRDARQQRFAHFTELAIISVQ
EIVDFAKQVPGFLQLGREDQIALLKASTIEIMLLETARRYNHETECITFLKDFTYSKDDFHRAGLQVEFI
NPIFEFSRAMRRLGLDDAEYALLIAINIFSADRPTCRAGPRGGVAAALRGGAAVLHAHQEAADQLRFPRM
LMKLVSLRTLSSVHSEQVFALRLQDKKLPPLLSEIWDVHE

>Q5XI75_RAT
MVMQFQGLENPIQISLQHSHRLSGFASDRMSSKPAKGVLTEHAAGPLGQNLDLESYSPYNNVQFPQVQPQ
ISSSSYYSNLGFYPQQPEDWYSPGLYELRRMPTESVYQGETEVSEMPVTKKPRMAASSAGRIKGDELCVV
CGDRASGYHYNALTCEGCKGFFRRSITKNAVYKCKNGGNCVMDMYMRRKCQDCRLRKCREMGMLAECLLT
EIQCKSKRLRKNVKQHADQTVNEDSEGRDLRQVTSTTKLCREKTELTVDQQTLLDYIMDSYSKQRMPQEI
TNKILKEEFSAEENFLILTEMATSHVQILVEFTKRLPGFQTLDHEDQIALLKGSAVEAMFLRSAEIFNKK
LPAGHADLLEERIRKSGISDEYITPMFSFYKSVGELKMTQEEYALLTAIVILSPDRQYIKDREAVEKLQE
PLLDVLQKLCKIYQPENPQHFACLLGRLTELRTFNHHHAEMLMSWRVNDHKFTPLLCEIWDVQ

>NR1H4_RAT
MNLIGPSHLQATDEFALSENLFGVLTEHAAGPLGQNLDLESYSPYNNVQFPQVQPQISSSSYYSNLGFYP
QQPEDWYSPGLYELRRMPTESVYQGETEVSEMPVTKKPRMAASSAGRIKGDELCVVCGDRASGYHYNALT
CEGCKGFFRRSITKNAVYKCKNGGNCVMDMYMRRKCQDCRLRKCREMGMLAECLLTEIQCKSKRLRKNVK
QHADQTVNEDSEGRDLRQVTSTTKLCREKTELTVDQQTLLDYIMDSYSKQRMPQEITNKILKEEFSAEEN
FLILTEMATSHVQILVEFTKRLPGFQTLDHEDQIALLKGSAVEAMFLRSAEIFNKKLPAGHADLLEERIR
KSGISDEYITPMFSFYKSVGELKMTQEEYALLTAIVILSPDRQYIKDREAVEKLQEPLLDVLQKLCKIYQ
PENPQHFACLLGRLTELRTFNHHHAEMLMSWRVNDHKFTPLLCEIWDVQ

>ECR_HELVI
MSLGARGYRRCDTLADMRRRWYNNGPFQTLRMLEESSSEVTSSSALGLPPAMVMSPESLASPEIGGLELW
GYDDGITYSMAQSLGTCTMEQQQPQPQQQPQQTQPLPSMPLPMPPTTPKSENESMSSGREELSPASSVNG
CSTDGEARRQKKGPAPRQQEELCLVCGDRASGYHYNALTCEGCKGFFRRSVTKNAVYICKFGHACEMDIY
MRRKCQECRLKKCLAVGMRPECVVPENQCAMKRKEKKAQREKDKLPVSTTTVDDHMPPIMQCDPPPPEAA
RILECVQHEVVPRFLNEKLMEQNRLKNVPPLTANQKSLIARLVWYQEGYEQPSEEDLKRVTQSDEDDEDS
DMPFRQITEMTILTVQLIVEFAKGLPGFAKISQSDQITLLKACSSEVMMLRVARRYDAATDSVLFANNQA
YTRDNYRKAGMAYVIEDLLHFCRCMYSMMMDNVHYALLTAIVIFSDRPGLEQPLLVEEIQRYYLNTLRVY
ILNQNSASPRGAVIFGEILGILTEIRTLGMQNSNMCISLKLKNRKLPPFLEEIWDVADVATTATPVAAEA
PAPLAPAPPARPPATV

>O44336_9ACAR

MNYHPGYEDVPPPYTAAAPEDFWRKEEMSPSSGGGGLNGYFVDSFGDPKKKKGPAPRQQEELCLVCGDRA
SGYHYNALTCEGCKGFFRRSITKNAVYQCKYGNNCDIDMYMRRKCQECRLKKCLSVGMRPECVVPEYQCA
IKRESKKHQKDRPNSTTRESPSALMAPSSVGGVSPTSQPMGGGGSSLGSSNHEEDKKPVVLSPGVKPLSS
SQEDLINKLVYYQQEFESPSEEDMKKTTPFPLGDSEEDNQRRFQHITEITILTVQLIVEFSKRVPGFDTL
AREDQITLLKACSSEVMMLRGARKYDVKTDSIVFANNQPYTRDNYRSASVGDSADALFRFCRKMCQLRVD
NAEYALLTAIVIFSERPSLVDPHKVERIQEYYIETLRMYSENHRPPGKNYFARLLSILTELRTLGNMNAE
MCFSLKVQNKKLPPFLAEIWDIQE

>O02035_TENMO

MKRRWSGLQAVRVTPEESSSEVTSSSTTLVMSPANSLASTDIGDVDLEFWDLDLNGAKSRQRTHYHSKRI
WIPGHTIIASNHHLAKSDTSSMSGREDLSPSSLNGYSADSCDSKKKKGPTPRQQEELCLVCGDRASGYHY
NALTCEGCKGFFRRSITKNAVYQCKYGNNCEIDMYMRRKCQECRLKKCLSVGMRPECVVPEVQCAVKRKE
KKAQKEKDKPNSTTNGSPDVIKIEPELSDSEKTLTNGRNRISPEQEELILIHRLVYFQNEYEHPSEEDVK
RIINQPIDGEDQCEIRFRHTTEITILTVQLIVEFAKRLPGFDKLLQEDQIALLKACSSEVMMFRMARRYD
VQSDSILFVNNQPYPRDSYNLAGMGETIEDLLHFCRTMYSMKVDNAEYALLTAIVIFSERPSLIEGWKVE
KIQEIYLEALRAYVDNRRSPSRGTIFAKLLSVLTELRTLGNQNSEMCISLKLKNKKLPPFLDEIWDVDLK
A

>Q8K476_MESAU

MNLIEHSHLQAMDEFSLSENVFGVLTEQEAGALGQNLDLEAYSPYSNVQFPQVQPQISSSSYYSNLGFYP
QQPEEWYSPGIYELRRMPAETVYQGETEVSEMPVTKKPRMATAAAGRIKGDELCVVCGDRASGYHYNALT
CEGCKGFFRRSITKNAVYKCKNGGSCVMDMYMRRKCQECRLRKCKEMGMLAECLLTEIQCKSKRLRKNVK
QHADKTVNEDREGRDLRQVTSTTKSCREKTELTPDQQNLLDYITDSYSKQRMPQEITNKILKEEFSAEEN
FLILTEMATSHVQVLVEFTKKLPGFQTLDHEDQIALLKGSAVEAMFLRSAEIFNKKLPAGHADLLEERIR
NSGISAEYITPMFSFYKSIGELKMTQEEYALLTGIVILSPDRQYIKDREAVERLQEPLLEVLQKLCKIYQ
PENPQHFACLLGRLTELRTFNHHHAEMLMSWRVNDHKFTPLLCEIWDVQ

>Q8K473_MESAU

MVMQRQGLENPVQVSLHHSHRLSGFVPDVMSLKPAKGVLTEQEAGALGQNLDLEAYSPYSNVQFPQVQPQ
ISSSSYYSNLGFYPQQPEEWYSPGIYELRRMPAETVYQGETEVSEMPVTKKPRMATAAAGRIKGDELCVV
CGDRASGYHYNALTCEGCKGFFRRSITKNAVYKCKNGGSCVMDMYMRRKCQECRLRKCKEMGMLAECMYT
GLLTEIQCKSKRLRKNVKQHADKTVNEDREGRDLRQVTSTTKSCREKTELTPDQQNLLDYITDSYSKQRM
PQEITNKILKEEFSAEENFLILTEMATSHVQVLVEFTKKLPGFQTLDHEDQIALLKGSAVEAMFLRSAEI
FNKKLPAGHADLLEERIRNSGISAEYITPMFSFYKSIGELKMTQEEYALLTGIVILSPDRQYIKDREAVE
RLQEPLLEVLQKLCKIYQPENPQHFACLLGRLTELRTFNHHHAEMLMSWRVNDHKFTPLLCEIWDVQ

>Q8K474_MESAU

MVMQRQGLENPVQVSLHHSHRLSGFVPDVMSLKPAKGVLTEQEAGALGQNLDLEAYSPYSNVQFPQVQPQ
ISSSSYYSNLGFYPQQPEEWYSPGIYELRRMPAETVYQGETEVSEMPVTKKPRMATAAAGRIKGDELCVV
CGDRASGYHYNALTCEGCKGFFRRSITKNAVYKCKNGGSCVMDMYMRRKCQECRLRKCKEMGMLAECLLT
EIQCKSKRLRKNVKQHADKTVNEDREGRDLRQVTSTTKSCREKTELTPDQQNLLDYITDSYSKQRMPQEI
TNKILKEEFSAEENFLILTEMATSHVQVLVEFTKKLPGFQTLDHEDQIALLKGSAVEAMFLRSAEIFNKK
LPAGHADLLEERIRNSGISAEYITPMFSFYKSIGELKMTQEEYALLTGIVILSPDRQYIKDREAVERLQE

PLLEVLQKLCKIYQPENPQHFACLLGRLTELRTFNHHHAEMLMSWRVNDHKFTPLLCEIWDVQ

>Q8K475_MESAU
MNLIEHSHLQAMDEFSLSENVFGVLTEQEAGALGQNLDLEAYSPYSNVQFPQVQPQISSSSYYSNLGFYP
QQPEEWYSPGIYELRRMPAETVYQGETEVSEMPVTKKPRMATAAAGRIKGDELCVVCGDRASGYHYNALT
CEGCKGFFRRSITKNAVYKCKNGGSCVMDMYMRRKCQECRLRKCKEMGMLAECMYTGLLTEIQCKSKRLR
KNVKQHADKTVNEDREGRDLRQVTSTTKSCREKTELTPDQQNLLDYITDSYSKQRMPQEITNKILKEEFS
AEENFLILTEMATSHVQVLVEFTKKLPGFQTLDHEDQIALLKGSAVEAMFLRSAEIFNKKLPAGHADLLE
ERIRNSGISAEYITPMFSFYKSIGELKMTQEEYALLTGIVILSPDRQYIKDREAVERLQEPLLEVLQKLC
KIYQPENPQHFACLLGRLTELRTFNHHHAEMLMSWRVNDHKFTPLLCEIWDVQ

>HR3_GALME
MNNSQFHELFGSQWPPDQHGGHSSASTMLHQAQSPLPQGMRLKREPHTDVQGVMHNQMGMDITSGSVADS
TSPPPGSSEGMFGSSISGMFMDKKAANSIRAQIEIIPCKVCGDKSSGVHYGVITCEGCKGFFRRSQSTVV
NYQCPRNKACVVDRVNRNRCQYCRLQKCLKLGMSRDAVKFGRMSKKQREKVEDEVRYHRAQMRAQTDAAP
DSVYDAQQQTPSSSDQFHGHYNGYPGYRSPLSSYGYGGNAGPALTSNMNIQPQAPQQQPYDVSADYVDST
TAYEPKQTGGFLDADFIGHAEGDISKVLVKSLAEAHANTNPKLEYVHEMFRKPPDVSKLLFYNSMTYEEM
WLDCADKLTGMIQNIIEFAKLIPGFMKLSQDDQILLLKSGSFELAIVRLSRLIDVNREQVLYGDVVLPIR
ECVHARDPRDVTLVVGIFDAAKTIERLKLTETELALYQSLVLLWPERHGVRGNPEIQCLFNMSMAAMRHE
IESNHAPLKGDVTVLDTLLAKIPTFRELSLMHLEALCRFKAAHPHHVFPALYKELFSLDSVLDYTHG

>ECR_CHITE
MKTENLIVTTVKVEPLNYASQSFGDNNIYGGATKKQRLESDEWMNHNQTNMNLESSNMNHNTISGFSSPD
VNYEAYSPNSKLDDGNMSVHMGDGLDGKKSSSKKGPVPRQQEELCLVCGDRASGYHYNALTCEGCKGFFR
RSVTKNAVYCCKFGHECEMDMYMRRKCQECRLKKCLAVGMRPECVVPENQCAIKRKEKKAQKEKDKVPGI
VGSNTSSSSLLNQSLNNGSLKNLEISYREELLEQLMKCDPPPHPMQQLLPEKLLMENRAKGTPQLTANQV
AVIYKLIWYQDGYEQPSEEDLKRITTELEEEEDQEHEANFRYITEVTILTVQLIVEFAKGLPAFIKIPQE
DQITLLKACSSEVMMLRMARRYDHDSDSILFANNTAYTKQTYQLAGMEETIDDLLHFCRQMYALSIDNVE
YALLTAIVIFSDRPGLEKAEMVDIIQSYYTETLKVYIVNRHGGESRCSVQFAKLLGILTELRTMGNKNSE
MCFSLKLRNRKLPRFLEEVWDVGDVNNQTTATTNTENIVRERINRN

>Q9BMC6_HELAM
MNNNQFHDLFGSQWPPDQHGGHSSASTMLHQSQGLPQGMQLKREPHTDVQPVMHNQMGTDITSGSVADST
SPPPGSSDGMFGSSISGMFMDKKAANSIRAQIEIIPCKVCGDKSSGVHYGVITCEGCKGFFRRSQSTVVN
YQCPRNKACVVDRVNRNRCQYCRLQKCLKLGMSRDAVKFGRMSKKQREKVEDEVRFHRAQMRAQTDTAPD
SVYDAQQQTPSSSDQFHGHYNGYPGYGSPLSSYGYNNAGPALQSNMGGIQPQAPQQQPYDVSADYVDSTT
AYEPKQTEGFLDPDFISHAEGDISKVLVKSLAEAHANTNPKLEFIHEMFRKPQDVSKLLYYSSMTYEEMW
LDCADKLTGMIQNIIEFAKLIPGFMKLTQDDQILLLKSGSFELAIVRLSRLIDVNRDQVLYGDAVLSIRE
CVHARDPRDVALVVGIFDAAKTIARLKLTETELALYQSLVLLWPERHGVRGNPEIQCLFNMSMAAMRHEI
ETNHAPLKGDVTVLDTLLAKIPTFRELSLMHLEALCRFKAAHPHHVFPALYKELFSLDSVLDYTHG

>E75_GALME
MTLVMSPDSSYGRYDAPAPADNRIMSPVHKEREPELHIEFDGTTVLCRVCGDKASGFHYGVHSCEGCKGF
FRRSIQQKIQYRPCTKNQQCSILRINRNRCQYCRLKKCIAVGMSRDAVRFGRVPKREKARILAAMQSSTT

RAHEQAAAAAELDDGPRLLARVVRAHLDTCEFTRDRVAAMRNGARDCPTYSQPTLACPLNPAPELQSEKEF
SQRFAHVIRGVIDFAGLIPGFQLLTQDDKFTLLKSGLFDALFVRLICMFDAPLNSIICLNGQLMKRDSIQ
SGANARFLVDSTFKFAERMNSMNLTDAEIGLFCAIVLITPDRPGLRNVELVERMHSRLKSCLQTVIAQNR
SDGPGFLRELMDTLPDLRTLSTLHTEKLVVFRTEHKELLRQQMWVEDEGALWADSGADDSARSPIGSVSS
SESSETTGDCGTPLLAATLAGRRRLDSRGSVDEEALGVAHLAHNGLTVTPVRPPPRYRKLDSPTDSGIES
GNEKHERIVGPESGCSSPRSSLEEHSDDRRPIAPADDMPVLKRVLQAPPLYDASSLMDEAYKPHKKFRAM
RRDTWSEAEARPGRPTPSPQPPHHPHPASPAHPAHSPRPIRAPLSSTHSVLAKSLMEGPRMTPEQLKRTD
IIQQYMRRGETGAPTEGCPLRAGGLLTCFRGASPAPQPVIALQVDVAETDAPQPLNLSKKSPSPSPPPPP
PRSYMPPMLPA

>Q7Z2W0_HUMAN
MVMQFQGLENPIQISPHCSCTPSGFFMEMMSMKPAKGVLTEQVAGPLGQNLEVEPYSQYSNVQFPQAQPQ
ISSSSYYSNLGFYPQQPEEWYSPGIYELRRMPAETLYQGETEVAEMPVTKKPRMGASAGRIKGDELCVVC
GDRASGYHYNALTCEGCKGFFRRSITKNAVYKCKNGGNCVMDMYMRRKCQECRLRKCKEMGMLAECLLTE
IQCKSKRLRKNVKQHADQTVNEDSEGRDLRQVTSTTKSCREKTELTPDQQTLLHFIMDSYNKQRMPQEIT
NKILKEEFSAEENFLILTEMATNHVQVLVEFTKKLPGFQTLDHEDQIALLKGSAVEAMFLRSAEIFNKKL
PSGHSDLLEERIRNSGISDEYITPMFSFYKSIGELKMTQEEYALLTAIVILSPDRQYIKDREAVEKLQEP
LLDVLQKLCKIHQPENPQHFACLLGRLTELRTFNHHHAEMLMSWRVNDHKFTPLLCEIWDVQ

>NR1H4_HUMAN
MVMQFQGLENPIQISPHCSCTPSGFFMEMMSMKPAKGVLTEQVAGPLGQNLEVEPYSQYSNVQFPQVQPQ
ISSSSYYSNLGFYPQQPEEWYSPGIYELRRMPAETLYQGETEVAEMPVTKKPRMGASAGRIKGDELCVVC
GDRASGYHYNALTCEGCKGFFRRSITKNAVYKCKNGGNCVMDMYMRRKCQECRLRKCKEMGMLAECMYTG
LLTEIQCKSKRLRKNVKQHADQTVNEDSEGRDLRQVTSTTKSCREKTELTPDQQTLLHFIMDSYNKQRMP
QEITNKILKEEFSAEENFLILTEMATNHVQVLVEFTKKLPGFQTLDHEDQIALLKGSAVEAMFLRSAEIF
NKKLPSGHSDLLEERIRNSGISDEYITPMFSFYKSIGELKMTQEEYALLTAIVILSPDRQYIKDREAVEK
LQEPLLDVLQKLCKIHQPENPQHFACLLGRLTELRTFNHHHAEMLMSWRVNDHKFTPLLCEIWDVQ

>Q8SPF5_RABIT
MVMQFQGLENPVHISPHHSCSPPGFVTDMMSMKPAKGVLTEQVTSPLGQNLEVEPYSQYNNVQFPQVQPQ
ISSSSYYSNLGFYPQQPEEWYSPGIYELRRTPADTLYQGETEVADMPVPKKPRMGASAGRIKGDELCVVC
GDRASGYHYNALTCEGCKGFFRRSITKNAVYKCKNGGNCVMDMYMRRKCQECRLRKCKEMGMLAECLLTE
IQCKSKRLRKNVKQHTDQTTNDDSEGPDLRQVTSTTKSCREKTELTPDQQNLLHYITDSYNKQRMPQEVT
NKILKEEFSAEENFLILTEMATSHVQVLVEFTKKLPGFQTLDHEDQIALLKGSAVEAMFLRSAEIFNKKL
PVGHTDLLEERIRKSGISDEYITPMFSFYKSVGELKMTQEEYALLTAIVILSPDRQYIKDREAVEKLQEP
LLDVLQKLCKIHQPENPQHFACLLGRLTELRTFNHHHAEMLMSWRVNDHKFTPLLCEIWDVQ

>VDR_CHICK
MSELRGSWDEQQQSMAYLPDADMDTVAASTSLPDPAGDFDRNVPRICGVCGDRATGFHFNAMTCEGCKGF
FRRSMKRKAMFTCPFNGDCKITKDNRRHCQACRLKRCVDIGMMKEFILTDEEVQRKREMILKRKEEEALK
ESLKPKLSEEQQKVIDTLLEAHHKTFDTTYSDFNKFRPPVRSKFSSRMATHSSSVVSQDFSSEDSNDVFG
SDAFAAFPEPMEPQMFSNLDLSEESDESPSMNIELPHLPMLPHLADLVSYSIQKVIGFAKMIPGFRDLTA
EDQIALLKSSAIEVIMLRSNQSFTMEDMSWTCGSNDFKYKVSDVTQAGHSMDLLEPLVKFQVGLKKLNLH
EEEHVLLMAICILSPDRPGVQDTSLVESIQDRLSDILQTYIRCRHPPPGSRLLYAKMIQKLADLRSLNEE

HSKQYRCLSFQPEHSMQLTPLVLEVFGNEIS

>Q8WSA2_BOMMO
MSPDSSYGRYDVPTSVDHSLMSSMHKEREPELHIEFDGTTVLCRVCGDKASGFHYGVHSCEGCKGFFRRS
IQQKIQYRPCTKNQQCSILRINRNRCQYCRLKKCIAVGMSRDAVRFGRVPKREKARILAAMQQSSSSRAH
EQAAAAELDDAPRLLARVVRAHLDTCEFTRDRVASMRARARDCPTYSQPTLACPLNPAPELQSEKEFSQR
FAHVIRGVIDFAGLIPGFQLLTQDDKFTLLKSGLFDALFVRLICMFDAPLNSIICLNGQLMKRDSIQSGA
NARFLVDSTFKFAERMNSMNLTDAEIGLFCAIVLITPDRPGLRNIELVERMHSRLKACLQTVIAQNRPER
PGFLRELMDTLPDLRTLSTLHTEKLVVFRTEHKELLRQQMWNEEEGVSWADSVVEESARSPIGSVSSSES
GEVPSDCGTPLLAATLAGRRRLDSRGSVDEEALGVAHLAHNGLTVTPVRPPPRYRKLDSPTDSGIESGNE
KHERIIGPGSGCSSPRSSLEEHTEDRRPTAPADDMPVLKRVLQAPPLYGGTSTLMDETYKPHKKFRAMRR
DTGEAEARPVQPTPSPQPLHPHPASPAHPAHSPRPPRISLSSTHSVLAKSLMEGPRMTPEQLKRTDMIQQ
YMRRNEAGSSVEGCTLRTGGLLTCYRGASPAPPPVLALQVDVTDAPLNLSKKSPSPPRSYMPQMLEA

>Q9U5G4_BOMMO
MSPDSSYGRYDVPTSVDHSLMSSMHKEREPELHIEFDGTTVLCRVCGDKASGFHYGVHSCEGCKGFFRRS
IQQKIQYRPCTKNQQCSILRINRNRCQYCRLKKCIAVGMSRDAVRFGRVPKREKARILAAMQQSSSSRAH
EQAAAAELDDAPRLLARVVRAHLDTCEFTRDRVASMRARARDCPTYSQPTLACPLNPAPELQSEKEFSQR
FAHVIRGVIDFAGLIPGFQLLTQDDKFTLLKSGLFDALFVRLICMFDAPLNSIICLNGQLMKRDSIQSGA
NARFLVDSTFKFAERMNSMNLTDAEIGLFCAIVLITPDRPGLRNIELVERMHSRLKACLQAVIAQNRPER
PGFLRELMDTLPDLRTLSTLHTEKLVVFRTEHKELLRQQMWNEEEGVSWADSVVEESARSPSGSVSSSES
GEVPSDCGTPLLAATLAGRRRLDSRGSVDEEALGVAHLAHNGLTVTPVRPPPRYRKLDSPTDSGIESGNE
KHERIIGPGSGCSSPRSSLEEHTEDRRPTAPADDMPVLKRVLQAPPLYGGTSTLMDETYKPHKKFRAMRR
DTGEAEARPVQPTPSPQPLHPHPASPAHPAHSPRPPRISLSSTHSVLAKSLMEGPRMTPEQLKRTDMIQQ
YMRRNEAGSSVEGCPLRTGGLLTCYRGASPAPPPVLALQVDVTDAPLNLSKKSPSPPRSYMPQMLEA

>E75_CHOFU
MTLVMSPDSSYGRYDAQPPVDGGMVNPVHREREPELHIEFDGTTVLCRVCGDKASGFHYGVHSCEGCKGF
FRRSIQQKIQYRPCTKNQQCSILRINRNRCQYCRLKKCIAVGMSRDAVRFGRVPKREKARILAAMQQSSS
SRAHEQAAAAELDDAPRLLARVVRAHLDTCEFTRDRVAAMRARARDCPTYSQPTLACPLNPAPELQSEKE
FSQRFAHVIRGVIDFAGLIPGFQLLTQDDKFTLLKSGLFDALFVRLICMFDAPLNSIICLNGQLMKRDSI
QSGANARFLVDSTFKFAERMNSMNLTDAEIGLFCAIVLITPDRPGLRNIELVERMHARLKSCLQTVIAQN
RADRPGFLRELMDTLPDLRTLSTLHTEKLVVFRTEHKELLRQQMWGDEEVCPWADSGVDDSARSPLGSVS
SSESGEAPSDCGTPLLAATLAGRRRLDSRGSVDEEALGVAHLAHNGLTVTPVRPPPRYRKLDSPTDSGIE
SGNEKHERIVGPGSGCSSPRSSLEEHMEDRRPLAADDMPVLKRVLQAPPLYDASSLMDEAYKPHKKFRAM
RRDTGEAEARPMRPTPSPQPMHPHPGSPAHPAHPAHSPRPLRAPLSSTHSVLAKSLMEGPRMTPEQLKRT
DIIQQYMRRGEAGEECRAGLLLYRGASPLQVDVADAPQPLNLSKKSPSPPRSFMPPMLEA

>E75_MANSE
MTLVMSPDSSYGRYDAPTPTDVTSPVHREREPELHIEFDGTTVLCRVCGDKASGFHYGVHSCEGCKGFFR
RSIQQKIQYRPCTKNQQCSILRINRNRCQYCRLKKCIAVGMSRDAVRFGRVPKREKARILAAMQQSSTSR
AHEQAAAAELDDAPRLLARVVRAHLDTCEFTRDRVAAMRARARDCPTYSQPTLACPLNPAPELQSEKEFS
QRFAHVIRGVIDFAGLIPGFQLLTQDDKFTLLKSGLFDALFVRLICMFDAPLNSIICLNGQLMKRDSIQS
GANARFLVDSTFKFAERMNSMNLTDAEIGLFCAIVLITPDRPGLRNVELVERMHTRLKACLQTVIAQNRP

DRPGFLRELMDTLPDLRTLSTLHTEKLVVFRTEHKELLRQQMWSEEEAVSWVDSGADELARSPIGSVSSS
ESGEAVGDCGTPLLAATLAGRRRLDSRGSVDEEALGVAHLAHNGLTVTPVRQPPRYRKLDSPTDSGIESG
NEKHERIVGTGSGCSSPRSSLEEHNEDRRPPVSADDMPVLKRVLQAPPLYGGTPSLMDEAYRRHKKFRAL
RRDTGEAEARTVRPTPSPQPQHPHPANPAHPAHSPRPQRASLSSTHSVLAKSLMEGPRMTPEQLKRTDII
QQYMRRGESSAPAEGCPLRAGGLLTCYRGASPAPQPVLALQVDVTDAPLNLSKKSPSPPRTYMPQMLEA

>Q8JHU2_CHICK
MGSEMNLIGHPQLATADGFSLAEGPHLFGILSEPMSSPVQEADVSPYTQYNSVPFPQVQPQISSPPYYSN
LGFYPPQHEEWYSPGMYELRRIPSETFFTRETEIMDIPAAKKPRLGHSTGRMKGEELCVVCGDKASGYHY
NALTCEGCKGFFRRSITKNAVYKCKNGGNCEMDMYMRRKCQECRLRKCKQMGMLAECLLTEIQCKSKRLR
KNVKQLPDQTVNEDNEGHDMKQVTSTTKMYREKVEFTPEQQNLLDYIMDSYSKQQIPQEVSKKLLHEEFS
AEGNFLILTEMATSHVQVLVEFTKKLPGFQTLDHEDQIALLKGSAVEAMFLRSAEIFSRKLPTGHTVLLE
ERIRNSGISDEFITPMFNFYKSIGELKMTQEEYALLTAIVILSPDRQYIKDRESVERLQEPLLDILQKFC
KLHHPDNPQHFACLLGRLTELRTFNHHHAEMLMSWRVNDHKFTPLLCEIWDVQ

>Q8JJ28_XENLA
MRQWEDLEQTMANSYVTVSDAYCLAEPLSYYDVLPDHINYQLPESEFQTASCCQYTNMAYSPGLQSPSSQ
CHYTSYGLEAAYGDGQYLLSTCELSKPSTLMTHGVDDVYPSMKRPRGSHASIRMKGHEELCVVCGDKASG
YHYNALTCEGCKGFFRRSITKNAVYRCKNGGHCEMDMYMRRKCQECRLKKCKAVGMLAECLLTEVQCKSK
RLRKNCKQNNSILSNVKVEDDGSDSRHVSSTTKLTKLPSQLELTGEECKLIDHIVTAHQKCGIPLDDLKI
FLEESADPEEIFYHFSEAAMLHVQAFVEFTKRLPGFEMLDHEDQIALLKGSTVETMLLRSAQLYNQPATG
SSLQSTEGLARYQSHSVDFSQIQEFDRCPLYSLEANSHQVVSTSTTDLTEEFITPLFNFFRSMGSLNVTE
AEYALLSAVTVLFSDRPLLQNKPPVEKLQEPLLGILHKYSKLYHPEDPQHFARLIGRLTELRTLNHNHSE
VLISWKARDTKLTPLLYGFWNLQ

>Q6P7H5_XENLA
MANSYVTVSDAYCLAEPLSYYDVLPDHINYQLPESEFQTASCCQYTNMAYSPGLQSPSSQCHYTSYGLEA
AYGDGQYLLSTCELSKPSTLMTHGVDDVYPSMKRPRGSHASIRMKGHEELCVVCGDKASGYHYNALTCEG
CKGFFRRSITKNAVYRCKNGGHCEMDMYMRRKCQECRLKKCKAVGMLAECLLTEVQCKSKRLRKNCKQNN
SILSNVKVEDDGSDSRHVSSTTKLTKLPSQLELTGEECKLIDHIVTAHQKCGIPLDDLKIFLEESADPEE
IFYHFSEAAMLHVQAFVEFTKRLPGFEMLDHEDQIALLKGSTVETMLLRSAQLYNQPATGSSLQSTEGLA
RYQSHSVDFSQIQEFDRCPLYSLEANSHQVVSTSTTDLTEEFITPLFNFFRSMGSLNVTEAEYALLSAVT
VLFSDRPLLQNKPPVEKLQEPLLGILHKYSKLYHPEDPQHFARLIGRLTELRTLNHNHSEVLISWKARDT
KLTPLLYGFWNLQ

>VDR_COTJA
MVSISASGGYAMPCCCESQELQSSDMETPAVGTPEFDRNVPRICGVCGDRATGFHFNAMTCEGCKGFFRR
SMKRKAMFTCPFSGDCKITKDNRRHCQACRLKRCVDIGMMKEFILTDEEVQRKREMILKRKEEEALKESL
KPKLSEEQQKVINILLEAHHKTFDTTYSDFNKFRPPVRSKFSSSTATHSSSVVSQDFSSEDSNDVFGSDA
FGAFPEPMEPQMFSNLDLSEESDESPSMNIELPHLPMLPHLADLVSYSIQKVIGFAKMIPGFRDLTAEDQ
IALLKSSAIEVIMLRSNQSFTMEDMSWTCGSNDFKYKVSDVTQAGHSMDLLEPLVKFQVGLKKLNLHEEE
HVLLMAICILSPDRPGVQDTSLVESIQDRLSDTLQTYIRCRHPPPGSRLLYAKMIQKLADLRSLNEEHSK
QYRCLSFQPEHSMQLTPLVLEVFGNEIS

>Q27547_CHOFU
MMNNGQFPELFGSQWPPDHHGGHSSANTMLHPSMQSTMVKREPHTDVMHSQMGMGMQDIASVADSTSPPP
GGDGMFSTSMNAMFMDKKGANSIRAQIEIIPCKVCGDKSSGVHYGVITCEGCKGFFRRSQSTVVNYQCPR
NKACVVDRVNRNRCQYCRLQKCLKLGMRRDAVKFGRMSKKQREKVEDEGRFHRAQMRGQTDAPLDSVYDA
QQQTPSSSDQFHGHYNSYPGYASPISSYGYNNTAPSLTSTMIQPHADSAAIRRTSGLCGLTTAYEPKQGG
FLDADFNWSRRSDISKVLVKSLAEAHANTIPKLEYIHEMLRKPVDVAKQLFYSSMTYEAMWLDCADKLTT
MIQSIIEFAKLMPGFMKLTQDDQILLLKSGSFELAIVRLSRLIDVNRDTVLYGDVVLPIRDCVHARDPRD
MALIVGIFDAAKTIARLKLTETELALYQSLVHLWPEPHGVRGNHEIQCLFNMSMTAMRHEIETNHHPLKG
DVTVLDTLLAKLPTFRELSLMHLEALCRFKAAHPLHIFPALYKELFSLDSVLDYHV

>NR1D1_RAT
MEDSSRVSPSKGTSNITKLNGMVLLCKVCGDVASGFHYGVHACEGCKGFFRRSIQQNIQYKRCLKNENCS
IVRINRNRCQQCRFKKCLSVGMSRDAVRFGRIPKREKQRMLAEMQNAMNLANNQLSSLCPLETSPAPHPT
SGSVGPSPPPAPAPTPLVGFSQFPQQLTPPRSPSPEPTVEDVISQVARAHREIFTYAHDKLGTSPGNFNA
NHASGSPPATTPQCWESQGCPSTPNDNNLLAAQRHNEALNGLRQGPSSYPPTWPSGPAHHSCHQPNSNGH
RLCPTHVYSAPEGKAPANGLRQGNTKNVLLACPMNMYPHGRSGRTVQEIWEDFSMSFTPAVREVVEFAKQ
IPGFRDLSQHDQVTLLKAGTFEVLMVRFASLFNVKDQTVMFLSRTTYSLQELGAMGMGDLLNAMFDFSEK
LNSLALTEEELGLFTAVVLVSAEGSGMENSASVEQLQETLLGALRALVLKNRPSETSRFTKLLLKLPDLR
TLNNMHSEKLLSFRVDAQ

>HR3_DROME
MYTQRMFDMWSSVTSKLEAHANNLGQSNVQSPAGQNNSSGSIKAQIEIIPCKVCGDKSSGVHYGVITCEG
CKGFFRRSQSSVVNYQCPRNKQCVVDRVNRNRCQYCRLQKCLKLGMSRDAVKFGRMSKKQREKVEDEVRF
HRAQMRAQSDAAPDSSVYDTQTPSSSDQLHHNNYNSYSGGYSNNEVGYGSPYGYSASVTPQQTMQYDISA
DYVDSTTYEPRSTIIDPEFISHADGDINDVLIKTLAEAHANTNTKLEAVHDMFRKQPDVSRILYYKNLGQ
EELWLDCAEKLTQMIQNIIEFAKLIPGFMRLSQDDQILLLKTGSFELAIVRMSRLLDLSQNAVLYGDVML
PQEAFYTSDSEEMRLVSRIFQTAKSIAELKLTETELALYQSLVLLWPERNGVRGNTEIQRLFNLSMNAIR
QELETNHAPLKGDVTVLDTLLNNIPNFRDISILHMESLSKFKLQHPNVVFPALYKELFSIDSQQDLT

>Q6DGW7_BRARE
MNDWVGHDVNVVGPLQIPPNDAFPLSESSHFFDILAEQNSPLLQDQEVMPFTSYPSMQYTSVEPSMSSPS
YYSSQHCYSQYGAEEWYSPSAMFEMRKGPLDGGFDNELDESCPVIPTVCKRSRHAGHSGKSKGEELCVVC
GDKASGYHYNALTCEGCKVFFRRSITKNAVYKCKSGGNCEMDMYMRRKCQECRLRKCKEMGMLAECLLTE
IQCKSKRLRKNTKASSDGSIGDDVVDSRDPKQVVSTTKPSKENIELSQDQQALINYIVDAHNKHRIPQDM
AKKLLQEQFNAEENFLLLTEMATSHVQVLVEFTKNIPGFQSLDHEDQIALLKGSAVEAMFLRSAQVFSKK
LPNGHTEVLEDRIRRSGISEEFITPMFNFYKSIGELQMMQEEHALLTAITILSPDRPYVKDQQAVERLQE
PMLEVLRKICKLQHPQEPQHFARLLGRLTELRTLNHHHAEMLESWRMSDHKFNPLLCEIWDVQ

>Q95P94_BOMMO
MLNMFDMWNSVSKLEAQSNVQQSQQPHTSGGSIKAQIEIIPCKVCGDKSSGVHYGVITCEGCKGFFRRSQ
STVVNYQCPRNKACVVDRVNRNRCQYCRLQKCLKLGMSRDAVKFGRMSKKQREKVEDEVRYHKAQMRVQA
DAAPDSVYDAQQQTPSSSDQFHGHYNSYPGYGSPLSSYGYNNAGPALPSNMSGMQPQPPAQPPYEVSGDY
VDSTTTYEPKQTGFLDADFISHVEGDISKVLVKSLTEAHANTNPKLDYIHEMFGKPQDVSKLLFYNSMTY
EEMWLDCADKLTAMIQNIIEFAKLIPGFMKLTQDDQILLLKSGSFELAIVRLSRLIDVNRDQVLYGDVVL

PVRECVHARDPRDVALVQGIFEAAKSIARLKLTETELALYQSLVLLWPERHGVMGNSEIRCLFNMSMSAM
RHEIEVNHAPLKGDVTVLDTLLAKIPTFRDLSLMHLGALSRFKATHPHHVFPALYKELFSLDSVLDYTHG

>PXR_HUMAN
MEVRPKESWNHADFVHCEDTESVPGKPSVNADEEVGGPQICRVCGDKATGYHFNVMTCEGCKGFFRRAMK
RNARLRCPFRKGACEITRKTRRQCQACRLRKCLESGMKKEMIMSDEAVEERRALIKRKKSERTGTQPLGV
QGLTEEQRMMIRELMDAQMKTFDTTFSHFKNFRLPGVLSSGCELPESLQAPSREEAAKWSQVRKDLCSLK
VSLQLRGEDGSVWNYKPPADSGGKEIFSLLPHMADMSTYMFKGIISFAKVISYFRDLPIEDQISLLKGAA
FELCQLRFNTVFNAETGTWECGRLSYCLEDTAGGFQQLLLEPMLKFHYMLKKLQLHEEEYVLMQAISLFS
PDRPGVLQHRVVDQLQEQFAITLKSYIECNRPQPAHRFLFLKIMAMLTELRSINAQHTQRLLRIQDIHPF
ATPLMQELFGITGS

>Q811X2_MOUSE
MANTYVATSDGYYLAEPTQYYDILPEQFHYQLCDTDFQEPPYCQYSTAQFPPALQSPSLQSHFNTHGLDP
QYSGGSWCGLDARESGQSTYVVVHDDEDEFPGAQRCRATCSLRWKGQDDMLCMVCGDKASGYHYNALTCE
GCKGFFRRSITKNAVYSCKNGGHCEMDMYMRRKCQECRLKKCKAVGMLAECLLTEIQCKSKRLRKNFKHG
PALYPAIQVEDEGADTKHVSSSTRSGKGVQDNMTLTQEEHRLLNTIVTAHQKSMIPLGETSKLLQEGSNP
ELSFLRLSEVSVLHIQGLMKFTKGLPGFENLTTEDQAALQKASKTEVMFLHVAQLYGTMRPAKPSAGTLE
VHNPSADESVHSPENFLKEGYPSAPLTDITKEFIASLSYFYRRMSELHVSDTEYALLTATTVLFSDRPCL
KNKQHIENLQEPVLQLLFKFSKMYHPEDPQHFAHLIGRLTELRTLSHSHSEILRMWKTKDPRLVMLFSEK
WDLHSFS

>VDR_SAGOE
MEAMAASTSLPDPGDFDRNVPRICGVCGDRATGFHFNAMTCEGCKGFFRRSMKRKALFTCPFNGDCRITK
DNRRHCQACRLKRCVDIGMMKEFILTDEEVQRKREMILKRKEEEALKDSLRPKLSEEQQRIIAILLDAHH
KTYDPTYSDFCQFRPPVRVNDGGGSHPSRPNSRHTPSFSGDSSSCCSDHYITSPDMMDSSSFSNLDLSEE
DSDDPSLTLELSQLSMLPHLADLVSYSIQKVIGFAKMIPGFRDLTSEDQIVLLKSSAIEVIMLRSNESFT
MDDMSWTCGNPDYKYRISDVTKAGHNLELIEPLIKFQVGLKKLNLHEEEHVLLMAICIVSPDRPGVQDAA
LIEAIQDRLSNTLQTYIRCRHPPPGSHLLYAKMIQKLADLRSLNEEHSKQYRCLSFQPESSMKLTPLVLE
VFGNEIS

>E75B_DROME
MVCAMQEVAAVQHQQQQQLQLPQQQQQQQQTTQQQHATTIVLLTGNGGGNLHIVATPQQHQPMHQLHHQ
HQHQHQHQQQAKSQQLKQQHSALVKLLESAPIKQQQQTPKQIVYLQQQQQQPQRKRLKNEAAIVQQQQQT
PATLVKTTTTSNSNSNSNNTQTTNSISQQQQQHQIVLQHQQPAAAATPKPCADLSAKNDSESGIDEDCPNSD
EDCPNANPAGTSLEDSSYEQYQCPWKKIRYARELLKQRELEQQQTTGGSNAQQQVEAKPAAIPTSNIKQL
HCDSPFSAQTHKEIANLLRQQSQQQQVVATQQQQQQQQQHQHQQQRRDSSDSNCSLMSNSSNSSAGNCCT
CNAGDDQQLEEMDEAHDSGCDDELCEQHHQRLDSSQLNYLCQKFDEKLDTALSNSSANTGRNTPAVTANE
DADGFFRRSIQQKIQYRPCTKNQQCSILRINRNRCQYCRLKKCIAVGMSRDAVRFGRVPKREKARILAAM
QQSTQNRGQQRALATELDDQPRLLAAVLRAHLETCEFTKEKVSAMRQRARDCPSYSMPTLLACPLNPAPE
LQSEQEFSQRFAHVIRGVIDFAGMIPGFQLLTQDDKFTLLKAGLFDALFVRLICMFDSSINSIICLNGQV
MRRDAIQNGANARFLVDSTFNFAERMNSMNLTDAEIGLFCAIVLITPDRPGLRNLELIEKMYSRLKGCLQ
YIVAQNRPDQPEFLAKLLETMPDLRTLSTLHTEKLVVFRTEHKELLRQQMWSMEDGNNSDGQQNKSPSGS
WADAMDVEAAKSPLGSVSSTESADLDYGSPSSSQPQGVSLPSPPQQQPSALASSAPLLAATLSGGCPLRN

RANSGSSGDSGAAEMDIVGSHAHLTQNGLTITPIVRHQQQQQQQQIGILNNAHSRNLNGGHAMCQQQQQ
HPQLHHHLTAGAARYRKLDSPTDSGIESGNEKNECKAVSSGGSSSCSSPRSSVDDALDCSDAAANHNQVV
QHPQLSVVSVSPVRSPQPSTSSHLKRQIVEDMPVLKRVLQAPPLYDTNSLMDEAYKPHKKFRALRHREFE
TAEADASSSTSGSNSLSAGSPRQSPVPNSVATPPPSAASAAAGNPAQSQLHMHLTRSSPKASMASSHSVL
AKSLMAEPRMTPEQMKRSDIIQNYLKRENSTAASSTTNGVGNRSPSSSSTPPPSAVQNQQRWGSSSVITT
TCQQRQQSVSPHSNGSSSSSSSSSSSSSSSSSTSSNCSSSSASSCQYFQSPHSTSNGTSAPASSSSGSNS
ATPLLELQVDIADSAQPLNLSKKSPTPPPSKLHALVAAANAVQRYPTLSADVTVTASNGGSSVGGGESGR
QQQSAGECGLPQSGPERRRAQGNAGGVRAGGGRWFYAEKWERQRLGVAVQRSRKQDHLERRELN

>Q8IQS2_DROME
MVCAMQEVAAVQHQQQQQQLQLPQQQQQQQQTTQQQHATTIVLLTGNGGGNLHIVATPQQHQPMHQLHHQ
HQHQHQHQQQAKSQQLKQQHSALVKLLESAPIKQQQQTPKQIVYLQQQQQQPQRKRLKNEAAIVQQQQQT
PATLVKTTTTSNSNSNSNNTQTTNSISQQQQQHQIVLQHQQPAAAATPKPCADLSAKNDSESGIDEDSPNSD
EDCPNANPAGTSLEDSSYEQYQCPWKKIRYARELKQRELEQQQTTGGSNAQQQVEAKPAAIPTSNIKQLH
CDSPFSAQTHKEIANLLRQQSQQQQVVATQQQQQQQQQHQHQQQRRDSSDSNCSLMSNSSNSSAGNCCTC
NAGDDQQLEEMDEAHDSGCDDELCEQHHQRLDSSQLNYLCQKFDEKLDTALSNSSANTGRNTPAVTANED
ADGFFRRSIQQKIQYRPCTKNQQCSILRINRNRCQYCRLKKCIAVGMSRDAVRFGRVPKREKARILAAMQ
QSTQNRGQQRALATELDDQPRLLAAVLRAHLETCEFTKEKVSAMRQRARDCPSYSMPTLLACPLNPAPEL
QSEQEFSQRFAHVIRGVIDFAGMIPGFQLLTQDDKFTLLKAGLFDALFVRLICMFDSSINSIICLNGQVM
RRDAIQNGANARFLVDSTFNFAERMNSMNLTDAEIGLFCAIVLITPDRPGLRNLELIEKMYSRLKGCLQY
IVAQNRPDQPEFLAKLLETMPDLRTLSTLHTEKLVVFRTEHKELLRQQMWSMEDGNNSDGQQNKSPSGSW
ADAMDVEAAKSPLGSVSSTESADLDYGSPSSSQPQGVSLPSPPQQQPSALASSAPLLAATLSGGCPLRNR
ANSGSSGDSGAAEMDIVGSHAHLTQNGLTITPIVRHQQQQQQQQIGILNNAHSRNLNGGHAMCQQQQQH
PQLHHHLTAGAARYRKLDSPTDSGIESGNEKNECKAVSSGGSSSCSSPRSSVDDALDCSDAAANHNQVVQ
HPQLSVVSVSPVRSPQPSTSSHLKRQIVEDMPVLKRVLQAPPLYDTNSLMDEAYKPHKKFRALRHREFET
AEADASSSTSGSNSLSAGSPRQSPVPNSVATPPPSAASAAAGNPAQSQLHMHLTRSSPKASMASSHSVLA
KSLMAEPRMTPEQMKRSDIIQNYLKRENSTAASSTTNGVGNRSPSSSSTPPPSAVQNQQRWGSSSVITTT
CQQRQQSVSPHSNGSSSSSSSSSSSSSSSSSTSSNCSSSSASSCQYFQSPHSTSNGTSAPASSSSGSNSA
TPLLELQVDIADSAQPLNLSKKSPTPPPSKLHALVAAANAVQRYPTLSADVTVTASNGGPPSAAASPAPS
SSPPASVGSPNPGLSAAVHKVMLEA

>VDR_HUMAN
MEAMAASTSLPDPGDFDRNVPRICGVCGDRATGFHFNAMTCEGCKGFFRRSMKRKALFTCPFNGDCRITK
DNRRHCQACRLKRCVDIGMMKEFILTDEEVQRKREMILKRKEEEALKDSLRPKLSEEQQRIIAILLDAHH
KTYDPTYSDFCQFRPPVRVNDGGGSHPSRPNSRHTPSFSGDSSSSCSDHCITSSDMMDSSSFSNLDLSEE
DSDDPSVTLELSQLSMLPHLADLVSYSIQKVIGFAKMIPGFRDLTSEDQIVLLKSSAIEVIMLRSNESFT
MDDMSWTCGNQDYKYRVSDVTKAGHSLELIEPLIKFQVGLKKLNLHEEEHVLLMAICIVSPDRPGVQDAA
LIEAIQDRLSNTLQTYIRCRHPPPGSHLLYAKMIQKLADLRSLNEEHSKQYRCLSFQPECSMKLTPLVLE
VFGNEIS

>Q9I8F7_SALSA
MDNKMNSFDMKTLSTLDCPYLPSLEYSHNSPHHHHSPDRSHSCNHSPDRSHSFNHSPDRSHSFNHSPDRN
HSFNHSPDRSHSFNHSPDRSHSYNDTYSVYQGSVNDKPLSPSQSSDCSIVSLSRPRPHSNPPTYTDASSL
LNIDCRVCGDKASGFHYGVHVCEGCKGFFRRTVRLKLEYDHCDLHCRIHKKSRNKCQYCRFQKCLLVGMS

HDAIRFGRMPQVEREKLLQAEFMDVEPRNPESADLRALSRQLCLSYHRHFPLTKSKAKAILSGKTHGNSP
FVIHDMKSLTAGQYFINCRQLPVLERQRSVLPPEEPAEELELSVFRRIQFRSAEAVQEVTEFTKSIPGFT
ELDMNDQVILLKYGVIEVMTTMLAPLMNKDGTLFAYGQIFMTREFLKSLRKPFCEMMEPKFEFAAKFNLL
ELDDSDMALFFAVIILSGDRPGLVNVKPIEDLQETVLQALELQLKTIHPDCPQLFAKLLMQQ

>Q8SQ01_MACMU
MEVRPKEGWNHADFVYCEDTEFAPGKPTVNADEEVGGPQICRVCGDKATGYHFNVMTCEGCKGFFRRAMK
RNARLRCPFRKGACEITRKTRRQCQACRLRKCLESGMKKEMIMSDAAVEERRALIKRKKRERIGTQPPGV
QGLTEEQRMMIRELMDAQMKTFDTTFSHFKNFRLPGVLSSGCEMPESLQAPSREEAAKWNQVRKDLWSVK
VSVQLRGEDGSVWNYKPPADNGGKEIFSLLPHMADMSTYMFKGIINFAKVISYFRDLPIEDQISLLKGAT
FELCQLRFNTVFNAETGTWECGRLSYCLEDPAGGFQQLLLEPMLKFHYMLKKLQLHEEEYVLMQAISLFS
PDRPGVVQHRVVDQLQEQYAITLKSYIECNRPQPAHRFLFLKIMAMLTELRSINAQHTQRLLRIQDIHPF
ATPLMQELFGITGS

>Q9IB74_PAROL
MEPTVVSTSSLASDEFDRNMPRICGVCGDKATGFHFNAMTCEGCKGFFRRSMKRKATFTCPFNGSCTITK
DNRRHCQACRLKRCVDIGMMREFILTDEEVQRKKDLIQRRKDEEAQREAEREARRPRLTDEQSQVIAMLV
EAHHKTYDDSYSDFCRFRPPVREGPVTRSASRAASLHSLSDASSDSFSHSPESVDTKVNFNNLLMMYQEQ
GSSPDSSEEEGSSFSMLPHLADLVSYSIQKVIGFAKMIPGFRELTAEDQIALLKSSAIEVIMLRSNQSFN
LEDMSWSCGAPDFKYQISDVTKAGHTLELLEPLVKFQVGLKKLNLQEEEHVMLMAICLLSPDRPGVQDHA
RIEALQDRLSETLQAYIQLHHPGGRLLYAKMIQKLADLRSLNEEHSKQYRSLSFRPEHSMQLTPLVLEVS
GSEVS

>VDR_RAT
MEATAASTSLPDPGDFDRNVPRICGVCGDRATGFHFNAMTCEGCKGFFRRSMKRKALFTCPFNGDCRITK
DNRRHCQACRLKRCVDIGMMKEFILTDEEVQRKREMIMKRKEEEALKDSLRPKLSEEQQHIIAILLDAHH
KTYDPTYADFRDFRPPVRMDGSTGSYSPRPTLSFSGNSSSSSSDLYTTSLDMMEPSGFSNLDLNGEDSDD
PSVTLDLSPLSMLPHLADLVSYSIQKVIGFAKMIPGFRDLTSDDQIVLLKSSAIEVIMLRSNQSFTMDDM
SWDCGSQDYKYDVTDVSKAGHTLELIEPLIKFQVGLKKLNLHEEEHVLLMAICIVSPDRPGVQDAKLVEA
IQDRLSNTLQTYIRCRHPPPGSHQLYAKMIQKLADLRSLNEEHSKQYRSLSFQPENSMKLTPLVLEVFGN
EIS

>VDR_BOVIN
MAASTSLPDPGDFDRNVPRICGVCGDRATGFHFNAMTCEGCKGFFRRSMKRKALFTCPFNGDCRITKDNR
RHCQACRLKRCVDIGMMKEFILTDEEVQRKREMILKRKEEEALKDSLRPKLSEEQQRIIAILLDAHHKTY
DPTYSDFCQFRPPVRVNDGGGSHPSRPNSRHTPSFSGDSSSSCSDHCITSSDMMDSSSFSNLDLSEEDSD
DPSVTLELSQLSMLPHLADLVSYSIQKVIGFAKMIPGFRDLTSEDQIVLLKSSAIEVIMLRSNESFTMDD
MSWTCGNQDYKYRVSDVTKAGHSLELIEPLIKFQVGLKKLNLHEEEHVLLMAICIVSPDRPGVQDAALIE
AIQDRLSNTLQTYIRCRHPPPGSHLLYAKMIQKLADLRSLNEEHSKQYRCLSFQPECSMKLTPLVLEVFG
NEIS

>Q922X0_MOUSE
MEAMAASTSLPDPGDFDRNVPRICGVCGDRATGFHFNAMTCEGCKGFFRRSMKRKALFTCPFNGDCRITK
DNRRHCQACRLKRCVDIGMMKEFILTDEEVQRKREMIMKRKEEEALKDSLRPKLSEEQQHIIAILLDAHH

KTYDPTYADFRDFRPPIRADVSTGSYSPRPTLSFSGDSSSNSDLYTPSLDMMEPASFSTMDLNEEGSDDP
SVTLDLSPLSMLPHLADLVSYSIQKVIGFAKMIPGFRDLTSDDQIVLLKSSAIEVIMLRSNQSFTLDDMS
WDCGSQDYKYDITDVSRAGHTLELIEPLIKFQVGLKKLNLHEEEHVLLMAICIVSPDRPGVQDAKLVEAI
QDRLSNTLQTYIRCRHPPPGSHQLYAKMIQKLADLRSLNEEHSKQYRSLSFQPENSMKLTPLVLEVFGNE
IS

>VDR_MOUSE
MEAMAASTSLPDPGDFDRNVPRICGVCGDRATGFHFNAMTCEGCKGFFRRSMKRKALFTCPFNGDCRITK
DNRRHCQACRLKRCVDIGMMKEFILTDEEVQRKREMIMKRKEEEALKDSLRPKLSEEQQHIIAILLDAHH
KTYDPTYADFRDFRPPIRADVSTGSYSPRPTLSFSGDSSSNSDLYTPSLDMMEPASFSTMDLNEEGSDDP
SVTLDLSPLSMLPHLADLVSYSIQKVIGFAKMIPGFRDLTSDDQIVLLKSSAIEVIMLRSNQSFTMDDMS
WDCGSQDYKYDITDVSRAGHTLELIEPLIKFQVGLKKLNLHEEEHVLLMAICIVSPDRPGVQDAKLVEAI
QDRLSNTLQTYIRCRHPPPGSHQLYAKMIQKLADLRSLNEEHSKQYRSLSFQPENSMKLTPLVLEVFGNE
IS

>PXR_RAT
MRPEERWNHVGLVQREEADSVLEEPINVDEEDGGLQICRVCGDKANGYHFNVMTCEGCKGFFRRAMKRNV
RLRCPFRKGTCEITRKTRRQCQACRLRKCLESGMKKEMIMSDAAVEQRRALIKRKKREKIEAPPPGGQGL
TEEQQALIQELMDAQMQTFDTTFSHFKDFRLPAVFHSDCELPEVLQASLLEDPATWSQIMKDSVPMKISV
QLRGEDGSIWNYQPPSKSDGKEIIPLLPHLADVSTYMFKGVINFAKVISHFRELPIEDQISLLKGATFEM
CILRFNTMFDTETGTWECGRLAYCFEDPNGGFQKLLLDPLMKFHCMLKKLQLREEEYVLMQAISLFSPDR
PGVVQRSVVDQLQERFALTLKAYIECSRPYPAHRFLFLKIMAVLTELRSINAQQTQQLLRIQDTHPFATP
LMQELFSSTDG

>Q5RAP4_PONPY
MDRAPQRQHQASRELLAAKKTHTSQIEVIPCKICGDKSSGIHYGVITCEGCKGFFRRSQRCNAAYSCTRQ
QNCPIDRTSRNRCQHCRLQKCLALGMSRDAVKFGRMSKKQRDSLHAEVQKQLQQRQQQQEPVVKTPPAG
AQGADTLTYTLGLPDGQLPLGSSPDLPEASACPPGLLKASGSGPSYSNNLAKAGLNGASCHLEYSPERGK
AEGRESFYSTGSQLTPDRCGLRFEEHRHPGLGELGQGPDSYGSPSFRSTPEAPYASLTEIEHLVQSVCKS
YRETCQLRLEDLLRQRSNIFSREEVTGYQRKSMWEMWERCAHHLTEAIQYVVEFAKRLSGFMELCQNDQI
VLLKAGAVEVVLVRMCRAYNADNRTVFFEGKYGGMELFRALGCSELISSIFDFSHSLSALHFSEDEIALY
TALVLINAYRPGLQEKRKVEQLQYNLELAFHHHLCKTHRQSILAKLPPKGKLRSLCSQHVERLQIFQHLH
PIVVQATFPPLYKELFSTETESPVGLSK

>PPAS_XENLA
MKEEIPPRSPILDEQPSTPLEHQETSQSVDCKICGDRASGFHYGVHACEGCKGFFRRTIRMRLQYEHCDR
NCKIQKKNRNKCQYCRFNKCLSLGMSHNAIRFGRMPESEKRKLVQAPVSDSAAPDSPVSDLDVLSQLIHS
SYMNTFTMTKKRARDILTGRNSISPFVIHDMDTLWQAEQGTVWEQLPTQNLTGTEIGVHVFYRCQCTSVE
TVRALTDFAKRIPGFGTLYLNDQVTLLKYGVHEAIFCMLASLMNKDGLLVAGGRGFVTREFLRSLRQPFC
HIMEPKFHFASKFNALELNDSDLALFVASIILCGDRPGLINPSQVEDIQEGILGALRRHLKASHTDAPFL
FPKLLHKMADLRQLVTEHAELVQSIKRTESSAALHPLLQEIYRDMY

>Q9IB73_PAROL
MEPMTVTTSMVGPDEFDRNAPRICGVCGDKATGFHFNAMTCEGCKGFFRRSMKRKASFTCPFNGSCTITK

DNRRHCQACRLKRCIDIGMMKEFILTDEEVQREKEMILKRKEEEAAREAMRPRLNEEQARMISSLVEAHH
KTYDASYSDFSRFRPPVREGPVTRSASRAASLHSLSDASSDSFNHSPESVDTKMNFSNLLMMYQDGASSP
DSSEENTKLSMLPHLADLVSYSIQKVIGFAKMIPGFRDLTAEDQIALLKSSAIEIIMLRSNQSFSLEDMS
WSCGGPDFKYCINDVTKAGHTLELLEPLVKFQVGLKKLNLHEEEHVLLMGICLLSPDRPGVQDHARVEQL
QDRLPEALQAYIRINHPGGRLLYAKMIQKLADLRSLNEEHSKQYRSLSFQPEHSMQLTPLVLEVFGSEVS

>Q62702_RAT
MTKSHSGMTKFSGMVLLCKVCGDVASGFHYGVHACEGCKGFFRRSIQQNIQYKKCLKNENCSIMRMNRNR
CQQCRFKKCLSVGMSRDRVRFGRIPKREKQRMLIEMQSAMKTMMSTQFGGHLQSDTLAEPHEQSVPPAQE
QLRPSPSWSKKTSKAPLLLLILQRRKVIGMVTRAHKDTFLYNQEHRENSSESMPPHRGERIPRNVEQYNL
NHDHRGGGLHSHFPCSESQQHLSGQYKGRNMMHYPNGHTVCISNGHCVNFSSAYPQRVCDRIPVGGCSQT
ESRNSYLCSTGGRMHLVCPMSKSPYVDPQKSGHEIWEEFSMSFTPAVKEVVEFAKCIPGFRDLSQHDQVN
LLKAGTFEVLMVRFASLFDAKERTVTFLSGKKYSVDDLHSMGAGDLLSSMFEFSEKLNGLQLSDEEMSLF
TAVVLVSADRSGIENVNSVEALQETLIRGRGRTLIMKNHPNEASIFTKLLLKLPDLRSLNNMHSEELLAF
KVHP

>PXR_MOUSE
MRPEESWSRVGLVQCEEADSALEEPINVEEEDGGLQICRVCGDKANGYHFNVMTCEGCKGFFRRAMKRNV
RLRCPFRKGTCEITRKTRRQCQACRLRKCLESGMKKEMIMSDAAVEQRRALIKRKKREKIEAPPPGGQGL
TEEQQALIQELMDAQMQTFDTTFSHFKDFRLPAVFHSGCELPEFLQASLLEDPATWSQIMKDRVPMKISL
QLRGEDGSIWNYQPPSKSDGKEIIPLLPHLADVSTYMFKGVINFAKVISYFRDLPIEDQISLLKGATFEM
CILRFNTMFDTETGTWECGRLAYCFEDPNGGFQKLLLDPLMKFHCMLKKLQLHKEEYVLMQAISLFSPDR
PGVVQRSVVDQLQERFALTLKAYIECSRPYPAHRFLFLKIMAVLTELRSINAQQTQQLLRIQDSHPFATP
LMQELFSSTDG

>RORG_MOUSE
MDRAPQRHHRTSRELLAAKKTHTSQIEVIPCKICGDKSSGIHYGVITCEGCKGFFRRSQQCNVAYSCTRQ
QNCPIDRTSRNRCQHCRLQKCLALGMSRDAVKFGRMSKKQRDSLHAEVQKQLQQQQQEQVAKTPPAGSR
GADTLTYTLGLSDGQLPLGASPDLPEASACPPGLLRASGSGPPYSNTLAKTEVQGASCHLEYSPERGKAE
GRDSIYSTDGQLTLGRCGLRFEETRHPELGEPEQGPDSHCIPSFCSAPEVPYASLTDIEYLVQNVCKSFR
ETCQLRLEDLLRQRTNLFSREEVTSYQRKSMWEMWERCAHHLTEAIQYVVEFAKRLSGFMELCQNDQIIL
LTAGAMEVVLVRMCRAYNANNHTVFFEGKYGGVELFRALGCSELISSIFDFSHFLSALCFSEDEIALYTA
LVLINANRPGLQEKRRVEHLQYNLELAFHHHLCKTHRQGLLAKLPPKGKLRSLCSQHVEKLQIFQHLHPI
VVQAAFPPLYKELFSTDVESPEGLSK

>Q9NH86_AEDAE
MLRDAPNRSELEMAVSSTVFDSMLAQIEIIPCKVCGDKSSGVHYGVITCEGCKGFFRRSQSSVVNYQCPR
NKQCVVDRVNRNRCQYCRLQKCLKLGMSRDAVKFGRMSKKQREKVEDEVRFHRAQMRAQSDAAPDSSVFD
TQTPSSSDQLHHGGYNGYAYNNEVGYGSPYGYSTSVTPQQTMGYDISADYVDSTTTYEPRSTIIDSDFIS
GHTEGDINDVLIKTLAEAHANTNHKLEIVHDMFRKSQDVTRIMYYKNMSQEELWLDCAEKLTAMIQQIIE
FAKLIPGFMRLSQDDQILLLKTGSFELAIVRMSRLMDLSTNSVLYGDIMLPQEVFYTSDSFEMKLVACIF
ETAKSIAELKLTETELALYQSLVLLWPERNGVRGNTEIQRLFEMSMSAIRQEIEANHAPLKGDVTVLEIL
LNKIPTFRELSIMHMEALQKFKQDHPQYVFPALYKELFSIDSQQDLMT

>VDR_XENLA
MEFMAATTSIADTDMEFDKNVPRICGVCGDKATGFHFNAMTCEGCKGFFRRSMKRKAMFTCPFNGDCRIT
KDNRRHCQSCRLKRCVDIGMMKEFILTDEEVQRKRQMINKRKSEEALKESMRPKISDEQQKMIDILLEAH
RKTFDTTYSDFNKFRPPVRENVDPFRRITRSSSVHTQGSPSEDSDVFTSSPDSSEHGFFSASLFGQFEYS
SMGGKSGELSMLPHIADLVSYSIQKIIGFAKMIPGFRDLIAEDQIALLKSSVIEVIMLRSNQSFSLDDMS
WTCGSEDFKYKVDDVTQAGHNMELLEPLVKFQVGLKKLDLHEEEHVLLMAICILSPDRPGLQDKALVESI
QDRLSSTLQTYILCKHPPPGSRLLYAKMIQKLADLRSLNEEHSKQYRSISFLPEHSMKLTPLMLEVFSDE
IP

>Q98934_CHICK
MRAQIEVIPCKICGDKSSGIHYGVITCEGCKGFFRRSQQNNASYSCPRQRNCLIDRTNRNRCQHCRLQKC
LALGMSRDAVKFGRMSKKQRDSLYAEVQKHQQRLQEQRQQQSGEAEALARVYSNSISNGLGNLNNETGST
YSNGHVIDLPKSEGYYNVDSAQPSPDQSGLDMTGIKQIKQEPIYDLTSVPNLFTYSSFNNGQLAPGISMT
EIDRIAQNIIKSHLETCQYTMEELHQLAWQTHTYEEIKVYQSKTREALWQQCAIQITHAIQYVVEFAKRI
TGFMELCQNDQILLLKSGCLEVVLVRMCRAFNPLNNTVLFEGKYGGMQMFKALGSDDLVNEAFDFAKNLC
SLQLTEEEIALFSSAVLISPDRAWLIEPRKVQKLQEKIYFALQHVIQKNHLDDETLTKLIAKIPTITALC
NLHGEKLQVFKQSHPDIVNTLFPPLYKELFNPDSTTGCK

>RORB_MOUSE
MRAQIEVIPCKICGDKSSGIHYGVITCEGCKGFFRRSQQNNASYSCPRQRNCLIDRTNRNRCQHCRLQKC
LALGMSRDAVKFGRMSKKQRDSLYAEVQKHQQRLQEQRQQQSGEAEALARVYSSSISNGLSNLNTETGGT
YANGHVIDLPKSEGYYSIDSGQPSPDQSGLDMTGIKQIKQEPIYDLTSVPNLFTYSSFNNGQLAPGITMS
EIDRIAQNIIKSHLETCQYTMEELHQLAWQTHTYEEIKAYQSKSREALWQQCAIQITHAIQYVVEFAKRI
TGFMELCQNDQILLLKSGCLEVVLVRMCRAFNPLNNTVLFEGKYGGMQMFKALGSDDLVNEAFDFAKNLC
SLQLTEEEIALFSSAVLISPDRAWLIEPRKVQKLQEKIYFALQHVIQKNHLDDETLAKLIAKIPTITAVC
NLHGEKLQVFKQSHPDIVNTLFPPLYKELFNPDCAAVCK

>RORB_RAT
MRAQIEVIPCKICGDKSSGIHYGVITCEGCKGFFRRSQQNNASYSCPRQRNCLIDRTNRNRCQHCRLQKC
LALGMSRDAVKFGRMSKKQRDSLYAEVQKHQQRLQEQRQQQSGEAEALARVYSSSISNGLSNLNTETGGT
YANGHVIDLPKSEGYYNIDSGQPSPDQSGLDMTGIKQIKQEPIYDLTSVHNLFTYSSFNNGQLAPGITMS
EIDRIAQNIIKSHLETCQYTMEELHQLAWQTHTYEEIKAYQSKSREALWQQCAIQITHAIQYVVEFAKRI
TGFMELCQNDQILLLKSGCLEVVLVRMCRAFNPLNNTVLFEGKYGGMQMFKALGSDDLVNEAFDFAKNLC
SLQLTEEEIALFSSAVLISPDRAWLLEPRKVQKLQEKIYFALQHVIQKNHLDDETLAKLIAKIPTITAVC
NLHGEKLQVFKQSHPDIVNTLFPPLYKELFNPDCAAVCK

>RORB_HUMAN
MRAQIEVIPCKICGDKSSGIHYGVITCEGCKGFFRRSQQNNASYSCPRQRNCLIDRTNRNRCQHCRLQKC
LALGMSRDAVKFGRMSKKQRDSLYAEVQKHQQRLQEQRQQQSGEAEALARVYSSSISNGLSNLNNETSGT
YANGHVIDLPKSEGYYNVDSGQPSPDQSGLDMTGIKQIKQEPIYDLTSVPNLFTYSSFNNGQLAPGITMT
EIDRIAQNIIKSHLETCQYTMEELHQLAWQTHTYEEIKAYQSKSREALWQQCAIQITHAIQYVVEFAKRI
TGFMELCQNDQILLLKSGCLEVVLVRMCRAFNPLNNTVLFEGKYGGMQMFKALGSDDLVNEAFDFAKNLC
SLQLTEEEIALFSSAVLISPDRAWLIEPRKVQKLQEKIYFALQHVIQKNHLDDETLAKLIAKIPTITAVC
NLHGEKLQVFKQSHPEIVNTLFPPLYKELFNPDCATGCK

>Q7ZU39_BRARE

MYLMITAMKAQIESIPCKICGDKSSGIHYGVITCEGCKGFFRGSQQGTVSYSCPRQKSCLIDRTSRNRCQ
HCRLQKCLAVGMSRDAVKFGRMSKKQRDSLFAEVQKHRQQQQDDKTGDESEKNQESQAPGEAEPLTPSYA
LSSSGVTEIPDDLSGYVNGQTQEEGKADSAIGGFYLDIQPSPDQSGLDMDGIKLEPVCDLSSDSGLDQYC
CYSNGDASPPHDDLEHLSENICKSHLETCQYLREELQPSNWQTVLQSNLEAYQKKSQEDMWQLCAVKVTE
AVQYVVEFAKRIDGFMELCQNDQIVLLKAGSLEVVFVRMCRAYNSQNNTVFFDSKYAGPEVFKALGCDDL
ISSVFEFAKSLNSLQLSEDEIGLFSAYVLMSADRSWLQEKTRVEKLQQKIKIALQNLLQKNQRDEGILTK
LVCKVSTVRMLCRHHMEKLSAFRALYPEMVHTRFPPLYKELFGSDFEQILPQEA

>Q91839_XENLA

MWKVQETLVLEEEEEEEDASNSCGTGEDEDDGDPKICRACGDRATGYHFNAMTCEGCKGFFRRAVKRNLR
LSCPFQNSCVINKSNRRHCQACRLKKCLDIGMRKELIMSDAAVEQRRALIKRKHKLTKLPPTPPGASLTP
EQQHFLTQLVGAHTKTFDFNFTFSKNFRPIRRSSDPTQEPQATSSEAFLMLPHISDLVTYMIKGIISFAK
MLPYFKSLDIEDQIALLKGSVAEVSVIRFNTVFNSDTNTWECGPFTYDTEDMFLAGFRQLFLEPLVRIHR
MMRKLNLQSEEYAMMAALSIFASDRPGVCDWEKIQKLQEHIALTLKDFIDSQRPPSPQNRLLYPKIMECL
TELRTVNDIHSKQLLEIWDIQPDATPLMREVFGSPE

>Q8AVZ2_XENLA

MWKVQETLVLEEEEEEEDASNSCGTGEDEDDGDPKICRACGDRATGYHFNAMTCEGCKGFFRRAMKRNLR
LSCPFQNSCVINKSNRRHCQACRLKKCLDIGMRKELIMSDAAVEQRRALIKRKHKLTKSPPTPPGASLTP
EQQHFLTQLVGAHTKTFDFNFTFSKNFRPIRRSSDPTQEPQATSSEAFLMLPHISDLVTYMIKGIISFAK
MLPYFKSLDIEDQIALLKGSVAEVSVIRFNTVFNPDTNTWECGPFTYDTEDMFLAGFRQLFLEPLVRIHR
MMRKLNLQSEEYAMMAALSIFASDRPGVCDWEKIQKLQEHIALTLKDFIDSQRPPSLQNRLLYPKIMECL
TELRTVNDIHSKQLLEIWDIQPDATPLMREVFGSPE

>Q9TU02_RABIT

MGGKPTISADEEEGPQTCRVCGDKANGYHFNVLTCEGCKGFFRRTVKRNARLRCPFRKGACEITRKTRRQ
CQACRLRKCLESGMKKEMIMSDVAVAQRRALIKRKKRERMEAQPPGMQGLTGEQRMIIEELMDAQMKTFD
TTFSHFKNFRLPEVLGSGCEIPESLQALTEEEAGRWRQIQEELGTMKLSLQLRGEDGSVWNYTPPADRSG
KKLFSLLPHLADMSTYMFKGIINFAKVISYFRDLPIEDQISLLKGATLELCLLRFNTVFNAETGTWECGR
LSYCVEDPEGGFQQLLVDPLLKFHYMLKKLQLHKEEYVLMQAISLFSPDRPGVVQREVVDQLQERFAITL
KAYIECSRPQPTHRFLFLKIMAVLTELRTINAQHTQRLLRIQDTHPFATPLMRELFSTTDD

>Q9U5G3_BOMMO

MVRTMSCGAELRERHSVLVSMLEARRESSDSGCSSDDSSDVERDSTKCSCDRGFFRRSIQQKIQYRPCTK
NQQCSILRINRNRCQYCRLKKCIAVGMSRDAVRFGRVPKREKARILAAMQQSSSSRAHEQAAAAELDDAP
RLLARVVRAHLDTCEFTRDRVASMRARARDCPTYSQPTLACPLNPAPELQSEKEFSQRFAHVIRGVIDFA
GLIPGFQLLTQDDKFTLLKSGLFDALFVRLICMFDAPLNSIICLNGQLMKRDSIQSGANARFLVDSTFKF
AERMNSMNLTDAEIGLFCAIVLITPDRPGLRNIELVERMHSRLKACLQAVIAQNRPERPGFLRELMDTLP
DLRTLSTLHTEKLVVFRTEHKELLRQQMWNEEEGVSWADSVVEESARSPSGSVSSSESGEVPSDCGTPLL
AATLAGRRRLDSRGSVDEEALGVAHLAHNGLTVTPVRPPPRYRKLDSPTDSGIESGNEKHERIIGPGSGC
SSPRSSLEEHTEDRRPTAPADDMPVLKRVLQAPPLYGGTSTLMDETYKPHKKFRAMRRDTGEAEARPVQP
TPSPQPLHPHPASPAHPAHSPRPPRISLSSTHSVLAKSLMEGPRMTPEQLKRTDMIQQYMRRNEAGSSVE

GCPLRTGGLLTCYRGASPAPPPVLALQVDVTDAPLNLSKKSPSPPRSYMPQMLEA

>NHR23_CAEEL
MACKVTGPNRHLTSVLEMSSFVYWPRSRQQAHNFPMQAVEQKLADATRTLHAKSSLQPSLSIETPKSKEN
DESGCESSNCMFHPHTIKSEPNFCFAREFKSVPDDFRIGGGDLQMGNNSKRLTCVIDTNRVDMAGILPDN
MSFRGLPENKSLLVSAQIEVIPCKVCGDKSSGVHYGVITCEGCKGFFRRSQSSIVNYQCPRQKNCVVDRV
NRNRCQYCRLKKCIELGMSRDAVKFGRMSKKQREKVEDEVRMHKELAANGLGYQAIYGDYSPPPSHPSYC
FDQSMYGHYPSGTSTPVNGYSIAVAATPTTPMPQNMYGATPSSTNGTQYVAHQATGGSFPSPQVPEEDVA
TRVIRAFNQQHSSYTTQHGVCNVDPDCIPHLSRAGGWELFARELNPLIQAIIEFAKSIDGFMNLPQETQI
QLLKGSVFELSLVFAAMYYNVDAQAVCGERYSVPFACLIAEDDAEMQLIVEVNNTLQEIVHLQPHQSELA
LLAAGLILEQVSSSHGIGILDTATIATAETLKNALYQSVMPRIGCMEDTIHRIQDVETRIRQTARLHQEA
LQNFRMSDPTSSEKLPALYKELFTADRP

>Q6Q2C9_PLOIN
MVRAMSCGAELRERHSVLVSMLEARRESSDSGCSSDESSDIERDSNCRCDSQGFFRRSIQQKIQYRPCTK
NQQCSILRINRNRCQYCRLKKCIAVGMSRDAVRFGRVPKREKARILAAMQQSSTTRAHEALAAAELDDGP
RLLARVVRAHLDTCEFTRERVAAMRKRARDCPTYSQPTLACPLNPAPELQSEKEFSQRFAHVIRGVIDFA
GLIPGFQLLTQDDKFTLLKSGLFDALFVRLICMFDAPLNSIICLNGQLMKRDSIQSGANARFLVDSTFKF
AERMNSMNLTDAEIGLFCAIVLITPDRPGLRNIELVERMHSRLKSCLQTVIAQNRSDRPGFLRELMDTLP
DLRTLSTLHTEKLVVFRTEHKELLRQQMWGEEEGVSWADSGADESARSPIGSVSSSESGETTGDCGTPLL
AATLAGRRRLDSRGSVDEEALGVAHLAHNGLTVTPVRPPPRYRKLDSPTDSGIESGNEKHERIVGPGSGC
SSPRSSLEEHSEDRRPMAPADDMPVLKRVLQAPPLYDAGLLMDEAYKPHKKFRAMRRDTGEAEARPVRPT
PSPQPPQHPHPASPVHPAHSPRPMRASLSSTHSVLAKSLMEGPRMTPEQLKRTDTIQQYMRRGETGAAEG
CPLRAGGLLTCYRGASPAPQSVLALQVDVAEADAPQPLNLSKKSPSPSPPPPPPPRSYMPPMLPA

>Q8SXA4_DROME
MTAQIEIIPCKVCGDKSSGVHYGVITCEGCKGFFRRSQSSVVNYQCPRNKQCVVDRVNRNRCQYCRLQKC
LKLGMSRDAVKFGRMSKKQREKVEDEVRFHRAQMRAQSDAAPDSSVYDTQTPSSSDQLHHNNYNSGGYSN
NEVGYGSPYGYSASVTPQQTMQYDISADYVDSTTYEPRSTIIDPEFISHADGDINDVLIKTLAEAHANTN
TKLEAVHDMFRKQPDVSRILYYKNLGQEELWLDCAEKLTQMIQNIIEFAKLIPGFMRLSQDDQILLLKTG
SFELAIVRMSRLLDLSQNAVLYGDVMLPQEAFYTSDSEEMRLVSRIFQTAKSIAELKLTETELALYQSLV
LLWPERNGVRGNTEIQRLFNLSMNAIRQELETNHAPLKGDVTVLDTLLNNIPNFRDISILHMESLSKFKL
QHPNVVFPALYKELFSIDSQQDLT

>Q5REL4_PONPY
MLGHELCRVCGDKASGFHYNVLSCEGCKGFFRRSVVRGGARRYACRGGGTCQMDAFMRRKCQQCRLRKCK
EAGMREQCVLSEEQIRKKKIRKQQQQQSQSQSQSPAGPQGSCSSTSGPGASPGGSEAGSQGSGEGEGVQL
TAAQELMIQQLVAAQLQCNKRSFSDQPKVTEIVDFAKQVPGFLQLGREDQIALLKASTIEIMLLETARRY
NHETECITFLKDFTYSKDDFHRAGLQVEFINPIFEFSRAMRRLGLDDAEYALLIAINIFSADRPNVQEPG
RVEALQQPYVEALLSYTRIKRPQDQLRFPRMLMKLVSLRTLSSVHSEQVFALRLQDKKLPPLLSEIWDVH
E

>Q5R8V3_PONPY
MEAMAASTSLPDPGDFDRNVPRICGVCGDRATGFHFNAMTCEGCKGFFRRSMKRKALFTCPFNGDCRITK

DNRRHCQACRLKRCVDIGMMKEFILTDEEVQRKREMILKRKEEEALKDSLRPKLSEEQQRIIAILLDAHH
KTYDPTYSDFCQFRPPVRVNDGGGSHPSRPNSRHTPSFSGDSSSSCSDNCITSPDMMDSSSFSNLDLSEE
DSDDPSVTLELSQLSMLPHLADLVSYSIQKVIGFAKMIPGFRDLTSEDQIVLLKSSAIEVIMLRSNESFT
MDDMSWTCGNQDYKYRISDVTKAGHSLELIEPLIKFQVGLKKLNLHEEEHVLLMAICIVSPDRPGVQDAA
LIEAIQDRLSNTLQTYIRCRHPPPGSHLLYA

>NR1I3_MOUSE
MTAMLTLETMASEEEYGPRNCVVCGDRATGYHFHALTCEGCKGFFRRTVSKTIGPICPFAGRCEVSKAQR
RHCPACRLQKCLNVGMRKDMILSAEALALRRARQAQRRAEKASLQLNQQQKELVQILLGAHTRHVGPLFD
QFVQFKPPAYLFMHHRPFQPRGPVLPLLTHFADINTFMVQQIIKFTKDLPLFRSLTMEDQISLLKGAAVE
ILHISLNTTFCLQTENFFCGPLCYKMEDAVHAGFQYEFLESILHFHKNLKGLHLQEPEYVLMAATALFSP
DRPGVTQREEIDQLQEEMALILNNHIMEQQSRLQSRFLYAKLMGLLADLRSINNAYSYELQRLEELSAMT
PLLGEICS

>Q811W9_MOUSE
MANTYVATSDGYYLAEPTQYYDILPEQFHYQLCDTDFQEPPYCQYSTAQFPPALQSPSLQSHFNTHGLDP
QYSGGSWCGLDARESGFFRRSITKNAVYSCKNGGHCEMDMYMRRKCQECRLKKCKAVGMLAECLLTEIQC
KSKRLRKNFKHGPALYPAIQVEDEGADTKHVSSSTRSGKGVQDNMTLTQEEHRLLNTIVTAHQKSMIPLG
ETSKLLQEGSNPELSFLRLSEVSVLHIQGLMKFTKGLPGFENLTTEDQAALQKASKTEVMFLHVAQLYGG
KDSTSGSTMRPAKPSAGTLEVHNPSADESVHSPENFLKEGYPSAPLTDITKEFIASLSYFYRRMSELHVS
DTEYALLTATTVLFSDRPCLKNKQHIENLQEPVLQLLFKFSKMYHPEDPQHFAHLIGRLTELRTLSHSHS
EILRMWKTKDPRLVMLFSEKWDLHSFS

>NR1I3_HUMAN
MASREDELRNCVVCGDQATGYHFNALTCEGCKGFFRRTVSKSIGPTCPFAGSCEVSKTQRRHCPACRLQK
CLDAGMRKDMILSAEALALRRAKQAQRRAQQTPVQLSKEQEELIRTLLGAHTRHMGTMFEQFVQFRPPAH
LFIHHQPLPTLAPVLPLVTHFADINTFMVLQVIKFTKDLPVFRSLPIEDQISLLKGAAVEICHIVLNTTF
CLQTQNFLCGPLRYTIEDGARVSPTVGFQVEFLELLFHFHGTLRKLQLQEPEYVLLAAMALFSPDRPGVT
QRDEIDQLQEEMALTLQSYIKGQQRRPRDRFLYAKLLGLLAELRSINEAYGYQIQHIQGLSAMMPLLQEI
CS

>NR1I3_MACMU
MASREDELRNCVVCGDQATGYHFNALTCEGCKGFFRRTVSKSIGPTCPFAGSCEVSKIQRRHCPACRLQK
CLDAGMRKDMILSAEALALRRAKQAQRRAQQTPMQLSNEQEELIQTLLGAHTRHMGTMFEQFVQFRPPAH
LFIHHQPLPTLAPVLPLVTHFADVNTFMVQQVIKFTKDLPVFRSLPIEDQISLLKGAAVEICHIVLNTTF
CLQTQNFLCGPLRYTIEDAARVSPAVGFQVEFLELLFHFHGTLRKLQLQEPEYVLLAAMALFSPDRPGVT
QRHEIDQLQEEMALTLQSYIKGQQQRPRDRFLYAKLLGLLAELRSINEAYGYQIQHIQGLSAMMPLLQEI
CS

>CNR14_CAEEL
MSFETKPNYLLLTNPDTPLSVCTSPYYSPSGKTASIPSSEASKPEGTNGQWSHLPTGATYVTDEFSSEFQ
IQNGSTAAQSGNANNYADPLSHRRYFNNVNGYNHHQFYDTASQASVSSPATSVTSSLSPPDSLSNGHTTQ
RHHIGKAISFCKVCGDKASGYHYGVTSCEGCKGFFRRSIQRKIDYRCLKQQVCEIKRESRNRCQYCRFKK
CLDSGMSKDSVRQMKFRNAMRDDKSPDSVFVPEISTLERQEEVDAVYEAVLRAHTTFSFYTDIKIRSIVA

RPFNVRINEDSKMNRLNAWQIYAHEIDVDIKEVVNFVKEIPKFNFINGNDKAVLLRKNAFPLYLLRIVRG
MSNRGLMLRDGRLIDFKSLQLLYGSLADEMLAFANHIITIGCTDGDIALFIVLILCQPLTTEQQFSTNFK
SQLQLLEMFDFYKKVLFQKMTCRIDGCDTYKQLMKCIHELNRLNELHKQQLNILRENLSFLNLPPLVVEM
FQLSTLPLPVNHNNQENQYTPAPEHQSPQPQQPTPNQQQTPVHC

>Q9U3F1_CAEEL
MSFETKPNYLLLTNPDTPLSVCTSPYYSPSGKTASIPSSEASKPEGTNGQWSHLPTGATYVTDEFSSEFQ
IQNGSTAAQSGNANNYADPLSHRRYFNNVNGYNHHQFYDTASQASVSSPATSVTSSLSPPDSLSNGHTTQ
RHHIGKAISFCKVCGDKASGYHYGVTSCEGCKGFFRRSIQRKIDYRCLKQQVCEIKRESRNRCQYCRFKK
CLDSGMSKDSVRQMKFRNAMRDDKSPDSVFVPEISTLERQEEVDAVYEAVLRAHTTFSFYTDIKIRSIVA
RPFNVRINEDSKMNRLNAWQIYAHEIDVDIKEVVNFVKEIPKFNFINGNDKAVLLRKNAFPLYLLRIVRG
MSNRGLMLRDGRLIDFKSLQLLYGSLADEMLAFANHIITIGCTDGDIALFIVLILCQPLTTEQQFSTNFK
SQLQLLEMFDFYKKVLFQKMTCRIDGCDTYKQLMKCIHELNRLNELHKQQLNILRENLSFLNLPPLVVEM
FQLSTLPLPVNHNNQESIRALNHSSQRQINNRLPFTASHLCQILIRFHSNTIHLQIISL

>NR1I3_PHOSI
MASGEDGPRSCMVCGDRATGYHFHALTCEGCKGFFRRTVSKNTGLTCPFAGSCKVNKAQRRHCPACRLQK
CLDAGMKKEMILSAEALAQRRAKQAQRRAQWAAVQLSKGQQELVQTLLGAHARHVGTMFDQFVQFRPPAH
LFIHHQRLPIPLPPLPLLEHFAEVNTFMVQQVIKFTKDLPLFRSLPMEDQISLLKGAAVEICHIALNTTF
CLQTRNFLCGPLRYTLEDGVHVGFQEEFLELLFRFHATLRRFQLQEPEYVLMAAMALFSPDRPGVTQKEE
IDRLQEMMALTLQSYIKGQPPRHRDRFLYAKLLGLLAELRSINNAYGYQIQHIQGLSAMMPLLQEICS

>NR1I3_CALUR
MASGEDGPRSCMVCGDRATGYHFHALTCEGCKGFFRRTVSKNTGLTCPFAGNCKVNKAQRRHCPACRLQK
CLDAGMKKEMILSAEALVQRRAKQAQRRAQWAPVQLSKGQQELVQTLLGAHARHVGTMFDQFVQFRPPAH
LFIHHQRLPIPVPALPLLKHFAEVNTFMVQEVIKFTKDLPLFRSLPMEDQISLLKGAAVEICHIALNTTF
CLQTRNFLCGPLCYALEDGVHVGFQEEFLELLFRFHATLRRLQLQEPEYVLMAAMALFSPDRPGVTRREE
IDRLQEVTALTLQSYIKGQPPRPRDRFLYAKLLGLLAELRSIDNAYGYQIQHIQGLSAMMPLLQEICS

>Q811X1_MOUSE
MANTYVATSDGYYLAEPTQYYDILPEQFHYQLCDTDFQEPPYCQYSTAQFPPALQSPSLQSHFNTHGLDP
QYSGGSWCGLDARESGQSTYVVVHDDEDEFPGAQRCRATCSLRWKGQDDMLCMVCGDKASGYHYNALTCE
GCKGFFRRSITKNAVYSCKNGGHCEMDMYMRRKCQECRLKKCKAVGMLAECLLTEIQCKSKRLRKNFKHG
PALYPAIQVEDEGADTKHVSSSTRSGKGVQDNMTLTQEEHRLLNTIVTAHQKSMIPLGETSKLLQEGSNP
ELSFLRLSEVSVLHIQGLMKFTKGLPGFENLTTEDQAALQKASKTEVMFLHVAQLYGGKDSTSGSTMRPA
KPSAGTLEVHNPSADESVHSPENFLKEGYPSAPLTEE

>Q811X0_MOUSE
MANTYVATSDGYYLAEPTQYYDILPEQFHYQLCDTDFQEPPYCQYSTAQFPPALQSPSLQSHFNTHGLDP
QYSGGSWCGLDARESGQSTYVVVHDDEDEFPGAQRCRATCSLRWKGQDDMLCMVCGDKASGYHYNALTCE
GCKGFFRRSITKNAVYSCKNGGHCEMDMYMRRKCQECRLKKCKAVGMLAECLLTEIQCKSKRLRKNFKHG
PALYPAIQVEDEGADTKHVSSSTRSGKGVQDNMTLTQEEHRLLNTIVTAHQKSMIPLGETSKLLQEGSNP
ELSFLRLSEVSVLHIQGLMKFTKGLPGFENLTTEDQAALQKASKTEVMFLHVAQLYGTMRPAKPSAGTLE
VHNPSADESVHSPENFLKEGYPSAPLTEE

>Q6GZ84_HUMAN

MLPKRSRRTVSKSIGPTCPFAGSCEVSKTQRRHCPACRLQKCLDAGMRKDMILSAEALALRRAKQAQRRA
QQTPVQLSKEQEELIRTLLGAHTRHMGTMFEQFVQFRPPAHLFIHHQPLPTLAPVLPLVTHFADINTFMV
LQVIKFTKDLPVFRSLPIEDQISLLKGAAVEICHIVLNTTFCLQTQNFLCGPLRYTIEDGARVGFQVEFL
ELLFHFHGTLRKLQLQEPEYVLLAAMALFSPAPYLTDRPGVTQRDEIDQLQEEMALTLQSYIKGQQRRPR
DRFLYAKLLGLLAELRSINEAYGYQIQHIQGLSAMMPLLQEICS

>Q6GZ68_HUMAN

MLPKRSRRTVSKSIGPTCPFAGSCEVSKTQRRHCPACRLQKCLDAGMRKDMILSAEALALRRAKQAQRRA
QQTPVQLSKEQEELIRTLLGAHTRHMGTMFEQFVQFRPPAHLFIHHQPLPTLAPVLPLVTHFADINTFMV
LQVIKFTKDLPVFRSLPIEDQISLLKGAAVEICHIVLNTTFCLQTQNFLCGPLRYTIEDGARVGFQVEFL
ELLFHFHGTLRKLQLQEPEYVLLAAMALFSPDRPGVTQRDEIDQLQEEMALTLQSYIKGQQRRPRDRFLY
AKLLGLLAELRSINEAYGYQIQHIQGLSAMMPLLQEICS

>Q8IQS1_DROME

MGEELPILKGILKGNVNYHNAPVRFGRVPKREKARILAAMQQSTQNRGQQRALATELDDQPRLLAAVLRA
HLETCEFTKEKVSAMRQRARDCPSYSMPTLLACPLNPAPELQSEQEFSQRFAHVIRGVIDFAGMIPGFQL
LTQDDKFTLLKAGLFDALFVRLICMFDSSINSIICLNGQVMRRDAIQNGANARFLVDSTFNFAERMNSMN
LTDAEIGLFCAIVLITPDRPGLRNLELIEKMYSRLKGCLQYIVAQNRPDQPEFLAKLLETMPDLRTLSTL
HTEKLVVFRTEHKELLRQQMWSMEDGNNSDGQQNKSPSGSWADAMDVEAAKSPLGSVSSTESADLDYGSP
SSSQPQGVSLPSPPQQQPSALASSAPLLAATLSGGCPLRNRANSGSSGDSGAAEMDIVGSHAHLTQNGLT
ITPIVRHQQQQQQQQQIGILNNAHSRNLNGGHAMCQQQQQHPQLHHHLTAGAARYRKLDSPTDSGIESGN
EKNECKAVSSGGSSSCSSPRSSVDDALDCSDAAANHNQVVQHPQLSVVSVSPVRSPQPSTSSHLKRQIVE
DMPVLKRVLQAPPLYDTNSLMDEAYKPHKKFRALRHREFETAEADASSSTSGSNSLSAGSPRQSPVPNSV
ATPPPSAASAAAGNPAQSQLHMHLTRSSPKASMASSHSVLAKSLMAEPRMTPEQMKRSDIIQNYLKRENS
TAASSTTNGVGNRSPSSSSTPPPSAVQNQQRWGSSSVITTTCQQRQQSVSPHSNGSSSSSSSSSSSSSSS
SSTSSNCSSSSASSCQYFQSPHSTSNGTSAPASSSSGSNSATPLLELQVDIADSAQPLNLSKKSPTPPPS
KLHALVAAANAVQRYPTLSADVTVTASNGGPPSAAASPAPSSSPPASVGSPNPGLSAAVHKVMLEA

>Q76FN2_RAT

MTATLTLETMTSEEEYGPRNCVVCGDRATGYHFHALTCEGCKGFFRRTVSKTIGPICPFAGRCEVSKAQR
RHCPACRLQKCLNVGMRKDMILSAEALALRRARQARRRAQKASLQLSQQQKELIQTLLGAHTRHVGPMFD
QFVQFRPPAYLFSHHRPFQPLAPVLPLLTHFADINTFMVQQIIKFTKDLPLFRSLTMEDQISLLKGAAVE
ILHISLNTTFCLQTQNFFCGPLCYKMEDAVHGETVVQNTVCTLWYEVWSNGQEAAQPTISHSRVPVRVFG
VDHPLPQNPEKIAAPGARVCAHGCHGSLLS

(2) 127 NR2

>RXRA_RAT

MDTKHFLPLDFSTQVNSSSLSSPTGRGSMAAPSLHPSLGPGLGSPLGSPGQLHSPISTLSSPINGMGPPF
SVISSPMGPHSMSVPTTPTLGFETGSPQLNSPMNPVSSSEDIKPPLGLNGVLKVPAHPSGNMSSFTKHIC
AICGDRSSGKHYGVYSCEGCKGFFKRTVRKDLTYTCRDNKDCLIDKRQRNRCQYCRYQKCLAMGMKREAV
QEERQRGKDRNENEVESTSSANEDMPVEKILEAELAVEPKTETYVEANMGLNPSSPNDPVTNICQAADKQ
LFTLVEWAKRIPHFSELPLDDQVILLRAGWNELLIASFSHRSIAVKDGILLATGLHVHRNSAHSAGVGAI

FDRVLTELVSKMRDMQMDKTELGCLRAIVLFNPDSKGLSNPAEVEALREKVYASLEAYCKHKYPEQPGRF
AKLLLRLPALRSIGLKCLEHLFFFKLIGDTPIDTFLMEMLEAPHQTT

>RXRA_MOUSE
MDTKHFLPLDFSTQVNSSSLNSPTGRGSMAVPSLHPSLGPGIGSPLGSPGQLHSPISTLSSPINGMGPPF
SVISSPMGPHSMSVPTTPTLGFGTGSPQLNSPMNPVSSTEDIKPPLGLNGVLKVPAHPSGNMASFTKHIC
AICGDRSSGKHYGVYSCEGCKGFFKRTVRKDLTYTCRDNKDCLIDKRQRNRCQYCRYQKCLAMGMKREAV
QEERQRGKDRNENEVESTSSANEDMPVEKILEAELAVEPKTETYVEANMGLNPSSPNDPVTNICQAADKQ
LFTLVEWAKRIPHFSELPLDDQVILLRAGWNELLIASFSHRSIAVKDGILLATGLHVHRNSAHSAGVGAI
FDRVLTELVSKMRDMQMDKTELGCLRAIVLFNPDSKGLSNPAEVEALREKVYASLEAYCKHKYPEQPGRF
AKLLLRLPALRSIGLKCLEHLFFFKLIGDTPIDTFLMEMLEAPHQAT

>RXRA_HUMAN
MDTKHFLPLDFSTQVNSSLTSPTGRGSMAAPSLHPSLGPGIGSPGQLHSPISTLSSPINGMGPPFSVISS
PMGPHSMSVPTTPTLGFSTGSPQLSSPMNPVSSSEDIKPPLGLNGVLKVPAHPSGNMASFTKHICAICGD
RSSGKHYGVYSCEGCKGFFKRTVRKDLTYTCRDNKDCLIDKRQRNRCQYCRYQKCLAMGMKREAVQEERQ
RGKDRNENEVESTSSANEDMPVERILEAELAVEPKTETYVEANMGLNPSSPNDPVTNICQAADKQLFTLV
EWAKRIPHFSELPLDDQVILLRAGWNELLIASFSHRSIAVKDGILLATGLHVHRNSAHSAGVGAIFDRVL
TELVSKMRDMQMDKTELGCLRAIVLFNPDSKGLSNPAEVEALREKVYASLEAYCKHKYPEQPGRFAKLLL
RLPALRSIGLKCLEHLFFFKLIGDTPIDTFLMEMLEAPHQMT

>Q5TJF7_CANFA
MSWAARPPFLPQRHAAGQCGPVGVRKEMHCGVASRWRRRRPWLDPAAAAAAAAAAGEQQTPEPEPGEAGR
DGMGDSGRDSRSPDSSSPNPLSQGAPPPSPPGLPLPPSSAPTLGGSGAPPPPPMPPPQLGSPFPVISSSM
GSPGLPPPAPPGFSGPVSSPQINSTVSLPGGGSGPPEDVKPPVLGVRGLHCPPPPGGPGAGKRLCAICGD
RSSGKHYGVYSCEGCKGFFKRTIRKDLTYSCRDNKDCTVDKRQRNRCQYCRYQKCLATGMKREAVQEERQ
RGKDKDGDGEGAGGAPEEMPVDRILEAELAVEQKSDQGVEGPGGTGGSGSSPNDPVTNICQAADKQLFTL
VEWAKRIPHFSSLPLDDQVILLRAGWNELLIASFSHRSIDVRDGILLATGLHVHRNSAHSAGVGAIFDRV
LTELVSKMRDMRMDKTELGCLRAIILFNPDAKGLSNPSEVEVLREKVYASLETYCKQKYPEQQGRFAKLL
LRLPALRSIGLKCLEHLFFFKLIGDTPIDTFLMEMLEAPHQLA

>RXRB_MOUSE
MSWATRPPFLPPRHAAGQCGPVGVRKEMHCGVASRWRRRRPWLDPAAAAAAAGEQQALEPEPGEAGRDGM
GDSGRDSRSPDSSSPNPLSQGIRPSSPPGPPLTPSAPPPPMPPPPLGSPFPVISSSMGSPGLPPPAPPGF
SGPVSSPQINSTVSLPGGGSGPPEDVKPPVLGVRGLHCPPPPGGPGAGKRLCAICGDRSSGKHYGVYSCE
GCKGFFKRTIRKDLTYSCRDNKDCTVDKRQRNRCQYCRYQKCLATGMKREAVQEERQRGKDKDGDGDGAG
GAPEEMPVDRILEAELAVEQKSDQGVEGPGATGGGGSSPNDPVTNICQAADKQLFTLVEWAKRIPHFSSL
PLDDQVILLRAGWNELLIASFSHRSIDVRDGILLATGLHVHRNSAHSAGVGAIFDRVLTELVSKMRDMRM
DKTELGCLRAIILFNPDAKGLSNPGEVEILREKVYASLETYCKQKYPEQQGRFAKLLLRLPALRSIGLKC
LEHLFFFKLIGDTPIDTFLMEMLEAPHQLA

>Q6LC96_MOUSE
MAVPSLHPSLGPGIGSPLGSPGQLHSPISTLSSPINGMGPPFSVISSPMGPHSMSVPTTPTLGFGTGSPQ
LNSPMNPVSSTEDIKPPLGLNGVLKVPAHPSGNMASFTKHICAICGDRSSGKHYGVYSCEGCKGFFKRTV

RKDLTYTCRDNKDCLIDKRQRNRCQYCRYQKCLAMGMKREAVQEERQRGKDRNENEVESTSSANEDMPVE
KILEAELAVEPKTETYVEANMGLNPSSPNDPVTNICQAADKQLFTLVEWAKRIPHFSELPLDDQVILLRA
GWNELLIASFSHRSIAVKDGILLATGLHVHRNSAHSAGVGAIFDRVLTELVSKMRDMQMDKTELGCLRAI
VLFNPDSKGLSNPAEVEALREKVYASLEAYCKHKYPEQPGRFAKLLLRLPALRSIGLKCLEHLFFFKLIG
DTPIDTFLMEMLEAPHQAT

>Q6MGB3_RAT
MGDSGRDSRSPDSSSPNPLSQGIPPSSPPGPPHTPSAPPPPMPPPPLGSPFPVISSSMGSPGLPPPAPPG
FSGPVSSPQINSTVSLPGGGSGPPEDVKPPVLGVRGLHCPPPPGGPGAGKRLCAICGDRSSGKHYGVYSC
EGCKGFFKRTIRKDLTYSCRDNKDCTVDKRQRNRCQYCRYQKCLATGMKREAVQEERQRGKDKDGDGDGA
GGAPEEMPVDRILEAELAVEQKSDQGVEGPGGTGGSGSSPNDPVTNICQAADKQLFTLVEWAKRIPHFSS
LPLDDQVILLRAGWNELLIASFSHRSIDVRDGILLATGLHVHRNSAHSAGVGAIFDRVLTELVSKMRDMR
MDKTELGCLRAIILFNPDAKGLSNPGEVEILREKVYASLETYCKQKYPEQQGRFAKLLLRLPALRSIGLK
CLEHLFFFKLIGDTPIDTFLMEMLEAPHQLA

>RXRG_MOUSE
MYGNYSHFMKFPTGFGGSPGHTGSTSMSPSVALPTGKPMDSHPSYTDTPVSAPRTLSAVGTPLNALGSPY
RVITSAMGPPSGALAAPPGINLVAPPSSQLNVVNSVSSSEDIKPLPGLPGIGNMNYPSTSPGSLVKHICA
ICGDRSSGKHYGVYSCEGCKGFFKRTIRKDLIYTCRDNKDCLIDKRQRNRCQYCRYQKCLVMGMKREAVQ
EERQRSRERAESEAECASSSHEDMPVERILEAELAVEPKTESYGDMNVENSTNDPVTNICHAADKQLFTL
VEWAKRIPHFSDLTLEDQVILLRAGWNELLIASFSHRSVSVQDGILLATGLHVHRSSAHSAGVGSIFDRV
LTELVSKMKDMQMDKSELGCLRAIVLFNPDAKGLSNPSEVETLREKVYATLEAYTKQKYPEQPGRFAKLL
LRLPALRSIGLKCLEHLFFFKLIGDTPIDSFLMEMLETPLQIT

>RXRG_HUMAN
MYGNYSHFMKFPAGYGGSPGHTGSTSMSPSAALSTGKPMDSHPSYTDTPVSAPRTLSAVGTPLNALGSPY
RVITSAMGPPSGALAAPPGINLVAPPSSQLNVVNSVSSSEDIKPLPGLPGIGNMNYPSTSPGSLVKHICA
ICGDRSSGKHYGVYSCEGCKGFFKRTIRKDLIYTCRDNKDCLIDKRQRNRCQYCRYQKCLVMGMKREAVQ
EERQRSRERAESEAECATSGHEDMPVERILEAELAVEPKTESYGDMNMENSTNDPVTNICHAADKQLFTL
VEWAKRIPHFSDLTLEDQVILLRAGWNELLIASFSHRSVSVQDGILLATGLHVHRSSAHSAGVGSIFDRV
LTELVSKMKDMQMDKSELGCLRAIVLFNPDAKGLSNPSEVETLREKVYATLEAYTKQKYPEQPGRFAKLL
LRLPALRSIGLKCLEHLFFFKLIGDTPIDTFLMEMLETPLQIT

>Q5REL6_PONPY
MYGNYSHFMKFPAGYGGSPGHTGSTSMSPSAALSTGKPMDSHPSYTDTPVSAPRTLSAVGTPLNALGSPY
RVITSAMGPPSGALAAPPGINLVAPPSSQLNVVNSVSSSEDIKPLPGLPGIGNMNYPSTSPGSLVKHICA
ICGDRSSGKHYGVYSCEGCKGFFKRTIRKDLIYTCRDNKDCLIDKRQRNRCQYCRYQKCLVMGMKREAVQ
EERQRSRERAESEAECASSGHEDMPVERILEAELAVEPKTESYGDMNMENSTNDPVTNICHAADKQLFTL
VEWAKRIPHFSDLTLEDQVILLRAGWNELLIASFSHRSVSVQDGILLATGLHVHRSSAHSAGVGSIFDRV
LTELVSKMKDMQMDKSELGCLRAIVLFNPDAKGLSNPSEVETLREKVYATLEAYTKQKYPEQPGRFAKLL
LRLPALRSIGLKCLEHLFFFKLIGDTPIDTFLMEMLETPLQIT

>RXRA_XENLA
MSSAAMDTKHFLPLGGRTCADTLRCTTSWTAGYDFSSQVNSSSLSSSGLRGSMTAPLLHPSLGNSGLNNS

LGSPTQLPSPLSSPINGMGPPFSVISPPLGPSMAIPSTPGLGYGTGSPQIHSPMNSVSSTEDIKPPPGIN
GILKVPMHPSGAMASFTKHICAICGDRSSGKHYGVYSCEGCKGFFKRTVRKDLTYTCRDSKDCMIDKRQR
NRCQYCRYQKCLAMGMKREAVQEERQRGKERNENEVESSNSANEDMPVEKILEAEHAVEPKTETYTEANM
GLAPNSPSDPVTNICQAADKQLFTLVEWAKRIPHFSELPLDDQVILLRAGWNELLIASFSHRSIAVKDGI
LLATGLHVHRNSAHSAGVGAIFDRVLTELVSKMRDMQMDKTELGCLRAIVLFNPDSKGLSNPLEVEALRE
KVYASLEAYCKQKYPEQPGRFAKLLLRLPALRSIGLKCLEHLFFFKLIGDTPIDTFLMEMLEAPHQMT

>7UP1_DROME
MCASPSTAPGFFNPRPQSGAELSAFDIGLSRSMGLGVPPHSAWHEPPASLGGHLHAASAGPGTTTGSVAT
GGGGTTPSSVASQQSAVIKQDLSCPSLNQAGSGHHPGIKEDLSSSLPSANGGSAGGHHSGSGSGSGSGVN
PGHGSDMLPLIKGHGQDMLTSIKGQPTGCGSTTPSSQANSSHSQSSNSGSQIDSKQNIECVVCGDKSSGK
HYGQFTCEGCKSFFKRSVRRNLTYSCRGSRNCPIDQHHRNQCQYCRLKKCLKMGMRREAVQRGRVPPTQP
GLAGMHGQYQIANGDPMGIAGFNGHSYLSSYISLLLRAEPYPTSRYGQCMQPNNIMGIDNICELAARLLF
SAVEWAKNIPFFPELQVTDQVALLRLVWSELFVLNASQCSMPLHVAPLLAAAGLHASPMAADRVVAFMDH
IRIFQEQVEKLKALHVDSAEYSCLKAIVLFTTDACGLSDVTHIESLQEKSQCALEEYCRTQYPNQPTRFG
KLLLRLPSLRTVSSQVIEQLFFVRLVGKTPIETLIRDMLLSGNSFSWPYLPSM

>RXRG_CHICK
MYGNYPHFIKFPAGFGNSPVHASSTSVSPSSSLSVGSTVDGHHNYLEAPTNASRALPSPMNTIGSPVNAL
GSPYRVIASSIGSHPVALSSSAPGMNFVTHSPQPNVLNNVSSSEDIKPLPGLPGIGNMNYPSTSPGSLAK
HICAICGDRSSGKHYGVYSCEGCKGFFKRTIRKDLIYTCRDNKDCLIDKRQRNRCQYCRYQKCLAMGMKR
EAVQEERQGSRERSENEAESTSGGSEDMPVERILEAELAVEPKTEAYSDVNTESSTNDPVTNICHAADKQ
LFTLVEWAKRIPHFSDLTLEDQVILLRAGWNELLIASFSHRSVSVQDGILLATGLHVHRSSAHSAGVGSI
FDRVLTELVSKMKDMQMDKSELGCLRAIVLFNPDAKGLSSPSEVESLREKVYATLEAYTKQKYPEQPGRF
AKLLLRLPALRSIGLKCLEHLFFFKLIGDTPIDTFLMEMLETPLQVT

>RXRB_BRARE
MGDSRDSRSPDSSSVSSPPSGQRSPPLAPSAAAMTSLPPITSAVNSPISSMGSPFSVISSSLGSPCLPGT
PSVGYGPISSPQINSTVSMSGLHAVSSSDDVKPPFGLKPLSSHSPGPMVSQKRLCAICGDRSSGKHYGVY
SCEGCKGFFKRTVRKDLSYTCRDNKDCLVDKRQRNRCQYCRYQKCLAMGMKREVVQDERQRSVQEERQRN
KERDGEVESSSAANEEMPVEKILEAEMAVEQKTELHADGSSGGSSPNDPVTNICQAADKQLFTLVEWAKR
IPHFSELSLDDQVILLRAGWNELLIASFSHRSITVKDGILLATGLHVHRNSAHSAGVGAIFDRESAHNAE
VGAIFDRVLTELVSKMRDMQMDKTELGCLRAIILFNPDAKGLSSPSEVELLREKVYASLEAYCKQRYPDQ
QGRFAKLLLRLPALRSIGLKCLEHLFFFKLIGDTPIDTFLMEMLEAPHQLT

>Q6INZ0_XENLA
MGDSRVCQSPDTSSLSPPLGHSFSDTPPPPSAPLHPSMVGSAMTSSVNSPLGSIGSPFPVINCSVGSPGI
PGTPSIGYGPVSSPQINSTVNLSGLHHVGSSEDVKPPLGMRSMQSHPNGGTVSGKRLCAICGDRSSGKHY
GVHSCEGCKGFFKRTIRKDLTYTCRDSKDCIVDKRQRNRCQYCRYQKCLATGMKREAVQEERQRGRERDG
EAELSGAINEEMPVEKILEAELAVEQKSDQSLEGGGSPSDPVTNICQAADKQLFTLVEWAKRIPHFSELP
LDDQVILLRAGWNELLIASFSHRSISVKDGILLATGLHVHRNSAHSAGVGAIFDRVLTELVSKMRDMRMD
KTELGCLRAIILFNPDAKGLSNPGDVEVLREKVYASLESYCKQKYPDQQGRFAKLLLRLPALRSIGLKCL
EHLFFFKLIGDTPIDTFLMEMLEAPHQLS

>Q91613_XENLA
MGDSRVCQSPDTSSLSPPLGHSFSDTPPPPSAPLHPSMVGSAMTSSVNSPLGSIGSPFPVINCSVGSPGI
PGTPSIGYGPVSSPQINSTVNLSGLHHVGSSEDVKPPLGMRSMQSHPNGGTVSGKRLCAICGDRSSGKHY
GVHSCEGCKGFFKRTIRKDLTYTCRDSKDCIVDKRQRNRCQYCRYQKCLATGMKREAVQEERQRGRERDG
EAELSGAINEEMPVEKILEAELAVEQKSDQSLEGGGSPSDPVTNICQDADKQLFTLVEWAKRIPHFSELP
ELPLDDQVILLRAGWNELLIASFSHRSISEKDGILLATGLHVHRNSAHSAGVGAIFERVLTELVSKMRDM
RMDKTELGCLRAIILFNPDAKGLSNPGDVEVLREKVYACLESYCKQKYPDQQGRFAKLLLRLPALRSIGL
KCLEHLFFFKLIGDTPIDTFLMEMLEAPHQLS

>RXRA_BRARE
MDNNDTYLHLSSSLQVAHGHLSSPPSQPPLSSMVSHHHPSIINGLGSPYSVITSSSLGSPSASMPTTSNM
GYGALNSPQMNSLNSVSSSEDIKPPPGLAGLGSYPCGSPGSLSKHICAICGDRSSGKHYGVYSCEGCKGF
FKRTIRKDLTYTCRDNKDCQIDKRQRNRCQYCRYQKCLAMGMKREAVQEERQRGRERSDNEVDSSSSFNE
EMPVEKILDAELAVEPKTEAYMESSMSNSTNDPVTNICQAADKQLFTLVEWAKRIPHFSDLPLDDQVILL
RAGWNELLIASFSHRSVTVKDGILLATGLHVHRSSAHSAGVGSIFDRVLTELVSKMRDMQMDKTELGCLR
AIVLFNPDAKGLSNPSEVEALREKVYASLEGYTKHNYPDQPGRFAKLLLRLPALRSIGLKCLEHLFFFKL
IGDTPIDTFLMEMLEAPHQIT

>Q86GV5_BRAFL
MAMAVSTWREPGEDLSSPDKQQQQQPVPAPVPGPGPQQPPPSQPPGQTQQSTPGPQQPGPPTPNQQTPNG
GSMPQQTPTAPTQPSSQQGQQQSTPTSGGQSQHIECVVCGDKSSGKHYGQFTCEGCKSFFKRSVRRNLTY
SCRGNRTCPIDQHHRNQCQYCRLKKCLKMGMRREAVQRGRIPPTQHPAGAQYALTNGVDPINGHSYLSGY
ISLLLRAEPYPTSRYGTQCMQPNNIMGIDNICELAARLLFSAVEWARNIPYFPELQVTDQVALLRLVWSE
LFVLNASQCSMPLHVAPLLAAAGLHASPMSADRVVAFMDHIRIFQEQVEKLKALHVDSAEYSCLKAIVLF
TSDACGLSDAAHIDSLQEKSQCALEEYVRSQYPNQPTRFGKLLLRLPSLRTVSSSVIEQLFFVRLVGKTP
IETLIRDMLLSGSSFNWPYMSIQ

>COT1_MOUSE
MAMVVSSWRDPQDDVAGGNPGGPNPAAQAARGGGGGAGEQQQAGSGAPHTPQTPGQPGAPATPGTQGDKG
QGPPGSGQSQQHIECVVCGDKSSGKHYGQFTCEGCKSFFKRSVRRNLTYTCRANRNCPIDQHHRNQCQYC
RLKKCLKVGMRREAVQRGRMPPTQPNPGQYALTNGDPLNGHCYLSGYISLLLRAEPYPTSRYGSQCMQPN
NIMGIENICELAARLLFSAVEWARNIPFFPDLQITDQVSLLRLTWSELFVLNAAQCSMPLHVAPLLAAAG
LHASPMSADRVVAFMDHIRIFQEQVEKLKALHVDSAEYSCLKAIVLFTSDACGLSDAAHIESLQEKSQCA
LEEYVRSQYPNQPSRFGKLLLRLPSLRTVSSSVIEQLFFVRLVGKTPIETLIRDMLLSGSSFNWPYMSIQ
CS

>COT1_HUMAN
MAMVVSSWRDPQDDVAGGNPGGPNPAAQAARGGGGGAGEQQQQAGSGAPHTPQTPGQPGAPATPGTAGDK
GQGPPGSGQSQQHIECVVCGDKSSGKHYGQFTCEGCKSFFKRSVRRNLTYTCRANRNCPIDQHHRNQCQY
CRLKKCLKVGMRREAVQRGRMPPTQPNPGQYALTNGDPLNGHCYLSGYISLLLRAEPYPTSRYGSQCMQP
NNIMGIENICELAARLLFSAVEWARNIPFFPDLQITDQVSLLRLTWSELFVLNAAQCSMPLHVAPLLAAA
GLHASPMSADRVVAFMDHIRIFQEQVEKLKALHVDSAEYSCLKAIVLFTSDACGLSDAAHIESLQEKSQC
ALEEYVRSQYPNQPSRFGKLLLRLPSLRTVSSSVIEQLFFVRLVGKTPIETLIRDMLLSGSSFNWPYMSI
QCS

>COT1_BOVIN
MAMVVSSWRDPQDDVAGGNPGGPNPAAQAARGGGGGAGEQQQQQAGSGAPHTPQTPGQPGAPATPGTAGD
KGQGPPGSGQSQQHIECVVCGDKSSGKHYGQFTCEGCKSFFKRSVRRNLTYTCRANRNCPIDQHHRNQCQ
YCRLKKCLKVGMRREAVQRGRMPPTQPNPGQYALTNGDPLNGHCYLSGYISLLLRAEPYPTSRYGSQCMQ
PNNIMGIENICELAARLLFSAVEWARNIPFFPDLQITDQVSLLRLTWSELFVLNAAQCSMPLHVAPLLAA
AGLHASPMSADRVVAFMDHIRIFQEQVEKLKALHVDSAEYSCLKAIVLFTSDACGLSDAAHIESLQEKSQ
CALEEYVRSQYPNQPSRFGKLLLRLPSLRTVSSSVIEQLFFVRLVGKTPIETLIRDMLLSGSSFNWPYMS
IQCS

>Q62681_RAT
MAMVVSSWRDPQDDVAGGNPGGPNPAAQAARGGGGGEQQQAGSGAPHTPQTPGQPGAPATPGTAGDKGQG
PPGSGQSQQHIECVVCGDKSSGKHYGQFTCEGCKSFFKRSVRRNLTYTCRANRNCPIDQHHRNQCQYCRL
KKCLKVGMRREAVQRGRMPPTQPNPGQYALTNGDPLNGHCYLSGYISLLLRAEPYPTSRYGSQCMQPNNI
MGIENICELAARLLFSAVEWARNIPFFPDLQITDQVSLLRLTWSELFVLNAAQCSMPLHVAVLAAAGLHA
SPMSADRVVAFMDHIRIFQEQVEKLKALHVDSAEYSCLKAIVLFTSDACGLSDAAHIESLQEKSQCALEE
YVRSQYPNQPSRFGKLLLRLPSLRTVSSSVIEQLFFVRLVGKTPIETLIRDMLLSGSSFNWPYMSIQCS

>Q6DHP9_BRARE
MDTHDTYLHLHSSPLNSSPSQPPVMSSMVGHPSVISSSRPLPSPMSTLGSSMNGLPSPYSVITPSLSSPS
ISLPSTPSMGFNTLNSPQMNSLSMNGNEDIKPPPGLAPLGNMSSYQCTSPGSLSKHICAICGDRSSGKHY
GVYSCEGCKGFFKRTIRKDLTYTCRDIKECLIDKRQRNRCQYCRYQKCLAMGMKREAVQEERQRGKEKSD
TEVETTSRFNEDMPVDKILDAELSVEPKTETYTESSPSNSTNDPVTNICHAADKQLFTLVEWAKRIPHFS
DLPLDDQVILLRAGWNELLIASFSHRSITVKDGILLGTGLHVHRSSAHSAGVGSIFNRVLTELVSKMKDM
QMDKTELGCLRAIVLFNPDAKGLSNSLEVEALREKVYASLETYTKQKYPDQPGRFAKLLLRLPALRSIGL
KCLEHLFFFKLIGDTPIDTFLMEMLEAPHQIT

>COT2_BOVIN
MAMVVSTWRDPQDEVPGSQGSQASQAPPVPGPPPGAPHTPQTPGQGGPASTPAQTAAGGQGGPGGPGSDK
QQQQQHIECVVCGDKSSGKHYGQFTCEGCKSFFKRSVRRNLSYTCRANRNCPIDQHHRNQCQYCRLKKCL
KVGMRREAVQRGRMPPTQPSHGQFALTNGDPLNCHSYLSGYISLLLRAEPYPTSRFGSQCMQPNNIMGIE
NICELAARMLFSAVEWARNIPFFPDLQITDQVALLRLTWSELFVLNAAQCSMPLHVAPLLAAAGLHASPM
SADRVVAFMDHIRIFQEQVEKLKALHVDSAEYSCLKAIVLFTSDACGLSDVAHVESLQEKSQCALEEYVR
SQYPNQPTRFGKLLLRLPSLRTVSSSVIEQLFFVRLVGKTPIETLIRDMLLSGSSFNWPYMAIQ

>COT2_HUMAN
MAMVVSTWRDPQDEVPGSQGSQASQAPPVPGPPPGAPHTPQTPGQGGPASTPAQTAAGGQGGPGGPGSDK
QQQQQHIECVVCGDKSSGKHYGQFTCEGCKSFFKRSVRRNLSYTCRANRNCPIDQHHRNQCQYCRLKKCL
KVGMRREAVQRGRMPPTQPTHGQFALTNGDPLNCHSYLSGYISLLLRAEPYPTSRFGSQCMQPNNIMGIE
NICELAARMLFSAVEWARNIPFFPDLQITDQVALLRLTWSELFVLNAAQCSMPLHVAPLLAAAGLHASPM
SADRVVAFMDHIRIFQEQVEKLKALHVDSAEYSCLKAIVLFTSDACGLSDVAHVESLQEKSQCALEEYVR
SQYPNQPTRFGKLLLRLPSLRTVSSSVIEQLFFVRLVGKTPIETLIRDMLLSGSSFNWPYMAIQ

>COT2_MOUSE

MAMVVSTWRDPQDEVPGSQGSQASQAPPVPGPPPGAPHTPQTPGQGGPASTPAQTAAGGQGGPGGPGSDK
QQQQQHIECVVCGDKSSGKHYGQFTCEGCKSFFKRSVRRNLSYTCRANRNCPIDQHHRNQCQYCRLKKCL
KVGMRREAVQRGRMPPTQPTHGQFALTNGDPLNCHSYLSGYISLLLRAEPYPTSRFGSQCMQPNNIMGIE
NICELAARMLFSAVEWARNIPFFPDLQITDQVALLRLTWSELFVLNAAQCSMPLHVAPLLAAAGLHASPM
SADRVVAFMDHIRIFQEQVEKLKALHVDSAEYSCLKAIVLFTSDACGLSDVAHVESLQEKSQCALEEYVR
SQYPNQPTRFGKLLLRLPSLRTVSSSVIEQLFFVRLVGKTPIETLIRDMLLSGSSFNWPYMAIQ

>COT2_RAT
MAMVVSTWRDPQDEVPGSQGSQASQAPPVPGPPPGAPHTPQTPGQGGPASTPAQTAAGSQGGPGGPGSDK
QQQQQHIECVVCGDKSSGKHYGQFTCEGCKSFFKRSVRRNLSYTCRANRNCPIDQHHRNQCQYCRLKKCL
KVGMRREAVQRGRMPPTQPTHGQFALTNGDPLNCHSYLSGYISLLLRAEPYPTSRFGSQCMQPNNIMGIE
NICELAARMLFSAVEWARNIPFFPDLQITDQVALLRLTWSELFVLNAAQCSMPLHVAPLLAAAGLHASPM
SADRVVAFMDHIRIFQEQVEKLKALHVDSAEYSCLKAIVLFTSDACGLSDVAHVESLQEKSQCALEEYVR
SQYPNQPTRFGKLLLRLPSLRTVSSSVIEQLFFVRLVGKTPIETLIRDMLLSGSSFNWPYMAIQ

>NR2F1_BRARE
MAMVVSVWRDPQEDVAGGPPSGPNPAAQPAREQQQAASAAPHTPQTPSQPGPPSTPGTAGDKGSQNSGQS
QQHIECVVCGDKSSGKHYGQFTCEGCKSFFKRSVRRNLTYTCRANRNCPIDQHHRNQCQYCRLKKCLKVG
MRREAVQRGRMPPTQPNPGQYALTNGDPLNGHCYLSGYISLLLRAEPYPTSRYGSQCMQPNNIMGIENIC
ELAARLLFSAVEWARNIPFFPDLQITDQVSLLRLTWSELFVLNAAQCSMPLHVAPLLAAAGLHASPMSAD
RVVAFMDHIRIFQEQVEKLKALHVDSAEYSCIKAIVLFTSDACGLSDAAHIESLQEKSQCALEEYVRSQY
PNQPSRFGKLLLRLPSLRTVSSSVIEQLFFVRLVGKTPIETLIRDMLLSGSSFNWPYMSIQ

>Q91430_BRARE
MAMVVWRGSQDDVAETHGTLSSQTQGGLSLPTPQPGQLGLTASQVAPPTPQTPVQGPPNNNNNTQSTPTN
QTTQSQSEKQQPQHIECVVCGDKSSGKHYGQFTCEGCKSFFKRSVRRNLTYTCRANRNCPIDQHHRNQCQ
YCRLKKCLKVGMRREAVQRGRMPPTQPHHGQFALTNGDPLHCHSYLSGYISLLLRAEPYPTSRYGSQCMQ
PNNIMGIENICELAARMLFSAVEWARNIPFFPDLQITDQVALLRLTWSELFVLNAAQCSMPLHVAPLLAA
AGLHASPMSADRVVAFMDHIRIFQEQVEKLKALHVDSAEYSCLKAIVLFTSDACGLSDVAHVESLQEKSQ
CALEEYVRSQYPNQPTRFGKLLLRLPSLRTVSSSVIEQLFFVRLVGKTPIETLIRDMLLSGSSFNWPYMS
IQ

>Q6PHF2_BRARE
MAMVVSVWRDPQEDVAGGPPSGPNPAAQPAREQQQAASAAPHTPQTPSQPGPPSTPGTAGDKGSQNSGQS
QQHIECVVCGDKSSGKHYGQFTCEGCKSFFKRSVRRNLTYTCRANRNCPIDQHHRNQCQYCRLKKCLKVG
MRREVQRGRMPPTQPNPGQYALTNGDPLNGHCYLSGYISLLLRAEPYPTSRYGSQCMQPNNIMGIENICE
LAARLLFSAVEWARNIPFFPDLQITDQVSLLRLTWSELFVLNAAQCSMPLHVAPLLAAAGLHASPMSADR
VVAFMDHIRIFQEQVEKLKALHVDSAEYSCIKAIVLFTSDACGLSDAAHIESLQEKSQCALEEYVRSQYP
NQPSRFGKLLLRLPSLRTVSSSVIEQLFFVRLVGKTPIETLIRDMLLSGSSFNWPYMSIQ

>Q9W745_XENLA
MAMVVSSWRDPQEDVAGGNPGGPNPGVREQQQAPSAAPHTPQTPSQPGPPSTPGAAGDKGQQGSGQSQQQ
HIECVVCGDKSSGKHYGQFTCEGCKSFFKRSVRRNLTYTCRANRNCPIDQHHRNQCQYCRLKKCLKVGMR
REAVQRGRMPPTQPNPGQYALTNGDPLNGHCYLSGYISLLLRAEPYPTSRYGSQCMQPNNIMGIENICEL

AARLLFSAVEWARNIPFFPDLQITDQVALLRLTWSELFVLNAAQCSMPLHVAPLLAAAGLHASPMSADRV
VAFMDHIRIFQEQVEKLKALHVDSAEYSCIKAIVLFTSDACGLSDAAHIESLQEKSQCALEEYVRSQYPN
QPSRFGKLLLRLPSLRTVSSSVIEQLFFVRLVGKTPIETLIRDMLLSGSSFNWPYMPIQCS

>Q91840_XENLA
MVGSAMTSSVNSPLGSIGSPFPVINCSVGSPGIPGTPSIGYGPVSSPQINSTVNLSGLHSVSSSEDVKPP
LGMRSMPSHPNGGAVSGKRLCAICGDRSSGKHYGVYSCEGCKGFFKRTIRKDLTYTCRDSKDCIVDKRQR
NRCQYCRYQKCLATGMKREAVQEERQRGKERDGEAELSGAINEEMPVEKILEAELAVEQKSDQSLEGGGS
PSDPVTNICQAADKQLFTLVEWAKRIPHFSELALDDQVILLRAGWNELLIASFSHRSISVKDGILLATGL
HVHRNSAHSAGVGAIFDRVLTELVSKMRDMRMDKTELGCLRAIILFNPDAKGLSNPGDVEVLREKVYASL
ESYCKQKYPDQQGRFAKLLLRLPALRSIGLKCLEHLFFFKLIGDTPIDTFLMEMLEAPHQLS

>Q8T5C6_BIOGL
MDRSEGMDTLENSMPSGMSMGMTMGGHQGHPPPDIKPDISSLTSPTSTHGYYGFGPGGMPSMASSTQPSP
GPQQMHSPGMHSPTSSMGSPPMLCLSPSGPSPSPGLPHSSLHTKHICAICGDRASGKHYGVYSCEGCKGF
FKRTVRKDLTYACRDDKNCMIDKRQRNRCQYCRYMKCLSMGMKREAVQEERQRVKEKGDGEVESTSGANN
DMPVEQILEAELAVDPKIDTYIDAQKDPVTNICQAADKQLFTLVEWAKRIPHFTELPLEDQVILLRAGWN
ELLIAGFSHRSIMAKDGILLATGLHVHRSSAHQAGVGTIFDRVLTELVAKMRDMKMDKTELGCLRAVVLF
NPDAKGLTAVQEVEQLREKVYASLEEYTKSRYPEEPGRFAKLLLRLPALRSIGLKCLEHLFFFKLIGDQP
IDTFLMEMLENPSPAT

>COT2_CHICK
MAMVVGAWRDPQDDVPGAQGTQPAPAPPVQGPPAGTPHTPQTPGPGGPPSTPAQSNAASQQSQGDKQQQQ
QHIECVVCGDKSSGKHYGQFTCEGCKSFFKRSVRRNLSYTCRANRNCPIDQHHRNQCQYCRLKKCLKVGM
RREAVQRGRMPPTQPTHGQFALTNGDPLNCHSYLSGYISLLLRAEPYPTSRFGSQCMQPNNIMGIENICE
LAARMLFSAVEWARNIPFFPDLQITDQVALLRLTWSELFVLNAAQCSMPLHVAPLLAAAGLHASPMSADR
VVAFMDHIRIFQEQVEKLKALHVDSAEYSCLKAIVLFTSDACGLSDVAHVESLQEKSQCALEEYVRSQYP
NQPTRFGKLLLRLPSLRTVSSSVIEQLFFVRLVGKTPIETLIRDMLLSGSSFNWPYMSIQ

>Q6DCH6_XENLA
MAMVVSGWRDPQEDVAGGNPGGPNPGVREQQQAPSAAPHTPQTPSQPGPPSTPGAAGDKTGQSQHIECVV
CGDKSSGKHYGQFTCEGCKSFFKRSVRRNLTYTCRANRNCPIDQHHRNQCQYCRFKKCLKVGMRREVQRG
RMPPTQPNPGQYALTNGDPLNGHCYLSGYISLLLRAEPYPTSRYGSQCMQPNNIMGIENICELAARLLFS
AVEWARNIPFFPDLQITDQVALLRLTWSELFVLNAAQCSMPLHVAPLLAAAGLHASPMSADRVVAFMDHI
RIFQEQVEKLKALHVDSAEYSCLKAIVLFTPDACGLSDAAHIESLQEKSQCALEEYVRSQYPNQPSRFGK
LLLRLPSLRTVSSSVIEQLFFVRLVGKTPIETLIRDMLLSGSSFNWPYMPIQCS

>Q8VCR0_MOUSE
MPPPPLGSPFPVISSSMGSPGLPPPAPPGFSGPVSSPQINSTVSLPGGGSGPPEDVKPPVLGVRGLHCPP
PPGGPGAGKRLCAICGDRSSGKHYGVYSCEGCKGFFKRTIRKDLTYSCRDNKDCTVDKRQRNRCQYCRYQ
KCLATGMKREAVQEERQRGKDKDGDGDGAGGAPEEMPVDRILEAELAVEQKSDQGVEGPGATGGGGSSPN
DPVTNICQAADKQLFTLVEWAKRIPHFSSLPLDDQVILLRAGWNELLIASFSHRSIDVRDGILLATGLHV
HRNSAHSAGVGAIFDRSLSRVLTELVSKMRDMRMDKTELGCLRAIILFNPDAKGLSNPGEVEILREKVYA
SLETYCKQKYPEQQGRFAKLLLRLPALRSIGLKCLEHLFFFKLIGDTPIDTFLMEMLEAPHQLA

>Q7ZXI7_XENLA
MAMVVGAWRDPQDDMPGSQTTQAPPGPTGAPHTPQTPGQGVPPTTPAQSNPSSQSSQNQGEKQQQQHIEC
VVCGDKSSGKHYGQFTCEGCKSFFKRSVRRNLTYTCRANRNCPIDQHHRNQCQYCRLKKCLKVGMRREAV
QRGRMPPTQPTHGQFALTNGDPLNCHSYLSGYISLLLRAEPYPTSRFGSQCMQPNNIMGIENICELAARM
LFSAVEWARNIPFFPDLQITDQVALLRLTWSELFVLNAAQCSMPLHVAPLLAAAGLHASPMSADRVVAFM
DHIRIFQEQVEKLKALHVDSAEYSCLKAIVLFTSDACGLSDVAHVESLQEKSQCALEEYVRSQYPNQPTR
FGKLLLRLPSLRTVSSSVIEQLFFVRLVGKTPIETLIRDMLLSGSSFNWPYMSIQ

>Q9PS79_9PIPI
MAMVVGAWRDPQDDMPGSQTTQAPPGPTRAPHTPQTPGQGVPPTTPAQSNPSSQSSQNQGEKQQQQHIEC
VVCGDKSSGKHYGQFTCEGCKSFFKRSVRRNLTYTCRANRNCPIDQHHRNQCQYCRLKKCLKVGMRREAV
QRGRMPPTQPTHGQFALTNGDPLNCHSYLSGYISLVLRAEPYPTSRFGSQCMQPNNIMGIENICELAARM
LFSAVEWARNIPFFPDLQITDQVALLRLTWSELFVLNAAQCSMPLHVAPLSAAAGLHASPMSADRVVAFM
DHIRIFQEQVEKLKALHVDSAEYSCLKAIVLFTSDACGLSDVAHVESLQEKSQCALEEYVRSQYPNQPTR
FGKLLLRLPSLRTVSSSVIEQLFFVRLVGKTPIETLIRDMLLSGSSFNWPYMSIQ

>Q8UUM6_ORYLA
MTSLPPISTSGVNSPVSSIGSPFSVISSSLGSPCLPGTPSVGYGPISSPQINSTVSMSGLHTVSSSDDVK
PPLGLKQLSSHSPGPMLSQKRLCSICGDRSSGKHYGVYSCEGCKGFFKRTVRKDLTYTCRDNKDCTVDKR
QRNRCQYCRYQKCLAMGMKREAVQEERQRNKEREGEVESTSAVNEEMPVEKILEAEMAVEQKTELHSDGG
SGGSSPNDPVTNISQAADKQLFTLVEWAKRIPHFSELPLDDQVILLRWNELLIASFSHRSISVKDGILLA
TGLHVHRNSAHSAGVGAIFDRAHNAEVGAIFERVLTELVSKMRDMQMDKTELGCLRAIILFNPDAKGLSN
SSEVELLRERVYASLETYCKHKYPDQQGRFAKLLLRLPALRSIGLKCLEHLFFFKLIGDTPIDTFLMEML
EAPHQLT

>RXRD_BRARE
MNSLPPSTSAVSSPVSSVDSPLSAVSSSIGSPGVPGTPSIGYGPISNSQINSSMSVSRLHAVSSSDDVKP
PFGLKSVSGSGPMLSQKRMCAICGDRSSGKHYGVYSCEGCKGFFKRTVRKDLSYTCRDNKECLVDKRQRN
RCQYCRYQKCLAMGMKREAVQEERQKNKERDGDYECSSSANEEMPVEKILEAETAVEHRTDLHSDATGSP
NDPVTNICQAADKQLFTLVEWAKRVPHFSDVPLDDQVILLRAGWNELLIAAFSHRSISVKDEILLATGLH
VPKESTHNLGVEAFFDRESSHSAEVGALFDRVLTELVCKMRDMQMDKTELGCLRAIVLFNPDAKGLTSSS
EVELLREKVYASLESYCKQKYPDQQGRFAKLLLRLPALRSIGLKCLEHLFFFKLIGNTPIDTFLMEMLES
PH

>Q66TQ0_9CAEN
MGHQVEACQVAMHMGVPGMGGMGGPHQPDIKPDISTLNPPSSTHPGFSYPGYGGMPGMPSSTQASPGGPN
MTSPQMHSPTSSLGSPTMMCLSPTGTSSPGMPHSGLSKHICAICGDRASGKHYGVYSCEGCKGFFKRTVR
KDLTYACRDDKNCMIDKRQRNRCQYCRYMKCLAQGMKREAVQEERQRVKEKGDGEVESTSGANSDMPVEQ
ILEAEIAVEPKIDTYIDAQKEPVTNICQAADKQLFTLVDWAKRIPHFVELPLEDQVILLRAGWNELLIGG
FSHRSTQVTDGILLATGLHVHRSSAHQAGVGTIFDRVLTELVAKMREMKMDKTELGCLRAIVLFNPDAKG
LQSVQEVEQLREKVYASLEEYCKQRYPDEPGRFAKLLLRLPALRSIGLKCLEHLFFFKLIGQTPIDTFLM
EMLESPSHPAT

>Q6V7U7_LOCMI
MEGSERGISLENNLSISSMGPQSPLDMKPDTASLISSGSFSPTGGPNSPGSFTIGHSSLLNNSSSNQAKG
SSSQYPPNHPLSGSKHLCSICGDRASGKHYGVYSCEGCKGFFKRTVRKDLSYACREDKNCIIDKRQRNRC
QYCRYQKCLAMGMKREAVQEERQRTKERDQNEVESTSSLHTDMPVERILEAEKRVECKAENQVEYESTMN
NICQAANICQATNKQLFQLVEWAKHIPHFTSLPLEDQVLLLRAGWNELLIAAFSHRSVDVKDGIVLATGL
TVHRNSAHQAGVGTIFDRVLTELVAKMREMKMDKTELGCLRSVILFNPEVRGLKSAQEVELLREKVYAAL
EEYTRTTHPDEPGRFAKLLLRLPSLRSIGLKCLEHLFFFRLIGDVPIDTFLMEMLESPSDS

>Q66J63_XENLA
MASIEEIAHQIIEQQMGEISRSHTEVSQTALMDGTTQRIQLVPSESSVSVPQRIQIVTDPQTGQKIQIVT
ALDQSGTNKQFIFTNNDGSLPSKVILARQDSSQGKVFLTTPDAAGVNQLFFSTPDVPAQHIQILSDTQSL
DQNLNKQFVELCVVCGDKASGRHYGAVTCEGCKGFFKRSIRKNLIYTCRGSKDCVINKHYRNRCQYCRLQ
RCISLGMKQDSVQCERKPIEVSREKSSNCAASTEKIYIRKDLRSPLAATTTFVTESKTSRTTSLLDSSML
VNIQQSGVKNESILLTPNKVEACQGDLSTLANVVTSLANLNKSKDLPQTNTEFSIIESLSNGDSSLSDLA
QDDQSNSEVTRAFDTLAKALNQSENSTQGSSECVGSGSNLLPDVNVEIEGPLLNDVHIAFRLTMPSPMPE
YLNVHYICESASRLLFLSMHWARSIPSFLSLGQENSISLVKACWNELFSLGLAQCCQVMNVETILAAFVN
HLHNSMQHDKLSSDKVKLVMDHIFKLQEFCNSMVKRCVDGYEYAYLKAIALFSPDHPGLENVSHIEKLQE
KAYMEFQDYVTKTYPEDTYRLSRLLLRLPALRLLNAAITEELFFAGLIGNVQIDSIIPYILRMETSDYNS
QIIGLTA

>Q91720_XENLA
MAMVVNPWQEDIPGVPGSQMNNPPGLCNQDPGGTPQTPTTPKGGIPGQDPVHSGDKGVPNVDCLVCGDKS
SGKHYGQFTCEGCKSFFKRSVRRNLTYTCRSNRDCPIDQHHRNQCQYCRLKKCLKVGMRREVQRGRMSHP
QTSPGQYTLNNVDPYNGHSYLTGFISLLLRAEPYPTSRYGAQCLQPNNIMGIENICELAARLLFSAIEWA
KNIPFFPDFQLSDQVSLLRMTWSELFVLNAAQCSMPLHVAPLLARAGLHASPMSADRVVAFMDHIRIFQE
QVEKLKALHVDSAEYSCLKAIALFTPDAVGLSDIGHVESIQEKSQCALEEYVRNQYPNQPTRFGRLLLRL
PSLRIVSAPVIEQLFFVRLVGKTPIETLIRDMLLSGSSFNWPYMPMQ

>Q5RCZ5_PONPY
MATIEEIAHQIIEQQMGEIVTEQQTGQKIQIVTALDHNTQGKQFILTNHDGSTPSKVILARQDSTPGKVF
LTTPDAAGVNQLFFTTPDLSAQHLQLLTDNSSPDQGPNKVFDLCVVCGDKASGRHYGAVTCEGCKGFFKR
SIRKNLVYSCRGSKDCIINKHHRNRCQYCRLQRCIAFGMKQDSVQCERKPIEVSREKSSNCAASTEKIYI
RKDLRSPLTATPTFVTDSESTRSTGLLDSGMFVNIHPSGVKTESTVLMTSDKAESCQGDLSTLASVVTSL
ANLGKTKDLSQNSNEMSMIESLSNDDTSLCEFQEMHTNGDVSRAFDTLAKALNPGESTACQSSVAGMEGS
VHLITGDSSINYTEKEGPLLSDSHVAFRLTMPSPMPEYLNVHYIGESASRLLFLSMHWALSIPSFQALGQ
ENSISLVKAYWNELFTLGLAQCWQVMNVATILATFVNCLHNSLQQDKMSTERRKLLMEHIFKLQEFCNSM
VKLCIDGYEYAYLKAIVLFSPDHPGLENMEQIEKFQEKAYVEFQDYITKTYPDDTYRLSRLLLRLPALRL
MNATITEELFFKGLIGNIRIDSVIPHILKMEPGQYSKTSSL

>Q95K90_MACFA
MATIEEIAHQIIEQQMGEIVTEQQTGQKIQIVTALDHNTQGKQFILTNHDGSTPSKVILARQDSTPGKVF
LTPDAAGVNQLFFTTPDLSAQHLQLLTDNSSPDQGPNKVFDLCVVCGDKASGRHYGAVTCEGCKGFFKRS
IRKNLVYSCRGSKDCIINKHHRNRCQYCRLQRCIAFGMKQDSVQCERKPIEVSREKSSNCAASTEKIYIR
KDLRSPLTATPTFVTDSETTRSTGLLDSGMFVNIHPSGVKTESTVLMTSDKAESCQGDLSTLASVVTSLA

NLGKTKDLSQNSNEMSMIESLSNDDTSLCEFQEMQTNGDVSRAFDTLAKALNPGESTACQSSVAGMEGSV
HLITGDSSINYTEKEGPLLSDSHVAFRLTMPSPMPEYLNVHYIGESASRLLFLSMHWALSIPSFQALGQE
NSISLVKAYWNELFTLGLAQCWQVMNVATILATFVNCLHNSLQQDKMSTERRKLLMEHIFKLQEFCNSMV
KLCIDGYEYAYLKAIVLFSPDHPGLENMEQIEKFQEKAYVEFQDYITKTYPDDTYRLSRLLLRLPALRLM
NATITEELFFKGLIGNIRIDSVIPHILKMEPADYNSQIIGHSI

>RXRG_BRARE
MPVPEQKQTVQLSSPMNAVSSSEDIKPPLGLNGVMKVPAHRIGTLSLSLTKHICAICGDRSSGKHYGVYS
CEGCKGFFKRTVRKDLTYTCRDNKDCMIDKRQRNRCQYCRYQKCLAMGMKREAVQEERQRAKERSEAEFG
GCANEDMPVEKILEAELAVEPKTETYVEANLSPSANSPNDPVTNICQAADKQLFTLVEWAKRIPHFSDLP
LDDQVILLRAGWNELLIASFSHRSIAVKDGILLATGLHVHRNSAHTAGVGAIFDRVLTELVSKMRDMQMD
KTELGCLRAIVLFNPDSKGLSNPSEVEALRERVYASLEAYCKHKYPDQPGRFAKLLLRLPALRSIGLKCL
EHLFFFKLIGDTPIDTFLMEMLEAPHQIT

>Q15625_HUMAN
MATIEEIAHQIIEQQMGEIVTEQQTGQKIQIVTALDHNTQGKQFILTNHDGSTPSKVILARQDSTPGKVF
LTTPDAAGVNQLFFTTPDLSAQHLQLLTDNSPDQGPNKVFDLCVVCGDKASGRHYGAVTCEGCKGFFKRS
IRKNLVYSCRGSKDCIINKHHRNRCQYCRLQRCIAFGMKQDSVQCERKPIEVSREKSSNCAASTEKIYIR
KDLRSPLTATPTFVTDSESTRSTGLLDSGMFMNIHPSGVKTESAVLMTSDKAESCQGDLSTLANVVTSLA
NLGKTKDLSQNSNEMSMIESLSNDDTSLCEFQEMQTNGDVSRAFDTLAKALNPGESTACQSSVAGMEGSV
HLITGDSSINYTEKEGPLLSDSHVAFRLTMPSPMPEYLNVHYIGESASRLLFLSMHWALSIPSFQALGQE
NSISLVKAYWNELFTLGLAQCWQVMNVATILATFVNCLHNSLQQDKMSTERRKLLMEHIFKLQEFCNSMV
KLCIDGYEYAYLKAIVLFSPDHPSLENMELIEKFQEKAYVEFQDYITKTYPDDTYRLSRLLLRLPALRLM
NATITEELFFKGLIGNIRIDSVIPHILKMEPADYNSQIIGHSI

>Q9VLI7_DROME
MMKHPQDLSVTDDQQLMKVNKVEKMEQELHDPESESHIMHADALASAYPAASQPHSPIGLALSPNGGGLG
LSNSSNQSSENFALCNGNGNAGSAGGGSASSGSNNNNSMFSPNNNLSGSGSGTNSSQQQLQQQQQQSPT
VCAICGDRATGKHYGASSCDGCKGFFRRSVRKNHQYTCRFARNCVVDKDKRNQCRYCRLRKCFKAGMKKE
AVQNERDRISCRRTSNDDPDPGNGLSVISLVKAENESRQSKAGAAMEPNINEDLSNKQFASINDVCESMK
QQLLTLVEWAKQIPAFNELQLDDQVALLRAHAGEHLLLGLSRRSMHLKDVLLLSNNCVITRHCPDPLVSP
NLDISRIGARIIDELVTVMKDVGIDDTEFACIKALVFFDPNAKGLNEPHRIKSLRHQILNNLEDYISDRQ
YESRGRFGEILLILPVLQSITWQMIEQIQFAKIFGVAHIDSLLQEMLLGGELADNPLPLSPPNQSNDYQS
PTHTGNMEGGNQVNSSLDSLATSGGPGSHSLDLEVQHIQALIEANSADDSFRAYAASTAAAAAAAVSSSS
SAPASVAPASISPPLNSPKSQHQHQQHATHQQQQESSYLDMPVKHYNGSRSGPLPTQHSPQRMHPYQRAV
ASPVEVSSGGGGLGLRNPADITLNEYNRSEGSSAEELLRRTPLKIRAPEMLTAPAGYGTEPCRMTLKQEP
ETGY

>NR2F5_BRARE
MAMVVNQWQENISADPGSQLQMCSQEPGGTPGTPSGSTPGNDALSGDKIPNVDCMVCGDKSSGKHYGQFT
CEGCKSFFKRSVRRNLSYTCRGNRDCPIDQHHRNQCQYCRLKKCLKVGMRREAVQRGRMSNSQSSPGQYL
SNGSDPYNGQPYLSGFISLLLRAEPYPTSRYGAQCMQSNNLMGIENICELAARLLFSAVEWAKNIPFFPD
LQLMDQVALLRMSWSELFVLNAAQCSMPLHVAPLLAAAGLHASPMSAERVVAFMDHIRVFQEQVEKLKAL
QVDTAEYSCLKSIVLFTSDAMGLSDVAHVESIQEKSQCALEEYVRNQYPNQPNRFGRLLLRLPSLRIVSS

PVIEQLFFVRLVGKTPIETLLRDMLLSGSSYNWPYMPVQRDRPISIHYNENGP

>Q9GSG8_AEDAE
MLKKEKPMLSVAAIIQAQGRWDRTLPLAGLAGFDAALVGHMGPVSPQDMKPDLKPDISLLNGSVGPFSPG
NNCGPASPGAFNQQVAAALQQQQQNVNSLNSQQSGGGGGAGGGTPTTPTNMSQQYPPNHPLSGSKHLCSI
CGDRASGKHYGVYSCEGCKGFFKRTVRKDLSYACREDKNCTIDKRQRNRCQYCRYQKCLACGMKREAVQE
ERQRSSKFSIKSEEINSTSSVRDVTIERIHEAEQLSEQKSGDNAIPYLRVGSNSMIPPEYKGAVSHLCQM
VNKQIYQLIDFARRVPHFINLPRDDQVMLLRCGWNEMLIAAVAWRSMEYIETERSSDGSRITVRQPQLMC
LGPNFTLHRNSAQQAGVDTLFDRILCELGIKMKRLDVTRAELGVLKAIILFNPDIRGLKCQKEIDGMREK
IYACLDEHCKQQHPSEDGRFAQLLLRLPALRSISLKCLDHLNFIRLLSDKHLDSFIVEMLDMPI

>USP_MANSE
MSSVAKKDKRTMSVTALINRAWPLTPAPHQQQSMPSSQPSNFLQPLATPSTTPSVELDIQWLNIEPGFMS
PMSPPEMKPDTAMLDGLRDDSTPPPAFKNYPPNHPLSGSKHLCSICGDRASGKHYGVYSCEGCKGFFKRT
VRKDLTYACREDRNCIIDKRQRNRCQYCRYQKCLACGMKREAVQEERQRAARGTEDAHPSSSVQELSIER
LLEIESLVADPPEEFQFLRVGPESGVPAKYRAPVSSLCQIGNKQIAALVVWARDIPHFGQLELEDQILLI
KNSWNELLLFAIAWRSMEYLTDERENVDSRSTAPPQLMCLMPGMTLHRNSALQAGVGQIFDRVLSELSLK
MRTLRMDQAEYVALKAIILLNPDVKGLKNKPEVVVLREKMFSCLDEYVRRSRCAEEGRFAALLLRLPALR
SISLKCFEHLYFFHLVADTSIASYIHDALRNHAPSIDTSIL

>Q6PH18_BRARE
MAMVVSAWRDPQEELAAVDDQSAAGREHLQHRHSPKSAEEKAQIAAQNQQHVECVVCGDKSSGKHYGQFT
CEGCKSFFKRSVRRNLSYTCRANRNCPVDQHHRNQCQYCRLKKCLKVGMRREAVQRGRMPPNQPNPSHYA
LTNGDHLNGQCYLSGYISLLLRAEPYPASRYGNQCMQSGNIMGIENICELAARLLFSAVEWARNIPFFPD
LQITDQVSLLRLTWSELFVLNAAQSSMPLHVAPLLAAAGLHASPMSADRVVAFMDHIRFFQEQVEKLKAL
QVDSAEYSCAKAIVLFTSDACGLSDIPHIEGLQEKSQCALEEYVRSQYPNQPTRFGKLLLRLPALRMVSS
SVIEQLFFVRLVGKTPIETLIRDMLLSGSSFNWPYMPIQ

>TR4_HUMAN
MTSPSPRIQIISTDSAVASPQRIQIVTDQQTGQKIQIVTAVDASGSPKQQFILTSPDGAGTGKVILASPE
TSSAKQLIFTTSDNLVPGRIQIVTDSASVERLLGKTDVQRPQVVEYCVVCGDKASGRHYGAVSCEGCKGF
FKRSVRKNLTYSCRSNQDCIINKHHRNRCQFCRLKKCLEMGMKMESVQSERKPFDVQREKPSNCAASTEK
IYIRKDLRSPLIATPTFVADKDGARQTGLLDPGMLVNIQQPLIREDGTVLLATDSKAETSQGALGTLANV
VTSLANLSESLNNGDTSEIQPEDQSASEITRAFDTLAKALNTTDSSSSPSLADGIDTSGGGSIHVISRDQ
STPIIEVEGPLLSDTHVTFKLTMPSPMPEYLNVHYICESASRLLFLSMHWARSIPAFQALGQDCNTSLVR
ACWNELFTLGLAQCAQVMSLSTILAAIVNHLQNSIQEDKLSGDRIKQVMEHIWKLQEFCNSMAKLDIDGY
EYAYLKAIVLFSPDHPGLTSTSQIEKFQEKAQMELQDYVQKTYSEDTYRLARILVRLPALRLMSSNITEE
LFFTGLIGNVSIDSIIPYILKMETAEYNGQITGASL

>TR4_MOUSE
MTSPSPRIQIISTDSAVASPQRIQIVTDQQTGQKIQIVTAVDASGSSKQQFILTSPDGAGTGKVILASPE
TSSAKQLIFTTSDNLVPGRIQIVTDSASVERLLGKADVQRPQVVEYCVVCGDKASGRHYGAVSCEGCKGF
FKRSVRKNLTYSCRSSQDCIINKHHRNRCQFCRLKKCLEMGMKMESVQSERKPFDVQREKPSNCAASTEK
IYIRKDLRSPLIATPTFVADKDGARQTGLLDPGMLVNIQQPLIREDGTVLLAADSKAETSQGALGTLANV

VTSLANLSESLNNGDASEMQPEDQSASEITRAFDTLAKALNTTDSASPPSLADGIDASGGGSIHVISRDQ
STPIIEVEGPLLSDTHVTFKLTMPSPMPEYLNVHYICESASRLLFLSMHWARSIPAFQALGQDCNTSLVR
ACWNELFTLGLAQCAQVMSLSTILAAIVNHLQNSIQEDKLSGDRIKQVMEHIWKLQEFCNSMAKLDIDGY
EYAYLKAIVLFSPDHPGLTGTSQIEKFQEKAQMELQDYVQKTYSEDTYRLARILVRLPALRLMSSNITEE
LFFTGLIGNVSIDSIIPYILKMETAEYNGQITGASL

>Q6P0E6_BRARE
MAMVVSAWRDPQEELAAVDDQSAAGREHLQHRHSPKSAEEKAQIAAQNQQHVECVVCGDKSSGKHYGQFT
CEGCKSFFKRSVRRNLSYTCRANRNCPVDQHHRNQCQYCRLKKCLKVGMRREVQRGRMPPNQPNPSHYAL
TNGDHLNGQCYLSGYISLLLRAEPYPASRYGNQCMQSGNIMGIENICELAARLLFSAVEWARNIPFFPDL
QITDQVSLLRLTWSELFVLNAAQSSMPLHVAPLLAAAGLHASPMSADRVVAFMDHIRFFQEQVEKLKALQ
VDSAEYSCAKAIVLFTSDACGLSDIPHIEGLQEKSQCALEEYVRSQYPNQPTRFGKLLLRLPALRMVSSS
VIEQLFFVRLVGKTPIETLIRDMLLSGSSFNWPYMPIQ

>TR4_RAT
MTSPSPRIQIISTDSAVRSPQRIQIVTDQQTGQKLQIVTAVDASGSSKQQFILTSPDGAGTGKVILASPE
TSSAKQLIFTTSDNLVPGRIQIVTDSASVERLLGKADVQRPQVVEYCVVCGDKASGRHYGAVSCEGCKGF
FKRSVRKNLTYSCRSSQDCIINKHHRNRCQFCRLKKCLEMGMKMESVQSERKPFDVQREKPSNCAASTEK
IYIRKDLRSPLIATPTFVADKDGSRQTGLLDPGMLVNIQQPLIREDGTVLLATDSKAETSQGALGTLANV
VTSLANLSESLNNGDASEMQPEDQSASEITRAFDTLAKALNTTDSASPPSLADGIDASGGGSIHVISRDQ
STPIIEVEGPLLSDTHVTFKLTMPSPMPEYLNVHYICESASRLLFLSMHWARSIPAFQALGQDCNTSLVR
ACWNELFTLGLAQCAQVMSLSTILAAIVNHLQNSIQEDKLSGDRIKQVMEHIWKLQEFCNSMAKLDIDGH
EYAYLKAIVLFSPDHPGLTGTSQIEKFQEKAQMELQDYVQKTYSEDTYRLARILVRLPALRLMSSNITEE
LFFTGLIGNVSIDSIIPYILKMETAEYNGQITGASL

>Q9GSG7_AEDAE
MDPSDRGFDAALVGHMGPLSPQDMKPDLKPDISLLNGSVGPFSPGNNCGPASPGAFNQQVAAALQQQQQN
VNSLNSQQSGGGGGGGGGTPTTPTNMSQQYPPNHPLSGSKHLCSICGDRASGKHYGVYSCEGCKGFFKRT
VRKDLSYACREDKNCTIDKRQRNRCQYCRYQKCLACGMKREAVQEERQRSSKFSIKSEEINSTSSVRDVT
IERIHEAEQLSEQKSGDNAIPYLRVGSNSMIPPEYKGAVSHLCQMVNKQIYQLIDFARRVPHFINLPRDD
QVMLLRCGWNEMLIAAVAWRSMEYIETERSSDGSRITVRQPQLMCLGPNFTLHRNSAQQAGVDTLFDRIL
CELGIKMKRLDVTRAELGVLKAIILFNPDIRGLKCQKEIDGMREKIYACLDEHCKQQHPSEDGRFAQLLL
RLPALRSISLKCLDHLNFIRLLSDKHLDSFIVEMLDMPI

>Q9U7D9_LOCMI
MEGSERGISLENNLSISSMGPQSPLDMKPDTASLISSGSFSPTGGPNSPGSFTIGHSSLLNNSSSNQAKG
SSSQYPPNHPLSGSKHLCSICGDRASGKHYGVYSCEGCKGFFKRTVRKDLSYACREDKNCIIDKRQRNRC
QYCRYQKCLAMGMKREAVQEERQRTKERDQNEVESTSSLHTDMPVERILEAEKRVECKAENQVEYELVEW
AKHIPHFTSLPLEDQVLLLRAGWNELLIAAFSHRSVDVKDGIVLATGLTVHRNSAHQAGVGTIFDRVLTE
LVAKMREMKMDKTELGCLRSVILFNPEVRGLKSAQEVELLREKVYAALEEYTRTTHPDEPGRFAKLLLRL
PSLRSIGLKCLEHLFFFRLIGDVPIDTFLMEMLESPSDS

>USP_BOMMO
MSSVAKKDKRTMSVTALINRAWPMTPSPQQQQQMVPSTQHSNFLHAMATPSTTPNVELDIQWLNIESGFM

SPMSPPEMKPDTAMLDGFRDDSTPPPPFKNYPPNHPLSGSKHLCSICGDRASGKHYGVYSCEGCKGFFKR
TVRKDLTYACREDKNCIIDKRQRNRCQYCRYQKCLACGMKREAVQEERQRAARRTEDAHPSSSVQELSIE
RLLELEALVADSAEELQILRVGPESGVPAKYRAPVSSLCQIGNKQIAALIVWARDIPHFGQLEIDDQILL
IKGSWNELLLFAIAWRSMEFLNDERENVDSRNTAPPQLICLMPGMTLHRNSALQAGVGQIFDRVLSELSL
KMRSLRMDQAECVALKAIILLNPDVKGLKNKQEVDVLREKMFLCLDEYCRRSRGGEEGRFAALLLRLPAL
RSISLKSFEHLYLFHLVAEGSVSSYIRDALCNHAPPIDTNIM

>Q8VIJ3_MOUSE
MATIEEIAHQIIDQQMGEIVTEQQTGQKIQIVTALDHSTQGKQFILANHEGSTPGKVFLTTPDAAGVNQL
FFTSPDLSAPHLQLLTENSPDQGPNKVFDLCVVCGDKASGRHYGAITCEGCKGFFKRSIRKNLVYSCRGS
KDCVINKHHRNRCQYCRLQRCIAFGMKQDSVQCERKPIEVSREKSSNCAASTEKIYIRKNLRSPLAATPT
FVTDSETARSAGLLDSGMFVNIHPSGIKTEPAMLMAPDKAESCQGDLSTLASVVTSLANLGKTKDLSHCG
GDMPVVQSLRNGDTSFGAFHHDIQTNGDVSRAFDTLAKALTPGESTSCQSSEEGMEGSPHLIAGEPSFVE
KEGPLLSESHVAFRLTMPSPMPEYLNVHYIGESASRLLFLSMHWALSIPSFQALGQENSISLVKAYWNEL
FTLGLAQCWQVMNVATILATFVNCLHSSLQQDKMSPERRKSLMEHIFKLQEFCNSMVKLCIDGHEYAYLK
AIVLFSPDHPGLENMELIERFQEKAYVEFQDYITRTYPDDTYRLSRLLLRLPALRLMNATITEELFFKGL
IGNVRIDSVIPHILKMEPADYNSQIIGHSL

>Q8VIJ4_RAT
MATIEEIAHQIIDQQMGEIVTEQQTGQKIQIVTALDHSTQGKQFILANHEGSTPGKVFLTTPDAAGVNQL
FFASPDLSTPHLQLLTENSPDQGPNKVFDLCVVCGDKASGRHYGAITCEGCKGFFKRSIRKNLVYSCRGS
KDCIINKHHRNRCQYCRLQRCIAFGMKQDSVQCERKPIEVSREKSSNCAASTEKIYIRKDLRSPLAATPT
FVTDSETARSTGLLDSGMFVNIHPSGIKTEPALLMTPDKAESCQGDLGTLASVVTSLANLGKAKDLSHCG
GDLPVVQSLRNGDTSFGAFHQDIQTNGDVSRAFDNLAKALTPGENPACQSPGESMEGSTHLIAGEPSCME
REGPLLSDSHVVFRLTMPSPMPEYLNVHYIGESASRLLFLSMHWALSIPSFQALGQENSISLVKAYWNEL
FTLGLAQCWQVMNVATILATFVNCLHNSLQQDKMSPERRKLLMEHIFKLQEFCNSMVKLCIDGHEYAYLK
AIVLFSPDHPGLENMELIEKFQEKAYVEFQDYITRTYPDDTYRLSRLLLRLPALRLMNATITEELFFKGL
IGNVRIDSVIPHILKMEPADYNSQIIGHSL

>HNF4_DROME
MHADALASAYPAASQPHSPIGLALSPNGGGLGLSNSSNQSSENFALCNGNGNAGSAGGGSASSGSNNNNS
MFSPNNNLSGSGSGTNSSQQQLQQQQQQQSPTVCAICGDRATGKHYGASSCDGCKGFFRRSVRKNHQYTC
RFARNCVVDKDKRNQCRYCRLRKCFKAGMKKEAVQNERDRISCRRTSNDDPDPGNGLSVISLVKAENESR
QSKAGAAMEPNINEDLSNKQFASINDVCESMKQQLLTLVEWAKQIPAFNELQLDDQVALLRAHAGEHLLL
GLSRRSMHLKDVLLLSNNCVITRHCPDPLVSPNLDISRIGARIIDELVTVMKDVGIDDTEFACIKALVFF
DPNAKGLNEPHRIKSLRHQILNNLEDYISDRQYESRGRFGEILLILPVLQSITWQMIEQIQFAKIFGVAH
IDSLLQEMLLGGELADNPLPLSPPNQSNDYQSPTHTGNMEGGNQVNSSLDSLATSGGPGSHSLDLEVQHI
QALIEANSADDSFRAYAASTAAAAAAAVSSSSSAPASVAPASISPPLNSPKSQHQHQQHATHQQQQESSY
LDMPVKHYNGSRSGPLPTQHSPQRMHPYQRAVASPVEVSSGGGGLGLRNPADITLNEYNRSEGSSAEELL
RRTPLKIRAPEMLTAPAGYGTEPCRMTLKQEPETGY

>Q62152_MOUSE
MATIEEIAHQIIDQQMGEIVTEQQTGQKMQIVTALDHSTQGKQFILANHEGSTPGKVFLTTPDAAGVNQL
FFTSPDLSAPHLQLLTEKSPDQGPNKVFDLCVVCGDKASGRHYGAITCEGCKGFFKRSIRKNLVYSCRGS

KDCVMNKHHRNRCQYCRLQRCIAFGMKQDSVQCERKPIEVSREKSSNCAASTEKIYIRKDLRSPLAATPT
FVTDSETARSAGLLDSGMFVNIHPSGIKTEPAMLMAPDKAESCQGDLSTLASVVTSLANLGKAKDLSHCG
GDMPVVQSLRNGDTSSVLFIMIFKTNGDVSRAFDTLAKALTPGESTSCQSPEEGMEGSPHLIAGEPSFVE
KEGPLLSESHIAFRLTMPSPMPEYLNAHYIGESASRLLFLSMHWALSIPSFQALGQENSISLVKAYWNEL
FTLGLAQCWQVMNVATILATFVNCLHSSLQQDKMSAERRKSLMEHIFKLQEFCNSMVKLCIDGHEYAYLK
AIVLFSPDHPGLENMELIERFQEKAYVEFQDYITRTYPDDTYRLSRLLLRLPALRLMNATITEELFFKGL
IGNVRIDSVIPHILKMEPADYNSQIIGHSL

>Q6GL59_XENTR
MAMVSGGWGDPNGDTNGVGKGYPRNSEEEEASPQGGMSDPEQGDEERPGIQVDCVVCGDKSSGKHYGVFT
CEGCKSFFKRSVRRNLSYTCRSNRDCQIDQHHRNQCQYCRLKKCFRVGMRKEAVQRGRIPPAHSSASPTS
APGAGEYFNGQPVSELISQLLRAEPYPASRYGSQYAQQGSVMGIDNICELAARLLFSTVEWSRNIPYFPE
LAMADQVSLLRLSWSELFVLSAAQSALPLHMAPLLAAAGFHASPMSADRVVSFMDQIRLFQDQVEKLNRL
QVDSAEYACLKAIALFTSDACGLTDPAHVESLQEKAQVALTEYVRAQYPSQPQRFGRLLLRLPALRAVPA
SLISQLFFMRLVGKTPIETLIRDMLLSGSSFNWPYSSGQ

>Q7T0T7_XENLA
MAMVSGGWGDPNGDTNGVGKGYPRNSEEEEASPQGGMSDPEQGDEERPGIQVDCVVCGDKSSGKHYGVFT
CEGCKSFFKRSVRRNLSYTCRSNRDCQIDQHHRNQCQYCRLKKCFRVGMRKEAVQRGRIPPAHSSASPTS
APGAGEYFNGQPVSELISQLLRAEPYPASRYGSQYTQQGSVMGIDNICELAARLLFSTVEWSRSIPYFPE
LAVADQVSLLRLSWSELFVLSAAQSALPLHMAPLLAAAGFHSSPMSADRVVSFMDQIRLFQDQVEKLNRL
QVDSAEYACLKAIALFTSDACGLTDPAHVESLQEKAQVALTEYVRAQYPSQPQRFGRLLLRLPALRAVPA
SLISQLFFMRLVGKTPIETLIRDMLLSGSSFNWPYSSGQ

>Q922G8_MOUSE
MAMVTGGWGDPGGDTNGVDKAGGSYPRATEDDSASPPGATSDAEPGDEERPGLQVDCVVCGDKSSGKHYG
VFTCEGCKSFFKRSIRRNLSYTCRSNRDCQIDQHHRNQCQYCRLKKCFRVGMRKEAVQRGRIPHALPGPA
ACSPPGATGVEPFTGPPVSELIAQLLRAEPYPAAGRFGGGGAVLGIDNVCELAARLLFSTVEWARHAPFF
PELPAADQVALLRLSWSELFVLNAAQAALPLHTAPLLAAAGLHAAPMAAERAVAFMDQVRAFQEQVDKLG
RLQVDAAEYGCLKAIALFTPDACGLSDPAHVESLQEKAQVALTEYVRAQYPSQPQRFGRLLLRLPALRAV
PASLISQLFFMRLVGKTPIETLIRDMLLSGSTFNWPYGSG

>Q9NFY1_TENMO
MTMESTDRALSLDQNLSMGSLGAPHSPLDMKPDASTLGQNSPVSFASGHGSLLSFSPQGPPSGGTPNKSC
GSLYPPNHPLSGSKHLCSICGDRASGKHYGVYSCEGCKGFFKRTVRKDLSYACREEKNCIIDKRQRNRCQ
YCRYQKCLNMGMKREAVQEERQRTKDRDTSEVESTSNMQAEMPLDRIIEAEKRIECTPAGGSGGVGEQHD
GVNNICQATNKQLFQLVQWAKLIPHFTSLPMSDQVLLLRAGWNELLIAAFSHRSIQAQDAIVLATGLTVN
KTSAHAVGVGNIYDRVLSELVNKMKEMKMDKTELGCLRAIILYNPTCRGIKSVQEVEMLREKIYGVLEEY
TRTTHPNEPGRFAKLLLRLPALRSIGLKCSEHLFFFKLIGDVPIDTFLMEMLESPADA

>EAR2_MOUSE
MAMVTGGWGDPGGDTNGVDKAGGSYPRATEDDSASPPGATSDAEPGDEERPGLQVDCVVCGDKSSGKHYG
VFTCEGCKSFFKRTIRRNLSYTCRSNRDCQIDQHHRNQCQYCRLKKCFRVGMRKEAVQRGRIPHALPGPA
ACSPPGATGVEPFTGPPVSELIAQLLRAEPYPAAGRFGGGGAVLGIDNVCELAARLLFSTVEWARHAPFF

PELPAADQVALLRLSWSELFVLNAAQAALPLHTAPLLAAAGLHAAPMAAERAVAFMDQVRAFQEQVDKLG
RLQVDAAEYGCLKAIALFTPDACGLSDPAHVESLQEKAQVALTEYVRAQYPSQPQRFGRLLLRLPALRAV
PASLISKLFFMRLVGKTPIETLIRDMLLSGSTFNWPYGSG

>Q6P117_BRARE
MAMVRGGWGDPNGETNGLGDKGYLRGDEDDGSPQGGGSDMEAGEDDKGCVVDCVVCGDKSSGKHYGVFTC
EGCKSFFKRSVRRNLNYTCRSNRDCQIDQHHRNQCQYCRLKKCFRVGMRKEAVQRGRIPPSHSSLSPSTT
PVGGNAGGGVSEFYNGQPVSELISQLLRAEPYPNSRYSHQYNQQMQGGGGGGSGMGIDSICELAARLLFS
IIEWARNIPYFPELPVSEQVALLRLSWSELFILNAAQSALPLHMAPLLAAAGFHSSPMSAERVVSFMDQV
RVFQDQVEKLTRLQVDSAEYSCLKAIALFSPDACGLTDPAHVESLQEKAQVALTEYERMQYPGQPQRFGR
LLLRLPALRAVPASLISQLFFMRLVGKTPIETLIRDMQLSGSSISWPYAPGQ

>EAR2_RAT
MAMVTGGWGGPGGDTNGVDKAGGSYPRATEDDSASPPGATSDAEPGDEERPGLQVDCVVCGDKSSGKHYG
VFTCEGCKSFFKRTIRRNLSYTCRSNRDCQIDQHHRNQCQYCRLKKCFRVGMRKEAVQPGPIPHALPGPA
ACSPPGAAGVEPFAGPPVSELIAQLLRAEPYPAAGRFGGGGAVLGIDNVCELAARLLFSTVEWARHAPFF
PELPAADQVGLLRLSWSELFVLNAAQAPVPLHTAPLLAAAGLHAGPMAAERAVAFMDQVRAFQEQVDKLG
RLQVDAAEYGCLKAIALFTPDACGLSDPAHVESLQEKAQVALTEYVRAQYPSQPQRFGRLLLRLPALRAV
PASLISQLFFMRLVGKTPIETLIRDMLLSGSTFNWPYGSG

>Q5QPB8_HUMAN
MRLSKTLVDMDMADYSAALDPAYTTLEFENVQVLTMGNDTSPSEGTNLNAPNSLGVSALCAICGDRATGK
HYGASSCDGCKGFFRRSVRKNHMYSCRFSRQCVVDKDKRNQCRYCRLKKCFRAGMKKEAVQNERDRISTR
RSSYEDSSLPSINALLQAEVLSRQITSPVSGINGDIRAKKIASIADVCESMKEQLLVLVEWAKYIPAFCE
LPLDDQVALLRAHAGEHLLLGATKRSMVFKDVLLLGNDYIVPRHCPELAEMSRVSIRILDELVLPFQELQ
IDDNEYAYLKAIIFFDPDAKGLSDPGKIKRLRSQVQVSLEDYINDRQYDSRGRFGELLLLLPTLQSITWQ
MIEQIQFIKLFGMAKIDNLLQEMLLGGPCQAQEGRGWSGDSPGDRPHTVSSPLSSLASPLCRFGQVA

>Q962I5_LUCCU
MDNGEQDAGFRLAPMSPQEIKPDISLLNENNTSSYSPKPGSPNPFAIGLQAINAVAAANANNQNQMLQTT
PPQQQQYPPNHPLSGSKHLCSICGDRASGKHYGVYSCEGCKGFFKRTVRKDLTYACREDRNCIIDKRQRN
RCQYCRYQKCLACGMKREAVQEERQRGTRAANARAAGAGGGGGGGGGVSNVVGAGGEDFKPSSSLRDLTI
ERIIEAEQKAESLSGDNVLPFLRVGNNSMVQHDYKGAVSHLCQMVNKQLYQMVEYARRTPHFTHLQREDQ
ILLLKAGWNELLIANVAWCSIESLDAEYASPGTVHDGSFGRRSPVRQPQQLFLNQNFSYHRNSAIKANVV
SIFDRILSELSIKMKRLNIDRSELSCLKAIILFNPDIRGLKCRADVEVCREKIYACLDEHCRTEHPGDDG
RFAQLLLRLPALRSISLKCLDHLFFFRLIGERALEELIAEQLEAPIC

>Q6P115_BRARE
MAMVSGGWANPNGSANGLGEKGYLRGEEEGSSPQAGNSDVEGGEEDKACVVDCVVCGDKSSGKHYGVFTC
EGCKSFFKRSIRRNLNYTCRSNRECQIDQHHRNQCQYCRLKKCFRVGMRKEAVQRGRIPPSHAGISPASM
VGAGGDVGGGPGMGADFFNGQPVSELISQLLRAEPYPNSRYGAQCGQQLQGANSSMMGIDNICELAARLL
FSTIEWARNIPYFPDLPVSEQVALLRLSWSELFILNAAQSALPLHTAPLLAAAGFHSSPMPADRVVSFMD
QVRVFQDQVDKLTRLQVDSVEYSCLKAIALFSPDACGLSDPAHVESLQEKAQVALTEYERMQYPGQPQRF
GRLLLRLPALRAVPANLISQLFFMRLVGKTPIETLIRDMQLSGSSISWPYVPGQ

>O61449_9ACAR
MAYQEPTRNLNGGGASNGVSSSLLPQPSTYLSGGGYGGTLSVNRAPADGQPTLSNGPSSATAPGGDSRFP
ATHPLSGSKHLCSICGDRASGKHYGVYSCEGCKGFFKRTVRKDLTYACREERRCVVDKRQRNRCQYCRYQ
KCLMCGMKREAVQEERQRAKDRNDNEVESTSGGVGVSGGVGGPGSPDMPLERILEAEMRVEQPAPSVLAQ
TAASGRDPVNSMCQAAPPLHELVQWARRIPHFEELPIEDRTALLKAGWNELLIAAFSHRSVAVRDGIVLA
TGLVVQRHSAHGAGVGDIFDRVLAELVAKMRDMKMDKTELGCLRAVVLFNPDAKGLRNATRVEALREKVY
AALEEHCRRHHPDQPGRFGKLLLRLPALRSIGLKCLEHLFFFKLIGDTPIDSFLLNMLEAPADP

>O61448_9ACAR
MATQERAPGLNGGTSNGVSSSLPPQPSLPAERLRRALGSITVGRPAAQQRSSGDTATAAAATSHHALSTK
PPLSGSKHLCSICGDRASGKHYGVYSCEGCKGFFKRTVRKDLSYACREERTCIIDKRQRNRCQYCRYQKC
LACGMKREAVQEERQRTKDRADSEVESTSGGAPPEMPLERILEAELRVESQTGTLSESAQQQDPVSSICQ
AADRQLHQLVQWAKHIPHFEELPLEDRMVLLKAGWNELLIAAFSHRSVDVRDGIVLATGLVVQRHSAHGA
GVGAIFDRVLTELVAKMREMKMDRTELGCLLAVVLFNPEAKGLRTCPSGGPEGESVSALEEHCRQQYPDQ
PGRFAKLLLRLPALRSIGLKCLEHLFFFKLIGDTPIDNFLLSMLEAPSDP

>HNF4A_MOUSE
MDMADYSAALDPAYTTLEFENVQVLTMGNDTSPSEGANLNSSNSLSVSALCAICGDRATGKHYGASSCDG
CKGFFRRSVRKNHMYSCRFSRQCVVDKDKRNQCRYCRLKKCFRAGMKKEAVQNERDRISTRRSSYEDSSL
PSINALLQAEVLSQQITSPISGINGDIRAKKIANITDVCESMKEQLLVLVEWAKYIPAFCELLLDDQVAL
LRAHAGEHLLLGATKRSMVFKDVLLLGNDYIVPRHCPELAEMSRVSIRILDELVLPFQELQIDDNEYACL
KAIIFFDPDAKGLSDPGKIKRLRSQVQVSLEDYINDRQYDSRGRFGELLLLLPTLQSITWQMIEQIQFIK
LFGMAKIDNLLQEMLLGGSASDAPHTHHPLHPHLMQEHMGTNVIVANTMPSHLSNGQMCEWPRPRGQAAT
PETPQPSPPSGSGSESYKLLPGAITTIVKPPSAIPQPTITKQEAI

>HNF4A_RAT
MDMADYSAALDPAYTTLEFENVQVLTMGNDTSPSEGANLNSSNSLGVSALCAICGDRATGKHYGASSCDG
CKGFFRRSVRKNHMYSCRFSRQCVVDKDKRNQCRYCRLKKCFRAGMKKEAVQNERDRISTRRSSYEDSSL
PSINALLQAEVLSQQITSPISGINGDIRAKKIANITDVCESMKEQLLVLVEWAKYIPAFCELLLDDQVAL
LRAHAGEHLLLGATKRSMVFKDVLLLGNDYIVPRHCPELAEMSRVSIRILDELVLPFQELQIDDNEYACL
KAIIFFDPDAKGLSDPGKIKRLRSQVQVSLEDYINDRQYDSRGRFGELLLLLPTLQSITWQMIEQIQFIK
LFGMAKIDNLLQEMLLGGSASDAPHAHHPLHPHLMQEHMGTNVIVANTMPSHLSNGQMCEWPRPRGQAAT
PETPQPSPPSGSGSESYKLLPGAITTIVKPPSAIPQPTITKQEAI

>Q6PHH5_BRARE
MRLSKPLVDMEMADYSEALDPAYTTLEFENMQVLAMSTDSSPQESANMNAANHLGAGTLCAICGDRATGK
HYGASSCDGCKGFFRRSVRKNHMYSCRFNRQCIVDKDKRNQCRYCRLKKCFRAGMKKEAVQNERDRISTR
RSSYEDSSLPSINALIQADVLSRQISSPGPILNGDIRTKKVAAIMDVCESMKQQLLVLVEWAKYIPAFCD
LPLDDQVALLRAHAGEHLLLGAAKRSMMYKDILLLGNDHIIPRNCPELEVSRVAVRILDELVLPFQDLQI
DDNEYACLKAIVFFDPDAKGLSDPSKIKRMRYQVQVSLEDYINDRQYDSRGRFGELLLLLPTLQSITWQM
IEQIQFVKLFGMAKIDNLLQEMLLGGSANEAPHAHHSLHPHLVQEHLSNNVIVTANMATPLHNGQMSTPE
TPIPSPPTASGSDHYKMASGVIATVPKQPSSIPQPTITKQEAI

>Q9JJI9_TAMSI
MDMADYSAALDPAYTTLEFENVQVLAMGNDTSPSEGTNLAAPNSLGVSALCAICGDRATGKHYGASSCDG
CKGFFRRSVRKNHMYSCRFSRQCVVDKDKRNQCRYCRLKKCFRAGMKKEAVQNERDRISTRRSSYEDSSL
PSINALLQAEVLSQQITSPVSGINGDIRAKKIANIADVCESMKEQLLVLVEWAKYIPAFCELLLDDQVAL
LRAHAGEHLLLGATKRSMVFKDVLLLGNDYIVPRHCPELAEMSRVSIRILDELVLPFQELQIDDNEYACL
KAIIFFDPDAKGLSDPGKIKRLRSQVQVSLEDYINDRQYDSRGRFGELLLLLPTLQSITWQMIEQIQFIK
LFGMAKIDNLLQEMLLGGSSSDAPHAHHPLHPHLMQEHMGTNVIVANTMPTHLSNGQMSTPETPQPSPPG
GSGSEPYKLLPGAITTIVKPPSAIPQPTITKQEVI

>Q7YRQ5_BOVIN
MDMADYSAALDPAYTTLEFENVQVLTMGNDTSPSEGANLNAPNSLGVSALCAICGDRATGKHYGASSCDG
CKGFFRRSVRKNHMYSCRFSRQCVVDKDKRNQCRYCRLKKCFRAGMKKEAVQNERDRISTRRSSYEDSSL
PSINALLQAEVLSQQITSPVSGINGDIRAKKIASIADVCESMKEQLLVLVEWAKYIPAFCELPLDDQVAL
LRAHAGEHLLLGATKRSMVFKDVLLLGNDYIVPRHCPELAEMSRVSVRILDELVLPFQELQIDDNEYACL
KAIIFFDPDAKGLSDPGKIKRLRSQVQVSLEDYINDRQYDSRGRFGELLLLLPTLQSITWQMIEQIQFIK
LFGMAKIDNLLQEMLLGGSSSEAPHAHHPLHPHLMPEHMGTNVIVANTMPAHLSNGQMSTPETPQPSPPG
GSGSEPYKLLPGAIATIVKPPSAIPQPTITKQEVI

>HNF4A_HUMAN
MDMADYSAALDPAYTTLEFENVQVLTMGNDTSPSEGTNLNAPNSLGVSALCAICGDRATGKHYGASSCDG
CKGFFRRSVRKNHMYSCRFSRQCVVDKDKRNQCRYCRLKKCFRAGMKKEAVQNERDRISTRRSSYEDSSL
PSINALLQAEVLSRQITSPVSGINGDIRAKKIASIADVCESMKEQLLVLVEWAKYIPAFCELPLDDQVAL
LRAHAGEHLLLGATKRSMVFKDVLLLGNDYIVPRHCPELAEMSRVSIRILDELVLPFQELQIDDNEYAYL
KAIIFFDPDAKGLSDPGKIKRLRSQVQVSLEDYINDRQYDSRGRFGELLLLLPTLQSITWQMIEQIQFIK
LFGMAKIDNLLQEMLLGGSPSDAPHAHHPLHPHLMQEHMGTNVIVANTMPTHLSNGQMCEWPRPRGQAAT
PETPQPSPPGGSGSEPYKLLPGAVATIVKPLSAIPQPTITKQEVI

>Q5RH32_BRARE
MEGESPRIQLVSADGGLQIVTEQQLAQKVQIVTAIDQAGAGKQQFILANLDYPNQEKLFIKQENSPAKVI
LTSSDGSAVNQLLFASPELSGQQIQFVTDGSDQGSMKPPVEYCVVCGDKASGRHYGAVSCEGCKGFFKRS
IRKNLVYTCRGSGECVINKHHRNRCQYCRLQRCMALGMKQDSVQCERKPVEVSREKPANCAPSIEKIYIR
KNLCSPLAAMPTFVSQKESTRPTSLLDSNMLLNIQQSISKLDNTILIPSSPDQNDSSQGDLGTLANVVTS
LGHLNKSREMMDSSTDLSGIDTMSNDDSVMTDIQREESNDVTRAFDTLTKVLKAEESCGEEVADGAVAVR
DDEQTSALLELEGPLLSDMHVPFKLMMPLPMPDFLNLNYICESASRLLFLSMHWARSIPAFQALGSENGI
TLMKACWNELFALGLAQCSHIMNVETILTAIINHLQTSLDEEKLSPERVKQVMEHIWRMQEFCNSMSRMS
PDAYEYAYLKAVVLFSPDHSAVDGTLQIERFQEKAYMELQDYVSKVYPEDTYRLSKLLVRLPALRLMSAA
VTEELFFAGLIGNVQIDSIIPYILKMESTDYNSQPISGVE

>Q7SZG3_FUGRU
MHSISSSEDIKPPFGLRPVPAHSPGLMMSQKRMCVICGDRSSGKHYGVYSCEGCKGFFKRTVRKDLSYTC
RDNKECLVDKRQRNRCQYCRYQKCLAMGMKREAVQEERQRNREREGELEFSVSVNEEMPVEKILAAETAV
EQKTELHSDGVSAGNSPHDAVSNICQTADKQLFALVEWAKRIPHFSELPLEDQVILLRAGWNELLIASFS
HRSINSKDGVLLASELQRDSANSAGVGAIFDRENVQSAEVGAIFDRVLTELVNKMRDMQMDKTELGCLRA
IVLFNPDAKGLSKSSEVELLREKVYASLEAYCKQRYPEQQGRFAKLLLRLPALRSIGLKCLEHLFFFKLI

GDTPIDTFLMEMLEAPHHLS

>Q8AVK3_XENLA
MRLSKALIDMDMADYTEALDPAYTTLEFENMQVLSIGTDTSTSDVTSLSASNSIGINSLCAICGDRATGK
HYGASSCDGCKGFFRRSVRKNHMYSCRFSRQCVVDKDKRNQCRYCRLKKCFRAGMKKEAVQNERDRISTR
RSSYEDSSLPSINVLIQAEVLSQQITSSVGVLNTDIRGKKIACIIDVCDSMKQQLLVLVEWAKYIPAFCE
LPLDDQVALLRAHAGEHLLLGATKRSMMFKDILLLGNDRLIPRNCPELEVGRVAVRILDELVLPFQELQI
DDNEYACLKAIIFFDPDAKGLSDPTKIKRMRYQVQVSLEDYINDRQYDSRGRFGDLLLLLPTLQSITWQM
IEQIQFVKLFGMAKIDNLLQEMLLGGSANEASHTHHHLHPHLVQDHLATNVIVANNTLPSQLHNGQMSTP
ETPQPSPPAGSGAEQYKIVHGTIASINKQPTSIPQSTITKQEAM

>Q5QPB7_HUMAN
MILLPLRLARLRHPLRHHWSISGGVDSSPQGDTSPSEGTNLNAPNSLGVSALCAICGDRATGKHYGASSC
DGCKGFFRRSVRKNHMYSCRFSRQCVVDKDKRNQCRYCRLKKCFRAGMKKEAVQNERDRISTRRSSYEDS
SLPSINALLQAEVLSRQITSPVSGINGDIRAKKIASIADVCESMKEQLLVLVEWAKYIPAFCELPLDDQV
ALLRAHAGEHLLLGATKRSMVFKDVLLLGNDYIVPRHCPELAEMSRVSIRILDELVLPFQELQIDDNEYA
YLKAIIFFDPDAKGLSDPGKIKRLRSQVQVSLEDYINDRQYDSRGRFGELLLLLPTLQSITWQMIEQIQF
IKLFGMAKIDNLLQEMLLGGSPSDAPHAHHPLHPHLMQEHMGTNVIVANTMPTHLSNGQMCEWPRPRGQA
ATPETPQPSPPGGSGSEPYKLLPGAVATIVKPLSAIPQPTITKQEVI

>Q6IVK1_PLOIN
MEPSRESGLNLENSTFMSPMSPPEMKPDTAMLDGLRDDATSPPSFKNYPPNHPLSGSKHLCSICGDRASG
KHYGVYSCEGCKGFFKRTVRKDLSYACREERNCIIDKRQRNRCQYCRYQKCLACGMKREAVQEERQRAAR
GTEDAHPSSSVQVSAEELSIERLLEMEALVADTSEEFQFLRVGPDSNVPPKFRAPVSSLCQIGNKQIAAL
VVWARDIPHFSQLELEDQVTLIKASWNELLLFAIAWRSMEYLTDERDNVDGSRTTSPPQLMCLMPGMTLH
RNSALQAGVGQIFDRVLSELALKMRSLPVDQAEYVALKAVILLNPDVKGLNSRQEVEVLREKMYSCLDEY
CRRSRGSEEGRFASLLLRLPALRSISLKSFEHLFFFHLVADGSIPGYIRDALRSHKPPLDASSIM

>Q95WF7_ACRMI
MAGVPAQSWCTEKTEPLEESAEKNVQQVECAVCGDKSSGKHYGVFTCEGCKSFFKRSVRRNLSYTCRASR
NCPIDQHHRNQCQYCRLRKCMKVGMRREAVQRGRIPPTQVPQPSPQHSALNGNDVSNGHSFLSGFISLLL
RAEPYPTTRFQQGMNMPCGIMGIENICELAARLLFSAVEWARNIPFFPDLAVTDQVALLRLVWSELFVLN
AAQCPMPLQVAPLLASAGIHSNHMSPDRMVTFMDNVRIFQEQIEKYRNLHVDAAEFACLKAIVLFTSDAS
GLTDPQYIESLQEKTQCALEEYTRNQYPNQPTRFGKLLLRLPSLRSINSSIVEQLFFVRLVGKTPIDTLL
RDMLLSGTPTSWPYLPCS

>Q6LDB2_9MURI
MNYPSTSPGSLVKHICAICGDRSSGKHYGVYSCEGCKGFFKRTIRKDLIYTCRDNKDCLIDKRQRNRCQY
CRYQKCLVMGMKREAVQEERQRSRERAESEAECASSSHEDMPVERILEAELAVEPKTESYGDMNVENSTN
DPVTNICHAADKQLFTLVEWAKRIPHFSDLTLEDQVILLRAGWNELLIASFSHRSVSVQDGILLATGLHV
HRSSAHSAGVGSIFDRVLTELVSKMKDMQMDKSELGCLRAIVLFNPDAKGLSNPSEVETLREKVYATLEA
YTKQKYPEQPGRFAKLLLRLPALRSIGLKCLEHLFFFKLIGDTPIDSFLMEMLETPLQIT

>Q8AXB6_BRARE

MEMADYSEALDPAYTTLEFENMQVLAMSTDSSPQESANMNAANHLGAGTLCAICGDRATGKHYGASSCDG
CKGFFRRSVRKNHMYSCRFNRQCIVDKDKRNQCRYCRLKKCFRAGMKKEAVQNERDRISTRRSSYEDSSL
PSINALIQADVLSRQISSPGPILNGDIRTKKVAAIMDVCESMKQQLLVLVEWAKYIPAFCDLPLDDQVAL
LRAHAGEHLLLGAAKRSMMYKDILLLGNDHIIPRNCPELEVSRVAVRILDELVLPFQDLQIDDNEYACLK
AIVFFDPDAKGLSDPSKIKRMRYQVQVSLEDYINDRQYDSRGRFGELLLLLPTLQSITWQMIEQIQFVKL
FGMAKIDNLLQEMLLGGSANEAPHAHHSLHPHLVQEHLSNNVIVTANMATPLHNGQMSTPETPIPSPPTA
SGSDHYKMASGVIATVPKQPSSIPQPTITKQEAI

>Q8I1M8_9NEOP
MEPSREPGLNLEGSFMSPMSPPEMKPDTAMLDVLRDDATSPPSFKNYPPNHPLSGSKHLCSICGDRASGK
HYGVYSCEGCKGFFKRTVRKDLTYACREERNCIIDKRQRNRCQYCRYQKCLACGMKREAVQEERQRAARG
TEDPLPSSSVQELSIERLLEMESLVADTSEECQFLRVGPESNVPPKFRAPVSSLCQIGNKQIAALVVWAR
DIPHFSQLEMDDQVLLIKGAWNELLLFAIAWRSMEFLNDERENMDGSRTTSPPQLMCLMPGMTLHRNSAL
QAGVGQIFDRVLSELSLKMRHLRMDQAEYVALKAIILLNPDIKGLGNRQEVEVLREKMYSCLDEYCRRVR
VSEEGRFASLLLRLPALRSISLKSFEHLFFFHLVADSSIAGYIRDLLRHHAPPIDASALM

>HNF4A_XENLA
MDMADYTEALDPAYTTLEFENMQVLSIGTDTSTSDVTSLSASNSIGINSLCAICGDRATGKHYGASSCDG
CKGFFRRSVRKNHMYSCRFSRQCVVDKDKRNQCRYCRLKKCFRAGMKKEAVQNERDRISTRRSSYEDSSL
PSINVLIQAEVLSQQITSSVGVLNTDIRGKKIACIIDVCDSMKQQLLVLVEWAKYIPAFCELPLDDQVAL
LRAHAGEHLLLGATKRSMMFKDILLLGNDRLIPRNCPELEVGRVAVRILDELVLPFQELQIDDNEYACLK
AIIFFDPDAKGLSDPTKIKRMRYQVQVSLEDYINDRQYDSRGRFGELLLLLPTLQSITWQMIEQIQFVKL
FGMAKIDNLLQEMLLGGSANEASHTHHHLHPHLVQDHLATNVIVANNTLPSQLHNGQMSTPETPQPSPPA
GSGAEQYKIVHGTIASINKQPTSIPQSTITKQEAM

>Q8JID3_CHICK
MATIEELAHQIIEQQMGEITHSQGAVTQTLMDGAAQRIQIVPSDSGLSLPQRIQILTDNSSNEQALNKVF
DLCVVCGDKASGRHYGAVTCEGCKGFFKRSIRKNLVYSCRGTKDCVINKHHRNRCQYCRLQRCIAFGMKQ
DSVQCERKPIEVSREKSSNCAASTEKIYIRKDLRSPLAATPTFVTDNETARSTGLLESGMFVNIQSGIKS
EPTVLMTPDKVEACQGDLSTLANVVTSLANLSKSKDLSQSGTELSMIESLSNGDGSLPELQQEEQGSSDV
TRAFDTLAKALNPGESAACQNSESAEANVQLGAEETSMNIVETSMNIVEIEGPLLSDAHVAFRLTMPSPM
PEYLNVHYICETASRLLFLSMHWARSIPSFQALGQDNSISLVKACWNELFTLGLAQCSQVMNVATILAAF
VNHLHDSLQQDKLPTDRGKLVMEHIFKLQEFCNSMVKLCLDGYEYAYLKAIVLLSPDHPGLENVVQIEKF
QEKAYMEFQDYVTKAYPDDTYRLSRLLLRLPALRLMSAAITEELFFAGLIGNVQIDSIIPYILRMETADY
NTQIIGHAV

>Q6B4V6_HUMAN
MVSVNAPLGAPVESSYDTSPSEGTNLNAPNSLGVSALCAICGDRATGKHYGASSCDGCKGFFRRSVRKNH
MYSCRFSRQCVVDKDKRNQCRYCRLKKCFRAGMKKEAVQNERDRISTRRSSYEDSSLPSINALLQAEVLS
RQITSPVSGINGDIRAKKIASIADVCESMKEQLLVLVEWAKYIPAFCELPLDDQVALLRAHAGEHLLLGA
TKRSMVFKDVLLLGNDYIVPRHCPELAEMSRVSIRILDELVLPFQELQIDDNEYAYLKAIIFFDPDAKGL
SDPGKIKRLRSQVQVSLEDYINDRQYDSRGRFGELLLLLPTLQSITWQMIEQIQFIKLFGMAKIDNLLQE
MLLGGSPSDAPHAHHPLHPHLMQEHMGTNVIVANTMPTHLSNGQMCEWPRPRGQAATPETPQPSPPGGSG
SEPYKLLPGAVATIVKPLSAIPQPTITKQEVI

>Q6B4V7_HUMAN
MVSVNAPLGAPVESSYDTSPSEGTNLNAPNSLGVSALCAICGDRATGKHYGASSCDGCKGFFRRSVRKNH
MYSCRFSRQCVVDKDKRNQCRYCRLKKCFRAGMKKEAVQNERDRISTRRSSYEDSSLPSINALLQAEVLS
RQITSPVSGINGDIRAKKIASIADVCESMKEQLLVLVEWAKYIPAFCELPLDDQVALLRAHAGEHLLLGA
TKRSMVFKDVLLLGNDYIVPRHCPELAEMSRVSIRILDELVLPFQELQIDDNEYAYLKAIIFFDPDAKGL
SDPGKIKRLRSQVQVSLEDYINDRQYDSRGRFGELLLLLPTLQSITWQMIEQIQFIKLFGMAKIDNLLQE
MLLGGSPSDAPHAHHPLHPHLMQEHMGTNVIVANTMPTHLSNGQMSTPETPQPSPPGGSGSEPYKLLPGA
VATIVKPLSAIPQPTITKQEVI

>Q6B4V5_HUMAN
MVSVNAPLGAPVESSYDTSPSEGTNLNAPNSLGVSALCAICGDRATGKHYGASSCDGCKGFFRRSVRKNH
MYSCRFSRQCVVDKDKRNQCRYCRLKKCFRAGMKKEAVQNERDRISTRRSSYEDSSLPSINALLQAEVLS
RQITSPVSGINGDIRAKKIASIADVCESMKEQLLVLVEWAKYIPAFCELPLDDQVALLRAHAGEHLLLGA
TKRSMVFKDVLLLGNDYIVPRHCPELAEMSRVSIRILDELVLPFQELQIDDNEYAYLKAIIFFDPDAKGL
SDPGKIKRLRSQVQVSLEDYINDRQYDSRGRFGELLLLLPTLQSITWQMIEQIQFIKLFGMAKIDNLLQE
MLLGGPCQAQEGRGWSGDSPGDRPHTVSSPLSSLASPLCRFGQVA

>HNF4B_XENLA
MDMPDYTETLDSSYTMLEFDSIRVLPSNTEIITVETASPGLLNNGINSFCAICGDRATGKHYGASSCDGC
KGFFRRSVRKNHVYACRFSRQCIVDKDKRNQCRYCRLRKCFRAGMKKEAVQNERDRISMRRSSYEDNGSL
SINVLTQAEAMVHQYSPVSPVHSSDISMKKVASISDVCESMKQQLLLVEWAKYIPAFCELPLDDQVALL
RAHAGAHLLLGVAKRSLPYKDFLLLGNDFIMPMHCPELEIARVPCRILDELVKPLREIQIDDNEYVCLKA
IIFFDPDCKGLSDQTKVKNMRFQVQVNLEDYINDRQFDSRGRFSDILLLLPPLQSITWQMIEQVQFAKLF
GVARIDSLLQELLLGGTTMDGGQYINSGHSSLNLDLLPGPTVHSHNLHSVIHTVSSLSPETSPPTNSTSE
DYKMNTATVSSIPLMQRTVIAKKEIL

>O77100_AEDAE
MDINQNNHDANDSLSPTHTSESRFTPEQSVDSNKNYTLDNCQSSANNLCTICSDRATGKHYGAASCDGCK
GFFRRTVRKNHSYTCRFSRQCVVDKDKRNQCRYCRLRKCFKAGMKKEAVQNERDRISCRRPSMEDIDTSN
GLSVKFLLLAENRSRHFGAALDDAYDGDGDLSNKRFASINDVCDSMKQQLLILVEWAKSIPAFAELQLDD
QVALLRAHAGEHLLLGLSRRSMHLEEMLLLGNNCIITKQSPDSKMAPNLDISRIGARIIDELVSAIKDIK
LDDSELACIKALVFFDPTVRGLNQPQKIKALRHQVLNNLEDYVSDKQYDSRGRFGEILLLLPVLQSITWQ
MIQQIELAKMFGVAHIDSLLQEMLLGGETIENTAPPTPPLNSFPNSSNSPPHMMSCDTTQRPSNAMEISR
SNPTTTSSNCDAIDSESIDGANDMMAPAIIEDISNYNIPQTTNSFQRDENVQNYIHPSNDDVYSNQYISP
ASSGMHPVHASANRHQQTNVLLPVNQLSREDYLKLERELKREPEANGY

>Q7YRQ4_BOVIN
MQILYNSNDSSVPETTSMNTADNGVNCLCAICGDRATGKHYGASSCDGCKGFFRRSIRKSHVYSCRFSRQ
CVVDKDKRNQCRYCRLRKCFRAGMKKEAVQNERDRISTRRSTFDGSNIPSINTLAQAEVRSRQISVSSPC
ASADINVKKIASIGDVCESMKQQLLVLVEWAKYIPAFCELPLDDQVALLRAHAGEHLLLGATKRSMMYKD
ILLLGNNYVIHRNSCEVEISRVANRVLDELVRPFQEIQIDDNEYACLKAIVFFDPDAKGLSDPVKIKNMR
FQVQLSLEDYINDRQYDSRGRFGELLLLLPTLQSITWQMIEQIQFVKLFGMVKIDNLLQEMLLGGASTEP
NHLHHPMHPHLSQDPLTGQTILLGSMSTLVHTDQISTPETPLPSPPQGSGQEPYKIAANQASVISHQSLS

KQKQL

>TR2_HUMAN
MATIEEIAHQIIEQQMGEIVTEQQTGQKIQIVTALDHNTQGKQFILTNHDGSTPSKVILARQDSTPGKVF
LTTPDAAGVNQLFFTTPDLSAQHLQLLTDNSPDQGPNKVFDLCVVCGDKASGRHYGAVTCEGCKGFFKRS
IRKNLVYSCRGSKDCIINKHHRNRCQYCRLQRCIAFGMKQDSVQCERKPIEVSREKSSNCAASTEKIYIR
KDLRSPLTATPTFVTDSESTRSTGLLDSGMFMNIHPSGVKTESAVLMTSDKAESCQGDLSTLANVVTSLA
NLGKTKDLSQNSNEMSMIESLSNDDTSLCEFQEMQTNGDVSRAFDTLAKALNPGESTACQSSVAGMEGSV
HLITGDSSINYTEKEGPLLSDSHVAFRLTMPSPMPEYLNVHYIGESASRLLFLSMHWALSIPSFQALGQE
NSISLVKAYWNELFTLGLAQCWQVMNVATILATFVNCLHNSLQQDAKVIAALIHFTRRAITDL

>Q15626_HUMAN
MATIEEIAHQIIEQQMGEIVTEQQTGQKIQIVTALDHNTQGKQFILTNHDGSTPSKVILARQDSTPGKVF
LTTPDAAGVNQLFFTTPDLSAQHLQLLTDNSPDQGPNKVFDLCVVCGDKASGRHYGAVTCEGCKGFFKRS
IRKNLVYSCRGSKDCIINKHHRNRCQYCRLQRCIAFGMKQDSVQCERKPIEVSREKSSNCAASTEKIYIR
KDLRSPLTATPTFVTDSESTRSTGLLDSGMFMNIHPSGVKTESAVLMTSDKAESCQGDLSTLANVVTSLA
NLGKTKDLSQNSNEMSMIESLSNDDTSLCEFQEMQTNGDVSRAFDTLAKALNPGESTACQSSVAGMEGSV
HLITGDSSINYTEKEGPLLSDSHVAFRLTMPSPMPEYLNVHYIGESASRLLFLSMHWALSIPSFQALGQE
NSISLVKAYWNELFTLGLAQCWQVMNVATILATFVNCLHNSLQQAEG

>NR2E3_HUMAN
METRPTALMSSTVAAAAPAAGAASRKESPGRWGLGEDPTGVSPSLQCRVCGDSSSGKHYGIYACNGCSGF
FKRSVRRRLIYRCQVGAGMCPVDKAHRNQCQACRLKKCLQAGMNQDAVQNERQPRSTAQVHLDSMESNTE
SRPESLVAPPAPAGRSPRGPTPMSAARALGHHFMASLITAETCAKLEPEDADENIDVTSNDPEFPSSPYS
SSSPCGLDSIHETSARLLFMAVKWAKNLPVFSSLPFRDQVILLEEAWSELFLLGAIQWSLPLDSCPLLAP
PEASAAGGAQGRLTLASMETRVLQETISRFRALAVDPTEFACMKALVLFKPETRGLKDPEHVEALQDQSQ
VMLSQHSKAHHPSQPVRFGKLLLLLPSLRFITAERIELLFFRKTIGNTPMEKLLCDMFKN

>Q6DEH0_BRARE
MSKPAGSTSGCLDILNVKSSRILDIPCKVCGDRSSGKHYGVYACDGCSGFFKRSIRRNRTYVCKSGTQGG
CPVDKTHRNQCRACRLKKCLEVNMNKDAVQHERGPRTSTIRKQVALYFRGHKEVNGSSTHFPSTSIPGPP
FFTTVTQLEPHNLELNTVTSTPERQAIVGLAQPTPKYPHEVSGTPMYLYEVATESVCESAARLLFMSIKW
AKSVPAFSTLPLPDQLILLEDAWRELFVLGIAQWAIPVDSSTLLAVSGMNTENTDSQRLNKIISEIQALQ
EVVTRFRQLRLDATEFACLKCIVTFKAVPTHSGSELRSFRNASAIAALQDEAQLTLNSYIHTRYPTQPCR
FGKLLLLLPALRSVGPSTIEEVFFKKTIGNVPITRLLSDMYKSSDI

>O46175_BOMMO
MGLRDSDSDMQLETSSSEASAASSTTISQHCAICGDRATGKHYGASSCDGCKGFFRRSVRKNHLYTCRFS
RNCVVDKDKRNQCRYCRLRKCFKAGMKKEAVQNERDRINCRRPSYEEPTQANGLSVVSLLNAELLSRKVI
DETVNVSDAEINNRKLAKINDVCDSIKQQLLILVEWAKYIPAFTVLHLDDQVALLRAHAGEHLLLGCARR
SLHLKDILLLGNNCIITKHNIDGRMDIDISMIGMRVMDEIVKPLREIDIDDTEFACLKAIVFFDPNAKGL
SQPQKIKQLRYQIQINLEDYISDRQYDGRGRFGELLLCLPPLQSITWQMIEQIQFAKLFGVAHVDSLLQE
MLLGGATTEAVLEEPSPEPAAASPSPPLVPLVPLVPQLPQLPGNDSAFLEPMPFKQEPNI

>NR2E1_ORYLA
MSKPTGSTSGCLDILNVKSSRILDIPCKVCGDRSSGKHYGVYACDGCSGFFKRSIRRNRIYLCKSGSQGG
CPVDKTHRNQCRACRLKKCLEVNMNKDAVQHERGPRTSTIRKQVALYFRGHKEVNGSSTHFPGSSLPGPP
FFTTVTQLEPHNLEMSSVATTPERQAIVGLAQPTPKYPHEVSGTPMYLYEVATESVCESAARLLFMSIKW
AKSVPAFSTLPLSDQLILLEDAWRELFVLGIAQWAIPVDSTTLLAVSGLNSENMEAQRMNKIMAEIQALQ
EVVTRFRQMRLDATEFACLKCIVTFKAVPTQGSAELRAFRNASAIAALQDEAQLTLNSYIHTRYPTQPCR
FGKLLLLLPALRSVSPSTIEEVFFKKNIGNVPITRLLSDMYKSSDI

>Q8JHW6_FUGRU
MSKAAGSTSGCLDILNVKSSRILDIPCKVCGDRSSGKHYGVYACDGCSGFFKRSIRRNRTYVCKSGSQGG
CPVDKTHRNQCRACRLKKCLEVNMNKDAVQHERGPRTSTIRKQVALYFRGHKEVNGSAAHFPGSSLPGPP
FFTTVAQLEPHNLDLSTVASTPERQTIVGLAQPTPKYPHEVSGTPMYLYEVATESVCESAARLLFMSIKW
AKSVPAFSTLPLSDQLILLEDAWRELFVLGIAQWAIPVDPTTLLAVSGMNTENTESQRMTKIMSEIQALQ
EVVTRFRQMRLDATEFACLKCIVTFKAVPTQGNAEIRSFRNASAIAALQDEAQLTLNSYIHTRYPTQPCR
FGKLLLLLPSLHSISPSTIEEVFFKKTIGNVPITRLLSDMYKSSDI

>Q9Y1J4_SCHMA
MNFSETAAHQSECSIVVSQLSTKKLTTPVLCCISSNSNNIPIISQQSPNNNNNNNNNNNVVFKTDNTKQH
IIDQNILTKTATTTSIVQPSELCHIQPILISSPVKNNNNNTHNLGTGSYIDSNCLSNQSNNDKSHSITPS
SPLLTNCNSPSTTLQSSTVCCLLRPSGRSTSTSSGSSCGSSCSGTGGGVSVSSGQYICSICSDRASGKHY
GVFSCEGCKGFFKRTVRKELTYICRDSQECQIDKRLRNRCQYCRYQKCLRAGMRREAVQEERQQQQLQSE
VQRSPTPPEQNCDLSVNSMIMSDTKITNAMNHSCLAEKQEIMLKTTNCTSSSSPHLLSNCSDSSINYFYS
ASNEKSQQPSINDNFNLTVNDAATYPPQELSLIGNKDSNNVTLPLADIHALKLPTTTSAAIPPPPDALEF
IRTAESTISSRRKQWLSAFNKQQCHAEIAKCFQDSMENLKWLENNFEKCTTNHLPLFDLVIWSSKLPYIC
QLSCGVHLDLLKSACMQLIIVNLVYWLANDHKPRSLSTSNSTSKLPDTTPTINSTDISNITDDPPENSIS
DISKDCTIQMKKINKSVPLDEKMDYYYSNFPEFHLLNNLTKPMDNNNNDSISSKPTNINDNSVDDDMIRK
RNTNVYKLIYNLAIKLRMLNLDPVELGCLKLILLLNPDSMTCLNNIRSLIELLRDQVYAGLKYYCNQVWP
NAPHGRMGRLLLKLSNFQSVAARIEKLICSNELNNLLNNLESIFSYLSKKKVDHSNYISTTTTTTTTMSS
TTTSTSNSIHLEFS

>NR2E1_MOUSE
MSKPAGSTSRILDIPCKVCGDRSSGKHYGVYACDGCSGFFKRSIRRNRTYVCKSGNQGGCPVDKTHRNQC
RACRLKKCLEVNMNKDAVQHERGPRTSTIRKQVALYFRGHKEDNGAAAHFPSTALPAPAFFTAVTQLEPH
GLELAAVSATPERQTLVSLAQPTPKYPHEVNGTPMYLYEVATESVCESAARLLFMSIKWAKSVPAFSTLS
LQDQLMLLEDAWRELFVLGIAQWAIPVDANTLLAVSGMNTDNTDSQKLNKIISEIQALQEVVARFRQLRL
DATEFACLKCIVTFKAVPTHSGSELRSFRNAAAIAALQDEAQLTLNSYIHTRYPTQPCRFGKLLLLLPAL
RSISPSTIEEVFFKKTIGNVPITRLLSDMYKSSDI

>HNF4G_MOUSE
MNTTDSGVNCLCAICGDRATGKHYGASSCDGCKGFFRRSIRKSHVYSCRFSRQCVVDKDKRNQCRYCRLR
KCFRAGMKKEAVQNERDRISTRRSTYEGSNIPSINTLAQAEVRSCQISVPSPSSSTDINIKKIASISDVC
ESMKQQLLVLVEWAKYIPAFCELPLDDQVALLRAHAGEHLLLGATKRSMMYKDILLLGNHYVIHRNSCEV
EVSRVANRVLDELVRPFQEIQIDDNEYACLKAIVFFDPDAKGLSDPVKIKNMRFQVQISLEDYINDRQYD
SRGRFGELLLLLPTLQSITWQMIEQIQFVKLFGMVKIDNLLQEMLLGGAANDGSHLHHPMHPHLSQDPLT

GQTILLGPMSTLVHTDQIATPETPLPSPPQGSGQEPYKITANQASVISHQSLSKQKQL

>Q7Z2V9_HUMAN

MNTTDNGVNCLCAICGDRATGKHYGASSCDGCKGFFRRSIRKSHVYSCRFSRQCVVDKDKRNQCRYCRLR
KCFRAGMKKEAVQNERDRISTRRSTFDGSNIPSINTLAQAEVRSRQISVSSPGSSTDINVKKIASIGDVC
ESMKQQLLVLVEWAKYIPAFCELPLDDQVALLRAHAGEHLLLGATKRSMMYKDILLLGNNYVIHRNSCEV
EISRVANRVLDELVRPFQEIQIDDNEYACLKAIVFFDPDAKGLSDPVKIKNMRFQVQIGLEDYINDRQYD
SRGRFGELLLLLPTLQSITWQMIEQIQFVKLFGMVKIDNLLQEMLLGGASNDGSHLHHPMHPHLSQDPLT
GQTILLGPMSTLVHADQISTPETPLPSPPQGSGQEQYKIAANQASVISHQHLSKQKQL

>NR2E1_CHICK

MSKPAGSTSRILDIPCKVCGDRSSGKHYGVYACDGCSGFFKRSIRRNRTYVCKSGNQGGCPVDKTHRNQC
RACRLKKCLEVNMNKDAVQHERGPRTSTIRKQVALYFRGHKEESSGAPHFPATALPAPAFFTAVSQLEPH
GLELAAVAGTPERQALVGLAQPTPKYPHEVNGTPMYLYEVATESVCESAARLLFMSIKWAKSVPAFSTLS
LQDQLMLLEDAWRELFVLGIAQWAIPVDANTLLAVSGMNGDNTDSQKLNKIISEIQALQEVVARFRQLRL
DATEFACLKCIVTFKAVPTHSGSELRSFRNAAAIAALQDEAQLTLNSYIHTRYPTQPCRFGKLLLLLPAL
RSISPSTIEEVFFKKTIGNVPITRLLSDMYKSSDI

>NR2E1_HUMAN

MSKPAGSTSRILDIPCKVCGDRSSGKHYGVYACDGCSGFFKRSIRRNRTYVCKSGNQGGCPVDKTHRNQC
RACRLKKCLEVNMNKDAVQHERGPRTSTIRKQVALYFRGHKEENGAAAHFPSAALPAPAFFTAVTQLEPH
GLELAAVSTTPERQTLVSLAQPTPKYPHEVNGTPMYLYEVATESVCESAARLLFMSIKWAKSVPAFSTLS
LQDQLMLLEDAWRELFVLGIAQWAIPVDANTLLAVSGMNGDNTDSQKLNKIISEIQALQEVVARFRQLRL
DATEFACLKCIVTFKAVPTHSGSELRSFRNAAAIAALQDEAQLTLNSYIHTRYPTQPCRFGKLLLLLPAL
RSISPSTIEEVFFKKTIGNVPITRLLSDMYKSSDI

>HNF4G_HUMAN

MNTTDNGVNCLCAICGDRATGKHYGASTCDGCKGFFRRSIRKSHIYSCRFSRQCVVDKDKRNQCRYCRLR
KCFRAGMKKEAVQNERDRISTRRSTFDGSNIPSINTLAQAEVRSRQISVSSPGSSTDINVKKIASIGDVC
ESMKQQLLVLVEWAKYIPAFCELPLDDQVALLRAHAGEHLLLGATKRSMIYKDILLLGNNYVIHRNSCEV
EISRVANRVLDELVRPFQEIQIDDNEYACLKAIVFFDPDAKGLSDPVKIKNMRFQVQIGLEDYINDRQYD
SRGRFGELLLLLPTLQSITWQMIEQIQFVKLFGMVKIDNLLQEMLLGGASNDGSHLHHPMHPHLSQDPLT
GQTILLGPMSTLVHADQISTPETPLPSPPQGSGQEQYKIAANQASVISHQHLSKQKQL

>Q8MX79_BRAFL

MMSPNSVLFTPGSGDTKPQELCVVCGDKASGRHYGAISCEGCKGFFKRSIRKNLGYVCRSSKECPINKHH
RNRCQYCRLQKCLSVGMRSESVQCERKPLDPTARERPPNCATSTEKIYIRKDLRSPLAATPTFVTEREPK
SGGLFDAGMLANIQQGDSNAPILLAVDPTKSDGKEGDGNTDLSTLANVVTTLASMSKNKEGQAQQNGGEG
DAAISGDGTVMEVQTSTDISKAFDNLTKALNTSQTGEQGITSDGSQGMEGTSDQDQTTVIELEGPLLTDA
HTAFKLTTPSPMPEFLNVHYICESASRLLFLTMHWARSIPAFQALGQECHTGLVRVCWSELFTLGLSQCS
AVMSLPTILTAIVNHLQSSVQADKLSADRVKVVMEHIWKLQEFINKMAALTVDPTEYAYMKAIVLFSTDH
PGLLNPRQIEKFQEKAHEELKEYEAQAHPDDTERFAKLLLRLPALRLLSPSIMEELFFAGLIGNVQIDSI
IPYILRMETADYNSQIGGTSQ

>Q90WV5_CHICK
MAASPAGSVVSAGLEDSPSGLSPAPGKALSPVLLCKVCGDTSSGKHYGIYACNGCSGFFKRSVRRKLIYR
CQAGTGLCPVDKAHRNQCQACRLKKCLQAGMNKDAVQNERQPRSTAQVRLDSIELDAELPPEHVAATRDV
PPVPCPAPRGHGATAATAAVPRTPTPPTNHRFMASLMTAETCAKLEPEDVDETVDVTGGEPERAGGEYQV
APYPAAGPENVYETSARLLFMAVKWAKNLPVFSNLPFRDQVILLEEAWSELFLLCAIQWSMPLESCPLLA
VPEPSPGKLLPAAVDVRALQETLGRFKALAVDPTEFACMKAVVLFKPETRGLKDPEQVENLQDQSQVMLG
QHNRSHYPGQPVRFGKLLLLLPALRFLSSERVELLFFRRTIGNTPMEKLLCDMFKN

>NR2E1_XENLA
MSNPTGSTSRILDIPCKVCGDRSSGKHYGVYACDGCSGFFKRSIRRNRSYVCKSGNQGGCPVDKTHRNQC
RACRLKKCLEVNMNKDAVQHERGPRTSTIRKQVALYFRGHKEVNGSTQHFSSTALPTPTFFTTVRQLEAH
NLELAAISTVPERQTLVGLAQPTPKYPHEVNGAPLYLYEFATESVCESAARLLFMSIKWAKSVPAFSTLS
LQDQLMLLEDAWRELFVLGIAQWAIPVDASTLLAVSGMNNENTESPKLNKIISEIQALQDVVSRFRQLRL
DATEFACLKCIVTFKAGVSTHSGSELRNFRNAAAISALQDEAQLTLNSYIHTRYPTQPCRFGKLLLLLPA
LRSINPSTIEEVFFKKTIGNVPITRVLSDMYKSSDI

>Q9VP28_DROME
MDGVKVETFIKSEENRAMPLIGGGSASGGTPLPGGGVGMGAGASATLSVELCLVCGDRASGRHYGAISCE
GCKGFFKRSIRKQLGYQCRGAMNCEVTKHHRNRCQFCRLQKCLASGMRTVQHERKPIVDRKEGIIAAAGS
SSTSGGGNGSSTYLSGKSGYQQGRGKGHSVKAESAATPPVHSAPATAFNLNENIFPMGLNFAELTQTLMF
ATQQQQQQQQHQQSGSYSPDIPKADPEDDEDDSMDNSSTLCLQLLANSASNNNSQHLNFNAGEVPTALP
TTSTMGLIQSSLDMRVIHKGLQILQPIQNQLERNGNLSVKPECDSEAEDSGTEDAVDAELEHMELDFECG
GNRSGGSDFAINEAVFEQDLLTDVQCAFHVQPPTLVHSYLNIHYVCETGSRIIFLTIHTLRKVPVFEQLE
AHTQVKLLRGVWPALMAIALAQCQGQLSVPTIIGQFIQSTRQLADIDKIEPLKISKMANLTRTLHDFVQE
LQSLDVTDMEFGLLRLILLFNPTLLQQRKERSLRGYVRRVQLYALSSLRRQGGIGGGEERFNVLVARLLP
LSSLDAEAMEELFFANLVGQMQMDALIPFILMTSNTSGL

>HR78_DROME
MDGVKVETFIKSEENRAMPLIGGGSASGGTPLPGGGVGMGAGASATLSVELCLVCGDRASGRHYGAISCE
GCKGFFKRSIRKQLGYQCRGAMNCEVTKHHRNRCQFCRLQKCLASGMRSDSVQHERKPIVDRKEGIIAAA
GGSSTSGGGNGSSTYLSGKSGYQQGRGKGHSVKAESAATPPVHSAPATAFNLNENIFPMGLNFAELTQTL
MFATQQQQQQQQHQQSGSYSPDIPKADPEDDEDDSMDNSSTLCLQLLANSASNNNSQHLNFNAGEAPTA
LPTTSTMGLIQSSLDMRVIHKGLQILQPIQNQLERNGNLSVKPECDSEAEDSGTEDAVDAELEHMELDFE
CGGNRSGGSDFAINEAVFEQDLLTDVQCAFHVQPPTLVHSYLNIHYVCETGSRIIFLTIHTLRKVPVFEQ
LEAHTQVKLLRGVWPALMAIALAQCQGQLSVPTIIGQFIQSTRQLADIDKIEPLKISKMANLTRTLHDFV
QELQSLDVTDMEFGLLRLILLFNPTLLQQRKERSLRGYVRRVQLYALSSLRRQGGIGGGEERFNVLVARL
LPLSSLDAEAMEELFFANLVGQMQMDALIPFILMTSNTSGL

>TLL_DROVI
MQSSEGSPDMMDQKYNSVRLSPAASSRILYHVPCKVCRDHSSGKHYGIYACDGCAGFFKRSIRRSRQYVC
KSQKQGLCVVDKTHRNQCRACRLRKCFEVGMNKDAVQHERGPRNSTLRRHMAMYKDAMMGAAEMPQIPPE
ILMNTAALTGFPGLPMPMPGVQRSHHHAALSAAFQPPPSAAVLDLSVPRVPHHPVHQGHHGFFSPTAAYM
NALATRALPPTPPLMAAEHIKETAAEHLFKNVNWIKSVRAFTELPMPDQLLLLEESWKEFFILAMAQYLM
PMNFAQLLFVYESENANREIVTIVAREVHAFQAVPNRLCHLNIDSTEYECLRAISLFRKSPPAASSTEDL

ANSSILTGSGSPNSSASAESRGLLESSKVAAMHNDARNALHNYISRTHPNQPLRFQTLLGVVTLMHKVSS
FTIEELFFRKTIGDITIVRLISDMYSQRKI

>Q9VML1_DROME
MGTAGDRLLDIPCKVCGDRSSGKHYGIYSCDGCSGFFKRSIHRNRIYTCKATGDLKGRCPVDKTHRNQCR
ACRLAKCFQSAMNKDAVQHERGPRKPKLHPQLHHHHHHAAAAAAAAHHAAAAHHHHHHHHHAHAAAAHHA
AVAAAAASGLHHHHHAMPVSLVTNVSASFNYTQHISTHPPAPAAPPSGFHLTASGAQQGPAPPAGHLHHG
GAGHQHATAFHHPGHGHALPAPHGGVVSNPGGNSSAISGSGPGSTLPFPSHLLHHNLIAEAASKLPGITA
TAVAAVVSSTSTPYASAAQTSSPSSNNHNYSSPSPSNSIQSISSIGSRSGGGEEGLSLGSESPRVNVETE
TPSPSNSPPLSAGSISPAPTLTTSSGSPQHRQMSRHSLSEATTPPSHASLMICASNNNNNNNNNNNNGEH
KQSSYTSGSPTPTTPTPPPPRSGVGSTCNTASSSSGFLELLLSPDKCQELIQYQVQHNTLLFPQQLLDSR
LLSWEMLQETTARLLFMAVRWVKCLMPFQTLSKNDQHLLLQESWKELFLLNLAQWTIPLDLTPILESPLI
RERVLQDEATQTEMKTIQEILCRFRQITPDGSEVGCMKAIALFAPETAGLCDVQPVEMLQDQAQCILSDH
VRLRYPRQATRFGRLLLLLPSLRTIRAATIEALFFKETIGNVPIARLLRDMYTMEPAQVDK

>Q5U7E0_MUSDO
MQTTEGSPDIMDQKYNSVRLSPAASSRILYHVPCKVCRDHSSGKHYGIYACDGCAGFFKRSIRRSRQYVC
KSQKQGLCVVDKTHRNQCRACRLRKCFEVGMNKDAVQHERGPRNSTLRRHMAMYKDAMMGGSEMPQIPAE
ILMNTAALTGFPGLPMPIPGSHHMHPSLAGAFPAPPSVLDLSVPRVPQHPMHQAHPGFFAPTAAYMNALA
ATRVLPPTPPLMAAEHIKETAAEHLFKNINWIKNVPSFGELPLPDQLQLLEDSWKEFFILAMAQYLMPMN
FTQLLFVYESENPNRDVTGLVTREVHAFQDVLNQLCHLNIDSHEYELIRALTLFRRPGSDDLANSSLSTS
NGSPNSSISAESRGLIESTKIAALHDESRNALIGYIARLHPGQPMRFQSIMSVLTQMHKVSSFAIEELFF
RKTIGDITIVRLIGDMYSQRKI

>Q86PK5_CAEEL
MTLEEKEEVSTSTSQSPQSSSFENVFCAICGDRATGKHYGAMSCDGCKGFFRRTIRKRHSYVCRFGEKCQ
VDKAKRNSCRKCRFDVCLRKGMRRDAVQTERDRIRPANPLSNGSNGGIVPDDPLLDTLIRAEASTRGLRT
TVITKTAEARKQATTNDVTDSMNQQLTLMVEWAKVLEGFQRVDNITQVALLRHFSAQHLVMCAAFRSIHL
SDAVWLTNETCLHKDSPKIPDMNRVAERIIDQVTNPMRSLHMNEIEYIALKAIAFFDPLAKGITSESYSD
VEEMRQRILESFERHVRYVSPYKDMPLRFANLLLLLPPMLAISRDLVEDVQLAKLFGLASIDNLMLELML
PNEGKNTTDKTSPPIMCHQ

>Q61WY2_CAEBR
MTPVDKEEPATSSTSQSPNSFENVFCAICGDKATGKHYGALSCDGCKGFFRRTIRKHHSYVCRFGEKCQV
DKAKRNSCRKCRFDVCIRKGMKRDAVQTERDRIRPLNVFLNGKLTEDPLLETLQRAEQATRGLRTTVITK
TSEARKQATTVDVTDSMNQQLRLMVEWAKMLEGFIRVDCITQAALLRHFSAQHLVMCAAFRSIHLSDAVW
LTNETCLHKDSPKIPDMNRVAERIIDQVTTPMRQLHMSEIEYIALKAITFFDPLAKGISSESHGDVEDMR
QKILEAFERHVRFSSPHKDMPLRFANLLLLLPPMLAISRDLVEDVQLAKLFGLASIDDLMLELMLPNEKS
VGSDKGESPLTGQQ

>Q8IVZ9_HUMAN
MCPVDKAHRNQCQACRLKKCLQAGMNQDAVQNERQPRSTAQVHLDSMESNTESRPESLVAPPAPAGRSPR
GPTPMSAARALGHHFMASLITAETCAKLEPEDADENIDVTSNDPEFPSSPYSSSSPCGLDSIHETSARLL
FMAVKWAKNLPVFSSLPFRDQVILLEEAWSELFLLGAIQWSLPLDSCPLLAPPEASAAGGAQGRLTLASM

ETRVLQETISRFRALAVDPTEFACMKALVLFKPETRGLKDPEHVEALQDQSQVMLSQHSKAHHPSQPVRF
GKLLLLLPSLRFITAERIELLFFRKTIGNTPMEKLLCDMFKN

>Q688B5_CAEEL
MIFFQNFDHTKLKIPKNQFLSKFQLFPTHFPFFDSKTVLFSEEMTLEEKEEVSTSTSQSPQSSSFENVFC
AICGDRATGKHYGAMSCDGCKAKRNSCRKCRFDVCLRKGMRRDAVQTERDRIRPANPLSNGSNGGIVPDD
PLLDTLIRAEASTRGLRTTVITKTAEARKQATTNDVTDSMNQQLTLMVEWAKVLEGFQRVDNITQVALLR
HFSAQHLVMCAAFRSIHLSDAVWLTNETCLHKDSPKIPDMNRVAERIIDQVTNPMRSLHMNEIEYIALKA
IAFFDPLAKGITSESYSDVEEMRQRILESFERHVRYVSPYKDMPLRFANLLLLLPPMLAISRDLVEDVQL
AKLFGLASIDNLMLELMLPNEGKNTTDKTSPPIMCHQ

>O77101_AEDAE
MDINQNTVQNERDRISCRRPSMEDIDTSNGLSVKFLLLAENRSRHFGAALDDAYDGDGDLSNKRFASIND
VCDSMKQQLLILVEWAKSIPAFAELQLDDQVALLRAHAGEHLLLGLSRRSMHLEEMLLLGNNCIITKQSP
DSKMAPNLDISRIGARIIDELVSAIKDIKLDDSELACIKALVFFDPTVRGLNQPQKIKALRHQVLNNLED
YVSDKQYDSRGRFGEILLLLPVLQSITWQMIQQIELAKMFGVAHIDSLLQEMLLGGETIENTAPPTPPLN
SFPNSSNSPPHMMSCDTTQRPSNAMEISRSNPTTTSSNCDAIDSESIDGANDMMAPAIIEDISNYNIPQT
TNSFQRDENVQNYIHPSNDDVYSNQYISPASSGMHPVHASANRHQQTNVLLPVNQLSREDYLKLERELKR
EPEANGY

>Q86PK4_CAEEL
MTLEEKEEVSTSTSQSPQSSSFENVFCAICGDRATGKHYGAMSCDGCKAKRNSCRKCRFDVCLRKGMRRD
AVQTERDRIRPANPLSNGSNGGIVPDDPLLDTLIRAEASTRGLRTTVITKTAEARKQATTNDVTDSMNQQ
LTLMVEWAKVLEGFQRVDNITQVALLRHFSAQHLVMCAAFRSIHLSDAVWLTNETCLHKDSPKIPDMNRV
AERIIDQVTNPMRSLHMNEIEYIALKAIAFFDPLAKGITSESYSDVEEMRQRILESFERHVRYVSPYKDM
PLRFANLLLLLPPMLAISRDLVEDVQLAKLFGLASIDNLMLELMLPNEGKNTTDKTSPPIMCHQ

(3) 148 NR3

>GCR_SAISC
MDSKESLTPGKEENPSSVLTQERGNVMDFCKILRGGATLKVSVSSTSLAAASQSDSKQQRLLVDFPKGSV
SNAQQPDLSKAVSLSMGLYMGETETKVMGNDLGFPQQGQISLSSGETDLQLLEESIANLNRSTSVPENPK
SSASSSVSAAPKEKEFPKTHSDVSSEQQNLKGQTGTNGGNVKLYTADQSTFDILQDLEFSSGSPGKETNQ
SPWKSDLLIDENCLLSPLAGEEDSFLLEGNSNEDCKPLILPDTKPKIKDNGDLVLSSSSNVTLPQVKTEK
EDFIELCTPGVIKQEKLSTVYCQASFPGANIIGNKMSAISIHGVSTSGGQMYHYDMNTASLSQQQDQKPI
FNVIPPIPVGSENWNRCQGSGDDNLTSLGTLNFPGRTVFSNGYSSPSMRPDVSSPPSSSSTATTGPPPKL
CLVCSDEASGCHYGVLTCGSCKVFFKRAVEGQHNYLCAGRNDCIIDKIRRKNCPACRYRKCLQAGMNLEA
RKTKKKIIKGIQQATTGVSQETSENPANKTIVPATLPQLTPTLVSLLEVIEPEVLYAGYDSTVPDSTWRI
MTTLNMLGGRQVIAAVKWAKAIPGFRNLHLDDQMTLLQYSWMFLMAFALGWRSYRQASSNLLCFAPDLII
NEQRMTLPCMYDQCKHMLYVSSELHRLQVSYEEYLCMKTLLLLSSVPKDGLKSQELFDEIRMTYIKELGK
AIVKREGNSSQNWQRFYQLTKLLDSMHEVVENLLNYCFQTFLDKTMSIEFPEMLAEIITNQLPKYSNGNI
KKLLFHQK

>GCR_HUMAN
MDSKESLTPGREENPSSVLAQERGDVMDFYKTLRGGATVKVSASSPSLAVASQSDSKQRRLLVDFPKGSV

SNAQQPDLSKAVSLSMGLYMGETETKVMGNDLGFPQQGQISLSSGETDLKLLEESIANLNRSTSVPENPK
SSASTAVSAAPTEKEFPKTHSDVSSEQQHLKGQTGTNGGNVKLYTTDQSTFDILQDLEFSSGSPGKETNE
SPWRSDLLIDENCLLSPLAGEDDSFLLEGNSNEDCKPLILPDTKPKIKDNGDLVLSSPSNVTLPQVKTEK
EDFIELCTPGVIKQEKLGTVYCQASFPGANIIGNKMSAISVHGVSTSGGQMYHYDMNTASLSQQQDQKPI
FNVIPPIPVGSENWNRCQGSGDDNLTSLGTLNFPGRTVFSNGYSSPSMRPDVSSPPSSSSTATTGPPPKL
CLVCSDEASGCHYGVLTCGSCKVFFKRAVEGQHNYLCAGRNDCIIDKIRRKNCPACRYRKCLQAGMNLEA
RKTKKKIKGIQQATTGVSQETSENPGNKTIVPATLPQLTPTLVSLLEVIEPEVLYAGYDSSVPDSTWRIM
TTLNMLGGRQVIAAVKWAKAIPGFRNLHLDDQMTLLQYSWMFLMAFALGWRSYRQSSANLLCFAPDLIIN
EQRMTLPCMYDQCKHMLYVSSELHRLQVSYEEYLCMKTLLLLSSVPKDGLKSQELFDEIRMTYIKELGKA
IVKREGNSSQNWQRFYQLTKLLDSMHEVVENLLNYCFQTFLDKTMSIEFPEMLAEIITNQIPKYSNGNIK
KLLFHQK

>GCR_SAIBB
MDSKESLTPGKEENPSSVLTQERGNVMDFCKILRGGATLKVSVSSTSLAAASQSDSKQQRLLVDFPKGSV
SNAQQPDLSKAVSLSMGLYMGETETKVMGNDLGFPQQGQISLSSGETDLQLLEESIANLNRSTSVPENPK
SSASSSVSAAPKEKEFPKTHSDVSSEQQNLKGQTGSNGGNVKLYTADQSTFDILQDLEFSSGSPGKETNQ
SPWKSDLLIDENCLLSPLAGEEDSFLLEGNSNEDCKPLILPDTKPKIKDNGDLVLSSSSNVTLPQVKTEK
EDFIELCTPGVIKQEKLSTVYCQASFPGANIIGNKMSAISIHGVSTSGGQMYHYDMNTASLSQQQDQKPI
FNVIPPIPVGSENWNRCQGSGDDNLTSLGTLNFPGRTVFSNGYSSPSMRPDVSSPPSSSSTATTGPPPKL
CLVCSDEASGCHYGVLTCGSCKVFFKRAVEGQHNYLCAGRNDCIIDKIRRKNCPACRYRKCLQAGMNLEA
RKTKKKIKGIQQATTGVSQETSENPANKTIVPATLPQLTPTLVSLLEVIEPEVLYAGYDSTVPDSTWRIM
TTLNMLGGRQVIAAVKWAKAIPGFRNLHLDDQMTLLQYSWMFLMAFALGWRSYRQASSNLLCFAPDLIIN
EQRMTLPCMYDQCKHMLYVSSELHRLQVSYEEYLCMKTLLLLSSVPKDGLKSQELFDEIRMTYIKELGKA
IVKREGNSSQNWQRFYQLTKLLDSMHEVVENLLNYCFQTFLDKTMSIEFPEMLAEIITNQLPKYSNGNIK
KLLFHQK

>GCR_AOTNA
MDSKESLTPGKEENPSSVLTQERGNVMDFSKILRGGATLKVSVSSTSLAAASQSDSKQQRLLVDFPKGSV
SNAQQPDLSKAVSLSMGLYMGETETKVMGNDLGFPQQGQISLSSGETDLQLLEESIANLNRSTSVPENPK
SSASSSVSAAPKEKEFPKTHSDVSSEQQNLKGQTGTNGGNVKLYTADQSTFDILQDLEFSSGSPGKETNQ
SPWRSDLLIDENCLLSPLAGEEDSFLLEGNSNEDCKPLILPDTKPKIKDNGDLVLSSSSNVTLPQVKTEK
EDFIELCTPGVIKQEKLSTVYCQASFPGANVIGNKMSAISIHGVSTSGGQMYHYDMNTASLSQQQDQKPI
FNVIPPIPVGSENWNRCQGSGDDNLTSLGTLNFPGRTVFSNGYSSPSMRPDVSSPPSSSSTATTGPPPKL
CLVCSDEASGCHYGVLTCGSCKVFFKRAVEGQHNYLCAGRNDCIIDKIRRKNCPACRYRKCLQAGMNLEA
RKTKKKIKGIQQATTGVSQETSENPANKTIVPATLPQLTPTLVSLLEVIEPEVLYAGYDSTVPDSTWRIM
TTLNMLGGRQVIAAVKWAKAIPGFRNLHLDDQMTLLQYSWMFLMAFALGWRSYRQASSNLLCFAPDLIIN
EQRMTLPCMYDQCKHMLYVSSELHRLQVSYEEYLCMKTLLLLSSVPKDGLKSQELFDEIRMTYIKELGKA
IVKREGNSSQNWQRFYQLTKLLDSMHEVVENLLNYCFQTFLDKTMSIEFPEMLAEIITNQLPKYSNGNIK
KLLFHQK

>Q6N0A4_HUMAN
MDSKESLTPGREENPSSVLAQERGDVMDFYKTLRGGATVKVSASSPSLAVASQSDSKQRRLLVDFPKGSV
SNAQQPDLSKAVSLSMGLYMGETETKVMGNDLGFPQQGQISLSSGETDLKLLEESIANLNRSTSVPENPK
SSASTAVSAAPTEKEFPKTHSDVSSEQQHLKGQTGTNGGNVKLYTTDQSTFDILQDLEFSSGSPGKETNE

SPWRSDLLIDENCLLSPLAGEDDSFLLEGNSNEDCKPLILPDTKPKIKDNGDLVLSSPSNVTLPQVKTEK
EDFIELCTPGVIKQEKLGTVYCQASFPGANIIGNKMSAISVHGVSTSGGQMYHYDMNTASLSQQQDQKPI
FNVIPPIPVGSENWNRCQGSGDDNLTSLGTLNFPGRTVFSNGYSSPSMGPDVSSPPSSSSTATTGPPPKL
CLVCSDEASGCHYGVLTCGSCKVFFKRAVEGQHNYLCAGRNDCIIDKIRRKNCPACRYRKCLQAGMNLEA
RKTKKKIKGIQQATTGVSQETSENPGNKTIVPATLPQLTPTLVSLLEVIEPEVLYAGYDSSVPDSTWRIM
TTLNMLGGRQVIAAVKWAKAIPGFRNLHLDDQMTLLQYSWMFLMAFALGWRSYRQSSANLLCFAPDLIIN
EQRMTLPCMYDQCKHMLYVSSELHRLQVSYEEYLCMKTLLLLSSVPKDGLKSQELFDEIRMTYIKELGKA
IVKREGNSSQNWQRFYQLTKLLDSMHEVVENLLNYCFQTFLDKTMSIEFPEMLAEIITNQIPKYSNGNIK
KLLFHQK

>GCR_SAGOE
MDSKESLTPGKEENPSSVLTQERGNVMDFCKILRGGATLKVSVSSTSLAAASQSDSKQQRLLVDFPKGSV
SNAQQPDLSKAVSLSMGLYMGETETKVMGNDLGFPQQGQISLSSGETDLQLLEESIANLNRSTSVPENPK
SSASSSVSAAPKEKEFPKTHSDVSSEQQNLKGQTGTNGGNAKLCTADQSTFDILQDLEFSSGSPGKETNQ
SPWRSDLLIDENCLLSPLAGEEDSFLLEGNSNEDCKPLILPDTKPKIKDNGDLVLSSSSNVTLPQVKTEK
EDFIELCTPGVIKQEKLSTVYCQASFPGANIIGNKMSAISIHGVSTSGGQMYHYDMNTASLSQQQDQKPI
FNVIPPIPVGSENWNRCQGSGDDNLTSLGTLNFPGRTVFSNGYSSPSMRPDVSSPPSSSSTATTGPPPKL
CLVCSDEASGCHYGVLTCGSCKVFFKRAVEGQHNYLCAGRNDCIIDKIRRKNCPACRYRKCLQAGMNLEA
RKTKKKIKGIQQATTGVSQETSENPANKTIVPATLPQLTPTLVSLLEVIEPEVLYAGYDSTVPDSTWRIM
TTLNMLGGRQVIAAVKWAKAIPGFRNLHLDDQMTLLQYSWMFLMAFALGWRSYRQASSNLLCFAPDLIIN
EQRMTLPCMYDQCKHMLYVSSELHRLQVSYEEYLCMKTLLLLSSVPKDGLKSQELFDEIRMTYIKELGKA
IVKREGNSSQNWQRFYQLTKLLDSMHEVVENLLNYCFQTFLDKTMSIEFPEMLAEIITNQLPKYSNGNIR
KLLFHQK

>Q6XLJ0_CALJA
MDSKESLTPGKEENPSSVLTQERGNVMDFCKILRGGATLKVSVSSTSLAAASQSDPKQQRLLVDFPKGSV
SNVQQPDLSKAVSLSMGLYMGETETKVMGNDLGFPQQGQISLSSGETDLQLLEESIANLNRSTSVPENPK
SSASSSVSAAPKEKEFPKTHSDVSSEQQNLKGQTGTNGGNVKLYTADQSTFDILQDLEFSSGSPGKERNQ
SPWRSDLLIDENCLLSPLAGEEDSFLLEGNSNEDCKPLILPDTKPKIKDNGDLVLSSSSNVTLPQVKTEK
EDFIELCTPGVIKQEKLSTVYCQASFPGANIIGNKMSAISIHGVSTSGGQMYHYDMNTASLSQQQDQKPI
FNVIPPIPVGSENWNRCQGSGDDNLTSLGTLNFPGRTVFSNGYSSPSMRPDVSSPPSSSSTATTGPPPKL
CLVCSDEASGCHYGVLTCGSCKVFFKRAVEGQHNYLCAGRNDCIIDKIRRKNCPACRYRKCLQAGMNLEA
RKTKKKIKGIQQATTGVSQETSENPANKTIVPATLPQLTPTLVSLLEVIEPEVLYAGYDSTVPDSTWRIM
TTLNMLGGRQVIAAVKWAKAIPGFRNLHLDDQMTLLQYSWMFLMAFALGWRSYRQASSNLLCFAPDLIIN
EQRMTLPCMYDQCKHMLYVSSELHRLQVSYEEYLCMKTLLLLSSVPKDGLKSQELFDEIRMTYIKELGKA
IVKREGNSSQNWQRFYQLTKLLDSMHEVVENLLNYCFQTFLDKTMSIEFPEMLAEIITNQLPKYSNGNIK
KLLFHQK

>Q5R7M7_PONPY
MDSKESLTPGREENPSSVLAQERGNVMDFYKTLRGGATVKVSASSPSLAVASQSDSKQRRLLVDFPKGSV
SNAQQPDLSKAVSLSMGLYMGETETKVMGNDLGFPQQGQISLSSGETDLKLLEESIANLNRSTSVPENPK
SSASTAVSAAPTEKEFPKTHSDISSEQQHLKGQTGTNGGNVKLYTTDQSTFDILQDLEFSSGSPGKETNE
SPWRSDLLIDENCLLSPLAGEDDSFLLEGNSNEDCKPLILPDTKPKIKDNGDLVLSSPSNVTLPQVKTEK
EDFIELCTPGVIKQEKLGTVYCQAGFPGANIIGNKMSAISVHGVSTSGGQMYHYDMNTASLSQQQDQKPI

FNVIPPIPVGSENWNRCQGSGDDNLTSLGTLNFPGRTVFSNGYSSPSMRPDVSSPPSSSSTATTGPPPKL
CLVCSDEASGCHYGVLTCGSCKVFFKRAVEGRQHNYLCAGRNDCIIDKIRRKNCPACRYRKCLQAGMNLE
ARKTKKKIKGIQQATTGVSQETPENPANKTIVPATLPQLTPTLVSLLEVIEPEVLYAGYDSSVPDSTWRI
MTTLNMLGGRQVIAAVKWAKAIPGFRNLHLDDQMTLLQYSWMFLMAFALGWRSYRQSSANLLCSAPDLII
NEQRMTLPCMYDQCKHMLYVSSELHRLQVSYEEYLCMKTLLLLSSVPKDGLKSQELFDEIRMTYIKELGK
AIVKREGNSSQNWQRFYQLTKLLDSMHEVVENLLNYCFQTFLDKTMSIEFPEMLAEIITNQIPKYSNGNI
KKLLFHQK

>Q5R9P5_PONPY
MDSKESLTPGREENPSSVLAQERGNVMDFYKTLRGGATVKVSASSPSLAVASQSDSKQRRLLVDFPKGSV
SNAQQPDLSKAVSLSMGLYMGETETKVMGNDLGFPQQGQISLSSGETDLKLLEESIANLNRSTSVPENPK
SSASTAVSAAPTEKEFPKTHSDISSEQQHLKGQTGTNGGNVKLYTTDQSTFDILQDLEFSSGSPGKETNE
SPWRSDLLIDENCLLSPLAGEDDSFLLEGNSNEDCKPLILPDTKPKIKDNGDLVLSSPSNVTLPQVKTEK
EDFIELCTPGVIKQEKLGTVYCQAGFPGANIIGNKMSAISVHGVSTSGGQMYHYDMNTASLSQQQDQKPI
FNVIPPIPVGSENWNRCQGSGDDNLTSLGTLNFPGRTVFSNGYSSPSMRPDVSSPPSSSSTATTGPPPEL
CLVCSDEASGCHYGVLTCGSCKVFFKRAVEGQHNYLCAGRNDCIIDKIRRKNCPACRYRKCLQAGMNLEA
RKTKKKMKGIQQATTGVSQETPENPANKTIVPATLPQLTPTLVSLLEVIEPEVLYAGYDSSVPDSTWRIM
TTLNMLGGRQVIAAVKWAKAIPGFRNLHLDDQMTLLQYSWMFLMAFALGWRSYRQSSANLLCFAPDLIIN
EQRMTLPCMYDQCKHMLYVSSELHRLQVSYEEYLCMKTLLLLSSVPKDGLKSQELFDEIRMTYIKELGKA
IVKREGNSSQNWQRFYQLTKLLDSMHEVVENLLNYCFQTFLDKTMSIEFPEMLAEIITNQIPKYSNGNIK
KLLFHQK

>ANDR_PANTR
MEVQLGLGRVYPRPPSKTYRGAFQNLFQSVREVIQNPGPRHPEAASAAPPGASLLLQQQQQQQQQQQQQQ
QQQQQQQQETSPRQQQQQGEDGSPQAHRRGPTGYLVLDEEQQPSQPQSAPECHPERGCVPEPGAAVAASK
GLPQQLPAPPDEDDSAAPSTLSLLGPTFPGLSSCSADLKDILSEASTMQLLQQQQEAVSEGSSSGRARE
ASGAPTSSKDNYLGGTSTISDSAKELCKAVSVSMGLGVEALEHLSPGEQLRGDCMYAPLLGVPPAVRPTP
CAPLAECKGSLLDDSAGKSTEDTAEYSPFKGGYTKGLEGESLGCSGSAAAGSSGTLELPSTLSLYKSGAL
DEAAAYQSRDYYNFPLALAGPPPPPPPPHPHARIKLENPLDYGSAWAAAAAQCRYGDLASLHGAGAAGPG
SGSPSAAASSSWHTLFTAEEGQLYGPCGGGGGGGGGGGGGGGGGEAGAVAPYGYTRPPQGLAGQEGDFTA
PDVWYPGGMVSRVPYPSPTCVKSEMGPWMDSYSGPYGDMRLETARDHVLPIDYYFPPQKTCLICGDEASG
CHYGALTCGSCKVFFKRAAEGKQKYLCASRNDCTIDKFRRKNCPSCRLRKCYEAGMTLGARKLKKLGNLK
LQEEGEASSTTSPTEETTQKLTVSHIEGYECQPIFLNVLEAIEPGVVCAGHDNNQPDSFAALLSSLNELG
ERQLVHVVKWAKALPGFRNLHVDDQMAVIQYSWMGLMVFAMGWRSFTNVNSRMLYFAPDLVFNEYRMHKS
RMYSQCVRMRHLSQEFGWLQITPQEFLCMKALLLFSIIPVDGLKNQKFFDELRMNYIKELDRIIACKRKN
PTSCSRRFYQLTKLLDSVQPIARELHQFTFDLLIKSHMVSVDFPEMMAEIISVQVPKILSGKVKPIYFHT
Q

>ANDR_PAPHA
MEVQLGLGRVYPRPPSKTYRGAFQNLFQSVREVIQNPGPRHPEAASAAPPGASLQQQQQQQQQETSPRQQ
QQQQGEDGSPQAHRRGPTGYLVLDEEQQPSQPQSAPECHPERGCVPEPGAAVAAGKGLPQQLPAPPDEDD
SAAPSTLSLLGPTFPGLSSCSADLKDILSEASTMQLLQQQQEAVSEGSSSGRAREASGAPTSSKDNYLG
GTSTISDSAKELCKAVSVSMGLGVEALEHLSPGEQLRGDCMYAPVLGVPPAVRPTPCAPLAECKGSLLDD
SAGKSTEDTAEYSPFKGGYTKGLEGESLGCSGSAAAGSSGTLELPSTLSLYKSGALDEAAAYQSRDYYNF

PLALAGPPPPPPPPHPHARIKLENPLDYGSAWAAAAAQCRYGELASLHGAGAAGPGSGSPSAAASSSWHT
LFTAEEGQLYGPCGGGGGGGGGGGGGGAGEAGAVAPYGYTRPPQGLAGQEGDFTAPDVWYPGGMVSRVPYP
SPTCVKSEMGPWMDSYSGPYGDMRLETARDHVLPIDYYFPPQKTCLICGDEASGCHYGALTCGSCKVFFK
RAAEGKQKYLCASRNDCTIDKFRRKNCPSCRLRKCYEAGMTLGARKLKKLGNLKLQEEGEASSTTSPTEE
TAQKLTVSHIEGYECQPIFLNVLEAIEPGVVCAGHDNNQPDSFAALLSSLNELGERQLVHVVKWAKALPG
FRNLHVDDQMAVIQYSWMGLMVFAMGWRSFTNVNSRMLYFAPDLVFNEYRMHKSRMYSQCVRMRHLSQEF
GWLQITPQEFLCMKALLLFSIIPVDGLKNQKFFDELRMNYIKELDRIIACKRKNPTSCSRRFYQLTKLLD
SVQPIARELHQFTFDLLIKSHMVSVDFPEMMAEIISVQVPKILSGKVKPIYFHTQ

>ANDR_MACFA
MEVQLGLGRVYPRPPSKTYRGAFQNLFQSVREVIQNPGPRHPEAASAAPPGASLQQQQQQQQETSPRQQQ
QQQQGEDGSPQAHRRGPTGYLVLDEEQQPSQPQSAPECHPERGCVPEPGAAVAAGKGLPQQLPAPPDEDD
SAAPSTLSLLGPTFPGLSSCSTDLKDILSEASTMQLLQQQQQEAVSEGSSSGRAREASGAPTSSKDNYLG
GTSTISDSAKELCKAVSVSMGLGVEALEHLSPGEQLRGDCMYAPVLGVPPAVRPTPCAPLAECKGSLLDD
SAGKSTEDTAEYSPFKGGYTKGLEGESLGCSGSAAAGSSGTLELPSTLSLYKSGALDEAAAYQSRDYYNF
PLALAGPPPPPPPPHPHARIKLENPLDYGSAWAAAAAQCRYGDLASLHGAGAAGPGSGSPSAAASSSWHT
LFTAEEGQLYGPCGGGGGGGGGGGGGGAGEAGAVAPYGYTRPPQGLAGQEGDFTAPDVWYPGGMVSRVPYP
SPTCVKSEMGPWMDSYSGPYGDMRLETARDHVLPIDYYFPPQKTCLICGDEASGCHYGALTCGSCKVFFK
RAAEGKQKYLCASRNDCTIDKFRRKNCPSCRLRKCYEAGMTLGARKLKKLGNLKLQEEGEASSTTSPTEE
TAQKLTVSHIEGYECQPIFLNVLEAIEPGVVCAGHDNNQPDSFAALLSSLNELGERQLVHVVKWAKALPG
FRNLHVDDQMAVIQYSWMGLMVFAMGWRSFTNVNSRMLYFAPDLVFNEYRMHKSRMYSQCVRMRHLSQEF
GWLQITPQEFLCMKALLLFSIIPVDGLKNQKFFDELRMNYIKELDRIIACKRKNPTSCSRRFYQLTKLLD
SVQPIARELHQFTFDLLIKSHMVSVDFPEMMAEIISVQVPKILSGKVKPIYFHTQ

>ANDR_MACMU
MEVQLGLGRVYPRPPSKTYRGAFQNLFQSVREVIQNPGPRHPEAASAAPPGASLQQQQQQQQETSPRQQQ
QQQQGEDGSPQAHRRGPTGYLVLDEEQQPSQPQSAPECHPERGCVPEPGAAVAAGKGLPQQLPAPPDEDD
SAAPSTLSLLGPTFPGLSSCSADLKDILSEASTMQLLQQQQQEAVSEGSSSGRAREASGAPTSSKDNYLE
GTSTISDSAKELCKAVSVSMGLGVEALEHLSPGEQLRGDCMYAPVLGVPPAVRPTPCAPLAECKGSLLDD
SAGKSTEDTAEYSPFKGGYTKGLEGESLGCSGSAAAGSSGTLELPSTLSLYKSGALDEAAAYQSRDYYNF
PLALAGPPPPPPPPHPHARIKLENPLDYGSAWAAAAAQCRYGDLASLHGAGAAGPGSGSPSAAASSSWHT
LFTAEEGQLYGPCGGGGGGGGGGGGGGAGEAGAVAPYGYTRPPQGLAGQEGDFTAPDVWYPGGMVSRVPYP
SPTCVKSEMGPWMDSYSGPYGDMRLETARDHVLPIDYYFPPQKTCLICGDEASGCHYGALTCGSCKVFFK
RAAEGKQKYLCASRNDCTIDKFRRKNCPSCRLRKCYEAGMTLGARKLKKLGNLKLQEEGEASSTTSPTEE
TAQKLTVSHIEGYECQPIFLNVLEAIEPGVVCAGHDNNQPDSFAALLSSLNELGERQLVHVVKWAKALPG
FRNLHVDDQMAVIQYSWMGLMVFAMGWRSFTNVNSRMLYFAPDLVFNEYRMHKSRMYSQCVRMRHLSQEF
GWLQITPQEFLCMKALLLFSIIPVDGLKNQKFFDELRMNYIKELDRIIACKRKNPTSCSRRFYQLTKLLD
SVQPIARELHQFTFDLLIKSHMVSVDFPEMMAEIISVQVPKILSGKVKPIYFHTQ

>Q9UN21_HUMAN
MEVQLGLGRVYPRPPSKTYRGAFQNLFQSVREVIQNPGPRHPEAASAAPPGASLLLLQQQQQQQQQQQQ
QQQETSPRQQQQQQGEDGSPQAHRRGPTGYLVLDEEQQPSQPQSALECHPERGCVPEPGAAVAASKGLPQ
QLPAPPDEDDSAAPSTLSLLGPTFPGLSSCSADLKDILSEASTMQLLQQQQQEAVSEGSSSGRAREASGA
PTSSKDNYLGGTSTISDNAKELCKAVSVSMGLGVEALEHLSPGEQLRGDCMYAPLLGVPPAVRPTPCAPL

AECKGSLLDDSAGKSTEDTAEYSPFKGGYTKGLEGESLGCSGSAAAGSSGTLELPSTLSLYKSGALDEAA
AYQSRDYYNFPLALAGPPPPPPPPHPHARIKLENPLDYGSAWAAAAAQCRYGDLASLHGAGAAGPGSGSP
SAAASSSWHTLFTAEEGQLYGPCGGGGGGGGGGGGGGGGGEAGAVAPYGYTRPPQGLAGQESDFTAPDVWY
PGGMVSRVPYPSPTCVKSEMGPWMDSYSGPYGDMRLETARDHVLPIDYYFPPQKTCLICGDEASGCHYGA
LTCGSCKVFFKRAAEGKQKYLCASRNDCTIDKFRRKNCPSCRLRKCYEAGMTLGARKLKKLGNLKLQEEG
EASSTTSPTEETTQKLTVSHIEGYECQPIFLNVLEAIEPGVVCAGHDNNQPDSFAALLSSLNELGERQLV
HVVKWAKALPGLRNLHVDDQMAVIQYSWMGLMVFAMGWRSFTNVNSRMLYFAPDLVFNEYRMHKSRMYSQ
CVRMRHLSQEFGWLQITPQEFLCMKAMLLFSIIPVDGLKNQKFFDELRMNYIKELDRIIACKRKNPTSCS
RRFYQLTKLLDSVHPIARELHQFTFDLLIKSHMVSVDFPEMMAEIISVQVPKILSGKVKPIYFHTQ

>Q5S4M0_PIG
MDPKESLTPPSREEIPSSVLGRERAHVMDFYKSLRGGTPVKVSAASPSLAAVSQPDSKQQRLAVDFPKGS
GSNAQQPDLSKAVSLSMGLYMGETETKVMGSDLGFPQQGQISLSSGETDFRLLEESIANLSRSTSVPENP
KSSASAAGPAAPAEKAFPKTHSDGAPEQPNVKGQTGTNGGNVKLFTTDQSTFDIWRKKLQDLELPSGSPG
KETSESPWSSDLLIDENCLLSPLAGEEDPFLLEGSSTEDCKPLVLPDTKPKVKDNGELILPSPNSVPLPQ
VKTEKEDFIELCTPGVIKQEKLGPAYCQASFSGANIIGGKMSAISVHGVSTSGGQLYHYDMNTAASLSKQ
QEQKPLFNVIPPIPVGSENWNRCQGSGDDNLTSLGTLNFSGRSVFSNGYSSPGMRPDVSSPPSSSSAATG
PPPKLCLVCSDEASGCHYGVLTCGSCKVFFKRAVEGQHNYLCAGRNDCIIDKIRRKNCPACRYRKCLQAG
MNLEARKTKKKIKGIQQATTGVSQETSENSANKTIVPATLPQLTPTLVSLLEVIEPEVLYAGYDSSIPDS
TWRIMTALNMLGGRQVIAAVKWAKAIPGFRNLHLDDQMTLLQYSWMFLMVFALGWRSYRQSSASLLCFAP
DLVINEQRMALPCMYDQCRHMLYVSSELQRLQVSYEEYLCMKTLLLLSSVPKDGLKSQELFDEIRMTYIK
ELGKAIVKREGNSSQNWQRFYQLTKLLDSMHDVVENLLNYCFQTFLDKTMSIEFPEMLAEIITNQLPKYS
SGNIKKLLFHQK

>GCR_MOUSE
MDSKESLAPPGRDEVPSSLLGRGRGSVMDLYKTLRGGATVKVSASSPSVAAASQADSKQQRILLDFSKGS
ASNAQQQQQQQQPQPDLSKAVSLSMGLYMGETETKVMGNDLGYPQQGQLGLSSGETDFRLLEESIANLNR
STSRPENPKSSTPAAGCATPTEKEFPQTHSDPSSEQQNRKSQPGTNGGSVKLYTTDQSTFDILQDLEFSA
GSPGKETNESPWRSDLLIDENLLSPLAGEDDPFLLEGDVNEDCKPLILPDTKPKIQDTGDTILSSPSSVA
LPQVKTEKDDFIELCTPGVIKQEKLGPVYCQASFSGTNIIGNKMSAISVHGVSTSGGQMYHYDMNTASLS
QQQDQKPVFNVIPPIPVGSENWNRCQGSGEDNLTSLGAMNFAGRSVFSNGYSSPGMRPDVSSPPSSSSTA
TGPPPKLCLVCSDEASVCHYGVLTCGSCKVFFKRAVEGQHNYLCAGRNDCIIDKIRRKNCPACRYRKCLQ
AGMNLEARKTKKKIKGIQQATAGVSQDTSENANKTIVPAALPQLTPTLVSLLEVIEPEVLYAGYDSSVPD
SAWRIMTTLNMLGGRQVIAAVKWAKAIPGFRNLHLDDQMTLLQYSWMFLMAFALGWRSYRQASGNLLCFA
PDLIINEQRMTLPCMYDQCKHMLFISTELQRLQVSYEEYLCMKTLLLLSSVPKEGLKSQELFDEIRMTYI
KELGKAIVKREGNSSQNWQRFYQLTKLLDSMHDVVENLLSYCFQTFLDKSMSIEFPEMLAEIITNQIPKY
SNGNIKKLLFHQK

>ANDR_EULFC
MEVQLGLGRVYPRPPSKTYRGAFQNLFQSVREVIQNPGPRHPEAASAAPPGARLQQQQETSPPQQQQQQQ
GEDGSPQAQSRGPTGYLALDEEQQPSQQQSALECHPESGCVPEPGAAAAASKGLQQQPPAPSDEDDSAVP
STLSLLGPTFPGLSSCSADLKDILSEAGTMQLLQQQQQEAVSEGSSSGRAREAAGAPTSSKDSYLGGTST
ISDSAKELCKAVSVSMGLGVETLEHLSPGEQLRGDCMYAPLLGGPPAVRPTPCAPLAECKGSLLDDSADK
GTEEPAEYTPFKGSYTQGLEGESLGCSGSSEAGSSGTLELPSTLSLYKSGALEEAASYQSRDYYNFPLAL

AGPPPPPLPPHPHARIKLENPLDYGSSWAAAAAQCRFGDLASLHGGGATGPGSGSPSAAAASSWHTLFTA
EEGQLYGPCGGGGGGTSEAGAVTPYGYSRPPQGLAGQEGDFPAPDVWYPSGVVSRVPYPSPSCVKSEMGP
WMESYSGPYGDVRLETARDHVLPIDYYFPPQKTCLICGDEASGCHYGALTCGSCKVFFKRAAEGKQKYLC
ASRNDCTIDKFRRKNCPSCRLRKCYEAGMTLGARKLKKLGNLKLQEEGEASSATSPTEESSQKLTVSHIE
GYECQPIFLNVLEAIEPGVVCAGHDNNQPDSFAALLSSLNELGERQLVHVVKWAKALPGFRNLHVDDQMA
VIQYSWMGLMVFAMGWRSFTNVNSRMLYFAPDLVFNEYRMHKSRMYSQCVRMRHLSQEFGWLQITPQEFL
CMKALLLFSIIPVDGLKNQKFFDELRMNYIKELDRIIACKRKNPTSCSRRFYQLTKLLDSVQPIARELHQ
FTFDLLIKSHMVSVDFPEMMAEIISVQVPKILSGKVKPIYFHTQ

>GCR_TUPGB
MDSKESLTSPSEEIPSSVHGQERGNVMDFYKTRRGGATVKVFMPSPSLGGSSQSDSKQQRLLVDFPKGSV
SNVQQPDLSKAVSLSMGLYMGETETKVMGNDLGFPQQGQITLSSGETNLQLLEESIANLNRSTSVPEHPK
ISASVAVSAALLKKELPETPSDVSSEQQNLKGQTGTNGGNVKLCTADQSTFDILQDLEFSSASPGRETNE
SPWRSDLLLDENCLLSPLAVEDDPFLSEGNLKEDCKPLILPDTKPKIKDNGDLILSSPKNVPLPQVKTEK
EDFIELCTPGVIKQEKLGPVYCQANFSGANIIGNKMSAISVHGVSTSGGQMYHYDMNTATLSQQQDQKPI
FNVIPPIPVSSENWNRCQGSGDENLTSLGTLNFSGRSVFSNGYSSPGMRPDVSSPPSNSLSAVGPPPKFC
LVCSDEASGCHYGVLTCGSCKVFFKRAVEGQHNYLCAGRNDCIIDKIRRKNCPACRYRKCLQAGMNLEAR
KTKKKIKGIQQTTTGISQETPENSANKTIVPATLPQLTPTPVSLLEVIEPEVLYAGYDSSLPDTTWRIMS
ALNMLGGRQVIAAVKWAKAIPGFRNLHLDDQMTLLQYSWMFLMAFALGWRSYKQASANLLCFAPDLIINE
QRMSLPFMYDQCKHMLFVSSELQRLQVSYEEYLCMKTLLLLSSVPKEGLKSQELFDEIRMTYIKELGKAI
VKREGNSSQNWQRFYQLTKLLDSMHDVVENLLNYCFQTFLDKTMRIEFPEMLAEIITNQIPKYSSGNIKK
LLFHQK

>ANDR_PIG
MEVQLGLGRVYPRPPSKTFRGAFQNLFQSVREVIQNPGPRHPEAASAAPPGARLQQQQLQQQETSPRRQQ
QQQQQPSEDGSPQVQSRGPTGYLALDEKQQPSQQQSAPECHPESGCTPEPGAASAASKGLQQQPPAPPDE
DDSAAPSTLSLLGPTFPGLSSCSTDLKDILSEAGTMQLLQQQQQQQQQEAVSEGNSSGRAREATGAPIS
SKDSYLGGSSTISDSAKELCKAVSVSMGLGVEALEHLSPGEQLRGDCMYAPLLTGPPSVRPTPCAPLAEC
KGSLLDDGPGKSNEETAEYSPFKAGYTKGLDSESLGCSSGGEAGGSGTLELPSALSLYKSGALDDVAAYP
SRDYYNFPLALAGPPPPPPPPHPHARIKLENPLDYGSAWAAAAAQCRYGDLASLHGGGAPGPGSGSPSAT
SSSSWHTLFTAEESQLYGPCGGGGGGSAGEAGAVAPYGYTRPPQGLAGQEGDLAIPDIWYPGGVVSRVPY
PSPSCVKSEMGPWMESYSGPYGDMRLEPTRDHVLPIDYYFPPQKTCLICGDEASGCHYGALTCGSCKVFF
KRAAEGKQKYLCASRNDCTIDKFRRKNCPSCRLRKCYEAGMTLGARKLKKLGNLKLQEEGEASSATSPTE
EPAQKLTVSHIEGYECQPIFLNVLEAIEPGVVCAGHDNNQPDSFAALLSSLNELGERQLVHVVKWAKALP
GFRNLHVDDQMAVIQYSWMGLMVFAMGWRSFTNVNSRMLYFAPDLVFNEYRMHKSRMYSQCVRMRHLSQE
FGWLQITPQEFLCMKALLLFSIIPVDGLKNQKFFDELRMNYIKELDRIIVMQEKNPTSCSRRFYQLTKLL
DSVQPIARELHQFTFDLLIKSHMVSVDFPEMMAEIISVQVPKILSGKVKPIYFHTQ

>GCR_CAVPO
MDLKESVTSSKEVPSSVLGSERRNVIDFYKTVRGGATVKVSASSPSLAAAAQSDSKQRRLLVDFPKGSGS
NAQQPDLSKAVSLSMGLYMGETETKVMGNDLGFPQQGQISLPSGETDFRLLEESIANLSRSTSVPENPKN
SASAVSGTPTEEFPKTQSDLSSEQENLKSQAGTNGGNVKFPPDQSTFDILKDLEFSSGSPGKERSESPWR
PDLLMDESCLLSPLAGEDDPFLLEGNSNEDCKPLILPDTKPKIKDNGDGILSSSNSVPQPQVKIGKEDFI
ELCTPGVIKQEKLGPVYCQASFSGANIIGNKMSAISVHGVSTSGGQMYHYDMNTASLSQQQDQKPIFNVI

PPIPVGSENWNRCQGSGEDNLTSLGTVNFPGRSVFSNGYSSPGLRPDVSSPPSSSSTTTGPPPKLCLVCS
DELSGCHYGVLTCGSCKVFFKRAVEGQHNYLCAGRNDCIIDKIRRENCPACRYRKCLQAGMNLQARKTKK
KIKGIQQATTGVSQNTSENPNKTIVPATLPQLTPTLVSLLEVIEPEVIHSGYDSTSPDSTWRIMTTLNML
GGRQVIAAVKWAKAIPGFKNLHLDDQMTLLQYSWMFLMAFALGWRSYKQSNGSLLCFAPDLIINEQRMSL
PWMYDQCRYMLYVSSELKRLQVSYEEYLCMKTLLLLSSVPKEGLKSQELFDEIRMTYIKELGKAIVKREG
NSSQNWQRFYQLTKLLDSLHEIVGNLLNICFKTFLDKTMNIEFPEMLAEIITNQLPKYSNGDIKKLLFHQ
K

>PRGR_HUMAN
MTELKAKGPRAPHVAGGPPSPEVGSPLLCRPAAGPFPGSQTSDTLPEVSAIPISLDGLLFPRPCQGQDPS
DEKTQDQQSLSDVEGAYSRAEATRGAGGSSSSPPEKDSGLLDSVLDTLLAPSGPGQSQPSPPACEVTSSW
CLFGPELPEDPPAAPATQRVLSPLMSRSGCKVGDSSGTAAAHKVLPRGLSPARQLLLPASESPHWSGAPV
KPSPQAAAVEVEEEDGSESEESAGPLLKGKPRALGGAAAGGGAAAVPPGAAAGGVALVPKEDSRFSAPRV
ALVEQDAPMAPGRSPLATTVMDFIHVPILPLNHALLAARTRQLLEDESYDGGAGAASAFAPPRSSPCASS
TPVAVGDFPDCAYPPDAEPKDDAYPLYSDFQPPALKIKEEEEGAEASARSPRSYLVAGANPAAFPDFPLG
PPPPLPPRATPSRPGEAAVTAAPASASVSSASSSGSTLECILYKAEGAPPQQGPFAPPPCKAPGASGCLL
PRDGLPSTSASAAAAGAAPALYPALGLNGLPQLGYQAAVLKEGLPQVYPPYLNYLRPDSEASQSPQYSFE
SLPQKICLICGDEASGCHYGVLTCGSCKVFFKRAMEGQHNYLCAGRNDCIVDKIRRKNCPACRLRKCCQA
GMVLGGRKFKKFNKVRVVRALDAVALPQPLGVPNESQALSQRFTFSPGQDIQLIPPLINLLMSIEPDVIY
AGHDNTKPDTSSSLLTSLNQLGERQLLSVVKWSKSLPGFRNLHIDDQITLIQYSWMSLMVFGLGWRSYKH
VSGQMLYFAPDLILNEQRMKESSFYSLCLTMWQIPQEFVKLQVSQEEFLCMKVLLLLNTIPLEGLRSQTQ
FEEMRSSYIRELIKAIGLRQKGVVSSSQRFYQLTKLLDNLHDLVKQLHLYCLNTFIQSRALSVEFPEMMS
EVIAAQLPKILAGMVKPLLFHKK

>ANDR_CANFA
MEVQLGLGRVYPRPPSKTYRGAFQNLFQSVREVIQNPGPRHPEAVSAAPPGAHLQQQQQQQQQETSPRQ
QQQQQQGDDGSPQAQSRGPTGYLALDEEQQPSQQRSASKGHPESACVPEPGVTSATGKGLQQQQPAPPDE
NDSAAPSTLSLLGPTFPGLSSCSTDLKDILSEAGTMQLLQQQRQQQQQQQQQQQQQQQQQQQEVVSEGSS
SGRAREAAGASTSSKDSYLGGSSTISDSAKELCKAVSVSMGLGVEALEHLSPGEQLRGDCMYAPLLGGPP
AVRPCAPLAECKGSLLDDGPGKGTEETAEYSPFKAGYAKGLDGDSLGCSSSSEAGGSGTLEMPSTLSLYK
SGALDEAAAYQSRDYYNFPLSLGGPPPHPPPPHPHTRIKLENPLDYGSAWAAAAAQCRYGDLASLHGAGA
AGPSSGSPSATTSSSWHTLFTAEEGQLYGPCGGSGGGSAGDGGSVAPYGYTRPPQGLAGQEGDFPPPDVW
YPGGVVSRVPFPSPSCVKSEMGSWMESYSGPYGDMRLETARDHVLPIDYYFPPQKTCLICGDEASGCHYG
ALTCGSCKVFFKRAAEGKQKYLCASRNDCTIDKFRRKNCPSCRLRKCYEAGMTLGARKLKKLGNLKLQEE
GEASNVTSPTEEPTQKLTVSHIEGYECQPIFLNVLEAIEPGVVCAGHDNNQPDSFAALLSSLNELGERQL
VHVVKWAKALPGFRNLHVDDQMAVIQYSWMGLMVFAMGWRSFTNVNSRMLYFAPDLVFNEYRMHKSRMYS
QCVRMRHLSQEFGWLQITPQEFLCMKALLLFSIIPVDGLKNQKFFDELRMNYIKELDRIIACKRKNPTSC
SRRFYQLTKLLDSVQPIARELHQFTFDLLIKSHMVSVDFPEMMAEIISVQVPKILSGKVKPIYFHTQ

>ANDR_CROCR
MEVQLGLGRVYPRPPSKTYRGAFQNLFQSVREVIQNPGPRHPEATSAAPPGARLQQQHQHQQQHQHETSP
RRQQQQQPEDGSPQRPSRGPTSYLALDEEQQPSQHQSAKGHPESGCVPEPVAMSRTGKGLEQQQPAPPDE
DDSAAPSTLSLLGPTFPGLSSCSTDLKDILSEAGTMQLLQRQRQRQQQRQQQQQQQQQQQQQQQQQEVV
SEGGSSGRAREAAGAPTSSKDSYLGGSSTISDSAKELCKAVSVSMGLGVEALEHLSPGEQLRGDCMYAPL

LGGPPPVCPCAPLTECKGSVLDDGPSKGTEETAEYSPFKTGYAKGLDGDSLGCSGSSQAGGSGTLEIPST
LSLYKSGTLDEAAAYQSRDYYNFQLSLAGPPPPPPSPHPHARIKLENPLDYGSAWAAAAAQCRYGDLASL
HGGGAAGPGSGSPSATASSSWHTLFTAEEGQLYGPCGGSGGGGTGESVSVTPYGYTRPQQGLTGQEGDFP
PPDVWYPGGVVSRMPYPSASCVKSEMGPWMESYSGPYGDMRLETTRDHVLPIDYYFPPQKTCLICGDEAS
GCHYGALTCGSCKVFFKRAAEGKQKYLCASRNDCTIDKFRRKNCPPCRLRKCYEAGMTLGARRLKKLGNL
KLQEEGEASSTTSPTEETTQKLTVSHIEGYECQPIFLNVLEAIEPGVVCAGHDNNQPDSFAALLSSLNEL
GERQLVHVVKWAKALPGFRNLHVDDQMAVIQYSWMGLMVFAMGWRSFTNVNSRMLYFAPDLVFNEYRMHK
SRMYSQCVRMRHLSQEFGWLQITPQEFLCMKALLLFSIIPVDGLKNQKFFDELRMNYIKDLDRIIACKRK
NPTSCSRRFYQLTKLLDSVQPIARELHQFTFDLLIKSHMVSVDFPEMMAEIISVQVPKILSGKVKPIYFH
TQ

>GCR_RABIT
MDSKESLSPPGREEVPSSVLRPAERGNVMDLYKTLRGGAPVRVPASSPSLAPAAQPDSKQQRLAVDFPKG
SASNAQQPDLSRAVSLSMGLYMGETETKVMGSDLAFPQQGQTSLSSGETDFRLLEESIASLNRSASGADN
PRSTAPAAGSAAPTEGFPKTHSDLASERQNPKGQTGGSAGSAKLHPTDQSTFDILQDLEFSGSPSKDRSE
SPWRSDLLMDENCLLSPLAGEDDPFLLEGNSSEDCKPLILPDTKPKIKDNGDLILSNSNNVPLPQVKTEK
EDFIELCTPGVIKQEKLGPVYCQASFSGANIIGNKISAISVHGVSTSGGQMYHYDMNAQQQEQKPLFNVI
PPIPVGSENWNRCQGSGDDNLTSLGTMNFPGRSVFSNGYSSPGMRPDVSSPPSNSTTAAGPPPKLCLVCS
DEASGCHYGVLTCGSCKVFFKRAVKGQHNYLCAGRNDCIIDKIRRKNCPACRYRKCLQAGMNLEARKTKK
KIKGIQQTSTGVSQETSENPSNRTVVPAALPQLTPTLVSLLEVIEPEVLYAGYDSSVPDSTWRIMTTLNM
LGGRQVIAAVKWAKAIPGFRNLHLDDQMTLLQYSWMFLMAFALGWRSYKQSSGNMLCFAPDLVINEQRMT
LPYMYDQCKHMLFVSSELKRLQVSYEEYLCMKTLLLLSTVPKEGLKSQELFDEIRMTYIKELGKAIVKRE
GNSSQNWQRFYQLTKLLDSMHEVVENLLHYCFQTFLDKTMSIEFPEMLAEIITNQIPKYSNGNIKKLLFH
QK

>ANDR_RAT
MEVQLGLGRVYPRPPSKTYRGAFQNLFQSVREAIQNPGPRHPEAASIAPPGACLQQRQETSPRRRRRQQH
PEDGSPQAHIRGTTGYLALEEEQQPSQQQSASEGHPESGCLPEPGAATAPGKGLPQQPPAPPDQDDSAAP
STLSLLGPTFPGLSSCSADIKDILSEAGTMQLLQQQQQQQQQQQQQQQQQQQQQQEVISEGSSSVRAREA
TGAPSSSKDSYLGGNSTISDSAKELCKAVSVSMGLGVEALEHLSPGEQLRGDCMYASLLGGPPAVRPTPC
APLAECKGLSLDEGPGKGTEETAEYSSFKGGYAKGLEGESLGCSGSSEAGSSGTLEIPSSLSLYKSGAVD
EAAAYQNRDYYNFPLALSGPPHPPPPTHPHARIKLENPSDYGSAWAAAAAQCRYGDLASLHGGSVAGPST
GSPPATASSSWHTLFTAEEGQLYGPGGGGGSSSPSDAGPVAPYGYTRPPQGLASQEGDFSASEVWYPGGV
VNRVPYPSPSCVKSEMGPWMENYSGPYGDMRLDSTRDHVLPIDYYFPPQKTCLICGDEASGCHYGALTCG
SCKVFFKRAAEGKQKYLCASRNDCTIDKFRRKNCPSCRLRKCYEAGMTLGARKLKKLGNLKLQEEGENSS
AGSPTEDPSQKMTVSHIEGYECQPIFLNVLEAIEPGVVCAGHDNNQPDSFAALLSSLNELGERQLVHVVK
WAKALPGFRNLHVDDQMAVIQYSWMGLMVFAMGWRSFTNVNSRMLYFAPDLVFNEYRMHKSRMYSQCVRM
RHLSQEFGWLQITPQEFLCMKALLLFSIIPVDGLKNQKFFDELRMNYIKELDRIIACKRKNPTSCSRRFY
QLTKLLDSVQPIARELHQFTFDLLIKSHMVSVDFPEMMAEIISVQVPKILSGKVKPIYFHTQ

>ANDR_MOUSE
MEVQLGLGRVYPRPPSKTYRGAFQNLFQSVREAIQNPGPRHPEAANIAPPGACLQQRQETSPRRRRRQQH
TEDGSPQAHIRGPTGYLALEEEQQPSQQQAASEGHPESSCLPEPGAATAPGKGLPQQPPAPPDQDDSAAP
STLSLLGPTFPGLSSCSADIKDILNEAGTMQLLQQQQQQQQHQQQHQQHQQQQEVISEGSSARAREATGA

PSSSKDSYLGGNSTISDSAKELCKAVSVSMGLGVEALEHLSPGEQLRGDCMYASLLGGPPAVRPTPCAPL
PECKGLPLDEGPGKSTEETAEYSSFKGGYAKGLEGESLGCSGSSEAGSSGTLEIPSSLSLYKSGALDEAA
AYQNRDYYNFPLALSGPPHPPPPTHPHARIKLENPLDYGSAWAAAAAQCRYGDLGSLHGGSVAGPSTGSP
PATTSSSWHTLFTAEEGQLYGPGGGGGSSSPSDAGPVAPYGYTRPPQGLTSQESDYSASEVWYPGGVVNR
VPYPSPNCVKSEMGPWMENYSGPYGDMRLDSTRDHVLPIDYYFPPQKTCLICGDEASGCHYGALTCGSCK
VFFKRAAEGKQKYLCASRNDCTIDKFRRKNCPSCRLRKCYEAGMTLGARKLKKLGNLKLQEEGENSNAGS
PTEDPSQKMTVSHIEGYECQPIFLNVLEAIEPGVVCAGHDNNQPDSFAALLSSLNELGERQLVHVVKWAK
ALPGFRNLHVDDQMAVIQYSWMGLMVFAMGWRSFTNVNSRMLYFAPDLVFNEYRMHKSRMYSQCVRMRHL
SQEFGWLQITPQEFLCMKALLLFSIIPVDGLKNQKFFDELRMNYIKELDRIIACKRKNPTSCSRRFYQLT
KLLDSVQPIARELHQFTFDLLIKSHMVSVDFPEMMAEIISVQVPKILSGKVKPIYFHTQ

>PRGR_RABIT
MTELKAKEPRAPHVAGGAPSPTEVGSQLLGRPDPGPFQGSQTSEASSVVSAIPISLDGLLFPRPCQGQNP
PDGKTQDPPSLSDVEGAFPGVEAPEGAGDSSSRPPEKDSGLLDSVLDTLLAPSGPGQSHASPATCEAISP
WCLFGPDLPEDPRAAPATKGVLAPLMSRPEDKAGDSSGTAAAHKVLPRGLSPSRQLLLPSSGSPHWPAVK
PSPQPAAVQVDEEDSSESEGTVGPLLKGQPRALGGTAAGGGAAPVASGAAAGGVALVPKEDSRFSAPRVS
LAEQDAPVAPGRSPLATSVVDFIHVPILPLNHAFLATRTRQLLEGESYDGGAAAASPFVPQRGSPSASST
PVAGGDFPDCTYPPDAEPKDDAFPLYGDFQPPALKIKEEEEAAEAAARSPRTYLVAGANPAAFPDFQLAA
PPPPSLPPRVPSSRPGEAAVAASPGSASVSSSSSSGSTLECILYKAEGAPPQQGPFAPLPCKPPGAGACL
LPRDGLPSTSASGAAAGAAPALYPTLGLNGLPQLGYQAAVLKEGLPQVYTPYLNYLRPDSEASQSPQYSF
ESLPQKICLICGDEASGCHYGVLTCGSCKVFFKRAMEGQHNYLCAGRNDCIVDKIRRKNCPACRLRKCCQ
AGMVLGGRKFKKFNKVRVMRALDAVALPQPVGIPNESQRITFSPSQEIQLIPPLINLLMSIEPDVIYAGH
DNTKPDTSSSLLTSLNQLGERQLLSVVKWSKSLPGFRNLHIDDQITLIQYSWMSLMVFGLGWRSYKHVSG
QMLYFAPDLILNEQRMKESSFYSLCLTMWQIPQEFVKLQVSQEEFLCMKVLLLLNTIPLEGLRSQSQFEE
MRSSYIRELIKAIGLRQKGVVSSSQRFYQLTKLLDNLHDLVKQLHLYCLNTFIQSRALSVEFPEMMSEVI
AAQLPKILAGMVKPLLFHKK

>MCR_SAISC
METKGYHSLPEGLDMERRWGQVSQAVEHSSLGSTERTDENNYMEIVNVSCVSGAIPNNSTQGSSKEKHEL
LPCLQQDNNRPGILTSDIKTELESKELSATVAESMGLYMDSVRDADYYEQQNQQRSMSPAKIYQNVEQLV
KFYKENGHRPSTLSCVNRPLRSFMSDSVSSVNGGVMRAIVKSPIMCHEKSPSVCSPLNMTSSVCSPAGIN
SVSSTTASFGSFPVHSPITQGTPLTCSPNVENRGSRSHSPAHASNVGSPLSSPLSSMKSSISSPPSHCSV
KSPVSSPNNVTPRSSVSSPANINNSRCSVSSPSNTNNRSTLSSPAASTVGSICSPVNNAFSYTASGTSAG
SSTSRDVVPSPDTQEKGAQEVPFPKTEEVESAISNGVTGQLNIVQYIKPEPDGAFSSSCLGGNSKINSDS
PFSVPIKQESTKHSCSGTSFKGNPTVNPFPFMDGSYFSFMDDKDYYSLSGILGPPVPGFDGTCEGSGFPV
GIKQEPDDGSYYPEASIPSSAIVGVNSGGQSFHYRIGAQGTISLSRSARDQSFQHLSSFPPVNTLVESWK
SHGDLSSRRSDGYPVLEYIPENVSSSTLRSVSTGSSRPSKICLVCGDEASGCHYGVVTCGSCKVFFKRAV
EGQHNYLCAGRNDCIIDKIRRKNCPACRLQKCLQAGMNLGARRSKKLGKLKGIHEEQPQQQPPPPPPPPQ
SPEEGTTYIAPAKEPSVNTALVPQLSAISRALTPSPAMVLENIEPEVVYAGYDNSKPDTAENLLSTLNRL
AGKQMIQVVKWAKVLPGFKNLPLEDQITLIQYSWMCLSSFALSWRSYKHTNSQFLYFAPDLVFNEEKMHQ
SAMYELCQGMHQISLQFIRLQLTFEEYTIMKVLLLLSTVPKDGLKSQAAFEEMRTNYIKELRKMVTKCPN
NSGQSWQRFYQLTKLLDSMHDLVNDLLEFCFYTFRESQALKVEFPAMLVEIISDQLPKVESGNAKPLYFH
RK

>Q6XLI9_CALJA
METKGYHSLPEGLDMERRWGQVSQAVEHSSLGSTERTDENNYMEIVNVSCVSGAIPNNSTQGSSKEKHEL
LPCLQQDNNRPGVLTSDIKTELESKELSATVAESMGLYMDSVRDADYYEQQNQQRSMSPAKIYQNVEQLV
KFYKENGHRPSTLSCVNRPLRSFMSDSGSSVNGGVMRAIVRSPIMCHEKSPSVCSPLNMTSSVCSPAGIN
SESSTTASFGSFPVHSPITQGTPLTCSPNVENRGSRSHSPAHASNVGSPLSSPLSSMKSSISSPPSHCSV
KSPVSSPNNVTLRSSVSSPANINNSRCSVSSPSNTNNRSTLSSPAASTVGSICSPVNNAFSYTASGTSAG
SSTSRDVVPSPDTQEKGAQEVPFPKTEEVESAISNGVTGQLNIVQYIKPEPDGAFSSSCLGGNSKINSDS
PFSVPIKQESTKHSCSGTSFKGNPTVNPFPFMDGSYFSFMDDKDYYSLSGILGPPVPGFDGNCEGSGFPV
GIKQEPDDGSYYPEASIPSSAIVGVNSGGQSFHYRIGAQGTISLSRSARDQSFQHLSSFPPVNTLVESWK
SHGDLSSRRSDGYPVLEYIPENVSSSTLRSVSTGSSRPSKICLVCGDEASGCHYGVVTCGSCKVFFKRAV
EGQHNYLCAGRNDCIIDKIRRKNCPACRLQKCLQAGMNLGARKSKKLGKLKGIHEEQPQQQPPPPPPPP
QSPEEGTTYIAPAKEPSVNTALVPQLSTISRALTPSPAMVLENIEPEVVYAGYDNSKPDTAENLLSTLNR
LAGKQMIQVVKWAKVLPGFKNLPLEDQITLIQYSWMCLSSFALSWRSYKHTNSQFLYFAPDLVFNEEKMH
QSAMYELCQGMHQISLQFIRLQLTFEEYTIMKVLLLLSTVPKDGLKSQAAFEEMRTNYIKELRKMVTKCP
NNSGQSWQRFYQLTKLLDSMHDLVNDLLEFCFYTFRESQALKVEFPAMLVEIISDQLPKVESGNAKPLYF
HRK

>MCR_HUMAN
METKGYHSLPEGLDMERRWGQVSQAVERSSLGPTERTDENNYMEIVNVSCVSGAIPNNSTQGSSKEKQEL
LPCLQQDNNRPGILTSDIKTELESKELSATVAESMGLYMDSVRDADYSYEQQNQQGSMSPAKIYQNVEQL
VKFYKGNGHRPSTLSCVNTPLRSFMSDSGSSVNGGVMRAIVKSPIMCHEKSPSVCSPLNMTSSVCSPAGI
NSVSSTTASFGSFPVHSPITQGTPLTCSPNAENRGSRSHSPAHASNVGSPLSSPLSSMKSSISSPPSHCS
VKSPVSSPNNVTLRSSVSSPANINNSRCSVSSPSNTNNRSTLSSPAASTVGSICSPVNNAFSYTASGTSA
GSSTLRDVVPSPDTQEKGAQEVPFPKTEEVESAISNGVTGQLNIVQYIKPEPDGAFSSSCLGGNSKINSD
SSFSVPIKQESTKHSCSGTSFKGNPTVNPFPFMDGSYFSFMDDKDYYSLSGILGPPVPGFDGNCEGSGFP
VGIKQEPDDGSYYPEASIPSSAIVGVNSGGQSFHYRIGAQGTISLSRSARDQSFQHLSSFPPVNTLVESW
KSHGDLSSRRSDGYPVLEYIPENVSSSTLRSVSTGSSRPSKICLVCGDEASGCHYGVVTCGSCKVFFKRA
VEGQHNYLCAGRNDCIIDKIRRKNCPACRLQKCLQAGMNLGARKSKKLGKLKGIHEEQPQQQPPPPPPP
PQSPEEGTTYIAPAKEPSVNTALVPQLSTISRALTPSPVMVLENIEPEIVYAGYDSSKPDTAENLLSTLN
RLAGKQMIQVVKWAKVLPGFKNLPLEDQITLIQYSWMCLSSFALSWRSYKHTNSQFLYFAPDLVFNEEKM
HQSAMYELCQGMHQISLQFVRLQLTFEEYTIMKVLLLLSTIPKDGLKSQAAFEEMRTNYIKELRKMVTKC
PNNSGQSWQRFYQLTKLLDSMHDLVSDLLEFCFYTFRESHALKVEFPAMLVEIISDQLPKVESGNAKPLY
FHRK

>MCR_RAT
METKGYHSLPEGLDMERRWSQVSQTLERSSLGPAERTTENNYMEIVNVSCVSGAIPNNSTQGSSKEKHEL
LPYIQQDNSRSGILPSDIKTELESKELSATVAESMGLYMDSVRDAEYTYDQQNQQGSLSPTKIYQNMEQL
VKFYKENGHRSSTLSAMSRPLRSFMPDSAASMNGGALRAIVKSPIICHEKSSSVSSPLNMASSVCSPVGI
NSMSSSTTSFGSFPVHSPITQGTSLTCSPSVENRGSRSHSPTHASNVGSPLSSPLSSMKSPISSPPSHCS
VKSPVSSPNNVPLRSSVSSPANLNNSRCSVSSPSNNTNNRSTLSSPTASTVGSIGSPISNAFSYATSGAS
AGAGAIQDVVPSPDTHEKGAHDVPFPKTEEVEKAISNGVTGPLNIVQYIKSEPDGAFSSSCLGGNSKISP
SSPFSVPIKQESSKHSCSGASFKGNPTVNPFPFMDGSYFSFMDDKDYYSLSGILGPPVPGFDGSCEDSAF
PVGIKQEPDDGSYYPEASIPSSAIVGVNSGGQSFHYRIGAQGTISLSRSPRDQSFQHLSSFPPVNTLVES
WKPHGDLSSRRSDGYPVLEYIPENVSSSTLRSVSTGSSRPSKICLVCGDEASGCHYGVVTCGSCKVFFKR

AVEGQHNYLCAGRNDCIIDKIRRKNCPACRLQKCLQAGMNLGARKSKKLGKLKGLHEEQPQQPPPPPPQS
PEEGTTYIAPTKEPSVNSALVPQLTSITHALTPSPAMILENIEPETVYAGYDNSKPDTAESLLSTLNRLA
AKQMIQVVKWAKVLPGFKNLPLEDQITLIQYSWMCLSSFALSWRSYKHTNSQLLYFAPDLVFNEEKMHQS
AMYELCQGMRQISLQFVRLQLTFEEYSIMKVLLLLSTVPKDGLKSQAAFEEMRTNYIKELRKMVTKCPNS
SGQSWQRFYQLTKLLDSMHDLVSDLLEFCFYTFRESQALKVEFPAMLVEIITDQLPKVESGNAKPLYFHR
K

>MCR_MOUSE
METKGYHSLPEGLDMERRWSQVSQTLERSSLGPAERTNENSYMEIVNVSCVSGATPNNSTQGSSKEKHEL
LPCLQQDNSRSGILPSDIKTELESKELSATVAESMGLYMDSVRDAEYTYDQQNQQGSLSPAKIYQNMEQL
VKFYKENGHRSSTLSAISRPLRSFMPDSGTSMNGGALRAIVKSPIICHEKSPSVCSPLNMPSSVCSPAGI
NSMSSSTASFGSFPVHSPITQGTSLTCSPSVENRGSRSHSPVHASNVGSPLSSPLSSMKSPISSPPSHCS
VKSPVSSPNNVPLRSSVSSPANLNNSRCSVSSPSNTNNRSTLSSPTASTVGSIGSPISNAFSYTTSGASA
GAGAIQDMVPSPDTHEKGAHDVPFPKTEEVEKAISNGVTGQLNIVQYIKPEPDGAFSSSCLGGNNKINPS
SPFSVPIKQESSKHSCSGASFKGNPTVNPFPFMDGSYFSFMDDKDYYSLSGILGPPVPGFDSSCEGSAFP
GGIKQEPDDGSYFPETSIPSSAIIGVNSGGQSFHYRIGAQGTISLSRSPRDQSFQHLSSFPPVNALVESW
KPHGDLSSRRSDGYPVLEYIPENVSSSTLRSVSTGSSRPSKICLVCGDEASGCHYGVVTCGSCKVFFKRA
VEHNYLCAGRNDCIIDKIRRKNCPACRLQKCLQAGMNLGARKSKKLGKLKGLHEEQPQQPPPPPPQSPEE
GTTYIAPTKEPSVNSALVPQLASITRALTPSPSMILENIEPEIVYAGYDNSKPDTAESLLSTLNRLAGKQ
MIQVVKWAKVLPGFKNLPLEDQITLIQYSWMCLSSFALSWRSYKHTNSQFLYFAPDLVFNEEKMHQSAMY
ELCQGMRQISLQFVRLQLTFEEYSIMKVLLLLSTVPKDGLKSQAAFEEMRTNYIKELRKMVTKCPNSSGQ
SWQRFYQLTKLLDSMHDLVNDLLEFCFYTFRESQALKVEFPAMLVEIISDQLPKVESGNAKPLYFHRK

>PRGR_RAT
MTELQAKDPRTLHTSGAAPSPTHVGSPLLARLDPDPFQGSQHSDASSVVSPIPISLDRLLFSRSCQAQEL
PDEKTQNQQSLSDVEGAFSGVEASRRRSRNPRAPEKDSRLLDSVLDTLLAPSGPEQSQTSPPACEAITSW
CLFGPELPEDPRSVPATKGLLSPLMSRPESKAGDSSGTGAGQKVLPKAVSPPRQLLLPTSGSAHWPGAGV
KPSQQPATVEVEEDGGLETEGSAGPLLKSKPRALEGMCSGGGVTANAPGAAPGGVTLVPKEDSRFSAPRV
SLEQDAPVAPGRSPLATTVVDFIHVPILPLNHALLAARTRQLLEGDSYDGGAAAQVPFAPPRGSPSAPSP
PVPCGDFPDCTYPPEGDPKEDGFPVYGEFQPPGLKIKEEEEGTEAASRSPRPYLLAGASAATFPDFPLPP
RPPRAPPSRPGEAAVAAPSAAVSPVSSSGSALECILYKAEGAPPTQGSFAPLPCKPPAASSCLLPRDSLP
AAPTSSAAPAIYPPLGLNGLPQLGYQAAVLKDSLPQVYPPYLNYLRPDSEASQSPQYGFDSLPQKICLIC
GDEASGCHYGVLTCGSCKVFFKRAMEGQHNYLCAGRNDCIVDKIRRKNCPACRLRKCCQAGMVLGGRKFK
KFNKVRVMRALDGVALPQSVAFPNESQTLGQRITFSPNQEIQLVPPLINLLMSIEPDVVYAGHDNTKPDT
SSSLLTSLNQLGERQLLSVVKWSKSLPGFRNLHIDDQITLIQYSWMSLMVFGLGWRSYKHVSGQMLYFAP
DLILNEQRMKELSFYSLCLTMWQIPQEFVKLQVTHEEFLCMKVLLLLNTIPLEGLRSQSQFEEMRSSYIR
ELIKAIGLRQKGVVPSSQRFYQLTKLLDSLHDLVKQLHLYCLNTFIQSRALAVEFPEMMSEVIAAQLPKI
LAGMVKPLLFHKK

>PRGR_MOUSE
MTELQAKDPQVLHTSGASPSPPHIGSPLLARLDSGPFQGSQHSDVSSVVSPIPISLDGLLFPRSCRGPEL
PDGKTGDQQSLSDVEGAFSGVEATHREGGRNSRPPEKDSRLLDSVLDSLLTPSGPEQSHASPPACEAITS
WCLFGPELPEDPRSVPATKGLLSPLMSRPEIKVGDQSGTGRGQKVLPKGLSPPRQLLLPTSGSAHWPGAG
VKPSPQPAAGEVEEDSGLETEGSASPLLKSKPRALEGTGQGGGVAANAPSAAPGGVTLVPKEDSRFSAPR

VSLEQDSPIAPGRSPLATTVVDFIHVPILPLNHALLAARTRQLLEGESYDGGATAGPFCPPRSPSAPSTP
VPRGDFPDCTYPLEGDPKEDVFPLYGDFQTPGLKIKEEEEGADAAVRSPRPYLSAGASSSTFPDFPLAPA
PQAAPSSRPGEAAVAGGPSSAAVSPASSSGSALECILYKAEAPPTQGSFAPLPCKPPAAASCLLPRDSLP
AAPGTAAAPAIYQPLGLNGLPQLGYQAAVLKDSLPQVYPPYLNYLRPDSEASQSPQYGFDSLPQKICLIC
GDEASGCHYGVLTCGSCKVFFKRAMEGQHNYLCAGRNDCIVDKIRRKNCPACRLRKCCQAGMVLGGRKFK
KFNKVRVMRTLDGVALPQSVGLPNESQALSQRITFSPNQEIQLVPPLINLLMSIEPDVIYAGHDNTKPDT
SSSLLTSLNQLGERQLLSVVKWSKSLPGFRNLHIDDQITLIQYSWMSLMVFGLGWRSYKHVSGQMLYFAP
DLILNEQRMKELSFYSLCLTMWQIPQEFVKLQVTHEEFLCMKVLLLLNTIPLEGLRSQSQFEEMRSSYIR
ELIKAIGLRQKGVVPTSQRFYQLTKLLDSLHDLVKQLHLYCLNTFIQSRTLAVEFPEMMSEVIAAQLPKI
LAGMVKPLLFHKK

>MCR_TUPGB
METKGYHSRPEGLDMERRWGQVSQPVDRPSLGPAERTEENNYMEIVNVSCVSGAIPNNSTQGSSKEKQEL
LPCLQQDNTQSGILTSEIKTELEPKELSATVAESMGLYMDSVREADYAFEQHAQQGSLSPAKIYQNVEQL
MKFYKENGHRSSTLSNVSRPSRSFLPDPGSAVNGGVMRAMVKSPILCHEKSPSVCSPLNMTSSVCSPAGI
NSVSSTTVRFGSFPVHSPITQGTPLTCSPTVDNRGSRSHSPAHASNVGSPLSSPLSSMKSPISSPPSHCS
VKSPVSSPNNVTLRSCVSSPANINNSRCSVSSPSKANNRSTLSSPAASTVGSICSPNAFSYPASGASVGS
SATRDVIPSPDTHEKGAHEVPFPKTEEVENAISNGVTGQLNIVQYIKPEPDGAFSSSCLGGNSKIHSDSP
FSSVPIKQESTKHSCSGASFKGNPTVNPFPFMDGSYFSFMDDKDYYSLSGILGPPVPGFEGNCEGTGFPM
GIKQEPDYGIYYPEASIPSSAIVGVNSGGQSFHYRIGAQGTISLSRSARDQSFQHLSSFPPVNTLVESWK
SHGDLSARKIDGYPVLEYIPENVSSSTLRSVSTGSSRPSKICLVCGDGASGCHYGVVTCGSCKVFFKRAV
EGQHNYLCAGRNDCIIDKIRRKNCPACRLQKCLQAGMNLGARKSKKLGKLKGLHEEQPQQPPPPQSPEEG
TTYIAPAKEPSVNTALVPQLSSISRALTPSPVMVLENIEPEIVYAGYDSSKPDTAENLLSTLNRLAGKQM
IQVVKWAKVLPGFKNLPLEDQITLIQYSRMCLSSFALSWRSYKHTNSQFFYFAPDLVFNEEKMHQSAMYE
LCQGMHQISLQFVRLQFTFEEYTFMEVLLLLSTIPKDGLKSQAAFEEMRANYIKELRKMVTKCPNNSGQS
WQGFYQLTKFLDSMHDLVSDLLEFCFYTFRELQALKVEFPAMLVEIISDQLPKVESGNAKPLYFHRK

>Q13771_HUMAN
MQLLQQQQQEAVSEGSSSGRAREASGAPTSSKDNYLGGTSTISDNAKELCKAVSVSMGLGVEALEHLSPG
EQLRGDCMYAPLLGVPPAVRPTPCAPLAECKGSLLDDSAGKSTEDTAEYSPFKGGYTKGLEGESLGCSGS
AAAGSSGTLELPSTLSLYKSGALDEAAAYQSRDYYNFPLALAGPPPPPPPPHPHARIKLENLLDYGSAWA
AAAAQCRYGDLASLHGAGAAGPGSGSPSAAASSSWHTLFTAEEGQLYGPCGGGGGGGGGGGGGGGGEAGA
VAPYGYTRPPQGLAGQESDFTAPDVWYPGGMVSRVPYPSPTCVKSEMGPWMDSYSGPYGDMRLETARDHV
LPIDYYFPPQKTCLICGDEASGCHYGALTCGSCKVFFKRAAEGKQKYLCASRNDCTIDKFRRKNCPSCRL
RKCYEAGMTLGARKLKKLGNLKLQEEGEASSTTSPTEETTQKLTVSHIEGYECQPIFLNVLEAIEPGVVC
AGHDNNQPDSFAALLSSLNELGERQLVHVVKWAKALPGLRNLHVDDQMAVIQYSWMGLMVFAMGWRSFTN
VNSRMLYFAPDLVFNEYRMHKSRMYSQCVRMRHLSQEFGWLQITPQEFLCMKAMLIFFFFLLLFSIIPVD
GLKNQKFFDELRMNYIKELDRIIACKRKNPTSCSRRFYQLTKLLDSVQPIARELHQFTFDLLIKSHMVSV
DFPEMMAEIISVQVPKILSGKVKPIYFHTQ

>Q6DDL0_XENLA
MDPKDLLKPSSGSPAVRGSPHYNDKPGNVIEFFGNYRGGVSVSVSASCPTSTASQSNTRQQQHFQKQLTA
TGDSTNGLNNNVPQPDLSKAVSLSMGLYMGESDTKVMSSDIAFPSQEQIGISTGETDFSLLEESIANLQA
KSLAPDKLIEISEDPGGFKCDISAQPRPSMGQGGSNGSSSTNLFPKDQCTFDLLRDLGISPDSPLDGKSN

PWLDPLFDEQEAFNLLSPLGTGDPFFMKSEVLSEGSKPLSLEDGTQRLGDHAKDMLLPSADLPISQVKTE
KEDYIELCTPGVVKQEKFGPVYCVGNFSGSGLFGNKSSAISVHGVSTSGGQMYHYDLNTATISQQDVKPV
FNLGSPGTSIAEGWNRCHGSGNDTAASPGNVNFPNRSVFSNGYSSPGIRSDASPSPSTSSTSTGPPPKLC
LVCSDEASGCHYGVLTCGSCKVFFKRAVEGQHNYLCAGRNDCIIDKIRRKNCPACRYRKCLQAGMNLEAR
KTKKKIKGIQQSTTATARESPETSMTRTLVPASVAQLTPTLISLLEVIEPEVLYSGYDSSIPDTTRRLMS
SLNMLGGRQVVSAVRWAKAIPGFRNLHLDDQMTLLQYSWMFLMVFALGWRSYKQTNGSILYFAPDLVITE
DRMHLPFMQERCQEMLKIAGEMSSLQISYDEYLCMKVLLLMCTIPKEGLKSHALFEEIRMTYIKELGKAI
VKREGNSSQNWQRFYQLTKLLDSMHEVAENLLAFCFLSFLDKSMSIEFPDMLSEIISNQIPKYSSGNLKK
LLFHQK

>GCR_XENLA
MDPKDLLKPSSGSPAVRGSPHYNDKPGNVIEFFGNYRGGVSVSVSASCPTSTASQSNTRQQQHFQKQLTA
TGDSTNGLNNNVPQPDLSKAVSLSMGLYMGESDTKVMSSDIAFPSQEQIGISTGETDFSLLEESIANLQA
KSLAPDKLIEISEDPGGFKCDISAQPRPSMGQGGSNGSSSTNLFPKDQCTFDLLRDLGISPDSPLDGKSN
PWLDPLFDEQEAFNLLSPLGTGDPFFMKSEVLSEGSKTLSLEDGTQRLGDHAKDMLLPSADRPISQVKTE
KEDYIELCTPGVVNEEKFGPVYCVGNFSGSGLFGNKSSAISVHGVSTSGGQMYHYDLNTATISQQDVKPV
FNLGSPGTSIAEGWNRCHGSGNDTAASPGNVNFPNRSVFSNGYSSPGIRSDASPSPSTSSTSTGPPPKLC
LVCSDEASGCHYGVLTCGSCKVFFKRAVEGQHNYLCAGRNDCIIDKIRRKNCPACRYRKCLQAGMNLEAR
KTKKKIKGIQQSTTATARESPETSMTRTLVPASVAQLTPTLISLLEVIEPEVLYSGYDSSIPDTTRRLMS
SLNMLGGRQVVSAVRWAKAIPGFRNLHLDDQMTLLQYSWMFLMVFALGWRSYKQTNGSILYFAPDLVITE
DRMHLPFMQERCQEMLKIAGEMSSLQISYDEYLCMKVLLLMCTIPKEGLKSHALFEEIRMTYIKELGKAI
VKREGNSSQNWQRFYQLTKLLDSMHEVAENLLAFCFLSFLDKSMSIEFPDMLSEIISNQIPKYSSGNLKK
LLFHQK

>Q66J29_XENLA
METKGYKNFQEGVNMERQWGQVPRDFSSPNSRQQTPENTYMEIVNVNCDSDSFPQNNAKADSKEKQDLLP
CLQQDSSKPSLLTQELKPELESKELSAAVAESMGLYMDSVRDSDYPYDQQNQQGTNGSGTIFQSMNPFYK
QTTQGTFPADCQTRSLRSAIESDKSMNGKLKSNTVKSPLSYSEKNLSVGSPSVMALPVCSPTGISSTSCS
TTFGNFTVHSPVNQVTPKSCSPHTDNRCSIAHSPAGTVESPLSSPVSSMRSPISSPPSHASLKSPVSSPN
NITVRPSVSSPGNINTRSSLSSPSNANNRSTISSPAASSMGSSICSPASSTLGFLPGVMPTDGGTASDGI
ISAETKDKGAQQIMFPKVEEMGNEIADSTVSLVKFIKPDPDAIFSSTCFGDTVSSDPAFSIPIKQESCKN
TCSSALFKGSQSANPFPFMDGSYFAFMDDKDYYSLSGILGPPVSSFGDGFEGNGFSNQSLNVAIKQETED
SSFYPENNMPSSAIVGVNSCGQSFHYRIGAQGTISLSRPLNRDQSFQNLSSFPPMSSLVESWKTQSELAQ
NTLSSRRNDGFPVPGYIPENMSSTTLRSMSTGPSRPSKVCLVCGDEASGCHYGVVTCGSCKVFFKRAVEG
QHSYLCAGRNDCIIDKIRRKNCPACRLQKCLQAGMNLGARKSKKLGKLKGVHEEHPQQPLQQTPTASPKE
DTTLTSSSKEPSANSNSLVPLISAVSPAITLSAAVILENIEPEIVYAGYDNTQPDTAENLLSSLNQLAGK
QMVQVVKWAKVIPGFRNLPLEDQITLIQYSWMCLSSFALSWRSYKHASSQFLYFAPDLIFNEERMRQSAM
YDLCQGMQQISLEFSRLQLTFEEYTLMKVLLLLSTVPKDGLKCQAAFEEMRVNYIKELRKVLLKSPHNSG
QSWQRYFQLTKLLDSMQDLVGDLLEFCFYTFRESQALKVEFPAMLVEIISDQLPKVESGIAKPLYFHRK

>Q8NG42_HUMAN
MTELKAKGPRAPHVAGGPPSPEVGSPLLCRPAAGPFPGSQTSDTLPEVSAIPISLDGLLFPRPCQGQDPS
DEKTQDQQSLSDVEGAYSRAEATRGAGGSSSSPPEKDSGLLDSVLDTLLAPSGPGQSQPSPPACEVTSSW
CLFGPELPEDPPAAPATQRVLSPLMSRSGCKVGDSSGTAAAHKVLPRGLSPARQLLLPASESPHWSGAPV

```
KPSPQAAAVEVEEEDGSESEESAGPLLKGKPRALGGAAAGGGAAAVPPGAAAGGVALVPKEDSRFSAPRV
ALVEQDAPMAPGRSPLATTVMDFIHVPILPLNHALLAARTRQLLEDESYDGGAGAASAFAPPRSSPCASS
TPVAVGDFPDCAYPPDAEPKDDAYPLYSDFQPPALKIKEEEEGAEASARSPRSYLVAGANPAAFPDFPLG
PPPPLPPRATPSRPGEAAVTAAPASASVSSASSSGSTLECILYKAEGAPPQQGPFAPPPCKAPGASGCLL
PRDGLPSTSASAAAAGAAPALYPALGLNGLPQLGYQAAVLKEGLPQVYPPYLNYLRPDSEASQSPQYSFE
SLPQKICLICGDEASGCHYGVLTCGSCKVFFKRAMEGQHNYLCAGRNDCIVDKIRRKNCPACRLRKCCQA
GMVLGGRKFKKFNKVRVVRALDAVALPQPVGVPNESQALSQRFTFSPGQDIQLIPPLINLLMSIEPDVIY
AGHDNTKPDTSSSLLTSLNQLGERQLLSVVKWSKSLPGFRNLHIDDQITLIQYSWMSLMVFGLGWRSYKH
VSGQMLYFAPDLILNESHRSLSSFKLAKKSSSV

>GCR_PAROL
MDQGGLKRNCNRDDSLTFGETAVGVGSDTGDTAGSLLQPAAMHLPSPSSLPQLTVAPNGGAGTKDQGEFG
GLFESPRGQCEGSEMKEGKIIRLQKRKHHLDIGMFNMEDNLSLLNQNISDLNRTSTSVISTSDTSVLGKL
PLPNLFPQHIKQEGGFSLEKELGTYGGHTGGGPCDLDGNSGHLIEDTEIWQDLDLPNSLPEISDFELDSE
VAHLDNILHDSSGGCGPDGSLLKETKVLVGNGGNCTDVNGTDQQHPLQHHQHQQQQHRHLLQHQQHQLHH
QHQQPPSLLSSVMIKEEKDHDNSFIHIRTPGVVKQEKQENGSFCQSQCLQSSMSSLHGGGPMSSTMGAGA
VPGYHYKASPSSTVGLQDQKPFGIFSNLPAVAESWTRGGRFGEPSGIQRGNDGLPSAAMSPFSVSFSSSS
PRTGENSSSAVPGLSKPSGPTHKICLVCSDEASGCHYGVVTCGSCKVFFKRAVEGWRARQNTDGQHNYLC
AGRNDCIIDKIRRKNCPACRFRKCLQAGMNLEARKNKKLIKMKVHRPTGSAEPISNMPVPVIPRMPQLVP
TMLSVLKAIEPEIIYSGYDSTLPDTSTRLMTTLNRLGGQQVISAVKWAKSLPGFRNLHLDDQMTLLQCSW
LFLMSFSLGWRSYEQCNGNMLCFAPDLVINKERMKLPFMTDQCEQMLKICNEFVRLQVSYDEYLCMKVLL
LLSTVPKDGLKSQAVFDEIRMTYIKELGKAIVKREENASQNWQRFYQLTKLLDSMQEMVEGLLQICFYTF
VNKTLSVEFPEMLAEIITNQIPKFKDGSVKPLLFHQK

>Q8JJ91_HAPBU
MDQGGLKRNGNRDDGLTFAEIEGTGDTPGSLFQTAMHLPGSPPPATVAPNRQGGTNGQGELGGLFESHQH
HVLGEGTDMKEGKMIRMQKQQQQQQQDIGIFGMGDSLPLFNQCISDTPTSVINTSDTSVLGNLPLPDLF
SHPIKTESILSLDKDLGTYSGHTGTGPCDLDGNSGRLIEDTEIWQDLDLPSSLPEISAFELDSEVAHLDN
ILQESTGGGCPVGGLPKEIKPLMGNGENCTSVNGTKQQHHALHPHQQQQHHQLIHHQQHQPQHHQPQAL
LSTIIIKEEKDPDESFIQICTPGVIKQEKQDNGFCQPQCLQSGISSLHGGGPRPMSSPVSVGAVPGYHYT
ANLSSTMDIQDQKPFDMYSNMPLMGDGWARGKRYGETSGIQSSDDGPTPVASLAPFSVGFSGSSPREGEI
SSSVVPAQSKTSGQTHKICLVCSDEASGCHYGVVTCGSCKVFFKRAVEGQHNYLCAGRNDCIIDKIRRKN
CPACRFRKCLQAGMNLEARKNKKLIKMKVHRAGASEPTISNMPVPVVPRSMPQLVPTMLSILKAIEPEII
YSGYDSTLPDTSSRLMSTLNRLGGQQVVSAVKWAKSLPGFRNLHLDDQMTLLQCSWLFLMSFSLGWRSYE
QCNGSMLCFAPDLVINKDRMKLPFMTDQCEQMLKICNEFVRLQVSYEEYLCMKVLLLLSTVPKDGLKSQA
VFDEIRMTYIKELGKAIVKREENPSQNWQRFYQLTKLLDSMQEMVEGLLQICFYTFVNKTLSVEFPEMLA
EIISNQIPKFKDGNVKALLFHQK

>Q8JJ90_HAPBU
MDQGGLKRNGNRDDGLTFAEIEGTGDTPGSLFQTAMHLPGSPPPATVAPNRQGGTNGQGELGGLFESHQH
HVLGEGTDMKEGKMIRMQKQQQQQQQDIGIFGMGDSLPLFNQCISDTPTSVINTSDTSVLGNLPLPDLF
SHPIKTESILSLDKDLGTYSGHTGTGPCDLDGNSGRLIEDTEIWQDLDLPSSLPEISAFELDSEVAHLDN
ILQESTGGGCPVGGLPKEIKPLMGNGENCTSVNGTKQQHHALHPHQQQQHHQLIHHQQHQPQHHQPQAL
LSTIIIKEEKDPDESFIQICTPGVIKQEKQDNGFCQPQCLQSGISSLHGGGPRPMSSPVSVGAVPGYHYT
```

ANLSSTMDIQDQKPFDMYSNMPLMGDGWARGKRYGETSGIQSSDDGPTPVASLAPFSVGFSGSSPREGEI
SSSVVPAQSKTSGQTHKICLVCSDEASGCHYGVVTCGSCKVFFKRAVEGWRARQNTDGQHNYLCAGRNDC
IIDKIRRKNCPACRFRKCLQAGMNLEARKNKKLIKMKVHRAGASEPTISNMPVPVVPRSMPQLVPTMLSI
LKAIEPEIIYSGYDSTLPDTSSRLMSTLNRLGGQQVVSAVKWAKSLPGFRNLHLDDQMTLLQCSWLFLMS
FSLGWRSYEQCNGSMLCFAPDLVINKDRMKLPFMTDQCEQMLKICNEFVRLQVSYEEYLCMKVLLLLSTV
PKDGLKSQAVFDEIRMTYIKELGKAIVKREENPSQNWQRFYQLTKLLDSMQEMVEGLLQICFYTFVNKTL
SVEFPEMLAEIISNQIPKFKDGNVKALLFHQK

>Q5WP01_ONCMY
METKRYPSFFEGSTDTEKNRWSHVPSSAMDYCCSGAEEADSLNLNSNSDVLMDIVNVSCSPSSNTADSKE
SNNNNEEKKKPEQQPTLKLTQNQHQPFVLPLFNSSLHGRKPEMMDSKELSKTVAESMGLYMNAAREATDF
GGFGQQGGHCSPGKMYPAGVCGRPCLEDSQCAASTGSPKLMSPSTGFLKQPSSTPGDCCSSGTPSGSAVL
GLSLSCSPQAPSSISSPGGSNNLVSSTTSPTCFGGPFTCTTISNPVNQHNAAGPALAHNHVHRRNSATCS
PAGSSTVGSPPLTSPLNVMRSPISSPQSMSSVRSPPSCSTSTNMRSSSVSSPTGSTNNMRASSSISSPTS
GGPMAMSSSPRNPSGGGGGFAVSSPASELGLVQNDSNSPEGRRDQQDFKEFEFPKVESVDGEMFNVGLDH
MGMVKFIKNEPDTDYRSMCLGNGSNNTKCNQATGCPGNGSPFITQIKSEPNKDGGGCMNPQCYTEQQQQH
SMGLFQSGPSEITYLSLRDNIDEYSLSGILGPPGTEMNGSYEAGVFPHNLLSKVKQENNDGSYYQENNNN
VVPTSAIVGVNSGGHSFHYQIGAQGTMSFSRHDPRDHGTNPLLNLISPVTALMESWKSRPGMSQGPRGEG
YPGHGCMPDSMSSASLRHPSSTAKVCLVCGDEASGCHYGVVTCGSCKVFFKRAVEGQHNYLCAGRNDCII
DKIRRKNCPACRVRKCLQAGMNLGARKSKKLGKLKGVNEDSTPTKEGGQTCPGSGGGYLSSGEKELSTSP
TNALVPHGPGGGLVTPYLPPSICSVLELIEPEVVFAGYDNTQPDTTDHLLSSLNQLAGKQMIRVVKWAKV
PPGFRGLPIEDQITLIQYSWMCLSSFSLSWRSYKHTNGQMLYFAPDLVFNEDRMQQSAMYDLCLGMRQVS
QEFVRLQLTYQEFLSMKVLLLLSTVPKEGLKNQAAFEEMRVNYIKELRRSVGKATNNSGQTWQRFFQLTK
LLDAMHDLVGNLLDFCFYTFRESQALKVEFPEMLVEIISDQIPKVESGNTHTLYFHKK

>Q9I8F5_9TELE
MEACESPEAVFHGSYQSLFQNVRVKRANNESLDISSSKKCGFLQETASREMRLSKLSPRRGILRSPEKEC
ESTISVIQLAASRIHFLKSSTESKSDSSLSSSGSGLADATESSDSRAGFLRGAESGQKGYGAAEVHKHEL
GSGRDASVASCSRACSSGITISETARELCKAVSVSLGLAMESSELGEVGPHHAPPLTTESSSDEMYMPLL
DCSVSEPGAGAKDREYALAMGQRDRGLELRGRDKVLGMFKSSDLVQLAGDVTTSQYSNASKSHLTSALQE
VHEFASMSGDIANPSSEGTAASDMDATHAASCQFEQLLPASMAHFVHPEFENGPSQSFAKPSAMSGEFTE
NYANQYNVKIKAEMMPRDLNGTWAYPHRYADDSNGQYGPPKQRTTYATDHETPFISNPYEYGQSGSLVPR
ERPPPEQWYPVGMLGRPPYPNVPCVKNEVGEWLDVTSLTDGRFDAGRSDIFPMEFFLPPQRTCLICSDEA
SGCHYGALTCGSCKVFFKRAAEGKQKYLCASRNDCTIDKLRRKNCPSCRLRRCFEAGMTLGARKLRKIGQ
VKGPDEVGSVQGPSESAQCLSPKPCLTFHSQLIFLNILEAIEPEVVNAGHDHAQPDSAAALLTSLNELGE
RQLVKVVKWAKGLPGFRNLHVDDQMTVIQHTWMGVMVFALGWRSYKNANARMLYFAPDLVFNDRRMHISS
MYEHCIQMKHLSQEFVLLQVTQEEFLCMKALLLFSIIPVEGLKSQKYFDELRLTYINELDRLINYGRKSN
CAMRFQQLTRMMDSLQPIVQKLHQFTFDLFVQARSLPTKVSFPEMIAEIISVQVPKILAGLSKPILFHK

>Q8UWB7_ORENI
MSQTTRELFCAKIWPEDRKSYTGDVVSAASMAQKTEESRVYFTKTPAGSSAASLAESENANLYGHIIPLA
CDMEKRCCQTAAAPQEELSAECRVGDSRSFSACATISETARELCKAVSVSLGLAMESGDTSETHVSLPQC
AANDQMSAEYLFGVEAVPANCPGAPASLSEYRCPERDERPPHDHKQQVKMLKSSEPAAAFHHLSSTATSV
NAQNFTLCEADDITPKEIDHLDIACAASCPYAPAAPEPLAQFGPAAAQRPCRAYNPVEEAGDVGDAGRIG

PWGFRQNGTASKSNLREVHLQGRYGSPGLITAFGDKYNSQFWGSRQCMNLQSSGANSAFICNPYDGTVMR
REQWYPGGMLRTPYPNSNYMKGEVGEWLDVTYNDTRFESSREHMFPMEFFFPPQRTCLICSDEASGCHYG
ALTCGSCKVFFKRAAEGKQKYLCASKNDCTIDKLRRKNCPSCRLKKCFEAGMTLGARKLKKIGQQKSPKE
DHGAQDPVEVIPNVSPKTGVQLNSHLVFLNILESIEPEVVNAGHDCGQPDSADTLLTSLNELGERQLVKV
VKWAKGLPGFRNLHVDDQMTIIQQSWMGVMVFALGWKSYKNASGRILYFAPDLVFNEHRMHVSTMYEHCI
RMRHLSQEFEMLQITQEEFHCMKALLLFSIIPVEGLKSQKYFDELRLTYINELDRLINFQTTTNTSQRFY
QLTRLMDSLQMTVKKLHQFTFDLFVQAQSLHTKVSFPEMIGEIISVHVPKILTGLAKPILFHK

>O93245_ONCMY
MEIPVGLGGVCDSPNIVFRGPFQNVFHNVKATLPSNTTVTETLDFSSSYSFMQNKHPWEMRQTNRQSPRK
EISSGTARNSDIEVKEDDSISFSRTLESDARRIHFAKSSTGNKTGFSSVNELDYPNANGYGSGRPGPLAC
NTKQQCCQPAVPHHGVELSPNSYARVANSCSNSACTTISETARELCNAVSVSLGLTMDLNEMNDLGPNYA
PSSANDQSQGNYLFQVPLLNCSGAEENVSITEYKCPSERNARPLQSDTRVKMFKSSPANDLTEEVATMEH
LSSRHPSTGEQEFRLNEKSDDPTSKETENSLSTRARSASCHFDPLLPAHLAHFSQTDPDRISSHVIPAHV
CETGETMEDKYADYLQQQYSVKIKYEAISNEPAGTSWGSQYNGYNDNDNTQYGPRQGMNPYSAGPDSGFI
CNPYEYERGGGLVRRERPTSEQWYPGGMLGRMPYPNSPYLKNEVGDWLDVSYTDARFEGGRDHMFPMEFF
FPPQRTCLICADEASGCHYGALTCGSCKVFFKRAAEGKQKYLCASRNDCTIDKLRRKNCPSCRLRKCFEA
GMTLGARKLKKIGQLKSPEEDLPTQGPTDAIQCVSPQSGLTFHSQLVFLNILESIEPEVVNAGHDHCQPD
SAAVLLTSLNELGERQLVKVVKWAKGMPGFRNLHVDDQMTVIQHSWMGVMVFGLGWRSYKNVNARMLYFA
PDLVFNDHRMHISSMFEHCIRMRHLSQQFVLLQVTQEEFLCMKALLLFSIIPVDGLKSQKYFDELRLTYI
NELDRVINYGRKSNCSQRFYQLTRLMDSLQPIVRKLQQFTFDLFIQAQSLPTKVSFPEMIAEIISVHVPK
ILAGLAKPILFHK

>Q8QFV7_HAPBU
MSQTSRQLFCAKIWPEDRKSYTGNVVTAASMAQKTEESRVYFTKTPTGSSAGSLAESENANLYGSGNIIP
LACDMEKRCCQTAAAPQEELSSECRVGDIRSFSACATISETARELCKAVSVSLGLTMESGDTSEAHVSLP
PCAANDQMSAEYLFGVEAVPANCPGAPASLSDYRCPERDERPPHDHKQQVKMFKSSEPAAAFHHLTSTAT
SVNAQNFTLCLADDITPKEIDHLDLACAASCPYAPAAPEPLAQFGPAAAQRPCGAYNSVEEAGDVVDAVE
NRSTGFQAERYGVKVKSEGSASAGASWVSRPNYSFGDKYNSQFWGSRQCMNVQSSGANPPFICNPYDGTV
MRREQWYPGGMLRTPYPNSNYMKGEVGEWLDVTYNDTRFESSREHMFPMEFFFPPQRTCLICSDEASGCH
YGALTCGSCKVFFKRAAEGKQKYLCASKNDCTIDKLRRKNCPSCRLKKCFEAGMTLGARKLKKIGQQKSP
EEEHGAQDPVDPHVSPKSGVQLNSHLVFLNILESIEPEVVNAGHDYGQPDSAATLLTSLNELGERQLVKV
VKWAKGLPGFRNLHVDDQMTIIQQSWMGVMVCALGWRSYKNAGGRMLYFAPDLVFNEHRMHVSTMYEHCI
RMRHLSQEFEMLQITQEEFLCMKALLLFSIIPVEGLKSQKYFDELRLTYINELDRLINFQTTTNTSQRFY
QLTRLMDSLQMTVKKLHQFTFDLFVQAQSLHTKVSFPEMIGEIISVHVPKILAGLAKPILFHK

>O93497_PAGMA
MSQTSGQLSCTKIWSRGEKVKTGDAVSAPSMAQNTEESPLRVSRNSTGNGAGRMREADNADPNTYESGHM
IPLVCDMEKHCCQTAAAPQEELFNADCRVGDSRSFSACATISETARELCKAVSVSLGLAMESNDPSDMDA
ALSQCAANDQLRGEYLFGVGAAPLSCPGAQAAVSEYKCPEERPLHGHKQQQQLMDMFKSSETGAHLQHLT
STRTPVDEHNFTLCKAEDLTPEETAHQDSVRAAACPYAQSALPGNMAHFGSPAPERPWQLYKPPDEAGDF
GEVMESRFVTSGYQPEQYSVKIKCEDTESAGALWGGNYTFNDRYNSQCWGPRQCMNAHSTGANSALCHPY
ERSVARPEHWYPGGMLRSPYPNSSYVKSEVGEWLDVPYSDPRFDSSSEHMFPMEFFFPAQRMCLICSDEA
SGCHYGALTCGSCKVFFKRAAEGKQKYLCASKNDCTIDKLRRKNCPSCRLRKCFEAGMTLGARKLKKIGQ

HKNSDEDHPLQEPAEVMPNISPKSGLSFNSQVVFLNVLESIEPEVVNAGHDYGQPDSAATLLTSLNELGE
RQLVKVVKWAKGLPGFRNLHVDDQMTVIQHSWMGVMVFGLGWRSYKNVNGRMLYFAPDLVFNEHRMHIST
MYEHCIRMRHLSQEFLLLQITQEEFLCMKALLLFSIIPVEGLKSQKYFDELRLTYINELDRLINYRMNTN
CSQRFYQLTRLLDSLQMTVKKLHQFTFDLFVQAQSLPTKVSFPEMIGEIISVHVPKILAGLAKPILFHE

>ANDR_RANCA
MEVHIGLGGVYKQPPGKMIRGAFENLFLSVREALQGERRSAASLDTSSPISACVHPHPTWNEPSTWTEVR
GTPWREPQGAQPDPPPCSPRSQAPQFTLSSCTTELKEILGEQGGMPEEGNSESASKEGYPESISDSAKEI
CKAVSVSLGLSMEALEHLSAAGEWQRGDCMFAGPPHHTMGAQTCQVAEEDKSDTSFSQYREGAFRRAGQS
TYSAGKAPEDGSSLPTEDKEQPCTDMALSEPGSLRSRGMEVMPSLTLYKPTAFMEDASAYPGRDYYSFQM
ALAPHGRIKVESPIEFAGSAWGGPSRYSEFPGFSHCGPSANWHSLFEEGQATASYTDSSLYSYPRSHVPA
GPDGEFSAEAWYPATAMLGRVHMAVPMRPRMTHGWTATLGIRRRLGWTGVESTFYPIDYYFPPQKPCLSC
EDEASGCHYEALTCGSCKVFFKRAAEGNQKYLCASRNDCTIDKFRRKNCPSCRLRKCYEAGMTLGARKLK
KLGNLKAQEELEGSPGQSEGREMPPNMSIPQLEGYSCQPIFLNVLEAIEPMVVCSGHDNNQPDSFALLLS
SLNELGERQLVHVVKWAKALPGFRNLHVNDQMTVIQYSWMGLMIFAMGWRSFKNVNSRMLYFAPDLVFNE
YRMHKSRMYSQCVRMRHLSQEFGWLQVTPEEFLCDEGPSALSIIPVEGLKDQKCFDELRMNYIKELDRVI
SCKRNNPASSSPRFFNLPKLLGSVQPIDVNLVQFTFGLFGKAQMVSVDFPEMMSEIISVQVPKILSGRVK
PLYFHSS

>Q66VR6_MICUN
MSQTNRELSCNTIWPEGEKVKTSDAVRAPSMAQNTEESRGYFTKNSTGNGAGKADSITYRSGHIHPPVCD
MEKQCCQTAAAPQEELLNAECRVGDSRSFSACATISETARELCKAVSVSLGLTMESNDTSDMDPALTQCA
SNGHTRGGDYLFGVGVVPPNCPGAQGAVTDRDDRPMRGQKQLVEMFKSSETVAAAASARLQHPGSTRTSA
DEQNFTLCKVDDITSEEIDHLDTARAASCHYAQSAPSNLAHFSHAERPCRVYKPPDEERDFGETMENKFG
GYQPEQYGVKVKSEDESESLWGTNYTFNDKYNSQLWGTRQCMNAHNAGANTTFICTPYERSMVRPEQWYP
GGMLRPTYPNSNYVKTEVGEWLDVAYNDTRFEAGREHMFPMEFFFPPQRMCLICSDEASGCHYGALTCGS
CKVFFKRAAEGKQKYLCASKNDCTIDKLRRKNCPSCRLRKCFEAGMTLRARKLKKIGQQKNPEEDHSVQD
PSEVMQNISPKSGLNFNSQTVFLNILESIEPEVVNAGHDYGQPDSAATLLTSLNELGERQLVKVVKWAKG
LPGFRNLHVDDQMTVIQHSWMGVMVFALGWRSYKNVNGRMLYFAPDLVFNEHRMHISTMYEHCIRMRHLS
QEFQLLQITQEEFLCMKALLLFSIIPVEGLKSQKYFDELRLTYINELDRLVNYRMTTNCSQRFYQLTRLL
DSLQMTVKKLHQFTFDLFVQAQSLPTKVSFPEMIGEIISVHVPKILAGLAKPILFHE

>Q60I32_GAMAF
MSQTSRQLSCSSVWSGVKKIKAGDAVRALGMAQKTEENPDGFTKSCAGNARFDESDNNARSCGSGCVNPP
ARDMEARCCQTAAAPQEELLNADTRSFSACATISETARELCRAVSVSLGLTMESSDMSDVDAALPPCAAN
DQISGEYFFGVDAAAVSCPDAQTQITYRCPDREERPVHGQKPAVKMYKSSETPAHFHHLASSRTSVNAQN
FTPCEAEDTDHLNAARTVSCPYAPDHLVQYAHTSTAAAANERPCRAAYNPQEGVRDFVEAPESDSGGYQP
DQYSVKIKSEGNDGWGSFWGGGYTFNKRYNTQFWGSRQCMNAHESGPNAAFICNPYEGSVVRPEQWYPGG
MLRTPYPNSGDMKSQVGEWLDVVYNDTRFEAGREHMFPMEFFFPPQRTCLICSDEASGCHYGALTCGSCK
VFFKRAAEGKQKYLCASKNDCTIDKLRRKNCPSCRLKKCFEAGMTLGARKLKKIGQQKNPEEEHPGQEAP
EVPHNMSPKSGPSLNSQMVFLNILESIEPEVVNAGHDCGQPDSAAGLLTSLNELGERQLVKVVKWAKGLP
GFRNLHVDDQMTVIQQSWMGVMVFALVWRSYKNVNGRMLYFAPDLVFNEHRMQISTMYEHCMRMRHLSQE
FVLLQITQEEFLCMKALLLFSIIPVEGLKSQKYFDELRLTYIKELDRLINYQMTTNCPQRFYQLTRLLDS
LQMTVKKLHQFTFDLFVQAQSLHTKVSFPEMIGEIISVHVPKILAGLAKPILFHK

>Q9PWG5_ANGJA
MSQTNGQIFSKGQFHGTSRNLDTELQEADSANFPMQPEAEARRIHFTKSSDSKGDSSILEPDNAQENAYG
PFLSDLHDCCTDQKSSLPTAPRNRREINPHSDACVSNSCSESACTTISETARELCKAVSVSLGLNMDSNE
MNEPGHNLASSLGSDLRKNFMFEVPFLGSSGTQESVPRAEYKCARLHDGQLLQNDELSGMFKTTHVQGLT
NEVAPSHLSSSYSNWDTQETRLNAETETSASKEQASCMTMDAARSGFCQFDQLLPTTLAQYSQIDPLSMG
RSNFRSQFFYKTLALANEAVEHAEGRYVDSSIQYSPKIKTENLQNQSGGSWDHQYRYNENWHSQYGPSRH
GLHPYCTETNNQFVVEPFDYQRGGGLLPRDRSASESWFPGGMLSKMPSTNLPCLKTEVADWLEVPCSDRM
LLEGGREHVFPMEFFFPPQRTCLICADEASGCHYGALTCGSCKVFFKRAAEGKQRYLCASRNDCTIDKLR
RKNCPSCRLKKCFEAGMTLGARKLKKIGQLKPPDELPTQAPTDAIQCISPKPGLSFPTQSVFLNILESIE
PEVVNAGHDYGQTDSAATLLTSLNELGERQLFKVVKWAKGLPGFRNMHVDDQMTVIQHAWMGVMVFALGW
RSYKNVNARMLYFAPDLVFNDRRMRVSSMYEHCIRMRHMSQEFVLLQVTHQEFLCMKALLLFSIIPVEGL
KNQKYFDDLRNTYINELDRLINCSRKTNCSQRFIQLTRLMDSLQPIVKKLHQFTFDLFVQAQSLHTKVNF
PEMIAEIISVHVPRILAGMAKPILFHN

>Q6IVJ3_DICLA
MDKGGVKKITYRRDDHLSKLVYTESPEEGGLLKVAPHSAMSIASATSVNLPSSPLMQPGQVPNGLSNSPL
PEELTSVTATVGSLLEDHESRGLTRDQKLQQQLLQTQTSTTFGRQTLRENLPHLEASIADITSMDSLIGG
SDPNFFPMKTEDFSMDEGDQEPIDLDHAFEHIGKDVDVHQKLFSDNALDLLQDFELTGSPSDFYVGDDAF
LSSLADDSLLGDVSSERDIKPAVVESMNGCGAVSVSLNGNNMTSPDQSCSSISTTASLTPTTTLSALVKK
EKDAGFIQLCTSGVIKQEKSSAGQSYCQMSGTSSTDMPNSNPISICGVSTSGGQGYRFGVNPTSNEAQQH
KDQKLVSSIYLPVTTISGPWNRSQGVGDNSAMHGASEAFSSSYPISFASSTSRQEGVIAPSSAQTKSGTH
KICLVCSDEASGCHYGVFTCGSCKVFFKRAVKGQHNYLCAGRNDCIIDKIRRKNCPACRFRKCLMAGMNL
EARKTKKLNRLKGAQPSNPPEMTTPPPIEARSLVPKCMPQLVPTMLSLLKAIEPDTIYAGYDSTLPDTST
RLMTTLNRLGGRQVISAVKWAKSLPGFRNLHLDDQMTLLQCSWLFLMSFGLGWRSYQQCNGSMLCFAPDL
VINEERMKLPYMADQCEQMLKISSEFVRLQVSHDEYLCMKVLLLLSTVPKDGLKSQAVFEDIRMSYIKEL
GKAIVKREENSSQNWQRFYHIPLLDYWGRSGGLNIINIRVGAIFSTSHLKSYTKDWYSITVSLHSLLKPL
QGTKCSYRT

>Q8NG44_HUMAN
MTELKAKGPRAPHVAGGPPSPEVGSPLLCRPAAGPFPGSQTSDTLPEVSAIPISLDGLLFPRPCQGQDPS
DEKTQDQQSLSDVEGAYSRAEATRGAGGSSSSPPEKDSGLLDSVLDTLLAPSGPGQSQPSPPACEVTSSW
CLFGPELPEDPPAAPATQRVLSPLMSRSGCKVGDSSGTAAAHKVLPRGLSPARQLLLPASESPHWSGAPV
KPSPQAAAVEVEEEDGSESEESAGPLLKGKPRALGGAAAGGGAAAVPPGAAAGGVALVPKEDSRFSAPRV
ALVEQDAPMAPGRSPLATTVMDFIHVPILPLNHALLAARTRQLLEDESYDGGAGAASAFAPPRSSPCASS
TPVAVGDFPDCAYPPDAEPKDDAYPLYSDFQPPALKIKEEEEGAEASARSPRSYLVAGANPAAFPDFPLG
PPPPLPPRATPSRPGEAAVTAAPASASVSSASSSGSTLECILYKAEGAPPQQGPFAPPPCKAPGASGCLL
PRDGLPSTSASAAAAGAAPALYPALGLNGLPQLGYQAAVLKEGLPQVYPPYLNYLRPDSEASQSPQYSFE
SLPQKICLICGDEASGCHYGVLTCGSCKVFFKRAMEGRKFKKFNKVRVVRALDAVALPQPVGVPNESQAL
SQRFTFSPGQDIQLIPPLINLLMSIEPDVIYAGHDNTKPDTSSSLLTSLNQLGERQLLSVVKWSKSLPGF
RNLHIDDQITLIQYSWMSLMVFGLGWRSYKHVSGQMLYFAPDLILNESHRSLSSFKLAKKSSSV

>Q801Z1_GASAC
MENPCCQTAAAPQEELLNADCRVGDSRSFSACATISETARELCKAVSVSLGLTMEPNDASDLDVAFPPCA

ASEQTRGEYLFMNVNCPGGAYKYPDRDDRPLHGQKQLVEMFKSSETAARLQHVGTPTRASAHERNFAPCE
ADEVTSEEIEHLDAAHASSCEYAPAAPGNFAHFGRSAAPRACRIYEHPDSAADFGGATENKFGGYQPAQY
GVKVKCEDGESAGAPWAGVYTPNEKYDAQCWGSRQCMNAHGTDTNPAFACNPHERSVVRPEQWYPGGMLR
PSYPNSNYVKTEVGEWLDVTYNDTRFEAGREHMFPMEFFFPPQRMCLICSDEASGCHYGALTCGSCKVFF
KRAAEGKQKYLCASKNDCTIDKLRRKNCPSCRLKKCFEAGMTLGARKLKKIGQQKIPEEEPPLKDPLEVI
QNFSPKSGLNFNTQMVFLNILESIEPEVVNAGHDYGQPDSAASLLTSLNELGERQLVKVVKWAKGLPGFR
NLHVDDQMTVIQHSWMGVMVFALGWRSYKNVNGRMLYFAPDLVFNEHRMQMSTMYEHCIRMRHLSQEFLL
LQISQEEFLCMKALLLFSILPVEGLKSQKYFDELRLTYINELDRLISYRMAANCPQRFYQLTRLLDSLQM
TVKKLHQFTFDLFVQAQSLPTKVSFPEMIGEIISVHVPKMLAGLAKPILFHE

>Q8NG45_HUMAN
MTELKAKGPRAPHVAGGPPSPEVGSPLLCRPAAGPFPGSQTSDTLPEVSAIPISLDGLLFPRPCQGQDPS
DEKTQDQQSLSDVEGAYSRAEATRGAGGSSSSPPEKDSGLLDSVLDTLLAPSGPGQSQPSPPACEVTSSW
CLFGPELPEDPPAAPATQRVLSPLMSRSGCKVGDSSGTAAAHKVLPRGLSPARQLLLPASESPHWSGAPV
KPSPQAAAVEVEEEDGSESEESAGPLLKGKPRALGGAAAGGGAAAVPPGAAAGGVALVPKEDSRFSAPRV
ALVEQDAPMAPGRSPLATTVMDFIHVPILPLNHALLAARTRQLLEDESYDGGAGAASAFAPPRSSPCASS
TPVAVGDFPDCAYPPDAEPKDDAYPLYSDFQPPALKIKEEEEGAEASARSPRSYLVAGANPAAFPDFPLG
PPPPLPPRATPSRPGEAAVTAAPASASVSSASSSGSTLECILYKAEGAPPQQGPFAPPPCKAPGASGCLL
PRDGLPSTSASAAAAGAAPALYPALGLNGLPQLGYQAAVLKEGLPQVYPPYLNYLRPDSEASQSPQYSFE
SLPQKICLICGDEASGCHYGVLTCGSCKVFFKRAMEGQHNYLCAGRNDCIVDKIRRKNCPACRLRKCCQA
GMVLGGFRNLHIDDQITLIQYSWMSLMVFGLGWRSYKHVSGQMLYFAPDLILNDSFGRATKSNPV

>Q8NG43_HUMAN
MTELKAKGPRAPHVAGGPPSPEVGSPLLCRPAAGPFPGSQTSDTLPEVSAIPISLDGLLFPRPCQGQDPS
DEKTQDQQSLSDVEGAYSRAEATRGAGGSSSSPPEKDSGLLDSVLDTLLAPSGPGQSQPSPPACEVTSSW
CLFGPELPEDPPAAPATQRVLSPLMSRSGCKVGDSSGTAAAHKVLPRGLSPARQLLLPASESPHWSGAPV
KPSPQAAAVEVEEEDGSESEESAGPLLKGKPRALGGAAAGGGAAAVPPGAAAGGVALVPKEDSRFSAPRV
ALVEQDAPMAPGRSPLATTVMDFIHVPILPLNHALLAARTRQLLEDESYDGGAGAASAFAPPRSSPCASS
TPVAVGDFPDCAYPPDAEPKDDAYPLYSDFQPPALKIKEEEEGAEASARSPRSYLVAGANPAAFPDFPLG
PPPPLPPRATPSRPGEAAVTAAPASASVSSASSSGSTLECILYKAEGAPPQQGPFAPPPCKAPGASGCLL
PRDGLPSTSASAAAAGAAPALYPALGLNGLPQLGYQAAVLKEGLPQVYPPYLNYLRPDSEASQSPQYSFE
SLPQKICLICGDEASGCHYGVLTCGSCKVFFKRAMEGQHNYLCAGRNDCIVDKIRRKNCPACRLRKCCQA
GMVLGGFRNLHIDDQITLIQYSWMSLMVFGLGWRSYKHVSGQMLYFAPDLILNEQSIVTS

>ESR1_PIG
MTMTLHTKASGMALLHQIQANELEPLNRPQLKIPLERPLGEVYVDSSKPAVYNYPEGAAYDFNAAAAASA
PVYGQSGLAYGPGSEAAAFGANGLGGFQPLNSVSPSPLVLLHPPPQLSPFLHPHGQQVPYYLENEPSGYA
VREAGPPAFYRPNSDNRRQGGRERLASTSDKGSMAMESAKETRYCAVCNDYASGYHYGVWSCEGCKAFFK
RSIQGHNDYMCPATNQCTIDKNRRKSCQACRLRKCYEVGMMKGGIRKDRRGGRMLKHKRQRDDGEGRNEA
VPPGDMRSANLWPSPLLIKHTKKNSPVLSLTADQMISALLEAEPPIIYSEYDPTRPLSEASMMGLLTNLA
DRELVHMINWAKRVPGFLDLSLHDQVHLLECAWLEILMIGLVWRSMEHPGKLLFAPNLLLDRNQGKCVEG
MVEIFDMLLATSSRFRMMNLQGEEFVCLKSIILLNSGVYTFLSSTLKSLEEKDHIHRVLDKITDTLIHLM
AKAGLTLQQQHRRLAQLLLILSHFRHMSNKGMEHLYNMKCKNVVPLYDLLLEMLDAHRLHAPTNLGGPPP
EDMSQSQLATSGSTPSHSLQMYYITGEAENFPTTI

>Q9DDZ4_MICSA

MCKRQSPAQSKQPCGTVLRPRIGPAFTELETLSPQHPSPPLRAPLSDMYPEESRGSGGGATVDFLEGTYD
YVAPTPVPTPLYSHSGYYSAPLDAQGPPSDGSLQSLGSGPTSPLVFVPSSPRLSPFMHPPSHHYLETTST
PVYRSSVLSSQQPVPREDQCATSDESYCVGESGAGAGGFEMAKEMRFCAVCSDYASGYHYGVWSCEGCKA
FFKRSIQGHNDYMCPATNQCTIDRNRRKSCQACRLRKCYEVGMMKGGVRKDRGRVLRRDKRRAGTNDRDK
ASKDLEYRTVPPQDRRKHSSSSAGGGGGKSSVTGMSPDQVLLLLQGAEPPMLCSRQKLSRPYTEVTIMTL
LTSMADKELVHMITWAKKLPGFLQLSLHDQVQLLESSWLEVLMIGLIWRSIHCPGKLIFAQDLILDRNEG
DCVEGFVEIFDMLLATASRFRMLKLKPEEFVCLKAIILLNSGAFSFCTGTMEPLHNSVEVHNMLDTITDA
LIHHISQSGCSAQQQSRRQAQLLLLLSHIRHMSNKGMEHLYSMKCKNKVPLYDLLLEMLDAHRIHRPDRP
AQFWSQADGEPPFITVNNCNSSSNGGVSSSVGSSSGPRVSHESPSRGPTGPGVLQYGGSRSDCTHIL

>ESR1_BOVIN

MTMTLHTKASGMALLHQIQANELEPLNRPQLKIPLERPLGEVYMDSSKPAVYNYPEGAAYDFNAAAPASA
PVYGQSGLPYGPGSEAAAFGANGLGAFPPLNSVSPSPLVLLHPPPQPLSPFLHPHGQQVPYYLENESSGY
AVREAGPPAYYRPNSDNRRQGGRERLASTSDKGSMAMESAKETRYCAVCNDYASGYHYGVWSCEGCKAFF
KRSIQGHNDYMCPATNQCTIDKNRRKSCQACRLRKCYEVGMMKGGIRKDRRGGRMLKHKRQRDDGEGRNE
AVPSGDMRAANLWPSPIMIKHTKKNSPVLSLTADQMISALLEAEPPIIYSEYDPTRPFSEASMMGLLTNL
ADRELVHMINWAKRVPGFVDLALHDQVHLLECAWLEILMIGLVWRSMEHPGKLLFAPNLLLDRNQGKCVE
GMVEIFDMLLATSSRFRMMNLQGEEFVCLKSIILLNSGVYTFLSSTLRSLEEKDHIHRVLDKITDTLIHL
MAKAGLTLQQQHRRLAQLLLILSHFRHMSNKGMEHLYSMKCKNVVPLYDLLLEMLDAHRLHAPANFGSAP
PEDVNQSQLAPTGCTSSHSLQTYYITGEAENFPSTV

>ESR1_HUMAN

MTMTLHTKASGMALLHQIQGNELEPLNRPQLKIPLERPLGEVYLDSSKPAVYNYPEGAAYEFNAAAAANA
QVYGQTGLPYGPGSEAAAFGSNGLGGFPPLNSVSPSPLMLLHPPPQLSPFLQPHGQQVPYYLENEPSGYT
VREAGPPAFYRPNSDNRRQGGRERLASTNDKGSMAMESAKETRYCAVCNDYASGYHYGVWSCEGCKAFFK
RSIQGHNDYMCPATNQCTIDKNRRKSCQACRLRKCYEVGMMKGGIRKDRRGGRMLKHKRQRDDGEGRGEV
GSAGDMRAANLWPSPLMIKRSKKNSLALSLTADQMVSALLDAEPPILYSEYDPTRPFSEASMMGLLTNLA
DRELVHMINWAKRVPGFVDLTLHDQVHLLECAWLEILMIGLVWRSMEHPGKLLFAPNLLLDRNQGKCVEG
MVEIFDMLLATSSRFRMMNLQGEEFVCLKSIILLNSGVYTFLSSTLKSLEEKDHIHRVLDKITDTLIHLM
AKAGLTLQQQHQRLAQLLLILSHIRHMSNKGMEHLYSMKCKNVVPLYDLLLEMLDAHRLHAPTSRGGASV
EETDQSHLATAGSTSSHSLQKYYITGEAEGFPATV

>ESR1_MOUSE

MTMTLHTKASGMALLHQIQGNELEPLNRPQLKMPMERALGEVYVDNSKPTVFNYPEGAAYEFNAAAAAAA
AASAPVYGQSGIAYGPGSEAAAFSANSLGAFPQLNSVSPSPLMLLHPPPQLSPFLHPHGQQVPYYLENEP
SAYAVRDTGPPAFYRSNSDNRRQNGRERLSSSNEKGNMIMESAKETRYCAVCNDYASGYHYGVWSCEGCK
AFFKRSIQGHNDYMCPATNQCTIDKNRRKSCQACRLRKCYEVGMMKGGIRKDRRGGRMLKHKRQRDDLEG
RNEMGASGDMRAANLWPSPLVIKHTKKNSPALSLTADQMVSALLDAEPPMIYSEYDPSRPFSEASMMGLL
TNLADRELVHMINWAKRVPGFGDLNLHDQVHLLECAWLEILMIGLVWRSMEHPGKLLFAPNLLLDRNQGK
CVEGMVEIFDMLLATSSRFRMMNLQGEEFVCLKSIILLNSGVYTFLSSTLKSLEEKDHIHRVLDKITDTL
IHLMAKAGLTLQQQHRRLAQLLLILSHIRHMSNKGMEHLYNMKCKNVVPLYDLLLEMLDAHRLHAPASRM
GVPPEEPSQTQLATTSSTSAHSLQTYYIPPEAEGFPNTI

```
>Q7SZI0_FUNHE
MYKGQNPVQSKEAFGPALRPRLSPASSELETLSPPRLPPSPRASLGDMYPEESRGSGGVAAVDFLEGTYD
YATPTPAPTPLYSHSTTGYYSAPLDAQGPPSDGSLHSLGSGPTSPLVFVPTSPRLSLFMHAPSQHYLETA
STPVYRSSHQPASREDQCDTRDEACSVGELGAGAGAGAAAGGFEMAKETRFCAVCSDYASGYHYGVWSCE
GCKAFFKRSIQGHNDYMCPATNQCTIDRNRRKSCQACRLRKCYEVGMMKGGVRKERGRVLRRDKRRTAIS
DREKAVKGLEPKTSPHQDKRKRGSALGGDRSSVASLPSEQVLLLFQGAEPPILCSRQKLSRPYTEVTMMT
LLTSMADKELVHMIAWAKKLPGFLQLALHDQVLLLESSWLEVLMIGLIWRSIHCPGKLIFAQDLILDRNE
GDCVEGMTEIFDMLLATASRFRMLKLKPEEFVCLKAIILLNSGAFSFCTGTMEPLHDSVAVQNMLDTITD
ALIHHISQSGFSVQQQARRQAQLLLLLSHIRHMSNKGMEHLYSMKCKNKVPLYDLLLEMLDAHRHHPVKP
SQDGKSPPSTSSFGAGCEGGSSSAGSSSGPRGSGDNLMRIPSAPGVLQYGGSRSDCAQVL

>ESR1_HORSE
MTMTLHTKASGMALLHQIQGNELETLNLPQFKIPLERPLGEVYVESSKPPVYDYPEGAAYDFNAAAAASA
SVYGQSGLAYGPGSEAAAFGANGLGGFPPLNSVSPSQLMLLHPPPQLSPYLHPPGQQVPYYLENEPSGYS
VCEAGPQAFYRPNADNRRQGGRERLASSGDKGSMAMESAKETRYCAVCNDYASGYHYGVWSCEGCKAFFK
RSIQGHNDYMCPATNQCTIDKNRRKSCQACRLRKCYEVGMMKGGIRKDRRGGRMLKHKRQRDDGEGRNEA
GPSGDRRPANFWPSPLLIKHTKKISPVLSLTAEQMISALLDAEPPVLYSEYDATRPFNEASMMGLLTNLA
DRELVHMINWAKRVPGFVDLSLHDQVHLLECAWLEILMIGLVWRSMEHPGKLLFAPNLLLDRNQGKCVEG
MVEIFDMLLATSSRLRMMNLQGEEFVCLKSIILLNSGVYTFLSSTLKSLEEKDHIHRVLDKMTDTLIHLM
AKAGLTLQQHRRLAQLLLILSHIRHMSNKGMEHLYSMKCKNVVPLYDLLLEMLDAHRLHAPANHGGAPME
ETNQSQLATTGSTSPHSMQTYYITGEAEGFPNTI

>ESR2_ONCMY
MHQQSPVDDVTALNSSALTMSEYPEGESPLQLQDVDSSRVGGHILSPIFNSSSPSLPVESHPVCIQSPYT
DLGHDFTTLPFYSPALLGYGTSPLSECSSVRQSLSPTLFWPPHSQVSSLALHQQHTRLQQNHPTGGTWTE
LTPHDHSEEEYRKPLVKRVADAEETSTSLRGKADMHYCAVCSDYASGYHYGVWSCEGCKAFFKRSIQGHN
DYICPATNQCTIDKNRRKSCQACRLRKCYEVGMMKCGLRRDRGSYQQRGAQQKRLARFSGRMRTSGPRSQ
EMKSVPCPLSGNEVVNMALTPEELIARIMDAEPPEIYLMKDMKKPFTEANVMMSLTNLADKELVHMISWA
KKVPGFVELSLFDQVHLLECCWLEVLMLGLMWRSVNHPGKLIFSPDLSLSRDEGSCVQGFVEIFDMLLAA
TSRFRELKLQREEYVCLKAMILLNSNMCLSSSEGGEELQRRSKLLCLLDSVTDALVWAISKTGLSFQQRS
TRLAHLLMLLSHIRHLSNKGMDHLHCMKMKKMVPLYDLLLEMLDAHIMHGSRLSHSGPAPKESTGVQEAT
LSVLKNDL

>ESR1_MESAU
MTMTLHTKASGMALLHQIQGNELEPLSRPQLKMPLERALSEVYVDSSKPAMFNYPEGAAYEFNAATAPAP
VYGQTGIAYGSGSEATAFGSNSLGLFPQLNSVSPSPLMLLHPPPPQLSPFLHPHGQQVPYYLENEPSAYA
VRDSGPPAFYRSNSDNRRQSGRERLSSSSEKGSMAMESVKETRYCAVCNDYASGYHYGVWSCEGCKAFFK
RSIQGHNDYMCPATNQCTIDKNRRKSCQACRLRKCYEVGMMKGGIRKDRRGGRMLKHKRQRDDLEGRNDM
GPSGDMRATNLWPSPLVIKHTKKNSPALSLTADQMVSALLDAEPPLIYSEYDPSRPFSEASMMGLLTNLA
DRELVHMINWAKRVPGFGDLNLHDQVHLLECAWLEILMIGLIWRSMEHPGKLLFAPNLLLDRNQGKCVEG
MVEIFDMLLATSARFRMMDLQGEEFVCLKSIILLNSGVYTFLSSTLKSLEEKDHIHRVLDKITDTLIHLM
AKAGLTLQQQHRRLAQLLLILSHIRHMSNKGMEHLYNMKCKNVVPFYDLLLEMLDAHRLHTPVSRMGVSP
EEPSQSQLTTTNSTSSHSLQTYYIPSEAESFPNTI
```

>ESR1_ORYLA
MSKRQSSVQIRQLFGPALRSRISPASSELETLSPPRLSPRDPLGDMYPEESRGSGGVAAVDFLEGTYDYA
APNPATTPLYSQSSTGYYSAPLETNGPPSEGSLQSLGSGPTSPLVFVPSSPRLSPFMHPPSHHYLETTST
PVYRSSHQGASREDQCGSREDTCSLGELGAGAGAGGFEMAKDTRFCAVCSDYASGYHYGVWSCEGCKAFF
KRSIQGHNDYMCPATNQCTIDRNRRKSCQACRLRKCYEVGMMKGGVRKDRIRILRRDKRRTGVGDGDKVV
KGQEHKTVHYDGRKRSSTGGGGGGGGGRLSVTSIPPEQVLLLLQGAEPPILCSRQKLSRPYTEVTMMTLL
TSMADKELVHMIAWAKKLPGFLQLSLHDQVLLLESSWLEVLMIGLIWRSIHCPGKLIFAQDLILDRNEGD
CVEGMTEIFDMLLATASRFRVLKLKPEEFVCLKAIILLNSGAFSFCTGTMEPLHNSAAVQSMLDTITDAL
IHYISQSGYLAQEQARRQAQLLLLLSHIRHMSNKGMEHLYSMKCKNKVPLYDLLLEMLDAHRLHHPVRAP
QSLSQVDRDPPSTSSGGGGIAPGSISASRGRIESPSRGPFAPSVLQYGGSRPDCTPALQD

>Q765N7_ALLMI
MTMTLHTKTSGVTLLHQIQGTELETLSRPQLKIPLDRSLSEMYVESNKTGIFNYPEGTTYDFATAAPVYS
STSLSYAPTSESYGSSSLGGFHSLNNVPPSPVVFLQTAPQLSPFIHHHSQQVPYYLENDQSGFGMREAAP
STFYRPGADSRRQSGRERMSSTSEKTSLSMESTKETRYCAVCNDYASGYHYGVWSCEGCKAFFKRSIQGH
NDYMCPATNQCTIDKNRRKSCQACRLRKCYEVGMMKGGIRKDRRGGRMLKQKRQREEQDARNGETATAEM
RTPTLWTSPLVIKHTKKNSPALSLTAEQMVSALLEAEPPIVYSEYDPNRPFNEASMMTLLTNLADRELVH
MINWAKRVPGFVDLTLHDQVHLLECAWLEILMIGLVWRSVEHPGKLLFAPNLLLDRNQGKCVEGMVEIFD
MLLATAARFRMMNLQGEEFVCLKSIILLNSGVYTFLSSTLKSLEEKDYIHRVLDKITDTLIHLMAKSGLS
LQQQHRRLAQLLLILSHIRHMSNKGMEHLYNMKCKNVVPLYDLLLEMLDAHRLHAPAARNAAQVEEETRL
TTASASSHSLQSFYINNREDENLQNTI

>Q8AYH0_COTJA
MTMTLHTKASGVTLLHQIQGTELETLSRPQLKIPLERSLSDMYVESNKTGVFNYPEGATYDFGTTAPVYG
STTLSYAPTSESFGSSSLAGFHSLNNVPPSPVVFLQTAPQLSPFIHHHSQQVPYYLENEQGSFGMRETAP
PAFYRPSSDNRRHSIRERMSSASEKGSLSMESTKETRYCAVCNDYASGYHYGVWSCEGCKAFFKRSIQGH
NDYMCPATNQCTIDKNRRKSCQACRLRKCYEVGMMKGGIRKDRRGGRMMKQKRQREEQESRNGEASSTEL
RAPTLWTSPLVVKHNKKNSPALSLTAEQMVSALLEAEPPIVYSEYDPNRPFNEASMMTLLTNLADRELVH
MINWAKRVPGFVDLTLHDQVHLLECAWLEILMIGLVWRSMEHPGKLLFAPNLLLDRNQGKCVEGMVEIFD
MLLATAARFRMMNLQGEEFVCLKSIILLNSGVYTFLSSTLKSLEERDYIHRVLDKITDTLIHFMAKSGLS
LQQQHRRLAQLLLILSHIRHMSNKGMEHLYNMKCKNVVPLYDLLLEMLDAHRLHAPAARSAAPMEEENRS
QLTTAPASSHSLQSFYINSKEEESMQNTI

>ESR1_CHICK
MTMTLHTKASGVTLLHQIQGTELETLSRPQLKIPLERSLSDMYVESNKTGVFNYPEGATYDFGTTAPVYG
STTLSYAPTSESFGSSSLAGFHSLNNVPPSPVVFLQTAPQLSPFIHHHSQQVPYYLENEQGSFGMREAAP
PAFYRPSSDNRRHSIRERMSSTNEKGSLSMESTKETRYCAVCNDYASGYHYGVWSCEGCKAFFKRSIQGH
NDYMCPATNQCTIDKNRRKSCQACRLRKCYEVGMMKGGIRKDRRGGEMMKQKRQREEQDSRNGEASSTEL
RAPTLWTSPLVVKHNKKNSPALSLTAEQMVSALLEAEPPIVYSEYDPNRPFNEASMMTLLTNLADRELVH
MINWAKRVPGFVDLTLHDQVHLLECAWLEILMIGLVWRSMEHPGKLLFAPNLLLDRNQGKCVEGMVEIFD
MLLATAARFRMMNLQGEEFVCLKSIILLNSGVYTFLSSTLKSLEERDYIHRVLDKITDTLIHLMAKSGLS
LQQQHRRLAQLLLILSHIRHMSNKGMEHLYNMKCKNVVPLYDLLLEMLDAHRLHAPAARSAAPMEEENRN
QLTTAPASSHSLQSFYINSKEEESMQNTI

>Q8UWB0_CAICR
MTMTLHTKTSGVTLLHQIQGTELETLSRPQLKIPLDRSLSEMYVENNKTGIFNYPEGTTYDFATAAPVYS
STSLSYAPTSESYGSSSLGGFHSLNNVPPSPVVFLQTAPQLSPFVHHHSQQVPYYLENDQSGFGMREAAS
STFYRPSADSRHQSGRERMSSTSEKASLSMESTKETRYCAVCNDYASGYHYGVWSCEGCKAFFKRSIQGH
NDYMCPATNQCTIDKNRRKSCQACRLRKCYEVGMMKGGIRKDRRGGRMLKQKRQREEQDARNGETATAEM
RTPTLWTSPLVIKHTKKNSPALSLTAEQMVSALLEAEPPIVYSEYDPNRPFNEASMMTLLTNLADRELVH
MINWAKRVPGFVDLTLHDQVHLLECAWLEILMIGLVWRSMEHPGKLLFAPNLLLDRNQGKCVEGMVEIFD
MLLATAARFRMMNLQGEEFVCLKSIILLNSGVYTFLSSTLKSLEEKDYIHRVLDKITDTLIHLMAKSGLS
LQQQHRRLAQLLLILSHIRHMSNKGMEHLYNMKCKNVVPLYDLLLEMLDAHRLHAPAARNAAQVEEETRL
TTASASSHSLQSFYINNREDENLQNTI

>Q6W5G7_XENLA
MTMPLPNKTTGVTFLHQIQSSELETLTRPPLKISLERPLGEMYVENNRTGIFNYPEGTTYDFAAAAAPVY
SSASLSYAASSETFGSSSLTGLHTLNNVPPSPVVFLQTPQLSPFIHHHGQQVPYYLESEQGTFAVREAAP
PTFYRSSSDNRRQSGRERMSSANDKGPPSMESTKETRYCAVCSDYASGYHYGVWSCEGCKAFFKRSIQGH
NDYMCPATNQCTIDKNRRKSCQACRLRKCYEVGMMKGGIRKDRRGGRMLKHKRQKEEQEQKNDVDPSEIR
TASIWVNPSVKSMKLSPVLSLTAEQLISALMEAEPPIVYSEHDSTKPLSEASMMTLLTNLADKELVHMIN
WAKRVPGFVDLTLHDQVHLLECAWLEILMVGLIWRSVEHPGKLSFAPNLLLDRNQGRCVEGLVEIFDMLV
TTATRFRMMRLRGEEFICLKSIILLNSGVYTFLSSTLESLEDTDLIHIILDKIIDTLVHFMAKSGLSLQQ
QQRRLAQLLLILSHIRHMSNKGMEHLYSMKCKNVVPLYDLLLEMLDAHRIHTPKDKTTTQEEDSRSPPTT
TVNGASPCLQPYYTNTEEVSLQSTV

>ESR1_POEGU
MTLHTKTSGVTLLHQIQGTELETLSRPQLKIPLERSLSDMYVETNKTGVFNYPEGATYDFGTTAPVYSST
TLSYAPTSESFGSSSLAGFHSLNSVPPSPVVFLQTAPHWSPFIHHHSQQVPYYLENDQGSFGMREAAPPA
FYRPNSDNRRHSIRERMSSANEKGSLSMESTKETRYCAVCNDYASGYHYGVWSCEGCKAFFKRSIQGHND
YMCPATNQCTIDKNRRKSCQACRLRKCYEVGMMKGGIRKDRRGGRVMKQKRQREEQDSRNGEASSTELRA
PTLWASPLVVKHNKKNSPALSLTAEQMVSALLEAEPPLVYSEYDPNRPFNEASMMTLLTNLADRELVHMI
NWAKRVPGFVDLTLHDQVHLLECAWLEILMIGLVWRSMEHPGKLLFAPNLLLDRNQGKCVEGMVEIFDML
LATAARFRMMNLQGEEFVCLKSIILLNSGVYTFLSSTLKSLEEKDYIHRVLDKITDTLIHLMAKSGLSLQ
QQHRRLAQLLLILSHIRHMSNKGMEHLYNMKCKNVVPLYDLLLEMLDAHRLHAPAARSAAPMEEENRSQL
TTASASSHSLQSFYINSKEEENMQNTL

>Q90WS8_BRARE
MSEYPEGDSPLLQLQEVDSGRVGGHILSPIFNSSSPSLPVENHPICIPSPYTDLGHDFSTLPFYSPALLG
YSTSPLSDCSSVRQSLSPTLFWPPHSHVSSLTLQQQSRLQQNHATSGTWTEHTPHDHVEEENSKPLVKRV
ADTEETSVSLRGKADMHYCAVCSDYASGYHYGVWSCEGCKAFFKRSIQGHNDYICPATNQCTIDKNRRKS
CQACRLRKCYEVGMMKCGLRRDRSSYQQRGAQQKRLVRFSGRMRMTGPRSQEIKSIPRPLSGNEVVRISL
SPEELISRIMEAEPPEIYLMKDMKKPFTEANVMMSLTNLADKELVHMISWAKKIPGFVELSLFDQVHLLE
CCWLEVLMLGLMWRSVNHPGKLIFSPDLSLSRDESSCVQGLVEIFDMLLAATSRFRELKLQREEYVCLKA
MILLNSNMCLGSSEGGEDLQSRSKLLCLLDSVTDALVWAISKTGLSFQQRSTRLAHLLMLLSHIRHVSNK
GMDHLHCMKMKKMVPLYDLLLEMLDAHIMHSSRLSHSGPRAPAAHKDNKSVQEAFPCSSQHGP

>ESR1_XENLA
MTMPLPNKTTGVTFLHQIQSSELETLTRPPLKISLERPLGEMYVENNRTGIFNYPEGTTYDFAAAAAPVY
SSASLSYAASSETFGSSSLTGLHTLNNVPPSPVVFLAKLPQLSPFIHHHGQQVPYYLESEQGTFAVREAA
PPTFYRSSSDNRRQSGRERMSSANDKGPPSMESTKETRYCAVCSDYASGYHYGVWSCEGCKAFFKRSIQG
HNDYMCPATNQCTIDKNRRKSCQACRLRKCYEVGMMKGGIRKDRRGGRLLKHKRQKEEQEQKNDVDPSEI
RTASIWVNPSVKSMKLSPVLSLTAEQLISALMEAEAPIVYSEHDSTKPLSEASMMTLLTNLADRELVHMI
NWAKRVPGFVDLTLHDQVHLLECAWLEILMVGLIWRSVEHPGKLSFAPNLLLDRNQGRCVEGLVEIFDML
VTTATRFRMMRLRGEEFICLKSIILLNSGVYTFLSSTLESLEDTDLIHIILDKIIDTLVHFMAKSGLSLQ
QQQRRLAQLLLILSHIRHMSNKGMEHLYSMKCKNVVPLYDLLLEMLDAHRIHTPKDKTTTQEEDSRSPPT
TTVNGASPCLQPYYTNTEEVSLQSTV

>ESRB1_CARAU
MTALNSYAFAMSEYAEGDSSLLQLQEVDSSRMGGHVLSPTFNSSSPSLPVESHPICIPSPYTDLGHDFTT
LPFYSPSLLGYGTSPLSDCPSVRQSLSPTLFWPPHSHVSSLALHQQQTRLQPNHPTGGTWAELTPHDHGE
EENCKPLSKRVAVAEETSTSLRGKADMHYCAVCSDYASGYHYGVWSCEGCKAFFKRSIQGHNDYICPATN
QCTIDKNRRKSCQACRLRKCYEVGMMKCGLRRDRSSYQQRGAQQNRLTRFSGRMRTSGPRSQEIKTVQRP
LSGNKVVTMALSPEELIARIMDAEPPEIYLMKDVKKPFTEANVMMSLTNLADKELVHMISWAKKIPGFVE
IGLFDQVHLLECCWLEVLMLGLMWRSVNHPGKLVFSPDLSLSRDEGSCVQGFAEIFDMLLAATSRFRELK
LQREEYACLKAMILLNSNMCLSSAEGGEELQSRSKLLCLLDSVTDALVWAISKTGLSFQQRSTRLAHLLM
LLSHIRHVSNKGMDHLHSMKMKKMVPLYDLLLEMLDAHIMHGSRLSHSGPQADPVPKESNCVQETFTCTS
QHGGTLRP

>Q7ZU32_BRARE
MSEYPEGDSPLLQLQEVDSGRVGGHILSPIFNSSSPSLPVENHPICIPSPYTDLGHDFSTLPFYSPALLG
YSTSPLSDCSSVRQSLSPTLFWPPHSHVSSLTLQQQSRLQQNHATSGTWTEHTPHDHVEEENSKPLVKRV
ADTEETSVSLRGKADMHYCAVCSDYASGYHYGVWSCEGCKAFFKRSIQGHNDYICPATNQCTIDKNRRKS
CQACRLRKCYEVGMMKCGLRRDRSSYQQRGAQQKRLVRFSGRMRMTGPRSQEIKSIPRPLSGNEVVRISL
SPEELISRIMEAEPPEIYLMKDMKKPFTEANVMMSLTNLADKELVHMISWAKKIPGFVELSLFDQVHLLE
CCWLEVLMLGLMWRSVNHPGKLIFSPDLCLSRDESSCVQGLVEIFDMLLAATSRFRELKLQREEYVCLKA
MILLNSNMCLGSSEGGEDLQSRSKLLCLLDSVTDALVWAISKTGLSFQQRSTRLAHLLMLLSHIRHVSNK
GMDHLHCMKMKKMVPLYDLLLEMLDAHIMHSSRLSHSGPRAPAAHKDNKSVQEAFPCSSQHGP

>Q8AV62_BRARE
MSEYPEGDSPLLQLQEVDSGRVGGHILSPIFNSSSPSLPVENHPICIPSPYTDLGHDFSTLPFYSPALLG
YSTSPLSDCSSVRQSLSPTLFWPPHSHVSSLTLQQQSRLQQNHATSGTWTEHTPHDHVEEENSKPLVKPV
AETEETSVSLRGKADMHYCAVCSDYASGYHYGVWSCEGCKAFFKRSIQGHNDYICPATNQCTIDKNRRKS
CQACRLRKCYEVGMMKCGLRRDRSSYQQRGAQQKRLVRFSGRMRMTGPRSQEIKSIPRPLSGNEGARISL
SPEELISRIMEAEPPEIYLMKDMKKPFTEANVMMSLTNLADKELVHMISWAKKIPGFVELSLFDQVHLLE
CCWLEVLMLGLMWRSVNHPGKLIFSPDLCLSRDESSCVQGLVEIFDMLLAATSRFRELKLQREEYVCLKA
MILLNSNMCLGSSEGGEDLQSRSKLLCLLDSVTDALVWAISKTGLSFQQRSTRLAHLLMLLSHIRHVSNK
GMDHLHCMKMKKMAPLYDLLLEMLDAHIMHSSRLSHSGPRAPAAHKDNKSVQEAFPCSSQHGP

>Q60GT9_GAMAF
MAFRSRLPDRDETANSLRMTERDRMETIDAYACPTLELSRAVSVSLGLDPLSSPLGGLNQCPSSAFGDCE

PAAAGGNSSCGAPELGTPGSASSEGSRLARADSSLGVDEFGEVCHGLRQVSCVDQFSSGDMDGAQSVTRG
SVISRFVCKDSSMFMNSIAEVPTTPQVTEVGSLKPYSPYSAEANLYRETQGVWCATERAYGERSEPHRSA
YDGHSFLCKYCGQTSFGPRQECDCVWYRRGERGGKGGAPVSTVQAFGQVESYPDAIPQGQSAFSTIKTEP
SVWVHCTDRGFRHEDFFPGVFLSDRRVCQVCGDDASGCHYGAVTCGSCKVFFKRAAAGKQNHLCASRNDC
TIDKLRRKNCASCRLKRCFMSGMSLKGRRLKGAGQTRGGEEEQQPGAWGHGEREGKHNVVLEPGNAAARA
QGPQLLGIPPTMRSCLSLLTILQSIEPAVVNAGHDPAQPDSPASLLTSLNELGERQLVTVVRWAKAIPGF
RDLHVDDQMSVIQLSWMGVMVFALGWRSYTLTNCSMLYFAPDLVFNDQRMQVSSMYEHCVRMKLLAQRFC
KLEVTEEEFLCMKALVLFSIMPVEGLRSQRCFDELRTSYIKELDRLASHHGETTRTQRLFQLTQLLDYLQ
SVVRKLHQFTYDLFIQAQSLQMRVNFPEMISEIVSVHVPKILSGMVKPILFHNTA

>Q8UWA9_CNEUN
MTMTLHTKTSGVALLHQIQGSELEPLNRPQLKIPLERPISEMYVDSNKTGVFNYPEGATYDFSTAAPVYS
SASLSYASTNESFGSGNLGGLHSLNNVPPSPVVFLQTAPQLSPFIHHHNQQVPYYLENEPSSSAMREAFP
TAFYRPGSENRHHGGRASNSEKGSLSMESTKETRYCAVCNDYASGYHYGVWSCEGCKAFFKRSIQGHNDY
MCPATNQCTIDKNRRKSCQACRLRKCYEVGMMKGGIRKDRRGGRMLKHKRQRDELDGRNAVAVTEARNTT
LWPSPLMIKHSKKNSPALSLTAEQMVSALLDAEPPIVYSEYDPSSPFSEASVMTLLTNLADRELVHMITW
AKRVPGFVDLALHDQVHLLECAWLEILMIGLIWRSLEHPGKLLFAPNLLLDRSQGMCVEGFVEIFDMLLA
TSSRFRMMNIQGEEFVCLKSIILLNSGIYTFLSSTLRSLEEKEHIHRVLDKITDTLTHLMAKSGLSLQQQ
HRRLAQLLLMLSHIRHMSNKGMEHLYNMKCKNVVPLYDLLLEMLDAHRLHERRTPTSEQAMNQLTNASTS
VHSLPPCYVNKREEENEQEAV

>ESR2_STUVU
MSLCTSSHKDFSQLLPLQDIGCNKTEIKNSPAGVISPAPYSCNQSTLTAEHSPVYIPSSYMESRHEYSTM
AFCSPAMVNYNIASNFGDPEAVAARQTSSPGALWSAPGHLSPLSLHCQSSLLYAEQPKSLWCEARPMEPV
LPGSRETLKRKTNGNDCTSPIANNPGSKKDAHFCAVCSDYASGYHYGVWSCEGCKAFFKRSIQGHNDYIC
PATNQCTIDKNRRKSCQACRLRKCYEVGMMKCGSRRERCGYRILRSHRGAEERVHCLGRARRYSEAATRV
KEILLSTVSPEQFVLTLLEAEPPHVLVSRPSKPFTEASMMMSLTKLADKELVHMIGWAKKIPGFIDLSLY
DQVRLLESCWMEVLMIGLMWRSIDHPGKLIFAPDLVLDRDEGKCVEGILEIFDMLLAMTSRFRELKLQHK
EYLCVKAMILLNSSMFPLSAEEPESNRKLHHLLNVVTEALVWVIAKSGIPSQQQTTRLANLLMLLSHVRH
ASNKGMEHLLSMKCKNVVPVYDLLLEMLNAHTLRGQRKPLATHPEFGPLEQMEPGESLRKGEPQ

>ESR2_MOUSE
MEIKNSPSSLTSPASYNCSQSILPLEHGPIYIPSSYVESRHEYSAMTFYSPAVMNYSVPSSTGNLEGGPV
RQTASPNVLWPTSGHLSPLATHCQSSLLYAEPQKSPWCEARSLEHTLPVNRETLKRKLGGSGCASPVTSP
SAKRDAHFCAVCSDYASGYHYGVWSCEGCKAFFKRSIQGHNDYICPATNQCTIDKNRRKSCQACRLRKCY
EVGMVKCGSRRERCGYRIVRRQRSASEQVHCLNKAKRTSGHTPRVKELLLNSLSPEQLVLTLLEAEPPNV
LVSRPSMPFTEASMMMSLTKLADKELVHMIGWAKKIPGFVELSLLDQVRLLESCWMEVLMVGLMWRSIDH
PGKLIFAPDLVLDRDEGKCVEGILEIFGMLLATTARFRELKLQHKEYLCVKAMILLNSSMYPLATASQEA
ESSRKLTHLLNAVTDALVWVISKSGISSQQQSVRLANLLMLLSHVRHISNKGMEHLLSMKCKNVVPVYDL
LLEMLNAHTLRGYKSSISGSECCSTEDSKSKEGSQNLQSQ

>ESR2_RAT
MEIKNSPSSLSSPASYNCSQSILPLEHGPIYIPSSYVDNRHEYSAMTFYSPAVMNYSVPGSTSNLDGGPV
RLSTSPNVLWPTSGHLSPLATHCQSSLLYAEPQKSPWCEARSLEHTLPVNRETLKRKLSGSSCASPVTSP

NAKRDAHFCPVCSDYASGYHYGVWSCEGCKAFFKRSIQGHNDYICPATNQCTIDKNRRKSCQACRLRKCY
EVGMVKCGSRRERCGYRIVRRQRSSSEQVHCLSKAKRNGGHAPRVKELLLSTLSPEQLVLTLLEAEPPNV
LVSRPSMPFTEASMMMSLTKLADKELVHMIGWAKKIPGFVELSLLDQVRLLESCWMEVLMVGLMWRSIDH
PGKLIFAPDLVLDRDEGKCVEGILEIFDMLLATTSRFRELKLQHKEYLCVKAMILLNSSMYPLASANQEA
ESSRKLTHLLNAVTDALVWVIAKSGISSQQQSVRLANLLMLLSHVRHISNKGMEHLLSMKCKNVVPVYDL
LLEMLNAHTLRGYKSSISGSECSSTEDSKNKESSQNLQSQ

>Q8JJB9_CYPCA
MSVYPEGDSPLLQLQEVDSSRVGGHVLSPAFNSSSPSLPVESHPICIPSPYTDLSHDFTTLPFYSPALLG
YGAAPLSDCPSVRQSLSPSLFWPPHNHVSSVVLHQQQTRLQQNHPTGGSWAELTPHDHSEEENCKPLAKR
VADAEETSASLRGKADMHYCAVCSDYASGYHYGVWSCEGCKAFFKRSIQGHNDYICPATNQCTIDKNRRK
SCQACRLRKCYEVGMMKCGLRRDRGSYQQRGARQKRLARFSGRMRTCGPRSQEIKSVPRPLGGNKVVSIA
LSPEELIARIMDAEPPEIYLMNDVKKPFTEANIMMSLTNLADKELVHMISWAKKIPGFVELSLFDQVHLL
ECCWLEVLMLGLMWRSVNHPGKLIFSPDLSLSRDEGSCVQGFVEIFDMLLAATSRFRELKLQREEYACLK
AMILLNSNMCLSSAEGGEELQSRSKLLCLLDSVTDALVWAISKTGLSFQQRSTRLAHLLMLLSHIRHVSN
KGMDHLHCMKMKKMVPLYDLLLEMLDAHIMHSSRLSHSGPRAAPAPKESKGVQEALTRTSQSGGTLAGP

>ESR2_HUMAN
MDIKNSPSSLNSPSSYNCSQSILPLEHGSIYIPSSYVDSHHEYPAMTFYSPAVMNYSIPSNVTNLEGGPG
RQTTSPNVLWPTPGHLSPLVVHRQLSHLYAEPQKSPWCEARSLEHTLPVNRETLKRKVSGNRCASPVTGP
GSKRDAHFCAVCSDYASGYHYGVWSCEGCKAFFKRSIQGHNDYICPATNQCTIDKNRRKSCQACRLRKCY
EVGMVKCGSRRERCGYRLVRRQRSADEQLHCAGKAKRSGGHAPRVRELLLDALSPEQLVLTLLEAEPPHV
LISRPSAPFTEASMMMSLTKLADKELVHMISWAKKIPGFVELSLFDQVRLLESCWMEVLMMGLMWRSIDH
PGKLIFAPDLVLDRDEGKCVEGILEIFDMLLATTSRFRELKLQHKEYLCVKAMILLNSSMYPLVTATQDA
DSSRKLAHLLNAVTDALVWVIAKSGISSQQQSMRLANLLMLLSHVRHASNKGMEHLLNMKCKNVVPVYDL
LLEMLNAHVLRGCKSSITGSECSPAEDSKSKEGSQNPQSQ

>ESR1_ONCMY
MLVRQSHTQISKPLGAPLRSRTTLESHVISPPKLSPQQPTTPNSNMYPEETRGGGGAAAFNYLDGGYDYT
APAQGPAPLYYSTTPQDAHGPPSDGSMQSLGSSPTGPLVFVSSSPQLSPQLSPFLHPPSHHGLPSQSYYL
ETSSTPLYRSSVVTNQLSASEEKLCIASDRQQSYSAAGSGVRVFEMANETRYCAVCSDFASGYHYGVWSC
EGCKAFFKRSIQGHNDYMCPATNQCTMDRNRRKSCQACRLRKCYEVGMVKGGLRKDRGGRVLRKDKRYCG
PAGDREKPYGDLEHRTAPPQDGGRNSSSSLNGGGGWRGPRITMPPEQVLFLLQGAEPPALCSRQKVARPY
TEVTMMTLLTSMADKELVHMIAWAKKVPGFQELSLHDQVQLLESSWLEVLMIGLIWRSIHCPGKLIFAQD
LILDRSEGDCVEGMAEIFDMLLATVSRFRMLKLKPEEFVCLKAIILLNSGAFSFCSNSVESLHNSSAVES
MLDNITDALIHHISHSGASVQQQPRRQAQLLLLLSHIRHMSNKGMEHLYSIKCKNKVPLYDLLLEMLDGH
RLQSPGKVAQAGEQTEGPSTTTTTSTGSSIGPMRGSQDTHIRSPGSGVLQYGSPSSDQMPIP

>Q8UWB8_ORENI
MSFHSFLEETNSLTMNEGDRLDTTTYACPAYELSKAVSVSLGLDSVSSPPNNMNQSSSSAFAECDSGTVA
DSSRGVPELRRAGNMNSDGSMFVLGDSSLREDDFGEVCQGIQQVSCMDLFGSGEMDSAQTVTRGSVISRY
VCRESNVFMNPTPELPAPPQVPGVVPLKPPSSYCASSDLYRDLPPEMWCANERAYSDRSQPQPQRGESGE
HNFLCKYCNCGQAPRGNRQECRCIWYGRGDQGGKGGMRATAQGYGQMSSYPSAIPQGQNTFSTIKSGPSV
WMNCTDRSLRREDFFPGVYLSERRVCQVCGDGASGCHYGAVTCGSCKVFFKRAAAGKQNHLCASRNDCTI

DKLRRKNCASCRLKRCFMSGMSLKGRRLKGAGQARNGEEEQQPASWGQGEKEERAAKKDVVLESGNAGVR
AQGAASQALGAAIPPPLHSCLSLLSILQAIEPAVVNAGHDPAQPDSPMSLLTSLNKLGERQLVTVVRWAK
AIPGFRDLHVDDQMSVIQLSWMGVMVFALGWRSYTLTNSSMLYFAPDLIFNDQRMQASSMYEHCVRMKLL
SQRLCMLKVTQEEFLCMKALVLLSIMPEQGPKSQHCFDKLRTSYIKELDRLASHRGETTRTQRLFQLTQL
LDHLQSVVRKLHQFTYDLFIQAQSMHVSFPEMISEIVSIHVPKILSGMVKPILFHNVA

>Q98SM7_BRARE
MSEYPEGDSPLLQLQEVDSGRVGGHILSPIFNSSSPSLPVENHPICIPSPYTDLGTTSALCPFTVPLCWG
TSTSPLSDCSSVRQSLSPTLFWPPHSHVSSLTLQQQSRLQQNHATSGTWTEHTPHDHVEEENSKPLVKRV
ADTEETSVSLRGKADMHYCAVCSDYASGYHYGVWSCEGCKAFFKRSIQGHNDYICPATNQCTIDKNRRKS
CQACRLRKCYEVGMMKCGLRRDRSSYQQRGAQQKRLVRFSGRMRMTGPRSQEIKSIPRPLSGNEVVRISL
SPEELISRIMEAEPPEIYLMKDMKKPFTEANVMMSLTNLADKELVHMISWAKKIPGFVELSLFDQVHLLE
CCWLEVLMLGLMWRSVNHPGKLIFSPDLCLSRDESSCVQGLVEIFDMLLAATSRFRELKLQREEYVCLKA
MILLNSNMCLGSSEGGEDLQSRSKLLCVLDSVTDALVWAISKTGLSFQQRSTRLAHLLMLLSHIRHVSNK
GMDHQHCMKMKKMVPLYDLLLEMLDAHIMHSSRLSHSGPRAPAAHKDDKSVQEAFPCSSQHGP

>ESR2_BOVIN
MDVKNSPSSLNSPVSYNCGQSILPLEPGPIYLPSSYVESRHEYSAVTFYSPAVMNYSIPNNSEDGPGRQT
TSPNVLWPTPGHLSPLAIHCQPSVLYAEPQKSPWRETRSLEHTLPVNRETLKRKASGSSCASPATSPSSK
RDAHFCAVCSDYASGYHYGVWSCEGCKAFFKRSIQGHNDYICPATNQCTIDKNRRKSCQACRLRKCYEVG
MVKCGSRRERCGYRIVRRQRNSDEQLHCLSKTKRNGGPMTRVKELLLSALSPEQLVLTLLEAEPPHVLIS
RPSTPFTEASMMMSLTKLADKELVHMISWAKKIPGFVELSLYDQVRLLESCWLEVLMVGLMWRSIDHPGK
LIFAPDLILDRDEGKCVEGILEIFDMLLATTSRFRELKLQHKEYLCVKAMILLNSSMYPSATAPQEADSG
RKLTHLLNAVTDALVWVIAKSGMSSQQQSMRLANLLMLLSHVRHASNKGMEHLLNMKCKNVVPVYDLLLE
MLNAHTLRGNKSLVTGSERNLVEDSESKEGSQKPQAQ

>Q5PR29_BRARE
MSSSPGPAPVLDSSKADRGASPALLPRLYASPLGMDNQTVCIPSPYVEACQDYSPPHGGEFNHGALTLYS
PVSSAVLGFHRPPVSESLVPLSPTILWPPHSLHYPPPLAYSETRSHSAWEEAKTHTLSQSSSVLSHTKLL
GQQLEGDNGLNPSASIVGKGDTHFCAVCHDYASGYHYGVWSCEGCKAFFKRSIQGHNDYICPATNQCTID
KSRRKSCQACRLRKCYEVGMMKCGVRRERCSYRGARHRRNPQIRDSSGGVVGLRGQSQQHLEFPLSPSQH
LFPSGGRAEGRGLNYSPEQLVSCILEAEPPQIYLREPVKKPYTEASMMMSLTSLADKELVLMISWAKKIP
GFVELTLSDQVHLLECCWLDILMLGLMWRSVDHPGKLIFTPDLKLNREEGNCVEGIMEIFDMLLATTSRF
RELKLQREEYVCLKAMILLNSNNCSSLPQTPEDVESRGKVLNLLDSVTDALVWIISRTGLSSQQQSIRLA
HLLMLLSHIRHLSNKGIEHLSNMKRKNVVLLYDLLLEMLDANTSQSSRMLEDRQQSPENLHTSRPQPDLK
DSDQETPHSPRAEETVNKTLHSSLLREDMDTN

>Q6XSH2_MICSA
MASSPGLDADPLPLLQLQEVDSSKASQRPSSPGLLPAVYSPPLGMDSHTVCIPSPYTDSSHEYNHSHGPL
TFYSPSVLSYARPPITDSPSSLCPPLSPSAFWPSHSHPNMPSLTLRCPQPLVYNEPSPHAHWPEPKAHSI
NPSSSILGCNKPLGKRLEEGVEGVNSSLCSSAVGKADMHFCAVCHDYASGYHYGVWSCEGCKAFFKRSIQ
GHNDYICPATNQCTIDKNRRKSCQACRLRKCYEVGMMKCGVRRERCSYRGARHRRGGLQPRDPTGRGLVR
MGLGSRAQRHLHLEAPLAPLTSLPQANHVHPSAMSPEEFISRIMEAEPPEIYLMEDMKKPFTEASMMMSL
TNLADKELVLMISWAKKIPGFVDLSLADQIHLLKCCWLEILMLGLMWRSVDYPGKLIFSPDFKLNREEGQ

CVEGFMEIFDMLLAATSRFRELKLQREEYVCLKAMILLNSNLCTSSPQTAEELESRNKLLRLLDSVIDAL
VWAISKLGLSTQQQTLRLGHLTMLLSHIRHVSNKGMDHLSTMKRKNVVLVYDLLLEMLDANTSSSGSQPS
SSPSSDTYSDQHQYPQPPSHLQPDSDQTGADHTTVPPHEPAEAPILDGHLQTLTLHSSPPFQSLVVAHID
SNDYIHQEQWSLDTADAGPPVEPTDYIVSDRVVMETALLG

>ESR2_PIG
MDIKNSPSNLNSPVSYNCSQSVLPLEPGPIYIPSSYVESCHEYSAMTFYSPAVVNYSISSNSEVGPGRQA
TSPNVLWPTPGHLSPLAIHCQPSLLYAEPQKSPWCDTRSLEHTLPVNRETLKRKASGSSCASPVTSPSSK
RDAHFCAVCSDYASGYHYGVWSCEGCKAFFKRSIQGHNDYICPATNQCTIDKNRRKSCQACRLRKCYEVG
MVKCGSRRERCGYRIVRKQRNSEGHLHCLSRAKKNGDHTTRVKELLLSTLSPEQLVLTLLEAEPPHVLVS
RPSTPFTEASMMMSLTKLADKELVHMISWAKKIPGFMELSLYDQVRLLESCWLEVLMVGLMWRSIDHPGK
LIFAPDLVLDRDEGKCVEGILEIFDMLLATTSRFRELKLQHKEYLCVKAMILLNSSMYPSAAAQEAESSR
KLTHLLNAVTDALVWVIARSGISSQQQSVRLANLLMLLSHVRHASNKGTEHLLNMKCKNVVPVYDLLLEM
LNAHTLRGNKSLVTGSERSRMEESESKEGSQKPQAQ

>ESR2_SHEEP
MDVKNSPSSLNSPVSYNCGQSILPLEPGPIYLPSSYVESRHEYSAVTFYSPAVMNYSIPNNSEDGPGRQT
TSPNVLWPTPGHLSPLAIHCQSSLLYAEPQKSPWCETRSLEHTFPVNRETLKRKASGSSCASPVSSPSSK
RDAHFCAVCSDYASGYHYGVWSCEGCKAFFKRSIQGHNDYICPATNQCTIDKNRRKSCQACRLRKCYEVG
MVKCGSRRERCGYRIVRRQRNSDEQLHCLSKTKRNGAPMTRVKELLLSALSPEQLVLTLLEAEPPHVLMS
RPSAPFTEASMMMSLTKLADKELVHMISWAKKIPGFVELSLYDQVRLLESCWLEVLMVGLMWRSIDHPGK
LIFAPDLVLDRDEGKCVEGILEIFDMLLATTSRFRELKLQHKEYLCVKAMILLNSSMYPSATASQEADSG
RKLTHLLNAVTDALVWVIAKSGMSSQQQSMRLANLLMLLSHVRHASNKGMEHLLNMKCKNVVPVYDLLLE
MLNAHTLRSNKPLVTRSERNLAEDSESKEGSQKPQAQ

>Q8QHK9_PAROL
MAAASAEKDQPLLQLQEVDSSRVRSCVLSPILSTSSPGLSLDGSQPICIPSPYTELGHDFATIPFYGPTI
FSYAAPSIPDCPSVHQSLSPSLFWPSHGHMGPPMTLHRSQGRSQQGQPIQSPWGELTPRDGVLANSKGVR
RRSQESEDGVVSSGGKSDLHYCAVCHDYASGYHYGVWSCEGCKAFFKRSIQGHNDYICPATNQCTIDKNR
RKSCQACRLRKCYEVGMTKCGMRKDHGSYRNPKTRRLTRLSSQGRASGPKALTGPVVALMNELQPPALTP
EQLIERIMEAEPPVIYLMKDMSGPLTEANVMMSLTHLADKELVHMITWAKKIPGFVELGLLDQVHLLECC
WLEVLMMGLMWRSVDHPGKLIFSPDLSLSREEGSCVQGFSEIFDMLIAATSRVRELKLQREEYVCLKAMI
LLNSNMCLSSSEGSEELHSRSKLLLLLDAVTDALVWAIAKTGLTFRQQYTRLAHLLMLLSHIRHVSNKGM
DHLHCMKMKNMVPLYDLLLEMLDAHIMHSSRLPHHASPQPEFTDQGEVPARPGSSGNGSSNTWTPSTSGD
GGEPQ

>Q98SM8_BRARE
MSSSPGPAPVLDSSKADRGASPALLPRLYASPLGMDNQTVCIPSPYVEACQDYSPPHGGEFNHGALTLYS
PVSSAVLGFHRPPVSESLVPLSPTILWPPHSLHCPPPLAYSETRSHSAWEEAKTHTLSQSSSVLSHTKLL
GQQLEGDNGLNPSASIVGKGDTHFCAVCHDYASGYHYGVWSCEGCKAFFKRSIQGHNDYICPATNQCTID
KSRRKSCQACRLRKCYEVDMMKCGVRRERCSYRGARHRRNPQIRDSSGGVVGLRGQSQQHLEFPLSPSQH
LFPSGGRAEGRGLNYSPEQLVSCILEAEPPQIYLREPVKKPYTEASMMMSLTSLADKELVLMISWAKKIP
GFVELTLSDQVHLLECCWLDILMLGLMWRSVDHPGKLIFTPDLKLNREEGNCVEGIMEIFDMLLATTSRF
RELKLQREEYVCLKAMILLNSNNCSSLPQTPEDVESRGKVLNLLDSVTDALVWIISRTGLSSQQQSIRLA

HLLMLLSHIRHLSNKGIEHLSNMKRKNVVLLYDLLLEMLDANTSQSSRMLEDRQQSPENLHTSRPQPDLK
DSDQETPHSPRAEETLNKTLHSSLLREDMDTN

>ESR2_ANGJA
MAGSPGNELPLLQLQEVDSSKVGESGGSSGLLPTMYNGALPALSMESHAVCIPSPYTDSSHDYAALTFYS
PPILSHGGPAVPESPAARQSLSPSLFWPAHGHHGHVSPLALHFQQPLVYREPAHSPWAEPKPLEHGQAQT
SKLAGKRMAESEEGTSSVGGCFAGKGDMHFCAVCHDYASGYHYGVWSCEGCKAFFKRSIQGHNGYICPAT
NQCTIDKNRRKSCQACRLRKCYEVGMMKCGVRRERCTYRGARHRRMPHIRELAGTGGGARTQRRGEGVVP
QTQEAQSSALTPEQLINRIIEAEPPEIYLMKELKKPFTEDSMMMSLTNLADKELVLMISWAKKIPGFVEL
DLSDQVHLLECCWLEVLMLGLMWRSVDHPGKLIFSPDLKLNRDEGSCVEGILEIFDMVLAATSRFRELKL
QREEYVCLKAIILLNPNLCTTSSENREELESRNKLLHMLDSVTDALVWTIAKKGLTFQQQSARLAHLLML
LAHIRHLSNKGMEHLSNMKRKNVVPLYDLLLEMLDANTMHSSRMSASYSSQPSPWSQAAQSQPGPPPSCS
GECPCPPKESSTI

>Q90WS9_BRARE
MSSSPGPAPVLDSSKADRGASPALLPRLYASPLGMDNQTVCIPSPYVEACQDYSPPHGGEFNHGALTLYS
PVSSAVLGFHRPPVSESLVPLSPTILWPPHSLHCPPPLAYSETRSHSAWEEAKTHTLSQSSSVLSHTKLL
GQQLEGDNGLNPSASIVGKGDTHFCAVCHDYASGYHYGVWSCEGCKAFFKRSIQGHNDYICPATNQCTID
KSRRKSCQACRLRKCYEVGMMKCGVRRERCSYRGARHRRNPQIRDSSGGVVGLRGQSQQHLEFPLSPSQH
LFPSGGRAEGRALNYSPEQLVSCILEAEPPQIYLREPVKKPYTEASMMMSLTSLADKELVLMISWAKKIP
GFVELTLSDQVHLLECCWLDILMLGLMWRSVDHPGKLIFTPDLKLNREEGNCVEGIMEIFDMLLATTSRF
RELKLQREEYVCLKAMILLNSNNCSSLPQTPEDVESRGKVLNLLDSVTDALVWIISRTGLSSQQQSIRLA
HLLMLLSHIRHLSNKGIEHLSNMKRKNVVLLYDLLLEMLDANASQSSRMLEDRQQSPENLHTSRPQPDLK
DSDQETPHSPRAEEMVNKTLHSSLLREDMDTN

>ESR2_CALJA
MDIKNSPSSLNSPSSYNFGQSILPLEHGPIYIPSSYVESHHEYPAMTFYSPAVMNYSIPSSVTNLEEGPG
RQITSPNMLWSTPGHLSPLAVHHQLSHLYAEPQKSPWCEARSLEHTLPVSRETLKRKVSGNHCASPVTGP
SSKRDAHFCAVCSDYASGYHYGVWSCEGCKAFFKRSIQGHNDYICPATNQCTIDKNRRKSCQACRLRKCY
EVGMVKCGSRRERCGYRLVRRQGNAEEQLHCAGKAKRSGGHVPRVRELLLSALSPEQLVLTLLEAEPPHV
LISRPSVPFTEASMMMSLTKLADEELVHMISWAKKIPGFVELSLLDQVRLLESCWLEVLMVGLMWRSIDH
PGKLIFAPNLILDRDEGKCVEGILEVFDMLLATTSRFRELKLQHKEYLCVKAMVLLNSQYDPLVTATQDA
ESSQKLAHLLNAVTDALVWVIAKSGFSSQQQSVRLANLLMLLSHIRHASNKGMEHLLSMKCKNVVPVYDL
LLEMMNAHVVRGCKSSITGSECSPAEDSKSTEGSQNPQSP

>ESR1_PAGMA
MYPEDSRGSGGVATVDFLEGTYDYAAPTPAPTPLYSHSTPGYYSAPLDAHGPPSDGSLQSLGSGPNSPLV
FVPSSPRLSPFMHPPTHHYLETTSTPVYRSSVPSSQQSVSREDQCGTSDDSYSVGESGAGALAAGFEIAK
EMRFCAVCSDYASGYHYGVWSCEGCKAFFKRSIQGHNDYMCPATNQCTIDRNRRKSCQACRLRKCYEVGM
MKGGMRKDRGRVLRRDKQRTGTSDRDKASKGLEHRTAPPQDRRKHISSSAGGGGGKSSMISMPPDQVLLL
LQGAEPPMLCSRQKLNRPYTEVTMMTLLTSMADKELVHMIAWAKKLPGFLQLSLHDQVQLLESSWLEVLM
IGLIWRSIHCPGKLIFAQDLILDRSEGDCVEGMAEIFDMLLATASRFRMLKLKPEEFVCLKAIILLNSGA
FSFCTGTMEPLHDGAAVQNMLDTITDALIHHINQSGCSAQQQSRRQAQLLLLLSHIRHMSNKGMEHLYSM
KCKNKVPLYDLLLEMLDAHRIHRADRPAETWSQADREPPFTSRNSSGGGGGGGGGSSSAGSTSGPRVSHE

SPTSPGVLQYGGSRSECTHIL

>Q6H9M5_SPAAU

MAASPGLDSRSLLQLQEVDSSKPSERPSSPRQLPAAYSPPLGMDSHTVCIPSPYADSSHEYNHGHGPLNF
YSQSVLSYARQPVTDSPSYLCPSISPSAFWPSHNHPSMPSLTLQCPQPHVYNEPSPHAPWLEPKAHAVTT
SSAVISCNKLPGKRSDERGEGANSSSCSSAVEKADMHFCAVCHDYASGYHYGVWSCEGCKAFFKRSIQGH
NDYICPATNQCTIDKNRRKSCQACRLRKCYEVGMMKCGVRRERCSYRGARHRRGGLQARDPTGRGLVRVG
LGSRGQRHLHLEAPLTPLPQAKRVHHSAMSPEEFISRIMEAEPPEIYLMEDMNKPFTESSMMMSLTNLAD
KELVLMISWAKKIPGFVELSLADQIHLLKCCWLEILMLGLMWRSVDHPGKLIFSPDFKLNREEGQCVEGI
MEIFDMLLAATSRFRELKLQREEYVCLKAMILLNSYLCTNSPETAEELESRNKLLRLLDSVIDALVWAIS
KLGLTTQQQTLRLGHLTMLLSHIRHVSNKGMDHLSTMKRKNVVLVYDLLLEMLDANTTTSGSQASSSPTS
ETFPDQHQYPQAPSHLQPGSDQAAADHTAVPPRGPAEAPILDGHLQALTLQSSPHFQSLEMTHMDSNQYI
HPEQWSLETRDAALSVDGSVDYMSPDPSVMDTDLVNGL

>Q804Q6_ACASC

MYPEDSRVSGGVATVDFLEGTYDYAAPTPAPTPLYSHSTPGYYSAPLDAHGPPSDGSLQSLGSGPNSPLV
FVPSSPRLSPFMHPPTHHYLETTSTPIYRSSVPSSQHSASREDQCGTSDDSYSVGESGAGAGAAGFEMAK
EMRFCAVCSDYASGYHYGVWSCEGCKAFFKRSIQGHNDYMCPATNQCTIDRNRRKSCQACRLRKCYEVGM
MKGGVRKDRGRVLRRDKRRTGTSDRDKASKGLEHRTAPPQDRRKHISSSAAGGGGKSSVISMPPDQVLLL
LQGAEPPMLCSRQKVNRPYTEVTVMTLLTSMADKELVHMIAWAKKLPGFLQLSLHDQVQLLESSWLEVLM
IGLIWRSIHCPGKFIFAQDFILDRSEGDCVEGMAEIFDMLLATASRFRMLKLKPEEFVCLKAIVLLNSGA
FSFCTGTMEPLHDGAAVQNMLDTITDALIHHINQSGCTAQQQSRRQAQLLLLLSHIRHMSNKGMEHLYSM
KCKNKVPLYDLLLEMLDAHRVHRPDRPHETWSQADREPPFTSRNNRGSGGGGGSSSAGSTSGTRVSLENP
TGPGVLQYGRSAPSAPHPMKPTE

>Q6H9M4_SPAAU

MAASPELDSRSLLQLQEVDSSKPSERPSSPRQLPAAYSPPLGMDSHTVCIPSPYADSSHEYNHGHGPLNF
YSQSVLSYARQPVTDSPSYLCPSISPSAFWPSHNHPSMPSLTLQCPQPHVYNEPSPHAPWLEPKAHAVTT
SSAVISCNKLPGKRSDERGEGANSSSCSSAVEKADMHFCAVCHDYASGYHYGVWSCEGCKAFFKRSIQGH
NDYICPATNQCTIDKNRRKSCQACRLRKCYEVGMMKCGVRRERCSYRGARHRRGGLQARDPTGRGLVRVG
LGSRGQRHLHLEAPLTPLPQAKRVHHSAMSPEEFISRIMEAEPPEIYLMEDMNKPFTESSMMMSLTNLAD
KELVLMISWAKKIPGFVELSLADQIHLLKCCWLEILMLGLMWRSVDHPGKLIFSPDFKLNREEGQCVEGI
MEIFDMLLAATSRFRELKLQREEYVCLKAMILLNSYLCTNSPETAEELESRNKLLRLLDSVIDALVWAIS
KLGLTTQQQTLRLGHLTMLLSHIRHVSNKGMDHLSTMKRKNVVLVYDLLLEMLDANTTTSGSQASSSPTS
ETFPDQHQYPQAPSHLQPGSDQAAADHTAVPPRGPAEAPILDGHLQALTLQSSPHFQSLEMTHMDSNQYI
HPEQWSLETRDAALSVDGSVDYMSPDPTVMDTDLVNGL

>Q7T3U5_9TELE

MSSSPGPASASVSPALDSGKADRGDSPTFLPHLYTSPLGMDSQTICIPSPYLEACQDYSPSHGGEFNHGA
LTLYSPVSTSVIGYPHPPVSESLVPLSPTIFWPSHTTHTALSLHCPPQVAYSETHSHTAWKDARTHTLNQ
SSSVLTHAKLLAQQVEGDDGLNPSPGIVGNGDTHFCAVCHDYASGYHYGVWSCEGCKAFFKRSIQGHNDY
MCPATNQCTIDKSRRKSCQACRLRKCYEMGMMKCGVRRERCSYRGARHRRNPQIRDSSGGALGVRGCSQH
HLEIPLNPTHHLFPSGGRAEGCGLSFSPEQLVNCILEAEPPQICLREPVKKPYTEASMMMSLTSLADKEL
VLMISWAKKIPGFVELTLSDQVHLLECCWLDILMLGLMWRSVDHPGKLIFSPDLKLNRDEWNCVEGIMEI

FDMLLATTSRFRELKLQREEYVCLKAMILLNSNNCSSLSQSPEDVESRGKVLRLLDSVTDALVWSISRTG
LSSQQQSIRLAHLLMLLSHIRHLSNKGIEHLSSMKRKNVVLLYDLLLEMLDANTSQSSRMLVAHTEASLT
TDLHTSRPQPAPRESDQETQRSPQAEETLHSSIHREDMDTD

>ESRB2_CARAU
MSSSTGPAPASASPVQANRGNSPNILPLLYTSQLGMDSQTICIPSPYVEACQDYSPPHGGEISHGALTLY
SPVSSPVLGYTHPPVSESLVPLNSAIFWPPHPTHSTPSLHCPSPLAYRETHAHTTWEDAKTHINQSSSVL
THAKLLGQQLDGDDGLNPSPGILGKGDTHFCAVCHDYASGYHYGVWSCEGCKAFFKRSIQGHNDYICPAT
NQCTIDKSRRKSCQACRLRKCYEVGMMKCGVRRERCSYRGGRHRRNPPIRDSSGGAIGVRGHSQPHLEFP
LSPTHPLFPLGDRAEGCGQNLSPEQLVNCILEAEPPQIYLREPIKKPYTEASMMMSLTNLADKELVLMIS
WAKKIPGFVELTLSDQVHLLECCWLDILMLGLMWRSVDHPGKLIFSPDLKLNRDEGTCVEGIMEIFDMLL
ATTSRFRELKLQREEYVCLKAMILLNSSNCSRLPQTPEDVESRGKVLRLLDSVTDALVWTISRTGLSSHQ
QSIRLAHLLMLLSHIRHLSNKGIEHLSTMKRKNVVLLYDLLLEMLDANTSQSSRMLAAHTKASLRMDTQQ
TTEILHTSKQQPALKESNQDTRHSPQAEGTVDKTLHRVDKTLHRVDVDTD

>ESR1_SPAAU
MYPEDSRVSGGVATVDFLEGTYDYAAPTPAPTPLYSHSTPGYYSAPLDAHGPPSDGSLQSLGSGPNSPLV
FVPSSPHLSPFMQPANHHYLETTSTPIYSVPSSQHSVSREDQCGTSDDSYSVGESGAGAGAAGFEMAKEM
RFCAVCSDYASGYHYGVWSCEGCKAFFKRSIQGHNDYMCPATNQCTIDRNRRKSCQACRLRKCYEVGMMK
GGVRKDRGRVLRRDKRRTGTSDRDKASKGLEHRTAPPQDRRKHISSSAGGGGGKSSVISMPPDQVLLLLR
GAEPPMLCSRQKVNRPYTEVTVMTLLTSMADKELVHMIAWAKKLPGFLQLSLHDQVQLLESSWLEVLMIG
LIWRSIHCPGKLIFAQDLILDRSEGDCVEGMAEIFDMLLATASRFRMLKLKPEEFVCLKAIILLNSGAFS
FCTGTMEPLHDSAAVQNMLDTITDALIHHINQSGCSAQQQSRRQAQLLLLLSHIRHMSNKGMEHLYSMKC
KNKVPLYDLLLEMLDAHRVHRPDRPAETWSQADREPLFTSRNSSSSSGGGGGGSSSAGSTSGPQVNLESP
TGPGVLQLRVHPHPMKPTE

>Q8UW75_ORYLA
MGTTLDSEKHQPLLQLQEVDSSRAGSCVLSPNLSSSSPGLSHETSQPICIPAPYADLSHDFTSLPFYNPT
IFSYASPSMSECPSVHQSLSASLFWQSHGHVGPTIPLHRSQARAQHGQPIQSPWDGVLTTSKGVRRRSQE
SEEAVVSSGGKSELHYCAVCHDYASGYHYGVWSCEGCKAFFKRSIQGHTDYICPATNQCTIDKNRRKSCQ
ACRLRKCYEVGMTKCGVRKERSSYRSAPARRAGRLTSQGRMNGPKVSSGPKESSGNEQSSLHLTPEQLIA
RMMEAEPPDIYLMKDTKKPLTEAVVMMSLTNLADKELVHMITWAKKIPGFVELSLLDQVHLLECCWLEVL
MMGLMWRSVGHPGKLIFSPDLSLSREEGSCVQGFVEIFDMLIAATSRVRELNLQREEYVCLKAMILLNSN
MCLSSSEGGGELHSRSKLLCLLDAVTDALVWAIGKSGLNFRQQYTRLAHLLMLLSHIRHVSNKGMDHLHC
MKMKNMVPLYDLLLEMLDAHIMHSSRLPRQPPPQDAADPTETSAQGQRSCCDVSKAWTTSSAGTAEEPQK
SD

>ESR3_MICUN
MAVASSPEKDQPLLQLQKVDSSRVGGQVLSPTLSSSLETSQPICITSPYTDLGHDFPTIPFYSPTIFSYA
GPSISDCTSVHQSLNPSLFWPSRGHMGSPIPLHHSQHGQPIQSPWVEISPLDNVLKTKQDGASLPLAVVP
VRHKSARRRSQESEEAVVTSGGKTDLHYCAVCHDYASGYHYGVWSCEGCKAFFKRSIQRDNEYICPATNE
CTIDKNRRKSCQACRLRKCYEVGMTKCGMRKERGNYRSPQMRRMTRLTSQGRTDSSSVLTGSAVVSLNAP
QPSALTSEQLIERLMEAEPPEIYLMKDMKKPLTEAKVMMSLTNLADKELVHMITWAKKIPGFVELGLLDQ
VHLLECCWLEVLMVGLMWRSVDHPGKLVFSPDLSLSREEGSCVQGFAEIFDMLLAATSRVRELKLQREEY

VCLKAMILLNSNMCLSSSESSSKLLRLLDAVTDALVSAIGKTVLSFRQQYTRLAHLLMLLSHIRHVSNKG
MDHLHCMKMKNMVPLYDLLLEMLDAHIMHSSRLPRRSPEQEPEDQADAPAPPHSSGSGPSYTWTPSSSEG
AGEPQ

>Q7T2K7_9LABR
MASSPGLDTDPLPLLRLQEVDSSKASERPSSPGLLPAVYSPRQGMDSHTVYIPSPYTDNNQEYNHGSGSV
SFYSPSVLSYARPSATDSPSSLCGPLSPSAFWPPHSQPNLPSLTLRCPQPLGYNESGLHAPWLESKPHNI
SSSSSIIGCNKPLGKRSEEGVNGVNPSLCSSVVGKADMHFCAVCHDYASGYHYGVWSCEGCKAFFKRSIQ
GHNDYICPATNQCTIDKNRRKSCQACRLRKCYEVGMMKCGVRRERCSYRGTRHRRGGLQPRDPTGRGLVR
VGLGSRAQRHLHLEGPLTPVTPLPQMSHVHHAAMSPEEFIMRIMEAEPPEIYLMEEQKKPFTEASMMMSL
TNLADKELVLMISWAKKIPGFVELCLADQIHLLKCCWLEILMLGLMWRSVDHPGKLIFSPDFKLNREEGQ
CVEGIMEIFDMLLAATSRFRELKLQREEYVCLKAMILLNSNLCSSSPQTEEELESRNKLLRLLDSVIDAL
VWAISKLGLSTQQQTLRLGHLTMLLSHIRHVSNKGMDHLSTMKRKNVVLVYDLLLEMLDANTSSSGSSQS
SSSPNSDSYSDLHQYPQHPSHLQPHQTTADHNNMPAHRQAEGQILEEELHTLPLQSSPPFHSQMATHMDR
NEYVHPQQHWSMDAEDAGPSVGSYMTSDRGVMEGALEVAGL

>Q6XSH1_MICSA
MAGACSIEKDQPLLQLQKVDSSRVGSRVVSPILNSPLETSQPICITSPYTDHSHDFTTIPFYNPTIFSYA
SPGISDRPSVHQSLSPSLFWPSHGHVGSPIPLHHSQPRPQYRQPIQNPWAELSPLESTLTTSKSVRRRSQ
ESQESVVSSGGKADLHYCAVCQDYASGYHYGVWSCEGCKAFFKRSIQRHNDYICPATNQCTIDKNRRKSC
QACRLRKCNEVGMTKFGIRKERGNCRNPQMRRVTRLSTQGRTNRPAVLTGPAAGSLIVPHSPALTPEQLI
ERIMDAEPPEIDLMKDMRRPLTEANVMMSLTNLADKELVHMISWAKKIPGFVELGLLDQVHLLECCWLEV
LMVGLMWRSVDHPGKLIFSRDLSLSREEGSCVQGFAEIFDMLIAATSRVRELKLQREEYVCLKAMILLNS
NMCLSSSEGSEELQSRSKLLRLLDAVTDALVWAIAKTGLTFRQQYTRLAHLLMLLSHIRHVSNKGMDHLH
CMKMKNMVPLYDLLLEMLDAHIMHSSRLSHQPTQQDAEDQREAPARPHSSGSCPSNTWTPGGGEPQ

>Q9DEV4_XENLA
MDSHEAVTSILPILPPDLNLDNMPILPLNPAYLAARARQILGMEEGGQRSSPDTSQTSVPFTNLQPNVKQ
ISYFNPEIQPDPRIKKDSSIVSLYKVAAKESTLCQDYGSSPRNPSTPDTDPSLDFILYKNEDYDCFKISH
GNTNEDSGCILPSTSAQTIYQPLSLNGHQYVTFQPTPMKETYLPQIQLPYVTYIRSDGDPERGIPFSFEM
LPQKICLICGDEASGCHYGVLTCGSCKVFFKRAIEGHQNYLCAGRNDCIVDKIRRKNCPSCRLRKCCQAG
MVLGGRKFKKFGRIKTGREIDTVVLQSPPTLSLECQQILIRRISNSSAQEIQFTPELLQILQSIEPEVVY
AGYDTTQPETPSALLSSLNQLCERQLVCVVKWSKSLPGFRNLHIDDQITLLQYSWMSLMVFALGWRSYQH
VSGQMLYFAPDLILNEQRMKDSSFYTLCLSMWQLPQEFMKLQVTHEEFLCMKALLLLNTIPLEGLKSQTN
FDEMRSNYIRELAKAISLRHKGVIASSQRFYQLTKLMDSMHELVKQLHLYCLNTFLQSRSLSVEFPEMMS
EVISAQLPKILAGMVKPLVFHKK

>ESR2_ORENI
MMAAASSPEKLLQLQEVDSSRAGSRILSPILGSSSPGLSHETSQPICIRSPYTDLGHDFTTIPFYSPTIF
SYGGPSISECSSVHQSLSASLFWPSHGRVGTPITLHCPQGRSQQGQSAQTPWDSVITTSKSVRRRSQESE
ESMVSSGGKADLHYCAVCHDYASGYHYGVWSCEGCKAFFKRSIQGHNDYICPATNQCTIDKNRRKSCQAC
RLRKCYEVGMTKCGIRKERGNYRNSQARRLTRLSSQGKTAEPKGITGPAEGSLNKPEKPALTPEQLIERI
LEAEPPEIYLVKDAKRPLTEASVMMLLTNLADKELVHMISWAKKIPGFVELSLVDQVHLLECCWLEVLMI
GLMWRSVDHPGKLIFCPDLSLSREEGSCVQGFVEIFDMLIAATTRVRELKLQREEYVCLKAMILLNSNMC

LSSSDCSEDLQSRSKLLRLLDAMTDALVLAIGKTGLTFRQQYTRLAHLLMLLSHIRHVSNKGMDHLHCMK
MKNIVPLYDLLLEMLDAHIMHSSCLPHQPPQQDSKDQSEVPAPLHSSAGGPSNTWTPSSARAGGESQ

>ESR2_SPAAU
MAVACSPEKDQSLLQLQKVDSSRVILSPVLSSPMETNQPICIPSPYTDRGHDFPTIPFYSATNFSYANPP
AISDRPSVHQTLSPSLFWPSHGHVGTTLPLHHLQARPQHGQAVQSPWVELSPLDNVLTSSKSARRRSQEN
EEGEVSSGGKADLHFCAVCHDYASGYHYGVWSCEGCKAFFKRSIQRHNDYICPATNQCTIDKNRRKSCQA
CRLHKCYNVGMTKCGMRKERGNFRDPQMRRVTRLSSQGRTSGPSVLNGPAVGPLNTPQPPALTSKQLIER
IMEAEPPEIYLMKDMRRPLTEANIMMSLTNLADKELVHMITWAKKIPGFLELGLLDQVHLLECCWLEVLM
IGLMWRSVDHPGKLIFSPDLSLSREEGSCVQGFLEIFDMLIAATSRVRELKLQREEYVCLKAMILLNSNM
CLSSSEGSEELQSRSKLLRLLDAVTDALVWAIAKTGLTFRQQYTRLAHLLMLLSHIRHVSNKGMDHLHGM
KMKNMVPLYDLLLEMLDAHIMHSSRLPRRSPQQETVEQCDAPARPHSPGTSGPTNTWTPSCTGGRGEPQ

>Q7T2K8_9LABR
MYPEESRGSGGVGTVDFLEGTYDYTAPTPAPTLYSLSTQGYYSAALDTHGQPSDSSIQSLGSGPSSPLVF
VPSSPRLSPFMHLPSHHYLETSSTPVYRSSVSSSQQSISREEHCGTSDESYSMGESGAGAAAGCFEMAKE
MRYCAVCSDYASGYHYGVWSCEGCKAFFKRSIQGHNDYMCPATNQCTIDRNRRKSCQACRLRKCYEVGMM
KGGVRKDRGRVLRRDKRRTGTSDKDNGSKDREQRTVPPQGRRKHGSSVGGGKSPVISMPPDQVLLLLQGA
EPPILCSRQKLSRPYTEVTMMTLLTSMTDRELVHMIAWAKKLPGFLQLTLHDQVQLLESSWLEVLMIGLI
WRSIHCPGKLIFAQDLILDRSEGDCVEGMAEIFDMLLATTSRFRMLKLKPEEFVCLKAIILLNSGAFSFC
TGTMEPLHDNEAVQNMLDIITDALIHHISQSGCSAHQQSRRQAQLLLLLSHIRHMSNKGMEHLYSMKCKN
KVPLYDLLLEMLDAHRLHRPDRPAESWYQTDREPAYSSSATTTNDNSSSSPAGSRASQESPNRPPTGHSV
LQFGGSRSDCTHIL

>Q6TGB3_HAPBU
MYPEESRGSGGVATVDFLEGSYDYAAPTPAPTPLYSHSTTGCYSAPLDAHGPPSDGSLQSLGSGTTSPLV
FVPSSPRLSPFMHPPSHHYLETTSTPVYRSSHQPVPRDDQCGTRDEAYGLGELGAGAGGFEMTKETRFCA
VCSDYASGYHYGVWSCEGCKAFFKRSIQGHNDYMCPATNQCTIDKNRRKSCQACRLRKCYEVGMMKGGMR
KDRGRVLRREKRRAYDRDKPAKDLPHTKAPPHDGRKHATSSSSTSGGGGRSSLNSIPPDQVLLLLQGAEP
PTLCSRQKMNQPYTEVTMMTLLTSMADKELVHMIAWAKKLPGFLQLSLHDQVLLLESSWLEVLMIGLIWR
SIHCPGKLIFAQDLILDRTEGTCVEGMAEIFDMLLATASRFRMLKLKPEEFVCLKAIILLNSGAFSFCTG
TMEPLHDSAAVQHMLDTITDALIFHISQLGCSAQHQSRRQAQLLLLLSHIRHMSNKGMEHLYSMKCKNKV
PLYDLLLEMLDAQRIHRPVKPSQSWSQGDRDSPNTSSSGGGGSDDEGTSSAGSSSGPQGNHESPRCENLS
RAPTGPGVLQYRGSHSDCTPIL

>Q800Q2_ZOAVI
MYPEESRGSGGVTTVDFLEGTYDYAAPTPAPTPLYSHSTPGFYSAPLDSHRPPSDGSLQSLGSGPTSPIV
FVPSSPRLSPFLHPPSHHYLETTSTHVYSVPSSQQSVSREDQCGTSDESYSLVESGAGAGAGGFEMAKET
RFCAVCSDYASGYHYGVWSCEGCKAFFKRSIQGHNDYMCPATNQCTIDRNRRKSCQACRLRKCYEVGMMK
GGVRKDRGHVLRRDKRRSDLSDRDKASKDLEHRTVPSQDRRKRSSISSACVGGKSMLTSMPPDQVLLLLQ
CAEPPILCSRQKLSRPYTEVTMMTLLTSMADKELVHMIAWAKKLPGFLQLGLHDQVQLLESSWLEVLMIG
LIWRSIHCPGKLIFAQDLILDRNEGDCVEGMAEIFDMLLATTSRFRLLKLKPEEFVCLKAIILLNSGAFS
FCTATMEPLHDTAAVQHMLDTITDTLIHHIGQSGCSVQQQSRRQAQLLLLLSHIRHMSNKGMEHLYSMKC
KNKVPLYDLLLEMLDAHRLHRPDKPGESWFPADGETLCTTSDNISSGGSGSGPRVSHDSPSRAPTVLQYG

GSISDCTHIL

>ESR1_ORENI
MYPEESRGSGGVATVDFLEGTYDYAAPTPAPTPLYSHSTTGCYSAPLDAHGPLSDGSLQSLGSGPTSPLV
FVPSSPRLSPFMHPPSHHYLETTSTPVYRSSHQPVPREDQCGTRDEAYSVGELGAGAGGFEMTKDTRFCA
VCSDYASGYHYGVWSCEGCKAFFKRSIQGHNDYMCPATNQCTIDKNRRKSCQACRLRKCYEVGMMKGGMR
KDRGRVLRREKRRACDRDKPAKDLPHTRASPQDGRKRAMSSSSTSGGGGRSSLNNMPPDQVLLLLQGAEP
PILSSRQKMSRPYTEVTIMTLLTSMADKELVHMITWAKKLPGFLQLSLHDQVLLLESSWLEVLMIGLIWR
SIQCPGKLIFAQDLILDRNEGTCVEGMAEIFDMLLATASRFRVLKLKPEEFVCLKAIILLNSGAFSFCTG
TMEPLHDSAAVQHMLDTITDALIFHISHLGCSAQQQSRRQAQLLLLLSHIRHMSNKGMEHLYSMKCKNKV
PLYDLLLEMLDAHRIHRPVKPFQSWSQGDRDSPTASSTSSSGGGGGDDEGASSAGSSSGPQGSHESPRRE
NLSRAPTGPGVLQYRGSHSDCTRIP

>Q804Q7_ACASC
MAVACSPEKDQSLLQLQKVDSSRVILSPVLSSPMETNQPICIPSPYTDRGHDFPAIPFYSPTNFSYANPP
SISDRPSVHQTLSSSLFWPSHGHVGTTLPLHHLQARPQHGQPVQSPWVELSPLDNVLTSSKSARRRSQES
EEGEVSSGGKADLHFCAVCHDYASGYHYGVWSCEGCKAFFKRSIQRHNDYICPATNQCTIDKNRRKSCQA
CRLHKCYNVGMTKCGMRKERGNFRNPQMRRVTRLSSQGRTNGPAVGPLIAPQPPALTSKQLIERIIEAEP
PEIYLMKDVRRPLTEANIMMSLTNLADKELVHMITWAKKIPGFLELGLLDQVHLLECCWLEVLMIGLMWR
SVDHPGKLIFSPDLSLSREEGSCVQGFLEIFDMLIAATSRVRELKLQREEYVCLKAMILLNSNMCLSSSE
GSEELQSRSKLLHLLDAVTDALVWAIAKTGLTFRQQYTRLAHLLMLLSHIRHVSNKGMDHLHGMKMKNMV
PLYDLLLEMLDAHIMHSSRLPRRSPQQETGDQCDGPARPHSPGPSGPSNTWTPSSTGGRGEPQ

>Q8QHL0_PAROL
MYPEESRGSGGAATVDFLEGTYDYAAPTPAQTPLYSHSTSGYYSAPLDAHGPPSDGSRHSLGSGPTSPHV
YVPSSPRLSPFMHPPSHHYLETTATSVYRSSQQPVTREDHCGPRDESFSVGETGAAAGAEGFEMAKETRF
CAVCSDYASGYHYGVWSCEGCKAFFKRSIQGHNDYMCPATNQCTIDRNRRKSCQACRLRKCYEVGMMKGG
VRKDRSHVLRRDKRRAGTNDRDKASKDQDHKTVPLQDGRKSSSSTAGGKSSVTAMLPDQVLVLLQGAEPP
ILCSRQKLNQPYTEVTMMTLLTSMADRELVHMIAWAKKLPGFLQLSLHDQVQLLESSWLEVLMIGLIWRS
IHCPGKLIFAQDLILDRNEGNCVEGMAEIFDMLLATASRFRMLKLKSEEFFCLKAIILLNSGSFSFCTGT
MEPLHNTAAVQDMLETITDALIHHISQSGCPVQQQWRRQAQLLLLLSHIRHMSNKGMEHLYSMKCKNKVP
LYDLLLEMLDAHCLHRPARPAQSWLQADREPSAAGNNNNNSSSIIISGGGSSSASSGHRGSQESPSRATT
GPSVLQHGGSRPDCTHIL

>ESR1_ICTPU
MSEEQARAEAPAGARQRRRSELEGYSVSLASLKLSPMYPEEEQRTTGGISSTAHYLDGTFNYTTNPDATN
SSVDYYSVAPEPQEENLQPLPNGSSSPPVFVPSSPQLSPFLGHPPAGQHTAQQVPYYLEPSGTSIYRSSV
LASAGSRVELCSAPGRQDVYTAVGASGPSGASGPSGAIGLVKEIRYCSVCSDYASGYHYGVWSCEGCKAF
FKRSIQGHNDYVCPATNQCTIDRNRRKSCQACRLRKCYEVGMMKGGFRKERGGRIIKHNRRPSGLKERER
GYSKAQSGSDVREALPQDGQSSSGIGGGVADVVCMSPEQVLLLLLRAEPPTLCSRQKHSRPYSELTIMSL
LTNMADRELVHMIAWAKKVPGFQDLSLHDQVQLLESSWLEILMIGLIWRSIYTPGKLIFAQDLILDKSEG
ECVEGMAEIFDMLLATVARFRTLKLKSEEFVCLKAIILLNSGAFSFCSSPVEPLRDGFMVQCMMDNITDA
LIYYISQSGISVQLQSRRQAQLLLLLSHIRHMSYKGMEHLYSMKCKNKVPLYDLLLEMLDAHRLRPLGKV
PRIWADRVSSSPTTTATTPTTNTTTTTTTTTHHPSNGSTCPADLPSNPPGPGQSPSP

>Q6W5G9_XENTR
MLFFCLFIIMLSIPVASVFIAAAAAPVYSSASLSYAASSETFGSSSLTGLHTLNNVPPSPVVFLQTPQLS
PFIHHHGQQVPYYLESEQGTFAVREAAPPTFYRSSSDNRRQSGRERMTSANDKGPPSMESTKETRYCAVC
SDYASGYHYGVWSCEGCKAFFKRSIQGHNDYMCPATNQCTIDKNRRKSCQACRLRKCYEVGMMKGGIRKD
RRGGRMLKHKRQKEEQEQKNDVTPSEIRTTSIWVNPSVKSMKLSPVLSLTAEQLISALMEAEPPIVYSEH
DSTKPLSEASMMTLLTNLADKELVHMINWAKRVPGFVDLTLHDQVHLLECAWLEILMVGLIWRSVEHPGK
LSFAPNLLLDRNQGRCVEGLVEIFDMLVTTATRFRMMRLRGEEFICLKSIILLNSGVYTFLSSTLESLED
TDLIHIILDKIIDTLVHFMAKSGLSLQQQQRRLAQLLLILSHIRHMSNKGMEHLYSMKCKNVVPLYDLLL
EMLDAHRMHTPKDKATAQEEDSRSPLTTTANGASPCLQPFYTSTEEVSLQSTV

>ESR1_OREAU
MYPEESRGSGGVATVDFLEGLMTMTAPTPAPTPLYSHSTTGCYSAPLDAHGPLSDGSLQSLGSGPTSPLV
FVPSSPRLSPFMHPPSHHYLETTSTPVYRSSHQPVPREDQCGTRDEAYSVGELGAGAGGFEITKNTRFCA
VCSDYASGYHYGVWSCEGCKAFFKRSIQGHNDYMCPATNQCTIDKNRRKSCQACRLRKCYEVGMMKGGMR
KDRGRVLRREKHGPAQRQTSQNLPTHKASPQDGRKRAMSSSSTSGPGGRSSLNNMPPDQVLLLLQGAEPP
ILCSRQKMSRPYTEVTIMTLLTSMADKELVHMITWAKKLPGFLQLSLHDQVLLLESSWLEVLMIGLIWRS
IQCPGKLIFEEDLILDRNEGTCVEGMAEIFDMLLATVRFRVLKLKPEEFVCLKAIILLNSGAFSFCTGTM
EPLHDSVAVQHMLDTITDALIFHISHFGCSAQQQSRRQAQLLLLLSHIRHMSNKGMEHLYSMKCKNKVPL
YDLLLEMLDAHRIHRPVKPSQSWSQGDRDSPTASSTSSRGGGGGDDEGASSAGSSSGPQGSHESPRRENL
SRAPKGPGVLQYRGSHSDCTRIP

>Q69F36_CARAU
MYPKEEHSVGGISSSVNYLDGSYEFPNPTQTYGTSSPAEPASVGYYPAPPDPHVPPVEEHLQSLGSPRIF
APSSPQLSPYLSHPGGHPSTHQASYYLDTPSSSLYRSSVVSSQQAGGGLCEELCSASDRQEMYSGSRGTG
GFDSEKETRFCAVCSDYASGYHYGVWSCEGCKAFFKRSIQGHNDYVCPATNQCTIDRNRRKSCQACRLRK
CYEVGMVKGGIRKDRGGRAIRRERRRSGNEDLIKSYSEQSSHSGSRTAPPQDKRKKSSGGGVSALCLPPD
QVLLLLLEAEPPAVCSRQKHSRPFTEITMMSLLTNMADKELVHMIAWAKKVPGFQDLSLHDQVQLLESSW
LEVLMIGLIWRSIHSPGKLIFAQDLILDRSEGECVEGMTEIFDMLLATVARFRSLKLKLEEFVCLKAIIL
LNSGAFSFCSSSVEPLMDSFMVQCMLDNITDALIYCISKSGATPQLQSRRQAQLLLLLSHIRHMSNKGME
HLYRMKCKNRVPLYDLLLEMLDAQQFHSSRKVQRPWSQSEKDSWSTPTAGSSSPSRGPGAMQPNTARVQT
PDLCPVQQLKNVKTVNQSVLANRFEGGTEVSLCALSENTLDSRRPI

>Q95MF0_MACAR
MDIKNSPSSLNSPSSYNCSQSILPLEHGSIYIPSSYVESHHEYPAMTFYSPAVMNYSIPSNVTNLEGGPG
RQTASPNVLWPTPGHLSPLAVHRQLSHLYAEPQKSPWCEARSLEHTLPVNRETLKRKVSGNRCTSPVTSP
SSKRDAHFCAVCSDFASGYHYGVWSCEGCKAFFKRSIQGHNDYICPATNQCTIDKNRRKSCQACRLRKCY
EVGMVKCGSRRERCGYRLVRRQRSADEQVHCASKAKRSGSHTPLVRELLLDALSPEQLVLTLLEAEPPHV
LISRPSAPFTEASMMMSLTKLADKELVHMISWAKKIPGFVELSLFDQVRLLESCWMEVLMVGLMWRSIDH
PGKLIFAPDLVLDRDEGKCVEGILEIFDMLLATTSRFRELKLQHKEYLCVKAMILLNSNMYPLVTATQDA
DSSRKLAHLLNAVTDALVWVIAKSGISSQQQSMRLANLLMLLSHVRHARAEKASHSLTSFGMEDGDTLLP
EATDGSVTF

>ESR1_BRARE

MYPKEEHSAGGISSSVNYLDGAYEYPNPTQTFGTSSPAEPASVGYYPAPPDPHEEHLQTLGGGSSSPLMF
APSSPQLSPYLSHHGGHHTTPHQVSYYLDSSSSTVYRSSVVSSQQAAVGLCEELCSATDRQELYTGSRAA
GGFDSGKETRFCAVCSDYASGYHYGVWSCEGCKAFFKRSIQGHNDYVCPATNQCTIDRNRRKSCQACRLR
KCYEVGMMKGGIRKDRGGRSVRRERRRSSNEDRDKSSSDQCSRAGVRTTGPQDKRKKRSGGVVSTLCMSP
DQVLLLLLGAEPPAVCSRQKHSRPYTEITMMSLLTNMADKELVHMIAWAKKVPGFQDLSLHDQVQLLESS
WLEVLMIGLIWRSIHSPGKLIFAQDLILDRSEGECVEGMAEIFDMLLATVARFRSLKLKLEEFVCLKAII
LINSGAFSFCSSPVEPLMDNFMVQCMLDNITDALIYCISKSGASLQLQSRRQAQLLLLLSHIRHMSNKGM
EHLYRMKCKNRVPLYDLLLEMLDAQRFQSSGKVQRVWSQSEKNPPSTPTTSSSSSNNSPRGGAAAIQSNG
ACHSHSPDP

>Q5XXP1_9TELE
MYPKEEHSAEGISSSVNYLDGAYEYPDPTQTYGTTSPAEPLSVGYFLAPTDHHAPPVEEHMQTFSGESSS
PLMFAPTSPQLSPYLSHHGGHHSTHQVSYYLDTSSSTVYRSSVVSSQQAGVGLCEVLCSATDRQEMYTGS
RAAGGFDSEKETRFCAVCSDYASGYHYGVWSCEGCKAFFKRSIQGHNDYVCPATNQCTIDRNRRKSCQAC
RLRKCYEVGMMKGGIRKDRGGRAIRRERRKSDNEDRDKSYSEQSSRVGLRTPQDKRKKSSAEVVSALCMP
PDQVLVLLLGAEPPAVCSRQKHSPPYTEITMMSLLTNMADKELVHMIAWAKKVPGFQDLSLHDQVQLLES
SWLEVLMIGLIWRSIHSPGKLIFAQDLILDRNEGECVEGMAEIFDMLLATVARLRSLKLKLEEFVCLKAI
ILLNSGAFSFCSSPVEPLMDSFMVQCMLDNITDALIYGISKSGASLQLQSRRQAQLLLLLSHIRHMSNKG
MEHLYHMKCKNRVPLYDLLLEMLDAQRFQSPGEVQRLGAQSEKDPPSTPPTRGPGATQPNTGCLSQSPDP

>ESR2_ICTPU
MSSSLSPTLQTVNGMDQDEPLPLFPSHYPTALGVDRGTVCIPSPYADNGHAEMSFCGPSAPENPAIAPPL
SPSLFWSSHNPHAMPALPLHCPPALPYSEPHIHTAWVDTKPHTSGRHSSFLSRPKLFGKRPEDGDGDEAL
DDDDPSSSSSGAVVKRDMHFCVVCHDYASGYHYGVWSCEGCKAFFKRSIQGHNDYICPATNQCTIDKNRR
KSCQACRLRKCYEMGMMKCGARRERCGYRASRRTAPMRDGSARPVGVRGQSQRLPHTPLHVSLSIPVSRA
SAESGFSGLSPEQLVYCILEAEPPQIYLKQQMKKPYTESTVMMSLTQLADKELVLMISWAKKIPGFVELS
LAHQVQLLECCWLEVLMLGLMWRSIDHPGKLIFSLDLKLNRDEGNCVEGIMEIFDMLLAGSSRFRELKLQ
KEEYVCLKALILLNSSMYMTSSACEKDLESRTKLLRLLDAVTDALVWAISRTGLSTQQQSARLAHLLMLL
SHIRHLSNKGMEHLSSMKRKNVVLLYDLLLEMLDANMAQSRHVSTSVCTDPVTPATSPNTPLPPQLHSPV
AHHVTQVNESQCSQE

>Q90WV1_CARAU
MYPKEEHSTGGISSSLNYLDGAYDFSNLSETFGTSSPTEPASFGYYPAPPDPHAPPAEEHLQNLGTGPGS
PLMFAPSSPQLSPYLNHHGGHHSTHQVSYYLDSPSSTVYRSSVVCSQQAGVGLCEELCSAADRQELFSGS
RAAGGFDSEKETRFCAVCSDYASGYHYGVWSCEGCKAFFKRSIQGHNDYVCPATNQCTIDRNRRKSCQAC
RLRKCYEVGMMKGGIRKERGGRAIRRDRRRSGNEDRVKSYSEQSNRTGLRTAPPQDRRKSSSTGVVSTLC
MPPDQVLVLLLGAEPPAVCSRQKHSRPYTEITMMSLLTNMADKELVHMIAWAKKVPGFQDLSLHDKVQLL
ESTWLEVLMIGLIWRSIHSPGKLIFAQDLILDRNEGECVEGMAEIFDMLLATVTRFRSLKLKLEEFVCLK
ALILLNSGAFSFCSSPVEPLMDSFMVQCMLDNITDAFIYGISKSGASLQLQSRRQAQLLLLLSHIRHMSN
KGMEHLYRMKCKNRVPLYDLLLEMLDAQRFHSSGKVQRLWAQSEKDPPSTPTTSSRGPGTMLPNIACHEQ
SPDP

>Q95ME9_CALJA
MDIKNSPSSLNSPSSYNFGQSILPLEHGPIYIPSSYVESHHEYPAMTFYSPAVMNYSIPSSVTNLEEGPG

RQITSPNMLWSTPGHLSPLAVHHQLSHLYAEPQKSPWCEARSLEHTLPVSRETLKRKVSGNHCASPVTGP
SSKRDAHFCAVCSDYASGYHYGVWSCEGCKAFFKRSIQGHNDYICPATNQCTIDKNRRKSCQACRLRKCY
EVGMVKCGSRRERCGYRLVRRQGNAEEQLHCAGKAKRSGGHVPRVRELLLSALSPEQLVLTLLEAEPPHV
LISRPSVPFTEASMMMSLTKLADEELVHMISWAKKIPGFVELSLLDQVRLLESCWLEVLMVGLMWRSIDH
PGKLIFAPNLILDRDEGKCVEGILEVFDMLLATTSRFRELKLQHKEYLCVKAMVLLNSQYDPLVTATQDA
ESSQKLAHLLNAVTDALVWVIAKSGFSSQQQSVRLANLLMLLSHIRHARAEKASHSLTSFGMEDGD

>Q91Z86_MOUSE
MTFYSPAVMNYSVPSSTGNLEGGPVRQTASPNVLWPTSGHLSPLATHCQSSLLYAEPQKSPWCEARSLEH
TLPVNRETLKRKLGGSGCASPVTSPSAKRDAHFCAVCSDYASGYHYGVWSCEGCKAFFKRSIQGHNDYIC
PATNQCTIDKNRRKSCQACRLRKCYEVGMVKCGSRRERCGYRIVRRQRSASEQVHCLNKAKRTSGHTPRV
KELLLNSLSPEQLVLTLLEAEPPNVLVSRPSMPFTEASMMMSLTKLADKELVHMIGWAKKIPGFVELSLL
DQVRLLESCWMEVLMVGLMWRSIDHPGKLIFAPDLVLDRSSEDPHWHVAQTKSAVPRDEGKCVEGILEIF
GMLLATTARFRELKLQHKEYLCVKAMILLNSSMYPLATASQEAESSRKLTHLLNAVTDALVWVISKSGIS
SQQQSVRLANLLMLLSHVRHISNKGMEHLLSMKCKNVVPVYDLLLEMLNAHTLRGYKSSISGSECCSTED
SKSKEGSQNLQSQ

>ESR2_CHICK
MAFCSPAMLNYNIASNFGDSESASVRQTSSPSVLWSAPGHLSPLTLHCQSSLLYAEQPKSPWCEVRPLDP
VLPVTRETLKRKTNGSDCTSPIASNPGSKRDAHFCAVCSDYASGYHYGVWSCEGCKAFFKRSIQGHNDYI
CPATNQCTIDKNRRKSCQACRLRKCYEVGMMKCGSRRERCGYRILRRHRNSEDCMGKTKKYNEAATRVKE
ILLSTVSPEQFVLTLLEAEPPNVLVSRPSKPFTEASMMMSLTKLADKELVHMIGWAKKIPGFIDLSLYDQ
VRLLESCWMEVLMIGLMWRSIDHPGKLIFAPDLVLDRDEGKCVEGILEIFDMLLAMTSRFRELKLQHKEY
LCVKAMILLNSSMFPLSPEEPESNRKLHHLLNVVTDALVWVIAKSGIPSQQQTTRLANLLMLLSHVRHAS
NKGMEHLLSMKCKNVVPVYDLLLEMLNAHTLRGQRKSPVTHPEFEQVSHFQV

>ESR2_COTJA
MAFCSPAMMNYNIASNFGDSESASVRQTSSPSLLWSAPGHLSPLTLHCQLSLLYAEQPKSPWCEARPLEP
VLPVSRETLKRKTNGSDCTSPIASNPGSKRDAHFCAVCSDYASGYHYGVWSCEGCKAFFKRSIQGHNDYI
CPATNQCTIDKNRRKSCQACRLRKCYEVGMMKCGSRRERCGYRILRRHRNSEDCMGKTKKYNEAATRVKE
ILLSTVSPEQFVLTLLEAEPPNVLVSRPSKPFTEASMMMSLTKLADKELVHMIGWAKKIPGFIDLSLYDQ
VRLLESCWLEVLMIGLMWRSIDHPGKLIFAPDLVLDRDEGKCVEGILEIFDMLLAMTSRFRELKLQHKEY
LCVKAMILLNSSMFPLSAEEPESNRKLHHLLNVVTDALVWVIAKSGIPSQQQTTRLANLLMLLSHVRHAS
NKGMEHLLSMKCKNVVPVYDLLLEMLNAHTLRGQRKSPVTHPDFEQVSHFQV

>ERR3_HUMAN
MDSVELCLPESFSLHYEEELLCRMSNKDRHIDSSCSSFIKTEPSSPASLTDSVNHHSPGGSSDASGSYSS
TMNGHQNGLDSPPLYPSAPILGGSGPVRKLYDDCSSTIVEDPQTKCEYMLNSMPKRLCLVCGDIASGYHY
GVASCEACKAFFKRTIQGNIEYSCPATNECEITKRRRKSCQACRFMKCLKVGMLKEGVRLDRVRGGRQKY
KRRIDAENSPYLNPQLVQPAKKPYNKIVSHLLVAEPEKIYAMPDPTVPDSDIKALTTLCDLADRELVVII
GWAKHIPGFSTLSLADQMSLLQSAWMEILILGVVYRSLSFEDELVYADDYIMDEDQSKLAGLLDLNNAIL
QLVKKYKSMKLEKEEFVTLKAIALANSDSMHIEDVEAVQKLQDVLHEALQDYEAGQHMEDPRRAGKMLMT
LPLLRQTSTKAVQHFYNIKLEGKVPMHKLFLEMLEAKV

>ERR3_MOUSE
MDSVELCLPESFSLHYEEELLCRMSNKDRHIDSSCSSFIKTEPSSPASLTDSVNHHSPGGSSDASGSYSS
TMNGHQNGLDSPPLYPSAPILGGSGPVRKLYDDCSSTIVEDPQTKCEYMLNSMPKRLCLVCGDIASGYHY
GVASCEACKAFFKRTIQGNIEYSCPATNECEITKRRRKSCQACRFMKCLKVGMLKEGVRLDRVRGGRQKY
KRRIDAENSPYLNPQLVQPAKKPYNKIVSHLLVAEPEKIYAMPDPTVPDSDIKALTTLCDLADRELVVII
GWAKHIPGFSTLSLADQMSLLQSAWMEILILGVVYRSLSFEDELVYADDYIMDEDQSKLAGLLDLNNAIL
QLVKKYKSMKLEKEEFVTLKAIALANSDSMHIEDVEAVQKLQDVLHEALQDYEAGQHMEDPRRAGKMLMT
LPLLRQTSTKAVQHFYNIKLEGKVPMHKLFLEMLEAKV

>ERR1_MOUSE
DAARRGGAAGGGGPRSPRRSGPLGPHACPPALPEPKVTSTMSSQVVGIEPLYIKAEPASPDSPKGSSETE
TEPPVTLASGPAPARCLPGHKEEEDGEGAGSGEQGSGKLVLSSLPKRLCLVCGDVASGYHYGVASCEACK
AFFKRTIQGSIEYSCPASNECEITKRRRKACQACRFTKCLRVGMLKEGVRLDRVRGGRQKYKRRPEVDPL
PFPGPFPAGPLAVAGGPRKTAPVNALVSHLLVVEPEKLYAMPDPASPDGHLPAVATLCDLFDREIVVTIS
WAKSIPGFSSLSLSDQMSVLQSVWMEVLVLGVAQRSLPLQDELAFAEDLVLDEEGARAAGLGDLGAALLQ
LVRRLQALRLEREEYVLLKALALANSDSVHIEDAEAVEQLREALHEALLEYEAGRAGPGGGAERRRAGRL
PCTLPLLRQTAGKVLAHFYGVKLEGKVPMHKLFLEMLEAMMD

>Q5UKY7_CHICK
MSSKDRHIDSSCSSYIKTEPSSPASLTDSINHHSPGGSSDASGSYSSTMNGHQNGLDSPPLYPSATGLGG
NGPVRKRYDDCSSTIAEDSQTKCEYMLNSMPKRLCLVCGDIASGYHYGVASCEACKAFFKRTIQGNIEYS
CPATNECEITKRRRKSCQACRFMKCLKVGMLKEGVRLDRVRGGRQKYKRRIDAENSPYLNPQLVQPAKKP
YNKIVSHLLVAEPEKIYAMPDPTVPDSDIKALTTLCDLADRELVVIIGWAKHIPGFSTLSLADQMSLLQS
AWMEILILGVVYRSLSFEDELVYAEDYIMDEDQSKLAGLLDLNNAILQLVKKYKSMKLEKEEFVTLKAIA
LANSDSMHIEDVEAVQKLQDVLHEALQDYEAGQHMEDPRRAGKMLMTLPLLRQTSTKAVQHFYNIKLEGK
VPMHKLFLEMLEAKV

>ERR3_PONPY
MSNKDRHIDSSCSSFIKTEPSSPASLTDSVNHHSPGGSSDASGSYSSTMNGHQNGLDSPPLYPSAPILGG
SGPVRKLYDDCSSTIVEDPQTKCEYMLNSMPKRLCLVCGDIASGYHYGVASCEACKAFFKRTIQGNIEYS
CPATNECEITKRRRKSCQACRFMKCLKVGMLKEGVRLDRVRGGRQKYKRRIDAENSPYLNPQLVQPAKKP
YNKIVSHLLVAEPEKIYAMPDPTVPDSDIKALTTLCDLADRELVVIIGWAKHIPGFSTLSLADQMSLLQS
AWMEILILGVVYRSLSFEDELVYADDYIMDEDQSKLAGLLDLNNAILQLVKKYKSMKLEKEEFVTLKAIA
LANSDSMHIEDVEAVQKLQDVLHEALQDYEAGQHMEDPRRAGKMLMTLPLLRQTSTKAVQHFYNIKLEGK
VPMHKLFSEMLEAKV

>Q6W5G5_XENLA
MSSANDKGPPSMESTKETRYCAVCSDYASGYHYGVWSCEGCKAFFKRSIQGHNDYMCPATNQCTIDKNRR
KSCQACRLRKCYEVGMMKGGIRKDRRGGRMLKHKRQKEEQEQKNDVDPSEIRTASIWVNPSVKSMKLSPV
LSLTAEQLISALMEAEPPIVYSEHDSTKPLSEASMMTLLTNLADKELVHMINWAKRVPGFVDLTLHDQVH
LLECAWLEILMVGLIWRSVEHPGKLSFAPNLLLDRNQGRCVEGLVEIFDMLVTTATRFRMMRLRGEEFIC
LKSIILLNSGVYTFLSSTLESLEDTDLIHIILDKIIDTLVHFMAKSGLSLQQQQRRLAQLLLILSHIRHM
SNKGMEHLYSMKCKNVVPLYDLLLEMLDAHRIHTPKDKTTTQEEDSRSPPTTTVNGASPCLQPYYTNTEE
VSLQSTV

>Q6W5G6_XENLA
MSSANDKGPPSMESTKETRFCAVCSDYASGYHYGVWSCEGCKAFFKRSIQGHNDYMCPATNQCTIDKNRR
KSCQACRLRKCYEVGMMKGGIRKDRRGGRMLKHKQQKEEPEQKNDVNPSEIRTASIWVNPSVKSMKLSPV
LSLTAEQLISALMEAEPPIVYSEHDSTKPLSEASMMTLLTNLADKELVHMINWAKRVPGFVDLTLHDQVH
LLECAWLEILMVGLIWRSVEHPEKLSFAPNLLLDRNQGRCVEGLVEIFDMLVTTATRFRMMRLHGEEFIC
LKSIILLNSGVYTFLSSTLESLEDTDLIHIILDKIIDTLVHFMAKSGLSLQQQQRRLAQLLLILSHIRHM
SNKGMEHLYSMKCKNVVPLYDLLLEMLDAHRIHTPKDKTTTQEEESRSPLSTTVNGASPCLQPFYKNTEE
VSLQSTV

>ERR2_RAT
MSSEDRHLGSSCGSFIKTEPSSPSSGIDALSHHSPSGSSDASGGFGMALGTHANGLDSPPMFAGAGLGGN
PCRKSYEDCTSGIMEDSAIKCEYMLNAIPKRLCLVCGDIASGYHYGVASCEACKAFFKRTIQGNIEYSCP
ATNECEITKRRRKSCQACRFMKCLKVGMLKEGVRLDRVRGGRQKYKRRLDSENSPYLSLQISPPAKKPLT
KIVSYLLVAEPDKLYAMPPDDVPEGDIKALTTLCDLADRELVFLISWAKHIPGFSNLTLGDQMSLLQSAW
MEILILGIVYRSLPYDDKLAYAEDYIMDEEHSRLVGLLELYRAILQLVRRYKKLKVEKEEFVMLKALALA
NSDSMYIENLEAVQKLQDLLHEALQDYELSQRHEEPRRAGKLLLTLPLLRQTAAKAVQHFYSVKLQGKVP
MHKLFLEMLEAKV

>Q6Q6F4_BRARE
MSNKDRHIESSCPSYIKTEPSSPASLTDSVNHHSPGGSSDASGSYSSTMNGHQNGLDSPTLYGPTGALGP
SGTGAKRYEDCSSTITEDSQIKCEYMLNSMPKRLCLVCGDIASGYHYGVASCEACKAFFKRTIQGNIEYS
CPATNECEITKRRRKSCQACRFMKCLTVGMMREGVRLDRVRGGRQKYKRRIDAENSPYLNPQLALPPKKP
YNKIVSHLLVAEPEKIYAMPDPTVPDSDIKALTTLCDLADRELVVNIGWAKHIPGFSTLSLADQMSLLQS
AWMEILILRVVYRSLSFEDKLVYAEDYIMDEDQSKLAGLLDLNNAILQLVKKYKSMKLEKEEFVTLKAIA
LANSDSMHIEDVEAVQKLQDVLHEALQDYEAGQHVEDPRRAGKLLMTLPLLRQTSTKAVQHFYSIKQDGK
VPMHKLFLELLEAKV

>Q8C7A6_MOUSE
MSSEDRHLGSSCGSFIKTEPSSPSSGIDALSHHSPSGSSDASGGFGIALSTHANGLDSPPMFAGAGLGGN
PCRKSYEDCTSGIMEDSAIKCEYMLNAIPKRLCLVCGDIASGYHYGVASCEACKAFFKRTIQGNIEYNCP
ATNECEITKRRRKSCQACRFMKCLKVGMLKEGVRLDRVRGGRQKYKRRLDSENSPYLNLPISPPAKKPLT
KIVSNLLGVEQDKLYAMPPNDIPEGDIKALTTLCELADRELVFLINWAKHIPGFPSLTLGDQMSLLQSAW
MEILILGIVYRSLPYDDKLAYAEDYIMDEEHSRLVGLLDLYRAILQLVRRYKKLKVEKEEFMILKALALA
NSDSMYIENLEAVQKLQDLLHEALQDYELSQRHEEPRRAGKLLLTLPLLRQTAAKAVQHFYSVKLQGKVP
MHKLFLEMLEAKV

>ERR2_MOUSE
MSSEDRHLGSSCGSFIKTEPSSPSSGIDALSHHSPSGSSDASGGFGIALSTHANGLDSPPMFAGAGLGGN
PCRKSYEDCTSGIMEDSAIKCEYMLNAIPKRLCLVCGDIASGYHYGVASCEACKAFFKRTIQGNIEYNCP
ATNECEITKRRRKSCQACRFMKCLKVGMLKEGVRLDRVRGGRQKYKRRLDSENSPYLNLPISPPAKKPLT
KIVSNLLGVEQDKLYAMPPNDIPEGDIKALTTLCELADRELVFLINWAKHIPGFPSLTLGDQMSLLQSAW
MEILILGIVYRSLPYDDKLAYAEDYIMDEEHSRLVGLLDLYRAILQLVRRYKKLKVEKEEFMILKALALA
NSDSMYIENLEAVQKLQDLLHEALQDYELSQRHEEPRRAGKLLWTLPLLRQTAAKAVQHFYSVKLQGKVP

MHKLFLEMLEAKV

>Q8CCV5_MOUSE
MSSEDRHLGSSCGSFIKTEPSSPSSGIDALSHHSPSGSSDASGGFGIALSTHANGLDSPPMFAGAGLGGN
PCRKSYEDCTSGIMEDSAIKCEYMLNAIPKRLCLVCGDIASGYHYGVASCEACKAFFKRTIQGNIEYNCP
ATNECEITKRRRKSCQACRFMKCLKVGMLKEGVRLDRVRGGRQKYKRRLDSENSPYLNLPISPPAKKPLT
KIVSNLLGVEQDKLYAMPPNDIPEGDIKALTTLCELADRGLVFLINWAKHIPGFPSLTLGDQMSLLQSAW
MEILILGIVYRSLPYDDKLAYAEDYIMDEEHSRLVGLLDLYRAILQLVRRYKKLKVEKEEFMILKALALA
NSDSMYIENLEAVQKLQDLLHEALQDYELSQRHEEPRRAGKLLLTLPLLRQTAAKAVQHFYSVKLQGKVP
MHKLFLEMLEAKV

>Q6Q6F6_BRARE
MSSRERRSDLYIKAEPSSPEGGGGGGGGRTSPGGASSDSSQSGGGGSRGEGAGRYSPPLYTPALRCHFKE
EGADGAEEGSTGSGGGRCKYALSTLPKRLCLVCGDVASGYHYGVASCEACKAFFKRTIQGNIEYSCPASN
ECEITKRRRKACQACRFTKCLKVGMLKEGVRLDRVRGGRQKYKRRPEVENATYQSAPIPLRKEGEKGSSS
IIVSHLLVAEPEKLFAMPDPLQPDTAQRTLTTLCDLADRELVVIIGWAKHIPGFLSLSLADQMSVLQSVW
LEVLVLGVAYRSLGCEDEVVFAEDFVLDEEMSRVAGLTELNAAISQLARRFRALQLDREEFVMLKAIALT
NSDSVYIEDMEAVQKLRDLLHQALLELEVQRRPDDPQRAGRLLLTLPLLRQTAGRALTTFYSIKTRGGVP
MHKLFLEMLEAMMDSP

>Q6QMY5_CANFA
MSSQVVGIEPLYIKAEPASPDSPKGSSETETEPPVALAPGPAPTRCLPGHKEEEDGEGAGPGEQGGGKLV
LSSLPKRLCLVCGDVASGYHYGVASCEACKAFFKRTIQGSIEYSCPASNECEITKRRRKACQACRFTKCL
RVGMLKEGVRLDRVRGGRQKYKRRPEVDPLPFPGSFPAGPLAVAGGPRKTAPVNALVSHLLVVEPEKLYA
MPDPAGPDGHLPAVATLCDLFDREIVVTISWAKSIPGFSSLSLSDQMSVLQSVWMEVLVLGVAQRSLPLQ
DELAFAEDLVLDEEGARAAGLGELGAVLLQLVRRLQALRLEREEYVLLKALALANSDSVHIEDAEAVEQL
REALHEALLEYEAGRAGPGGGAERRRAGRLLLTLPLLRQTAGKVLAHFYGVKLEGKVPMHKLFLEMLEAM
MD

>Q9VSE9_DROME
MSDGVSILHIKQEVDTPSASCFSPSSKSTATQSGTNGLKSSPSVSPERQLCSSTTSLSCDLHNVSLSNDG
DSLKGSGTSGGNGGGGGGGTSGGNATNASAGAGSGSVRDELRRLCLVCGDVASGFHYGVASCEACKAFFK
RTIQGNIEYTCPANNECEINKRRRKACQACRFQKCLLMGMLKEGVRLDRVRGGRQKYRRNPVSNSYQTMQ
LLYQSNTTSLCDVKILEVLNSYEPDALSVQTPPPQVHTTSITNDEASSSSGSIKLESSVVTPNGTCIFQN
NNNNDPNEILSVLSDIYDKELVSVIGWAKQIPGFIDLPLNDQMKLLQVSWAEILTLQLTFRSLPFNGKLC
FATDVWMDEHLAKECGYTEFYYHCVQIAQRMERISPRREEYYLLKALLLANCDILLDDQSSLRAFRDTIL
NSLNDVVYLLRHSSAVSHQQQLLLLLPSLRQADDILRRFWRGIARDEVITMKKLFLEMLEPLAR

>Q6AX97_XENLA
MSSRDRRPDLCIKAEPGTPESIGRRSPSGSSDSSGHGPDPPGPRCCRDEEQDDVSGRGKYVLNSIPKRLC
LVCGDVASGYHYGVASCEACKAFFKRTIQGNIEYSCPASNECEITKRRRKACQACRFTKCLRVGMLKEGV
RLDRVRGGRQKYKRRPDGDILQYSSGGQAQQASATVVKKQTPVNAVVSHLLVAEPDKLFAMPDPALPDGD
LKYMSTLCDLADREIVIIISWAKNIPGFSSLSLSDQMSLLQSVWMEVLLLGVVFRSLPYEDEVVFAEDFV
LDEESSRSGRLTDLCSCVLHLVRKYRTLRVEKEEYVMLKALTLTNSDSVHIEDPDAVLRLRDALQEALSE

YESGRHPEEPCRDGKLLLTLPLLRQTAGRVLQHFHALREEGAVPMHKLFLEMLEAMMD

(4) 23 NR4

>NR4A1_HUMAN
MPCIQAQYGTPAPSPGPRDHLASDPLTPEFIKPTMDLASPEAAPAAPTALPSFSTFMDGYTGEFDTFLYQ
LPGTVQPCSSASSSASSTSSSSATSPASASFKFEDFQVYGCYPGPLSGPVDEALSSSGSDYYGSPCSAPS
PSTPSFQPPQLSPWDGSFGHFSPSQTYEGLRAWTEQLPKASGPPQPPAFFSFSPPTGPSPSLAQSPLKLF
PSQATHQLGEGESYSMPTAFPGLAPTSPHLEGSGILDTPVTSTKARSGAPGGSEGRCAVCGDNASCQHYG
VRTCEGCKGFFKRTVQKNAKYICLANKDCPVDKRRRNRCQFCRFQKCLAVGMVKEVVRTDSLKGRRGRLP
SKPKQPPDASPANLLTSLVRAHLDSGPSTAKLDYSKFQELVLPHFGKEDAGDVQQFYDLLSGSLEVIRKW
AEKIPGFAELSPADQDLLLESAFLELFILRLAYRSKPGEGKLIFCSGLVLHRLQCARGFGDWIDSILAFS
RSLHSLLVDVPAFACLSALVLITDRHGLQEPRRVEELQNRIASCLKEHVAAVAGEPQPASCLSRLLGKLP
ELRTLCTQGLQRIFYLKLEDLVPPPPIIDKIFMDTLPF

>Q6IBU8_HUMAN
MPCIQAQYGTPAPSPGPRDHLASDPLTPEFIKPTMDLASPEAAPAAPTALPSFSTFMDGYTGEFDTFLYQ
LPGTVQPCSSASSSASSTSSSSATSPASASFKFEDFQVYGCYPGPLSGPVDEALSSSGSDYYGSPCSAPS
PSTPSFQPPQLSPWDGSFGHFSPSQTYEGLRAWTEQLPKASGPPQPPAFFSFSPPTGPSPSLAQSPLKLF
PSQATHQLGEGESYSMPTAFPGLAPTSPHLEGSGILDTPVTSTKARSGAPGGSEGRCAVCGDNASCQHYG
VRTCEGCKGFFKRTVQKNAKYICLANKDCPVDKRRRNRCQFCRFQKCLAVGMVKEVVRTDSLKGRRGRLP
SKPKQPPDAFPANLLTSLVRAHLDSGPSTAKLDYSKFQELVLPHFGKEDAGDVQQFYDLLSGSLEVIRKW
AEKIPGFAELSPADQDLLLESAFLELFILRLAYRSKPGEGKLIFCSGLVLHRLQCARGFGDWIDSILAFS
RSLHSLLVDVPAFACLSALVLITDRHGLQEPRRVEELQNRIASCLKEHVAAVAGEPQPASCLSRLLGKLP
ELRTLCTQGLQRIFYLKLEDLVPPPPIIDKIFMDTLPF

>Q8N3V2_HUMAN
MPCIQAQYGTPAPSPGPRDHLASDPLTPEFIKPTMDLASPEAAPAAPTALPSFSTFMDGYTGEFDTFLYQ
LPGTVQPCSSASSSASSTSSSSATSPASASFKFEDFQVYGCYPGPRSGPVDEALSSSGSDYYGSPCSAPS
PSTPSFQPPQLSPWDGSFGHSSPSQTYEGLRAWTEQLPKASGPPQPPAFFSFSPPTGPSPSLAQSPLKLF
PSQATHQLGEGESYSMPTAFPGLAPTSPHLEGSGILDTPVTSTKARSGAPGGSEGRCAVCGDNASCQHYG
VRTCEGCKGFFKRTVQKNAKYICLANKDCPVDKRRRNRCQFCRFQKCLAVGMVKEVVRTDSLKGRRGRLP
SKPKQPPDASPANLLTSLVRAHLDSGPSTAKLDYSKFQELVLPHFGKEDAGDVQQFYDLLSGSLEVIRKW
AEKIPGFAELSPADQDLLLESAFLELFILRLAYRSKPGEGKLIFCSGLVLHRLQCARGFGDWIDSILAFS
RSLHSLLVDVPAFACLSALVLITDRHGLQEPRRVEELQNRIASCLKEHVAAVAGEPQPASCLSRLLGKLP
ELRTLCTQGLQRIFYLKLEDLVPPPPIIDKIFMDTLPF

>Q5RBB0_PONPY
MPCIQAQYGTPAPSPGPRDHLASDPLTPEFIKPTMDLASPEAAPAAPTALPSFSTFMDGYTGEFDTFLYQ
LPGTVQPCSSASSSASSTSSSSATSPASASFKFEDFQVYGCYPGPLSGPVDEALSSSGSDYYGSPCSAPS
PSTPSFQPPQLSPWDGSFGHFSPSQTYEGLRAWTEQLPKASGPPQPPAFFSFSPPTGPSPSLAQSPLKLF
PSQATHQLGEGESYSMPTAFPGLASTSPHLEGSGILDAPVTSTKARSGAPGGSEGRCAVCGDNASCQHYG
VRTCEGCKGFFKRTVQKNAKYICLANKDCPVDKRRRNRCQFCRFQKCLAVGMVKEVVRTDSLRGRRGRLP
SKPKQPPDASPANLLTSLVRAHLDSGPSTAKLDYSKFQELVLPRFGKEDAGDVQQFYDLLSGSLEVIRKW
AEKIPGFAELSPADQDLLLESAFLELFILRLAYRSKPGEGKLIFCSGLVLHRLQCARGFGDWIDSILAFS

RSLHSLVVDVPAFACLSALVLITDRHGLQEPRRVEELQNRIASCLKEHVAAVAGEPQPASCLSRLLGKLP
ELRTLCTQGLQRIFYLKLEDLVPPPPIIDKIFMDTLPF

>NR4A1_CANFA
MPCIQAQYGTPAPSPGPRDHLASDPLTPELSKPTMDLASPEAAPTAPTALPSFSTFMDGYTGEFDTFLYQ
LPGTAQPCSSASSSASSTSSSSATSPASASFKFEDFQVYGCYPGPLSGPLDETLSSSGSDYYGSPCSAPS
PSTPSFQPPQLSPWDGSFGPFSPSQTYEGLRAWTEQLPKASGHPQPPAFFSFSPPTGPSPSLAQSPLKLF
PSQATCQLGERESYSISTAFPGLAPTSPHLDGPGMLDAPVPSAKARSGAPSGSEGRCAVCGDNASCQHYG
VRTCEGCKGFFKRTVQKNAKYICLANKDCPVDKRRRNRCQFCRFQKCLAVGMVKEVVRTDSLKGRRGRLP
SKPKQPPDASPANLLTSLVRAHLDSGPSTAKLDYSKFQELVLPHFGKEDAGDVQQFYDLLSGSLEVIRKW
AEKIPGFAELSPGDQDLLLESAFLELFILRLAYRSKPAEGKLIFCSGLVLHRLQCARGFGDWIDSILAFS
RSLHGLVVDVPAFACLSALVLITDRHGLQEPRRVEELQNRIASCLKEHVSAVAGEPQPASCLSRLLGKLP
ELRTLCTQGLQRIFYLKLEDLVPPPPIVDKIFMDTLPF

>NR4A1_MOUSE
MPCIQAQYGTPATSPGPRDHLTGDPLALEFGKPTMDLASPETAPAAPATLPSFSTFMDGYTGEFDTFLYQ
LPGTTQPCSSACSSASSTSSSSSSATSPASASFKFEDFQVYGCYPGTLSGPLDETLSSSGSEYYGSPCSA
PSPSTPNFQPSQLSPWDGSFGHFSPSQTYEGLWAWTEQLPKASSGPPPPPTFFSFSPPTGPSPSLAQSSL
KLFPPPATHQLGEGESYSMPAAFPGLAPTSPNRDTSGILDAPVTSTKSRSGASGGSEGRCAVCGDNASCQ
HYGVRTCEGCKGFFKRTVQKSAKYICLANKDCPVDKRRRNRCQFCRFQKCLAVGMVKEVVRTDSLKGRRG
RLPSKPKQPPDASPTNLLTSLIRAHLDSGPSTAKLDYSKFQELVLPRFGKEDAGDVQQFYDLLSGSLDVI
RKWAEKIPGFIELCPGDQDLLLESAFLELFILRLAYRSKPGEGKLIFCSGLVLHQLQCARGFGDWIDNIL
AFSRSLHSLGVDVPAFACLSALVLITDRHGLQDPRRVEELQNRIASCLKEHMATVAGDPQPASCLSRLLG
KLPELRTLCTQGLQRIFCLKLEDLVPPPPIVDKIFMDTLSF

>NR4A2_MOUSE
MPCVQAQYGSSPQGASPASQSYSYHSSGEYSSDFLTPEFVKFSMDLTNTEITATTSLPSFSTFMDNYSTG
YDVKPPCLYQMPLSGQQSSIKVEDIQMHNYQQHSHLPPQSEEMMPHSGSVYYKPSSPPTPSTPSFQVQHS
PMWDDPGSLHNFHQNYVATTHMIEQRKTPVSRLSLFSFKQSPPGTPVSSCQMRFDGPLHVPMNPEPAGSH
HVVDGQTFAVPNPIRKPASMGFPGLQIGHASQLLDTQVPSPPSRGSPSNEGLCAVCGDNAACQHYGVRTC
EGCKGFFKRTVQKNAKYVCLANKNCPVDKRRRNRCQYCRFQKCLAVGMVKEVVRTDSLKGRRGRLPSKPK
SPQDPSPPSPPVSLISALVRAHVDSNPAMTSLDYSRFQANPDYQMSGDDTQHIQQFYDLLTGSMEIIRGW
AEKIPGFADLPKADQDLLFESAFLELFVLRLAYRSNPVEGKLIFCNGVVLHRLQCVRGFGEWIDSIVEFS
SNLQNMNIDISAFSCIAALAMVTERHGLKEPKRVEELQNKIVNCLKDHVTFNNGGLNRPNYLSKLLGKLP
ELRTLCTQGLQRIFYLKLEDLVPPPAIIDKLFLDTLPF

>NR4A2_HUMAN
MPCVQAQYGSSPQGASPASQSYSYHSSGEYSSDFLTPEFVKFSMDLTNTEITATTSLPSFSTFMDNYSTG
YDVKPPCLYQMPLSGQQSSIKVEDIQMHNYQQHSHLPPQSEEMMPHSGSVYYKPSSPPTPTTPGFQVQHS
PMWDDPGSLHNFHQNYVATTHMIEQRKTPVSRLSLFSFKQSPPGTPVSSCQMRFDGPLHVPMNPEPAGSH
HVVDGQTFAVPNPIRKPASMGFPGLQIGHASQLLDTQVPSPPSRGSPSNEGLCAVCGDNAACQHYGVRTC
EGCKGFFKRTVQKNAKYVCLANKNCPVDKRRRNRCQYCRFQKCLAVGMVKEVVRTDSLKGRRGRLPSKPK
SPQEPSPPSPPVSLISALVRAHVDSNPAMTSLDYSRFQANPDYQMSGDDTQHIQQFYDLLTGSMEIIRGW
AEKIPGFADLPKADQDLLFESAFLELFVLRLAYRSNPVEGKLIFCNGVVLHRLQCVRGFGEWIDSIVEFS

SNLQNMNIDISAFSCIAALAMVTERHGLKEPKRVEELQNKIVNCLKDHVTFNNGGLNRPNYLSKLLGKLP
ELRTLCTQGLQRIFYLKLEDLVPPPAIIDKLFLDTLPF

>NR4A2_RAT
MPCVQAQYGSSPQGASPASQSYSYHSSGEYSSDFLTPEFVKFSMDLTNTEITATTSLPSFSTFMDNYSTG
YDVKPPCLYQMPLSGQQSSIKVEDIQMHNYQQHSHLPPQSEEMMPHSGSVYYKPSSPPTPSTPGFQVQHS
PMWDDPGSLHNFHQNYVATTHMIEQRKTPVSRLSLFSFKQSRPGTPVSSCQMRFDGPLHVPMNPEPAGSH
HVVDGQTFAVPNPIRKPASMGFPGLQIGHASQLLDTQVPSPPSRGSPSNEGLCAVCGDNAACQHYGVRTC
EGCKGFFKRTVQKNAKYVCLANKNCPVDKRRRNRCQYCRFQKCLAVGMVKEVVRTDSLKGRRGRLPSKPK
SPQDPSPPSPPVSLISALVRAHVDSNPAMTSLDYSRFQANPDYQMSGDDTQHIQQFYDLLTGSMEIIRGW
AEKIPGFADLPKADQDLLFESAFLELFVLRLAYRSNPVEGKLIFCNGVVLHRLQCVRGFGEWIDSIVEFS
SNLQNMNIDISAFSCIAALAMVTERHGLKEPKRVEELQNKIVNCLKDHVTFNNGGLNRPNYLSKLLGKLP
ELRTLCTQGLQRIFYLKLEDLVPPPAIIDKLFLDTLPF

>Q5R5Y4_PONPY
MPCVQAQYGSSPQGASPASQGYSYHSSGEYSSDFLTPEFVKFSMDLTNTEITATTSLPSFSTFMDNYSTG
YDVKPPCLYQMPLSGQQSSIKVEDIQMHNYQQHSHLPPQSEEMMPHSGSVYYKPSSPPTPTTPGFQVQHS
PMWDDPGSLHNFHQNYVATTHMIEQRKTPVSRLSLFSFKQSPPGTPVSSCQMRFDGPLHVPMNPEPAGSH
HVVDGQTFAVPNPIRKPASMGFPGLQIGHASQLLDTQVPSPPSRGSPSNEGLCAVCGDNAACQHYGVRTC
EGCKGFFKRTVQKNAKYVCLANKNCPVDKRRRNRCQYCRFQKCLAVGMVKEVVRTDSLKGRRGRLPSKPK
SPQEPSPPSPPVSLISALVRAHVDSNPAMTSLDYSRFQANPDYQMSGDDTQHIQQFYDLLTGSMEIIRGW
AEKIPGFADLPKADQDLLFESAFLELFVLRLAYRSNPVEGKLIFCNGVVLHRLQCVRGFGEWIDSIVEFS
SNLQNMNIDISAFSCIAALAMVTERHWLKEPKRVEELQNKIVNCLKDHVTFNNGGLNRPNYLSKLLGKLP
ELRTLCTQGLQRIFYLKLEDLVPPPAIIDKLFLDTLPF

>NR4A1_RAT
MPCIQAQYGTPATSPGPRDHLTGDPLALEFSKPTMDLASPETAPTAPATLPSFSTFMDGGYTGEFDTFLY
QLPGTAQPCSSASSTSSSSSSATSPASASFKFEDFQVYGCYPGTLSGPLDETLSSSGSDYYGSPCSAPSP
PTPNFQPSQLSPWDGSFGHFSPSQTYEGLRVWTEQLPKASGPPPPPTFFSFSPPTGPSPSLAQSSLKLFP
APATHQLGEGESYSVPAAFPGLAPTSPNCDTSGILDAPVTSTKARSGSSGGSEGRCAVCGDNASCQHYGV
RTCEGCKGFFKRTVQKSAKYICLANKDCPVDKRRRNRCQFCRFQKCLAVGMVKEVVRTDSLKGRRGRLPS
KPKQPPDASPTNLLTSLIRAHLDSGPNTAKLDYSKFQELVLPRFGKEDAGDVQQFYDLLSGSLDVIRKWA
EKIPGFIELSPGDQDLLLESAFLELFILRLAYRSKPGEGKLIFCSGLVLHRLQCARGFGDWIDNILAFSR
SLHSLGVDVPAFACLSALVLITDRHGLQDPRRVEELQNRIASCLKEHMAAVAGDPQPASCLSRLLGKLPE
LRTLCTQGLQRIFCLKLEDLVPPPPIVDKIFMDTLSF

>NR4A3_MOUSE
MPCVQAQYSPSPPGSTYATQTYGSEYTTEIMNPDYTKLTMDLGSTGIMATATTSLPSFSTFMEGYPSSCE
LKPSCLYQMPPSGPRPLIKMEEGREHGYHHHHHHHHHHHHHQQQQPSIPPPSGPEDEVLPSTSMYFKQSP
PSTPTTPGFPPQAGALWDDELPSAPGCIAPGPLLDPQMKAVPPMAAAARFPIFFKPSPPHPPAPSPAGGH
HLGYDPTAAAALSLPLGAAAAAGSQAAALEGHPYGLPLAKRTATLTFPPLGLTASPTASSLLGESPSLPS
PPNRSSSSGEGTCAVCGDNAACQHYGVRTCEGCKGFFKRTVQKNAKYVCLANKNCPVDKRRRNRCQYCRF
QKCLSVGMVKEVVRTDSLKGRRGRLPSKPKSPLQQEPSQPSPPSPPICMMNALVRALTDATPRDLDYSRY
CPTDQATAGTDAEHVQQFYNLLTASIDVSRSWAEKIPGFTDLPKEDQTLLIESAFLELFVLRLSIRSNTA

EDKFVFCNGLVLHRLQCLRGFGEWLDSIKDFSLNLQSLNLDIQALACLSALSMITERHGLKEPKRVEELC
TKITSSLKDHQRKGQALEPSEPKVLRALVELRKICTQGLQRIFYLKLEDLVPPPSVIDKLFLDTLPF

>NR4A3_RAT
MPCVQAQYSPSPPGSTYATQTYGSEYTTEIMNPDYAKLTMDLGSTGIMATATTSLPSFSTFMEGYPSSCE
LKPSCLYQMPPSGPRPLIKMEEGREHGYHHHHHHHHHHHHHHQQQQPSIPPPSGPEDEVLPSTSMYFKQS
PPSTPTTPGFPPQAGALWDDELPSAPGCIAPGPLLDPQMKAVPPMAAAARFPIFFKPSPPHPPAPSPAGG
HHLGYDPTAAAALSLPLGAAAAAGSQAAALEGHPYGLPLAKRTATLTFPPLGLTASPTASSLLGESPSLP
SPPNRSSSSGEGTCAVCGDNAACQHYGVRTCEGCKGFFKRTVQKNAKYVCLANKNCPVDKRRRNRCQYCR
FQKCLSVGMVKEVVRTDSLKGRRGRLPSKPKSPLQQEPSQPSPPSPPICMMNALVRALTDATPRDLDYSR
YCPTDQATAGTDAEHVQQFYNLLTASIDVSRSWAEKIPGFTDLPKEDQTLLIESAFLELFVLRLSIRSNT
AEDKFVFCNGLVLHRLQCLRGFGEWLDSIKDFSLNLQSLNLDIQALACLSALSMITERHGLKEPKRVEEL
CNKITSSLKDHQRKGQALEPSEPKVLRALVELRKICTQGLQRIFYLKLEDLVSPPSVIDKLFLDTLPF

>Q6NXU0_HUMAN
MDNYSTGYDVKPPCLYQMPLSGQQSSIKVEDIQMHNYQQHSHLPPQSEEMMPHSGSVYYKPSSPPTPTTP
GFQVQHSPMWDDPGSLHNFHQNYVATTHMIEQRKTPVSRLSLFSFKQSPPGTPVSSCQMRFDGPLHVPMN
PEPAGSHHVVDGQTFAVPNPIRKPASMGFPGLQIGHASQLLDTQVPSPPSRGSPSNEGLCAVCGDNAACQ
HYGVRTCEGCKGFFKRTVQKNAKYVCLANKNCPVDKRRRNRCQYCRFQKCLAVGMVKEVVRTDSLKGRRG
RLPSKPKSPQEPSPPSPPVSLISALVRAHVDSNPAMTSLDYSRFQANPDYQMSGDDTQHIQQFYDLLTGS
MEIIRGWAEKIPGFADLPKADQDLLFESAFLELFVLRLAYRSNPVEGKLIFCNGVVLHRLQCVRGFGEWI
DSIVEFSSNLQNMNIDISAFSCIAALAMVTERHGLKEPKRVEELQNKIVNCLKDHVTFNNGGLNRPNYLS
KLLGKLPELRTLCTQGLQRIFYLKLEDLVPPPAIIDKLFLDTLPF

>NR4A3_HUMAN
MPCVQAQYSPSPPGSSYAAQTYSSEYTTEIMNPDYTKLTMDLGSTEITATATTSLPSISTFVEGYSSNYE
LKPSCVYQMQRPLIKVEEGRAPSYHHHHHHHHHHHHHHQQQHQQPSIPPASSPEDEVLPSTSMYFKQSPP
STPTTPAFPPQAGALWDEALPSAPGCIAPGPLLDPPMKAVPTVAGARFPLFHFKPSPPHPPAPSPAGGHH
LGYDPTAAAALSLPLGAAAAAGSQAAALEGHPYGLPLAKRAAPLAFPPLGLTPSPTASSLLGESPSLPSP
PSRSSSSGEGTCAVCGDNAACQHYGVRTCEGCKGFFKRTVQKNAKYVCLANKNCPVDKRRRNRCQYCRFQ
KCLSVGMVKEVVRTDSLKGRRGRLPSKPKSPLQQEPSQPSPPSPPICMMNALVRALTDSTPRDLDYSRYC
PTDQAAAGTDAEHVQQFYNLLTASIDVSRSWAEKIPGFTDLPKEDQTLLIESAFLELFVLRLSIRSNTAE
DKFVFCNGLVLHRLQCLRGFGEWLDSIKDFSLNLQSLNLDIQALACLSALSMITERHGLKEPKRVEELCN
KITSSLKDHQSKGQALEPTESKVLGALVELRKICTLGLQRIFYLKLEDLVSPPSIIDKLFLDTLPF

>NR4A2_XENLA
MPCIQAQHGSLSQCAGPCDNYVPDILNSEFGKFTMDLVNSEIAASTSLPSFSTFMDGYTGEFDAFLYQIP
SSNQQSSLKVEEFQVFGCYPGSFTNQLDETMSSSGSDYYGSPCSIPSPSTPGFQNPQLPTWECSYGAYSP
TQNYDNMRHWTEQQKNSISQQTFFSFGTPAHSPNMAANPLKIAPATHRLDQQLVDTDVFALAQNSSAGFP
AVPLGQAPGVLDSSVLLDSPLSPSKTRSPSSNEGRCAVCGDNASCQHYGVRTCEGCKGFFKRTVQKNAKY
ICLANKDCPVDKRRRNRCQFCRFQKCLVVGMVKEVVRTDSLKGRRGRLPSKPKQIAESSPVDLINSLVRA
HIDSIPSSSKLDYSKFQETVPLQLEKESSVDVQQFYDLLSGSLEVIRKWAEKIQGFVDLPKEDQDLLLES
AFLELFILRLAYRSRPEEGKLIFCNGVVLHRTQCVRGFGEWIDSIIEFSHSLQRMNIDVPSFSCLSALVI
VTDRHGLKEPKKVEELQSQIINCLKEHIPSSMNEQNRPNCLSKLLGKLPELRTLCTQGLQRIFYLKLEDL

VPPPPIVDKIFMDTLPF

>Q7T0V3_XENLA
MPCIQAQYGSLSQCAGPCDNYVPDILNSEFGKFTMDLVNSEIAASTSLPSFSTFMDGYTGEFDAFLYQIP
SSNQQSSLKVEEFQVFGCYPGSFTNQVDETMSSSGSDYYGSPCSIPSPSTPGFQNPQLPTWECSYGAYSP
NQNYDNMRHWTEQQKNSISQQTFFSFGTPAHSPNVAANPLKMAPATHRLDQQIVDADVFALAHNSSSGFP
AIPLGQAHGVLDSSVLLDSPLSPSKTLSPSSNEGRCAVCGDNASCQHYGVRTCEGCKGFFKRTVQKNAKY
ICLANKDCPVDKRRRNRCQFCRFQKCLAVGMVKEVVRTDNLKGRRGRLPSKPKQIAESSPVSLINSLVRA
HIDSIPSSSKLDYSKFQETVPVQLEKESSVDVQQFYDLLSGSLEVIRKWAEKIQGFVDLPKEDQDLLLES
AFLELFILRLAYRSRPEEEKLIFCNGVVLHRTQCVRGFGEWIDSIIEFSHNLQRTNIDVPSFSCLSALVI
ITDRHGLKEPKKVEELQSRIINCLKEHAPSSMNEQNRPNCLSKLLGKLPELRTLCTQGLQRIFYLKLEDL
VPPPPIVDKIFMDTLPF

>Q6DH08_BRARE
MPCVQAQYGTSPPGASPASQSYSYNTTGEYNCDFLTPEFVKFSMDLTNAEIAVTSSLPSFSTFVDTYSSN
YDVKPPCLYQMAHSGDQLSIKVEEIPAHGYHQQHHQPHQAEESIPHTGAIYYKPSSPNISQSPSYPTAP
HHTWEDTGSLHSFHQNYLATSHIMDQQRKNAMSRLFSFKQSPVDTPMPSCQMRFDGSLHVSMGPDTAHRA
LDSFALPAPPRKQHGVGLSHSLNVGHPLLESPVASPQARGSPSSEGLCAVCGDNAACQHYGVRTCEGCKG
FFKRTVQKNAKYVCLANKNCPVDKRRRNRCQYCRFQKCLVVGMVKEVVRTASLKGRRGRLPSKPKSPQDI
PVSFTPVNLLNALVRAHIDSNPSMARLDYSKFQTSPEYHSGGDESLHIQQFYDLLTASMSIIRGWAEKIP
GFTELPKCDQELLFESAFLELFVLRLAYRSNLPEDKLIFCNGMVLHKLQCVRGFGEWIDSIVEFSSNLQS
MSLDVSAFSCIAALTIVTERHGLKEPKKAEELQNKLINCLKDQVSCSGELSKLLEKLPEVRALCTQGLQR
IFYLKLEDLVPTPAIIDKLFHDTLPF

>Q6GMG3_BRARE
MTCVQSQHGVQSYESSLFSSEFLNPDFTSRLAMDVTEQREQLSAPSLPSITSLVGGYVGEFDTYSCQIAT
GAASTCVGSTSGQLDSFKLDDLQVYGCYPGTFALSYLDETLSSSGGSNCFGSPASAPSPSTPGYQHMSAW
DSTFGPCSPDEGCWGSEKPLNQAASFFTFGHVTADELSPLGQMQSQIPDQDPFSHTPQHGSPLPFSPLSL
EQGSRDGSGLMDGHLSPKVRSPTGNEGRCAVCGDNASCQHYGVRTCEGCKGFFKRTVQKNAKYVCLANKD
CPVDKRRRNRCQFCRFQKCLAVGMVKEVVRTDSLKGRRGRLPSKPKAVQDSVSVSSPVNIIASLVTAHID
SNPASANLDYSKYQESTSGLPEKEDANDIQQFYDLLTGSMDVIRKWAGSIPGFSAFCKEDQELLLESAFV
ELFILRLAYRSNPETDKLIFCNGVVLHRTQCVRSFGDWIDSIMEFSQSLHRLNLDISAFSCLATLVIITD
RHGLKEPKRIEDFQNRLITCLRDHVSTSALEVGRPNSLSRLLSKLPELRTLCTQGLQRIFYLKLEDLVPP
PPIVDKIFMDTLPF

>Q8INU7_DROME
MDEDCFPPLSGGWSASPPAPSQLQQLHTLQSQAQMSHPNSSNNSSNNAGNSHNNSGGYNYHGHFNAINAS
ANLSPSSSASSLYEYNGVSAADNFYGQQQQQQQQSYQQHNYNSHNGERYSLPTFPTISELAAATAAVEAA
AAATVSSPSVGGPPPVRRASLPVQRTVSPAGSTAQSPKLAKITLNQRHSHAHAHALQLNSAPNSAASSPA
SADLQAGRLLQAPSQLCAVCGDTAACQHYGVRTCEGCKGFFKRTVQKGSKYVCLADKNCPVDKRRRNRCQ
FCRFQKCLVVGMVKEVVRTDSLKGRRGRLPSKPKSPQESPPSPPISLITALVRSHVDTTPDPSCLDYSHY
EEQSMSEADKVQQFYQLLTSSVDVIKQFAEKIPGYFDLLPEDQELLFQSASLELFVLRLAYRARIDDTKL
IFCNGTVLHRTQCLRSFGEWLNDIMEFSRSLHNLEIDISAFACLCALTLITERHGLREPKKVEQLQMKII
GSLRDHVTYNAEAQKKQHYFSRLLGKLPELRSLSVQGLQRIFYLKLEDLVPAPALIENMFVTTLPF

>Q9R1W4_MOUSE
MDNYSTGYDVKPPCLYQMPLSGQQSSIKVEDIQMHNYQQHSHLPPQSEEMMPHSGSVYYKPSSPPTPSTP
SFQVQHSPMWDDPGSLHNFHQNYVATTHMIEQRKTPVSRLSLFSFKQSPPGTPVSSCQMRFDGPLHVPMN
PEPAGSHHVVDGQTFAVPNPIRKPASMGFPGLQIGHASQLLDTQVPSPPSRGSPSNEGLCAVCGDNAACQ
HYGVRTCEGCKGFFKRTVQKNAKYVCLANKNCPVDKRRRNRCQYCRFQKCLAVGMVKEVVRTDSLKGRRG
RLPSKPKSPQDPSPPSPPVSLISALVRAHVDSNPAMTSLDYSRFQANPDYQMSGDDTQHIQQFYDLLTGS
MEIIRGWAEKIPGFADLPKADQDLLFESAFLELFVLRLAYRI

>O97727_PIG
MPCVQAQYSPSPPGSSYAAQTYGSEYTTEIMNPDYTKLTMDLGSTEITATATTSLPSFSTFMEGYSSNYE
LKPSCLYQMQPSGPRPLIKMEEGRAHGYHHHHHDHHHHHHHQQQQQQQQPPPQQQQPSIPPPSGPEDEVL
PSTSMYFKQSPPSTPTTPVFPQQAGALWEDALPSAQGCIAPGPLLDPPMKAVPTVAGARFPLFHFKPSPP
HPPAPSPAGGHHLGYDPTAAAALGLPLGAAAAAAAAAAGSQAAALEGHPYGLPLAKRAAALAFSPLGLTT
SPTTSSLLGESPSLPSPPNRSTASGEGTCAVCGDNAACQHYGVRTCEGCKGFFKRTVQKNAKYVCLANKN
CPVDKRRRNRCQYCRFQKCLSVGMVKEVVRTDSLKGRRGRLPSKPKSPLQQEPSQPSPPSPPVCMMNALV
RALTDSTPRDLDYSRGHACGIWKFPG

>Q61JK3_CAEBR
MMSKRTASKLEKPSTLAALEEQLRASGVIINTEFASLQDTSDQANVVPKPEVKVKVETIPEEFEQPSSSA
PSSSNQRALPSQSQLQPSVAPNPEMNASIAHIKSELDPMQAFQMPPNDLIFGAPHYPFSLTHDFMGAPNA
LMPPFTSPFYPQHFPVPAEARRNSQGNTSSSNNTGGTPSPHSNSLPTSPPQLHGFLRSFLNHDNLQTPPT
PFGIQDEVLDADKMCAVCNDRAVCLHYGARTCEGCKGFFKRTVQKNSKYTCAGNKNCPIDKRYRSRCQYC
RFQKCLEVGMVKEIVRGGSLSGRRGRLSSKTKLARSVDQPSPPLPLLALMGKAIEDNTNMTVTRHFLAPF
GEDSAVSILHCEYIATRKLLMAMPQICEIPEPDFRILLARSFFPIMAIRCANRCANNTESIMFESGELFM
LNAFPICFQQILRFMINKAQNFSSLVEWEPQAFASFIALQFLSGNTEQNLLGLSNKVLVDTVQSTIINAL
KDHCSGSQNKLAKIVRLTDEFDVFHTMGIQVLDSMYPSTRFPEEFQHLISLTRAPLRTADTQPACGSPVV
PSTSALFAFQMGSTAF

(5) 33 NR5

>NR5A2_HUMAN
MSSNSDTGDLQESLKHGLTPIGAGLPDRHGSPIPARGRLVMLPKVETEALGLARSHGEQGQMPENMQVSQ
FKMVNYSYDEDLEELCPVCGDKVSGYHYGLLTCESCKGFFKRTVQNNKRYTCIENQNCQIDKTQRKRCPY
CRFQKCLSVGMKLEAVRADRMRGGRNKFGPMYKRDRALKQQKKALIRANGLKLEAMSQVIQAMPSDLTIS
SAIQNIHSASKGLPLNHAALPPTDYDRSPFVTSPISMTMPPHGSLQGYQTYGHFPSRAIKSEYPDPYTSS
PESIMGYSYMDSYQTSSPASIPHLILELLKCEPDEPQVQAKIMAYLQQEQANRSKHEKLSTFGLMCKMAD
QTLFSIVEWARSSIFFRELKVDDQMKLLQNCWSELLILDHIYRQVVHGKEGSIFLVTGQQVDYSIIASQA
GATLNNLMSHAQELVAKLRSLQFDQREFVCLKFLVLFSLDVKNLENFQLVEGVQEQVNAALLDYTMCNYP
QQTEKFGQLLLRLPEIRAISMQAEEYLYYKHLNGDVPYNNLLIEMLHAKRA

>Q9UEC0_HUMAN
MSSNSDTGDLQESLKHGLTPIGAGLPDRHGSPIPARGRLVMLPKVETEALGLARSHGEQGQMPENMQVSQ
FKMVNYSYDEDLEELCPVCGDKVSGYHYGLLTCESCKGFFKRTVQNNKRYTCIENQNCQIDKTQRKRCPY
CRFQKCLSVGMKLEAVRADRMRGGRNKFGPMYKRDRALKQQKKALIRANGLKLEAMSQVIQAMPSDLTIS

SAIQNIHSASKGLPLNHAALPPTDYDRSPFVTSPISMTMPPHGSLQGYQTYGHFPSRAIKSEYPDSYTSS
PESIMGYSYMDSYQTSSPASIPHLILELLKCEPDEPQVQAKIMAYLQQEQANRSKHEKLSTFGLMCKMAD
QTLFSIVEWARSSIFFRELKVDDQMKLLQNCWSELLILDHIYRQVVHGKEGSIFLVTGQQVDYSIIASQA
GATLNNLMSHAQELVAKLRSLQFDQREFVCLKFLVLFSLDVKNLENFQLVEGVQEQVNAALLDYTMCNYP
QQTEKFGQLLLRLPEIRAISMQAEEYLYYKHLNGDVPYNNLLIEMLHAKRA

>Q9QWM0_RAT
MSASSITGDFQDFLKHGLPAIGAGLPDRHRRPIPARSRLVMLPKVETEASGLVRSHGEQGQMPENMQVSQ
FKMVNYSYDEDLEELCPVCGDKVSGYHYGLLTCESCKGFFKRTVQNQKRYTCIENQNCQIDKTQRKRCPY
CRFKKCIDVGMKLEAVRADRMRGGRNKFGPMYKRDRALKQQKKALIRANGLKLEAMSQVIQAMPSDLTSA
IQNIHSASKGLPLSHVALPPTDYDRSPFVTSPISMTMPPHGSLHGYQPYGHFPNRAIKSEYPDPYSSSPE
SMMGYSYMDGYQTSSPASIPHLILELLKCEPDEPQVQAKIMAYLQQEQNNRNRQEKLSAFGLLCKMADQT
LFSIVEWARSSIFFRELKVDDQMKLLQNCWSELLILDHIYRQVAHGKEGTIFLVTGEHVDYSSIISNTEV
AFNNLLSLAQELVVRLRSLQFDQREFVCLKFLVLFSSDVKNLENFQLVEGVQEQVNAALLDYTLCNYPQQ
TEKFGQLLLRLPEIRAISKQAEDYLYYKHVNGDVPYNNLLIEMLHAKRA

>NR5A2_MOUSE
MSASLDTGDFQEFLKHGLTAIASAPGSETRHSPKREEQLREKRAGLPDRHRRPIPARSRLVMLPKVETEA
PGLVRSHGEQGQMPENMQVSQFKMVNYSYDEDLEELCPVCGDKVSGYHYGLLTCESCKGFFKRTVQNQKR
YTCIENQNCQIDKTQRKRCPYCRFKKCIDVGMKLEAVRADRMRGGRNKFGPMYKRDRALKQQKKALIRAN
GLKLEAMSQVIQAMPSDLTSAIQNIHSASKGLPLSHVALPPTDYDRSPFVTSPISMTMPPHSSLHGYQPY
GHFPSRAIKSEYPDPYSSSPESMMGYSYMDGYQTNSPASIPHLILELLKCEPDEPQVQAKIMAYLQQEQS
NRNRQEKLSAFGLLCKMADQTLFSIVEWARSSIFFRELKVDDQMKLLQNCWSELLILDHIYRQVAHGKEG
TIFLVTGEHVDYSTIISHTEVAFNNLLSLAQELVVRLRSLQFDQREFVCLKFLVLFSSDVKNLENLQLVE
GVQEQVNAALLDYTVCNYPQQTEKFGQLLLRLPEIRAISKQAEDYLYYKHVNGDVPYNNLLIEMLHAKRA

>NR5A2_CHICK
MLPKVETEALGLARSNGEQGQMPENMQVSQFKMVNYSYDEDLEELCPVCGDKVSGYHYGLLTCESCKGFF
KRTVQNNKRYTCIENQNCQIDKTQRKRCPYCRFQKCLSVGMKLEAVRADRMRGGRNKFGPMYKRDRALKQ
QKKALIRANGLKLEAMTQVIQAMPTDLTISSAIQNIHSASKGLPLNHTALPPTDYDRSPFVTSPISMTMP
PHGSLQGYQTYGHFPSRAIKSEYPDPYTSSPESIMGYSYMDGYQTSSPASIPHLILELQKCEPDEPQVQA
KIMAYLQQEQANRSKHEKLNTFGLMCKMADQTLFSIVEWARSSIFFRELKVDDQMKLLQNCWSELLILDH
IYRQVVHVKEGSILLVTGQQVDYSVIASQAGATLNNLMSHAQELVAKLRSLQFDLREFVCLKFLVLFSLD
VKNLENFQLVEGVQEQVNAALLDYTMCNYPQQTDKFGQLLLRLPEIRAISMQAEEYLYCKHLNGDVPCNN
LLIEMLHAKRA

>Q5XGE7_XENTR
MLPKVEPEALGLSRSHGEQGHMPENMQASQFKMMGYSYDEELEEMCPVCGDKVSGYHYGLLTCESCKGFF
KRTVQNNKRYTCIENQSCQIDKTQRKRCPYCRFQKCLSVGMKLEAVRADRMRGGRNKFGPMYKRDRALKQ
QKKALIRANGLKLEAMGQVIQSIPTDLTISSAIQNIHSASKGLPLNHAALPPSDYDRSPFVTSPISMAMP
PHGSLQGYQAYGHFPSRAIKSEYPDPYTSSPESIMGYSYMDSYQSSSPSNIPHLIVELLKCEPDEPQVQG
KIMAYLQQEQANRSKHDKLNTFGLMCKMADQTLFSIVEWARSSIFFRELKVDDQMKLLQNCWSELLILDH
IFRQVLHGKEGSILLVTGQQVDYSVIVTQAGATLNNLMSHAQDLVAKLRSLQFDLREFVCLKFLVLFSLD
VKNLENYQLVEGVQEQINAALLDYTMCNYPQQTDKFGQLLLRLPEIRAISLQAEEYLYYKHLNGDVPCNN

LLIEMLHAKRA

>Q9IB82_RANRU

MLPKVESEALGLSRSHGEHGQMPDNMQVSQFKMMGYSYDDDLEEMCPVCGDKVSGYHYGLLTCESCKGFF
KRTVQNNKRYTCIENQTCQIDKTQRKRCPYCRFQKCLSVGMKLEAVRADRMRGGRNKFGPMYKRDRALKQ
QKKALIRANGLKLEAMSQVIQAIPTDLTISSAIQNIHSASKGLPLNHTALPPTDYDRSPFVTSPISMAMP
PHGSLQSYQAYGHFPSRAIKSEYPDPYTSSPESLMGYSYMDSYQSSSPSNIPHLIVELLKCEPDEPQVQS
KIMSYLQQEQANRSKHDKLNTFGLMCKMADQTLFSIVEWARSSIFFRELKVDDQMKLLQNCWSELLILDH
IFRQVLHGKEGSILLVTGQQVDFSVIVSQAGTTLNNLMSHAQELVAKLRSLQFDMREFVCLKFLVLFSLD
VKNLENFQLVEGVQEQINAALLDYTLCYYPQQTDKFGQLLLRLPEIRAISLQAEEYLYYKHLNGDVPCNN
LLIEMLHAKRA

>Q91544_XENLA

MLPKVESEALGLSRLHGEQGHMPDNMQVSQYKMMGYSYDDELEELCPVCGDKVSGYHYGLLTCESCKGFF
KRTVQNNKRYTCIENQSCPIDKTQRKRCPYCRFQKCLSVGMKLEAVRADRMRGGRNKFGPMYKRDRAIKQ
QKKALIRANGLKLEAIGQVIQSIPTDLTISSAIQNIHSASKGLHLNHAALPPSDYDRSPFVTSPISMAMP
PHGSLQGYQSYGHFPSRAIKSEYPDPYTSSPESIMGYSYMDSYQSSSPSNIPHLIVELLKCEPDEPQVQG
KIMAYLQQEQANRSKHDKLNTFGLMCKMADQTLFSIVEWARSSIFFRDLKVDDQMKLLQNCWSELLILDH
IFRQVLHGKEGSILLVTGQQVDYSVIVTQAGATLNNLMSHAQDLVAKLRSLQFDLREFVCLKFLVLFSLD
VKTLENYQLVEGVQEQVNAALLDYTMCNYPQQTDKFGQLLLRLPEIRAISLQAEEYLYYKHLNGDVPCNN
LLIEMLHAKRA

>Q90YL6_BRARE

MLPKVESEYLGLARSHGEQGHMPGNMQAPQFKMMDYSYDEDLDEMCPVCGDKVSGYHYGLLTCESCKGFF
KRTVQNNKRYTCIENQSCQIDKTQRKRCPYCRFQKCLTVGMKLEAVRADRMRGGRNKFGPMYKRDRALKQ
QKKALIRANGLKLEAMTQVMQTVPADLTITSAIQNIHSASKGLPLSHHHHHHHHHHHSSSSAGLPPADF
DRSPFVTSPVSMAMPPHAGGLQGYQAYGHFQSRTIKSEYPDPYTSSPESLMGYPYVEAYAGGSPPSFPHL
VVELLKCEPDEPQVQAKILAYLQQEQASRGKHEKLNTFGLMCKMADQTLFSIVEWARSSIFFRELKVDDQ
MKLLQNCWSELLILDHVFRQVMHAKEGSILLVTGQQVDYALIASQAGATLNNLLSHAQELVSKLRSLQLD
QREFVCLKFLVLFSLDVKNLENFHLVESVQEQVNAALLDYVMCNYPQQTDKFGQLLLRLPEIRAISLQAE
EYLYYKHLNGDVPCNNLLIEMLHAKRA

>Q9GKL1_HORSE

MSSNLDTGDLRDSGKHGLTPIVSQFKMVNYSYDEDLEELCPVCGDKVSGYHYGLLTCESCKGFFKRTVQN
NKRYTCIENQNCQIDKTQRKRCPYCRFQKCLSVGMKLEAVRADRMRGGRNKFGPMYKRDRALKQQKKALI
RANGLKLEAMSQVIQAMPSELSISSAIQNIHSASKGLPLNHAALPPTDYDRSPFVTSPISMTMPPHGSLQ
GYQTYSHFPSRAIKSEYPDPYTSSPESIMGYSYMDGYQTSSPASIPHLILELLKCEPDEPQVQAKIMAYL
QQEQANRSKHEKLSTFGLMCKMADQTLFSIVEWARSSIFFRELKVDDQMKLLQNCWSELLILDHIYRQVV
HGKEGSIFLVTGQQVDYSIIASQAGATLNNLMSHAQELVAKLRSLQFDQREFVCLKFLVLFSLDVKNLEN
FQLVEGVQEQVNAALLDYTMCNYPQQTEKFGQLLLRLPEIRAISMQAEEYLYYKHLNGDVPYNNLLIEML
HAKRA

>O42186_BRARE

MLPKVESEYLGLARSHGEQGHMPGNMQAPQFKMMDYSYDEDLDEMCPVCGDKVSGYHYGLLTCESCKGFF

KRTVQNNKRYTCIENQSCQIDKTQRKRCPYCRFQKCLTVGMKLEAVRADRMRGGRNKFGPMYKRDRALKQ
QKKALIRANGLKLEAMTQVMQTVPADLTITSAIQNIHSASKGLPLSHHHHHHHHHHHHSSSSAGLPPADF
DRSPFVTSPVSMAMPPHAGGLQGYQAYGHFQSRTIKSEYPTPTQARQSPHGLPLRRSLRSGSPPSFPHLV
VELLKCEPDEPQVQAKILAYLQQEQASRGKHEKLNTFGLMCKMADQTLFSIVEWARSSIFFRELKVDDQM
KLLQKCWRELLILDHVFRQVMHAKEGSILLVTGQQVDYALIASQAGATLNNLLSHAQELVSKLRSLQLDQ
REFVCLKFLVLFSLDVKNLENFHLVESVQEQVNAALLDYVMCNYPQQTDKFGQLLLRLPEIRAISLQAEE
YLYYKHLNGDVPCNNLLIEMLHAKRA

>Q9IB81_RANRU
MMGYSYDDDLEEMCPVCGDKVSGYHYGLLTCESCKGFFKRTVQNNKRYTCIENQTCQIDKTQRKRCPYCR
FQKCLSVGMKLEAVRADRMRGGRNKFGPMYKRDRALKQQKKALIRANGLKLEAMSQVIQAIPTDLTISSA
IQNIHSASKGLPLNHTALPPTDYDRSPFVTSPISMAMPPHGSLQSYQAYGHFPSRAIKSEYPDPYTSSPE
SLMGYSYMDSYQSSSPSNIPHLIVELLKCEPDEPQVQSKIMSYLQQEQANRSKHDKLNTFGLMCKMADQT
LFSIVEWARSSIFFRELKVDDQMKLLQNCWSELLILDHIFRQVLHGKEGSILLVTGQQVDFSVIVSQAGT
TLNNLMSHAQELVAKLRSLQFDMREFVCLKFLVLFSLDVKNLENFQLVEGVQEQINAALLDYTLCYYPQQ
TDKFGQLLLRLPEIRAISLQAEEYLYYKHLNGDVPCNNLLIEMLHAKRA

>Q9PWI7_CHICK
MDYSYDEDLDELCPVCGDKVSGYHYGLLTCESCKGFFKRTVQNNKHYTCTESQNCKIDKTQRKRCPYCRF
QKCLTVGMRLEAVRADRMRGGRNKFGPMYKRDRALKQQKKALIRANGFKLETVPQIVSPVQNDYGLSSTI
HSIHAMAKTLPPNPAALTPADYERGPYGTPSLAMTVPGHTPLAGYHYPSFPNRAIKSEYPDHYSAAHEAV
PTYAYPETYPSSSPPDIPEVILKLLQLEPDEAQVKARILACLQQEQGKGRHEKLSTFGLMCKMADQTLFS
IVEWARSCIFFKELEVGDQMKLLQNCWSELLVFDHVYRQLQHGKEHSVLLVTGQEVDLSAVAAQAGSILH
SLVLRAQELVLHLHSLQVDRQEFVCLKFLILFSLDVKYLENHALAKDAQEKANAALLEYTVCHYPHCTDK
FRQLLLRLTEVRALSMQAEEYLYHKHLSGEVPCNNLLIEMLHAKRT

>STF1_RAT
MDYSYDEDLDELCPVCGDKVSGYHYGLLTCESCKGFFKRTVQNNKHYTCTESQSCKIDKTQRKRCPFCRF
QKCLTVGMRLEAVRADRMRGGRNKFGPMYKRDRALKQQKKAQIRANGFKLETGPPMGVPPPPPPPPDYML
PPSLHAPEPKALVSGPPSGPLGDFGAPSLPMAVPGPHGPLAGYLYPAFSNRTIKSEYPEPYASPPQQPGP
PYSYPEPFSGGPNVPELILQLLQLEPEEDQVRARIVGCLQEPAKSRPDQPAPFSLLCRMADQTFISIVDW
ARRCMVFKELEVADQMTLLQNCWSELLVLDHIYRQVQYGKEDSILLVTGQEVELSTVAVQAGSLLHSLVL
RAQELVLQLHALQLDRQEFVCLKFLILFSLDVKFLNNHSLVKDAQEKANAALLDYTLCHYPHCGDKFQQL
LLCLVEVRALSMQAKEYLYHKHLGNEMPRNNLLIEMLQAKQT

>Q812G5_MOUSE
MDYSYDEDLDELCPVCGDKVSGYHYGLLTCESCKGFFKRTVQNNKHYTCTESQSCKIDKTQRKRCPFCRF
QKCLTVGMRLEAVRADRMRGGRNKFGPMYKRDRALKQQKKAQIRANGFKLETGPPMGVPPPPPPPPDYML
PPSLHAPEPKALVSGPPSGPLGDFGAPSLPMAVPGPHGPLAGYLYPAFSNRTIKSEYPEPYASPPQQPGP
PYSYPEPFSGGPNVPELILQLLQLEPEEDQVRARIVGCLQEPAKSRSDQPAPFSLLCRMADQTFISIVDW
ARRCMVFKELEVADQMTLLQNCWSELLVLDHIYRQVQYGKEDSILLVTGQEVELSTVAVQAGSLLHSLVL
RAQELVLQLHALQLDRQEFVCLKFLILFSLDVKFLNNHSLVKDAQEKANAALLDYTLCHYPHCGDKFQQL
LLCLVEVRALSMQAKEYLYHKHLGNEMPRNNLLIEMLQAKQT

>O42102_CHICK
MDYSYDEDLDELCPVCGDKVSGYHYGLLTCESCKGFFKRTVQNNKHYTCTESQNCKIDKTQRKCCPYCRF
QKCLTVGMRLEAVRADRMRGGRNKFGPMYKRDRALKQQKKALIRANGFKLETVPQIVSPVQNDYGLSSTI
HSIHAMAKTLPPNPAALTPADYERGPYGTPSLAMTVPGHTPLAGYHYPSFPNRAIKSEYPDHYSAAHEAV
PTYAYPETYPSSSPPDIPEVILKLLQLEPDEAQVKARILACLQQEQGKGRHEKLSTFGLMCKMADQTLFS
IVEWARSCIFFKELEVGDQMKLLQNCWSELLVFDHVYRQLQHGKEHSVLLVTGQEVDLSAVAAQAGSILH
SLVLRAQELVLHLHSPQVDRQEFVCLKFLILFSLDVKYLENHALAKDAQEKANAALLEYTVCHYPHCTDK
FRQLLLRLTEVRALSMQAEEYLYHKHLSGEVPCNNLLIEMLHAKRT

>Q90XC4_POEGU
MDYSYDEDLDELCPVCGDKVSGYHYGLLTCESCKGFFKRTVQNNKHYTCTESQSCKIDKTQRKRCPYCRF
QKCLTVGMRLEAVRADRMRGGRNKFGPMYKRDRALKQQKKALIRANSFKLETVPQIMSPVQSDYSLSSTI
HSIHAMSKTLPPNPAALTPVDYERSPYGTPSLGMTVPGHAPLPGYHYPSFPNRTIKSEYPDHYTNAHEAV
PAYMYPETYPSSSPPDIPEVILKLLQLEPDEAQVKARILSCLQQEQGKGRHEKLSTFGLMCKMADQTLFS
IVEWARSCIFFKELEVGDQMKLLQNCWSELLVFDHIYRQLQHGKEHSVLLVTGQEVDMSAIAAQAGSILN
TLVLRAQELVLHLHSLQVDRHEFVCLKFLILFSLDVKYLENHTLAKDAQEKANAALLEYTVCHYPHSTDK
FRQLLLWLAEVRALSLQAEEYLYHKHLSGEVPCNNLLIEMLHAKRT

>STF1_BOVIN
MDYSYDEDLDELCPVCGDKVSGYHYGLLTCESCKGFFKRTVQNNKHYTCTESQSCKIDKTQRKRCPFCRF
QKCLTVGMRLEAVRADRMRGGRNKFGPMYKRDRALKQQKKAQIRANGFKLETGPPVGVPPPPPPPPDYML
PHGLHASEPKGLASGPPAGPLGDFGAPALPMAVPSAHGPLAGYLYPAFPGRAIKSEYPEPYASPPQPGPP
YGYPEPFSGGPGVPELILQLLQLEPDEDQVRARIVGCLQEPAKGRPDQPAPFSLLCRMADQTFISIVDWA
RRCMVFKELEVADQMTLLQNCWSELLVFDHIYRQIQHGKEGSILLVTGQEVELTTVAAQAGSLLHSLVLR
AQELVLQLHALQLDRQEFVCLKFLILFSLDVKFLNNHSLVKEAQEKANAALLDYTLCHYPHCGDKFQQLL
LCLVEVRALSMQAKEYLYHKHLGNEMPRNNLLIEMLQAKQT

>STF1_MOUSE
MDYSYDEDLDELCPVCGDKVSGYHYGLLTCESCKGFFKRTVQNNKHYTCTESQSCKIDKTQRKRCPFCRF
QKCLTVGMRLEAVRADRMRGGRNKFGPMYKRDRALKQQKKAQIRANGFKLETGPPMGVPPPPPPPPDYML
PPSLHAPEPKALVSGPPSGPLGDIGAPSLPMSVPGPHGPLAGYLYPAFSNRTIKSEYPEPYASPPQQPGP
PYSYPEPFSGGPNVPELILQLLQLEPEEDQVRARIVGCLQEPAKSGSDQPAPFSLLCRMADQTFISIVDW
ARRCMVFKELEVADQMTLLQNCWSELLVLDHIYRQVQYGKEDSILLVSGQEVELSTVAVQAGSLLHSLVL
RAQELVLQLHALQLDRQEFVCLKFLILFSLDVKFLNNHSLVKDAQEKANAALLDYTLCHYPHCGDKFQQL
LLCLVEVRALSMQAKEYLYHKHLGNEMPRNNLLIEMLQAKQT

>Q9YI54_TRASC
MDYSYDEDPNQLCPVCGDKVSGYHYGLLTCESCKGFFKRTVQNNKHYTCTESQNSKIDKTQRKRCPYCRF
QKCLTVGMRLEAVRADRMRGGRNKFGPMYKRDRALKQQKKALIRANGFKLETVPQIVSPVQTDYNLSSTI
HGIHSVSKSLPPNPATMTPVDYDRSPYGTPSLGMTVPSHGALSSYHYPSFPNRTIKSEYPDHYTNSHESV
ASYMYPDAYPNSAPPDIPEVILKLLQLEPDEPQVKVRILACLQQEQGKGRHEKLSTFGLMCKMADQTLFS
IVEWARSCIFFKELEVGDQMKLLQNCWSELLVFDHIYRQVQHGKEHSMLLVTGQEVEMATIAAQAGSNLN
NLVLRAQELVLHLHSLQVDRQEFVCLKFLILFSLDVKYLENHSLAKDAQEKANAALLEYTICHYPHAADK
FRQLLLRLAEIRSLSMQAEEYLYHKHLSGEVPCNNLLIEMLHAKRT

>STF1_PIG
MDYWYDEDLDELCPVCGDKVSGYHYGLLTCETCKGFFKRTVQNNKHYTCTESQSCKIDKTQRKRCPFCRF
QKCLTVGMRLEAVRADGMRGGRNKFGPMYKRDRALKQQKKAQIRANGFKLETGPPMGVAPPPPPPPDYML
PPGLHAPEPKGLAAGPPTGPLGDFGAPTLPMAVPSAHGPLAGYLYPAFPGRAIKSEYPEPYASPPQPGPP
YGYPEPFSGGPGVPELIVQLLQLEPDEDQVRARIVGCLQEPAKGRPDQPAPFSLLCRMADQTFISIVDWA
RRCMVFKELEVADQMTLLQNCWSELHVFDHIYRQIQHGKEGSILLVTGQEVELTTVAAQAGSLLHGLVLR
AQELVLQLHALQLDRQEFVCLKFLILFSLDVKFLNNHSLVKDAQEKANAALLDYTLCHYPHCGDKFQQLL
LCLVEVRALSMQAKEYLYHKHLGNEMPRNNLLIEMLQAKQT

>STF1_HUMAN
MDYSYDEDLDELCPVCGDKVSGYHYGLLTCESCKGFFKRTVQNNKHYTCTESQSCKIDKTQRKRCPFCRF
QKCLTVGMRLEAVRADRMRGGRNKFGPMYKRDRALKQQKKAQIRANGFKLETGPPMGVPPPPPPAPDYVL
PPSLHGPEPKGLAAGPPAGPLGDFGAPALPMAVPGAHGPLAGYLYPAFPGRAIKSEYPEPYASPPQPGLP
YGYPEPFSGGPNVPELILQLLQLEPDEDQVRARILGCLQEPTKSRPDQPAAFGLLCRMADQTFISIVDWA
RRCMVFKELEVADQMTLLQNCWSELLVFDHIYRQVQHGKEGSILLVTGQEVELTTVATQAGSLLHSLVLR
AQELVLQLLALQLDRQEFVCLKFIILFSLDLKFLNNHILVKDAQEKANAALLDYTLCHYPHCGDKFQQLL
LCLVEVRALSMQAKEYLYHKHLGNEMPRNNLLIEMLQAKQT

>STF1_MACEU
MDYSYDEDLDELCPVCGDKVSGYHYGLLTCESCKGFFKRTVQNNKHYTCTESQNCKIDKTQRKRCPYCRF
QKCLTVGMRLEAVRADRMRGGRNKFGPMYKRDRALKQQKKALIRANGFKLETGPSMGPPPQTDYPLAPAL
HPGAKGLAPAPPAGPPGDYERGPYGPPGVPMAVPTHGPLAGYLYPAFPGRAIKSEYPEPYASPHEPAPPY
GYPEPYPSGPGLPGVPELILKLLQLEPDEGQLKARILACLQEPSKGRPDRPTPFGLMCKMADQTLFSIVE
WARSCVVFKELEVADQMKLLQNCWSELLVFDHIYRQIQHGKEGSILLVTGQEVDLSTVAAQAGSLLHSLV
LRAQDLVQQLHSLQVDRQEFVCLKFLILFSLDVKFLENHGLAKDAQEKANSALLEYTMCHYPHCGDKFRQ
LLLRLAEVRSLSMQAEEYLYHKHLGGEVPCNNLLIEMLHAKRT

>STF1_HORSE
MDYSYDEDLDELCPVCGDKVSGYHYGLLTCESCKGFFKRTVQNNKHYTCTESQSCKIDKTLRKRCPFCRF
QKCLTVGMRLEAVRADRMRGGRNKFGPMYKRDRALKQQKKAQIRANGFKLETGPPMGVPPPPPPPPDYML
PPGLHVPEPKGLASGPPAGPLGDFGAPALPMAVPSTNGPLAGYLYPAFPGRAIKSEYPEPYASPPQPGPP
YGYPEPFSGGPGVPELILQLLQLEPDEDQVRARIIGCLQEPAKGRPDQPASFNLLCRMADQTFISIVDWA
RRCMVFKELEVADQMTLLQNCWSELLVFDHIYRQVQHGKEGSTLLVTGQEVELTTVAAQAGSLLHGLVLR
AQELVLQMHALQLDRQEFVCLKFLILFSLDVKFLNNHSLVKDAQEKANTALLDYTLCHYPHCGDKFQQLL
LCLVEVRALSMQAKEYLYHKHLGNEMPRNNLLIEMLQAKQT

>Q9YI95_RANRU
MDYSYDEDLDELCPVCGDKVSGYHYGLLTCESCKGFFKRTVQNNKRYTCTENQSCKIDKTQRKRCPYCRF
QKCLNVGMRLEAVRADRMRGGRNKFGPMYKRDRALKQQKKALIRANGIKLETVPQIVSQVQTDYSVANNI
HTIHPVSKNLPSNTAPMTPVEYDRGSYGPPPIAMTLPNHAPLSGYHYSSFQSRTIKSEYPDHYSNVHDPS
TAGGYVYPEAYTSTSQPDIPEVILKLLQLEPDEPQIKARIISCLQQEQNKSRHEKLSMFGLMCKMADQTL
FSIVEWARSCIYFKELEVSDQMILLQNCWSELLVFDHIYRQMQHSKENSILLVTGQEIELSAIAAQAGST
LNNLVLRAQELVILLHSLQVDRQEFVCLKFLILFSLDEKFLENHSLAKSAQEKVDSALMEYTMCHYPHCT

DKYRLLLLRLAEIRSISMQAEEYLYHKHLSGEVPCNNLLIEMLHAKRA

>Q6QHU4_PLEWA
MEYTYDEDLDELCPVCGDKVSGYHYGLLTCESCKGFFKRTVQNNKRYTCTENQTCKIDKTQRKRCPYCRF
QKCLTVGMRLEAVRADRMRGGRNKFGPMYKRDRALKQQKKALIRANGFKLETVPQIVSPTQTEYTIPSNI
HSIHSVSKSLPLNTVAMTQVNYDRSPYGTPSLGMTMPNHGALQGYTYPHFPNRTIKSEFPDHYSSPHESV
APAFVYSDAYQNSFPPDIPETILNLLQLEPDEPQIKARILACLQQEQGKSRHEKPPSTFGLMCKMADQTL
FSIVEWARSCIYFKELEVGDQMILLQNCWSELLVFDHIYRQIQHGKENSILLVTGQEIDVSTIAAQAGPA
LNNLVLKSQELVIQLHSLQVDRQEFVCLKFLILFSVDEKCLENNTLARNAQEKINAALHEYTMCHYPHCM
DKFRLLLLRLTDIRSISMLAEDYLYHKHMSGEVPCNNLLIEMLHAKRS

>O93258_ORYLA
MLGDKAHGVTLKVMEYTYDEDLEELCPVCGDKVSGYHYGLLTCESCKGFFKRTVQNNKRYTCAENQECKI
DKTQRKRCPFCRFQKCLNVGMRLEAVRADRMRGGRNKFGPMYKRDRALKQQKKALIRSNGFKLESTALPS
ASPLQTDYSFTGTLHTLPTISKSLLPSTTSSVTPTDYEANLYGPTSLGMAMQSHVPLNPQYQYTAFPSRA
IKAECPDYTSSPESLTGYPYPDVYPSASPQPPSLPPLVLELLRCDPDELVVQNKIVAHLQQEQSNRGRLD
KPSTFSLMCRMADQTLFSIVEWARSCIFFKELRVGDQMKLLHNCWSELLVLDHIFRQVQHGKEDSILLVT
GQEVQLSSILSQGEATLCSLVQRGQELAARLRALQVDRREIACLKFLLLFNPNVKLLENQAFVEGVQEQV
NGALLEYTLTTYPQFQEKFSQLVVRLPELRSLSTQAEDYLCYMHLSGEVPCNNLLIEMLHAKRACV

>Q7ZT68_ORENI
MLGDKAHGVTLKVMEYTYDEDLEELCPVCGDKVSGYHYGLLTCESCKGFFKRTVQNNKRYTCAENQECKI
DKTQRKRCPFCRFQKCLNVGMRLEAVRADRMRGGRNKFGPMYKRDRALKQQKKALIRSNGFKLESTAPPP
ASPLQADYGFTGTLHTLPTISKSLLPSTPSSITPTDYEANLYGPPSLGMAMQSHVPLTTQYQYTAFPGRA
IKAECPDYTSSPETLTGYPYPDMYPSASPQPPSLPPLVLELLRCDPDELVVQNKIVTHLQQEQNGRGRLE
KPSTFSLMCRMADQTLFSIVEWARSCIFFKELRVGDQMKLLHNCWSELLVLDHIFRQVQHAKEDSILLVT
GQEVELSSILSQAEGTLSSLVQRGQELAARLRVLQVDRREIACLKFLLLFNPNVKLLENQAFVEGVQEQV
NAALLEYTLSAYPQFQEKFSQLLVRLPELRSLSTQAEDYLCYMHVSGEVPCNNLLIEMLHAKRACV

>Q800U8_BRARE
MDYSYDADLEEMCPVCGDKVSGYHYGLLTCESCKGFFKRTVQNNKRYTCAESQDCKIDKTQRKRCPFCRF
QKCLNVGMRLEAVRADRMRGGRNKFGPMYKRDRALKQQKKALIRASGLKMEATPPLLTSPQPDYTFSTAL
SVPAPKNTHPNIVTSVAPTDYERSLYASSSLSLSVPIPAHTPLPAQYPYPNLPSRAIKSEYPDHYTSSEH
YTSASSPESVPGYTYIEQTRVSSSPQVVAPGLTVPPLVLEFVRCEQDELQVQSKISAHLAHLQQEQNSRS
TAANQEQSSRLAAKPERLSTFGLMCHMADQTLFSIVEWARSCIFFKELKVGDQMKLLHNCWSELLVLDYV
ARQLHHGKEDSVLLITGQEVELASLLAQAGVTLSGMIQRGQELVQRLQELQLDRRETACLKYLILFNPDV
KLLENQPYVESVYEQVNAALLEYTLCAYPQFPDRFSQILLRLPELRALSSQAEDYLCYKHLSGEVPCNNL
LIEMLHAKRTCI

>Q8UV27_CLAGA
MEFTTEEDLEELCPVCGDKVSGYHYGLLTCESCKGFFKRTVQNNKRYTCNQNQDCGIDKTQRKRCPFCRF
QKCLSVGMRLEAVRADRMRGGRNKFGPMYKRDRALKQQKRALIRASTFKLEHNPPLVPSNQAEYPFPGSV
PGLLAPLGPPDYDCPPACPPSLGVALHSYGSFPAQYQYTTPTVPGRSIKAEHPDPYSGSPDSSLGYSYAE
GCIAASPQTSPLNPAVPSLVLELLSCEPDEEQVRAKICAYLQQEQSGRGKLDKPRPSNLLCVMADQTLFS

IVEWARSCIFFKELKVGDQMQLLHNCWSELLLLDHVFRQVHHGRDNTLLLITGQEVELAGVVSDSGLTLS
SLVQRGQELARRLQLLQVDRREMACLKFLVLFNPNVKLLENQALVESVQEQVNSSLLEYTLTSYPQHVDR
FSQLVLRLPEVRALSAQAEEYLSSKHLSGDVPCNNLLIEMLHAKRATTI

>Q9IAI9_BRARE
MDFRADEDLEELCPVCGDKVSGYHYGLLTCESCKGFFKRTVQNNKRYTCTQNQDCGIDKTQRKRCPFCRF
QKCLSVGMRLEAVRADRMRGGRNKFGPMYKRDRALKQQKRALIRASGFKLEANGTLLSGQTEFSSVGLQS
PYDCPPPALGVALQNYGSFPAQYQYTAPSLPKSIKAEYPDAYPSSPDSTLGYSYADGCLSASPHSVPINP
ALPPLVLELQSCDPDEEQVRGKICAYLHQEQSGRGKLEKSRPSSLLCVMADQTLFSIVEWARSCVFFKEL
KVGDQMRLLHNCWSELLLLDHICRQVHHGRDGSLLLITGQEVELSAVLDAGPPLSSMVERGQDLSRRLQL
LQVDSREMACLKFLILFNPNVKLLENPQFVESVQEQVNGALLEYTLFSYPQCVERFSQLILRLPELRSLS
AEAEDFLCYKHLCGEVPCNNLLIEMLHAKGSSAQ

>FTZF1_BOMMO
MHEDAPKMSIAQSLAASTSQPKGDIVTEIPLEFAMSSMETKSIETTNVELKITYVDPTTGTGGEPGAYLP
TAGTVCDQTDTKDVIEELCPVCGDKVSGYHYGLLTCESCKGFFKRTVQNKKVYTCVAERACHIDKTQRKR
CPFCRFQKCLDVGMKLEAVRADRMRGGRNKFGPMYKRDRARKLQMMRQRQIAVQTLRGSLGDGGLVLGFG
SPYTAVSVKQEIQIPQVSSLTSSPESSPGPALLGAQPQPPQPPPPPTHDKWEAHSPHSASPDAFTFDTQS
NTAATPSSTAEATSTETLRVSPMIREFVQTVDDREWQNALFGLLQSQTYNQCEVDLFELMCKVLDQNLFS
QVDWARNTVFFKYLKVDDQMKLLQDSWSVMLVLDHLHQRMHNGLPDETTLHNGQKFDLLCLGLLGVPSLA
DHFNELQNKLAELKFDVPDYICVKFMLLLNPEVRGIVNVKCVREGYQTVQAALLDYTLTCYPTIQDKFGK
LVMVVPEIHALAARGEEHLYQRHCAGQAPTQTLLMEMLHAKRKS

>Q91601_XENLA
MLPKVESEALGLSRLHGEQGHMPDNMQVSQYKMMGYSYDDELEELCPVCGDKVSGYHYGLLTCESCKGFF
KRTVQNNKRYTCIENQSCPIDKTQRKRCPYCRFQKCLSVGMKLEAVRADRMRGGRNKFGPMYKRDRAIKQ
QKKALIRANGLKLEAIGQVIQSIPTDLTISSAIQNIHSASKGLHLNHAALPPSDYDRSPFVTSPISMAMP
PHGSLQGYQSYGHFPSRAIKSEYPDPYTSSPESIMGYSFMDSYQSSSPSNIPHLIVELLKCEPDEPQVQG
KIMAYLQQEQANRSKHDKLNTFGLMCKMADQTLFSIVEWARSSIFFRDLKKIK

(6) 0 NR6

(7) 6 NR0

>DAX1_PIG
MAGEDHQWQGSILYNMLMSAKQTHATREAPEARLRGSCWGCSCGSEPPVGREGQPGGPAVALLYRCCFCG
EDHPRQGSILYNMLTSAKQTQETPEAPEARLGGACWGCSCGSEPRVGREELPGGRATVLLYRCCFCGEEH
PRQGSILYSLLTSAKQTHVALEAPEARPGGAWWDRSYCAQRLGAREELPGGRPVTLPYRCCFCGEDHPRQ
SGILCNMPMSAKQTHVAPEAQPGAPWWDPSCAAQRVALKSPQVVCEAASAGLLKTLRFVKYLPCFQVLPL
DQQLVLVRSCWAPLLMLELAQDRLNFETVETLEPSLLQMILTTRRQETEGDEPPSPQPPVQPHLVLPSEA
EHLPSVAEVQAIKGFLAKCWSLDISTKEYAYLKGTVLFNPDLPGLQCVKYIQGLQWGTQQILSEHIRMTH
RGYQARFAELNSALFLLRFINANVLAELFFRPIIGTVSMDDMMLEMLCAKL

>DAX1_RAT
MAGEDHPWHGSILYNLLMSAKQKHGSREEREVRLGAQCWGCACGTQPVLGGEGLPGGQALSLLYRCCFCG

ENHPRQGGILYSMLTNARQPSGATEAPRARFRTPCWGCACSNAKPLVGRXGLPAGQVPSLLYRCCFCGKK
HPRQGSILYSLLTNAQQTHVSREVPEAHRGGEWWQLSYCTHNVGGPEGLQSTQAMAFLYRSYVCCEEQPQ
QSSVASDTPVRADQTPAAPQEQPRAPWWDTSSGVQRPIALKDPQVVCEAASAGLLKTLRFVKYLPCFQIL
PLDQQLVLVRSCWAPLLMLELAQDHLHFEMMEISEPNLMHEMLTTRRQETEGPEPADPQATEQPQTVSAE
AGHVLSVAAVQAIKSFFFKCWSLNIDTKEYAYLKGTVLFNPDLPGLQCVKYIESLQWRTQQILTEHIRLM
QREYQIRSAELNSALFLLRFINTDVVTELFFRPIIGAVSMDDMMLEMLCAKL

>DAX1_HUMAN
MAGENHQWQGSILYNMLMSAKQTRAAPEAPETRLVDQCWGCSCGDEPGVGREGLLGGRNVALLYRCCFCG
KDHPRQGSILYSMLTSAKQTYAAPKAPEATLGPCWGCSCGSDPGVGRAGLPGGRPVALLYRCCFCGEDHP
RQGSILYSLLTSSKQTHVAPAAPEARPGGAWWDRSYFAQRPGGKEALPGGRATALLYRCCFCGEDHPQQG
STLYCVPTSTNQAQAAPEERPRAPWWDTSSGALRPVALKSPQVVCEAASAGLLKTLRFVKYLPCFQVLPL
DQQLVLVRNCWASLLMLELAQDRLQFETVEVSEPSMLQKILTTRRRETGGNEPLPVPTLQHHLAPPAEAR
KVPSASQVQAIKCFLSKCWSLNISTKEYAYLKGTVLFNPDVPGLQCVKYIQGLQWGTQQILSEHTRMTHQ
GPHDRFIELNSTLFLLRFINANVIAELFFRPIIGTVSMDDMMLEMLCTKI

>Q9PTE9_CHICK
MACLERCHCCADGRRHGSILYSILKSHDQAAEGPGPRRGQAGRGCSCGSQRRVALKSPQVVCKAASAVLV
KTLRFVQNVPCFQELPLDEQLVLVRSCWAPLLVLGLAQERVHLETVESAEPSMLQRILTTRRLGEHAPAP
GRQHPPSAGEIQAIKGFLAKCWSLDISTKEYAYLKGTVLFNPDLPGLQCTQYIEGLQKEAQEALNEHVRL
IHRGDQARFAKLNVVLSLLRSINANVIAELFFRPIIGSVNMDDMLLEMLCAKL

>SHP_MOUSE
MSSGQSGVCPCQGSAGRPTILYALLSPSPRTRPVAPASHSHCLCQQQRPVRLCAPHRTCREALDVLAKTV
AFLRNLPSFCHLPHEDQRRLLECCWGPLFLLGLAQDAVTFEVAEAPVPSILKKILLEEASSGTQGAQPSD
RPQPSLAAVQWLQRCLESFWSLELGPKEYAYLKGTILFNPDVPGLRASCHIAHLQQEAHWALCEVLEPWY
PASQGRLARILLMASTLKNIPGTLLVDLFFRPIMGDVDITELLEDMLLLR

>SHP_HUMAN
MSTSQPGACPCQGAASRPAILYALLSSSLKAVPRPRSRCLCRQHRPVQLCAPHRTCREALDVLAKTVAFL
RNLPSFWQLPPQDQRRLLQGCWGPLFLLGLAQDAVTFEVAEAPVPSILKKILLEEPSSSGGSGQLPDRPQ
PSLAAVQWLQCCLESFWSLELSPKEYACLKGTILFNPDVPGLQAASHIGHLQQEAHWVLCEVLEPWCPAA
QGRLTRVLLTASTLKSIPTSLLGDLFFRPIIGDVDIAGLLGDMLLLR

2. 500 non-NR proteins

>E4YGX1_OIKDI
MNFIKLPFQSNLAVRRRPQLRLLRHARDQALH

>E4YGY5_OIKDI
MQKTPGSPTRAARRRSEPLHHHHHQQQRDEDNAVPSTPERDQPSTSRAAATRRLNLSNSPLQVQDDDAEP
NVFALYDMFSHLLNAQDQQRLQDEILKLEDRYITATNGIWHDIENNAAKITILTRGNKAFSVKGKNYVE

>E4YGY7_OIKDI

MDAEGRPIDLTMSADLSIIDGAGGWASSSMMVAPPMTPSVRRTQDFDEKDMSTDDLETTLKSTMKVRDEH
RSFIDDKHDQIVKGLRHCETNENIFFVAEHTIIKDQEWAIEDEITKMMKRLELLKKRETLADILKRYPSR
KDEIEEKLNEVNAELDSLDDTVTEEPSTDDKAFNSTNESTLLNRTQVSYIKIIHYQEPADEVVETTVTIA
AQPTFEFDIPKLTAEHVAAIRSKKVEHVKMCSKEAQTEAPARKKSVDYSSQTPQLQMRDEKSQAKVLAHE
MAMQTAKPRSGVDAPSQTSVQRNSTVEFTDSFSQTKEIQRKQTKTNDSGIDSAADHSDIEATPVQKAVIV
NQLQQNVNAAPVKKTMDMACDPIVWPKSNYNTIETSSSSDSESESNKTKPGEKGKKAAAAKMIKDKKDGK
KLKTKSCGTNPEKIKKKDQSTKTMPKTTSSKTMQTEKVKNEKMVHKQVNTLFDSRKIAELKQKAEDLKRE
NELLKTAALAQKLKKEERTSSSDEDTKPSKVKIKRVPSKKKTTVEKRALSTASMKSTDDLTRDPDSDSEQ
TKKLKEENERLIAENARLKKDRKVSSSSSSSDVDAPVVFIETSSGPPLPEASEEEGTQVDREKVKNAAIL
KKMKDREDEIVELKQNLAKSEAEKKVLEDKIFDADEAQLNEDELKAEIAWLTSENEALRKDLENGEKEEK
RKNAFLLKELSDKNDEVNNFNEELKGLQDNFENEKTEKNEMIKQLEKDIQKKDDEIKELQDEVAGFNEDL
EQERRNKAVLLKKLKDREDELENELNEALKDKDDQIGELEKLNDALIDANKELKSDNANNEDHKAAKILA
LKNLADKEKEIKKLNSQLQEIKEEKASLENDVEKLFLERNAAKEELDELANAHKDLMNELQHAADESNDH
KLAKLAALKKIKELEAEKKELQEQCDTYAEQLEAASEMSEIDEKSLADAKHQNKLLNQTLDSVKSSVEDL
KNASFQEDKETEEKIQALEEEKSEKIKVIKNLEETIESLEEQIEDLNGENEKSRDEKLKTLAKIKLLEDA
QNEKEDLEDELEKNRSNLAALEKKIKDQDEAIQDLEEELNNKTTEIVNLKQKVSELESELATDKGDKAKA
LLVTKELNDRKEEIDFLKEEIENLKSENSQLAKNQESEDDRKKKLLVAKELAERKEEIKKLNKELDELKK
SQTKIKTKDQSTKTLPKPTSSKTMQTEKIKNEKMVNKQVNTLFDMKRVEEIKQMAEELKRENAKLKETQE
SEEDGAKKAFVAKELVERKEEIKKLEKDLEKLDIENKDLLKQAEENKDNKAAKLLIAKELKDREDEISKL
KQALAVEEQNAKNAADPNKITELEDEIAALEDERDRALAKIKGLEKDLEFSKVLEDEVDKKEKEILAKDE
QIQAYEETIAENNRKLKDLLVLKKAAEKVSETEAANETLKTEISEIKEERDELKSELEVVRNAQDADSSA
EGLEGKKPLTGSTKTLDSGIFDKTHTLEDIPLGSSNENIRSEPQMIIIQRLQVENERLIAERDSVGDIET
LKKEYQDEKAHLEEDLDHQQQKLTQLTGELQKLLQDKAGLEEDLELKNEDLQAVEEDLEDVKSKLKKVQV
ENDELVDDNENRLKELVALKALKDRSGTIDDLENERDELKQALVAHKTVEQLKLLNENGEEKLAKLNDAY
QDLEAELREKDAEMEQIEEQLQNNKFEKAKTLASLKALRKSVETGDEESDSSEEESDIFVDANDQSKPEE
IINDIKKVLVVKKGADNRKDIENLEEQIADLEDENDELKAQNEKLEEIRKKHKDEIEKLTEENEILHEDV
QSRPSSIAIAPIVEQQQVQSTNPFLQTHSDIESSIQPDEPKTSTLLRELENENERLMNEIRDLKAQLDLS
RTEMKFITAEKSDSGSSSGDLAEENESLRAEVKRLAALVAMYKGRDSDDEVDLRPAKRTDSVPDLLEADT
QKNLKEGVLHELTNTQKKLKEEEDKRQDLERVVDKLNETIEGLQTPEAQQRLKDGALSELEREKKRRSEA
EKELEKAKNEAELAKELKEGALAEVDRLNSSLISSKLEELEKTDSKSSSRQSTPIPSKKLDELELQTMEN
EVQLQALRPDASELAKKSPEEQDKILEDLKKTLEAKARDIAELEQEIEELKDENKELKVEAYDAQNYKKK
FKKAVDEKHAQGQDLEELTTPGKVQFIRNLFFTTRTILNCIRRLSDSLLIRIPSPEGRMLKNNDEIFVDQ
EKLSQQKRDPVDDNALWNISKMKRDMRQLQAQLRELDDRNSTLENKQSRALEQTDILEGHLNRASREQSD
LLNMSISSSNRSLDRINDSMTDQMIRVRDEMRVTDDENNRLIYELDQLKRDHEETERELRQARNKLVWQQ
KELEALREATSKPNVDDYDKLMSEYTSALDKIEKQEEKLEQASVSSKADDLQSHLDQSRAECDLYEEEIG
ELKKKISVLEAKLKSSGRQSPNSEIHDISLGDSLVEKLDNLEIKEDWTSEERLKLEMVNELRYIFSEPEI
VREEVSGPSSLESSSSSGKKKKENIDVLKESKETIRLLQSALEKLADESAESKDVSSELKGLSDQSVRLT
EQLKKRQDFIDRLVEKYKSSEEKVREFIRSTMRAILQREQDRIAQRVSQDESVASMTFDL

>A8PAW1_BRUMA
MALALLSVNICQFSGCGLNFGNLHDLLQHIEETHIPIIEDEMKKKEENAKNSSLDDDPSKAAALTAVFPI
STICKLFSKLPPHKAVPINPEPVKLAIGHYRKKTLASAVVGANVFGQAGDKSRFDQHDEESNDLYRNEDC
DDYEQGTDERRHKCPVEGCNKRYKNLQGARYHARQVHGYSEDNGTNNAVSFDLTAVVPGQVAPLIQSGTP
SKYANLRPYKCSQCSKRYKTTVGLNNHVQQSHQRINNAGNPAMSTNLVPVSPSAVSISTTHNNVASPAAS

TYVTPEGDEHVAAPSGYLTSTVQNATQVRINTQPLPSLAPVTGQQYQQQMSHPTNVTKLVSTVNGMMTRN
MNALSSYGPPTSESLNPVISRPDTPQVTDFCVQMNKRH

>E4YGY9_OIKDI
MIVRNLKSVLSGHINRIIREYIDKDLPGVFKPLTDYLYVIVGMLMTVAVQSSSIVCAVITPLCGIGVISL
ERAYSLIVGCCIGTTTTGLIAALASMGDGFAESMQVSLAHLFFNMVGFMLWFVVPCARRLPIRLALWAGA
ETEKYRWWAFCYIIGLFLAFPGLAFILSLVAPIFSDKVIKESYSFQNALTFAKLYKLCSGNVYFFLNSIE
SDYTPKYKIQSAQAAPKVFLKQAVDSVDIDVDVDEFGRGKKKKNKNKNKNKLPKPKKITTTTAAPTTTGW
DKFTSVSANYDDYYFYPEKDWKIVETTTTDRYEPTTTSWPTTPALVYTTEAPQQLKPVKQYNLPAGLQLP
AHLSNFNAGVFAAPKPDFAISSEDEYEYYYEYYYDESDSYYDDLGMKKPTKKKRRKKRKKNQKKKKNQKN
YANYQNSYSQPAFECWECLSANSFEDCQNRGKYVQCNGGSIGCDITVRSRRNNRYRDWYIEKVQMGCKQM
QSCQTEHQFNFGYHPQCFPEDSTWPALLQWNHSTCRQCCGTSKCNFDWIQNKPDTTAEWNSTDTHVVSGY
GNYNSNSNTNSGSYNYNYNNNYNSQNNNNYANNGAYNGGYYNG

>E4YGZ7_OIKDI
MSDGVRNRNNIGGSGSKSSIRSKKPDNSAFKQQRLPAWQPVLTAKSVLPIFFIVGIIFIPIGSLILVASN
GVQEVEQMYTDCQAQFTFTTHPPTLADITDVSADEKSCKTIYDEWIDTFSSGTAPNGNPPTCICKQNFEI
AETMNTPIFAYYRLTNYYQNHRRYVKSRDDTQLLAEKSYISTEADGDCSPYDKIGERPIAPCGAIANSLF
NDTFFIRRCGDAGVECTALQPDNIIDPTDANGFNAIKMTGEDIAWKTDKSQKFDPNKETGNETFLSGTER
PLNWRTDVHKLGTADDDLTYRHLSGSSGVGFRNEDFIVWMRTAAFPTFRKLYRKIQDNGADLQPGNYELL
TYYNYPVHRFGGGKFFVLATTSWIGGKNLFLGWTYAIVGGICLIVMLFLLCISRRNHNRRSIV

>E4YGZ9_OIKDI
MLQECVFVSSTIRPTASGSVLSSVTAQLQIFQLNTQILSR

>E4YH01_OIKDI
MSIAELNRKLDSLEEQITEKNIISLATAQTKLDKIKKYANVDTLFKEISRLSHLLDLSCAEMTPEAMDQL
AISIEPIVKQISPYMDKVQSLEKYSHIGKLENMIEKKKDIQKLARLNSLQYERVQNLGENTGILLAHYNA
IIDNLSRDFAVLEAQLSELEDLSP

>E4YH05_OIKDI
MIRFRRAKVYDFNRMPELKLIALHEFLVKQDARVIDELYRHPATCMSVFRELPQIAKHIIIRILFINQQI
AKPLIESWVADEHREKFEVAMEIITGLRIWENTNDGIAFNLNNSFRTYLQEALFGGGETWRPAVETLGAD
KNAKTVEQLDTYTKERWDQILSFLTQEQGKLSEEVISLLKYAGLCDANGEKRFQFLLLDRSSQVWYLLVQ
YLGYVQKLGLSLVNVLAFVLQLGYCSFGTDYPCDNSNNEISRVIQHFREMGLIFKRKSKEQRFYPTRLAQ
SISIAGGKKASSEDVQEQFILVETNYRIYAYTDSELHYALISLFAEVQYRFPYMIVAQMSRDSIQQSADY
GISAEQILNYLRSSAHPIARKNKHWVPQVVEDNIHLWCKERERLKFNDGLLYHQFLDQEAFEMLKSYAQD
IRALVWANDERRFMVVAPWSHDQIKSYYKQIKDGMM

>E4YH09_OIKDI
MLDEMHRRKVEKDIADLKKLIDQHFVQRKKDEEDLDSLRGRIANRKIVRDQQIQARNQKLKAREAQLALE
KDKREAIENRRKAEEEERKKATMAAIQYATSNPKKRLQKKGGNKQEKKKILAARRKNGNIDHLTGPQLAE
KAQEFFKHFTEINEDKYNLEITLQKHQYTLKLLRQRVHDLVGRQNKINTRK

>E4YH11_OIKDI
MSSDDWTIVTRRSRLAANASPEPQKELLTRNSFESLTDSSGTESVLNETPETKRGPPVEMEENDPTSLHG
TPPPEATDAPRAKSATKTLAQRVLRLFGRSGEDLQQRPERIQFKVSKKSTSKSQKKSQKFKKGRNVLTKK
NSRVTRASMKNAPPQSDLQTDLPAPASSDSSSDFDEEITAPCPTIDELQAAEVTITGHSRPVNDNLAAVF
GPKEPDSTLPSPFSAIKDHGFGSYATILNDIDMEQEHTEPGDGGVDTQFRVFHGLTEFFQGCIFPQLQLH
FQDQSSDSSKVQLALFTLMAGYLSIIMPKRSLSFTSMFQYCTCILTGAESHSHFRLQRERATRGVLDVFS
RLLDVNSLKCHVEINDDDLTTATQELLDSMTALLPDQTVLKQSFENELRADLMLIDTELVVNAHLRQVIL
SSLYVCDALGNKDAELGPAVSYRYRVLKALPFHALQVLMNMEILKCDDRLTLHPSEVKEEFFLPYLEEVV
KLRSDEPASQQSDVRYPIPAFNHFKPLAKQIIQERQRQRLSSSANIEQPEDPTGSGIEVEESSINGSDHP
ESDSASYFVQFDGPTVRVFGMVEVDPRLLIHRLTQSRLDDHFPAQESDQTDHLANREDQPSPARASPTTE
TTENQVHNGDDLLPKTLPNDSIIPHGTRGAVQRKRQNSGADHEQFSKHPRLRASPITAPTSNTSHMQSRP
IEEPICSTDAGSSTTNQTIPETSLVEAMKSTSLTPVSSTTLPPLCELGPAESEPAPDTPQTDEVMIVVSG
DEVVPLRNILSNAVGDSRRSVRSWGPSSKPTLLLRTSHTREKERDPERVKLTMELLASKNLFLAVHHREP
SDKEIDDSILSSFQKYIQGQVMSKFSDAELTRAHRELETAEQSPENQLAFYDLWLPKLLEIEEYVQTPVL
RLGLFQSGYFQDHHSLKRPLNDNELSNDSTVFGRVRGSPALAALKKVVNHLILDHPKVQLQTEVCNTIAL
RSRTQAWCQSIPTKDQPSRATAVFPACHSLSCLTTGGCLFRSSTIIVGLYDDPCLDLLAVSQRLQHCAAT
GELRMLTESMENDKELCELVQSRLPFLSTVLSGNSYLAPRHPNLEPAGEVAAVTICGTERTFDHSGTYLN
LNFAPETQDINSPHYNDYLRRYALLRICLQQFGFTVIDHPASGNMKVELSSLTPPVANTFYQYVGLLTRG
SPTLTTIPDNTLAAQIKLRPSSPTQKIPSLFSTSGSAPEAITRGYISRHRYTHFPSTFLTEFSKRGPENL
LWKRFVDVQLAVYRKGPYAPGTSEASVLSEVQIRLARMPNPFRRCLDIADRQSKLTELNVVLHNNEEELN
YFLKHVFTPDVCGQTRQLCPVPYCCSTTCSDNVDGFSRFQAELDDYRHSELSEDTAKVPRHWKDVFTAEA
GKVVHLAEAACRLIDQGRHRPVSTGLESLATCLRSPPVILSLQENDWSYNDMLRMGVTFMHVVSGLYRRP
SKSGGTRKPDTKYLPMCAAFYFSNRRSQYFISLKEIIPTRLSRNYMKLLN

>E4YH03_OIKDI
MFFLDDFLELLEPFKNEFKQDIEQIGKLDEEAASFKRQMDEKSNEMFKRCQIKDKQGVKNIQEDPEITAM
LREVNVIKNKAKTAHEKKLEIITEISSKVDRYVRRCDEDLDAFKNEIEAEQPGITNQLEQRATDNTPELQ
RKNSVLGSDYEYDEKQFKHTTTRKKTSSASRARRPSEKDALAAASALESAAPSMLRQTSVPERGRKNSVS
AVRLDRSQSVGAPPIKAMRLDPGIPTTSAMSTVEVGVDGVTRPARTKKLTPKGVQYRQAIVSKTSQLDNG
FRVPLTESYNSTGSTQIVQLPSTSAMHFVQTVAAQKAGEIPDNANSSATQISPFKTLQIHPGMIQNINKV
QD

>E4YH14_OIKDI
MVLSRAIKIWSNLALVIEAYSDEVRRRQDDLLKRSNFHVIHPVQVAEDGRILKKNVLITSSNRRRRHVSD
DQAEFEDIFYKVKINGLEHRMELSRRDDFISQNFSIQFESSNNIERYGNRYQHCHFSGRFSKRKEDDHPS
RVALSNCNGLQGFMRFQGNDFFLEPLWNTTKEEHEEHPHVMIEKESSFMQFDDVNISGGDSCTIVEDDEH
KEKTVRSRRSLNSHRRYGLIESHENIVEAMVTLDKRVVMTHGREYVDLYTQTIMHIVSELYADPTLGNRV
KIVLVRIIVLVEDNPDLALVHRAESTLRDFCLWQNSKNIDSGLADESGILHHDNAILLTGYNICSQHDIP
CGTLGIAHRNGMCDPQKSCNINQDVGLGSAFIIAHEIGHNFGMHHDGYEDGSSKSCSRDVDKQIMAPQIN
SGAYPFSWSRCSRYSINNFLDSGEGHCLLNNPINMNGLAYTNDGRSGLVGRTFDADAQCRFIYGPTSRHC
MHGNICMQLYCYHSNMKCITNGIPAAQGTTCRILNPSYAEKGVTGWCYKGECVPDGFQPTPVDGRWNSWS
GWSHCTRSCGVGVSYQMRSCDRPRPAYGGKYCLGERRRYRTCNTQNCNPEAQSFREKQCSRYDSKRFSGK
YYDWVPHTSEKVPSCSLSCKPKDESWFFLELSPQVLDGTRCTNDPEKFDLCIRGGCEEIGCDRSLNGNKE
FDNCRICNGDNRACKLITKNVSICDDDAELDSCDREFPLSLEDIDKFLIPAEVTSISVSQKEGDKNLILV

ETGMGNRVIGTEYFENCENCKNKMIKYEAISASYFRVLMKEDFTRTWGVSSYGPLQAPMRIRLLPRQYSI
NLIVTYYIPIKREGTLGSLRWKFGNWTECSTECGGGYREKVPLCHLKAFPDSDEKIVDQEKCPSATQPDI
LLESCNNQACAPVWHMSDWNSCSQTCGSGFKHRTVVCKRRIDKYEEEVVADTECLSDKPEYFQSCNTKRC
PPIWTIGKWQECNPTCGRPGWTTRHVNCVDSTGTDPEVHLDMSSCNQALRPSRRKRCHRPCPAPQWRTSE
WSECSSECRTGSMTRNVECVDMRDRILPNYRCNMSTKPVSSKTCKGECMQPVVIDGNDDEELCVDTPETT
YCAMVLRFDFCVKSFFRTKCCATCSAQDDLR

>E4YH19_OIKDI
MSVGDVQFTFPQVNHQKQPGTVELSMTHLKWSPDMIGEVVLIEIQKVKRQKISPEGKSKVQIQLELTDGR
SLTFHFADDDPKATRNKVKAHLGKLMGKVLAKKVDKNLEERKRVLQNNPNLVKLYKQLVTTGQVTAEEFW
SSRAHMLDSSHEQDIGITADFLSEVKPQSDGTNSIRYNLTPEIVKVIFRTYPAVKKRYLEVCPTELSEKD
FWTQFFQSQYFHREKLAGEDEKSIFANCDSLEDKELSNQARNSLVNLERIFDMDYMNEYAENEITQKLND
KKQKAALSKKEAKKLLVKRINNHATQVLQSCQKREASDDTNGVQAKRMADDVRAHLSENIDLTKQNETTA
VPCIIVDKESFFNWGAEDDKKSILNRMDDKQVLDLKIHLEEKLNRMMNNIREFEPKLDSMEDDYRERNEC
SQMREMNDVMNIVHVAIFNLFVVFALVFCGGFIQSEYSFYWLISDAGLPSNISVTCANNATLAECDVYTK
DVVQVLARYGEYSALCSFGTGMVGTIIGTVSDVIGRKKVLVANKLGYFISIIVCTFITYYHLHPYYFLIP
STIWNLVGGYSVMLGVTFATVTSYSPDEKRFWYIVAADVGISIAGLTVTGIGYLINTLPTENYYMVIAVG
AAVMVIPLFLAIFGMKENVLPPEIAALDKLKKCFSDIKEIWFRRSHLNKFLWLVVLVPALDKLVSFGTSS
VTNLYLQSAPFAWDPEELSMYISASSLLGILSFILPGVFRHFGISSFAIIYLSLLMNIGRNIMFGKATST
VMIMCTLGFNVLTGSYIPVIRNLLAMNTEKHNHGAVFAFLAALEANCITAGGLLYSYLYHEFPVDTSWLF
WLLSPAIGAIGFVFAICAHFVYVEKIKFAEDQRCLINEIDDSASESFR

>E4YH20_OIKDI
MSFHLPKILYTEQKQLPPPLKDYVTLNVTQDEIVIHHIRICAASLRHPQMAGSMNKTHKASMSDFLNYGD
LENKIVDWFGQPTLDIVKERAKGNIDFLNRLPEEAKIAILTRLPLEELPKMGLLNKEFAELCKSNELWEK
MCEQNGITIDENVRSLGLQIGFKKIFFSNKVEIQRHIRKMKKQSTFITA

>E4YH22_OIKDI
MCHNFVPLVNVQEQSSNEEPNSDWGSYLTRGLQSAASYLPSGVSEVLQQGRDFATAKLHSCGLKNISTIH
EIGRKYYLFVACSDGHLYVYEIDPSGGECNLIKQHKLCLPRVPEVSSEESDLPPMIHTVDS

>E4YH28_OIKDI
MRKIRRKIINSGTSKSDSYSKAIGTDSPGIERIRPSRAPPGQPISEESSSEPADDRQLLDYYLTKRKSEY
SSPCDVNLLAGSFNVNGKEPKEALDHWLLRQNETPDIIVIGIQELQLDADAYFSEYVNAKESEKYQKCQE
SWKTSVMLALQKYNRPFQLIVDERLVGMYIMIFCSERIKPAISKVDTANVGCGLMGTFGNKGACSVSLKI
HETSFCFVTSHLAAHQNAIKKRNQDYESIRHRTVFRERLRILDHERVFWLGDLNYRLDIPNDDVRFICQG
DDISSLLPADQLHAEMNAGKIFKGFHEAPITFKPTYKFDSGTNNYDSSPKNRIPAYTDRILWLTKETDSV
ECLSYSSHPSITISDHKPISACFRVRTEQINKQIYYSVYKEVNSWLDKHENELIPQVAVSSKAIDFGKLK
FLERATANIQLQNTGKTPAQFQIIDSKVFSPDRDVYEAFNSNIGLSIDPNKGILPPDSGDASICTISFDL
NINKEVVSKFNFGVKQGGNYVNPLDDLILILKLRNGPDHYITLSGDWTYSAFGMPFIALTHSFFKDVSIS
SLEYPATCLVLNQLVKAECSSSNSPSEQNLHPPAFYVLLNHIVNHGFMERQLFQINGDPDEVRAIIDTID
AEEVVGNLESLPGSVASVAEVLLLFLNSNPFPIIPYSHFELCTDLVDDHNLCKSMIEKLDLRSQNNFQYL
IMFLKEVLKHGKINGCTSEFLAGLFSRCILRPQITLKNREKADLEKRKSVQFVRQFLNEDESSLI

>E4YH30_OIKDI
MSEFGGRMLFSGANYRRDHQVKVNPPLSEIGYLTRDDPKNPSKKYRKNESAGEIGWAVENLCPEEQQKPT
NIDGKFFGFTKGIFRRAADLRASHRHQSPYSEPLKTTKLPSPLNNTKPRITNATNASP

>E4YH32_OIKDI
MVKYSEVPMGDKSDLDRLTDGQEGREIIIVATWLRPTKLIGLIDLFLALICYFLAEILRSWVHPISDEQR
EYYYQGLWQICSVEDDVLNRCIVDPDSCEEIEAECAFYNSAYGLWTRIIFTSCFLVAFVSFIAGIIAYSN
RERKDWYKYLGWILVLTSLFVLVSLTIFPSGFIRNFPFYTKWFFGLGYGFGWAALCLTLLAGVLFILGHG
QNKKKRFVAEKRIRI

>E4YH33_OIKDI
MTSILESRVVGMKWKLAFLTLGVDSRSHEKGSRWALKQQRIELKYEANVNHNMQSVDAFAGRSGPSRPEG
PTLLDGGWTPCESNGAVSKKPGKKQTKFHTCVQKQGFGEDVLKCVNWKKGGGEERSADEKCKNACREVVF
EASKKNLVKAVAKCTDMDNLVNKLKQKKPETLATNGVLMFDDDEFASSGAKKPTKLTSSGKSKSPVNRGP
GRPQSESKGSMTFGDLPDELLLFSDDMEDFGEPELMPERMIAPQFESPKSSPNKKGKKGKKGKRGRSG

>E4YH37_OIKDI
MKRINFKNKIIYIFALLIIVTVFYQAIILAKFSDVKQARKYIEENSKTCDICADCATFSQKNFTQEKILI
PLLVWGPNNQIQGFRESLYIANALGRKLVAPPFFPNWNHLANGSSLIHPWNRIDTQLLSKHYDIVSPQKV
VEICGPRIRLNLSTGIVSYNPNRYSIFKKYLGLDSWSNAFVFPISDKTLSPEEENRPLSNAIDKTYLNSI
YKSDEKCVILEYPWNSIPAYDIFVQLKSQKISEEYKEIVDIIKNTKRPAKGSKLKAKLKINFSNTINTIN
MQ

>E4YH39_OIKDI
MQRNCAQKVVGGEGSGILPEVTSGRVVVPCELERDVLLASSEGSSPMSPREVFLNSRESPVDRSKVRLTT
ALILGLALLL

>E4YH44_OIKDI
MPKDRNKPRGRTTAYGYFVVDEKEKHAKANPGVKINFGEFSKLCGQKWQTKSEEDRIEFEKKASEDKIRY
EEEMASYEPPAGTKKTKKRKKDPNAPKRPATAFFLFSTANREKAKAQLEEGAKVGDVAKKLGEMWKLVSA
EEKENFAKIAKESKAKYDKAMEEYRANTPKESPPKRKKKKKVSSEEDEPSDDISDEDHDY

>E4YH50_OIKDI
MSTSHLQWAIINKFSAFDVKRAGRTWTKEPGHLNGRKSLRFNTLVEKKGLKVAANAEGGVNFSAGKMSTT
MKKSSRKTFKSIRNVAKQGAFRKDQTRAAVKKASAIIASQKRAAQK

>E4YH56_OIKDI
MPLISKLPSLDLKKVQSNPMFSNPHGGAMGMSPMMLIDQNRLLAQHQLQQQLQQQQMMQQHQMQLAALNN
QVMMGNPLIAQQGLNPSLPPSGYPAHLFQGTQPAAFDESSSTVQKIIKIALTNKSFFII

>E4YH66_OIKDI
MKLKNTIFIVLAFACPDDDLQKECINLCGEEYSHCGENCNGELNCFENDFCTEKYSSCVRECPCEKNCPE
GCVNCENEICKRAPFLTVIGDQAFAARSSDLKKIARLDDCEFKELPVRLIDRFDSVSGALLSFSNRVYLC
FFNYPYKNCEEFDGISSTNSFLITNHVHCFGGLGEYKNQMVVLGAGDLPGQEVRRKVEILEIGGWKEISD

FPVDITVASTVTVPEGMLVLGGWNPGWISGGIYYKSVYLLKNEIWSFVGNLSEPVSDSTVFRMSEWIILV
SGDSENNFVERFKWDEEQIKELEIIGENPNTILNPIVFQSSRNFCNTV

>E4YH68_OIKDI
MKISSVFIFLSATNGQKPAELKVFKTQSKLEWVIDTFYAESFPKWSRGMKAKTKRVGLKLVDEFKRKQTE
MESECFEGSGDGPEQKMAVEIEQENVANLRFVQDDPCRGSKQLTRNMIKWANENILDYGCDGAPKEEQTE
NGRKDRLVKKWEKMQRRFQTRLQCE

>E4YH74_OIKDI
MIFSISLSFFVKTTLKTSLFANPVVSVARLWSFKDEQLKGFNVNSIGFNSVGPEIVAVEYGHSQRAKGAS
KIPGIMLGELLLLIKVKRTSKKTKKNRSNTRKTDNAVISQLSPATSFNFNPTDGNT

>E4YH77_OIKDI
MTEKEPMLMCLRHTGRNHGAIYWGKYNVTYEMFPPAYKYPDYCNGQCAVMNRKSLEAIGAEVGKTYLGDF
RIEDIFFTGILREKAGVKNLQDFTRTVSELTSSSGITKNVKTKTCMHYSKEMIMTPKAHLIDDRFYPEMP
TGVTTKRMFEERKNFFKRIHREKLKETKTLEHEKKEKKAKEDAKAQLLKERNLEVAGIAPQNLGFHPINW
INHHFVRKN

>E4YH78_OIKDI
MELKISDSTDPETGKPMYTVAWSENLADGLITIVMTGKADNGTYTIYVENDHDSPVSVHINVVSYAREPD
REPVTLTPTLIQKKIAPSNTDIVVIYAAFAQGEFPILFADLWAEVTFPLERNGTIMDTETVILTDDGKHD
DVIANDGIYTGIFTNIFETGRYSIKVRANGDNGKAVVSKGHMLGIGELDDAEYEVVENAVHKEVWQTTQR
RKRRNVRTTRDRREKEDEEYEYDYEEVEEEKSNEDTTVAPIITLQTLPVSNQDKREYYKQLEVEKLARTT
RTSAQDTERVTNMDTIIIDTDDSDLCDENFKHFDGFCYKMVLDKKLNHPEAEAACQAVGAKLIDVRSLYV
AEYFLKRLRNVIKDFWADAVNDEFVKYLPRLVDNSLRIQEIQALMNKAEKRMAIERRIRKEDEKNPLARM
GSIRQFRQPIFERDMKWIYNNVTSNAQPPAAFIKDFILQKYEKSIDYATLSQIEEATASWKLNLPKKLDH
FLSETLDFETFEAQSRFSRAKRDLITVSSPCRYKDDFNRYRKNRKFAICNSERDVICQKITLRNVRKREI
SSCEATWSDFASNFASSEICYHQASKKHNFAEAEKYCKELGGSLLRINCENKEVFEIKEENLDSFWISNM
DTSSLNELKCSQTIEKEEEESENNEILYLPIRIDQKWPIRNIGQIHPFFNQSEFKEVDDEIYDDDDDNDA
LDASDSNFLSDYTDPQLGDVYYCMWFENGAVNEVYREGPCLNLKKSYICQKPKNDLLAPGPITDIDVQHF
VGSYKDPLGSFHIEFTATGNDQSIGQADRYEVRMVATEKGKEALREDFDKGYHLQDSDFSNEIDSRPDRP
KRSGYLERQTIRFEDIASCPRNQLMDLMVLVDTTTVMKLLY

>E4YH85_OIKDI
MQKFLFLIFIAAIKAEKRCGEDIKEISKDGMSSLHRAVHPLIRPDLECVKQLLQKGADVNLQTSQGSTSL
HLLFKGASDLKLTAAVLDLFLEKSADLEIKDENGREAVHYLYENVKISDEFKAEVVKKLVSEGNHVSEEM
PWLGIKLPFLEKKLELLPGPPIPAAVKAVFEAENERFDRIKIAHCLAENEKQAAKINELSVNNTELEKQA
QNLNRATEKIAELEVELGRFREVVLQSETKLDKKEENIEALSEKNKQQEAQLEKHLEEEMRLMRKIEELE
KEREVMQKKEAELLEKAHAKEEALEASANQFKAMLRADFEQESAKFEESLQNEKALVGLLSGELDAAKMQ
LASAKNQTCSSKNVTTDVSSCLNDLDLDEEIERLTSEKRQIVAFILQFLAVYVFWKLGRKFFKKAEDPEH
SEIPSELSRYIEKLEKTLEYNRGLQRVMNLSLDRKDEKIKSLETSLRDQSAMLFPSETDADETTEDPSSL
DSPLSF

>E4YH87_OIKDI

MKLLANFGIIAVTNGLSSKEWRTKRVEKSEKKNAKVEIFRIYQEDLSPKKNVDGVFILDWWGKKEAPPFL
KRLTEDCGGCYLTNDQKRGIF

>E4YH88_OIKDI
MIPEFPVVPGRDLNDGTVMPQFGLGTWLSPKGDVEAAVLTAIQLGYRHVDAAWVYGNEGEVGNAVAKSIK
DGFVKREQLYLTTKLWNIFHDPKDIDRACDMSLKNLQTDYIDCYLMHCPMHFVGPDDGPALLDESKPACA
ANGKKPHLIGDADYLDTYKAMEKLVQAGKVKSIGVSNFNQFQLQRIMNNCTIKPVVNQIEVHPYLTNDAL
VNFCQENDIQVMAYSPLGNPSKPVTRVWDENAKTILQDEKLLAMAKKYNKTVAQICIRFGIQRGLILIPK
TTNPARLQENAEVFDFALESGEMDTISAMNQNFRVVELPQNAGVRYYPFVDNYSE

>E4YH89_OIKDI
MPKELHDRLHVPKMACKPQQYSSHQTGIFKAAFEDDIEAVTEFIEKDTHLNIRNWRGDTPLNCAIRGKAE
NTIDWLLDNKASPEFGGWLYEPPIVAATAINNESAVETLLRKEIGTDVDGVDERGWTAVLVAAKAGFYSL
TKTLINFGADLSLRSKQGDSAAYWVAKHNWEDIYERITQESNFKYVLQDAMEFAEEQDDEQLPALLKNKD
QNVNTRRPPGSPEGPPAKKHCQ

>E4YH91_OIKDI
MSKETKELKTLDEELTKLESLPNRNLAAEKRFQLIAANLPAALLNKRIWVEERQNCLNVSFAKCSDPMAI
INACQAIQIDAAKMSDLYGRTLLHVAARVPDLAAADKLLKLGADVN

>E4YH99_OIKDI
MIRKAVRGSCARISCRPQSVTVNKGWFRPKVQIERVDLERYFIIVEDPSHVVWQKLTEISVIEDRYQIFD
QNGVDRNIFGDVVSRFPEILEYSLENIDNHLKFWSKMFQNSSDCSFHLKSYPEACFHLDNAAYLTRRNEM
LKFLFSNFGINQKMSIRLILAYPKFFNNEVSQIATKVEMLKNLYLSMGGKNFKVFGRTQLAQNVLILEKP
ETMSDFIDLMTKTGLSSEETLKIISKMAGVLPDLALENVEKTMQLLREELKLENSSEMKKICLEAPKMNR
MSFKPGAMLKNAGRFFERMTSDDKLVNDCQKAFEAENQDAINEIVRVLRAGHRSPEKLEIRELSGGYFGE
TRSLSHTQGGEVAGFTWNKRYITFYADFFVLMELDDLENWEKENGPNKDMLADYIFLPDVNFSWKRMSEM
EIMVTNRSRECTIRVKQKEHCSVILQHLCALKYLAMNNLNGCSSPIKQPISESSFPVRPHTPAKWYICGD
AYMSNLADSIEAASERVFLADWQISPMIYLKRNYEGGAFSTSGDTGTYWRLDQVLKRAANRGVRIYILVY
QDPTALGLKNYEATKYLREKCLWKKHANLFTLTHPTLDGPNKWSHHEKLAVIDDKIAFIGGLDLSMGRWD
VHGKYFMFDPERRTFKGFDYWSQFNSKPNQNLIDCNYEKGENFFRTNCRFDENKDYLDRMTEMRTPWHDI
AARLQGEAAFDVSLHFIERWNMTSHTAYQSAVKINLPVFRKILVTDGMPLAGGQVARQIDEVNTSCQIIR
SAAAWSAGLRSTERTIYDNYKALIESAERFIFIENQFFVTTTGDYKLDDSLPQNQIALFLCDRIIKAFKK
GEQFRVYIVIPCIPGSGGSLEENTAAGGQEILLHITYESICRHENSIYEYLRRCEPTIEVEDFIYFASYRT
HEEFNGGIHQAVVYPHSKLMIVDDRFTIIGSANINDRSLLGDRDSEIGAIIDSSPFAKSLRIRIWAVALG
DPEHSLETSFPPESKEFFDYWKSRAQFNFQLFEDVFCVLPNNTVFALPGRFEAESEDWKKWKKQYDNRMA
ITDVEKAKYKMSQRRGFLVSHPLDFLKNEDRVNEISDFFKNPSAAFTKEGITQFLDGTMFQ

>E4YHA2_OIKDI
MGSDFVEKTQQVLGKIIQKPALTEKLLSRPPFRFLHDIVMNLMKKTGFFTGLFPKELLDGKSIADKNDKL
KFLQLTIDCISIVSGKTVDVKAAKIVAGQESEQTNKLLQEIAMAILTKKSSDEAVAQIKSGGKAEKTEKS
SSKSSNKTKEAEKSERRGSRDESKQREKKSDKRAESKPRDDKKGSKERRDKSERRDRDKSKKRETSEERR
IRKEKERADRKEKERAERKDRKKGGEEKAERSERRSSRPVRPTSAKGERRRPPPVPENDKEEKKKPSKTA
IEETSARPTTKGASRARPGSARPTNKGRAQAPEPPAVVNPNVIRDDDDEEDDNIEIEEVQADSSAIKGAV

VAEGDHGALVQDIMESSKNMSKAANVKDKTNKISEAEQKKIEQQIGKLKADVQAITAQLPPVAHVLDLFQ
EDLDAMSLEYDQWSAELKKNTTLLNEAQNRNHANLDSLYSQLNRFQRETEQREHEIMSTRQQIKINEDRI
RKQMAAVCAN

>E4YHA7_OIKDI
MTVVGEIAHHTADIIAFAWGFVTYIPWFVISGAGYKLSMNKRLKSREIKPAVRRAVENTVLIHNFDQQHD
TLVDAFRAATARFGSRMAIGRRQLLSEDDELQPNGRTFKKNTYGDYQFKTFSEMETLVNNTAAGFASLGL
KKGDKIALYMETRSEWMIAAHAAFRAGLTVVTVYASLGEDRVIEAIEESEAKALVSSNALLSKCVKPVAD
AMQGQLVHIINCKDENNKPCQVDASITITAFGAISQTDASTFEAPEVSGDDLAVIMYTSGTTGKAKGVMI
LHKNICAAIGGLTSRLKLSGISFDDDVYIGYLPCAHVLELAAENTVLLNGSRIGYSSPLTLSDKSAKIKT
GTMGDARALRPTLMAAVPEILERIRKNVDQVVKQGSAVKQAVFKYAYNYKLDQVQNGGDSPVLNRLIFKK
TRDLLGGRLKAMIAGGAPVEAKTQQFMSICMSCPLVIGYGLTETCAAGAVQDAYDISSGRVGPPLGCTEI
RLIDWEEGNYTINNEPPQGEVLIGGPAITAGYFKMPEKTAEDYCEIDGLRYFKTGDIGEWDSDGALKIID
RNKMRHGEYVALGKVEAALKTSGAVANIVVYGSGTILMPIAIACPVEKVLLDWAKELGVEGDLVEICKDD
KINKKMMEELQATAKAANLSKLEIPNNVHIDGNFDAGWLPDTGLVTDAFKVP

>E4YHA8_OIKDI
MTVYHDEIEIEDMEFDEETEIYTYPCPCGDKFEISLEDLQIGEEVATCPSCSLLIRVIYDEDQFADYDSD
SEMPAFEKLKKMRADQIFGVIKDGVPQEFIDGWDFIGKLGEGAFGDVRLILNRKSKIKCAVKIINCENMS
ENQKKSLQREKNIHRACSKHPNIIRYINCRYEDRLKAMFIFMEYAEGGELFDKLVPDIGMPEAQAKFYFK
QLVNGVAYLHKKSIGHRDLKPENLLLTRDGQLKIIDFGSAVVAVKPDKTKRRFDSDTNRVGTPAYMAPEC
FLDHYCPLEVDIWSMAICLVAMLSGQLPWSKADEDEDEDYLAYGRQQFSDDACWSRISVPAMSLIIQMLK
FNGEERAKIEWMNDEKNGPKWLIMDHKDDVAFKYQHQERVKVYKGKKFEDFYGQKSISQPEMLTLFGTKD
NLTKFDEFETDEPFKYVLKFNEMESSTQEQEVGGQDSYSQPLDDISKDVLKGEKHPSDRNNIRVPRITRV
WFEPHKKTEIIAAMLKYFISYQSVEQIPEKYWSFESPVVKLEVRILKTGDVYMLDCKRCKGCGIHFKRLF
RDFRASVEDILAKKKIQPQQILTFDETPAGIIPQVPAEDGNEENAMVNSPNDAKSSKHKLEDNEHLDNEP
QAKRAALQEA

>E4YHA9_OIKDI
MPEISLKRSNSSLPPRPLGPRRFRGKQWRSEPNPDSVLESINPVSISSPYRSSSNDSVNSADKNQNNSLP
RKRKVVAIRPKPDELELNRPPTISKDSEKRVAILSEVVDTEQKYVVKYLETLLELFIKPLKSLRLCTQQEI
SQVFCNIEEIYHVVSPNQAYIKFMTNYASNESSWKNLAARQSIDDFLTDAYSQLSREDPPIKQRVEDMLG
TIRDRFYKYKMFTERLVKDTPEHHDDHKEILKAEREIGVILTRLDREVKIAQKNARNLVLVSLIEKAFEN
DFSASGLSDEVRNLKYLSYDPITIQTSKNRGTYYLWLFNSMVVISQISSKQTKLTVPPEVHQEFPEVDLS
HKYKANDYFLLQDTSLKEASKSQDTDDKLKRSQKEIEILDNAIEVLEEATRPDIMQKQQELKKLKKDLEK
ELRDLSIQHNTSIEIEVNTAEKGLETIPFEFQSIDPQKRYDFKKLFEAAKKESIGSRRAGPTMSHCLPLQ
KSRNGQDPSAAICVNGKMWVLSTSERQNSQICILNESKESSPHYEICDAQVNSMCHVMASDHYRLQRARN
DDSDNDSDESDCGVPLSLASTNQYVPDDCVWLGTDDNRVLIFETEEDAKRKSPRATINLDAPVLYILVHE
GHVIISIGTTESAAALCIFSRVSAGWKIDEWTTFSVAKDLCDRERKDEFLPMISCGFSKMIFVEGVGGFK
NEVWAASGPNIFILDTENNYKIKKVFDAKKSETSGSRPLQITAMASSHGIVYIAVDEKCKLYVLDGGFRS
ELDISPIDLDSHVNRQLNAFSTGKDDIIRQHKKNRLRIMCLLMTSSYLWVGTSAGVVLNIELHSGRPTTH
NSQQLIQALSHGHTGRVLVLAALAVNKTQMKKKQAEHRRRMSKTAAQKKIYLVSAGEGLENFTQNSCDAI
GKHDATQHLIFWSRA

>E4YHB5_OIKDI
MICSCFAVIASLFAISAHSESGQVRVRSTSRFRKSYYKNEYFEKPRKGACVNSKWAYGTSCILSLICTGS
AGFGAYLSIIYSNKAENSKTTGIHPSVQQYLGIESLTNEISILRSCLFIECACQGFLALMSLYNVYLCGR
LVIYHSRYFIRVSQEEHVQNNSVEIDASGDRCPSEHLPHATYSLPTTTNLDATNNYADSVFLEQGPEPSL
VPLSSCLGYTNFGPEMSRTNSFGSGKSENASESDSTQIVQPSAVRQPPVKIAPKVDNPLPRPGNSSLRAA
GRRHRPEKSVSFRGTALVAE

>E4YHB8_OIKDI
MTMKMELLKIFLLAQLANVSAQQKKECRSQAGGAGPEIKYVTSEGYPLKSKAVEECVWNLQVNYGQYIKI
VPSTISVPCAGGGGVVFKELLKDGKKERAVSSKMYCGANTKIPAITSTGVRLEIYFKAPPGTKLKIGYKM
TETSGGTTVAQAGGARAVVGGSIKYNPHSKHPGHSRPSTSVLSHSREIPLLGSKTPPPKVDERNPLVFLL
AGVVVCGLVGALMYKFMYKDEQLAEEKCPGEKPLEKKAETKEVSNSAARTCSQETTATNISSD

>E4YHB9_OIKDI
WNHTERCLALLKSSLLGCSHQPQKDSKNLNHYSKMKTFAFLAATAALAQKKNKNNYNNNNNNYNSGSAYG
DPHFMVSSNDQPAICFDYNPEANTQIELLRDPATSLEVMAETSSSGNLHHEEQVIMSKITLSTPSGAKIT
MDRDGLHLESAASDVAGEPHPFTGVFEYADAQIVEHKGANGGIDRAHIQVGEAVFIIKATKKGSMSFAIR
EPQGLSPRSTGLIGEFLEPNAYTVKFLDEDNAEIILNGGKLDATKKEFHRAAECWVVEDDLAQFNDLIKE

>E4YHC1_OIKDI
MSYSDFPSFHLAFFEYLKITALHHCPLLPFNFNATVVRIYY

>E4YHC5_OIKDI
MESVLLDDWSLIWDRTNNWIQLKGINGEFEIESSPVEAVLGPFVVQTGHSLYKLGVPSKNSCDWIVPKDI
RDEFSKGFPKEWVVKITALQERRVTRHTPKREGPIREKSSTLSKSSRISEKMEDSLYRGMSDKQKERARK
LDRLRALQKRFRKFLNLIFASEALLTPHFVFRVEFPYLFSEFRNNI

>E4YHC8_OIKDI
MVCVELTLKQIEFLLVRVEQIKQDENNDDFEENLRNRNTTNLIESEIQRNARGIVIRCGDATVKGRIAAL
ASNVDSGDTPIAQEIEHFIHIITGVAVFLGVTFFILSIFLGYKWLEAVIFLIGIIVANVPEGLLATVTVC
LTLTAKRMAKKNCLVKNLEAVETLGSTSTICSDKTGTLTQNRMTVAHLWFDHKIHEVDTTENSSGDTAGD
ASESALLKCCEKSMGNVEAIRASNPKVAEIPFNSTNKWQLSIHEVEGKNLLVMKGAPERIVDRCNKILVN
GVEEEKPADFQTTFENANAHLGGMVGFKFDTEEMNFPSEDLVFVGLMAMIDPPRAAVPDAVAKCRSAGIK
VIMVTGDHPVTAAAIARQVGIISDGSKTVDELAKELRELDDSDLDEILRNHPEIVFARTSPQQKLIIVDG
CQRAGQVVAVTGDGVNDSPALKKADIGVAMGIAGSDVSKQAADMILLDDNFASIVTGVEEGRLIFDNLKK
SIAYTLTSNIPEISPFLIFILFSIPLPLGTVTILCIDLGTDLLPAISLAYETAESDIMKRDPRNKSV

>E4YHD2_OIKDI
MNSEDKTKPKTIKVKEREFESSSSSSSDEGIQTRSKTMNKIQYRPTCEATLGGFPKNAKLTPAQIAKLQH
DHKLATEIFNQFFNPDQDRTKKEEKKVKKTERKVKEEPVFGDWDLNVTKANEVEVKEENLDFWQDMIDE

>E4YHD4_OIKDI
MDLETVLEDVEFEEEVYINETELEQTYYAGPEETLFPTVPAPPPGLAPIPTTLHYYFRRLFIHIEYFFVE
HWGYFLIGFGIFLVFIIVPGILLQVYCDRLYRKYKREQITRKILEYEAEMERDGADEDNRASRRYQASLE

SSSSSEESYRHHKRGRRSARKSGKKVRARSRPPVDRKKSPEKTPESSPVAEKPPALPPKESKKEKQEAKK
QEEEQKQGSYHHFRHGNGKARFELIRDSGVSMWDQETREIENDSRLQSAENSMPFASTSQKTVNALPYPD
QLDHFQTENELSNDFPEHNYQETITASTVTDHRLPTMREETAPSSDHRGAYWSAGIVSQFNGARTPPRPS
RSRPEPHNSTQITYDEIREMKQVMPHDQEVKKPSEIQNYNDALNSLSQQNDDTLFQKRAGSSMIPNLMPA
PEIGENDSEPPPLPPKESKSILTPVEMPKLDLKRETHLVRKKIISFF

>E4YHD5_OIKDI
MKNLLFLFLELILARECLERSDPLGLNYRGSQDRTGAGLRFPIRRCGAWKPKSGMKHNFCRNPDRSSQGP
WCWVDRITDPRDEFKSQRASFTFIENGRICMSTFEMCPIEQSRKRRTIDYELDDLYDAEHDYNTDDLSDD
FIEDHYEQLEEQRMNRTKNRNTHPESSHFIGSMRVLNGETGALPFFVAIRTSTNIHFCGGALYNEDTVIT
AAHCFGDRGTRASRFRVTMGHEKRKWREARRERFFQEFRVREVILHKGYNTRDFKNDIAIMRLRSPAKFT
DYVRPICVLPPGYNLPPSTNAIVGGWGKNEGDEFSQVFKFATAHVFSSAECNRFAGADYTRNDGTQFCIG
YEQGGIDTCQGDSGGPAIVKIDGSWTLIGVVSFGDGCGKSNKPGVFTNIAHRSISGFLQNNL

>E4YHE4_OIKDI
MLLFGDDKNEIDHCDKPCQRADLFMSALELEDIFGGWRPLMAKDDELINIGTCSGYCQFSSRRLRHSEVP
EFLNGCATISFSPLTIFKGDMIRIIDNMIVKNCGCQRVQYCS

>E4YHE5_OIKDI
MSVVEEDAQFIKDFNEFFDDKFKIDLVIASIKNKISREVDDTNKNVSKERGEISRKEETIEVLKKGVEFL
IAERDSEKTSIAYTKWSNENIDAVESAITSIKTKIDADFRKIQSIKEKLLSLKETKLLSDVVCNEIIPSC
SICFERYDKMDHSESALTVCGHKFGKSCIEKSFEKKKNCPNCDKAFEKANILVTYD

>E4YHE8_OIKDI
MAIRYPVQDYLSISIVNFYFQKFLSLPQKTSLLPKIFIAIFIILSGLYFMPRDESSKSSNLNETQNDENA
YLQPLDVPSAVRSLSKQDMSRFWGTYRSGHYLGLRTRSEKSLLAGLIWLNNKSPRNEIHGDILRHKCNQD
NQLKYGWKEHDGENFGVQSVEEKNYKIWTKFLKQQVGGHGGDWTVKFETEGTGTTTFAFYFVTDSDGYLK
VPLGHDSADELDKFVGETSSLGKFEVRFLHDAQDRAYSRLQENTAFWTYSDYLNKITDLEDDVMRRLAGE
YIELNNQKVPHFWFDEPKNDKNYRKPNHKNHQVVVHSITCEHPCSFEVNFNSISGNHERKRPLVGEFFDE
ELARHRKNFKRVFGSRIKLQEEFSGEEHLEFAMAAVSNMIGGIGYYHGHSLVQKKGEKVTPNWDAPLYTA
VPSRSFFPRGFLWDEGFHQLLVSRFNPKISIDSLCHWLDLLNKDGWIPREQILDDEARQRVPDPFIVQGT
DRANPPTLLLALDYLVSSEKLEKEQLQALYPRIKAWFSWFNTTQAGPISGTYTWQGRHIGSENLELNPKT
LTSGLDDFPRASHPTKDEYHLDLRCWMALAAQVLGKMAIKLGQSNYEYLQLEVELKDNTKLNKLHWDAEE
KQFSDFGLHSHNVKLVRKQVKDENGNPRVIKVRKVIEQPRLRLVPHTGYIQLFPFLMKILESDSEQLGAT
LDMIADPEKLWTDFGLRSLAKSDPMFNERNTEHDPPYWRGNIWININYLAVHALNFYSDEPGPYQTQAQT
LATRLRQNLIGNVFRNYKETGFIWENYSESDGKGQGCHPFTGWSALTVLLMEETKF

>E4YHG3_OIKDI
MDQGHAPTTGHLLRVQATLTSNTDHSVKGAKQSANVISPDKEPILIMRLILNTRDRKSAGTARHSKIF

>E4YHH4_OIKDI
MYKLEKRIKLEQDDNIENANKKIDDLHHTLHTSICHINSLHNMSKLGLQDLEEARTEFKKNFAKEKTVTP
SMKEDVGEKTRISIEETEAEITAIRNALDVIQNIRALQEERKLLSNEPRNTRRGVLMAQLQEAAKCTPLW
IGGPNDSKPPLCGALPAAAKHEIVVGSKVAAKVDGAGENEPPNWILAKFVSYDSANMCTVEDIDAVDLTK

AKISLNRKRVLPLPQWRANPSEDGDALHSEGSTVMALYPQTTCFYKGVVASIPEGPSDNYIVLFEDATYP
NGMSPPMQVAQKYILAFDNKPDKNPSAATAATSKKGSSKSGSKAKSKKDMATSKGKRKR

>E4YHH5_OIKDI
MSQSNLTSYFEKLGPLSSRTSPVKKTANENDVESVQPVEALLKKDVVENEPFVKTQPQEPPSKKRKRKRN
RKKAKQQQESSGSLVITIDDSSCSSADSNLLPEKTSPKGTTKTDQKYLHGSAKDEQANEPKSTKELEEKE
LTAAAREKEEHQPKAKRTGSTTKDPSEKEWVIEAILKHRFIDTAKIEYFIKWKDWPSSTNTWEPLEHMVN
CQKMIDDYHEKHREDCLEHNRHFPSRPQECGFGRGLKPEKILGFCDDFGDFAMLIKWKSTGSNTFIDVVS
ADEVYEKAPVFLCTYIESVISDQFMPDDEGTSEDEEEQLDSNANQDRDENGECSQFDDVCKVDVLKDPVI
LGNVVLNRKNYFTMKEGKSADESQYLVPTKSAYKNFRKLFNFLLLFLGGALAESTCNAGSVDRTKVPKSF
GADVTESTLRKVWPHIHATHPTVNFWDWYKFFTTYAAYYDHGKAIVGDIYQVWPIDINSYGMTRDIASYN
YSLPRALDLSWNEMVFDQLNIPMASLIAANIFYDVEVGFQNHFEGGWNTFSTNYGGTNQTRWEELESSFS
FEEYFDHDNVDLLVVVDDSSLISDSDFEEIKSFITNIQTVAGSSSLDISVHAATGGSFKIFQSGALDLSS
LAKSDSTDRNFDAIFDAISDDFDSSRSDAAKYVLLIVTGDPTDDVSRIHKKLPGHSQLIIVNIGSQHITL
EDDTTKNVYASSAEELNEDDHKVVNLLLEVTQGVIHLWNNGATNVRVHANSKRPFCINTESLGEITTSVA
AGDSSVEATILASINNYPSYALFDLKRPVLTGSSSLIKIGPSFKTTYLSIINENDEDILIDFSLNMRTPL
FGFYSPDIDLSNCNVHEYCSFDGTCDCISTYKRTRDQECTFSQFIKRPDLDQFVCDIKDDVVEFYINLTD
FIPPVTDPRYGEDNRGFCFVDNSTINVLMTKLELAGSSNNTFGNKSCDCSAWKNGKPGELQKYTSADCPD
IIKKELVEDEAGLLKEVYHFGVGYDDATIETGFGKIMVQPGGRWKLSCDVKRSAAVSMADGEGGTEITYE
IENMDEFDVENNQDEVGFDFEFFATADLEGNRRIGERPLLSNQNVYIQTRISDNNLMKVFTSYCEITVTT
NGTVEVFEDILNSGCISAQYEDSFLRPEGFIPSDSGADKFLLRPILPTGFQEASVNVACTVVACPSIGPN
EPFCEPGPSCGDIYSAQTLRSLKRFSNRPFQENVGMTLEYKQSTVGEDDGRYSDDEESSALSTCLSVTFA
LLTIALLG

>E4YHI0_OIKDI
MDHHEFENFIASRDNVDLGKWNPINSTYKVPIVVSPVEIGRGHQSEAGFSSQVPESPGKSSDEVFKDLIK
TSDPVSGKLVDDLSVAFIRDTLQASSLPALVGKLYAEALITIPIESLSSAVDHCSTVDDYLKVGYEIITC
WYVICTAYNPAQNTSVHDLASHFLKRLADPKIEHDGIKRFLRGMILCTKGVLHCIRVIATRKEFTVEELK
EVHAVQSLFNASAPLFSGSPTHGQKFRNFVTTLNRIRWSSIHPTSHFVVILRTIFCRVQDLENSGLDERL
EQLPFDTSQLLFGLKLLKANRRIAINGQKDLLCALSILDYRFCLEMHARDPTKYDLPSKKINLCNANVKM
LPTLTEVRRDRDLRRRAAEKQKKTPGSPSVASTNAASSSTASGFWVGTKIDGKFYFADIISIGENETIAN
ALNDFLTDSNGKSIEKGGKKRTHSDASLTSLTDNVQPMPRRKVTLVTERDPTVLDDTGSETNDSDIHREI
LNWAIRRAAPAPSEITATSGTFRQPAVPRRVSTSSVPLTVVEENDEVFDAPGHENVADAYTRPRTRSAVR
KMTGSNQPGAIRAIPANSSSDGSSSAGGNSPGGSEYRPPSGSDNEDRPPQQPDGARFHHGNIPNKLMARN
RINQTGLSTRTGRRHAQSDGLGLAQDSFSIIPFFQNDLPQAAAYQPINKRLWKMTDQKPSELTLLQTTLT
SAFLVGYTVVVAETPTRPTRLAGEQPDPELLVERIAAYLKEQIQHIPEEIAGAFNGADYEGSVADEQNFY
DNTLSQLINELDARLNVIFMIQLYPKVLVEQLFGTALNANVPVDQSLFREVDQDFATGHSLLVSKASYLF
RCDPVLSGNIEAVNNLILHHIPALRCKTDLRHLRCSFASLSCVDTRCKTGMACLFRGSIIAGGVLDPIVT
LFAIARRIQNAVTRGTLRGLYEVKHSPNYSFEISAPFEQLPGASLLDSNVLLHPSSRELSSEKTGITPTL
LLSIHPCLPSPGNSLRLEFPEEMTHADSPAKYTAQLNLVFFVLVAFGINVAFIPATGSLTIKLDRNQSER
VKQMLHYVNSLLRESQFMAGYEGALKTSQVMPSMMPLPADLVPPNGPNRLGRVPTRDDVLQLAEHLDAKL
RQPAHNLFGPQSPIMAQSQELRALLGERNPFVLGVSPHDSSAWLADIAEDLHNDNGRFFAFKDLIFPNGL
IYRPDDGCRIPFCKRLECPGEREGWELLQTHLLEYRAEENLSVSMLKYCIANATAAFKSENMRVLENALF
STLPSDDDPRGAESFVSPVLRYLNNAGWTISRLIHLLSKYAVRVYGLYTKPGGRSRAVWTIGQSLPMTWY

IPAHEYTVSLSADLPTKFSKRYLSVRPDDVPVIGERFMREGHRMRVNGRN

>E4YHI4_OIKDI
MSRTRRGGGRGWSSGRSRIRVRIHNDLGPRIFSADPGLPPLDVPEVVAVASTRPIDLSDTPDYDEPLPVL
SGELKEDGVRQLRYAPGRQGSGTRVWSPASSLLRGTGSLAGTTTVSYSDTGAPPICNDVYHGSPTATCSL
PVLQESSPERSDGFVSKELDPVWAPDPVLAEPGPPGTVKRSTGVGTVYHLADLTKLDESSTSATTTQLKV
ALKSKNLACVEPKAGLQSGPPYHYLAEYAGYLGLTDVIPVSELNLGSTDYAGEGLRTCSDSRFCQPRLPG
DLPRRVRSPDRFERHWPTNSSDYSVDPPESPLYVHRTSLARFYDHGGDVINLEESVLVLLPTYRREASTL
LPDPMEVGVWKSLTRRGVTGKSMLDRRDFGKSASFLGCPAFLDDQTGTEFLRKWVRSDDFCGVDSSDRLR
DLLLPLCDLRDSFDPSSGLFRWDRVELHSKNDALHWGYPLYQGSGDNPFGQQFADFHVPDVLFNAARFDH
VYPKEADRHLIRLLFGLTHYDSSWVYLVTPMDLYRVQMMAQIRAYSLSEEDGPCPPGRDRLRQIDPGNWH
AQITDVDSHGLGIIEFAALALTIITCEVALHPTELTEDGGQPAFFFDHSARGHDGLVLSGGEGGKKKCFQF
LEALICGVRGRERSDGDQYIGHRRSATSDDVLQPQEKFYYDAVTGRRYPPVTRYDRQRPHHQAEAYYQTR
FSRKFADGKFEPVGHVGLPDVHFEQLDGTDCTARTLMSIPTHLAIAPFEALCAGLYAFINHAFSRGPASR
TLQTSCRFSPDVLPTSWKECHSSGRHVGYYGGTVHDVVSTFEAVARDVETGTSRSRRLYRAYQVSVPQSA
HFAAQRAFCRGNTRGVDRRGSQLFHDCIIDRDGEPLGLPLEQIVARRCAFTAPAPVEDEVAASDCESAPS
SPVAKSARLTEDHEA

>E4YHI7_OIKDI
MNAESDKLIEMIPISSNNNGLVVEELAFHVHPLMNVASEIVDIAENITLNFTIFTDAQALAAYTDLLRKA
QFAKSWIKSIIHKHKSSENMFREFLMHFRSNASNTFDESYVHDLFVGCAHQFDFLIVNLSKQLTDVVSIR
KIHSVFQEIVKKRSVKMSDIISDIVHDGKNDQQSRKGSNHCIKIALAF

>E4YHI8_OIKDI
VTDKRNANPGGAAPLLIGLAACTIGLSYGANGGGAINPARDLGPRLFSLIMFGSEALHGSAHFFWIPLVA
PLVGGVIGALCYLFVISAHWPEEETKIEEEYQKVSDGVSIFPFSNNYFTIQGEIINDENINVEMIRTTSE
IQRQEFY

>E4YHJ2_OIKDI
MVLTGTPLQQLMECAGQGKANLVEEILKKTRRGKPMVTANEFDPAGYTVFGRVARESPAPLVRATLDTLK
KFGGEINAKCHSGYTPIQEAILNRKDPAACVSAFKGFEI

>E4YHJ3_OIKDI
MKLFSVFVIAAVSAQGFDNKQANLARNLDLTESDEDREKRFKQECKDSIGNKQDTPRWNEKMEACRAKKD
AKHAKRVAKAEAAEDRAQMKAEIKTKKAEKAPIKAERNLCKAACKQIANQITKSNCLSWWKYSSRVEIQK
ILDDINDIRNPAERNSVEHCYTKSGLSGSESTCKDFAVDFCDGLNLAKPSPILRDCKSFCVNQCMEEGEN
CDWTPADYVPDL

>E4YHJ4_OIKDI
MEEPDTGQNHVQQRVKTGSARLREIRENLVNSKTKPTITSTTADVFEQMTTTSQEIGIVASQVPEINEEK
ILLEEANWTRINTEPPEFSTVRNLKLALGAIGGIAILLGCVCSLLYISRQKRRQQARAQKRSPANNKFPG
SNQPPNALNPAYPPHYFNPSHLYPQTQAQGPLGAPHGIPEWSQVPPQLNGGSAFAGMQVQYDLAQQYPEA
YLEHFRQQGLYDPYYAVEYYQQFYQQHQQQAYPESQAEMSEIAFDPVLNYVETIGEGLKTPKRRPKQRKD
GSSTPHPIITRRASFKSVRSEVSAWTEDEGELGRKSPCERGEMRRGSEELEGERTPKGDSAYGSRGSIQS

PHSIHELTGVSDDEKVPDGHVVRFSSKVTTAETNEHGTSGSRELAVE

>E4YHJ5_OIKDI
MGLVLGLLVLVAFYDLFKYSKSGAKMCKEFSIFSGSWRFWRRSKSGRGRISVDEKTFSAKISSIETA

>E4YHJ7_OIKDI
MSNLLEHVLELYPKLDHDIADYLGGLLDDVDCFDSADDVSGGIGPFLIDCAQAKQDDVDTLAEKLWGLMD
KKSDEPIKLVKAVVFGDNAVDTSEFDSLLVTDIELRGNYNAKESAKADKRARAKLAKRQAEEEAEKNREK
DELLKEQAVASQAKTKSAISMSGDILIENFDLTFGAQQLIQNADLNLAAGKRYGCVGRNGAGKSTLLRAL
SSRGLSLPTNISVLHVEQEVIGDDTTALDSVLGVLTERTSLLKEAEDLGASGADNDRLLVVHERLEEIDA
DSKPSEAAGILDGLGFTTKMQKMTTKEFSGGWRMRLALAQALLMEPDLLLLDEPTNMLDMRAVLWLESRL
QNWPNTLLTVSHDRSYLNSVCTDIIHLSSRTLYYYKGDYDTFEKTRGERHKQQLRDYEAQKSYRDHIQIF
IDRFRYNANRAAQVQSKIKILEKLPVLYPPEPPEVVKFGFNAPAEERINGNLLQMDNVEFRYSSTNRQIF
TGIDLSCDSKSRIAIVGENGAGKSTLLKLLLGRLEPTKGYVTHNRNCRIAYFAQHHIEDFDLSLNPIETL
RDFKPGKTAEEYRRILGGFGCTGDVATRKMNVLSGGQKSRVAFAKLGFAMPHLLVLDEPTNHLDVETVAA
LAEALKQFKGGVILVSHDERLISVACNEVWNCADGTVKRIEGGLEAYRSALLADFNKGGISKHKE

>E4YHK3_OIKDI
MSASTYGECVQDGFQITCLTFYNFTVADYIIFSFMLCLSAGVGVFFAYRARNSETVEDYLLAGRTMHFFP
TSLSIMCTALSAITILGTPSEFYLYGGMYIWAVACFAINLSLSAEIFLPIFYHLKIRSTYEYLELRFHSI
TRKVTMAMFLLATIVSTGVAIYAPATAISAVTSIDLNLAILSCGLVCVFYTTLGGMKAVVWTDVVQSVWM
ISGLLAITIYSAVNIGYDEIWQKAKDSGRTEFFVTSWNPTVRNTIQAFLIGKTFGLDGYSFCVSQNFVQR
FLACRSLAHAKGSAYMSIVWFATIITFCLTSGFALIIYYELCDPAAAGFLETTDQLMPWLTTYLFQENAG
VSAIYVSGAFAASLSTVSSALSSMANALVSDFLYHWTNKLSEKKQLIICKSLVLFLGGCCIGFAYMAASL
RGGILEAALAIPAIVGGPTWGVFMLAVFFPFVEFIGVSVGMFTGVAACTWMYIGRNIAPIPTEIVELAGK
LEVNTDHCMYENGTMFPGNDVQPEYIENSPLLSFYNISVHYIGTLGFSTTVVFTLLTSTIVHLFVRKGKY
NAKQRDPRLHCHHFSNLWRFITRNKVRSEDDEFWQIPGIKEEKSALEKSSNTDRSSVMSKTSNNKVSPEE
VQTEF

>E4YHK8_OIKDI
MKAALLRSSRTLNRKAVLFLDFSQFEKETNDNHSKNQDNQFSTAVSMFTVGGMIGSFSTTFMVTKFGRKG
AQFVNCFASFIGAAFYVASFYLSSHYCLLLARLAVGFFAGLSTGICPMYVIEISTADFRGKAGVLPQLFI
TIGILTAQILSFPSILGKASLWGWFMALGSIPCIIWILYSPKMVESPRYTLIEKNNSEQAQEDLRKLRGV
EDVSAELAELEDEAAKLKAEQSEDDDGMTVMELFKDPSLRWQLLIVCVAQMGQQLSGINAIFFYTNDIFK
AAGFSTETSTMISALVGLENVAMTFVSLAVIEKFGRTGLHVGGNILMVIFCAGMFLCLKYLTAASFVPYL
SIVCILGYIVGFAVGPGPVPWIWNSEYFPQRARGPAGSVSCALNWTAAFLVGKFFPIAQSAIGEWVFIFF
CAVSAFLAVFLWKFAPETKGKSFAEINAEFAKMNGMEVQETEMLKSDEK

>E4YHK9_OIKDI
MKLSNLILSTVTATSSINELLVCSVWPGETVEQAQIKRSECDELRAIFSSSNGDVRFISRNTSHGLCIDL
DCVKQARENSRVENGAVDVVKTATIYEKSTEHKLLTPKRAMIPTIPLASVSGTYNENMLQDKSEEINDND
IKINDSKAEIKSFANSWNKIFSKAKEKAELPEDEFETQYSSSCSITDPESENTCCSAYSISPCCDYDQKI
RMNQGVSRFPFLRRPTGRIVGGVPAKELKTSVAFIAKISHDHNFCGGVLIHDRWVVTAAHCMLEYCGTSF
PGDLVVKFGKTKKINWELEDGEQTHYVESIHCHQENCKAKGNPRLNDIALIRLAKPVQMSKTVDIASIPE

PFAMPIMSQDCITLGWGDTKNTGHADLLKQVSIPLVSNAQCNDYNWRGCTIRSCMMCAGAKGRAPCAGDS
GGPLFCPWHDGSYHLHGIYSFGRCGTEVTKPAVFTRVSNYREWIESTLSQFGDFV

>E4YHL0_OIKDI
MRFSTFVLFAAQLANAMSDEELLRMFSRSSRNRHSGCYKTAKLSKTRLLSDEIEIFRRNKSKIGLLLNCF
QINDHQKLIRRGRRYFFENILEKLNTRIRKYKR

>E4YHL1_OIKDI
MRRSMDSPFKDRKAQLSIIREYKLSYDKMKREFPEDEEVIETKEESLNPVSCLRTTCGVFLLASGMIGVF
YVINLPEAGKSLI

>E4YHM0_OIKDI
MVLMASILLPLFASAYYVPSFDGDEQILRRSKRNSSFYRERTCLSGTEDPRILTPSAACSSALTTPTTPA
ARYTTLNGRPGLRTPAMDKMMIFDKLTLFRTFLSRETTNFNAANFEDKNLCIFLCKR

>E4YHM6_OIKDI
MKLFALNLSFCAAETLLVPVTPPPLCPDLDKNEICSTECSDGRDQCYNECAGDYSCVNRCNSTWLNCLES
CPCSSGCPDGCIGCPHPVCEATEQYLFMINYNWASENKALHLRLSDNSFIESNFNYHYEYMDVDDACVSY
IKGQHFFIGGYYNPRAIIKVTDCEFKLQSQELTIPHIGGMWGSCETYNDVAQLCFYDGGVGARDCQTFDS
ESEIGTFSESTHPHDWSAMAVYDDKMWVVGGCDPDDYSNCHNHVEYYTQGEYSWQMGPAHPSSEIYAGVV
LGDASGVYTFGGRGGNNARSVYRMRNGSWSFLGQMTEATAYSYYFTSGKIVGDYAYVGENSFEKVSLKDG
VLPLSTNIGPETDDLRLYYAAMILTDQIICIN

>E4YHM8_OIKDI
MRNKGASKRNASRITASKTRNHECAGDQRGGRRR

>E4YHN0_OIKDI
MSLARQNARKFLSLEQVCLVEEIFDDRQLRTHIFELLFDYSENAGTPEYQNWRLLLDEIDNEIYEPRHYR
IFKLILEICNVNHLRAIAIDPRVPHPNNNFAVFTNAFKTGTESLGLTLRNYVDPNGSPELYVASVTSGQI
SEQAGLKPGDRIHAVGKAIIKNLQDFKTAIMSAKTNLREFTITVEKIGFIPSTIGSAQHKKIVWNRVPDY
KFNQEEIPIRMNLSSFNLSYCSPGVFIMQASSKRDQNFTTARIGDEVTEINGIRTAEMNRDEFEVLQAQG
ITEVFYRESNSEELARLISRLNHAGINWNQTELDRLNERHQILFLREATEDKNKHLQRSTSRFTEMNSYV
KPSKYYSDNTSEDGIEENSDKTRNGFLGVPDVDMDKSESEIDKVVEDLEGLAEKELSRESVRSVNYHGSG
SDFVTAVALASRNSIDESDGSSSGVDEPHATSNVQSEPEPEESESSDSERETILLPDPEPESDEEPVQPP
DEVKKELIPKISELERKFLKMLQENSKMASTIRISIPSSMRPRADMVFHLKIDFDENTVLVFCRNKYSQI
AVKETSFQEIRPGDEIVKIRTLKVKEMNSSLSAVEEIFNNWKRSHQKFPLKISVSRRRGAVAGS

>E4YHN7_OIKDI
FGNIGTHQDLPLLDWLTNYTFPIESKFSDKNFSEKMNAAVVRSTLSRGTTTACYFGTIYKDDAVGIGSIK
TNFQLFSKINPKQALTKAKEEGFYRARSVLREKGQHQINNLRRQGKYIAAKKQNIERHYEEKIMAAEGDR
IFVGKIVVGTDPNEELTKNDWAIVVDSHGIIKASGSSEHILSRFDGEIVKLGPYSILMPGFIDAHLHAPQ
FGNIGTHQDLPLLDWLTNYTFPIESKFSDKNFSEKMNAAVVRSTLSRGTTTACYFGTIYKDDAVALATQC
AEQGGQRAYVGKVNMDINPLATYYVNEFIIFKLTYFFPTREIRGLGNSLVEPIITPRFAPSCSVSLMQSLG
KMAKEQDMAIQTHISENEGEIQLMSAFPNDGYLDVYEKNGLVSDRCLLAHSIYLTESEKKKMAEKGASIV

HCPDSNFALMSGVLDHQSATEANINVALGTDVAGGASASMVDAMRYAELASKINTINQKQTTSYLNFKKP
FIYGTLNGAKALKIDDKTGSLEVGKEFDAVIADVSASLEPVFLDNDSPDELLERFVHCSDPRSITNVFLR
GKQVFSS

>E4YHN8_OIKDI
MKLFALSAFSAGVAAHSWIACTDYLEKNGRYYDHDLCRAWPRDAHNYAAIGGTFGGDRGFDHKPNSGSSP
CKSSRNNNNYQGEHHSTVYYQGQQVVLAHPMKNHGTGPCTNPYIPDFGNWIYATPQTEPGQADQVLSVFK
QNEVADLGRSPTGNGVDTEAYPKIGYANAPAFCEDTDKSLGTYSFNIPVDYPAGSYTFSWVWAFNGPTDY
YSTCFEVEIVNDKAARDDIVLGRGQSITTAICDATGTSTGEAGSTVGCDGAETTQPPTTTQGTGTETGGN
GGHSGAGTGDAEIRTNQMTGNIILQPAASGITRREIHVVFFGECSDDAVPNFWYADMTGTTSGDGSLLRK
RRSDGTGLNEQHFVLVQTAAEDIQRAKIGFHWGFSNDGCELLEMPSVVHVTDS

>E4YHN9_OIKDI
MKITQKFYPLHAKNAWSEKITDISQTVSLIQNFLSENDFFSDFRFYAREDERNHGGTLKMASDSEGAFYC
VIWRRSWIRLSVGIRQSARSSGYQIRVFTIALCYIVATMASFQAFTMIAPGAALGARCSSRTFFAILLSF
FCLKEKILKFEILSVLFCTVGLLFYAGDSIMRSMSVNNGTALPQNLLVGLGFGLCAIAGLSRSVGMTMYR
KIKVEIDPVTIVLVHSLMTVIAVIPVMLEFESFNLPKRTTGWVYLVCACCSATIATFSSNWAIQYINPGL
ASVAQNTDIIVSVTLEHLVIKTLPSGLEIAAVIFVLLGTSSLPLFRYWESLKREQEDQYDDAKALLK

>E4YHP1_OIKDI
MSKRRIQNLDDSSDGEDNPKVTKLVTSESHTSDSDSEWNTNGKNKRIKKEKRSKPKIEDDSDSDSEASDA
FDDGWGDDLIGDEKDREEIEAMTEMEREQVLFARGERRDAERKRWEIEKRIKQRNREKKKDGGDDEDSQP
TNSRDKSSSPEPSDPALARARNQKREKSDALGRLKEQREQKEKRAKEGSSKSGPLNVGDYFSSDSEEEDE
EGNNLASSDSDSDDDRRRSSDDDKEDEELARKRKKVECKEELKPAKMSRFRLCQWMHMPWFRNTIVGTYV
RVSIGLGDESGARYRIAEIKDTGESSKTYKLDPGDPKATKIHTNKTLLLRIGNSDRAFRMCFISNSDWSD
TEFDFWKKHGLKSGLPTVGDIEKKQKELERMKKHVITDREVETMVKEKAKHRTNPINFALQKTTLLKMRD
AAETAGNIEEVASIQSKLEEIEKRANKLSRDRQAGIAGITEINERSRRKIRGMESVCEAEWAKFRNNDKV
DPFTRRTTAPILGN

>E4YHP4_OIKDI
MRSQLINILKQSVKTSQKFNDYNFRSYFVRKYSNELAAVESNEGMTVTQELLEQAQRDLALVERQTVVQG
LYNIEPTVVEMQK

>E4YHQ5_OIKDI
MKFIPRKYFHENVRVEVIETLPDEMDEDNINYHGTHLWASAVVLSAVLQKMRLCHDKIVLELGCGVGLPG
LVAAQEAKEVIFTDGFDSGLLSASEALKINQLEAKTEVRKLKWGDKEALKEFKSIDVVLAADCLYPDVSS
WNDFFQTVVLLLKKTSSFCLLGKNMLF

>E4YHQ6_OIKDI
LIYQGYLTQGPKMNGIENGNNLKRERGMEDFGASGAPQLYTQDGPPDAKRPMNDVFGGENLTTEEFPVPD
AMVGLIIGRGGDQITKIQADSGCRVAVVPQSTGGTSRPCTLTGNPEQIEAAKKMLADIITRGSVKEGDVF
NGHPAPSANDLMNQNVAATASLDGNCMEEIIIPHDKCGIVIGKAGNTLRNLRSQFGCSVNLDSTVNTGDP
KPLRIAGPPDKVNLVVAEVHKMMAAKENITHTKVPDGQDQVTFMIPKVSVGVVIGKAGETINRIQEQTQT
RIQFVPDDPKILERGCYIIGPQEGCLVAQKEVLEVVRKKMEEVEGSKQPMPKLVFDGKRYVKQDGMNASH

HNGGGEQQVDYPVPASRAGVVIGKGGETINGIKEKTGAFVQINKNPPAEHPDWKYFTIRGNSQQIAHAQK
LIQEKVGGPAPPGAAIANNSSGNGYSYTGGNSYGSNNSGGGGQQDYSAAWAQYYASISQQQAASAPAAAP
AATGQPDYSKAWEEYFKKTGQTPQSYQASLAAQQPAAAPAPTPAAAPAAGGQDYSAQWAEYYRKLAEYQK
AQGN

>E4YHR1_OIKDI
MMFFLFLFLPIRGNSQICESGRGKYGEPVTFCEGLSKPPDFGPLRKTINLVIRNSSFTDLYGRLIRRLPF
LRSITFENIPLSKIHKRAFISQPEKINFLHNDFQSIPIHAFEARLQNITLSGQKISKITNRTGSEIAKKS
PRLNKINLRENEISIVEPNAFVNLQNLEILDLGANKLMKLNFHPGKVTDLRLDNNEISHISEIFTKNSQK
IKRLILRSNNLLSIPVFNFERIEFLDMKWNSIWNTPFDEQNFTQFANTDYENAIFEVGQARIYCNCDETW
QEYVSKIAVVEIECRPEESRGWKKQVQISSFFKENCAESSADAPETTKFPEKSSSDIDSTLEWSDSVTAS
SVENTTFPESTSSPKMETTDLFLNFTMFPDTFAPTQTTVFSDGIPVVLDAPLWRSETDDIEETDNFFSGD
DSSENIEDSTEASRNDVWFWDMIDDLQTLEWDSEILEPIEVHLENDKSEGPHQTTKAVMESTKSYEATTK
IEAQTFQTFIDSEVLTKRNSQTKNETIKPLMTPSNPALTTFTTSLGTTSVDKTASDLAPSRTSSTTITSK
MTKTTFKTNSTTTTTTTTTTKTMTTTTTTTTTTTSRTLSAKRRRQLARRENQLKGRVNKMNNKYKKQQQN
KIKAVKDSAKWKQNQVKKLGTILQRSNDLANMAERKLIYEDVKAEKEFRALIEKLETTTTSTTTTTTTTT
TTTTTTSTTTATTTSTIITTTKNFLDMKHLIETLNRIALEDRLCLFFFAEVKNWFSNGGGRMTKSTFFKF
LRRCEVYEYKSKIFEFFGGNLVLTDETTTEGPLTLPNLTPATTRRHLFTLKPRLFTPRTTRSPPTTKSSV
KTSQSTKINDDELTTIEIATTTQELRLPTKPQTNPFGVTNKPAIVQATIETKASTDEIVTLDVSPEESTI
KATTDWKFGNSPASTTKRPFLNNSTLPEEKRIIIILTISFVAIMLFGSIGVTMIRNMLCLESPAARQARE
IREMELNEAAQANLFARRRRRGRSDAPPAYNDLFPAHR

>E4YHR6_OIKDI
MEYSTEIHSSEDFCTITADGEYFVQFCDPPVPSFVNDSVDAIGQYDYSITFVIDTSGSMFGEKLKASVDG
LLAALAGLSPADNFNIVSSSRKEEIFSQEALPATAHSIARAKEWLMTLRHGGVSSIMTGVQSAIWHITAS
DDKKRIPLIVILSDSVSDATEENFQIALNSISNSAAERISISALAVGYTDTSSWQFLQRLSTRNRGLAFR
LFDLTKFVEIIPEKVDGIKKFSRSLRGLTDVHFSFNSESVADLTDTSFSHFTAGEDLVVLGKFVGKAPAF
LNFTIEYRDVEDNLISFSKSVAIKKFDKTGERAETIAKFSAEQSDYNPIRQQWAVTAVHQLLKRRAQATS
QSDYDELTRQAAKLSRRFKIVSPVVSFLVRRPKSSANLIDPANENGLREKRSSEIAESHEIHYQYRKLWW
RSDEKDELEKTAEESFENNLQWAALTGYPLMFSRKLENSDEKVCLALPGGVVNGRVKLAENEQFSIIGDI
RKFKLISVTIRINEDTIKVTNTTIYVDGAPSSSHPFLRTEENFIHFSIPEAKSSFKVHRLGRRLDLTAEI
DLEEITGFLPSLLEVESLMLEEITEKHKTAMLWVHGKHLLAHREENCWRLNAGETDVLFA

>E4YHR7_OIKDI
MENFQEKIRVFFSERDRKTLLYEVGVGFGVVVVCKVTHFLLKNTLLHPIPTFEFPLSRDDNFKAIKKGLD
EGKLPKVFRPKIFPGSTFSDILILTDFEIMKEAFSKRAISNRQFSDNVRADIENMQRRLNYDEIPKSILD
ENDPLFKNGAGNVAGSAT

>E4YHR8_OIKDI
MTFLELAYLECFSSKNPSASAFFSHRSAISSAVSPSLVSTRKSKMRFGSDGPYDEFHKKTRVQWHDTMKR
LAGKNKIDEIIQQTAEKTTFYLEKLGSSPEGMDPRKVFMNASINVTSGFAFEKNYDFEDEEFQKLAEYIN
EFFRGLTHKSLQQMTWNILPRWICESDFYAKIWYKTPMKHFLDAVPPFHKFIYTMIKEQRKSLDHANPRN
YLETLLIDAESDPKWGYFTVVATIVGVFLGASDTLANTMTWLALVLADHPEVQEKMNNEIKAAREIDADL
KKENCPFTRSVLLESRRLNSVSDTLPHIVSEDITVKGFHIPRNSQIFGSLAAVMRDPKNFDNPSKFIPDR

FIKDGRFENDPKVCGFSVGLRNCIGKSLAIEEYFAFATAMVENFRIKRVAGNMDLAKHAFLRLPIDDIRV
QFLRR

>E4YHS5_OIKDI
MKLLCSFILAASSRDTRRDEFTVDKEYVDAVCAEKGEGSYAYSDSVCDHYIHCEKPGSQAVPHVMPCSPG
TVWNSVEGFCDHPFNTPPPCGTLESGEPTHQDCEQGDGVYAHHHNCEEYFICYMGQKYFGKCPAGFGFDQ
VGKMCSDDLFVTSFYCNPTKKVYGNPVTTQAMAFCDEKEDGYYVNPGVCGRFIKCWSQIGSEFTCESAQQ
NLNETLVWNSSEQTCDHPATLADQTRSCLSQADSIELEELLVKGSIN

>E4YHS7_OIKDI
MKLLGFFFGALSAQLVDVFFDDQGRQSDDGDSWRSLDVDEKGPGMCNGGKIPPVKNGKWKCQRNLNDDGN
KKGKGRKCKLQCDKGFQQFSNKQNKPWSDGVVKCKSSKGWKRKPGKISCQKK

>E4YHS9_OIKDI
MVGAIIKKLGKNTAELARPVPIGRSSPLWPHLQIAADGGDTCEVRVLPSPIEQVVGELNVDRFPCDFTQD
EVFYQHYGRAEPRSDRISLQVHYETRTTKKILPIQIEIEVLMAPHNLPNERVFIDNQDLLGEVYPIFEGS
PPFDRRFLSDNPKIGMWPKYGKLVFAPVRSTCDDFVNSGIFYRHSGVTGTPDKDHQPLLIEIEDKSGFVR
EREWFDLQINIRAAPKNEPPNVDPQAELKLYVDQLILTAITRDVLFGKDYETDSRLLVVEFDDQPKSGFF
VSTDDRTRPILSFYQSELLDNKIAFQPPQAGSDFDRLLSVNFRIHDEDGGTSPWVPFYIKIQAMETSTPF
AMENNGLILYEGSRRCIGPDELKLSDKDTPIGNLNLRVIDGNRHGQIYVGPMAIDMFSGTDIESCSVVYE
HDDSDTFVDNIIMEISDGEASIEILFPIWIVPIDDQPPQIVKNLGAEIYKNSNVTIDLEDVDIDSHGPTL
YTLQNPLSKGELFVLNDEGQWTVTRTFTSEQVQRKQVEYMHNAGVNQLNVEIEELELTASDTRGNTGLSF
NYTIFIHPRDDDPPRCNFYPCTLGMRVNEYETAPLRKSNFNFVDNVSPPEEVVIDIRTPPHDVSTGQPMG
RLLGADTKLPLTQFTQQMVNHLKVVYEAPNSDLGLVRRVVEFSFHVSDAMQNTLYDQTFTIELMPMDNAE
PIVTNMGLQVMQGEAVTITQDKLDVTDMDTSDEMLAFMLLEEPAHGELRIADMKIEVGYEFTKLDIVAGD
VSYANNGDDDNSDIIRLEVTDGMHSFPVNVDVVVIPRPVVPEPTQRNPSETTLVVPERSQTELTLAKYFL
GPPEAADKDLSFTIVKQPEKGQLLKDGLALPAMTQPVTTADMANGRVVYQHNNQEIGPEEVQDLFILQAQ
DIHGRAESMEIITHVRIIPVDNQFPLVTVLQSITVDEGSKTSFNPSHLEINDADTTPDDIICRIDPQPEF
GFLENVSPLPGSEKSRAGEAITDFRAADLPNEFINYVQSIHKGYEPTADEFWIRCRDKQNNESIQKKITV
IINPVNDEEPKIEYSRWRVREGDILNLDESILNCRDLDIPFDELTFIVTSPPKFGKIIYLGMHTLESTES
IDSFTCEQLRAHEIAYEHDDSENFEDSIKMQLTDGKHVTEETIPIDIIPVDDETPRVEINRGLQMEHEQR
NARIGADVLKVTDLDSVDEDLMIIITHPPRLGTLRMDDGSPDGRYLSEGDVFEMRQLWENRVIYKRDSLQ
SIDEQRDYFVFEVNDGLNRLIDRKFFIQIESGDKLYPMVFNEGLTLPEDGRRTITTALLQASDLNSNDLE
LTFKVNKMPIKGHLESTDSPGKKRTTFTMRELVGSKIRYVHTADDEIRMDAFEFSVSDGTNTVYRTFRVN
ILPIDNKLPVVKVEGIRMNEGSEKLISPFEVSIEDQDTDDSQVRVTIIGDPIHGSLNYDGSFVASEFTLA
DLKNNKITYKHDGSESTKDQFRFIVTDGTHDEFYLFPELKDTHSGAVTFPIDIVPVDDEIPKLVTNKPGS
YVQQNADGTKTFTISKKHLRATDRDSFNPDLKYKVKELPKHGRILKALPAQMPQEVDYFTQKDIDDKAIR
YSLNDDVSETEDFFIFDLVDQGGNKREDLKFICRWSFVSLAKEMITVDEADKELIVTVKRRGYLGETAFV
SVNTNDILAEKSLDFKNVAISSNQVQLNPGQTDAVWKIRILDDDIYEEAENFEVQLSDPIMTILEDPSRA
TVEIIDPDDESTVFLSEPQIETVENVGILRVPIQRVGDVSMELAVICSTVSGSAGGTGPIPLESFFDFIS
RPESHQSVIRFGPGQKEAFCEVTIIDDSLYEPEEEFTVVLSQPTGGRIDEEMDETKVVILKDPADIPICN
FNVKNIDVEENVGQVEFEVLRTGSDLTGESSVIVRSNDIIADLKYHAAEAGSDYVAISKVVTFEPEAVSA
TVTVTILDDLGNPVMEGLEQFELYLSMPEDCVIGEPKEIKITIDDREDDKPSVEFQKAEFEVSESEVHAT
ALIIRQGDLNQETTVRCYTRQIGAEVAKDYIERPNTDVSIVRFVPGQTVAKCDVELVNDQSFENTEDFRL

VLGTPLSPVGAKLGEQFDTLVNIKDDGDQPTVGFEKPLYEVVEPAEGNVQRLRICVVRTGDLAGELELRV
HTKDGNADSGLDYIPLSKMIKIERDDDKCCFEVDILHDKKKEIRESFTVWIKDPIPENGVDPDIVQPRTI
VYIKQRDILADVTFPTSPKVISLADYDKLKNAAATVPPPKSGYPVICVTPCDPKSDVYDEVLSLCEDEQI
ANEMTEYRWQVSAPSDANGATHSLQDVSATTFMTTVNQITLDSIYFGVGSRIQCHARAVKSNGEPGRESA
SEIVTISDEGICPPRQEGVMGADPFSAKIRYIDGDDDKHPNTIKVSIVLPHTDGIIPLISTKQLTNFEFT
MSEDSTRSSQHSCSNLLLPREVRTNFGFLTNATSNPKIYSDNDLMPYQFDRNLRSERTLQFYKNLNLEAC
IWEFESWYDMSELQQACGGEITTDGQVLNLIHSYVTLSVPLYVSYISHSPTRIPWRHFDLETNLRLTFVY
DTAKMWTDGIGTPPESSLPGTFYPTSMKIQPDGRLVVRFKTVAQFTGMFVLEHPGSAAESTVSSVQHPEL
TFTLNLIRSDKSFSTPEQQWSFVSNYAITDYSGDYKISLIPCTVSHDQTYSDPPKCNPRDAVEFALPLRA
QQVSDPVAEQFSLNTTFSLLSKKDLWTADSAIQVTESSDVAFPEGAQIYGRVQVDPVQSLGAGFTVNIEK
VFLCSGSEGYIPKYNPGNSEYGCLADSHTLMYRFKVIVSYRISLLGLQIFKDKEQPDTEDDSLNSVPFNA
VLAVDDPSATTLSEQPGADGFRMDSTPLFQVNVGAEWYLHTIYTVRSSTKRVRRHSIVRRAAAEDIGVGF
DRGTNMMPIRLMSDEAFEEMEAEKAEFNWMIILAVAGVLFIAAFVLVVFRRRNDGNYPTSGYAQARSNSD
AFKPIVRPNQGSDSSHSSIPSGTEV

>E4YHT0_OIKDI
MLRRVRSRLKNYQTRKNSVDVINAIEFAHATGKCSYGIRNGLHDVVFLDKRELPDVALYKSLEIIFRVAS
FDQQIDFKRFATTARKHVETSELSLETRTACLSYLNSSKKRINAHQSDLIRSHLFDNHTSIKVCSRMLEI
LIVDLLKDNVAQNANIVDKLLQKLYSERGPEAIRRSLARLLVIQKTNPQFSHLFEFANSKSSEIIESDIP
WVPLINTQPEDIRKLFIHKAGGNVAAFYDPMKTILASDFDKEKKHNLAKALIDVIVDKSLYSFNQKAYEL
NKFHTDRVLGTFRHQIKRIYEDPEFLHLMIPYLSKVNIRWIKAYTCLLRNMERKNGFDELERFILEQQKE
VKFGRIPSWELDMAINGVRTYDLNYVKPKELESWLDEILEIEGANEKIANQLLSDIPHATLSSNITELKV
LEKLYFFAKERPMDKKFMEKVTKSISTRANHAVPLCLLASGTSGKLAKSGIDFPSTSKMKFSPDFKKKLQ
QVSHPVRIRSFTLNFIDYSEVLSENELEELITTLPEILAESVRREMSAADSTLLFDVSGLHEFENLMNAI
LARSSRTRDSDIRIVMEHLIKTSNHKVGVRLLEALQTDARLLAQFDYFDNVHLEFAHFLYISDSYLEIVG
KQWEKTLFNEAKKYDISTRANHAVPLCLLASGTSGKLAKSGIDFPSTSQMKFSPDFKKKLQQVSHPVRIR
SFTLNFVDYSEVLSENELEELITTLPEILAESVRREMSAADSTLLFDVSGLHEFENLMNAILARSARTRD
SDIRMMMEHLIKTSNHEVGVRLLEALQTDARLLTQFDYFDNVHLEFAHFLYISDSYLEIVGKQWEKILFN
EAKKYDIRFMMISPILLEKRFDSWGNFFYKSVGRSFSIFSWNIKNNPDDDFFILRSMCDYQSLYKLCEKR
NEWGSFMDKAANHHCDYEYFVRSFIEFDNLEGLKHVLYSFKWKDFSLHPRLEYRMIHVVREIVRSKRVNF
IEMLVEYFDEVVPKYGCKELQSLHPVLIELDLVHLYDKIEPHLLAHWEYKRKSLEGIRKIENSENWL

>E4YHT6_OIKDI
MIKLLRMQVCKDAPAVKLLKLDTKEGVASVLAKSVTTRKNKKLKMVNFMKKQSPLSPEKLEDTFAETDYI
IDEKRGLRRVRPYYFTHHKFALKRWLGMSVFDMMIKEFSLHISTPEKLEQTAERGYMLFNMEIIHASELK
NRIIKDGDMISTKDHRHEPPVRIPLPEVIADTDEFYVVNKPSSLPIHPVAQYRHNSLIFLISREYKVNYH
VIHRLDRLTSGLVIFAKTKEVAKRVSKEISDRVVQKQYLLRVVGKFPDKAVCDKSGTKKGIYRVAKADDD
EKKVKTALTEFELVEYFKEADQSLVRAIPKTGRTHQIRVHTQYLGCPIVNDPVYNDPLFGKERFVERQQR
EDITEEMAVNGLTKTKNWGPKGYKISEDDNRLDMDFEKDPLCDKCLNPPADPDQHDLILYLHAHKYSGAN
WSFETPTPFWAESDFKTPEYYKKYLL

>E4YHT9_OIKDI
MTPKGANSQKKWKLKMEEKLRMHQLKMKEREIIAQRNEEKEAERIRIEEKKRQQEENEWKQAKSQGQVVT
DAKKIKKLEAKEIRQKKRAARRKSKMKGPSKLK

>E4YHU7_OIKDI
MLWAVSTLCSLTFAQSDVDKGCENLARDIGLLLSAGISDYSPKLESVFRRRTDESWRPDRVQNRVNLPQS
SYEVSEIVDYRTSISDLLEDSSEKIRKLLSGADQGVSELQETLLDLYKKKVRVTYNETEVKTLDKKYYFN
AGYTNTEVENLEEDDPYSSIRQRIESLRLNKCFGQGQINVSFEQSTVHVPFNVYSLSDDVLTTASYSTGL
DKTFENNAKNSKVLSWQYFASSKGLSRFYPGLKWKLEDEIKRPIDYDARFESWYASSINYPKEIIMVDS
SGSMKGYRKVLAILTIRTIIDSLSDQDFFNVIHFESTPSYLHGCFERTLVRATDFNKQQLKVLLQSMKAS
NVADFNKAINETYKVFKEFSESQFSLTQNYYPAARGIMMLSDGALEDYAEAFNNASIALNDSNSVEKTRT
FPAGAGADIRVFTYLIGKDLKHQMPLKNIACARRGYYTHIASPADVKNNVMEYFHTMNRPLVNQDVYGVK
PQWSPMYSSSLIGGLKVHGLVVSATQAVYAKDTVTNSGELLGVVGTDVPLVFLEQYLPKPYILGPHGYAF
ITNANGQLLSHPDLQIREENGSMKTTYRSMTLITMELYRETPSRKKDIMDAIRLAANGTSKVYKRGFEAR
RIIDCGSAKISCRKLAVDTENVLTDYSFIGLNENFVLGIAQPRLMKNGNLQFAQNTEKLLLPKNKMIRGS
VNLIQECSKPLGNPGQQSNRKQLICEFYNGVNSNTLKLAGNWEFCKFQYYLYTDGQRCAPIEQTQALLDY
VSSNRKGSTNVNYNCDDSMIDSLLYDIHMTAILPGMWADYLRPTGVTAVIDFAYVLTRTGLLRFYDNSRT
GNVVSIDLLEPYDIGFEKFSYSRLFRIAASYKKGTTVYLLNPKDSQKYLTARTWSLGESVNFKPSKFSSE
YHVAMLTEGYQIRRDYLVDLIRQEAGDLQCKNINAGNTTQAACNNNIKDCYLLDDYGTIFVSANKSETGL
TLAAADSKLMQAMKTDKIFIGKGAKDYQATCSEVQTVNDQELGWKTCPLFVFYFFISNEDGINLKTEFPI
NTKEDLKNPSKPSQDDDITNEYNKARPCLKEFTIYHINKAFTEDLGKKRAAFCKQEIERGQDECSTRSYS
LRKIKNTNLSLLVADSCCTLTGAGSCPASENILTPEKFLSELTPKEKSLTKDALCANLKEDNIRRVLLDK
LESTDNITQSPCSSSAIKIVFSNSLFFRMILFYLLNLMFT

>E4YHU9_OIKDI
MEVDETCKEESTQQEMSIEDQFRNRMRKLYPAVDESITPLPRFWTSHKELSSKLIIQQGTRVEYKGPGSK
GYKDAASARTNTEIPAVCGMYYFEITVISKGRDGYIGVGLVGHIDDSQHLNAQQGGYNRLPGWDQDSYGY
HGDDGNAFSKSAHGQKYGPIYGSGDIVGCCINIVNRTIFFTKNGSNLGVVFEDVPDKPMYPTVGLQTPGE
LIEANFGEKPFAFDFESYLADFRRDQIVQIKNFSVGDAFQQNIQKIIANYLHYQGYSKAAAAFEEATQTK
CQAEIEVTKKTRIGIVSCILRGELTEAIARLSTDFPNLLESNHELVFQLKFQQLIEIIGGTESEMKNYKD
TENSFGRLPGDALQDFIEKGRNLREYVLANIDNHESSKMNQIKKISTLMIDNDPSKSPVADLFEKTRRQR
VATAVSAAIRQSQKLSARPKVAVLVNHVRLLHDTLLQRDIPQAAFTLPENFLNE

>E4YHV3_OIKDI
MPRNSIFPREDLSLPTINEHKIDGEVFIIEVDNSASRAPKLHTRGKWQRGARLAESAVVQNPKISGLSKR
FQVSSLTSTDTVTCALNTKHV

>E4YHW4_OIKDI
MSSLMSPRVEINGEVHPYNKYFCNSANAKNTAYETWSSSKVFAAANAGSTLRKGSPTCSPRNFGLDAKVW
GKNGYTKLADLVTAVVSYDETAGYSSNGLSAYYHDIGGRDAINALVKKRVLVVFLNLQYRMTWLGQPQNS
LGGNYGASVPSDLGTTFINEYGEACAIPRDNPSQVYPNSLTMLARRDVNSRKRYNSWCTLKIRLILVSYF
PGFLRF

>E4YHW8_OIKDI
MGDRFHRAAKDGSSPQGKALLKEATSRDLDRRDKEGMTPTLIAAQEGHSEALMICIQRGGKPNLYNAIGQ
TALHLATDRGHFRIVDYICKYYEEELVKILFLMDLAGNTAKMLATTQGHKKLIALLDRNEAELIKRNPKK
CKKYKDKAKKEMEVCKKKYSKIMAKHKTEKQSSSAPAKKTVYGETSVTTKTTDGSNNGFLGTFTMGKRIG

TVRRKEMLDQFDFSAMDEVDGTMTVSSNKNVLFGQGTVKNTAKNGTSKARPDVRDIFGQNGDLDGDLAEN
GELGTVSAVGMISTNRFFFNNNALAEQFDVLGAATTTGATFIDKNEESDSEQGCHF

>E4YHX7_OIKDI
MGVSRQSDPKHEKLSQSYNLWVHQSTKFITRELCSLRKLLFYDFRLGIKKILLSCNLQDGGNFCITSLWK
FVTFGGEPAVNVTIYSSSVDKSGGNWAGLGFSMAGDMTIADLFICKRLGNATKLVSAYSSKNSRPTEYSN
YTGILAQTVETSLRNPKNSTLGFWCSFTMTTNHTKMDSANSSTTFFYNQTSKYHAIEAYGQLTTWDLTFH
DGTAVAKDVIDGFYINEKNKEYKVAATQGTKLFKSHGALMILAWGFFIPAGGLFAAARYVFQKGGLWFNL
HRAFMIMGVLLNIAGFVVIFVENGGFVDPGYALGYAHAVMGCMVMGYSLMNVIRGFFRPDLESPRRRKFK
VTHFLFAGLAIVLSNTNITTGLYMVSLKASAIAFGVLSGICMLFIPIFHFWLSSDDLPMPIHNLVLTGVF
VVLIANTIAAATEFLLNT

>E4YHY0_OIKDI
MKFDLHFYEFSRISRMQLLRIADRNIFQERINQKLNAEESRSRLKRRKEGAWPSRAKTSWWEASTAQRCK
RNRSA

>E4YHY3_OIKDI
MNQVVSCRTTSTRERRRCVRSAERTRLLQVGSRKAARSFCQRRQHLITVTNLSEELEDSIAENDTFSKQN
TKLSKQNSNLLAKVDELSKDNESLQKSLEDVDAVHQEEIQAVLAQRDQLKSQLDPTELALTKSQAAEIQK
LKHELDVFKRTESQTFPRSSSPTQSDTKPDIKLKMAKLQKQNKAKIQALEAKLKEREENCASYKTINEEL
EAKVAKNERDYLNLIYKESEMKLVFEKQVKELTARVLNQEEKNGEGIAEVDEIAKEHREKTQELEKKLEA
ATRQNDESSDRIGKMKEFVEILKNKKDLLETELVMLKKQLEDTRTIAEAANDHTNIEKKLEFYMDKCQSL
EAGVVMSDTAKKQAVDDNERTLKALHLKEEELQKTETMKMNIQRHCQTLIEQAEKQAQSQLEIEAELEKL
QNDKRDLVQSLTEQKERANELQTELKKTSKKLKNVENEHKKKYSMMETQLCEQVAEISQKLEEADAKILG
FKTMAVDNDEAQAAVADGISKLRRENSEKREQIQSLKDELDVSTKKLQNRLDGCLLREKDLKNKVSESKE
LVAQCEKEKSELAKKLKKAAKSDIIIADLRKQNDSLDKNFISQGHKLARIEEELNACQRILEPVNARKIE
LEEKLALYEKQYLANKDMNIEKERDMYRESSLRLEKERGDLEQKLLEFERISGETQVAVAQMHAQNKDET
KKLVELHEKTINKYEENIKELEEEVAEKMSRINELIASESSNTEQDEQIKLLRDSHKEELKRRDSLVKKM
EEDRALLTQMVSEAKDRADTNEDSAEKFEKVLRQRNEAYELVKNVEEMLTVKTKRMEQLEEMRQSLEADL
AEQTQEYEAETNSLLTAKAEAFNKLKTIEKELASEKKAKLRLKRDLEQDQKIKKLQSADLESEVTAYLRQ
EIGNLTSKVKTKDDELSRMAVRLTTLEEAENLLFSKADVLEKQLSEERFEKGAVQEQARILAEKLEALKG
KDSTVSADLEQPEKESLEEKLLSKTTDGPEIEKLNETIELLRDAESSMFSKIQSLEKELAEEQAKXXXXX
XXXXXXXXXXXXXXXXXXXXXXXXXXXXXXXXXXXXXXXXXXXXXXXXXXXXXXXXXXXXXXXXXXXXXX
XXXXXXXXXXXXXXXXXXXXXXXXXXXXXXXXXXXXXXXXXXXXXXXXXXXXXXXXXXXXXXXXXXXXXX
XXXXXXXXXXXXXXXXXXXXXXXXXXXXXXXXXXXXXXXXXXXXXXXXXXXXXXXXXXXXXXXXXXXXXX
XXXXXXXXXXXXXXXXXXXXXXXXXXXXXXXXXXXXXXXXXXXXXXXXXXXXXXXXXXXXXXXXXXXXXX
XXXXXXXXXXXXXXXXXXXXXXXXXXXXXXXXXXXXXXXXXXXXXXXXXXXXXXXXXXXXXXXXXXXXXX
XXXXXXXXXXXXXXXXXXXXXXXXXXXXXXXXXXXXXXXXXXXXXXXXXXXXXXXXXXXXXXXXXXXXXX
XGQLLQAKFKQLDEFATKIDEAKRISDRQEMTQEAMNKLSHLVRAKDDEIEALNSKNQSLTEILRSDDAS
SHINSKLEEIDALNKRVIEQKQENGKLKDAVRKLETSCLMVNKQFEEYNEAIVSERVDMRSLISSHEQLH
SQAEETELELAKLRIIVGKIRAHPQSSKIAESSSPSTVEINGDRDMLAHSVKNLRERCAGLEATNVAIKR
DNDALRDQLMDRRDKWDNLELLNKKMAAEISSRDERMAEHLESIAELKANMIKKDQQLKDTEKDISELDR
LRERLVQVDLDHSEELQLIQSNEERLQEQVDSLRSGLNDVTGARKDLEEKQQENARGQEIKEKQLLAELE
VAKKRDLANEKSISELRQFIDNFRDQKGKKEIYIF

>E4YHY5_OIKDI
MAAKPPKVTVEERSSLGRGVAKEIMSFSFRELFNVKGAIQADFVKSFGQYRMPMGVELLLDELIVCNMNN
ATVELVNLDGETEVSLPDQVAPNEDGQAGPLNYNKPSIATMLMDSTIFVCDNLGCHKYIWDFDNDTVRFV
KTIRPTPGEIYGMCVIREDNGGILFVVQDARDRLIRYRIYDLDLEEKKKDLPVVFPPSNEEGKEKKTIRF
AAGVGNTVALSDMSKLNQGIWLTDTDGVVRRKIGDKQSDSNGEFIQAAGICFDSEGNFLAICSKSSRIQC
FDSEGNFLCCLQFPEGAIQRPSDLSINEQGELAVVSLTGQCFLFKLKAGDPTNAYVTRGPRPDHKSGYKV
IGRHGRSNRYWGKPNGDRGRGRGRGRGRGRGRGRGGGRGCAV

>E4YHZ7_OIKDI
MGINEVKIAIAAIKAAPDNQKAIDTLEKILKNILTHPLEAKYRKLKVAGKVFTETLLPVDGALDFLYSLG
FAESDDGSWLEIAALTSESKTMLVRAVFLLKTNQSSNSESAPLRDSGSQSAPQIPPAISESPSSSRKLQN
FNSSFLEIQERILRLRSSAMMHVRQWEDPKTQKKVRDTIPVTALFKRASELLEQNDSADGFDRGELLKHS
LLITLTDWFKNEFFKWTDGNIIRYNNPEKLLETREGMLSYFCLCCFNYFPKIEYNIFITRKGRCGEWANC
FGAILRSFAFHVRETYNVLEDHVWVEVRTLGRWTHVDPCEDAIDKPLMYKHGWKKSCHLCLSFSVAEGAR
DTTWRYNNLHAELREARKQLVIESWMNSFIKKYEERKKKIMNWEKKMQEEDWITEAVEFLSPKQIGSDLK
FSDRTTGSLQWRTSRGETAKNIEAKVFFYTKIKEESPEFVFSYNCAKDTYSFNGESVSSWATFAHHGGRL
FRKEEKDWKMCYLAMQEESTGEAEVEFKFKLPNKQISSILVTAHSATYENGKVNWIFCSGESCTKFNPTE
QFVFPEPSPDSTVSIKASLVAPNFWQHAQLFRQALDDTSFMFEVRFKF

>E4YHZ8_OIKDI
MSLNGNWKKTATEGEVEFGKAISWAGASEEEWKAQCTIPVEVSYKIDGDSVESTRTYGGAKAISNKGAFN
AENEYNFLGNEMKVKISGKDGDVIMESVSGWATVTVKVDGDKMVETVKHNESGAVLVNTWARA

>E4YI01_OIKDI
MEKFMNFSSSSSESSEDYSDVSETTESYSGDLEEKGIRPDRVSDDEEINPIRRRKNPYCRKCEKFGGVCI
EDNVDSSFDSNSDDEDQAVKMICSCKYMECDPDFLEGVVAKPVCDQNQVKYESLCLLKLTRCTKQENLTR
ASCKGKPTRIVVYSKTDEDYISSYSENPLDFYETDDFEEVVLPKSTTTVRTTTEKIVTSTEAKKKIPAKD
KTLAIVIGIVEGLAFLTLVAVLTVSCYLAKEAKMNGHQHLESVETAPLTQVKEKRSKVSTIDSSI

>E4YI03_OIKDI
MSILQNRKKTVFPQRLQSKLHLNIKMKLLAGVLASWVSAQYSNFYDYDQTYYGKSQVAQSEGYSPEVASV
ARTLGNGRICWSCLEETYTKCLLDDFSATTMRGDEKRHGAVYCQGEDYFCYISERRIIRHSENEWNYENG
NPWQATNPIDVDHAELINGLAGADPSTEVRVQMGCQQPLSCLRQQWQNYQIQMGKSFQQGTVALVDGAYV
PVGSQTEVRSGLCRHHNAWVDHASGLHTVNDQWRKHSWMGDTSQPGNGKVQDQGWGDRRFGAIERHFQRG
KATESVCHHCCDPYLEYNAKGCNNVAATAYAVAAVDRVDETAEATDEANIFLLRPASETSLDWSSASGKP
QYHGAFRNPHTQREKEVLGTT

>E4YI10_OIKDI
MEFFLDTFWREKVRFFRQVSFCECAGSNYSFRDKLRGSSARAVTCQPKCISAAKPTCKVQTEKYHFFIYF
LNLYISLI

>E4YI12_OIKDI
MKNTDLEKVGRTSRAAALPTVIAKSNQSESAGDGQPRRGPLCQNQNLNCQLHKYFFEFFSRENHLKRQNG

WRITNASCEPDQREKNSQAWKYSFKSQRYKRKQSSRRSLPPRALHLRRLRLCYLRDHHESSGFRRTIIQS
LKLVQNISS

>E4YI21_OIKDI
MKTFKTSPSFEEAKKTGENSSSKSVTIKIDEDIQQVATDEHHARQAAQITGATPFHIAKQELSQEMSAAN
SLDILLRPTPSADDMIEEIEEIEEEPEPETIDIKTQPMEHEISYIPAPSQLQRNQQAFVKMAGGKIQQVM
MQNPVQKPRQIFMGANGQLFEIQSPEPKQEPQQPIVIVLPTSKNGETQKLILPQNLINNSNSGQQVIYVI
NNQNSNSSSKNTPSSSFGESSSSNNSNVNVIDLAATRAGIRTSQIEVKEEDSLVKADVQGHHAYPSPFSQ
VPVQERSHQCKFCHKRFARSDECKRHERIHTDTRPFSCTYCDRRFTRKDHLRTHTRCHTKEKPYKCPCCE
RSFARSDERIRHLKIHVKRGETTMEEAKSKINEALREQTVHKKNHIIFEEVKSESNIVPITNINFIQEDK

>E4YI25_OIKDI
MYYDSIPPEARDHSREQMTCQSIQSLNGNNRQPHMWTARSGASYIPSQYANPMSASVGYSYTPNHMRVSS
NLTDIKTQTKTSKEKSDKCFKWRGLIAILSAACIILLLLVAVVSFKFLLPAVGNSPGGDTQVTGGAGVRI
IDPAVSCSSNSPVWDATEEIYVGSAIESLITPGSNIPIVLYVPEAGHVNFYARTDRPECSLVLMARQTLP
PKLSAHDFLESLSGAYATSKADNSIMYESRISRGLAAATWFLLLTNDGSVTCQVTFQTEMTDSSCPNDCS
QNGICFESKCSCFAGWTGRDCSIGICAPVCSGNGIVAGFLDSCVCYPGFNGRNCEFKSMDKKPCDETCQN
GICDNNKECVCKSGFSGRNCDTKTCVNDCSGNGVCVSNGKCRCFNGYSGADCSFDNAADESAEVCSGNGL
LIQNECFCDDGFTGQVCEKEVVKEDVAHNCYPKCENDGRCVLNDSDWKCECKAGTTGVNCGAKIEQNCND
GLDNDNDWLVDCDDPDCCSSPSCAKNSGCKTSSLNFQINGDSTLSKIHALARQLKLSSRFSANEKLAIVT
GIVEDENLQGIRGVTVKSRNGEVFTAETGHFIQVQAIGCEIYEFERNGFEAKELMICAFENIEYQAPLRL
YPKNSPPDFFRLPPPALVTIQSDFSNSLLPNESLSDDFPVDLATRKVSFSSEILDGTLNLRLREDQDHFT
TVFVTFTSSTNQARVLITCDGSEVFSETFFKLEIGKTKQLAAEWNFGNIFGQQSESVGSCQVFIGSRYDL
ESPFTWSLARLALVKAKEPEMKQKTAIANFVPDFMSFLDRENGIIFHEKNSVIQRFSMQLEKELKATVQI
PVDAIFESDDNNEEIIIFSKKNGFFSKSPKSALRRVFTFQNAVGFSKSPLSRENVFFISSVDNIVKIENW
RETNVFRESGGKKIGQVVALSNDELVFVRGDNELVKLSNGKVTVISRQDGLEEPCRQGSDFRRLTSIKLQ
SIKDIQFDYSTKQLFVLDENNIWQVNLRTDDNWTRRIISSSCGQRNEIVSGASSLQIGQNSAILVSNQKQ
IFELVGDSKFVPILGSCTDEVCETISSSSAAKFGAILAFKRVNDGFFVVDESSGAVQIWSLKPSRATKSP
SGYEVGFPDENLIREYLPDGRLVVVKDIYTRKTLKILTYKNNRLEQIANYYKNTIKITYESKSRILLQNS
HNRARAQIMLRGQRPVKIEDEDGSTEFDWGKDGNLQKLGPYFLTYINSRIARVSLDNFTIDLSTISSFAG
LESYSVSEKCTTKLGTVQVGSYAVLLLDSNGRLTSISADATVSTSSSDFWKNVETFSRDPITSQPSLFGL
HESRGFTKIPDRTTEWISTLSNERKNHVTRMRREGATLMSYELDYSKSKPTGKTYDQLKTFTGTLDLDPA
GAIERLSLEYSETGQEGNKIEFSYSTSGALVGWKQLRDGVKIGMSFSRDRGNNIVEVNPHSSAPLRVDHR
RNQLKVTTAAGSTHELGYKLGNSKWSESEISRKYPSGQEFNRRREGKTSIYTRNGISFLQIKDAGLYRTI
TNTIDGQDYNTVELYSCSGDIRWRKSAVESVRFEINKDSSSTKVIYDDSQLAIESVKYKNLLSSETISIS
GSPKVSVTYAYDSWHRVNRVILEIPGFRSISRVIEYDGDQIYAIDKFSFFAFPEKALVQISSASPRMNIL
RKNHRSTGRLVASQLKIGEEPKFQSQIEYNSLKQVQKEVFETLSTNNETIFNTEYDEDYKPTRSSNGEKI
LYRGELADQLIFPNGKVVPIARDHLHRLTKFGDIRVSWTGAEQVNLGSDEIEVNYKGQPVKINGARVHFD
SENLPRAFGNKEFFVFSNSKLLYHVKSRDITEYFYDPSGHLFAIARKSTLSPQVDYFYVMTTGEASPALV
FDENGKIVKEVRYGAFGRIAFDSNPVFKLALGHRGRLCLTATVCQTKEKIWIQTLTGEAFNFAGHVAELE
KLGEIDKARSTLPSTTWWPYSAAEARELINSKRDRLVVEFFNSINIPIMPEEAGRLF

>E4YGV5_OIKDI
MRFTRTLRDPVKFQRAGNVGIVTLNRPKALNALNHPIVKMMRPQLKTWEESGIDRVILKAEGGKAFCAGG

DIKDLTLPTYNGDYDSGANFFREEYQLDYQLGTFPKPVISLIHGVVMGGGVGISCHTPIRIATEKTLFAM
PETSIGLFPDVGGGFFLPRIPIPGFGLFLGLTGQRLKGPDCQHGHVSTHTISSDKLADIEKFLIDLPENL
CAEEIGEAIQRFQISIGEFSLAGQIDNIQKYFENPKSLVELVQELESSTGEWEQKVAKKLRSMSPTSLAI
TFKQIQDGKNMSFYDVFSMEYRLAYQCLRHEFPEGVRALLVDRDNSPQWNPATIEEISDSLMQTFFEEKP
NTWHPTKPWE

>E4YGY3_OIKDI
MKIAVLFASIGNVYPSANDQNLQSLGQLYKAHGAIANFVYEQLPARPTVRKLGGRSKSATAQTQERLSKR
LLELQERQEHIVKRCGPGGFKSEKGTKDSDDQDGSPLILSRGKFDSNLDLLRPSHSTFLPDIKSQEISAL
DFDEPTDPFVPSGQAVAGGLIESPFFDRIGGRGPSYGPGFGPMAQGGYGGFQLEDFDPLGAGNFESSVRR
RRETVDLEDIRATRKEMIKKIKELEKVFSELEDFVASNLSDCSAQRVKLHKYKLDKVKGRFQKVIERQGT
AEIQRGRRRMRKTTKPRNMN

>E4YI28_OIKDI
MKIEPENHEIKGKPPKMEREDLLEESKLGFDFFFTKFPHVSKIQILWFFLVNYCWVVGGLVQSILDTRYN
TSYEEGLLFSPKDSCSQFSNIDSLCSSNMSLETCLQQLADNSGSLEIEACSSGYIFDKSVFTRTVTTDFE
LVCENAYIESTISSIFFAGLFFGVFIFGPITDKIGRTKASVIASFLNFERMISGFFSISAGTGTFVYTME
VIGPKYRTWFGCMTQGIFAVGYALLSLVGYLLKDWQDQMIVLTLAPLFVIPIFLYLPYSTAWIYSQKQYK
DARENVKLIGKKYAIETDEKFLDELENSVKAQQKSENTGKIYTQIGNWLFL

>E4YI31_OIKDI
MTYNLDRNRKSRIPRKRPAQFGITKSKVRIHEEKISEGFAASDSLCCFSDFSRPKFANGLNLTQNGIPVS
VERLENDPWTFEEETKPVLKYSSELEGILQENRRQMRDFVSRIPRISPKHEPPNPHCMTLAVWQPPPIIP
ADIPNAPILQEANSTPLQSPQRPRSPENFEITERGDNLSPERMDID

>E4YI36_OIKDI
MLRRGFFCARSVVRMSSEKAPAVQKVATIDNQVTHTGQAFPEGDFRMNRYLGGKKKALSARWAIDMIAEE
PVIMSTQRRHMCDGTYGGLNQAVGHPRVWINLDDGEIHDCSYCGLRYQMIKKEDK

>E4YI40_OIKDI
MKLSAALFVAATAQEVGSDRWTYSDYAVDSVSNYANGKAGAATSGAYGYGSGNGRFCHSTKDTVHIHRWD
VSKNGYFSHYNPVECVGEELYCFVEERAHFGQIIGIRAGCAQMMNHPQVERNDADDTNFDGLPYVEPVYN
QEAARGATFDQNSYGSISIYYGIGGCLAHPAQNGNDQLHEDFLTSMSQNHYNYRHGGWYQSQCLRTNGAN
GNAELLPFGVSVCRSCCLATYFDDLYKDADTTARVADTPTEEGVNRGICNFLPYPLPAGGPNIPDQTAFD
CKQGTQGSQSACVSTNDDDSDDCDFCTKQLAPSLTMYEVPSYLSTFSQNLFESNFVRPDTDTAGPCDSGT
CTPTAVISQPMA

>E4YI41_OIKDI
MIFCENSFLIVVRRHLLIRGLSTLPPLPPSQPNTIYNQPPPPLPGTVVVPRRGPAKIISPGAPIDLTDDK
QTQKREYSDSQTNRRKEKLLLRLLKRFVSEGKTFVPEKGGGLESLWTERGFDKKPPTAEEIINASQLPDH
SGAAGSNGSSGNSSSTMPQRTRAMDPNLGKIDDSRKPKPMGAGTGSPAKSGVVYGASGGQGYAESNAVEN
WSQDVQF

>E4YI43_OIKDI

MKCVMENVVGDSVMVVIEDEIGEHFKREECLPKRSRTSKDIRFALKKLIRMEFLWRHLNIRALKVSLMYT
KNMDVKLEPHERFNSPFFANKNNSTCVRFSKIEELNRARDVRWAKNEKISSVPRKKVTGKRVKKEDMKLA
NILTMFKNNDEVKKDENSMDSMKSEDSRSREPPLVNLTNIPGTSEMNYFQIMSEDSNSNQSINCEPRNPA
KNSTMNPKASALVEEYRNIDENSMDAMLAQIIDDPKEREALLSFSGAKAKPVVNHSKLQPTKSTKKTRGR
PKKTPPKKGSVQESTIFQMFKKADSKKSNK

>E4YI44_OIKDI
MAAGVPVIAMASGGPLETVKNGETGYLIPEPFGKIELAKTIAKFIEEFKQSEMANNCAKHVANNFSFEAF
TSQLETIVSQP

>E4YI46_OIKDI
MVYVTAKIFRELCERREDINNYYGVSLLHQKQNNGFAIDPVPEKNKATDESIAKATTFLAAFALEKSNQC
DRVPFSSILKPLFKDVLGGHLRNIIEMENNVRLDELDSQSHHITIYGDEDGRLEVISQFITIHEILSGEH
LPDGDDAQINETIESIFRDTKESTQNKRDFCALHYGVKRYLFNLRYLQKINIPRIRAANIQRGETSQKTM
APVPSATPPRIKNAFLSSFTAQGSRSPTPPAVISLLDDCEESHPRASAAQINPEDLGAKLIRDVKERFPD
WNREDAEMEWFKYMEECNISNRPVNYNEAMERVKSVAAPPAEPPFDGSVNEAIGQQVIKEISDRHRSWDN
VKAQMEMLQIIEEYNKRSERIDVNDAMRRIEAIANDIDFKDIEMEADDEPPETMGIDADVIEVWFQENYE
DTPAYNMAVYKSDLFKEFTSHFELYYSGDNHEKHYQNFIGVIHALLLKKKHVYPNVKTKGNTGVNARKRF
AHLRRKGTKTELQVEVRSPKTEENSIPGWTNLAAVVNPRIAPILVEQSFRTVQKAKKAESNPDKYTLRCP
WNERFSNLYPGGVDGDPTLRRIVIDGSNIARSHGKVGEIRRQHKCEVFSIIGIKITVEHFWNLGCRSVTV
FLPHNRQGNKGMPRIPEKERKLQEQMETEDIIKYTPGRYHKGTKQFIQAYDDRYILDMAKAEDGIVISND
HFRDLYDEFKDVINWRLLP

>E4YI47_OIKDI
MHALKTMTDEVVSQKDIDKVMKKLEAARDDEPDDFGFVSSDYKKPEIVESSDSEEETEFVEEEMSAYQLA
IRDDILDQLVETGKVDTKSKAFQTLRYSDQLGILDEIRDSFKFDRSYLTGHINSIKKEENSQKSQEEQDK
EFSKYQLDGLLSKNNIRKEIRKVEDESKKNNLRDIIENSQGKLRKAGKKYALFDHEDIEPKEKIAKSIFD
LEDKKIDQKGTLSESSSEDEFVSDDSEYDVTTEEEIKVPASQKHGILGLETSVGEIESSSTSDIVDIEIP
ESILMLARLAAQKRDKAATMEADFKKKILESSDSSDVQYVHDDKVRKAETLNGGDATPQMPLCALRQYLD
KSDDFVDVTDRNRISEVNESTSFKEFESFDHENDNQHQVKTNSKPTEDITDDSNSVQTTTDYAKLEEFVN
EDKKEHPEILEKCNDEIKEENLQKSSVFPINPLVQEKQIFLSDKKTEIKAENIKNRDDFPINTSLVSEKH
EFLSEDEPEMNVEPLSPYLNEFAKEVWEVLIMEMAEEQVEYAIEKVKFEKLQESEVLDESWSSFIRETIT
DIALLSLDKELQKKKSSAAVSDQIAEFTAASVLEENAVQLQKLSSALKITSVPSQQLYKDAAEMLTLFGC
GVFFAPGEAEAQCAEFEMTNITQGTITDDGDTFLFGGRTVIKGLTLGNMVPVKYDIQETEFSREFLIALA
QLTGSDYCNGIKSVGSKTAIKILEEFDDRRSEDPHLTLNTFSKWWNTHHKSLTTGTNGIPLRAKLKKLNI
DADFPSDRSRHAYLHPNVEKLKDKKIRFTVPDLNRIRQYAARKLEWQEEAIDQHIIPLLPEKMEKRKDIR
FYMKAK

>E4YI56_OIKDI
MKLFTGFTLFVAATSAFKFGGQREEGSKVQKFQAIKDKIQGLSEEERQEKKNKFQAIMDKVKGQAGQGKP
FQALKEKLEGLSEEEREAAKQKFMAGKKDKIEAMREKFQKLSDEDKEELIGADMLEKIKEIKESMKDMSE
EERAAKREEIREKIKAKFESLTDEEKKAKFTQFFAAKKDRFQAMKQKREQKRGSKSAWGKLQAMKEKLAG
LDGDQKEALQAKIEAMQKKREQKRANRA

>E4YI59_OIKDI
MEGGDDFFKKRLEKDLAELKKMIDSHFVQREKDEEELATLEQKIAERKEVLTTKRFHKPNKPFQMREQQN
AERARKNQERREPLQKEAEEARRLEEEARKKKEAMDKLNKLNSGQPRQRKGKGTARDVKKKILADRRKPL
NIDHMDLDKLKQKVTELYDYLSTLENERADFETDNEEKKYETSTLRLRVNMLSGAASKEKTKRIGRIGVK
K

>E4YI61_OIKDI
MREVASSDFEKKIEKNVKFYFQKMTKPKNPEKINRFLDAGQTVFDFGESFKNPEILCSILKNMKTSLELE
QHRKIEVDKNATVPFDIISSHLDALGAQERHELGLLTTPDALFGGFETQNVALLMMLKDFYENAPSPRSS
FSSSSSLSSVSSSEDETKAAYDQPESSPPSSRMSSEVENEVLPSFPASPEEEDSSLRGPRPETNVVDSPR
ENSTSPESEVQVESSGLVSKKQ

>E4YI65_OIKDI
MWILGLFLSLTAALKPLKYSSLESYSDFCSEISTFEAEYDHFSTRNTQKIEIRVITDDRFYQAGGPVLFY
TGNEGDVQLFCENTGFMRKAGKELNAKLVFMEHRYYGKSIPDDKNLYLSAEQALADYAEYLVHLKSSGVT
GPVIAMGGSYGGMLAAYFRIKYPNLVAGAIAGSAPVKFLPGLFDCRGFYRVTTRTFTNTPSEHFCSDNIR
KSWETIKLIGAHMVGKRTLSEVFRTCEPITDVEPLLDFLEDVWGTLAMMDYPYPTNFVGDVPGWPVNVAC
SHLDHDINQEELLEPLRDAASVYYNYTGDLACLDLGDEGGDLGYNNWYFQTCTEFVFPFCSDGKEDMFRV
HTYDFPTYSTNCQQTFGTTPREHWAEMFFSVETMKTIGGIIFSNGLLDPWSSGGVLTQEEAGPRNYIFIL
SKGAHHLDLRADNPADPEEVTLARTEYISIMKNWIAESISKTASSSISDCSKKLLFSSKKVHGA

>E4YI76_OIKDI
MRISSAVVIASAQASRKKEQDRKVPPRHPSQRLTTLINFGIEWCHDNLRNIDPDSVRGDRLADRFETRFT
IWGAKLRKLVEEDCFYFNPEAPNGGPGPSRARRAMFDESKLDTDDVHDDLQRYDQTNPIRGLKQITTGFW
KWSRRYIAECPGGQPKNKHHAKRAKTIYSKILDYYKLLVQRQEEKANKL

>E4YI85_OIKDI
MARTKYNARKAHHAHKKPGKPVKPKPKPESSSDEDEEPEQDTGLVDPDKAFPDEEDETDTIGQSIWADQM
ITNSVQALLRSKPDTPLAELAEEFCRWIEKTICRPSKPNGYKRWTATIKRSMLAFDDRQIEAASKFLITW
GQDSSKNLGSPEALRAASSGKISNERYH

>E4YH76_OIKDI
MSVKWHQNFDFSNFHRIFDILSCIIELNAALKPANPKALQFDDVLDWKDELEEKGHLRDEGKIKTIVLFM
TTRGSSCCKTPITQAERARIPELDFVSLLIGENDPPKEWQEAFKFIPETDEERLDRLLNIPSSTLSSQIV
NEPLLQTRKKRSTEIVAQDSERLKRSGQIVAQKNKEIHKEDEVAQYFLKVENFNTTFEYRMAQVTELICH
AKETETRYFSIRACDKAGNCGLPSPLAPVRVTLYYNSSLKNLVGLINSLPRTCPT

>E4YH98_OIKDI
MKIKKMLSKVFKFEMLVWFAWIHLTGSITDLIIDKYPIIWARSSWINFRDLCNSVNSKDFRKKLEKAQEV
YEIREKEVAEMEHQRDKYSDDEIDQARKNLREDKANLKAQEDIIDNGKDLAEMCENKEKQFEEYFGLKCD
DLDMRNNWLVAGSILGSFFCLTLLHSNAKPNFETLNGAKFVGAMLFAMIFTTANAVQCIHVIFYWFEARK
ELQSGDIHQKNLYKNLNYGEQPDKDEIKLICKYDFLRYNLIVHGCIFVSSFLMCVVLVCLNFKKAKYYRK
SMDLDNAPDQERAEEIKEKKHKEELKILNIPKAGENPKEQKKGGWNSDSKETTTGLAKNCDW

>E4YI86_OIKDI
MIIHNEGQTASFGPSLILAATFLSLIVVAVKSASCFKRKHEDDVPSTVDSFTISRWSCLCNWWGKSDPLR
WTDDDNKFTTDSVKSMNIKKEPKKVRDGKSYLPKGVDGVGSSTKKFLELAPEQMLPQADRRELEDNRLVM
KAYLISKLVAARYDFADGEMTFQEQAEEKQINVDDYKQRTQNMYDLLYAGQISVDTKATRKQQRNALKRL
KKSNMNNRPKKIKELAKQMKEEANRPAPAAVDRQSSGIGSTGDISKADTTTVATTTTNAASNSEYNASGS
KISQFLRKFKFKSSRDSAVGSSLGSIDQLGGVAVKHTEKNNNWATAKKPSHITFAGAQF

>E4YI92_OIKDI
MECSPLLDMFICSALSPACVDNDLSGTLPYLVPVPPCRELCELVTSSCAPLISEFDIQWPAEFSCDRLPR
SSEAQCIPPHDLSQPLPFQLSWTSTEIKEENSYSQKEEIAKLCPAEQITENDEVTYAGIKGCTGPCEPKE
TTQTETKLVRLIVGIVAAAGAIASCFSITCFYIDKKRFQYPEKPAVFFAFCYAGICLIVLVGIVSPFRIA
CASNKLENIATGEVFEQKGLINGIDKNTCTVIFMSQFYLAGAALCWWLILMISWALAAIFKWSSESISQA
APFFHLFCWTFPAILTSTALYHRLVAGDAYLNSCNLSSTTFSNWVLLFFPTVLFFFTGSAIFMAGLFALR
NDLTPKSKSALVVHRIILFTIFFMLPKSAFLLLKFFEADHKIDWEEAVLQGSNLRPGFVFILLEKLLPLL
PAFAPLVWAANRKSLERWGSSTASSVLTNSSFVGSGKGSETSLKSRKKAESIVSERSSRADASLLGRIPT
VPDNLLV

>E4YI93_OIKDI
MRLLYSLFLSALAGNYYETFGVEKTATTKEIKKAFRKLAMKFHPDKNKAADAEEKFREIAEAYETLTSEQ
KRASYDASGFADAKAENHHQKSDFEFNFDQFFKDFDEFFKTKDDPTKRQKRSSFDFDDIFEGMENEENEI
FRTFLGEDFFGGLDLGSMGMKMGGKKFKIKTSSTVTKDGNSETRSFTQDSKSNVKISKSSTNSGSSEINC
KYVRIEVSPGNFEQKYVCEKDEL

>E4YI94_OIKDI
MSRAKPALARKPSTVKCFRAHWGYEGAADDELSFEEGDLVYISEQHDGGWWRGTCKGRSGLVPSNYLVAD
EDEENGGSNGQIDFPLHEGAKRGNLEWVDECLENKVPINGQDRSGSTGLYWAAYGGHVEVVERLLQNKFT
DLNLQNKLGDSPLIGAAIRSHPEVVDLLLKAGADPSITNNTDGSALSVATNNQVKSLIKARLGLKTERKN
QSDYIGAESDNDSDA

>E4YI98_OIKDI
MKRGDSVLIDEDQIETPLVKTEIVDPDWIDELSQTKQKWLGVFLAVLSGAFYGMNFLPITLAVEQKIIAS
HMDGVLSHFSGVLIAQLSIFIVYSVYRKSKPDLYPSAVIPAMITGTTWAVAFLCWLYANSILGEAFTFPI
LATAPSVLGCVVGIYFFKEVSDKKSIILAVIGSLTCIAGVICTSFSR

>E4YI99_OIKDI
MSRKAEEHAKAYSSRTIDSAGALLLSAVYRKLDQPTAACEALKTGLERCPGSLDIMSTQARLEQARQNHE
KGDEIYSEILKHDPVNQEALCVLVKVKKSFEF

>E4YIA0_OIKDI
MVFYFIGLGLSNPEDISVRGLRLVQGAKRVYLEMYTAILADWNFTRPYPAVTRLIC

>E4YIA3_OIKDI
MAAKEEATRRIIPDLICLLQTSFQFPFSGKKAQADLE

>E4YIA7_OIKDI
MARFYQLEFIFSTRRNSDARRSLMMLLSNVSISPSSNCKVYSHERSESCSNSSLEIDSQCFEINKTQEEA
CQEFVFDLDLIYYTISIENNLVCNRNWVNKFITTLTMIGLVIGPTFSGSVSDKIGRTLCIIIFSFITFGF
ALTCGCLGTYHWGVFAVFRTLSVIGSAGVGVTYFVLVMETIGPKYRPICGLLYISTTIGLGTIMLTFVVA
FLKNWEYAQFFMAFTAIFVGFAARICSETPRWLYSVKKSEEAREALQRMAKWQGYRIDSAQFEAFEKNMK
DDDKRSAEESEQNKKNIKDLIASPFIRKVTIILMLNQFVRGIINYGFLFNIGSLKGNIFTNNIFNNLMGI
PAFVICACLINTRLGRIGNFIWTLYLAGLSSFLIFFGHLFELPLLVNISSYVGMFASTAATSVGYVYTAE
VYPTDVRNVGVGCCSSFAMFGALLSPSLAILEDYVWWFPSLFNGVIAVIAGSLSFMLPETIGNPMTATTE
EFTALYGKRSSKEIVEKYTKKEESDSKNGRHANGSLENPSFKDSEL

>E4YIA8_OIKDI
MTADLIDRQFKLGQRQFVWSCILIAIECLVVKLNNDLWLDPWRQMKNIHLSIFFTSVTVFFLMLAGIIKM
RNSNKKRLISKKKKMWLTIMIFLSINALFSYNIATLSTSSQPLSGHDYTGWILVVEMVVAGIIMIITLWP
YCGIEHYQSYRPAAPSQSCHRRSENIEIKYQNIVSREIDEDLPKYENLFSPSN

>E4YIB1_OIKDI
MKIEPENHEIKEKPPKMEREDLLEESKLGFDFFFTKFPHVSKIQILWFFLVNYCWVVGGLVQLLTIKRYQ
ILTDFRCRSTLDTRYNTSYEEGLLFSPKDSCSQFANIDSLCSSNMSLEACLRQLADNSGSLEIEACSSGY
IFDKSVFTRTVTTDFELVCENAYIESTISSIFFAAGTGTFVYTMEVIGPKYRTWFGCMTQGIFAVGYALL
SLVGYLLKDWQDQMIVLTLAPVFVIPIFLYLPYSTAWIYSQKQYKDARENVKLIGKKYAIETDEKFLDEL
ENSEIGCFFNEKNIKKILIIDQFKKDLFKPKKMRLITLVEMYQWFSTTLVFYGLGLGVGNLGSNLFLSNF
INAMIDIICYILLPFFMDMKCIGRKYGTVWTMLLGSVGCFLTAVFDYLENENPENSSYGILKATCAFAGK
FGVAGTFGIVYVHASEMFPTPVRGIGVGLSSAGGRLGGMIAPLINGLGNKTSWLPFIVFGVLGLGQIFTA
FLLPETLGVPMLTTIEEAEEFYHCPENFKKSAEEKQIE

>E4YIB3_OIKDI
MELQAAKYASSLHKLLTKFKGVKKQGKELAESICSRKTENKSSDNSFDKKLNQKCANLLELSESANSIGL
TMIENSKKWKSLPKAFSLMSESTDLISKSSCCEIANETESVAKQIIKQASLWINLCGKIYHLEERDDLVY
IVACWAHQPLVNENAFELLAAKSKYISAYSA

>E4YIB4_OIKDI
MKNDITEDVQIQALRFYDKIRNLDTNQYDILKGVALGYCLGRAQSCEFIELLNCFAINFSTFFHFCQFSV
SLTFKKDEIVSLKKLGEALKTEAFGGRLEKKGKPQSTSRDVRLRKAYSVSFGPRLAVDEQLSGRALRHMD
SVDFNSTKTQEELAQKKQRMSRVIDDSARHHYSISPELEAKTQEAIHNKYGGREKAENAAKVIQQFYRQY
RLRQSFRKLRAAKTRRLTLDSLNNELEQARKENQKQLEITQISTSSSESSTTTSSLKSPKRSPSSPPPNE
SGSKSDLDQIMPPEKAESRLSSISLDHRSDLSLLKHRESYADSDDSDEEEQSTKSRPPSEAKSFDSDSLI
RNQRLSWVSGSVGGNTDLLRKRMYRIGLNLLNKKPEKGLAFLIKQGFVEDSPNVISNFLITRKGISRQVI
GEYLGNGSDRSKKILKAVCNQMDLSDLDVDEALRKFQSSVRIQGEAQRVERLVEAFSQRYVESNPEITRK
LNNPDSIFVLAFAIIMLNTDAHSPSMRKEKRMSEEDFVNNLRGIDGGQDLDQKMLQNVYRRVKNKEFKSV
DDHINQVLVIEQRIIGKKPALALPFRRLVCYCRMYQVADVTRPQRPGLHTREVFLFNDLLLVTKVCAKRR
SQYTYRLDAPLTSLEVEYFGNEHYPHAVRIIKTGKVLITFSLPNQDDCKNFIQDISESILEVRQMESLRI
EESLESLEEQKEAQKSPLTASRKLSSSLHNISISRDLSSSSTVSTAIDASEK

>E4YIB8_OIKDI

MPLFGAPKLEDILLTLRMQEKTLEREAKRAEKEHNALKAKVKKAIAEKRIEFAQIHAESAVRKRNEALNY
LRLSSRLNAVGSKIKSAQSMKNVTKELGKTTTSMEKAMKSMNLEKITATMDNFEKQFEHLDVVNSTMENS
MEGAMATGGQTAQVDDLIGQVAAENGLQQELELLKAPEANKATAAPAAAEANDLNSRLQALRN

>E4YIC3_OIKDI
MSHLWQLSDGVKLERNGVERSDRSNSAPLHQNFGISLHRSATFNLLRGVKRSDD

>E4YID1_OIKDI
MDKLREILVDLLNPEKAAAATARMNFRPPNGGCSSAKRYRTKGKTVFTPDYHVQFSTLRQEEQNAIRDHI
LQSLTTEENASVWSAVAEISAGILRKNNEWPAFFMIVQQACENQPAKGAELLARMSTSAPAVVREKTTQA
KCLELIGACLNHSEPASLHQGLRAFSSILTELDESNQNAAKSLLQRSVGAVQSIAESGEGDWAAEGFEVF
QSALDCPISLLNADALKDLSTWAAQVFGTADADAGLRCCAGNFLTQAVSSKSKFVKKKKMTQDLMHLCFP
ILMEDEEDEDDDEEDTPRSIALQLLDTISIKLPNTEVLGVIMEFATRASQGQAHQKRAALSALAVICEGC
ASPLIEGGHVEPLVTFAGDSLQSDNPALRGAALYALGQFAEQMVGAMTEFAPRVLQLVTGLLESCPDDLM
KHSQLEKGLYCIEQLAETLDEDQIENLLPSLMSSMHRFMQMGNTDKCQVATLAVSALGVIIACAENVIGP
HLNNCISMLQALLPKSAAATAEETPENIMLQASALDTLATLASAAGESFVPMAADSLSLAGQLVSAETDP
DIRRAALNLSCSVVSLPNSPPQLLEAVPRILECLMETLKSTEGIGVKMIGDDENEIGSSAFDAVEDIDDD
IEDGDDGEEIEHFTIENSYMEEKHTALSSIIRMAQKIPQHIAPSSSELYQECKSLTDFVNEDVRKFACAA
LIHLGHAMFKVTGNETALVEAITKGIDTIGTFLIHTRNLSKLHEFSSTVRMAMMGELICMMDDDDDEDSG
VKLEEEASEIEREILDGAGELLVAVAAVDPQGVITEGLADILPAAAALMKKPDSGSKACAIGILGDMFDT
LGSNCAEGFVKQLLPVFMRFTSSEDDNIRNNSVYGVGVLIANSGETGIKMIPDALKSLPLNESKNLQVRD
NVTGAIARMLYAGHAETRAAYSSQFLARLPLTEDHTEWRMTLEVIEKLLTEGNQEVLGRLQELAGHCLNS
VNNLNPDITADKKTILKIGQFMSQLSNADPNMFAELKKSSPEKVWNKIVKVNTKAN

>E4YID3_OIKDI
MNILNNEGHNPLHKATIEGKDKFFHGLLDAGALPSVPSDSGRLPHLVLKHANSADKRRVDLLEKVLSYSE
SEINACDKKYGGTPLHWSRTKEDVRLLTKKGALVDATSADKSTPLHVMIKKQRTEAAYELVLHSADVNAV
GEQGNTPLHFAVMAGNLQLIKALILFGANYKMKNDEQKTPGLVALESKESSKEEILVALHEVDDLKEAKS
SRLNLRKNLRILSLDGGGIRGLVLTQLLIAIEQEAGRPIHTLFDYLVGTSTGGMAALGLMQKYKATDIQR
FYLKLKDECFHGKRPYHEAPLEAALQELYGQAKMLDINEPRVLVTSMLADRQPAELFWFRNYIPTGKVDN
FQAKTTDSEHKFEPTIEHRNDEVWKAARCTGAAPTFFPAMGRFLDGGLAANNPTIDALVEIIGEVKEYNE
TPESEESGKESIKIVVSLGTGNQPVVKSRATDRIWPTNALEAYKSLTATVELAKMFVDTVCECDRWVVYN
DETLTSSMAKFQIFGDDSDAKEIWGVLCQCLKDKNNNNDEDQLNNNETMSPEKIKWISRRLNMDENPNEV
ESYQINICCGDISLWYKFPTSSGSQHSMIENNPIKGNRIEPIDPRLEPVPALPYTEQEKQSYYSIPKRKW
RVSFDVALGQG

>E4YID7_OIKDI
MVEEELRKRATEKSEKKKDAPEMKKKKKKRKPLVYRGPETWVVMLWMAVIGGAYWFAVNVYFKEIRILAS
VQDDVDKFEEVKCSPEHAEKPQVPRCTPTKCGRLVLDGLLAPREIEVLKRMAIEGTGALGGGAGGASILE
LSSSTVSSGEKFQSLFQLREASVKNDNNDNFRIMVERLYSKDNLGVYYSAKTKVATAIETAFGLEDDVLS
LAAPTFFSQLSREPAKTKNDEYWHSHIDSKQYPSFDYTALVYLNDYEEDFTGGRFFFETSSIAGTEEEAA
VEPKAGRVSAFTSGAENPHRVEKVASSRRLAFTMGFTCNPEFAIADPALPPLPDQ

>E4YIE0_OIKDI

MGDQTSHALSDSDIAKLVTIVTLCLINIAVYLTLWRSYIRPKCILPYQDPNYQAADHNVFKPTNLRFQLV
IFMLGFIGAPIFILLGWLKVPGEHCFLFQLCFLTNAALYPLLLLQCMWSYRLYYRLLYMRGNFYLKVSAF
QIFTLVPFMLAFGYFNYQWVWIIQASQLAFNNKSDATTGSENGFCFSYLDNNGTETTLQYAIPIKVYVWS
SLGAEVFNMLLIVIFTINSYKEMKKIGYDMDALDGIGVMVKLVTAQIIHLIKWAALADPLIRLKWIDVIL
IYLVFVPIDTQMIFLPDSKWSRMVRNYVRQKLTAFANQVDLPMAPPKSSKVSTGNEAIVKVESEGKHPPK
GSAPPTNTDLRASKTSRESEVQGNQGQRVWDDDELATKAVKVFKEELAEMNRSGLPKIEDMVRPKIIEPT
ELRVVTKNPADKAPLGANYRINNSVYKGNKVFLVSQEDGYSLKVSDMDAGVFGAFKEAYTTKICENKYFL
KLFGITYTPDMNPVFIYGGRAKGSLLDVLRNKHQEVTYRLALKWLNQLLDCAYLLAKENIVYREFISANI
IIDSSEDVRIGSIYDYRNISDEAKIKMHDLTREDGLEIMRCFAPETLRDETKFTKESLMWQFGILYWEIL
TRGHVPYDQLQVSELAVNIEKGVRCRQPLECDLTLYKVMLACWHPDPMQRPILKHIIDFMKQYEIELTTR
EASMFIKKYSNKRACEIVQSVDAAKLGLELGYIDQEYFTNLKCLAQEIADKFNIDNEETLGFEMEKYDWP
NWALEVNSERVHVPIDIRKMEDPAKIQPMPRISHQKKYNLKMLMNQ

>E4YIE1_OIKDI
MRITFLLFFVGTLGQAPLFRSLQQALIELGATEPTEPTTIERRSVDGMKFADYVDDCLNRAFSFSAASTG
LDDLMSFIKTCQKEAMDDFF

>E4YIE3_OIKDI
MSLYPNLAEEIPIRRDEEEESEDVTIVLQIPDGSVEVTYVRSNAPIATEFPTISVEDSPLGPVLKFNSQK
IVLDQSIFVLHYDLEMYCIGGWQGPIDGTDYHLLLKLINPSEDLIVSTSCIFMNNARYKCLFSDEQIGPQ
AQEAGDIGDKVSAGILYAADGLSKGISWAGDKSKAMMEGRAQNYVETTPALSNPKEVSPGWKKTAEVASV
GTKYVAKGTGYLASAIGHVATYAGGKIADKVQEKYGAKDEKGETSSTWRNTTKIIGGALAGYGKVWEALE
HSGKKIAVASRDTTAKVVSHRSGEAAGEVTYNSMNVGVNLTKTFFHIEDMGMKKICKTVGKSAAKNYLQK
HLDAKKTRDNQQISQNQPMAIMQ

>E4YIE5_OIKDI
MEKHENKKEKKKFRIVHPFPPPSTEYYEEKFGGLIHSPILITVQSANSLNNITLLLKVGTRGYLFRGITV
KSELTFSSTDNPDFPFELHLICCQQQAYLCNVKFRVLVKEKDLESASFFNPSNFGAGKFHAKTSSEIDDH
LQICFCENALQKAREQFQKTKSKMNRTESEPKTQKEVRTMIAEESDLTASEANSIFEKRQMRRLALVSRR
FEKKINADAPIKRRKLQAERVSKFKSAEKKEKNEKNEIFLLKKKVEDHEKLIRELQRLVRPDDIPQSSDD
S

>E4YIE8_OIKDI
MSCVRLVLIEEIRHTREKFAQTKEPIFAGKGRQVFMPIPSSYWAIGNVQEETLSIIKWTRSRNFVLSAMI
HSGAWQEPCMHGADEFQEKGITNGAAWYSTIGTLQDYEYIAQGVMAFTLELGCQKTTAMANLPWHGYMGI
RGKVHDERGHVISNAEIFIEETYWNGTVALNPLSVLSDENGNFNKIFWSENATAKVSVHVTHDKFQEKTF
TLREPILRELKDFRHCRQTNSFLENSCEYKLPRAIKIKLLKQRK

>E4YIE9_OIKDI
MVINGVLDADFACLPEADVKLEKPQNCVVAGWGWSGSSFENQLQEKLVRVIPDEVCPRFDANFNKYTAAH
CSGTGACVGDFGGPLICAEKNSKTGVEEPVVRGIMSHTKGCENKPMIYTDTQGYLEWIRQKTAVIAAQDS
GTRPTISPTTTDRPLTAAPGEPLLPSGASCRNPIDGITRIVGGQTAKNGEWDWIVQFPQIGCGGSVIAKN
WVLTAAHCCKPFALSQLMTNFGDHNIGTSSDSNFLLRPILKIVHPQWNSKTFDNDVCLLKYSNIPYSDRV
APVCMPKPWDEEIKPGQVCYVAGWGKDHEDSNKLNNELKEAAIMGIDPTLCNSGWGLSKRLNPNTMICAG

DLRGGTDSCQGDSGGPMVCIDENNQPVLRGIVSWGLGCARSGMPGVYSKITRMLEWIHKEVGVKVPTTAS
PSSQFINIVQTALPMDLLCPITNDVAMAEKLEFSEVETDLMNKWADGFDDNYAPMARLVGGSIVQQKRTW
TFLTRMERVSASATILNIHWVLTAGHVCHGAKLPEFTLIPDQIRIHPEFDPDLLNNDVCLMFFRDIVSFV
KTGDSVSTACLPTDDTDVVPDGRRCFVAGWGSQDEHSAVQSDFLREITVAVIGGETCNKEEMYDGELDEA
SMFCAGSLDGAVDACLGDSGSPLICVENNTPVLRGMVSWGFGCGRAGFPGVYTDITKMSRWILQQTSRNI
ETEETNPALLPPNLKCTTRYQIEKQTKQGEIDDFGGRIVGGTVTEKGKWKWIVRLPTIGCGGTIIDDNWV
VTAAHCFANEYSQFTTTGDEVNRIPDRMIIHEGFDTKTLDFDICLLHFEQSFELEKDDRIDLACLANKGV
EPVDGKRCYVAGWGAVGEGEAQSPILQELSVNIIDREVCNSDEIYRGGIQPSMFCCGRLVGGFDSCQGDS
GGPLICVDNGEPVLTGIVSWGFGCARKGFPGVYAHVSKLADWIHTKTFGTTAPVESTSISLVNEFDTQTF
EGVEKTFKPAEFIIHENYDHRTIDNDICLIRTSEKINLENPDIDPVCISTREPPVGRKCFVAGWGAVKES
GQGATVLQEIQAAILDHEICNGPDAYDGQINKDTMMCAGTMSGGFDSCQGDSGGPLVCVSPGREPVLQGI
VSWGFGCARPNAPGVYTRMSNYEGWLREKISADTSFTPEAETLSTTAGTTQAPLTSQKPTSSAPTTQTVT
QIAPEIFPGGTCPVVRNSWTGSRAVGYSFSPADYEPGFGPSTVETEKGFVSGEIEAEDGRDGDRIVGGLE
VSEARQKAWSFIVHFDRVGCGGTVVARNWVLTAAHCCQPVIDATSDPAVRNLLMRTMLNRLNKSSLSGEA
VIPKSIHLHSAWNKGTLENDFCMVEYEDDLFAKTTKATCLPDSAETMNELRLPILENSVCNHPQSYKGFM
KEDSMFCAGHLQGGKDSCQVLVGVVSWGFGCARPNFPGVYAKVSHAAAWIENDTSSTSTTITTTATSTIT
ITSTTTTTTTTATTTSTTTATTTTTTTTTTTTTTSTTTTTTTTTTTTTTTTTTTTTSTSTTTTTTTTTTT
TTTTTSTTTTTTTTTTTTSTTANNVKTSCGDFIPGVGSFRNIKSKGGSLTTICNNNICKNSCSNPNEMLV
GPSIVKCQVKGKTKKVLPAKAFISCAEKTDTLCGNLAPNGFVFADGHEGSITCAKTGNFETCTAKCSDSS
LSASIPTIFCKTIKRVTKFIPASAPEEIISPPPTKCGHFASSFTFEKGSSGSIGDCRQVGKNQVCKVTCD
EELAPSLEEIQCRAAKKGKFNFFPKIAKISCRVPPPPLPKNVHANCGDIFQVGPRYNLDEQSLSAECDKV
KCVLSCKNAGASLKATPSKLPKNGEMIIKCGKKGFAPKKVKATCIGGSPQRALGGLFDDETATKQNKKTS
CLNSIYEKYAVSPDSINVNCTGNKCLVSCKNTGKPPRHVWPDGSSLPRSLFTCKGRFSWSPIRGRIVC

>E4YIF9_OIKDI
MKLSAIVLNGVSAFDMLSGTSHKCGGLNPKEELRIGDLNNELMSIEERMHMSLSTEKIVGGRQVDDIKNW
NWIGQLGGYCGGSLVADDMFLTAAHCCGSTRIGQTVYFGVLNPWEDQGKAQKRKVSEMLNHPDFDRPTLT
HDICMIKLDSPIDQDRNVRPICLADSASPKNTPAYVAGWGLTSEGGPQSRDLMEVSVPIVTNKECQNAYS
HRPVDDTMFCAGKKEGGEDGCQGDSGGPIVTVDGDGKVSLAGVVSWGVGCARPGKFGVYSRVDTQLDFIH
WSIQTLRGKYDGKLSNFDLNKNALSGAQTPNEGEPATPHNGKEAKNSHQSDEDVDCHD

>E4YIG2_OIKDI
MGDLDPRWDFFQDFTLRTLSLKADKWGKLVGNDDQKQLILDFLEKKDKLWLIITAQGSGALTASEIPPES
SKTKGVFFFKREEKSIPKEKIPNLVSFGDLSQIPLDHLSSFVDDVVDGILSYNDNFATWPSVVSSDVQAH
VHKLKSEVYEISGSVKGRTLLPMPAGVENVSSDGDLSHVLHKIEGIIIKWTHQVKSVLELKSGQALIDGK
NPGPQEELKFWADLSSNLNCIYEQLVSSKVKKMGRILKEKDSSYFQSLLAMYDDVRIHLEEARDISMHLK
PLQSLLDDIEQTDMSEIERKLSALYHLIALIWRSCDYYRTPARVVILLQEVCNFMIELTKNFLDPENLLK
VEIGGTRGHTLSAQVVVLYEEFQELHKKMSEATFDPLDPDDNRFGNLYAMFKEKLYDMDRRLASILCQAF
DDCASVDQMFKLLAIAGNLIERPVIAEDFNPKYKVLLETFDEELNTCKDIYDSCYEDPPLHKNQPPTAGK
LKWANELLRRIQEPRDRFNLVESPLVQTEDAQIIFKKFEQMVDLINDFKKQTYAKWIATVDEDCTFNLSK
PLLERDAETNLISVNFNPKLEAVLREVRYLNFMGEETIPDSAAQLYEKHDIFRQWVSSLRQTVHWYNKVR
KTVLDVEFPLISSQLDEIDQKLNQGETTLDWSGDVLSYITEIYNAVSDLEIRVQKAKDNVIKIQEIMDVW
AQSPIFERKHDSKEPGLLNLGDRTDRLKKRYDIITTDGQKIHTLLTENVGFFKATESSTEWEAYKEYIDE
MVIEGLFNSIITSLEFMQDNMCAEVSPFFASELELRSPDMVFQPSLDQKVHNGFYDLIEGLLDDIYHISS

LVPRVSGDEGYQNLMEEHHMLNELRGDIMGRVNKVMNDANNFCDNFEIYSNLWIDDRQEFLNQFLTYELT
QFKEQIDSYEDLYSQVSGFSATEIFDGWFRVSIKPFKQSLLNIIKKWSYMFKEHLIKHVENSLVDLQQFI
VKTDEGLKSEVVEGDLDGLVNIMGFIIACKDRVEATDNMFSPLKATIELLKAYNHEMPEAVYKQLSDLPE
KWNNTKKIMVSTKQEVAPLQAVEMADIRKKCAAFDVKQYEHREAFRKLPMFQFSCEAPYDEIDLANKELV
AMETYMANLSDNAKLFEVHVPEFKQLKQSRKDVGLVKEVWDLTNIVTSSMTEWKKTLWSAIDVETMETTL
GCLRWS

>E4YIG3_OIKDI
MISIIVKLLFTNSIVATVDLEKSIDQILACSDILGLDSEGHVPTICYEAAVGLTPNLRRGRRSIHQTPFQ
VPSMNQMCSSECKRYPRLIRIDFIEKYTNYVLSNELTSNPRQRRIDFIDIGSCAGTCQATFQHRNVHNKL
IEYFLSEMLRRITSRAFRRAASNYVPIEDQMFGINEEQIELRYQFRKFFENELPESLLRDIDKNDDFPGL
RDFLKTMGNMGLNGPGISEEYGAKDNPIKSAAIIGEELSRKSAAISMSYGAHNALNVRVLDANGSHEQKL
KYLPDLISGEKIGALSMTEPTAGSDVMSMKTTAVQPAGKDYYVLNGNKYWITNGPVCDTLICYAKTDPTS
NAGDSITAFIVDTDTEGFTANHIPEKMGMRGSPTGDLHFDNVKVPKENILLGEGAGAFVLMRGLNIERLL
GAAMPVGIMQAVVDQSFPYAHERKQFGRPIGKFQLMQGKMAEMYSSLSACRAYTYSMLRSADQDPEAMSN
HECASLIMFVSDACTKVALEGIQILGGNGYVNDYPVNRYMRDAKLMEIGAGTTEVRKVILGRYFNDYFLD

>E4YIG4_OIKDI
MWKLSIYKNFRQYFGAFIFSYGLSSVFFTDILFEKKPEGVTNAPRIAKEIMRDHEDTRAFFNYDKNEFEH
PVEHLNAFLNFEFGYTWPVLHKPSNCTKVHGFNQTTVLIGVKSHPSGIEKRNLIRRTWSQARYWTNQIFG
KTPIQLKTIFLVGSTPTTDLRDEAEEYNDIYQWDFTEDLFNLTNKDHQLITFFENHCSFANFIVKADDDI
FLNPKVIQRLIVKNLFDPKRSVNGTYRNIPTGQLVTFDAPIEMFGSMVIGAKPFRDPLNKYFIPNSFYSD
AFGDYPKYFSGALFVMTNRALSLIGPKARETPAFPIDDVWVGMVLERAGLDNHLNHEYLMYIGLMNEQEM
KQANLNNTCFLAGMPTYHRLNLEVIEMTYKMIYSMNLEKCMDESILYDVVGRFYKKSQFIARELIQGKDH
YKNKIYISQIIMMIEFKNGAAISSFTSEIHLPQTV

>E4YIG7_OIKDI
MENFATTYDDHLIQKSDGGGEMNQLGGHFVNGRPLPNHTRTKIVEMAREGTRPCDISRRLRVSHGCVSKI
LQRYHDTGSILPGSIGGSKPRVTTPQIVNKIRSYKRLDPGMFAWEIRDLLIEDKVCDTNSAPSVSSISRI
LRNKIGNIFYSRQEDEDETPPAKTQRTRKRTRPHQEVSSTTSAQAKENKLYSPPPAMNHLHGNVSNGSHG
RFIAGSPGSISEASLICSRSFHSSSKLCTSHITSHFLITPCPPTSISPSISITITIKVQVTKRPVSRRVT
NF

>E4YIH3_OIKDI
MLMIRRVAHFIQQLDDQSGQTHALISVTEEKIKALSKAADKSEKLGAELTSRAANLEKEISEARAKELGI
EDRMEEIAGYLRQERSTRTIDNRRLLDFQLDEEKKNRELKMELERAEQRTVHYEEIRVRIGLMEKQLANI
VARGDSAEERGNMNQELLEEQIALQKQRELESIEHNSNEDNLLEEVRQKRELVQAAEDRFDFAEKKRIEL
DVIAKALTEQLLQAKKQAQLLSN

>E4YIH5_OIKDI
MRKIFVLFCLLANCFSEGRFIKKRINNNPGSYRRAQNSYRKKERGSTPYDFSKVKSYSITPGRGRVMASQ
PVELRGIAPLYIPIHDRCSNIEIIGTSSKKHTRQKSTSKIYAGVYHKLPLAQKWTRMTSNRKGQKINLFI
RKANFRFGRRKYFGWIMGYELQGQIRIRYFSTTKADCPAKVGRWFSVQHLRKYSANPDKLIQLRRQKETR
PVIFEIREQHFL

>E4YIH6_OIKDI
MTTTASASNNWVGCHPDMNECYRTQLACSVSMTYLSGPDKYQFDSNGIPDHNAWGMAGNSATIVEQSNSF
RVPRVPVLLDIDSMTPAGQGAIAFAKNGVSIFGPYNSGCCDATFEEIRSMDYCLGHPANGNYHYHYFSKN
TEGGAGCLNSCAAGVVSDLVGVAKDGFPIYGPMQWWSPSEEKIYLDAAACADCELRQIREYQTDVCGGIE
VADGSAEDGTTYRYIVTGTFPFNLQCYRGDISLSRTPAGAGWREFTVNNSCGINNTEGGTCELMNEDFLQ
SLGCTPGNCPYNPNTLRKLNYWQRSRTHVVDDVYHQCDGCTDDNCITYTTTTSTTSTTTTPTTTTTSTTT
TTTTTTTSTTTTTTTTSTTTTTTTTTTTTTTTTTTTTTTSTTSTTTTTRKLKFRKL

>E4YIH9_OIKDI
MTGIEPVPESIDFPKTEEEILKYWKEIDAFQTSLKQSKGKPPYVFYDGPPFATGKPHYGHLLAGTIKDTV
TRYWHQNGYHVERRFGWDTHGLPVEHEIDKTHGITGPEDVAKMGIDKYNALCRSIVMRYSGEWKDTVDRI
GRWIDFENDYKTLYPWFMESVWWVFKQLFSKGLVYHGQKVMPFSTACNTPLSNFEAGQDYKDVNDPAVSV
SFPLRDEPDVALVAWTTTPWTLPSNLSLCVHPDMDYVKILQKKDEKKLIVLEARLSELFKNDTEYDVLEK
FKGRTLEGKKYVPLFDYFVADFPNAHKVCCDTYVTADSGTGVVHQAPYFGADDFRVGIENNIITKDGLTA
CPVDNSGKFTNKVPDYKGLHVKDADKEIIKTLKGKDRVIKQGQIKHSYPFCWRSGTPLIYKAVPSWFVRV
EHAREQLLKSNDMTTWVPSHVKEKRFGNWLKNANDWCISRNRYWGTPIPLWASEDLEEIVCIGSIKELAE
LSGVTITDLHRESIDHITIPSKTGRGVLKRIPEVFDCWFESGSMPYAQKHYPFDNKKAFEDTFPAHFIAE
GIDQTRGWFYTLIVLSTHLFGKAPFKNVIVNGLVLAEDGNKMSKSKRNFPDPKLLFDKYGADAIRLYLIS
SVAVAGGDLRFKEQEVKEMIRDVFLPWYNAYRFLVQNILRHQIDTNSTFTYDPALIGTSDNIMDKWILSY
SQSLIKSVKEEMETYHLNRVVPKLLKFVDQLTNWYVRLNRRRLKGETGAEDCVHALTTLYTVIDWMNRLM
APATPFLTEHLYQNLRRLQPANKQDGSIHFQMMPEVNDSLIQMDIERAVSNMTTVVQIGRALRDRKTLPE
KYPLPEIVVIRPEQVQLDELKIVETYIMEQLNVRKVTFSADRSAYGVELKAEPEIPILGKRLGKQAKALF
AEIRKMDTDTIEKLRADGKMVVGGHEITKDEIRIQFNAKSEGTVKYEADSEGSLLVLLNISLSKDMIDEG
VSREVVNRVQKLKKKAKLVVTDEVTVFYQTPKGYLDDVITSHFEKIQSAIRATFVKSDCPGYADVCGTTK
EEMKGEFFKTKTVLNLQVAHGTIDPSAPVPLCVKSSTDSGVTTLLENPKGRPAQKITVKSCGESSQSDFV
NLEFVGNDRYGVVQDPKAAIILNNFGHEVKSLEQVESLAADLFGWGGDATALKLFFDKNCTKPVKSYSDL
KNATVFVKNLAKPIA

>E4YII0_OIKDI
MSDTEDEPSSQDIIEEEVGDEMEVVDAKEAEGGENEVRKASGSESSEESDDEDEEYEVESILQSRFVKKK
KQYLVKWVNYSDEWNTWEPMENLEGSQDLVQAYEKKEEEKIQKEESEKAAANERKRQAKAEREAKRRERE
AEKEAKRAKKEAEKPKSEAKPKVDSKRKSRAIISSSDSDDIAENEKKPTEKPKIEKNKTLTDAERKKKER
KQDAKRKALKEKNEFRRLELEAERRKNMTNAYKNPGVGKDDIRAPTPEVPPEIQSERAEPEPKSTEEVTL
RFLFGSDKESYGWQLPRVAEEFSRHKIVKVQKKVKCNVIDSMTASNIPPSDKAKLSHGETLIELTGTSSL
LSNLVLSIVRDLAGFRKSLPKYKQSDNKHLVLSVIIRNEFWETVFGKGNAEFQGFRKQCFSKLACGLDLP
DSTERLVHIQGDDAGLAVSVRRIANKLGKAFGNNLENANADIYQPYLPRCYALSGDQRVYVHSKIESAPE
ESRLSILSTITSGSCEVALAHQDENDDHLDDLSLPPHPGFNFILTGKSSCISIMKYYLVEDGIMTKHSLS
EIELGIVKEEKEKSDREKESDDLTVDSSCFYSMSNLACDRSVNIYGFVGEFDTVAITEHMAQFGAVEKVS
RTEEEAFPVIVKFAESASALAALKKRDQVIRGIKILVIPFRFGDEKCSRVSRTVILENLPAMNESSLYTK
FRENGVAMARIWPENPKIAFVIFSTLKIAKSTENMSPISMQPIFHDEEGTKRVDAHLLTSRIFKDQLFTA
EQRLSIFEDFELIRIDTTDINRIQSIPWKEPIKLHSAKRPPDSSSIPRRAKKPRPPSESLSIETSPIKPP
VKRPPIAPKSIAPPSNDSVNLLATVMTTQIVETNKQLIDARNNKLKVYKSSVEWSYFVANADAKTQADMI
PPDPQKKYELNRIFFQRAFEWKHNIDEWVKNDKQRKMRTRTPEPDLNDFEEPMDGPWDPATAEPLNDSIE

TSLGPPSSGILCTDEPQNLAEVHEFEMMDTIEDDPFLEPPSIGSSGGWNLGSGSSRSRKNSNKTKPSPQP
VGQFMPQRGRRGKRGKPDDATPRLNLTSAQLDNVNIGSNLAATLKALGQNRNILDNLRNQPIEEEEPEMN
PIASLSANPPQEIRKILRNLKLGKSDLDLLLNIPLKVMTEHNVYALIDEKHHDREHVNKKDPAKLISLIR
MYLTPPDDAQDINKKTPEKDSPKTPDKDEIYKCVEFREILESSLKLLKRGKAIMTEIYDMNSIELEPGAD
F

>Q9I7L7_DROME
MRFRSLKNIWPKEKLEQFLFLFILCGLPAIYYVLMEIILPELSDYWSPGYVFQLLLGLFLFSNVMSNYVM
CILVDPSIDPKLMKNQLVRGQHSEDWHECDKCGILAPPRSRHCRKCGVCVLMRDHHCFFTGCCIGHENYR
YFFYFLIYFFLSCMISLTSSSIFIYVLHGGRYQLFMLTHPAPNSAYFNSLIIRIIYFKLPDIYELVFTLV
FVLLWIGVCVATYVAYDQWSRGYFCYDFELQNIPFDRKLRRNFKTFLGRRMKWTWISGFVPSQLDHDGFD
LDPDNERVADWCSETTIKDG

>Q9I7M1_DROME
MVTDAMEQLRHVFQLVIDEAELILPPNSKGHILRELKDFVAALDTLDFDDSFELNRLEDALEELESIISI
AGSKEFESEADEVVVQLFIQHGVEDLEQILERNLETTLKIVEQKVEKYMSTWSDSRLARNAAMVKQFNDF
KNEKDVYEKLDKLTNSDFI

>Q9I7M8_DROME
MASNKECLIIVLDVRTCAAEEVKLKSAKCVAEILKDKIVCDRKDYVSFVLVGCDTDEIKTEDASHPNVLP
FGEPRLCSWQLLLEFFQFVNKTACEDGEWLNGLQAALELQNVATTLRVARRRILLLFDFNDFPQDYEKFN
EITDELLGENIELIVGTHNIAYIDNAITSQPQAIFNFSRKCGPDELNNQKYALSLVPRCNATLCSFKEAL
HTVFKVTNRRPWVWNAKLNIGSKISISLQGIIAMKNQTPVKLVKVWAEKDEIVIRETRHYIKGTEITPLP
ENLITGYMLGGTPVPYDEAVLEPKEPHPPGLHFFGFIKRNAVPDEYFCGESLYLLVHQKHNQSAAVKLDA
LVRALVSSDRAILCWKIYSTKFNRPQMVVLLPRLADDTHPATLYMLEVSYTSQHHFWDFPALRTTKTECS
EEQLNAIDQLIDSTDLECTLRDTQQPRPWAQNDLLPFDALPSIFEQNVMDILERKVIYDNDKEDKMLKDK
NFADVFWRVPDPLEEKSKRAAAIVKKLFPLRYSRAWQEKLLAKEQAENGVAVKSEPAEKEIPLPSDGVGL
IDPISDFRRVLASVHTISNATERDARFQALAADTRVVIITLLQRRKQNIGQLGELITLYRQSCIDFNTFL
EYDKFAEELKKIALAKNRSEFWQDVMVDKQLGPLVLGEPTLDDELALKAYYTIENWVESGANDMEDVEM

>Q9I7N3_DROME
MVVCRFFQQGSCRYGAKCNNEHFDVKQYLKADMESSLNGKMWPLSAYGPFKDKANFPNFIQDQSFEEVRF
MCYEAKRQNQFEQFSQQFNREVLEANNKMKTMLQMSPQVLEMMIKIHETPEGGTVSAAPQQSSNPFAAAA
PAAANTSASIFGKPTLSAGNIFGNATSQASNTNSIFGGTISNANARNSIFGVGAAATGAQSVFGQPQQQQ
LQPAAQAFQGGNIFAQAQVQVQAQPVNPNPFGGFQQQQQQPQSTGIFGQAASQNMGGMFTQASQQLQQQQ
QAAASGLFAQAAASAFPNQQQMQQQQQMQQQQQMQQQQMHQQQQMQQQQMQQQQQQFQMQQQQQMMQQQQ
QQAQPTNEIQSSVYSRMEDLNEQEIEAFKADQFLPGALPFKPPPRNFC

>Q9I7P6_DROME
MMSEEVIAKNISLSSLTYPKRIPKIKFGPIKDEMGFTLSERLALRQAWNLVRPFERRYGQDVFYSFLNDY
YWGIKKFRNGAELNVKALHSHALRFINFFGLLIEEKDPVVFQLMINDNNHTHNRCHVGSVNIGHLAQALV
DYVLKVFHKVSSPSLEQGLSKLVEKFQNYQDQQSNTSGYNRLSKVNFDSRPPRGNP

>Q9I7Q5_DROME

MQSNFMWLVAFLAIGICLAFPAGEDAQAETIKLESENTGDKYSFAYETSNGISRTETGEVKPGAGEEDGS
LSVQGSTSWSAPDGKKYEISFTADETGYHPKFRLVA

>Q9I7Q9_DROME
MSLASRILQQGSRLWNGLSAARGFHLLTRPAAPAIVSIQSSQLVAATTGICQTSGLLTPGSTLVQQVAGF
KVKGRLKRRCKDCYIVVRQERGYVICPTHPRHKQMSMKKRDYKSWILTHATQSKERGY

>Q9I7R0_DROME
MAAPVIVEATVKQTATLIFMHGLGDTGHGWSSALAAIRPPFMKVICPTAPTQPVSLNAGFRMPSWFDLKT
LDIGGPEDEPGIQSARDSVHGMIQKEISAGIPANRIVLGGFSQGGALALYSALTYDQPLAGVVALSCWLP
LHKQFPGAKVNSDDVPIFQAHGDYDPVVPYKFGQLSASLLKSFMKNVTFKTYSGLSHSSSDDEMDDVKDI
ISKWVN

>Q9I7R3_DROME
MDLEGKNVVYLGGFGGIGKKCVQELLKKQIKGLAIFDLIVDDDLLAEWKKQHPDTEIFYQKMDITQKSDI
DAAYKATAEKLGHFDVVVNGSGLLDDRRIELTIQINLVILEIVLSKGFIYQYFLKVGVINSTLTALEYMD
KSKGGRGGLIVNISSVAGLQPTPLMAIYSTSKTGVTTFTRAMASPIHYAHSGVGFITICPGYTNTGILKD
IDKKTTFPFYETRMRTVFSKVKGGQTAEVCARNIVNAIETAKNGAVLMLELGEIHELDIPNLWNPQLDDCV
FVCLS

>Q9I7S4_DROME
MECSDFMQQLRSFKPQSLGCAVARRKSSTTLIFPLPPQACSDEFCEEYPHNLMPTPGYWIECSRQKITMP
TPHTIWDESDSEHEDIRAGSASDAYLERECSDLYEDDEDSEATPSPRKKTTIEEQWDQQGAFELISVEQE
TYEKYFYGTEHWNYFTSDEDLGPVILSIKQETLNGRDQFRILVRAGSYTVHGLIPASCVFADRYNREEVV
RSLGKEVNLNPPLTLGQLPDTPEELLKLDQVFIKSELKVGVIFVKEDQYTEEQILDNNENSPLFDEFLTL
LGDRVRLRGFDKYKGGLDTVHDLTGLFSVYTNWRNIEIMFHVSTLLPYEKHDPQKLQRKRHIGNDIVCVV
FLEADNTRFSPACIKSHFLHTFILVRVSARIKHKPTRYEVSVVTRDEVGAYKPYLWEQSVFEKGPMFREW
LLTKIVNGERASYSAPKFARMQERTRSQMLEDLVMNLSNHAETGQIPKPYRRGSWRPIGEDEYIKMLHAR
VLDNNTHHEQQQQLLLQQQHMQHTQHMQHLPSHNICCYCNNNATELHTAGAIHNNNDNNNTTTTTTFTHR
HNNNNNNYSEQQQQQLPQQQQHDSQQQQQLLQPQQHQLLHQLQLSTSSLTHTSCCAMLHLCARHSHMRPS
SPLLDSVRDQFEDYDQLAKDFTRVFLNEEPSCLTNAHLFDVVFLVGQSKQKARFIGVRAILGVRSRVFQE
MLYGIQTGFGSPQIPVAEIFARPAPSLVSPQNQKPKSNNYLTVPDSDSIRPKSVPSSPMVKRAFSRLGTI
TAGWGRSIRNKNTNQLNPDDKKKWISSTDYRDSKDKDKDKPGNNQLAVPRLSVCADAQKVDRAKLAQTEF
NIIEFDPDTFRVLLDYLHTGTCPLTCVSIPGLLCAAEHYDLPELLQACFHHCKQFLRIEVVCPMLISLEN
YYWRYTSASELVNMILSFVESKAHSLFKCPEFLHLSESMVQMIMCRELQTPEIRKFEAMLAWAQHKVGKL
KNHPNKDTQFEFECIMERLTRDLNLCRISPSELLTVVLPSKSMKNERIMETLMVQVNLGTYRMPELDAYR
QQLRQQESAEATVQVHRAHQNHHNPPPIDARAYAMASLAAALEMQNSSNPLQSNAARDAADEEVELYASF
DNAPSTSRVAAQMQAAKEARRKRWAADGASGSSGDGGCASGSSGSGRRY

>PUR6_DROME
MSTTTTASIEGYKLGKVIIEGKTKQVYDLPEQPGLCLLLSKDRITAGDGVKAHDLAGKAEISNTTNGQVF
RLLNEAGIRTAYVKQCGAKAFIARKCQMIPIEWVTRRLATGSFLKRNVGVPEGYRFSPPKQETFFKDDAN
HDPQWSEEQIVSAKFELNGLVIGQDEVDIMRRTTLLVFEILERAWQTKNCALIDMKVEFGICDDGNIVLA
DIIDSDSWRLWPAGDKRLMVDKQVYRNLASVTASDLDTVKRNFIWVAEQLADIVPKKDHLVVILMGSASD

ISHSEKIATSCRSLGLNVELRVSSAHKGPEETLRIVREYESVMSNLIFVAVAGRSNGLGPVVSGSTNYPV
INCPPVKSDNMQVDVWSSLNLPSGLGCATVLYPEAAALHAATILGLGNFMVWSKLRVKALNNFITLKKAD
KELRGVRNA

>Q9I7T3_DROME
MRALCLLPVFVLLGRFCSLEAANILCLVSTAKHNNPGWSKPLFDALLANGHSLLVISTAPNPEPKKQVDG
LVYYHLPNEYDVMKRHFLLEEPREYISMVTLNQLLVWYEVLLGSCRALLNSDTMSSKRPELTAQLSMEYD
LIITDVTQGIECLMDSVSGWRSKPVLGLSAGKLTPDLMSLLRAENTINAARIPHYISQVPKTMGFWNRLH
NHIMYYAEPLIHLVITRPVLSELMKTENAFPKLQLVLLNTHPTLDYVQNLPPGVIEVGGLHIKNQTSPLP
TYIQEFTEKFFDGIVYINMPYIEYMNDQGLKAMYTMIHGNPNVAFIWNVEQLEQLPAKKPNLLTLHVNQS
LQQDILAMQYVKGFLNHGDSFSLQEAIHYGVPVVVLPLKLEEFNNAQRVMERNLGVMLQVKEFNQSSLSD
ALTRILDEERFISALHQAQLKFRTRPQSALELAVWHAEQLIAEPRLFKHFAQTETLAQNFFVANSLDVLT
VPLIVLLAAVVSLANLVYVLYTGGSKRQQTLEKAVLKKRKKSKKSSPQTFTPVNVTLKLETSELLEDLNN
EILEGEEELLKGEEKPLEEKKED

>E4YGN2_OIKDI
MNYNNWIGGMPDPGFLDTDSIHLDDLFIQSQALKNEAGTKSKDDDSGISTGLNSDAGASSPESATYADTT
PLLSIKQEATESLQMGMPLPTLSSPETAQLSDSYYSNHSSSPETERTGSKKTAYNEKWGVKVLKAWAIEN
QEQSDFENLPPDELNAILAKFWSEVRKSNGDYYGRNSLFNLRAMINKHLKGKPYSVPFDIVTDERFRSSN
ERLEKQLKLLKGIGRTITHKQPISIGDLRRMYDSGVLGTSNPLSLLRKVWFEITLHFCHKGSESQEKLLK
SSFEIYRDASGRAYVSRGRPHQNRGEIDDVRMYETGGDLCPVKSYQLYLMKLHPLQPRLFQQPRRKATPD
SPMWYGKAPIGEKALQQMMANISAAAKLSKRYTNHCVRTTALEQFSTSRRVRPAQSSSVGSSPNSSPKMS
SPEQSSSRTSPVVQSPQMSPRQSLQLKAAQHAAQYQQSLQAYAQNQIMEHSLNQMKQNISQLNSFLAPQS
AHSINPLSSMGVDPLYDAPPATPVYHADEIEAGQHMSAHMSSPTNMSFDSGASSPNSRASSEHRDSICKI
SSEDLLNFQASAEAQALMLQSLNSLQPQDIQERIAAMGSLFGLDADPFSINLKHISSLSGLGSGSPVSC

>Q9I7U1_DROME
MSKDADVEQATKPESGVVQVEPAIPEQPQAAVPTTISTATTVIAVTNGEKPAAATPATTATPATAEQVQP
ATTIAGIELATPTAAATSPPTGSGKANMDPALINFKVLYVLTQLCGLTMIVLVATWIGQHFGGLAGTSNP
GVEFNWHPLFMTIGFIYLYGNSILIYRGFRTTRKKTLKLTHAGIHMGAFILTVIALKTVFDSHNLANPPI
PNMYSLHSWLGLSAVIVFSLQYVAGFVAFLAPGLRENYRIAMMPLHIYFGLFGFVLAIASALMGITEKAI
FAIKTPAYSTLPPAGVLANVIGVMYVVFGALVVYLATEPSYKRKPIPEDTALLNSSSVNE

>TITIN_DROME
MQRQNPNPYQQQNQQHQQVQQFSSQEYSHSSQEQHQEQRISRTEQHVQRSQVTTQRQVQQHHGGSIGGAY
VPPSLTHVYAQGDISPPVFEQIFKNARFAQGGNALFEGRLRGNPKPFVTWTRKGAPLLESQKFRMSYNEA
TGDVSLLINQIGPGDEGEYTCTARNQYGEAICSVYIQPEGAPMPALQPIQNLEKNIYSNGYSYTSIEEEF
RVDTFEYRLLREVSFREAITRRSGYEQDSQLSQELDRNQGPAQAPQISQKPRSSKLIEGSDAVFTARVGS
NPKPRLTWFHNGQRLVASQKYEISYSSGVATLRVKNATARDGGHYTLLAENLQGCVVSSAVLAVEPAAET
AYEPKPVDVMAEQLEAGKALPPAFVKAFGDREITEGRMTRFDCRVTGNPYPEVFWLINGRQVRDDASHKI
LVNESGSHSLMITNVTRLDAGAVQCLARNKAGEVAIEAQLNVLEKEQVVAPQFVQRFSTMTVREGEPITM
SANAIGTPQPRITWQKDGVQISSTAERFVGIDGGATCLEIPRVTANDAGWYQCTAQNIAGSTANRARLYV
EVPREQPNYEQRRLNLPRPTKVIEPEPIPGPEIIYLRHVERAKPHLRPGEEDRVYPPPQFIIPLQNVQQT
EGGRVHMEARIEPVGDPTMVVEWYLNGRPLAASARATSVFKFGFIALDLLSIMGHDSGEYMCRVTNASGV

AESRAILSVVQRPSIEQSSQNPNSLQYINQLEDYSRYQRTESIDEQLNQAPQFIRPLRDLGEFEEGKNVH
FEAQVTPVNDPSMRVEWYKDGLPITASSRITAIFNFGYVSLNILHLRAEDAGTYTVRAVNRIGEAISQSS
IRVHSRSQVTADLGIPEQQRYIEKVEELEDYRKSQQRRHVQEAAEAIAPPQFKTPIQNQLDLREHAHAHF
EARLEPVGDSTMRVEWLKDGQPLEASSRITTYHNFGYVALTIKQLTIYDAGTYTCRAYNAMGQDTTVAQL
TVISKNEIVSESQHPGGLQKIQHLEDSSRYGRREEEETYITQAPRFLGPLKGTTKILEGQRAHFEARVEP
QSDLGLVIEWYHNGRSITAANRIQTYYDFGYVALDISQVRAEDAGVYLVVARNKLGEAQQQATMIVETRS
SIDTSSMHRGLYEKTQNLENKPFVEPQYDIEEISKSKPVFVTPLSDPKPIHDGKNIHLECRLEPMGDPTM
RVEWFHNGRPVTVGSRFRTYYDFGFVALDIIKATAADSGEYTVRATNHLGTAHTSACVRVIDHTDVVTET
QNEQSLEQIQLLEDSRRRHHQEEDITIMQAPQFTRGLHNIETIEGTNVHLECRLQPVGDPSMRIEWFVNG
KPVKTGHRFRPAYEFDYVALDLLGCYAIDSGVYTCQARNQLGEAVTSCSVRIIAKNDLILETQNESGLQK
IQYLEDSTRHRRSEFVDEVVNIRPRFLTHPKSLTNTREGGHAHFECKIEPVTDPNLKVEWFKNGRPITVG
HRFRPIHDFGYVALDIVHLIAEDSGVYTCRAVNLIGSDETQVELQCRSGEQIVTVTQNEAGLEQIHYLED
RSRYTRREEIDESTKQAPVFTTSLKNVEIKENQRAHFECRLIPVSDPSMRVEWYHNNLPLKSGSRFTETN
NFGFVALDIMSTLPEDAGTYTCRAYNAVGEAITSAVAVVHTKKSIYLESQHETALPRLQHLEDGSKRQRI
SVQDEFVSQAPVFTMPVRDVRVAENQAVHFEARLIPVGDPKLTVEWLRNGQPIEASNRTTTMHDFGYVAL
NMKYVNPEDSGTYTCRAVNELGQAVTSASLIVQSKTSIQLETQHEAAMHKIHQLEDHSRYQRREEEEYTV
TTAPVFVTKLIGPSNLVEGQSAHYECRIEPYPDPNLKVEWFHNGKPLSTGHRFRTTYDFGFAALDILTVY
AEDSGEYTCRVTNNLGEAINSIVLNVTSRSSIIHETQHEEALTKIQHLEDTSRFQRKTDEEQFHAERPQF
GRPLRNAKVNEGAPVHLEATLIPVNDPTMKVEWYCNGRPIQTGHRFKTTYDFGFVALDILYAHAEDTGTY
MCKAKNAIGEAVTTCAVNVTANKTLDLDTLDAQRLEKIRQLETYAPPPKPVVEEKGQKPIFLTPLSNLEH
LKEGEHAHLECRVEPINDPNLKIEWFCNGKQLPTGHRYRTTHDFGYVALDILYVYGEDTGTYICKATNQL
GEAVNTCNVRVLNRRSMILDTQHPDALEKIQKLESKVPNARTEVGDAPISPPHFTAELRGSTEIYEGQTA
HFEAQVAPVHDPNLRIEFYHNGKPLPSASRFHITFDFGYVSLDITHAVAEDAGEYSVRAVNALGQAVSST
NLRVIPRGTIISDTQHPEGLEKIRKLESTAPHQRQEPETPGTRQRPVFTQPLQNIDRINEHQTAHFEARL
IPVGDPNLKVEWYRNEKIIEDSSRITKQHDFGFVSLDISHIRKEDEGVYMCRAVNPLGEAVTTASMRVVS
EASIQMDTQHPDSISRIHQLEKPLAPRPTEPERLFEKPIFTQLLTGPSELWEGTHAHFEARVVPVGDPSL
KFEWFINGVELQMGSRLRTTHDFGFVTLDITAVVPEDAGVYMCRAYNAAGEAVSSTAMKVKTKSNIDGQP
LIPESWEAIRLKEAAMNRVPEMFVDSTPQQAPVFTTHLQSYDKLHEGQHVLLEAQVEPRADPNLRIEWFK
NGISLTTGSRIRSTFDFGLVTLSINGLRADDSAIYTCKATNQVGEAVSTSSLKIEDRHWLQAESLHPDSL
PRIGELEAPKEGRPEAPEPTYETPVFITHLNNIECKESDNVRFECNVEPARDPTMSIEWFYNGQPLQAAA
KFKSIYDFGYCALDLTNSYAENSGVYTCKATNSKGSATTSGTLKCTGGKTMFLDTQHPQGEAGLEAVQET
EEELANRYTSKTTKPETQYPPPVWTKPLQAEFHLSEAQPIHLEANVEPKEDPNLFIEWYFNGKMLNHGSR
FKMTSEFGFVTMDMIEVYARDQGIYTCKAYNKAGEAFTSTTIFCSSKENIIESTQHPKGAEGLEQIQDLE
DSLRKDGSKPEQPDLGIPPRFTTEFVNIADIGEGELAHFEANLIPVGDQSMVIEWFYNGKVLEASHRVRT
IYAFGTVALEVLGTKIEDTGTYTCRATNKHGTAEISCNLECVDKPRGQKPRFTSHIQPLEGLKDGQSAHF
ECTLIPVNDPDLKVEWYHNGKLMRHSNRIKTVSDFGYVVLDISYLQDHDSGEYVCRAWNKYGEDFTRTTL
NCGGRGGVFYDSLQPDSLQRIRELECPQGQQADTSAPLVAEPPKFITQIVDVTKLVEGQSAHFEARLTPI
TDPDLVVEWYFNGKKLPHGHRFRTFHDFGIVILDILYCYEENSGVYEARARNKYGEDVTRASLKCASKSS
LILDSQLPRGMEGGLEKIANLEYSMVRTREETTEETKGKAPVFTVPLENIENLREGENAHFEARITPADD
PKLKVEWYWNGRPLKAGSRFRTFCDFGFVILEISPVYPEDSGEYSCRAINEYGEAVTTATMKIQGKRSII
MESQLPKGMEGTIDRIAELEGLGSRSTEFVPDDDTGKPPEFITSPFDMVIGENALAHFECRLQPINDPSM
RVDWFHNGKALWAGSRIKTINDFGFVILEIAGCYQRDSGLYTCKATNKHGEATVSCKLQVKGRQGIVMEP
QLPSNFRTGTESLQKLEETMHKREELVTEDEQPNPPKFTEEIKDNLDVPEGGPIHFDCRVEPVGDPTMRI
EWFYNGHVMATGSRVHQLNDFGFIALDVDYIYARDSGEYTCRATNKWGTATTSAKVTCKGKHNIVYESQL

PEGMTSEKLKELERGRIPEAPKVVEEVFGPPKFTTQITSVTVDEAEAVRFECQVEPKTDPSLRVEWYRNG
KPLPSGHRYRNIFDMGFVSLDILYVYGEDSGEYVCRAINNYGEDRTRATVSCKKLPTILLQNQVPRGMKR
SDALTQMEATIKKYTSEVHLTEDDLFDPDRKQPPRFVTQIKEQLTLTEMAVTKFECQLAPVGDPNMKVEW
FFNGKPLLHKNRFQPIYDFGYVAMNFGWVYPEDSGEYVCRATNLYGKDETRAIIKVSGKPGIVYDSQLPA
HMQSIDRIREMEASWQVVPDEVDPDAKPRTKPVFVSKLEPQTVEEGDPARFCVRVTGHPRPRVMWLINGH
TVVHGSRYKLTNDGMFHLDVPKTRQYDTGKVEVIARNSVGESIATTELKVVARSDDYRNVLKNSPRPWYD
YELAAYQKERQENELEKVFDERKQVLSEQSSHTLKGVEHLKPKQYKPPTPDWQQNVKAKKSEDYYNKLQT
LETEQLLKETNLRRDTHQYAIPGEKVVSSSQAKGMAQSYEENLQEKTSTTEVQAAPPKGIAQPSESSVHG
REVHMNKQQQVQKEIQGDLEITRKITATETTEVEHKGTIQERVVQGPVKPAKAPVFTKKIQPCRVFENEQ
AKFEVEFEGEPNPTVKWYRESFPIQNSPDLQIHTFSGKSILIIRQVFVEDSAVFSCVAENRGGTAKCSAN
LVVEERRRAGKGGIQPPSFVTTIQSTTVATGQLARFDAKVTGTRPLDVYWLKNGMKIQPSIKFKMLEEDS
VHTLLIIEPFAEDSGRYECVAVNAAGEARCDGDCIVQSPSKPEKPTTPGSEKAPHIVEQLKSQTVEEGSK
VIFRCRVDGKPTPTARWMRGENFVKPSRYFQMSRQGEYYQLVISEAFPEDEGTYKCVAENKLGSIQTSAQ
LKVRPIENLDAPPTITALKDVSVTEGMPAQFKTTVTGKVKATSVQWFREGQLIPETPDFQMIFDGNSAVL
LIGTTYEEDSGIFTVRVTSSTGQVESSAKLTVKKRRISAFQLRTIDSAEDESSSSGREDSAPESPHAFQP
GQQPGQQFGQFLGVNGQGQHQGRSRQKKPKVRSKSLQPATKVIPWRKSSRPTRGRSLDKGVFLPGFKPEP
VKSWTEETINLKATPIEKKKPAPKLEAAKVVLKSIKTERDQGIMSLGATLEQIIAGKTEKEAIPWITMRE
KLKAVESVQQQLNKFDLDEVYLQPLEGQIETEGQLPQQAQVEQVQRTKEIQRLKSMESVEIMEMTDQIDK
LITQQQNAKDLIPWKEMRQQLKSVQRVTKQIDKFKIEEVELRHLQAQQAITEEYQTGTAEETVVMIDESS
KGSISKVLRRDEQLQYEDQSNIYKQKFITTEDVNIMHVSEREKLEAQRLIREQQAVNWRQQQQRPQLQPL
TSVEDTVISQTSERQKLVQQQSFIEEAQRQQFVQVEDSQMMSLEEYEHQKIINQRTQQEAFSWRQPREPQ
KFIQVEDSTLLHLQERHDTQEQQLLQQQPVMWDRGRKKPDQPQYVQPQEQRVKEEFVEKPKTYEEMHDEL
VEPTPIEQPQPVPVMWERGKKKPQPQEKTFEEAHDELVEPTPVQQPEPVPVMWERGKKKVAQQETVLSQE
VVQTSQVVEQQIVEETKKTAVRRVIPPREPEQKVEQVTLKPTPRPRPKEAVKAEEIQLKPLRSTRPVPQP
VEAEQKAYEEATDELTEEPIPQPQPVMWERGKKKPQKPQEEVTEIPKTLEIAVDTLEEEVPKPTEPQPQP
VLWARGQKKPQKPDEQKQELPKSLEIAVDTIEEDLIKPVQPEPQPVLWERKKKKPQPQDVIEEKLDVAPT
KTYEKAVDVLPDEPKVEEKPEPVLWQRGKKKIPKSEPTEEVHPDEVDAQIETVVKEDEMIVEEKRRIKKT
KRPKSTKEVTEELFEEQPEEEISPEEEVPQKEVIEEIEEIVEEKRRLKKTKKPKLTQQVTEEETPHEEII
KESEEVVQEQEEIVEEKKKVKKVKKPKTVAEKQLKEEEIPTEETVEEEETAEDQQLVVEESKKVKKVKKP
TGTVEKTDVEELPGEEVPVEEVPVEEVPEDVAPEEELIEEQEEIVDQDEIQEQKRKVKKAKKPKKTIEKT
EIEIEEDQPEEEVLQEEIIGEQEEITERQRKVKSIKKPKKVVTEKTVDQTEQPEKPEESQAEEVKETVTE
EPKKPKPAPEEAKVEQVEKISLKPAPRKQRLLPEKEQVEEVLLKPVKKIVAVSEAEQPETPETEFEVKEF
AITTTEDILDVTKKRVKKKKPKTKVAAEESTEEPAEETEEFEEEATQPEEVQPVEEIPEEPQVKEVADER
KTAPKPKPRKEEIIEKVEEVALKRVTRPKKELPQEATIEEVRLKPTQRTSIKPEEVKLEEVDLQHVEKKE
DEIVQEEKRKTRKVKKPKHEDLPEIPDAEPTQLEEAEHIELEKQPKPEEDQPQVPWKRGEKKQPVEEVLE
EKKWPSGKRRPLPEQQPEEVQLKPIPSKPIEEQQKPEKAIPGPQLVPEEKPESEEEELELEPLKLPEDKK
PKEPKAKKEKKKKPKLKKATPSVDEVSEEVAEPFDEPIAEEDEVEEMPVDDVKVVAVSEDVLPEEEVVPT
EETPEAKQKAHKKRTKRLKEASVEGQPQLLEAAIAEIEKVDEISQEISQKTITLLKKTEDTRPQFITTEQ
LIELDVEDVRRDLEMKVTSNIIKKEKRRVVLDDSQPLPELELITQKRIQEGIDKVADEELIEDQQLIQNQ
QETTTSEVIGQERKLVKKKKKEIKPPRITEKLRPRQCVPEEPTVLECKVEGVPFPEIKWYFNDILLFASE
KYEITVMEQVAKLKIAKVTPSDVGVYTCEAKNEAGVATSRTNIILEKEQGVPPQFTKPLKIEFIEEKQPE
RLKVTVTCQVTGKPNPEVKWYRGIEEVIPSETVQMFYDEKTGDVALEVINPTPNEAVVYSVQAQNQFGRA
IGNANILSRVDEVPREILKAPTVTPLSAVVVPTGGTLFFEAKYDGLPRPEVKWMRNGREIIENEETIIET
TETTTTIKVVNMTRKRTGKYEVWAKNKVGEAKSSGSVVVSDQKPDEQIKPPRFIQPLEPKYFGEHEVAII

EAIVESEPLSSFQWFVHNEPIKSSNEVRIVSQANKSTLLIENFQSKFVGPFTCRAENVGGSVTSTATVNL
IPQEEAEEFESPRFVEELVQPVEVMDGEALLLTCQVTGKPTPKVEWYHNAEKITENKETTISQDLQGVCQ
LQITEVFPENEGQYECVATNKIGKSVSKTNVKIQAFEYIPDSEITGLTGSEEDLLDRTLSIDEQAPKIIK
KLPEKIEPKEGEQAKLEVKVVGKPKPKVKWLRDDEQIFASEEYQIENFEDGTSVLVINHVYPDDLGTISF
EAYNPLGVAVTTALFAVEGIVGSKDYRKPEWVSQMEEMQVALKAAKCSPSLLNEMRDCRAALGETAKFSI
QFAGNPIPDIQWYFNNVQLRASEKYRMVVQEQEATLEIMKITSEDCGYYNCKLINEIGMTMTRAKFDISS
TSTIVEETKAKTTVKKKSGKKTMVKRSGASESQNVQKTEIRIIPTSAVETSMNVIKVKQPVSVLVEKSEI
SEVLVVKDREVADAEERSSQLIEEIEEEEEIEEKVQHDEEDEVEVQVEQKETYTSSKKIEITKTVELIRT
KISEKIITIEDVQVLSHHEEVQWLLESIEAESFGQIGESALRDLATIGLLLRYGCEHYEITYMYEQNIFI
SLKKPESQSALVQLVEREGHEELISQILSESSNEDETILAATVGFKAFIRMIQTYEITIEIVIRKFVRED
FISQDWKICGKERIVETSQIIESHEAITHVKIETATTKVEKLFKKQEQEHVQNLEQQEQVKIQVQTKQIA
QMNTKIKKHKKHKQQEQEVSETTIQCEQKETLAHETSAELPQSETLEQIEESLSTYETLPIQNLSKDTLQ
TVAVSVTTELSTPSPTASRVQEEILPQKVLAINEEVLPLDEFGLRKESPRPKENKLTENIEVRLKHALNV
SHAKTAESSKELPSKIPKSVKAQRKMKESRSLVVEAPNAEEAIEDLKPLKAVSQEVQSDILFSHEITEEQ
HQALETIEKLKPTSAIEDTVQQKLLSQEELIIAEVLPSETVGRDVTDVRPPGETISPRLTPNMSLCITEC
QPEDSIGEMQQAAKERMETPSMSVTESKAVGGQELEVLENVDHMPLITQPTKGLADYTIKAEEVPVQVQE
IITFDSLERETVAKTQTAKSNALELFELSEGLVSSTADSHSPIAEDLPIFEKDVKEATIDMQMQHHVTTS
ETVSNENAVKDLKAVDTPKMAEGTLGQSSALTIGETQQMNLVETTVELIEPNVESTKPAKGALTEAYGTA
ESNEETLLESLGLVPDDNRKIEQGKVNISEGEYVAKVQTTTVTDTEGEFVSVAPKLVNPKFDFVEQSALQ
IKQDTTVEKEEILSSNIELAPQLATSNMFPAELKVTSIYEVQPGLTSSDIITEQTKSVSANQVFETMSIG
VTSKPDMLESTSHIDAFQHPEFKTGDTILDENQQPLEVTNVQITESSTDIIDVLPNQKLTKAETVTDGFK
YAEGLVVLPMESTIDKTEDTKPTAVNADISMHQQFGTDVREQEPLESTLTRTEDLKPQTQTTESQFGLLQ
SLETSSCVTLEGESVLSVKERHPEQSAAIGTSSALQVANITRPQHMESLDRLDEQKVPYYQANVNIGEIT
LPNVEKIDSFDVLSDLNTPDYNKSSKGRVQLIESTTSLKTTTAVVSESTEELKDLNITQPVHIKPKPYES
DQKISISEQTNVLEHVSSLNPVLPALETIQSSIKSLHEINVRETDILEKEESLKDVDHISGRLAKIILDC
TTGIAQVRQEETLEHEEDLKAPLIPLEKAIPASSELHRLPLTEYVQEQQGTSDMTDFKVSNKCASPNIDH
LYETKSSEMIVYDSSINSVDSEFPAGIVPQKSLVPFRHTMVTENVAFNASENFEILSADQQIATNVQDSL
SQSIIAEDQIAFETEQNLGLETTPTHKPKLLKDDQNLHAKLVDEATVYEAMGQEQKVDKYNIQQAEITHD
LPQVYATDLQQTFEAEKEITTREQSYVAATTDIIPSRLGLAMTTKTHPVEGIDVLLSSPPKPSLAQTNYE
ETQHEVRVRETQAIEESEELTDGRLLPVSAVESIDSTFKVTSDSQQPPVFDKELSIPTVSPLEARAKPSL
NLLQGTTTFDVIPLESSVLLKDTHVAVQKAQQEYVAQVESNKVHVQMDNLVMHKEDIFENAEIENFCKPI
TEGTQLETVVIEVVPIDNVGGIHLAPQPSTLLATLTSTDIVNQSHVIDTQVPLEMESEAQAPLDNIAQAR
IKSAEDHVHTNVSEVNVYEQTKAIQDQNKHGLFVKVSKNSDTSKAYLTTIQSTFLKEDILPKPNILQDTA
QAAADELQSLVTEEVVSVSSIQETYELKIPLQKTANLTQQTPQNSVNVCQQLAYEETPDIAFEPHALTRA
TTSSVPTFLKPAENATVNIYENIEGHGDFKPGTVNLTSNSNLNSELVVSVVQEVTSVPSLGSLATVEPQE
LKAMPVTKSSTNLAYSEEVKGNKQEFTKIETVEEDDKQPETTVTVEELPYEEEKPEEIQELPEEVCVVET
VTEDGKPKKKKIRTRVIKKVKGDKQEVTKIETVEEDDKQPETTVTVEEVPYEEEKPEEIQELPEEVRVVE
TVTEDGKPKKKKIRTRVIKKVKGDKQEVTKIETVEEDDKQPETTVTVEEVPYEEEKPEEIQELPEEVRVV
ETVTEDGKPKKKKIRTRFIKKVKGDKQEVTKIETVEEDDKQPETTVTVEEVPYEEEKPEEIQELPEEVRV
VETVTEDGKPKKKKIRTRVIKKVKGDKQEVTKIETVEEDDKQPETTVTVEEVPYEEEKPEEIQELPEEVR
VVETVTEDGKPKKKKIRTRVIKKVKGDKQEVTKIETVEEDDKQPETTVTVEEVPYEEEKPEEIQELPEEV
RVVETVTEDGKPKKKKIRTRVIKKVKGDKQEVTKIETVEEDDKQPETTVTVEEVPYEEEKPEEIQELPEE
VRVVETVTEDGKPKKKKIRTRVIKKVKGDKQEVTKIETAEEDDKQPETTVTVEEVPYEEEKPEEIQELPE
EVRVVETVTEDGKPKKKKIRTRVIKKVKGDKQEVTKIETVEEDDKQPETTVTVEEVPYEEEKPEEIQELP

EEVRVVETVTEDGKPKKKKIRTRVIKKVKGDKQEVTKIETVEEDDKQPETTVTVEEVPYEEEKPEEIQEL
PEEVRVVETVTEDGKPKKKKIRTRVIKKVKGDKQEVTKIETVEEDDKQPETTVTVEEVPYEEEKPEEIQE
LPEEVRVVETVTEDGKPKKKKIRTRVIKKVKGDKQEVTKIETVEENDKQPETTVTVEEVPYEEEKPEEIQ
ELPEEVRVVETVTEDGKPKKKKIRTRVIKKVKGDKQEVTKIETVEEDDKQPKTTVTVEEVPYEEEKPEEI
QELPEEVRVVETVTEDGKPKKKKIRTRVIKKVKGDNQEVTKIETVEEDDKQPETTVTVEEVPYEEEKPEE
IQELPEEVRVVETVTEDGKPKKKKIRTRVIKKVKGDKQEVTKIETVEEDDKQPETTVTVEEVPYEEEKPE
EIQELPEEVRVVETVTEDGKPKKKKIRTRVIKKVKGDMQEVTKIETVEEDDKQPETTVTVEEVPYEEEKP
EEIQELPEEVRVVETVTEDGKPKKKKIRTRVIKKVKGDKQEVTKIETVEEDDKQPETTVTVEEVPYEEEK
PEEIQELPEEVRVVETVTEDGKPKKKKIRTRVIKKVKGDKQEVTKIETVEEDDKQPETTVTVEEVPYEEE
KPEEIQELPEEVRVVETVTEDGKPKKKKIRTRVIKKVKGDKQEVTKIETVEEDDKQPETTVTVEEVPYEE
EKPEEIQELPEEVRVVETVTEDGKPKKKKIRTRVIKKVKGDKQEVTKIETVEEDDKQPETTVTVEEVPYE
EEKPEEIQELPEEVRVVETVTEDGKPKKKKIRTRVIKKVKGDKQEVTKIETVEEDDKQPETTVTVEEVPY
EEEKPEEIQELPEEVRVVETVTEDGKPKKKKIRTRVIKKVKGDKQEVTKIETVEEDDKQPETTVTVEEVP
YEEEKPEEIQELPEEVRVVETVTEDGKPKKKKIRTRVIKKVKGDKQEVTKIETVEEDDKQPETTVTVEEV
PYEEEKPEEIQELPEEVRVVETVTEDGKPKKKKIRTRVIKKVKGDKQEVTKIETVEEDDKQPETTVTVEE
VPYEVEKPDEIQELPEEVRVVETVTEDGKPKKKKIRTRVIKKVKGDKQEVTKIETVEEDDKQPETTVTVE
EVPYEEEKPEEIQELPEEVRVVETVTEDGKPKKKKIRTRVIKKVKGDKQEVTKIETVEEDDKQPETTVTV
EEVPYEEEKPEEIQELPEEVRVVETVTEDGKPKKKKIRTRVIKKVKGDKQEVTKIETVEEDDKQPETTVT
VEEVPYEEEKPEEIQELPEEVRVVETVTEDGKPKKKKIRTRVIKKVKGDKQEVTKIETVEEDDKQPETTV
TVEEVPYEEEKPEEIQELPEEVRVVETVTEDGKPKKKKIRTRVIKKVKGDKQEVTKIETVEEDDKQPETT
VTVEEVPYEEEKLEEIQELPEEVRVVETVTEDGKPKKKKIRTRVIKKVKGDKQEVTTIETVEEDDKKAET
TVTVEETELSAPSVGKVQLKKRVIVQKPEDAVTVFELPERKSVILSEKEDGTPTKTVIKTRIIKKIQGPN
MEVTKVQTVEEYEKAPQTIVSVEKFNTPFPELPEERLSEVVMLPDEVFESEAVDEEGRLKMIKTKKRIIR
KPALDNTEEVTEIGIIEQDNVEPIYSVKIQERPLTESKPEDSKLIELPEHVTELNVILPDGKKKRRTVKS
RAFKKSLDDDLDEVTTIHIIEEEDKEPLTKVNIEVVPSDEISITPIPIEELPEETVFTEELDENKKPKKK
TTKTRTFKKRGPDDDEYFQIQTIDEEGKEPISLIRVVSDENIADIIDISKLDDEKVLKHKQKPHKHKDQY
YKEYTITEPEEASADALQKPTKDKTPKQKKTLETPIEEVDETVIIDEGTGEQTDQIAIKRKPRKVQGNVQ
VEAVDEKPIEKKEKAKKKKVVKTKRDEMDDYIQFLIHQEIPKTVLQPYQRTEMELPQRARRDSSFKQPVK
LTPMKIEKVEFKKPKMVEISSVVEFPQMLKLKAPKQRPQEEKKKKNEASFKNKKLKSWIRFVPYAPYCFP
YVVTELETNREVGELSRNVDEAEEVLKLRPKKFKHSKPEKAELEEADLGAYESDHSDKSNKELLHPKYKR
GKKEKIETPDESRKLKFGKGKVPQNEEASEEVNLKPVKLDIAEIEDAEMPVRTQEEEVVKKKKPKKSSKP
EEGLQFEPIEFEEMERTSDIREESDTSVSVDTSTQEKPIYKKKKKVTPSPQKNQYKILPGQPREFEETPE
DDLNLRKRQGERPDDDKADTKLKPFYKFEVLDLEPEGVQAETVPLSETIAKEPKKKRKIKVKTEQEDNTI
EIVPLSPEDNDEQIFEITVTSSEIPQGDAKAKTIGKKKVKRMNKQELDDFVTELQEEPNQEVYETRMSDF
YEVKLTELPSEMDSDKPTKRILRHEKGDEVQVLEIVESVVAPGEEPFYEINVISSANTEGDSEEITTDKI
KKKSRKIKKDDLDAYIQQLINAEIPVTELEKYEKIDVDGKAKKPKKLKAKTKKPIIDEGETLQVGVTEHE
PTKKLKTKKPEEKKNVIEKELAEHEAVPEYDEFLINKTESERPQEKRVEETEKDIVPIVDKVLADLNDCL
PFVVVEEDLKDMPLATDVIALEDEKIIRKRKVRAKKDSKQYEIEIIETEKPGDIPDEARVIVITTEVSGD
TIDGPAPSTTEAPKKSVRKVKKEKLKEFIVNIVEEAPLDHVSEIYEDVLRTPFRESSEKEDIPSFTTTVV
EDEIVNPVLPEKIKTVDDVRVPKDKKKKIDNQKKIKISEFEPTPTSEDSTIEEYTPKLSEHDEDLQTDEY
SVDVKDSLPKSKKKSTKKQKKESLPGPISLYTIRIEETTPEPITEKIYEDGKEVVRVINKRRIKKKAGPK
EYLIEVIETYEDNNPEADVVIRTIETTPSIDSKPQEDHKIQVVQEKKPKTESLDNYIQKLIDQEIPQVDH
KEFKATVLETSPESKKAKKIKKHHKKTTEVIDGIPITVIEVTIQETETDDEDFKPDEVTLKEIDHENAEE
APKVLKSKVSEEKPKSKKEKSLEFKIAEEDKPKPVLEDISEDVQVVQIIEEDGTPKQVEIKKKKVSRKHG

PKEQVFEITETKAIDEPLSEVTVVEITDEQPQEEVLPAQEKKPIKKQKKLKPEDVNTYVVKVLEELTEPT
QFETIPEDADDKPQPVIEDISENVQVVQIIEEDGTPKQVEIKKKKVSPKHGPKEQVFEITETRPSDEPLA
EVTIVELTEEGLNKDIVIPQEKKTVKKPKKLKPEDIQSYVIRVLEEFNEPQWPASTEKPIIEDIAESIEI
VPVTEEDGITKEVEVKKKKVSRKQGTKNQVFEIIETKTSDEPLAEVTILELSGDKSQEVTILPKEKKPIK
KTIKLKPEDVESYVVNVLEEFCEPQSFESPEPTEGEAHETKTKTKKPKKPIVKAPENVILIEEMAPETVI
ENIVNEIGEEVKQVKTTKKLKKKEGPKEYLIEIKETYEENKPEGDIEITTTELVPEGSPDASDDQPVIVV
QKIKKKKPVKDDLDKYIQQLIEQEITKTPLEEYEPTEMDSKKKPKKKVKSHNKKTIEVIDGLPVTIHEFN
VEDIVSEPEDMETPKTLLDEIKEIPQLPDDSSKYLVNISDEFGEADKPIKQPTQDQPIKKEKPLKKKKDV
EYPVSLEAFDHTVKVVSEPTLEGTVKEVTVKKRKVSRRKGSKDHIFEITETTSEDRPTAEVTVVELSSDE
VLDSEEKPKHERKIVKKPKQLKKDDVEEYIINIIEEFIQPIPVGLVEDEVEKVQKEETKKPKKSPITYIA
TEQEDNDNNYDALVKEDLDQPIERALEKPSSPLEYTISVEEDSVGEEQKQPKPKKISKPKSIKQPSVDKS
PDYLVNVISEESIIDEPIPEDYVVTEAAEEKPSEEPTFKVEELETEAVEKEVTDDDKGETTKQSVTKRKI
KKLVGPKEEIIEIVETKTGDTPEYEVIVTTEEVQEKSKEAPEEKKAKTVRKAKKIPKDDLQDYIQKLIEQ
DIPKTELEKYEKIDLDEPVKMKRKPIKKVKQSEEQPKEETEEPIEDKPVEKISEYSEVDSDEPKLTVAVK
EFIPEKPEEKPFEIVVLEETVESKREPDEEGKVREKVVKTKKIKQNRGSVEVVHDIVEEIDTDTNESVIT
VTTTVPTETPDQDQPSVKQKRTKKIKKDEVEDFVKRVIEEEAPQPEGSVDLVVIEDFVPKPSSEKRKKKP
IKDKHTSVEEETPHEDEVLLIESVPEDSPLSDDLITVVDSVPIEEEPENKVNQIEDTKKPEKKKKPKPSA
KILEENVPEDTVEKPLEALHTDSDLEKPDVQEFSISIKEEEQKHTHPEKKKSSKISSEQPKQPSTEQYEI
SVTEHDLKPEEEKPFTVQVIQSETNVEETKDDTGKVHKQVTTKRMLRRPAGEGEIEIIEVVRDDQPEAEI
TIVEYEPEPVNQDEKPKEPKKKTRKVKKDDIHDYIQKLIELETPKTELEKYEKIEFEPIVKDKPLDSPID
VLDESPKEVQKKDKKSRSTKVPNEETPVQEQYAKVNVVEEEAPEQPEIPVQILEVKPVEVDVKEVITEDG
KPVQEKTTKRVLKKIGPEEQTTFKITMIESEDNDSVTVIVDEEPEIASPQSIEEHPEQSKEKLAPKPKKT
VRKVKKDDLSDYVKKLIEEEIPKVDLEKYEKVEMPEKPVKLTVSDSIPEEPKPDKSQPISVLPDTTKPKK
TKTPKTPKTEDTDQQVPDEPTETTVDTTDIPELTPTQTAQPEDTATAQITPSAQEEKSTQDDTKDTIQKT
VKHKKTKPDTQKSVETSELPEVHKDYQISIIHEELVEEEQPEKILEVRVIDEVAEVEESQPIVEEVEDEE
PQPATEETVEDVTKPKSKKKKVVKKKTDDHDELIKKMLEQEIEKTELEKYEKIEFDVPKKLKPEFAALEP
IKIERKEQKPTKVTILDATDVPKTVKLKPSKRKEKPAEELTVQLPKFRLKARMVLVEYPPAPLIPKTTDI
GAIKDNGELSRNIEEAEEILKFKPHKTKKIKKIKDDLEKVELEKYEKYISSEEEPEEKTPYKKPEKAPKP
EEKQEDVKLKLGKGKKKPKEEEAPENVTLKNIPQKPQEVEEEVELKQKPKEVEIVEEQTKKPKDGEFVVE
PFEPSEFDRPEYVPDELEQIEHPEIPEKVKKPSKTKYKPKDKSKSEPETIVSEIVAGVPKEEEAIPEQDV
KFRKPERDAPEETDSEIKLRPVPQASKDENPDEQALVTPKAEEPIPQEIEDKAIDDEKKPKKSKPKKVQP
KEQEIAKEEPEEFEVSVKEEEALVDKPIEIEKPKDVKVKEKKPKEAPVSEVVVIEEEPKPEEVPEEIPVE
YKITTTVLEPEDAPKEHQVKVIDFDERQETTEEVIEEKVVTRKKKPKPQQPEEFEVTLKEPKEEQIQPDV
VSAEISLPIEEPEQKPEQYEVELKITQTTPEEPNDVQIAVKEKVKTKPVKKVKEDKIVVVEAEEEKQPVE
ETIVEVEKQEEKKKSEKPKSYEFKISETQSIEEKPIEVAEEAPEETPKVVEKKVAEKFDSYEFTLKETDE
EKVITVDDQPEEEAPVEVVFKKKPKEPEAVEAEFVMTEPKIVEETSVETAIKQKKTKKPKKDEEEAQLAI
KVVESEAPVAEEVFSEAPESKIVEEEVIAEEKPKEFTIRVSESEPKPEEPSVEQFTVKKRKPSVTFADEP
ATEIVIKESKPAEVVTEDAHIKTKKPKKKVTDVEAEELKIKITEEVPQEIPILEEVSEEEVITETKKTAP
VVEEKTYKIGIKETEPEKPAEAIVEEEEPVVTEPIEEAPKPEVFEEHKVRVIEETPRELVEEVIEEEVKV
IRRKKPKPEIKEEPEAEVTVSTPKPVEEVEATSSIAVIPEQPTEEEAADLKITIIEEETPPQELVQEIEE
IEIVEEPKAPEEQPTDFTFATKDSEKKPTVEELPEEQVTIQKKKKKAPVPEVVEEPEAEFLVKPKTPVQE
VTEEAKITKSKKPVKEEEAAAELKVTITEEIPTEPEVQEIIEEIEEIEEEKPAEYVIEVKESQPEAVEDK
EVSLPKKKPKAPIVEEPEAEITLKPKVKSEEVQEEAKIVKKKPKKIDEVAVADELTVKVEEEVVPEPIVE
EEVIEEFEIKKKPKEPEPEDIVDAAIVKLKKPEPVDADEVVAEVTLKPKAKTEVTEEEFSVDVKLPKEKK

ERPVEIEEEEIIEEAVVIRKKPKKPFEPTVEDLEETEFSLSFKKPHTINEGVEEAATVLKKRPVKPTTLD
EAAAELSIKRQEEEYEEGEDIEEFVVSQQRKPKPLQITEEDEEAYTVKKLKRRKQVDIPEYADVENVTFR
ARSTKTKEDVDQEFNIALDSYAEEEISMSGKVKLKKPIKKTFSEAADEAKIKIIQDFDDGEEPIIEEIRD
DEDTIDEVEEPEEYFVEELPPDEVDFKLKPKKHPKPAYSVQDEEEEQFLIGIRHPKRDSVTYDEDSLTFK
KKRKVVQQLFNEDGASLNITREMNVEESENLNIMYSICNYIADNNEAINLVEGEKVTVVGRHSSEWWYVK
KSTTEEEGWVPAQYLMEPEEYAQYVQNKLHEKIDKLPVFERPGPEDKPIAPRFIEKLQPIHTPDGYTVQF
ECKVEGNPRPQIAWFRETAIIKPSQDFQMFYDDDNVATLIIREVFPEDAGQFTVVAKNAAGFTSSTTELI
VESPLSDHGSDATALSRRSMSRESSLADILEGIPPTFSKKPKAQYVDENTNVILECRLVAVPEPDIVWTF
NGEDIDEEEIKNVRIVTESDMHMYCSVVHISKVKKSQEGTYEVIATNREGEARLPITLKVRTTDKEAPQI
LEPLRNMVIREGESVVLSTQIVGNPPPKVTWYKDGKPVKNAKSDKDLHTLTLITPQKSEKGEYTVKAVNP
LGSVETTANLTIEEPAGGNAEPPLFVERFEEQNVPQKGEIRLPAKVSGNPVPEVQWLFNNTPLFPSERIQ
QVYDGENIELIIKDANPETDSGDYKCIASNPIGKTSHGARVIVEVDEVTFTKKLKKTITIEEVQSLTLEC
ETSHVVTTKWFFNGKELSGMDHRVVVEDGKTHKLVIRNTNLRDSGTYTCKVKKQETQSTVEVLQRKPDFI
KVLEDYEVTEKDTAILDVELTTEATEVTWYKDGEKITPENKNVEFIKDGKARRLVIRDVTIHDEGQYTCK
IEGQECSCELVVIELPPEIVEPLNDVAVTKGENAVFEVELSKGDALVKWFKNGKEIVFNERIQLAIDGKK
QSLRIVKAKPEDVGEYSVQVGEQTSKAKLTVEEPLVDFVIRLPDITLATKTTDAEFTVQLSQPDVEVTWC
KKGKPIKPNQKHEVFVEGTVRRLVIHDASDEDAGEISCVAENVTSSTKLCVEELKLPPVITSDKDQTIKV
KENDDVTFTVKYTGVPTPEACWTTRKVVIPKSKRTIPTIDEQSAKLTIKKVVDDDEGEYTVKLVNPVGEA
EASLHLVIMRKPTAPGTPQPLEIMHDSITLYWKAPEDDGKSEIIEYILEYQDVKEEKWTEIRKIKDTTYT
ISKLKIDTEYVFRSIAVNEVGPSPPSPLSPPIRLVPKVETKAPSVQEPLQDVVSELDKEVTLSCVFGGIP
EPKVTWKKNGQVFESRSIRYENRVAKYTIEKTTIETEATYTCVATNEKGSAETSCRLKLQQKPVLEVEDK
YLTQKLRTGSILTIPATVRGYPQPTVTWHKETIEQKTTKSVTIETTETTSTYTVKKVTREQSGKYKVTAT
NESGTTYVECTVQVIDKPSRPQSLEIKDIKKDSIVLEWTPPVDDGGLDIEKYTLEKCDVQNNVWMKVSDF
NKDIKSYAVQKLSMNAQYMFRVVAANPIGESEPTESDPVTITKKFEKPSPPRGPTTVSGMNDTSFNLAWE
PSETDGGSKIIEYIVEIREETETTYRSVGVTLGTVTNIHVEKVVRNKGYFFRIYARNEVGTSEAFETTEK
IVLGRKITPPSPPQNLRAPDVTSRSVTLDWEVPARNGGSEITGYCVEKRSSTSTNWTKVITLDAHQLHYT
IDNLKEKCEYWFRVSAENEVGLGAPAVTESISLKTHASEFILVVRNCGSYTEFGFLKAVPSPPTGPLEAR
VLAANAHIFEWGLPESDGGAPLLGYHIAIRDMKKTMWIEVGRVPAGVLKFQIRDLQENHEYMIRIFAKNE
IGLSEPLESEEPYKAMTAGHESLPDEPRTEMSSCNTSSWLRDHHMDADIHSYARGRLLQRDEYFFRLWAE
LPKSKKKKSSK

>E4YII7_OIKDI
MKFLLLFLIKNCSSRVLTVFCSNYSDWNCEDIATIFRGFPKYELIQEQKRCDNVEILSTGIRRFNKILPP
RTVLNETFEYKEEMSFAIGFSCPKGSETLLAQSAHWSFDNRNRPITVADERTGLPVVTVGHRLDNSYSNV
ISLGPSYKYIAKSMWSMKTYFNWNRIIMIILPLKPDNSPPYSSTESYDTGRRMLYIENYFNEQLEGTAKH
IISALVKGLTTFSPGSYSSEEEWSVLQSLEDIIESFDVIFAPPNVFRDIVDKEHQKFEDSTMNWFIKELL
KKTWILLNEYNLEDQLAPQGFLSYFDVYQEMIKKLKLLPQPLEKYGYLSKQKDSRTIGIETPKSPILRGY
IAAVDFYRICLDYYDKQIPLNGSKVIREMTEYIKDPISGRPTFTTRFSEQIELDKNNYQIGHFSLSYFDR
NLKSWKQLIRVDHGDQLYRVCNTDVCKIYLQRLNHTDPTYQPENENEFFTTEQIILLALGGLVGLVGVIF
MITKGSGLFMDFLYDRQLIKLEWEIPASRILMIRDANEMASGNSFRRSTSRSTTRRGSRFNLIEGVKIPS
RRFCELGVYNLKIEKSGQRKRILVGLLPLESKLLASRDSLQSIRRVIQIEGPNIAKFEGIFNSRSKSYRV
IHYCNRGSLNHFLSSNYYANLELTLEVKLVLAIDILCGLAHLESVLGFHGAFSSKSCLLDACFRVKISNF
DHLRPSKKTQRDIYKAPEVLRFDVSQKDTPNVLRRCDVYSFAIVFQEILYRRGPFYIGDESYSEEQKIEQ
VRAVQSDIFRPTLEKPADLSNEQWLTLDEIALIEACWSEDAYKRPAWFRDILPHFQFSHSSRFGQKSKSI

IDSFVGLLETYAENTERLVREKTKILERF

>E4YIJ5_OIKDI
MRFLLALLVSSSFALEKCVEPMENGEWDIITISRAKGGIGLTALGATHVKFNGETKKTPLRITHGNSRQE
LKHNRPGLMRSFWEALFSDPQTAISLEIDPTYNHFFSVQKMKTMFKTNEKLCYKAGFSLFSFWRVAPLVV
GLAIFFKARSVTDQVAFHYAGGISAGVVLFAIGLLFIVTKMMIPKRGMISFWGVFLGGSTFISYFMSKFY
AQAYELIEMYPLAVMIYVVVAASLSFTACYYYEDSVLRHPKWQNFVCWALQLFGLLLIGISSWNIKLNIF
IALCVLAKELLKYSRRHGTFTAAKVSYKTSPSHVKSRLNTSHFSPTPSTPKFNSTLPKTPQTPNFFSPIA
GLRRLPMRLGFMRNNEQNSTPEKKFLTKEEYEEQGERETKHAILTLKTSIENSPNPWKELSKLSKDTQSK
LITFMNTGDHLEKDSDELFNDLSDSEDESGSQDFAEENGEQNSFLKPTYSSLNKSYQNSPKRPASYHRQS
NGSQQQPPERHQTFNNTSRIPQKRWR

>E4YIJ6_OIKDI
MREKPQKKTEKIKEISSEPTKVFDDCSEESSDTIEWSAFTPKKMMSKAEYEEQGVIETNKAMEELRKIIE
NSQNPVKTFMKFGGETRAKLLDFINSGEHFSVEVNKNIPESQNFVEECEKVDQASPLKRKIDKLKKGQIY
ESPKRYPSFIHPSYIRTGEAALKRR

>E4YIK1_OIKDI
MGKAGHRPGQWKQQNKKHNTGAHRSKGAIRKSVSGAVSSSGSMHSGKSSKASSKMARKASALSFRHKKEE
TSRVAYRPIHLIFFPLGDDTNAMEITHANLQCSGACEQTVGGSYQLALDRKKFNITRPNDISSLLVQSQV
CDALVLTMRYSDDFLRENIKLLEMLAHGVGLPQVFLAIQGLSEIPAAKVMAARNEVSKRFKDLNVIPCDK
MKTFPVDTIDQTKSLLRQIATVRQTARGHINRPFMLAEKIAVDGDDLKISGYLSRGLSVNLPIHVPGVGD
FLIKQVEDKHGEVIKKSKEDKLPNLNPEAEVDMLDAEQTFPTEDELKDAENPQSQTVKRKVPKGYSAYQA
AWIPDDDSESGNEDDIIGDDDREYFEDEDKVIAEMDSGMGVDSKEEEEDDEEDKDGSKYDKNYDYDQEQQ
FLSLHRKAKDDRAFPDEIDTPQDARTRLQKYRGLTSFRTSPWDKNENLPADYARVFKFKRWAHAKIRAKK
WVPEIAETVAAGQMVTLVLKIREENKAALRAIGDMDSLVIFGLLANEHKMSVMHFVMKRANGFDAEIANK
SELYFTCGFRRFKARPIFSQHTNGNKHKMERFLPIDKTRIVMSCYAPIMFPPQGVLAFAKDSSGNFKLAA
TGSILSSDPDRINLKRIVLSGAPFKINKKSCVCRFLFFNRLDIHWFKPLELRSKYGRRGHIKEPVGTHGH
FKAIFDQHLTSMDTVLMTLYKRVYPKWTYESVPKLDGELLV

>E4YIK3_OIKDI
MSSDSELEEIMVPAKKKIKQKKIPEELRDWIEPVLWKFNKTCMKSEHRVDRKPLLLRNYRDLEDGYIVAI
MIKEYCPKVVQTHNLTRTLKRVSKIDNWAFVSQRLWIKTGFNLDTRKIDKILDHEPSVLFEVLLDLKHFL
EENYAKDEEGSDKSSEVAYVDVEKYIPAVLDSPKAKKPLSKVNVKVLDNKAKGALHRNDTIFKNNRKCVH
ELRARFKSLQSDVQERNSEIIMLESSARPASSINASESSKKKSGCCCVIS

>E4YIK7_OIKDI
MQSNLTKAGILITGIIGAIVGVVAWKFRQNSENEDDSRVEIISIDETPIDLEIETEPEEDEEEESDFYDD
EEDKDEEIRSAIGSIFNLLEQMLQFRKIPEVVQLLQDILFRTNDSAQLIHIAKQSRASLILKCISHENEK
VRMLAVRIIQNISSCKTGQDSAKFLVPKLVDIANAFLINSTESDVMLKSLTDCIANFSANYKPDTQASYM
SFFQTASSCDLSDDDDFFGSSLMKIVVNLSDQKLGQDLLLKPELEDTMVRVERMIEEKQGGTRVQARCLI
AVKNMISETDWQLRRQESLDIILEYSNSRLADKELVERSSQILFNLEDKSEPQEN

>E4YIL1_OIKDI

MKEITFALQKSKLQNLPKIGEIQFGNQALNSIGKETFKDIPSLTKLSFQSTHVGELRPDTFLGLENLVSI
DFRSSKLQKIHEGAFAGLFNLEQIALTANGINELPEIMFEDSSNLKILLMDSNKIESFENTTFSGLFQLE
RLELQNNNLKFLPEFGFKDFEKVTKLKLSNNKLHQISENDLIGLESLEDLTINDNELNFVHENAFASLKK
LKFLEMNNNKIQVIEPNWFLNIIPRSTQAAQFGLNDNIWNCGCNSTRFRLWYELDENKDLFTTLTKIEHM
NCSSPEYMKNFTINGLDLTWFPKSEDDPCEPPPRIWGHPNLSIKRKSEAQLICGIKEGKPQPKYFWKRPD
NSTVWTNTSTLTIEAISDADRGVYECKALNIAGESIEKHSVEVTWEDAITDELGNYIGIRGTNSAQSQFL
CSFFFYLFIILFFH

>E4YIL8_OIKDI
MVQNLMKDVNENTFDSHFEINPNPWIAGCEKNEKENDPKIIRPPKPSQSIKLLSTAKNIPRSAFDLPNIS
NSDRVVQCSKMPPFSENMRKQPEIEFMTMQNDDFSDSKYAKWLKKQICNPESVENLQTRPVHLKSIVSES
NMPRDAEEQRLSNLKNLLNESLYRLLKYPADYNSILTCLMDTIVIGSKGFGDQFMVYVAETIYSTCICEI
PFCHIASKLVAEICLKIKKDETEKGFRTILLMMLQIEYKKKDEFAKSMEISDRERLQTLTMFIAELLVNF
LIPMDNGKPSRIQIFSKLLPSLLTTLMNTPSQSNVRIASQVLRLGVGPIIEIEQNNQAALSEVWHLLSNL
ARKGNEHAKKVLIHRATGWKSYFPRREENKNVRELNSYEYGRGEPVFFTNVGIPYMVSSLADPKVLKELF
Y

>E4YIL9_OIKDI
MLFSLRLLTLFLVLGICQRSPADYPIFKNIDGEWTISLGRILAQIRKQVDDPSDIMIYG

>E4YIM2_OIKDI
MSGLVSHLSTSIGQDVVPGCVAIAKFNPEEPKDQIAVACRGGKVCVYNLSELAARTTTYYKPVTVIDVKD
EVISIAAAPWKTATQKYQLLLIASSSTIQLYDIYENHSLFHRILPDEIRTISIGCVAEKTESWILTGGES
SITGFDKTGSEVYWNVSSDAVNAICFVDLIGKNYDQIVVGTTDQEITIYDHEELIDTRTEVSQIALLTRV
GADQMAYALQNGTIGVYQRLNRLWRVKSKSTATCQCFYKPNKSLLTGWESGRWEMRKMESGEIIYKSQIE
CSVAKVVAHSFSTAGDDLLVFGKNGSIEVFLPPTEIDKKKRKAVDENEALRKIEHRRQILKKELDHVEMA
QKTMTVSERIEQNVIPRNVKLSVSISNSNDGLFVSVSVDSDLKIFLLVFLSEGLFGNEEAKTEVFPAGKQ
ELRVHIAEAVSTVTALGIKAGVGYSGSKTLHVLEEMIEIPIFAHFVLNDVGQMPLGKVEFPFSERPQRFF
LWLKENFLLADKRQLSSNNNAMCFKSPSGEKVCFHLAGDKLLIYADTVKWASDAVQSLASYFGINDMSSI
CDFPEIKEIEGLFQEIADIQASLVRIDSDIAEITQGLMSSLVRLEDSRLLEDWKSMEQVQSMIMNADSDL
MLKQRVRHANRDNLTKILKKLNHYIDSGSKLRIGKEPVKILNVCRQAVKTKKPKVIVRTLLHGAN

>E4YIM9_OIKDI
MLLFFEFFALFNCREPNPEERLHFDLEEYYYKDVSPLLVLPKNTSIDSKEYREEVCAEWIPKILENFDEN
VDAENALSHIFYCVDAVDGFDELEACDEWLDYIHFKNLRSTSYKGKCEVHDEHYLERKQSASLGKVADFG
MHDDASANEIVILNDIINRPLYDHVIDVDLYIELDTINEIDLRDALMDMYADWYDSRLEWNKTEYSGVMY
TVFDTSKIWTPPIEVMNLLKMINPKLESLCEVHHDGTVHEIIKMRALVSCNVDQNLYPFDTQECGLNFAT
PSMADYKLYFHLHKWNMLNNLGNLSASIANYEEVHLSVLKGSFYEKNQEWDLLGYKYESGTVVGHLKRSY
SLVRVYFLLERHTIYYEVTLFIPIVCINLLVCLGLWMPISCGEAAGFQVTLLLALILYLDLISTTTPVFD
SIGKSPRLLILFLITTIASVVAHIIITFSMWKQSCDDDTLRNLSPFKCKFTQKTAKVLEWLTREKYEIPK
AIQLIIDDAENIEKLEEEELKKAWKFYGKMSRKRFRNYLFFRVIKILQLFLNRKKSFLGFLVNFRRHFLH
FCDHS

>E4YIN2_OIKDI

MVENSLKTERRDEEEQEKNPPKFLKLNSINGATNSLANVLPVDQTPTPTKIIKLASELFPAESNNTVNTV
TAAKSEVNSSQPNPFDKSFKQSTSQSPENGISGNEPVIQELDNVDESIDEHDDGDHEFVEEIVDEEEIEE
ASEKFKVG

>E4YIP1_OIKDI
MVTAFADYTSAPFYPGCDAPEVLKKFLSICAIMFIMIVNGLSVKASERMQIIFTVVKLVLISAIIIGGFV
EIAKGNTENFENAFEGTNPSISAWAVAIYNGMWSYDGWNQLNFVSQELINPERNFPLVIIGGIPLVTLLY
LLVNVSYFTVMSPSELLQSPAVATTFGAKVFGSFAWIVPVGVACSTFGSSNGEAFTSARLVYAAGGNGHF
PRFLAYLSNERLTPLMAVVFNAIIGILMVLPESSNIENLLDYFSFAMWTIYALTFISIIVFRYRKPYCDV
ERKFKIWLPLPILAAAISLYLVIAPLIEEPSLAYLLATCIIFGGLIFYIPFVYLDWNLPFGIYNKVEIFC
QKYFEVVPVSAEELKTE

>E4YIP7_OIKDI
MKLGSWLCPIVASKLSPFLDDIHYEIKWVGEQVNFGELGYKYEEEDFVVMKTANQEEYKCFFPSDESVSL
EEKSNEETGESAETLLAPLFKETAKKDCSLRIETYWSYELCHGQHIRQFHEEKVKGSVKSTEYYLGRLKE
NPLNPANAAKNRAEVSKKKLDGLPTPYHSISMGDGTECDIHTGKRRKTDVRYICNPNAMKPEIMSITETT
TCEYEILVLTNLLCSSPLYSLHLDTETFGIPCQKIGDSPTVPKGYVKPIEPLSIEGMLDLLKDGVDLDKL
GAAFFKFDAETGALQPLSEDELGDFDLEANQVGFTFIFVILVFRSEGIVQTIIKHAQEMGVNVNEIQIDG
LDQKDNTAKEIKKKLSEHTRSQINAKMPSQKVQEAKKAQLSAETEKILNDFLAGDFCLRGGQGWWKYEFC
YGKHVKQYHEFQKEQGKPKEPTKEIIVGKWDESIHLEWAKNRQNVKHSKEIVKTGQQVKAGKTIHGDDGF
PISVWDSEDKVDEVQTIDGDVQEVELWYSNGEVCDITKKPREVLVRLKCKPNQDALSLYLLEPKTCSYIL
VLESKLLCSILKNADENGLLSLK

>E4YIP8_OIKDI
MRTGLSSYGLIFQALQCNINCPCQPDCKNRLVQQGCDRPLRVKYFGSEKGHGLVAENEIHKGEFIIEYMG
EYLNLEAVNERQVYQRENDKMNYILSLAEVFGNGEKEIVHIDAGKLGNAARFANHSCSPNSKLYPVRVEN
DIARIAIFAERFIEPGEEITYDYGSAESTLSERKCQCGSRCCRHFMPSDINL

>E4YIP9_OIKDI
MNEEQMKASVLLDLEQTGRLEQLRAKVRYEVYQSLAKEKVVAKKELSNREVLTVSIISDWLDKHGFTVTA
AQLNSETQMTGNPLPKTVVKSELRIDPSDSRAILDSLIDNHLGALPLQQNKNKRPSLDPPQHIKCQSQES
DMIVFKK

>E4YIQ4_OIKDI
MDWLNEQQEKNDSIPDTPSSPESVAALVIDESAHSIISSPESVSSSSSETTRRKNRQKSPSNGLQFPRPI
MRDHRIKRRRTSEQNHNQKKEKLKTDWPSSRRSKF

>E4YIQ7_OIKDI
MLFFLLFLRLNGATDRPDTIWLPEAETLFDSNFVHDLEQIKNLEAKHEFELPLEVSANKNDFWILHNDDY
ITIYNKKANQVYMVTGKGLRWNSFERVLRSKVVDPRSKTKNSGIQPLFLAELASSKLKTTHVRSSNNFMP
LSTNAHREFLYKFVPSIEDENIKFAFIPTYRRDKVVSIRYLFNQSNEISFGFDLDVTEDIEENSSLADIL
NRINDVEFDRNQAFSATDDLKSIINMFRKRRSTNRSKRSAFANDASIFLCKNMNGQVFFPVWDRCGEAPS
SKTYQQNTAPESVQCWCDDGCLDRQDCCTDFLTSCKGEQSFQNSECIAEKNFECSDSSFEGTTILISTDG
FRESYLSERKEFLPTYMKFRECGSFPEFMEPSYPSKTFPNHHTLMTGLYPGQHGIVANKWYDWKKDASCN

VFSANTGEHPCNVSSPEEGWWSGDPFWNTVERHDMISASCLWAGNDLTINGEQPTYFWDYGTYGEEPFGY
RIYQMLKWLNLPPYPGSGPKEGFRPKFLTLYFDEPDHIGHAFGPYDASGELDAALKRVDFQLQQLMDGLK
LYNLEKCVNIVLTTDHGMEQALCEDMVAIAPVFDAANPEIMKTTNFLTKTTTAQMGGLMGSNKSDSFNVM
EVIDTVKCTWHGNSFKVWDKYFAPKRLHYDNASPAIEVLLFYMKRDTKIIDYPWDSCSDEGYMGNHGWDT
KYGGMKVPLTLYGPKIRPNVVLPPIQNVEVFRLLMDLLGIKSNSPKLMQQFYSKQFSLDGRFEPALKRPT
NPGKQPDFSGDVSFPTGVTFNKSSEIAFDVNHKFATYAKCIELDECIQKLKLGFEENPMLEFFDDPFWWH
TLKDDMLPRIFTNNVKSLRFGAAFDTTGTGVYVPPTQWMSRTENKWCNRQGVYQPSHMYFILEDEEGSLY
PALYPWRHNTSASFESEYSPCIVINEEETRQLYFGNFNCRLTFFIPFVEWWQEKLEIHSVRLLEIERMTG
MEFLGEEDRVRRVALRSTMNVEGDLDWAFKEPSSSSVAALCSFLSFSFLFQLIIN

>E4YIQ8_OIKDI
MSRRIIANIKQNPIIKIEHFHVANSVVISLKDTNFFLAKTGGDDDGDQDQFTTLTPLTTTTSTADQTKLP
SIQTISSSRVAGSFSIVNCPSVESYEVPKINGMGHALPPLSGTMVVPIETKTQTIPVVSDINVTNGSQPM
TITTVNGNWNYASVLERDCGVVNIINTTAFHGVPDNAVFLEVVPQSAIETSSEMKQNDSLGNLIDQAEES
THLNSVMGNIKTEYRLEPARSSPARIAPVSRTQSESTHEMHIPIIQAHQVETECEYDIFKASPDLKMTDV
QEKKTRNSKVSDAPVEGIEEINTKVVAQQITAELKRYSIPQAIFAQRVLCRSQGTLSDLLRNPKPWSKLK
SGRETFRRMQSWLREPEFQRMSSLRLAACRRKEDERDRQNSTTKKPRLVFTDHQRRTLHQIFKENKRPSK
EIQITISAQLGLELSTVSNFFMNARRRSSDKWLDKVVLSSNKKIMNFKVDDYTPYD

>E4YH58_OIKDI
MSGIFELTELKDRIIIAGGGLVGGVAAAMLADKGHQIDLYELRDDPRNENAAAGRSINLSLSHRGMRSLR
QIGMEEECLANGVKMPARMIHKVSGETYEVPYGRTKEHFILSISRLRLNQMILTAAEKRENVNLHFNHRI
TDVDLSGTLKFSDKPDASGDVLIGADGAYSKVRSKMARGVFDFSQTYIPHGYKELVMDKAENGDWRLPKN
YLHIWPRGQFMMIALPNLDGSFTCTLFLPFEMFKTIETQETGIAFMEEHFPDSISIFGRDNLMKQFGPGT
TPGLPMISVKTSPHSVDNTVIVGDAAHAIVPFYGQGMNAGMEDIAVLMDTYAKHNDLKSTIKEYGVFRPK
DAHAIADLAMYNYIEMRDLVTSNWFLFRRSLDRLLATFLPGSVIPLYEMVVFRPQMGYSNAIERNKKQST
MIDWTLAAAATASLAFALKKLTREGNLSSLQFDKLSIGSAQDFVSSLLK

>E4YIQ9_OIKDI
MTESDPRKPIGKAFIENSKEQSFLDQKISFVFRGKLSQHSTHDLDRQSIQIAAALIKNGIVNMDVALVGG
FTITSTSLALALMMSRNRLIILDSAEADRFSFIERINAEAIFIDESSLRSDVTLNHGVKDIYSFESSSEF
KIVTDKKITKLELSESNQEAKDSVEKFIDENISFNEIGFYTTTSGSTGVPKIIKMRNSTFYAKLKWATNP
AGPQDLPKLAAKFGPDLPNVMGHINNIAAFANFVGHIVVFVFSILSEPEARKNILFCDQKDLKNSLAAAA
VCKSGSLWAYVPKMLEIVESKEFKEIKLPTVSLFLAGGAKITKEHIRRVRSNFAASQDGRKCHVLKLYGS
TEMGSFLFRTGLLETDEIIESTDGIFCSNEGYYAEVRDKDGKVLPAGEKGELFVKSPDLMMGYIGFPDQG
EWYEMGDEAYLTDDNHVVVLGRLKEQITFVNTKKSNSVELEDRMYKAGDFVKSVIVLAKRNENAFDDIYY
IVYCDEADDEKILKHVEDDVLLLTKERPKVIFVREPFKMLANGKFDKKSLAAKYLE

>E4YIR1_OIKDI
MNGVDVSVAQDSGILKAVLSEGVGTATPSFGSEVTVHYTGSLNDGSQFDSSRGRGVFKFTLGQGQVIKGW
DEGVKSMKKGEISVFTLRPEYAYGDAGSPPKIPANATLTFDIELISWKAEDISENSDGSILRTFVKKGKE
SWGNVVDCAEATVKCRIVKSSLDEVVHDFGKINFRVGEAELVNLPVGIDFAVKKMNRGDVARLTLSGKAD
LKAECRKALGLQEFGEYEYELELVEFEKVQEAWEMDDQTKIEQAELSKSKGTERLKDQKYDLSIKHYNRV
ISLLDHQETKENNEKFEEISSKFKSLKLAAFLNLSLVYPKIAENYKAISAADDAIKIDPENEKAFFRRGT

ARMAGNDLEAAISDFKKVVEVNKENKTAAKNMKICSERRKKQKEEEKKKYAGKLFG

>E4YIR8_OIKDI
MQQNNKCTVGERFWPRNLYAGVSWPKFTPSTRKTIEFSLRGSKVYRDEPSVERTLVDYASQRMKLWNDLI
PKMMQPLKKAKINFPSSSFIPEPTDETATIADDLKNMNNITIQSFEDKQKEEAEKEVGIGLEISVTLSIG
LMLFIINAFAFLQMAIKKQREKKNFRVKIEDQIKERLIKNKQNNSIPNRVNKLTLGNDLRRSQLSSEGHK
NYLSRKMSLERSQSPRKDSTGR

>E4YIR9_OIKDI
MFSDFGWTSNAMRTAELHSGLNYDTFVYLFDHRMGSETYRPSELDRAGTNQAIAFLFGIPFYGKSTIGTI
FDSILWSPEEKTLSCSMMTYFANFINYG

>E4YIT1_OIKDI
MILSDGLSGAAVKLTLLGISLFAFYIRTFGVRKFEPVLNGSEGYRILRKLEILENGGDLDFDEQTWYPFG
FLDDDKDLVLIRLIQAFRFITGVLWKTLTSHDACVYFGPFCGAVTPWISFFVVRSLVRETLPAICASFYV
AVLPGFAFSTVAGRLSSEALLPAIIFVFLHLTNKAMSQGCLKDVAMLSLFYYVSSSLWSDYFLLMNFVAV
SLFCKLFHGGYDCAIYTAYSSFYGLSTCLTLYKSNFLASEQFIPLFAFVYLQYYALSIRIGDKEEIQRPL
QASFTSMLLLLFYMKYTEIIAPLKEENAIYLNPSLQKTSLAVLTSSQSAQPSAWAALFFDNHYGPMLLPV
GLSILSKKFPKVKTNITVYSVIALYLAAVKTSNQILLVPALSITSSIAISQIIEVCLELLKSTEPPTGPT
KRQSQKYETEKNNLPMKRFFAGAAGTLCAAHLIYAARHGVWVTKNIYSDTTIFVTASTGGSDGRALIFDD
FRETYSWLKHNTPEDSKIAAWSDYGYQINQLAERGTLADNSASNKTSIAQIGKAFASNEEQGWSRLRHLG
ADFVYVTFGGQVGYSKDDINKFLWMLRIADSEHVTGEFNQKRYKNVNNEFRVDVEGSQTWLNSLLYKLSY
YRYGGQTVAKGKATGWDRVRKAEIGNKDFELDFFEEVYTSQRWMVRVYRLKTPDEISISLY

>E4YIU6_OIKDI
MKVEELLKSNDKKDIGIVVPDNWQSVSYDCDQFALPTHYKKYLERVILPRGLVLDRTEKLVLDLFHSLDR
SVPVVALCVLKGGFQYFADVCDNLKRHAAAHYPEAIQFNMDFIRLKSYEDDRANEEVQVIGGDNLEHLRG
KQVIVVEDIVDTGNTIRKLMNVLAKYDPKSVKVVTMLSKRTPLNTSGYVADFSAFSIPVFFAVGYALDYN
EYFRDLNHVCVLNNEGIEKFSAKNLPN

>E4YIU9_OIKDI
MSMERALFKNASFAFETSAMSFDEIENKLYWVKNGETVFSSNANGEEVTSHVTIAGAQISCLVSKGDFIF
AADELLDEESREYKIHVISKKNEQTAVNYRVDKKLTCVGLFTRF

>E4YIV1_OIKDI
MKLFSVLASTFAASETCSKKSTCNETKKLVPVEAINFARVQSHTNMKKWGGLFGLNRFHHMRKVSGPEDP
LQKPNADTLYSIGVIDLSAGPVELSIPDVGDKYLSLTCYDENMYITAYETAPAQFKLRKTENMTRYQVCA
LRLAVDNQEEIPELHKMQDKGQIISPLDSYPPVDLPEYEPESYKSVYSHLMELAYYMDDLGVGLGFKGEN
INDVSRILAAAAGWGGLGTSMVKYENGKVSSNDGKTEYMLKVIGEVPTDCFWSVTTYTEDRLSYGINNVN
SNNVKRDEDGNVIIVFSNDHNDDSLNYLNIEEEWSYIVRIYEPKQAVQDGTWKFPEAVLYQ

>E4YIV7_OIKDI
MASVASISDQKMSHHRNQHLRQTFQNSPYSQGSSRSQGCKFRQKILSESEAKAKSSKKSRAKAKRKRRVS
KNLERKRSESENFEIFLSESEANSMYHSFMPDAPAYEFPQQGMPRSTAGSVSSIRSESGRSSALHDFMSS

VKQEEKEPETYSKVLNLIEARLDDQQDATKGLMTKAVTEIKEESKSNAADLSSMLVSMNERITELEGKIT
SKEDLSSNQKMLLGEIDKMVDIKAQKWYKEFRVPKKETVCLGVQCSMSQESIPASPEKRQRRRKRHISTP
PASPSPKRISTGVQKRTDKKFISRKSEKAGLQFVDANRLRKKSPKKKVRYFSDRIGADEDEIFRSLRPRT
QEKRNNAFRRIPTFFDEREEDFRVYPGTCPAKPRTKVSKAIRPSQDLPRPVIRNEPLDRTEVFFESSDDE
ESNRKASKEEKFVIPRRRKRPPTCSVYDFTSSYVSSGDETQISIQDTSEYGSQ

>E4YIV9_OIKDI
PSLKTKLKLYMNTKGKTRNAFQSFRDLISKRPRDEFKYLSQLQLTNFQNHLTKTIALLNDYNFDEIDTLV
LLTSQLGNMQIWSEFIEKHCTNLIEFRTNALVASYKLEHLDIYYSALQSTMAGAEVLVISLNLPNLRFLQ
LANIALNERGLFNFLQTSKTFLGFPKLKFLSLRRSFIPQSTTPDVLSFFLKNAENIEAIDLQGCSCFNFA
DLLVGIGPLHRRQRLKSVDFSGTIAKNEDDFNRFFNVQGLDCAIEDLSLSGINTKANAEDFYLPFINKFV
LHADRLKNLDLGRTSIEKEEARLLLERKPHLNSLTLSGCYGIPRSWRKIIKTEEFSNAIRAFYE

>E4YIW0_OIKDI
MSSKIRVFARRLGSGTNKPIYLQEKYSNFVPRPLSADDLILFADKNSVEKSFEFLKQELPTRWMQIFAEI
NALPIEKPSPLMLEVKSMIQETLENLLPFTDQPATENVISKFNSKLEDYIHRHRTAFDEVALAILEFKDE
QVEMAEKGLTTFDLDDAEEKVHYFLDRYFTTLVSTNLIIHQHLVVCFHRNPVLILGMNSDRNKRETVQRT
NVTKEIRKIASRIEIECEKYYGKTPQVKITEFDMGKATNNSQINTIFIPTHLEQICAELIRNAVRATVEN
NLDLPPVEIIISRAKENITIKISDRGKGASLHEQAKWGAYLYTNPPEQSTSKMPIVEPLAGYGYGIPLAA
VYARYLGGDVVIQSLQNYGTDVVIYLRSNAEDLKEVLPVFTAKTKEYYNSQNRKKTWISGKSSEKGDIVF
NG

>E4YIW2_OIKDI
MSKLKETQETLKKTVLNQNDLLRNFQKSTLDSQTALSKEFEERLDKLEDVTSKINEDFATFNQEVRHKIT
NLDTLSTQNAVKMTTLEETDNSYHLRLVQQQLRTSQRDNEVLKKKIEALERQVDPVLLAFGDDPREKIRQ
ICQQEMMRHGVIDSYSRGRN

>E4YIW3_OIKDI
MDKLISETFEKRKEISELKASVEEDRATCLAVSDTAEENISKTENSLNEKIQQVNKEIQQKEKKFQEMLE
KATDDVERKNDKLVESVLNLDQNAEIKSLENQLLKFDNRLTRENEIIRKKLEKHNRRISEKTEIALEKLN
FKVEEVDDKMTKEKFLLSSEIEKLAETIKDQQKSMSELKEEQYLLQLETIGKSSALGTLEKSYNELQSAF
IHLGSRVQQKMNQTIINMSTNHRNYQRYNNEKIDELRDKLSEIIANHNLQGQRVRENSNLTMKLFLNEHM
LNRTQSHQGYQLLQLAQEAKRCTDETDNIIRKFNEQQAQLQSQKDEMRDLRTILLQVNCFFSINKQKLFL
GSGGSTRGKDKCRSSRAAKSDQ

>E4YIW4_OIKDI
MFLVTGACLAFFVLLIVILLMCYCRLYYACRKENPKERHNYLHKKFNLREVFLHVIQLTLYPRGRSIRLH
DLRDLEKRSKQLHKLTYLQVTLFKENMRGSFVLRNLARERCLVTTLQQLKPVKVDNVERKLLFNRKII

>E4YIW8_OIKDI
MREHHPMRYHRMTEILKTVASVKLSSTFLLKSYFLKKPLPMLMALYLFNVFAVGYIVFALERLRGKCMDY
TDVVWMMGVTITNLGFGDFTPCFWLSRIIISLLSLLGIFQTALIVGVLSDTLVIPPDEKRILASVEKQRA
DQIRRHAAAKLIQATWLHYRQQKDRDNEPRNGISRRMTFTRYRLRRHAHKSYVDALWQWRRVKTSTETVG
QSLEKEFLVDDTAITTTYISRKLDALEKMVRKGGSSTPVNENNTKLKWLSAGRNALNSKFKAKPKNVNGK

IKDKSALCSEAPSKKGSIRKKPHSPVSLMDKLKSLKTNQVESTNEDHSQRHPETISSEISSPEMSRDQER
RERQQSNIILQDHLIGKLSNQVKGLEEKQEVVKQEISSIKSLLAALKEVATESTIDSAETFSSNPESDQD
RLQTAFQKALRRMLRSFFNHFLNNFCHTIMFFLNNDLSLEKSM

>E4YIX0_OIKDI
MLEVSVGDVWELASTISSQLEKLTADFGIEKIQSFVDPTIRALELLESVVEERSQLIELKLTAEKIAENL
RNMSSSQENSKDQGTKNFCEKQVYEEQMSSKNIELHQFQQTVAQYKIKDQQQHSEITRQRQMLLTYEKRV
TELSDELRLKECAIRELEADCDTLEGEANRLLEINNELRSVHNLTIESSLPPSVINDSIAAELSHCEESF
SIDTENLPDRTGPEGEEYQEEEQIDQAWQSKKSLDEKDKPRYTLYELEEVLNEKNKYKERCFVLEEKLRD
LTNDESVLWEGLSIKDLEMVEPTTIPVQATTPSTPSDPKSIRKMMSKWFRTGPTAAYQNYQSKSSTQSLQ
MTPSASQNLYIRSDPIDDDIKTLKANHQKQ

>E4YIX9_OIKDI
MGCVNIEVEKGVRSRFFRSADMEGIAASSEGAEHLEILGDLEHGWEEVGGVFILSVYMIAAALLKLGFHQ
ICGNPFPVPEDSVCSLSAPDLLREGSAYLIQDDQNIADIYFTPTIFFVIILPPIVFEAGYFMPKDPFFDN
LGTILTYAIVGTFFNAMAIGGSIYLVVEYGLVPGFDEHISFIHCLLFGSIISAVDPVAVIAVFDEIHVNM
TLYICVFGESLLNDGVAVVLYRVFEGFATLENNEPPPSNWGPEGQLLILDLFSVKTVFVYVGSLMTKYTK
HAPIIEPLLIYACAYLAYLTAEWIQLSSIIAIVFAGFFMKPFCEANMAQSSISSLHYIQKLLSSIMDITI
FIFLGISAISDFWVHWNTGFVVCIQFKTIVFISIYRVISVYVLTFILNSGRLDRISYVDQFVMAYGGLRG
GIAFSLCKLMCINTVPSIQSMLCSTMVAVFFTSFVQGMTIKPIVEWLQVKKQGEEEQTVMKEMTESFVDH
LTVGIADIIQSRGRHWWMNRIANLTDEYLTPILVREPDKIKNQSIIDVWAQLNVEDAAKLVQKQTDVKNY
HGLDSDDDDLLKMRLLLPGALVEDERAGGDKEAPHGHEGFGGEREQTDIKMHHMLRDNLYANRQRIELSG
HMKYRNLGTNAEQQSLTKQQQEILVRRRIKSLKETPEQIEFRKQMRKTEAQRKTYRKTISEGRKTMSIDP
RKTVTTPVTPEPEMFEMSQRKTKILFEVPVDPTPSSDLPWRNSGEDDHAAARSTHGRPQSQLLEHEKSL

>E4YIY0_OIKDI
MRPTTTPRTTPVRTTRRTTPYVRPSSPASRPKGDGGKVNCPPGKNIKRCRDLEAQKKKQHDAPSNSPIFE
QHMAAPVMESPVKDRNLGATGAQFDSFIMDFLSPFLIEDPVTTPQTTTTRAPRTTKKTMIGGSSFMSFTG
YGAPENPSSGVCRISPKSTIYAAISSAVGSREDCGKCVFVWCPSFSGCKGAPIGKVQIVDVYDGPGHIAL
SQAAFKAAIGLPSTKGNKMKTYQGTYSFTSC

>E4YIY2_OIKDI
MWIYEPRLEYLKNNHKISCPYLPALQARFSTAVLFFFSCILFGMRFMCKPFLCPSMLVILSCLGVVHALF
MMGSSSTTATFSLVLGNIIWSFGFIFMMLWFYKYYAYYYRKIGENFYGWKACVYIQASLIFINAFVAIIW
QLPNLDEIATSSLSCNVAGYQQLKIYFYNFDTHERALTEIVL

>E4YIY3_OIKDI
MANRDEEILTQSQCLVEKPEKAVTYEESSLVDTANGQPNENKAREALGYKEEGNAGFMLSVFNLMNAILG
SGILGLSYAMAQLGFVGFFIICLAVAVLAYFAIHLLLSLCAKTGVKSYERLGYRAFGTPGKLVTAVCILM
QNIGAMSSYMFIIKNQLPSVLHTIICAAADTPEECPNLYGEDVPWYLNGNMMVIIVVVCVVAPLASLKSI
EFLGYTSGFSITCMVFFTIIIVSKYFIGVEDCPLFDDAIANGTWETSTEATCNLKFARLKFYEIPFSAPI
WDAENSKISDECGTTYKMPGPYCNLEAQECGASAFVMNEKTVYAMPTMTFSFVCHTAILPIFAELKSGSH
AEMKKVSKTSLGSCFLLYFIAALFGYFTFYNYVQSDLLLTYNHSDPTNPLTLVVRVCVLIGVILTLPLTH
FPARKAVNFLFFPKHPFAWTRHLGIMAALLALCVTLVINVPDIREIFGFVGASASGSLIFILPSLFYIKI

QRDEKLPFFESLKETRLLMASFCFLVFGCIFSLMTLGIMTAAKLS

>E4YIZ2_OIKDI
MWKNAARQIGVIKNHSYHPCFQIKADEPAKGVVFFMHGLGDSGMGWADAFANYCADPNVRYIFPSAKEMP
VTLNMGMNMPSWFDIKELSASASDRYDLDQLNRTSEEMVKIVDEILEEEGLTRENLVIGGFSQGGALALN
IALNHYENVAGILAMSTFLPIDEVSKNYKKSLPGPISQHHGTADGVLPFFFAQQSAEFFKKVASKEEDFQ
FFAYEGMEHSSCLEELQNVNDFVKKCLNL

>E4YIZ6_OIKDI
MTSGQDSLILRCLRSFFEGIKCCIFKCCGAFFRTPEQGAQTSLKCICDSALTTGKYFSNLQEAPVQIRGS
RKLEKIQADLWNITEDFLKGKD

>E4YIZ9_OIKDI
MSYPGSGNTWTRILLEDITGIYTGSIYRDGSLTRGGFKGEGTNPMKGQTMIVKSHFPSNQKVMHAAKAII
FVVRNPFDAIIAEYKRRRGHGHTSVVSEKVFKDKMWLGNAPRWLDGWGRLAKNVTSIAAANNLPLHIYSF
ERLKENPTYEMEKVLNFLEDAINWKPDNRYKRLKCLGELQKAATSFKREKTPPSFEYYTPEMIEIGNKAI
HEVHKNLLEAGFDVFDVEEYLRSS

>E4YJ07_OIKDI
MGFTITLQPESEEQNCELKMKSSTIQTIKDNLVGIRAGFMLAWLLYSYLVFREDPENRYEGQYFFDYVSN
ANEFCCAVWAVVTLFARHSTNKRLLFWEKWLSAQTATGSWLIALVYWVMMETTDKTFHSFWQHGILGIVT
SLDFLAREPFAFKWIFAPVLYCGLNVLYLFTIANLWPEYRSTMYSFTDYGPLGSDQMWSVLKLQSALTFL
IQPSIFSALWTLQHIVSAIRRRVSKNEYVMTSDSDQELIKT

>E4YJ09_OIKDI
MNIQFFNFEILEVSQEQKPMPANVRQMSGSQNVRHLNRQELATMSNLDKRLKGSRVGPQTLLDKHEAKKN
AQTKQKYKTGAIEEEYEVGQDLGAGQFAVVKRVRHRKTGKFYAAKYIRKRKMKTSRRGVPQEEIEKEIAV
LQDLDHPRIVKLRESWNTANEIILVLELVSGGELFDYLAEREQLTENEAAGIIKQVLETISYMHELKIAH
FDLKPENVMCLPGNVPAGGAPKIKLVDFGLSQRCDLGIEVTAMHGTPEFVAPEVLAFEPIGLEADLWSIG
VITYILLSGCSPFQGDDKAETFQRIAQMDYSFEDEDFAGISQDAKDFIEMLFTRNPLERATAKDCLKSSW
IKRFTPEGKAIDIEAEMRVLRREQKELEETVKIHEGQFEKQKEAVQSQERNRQASGDSSAAGLLRKLSEQ
RKNLQRDVDDFRTQLIGKEGARRRGSLIREPSEVQTQRHYDTSFNINGRTYDVQQTRQTIGSGISRLEQS
ANTLETHRTDIRRMTRTSQNTMDSLNRSLNSSLTRFSSLGSEVRSRFGSDLSTTAYSSLYSGRKKF

>E4YJ10_OIKDI
MPPPGYVGQNFVTNPHCIMPDLRDRGGTPDQLTPNEEEDEDDDEPIIESCCFCCNLGFGILFGALFFLIF
DVVFLVKKMKNLFFTEIVDTVFEIEENKITTAMEIISTIFSAFAVLASFILLVSVCVCGSPDKKKTMGIV
SRYWAFQIGLNALFQAVESLFNTMPRDDSEFFVSLPGIGVDEVVGGVAIAVTWIFTVGYIAAYFFMILIL
ISYAAVLKRSAKKKLESLYAKRKQREYAAMLNLPNFDNSDLQSQITVSDRISDMSAYESSISPTIGEGNY
QDVISLQGKENLEDIYQQRRIPVRRPTGYLPRNRSVSPDPILQAQLAMLHPMQKFQFLQQQLNARQQMML
PNRPLPKISEDQSDQISEISVKSKPNSSGHNVRFSNVNKEATIIEDSTQVAPSFGPRPANYVPEFSLQNG
QMGNYSRPIRPFPGPTRNLSFSMQRPGYTGSL

>E4YJ14_OIKDI

MGRISEEQHEEYLESEAFVSCDEELQNEQPRGIVRLKQQNNLKSKRKKILTSFERENQQLKRRISQFAKA
SKEIENILSSRKKIEIYSMPRDSAKKEEKKESTGGAGAGDELSGHSTLLPELQKQLSKAARKERRYRNGI
AIF

>E4YJ15_OIKDI
MEPIIFFILIGSCGLFVIIIVVVSIIYCRCPSKKKETPARNDTQRNVLLPDIDEEENFPLASSNRAETNW
KDSQDSFDQSEDEWVDQSSPNEAIKLCKYVTQTSASQSQNGGSRPISCSMHSISSNRPSVKPPAPPVPDH
SALLNAPPDFPPPPPPPAPESSGNMIANPKYPYHVPIANQQQH

>E4YJ17_OIKDI
MKLLATVLLGLSKGEIANCTAEYLTLGLDCTGLSADGNSCDTTTHRCFTSPDCHTEFGCRLLDECPCGES
IDNEKICLLCPEVTTSRPEMTTAPETLVEEPPFPEILRSFDCSVNEDFTLDLRMSTGFPRGRFLPDDREF
CIVNSDTSSAVKEMVSSRNVVTGIYGNSGECSCPFDPSNNGPISFESCPDVFGNSKRLVQEDGENFTIWT
FGIGFDDLFADNDWGASYLKYRGYSWEFECRIKTSGEVSVAAGQSIWERIIGNGVGEDELKFDLIALFEG
NPVNDQVIEILEDDTRLLQFDLDVLEVSDKYMPFYKSCDLIFYNTEDIMRQTSETSLPNIINNGCVNPSY
VSEFRMDTLDSLGRDVLTWFAPKIFETSGSQQTALLTCRVNGCTNFGVTGCTPDPQCASMYPVTRNNGSS
EVVGGEAKAEMKMSGADTFTFSLSLIITLFVFN

>E4YJ21_OIKDI
MKLLNFGFVVAAFGFDVSYDEESVNQMENAEIAEIPHHSENMDLGWAGAREPRGWGEYYDWVNILDATEE
FAEDKPILLLVIPEDCDDCVHCSAIREEFKNNQELLKISKHFHVVNFLGYEESQAHWDPKIANAHYFPRF
YFLEAHGDIIPWAVNTLRKDDRVFYYNHASEILNTAKEVFHGYYKYKHDEL

>E4YJ26_OIKDI
MESGDEEANENFFHEISKKQRFDRLLFTKNFPGANSKERMAHLLEITEEVPGTDGGVRITKESVNERGTG
ITVGDAVTTEAERMKELWHDYNLLPTAPLVSIHYAMWIGDDLLDKPTDYTYDRGNIETGASETVQIGNGS
ILAGLEKAILRMTQGSQHFVILSPEWGYGKWGNLPMIPGNATLVFQVYVRFVSYEIEDRFVRMPMRLREK
IPLKVILSTCTRLKFEAKKHFEKTSDVRKQVNRNTGEETYKKSGKNTGDFFGAERAGELLDCAANVLERA
MCKNLDEEIEMRKMLIHLHSDACLSFRKSKPSIFKKGIEHGEKCLANFDELKEIFMARMNCEEYAGKEQM
HDFLKVYYKRITKTRRNLMELYSRDIYNMFEDALRMYGAFDDEVHNDDDDEISFKDYITKQEQKDFLKMK
KLVDNRKDDWIKQQEGNEDEPEDENSDIPVAAGGQENDKFKVIKLSANRLNLGKCIEALNKEDMEAEDPT
SVPYPSSYEYYMSFIDRIIDEKDPNRTGHMFSHVHTSKKAVNFIIAYASSRGMKCFKRICDGMVNLVITS
DESKESSLLGNHQ

>E4YJ27_OIKDI
MKTSSLFGDAKSPNSEDLSPPSIDFSFGEEFTGGIFAAYFEAPESMIKPMPKDSSLEVNSQVVALQIGDS
ICDYKICSSYKIQEGDIKMVFPQFENRLEGETKCSYWNMTQKQWATNGVSMVARNETHITCTTNHLTNFA
TVHYMYPGNGRVSSKGLEITSNIVCGTSIISLIVVFLAMIRKIRMSKGSPVTWSRRSKQPILIAHLAIAL
FIKMASIMTLSIFDDSTVLTTDEIDFKCTTNGIILASMVQFAQLASICWMSCQAIDHYVKITRVAEGNLE
YPRRFMNFLLLLGWGFPTVIILAILILFFNKVEVFVDLSISDVERICWVNAGTLYVCWVIPFACFFMLNL
YIFGKVMSTIIQMRRTGGGLKRSDTDRLRRAMRIRSRKEVPTTNLS

>E4YJ28_OIKDI
MPRAEISIYLVVAGVIMVPLGFSAVLFVYLTTSHIWMVIPTSVTIGILGTVLAFQECTISNNELDSNESI

ISTRSTSVDSLAALSLDVPQASVTPHNASPIPLRPQRVKIQPEDQSLDQKEVQRGIENLARRSSDHEIIT
VSENLTEFPVLVFCFDKMAIDTKEEVILPNVPSIFDHGPVVETKGLRYDSNNFRLLRPFWKKNRFVKSSV
TPQQY

>E4YJ30_OIKDI
MTSLRVVAELQNPSIRDRHWHQLMNATGVRFTMDEKTSLDDLLKLNLHEFEDTVLMTIWQEVQRTWSYLE
SIFIGSEDIRRQLPEDSDRFDGIDKDFKACMEDAKGNLNVVHATNKPGLYEQLESMQDRLTLCEKSLAEY
LETKRLAFPRFYFVSSTDLLDILSNGNNPRVIAGHLAKLFDNTTDLKFADDTSKMAVGMYSSEREYVDFT
NECNCDGQVEVWLNRIMESMRSTIRALLSEAVVTYEEKAREKWLFDYAAQVSLTVTQIWWTTEVGIAFGR
LEEGYENSMKDYSKKQIQQLNNLITLLLGDLTKGDRQKIMTICTIDVHARDVFLYSYEYLGNTPRLVVTP
LTDRCYVTLTQSLHLIMGGAPAGPAGTGKTETTKDLGRALVLSVIAVQVKCIQDAIRDKKERFIFEGCDI
SLVPSVGIFITMNPGYAGRTELPENLKALFRPCAMVVPDFELIAENMLLAEGFLDAKYLAKKFITLYTLC
KELLSKQDHYDWGLRAIKSVLVVAGALKRSDRGRPEDEVLMRALRDFNVPKIVTDDMPVFLVLTLASNER
IPLNNTMRLLFEISNLRTATPATVSRAGILFLNASDLGWNPIVTSWIDTREVQAERANMIILFEKYVPPC
IEAFRTKFKTITPIPETAYLNTLCYLLECLLTPENVPFKTIKFPSQGTVFDYYINSEDKRFVHWSEMCAE
EKFIFDPEMPLQATIVPTAETTRVRFFMDMLMEHFVVGNVPFNFYTTSMMLQEILEKPLEKKAGRNFGPP
GSKRLVYFIDDMNMPEVDAYGTVQPHTLIRQHLDYSHWYDRTKLSLKEIHNCQYVSCMNPTSGSFTINPR
LQRHFSVFAISFPGMDALESIYKQIFEQHLLLNKFSANCTKLVEPLIKAALSVHQKVSTTYLPTAIKFHY
IFNLRDLSNIFQGMLFSSNDVFKAPTALARLYIHEATRVYCDKFTEKADIESFDALVTEICKKEFGDLDQ
EELFAKPLKFCHFSQGVGINKILTEALDSYNDVNAAMNLVLFEDAMSHILRINRILEMPRGNALLVGVGG
SGKQSLSRLAAFIASLDVFQIQLRKGYSITDLKVDLASQYIKAGVKNQPVMFLMTDAQVPQEDFLVLIND
LLASGEIPGLFADDELEEIISGVRNDVKATGMEDSRENCWSFFIDRVRRSLKVVLCFSPVGVKLRVRARK
FPALVNCTSIDWFHEWPQDALKSVSLRFLNEIEEVPEQLKTSVSEFMAFVHTTVNEKSNIYRDNDRKYNY
TTPKSFLEQIKLYENLLRSKKKELLGKMERLENGLEKLKATASQVDDLKGKLASQEVELKIKNENADKLI
IEVGIKTEIVEKERAFAEIEEEKVAVISKEVGIKRADCERDLAAAEPALLAAQGALDTLNKNNLTELKSF
GSPPPAVVNVVSAVMVLLAPGGKVPKDRSWKAAKSTSMANVAQFLDALVNYDKENIPDTCLKAIQPYLDD
PDFNPEFIKAKSSAGAGLCSWVVNIVSFYRIFCDVEPKRNALAQANADLAAATEKLEADATQSTISLANR
LVGGLASENVRWAESVALFKEQEKMLPGDVLLITAFVSYFGYFTKKYRVEMMNDMFLPFIGKLATPIPIT
PELDPISMLTDDADVASWNNEGLPADRMSKENATILCNCERWPLMVDPQLQGIKWIKKRYEGKQFQIVRL
GNRGYLETIERSVSNGDVVLIENLSEYTDPVLDPLLGRNTIKKGRYIKIGLEDQLLADVVRAERPDLEEL
KAELTTQQNQFKITLKKLEDDLLAPKITEQNINEAREHYRSAANRASLLYFILDDLNKIHPMYQFSLKAF
NVVFATSITRADKSEEVKARVNNLIDSVTFSVYVYATRGLFEKDKLTFTAQVAFQILLNKGEINPLELDF
LLRFPAKTDQGSPVDFLSNIGWGGLKTLSEMEEFRGLDRDIEGSAKRWKKFIESECPEKEKFPQEWKSKT
SLQKLCMMRCFRPDRMTYAVTEFIAEKLHSKYTENRSVPFATSYEETGPGTACFFILSPGVDPLKDVEAI
GKKIGFTFDKRNFHNVSLGQGQEIVAEQAMELAAKEGHWVILQNVHLSCSRESEFKSILNSVCYFHAVVC
ERRKFGPQGWNRPYPFNVGDLTISIDVLRNYLEVNSKVPWDDLRYLFGEIMYGYHAYIDETLPPESPYLY
GLHPNAEIGVLTKTSEELFRTLLEMQPKDSGGSGAGVMTMEETVKAMLDEIMEKLPEEFNLYEMQSKIEE
ITPYTVVAVQECERMNLLINEIRRSLKAVNLGLKGELTITPQMEALMNSFFIDVVPETWQKRAFPSMLGL
TAWYGDLLNRIRDLDAWSADFAMPNSLWLGGLFNPQSFLTAIMQSMARKNEWPLDKMALSVDVLKKSKDD
INAPPREGAYVHGLFMEGARWDTPAGCINESKLKELTPAMPVLFVKAVPIDRLETKNIYECPVYKTRDRG
PNYVWTFNLKTKESANKWVLGGVAILLAE

>E4YJ32_OIKDI
MLVYVGCPKFLSSRQEDELVSRRCQKRIKNLQICPLHARCVAFSLSSNSTAARPQAASIPRRMRR

>E4YJ34_OIKDI
MDGLLNLPGQEPTSGRKSGMQEFGMKLKGIKKSLRKTMTGESMRDDTLEETVEMNMDNWSMKSLDESVVG
WCSDRYDEKLAKYVKFVTDSMFFGLLINAVIVVNIIFLFLEISAKNDPIKLKFTIKPEKASLDTLSYYLI
ELADTDNFDYKKWLEDTDIYFFLSVYLVEFALKFYVELYGYFFAFSNLVDFFVLLVSFIQIALTTSDNSA
LGQVRFLKILRGLRALRTLKTLWMFGGAQVIIVSIFKSMGRTIKNIGVILILAIWIFAIMMYSTISLSNH
PEAVREDWGCFTACFSNLFIMTTADGWFGLTERAEKAVGSSSTSFASSSTFIRFCVGCALMMSHFIIFNL
FIAINIAQVDEANKEYMDSVNTEREEQLEIKKTKIIQRQYDDVKKLREQQDAKGCSFDELIENFKQGLKH
DDYTVTDGIVTDIDWIENHRNILDQLDTSTYKVQQLIFEYTNVLIVAQNEALKDKALNVFRTRMKGK

>E4YJ37_OIKDI
MNREAHQDQFTEADEDVMWSTCLFGVGAVVVLLVGSLAYKQISDNWQVIKMVLMLQIPLLLLYLYDQGYM
KFSPGRNSNYQQRSGSYWQSSRRN

>E4YJ38_OIKDI
MRLASFLFALSSADILGYSFNDNCPEEYRGRENACVRQCSSDYDFCKNNCSGNSFCENNCSRDMFDCGNS
CPCHTECFAGCIGCENQVCGVCFFPDENEDHNTCVLAADRSYVNCLTNCESTGLCNTQCLEAYQAEIRSC
PCGAECPNGCPCNNGYEGCPADTSLTIIGDSYFVLERVTLRLSNTDTVYKPTWEIPDRFVYDSGTALLKG
QQFILGGLTNLTQIAILKDCSVEMQSQKLEIGFSQHYGDMTILNEKSYLCFSTSVSKWARCETFDGETVE
VIEGRSDYGHYFGSLGHFENELYAFGGWNYSSPQSSTNYMEKKSLTGSWEEFGTFPSEVFIERAATVQVP
QGFLVIGGLTDAGTLSSIWLFDGKWVKIAIIIKFRFF

>E4YJ43_OIKDI
MAYYREYDKIYTKEAEGIHYLNFWMVIISWIFHITMILLPRFAWFNPDQGSGIFGEFNAMKGLFWECGTV
SDMNFLCYNIADSMFNIPTALLILQFMVCLAFVLLTIVIVLAFLGMEWSRAYEHDIEKKINIMRLCGALM
IIIGILTFGTVMWYGITIEREFRFPNMPDNILTVHTDVVRYEYGACVYLGFALSLIDFICAFIFIKYPGS
IEYEDEVESRIYEEKNDMPSYTVPALEYAAPTYNTRRFDKDDFREPADLKLGNLTSPKSTKSRFKSRNSR
RSQPGSIISKAPDFV

>E4YJ46_OIKDI
MTNPSGSAVREGAIYVEKPPSQKRNQYTLTYRYVKPDLTTGEGIYNMDLMGDLPPGLKIRAHIEGQNMLA
ESLAKNSPDYRYKDIFDQKIATPSEPSMIKGWNGWWTQEQFFVDLYATDPVKFPKLYVPDSPIFPREIRG
GLWQNTIMLNCNVSCLDHVFEMLELSVSKNLFIKIWYYRSSEETKRLETWTNMGDIEAPIFPNDSDVVVY
CLQCEMNYVLLLIWWFASAIMAKTEPSRHFSKNREFASLMIHCRVNEYETEWMPFAGSKTIAEISYHMLA
YAFALYEAVPEMITIQEQPKIKIIGLISLSQLMGENSAVMNRKVIDPSLDRNYENYYENYISHSIAYDAN
FQPVKKASGRHCATWELVTNPRKYNQATEPETMLMSIIRIYNEARTTWHQGGLGYEHASTREVLAHKSKI
FEKEVKKHVDIQSSPEYQEQVEKNKDLANVAKLHQLKILQSDEAHKALKLTELQASIAAMNEERNEMAHK
ITEMENKSKLEKQSLEEILKKKDAEGKAEREALTSQINALRTLVETYQASLEKDDIEMSEEPSSSASIAT
RSKVDANGRAKPPTRPNKRAQQDNGGTGASSLFTTTQTTARKDHMSGTFPSMNVRPPELSPSQIADSTET
PYQKPNFIDLTTTPDLLQMTNRSILNSLPGLPRKRRTELKNEILINYPKTPSDGNLTEDPPSSDSFIHQI
REMDNWPESDSETTNLTLCNENGESANSSRQGSFHEEPSTQDISIVKLDTSQESISPAKFSSLPIPREKS
GKQRKTEYTEILKSLKKSTPNSERKRNHEGKDLNSCSAKKRKITKSPPPIDKIIRKIKNKNGINESSPRD
TATPRKPIQLPTVVKMPEVVLPKIFGKKPKEKTNKVEQGERMKPVERYQITVSPIELTRSNRNITECTTD
SQATQRSKERDPKPKLKFDNEFIKLQRQFELDERSREQYMADLAEYKDLCNGSKTMKKNKDKKEENWNLF

GKGKAASQKNEQLSKQIMLLGGSMGIVDQWDYALAHEHEDIPRNVDKIMQKIKDCEAQKSELSAWFAHKD
HGVIKHSKKQYISALFASAFAGLTVIENIMDHRSNKRVIEYGFDTPESHKELKTFRRKMKELSKFATNGK
RVLSTWQSFPILLSGLIPVPPSLKIAKLVLDNFSLEQTYGEFPATSQNKTILRYLLRKLGIETIEKRYET
DVVRMVTNITGEYDKDHIPILIATKNSAKSKPQVQQNLKLIEETVLWLICINTELEPEQVNLNDLKTVRK
TKQGRVILTELAKISAYEPEGLGALLDIDFVNEFFSNRINIPVTKRQENKTTEAAQGGNQSRLDILEGRI
PKGPLEPDTTIPGPELFIKDVHGKQTMAINDIKARLGNKNRVSKKAEKKFKAQMKAQQTNPNNENEFAKK
ATTTGITNPSDQDIDLLFQTARTINENKANANEKIKILSTNPGKACSRTMREIADAEPEAHVYCLNELQI
KTETILDPGTWPPDHTVYSSKASSDGMSYTAIMIKNVLKHRVTPHSAPGNCTAIDIKINETITKRIVSTY
RHNNKDPPLCYYHKNWNKSKYIFVEWIREIVRRARQDKVQLVLCGDWNICLSKHRGQDDLQMVEGLKNAV
KSLVNLIMGNTHFRTWASPSEIDVFFVSHSTQATCTALNLHRPPCSYDGHTGHMVSMPFEKPLTQFEVKV
STIVEKDEMFKYMVDNYETHTNRLEKSENAEKKIETSYNILNEALETCSRKIGKIAQKGAGIFMPTPKDT
WKYRQAANMLADEWEKRKEDPEKEHDAKTHVLRINLIKVSVMCKKLHARDSKTRTDKIVGKVEACNPANF
WDTVNQLLAEPPVRELENNVKEHMKEVLALQQKTATDPSNYRGHTFKPLHKVKLSAFKTSLHGSKQKSSI
LKTFKSLKHYTKGHTGISRSTIDNLPIGCFRMFIYEPITLAIQEGHYPEKWRCNRTQILPKAKGIRPLSI
QEIFATIIEKLIIEQLNSFLENNNFMHTSQNGFRKNLSTSSSLATVINFIARRRSEGNYVVISALDARNA
FGSPPHESVIKCLSHAFEGKALKLLSESLARDAIVANKGIFSGREKLAPLGVPQGSTTSPTIFCLYAIEL
INSLDPHDPATKLSIFADDCILLTAGKTLNEAIERTEECYEKVGATMKSLGLSMVPEKTAILITGKEGCH
LHDKSSYPTHLTCQGNQVKIDRALTYLGTIIGEENRKLTYKYNIDAKVKKLSQTTNRARSLAGHIRRKDL
QEVQRALTLGSYCHNAEVTSKWPVAEHQRAQNSYIRGLGASKERAWFLKNYENFEETTTKMKIDTLTESG
HPTLFEAKISGFYGLMHRTLRESKAHDLLAEAKAGIKIIDTKSGHQIINMFNLYKTDRDLAKERYFKQEF
DIEPSVQTKEFKIAHHISAAFKILLARNIISIKIILPKNLSAEAGKIMWPYCLAKEFNELPQYLRGNVIN
KKHKEMLKGHLKRNHKHLTQGLSCGDCIEGKTNQLPFLVKNLTRNILDTEYKANLKKAVISEQEQQTEEL
RIKWVLDTISPIMEESTAGEEALLYWQEQVNWETDTLVTSLQKLFAVGVTFGAGLSTRIENELRHVVEKL
EDPFASTSFTITNLANGKQQYMNMYEMELWLVSKEAEGTYTVSNAHLIKPERLKRNSSRELQKVQIEQKM
FSTIFPNKDVFDTYAHKHFAGNKTKMAKWLYGENIAEIFNARFIHGETKNVWAETLEKLNKMGGNPSSLN
LNLNSSEKWKTCRDAFKTSPRLRNMRQMFYNEIQNTIAKQPIEKLDNFRICNTAYKICQNCGAEMHTFYA
LLDAMGWKACSTKDQQSIWRTIMDFAETEK

>E4YJ50_OIKDI
MSADALKNEGNELLKNNDLEGAIEKYTEAITINPSNKVFFSNRSAAYAKKSEYQKAHDDAVKAIELEPTW
PKGYSRKGAALVGLNRLEEAKIAYEESLKLDPNNAATKAEVESLKSKLSGPSGSQPMGNPFGGNPAEIFQ
KLATDPRTKEYMSDPSYMSMLQELSSNPANAMKHMADPRMQATLQVMFGINLGADADGNPTVNPTPENKA
NQPPPSEADPICEEKEEAKRAKAAGNDFYKKKDFENAIKNYKKAVELDPSEMIYVNNLATCYFEMAKSGK
VDNYDDCREYAQKAVDVGRENRADYKNIAKALKRIAMCFEKEKKFDDAIKWYNKSLSEHREKETLATVQK
LEKQAKEAKRVSYFDETKAQEAKDKGNELFKKGQFPDAIKAYEEGLKRTADGDSKLLSNRAGCYSKLMEF
HRAQKDCEEALKYKPDFVKCWIRKGAVLEAQKQLDNALESYRKAIELDPNAKEAQDGMNRVSSLKYAARN
DPEQVKARAMNDPEIQAIMGDPSMRMILEQMQQNPQAAMEHMKNPDIAQKIQKLVDCGLISVSSR

>E4YJ52_OIKDI
MALVSIAISIAEFIYMSSHMGAKGYKRRKTFNKEKNLLWTQDKTWNPYENPDYQQKLREHNMENGSLSRR
RMNSRSTSGYQTPTRRDRPFPPSKNNQHETSVLIETAPQRAKDKPHPLQP

>E4YJ56_OIKDI
MEATFDLLPDVSIEGTCPTGPSGSWGTKGEYSTTTAINNIDDFVKQWNISEQWLRCTDDLGDTDEEFSTL

HNYRVRFSIPTKTNPIPRASASVYFFIRVSKTEPANYPVEVVYQVEGNRLVFQPGRHIFREKWLHDVLRS
KQTMLEHVDF

>E4YJ60_OIKDI
MGGLDNEIGVYGRCGECNQIVHAELTAALVYCSGCGTTSNLNSLKNARESPVCAKLLQHTLEMSKSLELV
AFRGVHNFEAKLLSPFLSRYGMDRDGSPKLLQEMNLGEKFDCGLLANRHFRIKDKQKSVSGSGKDKTVYQ
SGYLDDTLAIVAEANRLPEALIAAHADPDGHCLVHAISRAISGTQLFWHPLRMSIKDHLQSNLLQYRRLM
VNYVEDSEWPVIIDEADPCYLSGGATLGLRNIHVFALANVLKRPIILLDSKNGMGSVGDYSAIFLPVLAG
PSKCRDKSNKPHPPIAIAWASPGRNHFIPILPKHNEQQIILPSTVLPPVWLVSQEDLQDYVTFKSTPSGA
KGIQLGGGIEFSDGYLLKLVSCMRRAFLTEEKVQTSMIIKLFRDVYRETDCTPANITANYKKAMAESRLF
ICHLCGSIGEVDLSDLQPLGDSYEMIKEQNGYLEDNMTYQLPNNMTGIYVAEHDQLQLVFESSHPECPYC
GTLTTSVKHKTMSLLDLKRRSASAAQRPTGKTTLSSESSSNISKKQKPIAETKSEVAGKSGNLRIKICLS
DGQTDVMEFSSDVNTSTFLSLISQKFQVPVSTLAQIRSGHPPKVMDQTKSNSPVEFSNGDRVQITLSSSP
ASNHVATPMPSSGARIPESLQNSTDSSFIPESVKNFWKEAQSNKKTFFNALLDDIGTGKFTSLFQKNGGY
AQLTENIKRTPLEEGQHYNLMNFQDVVFCWKDGEFQVCLGPDKHVPITDGFLYTVTTNDLKRNCISLNLP
RTSELGPGISRISGSSSAQIIDKTEELARRLAENRKS

>E4YJ61_OIKDI
MSDEKVTENEENSGWGGWASWATNAVTSAVEKVNNVAEESINAISDASEFMQRDLEEFAKVVKEDSTAIL
TAATEASYQSINHYAGEEIAKETASYTKAGVSILGSGLRTLVVGVEKVLLESDHESEDENQKSEIQSKNL
LPENKSNTNLEEPVYETNPQTYLRDPMDPNFDNWKVNFNKTIESRKNKLTEILIARTNVRLMYNKLVPKK
ANHNDFWARYLYRLELEGLVPDTEHVHAPDELELSTASMSALSETDKQESSSPESEPLEVVKKDEIKKIA
DSDEGDMDDWEKDLDLDITEEEMNKALAEVDDDWDLEEA

>E4YJ62_OIKDI
MTHEEPIGGTRGESPLTRFSEKLAVCIEESEPILIEEKPKEKMGDRHHMSTRGPNTSLYVRPIDGSTRPD
EIKLLFNEYGNVRDVHIPIDFNTRQPRGFAYIEFESIEDAEYAQKKLNRTRLGDKMLHVDFAQGDRKTPG
QMKTQKEATAIRLQIEEVKQMQTEAMKRAREAEMRREGIPIDKSDRDAYSDRRYTKREDRDDRRKDKSRD
KERSRSSRKDRDDRKDRDRGDRDDRRGRDDRDKRDRSRSRDRGSRRDRDDRRGRDDRDQGRDRRDRGDFK
MERSRSPRRDDRRDARGDRGVDGYSGQYNRGHDDRGHGGHDDRGFGSYGASSHYGHDDRGGNRPNYDP

>E4YJ63_OIKDI
MVDQSVANRELPKYDFNTDSDLCENQIGTVERAVCECDKMLTFTLQPYAMDLTYQRYKNVNFFRREAETN
CCEADGSVLDVGTCVNDIGIAYEEKVSIYRPEYVHKDAGDGTDNDGSSSSGSSGGTGSGPGTGSGNILDD
GTAEG

>E4YJ66_OIKDI
MESWTRIQNVESDISKMVSSMKKRDPDSMRLQSHSFRNSFSNDFESPRSSSPTRNRVSRSQTLPIQARRS
DIEDVRFRFTSVITNEVQSGFSAADWSSNFNIFKPPEVPKEPNNAPASRKLFQEAVREQNSHDSKEYELE
RIVSKINFSTSQFSDLTTCKTCNGDLKDAFCFIPCGHVICVDCAFCDHGGRCTRCWKDISSTNRVYLFG

>E4YJ67_OIKDI
MKLFQTISSIALVSAQAPPEPKNCADCIAFDNAYKDEVNKNPHLIDDYVNMLKKECASAGDLESICDTIA
GGAFKKYLEKIYHEDSKQYCTEITMCQNSIVRFYQQDDPDCTACLNFMTDLRLIARYDPPHFAEIFDAIA

KGICDETGKFESKICYHELKGHEENARKAIMNSNQQQVCVQLEYC

>E4YJ68_OIKDI
MEAECECFNGYESLEDRWSEGSGDDYACVLPCHKTIRDTEIICKTNRKVVKIPLCAIRENHIKPNHLFMG
NDQCSGTVNAADGTIDFEDGVNNCSMTEWVNATHINYSGLIYSQQGGNPGSVISYGHGLQLHHTCSFPKC
IHVDLDINVNIAVLNLNLGATGELTASVAVYDQPEMTSPVSHGKIFEGFDDVFVKIELDDTHDFLLSFQN
CEAYDDSISSRPTWPLINDGFAEGAQILQNYDNSISFNFKAFAFQDFNPNGPPDFGEEIKIQCTVCVCDP
NLDQNCIPGARRRRSIGEESKDFIVNTSFKRQLSTEIIFP

>E4YJ70_OIKDI
MQSSNAIVTPEINLTHIARIAYEQFGGLDVILADNGDNFKTKIIQSTDFSTDEKFEILEQLACVIKRSKD
ITQNVQASPNLILFPHQATVVSVISPFKNDTAGCQHEPSKVVQKRSSLFIQMNKSPVNFRQKIKLTLKGD
FKHIFNLISPKGQLKLVRGNCHSPNVLRTLTYKDFMRFELDKNDLRVALSLSLQEKQKEIGNLSDGEGLK
EDALVSLPRTVITALPIRSKVKMFPHVESSGENVKY

>E4YJ71_OIKDI
MCKQRTCKSIAEQATIEVQKNEIYHRSRLIRSQRKFLFYRAALSTYFLVWFITQNTAVELPKHYIYLTTW
GETLLNIYFTSAFGLSIYFYMCHEEVEKQQKGMIKIILWICELFRIFSWDVGIMLSIAYWVLLRTDLNPY
SWHCHLINSISIIADLIITDTQVKLYQFIWTIGIGCVFLLHSMILFFVSDRVDYQIIYEETLDWGNYPQR
SLLLCVIVVLFFIPIVHLGVYTIQRRRDRMLDRAGFKHLNPPVVIVSNHTHDSEGISISDISYAMPQ

>E4YJ73_OIKDI
MKLFGIILASGVTAQHWSYGFNPSGKKRSPDLAELDPEYPVKRMAAFNIHQFRRQLLPKAYYDGAQEALE
RYNIA

>E4YH51_OIKDI
MRREPRPRLEPKEQMLASMSGATITALTMTPFDVIKVRLQSGRLANGAKPRVIPFCNGLMDHMLCCQDPK
ECAHSSDPYRPATSKPWYLRAISCPVGESNPFRLMAHLARTEGIGSLWSGLPATMIMAFPATILYFTSYE
QFRDIFESLLPETSQKVAPFIGGAAARTLTTLIVSPMEMIRTRMQVDGLSWGATTSLFQQTFRAQGWRTL
GIGFSATLLRDVPFSALYFGIYETLKKQLPIESFHTKNIACASCAAAIAGILTLPFDVMKTRQQTMLGSD
MSRSPSISSIARLIREESGTRGFFRGFSPRLMKVVPACAIMMGSYEASKRYFSSAKYS

>E4YJ75_OIKDI
MLIQLKMKVFNFFFSLALANDEASYTEDVGGLFRTMEEHPERVSLSNIDGQVPQWLKGSMFRNGPGQYEY
GTDHFNHIFDPSAIIQKIQFDNGKVHYQSKYIDSTHRLANVEKNAIVYTEMGTWAEPERYEEMEDEELSM
ARCLHLNESFPSDNTIVSLYPIHGWMVGFTESRMVTLNDPETLETKHVLDLGKSAPEGYFIITVLAHGSL
DENGDFWTMSVGVQKESYAFMPKTAYGTLKIKNAMRDSENPFAYQATPEEFLASVEFGESLFNQDERDRT
VRYFHMFVQTTEYLVLPVTSIELDPAKMANACKEGQPPMDMMFYNEEKPGYFRIFDKKNMKWFPKKFTTE
AFMQIHMIQAYVNEEGSKIIFDTCETPKGDIMMAYYLETVNATGQALQEVHESMLPIGVPVRYEFGLDGL
NSKTEEYVEPTVLFEPDMENWPMYTTAGTDFPMINFDYIGVEYDHFWSVGMGNVMADRLYHSQISTGDRH
VWREVGYSPSEPFFVSRPGASSETDGVLLSLVSAFEGHSNLRPFMLILNPIEMTEIARIWLPEDYDISVS
FHGTFVKH

>E4YJ82_OIKDI

MWRNGEPRHTIRDIGALLEMAEEEALHTEKILREKQKRMSARESESGLKELNTGYGDSGKSLNGFYSILP
SNENQIRLASANLRKLWRKSNENTFVLEAFAASPSSSKNSSKNGKTLKELDNVYNKRLPARVVDFSAEYE
KDISSEAASRENKFRQAEQAYKKALAERAEQERIYEEHLQQKQMKERQKAKEEEAFAHAKQEADRATKQL
EKTPEESSVPPIQTESAAINTSGDPGRRFESFLTRKQIEDQIKLWEDSCRSLLHPKTDSDKQIAMKIKRL
IAVPIGALTQRSGEDLKIQIKKIGDLLDGKDLPNHPETEKYATLCASQRMVRLAEEQLSSNEKAACAAAA
AVIALWDYDSRFGQLFMAHLYLACPVLLPRDPQPQDIDETRMRSYVGYARLITCMASSDCAPGTNFHPFG
IENLWKILASILLLPTQNELGPHVVYEVLRYSGKRLAAYYNNSFSKLLAYIYNHYIPTATGGPAARLSSI
LDEVDQKGWQDPPGNLASNFWTTKDEHTGRIVDH

>E4YJ86_OIKDI
MSPSPKLSNCSTEHVNKDKNMNKSRMSDSDWSTDSEAESDSEVPLDLTAPKAIKLEVPVFDAKFHPEECL
VVAGDLEGRIQAWRIDEECSEAPKTHISVKAHADSIRGLCFAPSGKKLFSIGSDKQLLISDVETSSPILA
LQDAFDDAPYCISSTHEHIIATGDEAGVVRLFDIRKKNPIDELLIDDGRCEDTIKDVQMSEDGKYLIAIS
DDGTVSAYNCKRHMFLMESESMGTDLCSIATVKGETKTLVTTSSGQIQVYNYKGWGHPSDIIPGQGFHKN
TNAKLT

>E4YJ93_OIKDI
MTSLDSPTSEVNTFPYHAGKGQIMRSSIQAAESGDFIGEAIAAYDYKARSHKELGLQKGCRVFLKRKMRP
FATPGSKTPKTPPSELHSSQRNITLAQNAPQSPRSGPKKTQRFFFPSQFLPHVKS

>E4YJA0_OIKDI
MESYEESHGQSRTFNYPANRPEVDQNAQSLPVTDPTVQLSPDSLQKTALANDYDQKTAFHPAFFNIKHDP
QASLAAMASNYHNPSFFQACMNSYYSPTGLMPGPGQLEQWNFHDQLQIPAFHTSLRSALLASLRSATFKQ
TEHYKSHSSSDSEEENKTRRVTSLTSSHLKSVEELVKKSTSSRQHPLDVIDDAPHGKGLSKADLSANARK
KRRPYTKQQIAELEKEYMSSTYIALEKRQELGDRLNLSDRQVKVWFQNRRMKEKKLQRLVQRGQNNFYTQ
PTQMLNTAMDTFY

>E4YJA1_OIKDI
MSVAEFIKDFNEFFDDKFKIDQVIASIKNKISIEVDDTNKNVSKERSEISRKKDTIDRLKKGVEVLIAER
DAEKARAEYFETNKDLNSSLKNIASLLRVQNK

>E4YJA5_OIKDI
MTTPNAQSKPKTATFNPATGNKQKLPGHRATYSVRDAAKNGIKGAYVPPHMRSATKNNNNNKAEKTSQKE
GNTARKQLNKKSNTNSNYRNKWRQHDDRADDRSNSNSNFRNWTKKEQHKREKKEPKEIIKPHCELFLTNL
PPHMRNIGGLAAFFHPYGEVAQIQVIGINDDIPENVQKWCDVKQLVPKTHSAIVEFLTARTAKFVVGVLR
KRLGQLTFNVGLIKPGLGEELNFQKNTYGEIVHQPSSNLTQQYIVTKTVISDTSSDSSEVETNKPYKRVI
KRLVTANPARIDQAYWSQSSSQISSSDVSAVSSSESDYSDARAGSPISEAGSSCCKIPEDKPVDMITNKL
ALV

>E4YJA7_OIKDI
MRLPAIFFASTAVTAQFSSLDSTNKAADGAEIEETEFEGDLEFDEATDLQFDLILAEMSVRGRGRGGSNT
QQRINKQKKKKGKQLLRLLKYAEPERNSRDWLEYGCYCFLNIKEDILTPGIGKPIDPLDSACHALQECLK
CINIDHEESKRCHPYNGYKFTSAPGDEYNDMVCRDAENSCQRSTCECDREFIKLRVSEKYF

>E4YJA8_OIKDI
MVVRKEYRIPLPISLDEYNRAQIYTSIISERDFSKNGEGIERVQTIKEEEQTYVKRLYHVHSLMPSPIQK
VIKKDQSVLIEQGWLSYPLFKTTFTNTYMGENFLIEITTIHKDDLGESENIHDLSDDEWKNTEVVNIDIA
ENGPEEWDPKKIDLRKFGKANIEKGWIEKLKNEAHMEAGARFIVCSEAKNAQRSFASKFYVFLSLTRSCA
SRFYHRFAQPLLAKIKRIIEPQKAAHS

>E4YJB2_OIKDI
MTSNDDERAAKNRARRKSIDGNFIHEDLHEVMPSQNLLSEAFSMKPKVKKEKKRKTLSEMHAIRDRSDRS
VRRASVAPNFNYVVEEGILEDLCEIAGEEAEIGGEIMLGDDKSDLLKRISKNTATKDIFKNQDSYQSFVN
NKITGSGSSTLELATTTKESVTSGIASQPSTTICKSSSMDRKKSLAAVPKGKIATQIATQTNITSEEAAL
EIVEQNAKKEKSLFYKVKKAVLCYKS

>E4YJB6_OIKDI
MLIFCIFFQSIFGAEKNATIEFQIANTSKAAFSGELKIYVGKNCNTKVQKGKICDYSFDGSVTYAENKGL
TVNKGHYYEMTNPIEPSDDLIFMSYRNKDRAIFKGIRLRVETGIKKQTQIIELFNSKSGMSRRDKYLIFQ
YHNNYNSCYNPNGDRFPDNSRCIRDATFDLKEGTMSSTDSFLIGRNTGTQTNGNAGKGAKILLYTGFQSD
TRGNGLMFYHGFNCGEHICEYNKIGEIRASAQNTTYELDFNDFDVDNDQIIMDIRNGNIRVRDLKIKPCA
SCGKSDLLKEFSFSNTDGIWMRYIESERHYGSSYYDVIFDFKRNTALLDHSVFAGNATVVK

>E4YJC2_OIKDI
MGSGNSVPVDNSWKQFQQMSQFFNQNNQNQRGFNVHMPVHLDKIVDLSRSYSVASEDELARMAKKDIVVP
AVVSPADTGATAAQQEYSPMAPLRELELAMEPLATEIQEAVGGSGIGGPQAAAQVGGLDLEFDFSQKITL
PTPITVNWTADATGIGAGVSLSGFLAGLGGGAAVVCLVLMIMWKCFSRRSPARSDCHYVHVGGVDVRQQA
AQQRADIAALRPYEIHYF

>E4YJC3_OIKDI
MEKAMNMIKEEMDKKDREIECWKQQLQEKDVQINNMKRELEKKTGMIEKKKAKIMNFTQYVLDFNEELQN
EKKDDEPFANKDEKAVTDQETKTDEPTFWKDFLEIESSGSDTQQTAKPSNEYGGFVKYDNRRRRSLPRAA
RMRAKSTRGSTSGSESDQA

>E4YJC5_OIKDI
MDECDWTPPSAAEMEVMKQKRNRSDRVSSLMGQYMLKGYRMLDKYCPICSSILLQTPQAQGGTNYCVSCV
DVAAEEAAAEAKKQPKAAFVVPKSKPKATLVKSSQAVTPVVTKQETVKNVSSHDSTVTSNDNAMERVQQL
ISDKMVSLAEDLNNTTSATATRELLALIKECSETVTVVKNLC

>E4YJD4_OIKDI
MSPAGRESNHEENKYFKNIAHPAAHSIKALQHEEHEEQFLAHALDEDEEYEEDGNEQSHSHLETYHSFEI
IPNKTHLSGRR

>E4YJD7_OIKDI
MVLLALLAIISIANAEEDKIEIDFQKPIGIKEESLELNCRYRLPATSTLKEIYFILGEFSLDASDIIVKA
EGTSGPRHWYGDWEQRAELEHNEDLDGWRVALLRIAAAALEYDQAKFTCGVNAEDILSLSEKAIVTAEVQ
VRPDDLVFQHEIKEKTLFVSCGAENGKPFASSSIMIDESLGRTISGDGVVNASYQLTPAFDGKAVTCSVT
HPTYDDSSIVQDFKLGAPRPSSSCGQTQTEACETVESGELAIVAVEMTREGDECSFFVTSTSPTLQPPAK

LRWEGPEENPSFEFESVVGASVNFTTSAFDNTTAISVVAESILGASAVAFVFREHCLPIPTTTAAVSPDD
EAEMAGAIVAAIVLGSLAVVFIIAAAIYFTCFNKHEEAYRPNEKDSDLDDITSSVDLNEVAETKKTASVK
RSKSGTWNVPSGEENASLLGPSNSLRSAPKRTTSTQTLPESEKPKFASSAEKSLTIQKKHLYVCISTESL
LLFQCCRELVCNSCISQCMKMEYARDLICPLCERRPSPPRLVLPPSKFIQNAIDVSMLVCKVEGCNEIFP
FKDKENHRKRCPSEKCIHCEETYFASNKTKHLKECLPFLNNERRRLMIACDNLVAGLRDYNEYAEVTENI
SDLVL

>E4YJD8_OIKDI
MTHKFFFSQFDHGDLVFAKMRGFPYWPARIDCVRPRDCNVREQGNDPDSPDFCWPIFFFGTHQISWIPES
NLRVFEENRETLGKNKHIKEAMRESLANTAVKFQFGDGSGEIVPKMWDLGEAARRWFRFESDETTNITEW
DVHSRAKKRRLETPEPASRSSGSSSLSEQESSTENMSTEEEPLTSTPSLSEAGPSTIKSTESSSESEDDD
DPVITNYGDAIHKIADFNVGDFVGCMYESPYYGSVLSKNEDKRTVQIQFYTRSGQHKIYISKKKDTDVIT
EKRQGYIFCHIPSEKVQHGFTQVKKTKTMFLLDGVLHTDIDLRQARWELFTAEIREDEDDIVTCTAQVER
LEEDIDKLNRSTYCERSSFNVPIGCENHLNFSLNSTVSELPGLNDSQQDSGMDEANSQ

>E4YJE2_OIKDI
YLLYRTEIMTVAIENKDAFKSWLTETLSPICDADPKALAKYVLALIVKKDQSEKEIRKSCTDQLEVFLQR
DTERFIDKLFNAIKYKDYLKESPSKSSSRDRDRKRDRSPRRRSRSRERNDRRRRSRSRERRRRSRSRDRR
SRSRSPRRKSSDKVNTHRAQSRSPPSSKTSKTGQNGCAKKKECTDYHEKGYCLAGDQCTFDHGEDAISID
SAKPPPLPNMSLPPVPPNMPPPPHMIPPGMPPIPPFGLPPVPPGMPPFAPPNMPPPVVQGLTRTKNGTAP
GQISRTVLNPQQGCSLEVRKIPVHENTIQKLNDHFSKFGQITNIQVAFGHPENALVQFASPAMANQAYQS
PEPVLNNRFVRVFFYRPPPSQVQKGNIGQRVTRKRPNSDNAEESNTIDESDIGEIKRTVTNVTVEEPKIV
EEARNKQKEKRSEAVQKSIDLRNQYTGLLEGQQAEQKMLIKMLEKGNLGPKKRQVVLDTLKQLTNSIDQR
RSSIKALERDIELSERRQAKAELRRPRSQRFAQTATLDRRPRQLRITGFKESERDECITHFAQFGEVADI
SFEEEGVTMIVEYRTRQQAESAENRGKLFNGRQLVLVWHKQEDDREDQELEDIDDTLDLESTLDSVKADA
EAEEEVLAEN

>E4YJE4_OIKDI
MIEEGLQILLKCKNDLEVETIVENMSPSAKKEFPKTAALIDSPFSGLQLRLMLLAEVAQSAKNNNVDLVI
STELLSYAKLLLEQAVAFVISPALTINPEKDIISACIQLGFGRQSDNVTMYATRELGLEARQILLSFCDM
NPLADIFRDLCLIMSVEGAPFWCKIGCVKVIQRCIKQRGALTAFLCALLGEEGCLKSPKICEIKNLVGLA
GPLDGSEKFEEIKKILETPDRLQKNIALFLSIVVSQQKIYQMITFDENEAELSMRRIKVVLQHLPENTDI
IGLQELHKFVKSQTKIIYQYALALSNNVIETSNKQLLQDAVSVLSVMTSSEILSLLLYRADFSFDFDLTR
INPKPFSASVEIPIMAELVLKTSRECRLNILVDLIKFSFRSKDTELREAFCLLLSTFLESEPEFIIELMN
NEDNIPQILTVCELIFPHADDSDSRQFLLSIVALLLSDLVGYSLSTKKLVRERMAPLITQLIHSEENPEV
MRTAKDILTKVLTFGNTQNKEANEDEKKKEDEAKKKAGRRMPSNDKRMTLQTWSEYLDSREIPLRSGAIR
ELTAALRQNRLTEQQSDKLEKNCMAIVKMQTEPFLFLSAVQALTVFALKRREIFDNLVEFLKNQASQNPA
LAVQVGEVLSRACKELGDMAPCYASTLIPVLLSLAANKKLDDMVVSSAISAAADLLPLCKFLIQDIEHEI
GAAIVGFLSPSNEEGVRCAACLLAKQIFAVIGASFSTLVGGIVLDVYRALKKVVNDPLSSKWLYNDAALA
LESVDDAVKDYLTPSKKMEKKIQILD

>E4YJE6_OIKDI
MNLWRNMETERRKQQKDKGATNVIIFGGHKSGKSSVIRTLLNEDAPTECTVGLSYSYYRSKGDKNFNLWE
IGGELNVSGRNMALVPLEHSTNVVLVLLLDLATLSQIEDLVKKLGKFNAALKNKNVKLAIVANKYDQLRQ

>E4YJE8_OIKDI
MFRVFKNIQCLLISYSKRNTCSSKNKCHSFFRECKNVLASEDSNQSVIYDYALASLESSNKRSSLHEFLK
IINFVECEEKIIRLRDEHFKVVAKQMLEEKKLDELIHLLGGLEQCVKVFGGICGREMVKVLVRIFEEIDI
PPRAMCQALNAFLRRILTETDYEIWAEIEKLIKDGKYPDISTHLALYPHFHKFFAADVILRCVEKLISSS
AVERKESQNLFHQLIHNQNHPNGHQELAGCLEDKKEFLQLVMLLEENQQHIIDCQKGRFIRWLENKNCDR
LRLLPFSRSFNHENKKVVYSFCKLFLQNAKEESGNDEATRIFIINDFVPTTAQLTLFVDEETDENGLGIP
KMAEYMKSFIQANVDLLPVIIQALASFNQSPTPLFYWIYSISKERDLLSNSHNFKPTVLDNLVNIFYFRT
QKHQLKVKSATQSLIVSILGALKIDEDRQKLLAFKLSAEIGSPAYYSPANPLLNKKERQLCAFTSQLEKP
EDISASLKRHLDLSPKNQDVFTIITTAIELYRDRLHLMNAIDERVTLSVFELLIKFCEDWDTIKNFEGQT
KTHDALLSVCAHHAHLLNDQQKASVTVNINSIIARRKNKDPKFVLMALKTLKTLLVELGTKKKGDGFLNG
NDVFQLLDDSESRLKGTTELAWQCASYLNHEASVAECLEALSTCDITGQIAIARNLGKEGRVIDYNDEDI
SACMFTLWSEIQASFGNRHVHELYGHFCELLINKNSIANNVFEQSVIQPIFKEIQKKAITRHGMILPLLS
QYTAGIYCSFPNEHSYLIIALLTTGSAFNKDVLVDLHTAKIVDHLVEDGFIQKTVTDDDSVDVLKEMMQV
RRLGLQIIASLNVEKDANEKAVRLIHILIEEHAKESKRKPRYHLNSSSHLTKERCWQSILFVICNVLHEN
ELDVEEISDLVDAILKTLNPDEETSVRMFQSICLAKLIAIDSKQWQKFDEIIEADDHLGYGGAVSSVLVV
GMLFCIIVKCSQFTSKLIEQCIRLQQSNNFLIRIHAIATIKKLYSYCLTIPALKKKIDDVFPFLGRHVQF
SHSQVKSGNMGRNVERVMGNLLFSYCSDVIQDLSLTSIFRTIPLSVGGKEINLINEELLYELYEESFDKN
ATSSCFKFLSFETEIDRFYKLKTSKEDDKISRDLDDLSLDEPTNVQQKITPWIQDSLIDKKSCEGLVICA
SLIDKANNLGGITRTAEVFGAESLAISNLSILKSKDYTSLAVTAMKWIDIQEVKAINLQKWLLSMKMKGY
CLAGLEQTTNSISIEKFDFPKKCVVVLGNEREGIPTNIISLLDVVLEIPQKGIIRSLNVHVSAAISIWEY
TRGNL

>E4YJF3_OIKDI
MSDNFKTKEELSAFAKECKMNDFCHLLAKKIWRLRMKVKNLKKENKTLKLAVRRILDTDDKENPSELPNC
RICMKNHIEKIFNCGHTFCMQCACKMTDEGACPQCKTKITGFTTIFY

>A8PAW9_BRUMA
MARIATIRNQIGEISSDEALQNDLKILPSNYNFEIPKTIWKIRATKSKNVCLQFPEGLLLYSCVIADILR
KYTECEIVIMGDVTYGACCVGDQAARAFGCDLMVHYGHSCLIPIQETQGIEMLYIFVNIEMNVGHFIDVL
EANFEKHKKLALVSTIQFVPCLQSVKKELIGKGYNILIPQVKPLSPGEILGCTSPKLEEDVDAVIYLGDG
RFHLESVMIQNPSVVAYQYDPYSNWVQVACPRLSIDWGAQFKKPLLTPYELVTVLQYVSFRTDSYPMDYY
ANESLGPWTNNHKTHRECRPKRNHITISLQS

>A8PAX6_BRUMA
MTSLPSKSSAVSGNNSSGTYTWIPDSADNSCIAGIPMPPQNANIEVSTPRDDQTAAWEKAQEALKKVNVS
ATSTSSNISSMTSYHSQTQPDIHSQIMHYYPWMEQHSLGMSPFLPRLPPPPPPPPPSSSSSSLTSSLPPQ
PSQTYGITQENSIYVGMNYGFTPSGTHLQSANVNYHPDFMNPWASSNVTAKPRQFGFNGNRSTLVSGSGR
GEYGRNNTAPQHPIRFSINRPRGLNTAPAFHQNSQSFGLESPTIPDPVKRYVERSYMAVEKKEDRDKLEE
YFKQKLNPLLMSGAYKAVDWDREPLPSEVNFELKMGWTPASQLKKGLNAITEMEKRSLKKSKHEMSHRRD
SRSPTFREQSKRRVPFSPRVRLPSDEDNPVDEQPKFKQTAAQKKKKKQKKNSGKSGRWAADERSNAQREE
RARRFARDDEARRAKFTEMRRRLDWYIDREGEQGTSDNVVGTCMDIEKSYFRLTSAPEPSTVRPLKILEK
ALKLVQQKYATNRDYTYANDQLRSIRQDLMIQCIRTDFTVNVYETNARIALEQGDREEFNQCQSQLKLLY
KELPDSPNCHEFTSYRLLYYISVANTIDQTTLLSELDEKARKDPCLSFSLKTREAWALGNHVKLFRLYQE

APRMASYVMDLFLERERKAALNACLKSFRPTINITILASRLGLEEPKLCEWLATFGITVDDGKIDCRTYS
NTVLA

>E4YJF5_OIKDI
MGVPAFFRWLQRRCGSIVVNCVEEPQKEIDGVTVPIDLSGANPNGVEFDNLYIDMNGVIHPCSHPEDRPA
PRNEEEIFECIFDALDRMMNIVRPRKLLYLAIDGPAPRAKMNQQRSRRFRAAKESVQLSELMAEKKLEIE
RAGGRLPPPDPKKEKFDSNCITPGTEFMDRLAKALRFYISERLNKEAGWQELNVILSDASVPGEGEHKII
DYIRKQRNTPTHDPNTHHCIWGADADLIMLGLATHEPNFTIVREEFVYGKPKPCVLCGQCGHELIDCLGL
PEGVEDKEPPVFLKGNYIFIRIATVREWIAQELQIPNIPITWDLENIIDDWILLCFFVGNDFLPHLPSLS
IREGAIDKLVGIYKRTVCEMKSYMTKDGTVNLKAVEFVMRSIGRMEDAIFKKRRQEECQRRVRDERRKHQ
DRNKRLNRNQVHQTPVAKSGVMEPKMVNQHQRKAKAHDLNKTARQIRQEAAEARLTAAKLEDQDTEQITL
SALAKARAMQARQNDNSALDAEDEVRLFEDGWKDRYFRTKFDVSADDNDFKRAVVGAYTEGLCWVFAYYF
QGCLDWGWYYPYHYAPFASDFVHLDSVEINWQRDAKPRKPLEQLMCVFPAGSGEFLPPSWRKLMSDPDSQ
IIDMYPTDFVVDLNGKKFEWMGVALLPFLDEARLHRTLAKVYGDLTEDEKRRNGVGEDKLFVGRRNKIYG
KLRDVYETDKKEFHMAEEGADIEAKDIHGIQGIISIDAMCNPEDTFLQSPGDFCEDINNNFAISVTYNLP
EFPPGFVFPAKILPGAKFPESNLKPRGDRSNRAPQDWSKNNHRSGARVDQAGHRMLDHSMPQSHHQEKDT
SNDSRIGLFGIEY

>E4YJF6_OIKDI
MKRNLESKVEDFLSSGKDSYRFPAGMFNFERQYVHALCKKRGLISASHGSGEARRLTISRKQGREEKEIL
PQLIPDNAKHVIEALRNYQEKAWQNNKQPKLEPKFRLNFHSKPSLPNRQYNKPVYKLQAFRSALPIAEKK
TEIVRSIVNSKVTIIIGETGSGKTTQVPQFILEDAEERLRPTRIYFSQPRRLATITCASRIAEERGSVLG
KEIGYQIRLDNRISNDTNLILCTHGVLLRTLVGSKKENREKLSAMVSHVILDEIHERDKNADFLLIELRE
MLKSGELNRLVLMSATIDVNFFRNYFEKDGIQTEAVSVEGRTGYEKLFSRTIDNNLVKLLVADLWQKKEF
SNTGSILVFLPGYGEIKSLGLDLIEHFHKCNLTPLYDDESSVTPQCSSRNLFYLHSQINPRKQAEAFRVG
KKKIILSTNIAETSVTIPDVDFIVDCGKQRMKSFEPGSAVSMWLTTLISKSSARQRAGRCGRLPGTIGTC
YHLFSRKTFDEIMLNEQAPELVRIPIPEICLSAKMLGGDMKIEEFLSKAPDPPTQKTICNAVEELKALGA
IEQNEDLTPLGRLLVDMPLDPRLGKAVLASVLLRCVDPVITIVTSLGYRDPFVIASNGDDNHLTKKFKSA
MSGGLQSDHHLILQAFEAWNSGCKGNWPIHRILSMPTLQYIKDVRRTIFDHLKACNLQPSNDNSHDWNLV
TACLSIGSFPNVGRMTNPKSTGRGRKQFTVISANQRKLFQVAKASMYVENFQISESNHWVIFDEMFQIAR
GAVPNLKTVTAVPVLPIALFGGIDRPMIKSDEGEVYLQWNQVNLKLSCASHQVAAKFAHLHKDWNAFFNY
ALETRNTRDDGLVRVIQMLLSSDGNIRQAPNSNYSGARNESRSNGRYFRGAFNRGRGRGRGRGAPRGAAA
NALSDSAANYLMVNELNLCHMRGRGHQECLEHIRGQFESLHGVTVTCGFTTDGKNVAGMAFWFSSIENYG
AISCGELTYSNINTAALKRASDDAAAYGSPCYYEHGLKFNTCASMMISQRANEWCPTSDSHNIIVSDPAY
IYVSGYAFDFTYPGGQYGIAYCISR

>E4YJF7_OIKDI
MPLQFKLFVGVIFGTTFSVLYNKVWRAYFPLEQVETSLVCDQGTLWNPETSKCVSVQNQQSPVVALISYF
SDVYNEIIRPEEQAVVPYCNDGFVLNTETNVCDEIETPIDVVAKWLLEKHQTFVLPFTGDDHVSNFNALV
ALACAVFVGVSFMILPLFSGLAKLAYRVTGHEDEETSNTDENDNENEPLGEHYQKMAQEFLDGSVNTVGA
GDAPEL

>E4YJG1_OIKDI
MKLFNIVLATASAMGMGKLNPQAMGKLNPDDHEMGLVVPESQATGLIVPEILVPEDQPAIKIAHSCEWDY

QGENNRLINCEADEALDILDAWYGRENSSTCNVARGGGILYNAPGACRRDAKAVIEGRCNGQRACALAFG
NSFVGDPCVGTTKYLEVYYRCVKIAVPQQAPEIHIDEPKKMM

>E4YJG2_OIKDI
MHNPIIEHKQIECGCVMSIAYKLPYSPRPVFKPCNPCTDRMFSTEEHFKQSENLRKKMVLQPKTFTPLEI
VPEKQEKKIDEDDSNFELIQSTVEKIDAQERGPATMIVENSEGSFTFSIESQTVLEDEITIMRECISIHV
GQAGVQIGNACWELYCLEHGISPDGKMPGDNSVGVTDDAFNTFFSETGQGKHVPRAIFCDLEPTVVDEVR
RGDYKDLFHPEQMISGKEDAANNYARGHYTIGKEMIDAVIDRTRRLADNCSGLQGFLIFHSFGGGTGSGF
SSLLMERLSVDYGKKSKLEFAIYPAPHISSAVVEPYNSILTTHTTLEHSDCAFMVDNEAIYDICRKNLDI
ERPSYKNLNRLISQIVSSITASLRFDGALNVDLTEFQTNLVPYPRIHFPLATYAPVVGRAKAAHEQHTVM
EITGACFDPANQLVKCDPRSGKYMACCLLYRGDVVPKDVNAAIAHIKTKRTIQFVDWCPTGFKVGINYQP
PMTVPSGDLAKVRRAVCMLSNTTAIAEAWARLDHKFDLMYSKRAFVHWYVGEGMEEGEFAEAREDMAALE
KDYEEVGIDSVDEASRHEEDSIY

>E4YJG8_OIKDI
MKSAMVLLLNVDGLGMFENYMSLLNAKTQMEHVNSEFALGLDAADRAEQLLTVNSVSPQHGQTSVPSFFT
SGLVSPSSANKFYMDGMRNQALAGIQSLSPYTDLLFAYAQHQKNSRSRHGYHF

>E4YJH3_OIKDI
MWQLIVVSLFWGITNPFLKSGGSKLDEKMGIMQRTISLYSNLSFFIPFVVNQCGSLIYYYSLGLFPISLA
IPLVNSCTLFLTLSGLQLFYFSYIISDL

>E4YJH4_OIKDI
MDLSQFRWNAPRKKAGGPEKSRSTSRRRSSSPKKQHSRSRSRERGKYSDDQNTSENSRAGSPAKKRKKEK
RGRKSKRSRKSNKYRNDANSEDEVQPKERDESEPFGLGEGPMWTAPRGRGGPRGGRWPGADFPRGRGRGR
GLRGRGYYRPNYIAIGRNIFPGGTSDEDESSSSSSRSRSRRRSRKHKSRKSEKRGRRRRKKSYSSISSRS
TTPDDMKNTPEHKKGRSPTPEPSKEVGLLDKLKLKESQDDVSLDRVQDIMQQLASGATEIRRGQFVQSFS
NQDIFDNNETPILRKEMIDAPLQPSFPQIRSVAQPRLFEDLPNHLLDPNLDRGLGLRKQMQETENQQRQK
AIEENIQKQAEMDKRIKSRVVALNPEEVIAAQMKADKMASSFMARKRSAANIRAVADIKEEPDAPPKAVT
LPPLKTESIDDDAEFLKNLNKQFNNERGMKIMPINTKMSFAQLTTNASRKGKLTIGGTKGLVSKFQAPKI
LGAKPRLNTSSTETPESLQTQGQLSPVVQPDNLGNHETPFSTDILSNAPLLDPQNIGPKRPAIKIKPTSR
RATSGIVLPSAITSVKSEPVENSPNFESHVPQATTPTLSIKTDPSLYSSSKLNAGSSGFKIAPPTSNLPT
SIPSMGPKDSLESSKNPLDRPPPPVIRRDVSAPAVLPSSTPKTPFEFSAKKPAVEIDFTKPPPPFSIPPP
SFRAGIPFSTFIPPPSLAAPPILPTGSASQPQIYSSNAPPPLFPPVLSLSRSEPEMEAVRENPKFKDVYI
LYPSIWLGFVGMKSKAAMIQLHHIDGNQHIASRLLTKSLVHPSSDGSKHPIIKIKSRMKLDGKAIDTIKR
KWTDIPYKCVTMLALPYGVDPDDTEKQKQCLENHFSKYFAGKNCCGNLQINHGDQDWHIYVFPPCEFMNS
SLVEKASDLANAVEKLPKLMITIIPK

>E4YJI6_OIKDI
MKLSTVVISSVASQAALNRNPNGKEDIVYTPLQAEIFELNGLGSIPEILINRDSNEADELTPKILEKQFG
SNFRAELDRQKENIKKNGKGFINGVTIDGAFGGQWRIIRDINGIWHVPMFFGEGEVWADKSRNIPFWGDQ
ANVVMSGKDMIRKIQQEFERASCLTIDVFDDEAAVLSYFDSLDSDAMYDTANGAFIHPSFASTGTLSRLK
AINGSGCWSYVGKVFGTNQEISFVDEWCAGGVEPGLHEMMHALGFHHEQSRYDRDSKIWIDENVCGNAGL
FSALLYKAPRSAWPDMLNQWGREHDYTSIMHYESWVCSASGTGHTVDGSTNSADGKDWKRYGFLTNTGEY

LPTESSLDKRFEMSDIAQINEIYNCNDRSNDHHFKTFMLYCDNNPELAYPVGWRCDGSCDCSDDSSDCSD
ESNCNIVQKSGTGGTLGGGVGSLATGDWINLDQLKAMSVGVRTDRDGTVGYPCSKYGYRIVDDGSGIKCE
CAPWAGNGSQWGADVDLTIEGNNCDSVNPDQCTNECSSVTGANCSLDNTKFNGVDCCTDTATEYNIVNGA
CVERNRCEDGTHSCMSEAYGGVCTWTTDVGYTCGCRRGYNGQGCSYRAFQENEGGCDSTWEDILVGVQVS
EFPYYSTCRTNVAVCSDTGSQGHDCVATASCTMIEERYSKGLVGGTYTCTCPTGETGDGKLSGTGCSGDV
CFNDPTLCPTLDNLGNTISTCVANADGTHACICPDTHTGDASVDGSCILKIDECVEGTHDCGANQICIDR
ESLFTCDCFVNFETYGFSKTGSYENGDLVCTDVDECSTLTMPCAQRNSVCTNQVPSECDVSQDVGQCTSF
LNFFDPAYGGQNCPINAFCNTHTCACSAGFVSTDMIMFLDQGSVTFTGIDCDTPEEYNNCAVTGDGTGCVD
PFCDNQCSDAPCVDTGALSYNSELDGYARGYICDCRSIDMYGDGIGGCTAAACPANSSSIDTDSTDTVFH
SNSFPSVVEDWACACDEGFILSESDLGDPKDPFCIPDPCVALACDTATSECSRISDTEAVCSCLSGFRAV
NGETNVCEDIDECSEGAHNCPTVTTACVNNAGSFECGKPILSIFYTLYLFVLLLHIFILLNSLYIKNYKH
KNIIVPDSSFKALFLYQFQLIYFYTLQKIYFHTKMFSSYIQIIYSAYGFFHY

>E4YJI8_OIKDI
MKKLVSSIIYYSWIIVLLYSCLLQSRRARNRYSSRDLLTKSGRDNSDEGLNYFLFEKIYLQVPLLFLQLL
CQFLFPKRCRFDFMALGSIIILAVLVQFRTIILIASWSLVFFLFQAEAKLIRAIAALLSISFTGYLKSGF
KIRRIFFWSGAERWSGSREKRWSGAERWSGNLAKF

>E4YJJ2_OIKDI
MLLLVLLSLIETGQVAALRFPPILFTTTIPPIFSQQQDDSLMNYVKNQDVNTFCKNSDSNGADLEDNDFV
PIVPGIIRMLDENLNNLLMDRDFQALSLLDVSKIAKEFLAKFDTWCIELVHVPLPPDQYEGSSVNWDFSH
GGVMKMLRSIGINPQVDRLIAMMNIFKDFMLSFGPLLETMKPGLEKRLKMMNFTELQNLPIPKDDDELSS
SLCENQIVPFIQIFQKLLIPLLDDMTDAFRGNENLLSIGKMFQFYYRLFKEFLFHEKICKMAYNNALPMV
VDVDGRMQRKMASDSESTTELTYITNTSCFVGEFVKTLPPSDEEEPLIDDMKRMIKMAVIMVKLAPKMVI
TMKSIIEIMGGIPNPNPYEEKSEIGYLIANISNVVLFAPDEVIEFFTSQLATELSLSIEMLDNDSCAETP
SGLMGKELGISAHGKFLTLNIFVLLISLKIII

>E4YJJ8_OIKDI
MVPTYHLNVPSDFVADKRHGPNLKIIYDEEFQPESSSKSAQPMTSRVRSYTIGEVFVFYEKPEFAKRAVE
IFNEQKWSRFLPMMKTEKLHNKAFRIKNSEIRARIPTSTSDIEKCDSIWKFFDVKNPPDRAVQRAIYQES
LPVLEDHVKRAENGDPYKFLEKITNDLANGQNLHAAIVFVAKLLQIIHTSFDLSVSSQSETNIAFLTIHE
TKDLRELLQKQAVVQVLEGKEQFVRRSDLEKILSRQDLLE

>E4YJJ9_OIKDI
MSRKEFTSASLADIKDMLATPDDGRFREYIRNLDNYDPFSESYPPENRDFYQILGVSRTASIDEINDAST
AFNDPQKSRKEKHEIVTALYALKTPGVRKIYDQMGELGLIGYGKLLSQLGGRHVDDDEHGTMGQDLKLFQ
LMNIMKHSESQMP

>E4YJK2_OIKDI
MDGVDSFYEEVDDYYGSLNFFTNEPKDNRDDSRRIYHYSSLVWKDTSKLGIGIAKFTGYPAKNKIKQHYV
VVYRYSSAGNIKGSFGRKVAFPSKSLLDLRRIITDHKAMQNTLLAEEQLKDGFLSERGIKQWQIKGMKVF
NWIREKQFLPPVVLDLDLCDKNREFEYVYSHSLSTSSQQTDEQLEVFVEDFLKNHGSDVSLSDKKGGFYR
LRFSKIGFYVERTENKTKITYRLK

>E4YJK5_OIKDI
MGDEARDQLQKDLNSYQAIAKKLCNLTEDDDKALLTKTMVKLVHYLRSIDQEDTTRRDALAVAIHCIGKS
CFMESEDLVVRASTAGAMVHAFRIYVPNFPPFCLTDMATTDEERVLDSLNFLVDLIPHVTDEESGKKIGY
ALEYINTIQLFNCVFKLEGEDIGENEFIDKAIKYIIECKIKDAGKRKVLSNILRELILVSEHEDFNVIDN
MLSYLISGVANYNKNAAQIVKDAFVDTGKHFAVVLSLFFNTMFVQFDKIPIKIANKWHEVLITLFKIDTN
LISEDTLGYLHDLVHSTTTKITERSKAITCLCELYLSPEAAKMVELKDAIAKTLGRPVEDAKVRELIVKK
CSDILLKNGLDENILKIFLNFYNDTKDGVREKVAERLQAIICSSIDFVTEETVNILGMLTRDRVSIKVRL
AAIRSIATTFKTHRAHSDLPLLKQLLRSLLEVYWAFARSKQAKDLTELEESGRIEIMMAFQTLIVGGDSV
EPEERAVNLSRMWLDEDGHFEKFIRSMFIDLYAMRKPFERTLELIADETVEEGRIEESFRSTIDYTCVKA
KDSMNESIMKVMLQNENLLDIGRKITYLEMPVKSRIKNQKKFIDAIRTADSSINLTAISELCQRSTDCVM
EPKVIIKLFKLLSESTDERKKLRTMNLINYFSNTHEYSFANLDVFEIVISFLEKKECTKEMLILLKNIGG
LLSEVYQGRKDAEGVEDEDLDLSILKTLSSYLEEIIEGDDNKLSKLAIAAMISVGNGGDITIKLTQKILQ
KLTGNAIRKIKSCLNAGEYDDEFNNANLVLGCVMADTSSASRGQFLHIFEDYIIGNKLSMPETNAAAFIK
CCERYMRFILEERTANCERLVHFISSQLRENRAEAKAAASSALFRLSSFKAVRTCLTGDDIRQICFQLAS
KKEKALKHIENKDVPLMWVSILIILEFENDKVWVAKIQQIARIYREQSSDELANEAESVLCYVIDILQMS
FTLEELKNPEGQKKLEEIQDRLRVFISAFTSPPAYGLRKTENDRKKLWAKLSRSFGILHGIAQVCRQHLF
TVGCTGGLKVTQKTYCVCEIVMQEILRQSAESKMSIDPISPTAILKLFSNKLFQLAKLNGKLIPADKSVH
MPEYLTYATQGSSAGKRKQPKDIAKIKLDDHQSDEDNDDHVEEKIIPKETKTRSGRAIRRRIIDGTEEDE
KKKSELASQNTSNITTDSDISTRSQASSRNSSRSRSRSPIKKATPKVKKTVKXXXXXXXXXXXXXXXXXX
XXXXXXXXXXXXXXXXXXXXXXXXXXXXXXXXXXXXXXXXXXXXXXXXXXXXXXXXXXXXXXXXXXXXXX
XXXXXXXXXXXXXXXXXXXXXXXXXXXXXXXXXXXXXXXXXXXXXXXXXXXXXXXXXXXXXXXXXXXXXX
XXXXXXXXXXXXXXXXXXXXXXXXXXXXXXXXXXXXXXXXXXXXXXXXXXXXXXXXXXXXXXXXXXXXXX
XXXXXXXXXXXXXXXXXXXXXXXXXXXXXXXXXXXXXXXXXXXXXXXXXXXXXXXXXXXXXXXXXXXXXX
XXXXXXXXXXXXXXXXXXXXXXXXXXXXXXXXXXXXXXXXXXXXXXXXXXXXXXXXXXXXXXXXXXXXXX
XXXXXXXXXXXXXXXXXXXXXXXXXXXXXXXXXXXXXXXXXXXXXXXXXXXXXXXXXXXXXXXXXXXXXX
XXXXXXXXXXXXXXXXXXXXXXXXXXXXXXXXXXXXXXXXXXXXXXXXXXXXXXXXXXXXXXXXXXXXXX
XXXXXXXXXXXXXXXXXXXXXXXXXXXXXXXXXXXXXXXXXXXXXXXXXXXXXXXXXXXXXXXXXXXXXX
XXXXXXXXXXXXXXXXXXXXXXXXXXXXXXXXXXXXXXXXXXXXXXXXXXXXXXXXXXXXXXXXXXXXXX
XXXXXXXXXXXXXXXXXXXXXXXXXXXXXXXXXXXXXXXXXXXXXXXXXXXXXXXXXXXXXXXXXXXXXX
XXXXXXXXXXXXXXXXXXXXXXXXXXXXXXXXXXXXXXXXXXXXXXXXXXXXXXXXXXXXXXXXXXXXXX
XXXXXXXXXXXXXXXXXXXXXXXXXXXXXXXXXXXXXXXXXXXXXXXXXXXXXXXXXXXXXXXXXXXXXX
XXXXXXXXXXXXXXXXXXXXXXXXXXXXXXXXXXXXXXXXXXXXXXXXXXXXXXXXXXXXXXXXXXXXXX
XXXXXXXXXXXXXXXXXXXXXXXXXXXXXXXXXXXFDAQTIFMNRL

>E4YJK8_OIKDI
MKLPTFLAIYASGSEAAGRWTGERPELSKEDADSFLKRYTRMNSVPGAADDSLLEEMRTGNLERECNEET
CDWAEAYEIFGDKTQTDDFMYNRLHQCETKNPCFNLGTASCQNKWDSYWCQCLSGWYGKDCDFMDTKGRY
SLLYINLIKNSDGYMCQRATGCSSPLIAAPQIVAKDDPPPTTSTTTSSTTTTTLRSQKAIALPAYEPSVE
CDTINDKFTVRMPLSYDGDFLLGWDMMTANCGFTSEDPQGVVYSYKFTECGTQMKLDENDANKFIYENNL
SRKPVLANGVYRDYGAIFNFRCIIDRLGHVDNDFVNATTGEILGDIMPVYVVSDNMISAMGQYENNDDFI
FRLNVYSDATFSSRFKMQEFPLLKSLKERIFLGTEVITKMQHEYIFTKRCWATPSVDTESNIAYSPIIDN
GCPSDAFTSLQARSNHEDRFSTETLRFPESNYVYIQCDVIVCDLRKPNDKSRKTVHC

>E4YJL0_OIKDI

MKSIATLSALAAVGKAQYPFPSLTPSGDSWTLWSEWSGCSNIDIADFCAFPDNYLANSVRYRECRLPDDE
VHSWNAADTANTVQPPQGDPAMTLDFTGIPTAWTQGIEREQSSPSTRIACDPRLDDYMDNPPPGMPVYAD
VRDLEVAIATDPDMFQSYIPAPYKRIVFEFALCINVHKCPRPLPWSSWNCHCLGTGQANNDNLPLCNCGN
GQTKRSRVQLCKDWDRDDSVTCESVGESIANNPILGTTEFPNRCINTINTQVWTQEFYGNQAPKNWYQQI
LMGDSNSGWIQKNDAGHDDNLENTYQLSRNGQYSYTFFNSPNDGFSTSSGYINQCFDNSRFSSTLSGYVD
QFLMNYNDVRQDVEYGYQEMMCQSVEAAWSNWGAWGRCSQSCAGGITRRSRSCVSQGVVVDVLAGDEGCG
CPGSSFEDLACNTFCCPVWQICTGDDCGNDDWTPLTAAADGAFAFGACPECGDTTLELTRHCKCQEADGR
FRFGDLTTPAFSTTFANFQHDCVLADGETVENSFAGVPYIAKTSKVCDGPCCEGWQPWSEWGACDDSCFD
SNNGALDAPAAARTQTRNRSCGCDGTTEVATDFSNCPSLDGLSFLPAETETRACVATPSPCAYWAEWGAY
SACSATCRAVLYDGTAAVGARCNADPLDAGGLQVRERGCNFGDVGAAGCPEAGRTESQACVLSDCCQYDQ
WSLWSQCLTDVGESCSANGQPGKRTRQRNTVCGDPLICDNCNHVEDCELPKCPTWADWGMWSECSMSCGP
GTKSRTRDCCGDVIGSDACPCNGDFNSAVSQCDFGGNTCSNFYTTGTQLDLWNARVNAGAHDATDMGYID
IMSCEVKQVCPYLQKMENSNELMWTEWSTCSTSCGNGVERRQRVCNHGTLGVDPECSGLIHEDRPCSHLP
CPRVTEWQMWSDCSVTCGMDGERYRVRLCEKPANVTSDCIEPLDETVRCVARPCPMLTNWSQWTTCSEEC
DTRLGVTVRERTCEHGMIGEDGCDESAYQSQFCNQDKPCPSWGAWSSWTACSEVSQNIFAHSRTRNCENG
ALGSRECPVDGADQDAPCDTEFCAQTNIGTSENDVYIPRGSATGLLISFTSVVIALFL

>E4YJL1_OIKDI
MEVESGIINFYQFVEIAEREPEKLTGAAKRKKKITPRDRFRQRLVDLRRRMDEQKIIPEEKMAIYEKIFD
DYKQICDCYIKLENSDAFELKVAPDPNFVQAPPPEPKNKRRGRRKKQTRNEDEIILIEDDTPPPEVKTTR
KPPKERAPYEKVKRKYTKKTEEQLKKELDSSLEKMTIKNDIPEERKDDNDSQYSDYGEILARNSFGLLI

>E4YJM3_OIKDI
MESPLQLSVCSLGWFFFLRRAMSDCGKFDKLEKKKRTETINLAVSVFHSFFSGIGALIAITRMPEILEDM
VYAHSNLSACVTSFTFGYFVYDFFDLLAASRWSISKNNFDIILHHVLVICCFGSCVVQQRYMGLSMLSIL
MEVNSVFLHLRKISRNFDIRSGIVYHAIVALNFLTLIAFRIAVSYKMIVWLSTVDRSGIPTVVFYLGVIA
MPVIAAMNCTLFYRCLRVDILRPLSRPIKKD

>E4YJN5_OIKDI
MSILWIYVEANEIVNILTSFGVFWNVNTTIMGILILAPANSIGDFVADFGLSKIGKVETAMGAIYAGPLM
NVLIGVGLGCTIANLTNGRPVPLELTAIELSMAAAVELSLFMGIGVLIWYNFRFPKWVGPVFVASYLAVL
VITIVADTEVVESDPRMVCTAKRTDRVMVNFDGFCENFGFVKWLWEMCAFIVSLPSSPKLPTAVAASISY
FQRR

>E4YJP6_OIKDI
METEESSIGVQTIKLFGESVGITSIPEDAARHVAENLKFQLKRIIQDSAKFMRNAKRARLSPRDVDSALR
ARRIEPLYGFTSTDYLPWRFASGGGRELHFNEDREIDLQKFLENTASKLPPPIKIRAHWLVIDGVQPNIP
ENPAPARKPPHDLSAKKLPEDLGKEDKGKDKKNEKKPGDKGGQTQALEQKPLMRHELSIEQMKYYQEITQA
AVGRNEEIRKEALNSLAEDTGIHAMLPRFTNFISEGIKCNINENNLALIIYLMRMVKALLDNPTLSLDMY
LHEIIPVVISCVVSRQLCQRIGENHWALRQYAARVLAQISKNFTTTTSMLQTRIVQSLQKPLDRRDAALA
QIYGSIVGLSELGSDVTKKIIIPRLPKISERIENLQNDLQSDPIHSDGLEHILDQVRKRICPEMPSTHQP
DGVLEVDTLITEFGPYLGEKMYKRLLGAIEMR

>E4YJP7_OIKDI

MVLHKGAWRDALPKTRSTPFQKKYIALILFIIISGAILYGQSEAEINSIAEINYKNVVEDLDIAKLSKTD
RKSLLAKLLDEDPELQEIESWGEEFEDAIDDQEIFNEEVLEEEVEDESELSKEMMEDSDLDFALDLSHEE
VAYRFKIRKRIFDEECNLLNSPGPISTENLNFTLSSVTQVFYDDKYKVIMCAAPKAATTNWQKMMAVLKY
DGIYDTDHFRKSNVYNQLPRFSQLSLEHGEEEGKSFGYK

>E4YJP9_OIKDI
MVDVKHVEEMILEDVEAHRDFINAAKVVVLFLVQWISAGAMVFGGVVPYIPQYRTIARSGNTDGFSPMVC
FVLLMANSLRILFWFGKHFELPLLAQSFIMVIAMIAMVELCARVRSKQETATKEHNFLDFDLRYFWRWSR
FSDFMQCILAIWLTLSYVTWLFLGFAWYVETLGFLAVFIEAMIGAPQFLKNYQNKSTLGMSVHMVLMWTS
GDCFKTMYFILNSAPPQFWICGLIQIGVDVSILAQVWYYSIPKYTISYSKPRLAA

>E4YJQ5_OIKDI
MKYENLEGKKKFFVPGGSFLAIMRVFQMLVGCLAWFLPGIIIMENPQANPFFGSSVFYTFIISGVIAWLL
PCFKILSVVPWPISAGPESMVCFIAGFFSFLSGIAICIGQNDVQSYVLKIALVLNVIHSTILYGTPVVIC
SQSAFRFFFLSGADLETMQALNNMEARDRYERDMEHASIVKNYEVSEGNNGQSLSFTPKGFKKVVTTSEP
IFEDMNE

>E4YJR1_OIKDI
MKLSVAVISAALAARVPDAERRLAKINEKASELLDYANDSSASNKQKAALKKRVDRLLSQLDKIDTSACP
APGVDEDRFTVDDEFDGLCDGSGKIPSMMRSYARKFGCEAGYPNKKFLDRFINKSHKLKLRARKAGKCPG
SDPAPVDPTSGPTGTPVTNPPIADLCGENKYADCYDLTSGEIYFENQWTCRNCFRVKATYGQNTNFNAQF
NAARVSCDDPCDGRDNCDNLVDSCPITPEWSNIIGTVNGDAIYTASVDVFCHKSSGHNWQSIVHMTRGTS
RDQFGDRYFTVWQHADGRLKLNGPMKGNDFDEVEYYVDCEDKTWTTIMISQQEIDENMLELTMYQDGEAL
GSNIINAVNAYSGPITVYASNPYHPPATLFKVRNFWHESEPYVAPPCVPVDPCLGLDSCETYINNCMVAP
FAGNQVATTTGFEQFRISAEVRCTDRVAPTWENVLHVNTGTSRQNFGDRYFTIWKKHAPANNEIRIVAPV
FDNAYHELNGYVNCEEGWHSVAVQQTEFDNQQMNVEILFDGEVIESQLVRKQDLFNGSLNVEVASNFGWP
AASDHYVRNFIQQDLTCPNPCSGEENCETFVDACEINPSAENQISTIHASVNFKASIDIQCNHDMDFGAW
RNIFHMSAGGEHGNPGDRFFAAWRQPNDNQLYFGLGVPEGSYAHHKTVDCADGRWNTYSLEQRQDPDAPH
MVKLIFSKNDNELASYNYETAAVFTGSVDVFSSREDNDGNVASDFSVRNFFYQSFDYMPPISCDMSSAVD
PCDGLDDCTTFVDSCYVNPERQHNFGSIEATKNFRSSIDIQCNHNMDFGDWLNIFHMSAGGEHGNPGDRF
FAAWRKPNDHQLYFGLGVPEGSYVHHKTLECVDGEWNTYVLEQKQDPDAPAVVSLTFSMDGDDIASYNYE
SDTVLVDTNIDAFVSREDSVNNVAFKFQVRNFFHQTFADVVPEPCVSAIDPCDGKTDCTTFVNSCSVNPA
ASNNVGTIEATTNFRTSIDIQCNHNLAFGNWRNIFHMSGGGEHGNPGDRFFAAWRRPNDHQLYFALGYPE
GSYAHHKNVECVDGEWNRYVLEQRQDSKAPGMATLTFSMDGAEVASYDYASDDVLVDMNIDAFISRENSI
NKVGFQFDVRNFFHQSYDDVTEEECVGTTNPCDGKSSCTTFVNSCAANPSSANNLGTIEATVNFRTSIDI
QCNHNMAFGNWRNIFHMSGGGEHGNPGDRFFAAWRRPNDHRLYFALGVPVSSYAQHKNVECIDGEWNTYV
LEQRQDAETVTLKFSVDGNEISSDNYPSNDVLVDTNIDAFSSRESDNNKVAWQFEVRNFFHEDLS

>E4YJR2_OIKDI
MNRLSRVAVVGAGAGGLCTASRFKQLGVEKIKIFESASRLGGTWRYVDDPKDDPCSSMYKNLLTNLPTKV
MNFPDFPFPKNTDAFPSHTVILKYLEEYARRQNLNESINFDNPVETCSFDESTKSWKVNDENFDFVVVAN
GHYTKPSVPEIFQNSVFEGEIMHTHYYRKAESLAGKNVLVIGQGPSGQDISLDLLGIANSVALLGRSEIK
GTPDSLRKFIGWAKEMKANGIFTNNNELIECDYILLASGYCFDFPFLDKNLIEYSACKKKIQPLYKQIVH
SRYPSLAFIGIPCTIVPFPLMDCQGFFQTAYSSAYVF

>E4YJR5_OIKDI
MDRRLAFSNLQFLLSLSFWKLGLLWVASIKQMRFCRAQISKLLVSKRTTCLDAFSPSSDYFQFQTSE

>E4YJR7_OIKDI
MTSKEDILKELPADVLSAKVSAEDLLKFSPVLQQEQQKHLQRQRQSAGEVEYDDRPKTGIFEYAKKNPFA
IGGIFGAVYCFCKGVSNTHQQGSHKADMKWMQGRLYWQSFAIVSMLGGHYYYSMKHMEKLEVKRAQYERE
RDIVCKNAHISPEVYEEMKYRESVERNYGGNDMIPLQELKRRKQVDQDFAERGETVYLSPEKRIAIENSS
IGASLSSTDFMRLGIRSPSS

>E4YJR8_OIKDI
MAGTPTSNGERALHRSPEHPPRSHQPRLWQAQRLRHDHDRSCSVFSRLRRDLLLVHRWRVHRQGRHRRLL
GLS

>E4YJT4_OIKDI
MKIAGSLLSLLGLVLAEQAYWDDNPGFLKRSHSLIPPHSGNLPLWELKGDTMATSEYIRLTPDQQSKTGG
LWSRVPITFPWWELQLAYKIHGSGKSIAADGLGMFIIKERGKFGSALGGPSQFTGFAAFMDSYKNGQTTG
NFPQISGFVNDGSWSYNHDNDGADQNIGKCLSGHRNKKDSSLLRIRYFEDRLTVKVDPDGTGDFKKTCFE
VDGVKLPTGLYVGFTSATGDLTDNHDVVSFKIWQLDSKTDAGKRHEMKPEVLNLAPKEAVKEESSSGGGW
GFFTFLLVLIIGGGVGFYYYQEREKASAKRFY

>E4YJT6_OIKDI
MSPGGDDSSVKVAIRIRPQLAREQIEGSRICTFVTTGEPQITLGKDKQFTYDCVCDLDNSQEEVYSGTVK
NLVEGCFDGFNATVIAYGQTGAGKTHTMGTGFELNTQSEQEGMIPRALRQIFNGVQSRQAQAIKEDSIKP
EFYITVQFMEIYNEDIHDLFNPEGSDKIRIHEKEGAIVVDGLKALDIKSYEDTMRLLQTGALNRTTASTK
MNTQSSRSHAVFTLNIKQTRAAPCNPDEKENAKKIDLETVSSKFHFVDLAGSERLKRTGATGDRAKEGIS
INQGLLALGNVISALGDASQKRKHVPYRDSKITRLLQDSLGGNSRTIMIACISPSDSDFMETLNTLKYAN
RARNIQNKITQNQDSSSKQLALLRTQLSQALSELALFKTNGGGDSSSSVDIAGYNDMFQENYLLQEEIKK
LKEKIRELQDTVDRQKERLIEKAMQMENVDSTDEEGRKVVADYLAQIEDLKNRLTQAEIRNARPRIGSAR
ETATLNTQNTGSDTFSLSFLNYKLDDPKNRIQKSSLNGQNTTLSFVKLETCFKQALSCPRETRTMMKMSS
MRNEIDLKEQLVMQLEATMANLENQRKKYENQLSILETKIQEAEKERDLAMAKQKNKTDKDPKSKEKIRE
MEQKLSLLRTDLKQLQQQKIENEKLSRREEAKSRELSDAKNKLQDMKRQKVDLIKKMRDEAKLFREKEMK
TQKELHKLKKSMRLTETNAAAANMKQKSTEDKLHRSEKAVNELKLQEREQKYKIHKTHVRLFCKRQSTPP
LSFSLKNVKNAASKNALPTKSPRNGLRQQPGNIRPQTRSRASFNKMWSHTQQKIRSAIAAKETQKDVENE
LEKLLAERKALEDDKACSKTDLTNKPLQVDVENASRSQLIYLINQMVPFFVSANANINQEKRQMTANYQK
VQNELDSKESAFAMQKQLISHLMDKKTSKSEPLPTPRTTSKKKRTTTSLSSALGLTPSIETGDESTDRTP
SPTSTISSNETVSSKDTSSSSKLSSRFGSVFSRSFSSKVSVERSATGKFLPSHSPEVNDNTVGLKCTHAL
VGHQKSIITIVASGEFLFTSGRDKLIKMWDLVDDKEILQYQMPSAVELLAFDAKMRVLYTVDGGKISAWD
TETGSEKPLKVITESKSIQFVEIDECGRLWVAVDKTVKCYVNYSDTGIRCASPSEITALSVRTVSELEVA
VAVGGRDNSTRIFRHSFELPADSISPKITLSPHLDTVTAIVQFPDFIITGSRDNSLKLWSSIGDLIKTFS
MAHADWILSMKTIPGRDAFVSIARDGSLAIWSTRLEKIGHITAPDAVAVRSIGTSRTHVFTGSPVKKSGK
SWLICF

>E4YJT8_OIKDI

MLISGERIREIAARELERHVQDPDFKSVLKELVQQVEDAIKDDDLWILEIVKQLEEIQIIQQNQFYGERA
NLFRQQTVFLKKEYEKHENLLSREQSQVENXXXXXXXXXXXXXXXXXXXXXXXXXXXXXXXXXXXXXXXX
XXXXXXXXXXXXXXXXXXXXXXXXXXXXXXXXXXXXXXXAQTRPGPARTGHFQCPRKF

>E4YJU5_OIKDI
MTSYAYYVPICLYRLSITTQTGTESDVAAESFARNDVCHFPREPKYGSRNFRISRICLHQLGLIWISRGL
RVNILLLLQLRNTAPSQATSSN

>E4YJU7_OIKDI
MTIKLKKLFKWLRKGGAKEQETADITKSDDPAKSEELVLCSLPVHAQFTISPSLSRAPSLSAVQNNAALH
TSAPRKRVMRHTSDVSHRHRTARHFDFIKTRLEELHELAELDRHPIVSPRTRPGRTGLLKFAKTSHLTNT
RCSSLMDLRLPQVPSSPLIPLWKRSDPDLSGDADESGFSETSETDSNISGQGRIQPPLKNLLFSFFGLY

>E4YJV5_OIKDI
MGKGKPKTKGKKIQLGDFIGDTGNKIAVNGEMMELPSAPRATTLEIDVSLLPSRPPFTATVANLVYEIHE
DDLEKFFEDIDHISRISIPREEDDGDSRGRFNGVAYVTVDGDRDACIGSMAKIMAKNDLMLAGRRAKIEI
YDEFGSRGSRGGRDGERYGDRGGRGGDRERDPGFGRSDDADDWRRPAAAPSNDDRGSYGGDRYGSRGGDR
YGGGDRYGGGDRYGGDRDRGYGDRGGFGDRDRDRGGYGDRGGFGDRDRDRGGYGDRGGFGDRDRDRGGYG
DRDRGYGRDDRDRGYGSRFSSAADDDNQWRRAEPEIPRRDPRDEIRRDAPRERPKLQLSKRSVNEGSNST
SSASSIFGSAKPIDTTKKEREIEEKLSKLSVDDNRGPRRDDRNYSGDRKYDRNPSLTAGEKTRIAEEELK
KQVESEKTNINQEGDHNEELGAVSRFAALEDE

>E4YJV8_OIKDI
MSGTTDRNQLDDAVNQLQKYADPPRADFANLLGVNPDGGYSHTASVSGIQESDYPSIPATDSVPLLAQKK
RVPLPAELIEQFGHMQTDWKLGIFTEIQRAWLTIDADIFIWKYETGEDLAYYDGLSEPIITCALVKPVKQ
IFRDVKHILVVATAMEIILLGVCFNNDNTDEELNIIPDPLYTVSTDNIRFQSITGTDDGRIFLAGKDGCL
HEVAYTNDTGWFGRKCRRINHSSSSIGYLIPSVVQNVLFEDDRLSHLLVDRKRKTIWTLSEKGSISLYDL
GKNGAGMAKVASLSVSSIVNAATRAAPQFDKSVFSPIVAVSTIDAGSSQAIHFVAVTESGLRYYFSAGSK
ERPHHLLLAHVRLPPGFTSSTAELKPRKVHSALLTGEVSLLCSEETGLEADQMWSLIGISNGSANMPEEY
GVNRLDCRVWSCASISDIDDPLVPPETGTLGLAPHRDVCAVYQPSSEFVVMTKQGTSLYESLPSALQLLN
ILKRSGGMESAELERFMITRDQSAEGTASAIQLACDGAIDHVTSTWATGAYLRYGGAPTRGGAGAATANG
GITGAPLSPMANRAASPNASSFFSPIRGNHNTGNGFVQSTPFGQRSFGLPTPTQPNMNSTQLQPGPAGDI
SVVNSAKFRGLVKYLTRVLRPIWLYPITSKPDPAIKQLPHTTRLDSVSLARVLTALKKLKSWMDSNNIGT
VPSSVATASDIPSYENAQFSSVYALVCRSLEVAALWHLLTENQLHVVSKDLSINDKEMLDSVLFKDLVTT
EVGRVLCQNLITALLSCYIGDNASIDFVSRSLRDICPTLFSADDAICSRATEILALTRNATDQEAKERCN
EALSLYLTIAHTANLGAVVPQLKMAQYWEGIVTLCITAADKRDPSGMALHHFKQDCPEDDLEGKRAYSVR
RDCYESCLSVIKELVESAKHITSTPSLPISPGKKPQSRKENKNETIAEEAARAADEMLVQIINSEDELFH
IAVFDWMMQSKLSDRLLSVKSGFLENYLLQTVKSTPDNILAADMLWKYYEQMQQHGSAARTLMDLAQNAP
GLKLSTRLEYLSRAVIDAGAIMGPTAADEQLRRDAQEAAEVAGIQHLIQVQTRDNLLDDRLFDLNKLYQD
YADPLDLPEVKLAIVHSAGQYDEATVDGFWQEIIEKEVEQSKDLSPDAQVAAIRAKVLSVGQRYSASGRF
LPKLFIINFLEKVSCFMQWPNKGWVVAVFKDVGVSMLNILEAYFEIIQDGDDFWAQNGQPLHLIDVLKVI
FETFLERPNVVVPNERRLFVDKCLQIIASANVTLGGMTNADTSLAMNNLKQVQYRLERWLQ

>E4YJW4_OIKDI

MLIVPPPLMLENISIVINESVEDPRAFPSSDMRRSGRRLDMALLVLTLQSPCIVERSMKVESMSVSPEPS
PESKSERR

>E4YJX2_OIKDI
MGESSGHFAKKSTKHVKELITKLTNCMDGIITKHNHQHPGKMIALDCQMKNWRNLSFELKEMQQVWATLN
FAYNNLLHNEDGVVSRGPTQNAPRAEMFLSNPPPAEINKNSAEIKADFDAMDVADYAKAVKLEGVDDGYD
LDNLDMRDRVYDSEDSDKAPLGQLFGDVKLYETSDSDDSSMDVGQSYNNAGPSIVAGPSRKTDFAKKTKN
LKKKSAKDDSSKTTRKLDFSPKQSKKKTSQKKQTPEPVYDEDMSDDDEPLSRLVSEVNATSDVIIPVVRQ
SLRRALEISKTSTDPSKKKRGGRKASSKKKRLSKKSSVENQSSSDDEEAALVRKKAPKTASKRSADYDHD
VEDSPKKKTSKMVTKKKEKISRVSKTESSGKVSKRVASGMKKESAQKDRNETNEDKKAKKSGKTKDDNQE
IVPAKEKAGKGKPWSKTKERKTPIHILDRRKEEYHKKKLAKQNALANGAHQKNAKEDERAQKQQEELERQ
AEAIALKNSKIYPKPPSSIVGSNQSNSKPAVQEQFVTLPVPAVSAVPLSSGSVLLSPKTRKTPPHILQNK
KEYYQSNKEEILKKQREYKKRQSLQKKKADSQSPDDDSDSDDKKSSDEGNSRPGSPERSSSSKRKNEGKH
MMPKKRWTEELTTSKNGDSNVQNFIPIQIIPSMPTFELTPVEPVECVAPTQRGGSDGASRKVKGNTGSDS
NLGSKDSSVLKSDGDSGRIAVKVMNSRLCAQPDLYHEEPEAPPSPPSANDPADIVGSTNGSTTPQNQSSE
AGHKPNFKKKFVQDWENSLSNGPNDNLKSDAPSSEEKPSHKEAYTPKNATNMDSASL

>E4YJX6_OIKDI
MSPNAAESVVKSDSPNAEVKFDHPNCQLCTSKNLPYLFSKIRHVDTAPKEFEFYSLRLMKLIAEDALALM
ADKEVDIPTPCGTWRGVAADPESEAFAVSIVRAGDSMLQAVRELVPGIPVAKILIQRDETTKEKTPVLYY
KKWPKNVANKTALICDPMLATGGSVIMAIKSLLEIGIPEEKMIFVNVISCPEGLNRLGREYPSVKIVTGM
VDPMLNSERFIVPGLGDYGDRFFGTVE

>E4YJY0_OIKDI
MAETLDRAESDPDPDDIFASAVLAANFSDEENDDQSESESDGLLDQSFSIVQDFQTENFSNEERNFCFDS
EEISLDDYFHSANFEYFESGTYSLRTIAEEDEEELSREREEEENSKRKNPRMERKSSSDEQRKKISNANL
LESMLNDLDDTCEIQFQDEEEEFFEAETKILAEEIVADTINFAIKLIAAEIKEAQIAKRLEECRAEISSE
KLVRPSEVRKSQRYSKTSSKSKKIENKEEPPKLSKMPIGETEKKPENEQSKVINLSSDISVEETFEEHHR
IDDLEIQNQFKDETEIPDSPPLEASQNVKKNDKKLEIQSQNISDNDNIPKPEESKPEKVQEIPAKPAKDS
KIDKSNQNSSQKDPPIAQAQVPAPKSTPTKQEKVETNSTKLDCASISKSSAAGSSFTKSQRRKSFPRFPK
KRRGSLKESSLFSCIMPKVKDTEDASITSLKSAIPKSSRYEEKSSCQIM

>E4YJY9_OIKDI
MRRNSRILLLFGIIWFSGVYLYVRNSENTSGVFNAESNPRHGNRISKDAPVKHHKKDHKQKIKLVEDHNL
DADKLEHDIQDIEHGLEDLKDSNDDEFAAIEDSIRHDLADDDDKDYADMEALSEEDKDDENDAAKKESKA
KAEPTGDSKINKEIAEGMEDVEFEQDELQGPPTEKTIQDKDELDKGENDEDYEDDGKVDWWNFDDSAYLA
AGALGADEDPYEANKFNQAASDRIKSDRAVPDTRHKECSKQKYQVEQLPDTTVIITYHNEAHSTLLRTVI
SVLHRSPPNLIKEIILVDDFSKNPNIGPPLTKIKKVKAIRNPKREGLIRSRVRGAAIATGKVLTFLDSHV
EANEGWLEPLLGRIHESRTAVVSPIIDVIGMDDFHYVGASADLKGGFNWDLVFKWDYMSEQERRERRRAP
TSPIRTPMIAGGLFSIDKNWFHELGEYDMDMDVWGGENLEISFRVWQCHGTLEIIPCSRVGHVFRKKHPY
TFPGGSGNVFAKNTRRAAEVWMDEYKEFYFAAVPSAKMVKFGDISKRTEVRERLQCKSFSWFLENVYPEL
RIPNKDAIGWGAVSQTNKGLEECIGNTHGGGTLGMYRCHGDGGNQEFTLTKEGKEFRHNDLCIGYNAKEP
VGNPVKFNTCHQMSHQRWEYFSSQIKPEGHTNLCLDSKNHLEKGLTLEVCNHSKTQVFSFEMRNIK

>E4YJZ1_OIKDI
MTSIASRVRIVQGDITKLKTDAIVNAANRSLLGGGGVDGAIHRAAGPELFKECRTIKGCKTGDAKITHGY
NLPATWIIHTVGPNLNAGDDKEKLRDAYQNSLNLAIDTKEIKTIAFPCISTGIYGYPQEEAAHIALEVTR
KTLKEYEILEEVIFCVFLDSDREIYERLKPVYFPDDEKIRQNQIEDSGDDLILPEVPTKKRKEEKTAEIQ
EEETLTKADVLLKTVVNYEL

>E4YJZ5_OIKDI
MEIFKEEIKAKHEAAEALKAKQKEKAKLTGIPVDYNPNFNAQAISDQGDPHSTNIFIASLSNRCTEEDVT
HYFGRFGPLVSVKIMYPRTQEEKFKDRNCAFVAYCCRNDAERAMSKLQNNDFKGVDLKLGWGKAVPNIQL
QSPLYVPDRLKWLLTPPKQSNLPLNAQPPPDLINSQSEEELHKCTVRVVIPNDAALTRLINRTVEFVIKQ
GPMFEAMLMDKESNNPMFQFLYDYQCPAHSYYRWRLYSILNGESFTFWRTNRFKLYLDGPWWKPPILPFI
QHGMPDSDEDDFDVHEQAENKPGNEFDSALNTVTPVRDLVGDLMALCIDQVDHAQEITDAILEAVLVDEC
TLDQRLGRIFLISDILYNGSAAPKASRYRILFDQHLETIFEKLHVVQKEIKTAFVADQFKNRIKTLFQAW
TAWSLFTNETLIKLHNIFNGIEEEKNESNGSSDSDVDGKEIDEKRARVSSEEHREQEASSDEDEVDGVPL
DGEPLEQASTASEPPNNSAHNPSQPAKPAGVGSGFVASKWEVVDDDDVKNEAVTSAEIFAETKKMEQEKK
RNNDYEDKKEAKPEYLQSDAWRKAIRDIEVKVIEYCEQLKVLDDSVQAEAYRSGLITKLSEKFKDDPSIW
EDSPRSSPTAKKSKKDRRDGEKQKRKKRSRSRDRRKRSRSRSRERRRKRR

>E4YJZ7_OIKDI
MADQQFETTTAVVNTTTTTTTTTTTSKPTWTTTTKILARPPATQKPTAAPEPAKKPNNNNPNRFGIFQPT
CGKTPARASRFRRITNGSTARQGDHPWMVALAYKKSVGCGGTIMTPKLIITAAHCIGGDPRRADGVDDVP
DEMYDNIQVRNARSYYIHPEYNSISDEHDIAVLELDEPLTLTDYVQPACLPDGEPRVNEYCEVAGWGSST
PDNAHQDREHQSDNWLLQMFELGTSYPHLPSWGFELEESKQTGNELKSGFLQIISQLECQKKYENDDITG
NMFCAGSDRGVDTCLGDSGGPLVCHNPASQRWEITGVTSWGRGCGVEEFPGVYTKVVQYLGWFAQIEAGK
GTPSRVDHLQFDNDDDNNENDIFDYEASGDDLSADFYKCGFTIEPEIPHNGIPASGVITSPNFPKKYSSN
EKCRWLIIAPAGFHVELKFTNFKVEYDSSCRKDRVEIHHDGQGFFLCGNGIPEDTFTTTEKQMEIYFSSN
KLINFSGFSAIYTIKQNMQNIGDISASFERADNGVVVSEADGINGICGKPLIQPDRMVRMLGGQPIKPTQ
WPWLGMLLEEDGEYIHMKCGVALICRQWAITSGDCARELKYGEKYVHKVKFGNMRWDEQSKHQEELFIDQ
IIEHPLFNGGYDYDIALVKFARKVSFNNYIKPICMKDYVHKNKGGFNCFAAGWGMNSQTMSTRQAHSAKI
NIVNDGYCSNIYRKTYNTQQMVCTGGDNRPCQGDGGAPLICNADNGEWFLHGISIYGPGCNKPGGGPSVF
VKPSVFLTFIEDATGGCVKSY

>E4YJZ9_OIKDI
MNVLRVAKSGLILRNFSRRTSNLANAKSTVTLWIDDHTLPGQKYIRRRPQEFSVDGRGHVICTIGEFNCG
GVLGTGKHGVSVYWGPDNPLNYVDNLRFSPLGVTLKSKYSWEECDRLALQYSREMNMRAIKNAAETAKFI
GIDCLTITLQNFKLINQFNSMEASGSLMHSFKYGMKNVVKTNWYHMLSLAGLKVFLCRLEYQDAAARDLA
IKAANAGRFRPLSASQQIVSEEHKELENNMREYLSQVAVNNGQDNIAQVRENVKKNRKYVVTRQVSATEN
KSILLDHRRSVGLPEIPQHSFVEANVDMDWAKLQSMAEDDYDEFDELEKIFVSERKGPELSDEELLNTMK
YGVSIQDQINEANKKLQLHEGDLKAYKESTNQQMNENKQLIHELTVTNKQLHKQLASVLRGDEKVIEAAL
GDHKSEKQALKTKAGKDVVNIIQKRLNAIRAQNDKKRRQLQELQTSKNLIEKDIKREEDNCRGTSENARQ
LRTIENRLDKALLKQREAEHVKAIYEKMLAKLKEGALMFPTILEEKEHQVEAQEEELKRVQGMLAQAQSQ
RDRAKSQFHENEEELQRERRERDRMLQELRQRAEEKRGADALERRTTRMESRTGSTPGAVMDPVQNEALP
QNNLEEEIAKYEDIYRAIKEATGVSSIDEAVSRFESQGETSKHLQNLKEENEQKINGLKQQRIDLEAKFN
TFKYSDADEAAKIEEELFKMTQKTEENKKLHNEYQKKLERQNKLLLNVRSGVQHLCDKLDFVEGTGETVK

PENPDVVADINSILERSGKQIDSLLQSLGEEGVEGAQERAGTDLTSWAITRVEETAPYSAIKSPGHHAAR
APGDLSDDGSSGEEDGQMLTREEIKKQADALVKARNKKRGRPVKQKGRR

>E4YK00_OIKDI
MLLIGLAFIDLRTNFGSTFLCSPSRAQFLTGRYAFRYGLGSDPISFENPIGMSTKEKLLPEYLKEVGYET
HAVGKWHLGYCNESFQPHNRGFDTFLGHYGGGVDYHTHATQGALGSYLNHFLNGEPHIPEDGFEFASYAW
SNRTRKVLRENTDKPNFVYLAFNAPHEKVAAPQDLIEEAKSMYPGIPGTRRLQLAAVKSIDVAMQSVIEE
AKTLERETIVVFHTDNGGALLAYAGGFDGPRGCSYPYKGYKSVLAEGGTLSPTWVYSTKRQFHSRYIDGM
IHIMDFFPTILRWAGYDKPMPRDLDGIDQYDLFENEAVTQIRDRFIYGLLHEWDDDNLVWKTTYAVRYGD
YKFMNYQSELIGNTQCPEGWLNSKHAKIYLLASLFPSA

>E4YK01_OIKDI
MAPKNLETFLPDVSSPSIKQTSRVNYGKLPEDKNKQLKFISDTHVQSFDWFLEEGLPNIPNSVAPYEFEM
EANNDQMRFRLEVEHIEVGQPTLESSIVCKDRRLFPSECRQKKTDYTAPVHVTFNLINLSSGESQVVTKK
DLLQMPLMVMSSKCHLRGLSPEELVARKEDHFEIGGYFIASGNEKVIRLLNMNRTRRNFPLGLRRNGWKR
RREGYTDAGLMIRCMDNWERTSNMNVHVTKTNEMEVVFYIQKQLFHVPMMTVVRALTSWTDLEIYREFMQ
TMSDEPNYDSAVKRMLKKLVKIQHHFLTQAKFLGQSFRIFDICPAWKSDVEAGQAFLKRYCLIYLNNDED
KVRAFALMARKAFRISHNTCATDDIDSPESHDVLLPGHLFQSMTLEFFESYQRSVSMIMAKEYSKQLEKG
NDEVGQNMLSFAINKIGNMNQHFKYLIATGNLKTNDGLGMQQLAGIVVGAEKINFARFYSHFRCIHRGAF
FMEMRATSPRKLSTAAWGFICPVHTPDGPPCGLLNHLAHTVRINVKRENTAKLEELLLEIGVKPCSIKAN
EFDVPVIIDGKQIGWIANGWRAQAVEKYLRRLKTSSSDRVPDTLEIVHCPAPENKGEAVMFPGINLLTEP
ARMYRPLLNLREDKIEMIGIMEQVWLHVTVNQKESHELTEYQELYPAALLSELACMSPFSNMNAGARVIL
QCQMAKQTFGWPSYTLNHRSDNKMYRILTPQEPLVRSHLHDSWGMDDYPLGTNVMIAVISYTGYDLEDAT
CVSKMSRERGMMYGTIYKTKMVDLQDTAQSMGYRSGLRLGCRDRFDPNELKAYRKIAGDKLDIDGLPFPG
GIFTYQDAYYCYYNEHEQKYSVGKYDSHAEDAIVESVRVFTNKRPQEGGFQQAAIQFRIARPINIGDKMA
SRHGQKGICSRLWATTDMPWTENGIVPDLIFNPHGFPSRMTVGMMIEFIAGKAAMVNGRVYDSTPFVFDE
NYTADEHFGKVLKEAGLNYHGYERMYSGISGEELEADIFFGPVYYQRLRHMVYDKFQARADKGPIDPYTQ
QPVKGRARGGGVRMGEMERDGLISHGAAFICRDRLFHCSDFYKTRICTVCGSVLSVSKVKPDKTKDKNWK
LMSNGKWECRTCGPEAKTLLTDLPYVVKYLAAELAGMNVKLDFKFAKEDKTARTEVLDIVEKFER

>E4YK05_OIKDI
MKLFSAFISATLAARECGDYACIESIETVGCLDTCYEDDCHGTQMYCCGELAQNDWDAIIKFQSVTKAAA
EAKNIDHNILAAIMSRESRAGRKLESWNGEYGPGWGKCKNGSCYGWGLMQVDRRFHQPVGAWDSQEHLEQ
ATQILIDQINCISRKFDRWTLPMKTKGGICAYNRGCSHVQNYDGMDIGTDGNDYANDSVCRGQWFKNRGF

>E4YK07_OIKDI
MDRPALALDENTPGSDVTAETAAALAAAAVLFKDDADYSNLCLSHAMDLFEFAELYRGEYDENEAFATAR
QFHPSSEFGDELAWAALWLYYATGKRRIVILPIKSLNSKPLKGNNQYLTRAEDYIAEFGLDLSAPTEFSA
DDKTIAVMVYHALLTSDQSAAGVQMIIDFCTSFQSQYNADGYLRMTDWHNFGATSGVAYICLYAANHVVS
GQTEADFVDFSKSLIIDVLGAGEQSYMTGYGHNPPTRAHHRDSACPIGKDCGWGMFEDKYTPNEFTLEGA
IMGGPDATSGFWEDNRKDYTRNEPSLVANALFQATVAGIRERELYDEYPSQMILNSDPSGISLPTEDFSD
FFSSWPFPPGTNVVDYCNDKTCENDGICISDYTNYTFTCDCQDGYFGTRCDTITEPVVDFIRQWGHGGEI
KLNLPSERPLGAWQVLLEFPPGCALQSLDIWHGCMDPYASSLSNSTFYVYQVGWHHDHQNIGIVFETDQT
YDENSYWGNSASCFDESAYSLTLVARGSHEADNIEGMTLQWSTDPDKCPGELPWNSPSTDPAYPENIILG

GDEVIDQAFLDLMVNNYDGPGHVNGSLEVEWVSVGEEVGWMRIWLPDAMPPAENPWQLQIAFAEGCNVQT
VLTYQACINTVNSDLNTEANATNPYGENVANLLLYQNGTDLGTDFIGLRIKFPQADQMTCLDANSYVFNL
YEWNNPIEASSLLDTCEIMYPDRDTQLALLGDSGAVYVPESSVPSMDFLSGPLSWTQGANGHFYLPNPKP
MAPFGLQVQVPAGCNIQRINFWHACVVLANSNINSGYFELEQIGWQEEHGYIGFSVDFQPSQYSSDIPAS
CKDANLYTLTLEDRSLDPQMGISWNGFYNDYADNCPQALGEALPYSSYEVQLRDGAVMRTSLSNQMMSRL
GNDPFAGNAAGTGAATSNPGERPIYYSDRVSDGQLSQYQQASIATWAKRYDAEAKEEKMREAERQAAKLA
FQIENGLIDAPRQIQQNLQQSRNAGYNAGSGGLMSGGIESSSLPSSSIPVQFETRSMDATSVELFWQHPA
GKRERYKIEYKDWIYTVLQPCYITKRC

>E4YK08_OIKDI
MGKCECKKGYSGDGFSCSDVNECLTGKSECDEHASCTNTIGSHVCTCPNGFIDYNGDGTRCDDVNECDTI
RPRCHNLGQCVNYPGTYACECLPGYFGDGTSTCADVDECVQDNPCSDHAICTNSVGSVTCECKTGFTGDG
FTCKDINECETGEHNCTPLGGKCWNKPGGYGCMCIDGFKGNGWKCEDINECEKEGVCHERAECFNEPGSF
RCKCGAGYRGDGVKLCVDLDECAAGMHKCDSAATCKNYVGTHRCKCAKGYKDLGSGFRGECQDIDECAFN
NQCTGKANINCINVPGGYRCKCKDGMIGDLRRGCKDQDECVAGTHECSPYAICTNTLGSHKCACRAGFKG
DGLACEDINECATGNHNCNAKGSRCINIPGSFECQCAPGYSGNPKTGCYDVNECKNDDAVCPEDSSCLNI
LGSYKCNCAPGYQGDGANCIDINECEDGSHSCDAAAKCTNTIGDYECACPSGFTGDGFSCTDIDECATGS
HACGSHAVCVNFRGGYDCACPANFVKNGVGCDAPDLCSPSPCPPGAECRNEHGTYVCACPSGFVSRAGVG
CVNIDECAQGLAGCHEHAICIDTDGSFQCKCKSGYEGNGRDCSDIDECASSMGSDCDRNAKCKNVIGGHE
CTCKPGFIGDGLTCQLSDACLADEHNCRFPKVCIPLKKGGHECACDGGYFAPKNAPDTCVDIDECTMGTH
DCNDEETCENREGGFSCKCKEGQFRSGGVCRERDECVLGLHDCDVNASCLNTGKGFKCNCKDGYSGDGKT
CSDVDECVIGVMREMITPKKEIVDISGKKTETVLTAIARLVLSFQKIFIYCAFGKTISLRSACPGAECVN
TVGSYKCKCKEGFKVLAGKCVDINECENGLHTCKNVGQKCVNTPGSFKVSFFSSDQLKCDDIDKVLPNAI
FDTAFAATREARTSALVQKDSRATARHALKSTSALKELTIVTNKRNAPTRKEDLFALARTDSLALDRLVL
MKMNAEIRHFVASQICTVQTPLARTNVPAKMVSNCHPNAFCIGIGNDASCVCNAGFRGNGFHCEDLDECG
LETHECHGNATCVNTPGDYDCSCPPEMVGDGFDCQPSRACDNASCHTNAASGATDTAVPIATSVSMRRTT
AAARLLLARTPSARTYAPAKMATKATASSARTQTNARLEPTTVAKVRFVSTALEISSASASLVLTVMEEP
ALMSTNATHSSTIAPRSLLVKISEADIPANVQPATTAMDLNALILTNARRESTTVRRTRNASTLTVITPA
SVRMASSAMESARTSARTSTNALLTFTIATRQQSARTRLDRSLARAWKNTKCFNTAEVRAQRNFNFLTTL
FNNFKAYTCSCKAGYTGNGFACSDVNECQISSPCPADSDCTNTVGAYLCTCKTGFQGAAGACQDENECVN
GRHTCESASLCTNTIGSFKCACDIGFEDSGNGCVDINECAKQSCHPDATCTNLVGSYECACKDGFKGNGM
LCMNIDECATGAHTCAASEKCEDTKGSFNCVGVKCPPIGTFDLALLLDTNVDIGDAGLSATKAFAKEMIN
SFEVGASSAKVTVAGYSGEKVAPSVFLLDSATSPKPTLLSKVDSINFSGRDLRIERALAFLVNFSFLEAM
GRRPDKPGIAILTASKKTYSASALDAVVRRIRTDKSTRYITIGVGEASETEMASVSGNPQYVFEIASYDE
LPSLMPKIMDLICEIDAEFNQ

>E4YK13_OIKDI
MRFTLARLNYKVAYLGKWNKVPGDYDGGKHILPPKITAKDRERSLKELWREARNEMWLVRHKQEAIRQNI
APRVSFQRPRNDEKRTYESQRLTHDLALGPDVTFEDRFMNIAQRGNQWTKPTHVKYPYLQQHLETGINPK
KLHSHYPVAVEKTLSWRDVKGFGKK

>E4YK14_OIKDI
MSQVNSPTFTEKFVASVYLVMFIVGFTANMLVILVLLLTHGSLKAPAGLYVLHLAVADSLLLISLPFAAD
NRLRGTWIFGRIACKLMESMKLLNFFSSIFLLTLMVCRRVSYKWRQSVTNSICAGVWLVSILTVAPLVMY

SDIAYRDPEDNQNARCIVAFPDIMNQVNELDFNGNVDFDDAHHFYIADGDHGAVESHCLSNGSKVSSLFK
QLIKPLKQFRNYMYIITAVGFVIPLAIVTFCYANIISIMAASQKKLNKSDAKTKDVAGILKQMRKDEHRK
QKVSKLKGMPPRRKVTVLVANLVISFVCCWLPFHFWHIVRLSGLDLSEHHFCAKIRDLTFWLVENQLRSI
DVFSLAYSNSVLNPLLYSFLGYGFRNKLIAALTRIKLCFRSRSPYRLSQQGNERRSQNMLEYL

>E4YK15_OIKDI
MYEISCSMGCVLMSIVSGSGSSSISSISSIMSSAEGVGRSKMSSEFAADISCDNSCFILEEYYKKMEEVM
PVKKGRGRPKKSDEKRGRPKKSDSEEKIVASDSENSGEKRGPGRPRKTSAEKAAAKKAPKRKSSPKTATA
PKRKSVRERAEQKPVAKYQDESDSDGEFEVEKLLDVEKQGRKGTKYLVRWVGFSSKHDSWEPAKSLPKRK
INAFLKKQEHADPSSDEEENFEEENSSEEDEVYEVEKIVDEKLYYGKTRYLVSWKGYNKDHNTWQDVDSL
KSCKEALKEWELKKQQKEEKKQEREEKNMQKKSEREEAKLAKAAERAANKADSA

>E4YK25_OIKDI
MDRTESRNRSKLRDFYKSQARENAELVRNPQFNLDSSQFQKNEYIDGVLRQMPLAALLQREREIGKEIST
LDQSMQSLVYDNYNKFIKATDTIKQMRVDFKDIEKSMDELVSEMSEITKLSTSLNSKMKPCRTEVANLVE
QQETLTKMQFLFELPAELKRKIKEGDFESAVEDYIKANCVLKNYENHPSFKSIQEECELEYKELVAKLEE
PFSEKESEEELLIRCIRLLVKLDEDRSSSLADGFLKSRITKVKHRDNTLKSFSSEISAILADICVNITSF
KNVFHERNLNSSLAAQHAYQSLKRHVNDILEELTDDFSEILVTNDLSDDQLSTSLDLWYSKLSATAQILP
ELNLTGRSEELVVSQAKIRHDSIRKTLFMKLEEEILKIEKSESPGATNAAILTLIRDSQHQLDKFFTMSK
SYSQIEYFLVEWSDMAKTLKIDAVDHIASFCQKTLETAHPALLFPLSALASIWSETTVDQCIERKDRQTV
NRLKISFSQICSAALKELVFIRGCHCAELIYRSVMAKDWLNASEPRGPRPIVKTFTDEIRKIDEECSKFM
RPGVRPAKSTRSGSMRIDTLSQRSTLNIQRLFTDKIEIYDTVQSTTISVTSGIIKIALKAMLESVRLKTF
SSYGLQQIQVDLSCLHHILWRYCEDESHMGTISDEILQSAALRALERELMEADVIELIVEKAQLA

>E4YK26_OIKDI
MYTMNVEVPFESERHAEIALNSVIQDEEPRAGTHIERKITVEGNLLKIHWEAEQARILRTSAQSLLQLLI
LVTQTIEQFDGME

>E4YK27_OIKDI
MTEVKMADDPQYNNYLRIVYEKQALKKDLQDTYRQLHNNLANNSDPHEKAALEKVMDDINTTNELIFSYQ
HKVEKIRETKESQMPQRAQTIEIHLHNGTCNVTSHHEKTIEEVCKMNNRFFSIDLSKSHLEMHTFDSTGK
KERKLLNWSEPSSKYANSELWLKTTDEAYLKSVAANTHCFEVKRKSRKNCSICGGNFFSLPRKLWKCTYS
DPAKKKNCDLIVCTQCKENNITKPCISVEDPIGIYKKPEEENNDLDTWKASPEEVKDWNPVGQGTWGIVY
KAKWHGPVAVKELKCKNPTDEEVRIFENEVRMLRKTRHEYIILFMAYICEPEKSKLAIIMSWCESSLHKR
IHVRSEKMTRLEAISIASQIAQGMGYLHSKTVGILHRDLKSANIFLQSDAKRSVKIGDFGLATTSAARGS
SKGGSFRDKTLGSIPWMAPELFRTPAPYSHESDMYAFGCVLYEIFAGEIPYAAKPHLRQEMIIFRVGAGL
MKPNLTRLNPREGEEKIVGAESCPEEIQKVMKECYERKPENRPKFEVGDNNLVEMFNTLEASENDEINRI
NQQQRAAERQVLRSQSLNTAQHSYYGSS

>E4YK38_OIKDI
MANALLFCALMGQTSIVSRYKHGFVKPNDERALRIRGYEAAHGVMQIMPDCTIAYGQSDEYSFVLRPDTT
VHGRRRQKLVSLAVSKFTAVYQFYWAHFFLETALLYPPAFDGRLVLYPSDKILRDYLAWRQVDCHINNLY
NTTFHSLIQKQGLTASESEKRLSKTLSKEKNEILFQLGINYNDEKDIFKKGSVLIGAENRKDRLLFNPGQ
ENEGEIDLWEAQSSEESKHLQKRQVLVLHVDIIKDPFWQRYPNLLLPLNEVKKARKRQLQTDCNKKNNEY

LFILVFYHALVFVILDFNIIRNLYGNLKHKNERTVCITVLPRFLYLVSLPSRNEPVSFTCRPVTGTIGDL
LEQIKEEDRGVDHAAIYAQGEIRLASATTISQLLTMGDFQLRINDIRHQIQLPDDQFSANQVCKMAAIKQ
LVHSLHSSLGIAEFQFQHHKKLTAKRDEIMKKLKKCQLVKQKIDEDSIKFSQQMHAGAMIGMSVLWGIMA
RLTWWEYSWDVIEPVTFFVTYGGAICCYTFYLATKTLPEYEHMTNRWQLLKTHKLSSKRRFSIEDYNLLS
HEYALVCEQIDRLEDELQYPPDKAALERFLGGGGGARSGARCSRSERQLSTGVDHHIANCNSKEKSKNKQ
SEVTMKLLATLLASSAVVNADFWDGAEEQNHSTWYSETNIGTFSSQNDPEMLEMAAEEAAAEIDFGGIQM
RGKRKKPKGFLIDQAWSDYDLKAELFLQQIYPGVENDPDFQMNKAEIQKEDAKICSAGVDPVKGKPVSCA
TDERMGLSAPMTPFQTAPRKFRQLKLMVIFLQKEPGFGKFCYYGCYCLPEGAHDLSGGGYGEPVDRIDRT
CKQFNQCYSCAKLGNGKLEGTSEECIGEYTKYRFQMIEDEETGEKSILCRNKPGTCARHICECDKRMAED
LSRYEDDWDVTYHTGRNNGEWNFADNCKKKGLGRYNKPETCCGDEFPNMRPKQGGKECCGSQPWDPTVQV
DRQCCSDGRVRPSGYC

>E4YK41_OIKDI
MKLLTIIPILTKAQDIYYDYDGSGENYEYENSNVNARNRVSAGPTFDVTVKEIQEAKENLKLEFEGSGMT
NKDLIGTFSFETGGYSYEDGQAIAELLDEYGRAKKPNANGAFGLFGNSKEEQEQERGKKNKNKKSNKNQV
VGSYDNYQNYFQAQLKNEEDFSCWTCSRSYWEKDSTGDMYADCRTHGEMITCPKQKEDLGSDHFSSANSC
QVTERRFFGIVTELHVGCKQTRACQANFNQNKMNGSVYGRSCRPESTFGISTCRQCCSASECYSDSTILA
ESFNTEAEWLDDTHHATLITV

>E4YK47_OIKDI
MREFCILLVSFFESINSDSNCYVVISGGKIGSEFTDEAFKILLQNNSHHGNLEIRIEKSPYEFLPSKRAG
HKLALWHNIPMLTGGELYAAQRELKSSECYLKDTGQEIIKKDLDLIDEEVSQWQLLPDMNEGPRQYHSMV
VFRNSPYSGTAWVCGGELWKRAKASGKLRLEVVASCECFNRYYMKWIVCPTMIFERKFASSASSYFGIYM
IGGEDKKGRILSSVEYVEPLGGGKWKRKSSLPIKVKSAAAVCIHDKLFLFGGFDGEKSISTVYHLNRTNW
VDFGNLLTRRFGHTVTAMQSTVLIYGGEDDGEFKSAEILNIISKTSGADILIF

>E4YK54_OIKDI
MDAYFAHSMIEKEDFEKMEQYILDHVPAEDFAAVRKNLKPTSIILEEATDKAYHEGAVLEHGCPVEIRKS
LEPLEKRLPDVVGIGFAKCGTGTLQFLDCHPSVTMRSTEPKFYNSKNAFDIIQFDGEGTPEKYDYLKKIE
ITAGKDKQKFLKFTKTVLTVNPKKQYAGSAALDVAKAMKILNPKTKIIGIACDPIKRVYSQFAMKNRRTE
FKTTDGMDEEAIHKCKACLNKDLNETIKDFTENAQRLLDNGGNSPGFDEYARALEPFVEQFGDENVLLLD
GENLITQPNQEWARLLEFLGLNKESMKFYIDEEKGFPCLEKPVKYCLNGAKGTSRKIDVRQEYPEDTAIW
RKSFSPKIKEMILFKKICNKIDEKCCQKLRDEKSSFSWAHEYACLDV

>E4YK60_OIKDI
MPELTKESSNGRTHVDILERRLLQNQLHTITIQENYTDLIEDLLDELCQLRNLEVATDYLCGSEELLDER
ERLHEALEIIRSDLRMEKNLVKLEKKKKNELKAALADSEEKRKKLIKKKGKRKKLIADFWRLSGSFEMTF
LTLF

>E4YK86_OIKDI
MWTTWFWVVMNKGLLIAFFALGAMRGQQKVGSVYARNGLIPLTSIINLITQVLVLYVYYAYIYYKYDYYY
DDYYFFHCTSTCYSIRKASFGLAIVSLILSVVVIIDLIVASCKEPKPIPPPQNAVVPIPMMSIPQYAQTT
QVQYADPPQYGTTMNTKNPPTYRQDLLVYNPQPAQPVMTMQYAVQPVPQKNPNEFRNLFNAGHIALMTLN
AIFCSLIIVFMSDCLSY

>E4YK87_OIKDI
METCLQYDYDGQCVEYGVVPYDEFDVQSVKMSPPNEEEEDAPAFANKTLNGDNFKKYAPFIILTIVFVVF
LKCWCRRNTKIVEKMKKEGGAKSGGATSGGAKLGSVKKPPKEISVTIDPDSEDNDGVPPAPSAARTLRFK
EQAEKIGETFMEKVKAPFQKPMKGYQELGSESDSVAEERSEANFDVDKYGAISDRFF

>E4YK88_OIKDI
MGKFINVTTFTFAICTAMCFSVAGTSFTRAAFTTIERYFGVSSTKISIYASFNQMAAIIVTPTAVHLTRN
MHIPRILYFSFLAKAVGFIILVLPYFLGPTYHPDGVLNAERNDSLNEENIFCRNEAQQDTTRNSMGLWEY
CIPISKVIIGLGGCLIWPHSISFIDNNSKQGQSLVLFGLNFVGILFGPMLAYGFASYVLSLWVNFTYECA
PEWVDPNGSDWIGAWWVGYVIAAAALLVFSTPLAFFPRNLKDENKLDEETDDKDENLVLKDHFEDEDKNT
IKKTSSSMISETKALFKNPLFVMQVLAGIVNSWAMATKATFGQKYMEIQFGLSPADASQLKVYYIGSLCL
GFLVGGLIVKKLKLKPFNVAIFLLVVQTFYSVTFLLLWSLEGCSPQTPDMLFGANEEQNLADCDCRTDKF
VPVCGKNVTMDGYLFEELTILSPCKAGCSDVDVTYSTEEKELFGCPVISEGVHSRKYINCAVPVETGICG
AIECKSKVWIYLGLVCLGTFFSGINGSPGTVFLLRSVGSERKPYAMSILGTCMKLFGWLPAPIIYARLFD
SLCAVKGSLDNCVFYDNTAVRHFYFGINAVIAVLYTLINCGFLTVFWKRYKSDKPMWGDMDMDKPF

>E4YK96_OIKDI
MKAYHEHLRAQDFDCLFFNAWRDDASDKVFFDTILTDALDEKPAGSLENAVERLVAHYSSDDAFHLVIFL
NNIESNCIARSLDLLVELVTCKKISLVATIDKIYGAFAFDQCQRSQLNFISIETATFMPYVHETKSGHSI
WAKSAGKIGVASLQHVYDSLTANGQGVLKIIIKDQIAKIKEKKDGPSFKELYKLCRKSFLVNSEAALKAQ
LVELKDHQLIGESTQEIRIQLQTSQLEEFLRTNNK

>E4YK99_OIKDI
MRVDIDEAFAFARKLALENGEIVKNAFHKPKNVDTKSNPSDLVTETDRLVEKNILEAIRAKYPTHKLIGE
ESFVGPMSITLTDEPTWVIDPIDGTSNFVTGFPYVAVVIGLVLKQEPVFGVVFNPILGEMFTACKDKGSF
LNDEKIAVKSTKDINNSVILSGFSSGRNYEHLAKVRNNLESVLMNPAIGIRMLGSTACAMTMVAAGRADA
YFGARFHIWDIAAPSIIVTEAGGVVSNLAGNELNLMNRQIISACTNELINDIARKITPIEIPDPDGKLE

>E4YKB2_OIKDI
MIRRGVQRLGIGLALGVGYDRLVADRLWGMRRFSYFDRAWSTGVAAIGKLTFFDDGLGTPDQIRIIDKYR
FEILEKRAEEMIDSFMKSIVIKSKKIDGDYLLVEGELDSNLDLDDELWPKECRKGFFQLVLPVDGVIEGI
AIQTAGTGDHGFKRRREIIAKPLIQEHNIASCIMENPYYARRKPDKQQYSGLRSFVDLITLSMGVGIECN
ALAKWLKEELGVERICVTGLSLGGHTAALAASISPVPIAAAPGFAWSTSTGVWTTGALSNRVDWANLESD
INAHPGYEPLFESLGTGLDRWLERDQEIAQQIDSLPELQETQEVTKNLAKSKLKLLANHFSHLGNYPVPK
DASLIHYVLGEYDYFYSRDQMTGMDRVWPGTTFEVHPTGHVDGFLLYGRAYRNSINTVMNRLPEARSKSV
NLYALENVPYEEIVDGQGTDVFQKAFQYCTVQTVNFLQRS

>E4YKB4_OIKDI
MSEDQSKPVKQSKVPSHDQILEYLTMENTRNKRKLALVDEFIKAQARVDPYNYETLEAAVQRIYAECVRK
PQYASTYTIFCLKVYHKLGDIGFCLDETGKYVSTSYEDAAAANMKWRGFREILVELLRTNFYSENKEQGL
IRMIGELYNCNMISRRLLQRIYFDLMHRQTAQDLIALTQLTYAVNDRLINGPAKQFVKEEMHPSQKEAER
RFGHMIFEGLKIFIPKVLQEQQLHDNVAMELQNLYNTVVFSLTKIK

>E4YKB9_OIKDI
MACQFFRGKYLADRMLNNIKQEVFKRQVRPKLLAILVGEDPASQVYVVRHKENACNHVGIASEIIRVPETH
STAMVSEILVDAGEREDVDGILLQLPVPDHLDERFLCNLIPPTKDVDAHNAASIGNLVYGRGIFVPPTAS
AVAHCIQENGIDTFGKRVLVVGRSRHCGLPITLIMQGFRTHPELPDVADLYNIAASSDIIISAAGVPGLI
RPQMIKEGAILIDIGFSRKISGSSVGKVRLMGDIHPDCRARASFITPVPGGIGPLTVAHLVRNTLNAHLH
ANGLPRVSLASLCRPEFEDEFEYAS

>E4YKC3_OIKDI
MRKHTLKALLRRKKSHLGNKWSILFLVAVSLARVAMGIFNSITGPTLPVLAQHVCKSPTTVSWIFSGRSV
GFLIGSALSSVFDRFGISQMLMLSLSVLLCAVGLALMPILTDLWLLIIIVTITGIIAGYIDAAIQCIMLK
VWGSKKSASILQLHHFTFSIGAFLAPILVSPFYSGDDDICSDSVDDNNEPVCLGIGDNNSILTPFLISSA
FIAAVFFLMIWLYFVDITEKIKEDNQESADEREEDSIRDLWQYFIPVILFYFCIVSGENVYQSNIFSYAA
CKVGWESGKASILNSIFWAGFGIGRGSGIFFSRFLRPSTYIIIDLVFCAATATFLSIFPTNEIMLWIGSF
TYGLGIATLYSCGSVTVTS

>E4YKC4_OIKDI
MIIELFTIVLLGLADAQKSVKLKNVDVLTLYAGRMTTGRRSAPVPQLQCIGGSAGCSAFVPTVVQCYNRG
TDGQDIQWECKADMEMKYKFGQVQVTCEGFNYPDDPYVLAGSCGLEYTIDRVGSSSSYSSDTGGTSFWSS
SNTDSSEEGSWALFIIIGFVIYLLVTNLNSSSDDRPPPYNPDFIPPAGSNPSSNTGWFSGSGRNRASSNS
NDGPGFGTGFAAGAATGFAAGNAHGRSNRRRGWFGGNSWFGGNSWTNTNNSYSQGFSDGRRSSPSPRRSP
SPAATKTASGFGGTKRR

>A8PAZ4_BRUMA
MAEFRILAGSSMLKLATKPRYAKFVTADDLVMLSALGYDEEFEMRHRFFGKLNKHLMALQLHVEYLGLFA
LVSLYDDVDFQNKMRVLVDANITKRRKYMERSKMKNFAPYYQPEYCLAYAIYILAKLPSFESIKCEPELR
RLTESIWFLLGIFSARKEPGSLEFIYNIFQTVKNSTDSKLESCSREELQQKNEKIWALCDIGMLLMSYRV
KILMKDIQFKPLLSKRFFLNMGKANTKVYLPASFVKEMKTYKANTKHQKTDTTKAATKSFVRHSLTNNSK
HSTRVSSVLKVGSKRAVSKKRSNRNRKAKVNDFPISNETDEIVIKHPKLVSEEGKPQQRKMNEKKSVLEK
IFQTSSGSSWDLGMEVLIFSSYYFHDV

>E4YKC6_OIKDI
MAKGSRKRFNKSGRDQTVATVVTANNTLIDGENNESNDCNALILDKKTVSIKRAPILQPKLSKKKRKHLE
KLVEQKEKKAKRGELLEKLNDLRVPSSELARYHSIAHIGNKKQQEKSVWLAKRPDSEILKEEKINSIKGA
KQAKLEIDEKGEKEAESSDVSTDEESDSENGEKEWCKKKEETFETESIPKLIKKDEQESEESKSKKLLDP
KLDYVLINRPSEIQEARQELPILSDEGTVLEAVAENDFVVLCGETGSGKTTQVPQFLYEAGFCVRGMIGM
TEPRRIAATSAAERIRYEMCKTEAEVAHHIRYENKTTKETQICVMTDGVLLSQMSSDFLLSKFGAIIIDE
AHERSIHTDVLIGMLTRVVMLRRKRNIPLKLIIMSATLRIDDFIANKKLFPNLAPPVLKIESRQYPVTTS
FAKQTELRDYIAAAYRKICKIHREEPAGSILVFVTGQDEVKLDLDENLSSFDLSSTALDATLPMRALPLY
SVLPPSEQKNIFAEFEEPIRKVVVSTNVAETSLTIPGIKYVVDTGRHKAKKFSPITGVSKFEIEWISQAA
ADQRAGRAGRTGPGRCFRLYSSAVFQDFAKFPPPEITMKPLDDLVLGMKAFGIEEIKNFPFVTLPDEEAL
TNAEKLCVKLGALKRPHKRQVFGSITALGKTMNAFPVSPRFGRILSLSSQKNLLKFAIAIVAACTVRELV
DANNEKIKALKEIWADASKSQLGDLLILLSVVGATESSKEPETFISAIGVRSQGLKEVRKLRRLITHVVR
SMIVNEEEEKELAERKLPQPGAKECTLLCELFLAGFGDQIAFHSEKGEFKLPTGEKGKIYPSSVLAKKRP
NIICYQEKMENHKGNTYLKCCHEVTAEQIVRIVPEYVTFNRNKNSTPKYDANSGEIEALYTCYYTSTNTP

LGEHWKTLTGEDETLLRCFAHCLLIGEVFPAFKKLKTFWLSPPAVLLRTWANLHERTTNLIRCFSVHGLT
SRAAFLKKLQNEESRSEIAESLKEWLVPSHFVHIESIIESISE

>E4YKC7_OIKDI
MSNNSDTVSNISRDDLNESGYQEVRNSKRRVWVVAGSVTFVVLCTIMAVTLPPALKVEKTTSTAFSSTTT
STDATGTFSTLSTTATISIFSSTAEWPENITTTSKNNDKATFASTVLSSSTFPTTPETDTTTQALLPVTA
ERTLVQSTAITEIPDTSTTLKPPFMYTTSYETTTVSIVETEAECEFINELLELDICLLDQRYPLKHCNKN
NLGS

>E4YKC8_OIKDI
MASPQNDMSLVVLGSGGVGKSALTVQFHQQVFVQEYDPTLEDNYVKRHEVDGKTYKLSILDTAGQDEFSL
FRENYYKSGEGFIIVCSFDNRNSLDEIKKFFGDIERITEKRHGPIIIAANKADKENRKFDEEAVRNLCNQ
LNLRYIVTSAKTNLNVENLFTEVSLK

>E4YKC9_OIKDI
SNVTVALRDVESDVVIDASFTGQLLNDERTLNFNETFIFKSRSIETTVDIKSAFLQENLSLELTHQGLDS
DSEHMHAGLLVSFGDYKIGATIHLFKNPVMQILVSAFAQKKSWYLYEGLFKAYLPSFQQLEFGDALNVTV
AYDEDHESTWADNSFFIECSTRLNLEEDEDSKYYALSNHLTTKNIIAKNNQEVILDVEMWIVQRADMLRF
VVESENDRYLMLSYLLLSNDNDRFSTKLTTNLYIPYLVKLEDGYMLINTYKLLSSEHEINVDANIASVIL
SSDPAEYSQLPRSEYGFNLMQENYQKLSSEFKKDKEKYKIGYEFSYALDESQTFSNFTGNGQINFGNYLS
EGITLDINNNKNDAGLYGAYTSQNITIGFESEFDKKGIASTGVLSKVKDLYNTSSIPKIRKIGGWQWKFK
PESNIGSFQLFHPIELQTSLSKIAAVAAHRMIVNFRKYRRFGKVQITGKSKFPGKYNEADFVGNLDTQAR
SMNFTLETDEEVQTKIDFAVNDAIELHLVDNVFLPKYLRIDLFFISKILEKKRWSLRGRIGHDLAPVLDC
TSTGDVTDTTTVFHSKCKGTEFDAVGFDLQTINNNIQILAGIFGFDKELKLDVNLLDDIKKKMTLKYREN
DNELIKWKTEGALDLKNQPKAVISTSLINDKLTEIPKTIEFESGFRVNELQSSINRIDVASILASLRNFD
VEHWTNVAWNSEKVNFRPKIAFNLDDDAVSVRALLFLENTLKNLKQENGIEFAASRGGDSGEIFLQKLSE
ILNLKNSTNPRSRRSLVLGETDGLVKLNVKVAGVDHFIEISGNGRILRSGQVDMTSLFSTSMEFLEKFFG
PYLKLNVKGSLASKIIQIALDNRVKRQELELALETSSRVSENEQKIRLRNVKLAQSFDFLEKWPREISIR
GICQSTAKNIEADLVEVLLDDFSVRCDRLDVKIRKYKRDFVEALEIKTKEFDAVPKRMKVLITGTDISND
GYSADDSYCQLDFSNTTEISSEEENNQLSPEINLHLKEKIMCFFVAKLNLQFFKSANDFGDFESLGSGES
VISIANPSSGKTLFEASFHPDVGTSEILTFEKSFGHFLIGRDYEFVSRVFYQTRNFTEAIAYSFRHDFFP
RLENIGENYKYRTILIRKVDYNRKYYTPSYFDTYTELFAISTAQTKNDELAHLRLRLPSLQGSKDGYFIT
QYDNSNKIQFFVGLDKSRGLISFTKDLSKINLKIDSIYAPLKTFLFNYEKYDDMMFGLPFKCHLGLFIDS
ETIFKFDLINDLGISAIEFQGEEKRFSIKVSEESDTYSSSSQYFFTCALSSWISQYYGGAISAMVELNDR
EFKAMEFRIGDKAGFRKLLKASVIYDNQEEMKNVSSTINIYELSDEPMTFQFNILNNLDSLFDETERFEF
ITNIKAVPNLEVSDHLELELKGLGGVSKVTTECGLKNNENSVISSLVRCPRFFSRIGKIQFETENGPGFI
NWNVDHKTGKMVVPEANGQKIYDFDFEKKTVLFQNGNKSLAANWGDDEIFISNTFENSFLPFDEMKISST
EKGLLIELDSEETEIEAVIGDTENFSITIDNDGQISKIENSPENAFKIHFGDNFDIGVDSDASGSEFSWG
SETTEGSLYLGLGGVELNSRNKRTDEQFSTGMRCEITPLNPNCKAWLNKKFVEISSKINYQIDLNQRIKI
AAEILFEQNISDEISDFMRINTNAMVGIGKQQYFSTGFGFSEEREEPPTLSQIWSVTAKEELESVVSLLT
ANLTDSQNGFFHLLFGEDFEIMYQPKKYSAKVGIVGHDFKIGGIFNDEEQNHYFYKPSKYLSPWHVAANF
TKFISRDDYSAKLEVERKSQNQFVPFGGILEFEVEDSQLSVGNLKIRAANKNELETYFELANLDLFFLSK
SLASTNFGDLLWKNLNPQMNLNNSLLRLKLDLPCMEFILDSPIDDNYMFARELVNTVNCFGEPIGFHLGD
LAMIDDRVCYGNAEICRITFISDKLLDEFVASGFNENLTVAENFQDLLEHVLQNSIDFTIAHGGIKDFSR

EISTSLNVDSFFVKALILHINKILLDVEKAIDFIEENSSLSISPDSIKLTLPHPLFLLQVPYSSSLLSSL
TFLNDPAMHYFNQIASDFL

>E4YKD4_OIKDI
MKVLASIVASVMAKWSNNFDKLDNETQFSQFESCKEEVHAVIFSNEIENGGFNCEHLNDPVPYHTIICSA
SCDGDNYEHEWRTSRFSVQCKETPQVKTKFKGDQSCSLKPVDLCEEFYIEDLFPMGDFVLQRISKRGAHK
LARLSLQCHDSDKSAKATCMSNKGELKSKENLDNFCVPEPEYCEPPLVIEGSWEFIKTRKNGDKVFELNC
PNTDFDMRGKVICEKATGAFRNRHHTEDEFLNFCAPDSPSGDGSGDSSGEGSGEGSGNL

>E4YKD6_OIKDI
MINFFKLKLFIKAVSILISLALISKMTKTEDFDNKDTPTTEENKEKCATNISRMIENNQMNPDLQMVIKT
TKINNSPHINDLVYVVSLQIGLTKGTCSGVSVNENWIISSAHCYSSRVNIYFGQMNGTYSQIISSSRVIR
HPHYNSSSLENDIMLIKLSSIRVSNKYILQN

>E4YKD8_OIKDI
MALWDPEAAGNAAEYLRLPRLKMIDIQNAPFEGKTAVWVPYSETGYCKGYKMGEKDGKIEVKRLADDKVK
LFKPEDVEPQNPPKYELLEDMANMTYLSEAAVVHNLNSRYELFLIYTYSGLFCVTVNPYKWLPVYDNHVV
LCYYNKRRTEMPPHVYSISDNAYQDMLRACENQSMLITGESGAGKTVNTKRVIQYFAVVCALGSEKDKKA
LKGGGTLEDQIVAANPAMEAFGNAKTIRNDNSSRFGKFIRIHFQVTGKLASGDIDTYLLEKSRVTFQLKA
ERCFHIFYQMLTGHKPHVNEMCMISTDPYDYKWCSLGEIKVKSIDDREEFDATDESFDILGFSDDEKAGV
YKITASLMHAGNAVFKEKPREEQAEPDGTDAAEKIAYLLGVNPQEWLKAVCTPKVKVGTEYVVKGQTVQQ
VYYSIGAVVKASFNRLFEWLVVVVNRALSTDLPRNNFIGILDIAGFEIFEFNTFEQLCINYTNERLQQFF
NHHMFILEQEEYKREGIEWEFIDFGMDLQNTIELIEKPLGIMSLLEEECIVPKATDMTYRDKLFQQHLGK
TKSIGKVKKQGKFEAHFEIYHYAGTVAYNVTDWLLKNKDPLNNSLVTLFKNSTLSACKKIWESYVSADDA
PKGGGKKGGKRQESLLRLMTNLHATQPHFVRCIIPNEQKRPGFMDNNLVLHQLRCNGVLEGIRICRKGFP
SRVEYSDWKQRYCILNPNAVPKAGFIDPKKACEKILTGITAIDPAVYRFGHTKLFFKAGIIGALEDLRDD
KISEILTKLQTRMRFNLQREAFLKTIKERDGAVVIQSNWRAYTTLKDWEWQKLLFKIRPLLNTAEKKAEF
DELLKEYEEMKKELEVESKRRTLAKVEKKEKKALQEAHQQALDDLQAEEDKVNSLSKAKNKLEQQVDDLE
QSVETEKRSRLDLERLKRKLEGDLRLAQETIMDLENDKQPAKAKSEKSRSDLSRELEELSERLEEANAQT
QGQIEVNKRREAELAKLQRDLEEHNIAHESTLSGMRKKHADTSSELTETIDNLQRVKSKLEKEKSELKME
ADDLISNVESLTKSKIGYEKSCRQLEDQFAELKMKHEEQEKAISDSAATKARLNTEYNELRRTFEEKEQI
NSQLMRQKNSVSQANDEIRRSLDDEIKAKNELQRQLSKSNSESAQWRTKYETDAVQRTEELEDAKKKLSG
RLSEAEESVEAALAKCSSLEKSKGRLQSEIEDLTVELERANAAAGALEKKQRSFDKILEENKVKQDEINA
ELEKAQKDSREASNEVFKMRNAYEEAVDCLESSKRENKQVQEEIADLTDQVAEGAKSISELEKAKRNIEV
ERNELAASLEETEAAVESEEAKTLRITVELQQIKNESDRRLQEKDEEMDNNRRNASRTVETIQGDISDIE
IQLAHANRQLNDAQRQNKDIMGQIKDAQMALDESERIYDEVKEQTAVTDRRVNLLQAEIDELRSAVEQAE
KGRKAAEQELMEANERANLLHTQNTALANQKRKLEQELLAVANEVEEAIQEAKNAEDKAKKSILDASIMA
EDLKKEQDASAHLERMKKNQEGQLKELQARLDDAEQVALKGGKKHVQKLEGRLRELESELDNERRRGVDS
QKAVRKMERKVKETVYAGEEDKKNLSRLQDQADKLQLKVKQFKRMAEEQEEASTQNMSRYRKLQHELDEA
EERADMAESTLSKMRSKTSAF

>E4YKE0_OIKDI
MAANDRSPQWLEVTFDSDTVVSGIMIQVSEVNKWLEHVNGKRDFPEENNLTNFFISYSQKKKPLLGAGKW
IRGESFETNYKWDAQKGISYHAFKTPVTCRRIRLNCTKTLGGWCPAVKIEWIG

>E4YKE8_OIKDI
MKVFIISAASAFSTAQMRPCDIAIQECSLNADCVDWPYCTEDGLYFTGPVCSITGSCSCIDRLNGAVSVS
SEGVELTTQVFLPADFFGSWMSDNNFSCDMFSSDASASASLAETVPEYVLSQIDL

>E4YGL4_OIKDI
MAETAGFFGFDTALPGDSSAWDEDEASRSSNAKNDETFGSSETGATAWPKLGDPRNLVSKLDRMSVNNSG
GFPDDPAIMSLQEKPISMPDRSAQLQNMEKIWARPQQQQQLAQSNSLQNMEKIWNQAPPPSSVAPSVSST
IAPPPGFGPPPGFAPRPDAVAQQVNNVANSIVGPNLTGPAPQTAGVGNIGSGAVQQMQRGLNVEELERQH
FAEGLDALSRRGTLVKDAQSLEAVQVVAAQQIRQPPQGTIGQLHPEHRRALERNKNRNDNRNNRHNQQRR
RDPYANLMSRREREWITSMQLRALEIKNPETEDYYYVNYMKRLMQKGNAKNQRELILPAPKENNRNRKRN
DSEKESKKEGDEDKEKEKKERKPWSEGSLGKPVSSSGKSIYEYLIIKLNC

>E4YGL7_OIKDI
MPPQRFSVFLERDQIQRDRDEENRLTEAQRNKELHSQLEGGGTHYVTAHHLHGGGGGSTGSVQTTGSESG
HSAHAHFLPREQNQILYESQMEVDVEDVRDLPSYSFQFGLHEPSRRPFPLPIYPAPSTAPTSSQSTMDRS
QWTDATGSLFPNSARRGSFDSSISPATPNTQTKYQRHYKSINVRPDKRNLLTYHQRRAELSNPYHSSTSL
SSRPNIDQHYLSASDLTGPTAPITDLKKRRLMLHELKNQLGDLKDTNATTVMDQIHAENVKQGRDKYKTL
KQIRRGNTKSRVDQFEAL

>E4YKF2_OIKDI
MSQSKGTISGGTVSKIIKDPTLGNIQTKALGQSGFQQNIPTKASGFAQSYDIDVEVETTQTIHITNVQII
QSEVSIVES

>E4YKF3_OIKDI
MADRTYGIVGMPMNNSSSKGMTRDRKDQLDKNTLATGRYTVKSKTKLPFL

>E4YKF5_OIKDI
MKNKLFQTTITGRLELESNFDDGKEENEEQVHPAFENILLLREKILLCKAKILELKDYMSAIFFALCGAY
VVLASWIDFTRAFPLFMVFLVYLAVKFYELSKEKIKERLAGIDRTKIRKISAFGGIFLMLGIYAETLYTT
EYRSNVITASFGILVFMFISMMMCVSIIKIVWRPILVGLMLQFFIAAFVIKTEIGFAFVEICRSKRFLRF
ADAGGIFLFSGYKGDLTNFVYGVCPVIIFFSSLISMLYSIGVMDFVINFLSFALTATCNCTQSEAVACVG
NIFVGIAESPLLIRPYIDHMTYSEIFCVMTSGFSSIAGSVLGAYLKLGIDARALITSSFMSAPGSLMVAK
LMFPEVTPSKLTTKKTEQDKSRKSRNLLEAAVQGAMDAFPVCFNITAMLIVFVAAIECLNDIVVFMCGLF
DIHDIRLETIFGVVFRPLVLLMGVPYFEADFCARLLGEKIFFTEFMAFQSLATSKGYRDMGREKCDCDGN
QKWLSELSETIMTYALCGFANIPTMGIMLGTMAGLAPKRKKVFSKILAKTCVAGTICCLIRGCIACIFVR
ISFLPDLQLSFSSSLPVILNSARSTFTSSILNLSQVSSFFRKKNLQNFRSVQHFLPRRKSVHEF

>E4YKG1_OIKDI
MFSEEELLGRIEELELTIDEIRDEHEIQLEKITKKLKDEEQTRLRKYELIEKKIHRVAMICEKNRKDYCQ
ARENYTHERDRAAKLGT

>E4YKG3_OIKDI
MMEVVSPIEATNLSKNFLSSEWMGAKTKSEVNPNECENNNREEDRKLFVGMLSKTQTEEDVRRLFGKYGA

IEECTILRTPEGSSKGCAFIKLANVQHAQNAIAQMHGSTTMPGASSSIVVKLADNEKERALRKMQQMAAN
GMVSPIMNYAYPQYDIQSIGGFANNMWAGGIVAPMGMQMVNQMVHGVVPSAMMPQMPSAPQSPIAISTTG
STSADSAQAHAQVQVQAAAIAQPQPATAYSLQTAYDSTLAQYQAQTADLSTLAATAAYGGYQFVMVPNPA
GGYTQVPLSCGGPSMAALNQNPCAPQREGNFHSLVLHNFNKLLPGPDGCNLFIYHLPQEFTDADLANIFA
PFGAVISAKVFIDRATNQSKCFGFVSYDNAPSAANAITSMNGFQIGMKRLKVQLKRPKAGDKNF

>E4YKG7_OIKDI
METRSRPIALNIIKTDFADDDMERHDFVELDLGIEGSYGSEGGSTLVDDFTGETKFHYKDFGSIDWYREL
AIDQRKRAREKEKAENSPNIVNYVRSLADAASGWIIVTVIGLLAGFAAGVIDIATKWLSDLKSGYCRGHL
YLNQEQCCWSDNLHGIDEDNCTAWRDYGNPNSYGGHVLLFIMYCLMGTLFATIAVILVKMIAPYACGSGI
PEVKTILSGFIMKGYLCFGTLLVKTLTMPLAVSAGLMLGKEGPLVHVACCCGHAVSQFFPKYRNNQAKLR
EMLSASAAAGVSVAFGAPIGGVLFSLEEVSYYFPLKTLWRSFFCALIAAFSLRAMNPFGNQHLVLFYVEY
DKPYHLFELFPFIILGILGGLYGILFIHMNLSWCRFRKRSLLGSVVILALGTSILAYPNPYTRIQSGHLI
RLLFKECKRYDDNPLCDYDYSKNATTVFSDGITQASWQLILALIVKSALTVITFGIKVPAGLFIPSMVTG
AITGRLIGILTQKIIEAFPYSPIWEGVCADPTSCIEPGLYSMVGAAAALGGVTRMTVSLVVIMFEVTGGL
QYIVPFMVATMASKWIGDAFGKDGIYDGHITLNEYPYLDVKIDMQQQKVRSIANNWTLTSIPFQGSTTEK
LCTMLADHPYSGFPIVTTQGRLVAFITREDLHEGIAAGRMLISDDPNDEFIDQTVLFTMSTPLVSAGRVP
IRLNNVIDYSPVTISEQTPIALVLEMFRKLGCRQVFVIRQGELVGLITKKDLLQFIYETRNNDQTSYSQF
K

>E4YKH5_OIKDI
MNRISLVNRKIVRWSSMSTLNDFIRARTSRVASVEALTYQNMTPLKVSSEQIQLEYKLNTELDLNPMGNM
HGGRMAYILDSVMSALASDFDKTDVNSLNFSVSYLNGISKDLDSFLVTAKIVKGGRAMAFLTAELSSKAG
EIYTLSEGSFAKCA

>E4YKH8_OIKDI
MDQPGWTLTAPGPSPAPGSIARQESVIEEMVMSMAEAYKSAQQGKVPSSPATSTPPAPSPENQQYHPLAA
ANLHQQAAPGYLPTPYQPQPQPQMTPPTYPPQQHYATSPPTQYPYQYGAPQYGDPQYGVHPQIPPQQPYP
TPALPQNPLQPYPPQPSYQAAPQHPQQSQYQQYPPRGYAPAPAYPQAPPAGYGYSQPAAPYGYPIQQQR

>E4YKI0_OIKDI
MYKFIPPAIIAFISIYFQSWIGSFLVVVFLIGIERRKKNVIAGIEEKWNPTNELTKCIKGGAPWMFKVNA
SCFGLLTSYHESINWKSIIRCSIEYRETKIFDYLINSSQSNKLLYNCFVENLATWFKLTVHSGDPTLAKL
FFDVFGSFPEIRTLPPDFAVRPRQSLADNGTLIILKKQAIRAININFRVKNYSSAIRSCPLGDANGSTLA
LSFEDVELMLKEAAYFESRHRFKIDKMEIYFKTSGKTAVTALFSIINYLLQKDSETFQNLAKKYLQILEL
FLKLPLEIPEDIKCSDVLDFLRYSENTKIWKTDVGLSVFQLTQLWFGNRGNLQFRCKQVLLKSCKIENLE
KNTDIPEIIREYVTLNTSTENLVL

>E4YKI2_OIKDI
MSDWGGFSDAELRHLRSGGDANPTSAKPKSTASKSSKKALAAKKTSKPKPAKKLPEEALFPTSTSSNAAK
KETKEEIQVHKIDTSSKEDEMPDFIEEEVVEQKKKMDIMEVEKAQREMEEKNKRRRAALSQEILSRQKKA
ALESKMLATIQEELVKLDNLLNADVAVLRDQIDTACFEFSEANRRFQTAEKEYVDSKFDLQKKTETKDSL
TEHLMNLIQANEERKQKKLEELTFKLSMDPEIVAKREAEAAEKLKLEEEAKQKAIAEARKKAAEEAENAK
EADATAEIKEETTSTSSPAPQEISREQTPEQDLSEPVADTQSS

>E4YKJ9_OIKDI
MNRVAKEGFLKCTWGNVAYKIWNKGAPKRAVFLHGWMDHLSVFEPLISNLSNSEIEILAVDLPGHGHSDH
VSWPWNYSHSDLPKYMIEILDRMEYRKYHFVGHSFSGNSLTPSLAVSSDEVQSFTILDAHGVITMVDDMY
LFTQRKALEGTYRSKRVENPKQISREELKKRLLKSTIPLEFQDLWLERGVKWNVERTHGHFARDIRLNEN
SNLLDHLKMPILRINGKESDPFGGVDPNNPAMALFDIKSVQRFSEEISKKENVTEFHLPGNHHFFLPQAR
ETAKILEEFWSSVE

>E4YKK1_OIKDI
MEGLVKWAQALSDKEVVSYLGIGMIVNGLFVAPILTYIMPAYYGRYSGKSWFSISAKLSWLVQECPAFFV
PLYYLLTTPALELPSQMLLGMMILHYFQRSFIYALMQRSKNNVAISITFFAFIFCLYNGVMQGLALSSVY
QYPASWLSSPQFILGSFLFSLGFLGNIHSDSILRNLRKPGESGYKIPRGGVFEHVSAANYFCEALEWTGF
AIASWNFPAAAFATYTVANLLPRGLSHHQWYKNKFDDYPKNRKAFIPFLL

>E4YKK7_OIKDI
MKARKISNFFFKSDLFRENSESTGSLARVPSHRNKKSKHHKKKKKSKKESKFERLEDAVERHQELRLEGE
EPIYTDDDIADGRVLDNPMNKFQPMVGGELDPEIKKRIEAAQAELSMEAPEEIIKSSRDEMVDEGIEEDE
TTTTTSTTTTTTSQTTKKEDTTTAENEDTTTTEKDETTTMENEENEKELENVEYENEKVDEKTTSTTTEE
PTTTTESTDSESEPEKVSDESRNEDSEERIEETEETTSSTTTTTTTNANEAENEDIARETTTSTTSTSTS
KTAISPDNNQSKSEEKEELIDINDDEVEKTIEESEPDTKTDQDNPGQSIEAIKNPGELTLGNNVKKVISP
KKEEESIAAKQKAANDLAELMKRKAMAGHAQFGSHDVNNFEAIMPELRTINPVNRYEPYDFKKGTPGEAD
IFEKREKVKEMTLHAWNGYKLNSFGESEVNPLTGQPVNSELFGKAHTGLTIIDALGTLYLMGLEEQFLEA
REWIENDFKFDAEAIPAIVSFFESNIRHLGGLLSIYQLTGDQLFLEKAEMVGQVLEKAFKGEKGMPYGRL
DLPNGSGMSQGWVPGGCFVLAEIGTISMEWVTLGMETGDLHYKELAETVQNFTREHVGESGIYSNFVRPT
EEGLEGCSSAISFAGCADSYYEYILKFYLLGGRKDDLQLSWLEKTVKGVEEHLVLEQKDKEKYQIMMVEG
QQRRNPKTGSFDFSKGNRMGHLACFAGGFWGMTSQVLTDKSERFMELAKGVTQSCRDSYVDSPTNLGPET
FSNTGASNPGNDYYILRPETVESYFYLWRLTKDEKYREWAWDVVQALEKHCRTEFGFAGLKSVKTGQKNE
IQESFLLAETLKYLFLIFSDDELISLDEFVFNTEAHPLRINPSIHQ

>E4YKL0_OIKDI
MSDREPLIREEDDQEIISEIPEIENETESQTRTFTDTGVETETRLERNLGLYDAVGIGVGIMLGSGIFVS
PGGVMANAGSFGSSIIIWVLCGVFSLLGGLCYAELGTLIPESGGDYTYCNRIFPDIIGFLRLWVEVIIIR
PGCHAAVAVTFALHVLQPFFPGQSVPSIPKKLIAAACITLFSWLNMYSIKGSTMVNSYTAFAKTISLILI
IGVGIWKAGQGEIQNFMPDEFWKGSTTDFPKLCLACYSGLWSFAGWTDIVLVTEEIQNPAKNVPLAIIIS
CTLIIGLYCLVNLAYFTVLTVKASAIATAQVFIEKALPNLPGHQYIIPILVSLACFGGVNGSLFASGRYY
FVAGRNNHMPPILSMVQIHRNTPSVACFVNGLLSTAMLVNDDIYSLINYTNFIYFVCIILAIGGLAYLKI
TGQADGSTVKVPLPILILTLIMFSGVVLGAMVLTPYETLGGIILTLTGLPIYFIFVQPRGKLLEWKQNMQ
PRYENFSRFIQKLMVVVPETKEE

>E4YKL4_OIKDI
MEILYFLSSIIIGYYLGCGIQDVRVEKCNVFQSLQETRIVQEPKKESKKKIFIAIHSTPKYLETRGDAIR
STWLNEVDPNIATVKFISGPVAGFPTLTLPGVNDYDYPPQKKTFKLLAYFYSIAHEYDWFYRVDDDITLQ
FDNLIQLVSKLNANNEHYIGGTGFGRNAEDFIPSGNAFCMGGSGVLVSHALVRKIRPHLSTCVKNLMTEH
EDVEVGRCIWTHLNKNCTTSYETGQIFFQNWRRDALPGEVGYKNNIDAEKDLKIKTLDNAISLHAIKQPE

SQKAVRIRILKRRMQKLISETGVMRSIVSKNLKNRRLNECLWDEIRNVNNEHTPALYQNNVIPGMKLHRP
WPETLRKTDSFVPGTSLHSSLYRCLNTDGFSIIRPGMDNFGWKLILIAARFSEEIVVKESTNLLVDTQQR
PLTLIVPLYKREDAFMRFIKLYSRLLKTHQNLNFIVAIAGDDIDRQNIVEIIEDKIPQTLFNTHVRVKTC
KLPFDKAHCLQDAIDLLKEEDLFFVYDVDIIVTEQFIQRIYFLGHDNVYFPTLFSQYELTDHNSQNISEV
NGFWRSHGVGPVAMTKQTYLKSSGYNQNIHGWGNEDTELYDDFIKNGIQFVRSHDEGIFHPWHSKNCSGL
QSETGQYAACVKINKEHTMSQTRLATKVLDIFTFNKIK

>E4YKL5_OIKDI
MGGKLSNLHGSASSEPKDVYLDITIDEIPAGIIEIKLRDDIVPKTCRSFRALCIGERGLTLKGSKFHRII
PGFMAQGGDITKGDGTGGKCIYGSRFKDENFNLKHVGPGVLSMANSGPDTNSSQFFISLDKLDWLDDNHV
VFGEVKSGMDVVRLMEKTGSQSGGCSRLVEIIDCGVL

>E4YKM7_OIKDI
MRLLGLVSLLSNSVLNARELLVITVATEKTDGYLRWEESVRYSGLKSRTFGTGEDWLGGDITNGPGGGHK
VNLLKKELAGYKGNSELYFLFTDAYDVIINGKEEEIFSRFDDIVSKVEYKTNVLISAEDLIWPDASLEPK
YPLVLGKRFLCSGAILARADVFLDLLEYRAIGDRDDDQLFYTEAYLNKELKEKFGIALDHKAELFFNLNG
ALEEVGIDFARSATGDNTVENTKYRTKPLVIHGNGPSKNELNRISNYVPQGWRPDYGCPACSKVLNEEIK
EDIDTSKEIVIAFIIDGITPFVHNSLKRIASLDYPAEKTHLLIYSNTVWADERVDTFLEVFGSSYKSTKF
ISSKEKMSVTMARKFALQKTYEKFSVEFVFFVDGYVQLTNPAVIGELIKTNVELVAPGMSRYGKLWSNYW
GAVASDGFYSRSDDYLDIVQGTRVGIWNMPFVNGAYLVHKNLAADLIDIFAGISQSPWQGKFNDPDLDFA
SNLRTLGIFMHVTNQAYWGRLVDREHMPVDRIHPELWQPEWNRPDWEEDYLDSDYWRVLEPETEMDEPCP
DVVAFPFLSSKGGFDMIEEMEHYGKWSGGNEAHTDERLAGGYENVPTVDIHMNQIGLQDEWLYVVKTYAA
PMVSKFYTGYNPDNKPNLMFVVRYKPGEQDRLRPHHDSSTWTFQIALNRPNIDFEGGGTYFTRYKCSVVG
SATEQDSRSLEVKQGMGFAFPGRLTHQHAGLPTTKGTRYILVNFMDA

>E4YKN2_OIKDI
MTKQRDKIALCPAEIDFGLIKENRIERETIILGIQNVSKSSIRVSILFPEMRWLLVEQNWPVNQPVPPGL
EVNAVISIDPNHFDFAAFPFQLGDLRIKVDDGIVSFPLQATSSRPILEIEKSLNFGKVSDSNNIIKKKLK
ITNRGNERGFFEIIKNPENEAVQFSSYSGFVEPKNEIHLTAKMIADCRKEINEEIKINLGDRTERVKITG
AVISRKLRLNTEILQLGDFVASAQGRETVFLENPTPDSIQWISKIPFEADGVEIGPFESANEIIQIIPSS
GTLLPGEKCQLTVKVESKKKLEKFTELIFKVVFGVVGKAFSESSTVGLFVTANLFPLKLKISSPAKNVKQ
SSKITKIYFGEAVLGSSPNVILDFENKTGIEINLQSTKTAHFKVSPKNLIIGPDETKKVTFTMDPSQIGN
LSSRFDFLVVVGKTSKVVEKAKFLLEGHVKAKKADLHKTEEKIKKKETLAALKPP

>E4YKP0_OIKDI
MGDPRPDIPIPQGLQQLMQDFVVTVLREQPDDVVGMAAEYFTKLKESREEMSSGSKVNFEQPEEEDDEDM
PEDEEFLRRMREKQKYGRRKSVSAEGYDPDKDDEDDDAEERIVNPKSDEQRNRLIAACQKVLLFNRLDKD
QFNQVLDAMFEHVCQPGDSVINEGDDGDNFYVIESGNYDVYKVINGDNLKVASYDNKGSFGELALMYNAP
RAATVSSVDGGTLWALDRQTYVRIIVRANAKKRRMYEQFLQNVHILQTLEEFERQKVADAMEPKEFEDGE
IIIQQGDTKLDYFYFVMEGEVKYVLTDDDDVEKEIKRDTAGGYFGELALIQDKPRAVTVSSVGVTKCGCL
DVQAFERLLGPCKEIMKRNVDLYAKELEQVSSKQPAEASSPETSN

>E4YKP2_OIKDI
MKCVILVAGHGTLLENEIREKGPKELVGPAVGKKKILDIWWKIVNQRTLFSDVYLVTNADKYKYYERWAT

ANDFPVSNIINDGSTSFHNSLGAAEDLQLVLNTAEISEDIVVVAGDMLFEEKSVDMCQIIQFYRRFTKYQ
YELSVWYWRKWSRPQRFAVYSVKNMYKFWKIEKFILRNFEGFYN

>E4YKP5_OIKDI
MSLPPEQQITNFLKENPLVCSILEDDVEKMENILESNPNLNVSKQDNHKQTPLHYAAFIGNENTCVTLIR
HGAQIDVKDNIGLTPLHRACAADRAQAVEVLIENGADCSIRNRNWETAWHIAAAHGAVSCLQKLLSKTGN
VNIQDKCGRSALHHAAVKGQDQVTEFLIENGINVANCDRQDRRALHWAASAGHSQVVELLITHGADVNAK
DAFSNTALHFCARNGYQMVIEQLIHSNSNLDLQNSNGESALHLAAKYGHAECVDILLKCGARTCRADANG
KTPLHCAVQKKAAKHVEVLLQFENHKEEKAVNLADELGNTALHYAAELDDFDLVQRLLQNRAQPNLENKL
GITPFHVAAKMCSKDIINILLQAGAVPKVDKKERSILHFAALGGRIGVMDILANIDPDRASYSQFSADGP
FSADEPFFGGRVIFGRREIFGGRAFFRRTSDFRKTSHFSANEPFFGGRGCFRIRFSSVRSSVRANFLNAR
PVFELERVDSSGKSVLHYAAIGQKVNMVANLLSLLPKSFVNKIDKDGKTPLHYAATNSYFLHVQKSSKKS
AEIDNSRNGVFDHEQQSAAICEIFLQNGASVEIRDKNNLTALHCAAASNNNSSLEILHEQDHSVTNISLL
VVACLQKCIAVVEKLLQDNPDLILARDSTNKGEFFSRVAISDRQNRRNLVSGKLXXXXXXXXXXXXXXXX
XXXXXXXXXXXXXXXXXXXXXXXXXXXXXXXXXXXXXXXXXXXXXXXXXXXXXXXXXXXXXXXXXXXXXX
XXXXXXXXXXXXXXXXXXXXXXXXXXXXXXXXXXXXXXXXXXXXXXXXXXXXXXXXXXXXXXXXXXXXXX
XXXXXXXXXXXXXXXXXXXXXPKKFANSVENINRFF

>E4YKP8_OIKDI
MDTVTEWEMAIAMALMGGIGIIHSNNTAEEQASHVRRVKKYEQGFINNPVTLRPSDTVRDLLETKEKHGF
SGIPITESNEKHSKLLGLVTSRDIDFLKEHEHETKLEQVMTPRSELVTAPTSVTLNEANVILMKSKKGKL
PVLNDKEIHKYPHLQVIGGNVVTQNQAFNLIKAGVDCLRIGMGSGSICITQEVCAVGRPQGTAVFRVCEL
AKKYGVPCIADGGIKNVGHVTKALSLGASTVMMGSLLAATSESPGEYFYQDGVRLKKYRGMGSLDAMKHK
ASQSRYFSDKSQIKVAQGVSGAVQDRGSIYDYIPYLIAGVKHGKQDLGIKSIREMHKCLYSGELRFERRS
AAARGEGGVHGLHHFEKKLY

>E4YKQ0_OIKDI
MFSVAERPVGIDMGGIPMWDRNQAIRWLQSLDLGQYAEKVKDGSSIVSDPEKSLGMTNSFHKKKLRLAIR
ALDDELKSNNLGTGWVCNWLEEIGLPEYKKAFHEHGIDGRVLNLLTMEELLALGVTSQLHYLSIKRGIQV
LRQSKFNPHTMVCRAGSCDQIERWSIFRIMDWLRTIELSEYAPNLRGSGVHGGLIALEPGFGHDHLANLL
DINPKKTLLRKHLQTSFNQLLSDQQFETKRDYILRNGMPMPLTARLKRKKKTFFGSKKSAGSGGDDELVC
PLNLGRHDDPSRRPSHGSKT

>E4YKQ4_OIKDI
MSRRTSQATSRASFKSPPVQRDPNMKETVISFNIKGGQNLSATKDGSEMFLRLSFGSFTAESEYIEAKTE
ASNVKLAVKEEISGTNTAFYDNLVRFPLTISLLEMLPKEKKQKEDKIICHREVHRDLSPFLKGKRKISES
VREVSKEAPNIQVNLEISVSISPELKPEFLEKNRIMTLRLIHASGVPDSWLGSSFNACLPIQKEENETAA
VKFSGKAEGAFNTSEKMSLYWSGLKNTTEKIISKENYKLPENGAAPANSDGECPNAIVFSSERKMIMSAD
DCRAFEARIAKVSNLLPLEISKYFHPFRKDAAKKGKAKPDDGELHNHGIALINLESLLYPGVKTVSGKFK
VNPFTQNQLIRAGYGESITASSCNSGTILSMGSTSRNDSSSAGGVSDNRPESRQAAQDALPYKELNSSVF
FEISFSRALVDRATLQDLDKNLATLIPMRPKIPRKKYGAEISVEEYKSQITEVAKLVLAEIRAFAPSTKG
ESFEKSLLYQLNRTGKHAQFHENF

>E4YKQ5_OIKDI

MSGDFKRVTINFFFIKKSGCGDGSGSFDLRLNSFSSIYEIDYYESLTYFEAEKFCQIIGGTLLEKHSSFF
PFDLWTPKRDGKCVKIRAFGGGEIITDCEESLRFACKKQKCQLLSYVELDGFFEIKFDEKCRDKFFNFVK
KEEFFIVDERTGSRTQIKEIVTKYWPDDQTKPVNLRIVHETELDGKSYSLIDDFSIQSKTIQKRVRRFSV
QAKSSSSHVYLSKIYLLLFFLILIRFFNL

>E4YKR1_OIKDI
MKNIIFESNSVGQDIDGKISNIENFAKEFKIRKLIEEGVIMAVMAGPVEKNPVIGFDLRIKRIESQNCFF
KQIKGFIVQKMIIRAIQEALKRANCIVKEPRMKFQLSLYSIEHRGKVMELLAENDCEILSEDPEGEMWSI
SGLLNVSQELKIQKRLLAVTRGNAYLETSYYGL

>E4YKR2_OIKDI
MCGIGVFVNCEPDDQTVKSLKRRGPDFYHQETFTVAGQTVTVIATVLSHQGEGIIAQPIVTEDFIFLWNG
EVYDHPKFEPEKHEDTNWFFENVASHMTGENQAGIEIMKEIRGEFAFCTIDRRSGEIYFGRDRLGRRSLL
LYAYDNQLILTSSSSGYHFIELPADGFYKSNLQRMNKLERIAFQAKTKVDEHLLEEFVFSNASNDMLAIN
GVKEGKLHSLPVQTMKTIQCDLWNFDQNHGGQCTGDLLERLEIVFRKAVERRVMCRKGSIGVLFSGGLDC
SLVVATVLEIFRDKPKTATIVYLYSVAFGDNEDKCEKCPDRLTACSAYEEIKRNHPSAEIILVKENISKE
QLTKVRAEKIKHLISPRVSVLDDSIGSALYFAAHSQVGSDCRLLLSGLGADELFCGYSSHRRVHDREGLD
ALEFEVQSHIERIAERNCARDDRVISDSGREYRMPFLDLDFIRFSSSIPILDRCDLTKGRGEGEKQLLRQ
LAAKKGLKIISSFEKRAIQFGSRIAKMEDKKVKGNDICANLKK

>E4YKR7_OIKDI
MKSFSKSVAVLATVALAQTGDEFENYNLNLEEIADILGAYDLGEYGNDYGTNYGGQVESSAEDAPVTAEE
LLNVDYNTVETTEVIGEYEEAIDGQERRPLANYATFEEKINIAKPDHNFCRTCSGVSYGTCIGNDAYETC
NDAQDVCEVKYRFDSAGEVLFWSGCKQRNACLQDEQGNFVGGRFFLNRCKSSKVPNRWLAGSECTFCSLM
SPTGTHTSNILDDATNIVKDISSVTHSLLPAITKTQLLENPEDYMDASATNYILGGQSYV

>E4YKS8_OIKDI
MKLLSTLLGLSLAQNEEERSLFGTEEINFDEQNRFLQEQRFAEEIKGLISGSLSCIAEDARIIGGNEADK
LTWRWIAFLHKNNCGGTVLNSRMVATAGHCCTWDFNRYPEKYEMAIGRHYREENAPRNIAGPYSRIYEAE
KVVQHPDVNFWTFENDMCLIFTKEEIEFNPGVAPACTAEESPKLGKLCYIAGWGAQSTDGSSASEVLNEA
EVEILDSETCFHNIQTKLREVSPSHSNFVVPDSWHETMMCAGNWAGGVDSCQGDSGGPLVCIEDNRPILH
GVVSWGMQCALPGLPGVYMRVSNYSDWIAAEYEKFVNGDTDSSDICDPGQIYFDDVKGFGSWDCTDEMIC
EWVCNDGGRTGVVTTCDAGDWSKPSKCSILNRCKSSLKGGDCRKCHDADRPPPAVGATWDCSGSGSRKTC
HLVCDGSKNGQSTSCDRKNSAGWSDVNASCSQPSEGENTTPSVPLDLELLGDGTFEKEAKKKNWQGHLQF
KTKFDERKNKATAYLLCEDGETEIKKIKIDCTKKVKIHKKTGVETSSLKCKAKPYAQKHVDKAC

>E4YKT1_OIKDI
MHRNDFTTIQKDGTIFLFGGYSDLVQEYTHRIWKYLPESHSWTIEGNMLVNRMNFASALVNGNVFSVGGF
YELNHESYDFETGLSTALDTTLESKLFVELVPFSFLGC

>E4YKT4_OIKDI
MEKIKNVTVVSRNGTIFEGFTGRYTSYEETETLCLSKGAKMPWFEMIGSQFSEPVWIKSSADFMKRTINA
SDEIGREKLERLMNEFLDENAPLELNASLLECAKNDEELKKILPKNLDLFGRKGENNFEKFASENPLVDF
KRCFQQHVSAKQNGLSIRSKNPVSSQFISSKNRPKNYQRINFLSKIRSQKHAKLPKNILWNDQFWNTFQI

QAHTLGHLDQVNIITYDQTGKRIITGSDESLIKIWSAVTGMLLMTLRGHEGEINDLQVHPDNNLLASCDD
KKAIRIWCLRSGATVTCLMGQHTAHNITSISWGPGMLCVESTYIRPLVSTGNDGTVVFWSYDENDKSFIL
EAPPKFTERTKPGDRAVQQAWSKGGRLIAVGFSDGRIRVYMILKPDSPRGVDKIAELDVHTSCVSSIEFN
QMQSTKTPPALLSTSFDGFAIIWRMRNRRWKVQKIDCVRETDPNADPKKRPKVNDGIWLKNDELICVALV
GATHQNIIKIFNSATSDLVTTLCFHTQTIQNLKAHPKIPHVFASSGCDGLIVIWDVLRSKKIAEVYFPST
IESGEQMSIHDIRWSSDGTKIISADSQGHMSISGFGKDVKLSQLPDELFFETDYHPLIRDREGFVVDEQT
GLTPHLQNPGMFTNSQNEPYLGRVNDDRTRRQMAEEGQRDVLGDRIRQLDLEMTAAEIDQAGEGGTPMNV
DENSVHLDMSVLTFDVQRRSQSIIKKPSWCNKDFCEVLPLKEVQKRDSLQKKLSKAEERWFKKEGLDCDV
MEIEEIHVTEDSVPQINSSQRRANRRRQRATDVEEPELPPESDEDHSIQGSDEEFNLNSSAEQRELTRVN
RRRAPRGYVPVDDEPAEDDSDVEEASSSEESTTQENTSSEYVPTLEEPSTEANSTRSRRARTSREPQAPA
SSSRQRRKKRIILDESEGENEQANLDSSLEENKEEEQVPGPSSRILIPSSTSSFRKKVAFSESDDSDNSM
KKSDTSSIDPLVQMSNSKKRNRRRERTNKRLSEDSSSNEPHYHSLIESPGRGHLQWIRQTQPLRCPFIPQ
IGDEVYYIPSAHLQFVKELENQKLTSLRKFHRVPTQFKNSCLMDMDILCVVEELEIELMNEKKFDPPARW
CSISLKCIHDEIDLKKFTEALKLGSPK

>E4YKU0_OIKDI
MKFTAEFEATFIFALEVFLAKILVRVILAALLSVNSTRSPSQLVSSNADRTFFKASCHLEKDAAERAFLA
FTRI

>E4YKU4_OIKDI
MIRCLFILTNTLAFLGGTALIGLCAWIIVDAESIGIEANLITDKLQEILDDVYTQLNEIWEENVDIPEIF
EEIFEWIGYATYIALSAGVLVAIISQDFQICIFFFKKNENILLISEKFGHFLMNLLFFRQF

>E4YKU5_OIKDI
MKCFSNLFLPIFIQLIDPSYGAFQIFGSGGRSSANNVETRPFTPVRADVKREFGIKQPVVIERPPTRAPA
AAPAPQTSRSFPSRSFNSQKEPSSGPKFQTSSGPSATSLSKIAKPSTSGGWKPSSWSSARKPSSPHHGPS
FRELGKLGGGIEVPRSSVGNTASSSFANRNSNSFSFTSSVSEETGWKGISQSQPTASSFDRLDRFMPQEN
IRRPEAVNTFQSREPQGLPSWNGDNLWDQGRTPAPTGPSWRTTRATIAPTTQSTRLYAPTTRSPISFQTQ
KFQFDSNSRLESARKQLLLQSQQNERMSESRSSNTFLEQYSSGPQQSAGFSSNGKGDRSLLNYQNSFRAE
DVRSGMVAENRGQVRIEKPPEVRTTGNPFDAEVSDQWEGETEGAIYHGLEEKDRQEYKNKNPDKWADLDG
DGVLEFDPDMPDYPAYKFFTETRWESDTDGGYIGILYVPITRDVDGWSITLKFSMPVTKLENWEYSPTVQ
SPSDGVTCMLHNNHWNGVRMQGTQMRIRIMVEFDRTPGMPPGMIPQPAAALLGTIWYPDGSNANYDEGVS
RNTVSYNEGFSNLPKPETASNVKSYTDPVNERLEQTKSQGDNIKWNNAAMFQDNYRSQLSWLDTPLFGGE
PTEAPKASTTADPYAGLSSNLIKGRTSPNQINFSGNLAGVSGISSSAQKTLDEIAMRLRMLEENTQTDII
MNAFGEVRTTRSTTTTTTVATTKRTLTMCEKRSIAERRMCKQPSCFRHMCLENGDFSPSQCWPKVGLCWC
VTIDGLKLSTTIRKMIKFKSSSCQLTYDPTGIAASRDANTVSAPSNPQQSYGSSQDNTGYESSQISQSYE
EVQVKEAPTPRPTVKYDRYSKHDWSQVIKLSSQFYFAQMSVVLKLPIDHPVRWRLSAHENDGRPCGVDLS
GGFYDSGDFMKYTFPMAWTMTMLSWGAIEHRSTLENVGRWKDLSKIITTGAEYLLKTNPSPRVLYAIVGD
PEIDHQYWGRKPSKDQNSIRPCLKVTQWAHGTDLAAEVAGALAAASMVVSDEALASKCWHKAQELYDFGD
KHRGKYSDVVREVKDYFQTFKDYYPSNDYTDELGWGALWLYKASKVTRNFPARSAYTVFQLGKRVDSFNW
DQKHAGVQLLLAELTQEPKYIADISTFCDYNMPHGGAKYTPGGLLFIEKWGVLRHSLNIAYICLRAGTLM
GMDSIRQSNYRKFAMSQLDYMLGGQGRSFLVGYGKNFPDRPHHRTASCSLVGPCSWEAFKAMLMGALVGG
PDENDIFVNNRTDFVRNEVALDYNAALTSLAALMLDLERGNVG

>E4YKU8_OIKDI
MEGNAKTVVGLVDGGVKEFACFLSKINCARNHKEHNVDSFQYSKSVYFRTIKPIKCGQELLVRYGDVYEK
QLKIEFNNPNPKTTYFQENQDGLFFCPLCNICFHTRDAFNIHKSELNCKILKVIKKTGTPIFGSKDEPYI
CASCGQCYSDLCLLQYHQIEHDELRKFGCDKCCLRFLREEHYQKHMKWCKPDFPS

>E4YKU9_OIKDI
MNAADILLSVGGYTCLWALLFFLAPFVFRKCAFSADIYSKLTKAKQIDLCLRATSIFSATCAWSFGAWNL
HKNLSAYAPLNEPNETASLPLLFMTAFFTLDTIVCIYLREEYGATTYQYLCHHAVAILGFYNSLKYQECL
WFANYRLLSELSTPLINVRAIMNAFNLKEKYAGVYSANRVLTMFAFFFSRIATIPYFWMLTYRRYDDLIN
CDWKIIGMLIVSGIVLDFLNIQWFVLIMKIASVEFKKIPKIAKEKTETLKISLREKKDNVYQTIQTAKTD
FRENVSYRINRVRRRLSAMRPE

>E4YKV2_OIKDI
MSKNLSKSEDKKTTTLCGSHGYIAPEVYDSARYGTKADIWSFGVMIYEMTEKKLLIEAEDSSDLKEKTLE
YELDADTIESDAKGLLIKALKTDPKNRASARKLKRTSFFEEITFENLREQDSPVDFKPPEFGLTDPQPLR
SWFIK

>E4YKX2_OIKDI
MVNRRASHAGSWYSGDPRELENQLKNWISKAKFEKKAKAVIVPHAGYAYSAPTAAWSFLQLDAQTTKKIF
VIGPSHHVYLPNCALPVVKECETPLGNLRIDKDIVTELHATGLFCTMDVPTDEEEHSIEMQLPFLAHIFK
NRLEEVSVVPIMVGSIKQEKEADYAQVFAKYLNDPEVVFVISSDFCHWGKRFRYTYRLEEYEQIFESIDA
VDREGMDHIASKNLSNFHNYLRRTKNTICGRNPICLLLATINLLEQKVNMQSQIKFLKYAQSSQVRSPND
SSVSYASAVCYL

>E4YKX7_OIKDI
MQKLNFYFISCGVLYVSFPYVVLIVSVISLSAYIAAIQSIPSGKITKHVPFIYLNLLSMLFGLGALIYAN
SFSFWFLFLSAFPALFFFSTEKISDLHAMRAEMAYTIVE

>E4YKX8_OIKDI
MAAHPSYNPNFDSNYGSQWGSTAPPPPYGNAPLPGQPPVNIQPMPITLTNPDRLSTHSMVCVCPHCRYEG
ESVITKVTGWGNHLAACGTCVFGLWAGCCLISYYLDILKDTEHQCANCKKLIGIKKILY

>E4YKY6_OIKDI
MSNVEIFDESDNNPFDVDQYIVTNLLQKGLPGEAAPLRNLPLIHRSYSMNSPTIFKILYSFRRAAS

>E4YKY8_OIKDI
MQFYYIIILFANFSFFWRTWHLQLRLLDSKMEKKSQKLEADLRSSGKKHAERIQELQRHQQQAFQSFQDL
EERISAVAARVVHLGEQLEGVNTPREHAADAQRLMGYFSEFLSGNINSDVLTNPHRINETADVVRKLHLI
AQELPDTNFQEVKLSILNQYKKVEKELLNKFRSAGENNDIEQMQLTAETLSRYQNYQLCVDAYVEEALRQ
IDVDESIFEQITSLIDGKSSQISKVFNSPEQVLGKLIQSCYDNRLGEFIHHKLDPLKRKNLELYLSECQK
LYEKTSALSAALSKHRPGSGKDSTFTKKLQRSVFDDFISSYLTDEEMFLKKRSKELLDRYYERQGHEKRG
LGSEMASKVGSIFGSDNRKVEILISEELAASVLQETHSAFRRCGALSPSNLIAENGLKIFNILLEFLIKQ
HVQYAIDFGLTQAPSAEPKTEPDMSMFLLVQEANTVWHLFEKQFNDDLLPLTAQSPIHAEAVQARKSMKD
AMETSMDSALEKTITSAIGYAKNILKSEQKRNDFLSDELASQATPACRTAVEYLHKVISSMKKHLDGENI

DLILIALGGKFYTLIYEHLQAFQFSSMGGMLAICDIKEYRTAAKSLNSVLVDSKFDTLHALCNLLIAVPE
NLRQVCTGDQLANLDKNVLHNFVKLRTDYKANNLQRLFT

>TANC2_HUMAN
MFRNSLKMLLTGGKSSRKNRSSDGGSEEPPDRRQSSVDSRQSRSGQGGISTESDCAFEPDYAVPPLPVSE
GDAEQELGPPPSVDEAANTLMTRLGFLLGEKVTEVQPGDQYSMEVQDENQTSAITQRISPCSTLTSSTAS
PPASSPCSTLPPISTNATAKDCSYGAVTSPTSTLESRDSGIIATLTSYSENVERTKYAGESSKELGSGGN
IKPWQSQKSSMDSCLYRVDENMTASTYSLNKIPERNLETVLSQSVQSIPLYLMPRPNSVAATSSAHLEDL
AYLDEQRHTPLRTSLRMPRQSMGGARTQQDLRVRFAPYRPPDISLKPLLFEVPSITTESVFVGRDWVFHE
IDAQLQSSNASVNQGVVIVGNIGFGKTAIISRLVALSCHGTRMRQIASDSPHASPKHVDANRELPLTQPP
SAHSSITSGSCPGTPEMRRRQEEAMRRLASQVVAYHYCQADNAYTCLVPEFVHNVAALLCRSPQLTAYRE
QLLREPHLQSMLSLRSCVQDPMASFRRGVLEPLENLHKERKIPDEDFIILIDGLNEAEFHKPDYGDTIVS
FLSKMIGKFPSWLKLIVTVRTSLQEITKLLPFHRIFLDRLEENEAIDQDLQAYILHRIHSSSEIQNNISL
NGKMDNTTFGKLSSHLKTLSQGSYLYLKLTFDLIEKGYLVLKSSSYKVVPVSLSEVYLLQCNMKFPTQSS
FDRVMPLLNVAVASLHPLTDEHIFQAINAGSIEGTLEWEDFQQRMENLSMFLIKRRDMTRMFVHPSFREW
LIWREEGEKTKFLCDPRSGHTLLAFWFSRQEGKLNRQQTIELGHHILKAHIFKGLSKKVGVSSSILQGLW
ISYSTEGLSMALASLRNLYTPNIKVSRLLILGGANINYRTEVLNNAPILCVQSHLGYTEMVALLLEFGAN
VDASSESGLTPLGYAAAAGYLSIVVLLCKKRAKVDHLDKNGQCALVHAALRGHLEVVKFLIQCDWTMAGQ
QQGVFKKSHAIQQALIAAASMGYTEIVSYLLDLPEKDEEEVERAQINSFDSLWGETALTAAAGRGKLEVC
RLLLEQGAAVAQPNRRGAVPLFSTVRQGHWQIVDLLLTHGADVNMADKQGRTPLMMAASEGHLGTVDFLL
AQGASIALMDKEGLTALSWACLKGHLSVVRSLVDNGAATDHADKNGRTPLDLAAFYGDAEVVQFLVDHGA
MIEHVDYSGMRPLDRAVGCRNTSVVVTLLKKGAKIGPATWAMATSKPDIMIILLSKLMEEGDMFYKKGKV
KEAAQRYQYALKKFPREGFGEDLKTFRELKVSLLLNLSRCRRKMNDFGMAEEFATKALELKPKSYEAYYA
RARAKRSSRQFAAALEDLNEAIKLCPNNREIQRLLLRVEEECRQMQQPQQPPPPPQPQQQLPEEAEPEPQ
HEDIYSVQDIFEEEYLEQDVENVSIGLQTEARPSQGLPVIQSPPSSPPHRDSAYISSSPLGSHQVFDFRS
SSSVGSPTRQTYQSTSPALSPTHQNSHYRPSPPHTSPAHQGGSYRFSPPPVGGQGKEYPSPPPSPLRRGP
QYRASPPAESMSVYRSQSGSPVRYQQETSVSQLPGRPKSPLSKMAQRPYQMPQLPVAVPQQGLRLQPAKA
QIVRSNQPSPAVHSSTVIPTGAYGQVAHSMASKYQSSQGDIGVSQSRLVYQGSIGGIVGDGRPVQHVQAS
LSAGAICQHGGLTKEDLPQRPSSAYRGGVRYSQTPQIGRSQSASYYPVCHSKLDLERSSSQLGSPDVSHL
IRRPISVNPNEIKPHPPTPRPLLHSQSVGLRFSPSSNSISSTSNLTPTFRPSSSIQQMEIPLKPAYERSC
DELSPVSPTQGGYPSEPTRSRTTPFMGIIDKTARTQQYPHLHQQNRTWAVSSVDTVLSPTSPGNLPQPES
FSPPSSISNIAFYNKTNNAQNGHLLEDDYYSPHGMLANGSRGDLLERVSQASSYPDVKVARTLPVAQAYQ
DNLYRQLSRDSRQGQTSPIKPKRPFVESNV

>K1632_HUMAN
MAEAVKPQRRAKAKASRTKTKEKKKYETPQREESSEVSLPKTSREQEIPSLACEFKGDHLKVVTDSQLQD
DASGQNESEMFDVPLTSLTISNEESLTCNTEPPKEGGEARPCVGDSAVTPKVHPGDNVGTKVETPKNFTE
VEENMSVQGGLSESAPQSNFSYTQPAMENIQVRETQNSKEDKQGLVCSSEVPQNVGLQSSCPAKHGFQTP
RVKKLYPQLPAEIAGEAPALVAVKPLLRSERLYPELPSQLELVPFTKEQLKILEPGSWLENVESYLEEFD
SMAHQDRHEFYELLLNYSRCRKQLLLAEAELLTLTSDCQNAKSRLWQFKEEQMSVQGICADQVKVFSYHR
YQRVEMNENALVELKKLFDAKSEHLHQTLALHSYTSVLSRLQVESYIYALLSSSAVLRSSAIHQQGRASK
QTESIPSDLCQLKECISVLFMFTRRVNEDTQFHDDILLWLQKLVSVLQRVGCPGDHLFLLNHILRCPAGV
SKWAVPFIQIKVLHNPSGVFHFMQSLALLMSPVKNRAEFMCHMKPSERKPSSSGPGSGTWTLVDEGGEED
EDPETSWILLNEDDLVTILAQFPFHELFQHLLGFKAKGDYLPETTRPQEMMKIFAFANSLVELLAVGLET

FNRARYRQFVKRIGYMIRMTLGYVSDHWAQYVSHNQGSGLAQQPYSMEKLQVEFDELFLRAVLHVLKAKR
LGIWLFMSEMPFGTLSVQMLWKLFYLMHQVESENLQQLSSSLQPAQCKQQLQDPEHFTNFEKCLSSMNSS
EEICLLTTFAQMAQARRTNVDEDFIKIIVLEIYEVSYVTLSTRETFSKVGRELLGTITAVHPEIISVLLD
RVQETIDQVGMVSLYLFKELPLYLWQPSASEIAVIRDWLLNYNLTVVKNKLACVILEGLNWGFAKQATLH
LDQAVHAEVALMVLEAYQKYLAQKPYAGILSESMKQVSYLASIVRYGETPETSFNQWAWNLILRLKLHKN
DYGIQPNCPAVPFSVTVPDMTESPTFHPLLKAVKAGMPIGCYLALSMTAVGHSIEKFCAEGIPLLGILVQ
SRHLRTVVHVLDKILPLFYPCQYYLLKNEQFLSHLLLFLHLDSGVPQGVTQQVTHKVAQHLTGASHGDNV
KLLNSMIQAHISVSTQPNEVGPVAVLEFWVQALISQHLWYREQPILFLMDHLCKAAFQLMQEDCIQKLLY
QQHKNALGYHCDRSLLSSLVSWIVAGNITPSFVEGLATPTQVWFAWTVLNMESIFEEDSQLRRVIEGELV
INSAFTPDQALKKAQTQLKLPIVPSLQRLLIYRWAHQALVTPSDHPLLPLIWQKFFLLYLHRPGPQYGLP
IDGCIGRRFFQSPAHINLLKEMKRRLTEVADFHHAASKALRVPAEGSEGLPESHSGTPGYLTSPELHKEL
VRLFNVYILWLEDENFQKGDTYIPSLPKHYDIHRLAKVMQNQQDLWMEYLNMERIYHEFQETVGLWTQAK
LESHSTPCSLSVQLDFTDPLLAKERVLSNLRKHEAPQPPLALHPTKPPVPVISSAVLLSQKDATQLVCTD
LNLLQQQARTAALRESQQVALDGELLDTMPKQYVNREEQTTLHLECRGSSGKKCQGAAVVTVQFEGMHKN
EAISQQLHVLRKEVKQLQAEAAKPPSLNIVEAAVHAENLITALVNAYKLQPTPGIQKVGISLFFTIVDYV
SDETQRHPPTRQFFTSCIEILGQVFISGIKSECRKVLETILKNSRLCSLLSPFFTPNAAPAEFIQLYEQV
VKFLSEDNSDMIFMLLTKFDLKQWLSATKPPLSDRTRLLESIHLALTAWGLEPDEDILMPFNLFCKHWTY
LLLYQFPDQYSDILRLLMQSSAEQLLSPECWKATLRALGCCAPSCQQGAASTEGAVLPSSSDALLSDKQV
METIQWLSDFFYKLRLSKMDFKSFGLFSKWSPYMADVKTFLGYLVKRLIDLEMTCLAQDPTASRKTVLKS
LHSVIIQLFKPWILVLEDNESSQQRHYPWLESDTVVASSIVQLFTDCIDSLHESFKDKLLPGDAGALWLH
LMHYCEACTAPKMPEFILYAFHSTYRKLPWKDLHPDQMLMEAFFKVERGSPKSCFLFLGSVLCEVNWVSV
LSDAWNSSPHPETRSMIVCLLFMMILLAKEVQLVDQTDSPLLSLLGQTSSLSWHLVDIVSYQSVLSYFSS
HYPPSIILAKESYAELIMKLLKVSAGLSIPTDSQKHLDAVPKCQAFTHQMVQFLSTLEQNGKITLAVLEQ
EMSKLLDDIIVFNPPDMDSQTRHMALSSLFMEVLMMMNNATIPTAEFLRGSIRTWIGQKMHGLVVLPLLT
AACQSLASVRHMAETTEACITAYFKESPLNQNSGWGPILVSLQVPELTMEEFLQECLTLGSYLTLYVYLL
QCLNSEQTLRNEMKVLLILSKWLEQVYPSSVEEEAKLFLWWHQVLQLSLIQTEQNDSVLTESVIRILLLV
QSRQNLVAEERLSSGILGAIGFGRKSPLSNRFRVVARSMAAFLSVQVPMEDQIRLRPGSELHLTPKAQQA
LNALESMASSKQYVEYQDQILQATQFIRHPGHCLQDGKSFLALLVNCLYPEVHYLDHIR

>MOV10_HUMAN
MPSKFSCRQLREAGQCFESFLVVRGLDMETDRERLRTIYNRDFKISFGTPAPGFSSMLYGMKIANLAYVT
KTRVRFFRLDRWADVRFPEKRRMKLGSDISKHHKSLLAKIFYDRAEYLHGKHGVDVEVQGPHEARDGQLL
IRLDLNRKEVLTLRLRNGGTQSVTLTHLFPLCRTPQFAFYNEDQELPCPLGPGECYELHVHCKTSFVGYF
PATVLWELLGPGESGSEGAGTFYIARFLAAVAHSPLAAQLKPMTPFKRTRITGNPVVTNRIEEGERPDRA
KGYDLELSMALGTYYPPPRLRQLLPMLLQGTSIFTAPKEIAEIKAQLETALKWRNYEVKLRLLLHLEELQ
MEHDIRHYDLESVPMTWDPVDQNPRLLTLEVPGVTESRPSVLRGDHLFALLSSETHQEDPITYKGFVHKV
ELDRVKLSFSMSLLSRFVDGLTFKVNFTFNRQPLRVQHRALELTGRWLLWPMLFPVAPRDVPLLPSDVKL
KLYDRSLESNPEQLQAMRHIVTGTTRPAPYIIFGPPGTGKTVTLVEAIKQVVKHLPKAHILACAPSNSGA
DLLCQRLRVHLPSSIYRLLAPSRDIRMVPEDIKPCCNWDAKKGEYVFPAKKKLQEYRVLITTLITAGRLV
SAQFPIDHFTHIFIDEAGHCMEPESLVAIAGLMEVKETGDPGGQLVLAGDPRQLGPVLRSPLTQKHGLGY
SLLERLLTYNSLYKKGPDGYDPQFITKLLRNYRSHPTILDIPNQLYYEGELQACADVVDRERFCRWAGLP
RQGFPIIFHGVMGKDEREGNSPSFFNPEEAATVTSYLKLLLAPSSKKGKARLSPRSVGVISPYRKQVEKI
RYCITKLDRELRGLDDIKDLKVGSVEEFQGQERSVILISTVRSSQSFVQLDLDFNLGFLKNPKRFNVAVT
RAKALLIIVGNPLLLGHDPDWKVFLEFCKENGGYTGCPFPAKLDLQQGQNLLQGLSKLSPSTSGPHSHDY

LPQEREGEGGLSLQVEPEWRNEL

>ZN532_HUMAN
MTMGDMKTPDFDDLLAAFDIPDMVDPKAAIESGHDDHESHMKQNAHGEDDSHAPSSSDVGVSVIVKNVRN
IDSSEGGEKDGHNPTGNGLHNGFLTASSLDSYSKDGAKSLKGDVPASEVTLKDSTFSQFSPISSAEEFDD
DEKIEVDDPPDKEDMRSSFRSNVLTGSAPQQDYDKLKALGGENSSKTGLSTSGNVEKNKAVKRETEASSI
NLSVYEPFKVRKAEDKLKESSDKVLENRVLDGKLSSEKNDTSLPSVAPSKTKSSSKLSSCIAAIAALSAK
KAASDSCKEPVANSRESSPLPKEVNDSPRAADKSPESQNLIDGTKKPSLKQPDSPRSISSENSSKGSPSS
PAGSTPAIPKVRIKTIKTSSGEIKRTVTRVLPEVDLDSGKKPSEQTASVMASVTSLLSSPASAAVLSSPP
RAPLQSAVVTNAVSPAELTPKQVTIKPVATAFLPVSAVKTAGSQVINLKLANNTTVKATVISAASVQSAS
SAIIKAANAIQQQTVVVPASSLANAKLVPKTVHLANLNLLPQGAQATSELRQVLTKPQQQIKQAIINAAA
SQPPKKVSRVQVVSSLQSSVVEAFNKVLSSVNPVPVYIPNLSPPANAGITLPTRGYKCLECGDSFALEKS
LTQHYDRRSVRIEVTCNHCTKNLVFYNKCSLLSHARGHKEKGVVMQCSHLILKPVPADQMIVSPSSNTST
STSTLQSPVGAGTHTVTKIQSGITGTVISAPSSTPITPAMPLDEDPSKLCRHSLKCLECNEVFQDETSLA
THFQQAADTSGQKTCTICQMLLPNQCSYASHQRIHQHKSPYTCPECGAICRSVHFQTHVTKNCLHYTRRV
GFRCVHCNVVYSDVAALKSHIQGSHCEVFYKCPICPMAFKSAPSTHSHAYTQHPGIKIGEPKIIYKCSMC
DTVFTLQTLLYRHFDQHIENQKVSVFKCPDCSLLYAQKQLMMDHIKSMHGTLKSIEGPPNLGINLPLSIK
PATQNSANQNKEDTKSMNGKEKLEKKSPSPVKKSMETKKVASPGWTCWECDCLFMQRDVYISHVRKEHGK
QMKKHPCRQCDKSFSSSHSLCRHNRIKHKGIRKVYACSHCPDSRRTFTKRLMLEKHVQLMHGIKDPDLKE
MTDATNEEETEIKEDTKVPSPKRKLEEPVLEFRPPRGAITQPLKKLKINVFKVHKCAVCGFTTENLLQFH
EHIPQHKSDGSSYQCRECGLCYTSHVSLSRHLFIVHKLKEPQPVSKQNGAGEDNQQENKPSHEDESPDGA
VSDRKCKVCAKTFETEAALNTHMRTHGMAFIKSKRMSSAEK

>MTL14_HUMAN
MDSRLQEIRERQKLRRQLLAQQLGAESADSIGAVLNSKDEQREIAETRETCRASYDTSAPNAKRKYLDEG
ETDEDKMEEYKDELEMQQDEENLPYEEEIYKDSSTFLKGTQSLNPHNDYCQHFVDTGHRPQNFIRDVGLA
DRFEEYPKLRELIRLKDELIAKSNTPPMYLQADIEAFDIRELTPKFDVILLEPPLEEYYRETGITANEKC
WTWDDIMKLEIDEIAAPRSFIFLWCGSGEGLDLGRVCLRKWGYRRCEDICWIKTNKNNPGKTKTLDPKAV
FQRTKEHCLMGIKGTVKRSTDGDFIHANVDIDLIITEEPEIGNIEKPVEIFHIIEHFCLGRRRLHLFGRD
STIRPGWLTVGPTLTNSNYNAETYASYFSAPNSYLTGCTEEIERLRPKSPPPKSKSDRGGGAPRGGGRGG
TSAGRGRERNRSNFRGERGGFRGGRGGAHRGGFPPR

>ARGAL_HUMAN
MASSNPPPQPAIGDQLVPGVPGPSSEAEDDPGEAFEFDDSDDEEDTSAALGVPSLAPERDTDPPLIHLDS
IPVTDPDPAAAPPGTGVPAWVSNGDAADAAFSGARHSSWKRKSSRRIDRFTFPALEEDVIYDDVPCESPD
AHQPGAERNLLYEDAHRAGAPRQAEDLGWSSSEFESYSEDSGEEAKPEVEVEPAKHRVSFQPKLSPDLTR
LKERYARTKRDILALRVGGRDMQELKHKYDCKMTQLMKAAKSGTKDGLEKTRMAVMRKVSFLHRKDVLGD
SEEEDMGLLEVSVSDIKPPAPELGPMPEGLSPQQVVRRHILGSIVQSEGSYVESLKRILQDYRNPLMEME
PKALSARKCQVVFFRVKEILHCHSMFQIALSSRVAEWDSTEKIGDLFVASFSKSMVLDVYSDYVNNFTSA
MSIIKKACLTKPAFLEFLKRRQVCSPDRVTLYGLMVKPIQRFPQFILLLQDMLKNTPRGHPDRLSLQLAL
TELETLAEKLNEQKRLADQVAEIQQLTKSVSDRSSLNKLLTSGQRQLLLCETLTETVYGDRGQLIKSKER
RVFLLNDMLVCANINFKPANHRGQLEISSLVPLGPKYVVKWNTALPQVQVVEVGQDGGTYDKDNVLIQHS
GAKKASASGGQAQNKVYLGPPRLFQELQDLQKDLAVVEQITLLISTLHGTYQNLNMTVAQDWCLALQRLMR
VKEEEIHSANKCRLRLLLPGKPDKSGRPISFMVVFITPNPLSKISWVNRLHLAKIGLREENQPGWLCPDE

DKKSKAPFWCPILACCIPAFSSRALSLQLGALVHSPVNCPLLGFSAVSTSLPQGYLWVGGGQEGAGGQVE
IFSLNRPSPRTVKSFPLAAPVLCMEYIPELEEEAESRDESPTVADPSATVHPTICLGLQDGSILLYSSVD
TGTQCLVSCRSPGLQPVLCLRHSPFHLLAGLQDGTLAAYPRTSGGVLWDLESPPVCLTVGPGPVRTLLSL
EDAVWASCGPWVTVLEATTLQPQQSFEAHQDEAVSVTHMVKAGSGVWMAFSSGTSIRLFHTETLEHLQEI
NIATRTTFLLPGQKHLCVTSLLICQGLLWVGTDQGVIVLLPVPRLEGIPKITGKGMVSLNGHCGPVAFLA
VATSILAPDILRSDQEEAEGPRAEEDKPDGQAHEPMPDSHVGRELTRKKGILLQYRLRSTAHLPGPLLSM
REPAPADGAALEHSEEDGSIYEMADDPDIWVRSRPCARDAHRKEICSVAIISGGQGYRNFGSALGSSGRQ
APCGETDSTLLIWQVPLML

>SMUF1_HUMAN
MSNPGTRRNGSSIKIRLTVLCAKNLAKKDFFRLPDPFAKIVVDGSGQCHSTDTVKNTLDPKWNQHYDLYV
GKTDSITISVWNHKKIHKKQGAGFLGCVRLLSNAISRLKDTGYQRLDLCKLNPSDTDAVRGQIVVSLQTR
DRIGTGGSVVDCRGLLENEGTVYEDSGPGRPLSCFMEEPAPYTDSTGAAAGGGNCRFVESPSQDQRLQAQ
RLRNPDVRGSLQTPQNRPHGHQSPELPEGYEQRTTVQGQVYFLHTQTGVSTWHDPRIPSPSGTIPGGDAA
FLYEFLLQGHTSEPRDLNSVNCDELGPLPPGWEVRSTVSGRIYFVDHNNRTTQFTDPRLHHIMNHQCQLK
EPSQPLPLPSEGSLEDEELPAQRYERDLVQKLKVLRHELSLQQPQAGHCRIEVSREEIFEESYRQIMKMR
PKDLKKRLMVKFRGEEGLDYGGVAREWLYLLCHEMLNPYYGLFQYSTDNIYMLQINPDSSINPDHLSYFH
FVGRIMGLAVFHGHYINGGFTVPFYKQLLGKPIQLSDLESVDPELHKSLVWILENDITPVLDHTFCVEHN
AFGRILQHELKPNGRNVPVTEENKKEYVRLYVNWRFMRGIEAQFLALQKGFNELIPQHLLKPFDQKELEL
IIGGLDKIDLNDWKSNTRLKHCVADSNIVRWFWQAVETFDEERRARLLQFVTGSTRVPLQGFKALQGSTG
AAGPRLFTIHLIDANTDNLPKAHTCFNRIDIPPYESYEKLYEKLLTAVEETCGFAVE

>ANO8_HUMAN
MAEAASGAGGTSLEGERGKRPPPEGEPAAPASGVLDKLFGKRLLQAGRYLVSHKAWMKTVPTENCDVLMT
FPDTTDDHTLLWLLNHIRVGIPELIVQVRHHRHTRAYAFFVTATYESLLRGADELGLRKAVKAEFGGGTR
GFSCEEDFIYENVESELRFFTSQERQSIIRFWLQNLRAKQGEALHNVRFLEDQPIIPELAARGIIQQVFP
VHEQRILNRLMKSWVQAVCENQPLDDICDYFGVKIAMYFAWLGFYTSAMVYPAVFGSVLYTFTEADQTSR
DVSCVVFALFNVIWSTLFLEEWKRRGAELAYKWGTLDSPGEAVEEPRPQFRGVRRISPITRAEEFYYPPW
KRLLFQLLVSLPLCLACLVCVFLLMLGCFQLQELVLSVKGLPRLARFLPKVMLALLVSVSAEGYKKLAIW
LNDMENYRLESAYEKHLIIKVVLFQFVNSYLSLFYIGFYLKDMERLKEMLATLLITRQFLQNVREVLQPH
LYRRLGRGELGLRAVWELARALLGLLSLRRPAPRRLEPQADEGGGGGSGGGGRRCLSGGCGAPEEEEEAA
LVERRRAGEGGEEGDGPPGGKEEDEDDEEEEDEEEEEDEEEGEEGGLLDCGLRLKKVSFAERGAGRRRPG
PSPEALLEEGSPTMVEKGLEPGVFTLAEEDDEAEGAPGSPEREPPAILFRRAGGEGRDQGPDGGPDPEPG
SNSDSTRRQRRQNRSSWIDPPEEEHSPQLTQAELESCMKKYEDTFQDYQEMFVQFGYVVLFSSAFPLAAL
CALVNNLIEIRSDAFKLCTGLQRPFGQRVESIGQWQKVMEAMGVLAIVVNCYLIGQCGQLQRLFPWLSPE
AAIVSVVVLEHFALLLKYLIHVAIPDIPGWVAEEMAKLEYQRREAFKRHERQAQHRYQQQQRRRREEEER
QRHAEHHARREHDSGGREEARAEGSGLDPATSSEKASAKAKGSTAGGHGPERPKRPGSLLAPNNVMKLKQ
IIPLQGKFLSSGATSSLAAAGAGATTRPPPAQSPTGSDTRLPAFLSFKFLKSPETRRDSERSHSPPKAFH
AGKLFPFGGTRAEPGSNGAGGQARPDGTPSSGSSRVQRSGPVDEALAEELEAPRPEEEGSGTALAPVGAP
ALRTRRSRSPAPPPPMPLPRPPTPPAGCWQWDGPWGCGGEGAAPRQALAAAECPPCAMAGPPPAPQPLPG
DASFYSLPPPPLPPTSDPLETPAPSPSPSPSPQAVCWPSGWH

>TRPM3_HUMAN
MPEPWGTVYFLGIAQVFSFLFSWWNLEGVMNQADAPRPLNWTIRKLCHAAFLPSVRLLKAQKSWIERAFY

KRECVHIIPSTKDPHRCCCGRLIGQHVGLTPSISVLQNEKNESRLSRNDIQSEKWSISKHTQLSPTDAFG
TIEFQGGGHSNKAMYVRVSFDTKPDLLLHLMTKEWQLELPKLLISVHGGLQNFELQPKLKQVFGKGLIKA
AMTTGAWIFTGGVNTGVIRHVGDALKDHASKSRGKICTIGIAPWGIVENQEDLIGRDVVRPYQTMSNPMS
KLTVLNSMHSHFILADNGTTGKYGAEVKLRRQLEKHISLQKINTRCLPFFSLDSRLFYSFWGSCQLDSVG
IGQGVPVVALIVEGGPNVISIVLEYLRDTPPVPVVVCDGSGRASDILAFGHKYSEEGGLINESLRDQLLV
TIQKTFTYTRTQAQHLFIILMECMKKKELITVFRMGSEGHQDIDLAILTALLKGANASAPDQLSLALAWN
RVDIARSQIFIYGQQWPVGSLEQAMLDALVLDRVDFVKLLIENGVSMHRFLTISRLEELYNTRHGPSNTL
YHLVRDVKKGNLPPDYRISLIDIGLVIEYLMGGAYRCNYTRKRFRTLYHNLFGPKRPKALKLLGMEDDIP
LRRGRKTTKKREEEVDIDLDDPEINHFPFPFHELMVWAVLMKRQKMALFFWQHGEEAMAKALVACKLCKA
MAHEASENDMVDDISQELNHNSRDFGQLAVELLDQSYKQDEQLAMKLLTYELKNWSNATCLQLAVAAKHR
DFIAHTCSQMLLTDMWMGRLRMRKNSGLKVILGILLPPSILSLEFKNKDDMPYMSQAQEIHLQEKEAEEP
EKPTKEKEEEDMELTAMLGRNNGESSRKKDEEEVQSKHRLIPLGRKIYEFYNAPIVKFWFYTLAYIGYLM
LFNYIVLVKMERWPSTQEWIVISYIFTLGIEKMREILMSEPGKLLQKVKVWLQEYWNVTDLIAILLFSVG
MILRLQDQPFRSDGRVIYCVNIIYWYIRLLDIFGVNKYLGPYVMMIGKMMIDMMYFVIIMLVVLMSFGVA
RQAILFPNEEPSWKLAKNIFYMPYWMIYGEVFADQIDPPCGQNETREDGKIIQLPPCKTGAWIVPAIMAC
YLLVANILLVNLLIAVFNNTFFEVKSISNQVWKFQRYQLIMTFHERPVLPPPLIIFSHMTMIFQHLCCRW
RKHESDPDERDYGLKLFITDDELKKVHDFEEQCIEEYFREKDDRFNSSNDERIRVTSERVENMSMRLEEV
NEREHSMKASLQTVDIRLAQLEDLIGRMATALERLTGLERAESNKIRSRTSSDCTDAAYIVRQSSFNSQE
GNTFKLQESIDPAGEETMSPTSPTLMPRMRSHSFYSVNMKDKGGIEKLESIFKERSLSLHRATSSHSVAK
EPKAPAAPANTLAIVPDSRRPSSCIDIYVSAMDELHCDIDPLDNSVNILGLGEPSFSTPVPSTAPSSSAY
ATLAPTDRPPSRSIDFEDITSMDTRSFSSDYTHLPECQNPWDSEPPMYHTIERSKSSRYLATTPFLLEEA
PIVKSHSFMFSPSRSYYANFGVPVKTAEYTSITDCIDTRCVNAPQAIADRAAFPGGLGDKVEDLTCCHPE
REAELSHPSSDSEENEAKGRRATIAISSQEGDNSERTLSNNITVPKIERANSYSAEEPSAPYAHTRKSFS
ISDKLDRQRNTASLRNPFQRSKSSKPEGRGDSLSMRRLSRTSAFQSFESKHN

>GBA2_HUMAN
MGTQDPGNMGTGVPASEQISCAKEDPQVYCPEETGGTKDVQVTDCKSPEDSRPPKETDCCNPEDSGQLMV
SYEGKAMGYQVPPFGWRICLAHEFTEKRKPFQANNVSLSNMIKHIGMGLRYLQWWYRKTHVEKKTPFIDM
INSVPLRQIYGCPLGGIGGGTITRGWRGQFCRWQLNPGMYQHRTVIADQFTVCLRREGQTVYQQVLSLER
PSVLRSWNWGLCGYFAFYHALYPRAWTVYQLPGQNVTLTCRQITPILPHDYQDSSLPVGVFVWDVENEGD
EALDVSIMFSMRNGLGGGDDAPGGLWNEPFCLERSGETVRGLLLHHPTLPNPYTMAVAARVTAATTVTHI
TAFDPDSTGQQVWQDLLQDGQLDSPTGQSTPTQKGVGIAGAVCVSSKLRPRGQCRLEFSLAWDMPRIMFG
AKGQVHYRRYTRFFGQDGDAAPALSHYALCRYAEWEERISAWQSPVLDDRSLPAWYKSALFNELYFLADG
GTVWLEVLEDSLPEELGRNMCHLRPTLRDYGRFGYLEGQEYRMYNTYDVHFYASFALIMLWPKLELSLQY
DMALATLREDLTRRRYLMSGVMAPVKRRNVIPHDIGDPDDEPWLRVNAYLIHDTADWKDLNLKFVLQVYR
DYYLTGDQNFLKDMWPVCLAVMESEMKFDKDHDGLIENGGYADQTYDGWVTTGPSAYCGGLWLAAVAVMV
QMAALCGAQDIQDKFSSILSRGQEAYERLLWNGRYYNYDSSSRPQSRSVMSDQCAGQWFLKACGLGEGDT
EVFPTQHVVRALQTIFELNVQAFAGGAMGAVNGMQPHGVPDKSSVQSDEVWVGVVYGLAATMIQEGLTWE
GFQTAEGCYRTVWERLGLAFQTPEAYCQQRVFRSLAYMRPLSIWAMQLALQQQQHKKASWPKVKQGTGLR
TGPMFGPKEAMANLSPE

>CWC22_HUMAN
MKSSVAQIKPSSGHDRRENLNSYQRNSSPEDRYEEQERSPRDRDYFDYSRSDYEHSRRGRSYDSSMESRN
RDREKRRERERDTDRKRSRKSPSPGRRNPETSVTQSSSAQDEPATKKKKDELDPLLTRTGGAYIPPAKLR

MMQEQITDKNSLAYQRMSWEALKKSINGLINKVNISNISIIIQELLQENIVRGRGLLSRSVLQAQSASPI
FTHVYAALVAIINSKFPQIGELILKRLILNFRKGYRRNDKQLCLTASKFVAHLINQNVAHEVLCLEMLTL
LLERPTDDSVEVAIGFLKECGLKLTQVSPRGINAIFERLRNILHESEIDKRVQYMIEVMFAVRKDGFKDH
PIILEGLDLVEEDDQFTHMLPLEDDYNPEDVLNVFKMDPNFMENEEKYKAIKKEILDEGDTDSNTDQDAG
SSEEDEEEEEEEGEEDEEGQKVTIHDKTEINLVSFRRTIYLAIQSSLDFEECAHKLLKMEFPESQTKELC
NMILDCCAQQRTYEKFFGLLAGRFCMLKKEYMESFEGIFKEQYDTIHRLETNKLRNVAKMFAHLLYTDSL
PWSVLECIKLSEETTTSSSRIFVKIFFQELCEYMGLPKLNARLKDETLQPFFEGLLPRDNPRNTRFAINF
FTSIGLGGLTDELREHLKNTPKVIVAQKPDVEQNKSSPSSSSSASSSSESDSSDSDSDSSDSSSESSSEE
SDSSSISSHSSASANDVRKKGHGKTRSKEVDKLIRNQQTNDRKQKERRQEHGHQETRTERERRSEKHRDQ
NSSGSNWRDPITKYTSDKDVPSERNNYSRVANDRDQEMHIDLENKHGDPKKKRGERRNSFSENEKHTHRI
KDSENFRRKDRSKSKEMNRKHSGSRSDEDRYQNGAERRWEKSSRYSEQSRESKKNQDRRREKSPAKQK

>NCK5L_HUMAN
MDQPAGGPGNPRPGEGDDGSMEPGTCQELLHRLRELEAENSALAQANENQRETYERCLDEVANHVVQALL
NQKDLREECIKLKKRVFDLERQNQMLSALFQQKLQLTTGSLPQIPLTPLQPPSEPPASPSLSSTEGPAAP
LPLGHCAGQREVCWEQQLRPGGPGPPAAPPPALDALSPFLRKKAQILEVLRALEETDPLLLCSPATPWRP
PGQGPGSPEPINGELCGPPQPEPSPWAPCLLLGPGNLGGLLHWERLLGGLGGEEDTGRPWGPSRGPPQAQ
GTSSGPNCAPGSSSSSSSDEAGDPNEAPSPDTLLGALARRQLNLGQLLEDTESYLQAFLAGAAGPLNGDH
PGPGQSSSPDQAPPQLSKSKGLPKSAWGGGTPEAHRPGFGATSEGQGPLPFLSMFMGAGDAPLGSRPGHP
HSSSQVKSKLQIGPPSPGEAQGPLLPSPARGLKFLKLPPTSEKSPSPGGPQLSPQLPRNSRIPCRNSGSD
GSPSPLLARRGLGGGELSPEGAQGLPTSPSPCYTTPDSTQLRPPQSALSTTLSPGPVVSPCYENILDLSR
STFRGPSPEPPPSPLQVPTYPQLTLEVPQAPEVLRSPGVPPSPCLPESYPYGSPQEKSLDKAGSESPHPG
RRTPGNSSKKPSQGSGRRPGDPGSTPLRDRLAALGKLKTGPEGALGSEKNGVPARPGTEKTRGPGKSGES
AGDMVPSIHRPLEQLEAKGGIRGAVALGTNSLKQQEPGLMGDPGARVYSSHSMGARVDLEPVSPRSCLTK
VELAKSRLAGALCPQVPRTPAKVPTSAPSLGKPNKSPHSSPTKLPSKSPTKVVPRPGAPLVTKESPKPDK
GKGPPWADCGSTTAQSTPLVPGPTDPSQGPEGLAPHSAIEEKVMKGIEENVLRLQGQERAPGAEVKHRNT
SSIASWFGLKKSKLPALNRRTEATKNKEGAGGGSPLRREVKMEARKLEAESLNISKLMAKAEDLRRALEE
EKAYLSSRARPRPGGPAPGPNTGLGQVQGQLAGMYQGADTFMQQLLNRVDGKELPSKSWREPKPEYGDFQ
PVSSDPKSPWPACGPRNGLVGPLQGCGKPPGKPSSEPGRREETPSEDSLAEPVPTSHFTACGSLTRTLDS
GIGTFPPPDHGSSGTPSKNLPKTKPPRLDPPPGVPPARPPPLTKVPRRAHTLEREVPGIEELLVSGRHPS
MPAFPALLPAAPGHRGHETCPDDPCEDPGPTPPVQLAKNWTFPNTRAAGSSSDPLMCPPRQLEGLPRTPM
ALPVDRKRSQEPSRPSPTPQGPPFGGSRTPSTSDMAEEGRVASGGPPGLETSESLSDSLYDSLSSCGSQG

>CPNE5_HUMAN
MEQPEDMASLSEFDSLAGSIPATKVEITVSCRNLLDKDMFSKSDPLCVMYTQGMENKQWREFGRTEVIDN
TLNPDFVRKFIVDYFFEEKQNLRFDLYDVDSKSPDLSKHDFLGQAFCTLGEIVGSPGSRLEKPLTIGAFS
LNSRTGKPMPAVSNGGVPGKKCGTIILSAEELSNCRDVATMQFCANKLDKKDFFGKSDPFLVFYRSNEDG
TFTICHKTEVMKNTLNPVWQTFSIPVRALCNGDYDRTIKVEVYDWDRDGSHDFIGEFTTSYRELARGQSQ
FNIYEVVNPKKKMKKKKYVNSGTVTLLSFAVESECTFLDYIKGGTQINFTVAIDFTASNGNPSQSTSLHY
MSPYQLNAYALALTAVGEIIQHYDSDKMFPALGFGAKLPPDGRVSHEFPLNGNQENPSCCGIDGILEAYH
RSLRTVQLYGPTNFAPVVTHVARNAAAVQDGSQYSVLLIITDGVISDMAQTKEAIVNAAKLPMSIIIVGV
GQAEFDAMVELDGDDVRISSRGKLAERDIVQFVPFRDYVDRTGNHVLSMARLARDVLAEIPDQLVSYMKA
QGIRPRPPPAAPTHSPSQSPARTPPASPLHTHI

>SYTL2_HUMAN
MIDLSFLTEEEQEAIMKVLQRDAALKRAEEERVRHLPEKIKDDQQLKNMSGQWFYEAKAKRHRDKIHGAD
IIRASMRKKRPQIAAEQSKDRENGAKESWVNNVNKDAFLPPELAGVVEEPEEDAAPASPSSSVVNPASSV
IDMSQENTRKPNVSPEKRKNPFNSSKLPEGHSSQQTKNEQSKNGRTGLFQTSKEDELSESKEKSTVADTS
IQKLEKSKQTLPGLSNGSQIKAPIPKARKMIYKSTDLNKDDNQSFPRQRTDSLKARGAPRGILKRNSSSS
STDSETLRYNHNFEPKSKIVSPGLTIHERISEKEHSLEDNSSPNSLEPLKHVRFSAVKDELPQSPGLIHG
REVGEFSVLESDRLKNGMEDAGDTEEFQSDPKPSQYRKPSLFHQSTSSPYVSKSETHQPMTSGSFPINGL
HSHSEVLTARPQSMENSPTINEPKDKSSELTRLESVLPRSPADELSHCVEPEPSQVPGGSSRDRQQGSEE
EPSPVLKTLERSAARKMPSKSLEDISSDSSNQAKVDNQPEELVRSAEDVSTVPTQPDNPFSHPDKLKRMS
KSVPAFLQDESDDRETDTASESSYQLSRHKKSPSSLTNLSSSSGMTSLSSVSGSVMSVYSGDFGNLEVKG
NIQFAIEYVESLKELHVFVAQCKDLAAADVKKQRSDPYVKAYLLPDKGKMGKKKTLVVKKTLNPVYNEIL
RYKIEKQILKTQKLNLSIWHRDTFKRNSFLGEVELDLETWDWDNKQNKQLRWYPLKRKTAPVALEAENRG
EMKLALQYVPEPVPGKKLPTTGEVHIWVKECLDLPLLRGSHLNSFVKCTILPDTSRKSRQKTRAVGKTTN
PIFNHTMVYDGFRPEDLMEACVELTVWDHYKLTNQFLGGLRIGFGTGKSYGTEVDWMDSTSEEVALWEKM
VNSPNTWIEATLPLRMLLIAKISK

>MAGE1_HUMAN
MSLVSQNSRRRRRRVAKATAHNSSWGEMQAPNAPGLPADVPGSDVPQGPSDSQILQGLCASEGPSTSVLP
TSAEGPSTFVPPTISEASSASGQPTISEGPGTSVLPTPSEGLSTSGPPTISKGLCTSVTLAASEGRNTSR
PPTSSEEPSTSVPPTASEVPSTSLPPTPGEGTSTSVPPTAYEGPSTSVVPTPDEGPSTSVLPTPGEGPGT
SVPLAATEGLSTSVQATPDEGPSTSVPPTATEGLSTPVPPTRDEGPSTSVPATPGEGPSTSVLPAASDGQ
SISLVPTRGKGSSTSVPPTATEGLSTSVQPTAGEGSSTSVPPTPGGGLSTSVPPTATEELSTSVPPTPGE
GPSTSVLPIPGEGLSTSVPPTASDGSDTSVPPTPGEGASTLVQPTAPDGPGSSVLPNPGEGPSTLFSSSA
SVDRNPSKCSLVLPSPRVTKASVDSDSEGPKGAEGPIEFEVLRDCESPNSISIMGLNTSRVAITLKPQDP
MEQNVAELLQFLLVKDQSKYPIRESEMREYIVKEYRNQFPEILRRAAAHLECIFRFELRELDPEAHTYIL
LNKLGPVPFEGLEESPNGPKMGLLMMILGQIFLNGNQAKEAEIWEMLWRMGVQRERRLSIFGNPKRLLSV
EFVWQRYLDYRPVTDCKPVEYEFFWGPRSHLETTKMKILKFMAKIYNKDPMDWPEKYNEALEEDAARAFA
EGWQALPHFRRPFFEEAAAEVPSPDSEVSSYSSKYAPHSWPESRLESKARKLVQLFLLMDSTKLPIPKKG
ILYYIGRECSKVFPDLLNRAARTLNHVYGTELVVLDPRNHSYTLYNRREMEETEEIVDSPNRPGNNFLMQ
VLSFIFIMGNHARESAVWAFLRGLGVQAGRKHVITCRYLSQRYIDSLRVPDSDPVQYEFVWGPRARLETS
KMKALRYVARIHRKEPQDWPQQYREAMEDEANRADVGHRQIFVHNFR

>K1586_HUMAN
MGDPGSEIIESVPPAGPEASESTTDENEDDIQFVSEGPSRPVLEYIDLVCGDDENPSAYYSDILFPKMPK
RQGDFLHFLNVKKVKTDTENNEVSKNHCRLSKAKEPHFEYIEQPIIEEKPSLSSKKEIDNLVLPDCWNEK
QAFMFTEQYKWLEIKEGKLGCKDCSAVRHLGSKAEKHVHVSKEWIAYLVTPNGSNKTTRQASLRKKIREH
DVSKAHGKIQDLLKESTNDSICNLVHKQNNKNIDATVKVFNTVYSLVKHNRPLSDIEGARELQEKNGEVN
CLNTRYSATRIAEHIAKEMKMKIFKNIIEENAKICIIIDEASTVSKKTTLVIYLQCTIQSAPAPVMLFVA
LKELVSTIAECIVNTLLTTLNDCGFTNEYLKANLIAFCSDGANTILGRKSGVATKLLENFPEIIIWNCLN
HRLQLSLDDSISEIKQINHLKIFIDKIYSIYHQPNKNQTKLLGTVAKELETEIIKIGRVMGPRWAACSLQ
AATAVWHAYPILYMHFSHSYSGLAKRLANINFLQDLALMIDILEEFSVLSTALQSRSTNIKKAQKLIKRT
IRALENLKIGTGKYESQIEDLIKSDKFKDIPFNKNNKFNALPRSILLDNIIQHMNLRLLSDRNHEDIFNY
FDLLEPSTWPYEEITSPWIAGEKTLFHLCKILKYEVDLNDFREFVNNNIKSNNVSIPTTIYKAKKIVSTI
AINSAEAERGFNLMNIICTRVRNSLTIDHVSDLMTINLLGKELADWDATPFVKSWSNCNHRLATDTRVRQ

KSTKVFHENQLAIWNLK

>TNR6C_HUMAN
MATGSAQGNFTGHTKKTNGNNGTNGALVQSPSNQSALGAGGANSNGSAARVWGVATGSSSGLAHCSVSGG
DGKMDTMIGDGRSQNCWGASNSNAGINLNLNPNANPAAWPVLGHEGTVATGNPSSICSPVSAIGQNMGNQ
NGNPTGTLGAWGNLLPQESTEPQTSTSQNVSFSAQPQNLNTDGPNNTNPMNSSPNPINAMQTNGLPNWGM
AVGMGAIIPPHLQGLPGANGSSVSQVSGGSAEGISNSVWGLSPGNPATGNSNSGFSQGNGDTVNSALSAK
QNGSSSAVQKEGSGGNAWDSGPPAGPGILAWGRGSGNNGVGNIHSGAWGHPSRSTSNGVNGEWGKPPNQH
SNSDINGKGSTGWESPSVTSQNPTVQPGGEHMNSWAKAASSGTTASEGSSDGSGNHNEGSTGREGTGEGR
RRDKGIIDQGHIQLPRNDLDPRVLSNTGWGQTPVKQNTAWEFEESPRSERKNDNGTEAWGCAATQASNSG
GKNDGSIMNSTNTSSVSGWVNAPPAAVPANTGWGDSNNKAPSGPGVWGDSISSTAVSTAAAAKSGHAWSG
AANQEDKSPTWGEPPKPKSQHWGDGQRSNPAWSAGGGDWADSSSVLGHLGDGKKNGSGWDADSNRSGSGW
NDTTRSGNSGWGNSTNTKANPGTNWGETLKPGPQQNWASKPQDNNVSNWGGAASVKQTGTGWIGGPVPVK
QKDSSEATGWEEPSPPSIRRKMEIDDGTSAWGDPSNYNNKTVNMWDRNNPVIQSSTTTNTTTTTTTTTSN
TTHRVETPPPHQAGTQLNRSPLLGPGRKVSSGWGEMPNVHSKTENSWGEPSSPSTLVDNGTAAWGKPPSS
GSGWGDHPAEPPVAFGRAGAPVAASALCKPASKSMQEGWGSGGDEMNLSTSQWEDEEGDVWNNAASQEST
SSCSSWGNAPKKGLQKGMKTSGKQDEAWIMSRLIKQLTDMGFPREPAEEALKSNNMNLDQAMSALLEKKV
DVDKRGLGVTDHNGMAAKPLGCRPPISKESSVDRPTFLDKDGGLVEEPTPSPFLPSPSLKLPLSHSALPS
QALGGIASGLGMQNLNSSRQIPSGNLGMFGNSGAAQARTMQQPPQPPVQPLNSSQPSLRAQVPQFLSPQV
QAQLLQFAAKNIGLNPALLTSPINPQHMTMLNQLYQLQLAYQRLQIQQQMLQAQRNVSGSMRQQEQQVAR
TITNLQQQIQQHQRQLAQALLVKQPPPPPPPPHLSLHPSAGKSAMDSFPSHPQTPGLPDLQTKEQQSSPN
TFAPYPLAGLNPNMNVNSMDMTGGLSVKDPSQSQSRLPQWTHPNSMDNLPSAASPLEQNPSKHGAIPGGL
SIGPPGKSSIDDSYGRYDLIQNSESPASPPVAVPHSWSRAKSDSDKISNGSSINWPPEFHPGVPWKGLQN
IDPENDPDVTPGSVPTGPTINTTIQDVNRYLLKSGGKLSDIKSTWSSGPTSHTQASLSHELWKVPRNSTA
PTRPPPGLTNPKPSSTWGASPLGWTSSYSSGSAWSTDTSGRTSSWLVLRNLTPQIDGSTLRTLCLQHGPL
ITFHLNLTQGNAVVRYSSKEEAAKAQKSLHMCVLGNTTILAEFAGEEEVNRFLAQGQALPPTSSWQSSSA
SSQPRLSAAGSSHGLVRSDAGHWNAPCLGGKGSSELLWGGVPQYSSSLWGPPSADDSRVIGSPTPLTTLL
PGDLLSGESL

>ANKH_HUMAN
MVKFPALTHYWPLIRFLVPLGITNIAIDFGEQALNRGIAAVKEDAVEMLASYGLAYSLMKFFTGPMSDFK
NVGLVFVNSKRDRTKAVLCMVVAGAIAAVFHTLIAYSDLGYYIINKLHHVDESVGSKTRRAFLYLAAFPF
MDAMAWTHAGILLKHKYSFLVGCASISDVIAQVVFVAILLHSHLECREPLLIPILSLYMGALVRCTTLCL
GYYKNIHDIIPDRSGPELGGDATIRKMLSFWWPLALILATQRISRPIVNLFVSRDLGGSSAATEAVAILT
ATYPVGHMPYGWLTEIRAVYPAFDKNNPSNKLVSTSNTVTAAHIKKFTFVCMALSLTLCFVMFWTPNVSE
KILIDIIGVDFAFAELCVVPLRIFSFFPVPVTVRAHLTGWLMTLKKTFVLAPSSVLRIIVLIASLVVLPY
LGVHGATLGVGSLLAGFVGESTMVAIAACYVYRKQKKKMENESATEGEDSAMTDMPPTEEVTDIVEMREE
NE

>ZSWM6_HUMAN
MAERGQQPPPAKRLCCRPGGGGGGGGSSGGGGGAGGGYSSACRPGPRAGGAAAAAACGGGAALGLLPPGK
TQSPESLLDIAARRVAEKWPFQRVEERFERIPEPVQRRIVYWSFPRSEREICMYSSFNTGGGAAGGPGDD
SGGGGGAGGGGGGGSSSSPAATSAAATSAAAAAAAAAAAAAAAAGAGAPSVGAAGAADGGDETRLPFRRG
IALLESGCVDNVLQVGFHLSGTVTEPAIQSEPETVCNVAISFDRCKITSVTCSCGNKDIFYCAHVVALSL

YRIRKPDQVKLHLPISETLFQMNRDQLQKFVQYLITVHHTEVLPTAQKLADEILSQNSEINQVHGAPDPT
AGASIDDENCWHLDEEQVQEQVKLFLSQGGYHGSGKQLNLLFAKVREMLKMRDSNGARMLTLITEQFMAD
PRLSLWRQQGTAMTDKYRQLWDELGALWMCIVLNPHCKLEQKASWLKQLKKWNSVDVCPWEDGNHGSELP
NLTNALPQGANANQDSSNRPHRTVFTRAIEACDLHWQDSHLQHIISSDLYTNYCYHDDTENSLFDSRGWP
LWHEHVPTACARVDALRSHGYPREALRLAIAIVNTLRRQQQKQLEMFRTQKKELPHKNITSITNLEGWVG
HPLDPVGTLFSSLMEACRIDDENLSGFSDFTENMGQCKSLEYQHLPAHKFLEEGESYLTLAVEVALIGLG
QQRIMPDGLYTQEKVCRNEEQLISKLQEIELDDTLVKIFRKQAVFLLEAGPYSGLGEIIHRESVPMHTFA
KYLFTSLLPHDAELAYKIALRAMRLLVLESTAPSGDLTRPHHIASVVPNRYPRWFTLSHIESQQCELAST
MLTAAKGDVRRLETVLESIQKNIHSSSHIFKLAQDAFKIATLMDSLPDITLLKVSLELGLQVMRMTLSTL
NWRRREMVRWLVTCATEVGVYALDSIMQTWFTLFTPTEATSIVATTVMSNSTIVRLHLDCHQQEKLASSA
RTLALQCAMKDPQNCALSALTLCEKDHIAFETAYQIVLDAATTGMSYTQLFTIARYMEHRGYPMRAYKLA
TLAMTHLNLSYNQDTHPAINDVLWACALSHSLGKNELAAIIPLVVKSVKCATVLSDILRRCTLTTPGMVG
LHGRRNSGKLMSLDKAPLRQLLDATIGAYINTTHSRLTHISPRHYSEFIEFLSKARETFLMAHDGHIQFT
QFIDNLKQIYKGKKKLMMLVRERFG

>VAT1L_HUMAN
MAKEGVEKAEETEQMIEKEAGKEPAEGGGGDGSHRLGDAQEMRAVVLAGFGGLNKLRLFRKAMPEPQDGE
LKIRVKACGLNFIDLMVRQGNIDNPPKTPLVPGFECSGIVEALGDSVKGYEIGDRVMAFVNYNAWAEVVC
TPVEFVYKIPDDMSFSEAAAFPMNFVTAYVMLFEVANLREGMSVLVHSAGGGVGQAVAQLCSTVPNVTVF
GTASTFKHEAIKDSVTHLFDRNADYVQEVKRISAEGVDIVLDCLCGDNTGKGLSLLKPLGTYILYGSSNM
VTGETKSFFSFAKSWWQVEKVNPIKLYEENKVIAGFSLLNLLFKQGRAGLIRGVVEKLIGLYNQKKIKPV
VDSLWALEEVKEAMQRIHDRGNIGKLILDVEKTPTPLMANDSTETSEAGEEEEDHEGDSENKERMPFIQ

>ZDBF2_HUMAN
MQKRQGYCSYCRVQYNNLEQHLFSAQHRSLTRQSRRQICTSSLMERFLQDVLQHHPYHCQESSSTQDETH
VNTGSSSEVVHLDDAFSEEEEEDEDKVEDEDATEERPSEVSEPIEELHSRPHKSQEGTQEVSVRPSVIQK
LEKGQQQPLEFVHKIGASVRKCNLVDIGQATNNRSNLVRPPVICNAPASCLPESSNDRPVTANTTSLPPA
AHLDSVSKCDPNKVEKYLEQPDGASRNPVPSSHVETTSFSYQKHKESNRKSLRMNSDKLVLWKDVKSQGK
TLSAGLKFHERMGTKGSLRVKSPSKLAVNPNKTDMPSNKGIFEDTIAKNHEEFFSNMDCTQEEKHLVFNK
TAFWEQKCSVSSEMKFDCISLQSASDQPQETAQDLSLWKEEQIDQEDNYESRGSEMSFDCSSSFHSLTDQ
SKVSAKEVNLSKEVRTDVQYKNNKSYVSKISSDCDDILHLVTNQSQMIVKEISLQNARHISLVDQSYESS
SSETNFDCDASPQSTSDYPQQSVTEVNLPKEVHIGLVDKNYGSSSSEVSADSVFPLQSVVDRPPVAVTET
KLRKKAHTSLVDNYGSSCSETSFDCDVSLESVVDHPQLTVKGRNLKGRQVHLKHKKRKPSSAKAHLDCDV
SLGTVADESQRAVEKINLLKEKNADLMDMNCESHGPEMGFQADAQLADQSQVAEIERQKVDVDLENKSVQ
SSRSSLSSDSPASLYHSAHDEPQEALDEVNLKELNIDMEVRSYDCSSSELTFDSDPPLLSVTEQSHLDAE
GKERHIDLEDESCESDSSEITFDSDIPLYSVIDQPEVAVYEEETVDLESKSNESCVSEITFDSDIPLHSG
NDHPEVAVKEVIQKEEYIHLERKNDEPSGSEISSDSHAPLHSVTNSPEVAVKKLNPQKEEQVHLENKENE
PIDSEVSLDYNIIFHSVTGRSEDPIKEISLHTKEHMYLENKSVFETSLDSDVPLQAATHKPEVIVKETWL
QREKHAEFQGRSTEFSGSKTSLDSGVPHYSVTEPQVAVNKINRKKQYVLENKNDKCSGSEIILDSNVPPQ
SMTDQPQLAFLKEKHVNLKDKNSKSGDSKITFDSEQLQEAVKKIDQWKEEVIGLKNKINEPSTYKLIHHP
DVSVQSVADQPKVAIKHVNLGNENHMYLEVKNSQYSCSEMNLDSGFLGQSIVNRPQITILEQEHIELEGK
HNQCCGSEVSFDSDDPLQSVADRLRETVKEISLWKDEEVDTEDRRNEAKGFEIMYDSDVLQPVAGQPEEV
VKEVSLWKEHVDLENKIVKPTDSRINFDSHEPLQSVTNKIPGANKEINLLREEHVCLDDKGYVPSDSEII
YVSNIPLQSVIKQPHILEEEHASLEDKSSNSYSPEESSDSNDSFQAAADELQKPVKEINLWKEDHIYLED

KSYKLGDFDVSYASHIPVQFVTDQSSVPVKEINLQKKDHNDLENKNCEVCGSEIKCHSCVHLQSEVDQPQ
VSYKEADLQKEEHVVMEEKTDQPSDSEMMYDSDVPFQIVVNQFPGSVKETHLPKVVLVDLVPGDSDYEVI
SDDIPLQLVTDPPQLTVKDISCINTECIDIEDKSCDFFGSEVRCNCKASTPSMTNQCKETFKIINRKKDY
IILGEPSCQSCGSEMNFNVDASDQSMTYESQGPDEKMVKYIDSEDKSCGYNGSKGKFNLEDTSHRTTHRL
QKAHKEASLRKDPRNAGLKGKSCQSSASAVDFGASSKSALHRRADKKKRSKLKHRDLEVSCEPDGFEMNF
QCAPPLPSDTDQPQETVKKRHPCKKVSSDLKEKNHDSQSSSVLKVDSVRNLKKAKDVIEDNPDEPVLEAL
PHVPPSFVGKTWSQIMREDDIKINALVKEFREGRFHCYFDDDCETKKVSSKGKKKVTWADLQGKEDTAPT
QAVSESDDIVCGISDIDDLSVALDKPCHRHPPAERPPKQKGRVASQCQTAKISHSTQTSCKNYPVMKRKI
IRQEEDPPKSKCSRLQDDRKTKKKVKIGTVEFPASCTKVLKPMQPKALVCVLSSLNIKLKEGEGLPFPKM
RHHSWDNDIRFICKYKRNIFDYYEPLIKQIVISPPLSVIVPEFERRNWVKIHFNRSNQNSSAGDNDADGQ
GSASAPLMAVPARYGFNSHQGTSDSSLFLEESKVLHARELPKKRNFQLTFLNHDVVKISPKSVRNKLLES
QSKKKIHGKRVTTSSNKLGFPKKVYKPIILQQKPRKASEKQSIWIRTKPSDIIRKYISKYSVFLRHRYQS
RSAFLGRYLKKKKSVVSRLKKAKRTAKVLLNSSVPPAGAEELSSAMANPPPKRPVRASCRVARRRKKTDE
SYHGRQKGPSTPVRAYDLRSSSCLQQRERMMTRLANKLRGNEVK

>ROBO2_HUMAN
MSLLMFTQLLLCGFLYVRVDGSRLRQEDFPPRIVEHPSDVIVSKGEPTTLNCKAEGRPTPTIEWYKDGER
VETDKDDPRSHRMLLPSGSLFFLRIVHGRRSKPDEGSYVCVARNYLGEAVSRNASLEVALLRDDFRQNPT
DVVVAAGEPAILECQPPRGHPEPTIYWKKDKVRIDDKEERISIRGGKLMISNTRKSDAGMYTCVGTNMVG
ERDSDPAELTVFERPTFLRRPINQVVLEEEAVEFRCQVQGDPQPTVRWKKDDADLPRGRYDIKDDYTLRI
KKTMSTDEGTYMCIAENRVGKMEASATLTVRAPPQFVVRPRDQIVAQGRTVTFPCETKGNPQPAVFWQKE
GSQNLLFPNQPQQPNSRCSVSPTGDLTITNIQRSDAGYYICQALTVAGSILAKAQLEVTDVLTDRPPPII
LQGPANQTLAVDGTALLKCKATGDPLPVISWLKEGFTFPGRDPRATIQEQGTLQIKNLRISDTGTYTCVA
TSSSGETSWSAVLDVTESGATISKNYDLSDLPGPPSKPQVTDVTKNSVTLSWQPGTPGTLPASAYIIEAF
SQSVSNSWQTVANHVKTTLYTVRGLRPNTIYLFMVRAINPQGLSDPSPMSDPVRTQDISPPAQGVDHRQV
QKELGDVLVRLHNPVVLTPTTVQVTWTVDRQPQFIQGYRVMYRQTSGLQATSSWQNLDAKVPTERSAVLV
NLKKGVTYEIKVRPYFNEFQGMDSESKTVRTTEEAPSAPPQSVTVLTVGSYNSTSISVSWDPPPPDHQNG
IIQEYKIWCLGNETRFHINKTVDAAIRSVIIGGLFPGIQYRVEVAASTSAGVGVKSEPQPIIIGRRNEVV
ITENNNSITEQITDVVKQPAFIAGIGGACWVILMGFSIWLYWRRKKRKGLSNYAVTFQRGDGGLMSNGSR
PGLLNAGDPSYPWLADSWPATSLPVNNSNSGPNEIGNFGRGDVLPPVPGQGDKTATMLSDGAIYSSIDFT
TKTSYNSSSQITQATPYATTQILHSNSIHELAVDLPDPQWKSSIQQKTDLMGFGYSLPDQNKGNNGGKGG
KKKKNKNSSKPQKNNGSTWANVPLPPPPVQPLPGTELEHYAVEQQENGYDSDSWCPPLPVQTYLHQGLED
ELEEDDDRVPTPPVRGVASSPAISFGQQSTATLTPSPREEMQPMLQAHLDELTRAYQFDIAKQTWHIQSN
NQPPQPPVPPLGYVSGALISDLETDVADDDADDEEEALEIPRPLRALDQTPGSSMDNLDSSVTGKAFTSS
QRPRPTSPFSTDSNTSAALSQSQRPRPTKKHKGGRMDQQPALPHRREGMTDEEALVPYSKPSFPSPGGHS
SSGTASSKGSTGPRKTEVLRAGHQRNASDLLDIGYMGSNSQGQFTGEL

>AGO4_HUMAN
MEALGPGPPASLFQPPRRPGLGTVGKPIRLLANHFQVQIPKIDVYHYDVDIKPEKRPRRVNREVVDTMVR
HFKMQIFGDRQPGYDGKRNMYTAHPLPIGRDRVDMEVTLPGEGKDQTFKVSVQWVSVVSLQLLLEALAGH
LNEVPDDSVQALDVITRHLPSMRYTPVGRSFFSPPEGYYHPLGGGREVWFGFHQSVRPAMWNMMLNIDVS
ATAFYRAQPIIEFMCEVLDIQNINEQTKPLTDSQRVKFTKEIRGLKVEVTHCGQMKRKYRVCNVTRRPAS
HQTFPLQLENGQAMECTVAQYFKQKYSLQLKYPHLPCLQVGQEQKHTYLPLEVCNIVAGQRCIKKLTDNQ
TSTMIKATARSAPDRQEEISRLVKSNSMVGGPDPYLKEFGIVVHNEMTELTGRVLPAPMLQYGGRNKTVA

TPNQGVWDMRGKQFYAGIEIKVWAVACFAPQKQCREDLLKSFTDQLRKISKDAGMPIQGQPCFCKYAQGA
DSVEPMFKHLKMTYVGLQLIVVILPGKTPVYAEVKRVGDTLLGMATQCVQVKNVVKTSPQTLSNLCLKIN
AKLGGINNVLVPHQRPSVFQQPVIFLGADVTHPPAGDGKKPSIAAVVGSMDGHPSRYCATVRVQTSRQEI
SQELLYSQEVIQDLTNMVRELLIQFYKSTRFKPTRIIYYRGGVSEGQMKQVAWPELIAIRKACISLEEDY
RPGITYIVVQKRHHTRLFCADKTERVGKSGNVPAGTTVDSTITHPSEFDFYLCSHAGIQGTSRPSHYQVL
WDDNCFTADELQLLTYQLCHTYVRCTRSVSIPAPAYYARLVAFRARYHLVDKDHDSAEGSHVSGQSNGRD
PQALAKAVQIHHDTQHTMYFA

>CHD8_HUMAN
MADPIMDLFDDPNLFGLDSLTDDSFNQVTQDPIEEALGLPSSLDSLDQMNQDGGGGDVGNSSASELVPPP
EETAPTELSKESTAPAPESITLHDYTTQPASQEQPAQPVLQTSTPTSGLLQVSKSQEILSQGNPFMGVSA
TAVSSSSAGGQPPQSAPKIVILKAPPSSSVTGAHVAQIQAQGITSTAQPLVAGTANGGKVTFTKVLTGTP
LRPGVSIVSGNTVLAAKVPGNQAAVQRIVQPSRPVKQLVLQPVKGSAPAGNPGATGPPLKPAVTLTSTPT
QGESKRITLVLQQPQSGGPQGHRHVVLGSLPGKIVLQGNQLAALTQAKNAQGQPAKVVTIQLQVQQPQQK
IQIVPQPPSSQPQPQQPPSTQPVTLSSVQQAQIMGPGQSPGQRLSVPVKVVLQPQAGSSQGASSGLSVVK
VLSASEVAALSSPASSAPHSGGKTGMEENRRLEHQKKQEKANRIVAEAIARARARGEQNIPRVLNEDELP
SVRPEEEGEKKRRKKSAGERLKEEKPKKSKTSGASKTKGKSKLNTITPVVGKKRKRNTSSDNSDVEVMPA
QSPREDEESSIQKRRSNRQVKRKKYTEDLDIKITDDEEEEEVDVTGPIKPEPILPEPVQEPDGETLPSMQ
FFVENPSEEDAAIVDKVLSMRIVKKELPSGQYTEAEEFFVKYKNYSYLHCEWATISQLEKDKRIHQKLKR
FKTKMAQMRHFFHEDEEPFNPDYVEVDRILDESHSIDKDNGEPVIYYLVKWCSLPYEDSTWELKEDVDEG
KIREFKRIQSRHPELKRVNRPQASAWKKLELSHEYKNRNQLREYQLEGVNWLLFNWYNRQNCILADEMGL
GKTIQSIAFLQEVYNVGIHGPFLVIAPLSTITNWEREFNTWTEMNTIVYHGSLASRQMIQQYEMYCKDSR
GRLIPGAYKFDALITTFEMILSDCPELREIEWRCVIIDEAHRLKNRNCKLLDSLKHMDLEHKVLLTGTPL
QNTVEELFSLLHFLEPSQFPSESEFLKDFGDLKTEEQVQKLQAILKPMMLRRLKEDVEKNLAPKQETIIE
VELTNIQKKYYRAILEKNFSFLSKGAGHTNMPNLLNTMMELRKCCNHPYLINGAEEKILTEFREACHIIP
HDFHLQAMVRSAGKLVLIDKLLPKLKAGGHKVLIFSQMVRCLDILEDYLIQRRYLYERIDGRVRGNLRQA
AIDRFSKPDSDRFVFLLCTRAGGLGINLTAADTCIIFDSDWNPQNDLQAQARCHRIGQSKAVKVYRLITR
NSYEREMFDKASLKLGLDKAVLQSMSGRDGNITGIQQFSKKEIEDLLRKGAYAAIMEEDDEGSKFCEEDI
DQILLRRTTTITIESEGKGSTFAKASFVASENRTDISLDDPNFWQKWAKKADLDMDLLNSKNNLVIDTPR
VRKQTRHFSTLKDDDLVEFSDLESEDDERPRSRRHDRHHAYGRTDCFRVEKHLLVYGWGRWRDILSHGRF
KRRMTERDVETICRAILVYCLLHYRGDENIKGFIWDLISPAENGKTKELQNHSGLSIPVPRGRKGKKVKS
QSTFDIHKADWIRKYNPDTLFQDESYKKHLKHQCNKVLLRVRMLYYLRQEVIGDQAEKVLGGAIASEIDI
WFPVVDQLEVPTTWWDSEADKSLLIGVFKHGYEKYNTMRADPALCFLEKAGRPDDKAIAAEHRVLDNFSD
IVEGVDFDKDCEDPEYKPLQGPPKDQDDEGDPLMMMDEEISVIDGDEAQVTQQPGHLFWPPGSALTARLR
RLVTAYQRSYKREQMKIEAAERGDRRRRRCEAAFKLKEIARREKQQRWTRREQTDFYRVVSTFGVEYDPD
TMQFHWDRFRTFARLDKKTDESLTKYFHGFVAMCRQVCRLPPAAGDEPPDPNLFIEPITEERASRTLYRI
ELLRRLREQVLCHPLLEDRLALCQPPGPELPKWWEPVRHDGELLRGAARHGVSQTDCNIMQDPDFSFLAA
RMNYMQNHQAGAPAPSLSRCSTPLLHQQYTSRTASPLPLRPDAPVEKSPEETATQVPSLESLTLKLEHEV
VARSRPTPQDYEMRVSPSDTTPLVSRSVPPVKLEDEDDSDSELDLSKLSPSSSSSSSSSSSSSSTDESED
EKEEKLTDQSRSKLYDEESLLSLTMSQDGFPNEDGEQMTPELLLLQERQRASEWPKDRVLINRIDLVCQA
VLSGKWPSSRRSQEMVTGGILGPGNHLLDSPSLTPGEYGDSPVPTPRSSSAASMAEEEASAVSTAAAQFT
KLRRGMDEKEFTVQIKDEEGLKLTFQKHKLMANGVMGDGHPLFHKKKGNRKKLVELEVECMEEPNHLDVD
LETRIPVINKVDGTLLVGEDAPRRAELEMWLQGHPEFAVDPRFLAYMEDRRKQKWQRCKKNNKAELNCLG
MEPVQTANSRNGKKGHHTETVFNRVLPGPIAPESSKKRARRMRPDLSKMMALMQGGSTGSLSLHNTFQHS

SSGLQSVSSLGHSSATSASLPFMPFVMGGAPSSPHVDSSTMLHHHHHHPHPHHHHHHHPGLRAPGYPSSP
VTTASGTTLRLPPLQPEEDDDEDEEDDDDLSQGYDSSERDFSLIDDPMMPANSDSSEDADD

>PCD18_HUMAN
MHQMNAKMHFRFVFALLIVSFNHDVLGKNLKYRIYEEQRVGSVIARLSEDVADVLLKLPNPSTVRFRAMQ
RGNSPLLVVNEDNGEISIGATIDREQLCQKNLNCSIEFDVITLPTEHLQLFHIEVEVLDINDNSPQFSRS
LIPIEISESAAVGTRIPLDSAFDPDVGENSLHTYSLSANDFFNIEVRTRTDGAKYAELIVVRELDRELKS
SYELQLTASDMGVPQRSGSSILKISISDSNDNSPAFEQQSYIIQLLENSPVGTLLLDLNATDPDEGANGK
IVYSFSSHVSPKIMETFKIDSERGHLTLFKQVDYEITKSYEIDVQAQDLGPNSIPAHCKIIIKVVDVNDN
KPEININLMSPGKEEISYIFEGDPIDTFVALVRVQDKDSGLNGEIVCKLHGHGHFKLQKTYENNYLILTN
ATLDREKRSEYSLTVIAEDRGTPSLSTVKHFTVQINDINDNPPHFQRSRYEFVISENNSPGAYITTVTAT
DPDLGENGQVTYTILESFILGSSITTYVTIDPSNGAIYALRIFDHEEVSQITFVVEARDGGSPKQLVSNT
TVVLTIIDENDNVPVVIGPALRNNTAEITIPKGAESGFHVTRIRAIDRDSGVNAELSCAIVAGNEENIFI
IDPRSCDIHTNVSMDSVPYTEWELSVIIQDKGNPQLHTKVLLKCMIFEYAESVTSTAMTSVSQASLDVSM
IIIISLGAICAVLLVIMVLFATRCNREKKDTRSYNCRVAESTYQHHPKRPSRQIHKGDITLVPTINGTLP
IRSHHRSSPSSSPTLERGQMGSRQSHNSHQSLNSLVTISSNHVPENFSLELTHATPAVEQVSQLLSMLHQ
GQYQPRPSFRGNKYSRSYRYALQDMDKFSLKDSGRGDSEAGDSDYDLGRDSPIDRLLGEGFSDLFLTDGR
IPAAMRLCTEECRVLGHSDQCWMPPLPSPSSDYRSNMFIPGEEFPTQPQQQHPHQSLEDDAQPADSGEKK
KSFSTFGKDSPNDEDTGDTSTSSLLSEMSSVFQRLLPPSLDTYSECSEVDRSNSLERRKGPLPAKTVGYP
QGVAAWAASTHFQNPTTNCGPPLGTHSSVQPSSKWLPAMEEIPENYEEDDFDNVLNHLNDGKHELMDASE
LVAEINKLLQDVRQS

>GPAT1_HUMAN
MDESALTLGTIDVSYLPHSSEYSVGRCKHTSEEWGECGFRPTIFRSATLKWKESLMSRKRPFVGRCCYSC
TPQSWDKFFNPSIPSLGLRNVIYINETHTRHRGWLARRLSYVLFIQERDVHKGMFATNVTENVLNSSRVQ
EAIAEVAAELNPDGSAQQQSKAVNKVKKKAKRILQEMVATVSPAMIRLTGWVLLKLFNSFFWNIQIHKGQ
LEMVKAATETNLPLLFLPVHRSHIDYLLLTFILFCHNIKAPYIASGNNLNIPIFSTLIHKLGGFFIRRRL
DETPDGRKDVLYRALLHGHIVELLRQQQFLEIFLEGTRSRSGKTSCARAGLLSVVVDTLSTNVIPDILII
PVGISYDRIIEGHYNGEQLGKPKKNESLWSVARGVIRMLRKNYGCVRVDFAQPFSLKEYLESQSQKPVSA
LLSLEQALLPAILPSRPSDAADEGRDTSINESRNATDESLRRRLIANLAEHILFTASKSCAIMSTHIVAC
LLLYRHRQGIDLSTLVEDFFVMKEEVLARDFDLGFSGNSEDVVMHAIQLLGNCVTITHTSRNDEFFITPS
TTVPSVFELNFYSNGVLHVFIMEAIIACSLYAVLNKRGLGGPTSTPPNLISQEQLVRKAASLCYLLSNEG
TISLPCQTFYQVCHETVGKFIQYGILTVAEHDDQEDISPSLAEQQWDKKLPEPLSWRSDEEDEDSDFGEE
QRDCYLKVSQSKEHQQFITFLQRLLGPLLEAYSSAAIFVHNFSGPVPEPEYLQKLHKYLITRTERNVAVY
AESATYCLVKNAVKMFKDIGVFKETKQKRVSVLELSSTFLPQCNRQKLLEYILSFVVL

>CL035_HUMAN
MNWNEKPKSATLPPLYPKSQPPFLHQSLINQITTTSQSSFSYPGSNQEACMYPGNSNPISQPLLNIQNYP
QQISVSDMHNGTVVASHTSVERITYANVNGPKQLTHNLQMSSGVTQNVWLNSPMRNPVHSHIGATVSHQT
DFGANVPNMPALQSQLITSDTYSMQMQMIPSNSTRLPVAYQGNQGLNQSFSEQQVDWTQQCISKGLTYPD
YRPPPKLYRYSPQSFLPDSTIQKQNFIPHTSLQVKNSQLLNSVLTLPSRQTSAVPSQQYATQTDKRPPPP
PYNCRYGSQPLQSTQHITKHLSMEVPQSREMLSSEIRTSFQQQWQNPNENVSTIGNFTNLKVNTNSKQPF
NSPIRSSVDGVQTLAQTNEEKIMDSCNPTSNQVLDTSVAKEKLVRDIKTLVEIKQKFSELARKIKINKDL
LMAAGCIKMTNTSYSEPAQNSKLSLKQTAKIQSGPQITPVMPENAERQTPTVVESAETNKTQCMLNSDIQ

EVNCRRFNQVDSVLPNPVYSEKRPMPDSSHDVKVLTSKTSAVEMTQAVLNTQLSSENVTKVEQNSPAVCE
TISVPKSMSTEEYKSKIQNENMLLLALLSQARKTQKTVLKDANQTIQDSKPDSCEMNPNTQMTGNQLNLK
NMETPSTSNVSGRVLDNSFCSGQESSTKGMPAKSDSSCSMEVLATCLSLWKKQPSDTAKEKECDKLRTNT
TAVGISKPANIHVKSPCSVVGNSNSQNKISNPSQQTALSMVMHNYESSGINITKGTELQIAVVSPLVLSE
VKTLSVKGITPAVLPETVYPVIKEGSVCSLQNQLAENAKATAALKVDVSGPVASTATSTKIFPLTQKEKQ
NESTNGNSEVTPNVNQGKHNKLESAIHSPMNDQQISQESRNSTVVSSDTLQIDNICSLVEGDTSYNSQIA
KIFSSLPLKMVEPQKPSLPNQQGIGSREPEKQLDNTTENKDFGFQKDKPVQCTDVSHKICDQSKSEPPLE
SSFNNLETNRVILEKSSLEHATEKSTANDTCSSAAIQEDIYPQEIDASSNYTPQDPARNEIHSDKAPVLY
LHDQLSELLKEFPYGIEAVNTREGSVGQQTTYQTSEDQTADKTSSDSKDPADQIQITILSSEQMKEIFPE
QDDQPYVVDKLAEPQKEEPITEVVSQCDLQAPAAGQSRDSVILDSEKDDIHCCALGWLSMVYEGVPQCQC
NSIKNSSSEEEKQKEQCSPLDTNSCKQGERTSDRDVTVVQFKSLVNNPKTPPDGKSHFPELQDDSRKDTP
KTKHKSLPRTEQELVAGQFSSKCDKLNPLQNHKRKKLRFHEVTFHSSNKMTASYEQASQETRQKKHVTQN
SRPLKTKTAFLPNKDVYKKHSSLGQSLSPEKIKLKLKSVSFKQKRKLDQGNVLDMEVKKKKHDKQEQKGS
VGATFKLGDSLSNPNERAIVKEKMVSNTKSVDTKASSSKFSRILTPKEYLQRQKHKEALSNKASKKICVK
NVPCDSEHMRPSKLAVQVESCGKSNEKHSSGVQTSKESLNGLTSHGKNLKIHHSQESKTYNILRNVKEKV
GGKQPDKIWIDKTKLDKLTNISNEAQFSQMPPQVKDQKKLYLNRVGFKCTERESISLTKLESSPRKLHKD
KRQENKHKTFLPVKGNTEKSNMLEFKLCPDILLKNTNSVEERKDVKPHPRKEQAPLQVSGIKSTKEDWLK
FVATKKRTQKDSQERDNVNSRLSKRSFSADGFEMLQNPVKDSKEMFQTYKQMYLEKRSRSLGSSPVK

>PLXA4_HUMAN
MKAMPWNWTCLLSHLLMVGMGSSTLLTRQPAPLSQKQRSFVTFRGEPAEGFNHLVVDERTGHIYLGAVNR
IYKLSSDLKVLVTHETGPDEDNPKCYPPRIVQTCNEPLTTTNNVNKMLLIDYKENRLIACGSLYQGICKL
LRLEDLFKLGEPYHKKEHYLSGVNESGSVFGVIVSYSNLDDKLFIATAVDGKPEYFPTISSRKLTKNSEA
DGMFAYVFHDEFVASMIKIPSDTFTIIPDFDIYYVYGFSSGNFVYFLTLQPEMVSPPGSTTKEQVYTSKL
VRLCKEDTAFNSYVEVPIGCERSGVEYRLLQAAYLSKAGAVLGRTLGVHPDDDLLFTVFSKGQKRKMKSL
DESALCIFILKQINDRIKERLQSCYRGEGTLDLAWLKVKDIPCSSALLTIDDNFCGLDMNAPLGVSDMVR
GIPVFTEDRDRMTSVIAYVYKNHSLAFVGTKSGKLKKIRVDGPRGNALQYETVQVVDPGPVLRDMAFSKD
HEQLYIMSERQLTRVPVESCGQYQSCGECLGSGDPHCGWCVLHNTCTRKERCERSKEPRRFASEMKQCVR
LTVHPNNISVSQYNVLLVLETYNVPELSAGVNCTFEDLSEMDGLVVGNQIQCYSPAAKEVPRIITENGDH
HVVQLQLKSKETGMTFASTSFVFYNCSVHNSCLSCVESPYRCHWCKYRHVCTHDPKTCSFQEGRVKLPED
CPQLLRVDKILVPVEVIKPITLKAKNLPQPQSGQRGYECILNIQGSEQRVPALRFNSSSVQCQNTSYSYE
GMEINNLPVELTVVWNGHFNIDNPAQNKVHLYKCGAMRESCGLCLKADPDFACGWCQGPGQCTLRQHCPA
QESQWLELSGAKSKCTNPRITEIIPVTGPREGGTKVTIRGENLGLEFRDIASHVKVAGVECSPLVDGYIP
AEQIVCEMGEAKPSQHAGFVEICVAVCRPEFMARSSQLYYFMTLTLSDLKPSRGPMSGGTQVTITGTNLN
AGSNVVVMFGKQPCLFHRRSPSYIVCNTTSSDEVLEMKVSVQVDRAKIHQDLVFQYVEDPTIVRIEPEWS
IVSGNTPIAVWGTHLDLIQNPQIRAKHGGKEHINICEVLNATEMTCQAPALALGPDHQSDLTERPEEFGF
ILDNVQSLLILNKTNFTYYPNPVFEAFGPSGILELKPGTPIILKGKNLIPPVAGGNVKLNYTVLVGEKPC
TVTVSDVQLLCESPNLIGRHKVMARVGGMEYSPGMVYIAPDSPLSLPAIVSIAVAGGLLIIFIVAVLIAY
KRKSRESDLTLKRLQMQMDNLESRVALECKEAFAELQTDIHELTSDLDGAGIPFLDYRTYTMRVLFPGIE
DHPVLRDLEVPGYRQERVEKGLKLFAQLINNKVFLLSFIRTLESQRSFSMRDRGNVASLIMTVLQSKLEY
ATDVLKQLLADLIDKNLESKNHPKLLLRRTESVAEKMLTNWFTFLLYKFLKECAGEPLFSLFCAIKQQME
KGPIDAITGEARYSLSEDKLIRQQIDYKTLVLSCVSPDNANSPEVPVKILNCDTITQVKEKILDAIFKNV
PCSHRPKAADMDLEWRQGSGARMILQDEDITTKIENDWKRLNTLAHYQVPDGSVVALVSKQVTAYNAVNN
STVSRTSASKYENMIRYTGSPDSLRSRTPMITPDLESGVKMWHLVKNHEHGDQKEGDRGSKMVSEIYLTR

LLATKGTLQKFVDDLFETIFSTAHRGSALPLAIKYMFDFLDEQADKHGIHDPHVRHTWKSNCLPLRFWVN
MIKNPQFVFDIHKNSITDACLSVVAQTFMDSCSTSEHRLGKDSPSNKLLYAKDIPSYKNWVERYYSDIGK
MPAISDQDMNAYLAEQSRMHMNEFNTMSALSEIFSYVGKYSEEILGPLDHDDQCGKQKLAYKLEQVITLM
SLDS

>K1549_HUMAN
MELVLKKSTGHSAAQVALTETAPGSQHSSPLHVTAPPSATTFDTAFFNQGKQTKSTADPSIFVATYVSVT
SKEVAVNDDEMDNFLPDTHWTTPRMVSPIQYITVSPPGLPREALEPMLTPSLPMVSLQDEEVTSGWQNTT
RQPAAYAESASHFHTFRSAFRTSEGIVPTPGRNLVLYPTDAYSHLSSRTLPEIVASLTEGVETTLFLSSR
SLMPQPLGDGITIPLPSLGEVSQPPEEVWATSADRYTDVTTVLSQSLEETISPRTYPTVTASHAALAFSR
THSPLLSTPLAFASSASPTDVSSNPFLPSDSSKTSELHSNSALPGPVDNTHILSPVSSFRPYTWCAACTV
PSPQQVLATSLMEKDVGSGDGAETLCMTVLEESSISLMSSVVADFSEFEEDPQVFNTLFASRPIVPLSSR
SMEISETSVGISAEVDMSSVTTTQVPPAHGRLSVPASLDPTAGSLSVAETQVTPSSVTTAFFSVITSILL
DSSFSVIANKNTPSLAVRDPSVFTPYSLVPSVESSLFSDQERSSFSEHKPRGALDFASSFFSTPPLELSG
SISSPSEAPASLSLMPSDLSPFTSQSFSPLVETFTLFDSSDLQSSQLSLPSSTNLEFSQLQPSSELPLNT
IMLLPSRSEVSPWSSFPSDSLEFVEASTVSLTDSEAHFTSAFIETTSYLESSLISHESAVTALVPPGSES
FDILTAGIQATSPLTTVHTTPILTESSLFSTLTPPDDQISALDGHVSVLASFSKAIPTGTVLITDAYLPS
GSSFVSEATPFPLPTELTVVGPSLTPTEVPLNTSTEVSTTSTGAATGGPLDSTLMGDAASQSPPESSAAP
PLPSLRPVTAFTLEATVDTPTLATAKPPYVCDITVPDAYLITTVLARRAVQEYIITAIKEVLRIHFNRAV
ELKVYELFTDFTFLVTSGPFVYTAISVINVLINSKLVRDQTPLILSVKPSFLVPESRFQVQTVLQFVPPS
VDTGFCNFTQRIEKGLMTALFEVRKHHQGTYNLTVQILNITISSSRVTPRRGPVNIIFAVKSTQGFLNGS
EVSELLRNLSVVEFSFYLGYPVLQIAEPFQYPQLNLSQLLKSSWVRTVLLGVMEKQLQNEVFQAEMERKL
AQLLSEVSTRRRMWRRATVAAGNSVVQVVNVSRLEGDDNPVQLIYFVEDQDGERLSAVKSSDLINKMDLQ
RAAIILGYRIQGVIAQPVDRVKRPSPESQSNNLWVIVGVVIPVLVVMVIVVILYWKLCRTDKLDFQPDTV
ANIQQRQKLQIPSVKGFDFAKQHLGQHNKDDILIIHEPAPLPGPLKDHTTPSENGDVPSPKSKIPSKNVR
HRGRVSPSDADSTVSEESSERDAGDKTPGAVNDGRSHRAPQSGPPLPSSGNEQHSSASIFEHVDRISRPP
EASRRVPSKIQLIAMQPIPAPPVQRPSPADRVAESNKINKEIQTALRHKSEIEHHRNKIRLRAKRRGHYE
FPVVDDLSSGDTKERHRVYRRAQMQIDKILDPTASVPSVFIEPRKSSRIKRSPKPRRKHQVNGCPADAEK
DRLITTDSDGTYRRPPGVHNSAYIGCPSDPDLPADVQTPSSVELGRYPALPFPASQYIPPQPSIEEARQT
MHSLLDDAFALVAPSSQPASTAGVGPGVPPGLPANSTPSQEERRATQWGSFYSPAQTANNPCSRYEDYGM
TPPTGPLPRPGFGPGLLQSTELVPPDPQQPQASAEAPFAARGIYSEEMPSVARPRPVGGTTGSQIQHLTQ
VGIASRIGAQPVEIPPSRGSQYGGPGWPSYGEDEAGRREATHMLGHQEYSSSPLFQVPRTSGREPSAPSG
NLPHRGLQGPGLGYPTSSTEDLQPGHSSASLIKAIREELLRLSQKQSTVQNFHS

>E41L5_HUMAN
MLSFFRRTLGRRSMRKHAEKERLREAQRAATHIPAAGDSKSIITCRVSLLDGTDVSVDLPKKAKGQELFD
QIMYHLDLIESDYFGLRFMDSAQVAHWLDGTKSIKKQVKIGSPYCLHLRVKFYSSEPNNLREELTRYLFV
LQLKQDILSGKLDCPFDTAVQLAAYNLQAELGDYDLAEHSPELVSEFRFVPIQTEEMELAIFEKWKEYRG
QTPAQAETNYLNKAKWLEMYGVDMHVVKARDGNDYSLGLTPTGVLVFEGDTKIGLFFWPKITRLDFKKNK
LTLVVVEDDDQGKEQEHTFVFRLDHPKACKHLWKCAVEHHAFFRLRGPVQKSSHRSGFIRLGSRFRYSGK
TEYQTTKTNKARRSTSFERRPSKRYSRRTLQMKACATKPEELSVHNNVSTQSNGSQQAWGMRSALPVSPS
ISSAPVPVEIENLPQSPGTDQHDRKCIPLNIDLLNSPDLLEATIGDVIGASDTMETSQALNDVNVATRLP
GLGEPEVEYETLKDTSEKLKQLEMENSPLLSPRSNIDVNINSQEEVVKLTEKCLNNVIESPGLNVMRVPP
DFKSNILKAQVEAVHKVTKEDSLLSHKNANVQDAATNSAVLNENNVPLPKESLETLMLITPADSGSVLKE

ATDELDALLASLTENLIDHTVAPQVSSTSMITPRWIVPQSGAMSNGLAGCEMLLTGKEGHGNKDGISLIS
PPAPFLVDAVTSSGPILAEEAVLKQKCLLTTEL

>FBSL_HUMAN
MEAKVRPSRRSRAQRDRGRRREAARDARAQSPSSGDEPEPSPGKENAGLRGAPPRGAAPAPRTARPPRRR
RRESSSQEEEVIDGFAIASFSTLEALEKDMALKPHERKEKWERRLIKKPRESETCPPAEPSENRRPLEAG
SPGQDLEPACDGARKVPLQPSKQMKVTVSKGGDRDSDDDSVLEATSSRDPLSDSSAHAVSGRGYSCDSES
GPDDKASVGSEKLFAPGTDKGPALEKSEAKAGPVPKVSGLERSRELSAESFLPTASPAPHAAPCPGPPPG
SRANPLVKKEPPAPHRHTPQPPPPQPRGLLPTHVPASLGAFAGHSQAAANGLHGLSRSSSAPLGLGKHVS
LSPHGPGPHLSTSHLALRSQAQHQLHAAMFAAPPTLPPPPALPASSLVLPGHPADHELLRQELNTRFLVQ
SAERPGASLGPGALLRAEFHQHQHTHQHTHQHTHQHQHTFAPFPAGLPPTPPAAPPPFDKYAPKLDSPYF
RHSSVSFFPSFPPAIPGLPTLLPHPGPFGSLQGAFQPKVSDPYRAVVKVSTCWEGPWQGRTLVPPGRPRG
ARDSRSLQKTWVGVAPAPLSASILSQKPGRWCAVHVQIAWQIYRHQQKIKEMQLDPHKLEVGAKLDLFGR
PPAPGVFAGFHYPQDLARPLFPSTGAAHPASNPFGPSAHPGSFLPTGPLTDPFSRPSTFGGLGSLSSHAF
GGLGSHALAPGGSIFAPKEGSSVHGLPSPHEAWNRLHRAPPSFPAPPPWPKSVDAERVSALTNHDREPDN
GKEEQERDLLEKTRLLSRASPATPAGHPVSGLLLRAQSELGRSGAPAEREAEPRVKESRSPAKEEAAKMP
ARASPPHSKAAPGDVKVKEERGEDEASEPPAGGLHPAPLQLGLGRERLGAPGFAWEPFRGLELPRRAFPA
AAPAPGSAALLEPPERPYRDREPHGYSPERLRGELERARAPHLPPAAPALDGALLPSLGALHFPRLSPAA
LHNGLLARTPPAAAALGAPPPLVTAAGPPTPPGPPRSRTTPLGGLGPGEARDYSPSRNPPEVEAR

>TRI39_HUMAN
MAETSLLEAGASAASTAAALENLQVEASCSVCLEYLKEPVIIECGHNFCKACITRWWEDLERDFPCPVCR
KTSRYRSLRPNRQLGSMVEIAKQLQAVKRKIRDESLCPQHHEALSLFCYEDQEAVCLICAISHTHRAHTV
VPLDDATQEYKEKLQKCLEPLEQKLQEITRCKSSEEKKPGELKRLVESRRQQILREFEELHRRLDEEQQV
LLSRLEEEEQDILQRLRENAAHLGDKRRDLAHLAAEVEGKCLQSGFEMLKDVKSTLEKNIPRKFGGSLST
ICPRDHKALLGLVKEINRCEKVKTMEVTSVSIELEKNFSNFPRQYFALRKILKQLIADVTLDPETAHPNL
VLSEDRKSVKFVETRLRDLPDTPRRFTFYPCVLATEGFTSGRHYWEVEVGDKTHWAVGVCRDSVSRKGEL
TPLPETGYWRVRLWNGDKYAATTTPFTPLHIKVKPKRVGIFLDYEAGTLSFYNVTDRSHIYTFTDTFTEK
LWPLFYPGIRAGRKNAAPLTIRPPTDWE

>TPIP1_HUMAN
MGSSSEASFRSAQASCSGARRQGLGRGDQNLSVMPPNGRAQTHTPGWVSDPLVLGAQVHGGCRGIEALSV
SSGSWSSATVWILTGLGLGLSRPFLPGATVLRDRPLGSAFELSYDQKKAPLRLQ

>Q9HCN2-3
MGSSSEASFRSAQASCSGARRQGLGRGDQNLSVMPPNGRAQTHTPGWVSPCSENRDGLLPATAPGRLCSH
RGADIPSFQTHQDPVTASGSSELHADCPQFRALDRAGN

>TMM8A_HUMAN
MGRAGTGTGGEAVAAVVAGPLLLLLLLARPPPASAGYSGKSEVGLVSEHFSQAPQRLSFYSWYGSARLFRF
RVPPDAVLLRWLLQVSRESGAACTDAEITVHFRSGAPPVINPLGTSFPDDTAVQPSFQVGVPLSTTPRSN
ASVNVSHPAPGDWFVAAHLPPSSQKIELKGLAPTCAYVFQPELLVTRVVEISIMEPDVPLPQTLLSHPSY
LKVFVPDYTRELLLELRDCVSNGSLGCPVRLTVGPVTLPSNFQKVLTCTGAPWPCRLLLPSPPWDRWLQV
TAESLVGPLGTVAFSAVAALTACRPRSVTIQPLLQSSQNQSFNASSGLLSPSPDHQDLGRSGRVDRSPFC

LTNYPVTREDMDVVSVHFQPLDRVSVRVCSDTPSVMRLRLNTGMDSGGSLTISLRANKTEMRNETVVVAC
VNAASPFLGFNTSLNCTTAFFQGYPLSLSAWSRRANLIIPYPETDNWYLSLQLMCPENAEDCEQAVVHVE
TTLYLVPCLNDCGPYGQCLLLRRHSYLYASCSCKAGWRGWSCTDNSTAQTVAQQRAATLLLTLSNLMFLA
PIAVSVRRFFLVEASVYAYTMFFSTFYHACDQPGEAVLCILSYDTLQYCDFLGSGAAIWVTILCMARLKT
VLKYVLFLLGTLVIAMSLQLDRRGMWNMLGPCLFAFVIMASMWAYRCGHRRQCYPTSWQRWAFYLLPGVS
MASVGIAIYTSMMTSDNYYYTHSIWHILLAGSAALLLPPPDQPAEPWACSQKFPCHYQICKNDREELYAV
T

>GPN1_HUMAN
MAASAAAAELQASGGPRHPVCLLVLGMAGSGKTTFVQRLTGHLHAQGTPPYVINLDPAVHEVPFPANIDI
RDTVKYKEVMKQYGLGPNGGIVTSLNLFATRFDQVMKFIEKAQNMSKYVLIDTPGQIEVFTWSASGTIIT
EALASSFPTVVIYVMDTSRSTNPVTFMSNMLYACSILYKTKLPFIVVMNKTDIIDHSFAVEWMQDFEAFQ
DALNQETTYVSNLTRSMSLVLDEFYSSLRVVGVSAVLGTGLDELFVQVTSAAEEYEREYRPEYERLKKSL
ANAESQQQREQLERLRKDMGSVALDAGTAKDSLSPVLHPSDLILTRGTLDEEDEEADSDTDDIDHRVTEE
SHEEPAFQNFMQESMAQYWKRNNK

>Q9HCN5_HUMAN
MSPSPTALFCL

>SDF2L_HUMAN
MWSAGRGGAAWPVLLGLLLALLVPGGGAAKTGAELVTCGSVLKLLNTHHRVRLHSHDIKYGSGSGQQSVT
GVEASDDANSYWRIRGGSEGGCPRGSPVRCGQAVRLTHVLTGKNLHTHHFPSPLSNNQEVSAFGEDGEGD
DLDLWTVRCSGQHWEREAAVRFQHVGTSVFLSVTGEQYGSPIRGQHEVHGMPSANTHNTWKAMEGIFIKP
SVEPSAGHDEL

>HHATL_HUMAN
MGIKTALPAAELGLYSLVLSGALAYAGRGLLEASQDGAHRKAFRESVRPGWEYIGRKMDVADFEWVMWFT
SFRNVIIFALSGHVLFAKLCTMVAPKLRSWMYAVYGALAVMGTMGPWYLLLLLGHCVGLYVASLLGQPWL
CLGLGLASLASFKMDPLISWQSGFVTGTFDLQEVLFHGGSSFTVLRCTSFALESCAHPDRHYSLADLLKY
NFYLPFFFFGPIMTFDRFHAQVSQVEPVRREGELWHIRAQAGLSVVAIMAVDIFFHFFYILTIPSDLKFA
NRLPDSALAGLAYSNLVYDWVKAAVLFGVVNTVACLDHLDPPQPPKCITALYVFAETHFDRGINDWLCKY
VYNHIGGEHSAVIPELAATVATFAITTLWLGPCDIVYLWSFLNCFGLNFELWMQKLAEWGPLARIEASLS
VQMSRRVRALFGAMNFWAIIMYNLVSLNSLKFTELVARRLLLTGFPQTTLSILFVTYCGVQLVKERERTL
ALEEEQKQDKEKPE

>GALT9_HUMAN
MAVARKIRTLLTVNILVFVGIVLFSVYCRLQGRSQELVRIVSGDRRVRSRHAKVGTLGDREAILQRLDHL
EEVVYNQLNGLAKPIGLVEGPGGLGQGGLAATLRDDGQEAEGKYEEYGYNAQLSDRISLDRSIPDYRPRK
CRQMSYAQDLPQVSVVFIFVNEALSVILRSVHSVVNHTPSQLLKEVILVDDNSDNVELKFNLDQYVNKRY
PGLVKIVRNSRREGLIRARLQGWKAATAPVVGFFDAHVEFNTGWAEPALSRIREDRRRIVLPAIDNIKYS
TFEVQQYANAAHGYNWGLRCMYIIPPQDWLDRGDESAPIRTPAMIGCSFVVDREYFGDIGLLDPGMEVYG
GENVELGMRVWQCGGSMEVLPCSRVAHIERTRKPYNNDIDYYAKRNALRAAEVWMDDFKSHVYMAWNIPM
SNPGVDFGDVSERLALRQRLKCRSFKWYLENVYPEMRVYNNTLTYGEVRNSKASAYCLDQGAEDGDRAIL
YPCHGMSSQLVRYSADGLLQLGPLGSTAFLPDSKCLVDDGTGRMPTLKKCEDVARPTQRLWDFTQSGPIV

SRATGRCLEVEMSKDANFGLRLVVQRCSGQKWMIRNWIKHARH

>PDE11_HUMAN
MAASRLDFGEVETFLDRHPELFEDYLMRKGKQEMVEKWLQRHSQGQGALGPRPSLAGTSSLAHSTCRGGS
SVGGGTGPNGSAHSQPLPGGGDCGGVPLSPSWAGGSRGDGNLQRRASQKELRKSFARSKAIHVNRTYDEQ
VTSRAQEPLSSVRRRALLRKASSLPPTTAHILSALLESRVNLPRYPPTAIDYKCHLKKHNERQFFLELVK
DISNDLDLTSLSYKILIFVCLMVDADRCSLFLVEGAAAGKKTLVSKFFDVHAGTPLLPCSSTENSNEVQV
PWGKGIIGYVGEHGETVNIPDAYQDRRFNDEIDKLTGYKTKSLLCMPIRSSDGEIIGVAQAINKIPEGAP
FTEDDEKVMQMYLPFCGIAISNAQLFAASRKEYERSRALLEVVNDLFEEQTDLEKIVKKIMHRAQTLLKC
ERCSVLLLEDIESPVVKFTKSFELMSPKCSADAENSFKESMEKSSYSDWLINNSIAELVASTGLPVNISD
AYQDPRFDAEADQISGFHIRSVLCVPIWNSNHQIIGVAQVLNRLDGKPFDDADQRLFEAFVIFCGLGINN
TIMYDQVKKSWAKQSVALDVLSYHATCSKAEVDKFKAANIPLVSELAIDDIHFDDFSLDVDAMITAALRM
FMELGMVQKFKIDYETLCRWLLTVRKNYRMVLYHNWRHAFNVCQLMFAMLTTAGFQDILTEVEILAVIVG
CLCHDLDHRGTNNAFQAKSGSALAQLYGTSATLEHHHFNHAVMILQSEGHNIFANLSSKEYSDLMQLLKQ
SILATDLTLYFERRTEFFELVSKGEYDWNIKNHRDIFRSMLMTACDLGAVTKPWEISRQVAELVTSEFFE
QGDRERLELKLTPSAIFDRNRKDELPRLQLEWIDSICMPLYQALVKVNVKLKPMLDSVATNRSKWEELHQ
KRLLASTASSSPASVMVAKEDRN

>E41LA_HUMAN
MGCFCAVPEEFYCEVLLLDESKLTLTTQQQGIKKSTKGSVVLDHVFHHVNLVEIDYFGLRYCDRSHQTYW
LDPAKTLAEHKELINTGPPYTLYFGIKFYAEDPCKLKEEITRYQFFLQVKQDVLQGRLPCPVNTAAQLGA
YAIQSELGDYDPYKHTAGYVSEYRFVPDQKEELEEAIERIHKTLMGQIPSEAELNYLRTAKSLEMYGVDL
HPVYGENKSEYFLGLTPVGVVVYKNKKQVGKYFWPRITKVHFKETQFELRVLGKDCNETSFFFEARSKTA
CKHLWKCSVEHHTFFRMPENESNSLSRKLSKFGSIRYKHRYSGRTALQMSRDLSIQLPRPDQNVTRSRSK
TYPKRIAQTQPAESNSISRITANMENGENEGTIKIIAPSPVKSFKKAKNENSPDTQRSKSHAPWEENGPQ
SGLYNSPSDRTKSPKFPYTRRRNPSCGSDNDSVQPVRRRKAHNSGEDSDLKQRRRSRSRCNTSSGSESEN
SNREYRKKRNRIRQENDMVDSAPQWEAVLRRQKEKNQADPNNRRSRHRSRSRSPDIQAKEELWKHIQKEL
VDPSGLSEEQLKEIPYTKIETQGDPIRIRHSHSPRSYRQYRRSQCSDGERSVLSEVNSKTDLVPPLPVTR
SSDAQGSGDATVHQRRNGSKDSLMEEKPQTSTNNLAGKHTAKTIKTIQASRLKTET

>SYF1_HUMAN
MVVMARLSRPERPDLVFEEEDLPYEEEIMRNQFSVKCWLRYIEFKQGAPKPRLNQLYERALKLLPCSYKL
WYRYLKARRAQVKHRCVTDPAYEDVNNCHERAFVFMHKMPRLWLDYCQFLMDQGRVTHTRRTFDRALRAL
PITQHSRIWPLYLRFLRSHPLPETAVRGYRRFLKLSPESAEEYIEYLKSSDRLDEAAQRLATVVNDERFV
SKAGKSNYQLWHELCDLISQNPDKVQSLNVDAIIRGGLTRFTDQLGKLWCSLADYYIRSGHFEKARDVYE
EAIRTVMTVRDFTQVFDSYAQFEESMIAAKMETASELGREEEDDVDLELRLARFEQLISRRPLLLNSVLL
RQNPHHVHEWHKRVALHQGRPREIINTYTEAVQTVDPFKATGKPHTLWVAFAKFYEDNGQLDDARVILEK
ATKVNFKQVDDLASVWCQCGELELRHENYDEALRLLRKATALPARRAEYFDGSEPVQNRVYKSLKVWSML
ADLEESLGTFQSTKAVYDRILDLRIATPQIVINYAMFLEEHKYFEESFKAYERGISLFKWPNVSDIWSTY
LTKFIARYGGRKLERARDLFEQALDGCPPKYAKTLYLLYAQLEEEWGLARHAMAVYERATRAVEPAQQYD
MFNIYIKRAAEIYGVTHTRGIYQKAIEVLSDEHAREMCLRFADMECKLGEIDRARAIYSFCSQICDPRTT
GAFWQTWKDFEVRHGNEDTIKEMLRIRRSVQATYNTQVNFMASQMLKVSGSATGTVSDLAPGQSGMDDMK
LLEQRAEQLAAEAERDQPLRAQSKILFVRSDASREELAELAQQVNPEEIQLGEDEDEDEMDLEPNEVRLE
QQSVPAAVFGSLKED

>FGF22_HUMAN
MRRRLWLGLAWLLLARAPDAAGTPSASRGPRSYPHLEGDVRWRRLFSSTHFFLRVDPGGRVQGTRWRHGQ
DSILEIRSVHVGVVVIKAVSSGFYVAMNRRGRLYGSRLYTVDCRFRERIEENGHNTYASQRWRRRGQPMF
LALDRRGGPRPGGRTRRYHLSAHFLPVLVS

>CELR2_HUMAN
MRSPATGVPLPTPPPPLLLLLLLLLPPPLLGDQVGPCRSLGSRGRGSSGACAPMGWLCPSSASNLWLYTS
RCRDAGTELTGHLVPHHDGLRVWCPESEAHIPLPPAPEGCPWSCRLLGIGGHLSPQGKLTLPEEHPCLKA
PRLRCQSCKLAQAPGLRAGERSPEESLGGRRKRNVNTAPQFQPPSYQATVPENQPAGTPVASLRAIDPDE
GEAGRLEYTMDALFDSRSNQFFSLDPVTGAVTTAEELDRETKSTHVFRVTAQDHGMPRRSALATLTILVT
DTNDHDPVFEQQEYKESLRENLEVGYEVLTVRATDGDAPPNANILYRLLEGSGGSPSEVFEIDPRSGVIR
TRGPVDREEVESYQLTVEASDQGRDPGPRSTTAAVFLSVEDDNDNAPQFSEKRYVVQVREDVTPGAPVLR
VTASDRDKGSNAVVHYSIMSGNARGQFYLDAQTGALDVVSPLDYETTKEYTLRVRAQDGGRPPLSNVSGL
VTVQVLDINDNAPIFVSTPFQATVLESVPLGYLVLHVQAIDADAGDNARLEYRLAGVGHDFPFTINNGTG
WISVAAELDREEVDFYSFGVEARDHGTPALTASASVSVTVLDVNDNNPTFTQPEYTVRLNEDAAVGTSVV
TVSAVDRDAHSVITYQITSGNTRNRFSITSQSGGGLVSLALPLDYKLERQYVLAVTASDGTRQDTAQIVV
NVTDANTHRPVFQSSHYTVNVNEDRPAGTTVVLISATDEDTGENARITYFMEDSIPQFRIDADTGAVTTQ
AELDYEDQVSYTLAITARDNGIPQKSDTTYLEILVNDVNDNAPQFLRDSYQGSVYEDVPPFTSVLQISAT
DRDSGLNGRVFYTFQGGDDGDGDFIVESTSGIVRTLRRLDRENVAQYVLRAYAVDKGMPPARTPMEVTVT
VLDVNDNPPVFEQDEFDVFVEENSPIGLAVARVTATDPDEGTNAQIMYQIVEGNIPEVFQLDIFSGELTA
LVDLDYEDRPEYVLVIQATSAPLVSRATVHVRLLDRNDNPPVLGNFEILFNNYVTNRSSSFPGGAIGRVP
AHDPDISDSLTYSFERGNELSLVLLNASTGELKLSRALDNNRPLEAIMSVLVSDGVHSVTAQCALRVTII
TDEMLTHSITLRLEDMSPERFLSPLLGLFIQAVAATLATPPDHVVVFNVQRDTDAPGGHILNVSLSVGQP
PGPGGGPPFLPSEDLQERLYLNRSLLTAISAQRVLPFDDNICLREPCENYMRCVSVLRFDSSAPFIASSS
VLFRPIHPVGGLRCRCPPGFTGDYCETEVDLCYSRPCGPHGRCRSREGGYTCLCRDGYTGEHCEVSARSG
RCTPGVCKNGGTCVNLLVGGFKCDCPSGDFEKPYCQVTTRSFPAHSFITFRGLRQRFHFTLALSFATKER
DGLLLYNGRFNEKHDFVALEVIQEQVQLTFSAGESTTTVSPFVPGGVSDGQWHTVQLKYYNKPLLGQTGL
PQGPSEQKVAVVTVDGCDTGVALRFGSVLGNYSCAAQGTQGGSKKSLDLTGPLLLGGVPDLPESFPVRMR
QFVGCMRNLQVDSRHIDMADFIANNGTVPGCPAKKNVCDSNTCHNGGTCVNQWDAFSCECPLGFGGKSCA
QEMANPQHFLGSSLVAWHGLSLPISQPWYLSLMFRTRQADGVLLQAITRGRSTITLQLREGHVMLSVEGT
GLQASSLRLEPGRANDGDWHHAQLALGASGGPGHAILSFDYGQQRAEGNLGPRLHGLHLSNITVGGIPGP
AGGVARGFRGCLQGVRVSDTPEGVNSLDPSHGESINVEQGCSLPDPCDSNPCPANSYCSNDWDSYSCSCD
PGYYGDNCTNVCDLNPCEHQSVCTRKPSAPHGYTCECPPNYLGPYCETRIDQPCPRGWWGHPTCGPCNCD
VSKGFDPDCNKTSGECHCKENHYRPPGSPTCLLCDCYPTGSLSRVCDPEDGQCPCKPGVIGRQCDRCDNP
FAEVTTNGCEVNYDSCPRAIEAGIWWPRTRFGLPAAAPCPKGSFGTAVRHCDEHRGWLPPNLFNCTSITF
SELKGFAERLQRNESGLDSGRSQQLALLLRNATQHTAGYFGSDVKVAYQLATRLLAHESTQRGFGLSATQ
DVHFTENLLRVGSALLDTANKRHWELIQQTEGGTAWLLQHYEAYASALAQNMRHTYLSPFTIVTPNIVIS
VVRLDKGNFAGAKLPRYEALRGEQPPDLETTVILPESVFRETPPVVRPAGPGEAQEPEELARRQRRHPEL
SQGEAVASVIIYRTLAGLLPHNYDPDKRSLRVPKRPIINTPVVSISVHDDEELLPRALDKPVTVQFRLLE
TEERTKPICVFWNHSILVSGTGGWSARGCEVVFRNESHVSCQCNHMTSFAVLMDVSRRENGEILPLKTLT
YVALGVTLAALLLTFFFLTLLRILRSNQHGIRRNLTAALGLAQLVFLLGINQADLPFACTVIAILLHFLY
LCTFSWALLEALHLYRALTEVRDVNTGPMRFYYMLGWGVPAFITGLAVGLDPEGYGNPDFCWLSIYDTLI
WSFAGPVAFAVSMSVFLYILAARASCAAQRQGFEKKGPVSGLQPSFAVLLLLSATWLLALLSVNSDTLLF

HYLFATCNCIQGPFIFLSYVVLSKEVRKALKLACSRKPSPDPALTTKSTLTSSYNCPSPYADGRLYQPYG
DSAGSLHSTSRSGKSQPSYIPFLLREESALNPGQGPPGLGDPGSLFLEGQDQQHDPDTDSDSDLSLEDDQ
SGSYASTHSSDSEEEEEEEEEEEAAFPGEQGWDSLLGPGAERLPLHSTPKDGGPGPGKAPWPGDFGTTAKE
SSGNGAPEERLRENGDALSREGSLGPLPGSSAQPHKGILKKKCLPTISEKSSLLRLPLEQCTGSSRGSSA
SEGSRGGPPPRPPPRQSLQEQLNGVMPIAMSIKAGTVDEDSSGSEFLFFNFLH

>DPOD4_HUMAN
MGRKRLITDSYPVVKRREGPAGHSKGELAPELGEEPQPRDEEEAELELLRQFDLAWQYGPCTGITRLQRW
CRAKQMGLEPPPEVWQVLKTHPGDPRFQCSLWHLYPL

>BRMS1_HUMAN
MPVQPPSKDTEEMEAEGDSAAEMNGEEEESEEERSGSQTESEEESSEMDDEDYERRRSECVSEMLDLEKQ
FSELKEKLFRERLSQLRLRLEEVGAERAPEYTEPLGGLQRSLKIRIQVAGIYKGFCLDVIRNKYECELQG
AKQHLESEKLLLYDTLQGELQERIQRLEEDRQSLDLSSEWWDDKLHARGSSRSWDSLPPSKRKKAPLVSG
PYIVYMLQEIDILEDWTAIKKARAAVSPQKRKSDGP

>ZN304_HUMAN
MAAAVLMDRVQSCVTFEDVFVYFSREEWELLEEAQRFLYRDVMLENFALVATLGFWCEAEHEAPSEQSVS
VEGVSQVRTAESGLFQKAHPCEMCDPLLKDILHLAEHQGSHLTQKLCTRGLCRRRFSFSANFYQHQKQHN
GENCFRGDDGGASFVKSCTVHMLGRSFTCREEGMDLPDSSGLFQHQTTYNRVSPCRRTECMESFPHSSSL
RQHQGDYDGQMLFSCGDEGKAFLDTFTLLDSQMTHAEVRPFRCLPCGNVFKEKSALINHRKIHSGEISHV
CKECGKAFIHLHHLKMHQKFHTGKRHYTCSECGKAFSRKDTLVQHQRVHTGERSYDCSECGKAYSRSSHL
VQHQRIHTGERPYKCNKCGKAFSRKDTLVQHQRFHTGERPYECSECGKFFSQSSHLIEHWRIHTGARPYE
CIECGKFFSHNSSLIKHRRVHTGARSYVCSKCGKAFGCKDTLVQHQIIHTGARPYECSECGKAFSRKDTL
VQHQKIHTGERPYECGECGKFFSHSSNLIVHQRIHTGAKPYECNECGKCFSHNSSLILHQRVHTGARPYV
CSECGKAYISSSHLVQHKKVHTGARPYECSECGKFFSRNSGLILHQRVHTGEKPYVCSECGKAYSRSSHL
VRHQKAHTGERAHECNSFGGPLAASLKLV

>Q9HCX8_HUMAN
MMHVNNFPFRRHSWI

>Q9HCY4_HUMAN
LGMEAVSRVFPALAGQAPEEQGEIIKVKVKEEDHTWDQESALRRNLSYTRELSRQRFRQFCYQETPGPRE
ALSQLRELCRQWLNPEIHTKEQILELLVLEQFLTILPEELQSWVREHNPESGEEVVTLLEDLERELDEPR
QQ

>S10AE_HUMAN
MGQCRSANAEDAQEFSDVERAIETLIKNFHQYSVEGGKETLTPSELRDLVTQQLPHLMPSNCGLEEKIAN
LGSCNDSKLEFRSFWELIGEAAKSVKLERPVRGH

>ZN334_HUMAN
MKMKKFQIPVSFQDLTVNFTQEEWQQLDPAQRLLYRDVMLENYSNLVSVGYHVSKPDVIFKLEQGEEPWI
VEEFSNQNYPDIDDALEKNKEIQDKHLTQTVFFSNKTLITERENVFGKTLNLGMNSVPSRKMPYKCNPGG
NSLKTNSEVIVAKKSKENRKIPDGYSGFGKHEKSHLGMKKYRYNPMRKASNQNENLILHQNIQILKQPFD

YNKCGKTFFKRAILITQKGRQTERKPNECNECRKTFSKRSTLIVHQRIHTGEKPYVCSDCRKTFRVKTSL
TRHRRIHTGERPYECSECRKTFIDKSALIVHQKIHGGEKSYECNECGKTFFRKSALAEHFRSHTGEKPYE
CKECGNAFSKKSYLVVHQRTHRGEKPNECKECGKTFFCQSALTAHQRIHTGEKPYECSECEKTFFCQSAL
NVHRRSHTGEKPYECSQCGKFLCTKSALIAHQITHRGKKSYECNECGKFFCHKSTLTIHQRTHTGEKHGV
FNKCGRISIVKSNCSQCKRMNTKENLYECSEHGHAVSKNSHLIVHQRTIWERPYECNECGRTYCRKSALT
HHQRTHTGQRPYECNECGKTFCQKFSFVEHQRTHTGEKPYECNECGKSFCHKSAFRVHRRIHTGEKPYEC
NQCGKTYRRLWTLTEHQKIHTGEKPYECNKCEKTFRHKSNFLLHQKSHKE

>AT131_HUMAN
MAAAAAVGNAVPCGARPCGVRPDGQPKPGPQPRALLAAGPALIANGDELVAAVWPYRRLALLRRLTVLPF
AGLLYPAWLGAAAAGCWGWGSSWVQIPEAALLVLATICLAHALTVLSGHWSVHAHCALTCTPEYDPSKAT
FVKVVPTPNNGSTELVALHRNEGEDGLEVLSFEFQKIKYSYDALEKKQFLPVAFPVGNAFSYYQSNRGFQ
EDSEIRAAEKKFGSNKAEMVVPDFSELFKERATAPFFVFQVFCVGLWCLDEYWYYSVFTLSMLVAFEASL
VQQQMRNMSEIRKMGNKPHMIQVYRSRKWRPIASDEIVPGDIVSIGRSPQENLVPCDVLLLRGRCIVDEA
MLTGESVPQMKEPIEDLSPDRVLDLQADSRLHVIFGGTKVVQHIPPQKATTGLKPVDSGCVAYVLRTGFN
TSQGKLLRTILFGVKRVTANNLETFIFILFLLVFAIAAAAYVWIEGTKDPSRNRYKLFLECTLILTSVVP
PELPIELSLAVNTSLIALAKLYMYCTEPFRIPFAGKVEVCCFDKTGTLTSDSLVVRGVAGLRDGKEVTPV
SSIPVETHRALASCHSLMQLDDGTLVGDPLEKAMLTAVDWTLTKDEKVFPRSIKTQGLKIHQRFHFASAL
KRMSVLASYEKLGSTDLCYIAAVKGAPETLHSMFSQCPPDYHHIHTEISREGARVLALGYKELGHLTHQQ
AREVKREALECSLKFVGFIVVSCPLKADSKAVIREIQNASHRVVMITGDNPLTACHVAQELHFIEKAHTL
ILQPPSEKGRQCEWRSIDGSIVLPLARGSPKALALEYALCLTGDGLAHLQATDPQQLLRLIPHVQVFARV
APKQKEFVITSLKELGYVTLMCGDGTNDVGALKHADVGVALLANAPERVVERRRRPRDSPTLSNSGIRAT
SRTAKQRSGLPPSEEQPTSQRDRLSQVLRDLEDESTPIVKLGDASIAAPFTSKLSSIQCICHVIKQGRCT
LVTTLQMFKILALNALILAYSQSVLYLEGVKFSDFQATLQGLLLAGCFLFISRSKPLKTLSRERPLPNIF
NLYTILTVMLQFFVHFLSLVYLYREAQARSPEKQEQFVDLYKEFEPSLVNSTVYIMAMAMQMATFAINYK
GPPFMESLPENKPLVWSLAVSLLAIIGLLLGSSPDFNSQFGLVDIPVEFKLVIAQVLLLDFCLALLADRV
LQFFLGTPKLKVPS

>LYRM4_HUMAN
MAASSRAQVLSLYRAMLRESKRFSAYNYRTYAVRRIRDAFRENKNVKDPVEIQTLVNKAKRDLGVIRRQV
HIGQLYSTDKLIIENRDMPRT

>E4YH25_OIKDI
MSRLYTSANFIELKALKMNLRRKSADGFAIPTHFGHNWPSYDGKFQSPTHLYSKASKAIGYKVNKIYRLD
FLIRRCLSGPLTNEAVKSINNFVNQREALLETSVEDSEKSLLLYLCWIKLKNFCPLTGRLKKEEILLRRD
SFLDRFEAIEPSGLLQLIYMNHSQLVPREIMSDLVESLNPLFEESIFELLPIFNPKCGMFKLEETVDANP
VKALFMHKLKHNTGVFGDKSIQNFLKNPSIPMRYKHRHFQMISWVLTLSSDGKKSRKTDLLKKIYSPSLE
SDLSGLPLAEMVDLSNMIGLYEHEDYFTGAESLDEQTSLYYQSCTDSCGSLTKPTFAKFAENNTKVEYDG
KTIPRGEAAYKAMLNEKIEHYQRSLEKQGVGQLEIAYQTIKFISKPPDGSLRRWISKSWVRRRFVKKEKK
IIKDMCFVDSIRKKLLVIFIEDLRKYQEESFHLSFMSDFISQIYDDMEDKDSLLEDLRDIISQESECPIE
IVNRLKLAQILLAYFPDLYSKNDLQDLFTQNTAFPVYLVQADG

>SPCS_HUMAN
MNRESFAAGERLVSPAYVRQGCEARRSHEHLIRLLLEKGKCPENGWDESTLELFLHELAIMDSNNFLGNC

GVGEREGRVASALVARRHYRFIHGIGRSGDISAVQPKAAGSSLLNKITNSLVLDIIKLAGVHTVANCFVV
PMATGMSLTLCFLTLRHKRPKAKYIIWPRIDQKSCFKSMITAGFEPVVIENVLEGDELRTDLKAVEAKVQ
ELGPDCILCIHSTTSCFAPRVPDRLEELAVICANYDIPHIVNNAYGVQSSKCMHLIQQGARVGRIDAFVQ
SLDKNFMVPVGGAIIAGFNDSFIQEISKMYPGRASASPSLDVLITLLSLGSNGYKKLLKERKEMFSYLSN
QIKKLSEAYNERLLHTPHNPISLAMTLKTLDEHRDKAVTQLGSMLFTRQVSGARVVPLGSMQTVSGYTFR
GFMSHTNNYPCAYLNAASAIGMKMQDVDLFIKRLDRCLKAVRKERSKESDDNYDKTEDVDIEEMALKLDN
VLLDTYQDASS

>CHM1A_HUMAN
MDDTLFQLKFTAKQLEKLAKKAEKDSKAEQAKVKKALLQKNVECARVYAENAIRKKNEGVNWLRMASRVD
AVASKVQTAVTMKGVTKNMAQVTKALDKALSTMDLQKVSSVMDRFEQQVQNLDVHTSVMEDSMSSATTLT
TPQEQVDSLIMQIAEENGLEVLDQLSQLPEGASAVGESSVRSQEDQLSRRLAALRN

>PTPRH_HUMAN
MAGAGGGLGVWGNLVLLGLCSWTGARAPAPNPGRNLTVETQTTSSISLSWEVPDGLDSQNSNYWVQCTGD
GGTTETRNTTATNVTVDGLGPGSLYTCSVWVEKDGVNSSVGTVTTATAPNPVRNLRVEAQTNSSIALTWE
VPDGPDPQNSTYGVEYTGDGGRAGTRSTAHTNITVDGLEPGCLYAFSMWVGKNGINSSRETRNATTAHNP
VRNLRVEAQTTSSISLSWEVPDGTDPQNSTYCVQCTGDGGRTETRNTTDTRVTVDGLGPGSLYTCSVWVE
KDGVNSSVEIVTSATAPNPVRNLTVEAQTNSSIALTWEVPDGPDPQNSTYGVEYTGDGGRAGTRSTAHTN
ITVDRLEPGCLYVFSVWVGKNGINSSRETRNATTAPNPVRNLHMETQTNSSIALCWEVPDGPYPQDYTYW
VEYTGDGGGTETRNTTNTSVTAERLEPGTLYTFSVWAEKNGARGSRQNVSISTVPNAVTSLSKQDWTNST
IALRWTAPQGPGQSSYSYWVSWVREGMTDPRTQSTSGTDITLKELEAGSLYHLTVWAERNEVRGYNSTLT
AATAPNEVTDLQNETQTKNSVMLWWKAPGDPHSQLYVYWVQWASKGHPRRGQDPQANWVNQTSRTNETWY
KVEALEPGTLYNFTVWAERNDVASSTQSLCASTYPDTVTITSCVSTSAGYGVNLIWSCPQGGYEAFELEV
GGQRGSQDRSSCGEAVSVLGLGPARSYPATITTIWDGMKVVSHSVVCHTESAGVIAGAFVGILLFLILVG
LLIFFLKRRNKKKQQKPELRDLVFSSPGDIPAEDFADHVRKNERDSNCGFADKYQQLSLVGHSQSQMVAS
ASENNAKNRYRNVLPYDWSRVPLKPIHEEPGSDYINASFMPGLWSPQEFIATQGPLPQTVGDFWRLVWEQ
QSHTLVMLTNCMEAGRVKCEHYWPLDSQPCTHGHLRVTLVGEEVMENWTVRELLLLQVEEQKTLSVRQFH
YQAWPDHGVPSSPDTLLAFWRMLRQWLDQTMEGGPPIVHCSAGVGRTGTLIALDVLLRQLQSEGLLGPFS
FVRKMRESRPLMVQTEAQYVFLHQCILRFLQQSAQAPAEKEVPYEDVENLIYENVAAIQAHKLEV

>TM9S3_HUMAN
MRPLPGALGVAAAAALWLLLLLLPRTRADEHEHTYQDKEEVVLWMNTVGPYHNRQETYKYFSLPFCVGSK
KSISHYHETLGEALQGVELEFSGLDIKFKDDVMPATYCEIDLDKEKRDAFVYAIKNHYWYQMYIDDLPIW
GIVGEADENGEDYYLWTYKKLEIGFNGNRIVDVNLTSEGKVKLVPNTKIQMSYSVKWKKSDVKFEDRFDK
YLDPSFFQHRIHWFSIFNSFMMVIFLVGLVSMILMRTLRKDYARYSKEEEMDDMDRDLGDEYGWKQVHGD
VFRPSSHPLIFSSLIGSGCQIFAVSLIVIIVAMIEDLYTERGSMLSTAIFVYAATSPVNGYFGGSLYARQ
GGRRWIKQMFIGAFLIPAMVCGTAFFINFIAIYYHASRAIPFGTMVAVCCICFFVILPLNLVGTILGRNL
SGQPNFPCRVNAVPRPIPEKKWFMEPAVIVCLGGILPFGSIFIEMYFIFTSFWAYKIYYVYGFMMLVLVI
LCIVTVCVTIVCTYFLLNAEDYRWQWTSFLSAASTAIYVVMYSFYYYFFKTKMYGLFQTSFYFGYMAVFS
TALGIMCGAIGYMGTSAFVRKIYTNVKID

>MOG1_HUMAN
MEPTRDCPLFGGAFSAILPMGAIDVSDLRPVPDNQEVFCHPVTDQSLIVELLELQAHVRGEAAARYHFED

VGGVQGARAVHVESVQPLSLENLALRGRCQEAWVLSGKQQIAKENQQVAKDVTLHQALLRLPQYQTDLLL
TFNQPPPDNRSSLGPENLSPAPWSLGDFEQLVTSLTLHDPNIFGPQ

>E4YH36_OIKDI
MTSILEVLIQQRIFAQRGLPELTHCLLIRAELSHISSPIHFQETGFISSLDLRSFFFRPLSVQSRVVGMK
WKLAFLTLGVDSRSHEKGSRWALKQQRIELKYEANVNHNMQSVIFIKFRAFLDGEKILKSPQKIFKQTQE
FLGLPILIQDQHFFINKTSGYYCVHNPTTQEPHCMTTKSKGRTRKVDQEVLALSEKQQNSLEEFFLPFNV
QLCEIIGPVLNFLKNLC

>E4YH48_OIKDI
MQREPTALPPTTFTICFTGTTHVRLDGIESRLKEALDSVVPELNLTRVGPPTITVQSRDEDFIPLSDYVP
PPNTPIQESDQEPALSPTPPSECAQFNPADFPLPSDTDDLEQQFPENQEPEKVPIARDHFNHTRDELDEM
FTSFDSHDPDAQNDKKGNKQCTDLTEKAMRFSVYDKLSALTTDNDKNSLGNLASPEQNHSSIRRSAHSSS
DDNEEMTFVYEGSSTVQSEGKPKTSCLKKQKNSWSSDDDSRETRVEKKGNSLDIGDAKETKRQPCLKKSN
SRGEDLSDQSRKSSLSSRGESNKAHGSSSTTTSRANYKSSFSDRNNNDRQRSSTGYNPGSHRGSSVFHRN
GNRDDRFRRGSRESDRDDFRRRDQDRAGSGREFRPREKRNTSSEREVSVYNDANRFGEKDYASRRNEDRS
FSRFRDGGFQKYDARPSRDEHISNHNRPHFADREHRSYGGQSYRREFRGEHCTRSGRGAFGQTPTDGHRR
RRDRDSERNSDER

>Q9HD52_HUMAN
MISQGSLPGQGPNTIAKGLIVNQTSKGPRQQRAWKEMVQIKVGEGSKIRKFKNTSYGIMLTP

>GAGD2_HUMAN
MRCHAHGPSCLVTAITREEGGPRSGGAQAKLGCCWGYPSPRSTWNPDRRFWTPQTGPGEGRHERHTQTQN
HTASPRSPVMESPKKKNQQLKVGILHLGSRQKKIRIQLRSQCATWKVICKSCISQTPGINLDLGSGVKVK
IIPKEEHCKMPEAGEEQPQV

>MYO10_HUMAN
MDNFFTEGTRVWLRENGQHFPSTVNSCAEGIVVFRTDYGQVFTYKQSTITHQKVTAMHPTNEEGVDDMAS
LTELHGGSIMYNLFQRYKRNQIYTYIGSILASVNPYQPIAGLYEPATMEQYSRRHLGELPPHIFAIANEC
YRCLWKRHDNQCILISGESGAGKTESTKLILKFLSVISQQSLELSLKEKTSCVERAILESSPIMEAFGNA
KTVYNNNSSRFGKFVQLNICQKGNIQGGRIVDYLLEKNRVVRQNPGERNYHIFYALLAGLEHEEREEFYL
STPENYHYLNQSGCVEDKTISDQESFREVITAMDVMQFSKEEVREVSRLLAGILHLGNIEFITAGGAQVS
FKTALGRSAELLGLDPTQLTDALTQRSMFLRGEEILTPLNVQQAVDSRDSLAMALYACCFEWVIKKINSR
IKGNEDFKSIGILDIFGFENFEVNHFEQFNINYANEKLQEYFNKHIFSLEQLEYSREGLVWEDIDWIDNG
ECLDLIEKKLGLLALINEESHFPQATDSTLLEKLHSQHANNHFYVKPRVAVNNFGVKHYAGEVQYDVRGI
LEKNRDTFRDDLLNLLRESRFDFIYDLFEHVSSRNNQDTLKCGSKHRRPTVSSQFKDSLHSLMATLSSSN
PFFVRCIKPNMQKMPDQFDQAVVLNQLRYSGMLETVRIRKAGYAVRRPFQDFYKRYKVLMRNLALPEDVR
GKCTSLLQLYDASNSEWQLGKTKVFLRESLEQKLEKRREEEVSHAAMVIRAHVLGFLARKQYRKVLYCVV
IIQKNYRAFLLRRRFLHLKKAAIVFQKQLRGQIARRVYRQLLAEKREQEEKKKQEEEEKKKREEEERERE
RERREAELRAQQEEETRKQQELEALQKSQKEAELTRELEKQKENKQVEEILRLEKEIEDLQRMKEQQELS
LTEASLQKLQERRDQELRRLEEEACRAAQEFLESLNFDEIDECVRNIERSLSVGSEFSSELAESACEEKP
NFNFSQPYPEEEVDEGFEADDDAFKDSPNPSEHGHSDQRTSGIRTSDDSSEEDPYMNDTVVPTSPSADST
VLLAPSVQDSGSLHNSSSGESTYCMPQNAGDLPSPDGDYDYDQDDYEDGAITSGSSVTFSNSYGSQWSPD

YRCSVGTYNSSGAYRFSSEGAQSSFEDSEEDFDSRFDTDDELSYRRDSVYSCVTLPYFHSFLYMKGGLMN
SWKRRWCVLKDETFLWFRSKQEALKQGWLHKKGGGSSTLSRRNWKKRWFVLRQSKLMYFENDSEEKLKGT
VEVRTAKEIIDNTTKENGIDIIMADRTFHLIAESPEDASQWFSVLSQVHASTDQEIQEMHDEQANPQNAV
GTLDVGLIDSVCASDSPDRPNSFVIITANRVLHCNADTPEEMHHWITLLQRSKGDTRVEGQEFIVRGWLH
KEVKNSPKMSSLKLKKRWFVLTHNSLDYYKSSEKNALKLGTLVLNSLCSVVPPDEKIFKETGYWNVTVYG
RKHCYRLYTKLLNEATRWSSAIQNVTDTKAPIDTPTQQLIQDIKENCLNSDVVEQIYKRNPILRYTHHPL
HSPLLPLPYGDINLNLLKDKGYTTLQDEAIKIFNSLQQLESMSDPIPIIQGILQTGHDLRPLRDELYCQL
IKQTNKVPHPGSVGNLYSWQILTCLSCTFLPSRGILKYLKFHLKRIREQFPGSEMEKYALFTYESLKKTK
CREFVPSRDEIEALIHRQEMTSTVYCHGGGSCKITINSHTTAGEVVEKLIRGLAMEDSRNMFALFEYNGH
VDKAIESRTVVADVLAKFEKLAATSEVGDLPWKFYFKLYCFLDTDNVPKDSVEFAFMFEQAHEAVIHGHH
PAPEENLQVLAALRLQYLQGDYTLHAAIPPLEEVYSLQRLKARISQSTKTFTPCERLEKRRTSFLEGTLR
RSFRTGSVVRQKVEEEQMLDMWIKEEVSSARASIIDKWRKFQGMNQEQAMAKYMALIKEWPGYGSTLFDV
ECKEGGFPQELWLGVSADAVSVYKRGEGRPLEVFQYEHILSFGAPLANTYKIVVDERELLFETSEVVDVA
KLMKAYISMIVKKRYSTTRSASSQGSSR

>Q9HD86_HUMAN
MDGNGGHYLSEISQEHKHAHSHSYVEALKKKKKAGCGGSRLQSQHPGRLRRVDHLRSGVQDQPDQHGETL
SPPKTQN

>CF050_HUMAN
MANTQLDHLHYTTEFTRNDLLIICKKFNLMLMDEDIISLLAIFIKMCLWLWKQFLKRGSKCSETSELLEK
VKLQLAFTAYKYVDICFPEQMAYSRYIRWYIH

>RETN_HUMAN
MKALCLLLLPVLGLLVSSKTLCSMEEAINERIQEVAGSLIFRAISSIGLECQSVTSRGDLATCPRGFAVT
GCTCGSACGSWDVRAETTCHCQCAGMDWTGARCCRVQP

>Q9HDB3_HUMAN
MNSRPRRRDIDRLEHLHLTEFGLKLGQKIFLLNKRDFILFLSLF

>Q9HDC8_HUMAN
LGAGQASLFGNNQPKIGGPLGTGAFGAPGFNTTTATLGFGAPQAPV

>HRSL1_HUMAN
MAFNDCFSLNYPGNPCPGDLIEVFRPGYQHWALYLGDGYVINIAPVDGIPASFTSAKSVFSSKALVKMQL
LKDVVGNDTYRINNKYDETYPPLPVEEIIKRSEFVIGQEVAYNLLVNNCEHFVTLLRYGEGVSEQANRAI
STVEFVTAAVGVFSFLGLFPKGQRAKYY

>RHO1_ASHGO
MSQQMHNPSIRRKLVIVGDGACGKTCLLIVFAKGKFPQVYVPTVFDNYVADVEVDGRRVELALWDTAGQE
DYDRLRPLSYPDSNVVLICYSIDLPDSLENVMEKWISEVLYFCQGVPIILVGCKADLRNDPQVIEQLRQQ
GQQPVSQAQAQEVADQIGAVEYIECSAKTGFGVREVFEAATRASLMGKQGKSKAKSDKKKKKKCVVL

>PP2A1_EMENI

MDNNMEIDAARSPEPHHLSPTTDPGSIPTLDGWIESLMTCKQLAEEDVRRLCDRAREVLQEESNVQPVKC
PVTVCGDIHGQFHDLMELFRIGGPNPDTNYLFMGDYVDRGYYSVETVTLLVCLKIRYPQRITILRGNHES
RQITQVYGFYDECLRKYGNANVWKYFTDLFDYLPLTALIENQIFCLHGGLSPSIDTLDNIRSLDRIQEVP
HEGPMCDLLWSDPDDRCGWGISPRGAGYTFGQDISEAFNHNNGLTLVARAHQLVMEGYNWSQDRNVVTIF
SAPNYCYRCGNQAAIMEIDEHLKYTFLQFDPCPRAGEPMVSRRTPDYFL

>PEX6_PENCH
MDFEQYGQSSQQPRQRRRRAGKRRLNNKTPIAARLALDPQLRGKVGILSEDLANDLFQQQALQDVTTSDD
GVLYVAIAPHTPTYTSVEDQAWTILPVRIQPTERSPVAMSHSTVLFPESADSLQPFLQALGKVDSSRNSL
QAHRSVEIRILDVAPIHLDTIFVTVERHLLRNHDDVQTKFGGGFTNAQGPNGLWGKTGKSVEAKKYSKRA
AADAEQRLTAAVREALGAQRIVHTGDVLPLPLPSHPITYAPPPPARISFCEPVSQGLLMSTTKIVLVQAR
PQGIRAQQTMPSRSALLKQVAEDEADDTSNEQFYSAAEDKPGESGTEMEVTSAAEESETEGSAGSMSDSS
DDSLEDMISLSAPELPQPPSGVMSSLTSATPRAGGRRSDGIHTPGSVASNFTSATMRPGRGGGKTFKVEG
LLQQVPNEVLHPRPRDDEDVDSFVFVDISTLAKIGCFSGDWVRIEAAEEPQLNMFASLKFGSFNDSPEDS
GDWRPVKIFGLSGLPSSKPRYAINHSGERRPSISQRPPTRLTPSVFVPPLLLGNIENPKYLRISPMTFAT
PNGSSKPGILQHMKNTAAKNPPLAKEVTLLKVSTPLSMDRVLQPALFAGLKQYFESRRRILKSGDLVGIS
VDEGLGRAVFSGTGSGDSASQEEDITIRLGQGANATNAGTRKIGVAWFRVGQVAPTTVEELEETGEDQWG
GVAVLDPATTRMVQAGSDVSRVPGVLGNGWEYWLGVKTIPKTVHDAPTPHGIVADPPQSVIPPLQQRIRD
LMSAATSPRAIQLGMKPVFILLRSQQRHIGKATVATRACSDIGIHTFPIDAYDILTEGGANGGDVKTEAY
LKARAERAFHCGANCTALLIRHIEVLTADRIVTAMSDILNDARVVIATTTDVETIPEGIRSLITHEFEMG
APEEKEREGILQNAVTERGIRLSADVDLGSIALKTAALVAGDLVDVVERAAGARTARLESLAEASKKISG
SEVFVRDVLLAGGDGARGVTKADFDAAVEAARKNFADSIGAPKIPNVGWDDVGGLTNVKDALVETIQLPL
ERPELFAKGMKKRSGILFYGPPGTGKTLLAKAIATEFSLNFFSVKGPELLNMYIGESEANVRRVFQRARD
ARPCVVFFDELDSVAPKRGNQGDSGGVMDRIVSQLLAELDGMNGGEENSGGVFVIGATNRPDLLDTALLR
PGRFDKMLYLGVSDTHRKQATILEALTRKFALHPDVSLDRVAEQLPLTYTGADLYALCSDAMLKAITRKA
TAVDEKINALPNGPVSTAWFFDHLATKEDVNVMVTEEDFLSAQGELVPSVSAKELEHFERIRQTFEAVDK
SKQDPAAAAPQTIAEAMEAFSLGGSAIPEEAPTINGDSLTPGGIHGRIKGLNRWPGNPVRSTSGQSTTSS
KGKGKSVSKKGKSRTGAESDGSVDGDDEDMADANSKEDEDEDDYVVRTDHLRNPMEEVE

>RL14_THEAC
MKGIAGRQTRGLPLGANITCADNTGARSISLIDVKAWHGKARRIPAAGVGDMFMASVKKGTPEMRSKVVY
AVVIRQKRPYRRPDGTMVQFEDNAAVLVTPDGEVRGSEIKGPVAREAAERWPRIAAIASIIV

>LGUL_PSEAE
MSFNTEVQPGICMEPDAITQEYVFNHTMLRVKDPKRSLDFYSRVLGMRLLRRLDFEEGRFSLYFLAMTRG
EEVPDAVDERQRYTFGRQSVLELTHNWGSESDDSQYHNGNQDPRGFGHICFSVPDLVAACERFETLGVNF
VKPLDRGMKNVAFISDPDGYWVEIVQASLNGEMGRG

>ETFD_PSEAE
MEREYMEFDVVIVGAGPAGLSAACRLKQKAAEAGQEISVCVVEKGSEVGAHILSGAVFEPRALNELFPDW
KELGAPLNTPVTGDDIYVLKSAESATKVPNFFVPKTMHNEGNYIISLGNLCRWLAQQAEGLGVEIYPGFA
AQEALIDENGVVRGIVTGDLGVDREGNPKEGYYTPGMELRAKYTLFAEGCRGHIGKQLIKKYNLDSEADA
QHYGIGIKEIWDIDPSKHKPGLVVHTAGWPLNDENTGGSFLYHLENNQVFVGLIIDLSYSNPHLSPFDEF
QRYKHHPVVKQYLEGGKRVAYGARAICKGGLNSLPKMVFPGGALIGCDLGTLNFAKIKGSHTAMKSGMLA

ADAIAEALAAGREGGDELSSYVDAFKASWLYDELFRSRNFGAAIHKFGAIGGGAFNFIDQNIFGGKIPVT
LHDDKPDYACLKKASEAPKIDYPKPDGKLSFDKLSSVFLSNTNHEEDQPIHLKLADASIPIEKNLPLYDE
PAQRYCPAGVYEVVANDDGSKRFQINAQNCVHCKTCDIKDPAQNITWVAPEGTGGPNYPNM

>Y2418_PSEAE
MKKVLGIYGNANRHWVGDGFPVRSLFSYNTLGQHISPFLLLDYAGPADFPPAQQRRGVGQHPHRGFETVT
IVYQGEVEHHDSTGAGGRIGPGDVQWMTAASGILHEEYHSERFRSTGGTLEMVQLWVNLPSSDKMNPPRY
QTLLDADIPRVGLPDRAGELRVIAGRYGRHQGPALTHSPLAVWDVQLKAGKHLALDLPKGHTCAVVVLRG
TLAVGDEIVREAQVALLDRDDPRLELEANNDVQLLVLSGEPLDEPIIGYGPFVMSSREEIDQAIEDFENG
RFIRAH

>ACON1_PSEAE
MPALDSLKTLRSLAVDGKTYHYYSLPEAARTLGDLGKLPMSLKVLLENLLRWEDGSTVTGDDLKALAGWL
RERRSDREIQYRPARVLMQDFTGVPAVVDLAAMRAAMAKAGGDPQKINPLSPVDLVIDHSVMVDKFASES
AFEQNVEIEMQRNGERYAFLRWGQNAFDNFSVVPPGTGICHQVNLEYLGRTVWTKDEDGRTYAFPDTLVG
TDSHTTMINGLGVLGWGVGGIEAEAAMLGQPVSMLIPEVIGFKLTGKLREGITATDLVLTVTQMLRKKGV
VGKFVEFYGDGLADLPLADRATIANMAPEYGATCGFFPVDEITLGYLRLSGRPESTVKLVEAYSKEQGLW
REKGHEPVFTDTLHLDMGEVEASLAGPKRPQDRVALQNVASAFNEFLGLQLHPSSTEEGRLLSEGGGGTA
VGANAAFGEIDYQHDGQTHRLKNGAVVIAAITSCTNTSNPSVMMAAGLLAKKAVEKGLQRKPWVKSSLAP
GSKVVTDYFKAAGLTRYLDELGFDLVGYGCTTCIGNSGPLLEPIEKAIQQADLTVASVLSGNRNFEGRVH
PLVKTNWLASPPLVVAYALAGSVRINLSEEPLGTGKDGQPVYLKDIWPSQKEIAEAIQKVDTEMFHKEYA
EVFAGDEKWQAIQVPQSDTYEWQADSTYIQHPPFFEHIAEAPPAIADVEQARVLAVLGDSVTTDHISPAG
NIKADSPAGRYLREHGVEPKDFNSYGSRRGNHEVMMRGTFANIRIKNEMLGGEEGGNTLYVPSGEKLAIY
DAAMRYQEDGTPLVIVAGKEYGTGSSRDWAAKGTNLLGVKAVIAESFERIHRSNLVGMGVLPLQFENGQD
RKSLKLTGKEVLNIRGLGGELKPHMPLSVEVTREDGSQDSFKVLCRIDTLNEVEYFKAGGILHYVLRSML

>COBP_PSEAE
MRDLILGGARSGKSRLAERLAAESGLAVSYIATAQAGDGEMGRRIAEHRARRPAHWRTLEEPLALAATLR
SEAEAGRCLLVDCLTLWLTNLLLCDDPQRLDGEREALLECLGELPGRIILVSNETGLGVVPLGELSRRYV
DEAGWLHQAIAERCERVTFTVAGLPMPLKGEPL

>PR2_DROME
MEYPQIDLYEFLTESELQQYYNAVKNELKITNAAQFKYAADEDLRFIGLSRPEIRRLRKFYEKHFPHSYL
SKIKRLLQAPGTMVKREEAPGGGSQVALDGSSASACSSLAAKNGASSPSKVPNNKHIIPADSISVNKQLG
TGEFGIVQQGVWSNGNERIQVAIKCLCRERMQSNPMEFLKEAAIMHSIEHENIVRLYGVVLATDSLMLVT
ELAHLRSLLECLKDSGLRVSFLTIPTLCEFALQICNGMRYLEQKRLIHRDLAARNILVFSKDKVKISDFG
LSRALGVGKDYYKTNFNVNLKLPIAWCAPECINYLRFTNASDVWAFGVCLWEMFSYGFQPWAALTGLQIL
EAIDAPNYQRLEQPDCCPSEYYTLMMKCWQDDAAKRPRFGEIYDQLPDMKPEQLKAVVNCTEPKKDHLLY
RQGDIISVLDRNTGTPFWKGVLSTGKTGYFNPSNTVAFLEGLPSSTRDSFSRVSDHRISKRKLRTEMISK
PQNDFKHTGHVGIDGATFGDIAFLGSSQNYNHVPKQIVTPYKPSEDIEQTPLLLPPTPTSPDSLQTASGY
FPEGANSGGAMGTSMNPTFIPSAEHTPKLIATNGQSSFDFASGSTNPFFPNRGDDELEFGLHNYGADGKS
VHSETGWRPTSRSIVDDPHEYHEISDDEIAADKLDFGPSLLDEINSMFGSISAATGSHPKSPGFDHVNNK
NEITEMSAKLGQKSGDTNGNKHGHGLLPTLSKKKSSGTVKPISVKDEKILNHAIEIANEISARSMIDLVS
DQTPVIHSPKRKFSFRFPHLSNNGSGDKSGGLGTSGSAHTPTHGNASPFPKKKNFTEELQSIPDIQSLIG

KEGLEAYNSLIERKALLDIGPSPAATLLRHLDTDEFDLQSLHQSQRPMTLPTRGATQRVRKAELAAGLSR
HNDENSNSLEACESPSYMTHGSYKFPEAQPTEQLPEPESPNPIPLPPREGKKQVKTSTKRHVRKYPLIIP
ANGLQRTLSKLADFGDEAAKSPEISTSSQPQPGRAIEVVAAVRPSGMRRPSRPSEREYENMPTVGKESAH
TYQNLDKLTPADAAGLTDTASLQFESIMEADTSKEGILQSPDVTDGFYNFSIQKEHYNKGKDAEFEATQI
SGLYVNDDELRNLDIESSRRTATPCSSCSALESEHSQPDALPSTESVSEVSRFSSVDNELAGNALFKKVR
ASVNMAMNRKSVAETSLTSNQPGGASAKPQTEAEYFAATAARLADSNSVSCEDLLEFSDKKPKGCERGVD
SDEVRIMVKVLGKDSTPNRCLGALEFINWDVHKSIKLIKLQNLVSEANLSLEASFEALQQHEWDLHTTAH
KLNGLKL

>Q9I7H5_DROME
MLDRHCLYIGIFQLIIWVGVANGEFYSSVDSMQDLAQVEEELLNATRSYVESQQKQLDFYRRYVEQIKRE
HEWATSQLKLDDYLGHPLHAFRLIKRLVRDWDSLIFEPILANNAREEFRAFVEVLSRDLGYPDQSELQGA
IKGLARLQKVYNLATSDLADGIIGGLNYGSDLRWRECYEIGVQLFDLGEYQRSLEWLQVAFILLRNSPRE
EKDADHYLSDIREYASMANFELGNPKKAARLLSQILESQPTHSAQQTQKYLESRVPGKNVQETKPSWFSN
YTRLCQGRRLPEERSGDPLRCYLDGKRHAYFTLAPLQVEPVHLDPDINVYHGMLSSKQILSIFEEADKEE
MVRSAVAGSGGEGTVRDLRVSQQTWLDYKSPVMNSVGRIIQFVSGFDMAGAEHMQVANYGVGGQYEPHPD
YFEVNLPKNFEGDRISTSMFYLSDVEQGGYTVFTKLNVFLPPVKGALVMWHNLHRSLHVDARTLHAGCPV
IVGSKRIGNIWMHSGYQEFRRPCNLTSDSYKSLAYRD

>Q9I7H8_DROME
MPYEKFNKKRRQWEDSGVLELIKLWKVCAYELRTIKRNGHLYVAMAKQLTSLGVPVTALEVHFKVNNLTQ
RYRQEQKTFETTGIISTWKFYSQVDDVFKSLAAHTGYKDKRMTSASNTSSLPSTSESPVWKNPMSQQEFN
SNNTEGFYKTEYGMHRHFMDHSQPPPNAGSVDNFMASSAAVAAVAAAASARLADNNMDQQMNTAAGGSGG
SGGGGGNTNGGVANYQKIKKSHEDYDKFVDIVKNIVDTHKSTPDKVDTFGDFIKSYMKRWPERLQDEAIN
HITNYVIVKNMEHSMVSSHNPEGVNNRQ

>CCNB3_DROME
MAPTKATTRAAITSGHHQLQQAVNPILGALGAATRKGLTRRAAATGNIDPNVENMQTRAKRKADHSPIKN
DKIKRSALGNLTNNVKIMTLHPAQDEEQSGVGKKPTAQQLQALMDAKKQENLSVNVFGASKMTTRASSKV
EDSVENCHKVLDKLEEALARPKPRPKAVPAAKKTVLGEVQLPAMPNPMQIPVLLPPTHNLAAPQVAAVKP
VRRISNDFNKTEDSLYMSALEDVSSCDSMRLSGNFEAARRRSAKLQQKTEQQPQPLLLTLPETAPSQVVP
IPPVPEEVEDFDRKNWDDPFQVSHYAMDIFNYLKVREAEFPIADYMPRQIHLTTWMRTLLVDWMVEVQET
FELNHETLYLAVKIVDLYLCREVINKEKLQLLGAAAFFIACKYDERQPPLIEDFLYICDGAYNHDELVRM
ERETLRVIKYDLGIPLSYRFLRRYARCAKVPMPTLTLARYILELSLMDYANISFSDSQMASAALFMALRM
HGGPGQLDKQTWTSTLIYYTGYQLADFAEIVTALNAGLHRKPRATIKTIRNKYSHKIFHEVAKVPLLTNQ
ELFQGNLDLNESNLS

>Q9I7I1_DROME
MNILRPRGMTQAEKDVFAHRQCGLDPNGHELLHMVYVCCPELGDVLPNKQTCGQTTPVFRDRGAENAELN
EYPWMVLLLYENRLSLIRYVLTAAHCVIGGYLTQNDLVLKSVRLGESTTDCITSESRCPHLDVEVGQTTV
HQGFTSSGGTYRNDIALLRLQFPVRYTKKIQPICLLDAEFPLQDLNLQISGWDPTKSSQTLITSTVKERN
PADCLNRYPSFRSASQVCAGGQRKGDTCAGISGSPVMGIMGSGVDEFVFLAGIASYGQQYCYSAGIPGVY
TKIGHFSEWIKANLAP

>Q9I7I3_DROME
MLGRIAVLLLLVGLFGPEVRSAKVNDDQCGAFDEDQMLNMQSTFAIPTEHQWVARIVYGKGFEGKIRDNG
CLGVLVSKRTVLAPAHCFVQYNGVAEAFSVHLGVHNKSAPVGVRVCETDGYCVRPSQEIKLAEIAIHPDY
DSRTLKNSLAVLTLQRDAKIYPNVMPICMPPPSLLNETLVAQTFVVAGLRVFEDFRLKTWVNTLSRGFCQ
SKVKTLVTSSNTVCGYHKQPVAYYLGAPLVGLQKKGHVTQNYYLVGIMIDWRWENNRIMSSFLAIRNYMD
FIRQNSNSLIVRS

>Q9I7I4_DROME
MVMLPLTLVLILIMRLDGMLAALQLNEQRMRDLRNSHSEVPVYMEDYEALLPEGSTYNDLINEEFILPAS
KRTQLQILAAERARRCQPYRYGNGESMELEERNTLMKDSRTSFLPLGIPRECLGSGIELDIKPIDEEAYQ
RQKKRYQDIAPYWLEKIRIRERREAERHAEEASAEISEATAALQSFWNEEGTREGIRMTQAKTMKRYMDN
KVDPCVDFYKYACGNWERLHPIPKDKAGFDTFEMLRESLDLVLRNLLEKNTPVHSAAELRKSPVRNTLFK
LNEQGEGEGEADQAAELTAERLRRHIVSKRQLLNRVLVRYKRYTNGTKRKRLIETPRERTKEEEAAPPVV
LPKDKTKDKSDNEEQLHVPTDFLKPHQDAQLKAKNLYRSCVNSAVLAKRGLEPLHTLIRELGGWPVLESQ
WSESNFNWQVLAATLRRYNNDILIVQWVGADIKNSEENIVQFDQTGLGLPTREYFLQPSNAKYLQAYQRY
MAEVMHKMGASKADAQRVASELVAFETQLAGITAPAEQRLNVTKLYKRMTLDQLQAVVPEIKWRAYLQSL
QDREVLGTEEVVIYAVEYMSKLVTLLDETDPRTVSNYMMWRFVRHRINNVDDRFDDIKQSFYHALFGREE
SPQRWKVCIAQVNTNMGMAVGSMFVSRYFDNNSKRDTLRMTHDLQQAFRDILKTTDWLDDTTKQLAEEKV
NAMSLKIGYPDFILNPSELNSKYAGIEIYPEKYFENTLNVLLHTAKTEQAKLHERVNKTNWQTAPAIVNA
YYSRNKNQIMFPAGILQPPFYHRHFPKSLNFGGIGVVIGHELTHGFDDKGRLFDRNGNIHKWWTDSSIRG
FDERARCIIAQYSNYTVEEVGIVLNGESTQGENIADNGGLRQAFHAYQRWLKEHPSEVSDEILPGLNMTG
PQLFFLNFGQVWCGAMRPEAIRNKLNTAIHSPGRFRVIGTLSNSVDFAREFNCPLGSPMNPQKKCSVW

>Q9I7I6_DROME
MLKSIVTVIRLHLEELWLRILGYIMRRFLRSAMIVFSWFVVPYSRYTNIKVIRRKLPPIRSHLLEIPAVD
LAKLIRTRKIKSEEVVEAYIERCRQVNPLINAIVQDRFEEALEEAREIDNVIAMGINSVESMEELTPLLG
IPVTVKESIAVKGMTNQAGRVFKTPQIAKSDAPVVEQIKRSGGIILLVSNTPELCLLWETYNNVTGQTKN
PYDLKRTPGGSSGGEAALLASGASLLGLTSDIGGSSRLPAMFSGIWGHKPTPYAVSFKGHHPTSDFPKWG
DFFTIAPMTRYAKDLPLLLKCMSDPTGPKLTLDRPISVNGIRFFFMDNDGPSGMMRPLSRDLHAAINRVA
TDFNAKRVNIRKMKWSLDISLSAMLTMKNIETIYHKTEEGEQPKTVCKETVKYFFGCSDSILPSVIFGHL
QNFMKIIPNSRHKHLASIIEALKTEFKEMLGNDGVFLYPTFPNTAHQHYQIYHKLLEPMYMAIFNTLGLP
VTNCMIGLDRRNLPMGIQVVANPGQDHLSLAVAREMERRYGGWVRPPSEDSHSSGGSSKQRG

>Q9I7J0_DROME
MAPRNKEQEQEVLNWVFAVIGEKVPSGQYEDILKDGIWLCKLANKLAPGSVKKIQERGTNFQLMENIQRF
QAAVKKYGVPEEEIFQTADLFERRNIPQVTLSLYALGRITQKHPEYTGPTLGPKMADKNERSFTEEQLRA
HEGELNLQMGFNKGASQAGHGGMGNTRHM

>Q9I7J1_DROME
MQANSSTTTTTTTTKTKKLSSGTGTNMISSVAVTSAIKTTTTTRKPAGSGVSSKVSKAQGATNSTPASSD
SNAEIAELTKKINDHADAIYHTWKSQGIPPTELLTMYTSAAASGDLADVASPTADAEGLQKMVTSFVNKD
KEQRGKSNTLKKSDITTANGKVKKAAVSSFSPVPDQALQSPKKVAAVTVSKPLPDVNLNYDISLDLDVNN
LSSQQTQNLLKISELIQQQKDVAATVGKKRQNSKANSSSSINSNHIGNSASSNKLTASEQPTKKQSKANS
SGSTRTGSAAVSLDVVDGPSAARIANSTAVSDKGGEPAATGSRKSSKTKETSADSKPATKEKPTAVATVA

NARKSTSRIATDNAGNMAAATAAAAATSATSAATATSMDATPATAASTTMPTLNKVKPTSGARAALANGQ
DKMNLLTRGSVAERVLMFEKCPDVRHAFLNIKRPQTDPPPKSLMKVKLHATPPPPPQEQNLLQKEIRSTK
SVYIPRFYFPHGKPQPNIAMERVVRGILSAFDSFPNNQVTKDELPRILKICGLPFYWRMPVMVFCQSAST
GLVERQRFVEFWKQMNVYCHEAASRFVYILSRGQRFRSYIVPEDLVPMVQDVVDTHPGLAFLKEATEFHS
RYVHTVIARIFYSVNRSWSGKITIAELKRSDLLEMISLLEEEEDINQIMAFFSYEHFYVIYCKFWELDKD
HDLLINQEDLAKHSDHALSSRIVERIFSGCVTRSDNKKAPEDEAKMSYTDFVWFILSEEDKRTPTAIEYW
FRCMDVDGDGVLSMYELEYFYEEQQQRMEGIGIECLPFEDCLCQMLDMIKPANRDCITLGDLKRCKMTHV
FFDTFFNLEKYLDHEQRDPFASQRDEYTSDWDRFAAQEYELLISEEND

>Q9I7J2_DROME
MRSLEGSRRRQVEQLHLVLEAKDQDQELNTRQEQEQSVARPSMANERKQSSPKVELTKLSHDLIEECKRS
QEDSLLSRHLRHELEKLPRDSRRELVRKQRNGCAPLFIACKRGAVDIAEYLITICEANIEQRGHFEVPED
NSFHYVSPLWAAVVSGKLSMVKYLVRIGCDINATSDSGSTPVRSACYMTHVDIVKFLVENGADIKRPNIN
GGTCLINSVQSVQLCLYLVRKGADINARDIQDKTALHYAIQEHRLDTTKLLIEQGADPYARSRYGDDALR
TACLKGAHHIFDFLKKQLHYTPARLAEAHELMGSTFLDEHNESRVCILHWRMAHHIRAAYSPYIEKKPQV
PLRTAYENAVEFSTLEELDNIATDMDAMRTQSLLICERVLGLTHKDMLFRLTFRGASYADSLQLQRCIDL
WRFLLEVRVSNWSILHFETCFAAQALVRLMLDLHVQNSSHIRSDARARFVHQDKVLPRFEDVLGVFRTLS
ESAIVVKHLLLLRPVFRRQQENYDRVMRCLAHLIYLLINTVHTEAQNKLICQAVHEAVVVGNLRSASTAD
TMLHLCASRLNVIKSGYITDDNFADKTVFPNADVIKLLIQCGVDVNTKNEAKSTPLHVACQPYNYDNEIV
HLLLKCGGDIDQPNRADKRPYDLIASNPTSTIPLLNFVTLQCLAATAISKHRIPYHNQLHRQLEKFVRNH
EP

>JUPIT_DROME
MAAYAAFKHVELYNVGKAKKRVLRPPGGGSSDIFGSEMPQTPRNVKNRMASNIFAAEKDNGVKNNVRQGA
HRFYFIGDAPRRGQKTVDSHSRLFGEPTRPITPGKNHMKSSIPFGQNTEAVAAQKLLTTNGHYNGKSGSV
SSASSSVSSSTENLKMNSGSRSEGNPVTGEGYKVVANEYSQRQESSNGGTPVINKNRIPPGGYSSGLW

>Q9I7K0-3
MSTRPDTKETSPRVSLCPPEPARTETPIPPADDALSIDNSCRDSEVGDVPADNSTVTKSDQVNEGCQTRR
DSGNNPEQPYSLNKMAGVSNVKEPLGLCPNEIKEEQQACSKLDSRNPITGLGLNGDGVGGLKPKKLKIRE
GNPVTGEGYKVVANEYSQRQESSNGGTPVINKNRIPPGGYSSGLW

>Q9I7K4_DROME
MQPFIVIRNYTQDDELKCQELVRDYIMSFSNKSFFVYCFREITLQFIVITWAIFFIFLGVPLLFCALTVP
ACIFCLFTGTYFSFYSKAVELMRTKPSQSLVAECYEPFIFRCSPKEASYQIFTENCPYEETYTRKFRRRI
VAAISVKNHHAVYNAAWIYRFAIDPHYPCQTIMDPMIKLVVKNCIIGGYASLECTISEWQESERDFYDDF
GFVTRQIYHKKIIGSSLAVMKTQLTYGLRTDGAALHKQN

>NASP_DROME
MSAEAEAIVTTATADVSSPSKTVAVEPVAADTTPDNAPAVSTEGSGKAEQERAEKILKGKELFSQGSRNF
LVKSYDEAADELSQVCQLYEEVYGELADELGQPLLLYAKALIAMALDENKVIDVPDEAADDDDEDVDDDE
EESAEDGAAKKEEKKDTKEAANGASSSNGKELDTIKEGSDEADSTGEAEQAQSDEKPSKKVPTGVDEVSS
SNGGGGAAVNDDERPSTSNGEVTASCSNGAAPAVEEEPEEEEGVSGSLQLAWEILEAAAQIFSRQGLSGL
PYLAEVQTELANIEFENGILEAAREDYEKALKIHGELPTRNRRALAELHYKIGLTYLMQQLNKEGATALR

QSSVLIEEEIAEIKGKDEPSERDRNNMLDLEETKQEILAKIQEIEEMQAQTIAEVRAALDSYIKPMSSGD
AAAASSSSSSSANGAASSSSSSSSKGAAAASSSTISSSSAKPTDITHLIKRKKPEDPSSEAEALCSPAKRA
AV

>Q9I7K8_DROME
MVICEIEFCNNSQGIFYAGQLISGQVVIKTEKEKSVKAVILNIKGYAETHWADTEHDPDDQSNGESFNGH
VDYLATRAYLHGSSSSIEVLIEPGTSSYRFACQLPITCPSSFEGTLGRIRYLVNVRFVRPWKFDLNFNRC
FTVIKVMDLNSESLMLRVPSQVESQRTFCCFPCRSSPLSMRLSVPQSGFVPGQIVPVEVMVSNDSGVAVE
DITVKLTMVVIYYSQPPSADTNKDRFEMVLKTGGGVSTKCRQQFTFDLKVPPTPPTCFNLCSIIQIGYQV
EAEARVKGCHGGQSLHMPITIGSVPLTKQLQKEPRTWGEVLPPQQLDAKALILIGSEQNGEALGSPNPWA
ADPSIAPPSYAEAKHISPDPHKFSKSKKKSQKRGVKGSQERKAETIVFSPLYAVFDLSNQVDEMTLRANE
PKTDGGYVNEGVEKSTWL

>Q9I7L0_DROME
MTVTCEIDFDNNPHGTYFGGEVLTGRVTLKLDKMKLVKAITLNITGYAETRWIERVTTNRRRRRRTFCGR
EDYIASKTFLVGSNLSSQVSIEAGIHTYNFVCLIPTECPSSFEGSHGRVRYMATVTLVRPWKFDQSYTRC
FTVLKVMDLNFDSPLLRVPAHSETSKTYCCWPCRSDPLALQLTVPQTGFVPGQNVPLSVLVTNDSHIPVE
QLLISFVMLVTYHSKPPSMPNTTSERLVVNTFKGDAVQRNCKKLFSYEIRVPATPPTCFNLCGIIQIAYQ
VEVEARVKGCHNNEVVTIPLTIGSVPLAQHVPIQPRGFVPQLNVNELAVEEVATAPNSSSPWSVDASIPP
PNYQEAVHMRSTAATRSDDLDDPEPVPPNTLSLDGGAYKPLYPVFDIPSPSAPPPTDYTQNYMAERAFVN
PAMDVDKDKGTWL

>Q9I7L2_DROME
MALLEYVDNRGRRELSCGGSLINNRYVLTAAHCVIGAVETEVGHLTTVRLGEYDTSKDVDCIDDICNQPI
LQLGIEQATVHPQYDPANKNRIHDIALLRLDRPVVLNEYIQPVCLPLVSTRMAINTGELLVVSGWGRTTT
ARKSTIKQRLDLPVNDHDYCARKFATRNIHLISSQLCVGGEFYRDSCDGDSGGPLMRRGFDQAWYQEGVV
SFGNRCGLEGWPGVYTRVADYMDWIVETIRP

>E4YGN5_OIKDI
MVTPTPPRTEPDHMARGGLDTNCGPLHLRFEPCLFFEAIKIVEVESIKQENSADDDESSSPVESAITEEI
EQEEFNVDIAEEKRLLKNEIEEESSDSERLELTSPALINEHSEGAQSRCSRSDSDIKSTSSENQLKRKYS
HTIADIIPDKTARQDDSSSDVEEINSTPPIARFAANSNSWAFQPTASLPSRVTGGLNGLPGVPVAHQPVS
PALMEAFQRARQGVASIQSQVSPIGFALRVQQQLQANALFTMLRQQQQVKVKSSSFTNYSPLMKSFNHLN
HRFPAGMNPRVPPASLQFPPPQTQTRRRPNGQKNDRCEYCGKVFKNTSNLTVHRRMHTGERPYKCKLCDY
ACAQSSKLTRHMKTHGTSREHQHKCDICGVPFAVFSTLEKHMKKEHADQLNERMNGHFMTLPSSSK

>E4YGT7_OIKDI
MKSRFSIFVLLISSIRANTEIKDELKELLKDSTVVNTLVDILDYKLSKKTEELEKKVTVEKLKGEKIVGN
KWKEVNAYSDESCSTLCALYASCIGYNYLDSGKICVIFDSIANTEPDDLNFSGMIRRSYALAKIPSSPND
TAPHSSGNSDNYDLKIIKSAVAKHIHVCQKKFEFLSKKVKVTRDSLGAKIRSLEIDTSKSKCLKGGRPSA
CDNFAKKIEERNTDIYNAVLTYVEDQNAIKLIQEGMDTRLTEMGHLLATYQPVTVAQPAQDLIGSVLMQT
KEMQNNVRIELESKIERNDAEMRRNQGAISNLRSKIDKLPLAGQTSENEMSVLNQSQVFLVNLLKLRIS

>Q9I7L3_DROME

MRFAPSLSLKSTRGVHQSDTEVSKIMRSLDVIPDVIHIGPQEFLNVTYHGHLAAHCGKVLEPMQVRDEPS
VKWPSAPENYYALLMVDPDVPNAITPTHREFLHWMVLNIPGNLLALGDVRVGYMGATPLKGTGTHRFVFL
LYKQRDYTKFDFPKLPKHSVKGRSGFETKRFAKKYRFGHPVAGNFFTSQWSPDVPSLIKAISHNARQVAH
F

>Q9I7L5_DROME
MGFDMATRFMDILKLTFKVISFKYEQRKLSTAIYSVIKTKSGPVRGVKRNTIWGGSYFSFEKIPFAKPPV
GDLRFKAPEAVEPWDQELDCTSPADKPLQTHMFFRKYAGSEDCLYLNVYVKDLQPDKLRPVMVWIYGGGY
QVGEASRDMYSPDFFMSKDVVIVTVAYRLGALGFLSLDDPQLNVPGNAGLKDQIMALRWVQQNIEAFGGD
SNNITLFGESAGGASTHFLALSPQTEGLIHKAIVMSGSVLCPWTQPPRNNWAYRLAQKLGYTGDNKDKAI
FEFLRSMSGGEIVKATATVLSNDEKHHRILFAFGPVVEPYTTEHTVVAKQPHELMQNSWSHRIPMMFGGT
SFEGLLFYPEVSRRPATLDEVGNCKNLLPSDLGLNLDPKLRENYGLQLKKAYFGDEPCNQANMMKFLELC
SYREFWHPIYRAALNRVRQSSAPTYLYRFDHDSKLCNAIRIVLCGHQMRGVCHGDDLCYIFHSMLSHQSA
PDSPEHKVITGMVDVWTSFAAHGDPNCESIKSLKFAPIENVTNFKCLNIGDQFEVMALPELQKIEPVWNS
FYAPNKL
