# Supplementary material for: NR-2L: A Two-Level Predictor for Identifying Nuclear Receptor Subfamilies Based on Sequence-Derived Features
Source: PLoS One. 2011 Aug 15;6(8):e23505. doi: 10.1371/journal.pone.0023505 (PMC3156231; doi:10.1371/journal.pone.0023505)
Supplement: Supporting Information S3 — List of the jackknifing results obtained by NR-2L on the 159 NRs and 500 non-NRs in the dataset S (cf. Supporting Information S1), and the corresponding observed results as annotated in NucleaRDB or UniProt. (PDF) [file pone.0023505.s003.pdf]

Supporting Information S3. List of the jackknifing results obtained by **NR-2L** on the 159 NRs and 500 non-NRs in the dataset 𝕊 (cf. Supporting Information S1), and the corresponding observed results as annotated in NucleaRDB or UniProt.

I. Results of the 1st level prediction in identifying NRs or non-NRs

Protein code	Predicted	Observed
Q6TNS3_BRARE	NR	NR
O76246_UCAPU	NR	NR
RORA_HUMAN	NR	NR
Q9PTN2_BRARE	NR	NR
HR3_MANSE	NR	NR
Q9UNW4_HUMAN	NR	NR
Q86BC1_DROME	NR	NR
Q8JJ27_XENLA	NR	NR
E75_METEN	NR	NR
E78A_DROME	NR	NR
Q80ST6_MOUSE	NR	NR
RORG_HUMAN	NR	NR
O02643_CHOFU	NR	NR
Q7ZZY9_PETMA	NR	NR
Q9DF24_XENLA	NR	NR
Q9DFH3_CHICK	NR	NR
Q60Q78_CAEBR	NR	NR
NR1I3_RAT	NR	NR
Q9XXU7_CAEEL	NR	NR
Q6GZ85_HUMAN	NR	NR
NHR1_ONCVO	NR	NR
HR96_DROME	NR	NR
RXRB_HUMAN	NR	NR
Q9BMU6_AEDAE	NR	NR
Q8MX78_BRAFL	NR	NR
Q6GN21_XENLA	NR	NR
7UP2_DROME	NR	NR
O76241_UCAPU	NR	NR
Q8IPF2_DROME	NR	NR
USP_CHOFU	NR	NR
Q9U3Y3_AEDAL	NR	NR
EAR2_HUMAN	NR	NR
Q9NG48_APIME	NR	NR

USP_DROME	NR	NR
O45117_CHITE	NR	NR
Q8CFY1_MOUSE	NR	NR
O97120_SCHMA	NR	NR
O96562_TRICY	NR	NR
Q6ZMP8_HUMAN	NR	NR
O46174_BOMMO	NR	NR
O77099_AEDAE	NR	NR
Q26622_STRPU	NR	NR
Q8ST32_DIRIM	NR	NR
Q6PHZ7_HUMAN	NR	NR
Q8I748_SUBDO	NR	NR
Q8MLA0_DROME	NR	NR
Q6P0E0_BRARE	NR	NR
Q9V7B3_DROME	NR	NR
Q9U9R6_SCHMA	NR	NR
NR2E3_MOUSE	NR	NR
Q5U3F3_BRARE	NR	NR
NHR64_CAEEL	NR	NR
Q7YTB9_SACKO	NR	NR
Q8IPT4_DROME	NR	NR
TLL_DROME	NR	NR
Q9NCL0_TRICA	NR	NR
O96680_DROME	NR	NR
P90892_CAEEL	NR	NR
ANDR_HUMAN	NR	NR
GCR_RAT	NR	NR
Q6XLI8_CALJA	NR	NR
PRGR_CANFA	NR	NR
Q8TDS3_HUMAN	NR	NR
Q6QB13_DICLA	NR	NR
Q8JJ92_HAPBU	NR	NR
GCR_ONCMY	NR	NR
Q8JJ89_HAPBU	NR	NR
P70048_XENLA	NR	NR
Q5WP02_ONCMY	NR	NR
Q8QFV2_CARAU	NR	NR
PRGR_CHICK	NR	NR
Q800S7_ACASC	NR	NR
O93244_ONCMY	NR	NR
Q76LM5_ORYLA	NR	NR

Q9YGV9_ANGJA	NR	NR
Q9DDU9_XENLA	NR	NR
PRGR_RANDY	NR	NR
Q6RKQ3_ONCMY	NR	NR
Q9IBD5_ANGJA	NR	NR
Q6A4C2_DICLA	NR	NR
Q8QGX5_ANGJA	NR	NR
Q8BG65_MOUSE	NR	NR
ESR1_RAT	NR	NR
Q9W6F4_HAPBU	NR	NR
Q6R7S4_SALSA	NR	NR
Q7T3U4_9TELE	NR	NR
Q762D6_CONMY	NR	NR
ESR2_MICUN	NR	NR
Q90WH6_CLAGA	NR	NR
Q90ZE6_SQUAC	NR	NR
ERR1_HUMAN	NR	NR
ERR2_HUMAN	NR	NR
Q5XTQ9_BRAFL	NR	NR
Q5XTR0_BRAFL	NR	NR
Q8WS79_DROME	NR	NR
Q6ZMM6_HUMAN	NR	NR
Q98TQ3_ORYLA	NR	NR
O97726_PIG	NR	NR
Q6INY4_XENLA	NR	NR
HR38_DROME	non-NR	NR
Q9U4L1_AEDAE	NR	NR
NHR6_CAEEL	NR	NR
Q9QWM1_RAT	NR	NR
P97782_MOUSE	NR	NR
Q8JH98_CARAU	NR	NR
Q8UV26_CLAGA	NR	NR
Q98ST8_BRARE	NR	NR
Q6W954_EPICO	NR	NR
FTZF1_DROME	NR	NR
Q9NB03_AEDAE	NR	NR
Q8WSJ3_MANSE	NR	NR
Q9Y0D1_METEN	NR	NR
Q9BPL0_SCHMA	NR	NR
FTF1B_DROME	NR	NR
NR6A1_MOUSE	NR	NR

NR6A1_HUMAN	NR	NR
Q66JK1_XENTR	NR	NR
P70033_XENLA	NR	NR
Q9PU65_BRARE	NR	NR
A0A178_CARAU	non-NR	non-NR
A0A180_XENLA	non-NR	non-NR
A0A181_DANRE	non-NR	non-NR
LCE6A_HUMAN	non-NR	non-NR
EYS_DROME	non-NR	non-NR
A0A1G5_9BIVA	non-NR	non-NR
A0A4R1_HUMAN	non-NR	non-NR
A0A4W4_DANRE	non-NR	non-NR
B5DVZ7_DROPS	non-NR	non-NR
B5DW00_DROPS	non-NR	non-NR
B5DW05_DROPS	non-NR	non-NR
B5DW07_DROPS	non-NR	non-NR
B5DW09_DROPS	non-NR	non-NR
B5DW10_DROPS	non-NR	non-NR
B5DW13_DROPS	non-NR	non-NR
B5DW14_DROPS	non-NR	non-NR
B5DW17_DROPS	non-NR	non-NR
B5DW18_DROPS	non-NR	non-NR
B5DW19_DROPS	non-NR	non-NR
B5DW21_DROPS	non-NR	non-NR
B5DW23_DROPS	non-NR	non-NR
B5DW25_DROPS	non-NR	non-NR
B5DW26_DROPS	non-NR	non-NR
B5DW27_DROPS	non-NR	non-NR
B5DW28_DROPS	non-NR	non-NR
B5DW29_DROPS	non-NR	non-NR
B5DW32_DROPS	non-NR	non-NR
B5DW33_DROPS	non-NR	non-NR
B5DW38_DROPS	non-NR	non-NR
B5DW41_DROPS	non-NR	non-NR
B5DW42_DROPS	non-NR	non-NR
B5DW43_DROPS	non-NR	non-NR
B5DW44_DROPS	non-NR	non-NR
B5DW45_DROPS	non-NR	non-NR
B5DW46_DROPS	non-NR	non-NR
B5DW47_DROPS	non-NR	non-NR
B5DW48_DROPS	NR	non-NR

B5DW50_DROPS	non-NR	non-NR
B5DW51_DROPS	non-NR	non-NR
B5DW53_DROPS	non-NR	non-NR
B5DW54_DROPS	non-NR	non-NR
B5DW55_DROPS	non-NR	non-NR
B5DW63_DROPS	non-NR	non-NR
B5DW64_DROPS	non-NR	non-NR
B5DW66_DROPS	non-NR	non-NR
B5DW68_DROPS	non-NR	non-NR
B5DW70_DROPS	non-NR	non-NR
B5DW73_DROPS	non-NR	non-NR
B5DW74_DROPS	non-NR	non-NR
B5DW75_DROPS	non-NR	non-NR
B5DW76_DROPS	non-NR	non-NR
E1FMB3_LOALO	non-NR	non-NR
E1FMB5_LOALO	non-NR	non-NR
E1FMB7_LOALO	non-NR	non-NR
E1FMC1_LOALO	non-NR	non-NR
E1FMC2_LOALO	non-NR	non-NR
E1FMC3_LOALO	non-NR	non-NR
E1FMC4_LOALO	non-NR	non-NR
E1FMC5_LOALO	non-NR	non-NR
E1FMC9_LOALO	non-NR	non-NR
E1FMD1_LOALO	non-NR	non-NR
E1FMD2_LOALO	non-NR	non-NR
E1FMD4_LOALO	NR	non-NR
E1FMD5_LOALO	non-NR	non-NR
E1FMD6_LOALO	non-NR	non-NR
E1FMD7_LOALO	non-NR	non-NR
E1FMD8_LOALO	non-NR	non-NR
E1FME3_LOALO	non-NR	non-NR
E1FME4_LOALO	non-NR	non-NR
E1FME5_LOALO	non-NR	non-NR
E1FME8_LOALO	non-NR	non-NR
E1FMF1_LOALO	non-NR	non-NR
E1FMF2_LOALO	non-NR	non-NR
E1FMF3_LOALO	non-NR	non-NR
E1FMF4_LOALO	non-NR	non-NR
E1FMF5_LOALO	non-NR	non-NR
E1FMF7_LOALO	NR	non-NR
E1FMF9_LOALO	non-NR	non-NR

E1FMG1_LOAL0	non-NR	non-NR
E1FMG2_LOAL0	non-NR	non-NR
E1FMG3_LOAL0	non-NR	non-NR
E1FMG4_LOAL0	non-NR	non-NR
E1FMG5_LOAL0	non-NR	non-NR
E1FMH1_LOAL0	non-NR	non-NR
E1FMH5_LOAL0	non-NR	non-NR
E1FMH6_LOAL0	non-NR	non-NR
E1FMH7_LOAL0	non-NR	non-NR
E1FMI1_LOAL0	non-NR	non-NR
E1FMI3_LOAL0	NR	non-NR
E1FMI5_LOAL0	non-NR	non-NR
E1FMI9_LOAL0	non-NR	non-NR
E1FMJ0_LOAL0	non-NR	non-NR
E1FMJ3_LOAL0	non-NR	non-NR
E1FMJ5_LOAL0	non-NR	non-NR
E1FMK6_LOAL0	non-NR	non-NR
E1FMK9_LOAL0	non-NR	non-NR
E1FML0_LOAL0	non-NR	non-NR
E1FML2_LOAL0	non-NR	non-NR
E1FML9_LOAL0	non-NR	non-NR
E1FMM1_LOAL0	non-NR	non-NR
E1FMM4_LOAL0	non-NR	non-NR
E1FMM7_LOAL0	non-NR	non-NR
E1FMM9_LOAL0	non-NR	non-NR
E1FMN0_LOAL0	NR	non-NR
E1FMN1_LOAL0	non-NR	non-NR
E1FMN4_LOAL0	non-NR	non-NR
E1FMN7_LOAL0	non-NR	non-NR
E1FMP1_LOAL0	non-NR	non-NR
E1FMP4_LOAL0	non-NR	non-NR
E1FMP5_LOAL0	non-NR	non-NR
E1FMP6_LOAL0	non-NR	non-NR
E1FMP7_LOAL0	non-NR	non-NR
E1FMQ0_LOAL0	non-NR	non-NR
E1FMQ1_LOAL0	non-NR	non-NR
E1FMQ6_LOAL0	non-NR	non-NR
E1FMQ8_LOAL0	non-NR	non-NR
E1FMR0_LOAL0	non-NR	non-NR
E1FMR2_LOAL0	non-NR	non-NR
E1FMR8_LOAL0	non-NR	non-NR

E1FMS2_LOALO	non-NR	non-NR
E1FMS5_LOALO	non-NR	non-NR
E1FMS6_LOALO	non-NR	non-NR
E1FMS7_LOALO	non-NR	non-NR
E1FMS9_LOALO	non-NR	non-NR
E1FMT0_LOALO	non-NR	non-NR
E1FMT2_LOALO	non-NR	non-NR
E1FMT5_LOALO	non-NR	non-NR
E1FMT7_LOALO	non-NR	non-NR
E1FMT8_LOALO	non-NR	non-NR
E1FMU1_LOALO	non-NR	non-NR
E1FMU4_LOALO	non-NR	non-NR
E1FMU5_LOALO	non-NR	non-NR
E1FMU8_LOALO	non-NR	non-NR
E1FMU9_LOALO	non-NR	non-NR
E1FMV1_LOALO	non-NR	non-NR
E1FMV2_LOALO	non-NR	non-NR
E1FMV6_LOALO	non-NR	non-NR
E1FMV8_LOALO	non-NR	non-NR
E1FMV9_LOALO	non-NR	non-NR
E1FMW1_LOALO	non-NR	non-NR
E1FMW2_LOALO	non-NR	non-NR
E1FMW4_LOALO	non-NR	non-NR
E1FMW5_LOALO	non-NR	non-NR
E1FMW6_LOALO	non-NR	non-NR
E1FMW7_LOALO	non-NR	non-NR
E1FMX3_LOALO	non-NR	non-NR
E1FMX4_LOALO	non-NR	non-NR
E1FMX7_LOALO	non-NR	non-NR
E1FMX8_LOALO	non-NR	non-NR
E1FMY0_LOALO	non-NR	non-NR
E1FMY4_LOALO	non-NR	non-NR
E1FMY6_LOALO	non-NR	non-NR
E1FMY8_LOALO	non-NR	non-NR
E1FMZ2_LOALO	non-NR	non-NR
E1FMZ4_LOALO	non-NR	non-NR
E1FMZ6_LOALO	non-NR	non-NR
CLDZ_DANRE	non-NR	non-NR
CLD7B_DANRE	non-NR	non-NR
CND3_XENLA	non-NR	non-NR
BAG6A_XENLA	non-NR	non-NR

Q9YHT5_XENLA	non-NR	non-NR
NOGG3_DANRE	non-NR	non-NR
Q9YHV6_TAKRU	non-NR	non-NR
Q9YI01_DANRE	non-NR	non-NR
Q9YI32_CARAU	non-NR	non-NR
TYRO3_DANRE	non-NR	non-NR
Q9YI90_XENLA	non-NR	non-NR
Q9YIB1_ANGJA	non-NR	non-NR
LEG2_CONMY	non-NR	non-NR
SYUG_MOUSE	non-NR	non-NR
CDC7_MOUSE	non-NR	non-NR
WDR46_MOUSE	non-NR	non-NR
CST9_MOUSE	non-NR	non-NR
Q9Z0I5_MOUSE	non-NR	non-NR
ITSN2_MOUSE	non-NR	non-NR
CLD13_MOUSE	non-NR	non-NR
TI17A_MOUSE	non-NR	non-NR
PA21B_MOUSE	non-NR	non-NR
IRS4_MOUSE	non-NR	non-NR
STAU1_MOUSE	non-NR	non-NR
Q9Z117_MOUSE	non-NR	non-NR
Q9Z161_RAT	non-NR	non-NR
P2R3A_MOUSE	non-NR	non-NR
Q9Z190_MOUSE	non-NR	non-NR
EYA4_MOUSE	NR	non-NR
ZSC12_MOUSE	NR	non-NR
DEF3B_RAT	non-NR	non-NR
Q9Z1F3_MOUSE	non-NR	non-NR
SAE2_MOUSE	non-NR	non-NR
VPP1_MOUSE	non-NR	non-NR
NEK4_MOUSE	non-NR	non-NR
NDUA7_MOUSE	non-NR	non-NR
KANK3_MOUSE	non-NR	non-NR
BAG6_MOUSE	non-NR	non-NR
GRP1_MOUSE	non-NR	non-NR
SEPT3_MOUSE	non-NR	non-NR
AP3B1_MOUSE	non-NR	non-NR
Q9Z1W6-4	non-NR	non-NR
HNRPC_MOUSE	non-NR	non-NR
Q9Z271_MOUSE	non-NR	non-NR
TULP1_MOUSE	non-NR	non-NR

PLD1_MOUSE	non-NR	non-NR
Q9Z284_MOUSE	non-NR	non-NR
KR121_MOUSE	non-NR	non-NR
RGR_MOUSE	non-NR	non-NR
E2AK3_MOUSE	non-NR	non-NR
ATOH7_MOUSE	non-NR	non-NR
OASL2_MOUSE	non-NR	non-NR
Q9Z2G2_MOUSE	non-NR	non-NR
LETM1_MOUSE	non-NR	non-NR
PMM2_MOUSE	non-NR	non-NR
Q9Z2P0_MOUSE	non-NR	non-NR
VAMP5_MOUSE	non-NR	non-NR
Q9Z2R2_MOUSE	non-NR	non-NR
APOC3_CAVPO	non-NR	non-NR
Q9Z2R8_MOUSE	NR	non-NR
ZN292_MOUSE	non-NR	non-NR
Q9Z2U9_RAT	non-NR	non-NR
Q9Z2V0_MOUSE	non-NR	non-NR
HDAC6_MOUSE	non-NR	non-NR
Q9Z2X7_RAT	non-NR	non-NR
CORIN_MOUSE	non-NR	non-NR
Q9Z322_RAT	NR	non-NR
KCNQ2_MOUSE	non-NR	non-NR
THIO_RICPR	non-NR	non-NR
CSTA_HELPJ	non-NR	non-NR
DHE4_HELPJ	NR	non-NR
Y944_HELPJ	non-NR	non-NR
SCOB_HELPJ	non-NR	non-NR
Q9ZNT0_ARATH	non-NR	non-NR
APC10_ARATH	non-NR	non-NR
VATF_ARATH	non-NR	non-NR
RABD1_ARATH	non-NR	non-NR
Q9ZUX0_ARATH	NR	non-NR
Y1461_ARATH	non-NR	non-NR
PCNA2_ARATH	non-NR	non-NR
NU5M_RHISA	non-NR	non-NR
NU4LM_SQUAC	non-NR	non-NR
NU6M_CANFA	non-NR	non-NR
A3LT2_RAT	NR	non-NR
A0A504_MOUSE	non-NR	non-NR
A0A5F3_MOUSE	non-NR	non-NR

A0A7V1_PAROL	NR	non-NR
A0A8M4_9CRUS	non-NR	non-NR
A0A8M5_9CRUS	non-NR	non-NR
A0A9I9_MARSA	non-NR	non-NR
A0A9P7_TAKRU	non-NR	non-NR
A0A9Q6_9ASCI	non-NR	non-NR
A0A9Q7_9ASCI	NR	non-NR
A0A9Q9_9ASCI	non-NR	non-NR
A0A9R0_9ASCI	non-NR	non-NR
A0A9R2_9ASCI	non-NR	non-NR
A0A9R3_9ASCI	non-NR	non-NR
A0A9R4_9ASCI	non-NR	non-NR
A0A9S0_CAEEL	non-NR	non-NR
A0A9S1_CAEEL	non-NR	non-NR
A0A9S2_CAEEL	non-NR	non-NR
A0A9S4_CAEEL	non-NR	non-NR
A0A9S5_CAEEL	non-NR	non-NR
A0A9S6_CAEEL	NR	non-NR
A0A9S7_CAEEL	non-NR	non-NR
A0AAB8_CAEEL	non-NR	non-NR
A0AAB9_CAEEL	non-NR	non-NR
A0AAC0_CAEEL	non-NR	non-NR
A0AAC1_CAEEL	non-NR	non-NR
BR2GC_RANGU	non-NR	non-NR
A0AMI7_DROME	non-NR	non-NR
A0AMQ8_DROME	NR	non-NR
A0AMT9_DROME	non-NR	non-NR
A0AMX2_DROME	NR	non-NR
A0AN03_DROME	NR	non-NR
A0AN67_DROME	non-NR	non-NR
A0ANA1_DROME	non-NR	non-NR
A0ANB3_DROME	non-NR	non-NR
A0ANE1_DROME	non-NR	non-NR
A0ANM3_DROME	non-NR	non-NR
A0ANU3_DROME	NR	non-NR
A0AP24_DROME	non-NR	non-NR
A0AP42_DROME	non-NR	non-NR
A0APF4_DROME	non-NR	non-NR
A0APM6_DROME	non-NR	non-NR
A0APN6_DROME	non-NR	non-NR
A0APP3_DROME	non-NR	non-NR

A0APQ1_DROME	non-NR	non-NR
A0APW6_DROME	non-NR	non-NR
A0APX3_DROME	non-NR	non-NR
A0AQ14_DROME	non-NR	non-NR
A0AQ96_DROME	non-NR	non-NR
A0AQD3_DROME	non-NR	non-NR
A0AQD7_DROME	non-NR	non-NR
A0AQL9_TRISP	NR	non-NR
A0AR24_HUMAN	non-NR	non-NR
A0AR27_HUMAN	NR	non-NR
A0AUH1_HUMAN	NR	non-NR
A0AUJ2_HUMAN	non-NR	non-NR
A0AUP8_DANRE	non-NR	non-NR
A0AUQ0_DANRE	non-NR	non-NR
A0AUR7_DANRE	non-NR	non-NR
SMKY_MOUSE	NR	non-NR
CB067_HUMAN	non-NR	non-NR
S12A8_HUMAN	non-NR	non-NR
A0AV37_HUMAN	non-NR	non-NR
RBM47_HUMAN	NR	non-NR
TTC26_HUMAN	non-NR	non-NR
A0AVG2_HUMAN	non-NR	non-NR
FR1L5_HUMAN	non-NR	non-NR
TM129_HUMAN	NR	non-NR
E2F8_HUMAN	non-NR	non-NR
UBA6_HUMAN	non-NR	non-NR
A0AVV0_DROME	non-NR	non-NR
A0AVX5_DROME	NR	non-NR
A0AYH9_BURCH	NR	non-NR
A0B2G5_BURCH	non-NR	non-NR
RL72_PARTE	non-NR	non-NR
A0BJ67_PARTE	non-NR	non-NR
A0BST8_PARTE	NR	non-NR
A0BUY5_PARTE	non-NR	non-NR
A0C2K3_PARTE	non-NR	non-NR
PPX4_PARTE	non-NR	non-NR
A0CFU8_PARTE	non-NR	non-NR
A0CP12_PARTE	non-NR	non-NR
A0CPX7_PARTE	non-NR	non-NR
A0CT56_PARTE	non-NR	non-NR
A0CZW3_PARTE	non-NR	non-NR

A0CZY2_PARTE	non-NR	non-NR
A0D7E8_PARTE	non-NR	non-NR
A0DCA3_PARTE	non-NR	non-NR
A0DHD2_PARTE	non-NR	non-NR
A0DJ92_PARTE	non-NR	non-NR
A0DLP9_PARTE	non-NR	non-NR
A0DLR9_PARTE	NR	non-NR
A0DMR3_PARTE	non-NR	non-NR
A0DPG3_PARTE	non-NR	non-NR
A0DVJ0_PARTE	non-NR	non-NR
A0DVQ0_PARTE	non-NR	non-NR
A0DX60_PARTE	non-NR	non-NR
A0DZ45_PARTE	non-NR	non-NR
A0E3C0_PARTE	non-NR	non-NR
A0E9R7_PARTE	non-NR	non-NR
A0EAD3_PARTE	non-NR	non-NR
A0ECE3_PARTE	non-NR	non-NR
A0ELU3_9BIVA	NR	non-NR
A0EM56_BOMMO	non-NR	non-NR
A0EM58_APIME	non-NR	non-NR
A0EM59_APIME	non-NR	non-NR
A0EXG3_MOUSE	non-NR	non-NR
A0EYL8_NEMVE	NR	non-NR
A0EYM1_NEMVE	NR	non-NR
A0EYM2_9BIVA	non-NR	non-NR
A0EYM5_XENBC	non-NR	non-NR
A0EYM6_XENBC	non-NR	non-NR
A0EYM9_XENBC	non-NR	non-NR
A0EYN0_XENBC	non-NR	non-NR
A0EYN1_XENBC	non-NR	non-NR
A0EYN3_XENBC	non-NR	non-NR
A0EYN5_XENBC	non-NR	non-NR
A0EZR6_AEDAE	non-NR	non-NR
A0EZR7_AEDAE	non-NR	non-NR
A0EZR9_AEDAE	non-NR	non-NR
A0EZS2_AEDAE	non-NR	non-NR
A0EZS5_PSEMX	non-NR	non-NR
A0F019_PSEMX	non-NR	non-NR
A0F0C2_MESMA	non-NR	non-NR
A0FDP3_SACKO	NR	non-NR
A0FDP4_SACKO	non-NR	non-NR

A0FDP5_SACKO	non-NR	non-NR
A0FDP6_SACKO	non-NR	non-NR
A0FDP8_SACKO	non-NR	non-NR
A0FDP9_BOMMO	non-NR	non-NR
A0FDQ1_BOMMO	non-NR	non-NR
A0FDQ4_BOMMO	non-NR	non-NR
A0FDQ8_BOMMO	non-NR	non-NR
A0FDR0_BOMMO	non-NR	non-NR
A0FDR2_BOMMO	non-NR	non-NR
A0FDR3_BOMMO	NR	non-NR
A0FGR0_XENLA	NR	non-NR
ESYT2_HUMAN	non-NR	non-NR
ESYT3_HUMAN	non-NR	non-NR
A0FH50_DANRE	non-NR	non-NR
A0FIK6_HUMAN	non-NR	non-NR
A0FIU8_9DIPT	non-NR	non-NR
A0FIU9_9DIPT	non-NR	non-NR
A0FIV0_9DIPT	non-NR	non-NR
A0FIV1_9DIPT	non-NR	non-NR
A0FIV2_9DIPT	non-NR	non-NR
A0FIV3_9DIPT	non-NR	non-NR
A0FIV4_9DIPT	non-NR	non-NR
A0FIV5_9DIPT	non-NR	non-NR
A0FIV6_9DIPT	non-NR	non-NR
A0FJI1_DANRE	non-NR	non-NR
A0FJI2_DANRE	non-NR	non-NR
A0FJI3_DANRE	non-NR	non-NR
A0FK01_XENLA	NR	non-NR
A0FK59_CHICK	NR	non-NR
A0FK72_ARAVE	non-NR	non-NR
A0FKC7_CHICK	NR	non-NR
A0FKH2_LEPDE	non-NR	non-NR
A0FKN4_HUMAN	non-NR	non-NR
VMPA_LOXIN	non-NR	non-NR
A0FLQ5_CAEEL	non-NR	non-NR
A0FLR2_CAEEL	non-NR	non-NR
A0FLR4_CAEEL	non-NR	non-NR
A0FLR5_CAEEL	non-NR	non-NR
A0FLR6_CAEEL	NR	non-NR
A0FLR8_CAEEL	non-NR	non-NR
A0FLS3_CAEEL	non-NR	non-NR

A0FLS4_CAEEL	non-NR	non-NR
A0JCK6_PLUXY	non-NR	non-NR
A0JCS2_9HYME	non-NR	non-NR
A0JCS3_9HYME	non-NR	non-NR
A0JCS4_9HYME	NR	non-NR
A0JCS6_9HYME	non-NR	non-NR
A0JCS8_9HYME	non-NR	non-NR
A0JCS9_9HYME	non-NR	non-NR
A0JCT0_9HYME	non-NR	non-NR
A0JCT1_9HYME	non-NR	non-NR
A0JCT2_9HYME	non-NR	non-NR
A0JCT4_9HYME	non-NR	non-NR
A0JCT5_9HYME	non-NR	non-NR
A0JCT6_9HYME	non-NR	non-NR
A0JCT8_9HYME	non-NR	non-NR
A0JCU0_9HYME	non-NR	non-NR
A0JCU1_9HYME	non-NR	non-NR
A0JCU3_9HYME	non-NR	non-NR
A0JCU4_9HYME	non-NR	non-NR
A0JCU5_9HYME	non-NR	non-NR
A0JCU6_9HYME	non-NR	non-NR
A0JCU7_9HYME	non-NR	non-NR
A0JCU8_9HYME	non-NR	non-NR
A0JCV1_9HYME	non-NR	non-NR
A0JCV4_9HYME	non-NR	non-NR
A0JCV5_9HYME	non-NR	non-NR
A0JCV6_9HYME	non-NR	non-NR
A0JCV7_9HYME	non-NR	non-NR
A0JCV8_9HYME	non-NR	non-NR
A0JCV9_9HYME	non-NR	non-NR
A0JCW0_9HYME	non-NR	non-NR
A0JCW1_9HYME	non-NR	non-NR
A0JCW3_9HYME	non-NR	non-NR
A0JCW4_9HYME	non-NR	non-NR
A0JCW5_9HYME	NR	non-NR
A0JCW9_9HYME	non-NR	non-NR
A0JCX0_9HYME	non-NR	non-NR
A0JCX2_9HYME	non-NR	non-NR
A0JCX7_9HYME	non-NR	non-NR
A0JCX9_9HYME	non-NR	non-NR
A0JCY2_9HYME	non-NR	non-NR

A0JCY4_9HYME	NR	non-NR
A0JCY5_9HYME	non-NR	non-NR
A0JCY6_9HYME	non-NR	non-NR
A0JCY7_9HYME	non-NR	non-NR
A0JCZ0_9HYME	non-NR	non-NR
A0JCZ2_9HYME	non-NR	non-NR
A0JGX7_TAKRU	non-NR	non-NR
A0JGZ6_TETNG	non-NR	non-NR
A0JI74_SCHMA	NR	non-NR
A0JI76_SCHMA	non-NR	non-NR
A0JL26_DANRE	non-NR	non-NR
A0JLZ8_XENTR	non-NR	non-NR
K0284_XENTR	non-NR	non-NR
C2CD3_XENTR	non-NR	non-NR
A0JM29_XENTR	non-NR	non-NR
A0JM54_XENTR	non-NR	non-NR
UIMC1_XENTR	NR	non-NR
CA174_XENTR	non-NR	non-NR
TDRD5_XENTR	non-NR	non-NR
A0JMB8_DANRE	non-NR	non-NR
A0JMC6_DANRE	non-NR	non-NR
A0JMC9_DANRE	non-NR	non-NR
K0664_DANRE	non-NR	non-NR
LST2_DANRE	non-NR	non-NR
A0JMD3_DANRE	non-NR	non-NR
A0JMD5_DANRE	non-NR	non-NR
A0JMD6_DANRE	non-NR	non-NR
A0JMD7_DANRE	non-NR	non-NR
A0JMD8_DANRE	non-NR	non-NR
A0JME7_DANRE	non-NR	non-NR
CTC1_DANRE	non-NR	non-NR
A0JMF2_DANRE	non-NR	non-NR
A0JMF5_DANRE	non-NR	non-NR
HAUS6_DANRE	non-NR	non-NR
A0JMG0_DANRE	non-NR	non-NR
A0JMG4_DANRE	non-NR	non-NR
A0JMH5_DANRE	NR	non-NR
A0JMI1_DANRE	non-NR	non-NR
RRF2M_DANRE	non-NR	non-NR
A0JMK6_DANRE	non-NR	non-NR
BICR2_DANRE	non-NR	non-NR

CAF1A_DANRE	non-NR	non-NR
A0JML0_DANRE	non-NR	non-NR
A0JML6_DANRE	non-NR	non-NR
DALD3_DANRE	non-NR	non-NR
A0JMM0_DANRE	NR	non-NR
A0JMM2_DANRE	non-NR	non-NR
A0JMM5_DANRE	non-NR	non-NR
A0JMM6_DANRE	non-NR	non-NR
A0JMM7_DANRE	non-NR	non-NR
A2LDC_DANRE	non-NR	non-NR
ATHL1_DANRE	non-NR	non-NR
A0JMP5_DANRE	non-NR	non-NR

II. Results of the 2nd level prediction in identifying the subfamilies of NRs

Protein code	Predicted	Observed
THB2_RAT	NR1	NR1
Q64895_9RETR	NR3	NR1
Q5RZV7_PSEAM	NR1	NR1
RRG_NOTVI	NR1	NR1
Q15156_HUMAN	NR3	NR1
Q86WD1_HUMAN	NR1	NR1
Q658K5_HUMAN	NR4	NR1
NR1D2_HUMAN	NR1	NR1
Q6IBU6_HUMAN	NR1	NR1
Q922A5_MOUSE	NR1	NR1
Q6VA69_AEDAE	NR1	NR1
Q6PAY0_XENLA	NR1	NR1
NR1H3_HUMAN	NR1	NR1
Q9PW01_PLEPL	NR1	NR1
Q9GPH1_CALVI	NR1	NR1
O97095_LOCMI	NR1	NR1
Q7T030_PLEPL	NR1	NR1
ECR_DROME	NR1	NR1
Q90WP6_SALSA	NR1	NR1
Q6GMI3_BRARE	NR1	NR1
Q8WSA1_BOMMO	NR1	NR1
Q7T031_PLEPL	NR1	NR1
O44338_9ACAR	NR1	NR1
NR1H4_MOUSE	NR1	NR1
E75C_DROME	NR4	NR1

Q6GND0_XENLA	NR1	NR1
ECR_BOMMO	NR1	NR1
Q8MX80_BRAFL	NR1	NR1
Q6TNS3_BRARE	NR1	NR1
O76246_UCAPU	NR1	NR1
RORA_HUMAN	NR1	NR1
Q9PTN2_BRARE	NR1	NR1
HR3_MANSE	NR1	NR1
Q9UNW4_HUMAN	NR1	NR1
Q86BC1_DROME	NR1	NR1
Q8JJ27_XENLA	NR1	NR1
E75_METEN	NR1	NR1
E78A_DROME	NR3	NR1
Q80ST6_MOUSE	NR1	NR1
RORG_HUMAN	NR1	NR1
O02643_CHOFU	NR1	NR1
Q7ZZY9_PETMA	NR1	NR1
Q9DF24_XENLA	NR1	NR1
Q9DFH3_CHICK	NR1	NR1
Q60Q78_CAEBR	NR1	NR1
NR1I3_RAT	NR1	NR1
Q9XXU7_CAEEL	NR1	NR1
Q6GZ85_HUMAN	NR1	NR1
NHR1_ONCVO	NR2	NR1
HR96_DROME	NR3	NR1
RXRB_HUMAN	NR2	NR2
Q9BMU6_AEDAE	NR2	NR2
Q8MX78_BRAFL	NR2	NR2
Q6GN21_XENLA	NR2	NR2
7UP2_DROME	NR2	NR2
O76241_UCAPU	NR2	NR2
Q8IPF2_DROME	NR5	NR2
USP_CHOFU	NR2	NR2
Q9U3Y3_AEDAL	NR2	NR2
EAR2_HUMAN	NR2	NR2
Q9NG48_APIME	NR2	NR2
USP_DROME	NR2	NR2
O45117_CHITE	NR2	NR2
Q8CFY1_MOUSE	NR2	NR2
O97120_SCHMA	NR3	NR2
O96562_TRICY	NR2	NR2

Q6ZMP8_HUMAN	NR2	NR2
O46174_BOMMO	NR2	NR2
O77099_AEDAE	NR2	NR2
Q26622_STRPU	NR2	NR2
Q8ST32_DIRIM	NR2	NR2
Q6PHZ7_HUMAN	NR2	NR2
Q8I748_SUBDO	NR4	NR2
Q8MLA0_DROME	NR2	NR2
Q6P0E0_BRARE	NR2	NR2
Q9V7B3_DROME	NR2	NR2
Q9U9R6_SCHMA	NR2	NR2
NR2E3_MOUSE	NR2	NR2
Q5U3F3_BRARE	NR2	NR2
NHR64_CAEEL	NR2	NR2
Q7YTB9_SACKO	NR2	NR2
Q8IPT4_DROME	NR3	NR2
TLL_DROME	NR2	NR2
Q9NCL0_TRICA	NR2	NR2
O96680_DROME	NR4	NR2
P90892_CAEEL	NR2	NR2
ANDR_HUMAN	NR3	NR3
GCR_RAT	NR3	NR3
Q6XLI8_CALJA	NR3	NR3
PRGR_CANFA	NR3	NR3
Q8TDS3_HUMAN	NR3	NR3
Q6QB13_DICLA	NR3	NR3
Q8JJ92_HAPBU	NR3	NR3
GCR_ONCMY	NR3	NR3
Q8JJ89_HAPBU	NR3	NR3
P70048_XENLA	NR3	NR3
Q5WP02_ONCMY	NR3	NR3
Q8QFV2_CARAU	NR3	NR3
PRGR_CHICK	NR3	NR3
Q800S7_ACASC	NR3	NR3
O93244_ONCMY	NR3	NR3
Q76LM5_ORYLA	NR3	NR3
Q9YGV9_ANGJA	NR3	NR3
Q9DDU9_XENLA	NR3	NR3
PRGR_RANDY	NR3	NR3
Q6RKQ3_ONCMY	NR3	NR3
Q9IBD5_ANGJA	NR3	NR3

Q6A4C2_DICLA	NR3	NR3
Q8QGX5_ANGJA	NR3	NR3
Q8BG65_MOUSE	NR3	NR3
ESR1_RAT	NR3	NR3
Q9W6F4_HAPBU	NR3	NR3
Q6R7S4_SALSA	NR3	NR3
Q7T3U4_9TELE	NR3	NR3
Q762D6_CONMY	NR3	NR3
ESR2_MICUN	NR3	NR3
Q90WH6_CLAGA	NR3	NR3
Q90ZE6_SQUAC	NR3	NR3
ERR1_HUMAN	NR3	NR3
ERR2_HUMAN	NR3	NR3
Q5XTQ9_BRAFL	NR3	NR3
Q5XTR0_BRAFL	NR3	NR3
Q8WS79_DROME	NR3	NR3
Q6ZMM6_HUMAN	NR4	NR4
Q98TQ3_ORYLA	NR4	NR4
O97726_PIG	NR4	NR4
Q6INY4_XENLA	NR4	NR4
HR38_DROME	NR1	NR4
Q9U4L1_AEDAE	NR4	NR4
NHR6_CAEEL	NR4	NR4
Q9QWM1_RAT	NR5	NR5
P97782_MOUSE	NR5	NR5
Q8JH98_CARAU	NR5	NR5
Q8UV26_CLAGA	NR5	NR5
Q98ST8_BRARE	NR5	NR5
Q6W954_EPICO	NR5	NR5
FTZF1_DROME	NR5	NR5
Q9NB03_AEDAE	NR5	NR5
Q8WSJ3_MANSE	NR5	NR5
Q9Y0D1_METEN	NR5	NR5
Q9BPL0_SCHMA	NR3	NR5
FTF1B_DROME	NR3	NR5
NR6A1_MOUSE	NR6	NR6
NR6A1_HUMAN	NR6	NR6
Q66JK1_XENTR	NR6	NR6
P70033_XENLA	NR6	NR6
Q9PU65_BRARE	NR6	NR6
KNIR_DROVI	NR0	NR0

KNIR_DROME	NR0	NR0
KNRL_DROME	NR2	NR0
TRX_DROVI	NR0	NR0
TRX_DROME	NR0	NR0
EGON_DROME	NR0	NR0
ODR7_CAEEL	NR2	NR0
DAX1_MOUSE	NR3	NR0
Q8QGS4_RANRU	NR0	NR0
Q8AY13_ORENI	NR0	NR0
P97947_RAT	NR0	NR0
Q8AUM4_ORENI	NR0	NR0
KNIR_DROVI	NR	NR
KNIR_DROME	NR	NR
KNRL_DROME	NR	NR
TRX_DROVI	NR	NR
TRX_DROME	NR	NR
EGON_DROME	NR	NR
ODR7_CAEEL	NR	NR
DAX1_MOUSE	NR	NR
Q8QGS4_RANRU	NR	NR
Q8AY13_ORENI	NR	NR
P97947_RAT	NR	NR
Q8AUM4_ORENI	NR	NR
THB2_RAT	NR	NR
Q64895_9RETR	non-NR	NR
Q5RZV7_PSEAM	NR	NR
RRG_NOTVI	NR	NR
Q15156_HUMAN	NR	NR
Q86WD1_HUMAN	NR	NR
Q658K5_HUMAN	NR	NR
NR1D2_HUMAN	NR	NR
Q6IBU6_HUMAN	NR	NR
Q922A5_MOUSE	NR	NR
Q6VA69_AEDAE	NR	NR
Q6PAY0_XENLA	NR	NR
NR1H3_HUMAN	NR	NR
Q9PW01_PLEPL	NR	NR
Q9GPH1_CALVI	NR	NR
O97095_LOCMI	NR	NR
Q7T030_PLEPL	NR	NR
ECR_DROME	NR	NR

Q90WP6_SALSA	NR	NR
Q6GMI3_BRARE	NR	NR
Q8WSA1_BOMMO	NR	NR
Q7T031_PLEPL	NR	NR
O44338_9ACAR	NR	NR
NR1H4_MOUSE	NR	NR
E75C_DROME	non-NR	NR
Q6GND0_XENLA	NR	NR
ECR_BOMMO	NR	NR
Q8MX80_BRAFL	NR	NR
