# Supplementary material for: NR-2L: A Two-Level Predictor for Identifying Nuclear Receptor Subfamilies Based on Sequence-Derived Features
Source: PLoS One. 2011 Aug 15;6(8):e23505. doi: 10.1371/journal.pone.0023505 (PMC3156231; doi:10.1371/journal.pone.0023505)
Supplement: Supporting Information S4 — List of the results obtained by NR-2L on the 568 NRsand 500 non-NRs in the independent testing dataset ST (cf. Supporting Information S2), and the corresponding observed results as annotated in NucleaRDB or UniProt. (PDF) [file pone.0023505.s004.pdf]

Supporting Information S4. List of the results obtained by **NR-2L** on the 568 NRs and 500 non-NRs in the independent testing dataset $\mathbb{S}_T$ (cf. Supporting Information S2), and the corresponding observed results as annotated in NucleaRDB or UniProt.

I. Results of the 1st level prediction in identifying NRs or non-NRs

Protein code	Predicted	Observed
THA_CHICK	NR	NR
THA1_SHEEP	NR	NR
THB2_MOUSE	NR	NR
THB1_HUMAN	NR	NR
Q6F3J6_CONMY	NR	NR
THB1_RAT	NR	NR
THAA_XENLA	NR	NR
THB1_MOUSE	NR	NR
Q7T0K4_AMBME	NR	NR
THAB_XENLA	NR	NR
THA_RANCA	NR	NR
THA_SALSA	NR	NR
THB2_HUMAN	NR	NR
Q6F3J5_CONMY	NR	NR
THA_HIPHI	NR	NR
THA_NECMA	NR	NR
THA1_BRARE	NR	NR
Q766D2_ORYLA	NR	NR
Q5RZV6_PSEAM	NR	NR
THAA_PAROL	NR	NR
THBB_XENLA	NR	NR
THA_HUMAN	NR	NR
THA_PIG	NR	NR
THA_RAT	NR	NR
Q80Y90_MOUSE	NR	NR
THA_MOUSE	NR	NR
THAB_PAROL	NR	NR
Q800D8_SPAAU	NR	NR
THB_BRARE	NR	NR
THB_PAROL	NR	NR
ERBA_AVIER	NR	NR

Q6F3J8_CONMY	NR	NR
THB_RANCA	NR	NR
Q90Y21_SALSA	NR	NR
Q90Y22_SALSA	NR	NR
THBA_XENLA	NR	NR
THB_CHICK	NR	NR
THB_CAIMO	NR	NR
Q7T0K3_AMBME	NR	NR
Q6Y9T0_NECMA	NR	NR
Q766D1_ORYLA	NR	NR
P97513_MUSSP	NR	NR
RRA_MOUSE	NR	NR
Q6F3J7_CONMY	NR	NR
RRA_HUMAN	NR	NR
RRA_CHICK	NR	NR
RRG1_HUMAN	NR	NR
Q91YX2_MOUSE	NR	NR
RRG1_MOUSE	NR	NR
Q9I8T3_AMBME	NR	NR
RRB_HUMAN	NR	NR
RRB_CHICK	NR	NR
RRG2_HUMAN	NR	NR
RRB_COTJA	NR	NR
RRA_NOTVI	NR	NR
Q8VHB6_MESAU	NR	NR
Q5U645_HUMAN	NR	NR
RRG2_MOUSE	NR	NR
Q9I8T2_AMBME	NR	NR
Q9QWJ1_RAT	NR	NR
Q8VHB8_MESAU	NR	NR
Q8VHB7_MESAU	NR	NR
RRG_XENLA	NR	NR
PPAT_PIG	NR	NR
Q8HZ56_SHEEP	NR	NR
PPAT_BOVIN	NR	NR
PPAT_MACMU	NR	NR
RRA_FUGRU	NR	NR
Q95J17_MACFA	NR	NR
Q8MHZ0_MACFA	NR	NR
Q95KZ8_MACFA	NR	NR
PPAT_HUMAN	NR	NR

RRB_MOUSE	NR	NR
Q866S0_CAPHI	NR	NR
Q5RFE5_PONPY	NR	NR
PPAT_RABIT	NR	NR
Q92019_XENLA	NR	NR
Q8JHU6_ANAPL	NR	NR
Q8HXA4_RABIT	NR	NR
PPAT_RAT	NR	NR
PPAT_CRIGR	NR	NR
PPAT_MOUSE	NR	NR
Q7ZTI3_BRARE	NR	NR
Q90272_BRARE	NR	NR
Q91391_BRARE	NR	NR
Q90271_BRARE	NR	NR
RRG_BRARE	NR	NR
Q9I878_CHICK	NR	NR
RRA_XENLA	NR	NR
Q98SF8_XENLA	NR	NR
Q6PA31_XENLA	NR	NR
NR1D2_MOUSE	NR	NR
Q8C598_MOUSE	NR	NR
Q7TNH1_MESAU	NR	NR
PPAS_MOUSE	NR	NR
PPAS_HUMAN	NR	NR
Q99ND3_RAT	NR	NR
PPAR_RAT	NR	NR
PPAR_CANFA	NR	NR
NR1D2_RAT	NR	NR
Q6I9S1_HUMAN	NR	NR
PPAR_HUMAN	NR	NR
PPAR_MOUSE	NR	NR
Q62879_RAT	NR	NR
Q9I8W4_CHICK	NR	NR
NR1H2_HUMAN	NR	NR
PPAT_XENLA	NR	NR
Q8QGC1_9AVES	NR	NR
Q9QWI0_RAT	NR	NR
Q866Q2_PIG	NR	NR
Q9I8W3_CHICK	NR	NR
Q6P6S7_RAT	NR	NR
Q9N2H4_RABIT	NR	NR

PPAR_PHACI	NR	NR
Q8BP65_MOUSE	NR	NR
Q90970_CHICK	NR	NR
NR1H3_RAT	NR	NR
PPAR_CAVPO	NR	NR
NR1H3_MOUSE	NR	NR
Q91X41_MOUSE	NR	NR
Q9U3Y4_AEDAL	NR	NR
ECR_LUCCU	NR	NR
NR1H2_RAT	NR	NR
PPAR_XENLA	NR	NR
Q6AXA9_XENLA	NR	NR
Q8IW13_HUMAN	NR	NR
Q5U0N9_HUMAN	NR	NR
NR1D1_HUMAN	NR	NR
NR1H2_MOUSE	NR	NR
ECR_AEDAE	NR	NR
Q8MYA6_9NEOP	NR	NR
Q7T029_PLEPL	NR	NR
Q9W712_PLAFE	NR	NR
Q9VVM9_DROME	NR	NR
Q8MYA7_9NEOP	NR	NR
O76827_CERCA	NR	NR
Q8IQS3_DROME	non-NR	NR
E75A_DROME	non-NR	NR
Q9I8F6_SALSA	NR	NR
ECR_MANSE	NR	NR
RORA_MOUSE	NR	NR
Q6GMA2_XENLA	NR	NR
O77255_CHOFU	NR	NR
Q6RVD3_PLOIN	NR	NR
Q8JHU1_CHICK	NR	NR
Q5RGZ2_BRARE	NR	NR
O77240_CHOFU	NR	NR
Q8JIT8_SALSA	NR	NR
Q8AXU8_CHICK	NR	NR
O44337_9ACAR	NR	NR
Q68CY8_HUMAN	NR	NR
Q5XI75_RAT	NR	NR
NR1H4_RAT	NR	NR
ECR_HELVI	NR	NR

O44336_9ACAR	NR	NR
O02035_TENMO	NR	NR
Q8K476_MESAU	NR	NR
Q8K473_MESAU	NR	NR
Q8K474_MESAU	NR	NR
Q8K475_MESAU	NR	NR
HR3_GALME	NR	NR
ECR_CHITE	NR	NR
Q9BMC6_HELAM	NR	NR
E75_GALME	NR	NR
Q7Z2W0_HUMAN	NR	NR
NR1H4_HUMAN	NR	NR
Q8SPF5_RABIT	NR	NR
VDR_CHICK	NR	NR
Q8WSA2_BOMMO	NR	NR
Q9U5G4_BOMMO	NR	NR
E75_CHOFU	NR	NR
E75_MANSE	NR	NR
Q8JHU2_CHICK	NR	NR
Q8JJ28_XENLA	NR	NR
Q6P7H5_XENLA	NR	NR
VDR_COTJA	NR	NR
Q27547_CHOFU	NR	NR
NR1D1_RAT	NR	NR
HR3_DROME	NR	NR
Q6DGW7_BRARE	NR	NR
Q95P94_BOMMO	NR	NR
PXR_HUMAN	NR	NR
Q811X2_MOUSE	NR	NR
VDR_SAGOE	NR	NR
E75B_DROME	NR	NR
Q8IQS2_DROME	NR	NR
VDR_HUMAN	NR	NR
Q9I8F7_SALSA	NR	NR
Q8SQ01_MACMU	NR	NR
Q9IB74_PAROL	NR	NR
VDR_RAT	NR	NR
VDR_BOVIN	NR	NR
Q922X0_MOUSE	NR	NR
VDR_MOUSE	NR	NR
PXR_RAT	NR	NR

Q5RAP4_PONPY	NR	NR
PPAS_XENLA	NR	NR
Q9IB73_PAROL	NR	NR
Q62702_RAT	NR	NR
PXR_MOUSE	NR	NR
RORG_MOUSE	NR	NR
Q9NH86_AEDAE	NR	NR
VDR_XENLA	NR	NR
Q98934_CHICK	NR	NR
RORB_MOUSE	NR	NR
RORB_RAT	NR	NR
RORB_HUMAN	NR	NR
Q7ZU39_BRARE	NR	NR
Q91839_XENLA	NR	NR
Q8AVZ2_XENLA	NR	NR
Q9TU02_RABIT	NR	NR
Q9U5G3_BOMMO	NR	NR
NHR23_CAEEL	NR	NR
Q6Q2C9_PLOIN	NR	NR
Q8SXA4_DROME	NR	NR
Q5REL4_PONPY	NR	NR
Q5R8V3_PONPY	NR	NR
NR1I3_MOUSE	NR	NR
Q811W9_MOUSE	NR	NR
NR1I3_HUMAN	NR	NR
NR1I3_MACMU	NR	NR
CNR14_CAEEL	NR	NR
Q9U3F1_CAEEL	NR	NR
NR1I3_PHOSI	NR	NR
NR1I3_CALUR	NR	NR
Q811X1_MOUSE	NR	NR
Q811X0_MOUSE	NR	NR
Q6GZ84_HUMAN	NR	NR
Q6GZ68_HUMAN	NR	NR
Q8IQS1_DROME	NR	NR
Q76FN2_RAT	NR	NR
RXRA_RAT	NR	NR
RXRA_MOUSE	NR	NR
RXRA_HUMAN	NR	NR
Q5TJF7_CANFA	NR	NR
RXRB_MOUSE	NR	NR

Q6LC96_MOUSE	NR	NR
Q6MGB3_RAT	NR	NR
RXRG_MOUSE	NR	NR
RXRG_HUMAN	NR	NR
Q5REL6_PONPY	NR	NR
RXRA_XENLA	NR	NR
7UP1_DROME	NR	NR
RXRG_CHICK	NR	NR
RXRB_BRARE	NR	NR
Q6INZ0_XENLA	NR	NR
Q91613_XENLA	NR	NR
RXRA_BRARE	NR	NR
Q86GV5_BRAFL	NR	NR
COT1_MOUSE	NR	NR
COT1_HUMAN	NR	NR
COT1_BOVIN	NR	NR
Q62681_RAT	NR	NR
Q6DHP9_BRARE	NR	NR
COT2_BOVIN	NR	NR
COT2_HUMAN	NR	NR
COT2_MOUSE	NR	NR
COT2_RAT	NR	NR
NR2F1_BRARE	NR	NR
Q91430_BRARE	NR	NR
Q6PHF2_BRARE	NR	NR
Q9W745_XENLA	NR	NR
Q91840_XENLA	NR	NR
Q8T5C6_BIOGL	NR	NR
COT2_CHICK	NR	NR
Q6DCH6_XENLA	NR	NR
Q8VCR0_MOUSE	NR	NR
Q7ZXI7_XENLA	NR	NR
Q9PS79_9PIPI	NR	NR
Q8UUM6_ORYLA	NR	NR
RXRD_BRARE	NR	NR
Q66TQ0_9CAEN	NR	NR
Q6V7U7_LOCMI	NR	NR
Q66J63_XENLA	NR	NR
Q91720_XENLA	NR	NR
Q5RCZ5_PONPY	NR	NR
Q95K90_MACFA	NR	NR

RXRG_BRARE	NR	NR
Q15625_HUMAN	NR	NR
Q9VLI7_DROME	NR	NR
NR2F5_BRARE	NR	NR
Q9GSG8_AEDAE	NR	NR
USP_MANSE	NR	NR
Q6PH18_BRARE	NR	NR
TR4_HUMAN	NR	NR
TR4_MOUSE	NR	NR
Q6P0E6_BRARE	NR	NR
TR4_RAT	NR	NR
Q9GSG7_AEDAE	NR	NR
Q9U7D9_LOCMI	NR	NR
USP_BOMMO	NR	NR
Q8VIJ3_MOUSE	NR	NR
Q8VIJ4_RAT	NR	NR
HNF4_DROME	NR	NR
Q62152_MOUSE	NR	NR
Q6GL59_XENTR	NR	NR
Q7T0T7_XENLA	NR	NR
Q922G8_MOUSE	NR	NR
Q9NFY1_TENMO	NR	NR
EAR2_MOUSE	NR	NR
Q6P117_BRARE	NR	NR
EAR2_RAT	NR	NR
Q5QPB8_HUMAN	NR	NR
Q962I5_LUCCU	NR	NR
Q6P115_BRARE	NR	NR
O61449_9ACAR	NR	NR
O61448_9ACAR	NR	NR
HNF4A_MOUSE	NR	NR
HNF4A_RAT	NR	NR
Q6PHH5_BRARE	NR	NR
Q9JJI9_TAMSI	NR	NR
Q7YRQ5_BOVIN	NR	NR
HNF4A_HUMAN	NR	NR
Q5RH32_BRARE	NR	NR
Q7SZG3_FUGRU	NR	NR
Q8AVK3_XENLA	NR	NR
Q5QPB7_HUMAN	NR	NR
Q6IVK1_PLOIN	NR	NR

Q95WF7_ACRMI	NR	NR
Q6LDB2_9MURI	NR	NR
Q8AXB6_BRARE	NR	NR
Q8I1M8_9NEOP	NR	NR
HNF4A_XENLA	NR	NR
Q8JID3_CHICK	NR	NR
Q6B4V6_HUMAN	NR	NR
Q6B4V7_HUMAN	NR	NR
Q6B4V5_HUMAN	NR	NR
HNF4B_XENLA	NR	NR
O77100_AEDAE	NR	NR
Q7YRQ4_BOVIN	NR	NR
TR2_HUMAN	NR	NR
Q15626_HUMAN	NR	NR
NR2E3_HUMAN	NR	NR
Q6DEH0_BRARE	NR	NR
O46175_BOMMO	NR	NR
NR2E1_ORYLA	NR	NR
Q8JHW6_FUGRU	NR	NR
Q9Y1J4_SCHMA	NR	NR
NR2E1_MOUSE	NR	NR
HNF4G_MOUSE	NR	NR
Q7Z2V9_HUMAN	NR	NR
NR2E1_CHICK	NR	NR
NR2E1_HUMAN	NR	NR
HNF4G_HUMAN	NR	NR
Q8MX79_BRAFL	NR	NR
Q90WV5_CHICK	NR	NR
NR2E1_XENLA	NR	NR
Q9VP28_DROME	NR	NR
HR78_DROME	NR	NR
TLL_DROVI	NR	NR
Q9VML1_DROME	NR	NR
Q5U7E0_MUSDO	NR	NR
Q86PK5_CAEEL	NR	NR
Q61WY2_CAEBR	NR	NR
Q8IVZ9_HUMAN	NR	NR
Q688B5_CAEEL	NR	NR
O77101_AEDAE	NR	NR
Q86PK4_CAEEL	NR	NR
GCR_SAISC	NR	NR

GCR_HUMAN	NR	NR
GCR_SAIBB	NR	NR
GCR_AOTNA	NR	NR
Q6N0A4_HUMAN	NR	NR
GCR_SAGOE	NR	NR
Q6XLJ0_CALJA	NR	NR
Q5R7M7_PONPY	NR	NR
Q5R9P5_PONPY	NR	NR
ANDR_PANTR	NR	NR
ANDR_PAPHA	NR	NR
ANDR_MACFA	NR	NR
ANDR_MACMU	NR	NR
Q9UN21_HUMAN	NR	NR
Q5S4M0_PIG	NR	NR
GCR_MOUSE	NR	NR
ANDR_EULFC	NR	NR
GCR_TUPGB	NR	NR
ANDR_PIG	NR	NR
GCR_CAVPO	NR	NR
PRGR_HUMAN	NR	NR
ANDR_CANFA	NR	NR
ANDR_CROCR	NR	NR
GCR_RABIT	NR	NR
ANDR_RAT	NR	NR
ANDR_MOUSE	NR	NR
PRGR_RABIT	NR	NR
MCR_SAISC	NR	NR
Q6XLI9_CALJA	NR	NR
MCR_HUMAN	NR	NR
MCR_RAT	NR	NR
MCR_MOUSE	NR	NR
PRGR_RAT	NR	NR
PRGR_MOUSE	NR	NR
MCR_TUPGB	NR	NR
Q13771_HUMAN	NR	NR
Q6DDL0_XENLA	NR	NR
GCR_XENLA	NR	NR
Q66J29_XENLA	NR	NR
Q8NG42_HUMAN	NR	NR
GCR_PAROL	NR	NR
Q8JJ91_HAPBU	NR	NR

Q8JJ90_HAPBU	NR	NR
Q5WP01_ONCMY	NR	NR
Q9I8F5_9TELE	NR	NR
Q8UWB7_ORENI	NR	NR
O93245_ONCMY	NR	NR
Q8QFV7_HAPBU	NR	NR
O93497_PAGMA	NR	NR
ANDR_RANCA	NR	NR
Q66VR6_MICUN	NR	NR
Q60I32_GAMAF	NR	NR
Q9PWG5_ANGJA	NR	NR
Q6IVJ3_DICLA	NR	NR
Q8NG44_HUMAN	NR	NR
Q801Z1_GASAC	NR	NR
Q8NG45_HUMAN	NR	NR
Q8NG43_HUMAN	NR	NR
ESR1_PIG	NR	NR
Q9DDZ4_MICSA	NR	NR
ESR1_BOVIN	NR	NR
ESR1_HUMAN	NR	NR
ESR1_MOUSE	NR	NR
Q7SZI0_FUNHE	NR	NR
ESR1_HORSE	NR	NR
ESR2_ONCMY	NR	NR
ESR1_MESAU	NR	NR
ESR1_ORYLA	NR	NR
Q765N7_ALLMI	NR	NR
Q8AYH0_COTJA	NR	NR
ESR1_CHICK	NR	NR
Q8UWB0_CAICR	NR	NR
Q6W5G7_XENLA	NR	NR
ESR1_POEGU	NR	NR
Q90WS8_BRARE	NR	NR
ESR1_XENLA	NR	NR
ESRB1_CARAU	NR	NR
Q7ZU32_BRARE	NR	NR
Q8AV62_BRARE	NR	NR
Q60GT9_GAMAF	NR	NR
Q8UWA9_CNEUN	NR	NR
ESR2_STUVU	NR	NR
ESR2_MOUSE	NR	NR

ESR2_RAT	NR	NR
Q8JJB9_CYPCA	NR	NR
ESR2_HUMAN	NR	NR
ESR1_ONCMY	NR	NR
Q8UWB8_ORENI	NR	NR
Q98SM7_BRARE	NR	NR
ESR2_BOVIN	NR	NR
Q5PR29_BRARE	NR	NR
Q6XSH2_MICSA	NR	NR
ESR2_PIG	NR	NR
ESR2_SHEEP	NR	NR
Q8QHK9_PAROL	NR	NR
Q98SM8_BRARE	NR	NR
ESR2_ANGJA	NR	NR
Q90WS9_BRARE	NR	NR
ESR2_CALJA	NR	NR
ESR1_PAGMA	NR	NR
Q6H9M5_SPAAU	NR	NR
Q804Q6_ACASC	NR	NR
Q6H9M4_SPAAU	NR	NR
Q7T3U5_9TELE	NR	NR
ESRB2_CARAU	NR	NR
ESR1_SPAAU	NR	NR
Q8UW75_ORYLA	NR	NR
ESR3_MICUN	NR	NR
Q7T2K7_9LABR	NR	NR
Q6XSH1_MICSA	NR	NR
Q9DEV4_XENLA	NR	NR
ESR2_ORENI	NR	NR
ESR2_SPAAU	NR	NR
Q7T2K8_9LABR	NR	NR
Q6TGB3_HAPBU	NR	NR
Q800Q2_ZOAVI	NR	NR
ESR1_ORENI	NR	NR
Q804Q7_ACASC	NR	NR
Q8QHL0_PAROL	NR	NR
ESR1_ICTPU	NR	NR
Q6W5G9_XENTR	NR	NR
ESR1_OREAU	NR	NR
Q69F36_CARAU	NR	NR
Q95MF0_MACAR	NR	NR

ESR1_BRARE	NR	NR
Q5XXP1_9TELE	NR	NR
ESR2_ICTPU	NR	NR
Q90WV1_CARAU	NR	NR
Q95ME9_CALJA	NR	NR
Q91Z86_MOUSE	NR	NR
ESR2_CHICK	NR	NR
ESR2_COTJA	NR	NR
ERR3_HUMAN	NR	NR
ERR3_MOUSE	NR	NR
ERR1_MOUSE	NR	NR
Q5UKY7_CHICK	NR	NR
ERR3_PONPY	NR	NR
Q6W5G5_XENLA	NR	NR
Q6W5G6_XENLA	NR	NR
ERR2_RAT	NR	NR
Q6Q6F4_BRARE	NR	NR
Q8C7A6_MOUSE	NR	NR
ERR2_MOUSE	NR	NR
Q8CCV5_MOUSE	NR	NR
Q6Q6F6_BRARE	NR	NR
Q6QMY5_CANFA	NR	NR
Q9VSE9_DROME	NR	NR
Q6AX97_XENLA	NR	NR
NR4A1_HUMAN	NR	NR
Q6IBU8_HUMAN	NR	NR
Q8N3V2_HUMAN	NR	NR
Q5RBB0_PONPY	NR	NR
NR4A1_CANFA	NR	NR
NR4A1_MOUSE	NR	NR
NR4A2_MOUSE	NR	NR
NR4A2_HUMAN	NR	NR
NR4A2_RAT	NR	NR
Q5R5Y4_PONPY	NR	NR
NR4A1_RAT	NR	NR
NR4A3_MOUSE	NR	NR
NR4A3_RAT	NR	NR
Q6NXU0_HUMAN	NR	NR
NR4A3_HUMAN	NR	NR
NR4A2_XENLA	NR	NR
Q7T0V3_XENLA	NR	NR

Q6DH08_BRARE	NR	NR
Q6GMG3_BRARE	NR	NR
Q8INU7_DROME	NR	NR
Q9R1W4_MOUSE	NR	NR
O97727_PIG	NR	NR
Q61JK3_CAEBR	NR	NR
NR5A2_HUMAN	NR	NR
Q9UEC0_HUMAN	NR	NR
Q9QWM0_RAT	NR	NR
NR5A2_MOUSE	NR	NR
NR5A2_CHICK	NR	NR
Q5XGE7_XENTR	NR	NR
Q9IB82_RANRU	NR	NR
Q91544_XENLA	NR	NR
Q90YL6_BRARE	NR	NR
Q9GKL1_HORSE	NR	NR
O42186_BRARE	NR	NR
Q9IB81_RANRU	NR	NR
Q9PWI7_CHICK	NR	NR
STF1_RAT	NR	NR
Q812G5_MOUSE	NR	NR
O42102_CHICK	NR	NR
Q90XC4_POEGU	NR	NR
STF1_BOVIN	NR	NR
STF1_MOUSE	NR	NR
Q9YI54_TRASC	NR	NR
STF1_PIG	NR	NR
STF1_HUMAN	NR	NR
STF1_MACEU	NR	NR
STF1_HORSE	NR	NR
Q9YI95_RANRU	NR	NR
Q6QHU4_PLEWA	NR	NR
O93258_ORYLA	NR	NR
Q7ZT68_ORENI	NR	NR
Q800U8_BRARE	NR	NR
Q8UV27_CLAGA	NR	NR
Q9IAI9_BRARE	NR	NR
FTZF1_BOMMO	NR	NR
Q91601_XENLA	NR	NR
DAX1_PIG	NR	NR
DAX1_RAT	NR	NR

DAX1_HUMAN	NR	NR
Q9PTE9_CHICK	NR	NR
SHP_MOUSE	NR	NR
SHP_HUMAN	NR	NR
E4YGX1_OIKDI	non-NR	non-NR
E4YGY5_OIKDI	non-NR	non-NR
Q9I7K4_DROME	NR	non-NR
NASP_DROME	NR	non-NR
E4YGY7_OIKDI	non-NR	non-NR
A8PAW1_BRUMA	non-NR	non-NR
E4YGY9_OIKDI	non-NR	non-NR
E4YGZ7_OIKDI	non-NR	non-NR
E4YGZ9_OIKDI	non-NR	non-NR
E4YH01_OIKDI	non-NR	non-NR
E4YH05_OIKDI	non-NR	non-NR
E4YH09_OIKDI	non-NR	non-NR
E4YH11_OIKDI	non-NR	non-NR
E4YH03_OIKDI	non-NR	non-NR
E4YH14_OIKDI	non-NR	non-NR
E4YH19_OIKDI	non-NR	non-NR
E4YH20_OIKDI	non-NR	non-NR
E4YH22_OIKDI	non-NR	non-NR
E4YH28_OIKDI	non-NR	non-NR
E4YH30_OIKDI	non-NR	non-NR
E4YH32_OIKDI	non-NR	non-NR
Q9I7K8_DROME	NR	non-NR
Q9I7L0_DROME	NR	non-NR
E4YH33_OIKDI	non-NR	non-NR
E4YH37_OIKDI	non-NR	non-NR
E4YH39_OIKDI	non-NR	non-NR
E4YH44_OIKDI	non-NR	non-NR
E4YH50_OIKDI	non-NR	non-NR
E4YH56_OIKDI	non-NR	non-NR
E4YH66_OIKDI	non-NR	non-NR
E4YH68_OIKDI	non-NR	non-NR
E4YH74_OIKDI	non-NR	non-NR
E4YH77_OIKDI	non-NR	non-NR
E4YH78_OIKDI	non-NR	non-NR
E4YH85_OIKDI	non-NR	non-NR
E4YH87_OIKDI	non-NR	non-NR
E4YH88_OIKDI	non-NR	non-NR

E4YH89_OIKDI	non-NR	non-NR
Q9I7L2_DROME	NR	non-NR
E4YGN5_OIKDI	NR	non-NR
E4YH91_OIKDI	non-NR	non-NR
E4YH99_OIKDI	non-NR	non-NR
E4YHA2_OIKDI	non-NR	non-NR
E4YHA7_OIKDI	non-NR	non-NR
E4YHA8_OIKDI	non-NR	non-NR
E4YHA9_OIKDI	non-NR	non-NR
E4YHB5_OIKDI	non-NR	non-NR
E4YHB8_OIKDI	non-NR	non-NR
E4YHB9_OIKDI	non-NR	non-NR
E4YHC1_OIKDI	non-NR	non-NR
E4YHC5_OIKDI	non-NR	non-NR
E4YHC8_OIKDI	non-NR	non-NR
E4YHD2_OIKDI	non-NR	non-NR
E4YHD4_OIKDI	non-NR	non-NR
E4YHD5_OIKDI	non-NR	non-NR
E4YHE4_OIKDI	non-NR	non-NR
E4YHE5_OIKDI	non-NR	non-NR
E4YHE8_OIKDI	non-NR	non-NR
E4YGT7_OIKDI	NR	non-NR
E4YHG3_OIKDI	non-NR	non-NR
E4YHH4_OIKDI	non-NR	non-NR
E4YHH5_OIKDI	non-NR	non-NR
E4YHI0_OIKDI	non-NR	non-NR
E4YHI4_OIKDI	non-NR	non-NR
E4YHI7_OIKDI	non-NR	non-NR
E4YHI8_OIKDI	non-NR	non-NR
E4YHJ2_OIKDI	non-NR	non-NR
E4YHJ3_OIKDI	non-NR	non-NR
E4YHJ4_OIKDI	non-NR	non-NR
E4YHJ5_OIKDI	non-NR	non-NR
E4YHJ7_OIKDI	non-NR	non-NR
E4YHK3_OIKDI	non-NR	non-NR
E4YHK8_OIKDI	non-NR	non-NR
E4YHK9_OIKDI	non-NR	non-NR
E4YHL0_OIKDI	non-NR	non-NR
E4YHL1_OIKDI	non-NR	non-NR
E4YHM0_OIKDI	non-NR	non-NR
E4YHM6_OIKDI	non-NR	non-NR

E4YHM8_OIKDI	non-NR	non-NR
E4YHN0_OIKDI	non-NR	non-NR
E4YHN7_OIKDI	non-NR	non-NR
E4YHN8_OIKDI	non-NR	non-NR
E4YHN9_OIKDI	non-NR	non-NR
E4YHP1_OIKDI	non-NR	non-NR
E4YHP4_OIKDI	non-NR	non-NR
E4YHQ5_OIKDI	non-NR	non-NR
E4YHQ6_OIKDI	non-NR	non-NR
E4YHR1_OIKDI	non-NR	non-NR
E4YHR6_OIKDI	non-NR	non-NR
E4YHR7_OIKDI	non-NR	non-NR
E4YHR8_OIKDI	non-NR	non-NR
E4YHS5_OIKDI	non-NR	non-NR
E4YHS7_OIKDI	non-NR	non-NR
E4YHS9_OIKDI	non-NR	non-NR
E4YHT0_OIKDI	non-NR	non-NR
E4YHT6_OIKDI	non-NR	non-NR
E4YHT9_OIKDI	non-NR	non-NR
E4YHU7_OIKDI	non-NR	non-NR
E4YHU9_OIKDI	non-NR	non-NR
E4YHV3_OIKDI	non-NR	non-NR
E4YHW4_OIKDI	non-NR	non-NR
E4YHW8_OIKDI	non-NR	non-NR
E4YHX7_OIKDI	non-NR	non-NR
E4YHY0_OIKDI	non-NR	non-NR
E4YHY3_OIKDI	non-NR	non-NR
E4YHY5_OIKDI	non-NR	non-NR
E4YHZ7_OIKDI	non-NR	non-NR
E4YHZ8_OIKDI	non-NR	non-NR
E4YI01_OIKDI	non-NR	non-NR
E4YI03_OIKDI	non-NR	non-NR
E4YI10_OIKDI	non-NR	non-NR
E4YI12_OIKDI	non-NR	non-NR
E4YI21_OIKDI	non-NR	non-NR
E4YI25_OIKDI	non-NR	non-NR
E4YGV5_OIKDI	non-NR	non-NR
E4YGY3_OIKDI	non-NR	non-NR
E4YI28_OIKDI	non-NR	non-NR
E4YI31_OIKDI	non-NR	non-NR
E4YI36_OIKDI	non-NR	non-NR

E4YI40_OIKDI	non-NR	non-NR
E4YI41_OIKDI	non-NR	non-NR
E4YI43_OIKDI	non-NR	non-NR
E4YI44_OIKDI	non-NR	non-NR
E4YI46_OIKDI	non-NR	non-NR
E4YI47_OIKDI	non-NR	non-NR
E4YI56_OIKDI	non-NR	non-NR
E4YI59_OIKDI	non-NR	non-NR
E4YI61_OIKDI	non-NR	non-NR
E4YI65_OIKDI	non-NR	non-NR
E4YI76_OIKDI	non-NR	non-NR
E4YI85_OIKDI	non-NR	non-NR
E4YH76_OIKDI	non-NR	non-NR
E4YH98_OIKDI	non-NR	non-NR
E4YI86_OIKDI	non-NR	non-NR
E4YI92_OIKDI	non-NR	non-NR
E4YI93_OIKDI	non-NR	non-NR
E4YI94_OIKDI	non-NR	non-NR
E4YI98_OIKDI	non-NR	non-NR
E4YI99_OIKDI	non-NR	non-NR
E4YIA0_OIKDI	non-NR	non-NR
E4YIA3_OIKDI	non-NR	non-NR
E4YIA7_OIKDI	non-NR	non-NR
E4YIA8_OIKDI	non-NR	non-NR
E4YIB1_OIKDI	non-NR	non-NR
E4YIB3_OIKDI	non-NR	non-NR
E4YIB4_OIKDI	non-NR	non-NR
E4YIB8_OIKDI	non-NR	non-NR
E4YIC3_OIKDI	non-NR	non-NR
E4YID1_OIKDI	non-NR	non-NR
E4YID3_OIKDI	non-NR	non-NR
E4YID7_OIKDI	non-NR	non-NR
E4YIE0_OIKDI	non-NR	non-NR
E4YIE1_OIKDI	non-NR	non-NR
E4YIE3_OIKDI	non-NR	non-NR
E4YIE5_OIKDI	non-NR	non-NR
E4YIE8_OIKDI	non-NR	non-NR
E4YIE9_OIKDI	non-NR	non-NR
E4YIF9_OIKDI	non-NR	non-NR
E4YIG2_OIKDI	non-NR	non-NR
E4YIG3_OIKDI	non-NR	non-NR

E4YIG4_OIKDI	non-NR	non-NR
E4YIG7_OIKDI	non-NR	non-NR
E4YIH3_OIKDI	non-NR	non-NR
E4YIH5_OIKDI	non-NR	non-NR
E4YIH6_OIKDI	non-NR	non-NR
E4YIH9_OIKDI	non-NR	non-NR
E4YII0_OIKDI	non-NR	non-NR
Q9I7L7_DROME	non-NR	non-NR
Q9I7M1_DROME	non-NR	non-NR
Q9I7M8_DROME	non-NR	non-NR
Q9I7N3_DROME	non-NR	non-NR
Q9I7P6_DROME	non-NR	non-NR
Q9I7Q5_DROME	non-NR	non-NR
Q9I7Q9_DROME	non-NR	non-NR
Q9I7R0_DROME	non-NR	non-NR
Q9I7R3_DROME	non-NR	non-NR
Q9I7S4_DROME	non-NR	non-NR
PUR6_DROME	non-NR	non-NR
Q9I7T3_DROME	non-NR	non-NR
E4YGN2_OIKDI	non-NR	non-NR
Q9I7U1_DROME	non-NR	non-NR
TITIN_DROME	non-NR	non-NR
E4YII7_OIKDI	non-NR	non-NR
E4YIJ5_OIKDI	non-NR	non-NR
JUPIT_DROME	NR	non-NR
Q9I7K0-3	NR	non-NR
E4YIJ6_OIKDI	non-NR	non-NR
E4YIK1_OIKDI	non-NR	non-NR
E4YIK3_OIKDI	non-NR	non-NR
E4YIK7_OIKDI	non-NR	non-NR
E4YIL1_OIKDI	non-NR	non-NR
E4YIL8_OIKDI	non-NR	non-NR
E4YIL9_OIKDI	non-NR	non-NR
E4YIM2_OIKDI	non-NR	non-NR
E4YIM9_OIKDI	non-NR	non-NR
E4YIN2_OIKDI	non-NR	non-NR
E4YIP1_OIKDI	non-NR	non-NR
E4YIP7_OIKDI	non-NR	non-NR
E4YIP8_OIKDI	non-NR	non-NR
E4YIP9_OIKDI	non-NR	non-NR
E4YIQ4_OIKDI	non-NR	non-NR

E4YIQ7_OIKDI	non-NR	non-NR
E4YIQ8_OIKDI	non-NR	non-NR
E4YH58_OIKDI	non-NR	non-NR
E4YIQ9_OIKDI	non-NR	non-NR
E4YIR1_OIKDI	non-NR	non-NR
E4YIR8_OIKDI	non-NR	non-NR
E4YIR9_OIKDI	non-NR	non-NR
E4YIT1_OIKDI	non-NR	non-NR
E4YIU6_OIKDI	non-NR	non-NR
E4YIU9_OIKDI	non-NR	non-NR
E4YIV1_OIKDI	non-NR	non-NR
E4YIV7_OIKDI	non-NR	non-NR
E4YIV9_OIKDI	non-NR	non-NR
E4YIW0_OIKDI	non-NR	non-NR
E4YIW2_OIKDI	non-NR	non-NR
E4YIW3_OIKDI	non-NR	non-NR
E4YIW4_OIKDI	non-NR	non-NR
E4YIW8_OIKDI	non-NR	non-NR
E4YIX0_OIKDI	non-NR	non-NR
E4YIX9_OIKDI	non-NR	non-NR
E4YIY0_OIKDI	non-NR	non-NR
E4YIY2_OIKDI	non-NR	non-NR
E4YIY3_OIKDI	non-NR	non-NR
E4YIZ2_OIKDI	non-NR	non-NR
E4YIZ6_OIKDI	non-NR	non-NR
E4YIZ9_OIKDI	non-NR	non-NR
E4YJ07_OIKDI	non-NR	non-NR
E4YJ09_OIKDI	non-NR	non-NR
E4YJ10_OIKDI	non-NR	non-NR
E4YJ14_OIKDI	non-NR	non-NR
E4YJ15_OIKDI	non-NR	non-NR
E4YJ17_OIKDI	non-NR	non-NR
E4YJ21_OIKDI	non-NR	non-NR
E4YJ26_OIKDI	non-NR	non-NR
E4YJ27_OIKDI	non-NR	non-NR
E4YJ28_OIKDI	non-NR	non-NR
E4YJ30_OIKDI	non-NR	non-NR
E4YJ32_OIKDI	non-NR	non-NR
E4YJ34_OIKDI	non-NR	non-NR
E4YJ37_OIKDI	non-NR	non-NR
E4YJ38_OIKDI	non-NR	non-NR

E4YJ43_OIKDI	non-NR	non-NR
E4YJ46_OIKDI	non-NR	non-NR
E4YJ50_OIKDI	non-NR	non-NR
E4YJ52_OIKDI	non-NR	non-NR
E4YJ56_OIKDI	non-NR	non-NR
E4YJ60_OIKDI	non-NR	non-NR
E4YJ61_OIKDI	non-NR	non-NR
E4YJ62_OIKDI	non-NR	non-NR
E4YJ63_OIKDI	non-NR	non-NR
E4YJ66_OIKDI	non-NR	non-NR
E4YJ67_OIKDI	non-NR	non-NR
E4YJ68_OIKDI	non-NR	non-NR
E4YJ70_OIKDI	non-NR	non-NR
Q9I7L3_DROME	NR	non-NR
E4YJ71_OIKDI	non-NR	non-NR
E4YJ73_OIKDI	non-NR	non-NR
E4YH51_OIKDI	non-NR	non-NR
E4YJ75_OIKDI	non-NR	non-NR
E4YJ82_OIKDI	non-NR	non-NR
E4YJ86_OIKDI	non-NR	non-NR
E4YJ93_OIKDI	non-NR	non-NR
E4YJA0_OIKDI	non-NR	non-NR
E4YJA1_OIKDI	non-NR	non-NR
E4YJA5_OIKDI	non-NR	non-NR
E4YJA7_OIKDI	non-NR	non-NR
E4YJA8_OIKDI	non-NR	non-NR
E4YJB2_OIKDI	non-NR	non-NR
E4YJB6_OIKDI	non-NR	non-NR
E4YJC2_OIKDI	non-NR	non-NR
E4YJC3_OIKDI	non-NR	non-NR
E4YJC5_OIKDI	non-NR	non-NR
E4YJD4_OIKDI	non-NR	non-NR
E4YJD7_OIKDI	non-NR	non-NR
E4YJD8_OIKDI	non-NR	non-NR
E4YJE2_OIKDI	non-NR	non-NR
E4YJE4_OIKDI	non-NR	non-NR
E4YJE6_OIKDI	non-NR	non-NR
E4YJE8_OIKDI	non-NR	non-NR
E4YJF3_OIKDI	non-NR	non-NR
A8PAW9_BRUMA	non-NR	non-NR
A8PAX6_BRUMA	non-NR	non-NR

E4YJF5_OIKDI	non-NR	non-NR
E4YJF6_OIKDI	non-NR	non-NR
E4YJF7_OIKDI	non-NR	non-NR
E4YJG1_OIKDI	non-NR	non-NR
E4YJG2_OIKDI	non-NR	non-NR
E4YJG8_OIKDI	non-NR	non-NR
E4YJH3_OIKDI	non-NR	non-NR
E4YJH4_OIKDI	non-NR	non-NR
E4YJI6_OIKDI	non-NR	non-NR
E4YJI8_OIKDI	non-NR	non-NR
E4YJJ2_OIKDI	non-NR	non-NR
E4YJJ8_OIKDI	non-NR	non-NR
E4YJJ9_OIKDI	non-NR	non-NR
E4YJK2_OIKDI	non-NR	non-NR
E4YJK5_OIKDI	non-NR	non-NR
E4YJK8_OIKDI	non-NR	non-NR
E4YJL0_OIKDI	non-NR	non-NR
E4YJL1_OIKDI	non-NR	non-NR
E4YJM3_OIKDI	non-NR	non-NR
E4YJN5_OIKDI	non-NR	non-NR
E4YJP6_OIKDI	non-NR	non-NR
E4YJP7_OIKDI	non-NR	non-NR
E4YJP9_OIKDI	non-NR	non-NR
E4YJQ5_OIKDI	non-NR	non-NR
E4YJR1_OIKDI	non-NR	non-NR
E4YJR2_OIKDI	non-NR	non-NR
E4YJR5_OIKDI	non-NR	non-NR
E4YJR7_OIKDI	non-NR	non-NR
E4YJR8_OIKDI	non-NR	non-NR
E4YJT4_OIKDI	non-NR	non-NR
Q9I7J1_DROME	NR	non-NR
Q9I7J2_DROME	NR	non-NR
E4YJT6_OIKDI	non-NR	non-NR
E4YJT8_OIKDI	non-NR	non-NR
E4YJU5_OIKDI	non-NR	non-NR
E4YJU7_OIKDI	non-NR	non-NR
E4YJV5_OIKDI	non-NR	non-NR
E4YJV8_OIKDI	non-NR	non-NR
E4YJW4_OIKDI	non-NR	non-NR
E4YJX2_OIKDI	non-NR	non-NR
E4YJX6_OIKDI	non-NR	non-NR

E4YJY0_OIKDI	non-NR	non-NR
E4YJY9_OIKDI	non-NR	non-NR
E4YJZ1_OIKDI	non-NR	non-NR
E4YJZ5_OIKDI	non-NR	non-NR
E4YJZ7_OIKDI	non-NR	non-NR
E4YJZ9_OIKDI	non-NR	non-NR
E4YK00_OIKDI	non-NR	non-NR
E4YK01_OIKDI	non-NR	non-NR
E4YK05_OIKDI	non-NR	non-NR
E4YK07_OIKDI	non-NR	non-NR
E4YK08_OIKDI	non-NR	non-NR
E4YK13_OIKDI	non-NR	non-NR
E4YK14_OIKDI	non-NR	non-NR
E4YK15_OIKDI	non-NR	non-NR
E4YK25_OIKDI	non-NR	non-NR
E4YK26_OIKDI	non-NR	non-NR
E4YK27_OIKDI	non-NR	non-NR
E4YK38_OIKDI	non-NR	non-NR
E4YK41_OIKDI	non-NR	non-NR
E4YK47_OIKDI	non-NR	non-NR
E4YK54_OIKDI	non-NR	non-NR
Q9I7L5_DROME	NR	non-NR
E4YK60_OIKDI	non-NR	non-NR
E4YK86_OIKDI	non-NR	non-NR
E4YK87_OIKDI	non-NR	non-NR
E4YK88_OIKDI	non-NR	non-NR
E4YK96_OIKDI	non-NR	non-NR
E4YK99_OIKDI	non-NR	non-NR
E4YKB2_OIKDI	non-NR	non-NR
E4YKB4_OIKDI	non-NR	non-NR
E4YKB9_OIKDI	non-NR	non-NR
E4YKC3_OIKDI	non-NR	non-NR
E4YKC4_OIKDI	non-NR	non-NR
A8PAZ4_BRUMA	non-NR	non-NR
E4YKC6_OIKDI	non-NR	non-NR
E4YKC7_OIKDI	non-NR	non-NR
E4YKC8_OIKDI	non-NR	non-NR
E4YKC9_OIKDI	non-NR	non-NR
E4YKD4_OIKDI	non-NR	non-NR
E4YKD6_OIKDI	non-NR	non-NR
E4YKD8_OIKDI	non-NR	non-NR

E4YKE0_OIKDI	non-NR	non-NR
E4YKE8_OIKDI	non-NR	non-NR
E4YGL4_OIKDI	non-NR	non-NR
E4YGL7_OIKDI	non-NR	non-NR
E4YKF2_OIKDI	non-NR	non-NR
E4YKF3_OIKDI	non-NR	non-NR
E4YKF5_OIKDI	non-NR	non-NR
E4YKG1_OIKDI	non-NR	non-NR
E4YKG3_OIKDI	non-NR	non-NR
E4YKG7_OIKDI	non-NR	non-NR
E4YKH5_OIKDI	non-NR	non-NR
E4YKH8_OIKDI	non-NR	non-NR
E4YKI0_OIKDI	non-NR	non-NR
E4YKI2_OIKDI	non-NR	non-NR
E4YKJ9_OIKDI	non-NR	non-NR
E4YKK1_OIKDI	non-NR	non-NR
E4YKK7_OIKDI	non-NR	non-NR
E4YKL0_OIKDI	non-NR	non-NR
Q9I7I6_DROME	NR	non-NR
Q9I7J0_DROME	NR	non-NR
E4YKL4_OIKDI	non-NR	non-NR
E4YKL5_OIKDI	non-NR	non-NR
E4YKM7_OIKDI	non-NR	non-NR
E4YKN2_OIKDI	non-NR	non-NR
E4YKP0_OIKDI	non-NR	non-NR
E4YKP2_OIKDI	non-NR	non-NR
E4YKP5_OIKDI	non-NR	non-NR
E4YKP8_OIKDI	non-NR	non-NR
E4YKQ0_OIKDI	non-NR	non-NR
E4YKQ4_OIKDI	non-NR	non-NR
E4YKQ5_OIKDI	non-NR	non-NR
E4YKR1_OIKDI	non-NR	non-NR
E4YKR2_OIKDI	non-NR	non-NR
E4YKR7_OIKDI	non-NR	non-NR
E4YKS8_OIKDI	non-NR	non-NR
E4YKT1_OIKDI	non-NR	non-NR
E4YKT4_OIKDI	non-NR	non-NR
E4YKU0_OIKDI	non-NR	non-NR
E4YKU4_OIKDI	non-NR	non-NR
E4YKU5_OIKDI	non-NR	non-NR
E4YKU8_OIKDI	non-NR	non-NR

E4YKU9_OIKDI	non-NR	non-NR
Q9I7I4_DROME	NR	non-NR
E4YKV2_OIKDI	non-NR	non-NR
E4YKX2_OIKDI	non-NR	non-NR
E4YKX7_OIKDI	non-NR	non-NR
E4YKX8_OIKDI	non-NR	non-NR
E4YKY6_OIKDI	non-NR	non-NR
E4YKY8_OIKDI	non-NR	non-NR
TANC2_HUMAN	non-NR	non-NR
K1632_HUMAN	non-NR	non-NR
MOV10_HUMAN	non-NR	non-NR
ZN532_HUMAN	non-NR	non-NR
MTL14_HUMAN	non-NR	non-NR
ARGAL_HUMAN	non-NR	non-NR
SMUF1_HUMAN	non-NR	non-NR
ANO8_HUMAN	non-NR	non-NR
TRPM3_HUMAN	non-NR	non-NR
GBA2_HUMAN	non-NR	non-NR
CWC22_HUMAN	non-NR	non-NR
NCK5L_HUMAN	non-NR	non-NR
CPNE5_HUMAN	non-NR	non-NR
SYTL2_HUMAN	non-NR	non-NR
MAGE1_HUMAN	non-NR	non-NR
K1586_HUMAN	non-NR	non-NR
TNR6C_HUMAN	non-NR	non-NR
ANKH_HUMAN	non-NR	non-NR
ZSWM6_HUMAN	non-NR	non-NR
VAT1L_HUMAN	non-NR	non-NR
ZDBF2_HUMAN	non-NR	non-NR
Q9I7I3_DROME	NR	non-NR
ROBO2_HUMAN	non-NR	non-NR
AGO4_HUMAN	non-NR	non-NR
CHD8_HUMAN	non-NR	non-NR
PCD18_HUMAN	non-NR	non-NR
GPAT1_HUMAN	non-NR	non-NR
CL035_HUMAN	non-NR	non-NR
PLXA4_HUMAN	non-NR	non-NR
K1549_HUMAN	non-NR	non-NR
E41L5_HUMAN	non-NR	non-NR
FBSL_HUMAN	non-NR	non-NR
TRI39_HUMAN	non-NR	non-NR

TPIP1_HUMAN	non-NR	non-NR
Q9HCN2-3	non-NR	non-NR
TMM8A_HUMAN	non-NR	non-NR
GPN1_HUMAN	non-NR	non-NR
Q9HCN5_HUMAN	non-NR	non-NR
SDF2L_HUMAN	non-NR	non-NR
HHATL_HUMAN	non-NR	non-NR
GALT9_HUMAN	non-NR	non-NR
PDE11_HUMAN	non-NR	non-NR
E41LA_HUMAN	non-NR	non-NR
SYF1_HUMAN	non-NR	non-NR
FGF22_HUMAN	non-NR	non-NR
CELR2_HUMAN	non-NR	non-NR
DPOD4_HUMAN	non-NR	non-NR
BRMS1_HUMAN	non-NR	non-NR
ZN304_HUMAN	non-NR	non-NR
Q9HCX8_HUMAN	non-NR	non-NR
Q9HCY4_HUMAN	non-NR	non-NR
Q9I7I1_DROME	NR	non-NR
S10AE_HUMAN	non-NR	non-NR
ZN334_HUMAN	non-NR	non-NR
AT131_HUMAN	non-NR	non-NR
LYRM4_HUMAN	non-NR	non-NR
E4YH25_OIKDI	non-NR	non-NR
SPCS_HUMAN	non-NR	non-NR
CHM1A_HUMAN	non-NR	non-NR
PTPRH_HUMAN	non-NR	non-NR
TM9S3_HUMAN	non-NR	non-NR
MOG1_HUMAN	non-NR	non-NR
E4YH36_OIKDI	non-NR	non-NR
E4YH48_OIKDI	non-NR	non-NR
Q9HD52_HUMAN	non-NR	non-NR
GAGD2_HUMAN	non-NR	non-NR
MYO10_HUMAN	non-NR	non-NR
Q9HD86_HUMAN	non-NR	non-NR
CF050_HUMAN	non-NR	non-NR
RETN_HUMAN	non-NR	non-NR
Q9HDB3_HUMAN	non-NR	non-NR
Q9HDC8_HUMAN	non-NR	non-NR
HRSL1_HUMAN	non-NR	non-NR
RHO1_ASHGO	non-NR	non-NR

CCNB3_DROME	NR	non-NR
PP2A1_EMENI	non-NR	non-NR
PEX6_PENCH	non-NR	non-NR
RL14_THEAC	non-NR	non-NR
LGUL_PSEAE	non-NR	non-NR
ETFD_PSEAE	non-NR	non-NR
Y2418_PSEAE	non-NR	non-NR
ACON1_PSEAE	non-NR	non-NR
COBP_PSEAE	non-NR	non-NR
PR2_DROME	non-NR	non-NR
Q9I7H5_DROME	non-NR	non-NR
Q9I7H8_DROME	non-NR	non-NR

II. Results of the 2nd level prediction in identifying the subfamilies of NRs

Protein code	Predicted	Observed
THA_CHICK	NR1	NR1
THA1_SHEEP	NR1	NR1
THB2_MOUSE	NR1	NR1
THB1_HUMAN	NR1	NR1
Q6F3J6_CONMY	NR1	NR1
THB1_RAT	NR1	NR1
THAA_XENLA	NR1	NR1
THB1_MOUSE	NR1	NR1
Q7T0K4_AMBME	NR1	NR1
THAB_XENLA	NR1	NR1
THA_RANCA	NR1	NR1
THA_SALSA	NR1	NR1
THB2_HUMAN	NR1	NR1
Q6F3J5_CONMY	NR1	NR1
THA_HIPHI	NR1	NR1
THA_NECMA	NR1	NR1
THA1_BRARE	NR1	NR1
Q766D2_ORYLA	NR1	NR1
Q5RZV6_PSEAM	NR1	NR1
THAA_PAROL	NR1	NR1
THBB_XENLA	NR1	NR1
THA_HUMAN	NR1	NR1
THA_PIG	NR1	NR1
THA_RAT	NR1	NR1

Q80Y90_MOUSE	NR1	NR1
THA_MOUSE	NR1	NR1
THAB_PAROL	NR1	NR1
Q800D8_SPAAU	NR1	NR1
THB_BRARE	NR1	NR1
THB_PAROL	NR1	NR1
ERBA_AVIER	NR1	NR1
Q6F3J8_CONMY	NR1	NR1
THB_RANCA	NR1	NR1
Q90Y21_SALSA	NR1	NR1
Q90Y22_SALSA	NR1	NR1
THBA_XENLA	NR1	NR1
THB_CHICK	NR1	NR1
THB_CAIMO	NR1	NR1
Q7T0K3_AMBME	NR1	NR1
Q6Y9T0_NECMA	NR1	NR1
Q766D1_ORYLA	NR1	NR1
P97513_MUSSP	NR1	NR1
RRA_MOUSE	NR1	NR1
Q6F3J7_CONMY	NR1	NR1
RRA_HUMAN	NR1	NR1
RRA_CHICK	NR1	NR1
RRG1_HUMAN	NR1	NR1
Q91YX2_MOUSE	NR1	NR1
RRG1_MOUSE	NR1	NR1
Q9I8T3_AMBME	NR1	NR1
RRB_HUMAN	NR1	NR1
RRB_CHICK	NR1	NR1
RRG2_HUMAN	NR1	NR1
RRB_COTJA	NR1	NR1
RRA_NOTVI	NR1	NR1
Q8VHB6_MESAU	NR1	NR1
Q5U645_HUMAN	NR1	NR1
RRG2_MOUSE	NR1	NR1
Q9I8T2_AMBME	NR1	NR1
Q9QWJ1_RAT	NR1	NR1
Q8VHB8_MESAU	NR1	NR1
Q8VHB7_MESAU	NR1	NR1
RRG_XENLA	NR1	NR1
PPAT_PIG	NR1	NR1
Q8HZ56_SHEEP	NR1	NR1

PPAT_BOVIN	NR1	NR1
PPAT_MACMU	NR1	NR1
RRA_FUGRU	NR1	NR1
Q95J17_MACFA	NR1	NR1
Q8MHZ0_MACFA	NR1	NR1
Q95KZ8_MACFA	NR1	NR1
PPAT_HUMAN	NR1	NR1
RRB_MOUSE	NR1	NR1
Q866S0_CAPHI	NR1	NR1
Q5RFE5_PONPY	NR1	NR1
PPAT_RABIT	NR1	NR1
Q92019_XENLA	NR1	NR1
Q8JHU6_ANAPL	NR1	NR1
Q8HXA4_RABIT	NR1	NR1
PPAT_RAT	NR1	NR1
PPAT_CRIGR	NR1	NR1
PPAT_MOUSE	NR1	NR1
Q7ZTI3_BRARE	NR1	NR1
Q90272_BRARE	NR1	NR1
Q91391_BRARE	NR1	NR1
Q90271_BRARE	NR1	NR1
RRG_BRARE	NR1	NR1
Q9I878_CHICK	NR1	NR1
RRA_XENLA	NR1	NR1
Q98SF8_XENLA	NR1	NR1
Q6PA31_XENLA	NR1	NR1
NR1D2_MOUSE	NR1	NR1
Q8C598_MOUSE	NR1	NR1
Q7TNH1_MESAU	NR1	NR1
PPAS_MOUSE	NR1	NR1
PPAS_HUMAN	NR1	NR1
Q99ND3_RAT	NR1	NR1
PPAR_RAT	NR1	NR1
PPAR_CANFA	NR1	NR1
NR1D2_RAT	NR1	NR1
Q6I9S1_HUMAN	NR1	NR1
PPAR_HUMAN	NR1	NR1
PPAR_MOUSE	NR1	NR1
Q62879_RAT	NR1	NR1
Q9I8W4_CHICK	NR1	NR1
NR1H2_HUMAN	NR1	NR1

PPAT_XENLA	NR1	NR1
Q8QGC1_9AVES	NR1	NR1
Q9QWI0_RAT	NR1	NR1
Q866Q2_PIG	NR1	NR1
Q9I8W3_CHICK	NR1	NR1
Q6P6S7_RAT	NR1	NR1
Q9N2H4_RABIT	NR1	NR1
PPAR_PHACI	NR1	NR1
Q8BP65_MOUSE	NR1	NR1
Q90970_CHICK	NR1	NR1
NR1H3_RAT	NR1	NR1
PPAR_CAVPO	NR1	NR1
NR1H3_MOUSE	NR1	NR1
Q91X41_MOUSE	NR1	NR1
Q9U3Y4_AEDAL	NR1	NR1
ECR_LUCCU	NR1	NR1
NR1H2_RAT	NR1	NR1
PPAR_XENLA	NR1	NR1
Q6AXA9_XENLA	NR1	NR1
Q8IW13_HUMAN	NR1	NR1
Q5U0N9_HUMAN	NR1	NR1
NR1D1_HUMAN	NR1	NR1
NR1H2_MOUSE	NR1	NR1
ECR_AEDAE	NR1	NR1
Q8MYA6_9NEOP	NR1	NR1
Q7T029_PLEPL	NR1	NR1
Q9W712_PLAFE	NR1	NR1
Q9VVM9_DROME	NR1	NR1
Q8MYA7_9NEOP	NR1	NR1
O76827_CERCA	NR1	NR1
Q8IQS3_DROME	NR4	NR1
E75A_DROME	NR1	NR1
Q9I8F6_SALSA	NR1	NR1
ECR_MANSE	NR1	NR1
RORA_MOUSE	NR1	NR1
Q6GMA2_XENLA	NR1	NR1
O77255_CHOFU	NR1	NR1
Q6RVD3_PLOIN	NR1	NR1
Q8JHU1_CHICK	NR1	NR1
Q5RGZ2_BRARE	NR1	NR1
O77240_CHOFU	NR1	NR1

Q8JIT8_SALSA	NR1	NR1
Q8AXU8_CHICK	NR1	NR1
O44337_9ACAR	NR1	NR1
Q68CY8_HUMAN	NR1	NR1
Q5XI75_RAT	NR1	NR1
NR1H4_RAT	NR1	NR1
ECR_HELVI	NR1	NR1
O44336_9ACAR	NR1	NR1
O02035_TENMO	NR1	NR1
Q8K476_MESAU	NR1	NR1
Q8K473_MESAU	NR1	NR1
Q8K474_MESAU	NR1	NR1
Q8K475_MESAU	NR1	NR1
HR3_GALME	NR1	NR1
ECR_CHITE	NR1	NR1
Q9BMC6_HELAM	NR1	NR1
E75_GALME	NR1	NR1
Q7Z2W0_HUMAN	NR1	NR1
NR1H4_HUMAN	NR1	NR1
Q8SPF5_RABIT	NR1	NR1
VDR_CHICK	NR1	NR1
Q8WSA2_BOMMO	NR1	NR1
Q9U5G4_BOMMO	NR1	NR1
E75_CHOFU	NR1	NR1
E75_MANSE	NR1	NR1
Q8JHU2_CHICK	NR1	NR1
Q8JJ28_XENLA	NR1	NR1
Q6P7H5_XENLA	NR1	NR1
VDR_COTJA	NR1	NR1
Q27547_CHOFU	NR1	NR1
NR1D1_RAT	NR1	NR1
HR3_DROME	NR1	NR1
Q6DGW7_BRARE	NR1	NR1
Q95P94_BOMMO	NR1	NR1
PXR_HUMAN	NR1	NR1
Q811X2_MOUSE	NR1	NR1
VDR_SAGOE	NR1	NR1
E75B_DROME	NR1	NR1
Q8IQS2_DROME	NR1	NR1
VDR_HUMAN	NR1	NR1
Q9I8F7_SALSA	NR1	NR1

Q8SQ01_MACMU	NR1	NR1
Q9IB74_PAROL	NR1	NR1
VDR_RAT	NR1	NR1
VDR_BOVIN	NR1	NR1
Q922X0_MOUSE	NR1	NR1
VDR_MOUSE	NR1	NR1
PXR_RAT	NR1	NR1
Q5RAP4_PONPY	NR1	NR1
PPAS_XENLA	NR1	NR1
Q9IB73_PAROL	NR1	NR1
Q62702_RAT	NR1	NR1
PXR_MOUSE	NR1	NR1
RORG_MOUSE	NR1	NR1
Q9NH86_AEDAE	NR1	NR1
VDR_XENLA	NR1	NR1
Q98934_CHICK	NR1	NR1
RORB_MOUSE	NR1	NR1
RORB_RAT	NR1	NR1
RORB_HUMAN	NR1	NR1
Q7ZU39_BRARE	NR1	NR1
Q91839_XENLA	NR1	NR1
Q8AVZ2_XENLA	NR1	NR1
Q9TU02_RABIT	NR1	NR1
Q9U5G3_BOMMO	NR1	NR1
NHR23_CAEEL	NR1	NR1
Q6Q2C9_PLOIN	NR1	NR1
Q8SXA4_DROME	NR1	NR1
Q5REL4_PONPY	NR1	NR1
Q5R8V3_PONPY	NR1	NR1
NR1I3_MOUSE	NR1	NR1
Q811W9_MOUSE	NR1	NR1
NR1I3_HUMAN	NR1	NR1
NR1I3_MACMU	NR1	NR1
CNR14_CAEEL	NR1	NR1
Q9U3F1_CAEEL	NR1	NR1
NR1I3_PHOSI	NR1	NR1
NR1I3_CALUR	NR1	NR1
Q811X1_MOUSE	NR1	NR1
Q811X0_MOUSE	NR1	NR1
Q6GZ84_HUMAN	NR1	NR1
Q6GZ68_HUMAN	NR1	NR1

Q8IQS1_DROME	NR5	NR1
Q76FN2_RAT	NR1	NR1
RXRA_RAT	NR2	NR2
RXRA_MOUSE	NR2	NR2
RXRA_HUMAN	NR2	NR2
Q5TJF7_CANFA	NR2	NR2
RXRB_MOUSE	NR2	NR2
Q6LC96_MOUSE	NR2	NR2
Q6MGB3_RAT	NR2	NR2
RXRG_MOUSE	NR2	NR2
RXRG_HUMAN	NR2	NR2
Q5REL6_PONPY	NR2	NR2
RXRA_XENLA	NR2	NR2
7UP1_DROME	NR2	NR2
RXRG_CHICK	NR2	NR2
RXRB_BRARE	NR2	NR2
Q6INZ0_XENLA	NR2	NR2
Q91613_XENLA	NR2	NR2
RXRA_BRARE	NR2	NR2
Q86GV5_BRAFL	NR2	NR2
COT1_MOUSE	NR2	NR2
COT1_HUMAN	NR2	NR2
COT1_BOVIN	NR2	NR2
Q62681_RAT	NR2	NR2
Q6DHP9_BRARE	NR2	NR2
COT2_BOVIN	NR2	NR2
COT2_HUMAN	NR2	NR2
COT2_MOUSE	NR2	NR2
COT2_RAT	NR2	NR2
NR2F1_BRARE	NR2	NR2
Q91430_BRARE	NR2	NR2
Q6PHF2_BRARE	NR2	NR2
Q9W745_XENLA	NR2	NR2
Q91840_XENLA	NR2	NR2
Q8T5C6_BIOGL	NR2	NR2
COT2_CHICK	NR2	NR2
Q6DCH6_XENLA	NR2	NR2
Q8VCR0_MOUSE	NR2	NR2
Q7ZXI7_XENLA	NR2	NR2
Q9PS79_9PIPI	NR2	NR2
Q8UUM6_ORYLA	NR2	NR2

RXRD_BRARE	NR2	NR2
Q66TQ0_9CAEN	NR2	NR2
Q6V7U7_LOCMI	NR2	NR2
Q66J63_XENLA	NR2	NR2
Q91720_XENLA	NR2	NR2
Q5RCZ5_PONPY	NR2	NR2
Q95K90_MACFA	NR2	NR2
RXRG_BRARE	NR2	NR2
Q15625_HUMAN	NR2	NR2
Q9VLI7_DROME	NR2	NR2
NR2F5_BRARE	NR2	NR2
Q9GSG8_AEDAE	NR2	NR2
USP_MANSE	NR2	NR2
Q6PH18_BRARE	NR2	NR2
TR4_HUMAN	NR2	NR2
TR4_MOUSE	NR2	NR2
Q6P0E6_BRARE	NR2	NR2
TR4_RAT	NR2	NR2
Q9GSG7_AEDAE	NR2	NR2
Q9U7D9_LOCMI	NR2	NR2
USP_BOMMO	NR2	NR2
Q8VIJ3_MOUSE	NR2	NR2
Q8VIJ4_RAT	NR2	NR2
HNF4_DROME	NR2	NR2
Q62152_MOUSE	NR2	NR2
Q6GL59_XENTR	NR2	NR2
Q7T0T7_XENLA	NR2	NR2
Q922G8_MOUSE	NR2	NR2
Q9NFY1_TENMO	NR2	NR2
EAR2_MOUSE	NR2	NR2
Q6P117_BRARE	NR2	NR2
EAR2_RAT	NR2	NR2
Q5QPB8_HUMAN	NR2	NR2
Q962I5_LUCCU	NR2	NR2
Q6P115_BRARE	NR2	NR2
O61449_9ACAR	NR2	NR2
O61448_9ACAR	NR2	NR2
HNF4A_MOUSE	NR2	NR2
HNF4A_RAT	NR2	NR2
Q6PHH5_BRARE	NR2	NR2
Q9JJI9_TAMSI	NR2	NR2

Q7YRQ5_BOVIN	NR2	NR2
HNF4A_HUMAN	NR2	NR2
Q5RH32_BRARE	NR2	NR2
Q7SZG3_FUGRU	NR2	NR2
Q8AVK3_XENLA	NR2	NR2
Q5QPB7_HUMAN	NR2	NR2
Q6IVK1_PLOIN	NR2	NR2
Q95WF7_ACRMI	NR2	NR2
Q6LDB2_9MURI	NR2	NR2
Q8AXB6_BRARE	NR2	NR2
Q8I1M8_9NEOP	NR2	NR2
HNF4A_XENLA	NR2	NR2
Q8JID3_CHICK	NR2	NR2
Q6B4V6_HUMAN	NR2	NR2
Q6B4V7_HUMAN	NR2	NR2
Q6B4V5_HUMAN	NR2	NR2
HNF4B_XENLA	NR2	NR2
O77100_AEDAE	NR2	NR2
Q7YRQ4_BOVIN	NR2	NR2
TR2_HUMAN	NR2	NR2
Q15626_HUMAN	NR2	NR2
NR2E3_HUMAN	NR2	NR2
Q6DEH0_BRARE	NR2	NR2
O46175_BOMMO	NR2	NR2
NR2E1_ORYLA	NR2	NR2
Q8JHW6_FUGRU	NR2	NR2
Q9Y1J4_SCHMA	NR2	NR2
NR2E1_MOUSE	NR2	NR2
HNF4G_MOUSE	NR2	NR2
Q7Z2V9_HUMAN	NR2	NR2
NR2E1_CHICK	NR2	NR2
NR2E1_HUMAN	NR2	NR2
HNF4G_HUMAN	NR2	NR2
Q8MX79_BRAFL	NR2	NR2
Q90WV5_CHICK	NR2	NR2
NR2E1_XENLA	NR2	NR2
Q9VP28_DROME	NR2	NR2
HR78_DROME	NR2	NR2
TLL_DROVI	NR2	NR2
Q9VML1_DROME	NR2	NR2
Q5U7E0_MUSDO	NR2	NR2

Q86PK5_CAEEL	NR2	NR2
Q61WY2_CAEBR	NR2	NR2
Q8IVZ9_HUMAN	NR2	NR2
Q688B5_CAEEL	NR2	NR2
O77101_AEDAE	NR2	NR2
Q86PK4_CAEEL	NR2	NR2
GCR_SAISC	NR3	NR3
GCR_HUMAN	NR3	NR3
GCR_SAIBB	NR3	NR3
GCR_AOTNA	NR3	NR3
Q6N0A4_HUMAN	NR3	NR3
GCR_SAGOE	NR3	NR3
Q6XLJ0_CALJA	NR3	NR3
Q5R7M7_PONPY	NR3	NR3
Q5R9P5_PONPY	NR3	NR3
ANDR_PANTR	NR3	NR3
ANDR_PAPHA	NR3	NR3
ANDR_MACFA	NR3	NR3
ANDR_MACMU	NR3	NR3
Q9UN21_HUMAN	NR3	NR3
Q5S4M0_PIG	NR3	NR3
GCR_MOUSE	NR3	NR3
ANDR_EULFC	NR3	NR3
GCR_TUPGB	NR3	NR3
ANDR_PIG	NR3	NR3
GCR_CAVPO	NR3	NR3
PRGR_HUMAN	NR3	NR3
ANDR_CANFA	NR3	NR3
ANDR_CROCR	NR3	NR3
GCR_RABIT	NR3	NR3
ANDR_RAT	NR3	NR3
ANDR_MOUSE	NR3	NR3
PRGR_RABIT	NR3	NR3
MCR_SAISC	NR3	NR3
Q6XLI9_CALJA	NR3	NR3
MCR_HUMAN	NR3	NR3
MCR_RAT	NR3	NR3
MCR_MOUSE	NR3	NR3
PRGR_RAT	NR3	NR3
PRGR_MOUSE	NR3	NR3
MCR_TUPGB	NR3	NR3

Q13771_HUMAN	NR3	NR3
Q6DDL0_XENLA	NR3	NR3
GCR_XENLA	NR3	NR3
Q66J29_XENLA	NR3	NR3
Q8NG42_HUMAN	NR3	NR3
GCR_PAROL	NR3	NR3
Q8JJ91_HAPBU	NR3	NR3
Q8JJ90_HAPBU	NR3	NR3
Q5WP01_ONCMY	NR3	NR3
Q9I8F5_9TELE	NR3	NR3
Q8UWB7_ORENI	NR3	NR3
O93245_ONCMY	NR3	NR3
Q8QFV7_HAPBU	NR3	NR3
O93497_PAGMA	NR3	NR3
ANDR_RANCA	NR3	NR3
Q66VR6_MICUN	NR3	NR3
Q60I32_GAMAF	NR3	NR3
Q9PWG5_ANGJA	NR3	NR3
Q6IVJ3_DICLA	NR3	NR3
Q8NG44_HUMAN	NR3	NR3
Q801Z1_GASAC	NR3	NR3
Q8NG45_HUMAN	NR3	NR3
Q8NG43_HUMAN	NR3	NR3
ESR1_PIG	NR3	NR3
Q9DDZ4_MICSA	NR3	NR3
ESR1_BOVIN	NR3	NR3
ESR1_HUMAN	NR3	NR3
ESR1_MOUSE	NR3	NR3
Q7SZI0_FUNHE	NR3	NR3
ESR1_HORSE	NR3	NR3
ESR2_ONCMY	NR3	NR3
ESR1_MESAU	NR3	NR3
ESR1_ORYLA	NR3	NR3
Q765N7_ALLMI	NR3	NR3
Q8AYH0_COTJA	NR3	NR3
ESR1_CHICK	NR3	NR3
Q8UWB0_CAICR	NR3	NR3
Q6W5G7_XENLA	NR3	NR3
ESR1_POEGU	NR3	NR3
Q90WS8_BRARE	NR3	NR3
ESR1_XENLA	NR3	NR3

ESRB1_CARAU	NR3	NR3
Q7ZU32_BRARE	NR3	NR3
Q8AV62_BRARE	NR3	NR3
Q60GT9_GAMAF	NR3	NR3
Q8UWA9_CNEUN	NR3	NR3
ESR2_STUVU	NR3	NR3
ESR2_MOUSE	NR3	NR3
ESR2_RAT	NR3	NR3
Q8JJB9_CYPCA	NR3	NR3
ESR2_HUMAN	NR3	NR3
ESR1_ONCMY	NR3	NR3
Q8UWB8_ORENI	NR3	NR3
Q98SM7_BRARE	NR3	NR3
ESR2_BOVIN	NR3	NR3
Q5PR29_BRARE	NR3	NR3
Q6XSH2_MICSA	NR3	NR3
ESR2_PIG	NR3	NR3
ESR2_SHEEP	NR3	NR3
Q8QHK9_PAROL	NR3	NR3
Q98SM8_BRARE	NR3	NR3
ESR2_ANGJA	NR3	NR3
Q90WS9_BRARE	NR3	NR3
ESR2_CALJA	NR3	NR3
ESR1_PAGMA	NR3	NR3
Q6H9M5_SPAAU	NR3	NR3
Q804Q6_ACASC	NR3	NR3
Q6H9M4_SPAAU	NR3	NR3
Q7T3U5_9TELE	NR3	NR3
ESRB2_CARAU	NR3	NR3
ESR1_SPAAU	NR3	NR3
Q8UW75_ORYLA	NR3	NR3
ESR3_MICUN	NR3	NR3
Q7T2K7_9LABR	NR3	NR3
Q6XSH1_MICSA	NR3	NR3
Q9DEV4_XENLA	NR3	NR3
ESR2_ORENI	NR3	NR3
ESR2_SPAAU	NR3	NR3
Q7T2K8_9LABR	NR3	NR3
Q6TGB3_HAPBU	NR3	NR3
Q800Q2_ZOAVI	NR3	NR3
ESR1_ORENI	NR3	NR3

Q804Q7_ACASC	NR3	NR3
Q8QHL0_PAROL	NR3	NR3
ESR1_ICTPU	NR3	NR3
Q6W5G9_XENTR	NR3	NR3
ESR1_OREAU	NR3	NR3
Q69F36_CARAU	NR3	NR3
Q95MF0_MACAR	NR3	NR3
ESR1_BRARE	NR3	NR3
Q5XXP1_9TELE	NR3	NR3
ESR2_ICTPU	NR3	NR3
Q90WV1_CARAU	NR3	NR3
Q95ME9_CALJA	NR3	NR3
Q91Z86_MOUSE	NR3	NR3
ESR2_CHICK	NR3	NR3
ESR2_COTJA	NR3	NR3
ERR3_HUMAN	NR3	NR3
ERR3_MOUSE	NR3	NR3
ERR1_MOUSE	NR3	NR3
Q5UKY7_CHICK	NR3	NR3
ERR3_PONPY	NR3	NR3
Q6W5G5_XENLA	NR3	NR3
Q6W5G6_XENLA	NR3	NR3
ERR2_RAT	NR3	NR3
Q6Q6F4_BRARE	NR3	NR3
Q8C7A6_MOUSE	NR3	NR3
ERR2_MOUSE	NR3	NR3
Q8CCV5_MOUSE	NR3	NR3
Q6Q6F6_BRARE	NR3	NR3
Q6QMY5_CANFA	NR3	NR3
Q9VSE9_DROME	NR3	NR3
Q6AX97_XENLA	NR3	NR3
NR4A1_HUMAN	NR4	NR4
Q6IBU8_HUMAN	NR4	NR4
Q8N3V2_HUMAN	NR4	NR4
Q5RBB0_PONPY	NR4	NR4
NR4A1_CANFA	NR4	NR4
NR4A1_MOUSE	NR4	NR4
NR4A2_MOUSE	NR4	NR4
NR4A2_HUMAN	NR4	NR4
NR4A2_RAT	NR4	NR4
Q5R5Y4_PONPY	NR4	NR4

NR4A1_RAT	NR4	NR4
NR4A3_MOUSE	NR4	NR4
NR4A3_RAT	NR4	NR4
Q6NXU0_HUMAN	NR4	NR4
NR4A3_HUMAN	NR4	NR4
NR4A2_XENLA	NR4	NR4
Q7T0V3_XENLA	NR4	NR4
Q6DH08_BRARE	NR4	NR4
Q6GMG3_BRARE	NR4	NR4
Q8INU7_DROME	NR4	NR4
Q9R1W4_MOUSE	NR4	NR4
O97727_PIG	NR4	NR4
Q61JK3_CAEBR	NR4	NR4
NR5A2_HUMAN	NR5	NR5
Q9UEC0_HUMAN	NR5	NR5
Q9QWM0_RAT	NR5	NR5
NR5A2_MOUSE	NR5	NR5
NR5A2_CHICK	NR5	NR5
Q5XGE7_XENTR	NR5	NR5
Q9IB82_RANRU	NR5	NR5
Q91544_XENLA	NR5	NR5
Q90YL6_BRARE	NR5	NR5
Q9GKL1_HORSE	NR5	NR5
O42186_BRARE	NR5	NR5
Q9IB81_RANRU	NR5	NR5
Q9PWI7_CHICK	NR5	NR5
STF1_RAT	NR5	NR5
Q812G5_MOUSE	NR5	NR5
O42102_CHICK	NR5	NR5
Q90XC4_POEGU	NR5	NR5
STF1_BOVIN	NR5	NR5
STF1_MOUSE	NR5	NR5
Q9YI54_TRASC	NR5	NR5
STF1_PIG	NR5	NR5
STF1_HUMAN	NR5	NR5
STF1_MACEU	NR5	NR5
STF1_HORSE	NR5	NR5
Q9YI95_RANRU	NR5	NR5
Q6QHU4_PLEWA	NR5	NR5
O93258_ORYLA	NR5	NR5
Q7ZT68_ORENI	NR5	NR5

Q800U8_BRARE	NR5	NR5
Q8UV27_CLAGA	NR5	NR5
Q9IAI9_BRARE	NR5	NR5
FTZF1_BOMMO	NR5	NR5
Q91601_XENLA	NR5	NR5
DAX1_PIG	NR0	NR0
DAX1_RAT	NR0	NR0
DAX1_HUMAN	NR0	NR0
Q9PTE9_CHICK	NR0	NR0
SHP_MOUSE	NR0	NR0
SHP_HUMAN	NR0	NR0
